# Space-use, movement and dispersal of sub-adult cougars in a geographically isolated population

**DOI:** 10.7717/peerj.1118

**Published:** 2015-08-06

**Authors:** Carl D. Morrison, Mark S. Boyce, Scott E. Nielsen

**Affiliations:** 1 Department of Biological Sciences, University of Alberta, Edmonton, AB, Canada; 2 Department of Renewable Resources, University of Alberta, Edmonton, AB, Canada

**Keywords:** Cougar, Dispersal, Saskatchewan, Alberta, Resource selection function, Habitat, *Puma concolor*, Movement, Range expansion, Sub-adult

## Abstract

Cougar (*Puma concolor*) observations have increased in Midwest North America, with breeding populations re-establishing in several regions east of their contemporary range. The Cypress Hills Uplands, located in southwest Saskatchewan and southeast Alberta, was recently re-colonized by cougars and now supports the easternmost confirmed breeding population of cougars in Canada. A number of factors contribute to this cougar range expansion, but it is dispersal that provides the mechanism for re-colonization of historic range. We used GPS-collar data to examine space-use and movement behavior of sub-adult cougars, the age class associated with dispersal, in the Cypress Hills. Conditional logistic regression and a two-stage modeling approach were used to estimate resource selection functions (RSF) of sub-adult cougars during two distinct ranging behaviors: transient movements (i.e., dispersal and exploratory forays) and localizing movements (i.e., temporary home ranges). Linear regression was used to model movement rates, measured as the distance between consecutive 3-h GPS-relocations, of sub-adult cougars relative to different habitats, times of day and between transient and localizing behavior. All individual sub-adult cougars displayed bouts of transient and localizing behavior. All male cougars dispersed from their natal ranges and travelled considerably farther distances than female cougars. One male dispersed over 750 km eastward through the agricultural belt of northern Montana and southern Saskatchewan. Males occupied temporary home ranges in more open habitats on the fringes of the insular Cypress Hills, while females appeared to be recruited into the adult population, occupying treed habitat that provided more suitable cover. During both ranging behaviors, sub-adult cougars selected for rugged terrain and proximity to hydrological features (likely supporting riparian habitats) and avoided open cover types. Differences in habitat selection between ranging behaviors were observed in response to open water, roads and elevation. Although certain habitat characteristics were preferred, transient and localizing cougars used fast-paced nocturnal movements and shortened daytime movements when traversing open habitats to effectively limit their residency and exposure in less-suitable landscapes. Additionally, cougars moved greater distances at night during transient behavior compared to localizing behavior indicating cougars used cover of darkness to traverse novel terrain. In doing so, sub-adult cougars can successfully disperse several hundred kilometres across a matrix of open habitat in search of resources and mates.

## Introduction

Carnivore populations around the world have experienced extensive reductions in their ranges from anthropogenic stressors such as habitat loss, habitat fragmentation and over exploitation (Ripple et al., 2014). Maintaining or restoring connectivity between spatially segregated populations, often separated by matrices of suboptimal habitats, is a growing conservation priority. Natal dispersal provides a mechanism to maintain gene flow among populations, to resupply sink populations and to re-colonize vacant habitat patches (Clobert et al., 2012). Yet little is known regarding the fine-scale movements and selection of habitats made by dispersing large carnivores because far ranging individuals are inherently difficult to track. This limits our ability to manage metapopulation dynamics and to promote re-colonization of former range. Expanding cougar (*Puma concolor*) populations in parts of North America, coupled with advances in GPS-tracking technology, provide an opportunity to examine the dispersal ecology of a solitary large carnivore which has applications for carnivore conservation around the globe.

Cougars historically occurred across much of North America but were extirpated from large portions of their eastern range by direct persecution and reduced abundance of prey (Sunquist & Sunquist, 2002). A significant increase in cougar occurrences (e.g., sightings, genetic samples, individual specimens) throughout the Midwest in the past two decades indicates that cougars are re-colonizing portions of their former range and expanding their distribution eastward (LaRue et al., 2012). Isolated breeding populations of cougars are now confirmed in areas east of their contemporary range where there has not been an established population of cougars for the past century. This includes the North Dakota Badlands, the Black Hills in South Dakota, western Nebraska and the Cypress Hills which span the Alberta-Saskatchewan border (Cougar Network, 2007; LaRue et al., 2012; Morrison et al., 2014). In Canada, the presence of wild cougars also has been recently confirmed in Ontario (Rosatte, 2011) and Manitoba (Watkins, 2005), although the source of these cougars and their status there remain unclear.

Eastward range expansion can be explained by several factors including a shift towards conservation-based cougar management, an increase in deer abundance throughout midwestern North America (Cougar Management Guidelines Working Group, 2005), the cougars’ adaptability to moderate levels of human activity (Morrison et al., 2014), and perhaps most importantly, their dispersal ecology (Thompson & Jenks, 2005; LaRue et al., 2012). A recent study of confirmed cougar occurrences in the Midwest found that 76% (*n* = 29) of known-sex carcasses were males (sex class typically associated with dispersal in polygynous mammal species) and that cougar confirmations declined as distance from western source populations increased (LaRue et al., 2012). Furthermore, Thompson & Jenks (2010) observed several sub-adult cougars dispersing into the Midwest region from the Black Hills, which was re-colonized in the late 1990s. These findings lend support to the hypothesis that dispersal is facilitating cougar range expansion which may be unfolding via a stepping-stone process (LaRue et al., 2012).

Dispersal can be described as a three-stage process including emigration, transience and settlement (Clobert, Ims & Rousset, 2004; Van Dyck & Baguette, 2005). Each stage of dispersal has important implications for understanding metapopulation dynamics, which are increasingly being incorporated into conservation and management strategies for large carnivores (Fattebert et al., 2013; Stoner et al., 2013; Dolrenry et al., 2014). Reducing competition for mates and resources, as well as reducing inbreeding, have been hypothesized as promoting dispersal (Logan & Sweanor, 2001; Thompson & Jenks, 2010; Clobert et al., 2012). Breeding opportunities are a major determinant for young males establishing permanent home ranges in the post-dispersal stage (Stoner et al., 2013; Thompson & Jenks, 2010). While other studies have examined the ecological parameters influencing emigration and settlement behavior in cougars (e.g., Stoner et al., 2013), our study focuses on the “transient” behavior of dispersal because this provides the linkages within metapopulations, and more specifically in the context of range expansion, it provides the mechanism for re-colonization.

Transient behavior in cougars typically occurs in the sub-adult life stage (Logan & Sweanor, 2001). This includes natal dispersal but can also be exploratory forays by individuals who do not disperse. Cougar movements during this period are characterized by one-way directional bouts broken up by periods of localizing behavior which have been termed temporary or transient home ranges (THRs; Beier, 1995; Sweanor, Logan & Hornocker, 2000; Stoner et al., 2008). The use of THRs during natal dispersal has been documented for other large felids including leopards (Fattebert et al., 2013) and tigers (Smith, 1993), as well as other taxa such as spotted owls (Forsman et al., 2002). These localizing events may be an important component of dispersal during which individuals evaluate competition for resources and mates. Therefore THRs that are abandoned may represent aborted attempts to establish a permanent home range (Stoner et al., 2008).

Like other polygynous carnivores, cougars exhibit sex-biased dispersal; almost all males disperse while approximately 50% of sub-adult females remain philopatric (Logan & Sweanor, 2001; Stoner et al., 2013). Sweanor, Logan & Hornocker (2000) describe the onset of dispersal as the departure from the cougar’s natal range which occurs at an average age of 15 months. Congruent with differential dispersal behavior, males typically disperse greater distances than females, often covering several hundred kilometres (Sweanor & Logan, 2010). Instances of extreme long-distance dispersals exceeding 1,000 km have been documented for both sexes (female: 1,341 km (Stoner et al., 2008); male: 1,067 km straight-line (Thompson & Jenks, 2005)).

Dispersing cougars use habitats suited to their biological needs (i.e., providing cover and prey), but also have been documented using fast-paced movements (Sweanor, Logan & Hornocker, 2000; Dickson, Jenness & Beier, 2005) and corridors (Beier, 1995) to cross unsuitable habitat. Indeed, dispersal by panthers in Florida was impeded by anthropogenic landscape features that reduced connectivity (lack of corridors) eventually leading to juvenile cougars, including males, returning to the vicinity of their natal ranges (Maehr et al., 2002). Flat open expanses were also sufficient barriers to movement that limited gene flow among populations (McRea et al., 2005) with cougars selecting against grasslands, agriculture and pasturelands (Laing, 1988; Dickson, Jenness & Beier, 2005).

Past studies of dispersal in far ranging and cryptic species have often been based on coarse spatial and temporal data due largely to technological limitations. For cougars, data were often collected once per week (Beier, 1995; Thompson & Jenks, 2010) or opportunistically if the individual was located by other means, such as human-caused mortality, camera traps or genetic samples (Thompson & Jenks, 2005; Cougar Network, 2007; LaRue et al., 2012). As a result, the limited amount of research on cougar dispersal has focused primarily on coarse scales of movement such as the direction and straight-line distance travelled (Thompson & Jenks, 2005; Thompson & Jenks, 2010; Stoner et al., 2013). Other studies have relied on expert opinion (LaRue et al., 2012) and isotopic clues (Henaux et al., 2011) to examine potential dispersal corridors.

Advances in satellite collar telemetry have facilitated fine-scale analysis of movements and habitat selection of transient animals by collecting spatial data multiple times per day and transmitting these data to a remote receiver. Using this technology, our objective was to obtain a fine-scale quantitative assessment of the spatial ecology of sub-adult cougars in the Cypress Hills to examine factors influencing dispersal in an isolated population. More specifically, we quantify sex-biased differences in ranging behavior including cumulative distance travelled during transience, and the size, distribution and habitat composition of THRs used while localizing. In order to more thoroughly examine fine-scale habitat selection of sub-adult cougars, we used conditional logistic regression and a two-stage modelling approach to estimate population-level resource selection functions (RSF) for transient and localizing behaviors. Lastly, we estimated a linear regression model, using distances between consecutive GPS relocations as the response variable, to quantify factors influencing cougar movement rates during the sub-adult life stage.

## Materials and Methods

### Study area

**Figure 1:**
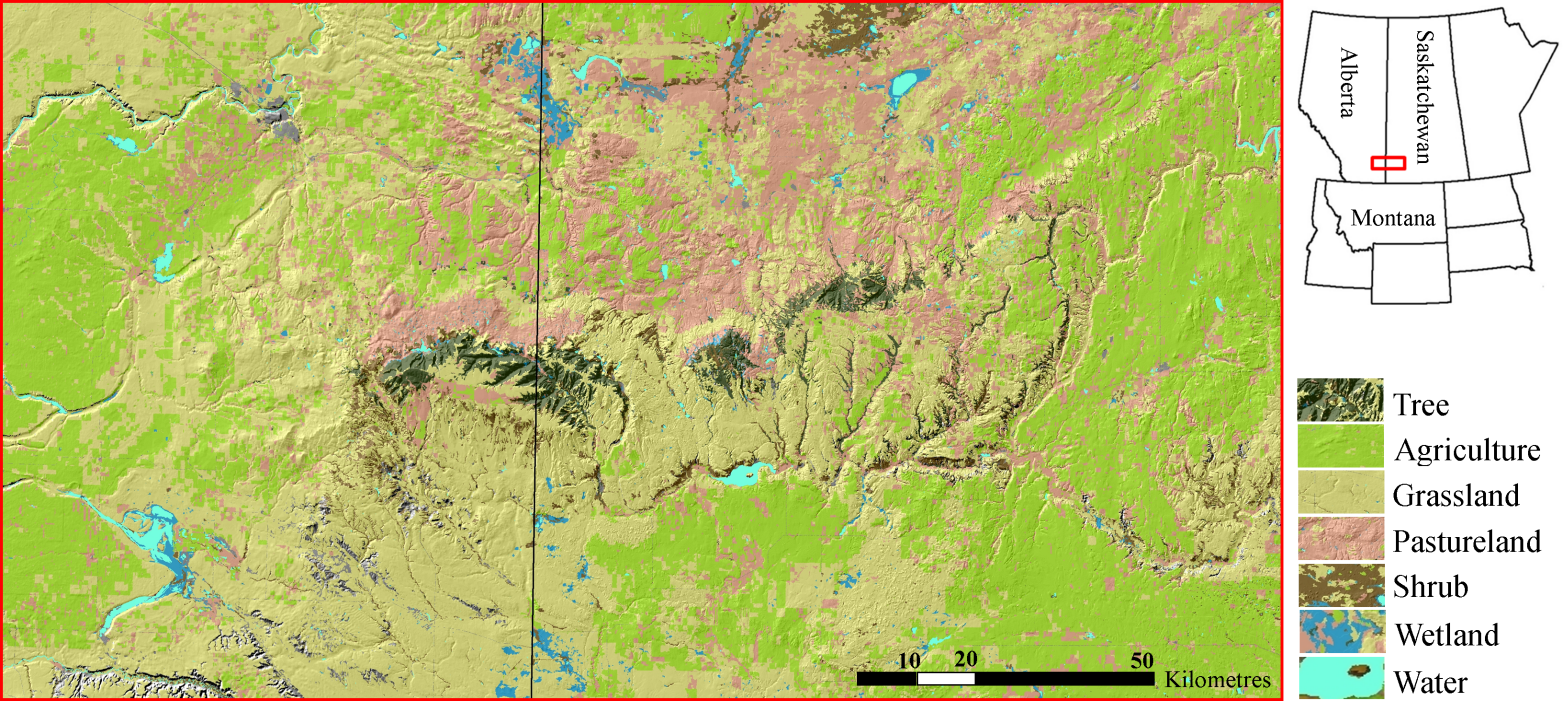
Cypress Hills study area. The Cypress Hills are an insular formation of foothills located in southeast Alberta and southwest Saskatchewan, Canada (Latitude 49.7°N, Longitude 109.5°W). The hills are distinguished by an abundance of tree cover (dark green) surrounded by a matrix dominated by mixed grasslands, pasturelands and agriculture development (shades of yellow, pink and light green, respectively). Cougars re-colonized the Cypress Hills in the late 1990s and early 2000s.

The Cypress Hills Uplands (∼2000 km^2^; Latitude 49.7°N, Longitude 109.5°W; Fig. 1) are an insular formation of foothills, located in southeastern Alberta and southwestern Saskatchewan, which rise several hundred meters above the surrounding landscape. The hills are distinguished from their surroundings by their abundance of tree cover consisting primarily of lodgepole pine (*Pinus contorta*), white spruce (*Picea glauca*) and trembling aspen (*Populus tremuloides*). The matrix surrounding the hills is an expanse of mixed grasslands, pasture lands and cultivated agricultural crops that dominate of much of midwestern North America (LaRue & Nielsen, 2008). The higher elevation of the Cypress Hills (1,234 m, Elkwater, AB) relative to the surrounding plains results in cooler summers and warmer winters. Average temperature of the Cypress Hills is 19.1 C in July and −3.3 C in January, while annual precipitation is 533.5 mm (much greater precipitation than the surrounding plains). Permission to conduct field work in this study area was provided by Saskatchewan Environment (permit no. 10FW236) and Alberta Sustainable Resource Development (permit no. 35339).

A resident population of cougars is believed to have re-colonized the Cypress Hills in the late 1990s and early 2000s coinciding with a marked increase in cougar sightings. Remote camera images of a family group in 2006 were the first evidence of breeding (Bacon, 2010). Outside the Cypress Hills, the closest known breeding populations of cougars are 200 km south in the Bear Paw Mountains in Montana and 250 km west in the Rocky Mountains of southwestern Alberta. The primary diet of cougars in the Cypress Hills is white-tailed deer (*Odocoileus virginianus*), mule deer (*O. hemionus*), porcupines (*Erethizon dorsatum*) and elk (*Cervus canadensis*) (Bacon et al., 2011; C Morrison, 2013, unpublished data).

### Cougar capture and collaring

Cougars (*n* = 7) were captured in the Cypress Hills during 2010 and 2011 with the assistance of a professional houndsmen and trained tracking hounds. All animal handling was done by trained personnel in accordance with Animal Use Protocol 568-02-11 approved by the University of Alberta Animal Care Committee. We targeted mature juveniles (>12 months old) that were still dependent on their mothers. These criteria ensured the cougars could support a GPS collar (with compressible foam padding to allow additional room for growth) and that cougars would be monitored through the onset of independence. Male cougars (*n* = 4) were fitted with Argos- (Lotek 4400s) or Iridium-based (Advanced Telemetry Systems) satellite-GPS collars that transmitted data to an email receiver every 2–6 days. Females (*n* = 3) were fitted with either a satellite-GPS collar or a standard GPS collar (Lotek 4400s) that required remote downloading of data with a hand-held command unit. All collars were programmed to take a fix every 3 h.

Cougar GPS data were analyzed during the sub-adult life stage, which is the age class typically associated with natal dispersal and/or exploratory ranging behavior. We considered the onset of sub-adulthood as the first independent foray away from the cougar’s natal range. Data collection continued until one of several criteria were met: (1) the cougar established a home range evidenced by breeding or localizing behavior for more than 12 months; (2) the cougar died; (3) the GPS-radio collar quit transmitting (e.g., dropped off or malfunctioned); or (4) the study period ended.

### Characterizing sub-adult ranging behavior

**Figure 2:**
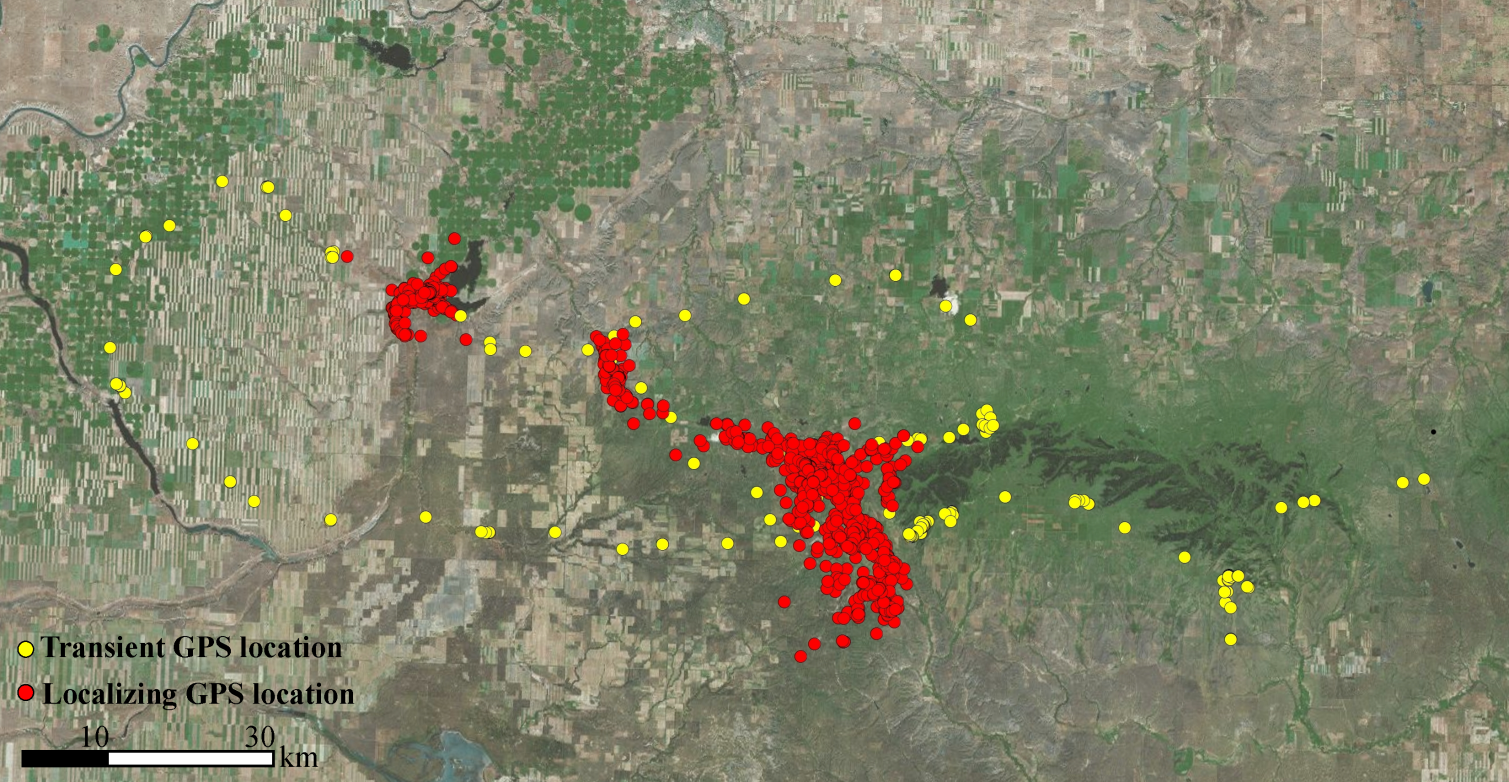
Ranging behaviors of sub-adult cougars. An example of satellite-telemetry data collected from a sub-adult male cougar in the Cypress Hill region of Alberta and Saskatchewan, Canada. Spatial data of sub-adult cougars were visually examined and classified as transient behavior (yellow points) or localizing behavior (red points). Transient behavior was characterized as unidirectional movements often into novel terrain. Localizing behavior was characterized by site fidelity to a general area (i.e., multi-directional movements) for a period exceeding 20 days, the extent of which formed the basis of temporary home ranges.

GPS locations collected during the sub-adult monitoring period were categorized into two ranging behaviors (“transience” and “localizing”) based on a visual assessment of the geographic point data (Fig. 2). Transience was characterized by unidirectional movements, often into novel terrain. Transient behavior included true natal dispersal events, where the animal never returned, and exploratory forays, where the cougar ultimately returned to its natal home range or a previous THR. Localizing was characterized by a cougar ceasing unidirectional movements and demonstrating site fidelity to an area for at least 20 days. We established 20 days as the minimum time required to define a THR based on a cougar kill rate of approximately one kill every 5–10 days (Anderson & Lindzey, 2003; Knopff et al., 2010). Thus, we made the assumption that localizing events less than 20 days could result from a feeding bout of a transient cougar; whereas localizing events exceeding 20 days encompassed enough time for a cougar to make multiple kills and could be indicative of a cougar finding sufficient resources to establish a more-permanent range.

Temporary home ranges were delineated using a 95% kernel density estimate. We calculated the area (km^2^) of each THR and the straight-line and cumulative distances travelled from the center-point of the last THR to the center-point of that cougar’s natal home range. Percent overlap of each THR with known occupied adult range was calculated to examine the spatial distribution of sub-adult THRs in relation to adults. Adult range was based on the aggregated distribution of GPS locations from nine adult cougars (*n* = 2 males, 7 females) monitored between 2008 and 2013 (Bacon, 2010; Morrison et al., 2014). GPS locations for adults exhibited considerable fidelity to the three distinct patches of treed habitat that comprise the insular Cypress Hills (C Morrison, 2013, unpublished data). Based on these data, and supported by field observations, we made the assumption that these patches are primary habitats and that the density of adult cougars is considerably higher in these patches than in the surrounding matrix. Landcover composition of sub-adult male and female THRs was quantified by calculating the average proportional composition of six primary cover types (shrub, wetland, grassland, agriculture, pasture and treed) in THRs for each sex class. Land cover was a categorical variable obtained from Land Cover for the Northern Sagebrush Steppe Initiative Area (30 m resolution; NSSI; Montana Fish, Wildlife & Parks, 2011). Finally, each sub-adult cougar was classified as a disperser or philopatric if their last known home range (THR or established) overlapped with their natal range by <5%, or >5%, respectively (Sweanor, Logan & Hornocker, 2000).

### Habitat selection

We quantified habitat selection by estimating resource selection functions (RSF) to identify landscape attributes selected by sub-adult cougars during transient and localizing behaviors. RSFs were estimated using conditional logistic regression (clogit; package = “survival”; R Core Team, 2013) to quantify the relative probability of a site being selected (Manly et al., 2002; Boyce et al., 2003). We used conditional logistic regression to pair one observed location with multiple random locations within an ecologically relevant extent (Compton, Rhymer & McCollough, 2002; Boyce et al., 2003). For species or individuals without well-defined home ranges, as is the case with sub-adult cougars, the conditional logistic design more accurately reflects the choices made during habitat selection (Compton, Rhymer & McCollough, 2002) compared to sampling availability at larger scales (e.g., level II & III, Johnson, 1980). In our case, each GPS location was buffered by 2,000 m, the approximate 90th percentile of observed 3-h step lengths for sub-adult cougars. Ten random points were generated within each buffer to sample availability. This case-control design applies a method similar to step selection functions for sampling availability in a biologically meaningful manner; available steps are paired to observed steps (i.e., share a starting location) and the end locations are drawn from the distribution of lengths and turning angles of observed steps (Fortin et al., 2005).

We used a two-stage modelling approach, whereby coefficients were first estimated for individual cougars and then averaged to obtain a population-level estimate (Nielsen et al., 2002; Sawyer et al., 2006; Fieberg et al., 2010). This method treats the individual animal as the sampling unit, instead of the GPS relocation, alleviating concerns regarding autocorrelation often associated with spatio-temporal data. Models of habitat selection by individual cougars were determined based on the combination of covariates that resulted in the lowest Akaike’s Information Criterion (AIC; Burnham & Anderson, 2002). In cases where a covariate was not included in an individual model, it received a beta value of 0 when calculating the population-level model. Due to the small number of GPS relocations during dispersal for two females, data for these two individuals were combined and a single model was estimated. Due to the overall limited number of cougars monitored, males and females were pooled when calculating population-level models.

Candidate landscape variables were chosen based on landscape characteristics deemed important to the biology of cougars. This included topographic roughness (Arundel, Mattson & Hart, 2007), elevation, distance to hydrological features (Dickson & Beier, 2002; LaRue & Nielsen, 2008), distance to open water (Arundel, Mattson & Hart, 2007), distance to paved and unpaved roads (Dickson, Jenness & Beier, 2005; Morrison et al., 2014), and land cover (Dickson, Jenness & Beier, 2005). Quadratic terms were included for all continuous variables to assess non-linearity. Continuous variables were also tested for collinearity using a threshold Pearson correlation coefficient of |*r*| ≥ 0.7, above which the two correlated variables were restricted from entering the same model. Land cover was condensed into six primary cover types which were shrub, wetland, grassland, agriculture, pasture and tree. Tree cover included deciduous, conifer and mixed woods and was used as a reference category in the models because it is the dominant cover type of the Cypress Hills Uplands and we were interested in examining selection related to more open habitats. Other land cover classes identified by NSSI but not included in the six primary categories were accounted for by other habitat covariates (e.g., water) or were masked from being sampled because they comprised a small fraction of habitat types available and were rarely, or never, encountered by cougars during this study. All covariates were standardized for statistical analysis.

### Movement

In addition to assessing the habitat characteristics that influenced cougar space-use, we also examined how these landscape variables affected movement rates of sub-adult cougars. Step length, defined as the distance between consecutive GPS relocations (Turchin, 1998), is a measure of speed that can be used to quantify cougar response to multiple habitat variables. Linear regression was used to estimate a population-level movement model using the same two-staged approach described for the habitat-selection models. Step length was right skewed so a natural log transformation was performed prior to statistical analysis. Only steps that linked two consecutive fixes were included in the analysis to ensure all steps were equal in duration.

Candidate explanatory variables in the step-length model included all the same covariates available in the RSF models but used linear unit measures. Specifically, for each step we calculated the length-weighted mean (using “isectlinerst” tool in Geospatial Modelling Environment; Beyer, 2012) for topographic roughness, elevation, distance to hydrological features, distance to open water, and distance to paved and unpaved roads. To characterize cover types we calculated the proportional composition of each cover type that a step intersected. To eliminate collinearity associated with proportional composition (i.e., all proportions sum to one), we excluded tree cover from the model to serve as a reference category for land cover categories. All covariates were standardized for statistical analysis.

In addition to habitat covariates, we also included day-period as a categorical variable by binning steps into either day (steps commencing at 900, 1200 and 1500 h), crepuscular (steps commencing at 600 and 1800 h), or night (steps commencing at 0300, 2100 and 2400 h) period. Also, instead of estimating separate models for transience and localizing, we included ranging behavior as a categorical variable (*ranging*) to test whether cougars moved faster during transience while accounting for the effects of other habitat characteristics. Finally, we included interaction terms to test for diel shifts in activity associated with land cover or ranging behavior.

## Results

### Cougar captures and GPS data

In total, seven sub-adult cougars, including four males and three females were monitored with GPS-radio collars. Six cougars were collared while still dependent on their mothers. One female was collared as an independent sub-adult and was estimated to be 18 months at time of capture. After independence, sub-adult cougars were monitored for an average of 240 days (min = 88, max = 411) during which an average of 1,481 GPS relocations (min = 385, max = 2,694) were collected per individual. Average fix success of sub-adult cougars was 82.1% (min = 79.1%, max = 92.7%).

### Characterizing sub-adult ranging behavior

**Figure 3:**
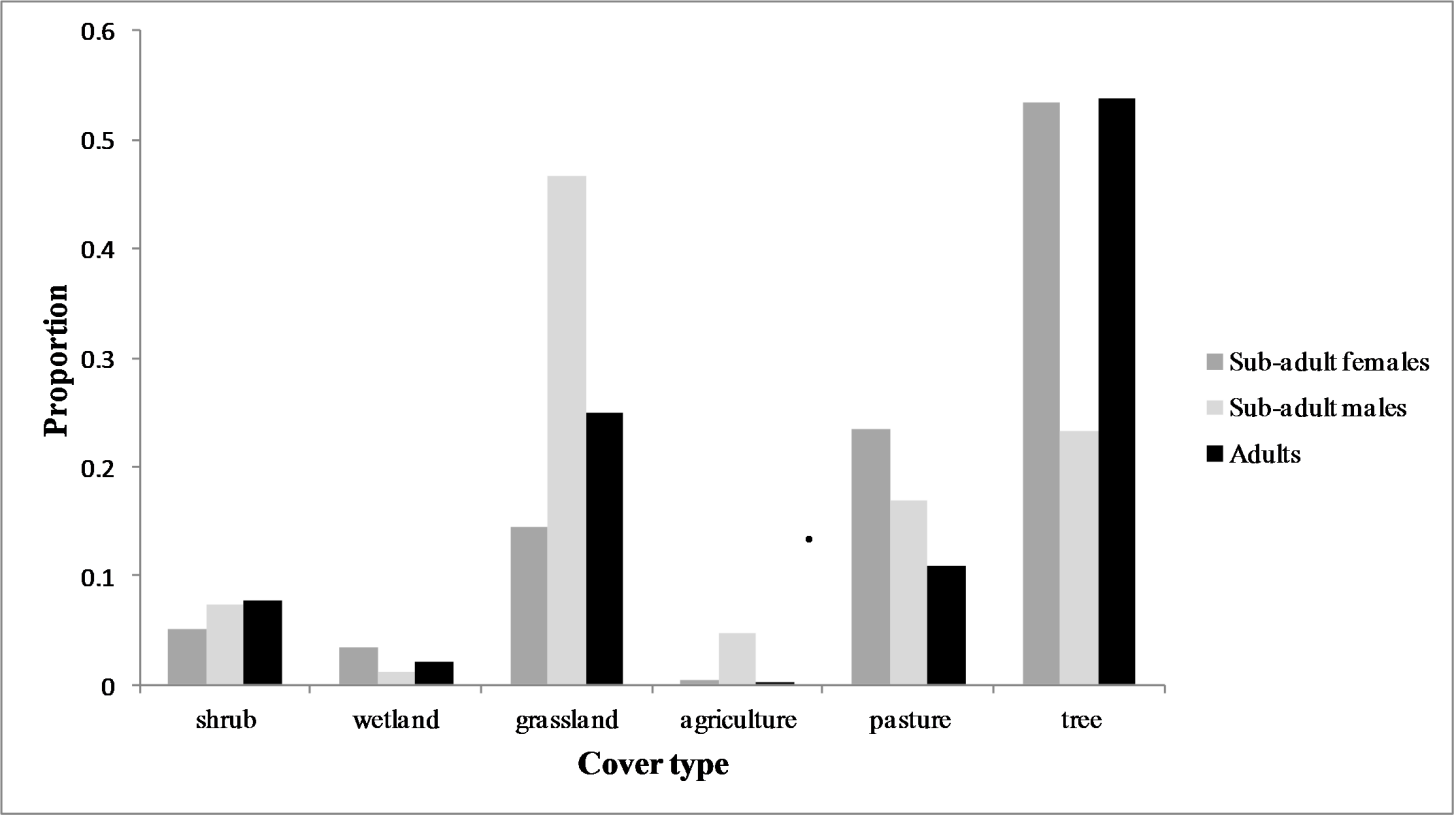
Land cover composition of temporary home ranges. Average land cover composition of temporary home ranges of male (*n* = 4) and female (*n* = 3) sub-adult cougars and of aggregated ranges of adult cougars (*n* = 9) observed in the Cypress Hills, Alberta and Saskatchewan, Canada, between 2009–2012.

**Figure 4:**
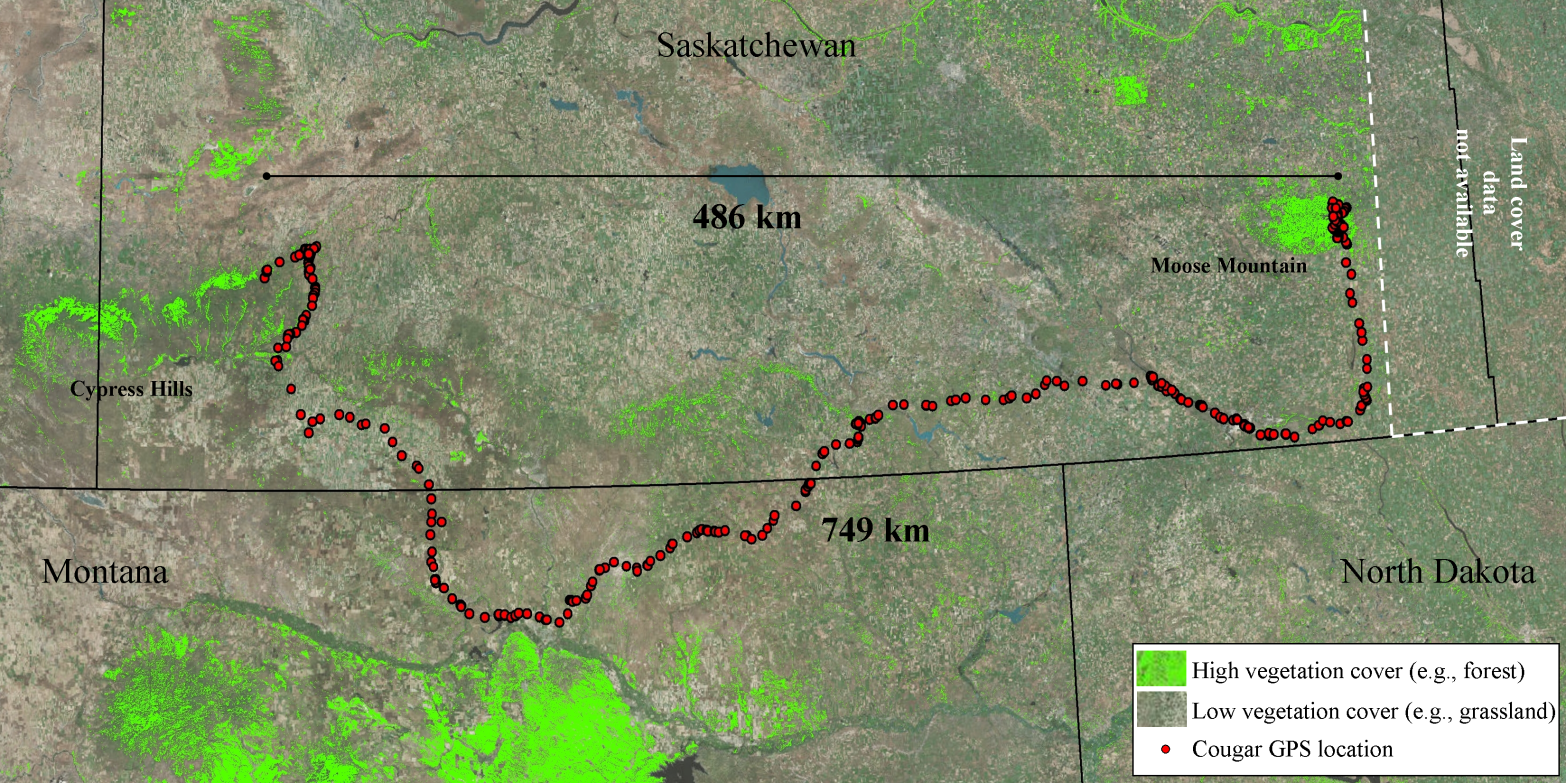
Long-distance dispersal of a sub-adult male cougar. Dispersal route of a sub-adult male cougar (M7) that was fitted with a GPS-satellite collar in the Cypress Hills (Aug 2011) in southwest Saskatchewan, Canada. Cougar M7 travelled a cumulative distance exceeding 749 km over 100 days (13 Feb 2012–22 May 2012) before localizing in the vicinity of Moose Mountain Provincial Park in southeast Saskatchewan. Pixels highlighted in green represent mid to high vegetation cover (e.g., forest and shrub). The remainder of the pixels (no highlights) overlaying the satellite image are classified as open cover (e.g., agriculture, grasslands and pasturelands).

All sub-adult cougars displayed movement bouts characteristic of transience and localizing. During transience, six cougars made exploratory forays into the surrounding grassland-dominated matrix. However, all but one cougar eventually returned to the vicinity of the Cypress Hills. We delineated seven THRs used by males and four THRs used by females (Table 1). The average female THR was 72.6 km^2^ and overlapped with observed adult range by 79.8%. Male THRs averaged 172.4 km^2^ with 16.1% overlap of adult range. Average land-cover composition of THRs used by males included significantly more grassland (*P* < 0.05) and significantly less forested cover (*P* < 0.05) than sub-adult females. Otherwise there was no significant difference (*P* > 0.05) observed between male and female THRs in the remaining land cover categories (Fig. 3). Average distance from the center point of the last THR to the center point of natal ranges was 13.7 km for females and 165.3 km for males. Average cumulative distance covered during transience was 132.1 km for females and 364.3 km for males (Table 1). One female remained philopatric to her natal range while one female dispersed. The third female was captured post-independence so no conclusions can be made in regards to her natal range. All four male cougars dispersed from their natal ranges. One male dispersed from the study area completely and traversed 749.3 km (487.7 km straight-line distance) covering portions of northeast Montana and much of southern Saskatchewan before establishing his first THR near Moose Mountain Provincial Park in southeast Saskatchewan (Latitude 49.8°N, Longitude 102.4°W; Fig. 4).

**Table 1:** Dispersal, movement and space-use of sub-adult cougars. Dispersal, movement and space-use statistics for sub-adult cougars in the Cypress Hills (southeast Alberta, southwest Saskatchewan) monitored between 2010 and 2012. Straight-line distance and cumulative distance were calculated from the center point of the last temporary home range (THR) to the center point of the cougar’s natal range. If multiple THRs were used by individual cougars they are denoted by cougar ID and then chronologically by letter code A or B.

Cougar	Dispersal status	Straight line distance (km)	Cumulative distance (km)	Temporary home range	THR area (km^2^)	THR overlap with known adult range
F1	Unk	N/A	N/A	F1_THR_A	47.4	90.04%
				F1_THR_B	71.4	78.58%
F4	Dispersed	24.5	209.6	F4_THR	95.9	66.02%
F5	Philopatric	2.9	54.6	F5_THR	75.6	84.56%
**Female average**		**13.7**	**132.1**		**72.6**	**79.8%**
M2	Dispersed	40.3	200.9	M2_THR_A	229.8	52.11%
				M2_THR_B	246.9	20.00%
M3	Dispersed	108.7	271.7	M3_THR_A	21.8	0.00%
				M3_THR_B	304.3	11.50%
M6	Dispersed	25.6	235.3	M6_THR_A	79.3	17.21%
				M6_THR_B	225.9	11.66%
M7	Dispersed	486.7	749.3	M7_THR	98.8	0.00%
**Male average**		**165.3**	**364.3**		**172.4**	**16.1%**

### Habitat use and selection

**Figure 5:**
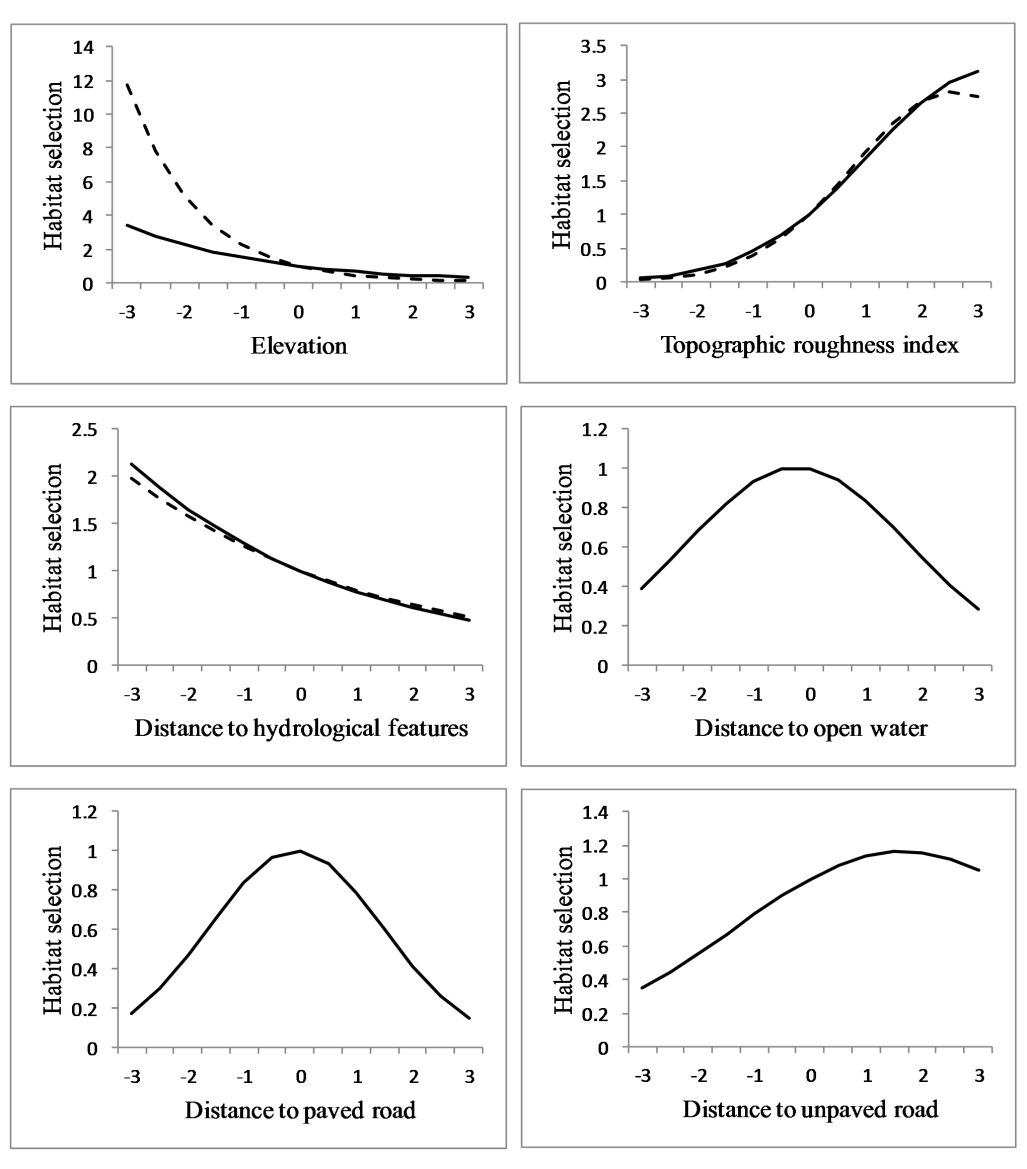
Habitat selection of sub-adult cougars. Habitat selection (estimated using resource selection functions; RSF) of sub-adult cougars in the Cypress Hills (Alberta and Saskatchewan, Canada) plotted over standardized range of habitat covariates (up to ±3 standard deviations). Note the differences in scale of the vertical axes. Response curves are plotted only for covariates that had a strong population-level response (i.e., 90% confidence interval of beta coefficients did not overlap zero). Dashed and solid lines indicate RSF estimates from transient and localizing models respectively.

At the population level, cougars selected rough terrain and proximity to hydrological features during both ranging behaviors (Table 2). Regression coefficients for elevation were negative during both ranging behaviors (Table 2) but with a markedly steeper slope during transience (Fig. 5). Proximity to roads did not greatly affect cougar habitat selection during transience, although cougars avoided areas in close proximity to paved and unpaved roads while localizing in THRs (Table 2). This avoidance became less pronounced as distance to both road types increased (Fig. 5). Response to paved roads was non-linear and the relative probability of selection began to decrease when distance to paved roads exceeded ∼12.5 km (Fig. 5). Distance to open water had little effect on habitat selection during transience, although cougars showed a non-linear response while localizing (Table 2, Fig. 5). Cougars avoided all land cover classes when compared to forests during both ranging behaviors (Table 2). This avoidance was greatest for agriculture followed by grasslands and then pasturelands. Cougars used wetlands during transience and shrubland while localizing at the same rate as the reference habitat of forests.

**Table 2:** Population-level habitat selection models for sub-adult cougars during transient and localizing behavior. Standardized population-level coefficients (*β*) and 90% confidence intervals (CI) for sub-adult cougars in the Cypress Hills, Alberta and Saskatchewan, Canada. Population coefficients were calculated by averaging individual-level betas obtained using conditional logistic regression models. Land cover was a categorical variable and used tree as a reference category.

	Transience				Localizing			
		90% CI				90% CI		
Covariate	*β*	Upper	Lower		*β*	Upper	Lower	
*elevation*	−0.821	−0.3343	−1.3082	^*^	−0.406	−0.067	−0.746	^*^
*elevation* ^2^	0.382	0.7723	−0.0093		0.278	0.935	−0.378	
*topographic roughness*	0.802	1.1315	0.4717	^*^	0.700	1.295	0.105	^*^
*topographic roughness* ^2^	−0.155	−0.0624	−0.2471	^*^	−0.107	−0.076	−0.137	^*^
*dist. to hydro. feature*	−0.227	−0.1202	−0.3329	^*^	−0.251	−0.120	−0.381	^*^
*dist. to hydro. feature* ^2^	0.109	0.2752	−0.0571		0.089	0.218	−0.040	
*dist. to open water*	N/A	N/A	N/A		−0.064	0.236	−0.364	
*dist. to open water* ^2^	N/A	N/A	N/A		−0.123	−0.104	−0.142	^*^
*dist. to paved road*	−0.080	0.2996	−0.4588		−0.028	0.249	−0.305	
*dist. to paved road* ^2^	0.073	0.1920	−0.0468		−0.205	−0.145	−0.265	^*^
*dist. to unpaved road*	0.201	0.5595	−0.1580		0.184	0.339	0.030	^*^
*dist. to unpaved road* ^2^	0.085	0.1808	−0.0105		−0.056	−0.041	−0.071	^*^
*cover*								
*shrub*	−1.080	−0.4920	−1.6688	^*^	−0.692	0.053	−1.437	
*wetland*	−0.312	0.2852	−0.9087		−0.938	−0.137	−1.738	^*^
*grassland*	−1.631	−0.9240	−2.3388	^*^	−1.646	−1.537	−1.755	^*^
*agriculture*	−1.831	−0.9607	−2.7005	^*^	−2.474	−2.342	−2.607	^*^
*pasture*	−1.096	−0.6954	−1.4965	^*^	−1.669	−1.322	−2.016	^*^

**Notes.**

* Indicate covariates that have a strong population-level effect based on 90% confidence intervals that do not overlap 0.

### Movement

**Figure 6:**
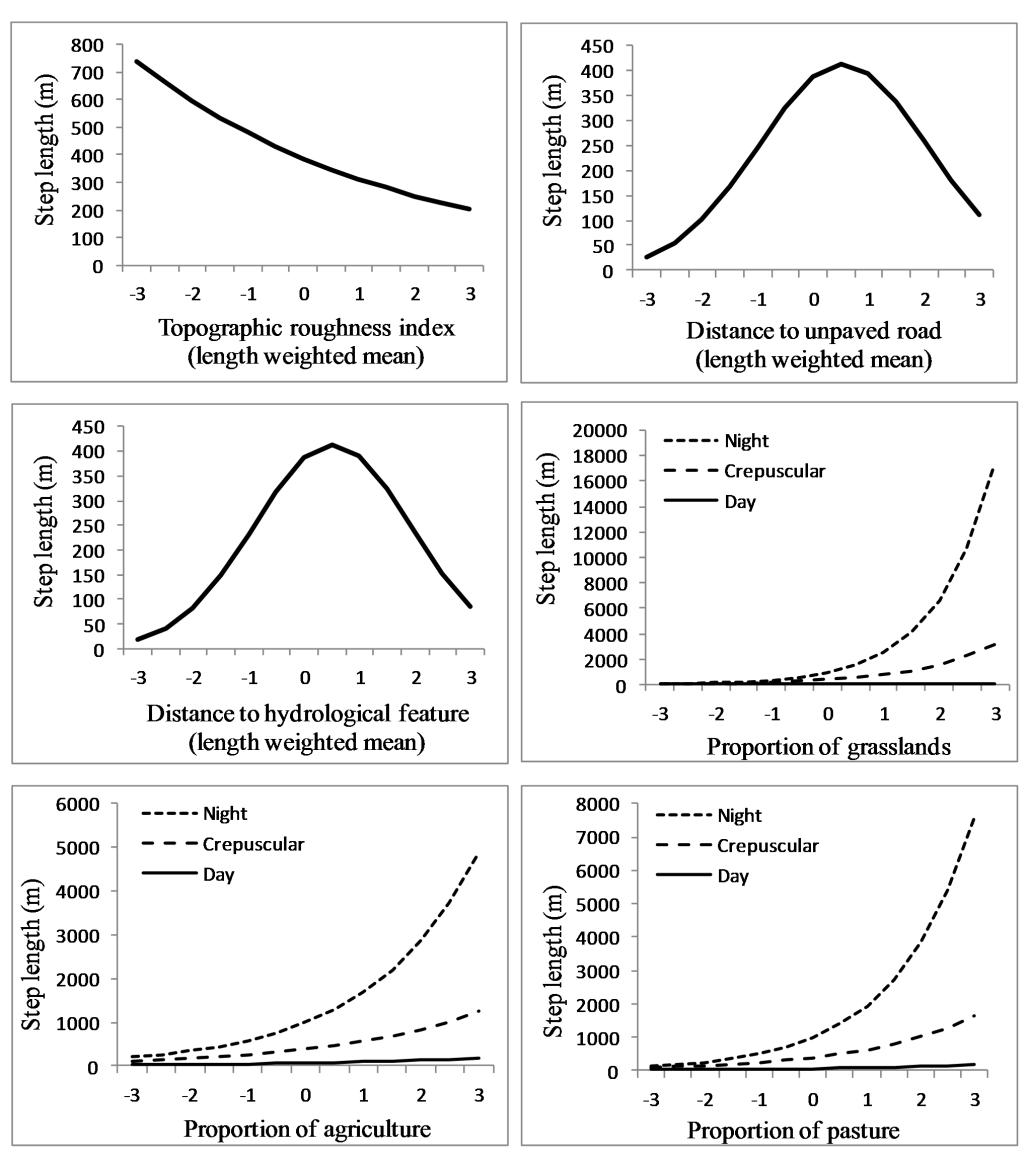
Movement rates of sub-adult cougars. Step length (calculated as distance between sequential 3-h GPS relocations) estimates for sub-adult cougars in the Cypress Hills (Alberta and Saskatchewan, Canada) plotted over standardized range of habitat covariates (up to ±3 standard deviations). Exponents of natural-log transformed step length are presented for ease of interpretation. Note the differences in scale of the vertical axes. Response curves are plotted only for covariates that had a strong population-level response (i.e., 90% confidence interval of beta coefficients did not overlap zero).

We analyzed an average of 1,344 steps per sub-adult cougar (min = 261, max = 2,309). The final population-level model included all candidate covariates but not all appeared to have a great effect on step length (Table 3). Specifically, length-weighted means for elevation, proximity to paved roads and proximity to open water did not appear to influence cougar step length (Table 3). Cougar response to roads and hydrological features was non-linear; step length (i.e., movement rates) increased as distance from hydrological features and unpaved roads increased, but eventually decreased as distance from these features became large (Fig. 6). Cougar movements slowed as topographic roughness increased (Table 3 and Fig. 6).

**Table 3:** Population-level movement model for sub-adult cougars. Step-length model results for sub-adult cougars in the Cypress Hills (Alberta and Saskatchewan, Canada) estimated using linear regression. Standardized population-level coefficients (*β*) and 90% confidence intervals (CI) are reported. Step length was natural log transformed for statistical analysis. Day_period used crepuscular as a reference category. Ranging used transience as a reference category. Tree cover was excluded from the model to act as a reference category for proportional cover.

		90% CI		
Covariate	*β*	Upper	Lower	
*constant*	5.958	6.994	4.923	^*^
*elevation*	0.656	1.605	−0.293	
*elevation* ^2^	0.092	0.546	−0.361	
*topographic roughness index*	−0.215	−0.104	−0.327	^*^
*topographic roughness index* ^2^	−0.052	0.006	−0.111	
*dist. to unpaved road*	0.236	0.308	0.164	^*^
*dist. to unpaved road* ^2^	−0.217	−0.065	−0.369	^*^
*dist. to paved road*	−0.647	0.292	−1.586	
*dist. to paved road* ^2^	−0.229	0.221	−0.678	
*dist. to hydrological feature*	0.260	0.423	0.098	^*^
*dist. to hydrological feature* ^2^	−0.254	−0.077	−0.431	^*^
*dist. to open water*	−0.017	0.141	−0.176	
*dist. to open water* ^2^	−0.039	0.046	−0.123	
*day_period*				
*day*	−1.790	−1.362	−2.218	^*^
*night*	0.938	1.589	0.287	^*^
*Proportional cover*				
*shrub*	0.182	0.540	−0.177	
*wetland*	0.073	0.276	−0.130	
*grassland*	0.702	0.948	0.457	^*^
*agriculture*	0.387	0.604	0.171	^*^
*pasture*	0.481	0.828	0.133	^*^
*ranging*				
*localizing*	−0.739	0.696	−2.173	
*day × shrub*	−0.193	0.186	−0.573	
*night × shrub*	−0.050	0.186	−0.287	
*day × wet*	−0.050	0.078	−0.178	
*night × wet*	0.047	0.108	−0.014	
*day × grassland*	−0.460	−0.249	−0.670	^*^
*night × grassland*	0.252	0.468	0.035	^*^
*day × agriculture*	−0.040	0.002	−0.082	
*night × agriculture*	0.144	0.297	−0.009	
*day × pasture*	−0.154	0.136	−0.444	
*night × pasture*	0.197	0.343	0.052	^*^
*day × localizing*	0.294	0.910	−0.322	
*night × localizing*	−1.056	−0.179	−1.932	^*^

**Notes.**

* Indicate covariates that have a strong population-level effect based on 90% confidence intervals that do not overlap 0.

Proportion of shrubland and wetland did not influence cougar step length in relation to proportion of tree cover (Table 3). Cougar step length increased in grasslands, agriculture and pasturelands and this pace quickened in these habitats at night (Table 3 and Fig. 6). In contrast, cougars had a shortened step length in grasslands during the day. Cougars moved greater distances at night during transient behavior compared to localizing behavior, while day-time step lengths during both ranging behaviors were comparable (Table 3).

## Discussion

Sub-adult cougars in the Cypress Hills demonstrated male-biased dispersal behavior which is common among polygynous mammals (Johnson, 1986) and reported in other cougar dispersal studies (Sweanor, Logan & Hornocker, 2000; Thompson & Jenks, 2010; Maehr et al., 2002). All males successfully dispersed from their natal range, while female dispersal varied. Average distance traversed by males during transience was also greater than females. As expected, total distances covered during transience for both sexes far exceeded the net displacement from the cougar’s natal range to its last known location (Table 1). Stoner et al. (2008) observed a similar discrepancy in measures of displacement for an exceptional young female that travelled 1,341 km during dispersal but was relocated only 357 km from her point of first capture. Straight-line distance is an often reported metric in dispersal studies of terrestrial mammals (Santini et al., 2013; Poole, 1997; Sweanor, Logan & Hornocker, 2000; Thompson & Jenks, 2010), but this fails to account for the complex movements of individuals between start and terminus locations. Our findings exemplify how our understanding of the spatial ecology of transient individuals will continue to improve with advancements in GPS-radio collar technology.

Only one marked cougar emigrated from the Cypress Hills Uplands during our study period. The remaining six sub-adult cougars made long distance exploratory forays into the surrounding grassland-dominated matrix, but eventually returned to the vicinity of the Cypress Hills. Thompson & Jenks (2010) hypothesize that the mate-procurement hypothesis is the leading mechanism driving long-distance dispersal in sub-adult males. Sub-adult males in the Cypress Hills, who did not ultimately disperse away from the study area, used THRs that overlapped minimally with observed adult range. Due to the insular nature of tree cover in the Cypress Hills, this avoidance tactic resulted in sub-adult males occupying THRs dominated by grasslands, a cover type that was less preferred to forested cover by males and females alike (Fig. 3). The tendency for sub-adult males to localize in less-suitable habitats on the periphery of adult range limits direct competition with resident males and has been observed in other large carnivores (e.g., tigers, Smith, 1993). On the other hand, it is unclear how this avoidance strategy provides access to mating opportunities which is of obvious importance to the mate procurement hypothesis. Additional long-term monitoring would be required to examine if these satellite males could eventually compete with dominant males in order to recruit into the population or if they would be required to emigrate to seek breeding opportunities elsewhere, as was observed in the Black Hills (Thompson & Jenks, 2010).

In contrast with sub-adult males, sub-adult females used THRs that overlapped considerably with known adult ranges with two of the three females eventually showing evidence of breeding. These data indicate sub-adult females were successfully recruited into the Cypress Hills population. True range expansion is dictated by the presence of females (Thompson & Jenks, 2010). As a result, male-biased dispersal patterns, in terms of distance and rates, still could be a major factor limiting the pace and extent of cougar range expansion and re-colonization. However, the high rate of long-distance exploratory forays observed in both sexes could indicate high levels of competition for resources in this insular landscape. Under the hypothesis that female dispersal is partially density dependent (Logan & Sweanor, 2001), geographically isolated populations of cougars could be expected to produce a greater number of female dispersers in which case these populations will serve as important stepping stones promoting, and potentially expediting, re-colonization into suitable areas further east.

The most notable dispersal event we documented was that of a male cougar (M7) who successfully traversed 749 km over 100 days, from 13 February 2012 to 22 May 2012 (Fig. 4). Cougar M7 maintained fast-paced directional movements until he arrived in the vicinity of Moose Mountain Provincial Park—the first large suitable habitat patch he encountered. Here he localized for 27 days until his collar quit transmitting. Although there is no known breeding population of cougars in the Moose Mountain area, the presence of at least one other cougar was confirmed during the winter of 2012 with remote cameras (J Karst, Saskatchewan Fish and Wildlife, pers. comm., 2012). The presence of this unknown cougar provides one more confirmation that cougars are reaching isolated patches of habitat several hundred kilometres from known source populations. Indeed, the capacity to disperse long distances has been well documented in cougars (Stoner et al., 2008; Thompson & Jenks, 2005) and several other species of large carnivore including wolf (*Canis lupus*; Andersen et al., 2015), lynx (*Lynx canadensis*; Poole, 1997) and leopard (*Panthera pardus*; Fattebert et al., 2013). By examining the fine-scale movements and selection of habitats of sub-adult cougars emanating from a geographically isolated population, our results, exemplified by this account of M7’s dispersal, provide insights into how long-distance dispersals occur.

By examining habitat selection of sub-adult cougars during different ranging behaviors, it is apparent that certain habitat characteristics have variable importance to cougars depending on whether they are localizing in a THR or traversing relatively novel landscapes. For example, cougars selected for lower elevations during both ranging behaviors but the slope of the response curves differed dramatically. Localizing cougars demonstrated a gradual response to elevation which could be explained by the sub-adults’ tendencies, especially males, to avoid the forested uplands dominated by adult cougars. During transience, the relative probability of use dropped quickly with increases in elevation. This could indicate low lying features are important travel corridors for cougars during dispersal, perhaps providing some form of lateral cover to offset the lack of vegetative cover in more open habitat types. In California, cougars were observed using canyon bottoms as preferred travel routes (Dickson & Beier, 2007).

Differences in habitat selection between ranging behaviors was also observed in response to open water and roads. Localizing sub-adult cougars had a strong (albeit non-linear) response to distance to open water whereas open water was not included in the most-supported transient models for any individual cougar and thus had no effect at the population-level. This difference could result from open water representing a localized resource that might be less important to a travelling cougar. During localizing behavior cougars avoided areas in proximity to both paved and unpaved roads but appeared indifferent to these features during transience. Morrison et al. (2014) documented temporal shifts in cougar space-use around roads and trails in response to fluctuating levels of human activity. So it is possible that localizing sub-adult cougars become accustomed to human activity levels on roads within their THRs and adjust their space-use accordingly. During transience however, cougars moved greater distances at night and therefore were more likely to encounter roads when traffic levels were reduced. Cougars in southwest Alberta appeared ambivalent to roads at night that received <1 vehicle/hour (Banfield, 2012). Although proximity to roads may not influence fine-scale cougar habitat selection during dispersal, it logically increases the risk of human-caused mortality. As such, cougars would still benefit from corridors with lower densities of roads and human development (LaRue & Nielsen, 2008).

Proximity to hydrological features was a selected habitat characteristic during both ranging behaviors which is consistent with other findings that cougars select for riparian areas (Dickson & Beier, 2002; Dickson, Jenness & Beier, 2005). Historically cougars were documented along rivers and in riparian areas throughout the Midwest, but land-use transformations over the past century might have affected the ability of these features to support populations or even to serve as corridors (Laundre, 2012). However, models by LaRue & Nielsen (2008) predicted high stream densities within dispersal corridors. Our results indicated that hydrological features were important for animals traversing relatively open landscapes. In many instances, small hydrological features likely supported riparian habitats that were not resolved at a land-cover resolution of 30 m, but which provided fine-scale linear corridors for cougars. These riparian habitats also likely provided access to water and prey for both localizing and transient cougars.

**Figure 7:**
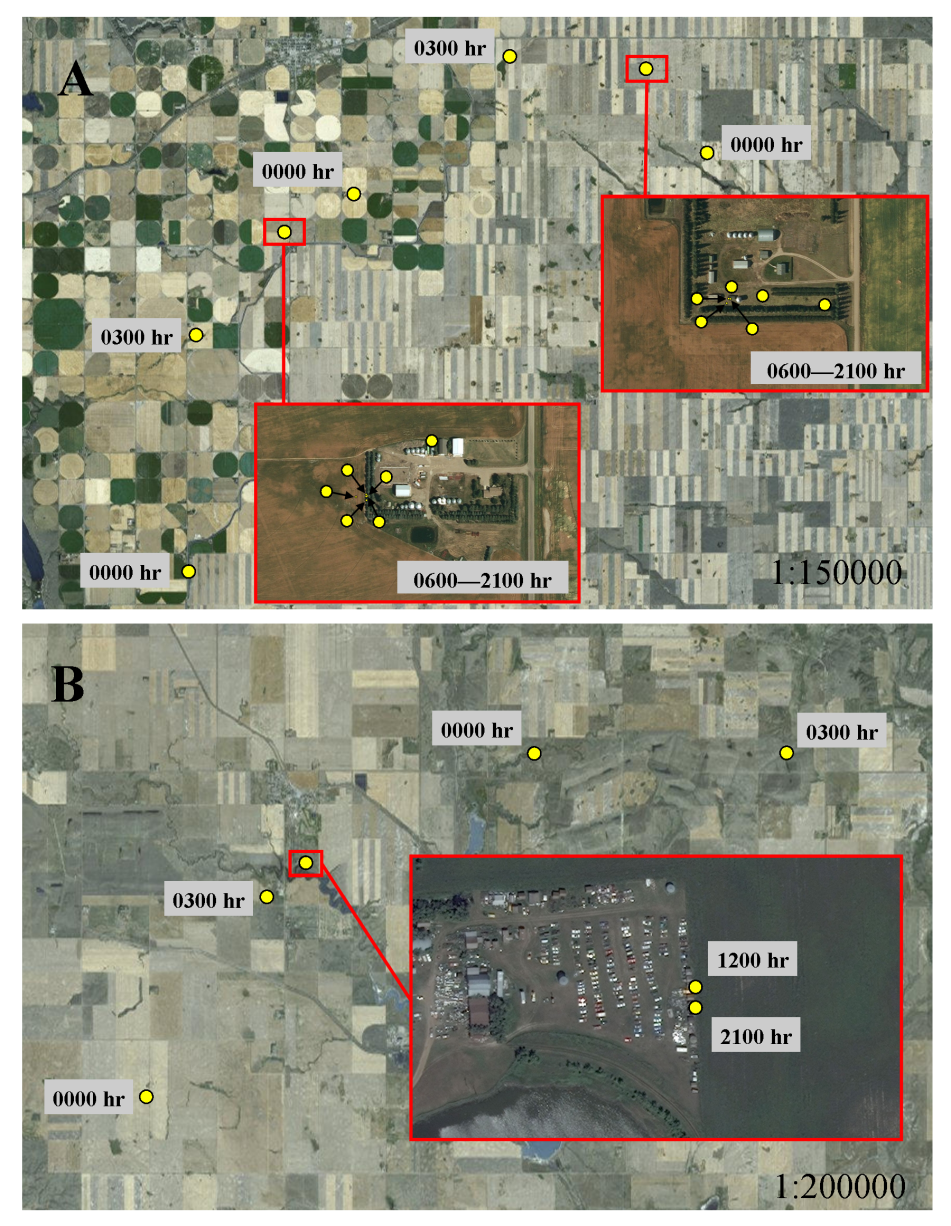
Movement behavior of sub-adult cougars during dispersal. Movement model results indicated cougars used face-paced movements at night and had shortened daytime movements when traversing open habitats. Illustrations A and B exemplify these model results and highlight the micro-scale cover, sometimes associated with human infrastructure, that cougars presumably sought for daytime resting locations. In both examples, cougars used directional face-paced movements at night to traverse large expanses of agricultural development. Cougar M3 (A) took cover at rural residences (located ∼75 km from the Cypress Hills) during the day. Cougar M7 (B) recorded daytime fixes at an abandoned-car yard (located ∼250 km from the Cypress Hills); fixes scheduled for 0600 h, 0900 h, 1500 h and 1800 h were unsuccessful presumably because the cougar was sheltered under a vehicle.

In addition to selecting habitat characteristics that likely facilitated movement through novel terrain, sub-adult cougars moved considerably farther at night during transience than while localizing, indicating cougars were also relying on the cover of darkness while traversing unfamiliar landscapes. Also, sub-adult cougars generally shifted to faster-paced nocturnal movements while traversing less-suitable habitats during both ranging behaviors. This was particularly evident when traversing agriculture, pasture and grasslands, but was less pronounced when crossing wetlands and shrublands, which likely afforded greater vegetative cover. Likewise, dispersing cougars in a range and basin landscape in New Mexico also used fast-paced directional movements to cross matrices of unsuitable habitat (Sweanor, Logan & Hornocker, 2000). In contrast to long-distance nocturnal movements in open habitats, cougars moved considerably less during the day in grasslands, presumably being “pinned-down” in any available cover. Indeed, using satellite imagery, a qualitative examination of cougar day-time resting locations in open habitats often revealed some landscape feature, at times man-made structures, which likely provided sufficient cover to hide for the day (Fig. 7). Thus, by adopting a leap-frog pattern of movement between available cover, interspersed with fast-paced long distances movements at night, even large open expanses offered some level of permeability for sub-adult cougars.

## Conclusions

The technological and analytical methods we utilized have applications for any species of wildlife that is prone to long distance dispersal or is far ranging by nature, and is capable of supporting a satellite-GPS collar. Although we were limited in the number of sub-adult cougars we sampled, our study focused on a relatively small and isolated population of cougars; therefore our sample likely represents a reasonable proportion of the sub-adult cougars present during this study. We also collected a large sample of GPS relocations and step segments for individual cougars which contributed to our population-level estimates using a two-stage modelling approach. Thus, the results of this study provide valuable insights into the fine-scale spatial ecology of a polygnynous carnivore which have typically been examined at much coarser scales due to technological limitations.

If cougars continue to re-colonize the prairie-dominated landscape of the Midwest, their distribution will likely be spatially structured as geographically isolated sub-populations separated by a matrix of less-suitable habitats. Dispersal is fundamental for re-colonizing former habitat patches/range and maintaining the long-term presence and genetic viability of metapopulations (Sweanor, Logan & Hornocker, 2000; Quigley & Hornocker, 2010). As such, understanding how cougars traverse the Midwest matrix and how re-established populations can serve as stepping stones for continued range expansion and gene-flow among isolated populations has conservation implications for a number of large carnivores facing similar challenges.

By co-examining space-use and movement of sub-adult cougars in an isolated population, our results provide insight into how cougar range expansion is progressing in the North American Midwest. Managers should realize that although certain habitat characteristics are preferred, cougars will not restrict their movements to these features. Instead, cougars will adopt faster, nocturnal movements to effectively limit their residency and exposure in these less-suitable landscapes. In doing so, cougars can successfully disperse several hundred kilometres across prairie-dominated landscapes in search of resources and mates.

## Supplemental Information

10.7717/peerj.1118/supp-1 Supplemental Information 1 Sub-adult cougar satellite-telemetry data Global positioning system (GPS) data for sub-adult cougars collected between 2010–2012 in the Cypress Hills region of Alberta and Saskatchewan, Canada. Data provide are: individual cougar identification (*coug_ID*); local date (*lmt_date*); local time (*hour*); geographic coordinates (*latitude* and *longitude*; *UTMx* and *UTMy*); *valid_step* (binary; 1 = starting point of a step connecting consecutive 3-h GPS relocations, 0 = false); *step_length* (meters) and ranging behavior (*ranging*; local = localizing, trans = transient). These raw data formed the basis for all spatial analyses presented in the manuscript.

## Additional Information and Declarations

### Field Study Permissions

The following information was supplied relating to field study approvals (i.e., approving body and any reference numbers): Saskatchewan Environment permit no. 10FW236 Alberta Sustainable Resource Development permit no. 35339.
