# Supplementary material for: Space-use, movement and dispersal of sub-adult cougars in a geographically isolated population
Source: PeerJ. 2015 Aug 6;3:e1118. doi: 10.7717/peerj.1118 (PMC4540023; doi:10.7717/peerj.1118)
Supplement: Supplemental Information 1 — Global positioning system (GPS) data for sub-adult cougars collected between 2010–2012 in the Cypress Hills region of Alberta and Saskatchewan, Canada. Data provide are: individual cougar identification (coug_ID); local date (lmt_date); local time (hour); geographic coordinates (latitude and longitude; UTMx and UTMy); valid_step (binary; 1 = starting point of a step connecting consecutive 3-h GPS relocations, 0 = false); step_length (meters) and ranging behavior (ranging; local = localizing, trans = transient). These raw data formed the basis for all spatial analyses presented in the manuscript. [file peerj-03-1118-s001.pdf]

| coug_ID | lmt_date | hour | latitude | longitude | UTMx | UTMy | valid_step | step_length | ranging |
|---|---|---|---|---|---|---|---|---|---|
| F1 | 02/16/2010 0:00 | 0 | 49.682008 | -109.517319 | 606957 | 5504331 | 1 | 17.70 | Local |
| F1 | 02/16/2010 0:00 | 3 | 49.681858 | -109.517237 | 606963 | 5504315 | 1 | 1926.17 | Local |
| F1 | 02/16/2010 0:00 | 6 | 49.665296 | -109.509415 | 607564 | 5502484 | 1 | 413.86 | Local |
| F1 | 02/16/2010 0:00 | 9 | 49.666910 | -109.514582 | 607187 | 5502657 | 0 | 416.12 | Local |
| F1 | 02/16/2010 0:00 | 21 | 49.665291 | -109.509383 | 607566 | 5502484 | 1 | 38.00 | Local |
| F1 | 02/17/2010 0:00 | 0 | 49.665006 | -109.509673 | 607546 | 5502452 | 1 | 28.62 | Local |
| F1 | 02/17/2010 0:00 | 3 | 49.665236 | -109.509496 | 607558 | 5502478 | 1 | 2.36 | Local |
| F1 | 02/17/2010 0:00 | 6 | 49.665249 | -109.509523 | 607556 | 5502479 | 1 | 1890.23 | Local |
| F1 | 02/17/2010 0:00 | 9 | 49.681365 | -109.517860 | 606919 | 5504259 | 1 | 4.39 | Local |
| F1 | 02/17/2010 0:00 | 12 | 49.681404 | -109.517858 | 606919 | 5504263 | 1 | 3.55 | Local |
| F1 | 02/17/2010 0:00 | 15 | 49.681405 | -109.517809 | 606923 | 5504263 | 1 | 16.91 | Local |
| F1 | 02/17/2010 0:00 | 18 | 49.681555 | -109.517851 | 606919 | 5504280 | 1 | 1907.93 | Local |
| F1 | 02/17/2010 0:00 | 21 | 49.665290 | -109.509430 | 607563 | 5502484 | 0 | 1871.34 | Local |
| F1 | 02/18/2010 0:00 | 9 | 49.681933 | -109.513284 | 607248 | 5504329 | 1 | 204.82 | Local |
| F1 | 02/18/2010 0:00 | 12 | 49.683667 | -109.514239 | 607175 | 5504520 | 1 | 11.71 | Local |
| F1 | 02/18/2010 0:00 | 15 | 49.683565 | -109.514201 | 607178 | 5504509 | 1 | 36.86 | Local |
| F1 | 02/18/2010 0:00 | 18 | 49.683523 | -109.513695 | 607215 | 5504505 | 1 | 2053.28 | Local |
| F1 | 02/18/2010 0:00 | 21 | 49.665270 | -109.509388 | 607566 | 5502482 | 0 | 418.97 | Local |
| F1 | 02/19/2010 0:00 | 15 | 49.667029 | -109.514522 | 607191 | 5502670 | 0 | 1473.43 | Local |
| F1 | 02/19/2010 0:00 | 21 | 49.679352 | -109.507012 | 607706 | 5504051 | 1 | 1067.25 | Local |
| F1 | 02/20/2010 0:00 | 0 | 49.687435 | -109.499036 | 608263 | 5504961 | 1 | 1407.33 | Local |
| F1 | 02/20/2010 0:00 | 3 | 49.686067 | -109.518430 | 606868 | 5504781 | 1 | 344.48 | Local |
| F1 | 02/20/2010 0:00 | 6 | 49.683010 | -109.519208 | 606818 | 5504440 | 1 | 369.54 | Local |
| F1 | 02/20/2010 0:00 | 9 | 49.683607 | -109.514169 | 607180 | 5504513 | 1 | 8.67 | Local |
| F1 | 02/20/2010 0:00 | 12 | 49.683535 | -109.514121 | 607184 | 5504506 | 1 | 10.38 | Local |
| F1 | 02/20/2010 0:00 | 15 | 49.683602 | -109.514222 | 607177 | 5504513 | 1 | 82.11 | Local |
| F1 | 02/20/2010 0:00 | 18 | 49.683462 | -109.513105 | 607258 | 5504499 | 1 | 629.17 | Local |
| F1 | 02/20/2010 0:00 | 21 | 49.679376 | -109.507073 | 607702 | 5504053 | 1 | 11.47 | Local |
| F1 | 02/21/2010 0:00 | 0 | 49.679280 | -109.507015 | 607706 | 5504043 | 1 | 9.80 | Local |
| F1 | 02/21/2010 0:00 | 3 | 49.679357 | -109.507080 | 607701 | 5504051 | 1 | 7.02 | Local |
| F1 | 02/21/2010 0:00 | 6 | 49.679402 | -109.507013 | 607706 | 5504056 | 1 | 243.65 | Local |
| F1 | 02/21/2010 0:00 | 9 | 49.680940 | -109.504608 | 607876 | 5504231 | 1 | 694.04 | Local |
| F1 | 02/21/2010 0:00 | 12 | 49.680746 | -109.494993 | 608570 | 5504223 | 1 | 5.14 | Local |
| F1 | 02/21/2010 0:00 | 15 | 49.680791 | -109.494977 | 608571 | 5504228 | 1 | 6.74 | Local |
| F1 | 02/21/2010 0:00 | 18 | 49.680734 | -109.494945 | 608574 | 5504222 | 1 | 656.45 | Local |
| F1 | 02/21/2010 0:00 | 21 | 49.682189 | -109.503762 | 607934 | 5504371 | 1 | 2.53 | Local |
| F1 | 02/22/2010 0:00 | 0 | 49.682167 | -109.503767 | 607934 | 5504368 | 1 | 8.53 | Local |
| F1 | 02/22/2010 0:00 | 3 | 49.682117 | -109.503858 | 607927 | 5504363 | 0 | 1837.78 | Local |
| F1 | 02/22/2010 0:00 | 12 | 49.667103 | -109.514502 | 607193 | 5502678 | 0 | 862.29 | Local |
| F1 | 02/22/2010 0:00 | 21 | 49.664641 | -109.503173 | 608016 | 5502421 | 0 | 2142.05 | Local |
| F1 | 02/23/2010 0:00 | 21 | 49.681430 | -109.517728 | 606928 | 5504266 | 1 | 707.27 | Local |
| F1 | 02/24/2010 0:00 | 0 | 49.686197 | -109.511239 | 607386 | 5504806 | 1 | 696.87 | Local |
| F1 | 02/24/2010 0:00 | 3 | 49.690760 | -109.517861 | 606898 | 5505303 | 1 | 493.36 | Local |
| F1 | 02/24/2010 0:00 | 6 | 49.694470 | -109.514109 | 607161 | 5505721 | 1 | 20.47 | Local |
| F1 | 02/24/2010 0:00 | 9 | 49.694650 | -109.514047 | 607165 | 5505741 | 1 | 17.80 | Local |
| F1 | 02/24/2010 0:00 | 12 | 49.694672 | -109.513803 | 607182 | 5505744 | 1 | 28.92 | Local |
| F1 | 02/24/2010 0:00 | 15 | 49.694683 | -109.514203 | 607154 | 5505745 | 1 | 208.28 | Local |
| F1 | 02/24/2010 0:00 | 18 | 49.695156 | -109.511409 | 607354 | 5505801 | 1 | 13.95 | Local |
| F1 | 02/24/2010 0:00 | 21 | 49.695221 | -109.511244 | 607366 | 5505809 | 1 | 36.18 | Local |
| F1 | 02/25/2010 0:00 | 0 | 49.694985 | -109.511589 | 607341 | 5505782 | 0 | 17.94 | Local |
| F1 | 02/25/2010 0:00 | 9 | 49.695120 | -109.511454 | 607351 | 5505797 | 1 | 2.84 | Local |
| F1 | 02/25/2010 0:00 | 12 | 49.695097 | -109.511439 | 607352 | 5505795 | 1 | 7.62 | Local |
| F1 | 02/25/2010 0:00 | 15 | 49.695165 | -109.511426 | 607353 | 5505802 | 1 | 0.91 | Local |
| F1 | 02/25/2010 0:00 | 18 | 49.695164 | -109.511413 | 607354 | 5505802 | 1 | 3.09 | Local |

| | | | | | | | | | |
|---|---|---|---|---|---|---|---|---|---|
| F1 | 02/25/2010 0:00 | 21 | 49.695136 | -109.511420 | 607353 | 5505799 | 1 | 3.04 | Local |
| F1 | 02/26/2010 0:00 | 0 | 49.695121 | -109.511456 | 607351 | 5505797 | 0 | 7.95 | Local |
| F1 | 02/26/2010 0:00 | 6 | 49.695051 | -109.511470 | 607350 | 5505789 | 1 | 16.78 | Local |
| F1 | 02/26/2010 0:00 | 9 | 49.695202 | -109.511464 | 607350 | 5505806 | 1 | 6.36 | Local |
| F1 | 02/26/2010 0:00 | 12 | 49.695155 | -109.511413 | 607354 | 5505801 | 1 | 17.93 | Local |
| F1 | 02/26/2010 0:00 | 15 | 49.695268 | -109.511590 | 607341 | 5505813 | 0 | 19.98 | Local |
| F1 | 02/27/2010 0:00 | 0 | 49.695220 | -109.511323 | 607360 | 5505809 | 1 | 20.24 | Local |
| F1 | 02/27/2010 0:00 | 3 | 49.695099 | -109.511532 | 607345 | 5505795 | 1 | 25.57 | Local |
| F1 | 02/27/2010 0:00 | 6 | 49.695287 | -109.511327 | 607360 | 5505816 | 1 | 14.17 | Local |
| F1 | 02/27/2010 0:00 | 9 | 49.695174 | -109.511420 | 607353 | 5505803 | 1 | 5.45 | Local |
| F1 | 02/27/2010 0:00 | 12 | 49.695132 | -109.511456 | 607351 | 5505798 | 1 | 8.25 | Local |
| F1 | 02/27/2010 0:00 | 15 | 49.695205 | -109.511443 | 607352 | 5505807 | 1 | 21.43 | Local |
| F1 | 02/27/2010 0:00 | 18 | 49.695231 | -109.511148 | 607373 | 5505810 | 0 | 12.38 | Local |
| F1 | 02/28/2010 0:00 | 3 | 49.695207 | -109.511316 | 607361 | 5505807 | 1 | 13.64 | Local |
| F1 | 02/28/2010 0:00 | 6 | 49.695128 | -109.511460 | 607350 | 5505798 | 0 | 4.95 | Local |
| F1 | 02/28/2010 0:00 | 12 | 49.695171 | -109.511445 | 607351 | 5505803 | 1 | 9.92 | Local |
| F1 | 02/28/2010 0:00 | 15 | 49.695254 | -109.511391 | 607355 | 5505812 | 1 | 20.73 | Local |
| F1 | 02/28/2010 0:00 | 18 | 49.695073 | -109.511461 | 607351 | 5505792 | 1 | 11.27 | Local |
| F1 | 02/28/2010 0:00 | 21 | 49.695164 | -109.511528 | 607345 | 5505802 | 1 | 17.28 | Local |
| F1 | 03/01/2010 0:00 | 0 | 49.695202 | -109.511296 | 607362 | 5505807 | 1 | 8.44 | Local |
| F1 | 03/01/2010 0:00 | 3 | 49.695155 | -109.511388 | 607356 | 5505801 | 1 | 6.31 | Local |
| F1 | 03/01/2010 0:00 | 6 | 49.695195 | -109.511326 | 607360 | 5505806 | 1 | 14.31 | Local |
| F1 | 03/01/2010 0:00 | 9 | 49.695137 | -109.511502 | 607347 | 5505799 | 0 | 2.11 | Local |
| F1 | 03/01/2010 0:00 | 15 | 49.695126 | -109.511478 | 607349 | 5505798 | 1 | 7.07 | Local |
| F1 | 03/01/2010 0:00 | 18 | 49.695161 | -109.511396 | 607355 | 5505802 | 0 | 6.45 | Local |
| F1 | 03/02/2010 0:00 | 3 | 49.695167 | -109.511485 | 607349 | 5505802 | 1 | 3.10 | Local |
| F1 | 03/02/2010 0:00 | 6 | 49.695166 | -109.511442 | 607352 | 5505802 | 1 | 9.90 | Local |
| F1 | 03/02/2010 0:00 | 9 | 49.695077 | -109.511434 | 607352 | 5505792 | 1 | 9.77 | Local |
| F1 | 03/02/2010 0:00 | 12 | 49.695114 | -109.511558 | 607343 | 5505796 | 0 | 23.14 | Local |
| F1 | 03/02/2010 0:00 | 18 | 49.694907 | -109.511517 | 607347 | 5505773 | 1 | 38.17 | Local |
| F1 | 03/02/2010 0:00 | 21 | 49.695250 | -109.511479 | 607349 | 5505812 | 1 | 21.20 | Local |
| F1 | 03/03/2010 0:00 | 0 | 49.695062 | -109.511432 | 607353 | 5505791 | 1 | 48.34 | Local |
| F1 | 03/03/2010 0:00 | 3 | 49.695427 | -109.511795 | 607326 | 5505831 | 1 | 38.59 | Local |
| F1 | 03/03/2010 0:00 | 6 | 49.695184 | -109.511413 | 607354 | 5505804 | 1 | 105.22 | Local |
| F1 | 03/03/2010 0:00 | 9 | 49.695025 | -109.512851 | 607250 | 5505785 | 1 | 274.23 | Local |
| F1 | 03/03/2010 0:00 | 12 | 49.694463 | -109.509149 | 607519 | 5505727 | 1 | 9.06 | Local |
| F1 | 03/03/2010 0:00 | 15 | 49.694514 | -109.509052 | 607525 | 5505733 | 1 | 166.17 | Local |
| F1 | 03/03/2010 0:00 | 18 | 49.694862 | -109.511293 | 607363 | 5505769 | 1 | 33.65 | Local |
| F1 | 03/03/2010 0:00 | 21 | 49.695148 | -109.511447 | 607351 | 5505800 | 1 | 15.77 | Local |
| F1 | 03/04/2010 0:00 | 0 | 49.695010 | -109.511394 | 607355 | 5505785 | 1 | 11.43 | Local |
| F1 | 03/04/2010 0:00 | 3 | 49.695058 | -109.511534 | 607345 | 5505790 | 1 | 19.61 | Local |
| F1 | 03/04/2010 0:00 | 6 | 49.695196 | -109.511364 | 607357 | 5505806 | 1 | 126.59 | Local |
| F1 | 03/04/2010 0:00 | 9 | 49.694959 | -109.513081 | 607234 | 5505777 | 1 | 2.58 | Local |
| F1 | 03/04/2010 0:00 | 12 | 49.694967 | -109.513114 | 607232 | 5505778 | 1 | 301.81 | Local |
| F1 | 03/04/2010 0:00 | 15 | 49.694428 | -109.509013 | 607528 | 5505724 | 1 | 71.32 | Local |
| F1 | 03/04/2010 0:00 | 18 | 49.695036 | -109.509328 | 607504 | 5505791 | 1 | 370.62 | Local |
| F1 | 03/04/2010 0:00 | 21 | 49.692074 | -109.506974 | 607681 | 5505465 | 1 | 16.48 | Local |
| F1 | 03/05/2010 0:00 | 0 | 49.691968 | -109.506814 | 607692 | 5505453 | 1 | 690.54 | Local |
| F1 | 03/05/2010 0:00 | 3 | 49.686526 | -109.502202 | 608037 | 5504855 | 1 | 636.54 | Local |
| F1 | 03/05/2010 0:00 | 6 | 49.682741 | -109.495582 | 608523 | 5504444 | 1 | 19.04 | Local |
| F1 | 03/05/2010 0:00 | 9 | 49.682906 | -109.495656 | 608517 | 5504462 | 1 | 11.88 | Local |
| F1 | 03/05/2010 0:00 | 12 | 49.682801 | -109.495621 | 608520 | 5504450 | 1 | 9.82 | Local |
| F1 | 03/05/2010 0:00 | 15 | 49.682765 | -109.495497 | 608529 | 5504447 | 1 | 39.95 | Local |
| F1 | 03/05/2010 0:00 | 18 | 49.683109 | -109.495340 | 608540 | 5504485 | 0 | 1614.89 | Local |
| F1 | 03/06/2010 0:00 | 0 | 49.668782 | -109.499005 | 608307 | 5502887 | 1 | 496.91 | Local |

| | | | | | | | | | |
|---|---|---|---|---|---|---|---|---|---|
| F1 | 03/06/2010 0:00 | 3 | 49.667602 | -109.492364 | 608789 | 5502765 | 1 | 511.66 | Local |
| F1 | 03/06/2010 0:00 | 6 | 49.663003 | -109.492602 | 608782 | 5502254 | 1 | 306.78 | Local |
| F1 | 03/06/2010 0:00 | 9 | 49.660257 | -109.492191 | 608818 | 5501949 | 1 | 6.26 | Local |
| F1 | 03/06/2010 0:00 | 12 | 49.660270 | -109.492275 | 608812 | 5501950 | 1 | 17.56 | Local |
| F1 | 03/06/2010 0:00 | 15 | 49.660365 | -109.492081 | 608826 | 5501961 | 0 | 151.37 | Local |
| F1 | 03/06/2010 0:00 | 21 | 49.659390 | -109.493545 | 608722 | 5501851 | 1 | 247.76 | Local |
| F1 | 03/07/2010 0:00 | 0 | 49.657162 | -109.493563 | 608726 | 5501603 | 1 | 133.85 | Local |
| F1 | 03/07/2010 0:00 | 3 | 49.656179 | -109.492493 | 608805 | 5501495 | 1 | 12.25 | Local |
| F1 | 03/07/2010 0:00 | 6 | 49.656113 | -109.492358 | 608815 | 5501488 | 1 | 1531.33 | Local |
| F1 | 03/07/2010 0:00 | 9 | 49.642737 | -109.487305 | 609210 | 5500009 | 1 | 234.11 | Local |
| F1 | 03/07/2010 0:00 | 12 | 49.641983 | -109.490332 | 608993 | 5499920 | 1 | 14.39 | Local |
| F1 | 03/07/2010 0:00 | 15 | 49.641908 | -109.490169 | 609005 | 5499912 | 1 | 11.10 | Local |
| F1 | 03/07/2010 0:00 | 18 | 49.642006 | -109.490200 | 609002 | 5499923 | 1 | 2.97 | Local |
| F1 | 03/07/2010 0:00 | 21 | 49.642023 | -109.490168 | 609005 | 5499925 | 1 | 74.66 | Local |
| F1 | 03/08/2010 0:00 | 0 | 49.642560 | -109.489546 | 609048 | 5499986 | 1 | 80.05 | Local |
| F1 | 03/08/2010 0:00 | 3 | 49.641981 | -109.490205 | 609002 | 5499920 | 1 | 9.97 | Local |
| F1 | 03/08/2010 0:00 | 6 | 49.642070 | -109.490200 | 609002 | 5499930 | 1 | 6.27 | Local |
| F1 | 03/08/2010 0:00 | 9 | 49.642023 | -109.490151 | 609006 | 5499925 | 1 | 7.09 | Local |
| F1 | 03/08/2010 0:00 | 12 | 49.642015 | -109.490249 | 608999 | 5499924 | 1 | 20.17 | Local |
| F1 | 03/08/2010 0:00 | 15 | 49.642028 | -109.489970 | 609019 | 5499926 | 1 | 57.35 | Local |
| F1 | 03/08/2010 0:00 | 18 | 49.641921 | -109.489193 | 609075 | 5499915 | 0 | 67.65 | Local |
| F1 | 03/09/2010 0:00 | 0 | 49.641911 | -109.490129 | 609008 | 5499913 | 1 | 22.17 | Local |
| F1 | 03/09/2010 0:00 | 3 | 49.642093 | -109.490004 | 609016 | 5499933 | 1 | 4.69 | Local |
| F1 | 03/09/2010 0:00 | 6 | 49.642134 | -109.489992 | 609017 | 5499938 | 1 | 21.20 | Local |
| F1 | 03/09/2010 0:00 | 9 | 49.641972 | -109.490146 | 609006 | 5499919 | 1 | 16.36 | Local |
| F1 | 03/09/2010 0:00 | 12 | 49.642095 | -109.490022 | 609015 | 5499933 | 1 | 3.33 | Local |
| F1 | 03/09/2010 0:00 | 15 | 49.642103 | -109.489978 | 609018 | 5499934 | 1 | 14.02 | Local |
| F1 | 03/09/2010 0:00 | 18 | 49.642043 | -109.490148 | 609006 | 5499927 | 1 | 14.34 | Local |
| F1 | 03/09/2010 0:00 | 21 | 49.642132 | -109.490005 | 609016 | 5499937 | 0 | 3.13 | Local |
| F1 | 03/10/2010 0:00 | 3 | 49.642105 | -109.489992 | 609017 | 5499934 | 1 | 0.99 | Local |
| F1 | 03/10/2010 0:00 | 6 | 49.642100 | -109.490003 | 609016 | 5499934 | 1 | 10.48 | Local |
| F1 | 03/10/2010 0:00 | 9 | 49.642017 | -109.490071 | 609012 | 5499924 | 1 | 13.17 | Local |
| F1 | 03/10/2010 0:00 | 12 | 49.642030 | -109.490253 | 608998 | 5499926 | 0 | 89.25 | Local |
| F1 | 03/10/2010 0:00 | 18 | 49.642653 | -109.489472 | 609053 | 5499996 | 1 | 6.95 | Local |
| F1 | 03/10/2010 0:00 | 21 | 49.642591 | -109.489461 | 609054 | 5499989 | 1 | 82.54 | Local |
| F1 | 03/11/2010 0:00 | 0 | 49.641967 | -109.490082 | 609011 | 5499919 | 1 | 16.61 | Local |
| F1 | 03/11/2010 0:00 | 3 | 49.642102 | -109.489983 | 609018 | 5499934 | 1 | 24.18 | Local |
| F1 | 03/11/2010 0:00 | 6 | 49.641982 | -109.490262 | 608998 | 5499920 | 1 | 23.15 | Local |
| F1 | 03/11/2010 0:00 | 9 | 49.642035 | -109.489952 | 609020 | 5499927 | 1 | 6.69 | Local |
| F1 | 03/11/2010 0:00 | 12 | 49.642086 | -109.490002 | 609016 | 5499932 | 1 | 13.42 | Local |
| F1 | 03/11/2010 0:00 | 15 | 49.642046 | -109.490178 | 609004 | 5499928 | 0 | 11.26 | Local |
| F1 | 03/11/2010 0:00 | 21 | 49.642082 | -109.490032 | 609014 | 5499932 | 1 | 4.21 | Local |
| F1 | 03/12/2010 0:00 | 0 | 49.642072 | -109.489976 | 609018 | 5499931 | 1 | 149.69 | Local |
| F1 | 03/12/2010 0:00 | 3 | 49.643410 | -109.490196 | 608999 | 5500079 | 1 | 175.63 | Local |
| F1 | 03/12/2010 0:00 | 6 | 49.641831 | -109.490206 | 609002 | 5499904 | 1 | 35.06 | Local |
| F1 | 03/12/2010 0:00 | 9 | 49.642127 | -109.490041 | 609013 | 5499937 | 0 | 3.46 | Local |
| F1 | 03/12/2010 0:00 | 18 | 49.642103 | -109.490009 | 609016 | 5499934 | 1 | 3.29 | Local |
| F1 | 03/12/2010 0:00 | 21 | 49.642081 | -109.489979 | 609018 | 5499932 | 1 | 10.33 | Local |
| F1 | 03/13/2010 0:00 | 0 | 49.642076 | -109.489836 | 609028 | 5499931 | 1 | 20.15 | Local |
| F1 | 03/13/2010 0:00 | 3 | 49.642060 | -109.490114 | 609008 | 5499929 | 1 | 15.70 | Local |
| F1 | 03/13/2010 0:00 | 6 | 49.642007 | -109.489913 | 609023 | 5499924 | 1 | 8.26 | Local |
| F1 | 03/13/2010 0:00 | 9 | 49.642071 | -109.489971 | 609019 | 5499931 | 1 | 10.19 | Local |
| F1 | 03/13/2010 0:00 | 12 | 49.642121 | -109.490089 | 609010 | 5499936 | 1 | 15.69 | Local |
| F1 | 03/13/2010 0:00 | 15 | 49.641994 | -109.489996 | 609017 | 5499922 | 1 | 97.41 | Local |
| F1 | 03/13/2010 0:00 | 18 | 49.642600 | -109.489021 | 609086 | 5499991 | 1 | 104.95 | Local |

| | | | | | | | | | |
|---|---|---|---|---|---|---|---|---|---|
| F1 | 03/13/2010 0:00 | 21 | 49.641978 | -109.490114 | 609009 | 5499920 | 1 | 7.55 | Local |
| F1 | 03/14/2010 0:00 | 0 | 49.642039 | -109.490070 | 609012 | 5499927 | 1 | 4.60 | Local |
| F1 | 03/14/2010 0:00 | 3 | 49.642080 | -109.490077 | 609011 | 5499931 | 1 | 4.08 | Local |
| F1 | 03/14/2010 0:00 | 6 | 49.642052 | -109.490113 | 609008 | 5499928 | 1 | 8.93 | Local |
| F1 | 03/14/2010 0:00 | 9 | 49.642131 | -109.490092 | 609010 | 5499937 | 1 | 3.01 | Local |
| F1 | 03/14/2010 0:00 | 12 | 49.642113 | -109.490061 | 609012 | 5499935 | 0 | 14.51 | Local |
| F1 | 03/14/2010 0:00 | 18 | 49.641984 | -109.490037 | 609014 | 5499921 | 1 | 5.36 | Local |
| F1 | 03/14/2010 0:00 | 21 | 49.642011 | -109.490098 | 609010 | 5499924 | 1 | 14.82 | Local |
| F1 | 03/15/2010 0:00 | 0 | 49.642089 | -109.489932 | 609021 | 5499933 | 0 | 30.36 | Local |
| F1 | 03/15/2010 0:00 | 6 | 49.641940 | -109.490285 | 608996 | 5499916 | 1 | 1122.00 | Local |
| F1 | 03/15/2010 0:00 | 9 | 49.632319 | -109.485603 | 609356 | 5498853 | 1 | 647.70 | Local |
| F1 | 03/15/2010 0:00 | 12 | 49.626586 | -109.484012 | 609484 | 5498218 | 1 | 6.18 | Local |
| F1 | 03/15/2010 0:00 | 15 | 49.626546 | -109.483954 | 609488 | 5498213 | 1 | 250.45 | Local |
| F1 | 03/15/2010 0:00 | 18 | 49.624304 | -109.484296 | 609468 | 5497964 | 1 | 177.54 | Local |
| F1 | 03/15/2010 0:00 | 21 | 49.622720 | -109.484601 | 609450 | 5497787 | 0 | 212.39 | Local |
| F1 | 03/16/2010 0:00 | 9 | 49.624296 | -109.482940 | 609566 | 5497965 | 1 | 14.38 | Local |
| F1 | 03/16/2010 0:00 | 12 | 49.624419 | -109.483001 | 609561 | 5497978 | 1 | 5.42 | Local |
| F1 | 03/16/2010 0:00 | 15 | 49.624448 | -109.483062 | 609557 | 5497981 | 1 | 207.63 | Local |
| F1 | 03/16/2010 0:00 | 18 | 49.622999 | -109.484875 | 609429 | 5497818 | 1 | 46.88 | Local |
| F1 | 03/16/2010 0:00 | 21 | 49.623415 | -109.484984 | 609420 | 5497864 | 1 | 198.89 | Local |
| F1 | 03/17/2010 0:00 | 0 | 49.623806 | -109.482297 | 609614 | 5497911 | 1 | 1153.97 | Local |
| F1 | 03/17/2010 0:00 | 3 | 49.633983 | -109.485427 | 609365 | 5499038 | 1 | 112.76 | Local |
| F1 | 03/17/2010 0:00 | 6 | 49.632981 | -109.485674 | 609349 | 5498926 | 1 | 439.67 | Local |
| F1 | 03/17/2010 0:00 | 9 | 49.629310 | -109.483411 | 609521 | 5498521 | 1 | 13.53 | Local |
| F1 | 03/17/2010 0:00 | 12 | 49.629410 | -109.483305 | 609528 | 5498533 | 0 | 355.62 | Local |
| F1 | 03/17/2010 0:00 | 18 | 49.629276 | -109.478385 | 609884 | 5498525 | 1 | 437.68 | Local |
| F1 | 03/17/2010 0:00 | 21 | 49.625480 | -109.476784 | 610008 | 5498105 | 1 | 1067.99 | Local |
| F1 | 03/18/2010 0:00 | 0 | 49.616656 | -109.482622 | 609606 | 5497116 | 1 | 119.05 | Local |
| F1 | 03/18/2010 0:00 | 3 | 49.616807 | -109.484254 | 609488 | 5497130 | 1 | 8.62 | Local |
| F1 | 03/18/2010 0:00 | 6 | 49.616774 | -109.484146 | 609496 | 5497127 | 1 | 284.69 | Local |
| F1 | 03/18/2010 0:00 | 9 | 49.619332 | -109.484301 | 609479 | 5497411 | 1 | 6.00 | Local |
| F1 | 03/18/2010 0:00 | 12 | 49.619360 | -109.484372 | 609474 | 5497414 | 1 | 1.82 | Local |
| F1 | 03/18/2010 0:00 | 15 | 49.619374 | -109.484359 | 609475 | 5497415 | 1 | 297.39 | Local |
| F1 | 03/18/2010 0:00 | 18 | 49.622047 | -109.484217 | 609479 | 5497713 | 1 | 1262.28 | Local |
| F1 | 03/18/2010 0:00 | 21 | 49.633097 | -109.480209 | 609744 | 5498947 | 1 | 1683.31 | Local |
| F1 | 03/19/2010 0:00 | 0 | 49.645848 | -109.492774 | 608808 | 5500346 | 1 | 782.33 | Local |
| F1 | 03/19/2010 0:00 | 3 | 49.650369 | -109.501076 | 608199 | 5500837 | 0 | 438.42 | Local |
| F1 | 03/19/2010 0:00 | 9 | 49.646438 | -109.501550 | 608173 | 5500399 | 1 | 1039.66 | Local |
| F1 | 03/19/2010 0:00 | 12 | 49.641938 | -109.488930 | 609094 | 5499917 | 1 | 188.32 | Local |
| F1 | 03/19/2010 0:00 | 15 | 49.642158 | -109.486344 | 609280 | 5499946 | 1 | 323.31 | Local |
| F1 | 03/19/2010 0:00 | 18 | 49.641881 | -109.481887 | 609603 | 5499921 | 1 | 986.62 | Local |
| F1 | 03/19/2010 0:00 | 21 | 49.638504 | -109.494521 | 608698 | 5499527 | 1 | 697.89 | Local |
| F1 | 03/20/2010 0:00 | 0 | 49.640025 | -109.503897 | 608018 | 5499683 | 0 | 1258.44 | Local |
| F1 | 03/20/2010 0:00 | 15 | 49.636719 | -109.487231 | 609228 | 5499340 | 1 | 130.07 | Local |
| F1 | 03/20/2010 0:00 | 18 | 49.636319 | -109.485539 | 609352 | 5499298 | 1 | 1488.58 | Local |
| F1 | 03/20/2010 0:00 | 21 | 49.622963 | -109.484115 | 609484 | 5497815 | 0 | 2620.61 | Local |
| F1 | 03/21/2010 0:00 | 6 | 49.600975 | -109.471060 | 610477 | 5495390 | 1 | 1107.43 | Local |
| F1 | 03/21/2010 0:00 | 9 | 49.608131 | -109.481718 | 609691 | 5496169 | 1 | 12.84 | Local |
| F1 | 03/21/2010 0:00 | 12 | 49.608246 | -109.481735 | 609689 | 5496182 | 0 | 9.76 | Local |
| F1 | 03/21/2010 0:00 | 18 | 49.608177 | -109.481817 | 609683 | 5496174 | 1 | 536.44 | Local |
| F1 | 03/21/2010 0:00 | 21 | 49.612450 | -109.485264 | 609425 | 5496644 | 0 | 731.21 | Local |
| F1 | 03/22/2010 0:00 | 6 | 49.618986 | -109.484158 | 609490 | 5497373 | 1 | 115.71 | Local |
| F1 | 03/22/2010 0:00 | 9 | 49.619977 | -109.483667 | 609523 | 5497483 | 1 | 7.71 | Local |
| F1 | 03/22/2010 0:00 | 12 | 49.619908 | -109.483679 | 609523 | 5497476 | 1 | 6.45 | Local |
| F1 | 03/22/2010 0:00 | 15 | 49.619888 | -109.483595 | 609529 | 5497474 | 1 | 482.48 | Local |

| | | | | | | | | | |
|---|---|---|---|---|---|---|---|---|---|
| F1 | 03/22/2010 0:00 | 18 | 49.621081 | -109.477174 | 609990 | 5497616 | 1 | 2317.24 | Local |
| F1 | 03/22/2010 0:00 | 21 | 49.641887 | -109.475341 | 610075 | 5499931 | 1 | 1091.75 | Local |
| F1 | 03/23/2010 0:00 | 0 | 49.648896 | -109.464752 | 610824 | 5500726 | 1 | 1083.34 | Local |
| F1 | 03/23/2010 0:00 | 3 | 49.648350 | -109.449771 | 611906 | 5500688 | 1 | 341.42 | Local |
| F1 | 03/23/2010 0:00 | 6 | 49.646300 | -109.446250 | 612165 | 5500465 | 1 | 494.76 | Local |
| F1 | 03/23/2010 0:00 | 9 | 49.645796 | -109.453058 | 611675 | 5500399 | 0 | 4.86 | Local |
| F1 | 03/23/2010 0:00 | 15 | 49.645828 | -109.453011 | 611678 | 5500402 | 1 | 4.12 | Local |
| F1 | 03/23/2010 0:00 | 18 | 49.645793 | -109.453033 | 611677 | 5500399 | 1 | 288.62 | Local |
| F1 | 03/23/2010 0:00 | 21 | 49.646278 | -109.449106 | 611959 | 5500458 | 1 | 66.82 | Local |
| F1 | 03/24/2010 0:00 | 0 | 49.646595 | -109.448320 | 612015 | 5500495 | 1 | 215.89 | Local |
| F1 | 03/24/2010 0:00 | 3 | 49.648284 | -109.449796 | 611905 | 5500680 | 1 | 1440.77 | Local |
| F1 | 03/24/2010 0:00 | 6 | 49.656775 | -109.464871 | 610797 | 5501602 | 1 | 224.62 | Local |
| F1 | 03/24/2010 0:00 | 9 | 49.655156 | -109.466733 | 610667 | 5501419 | 0 | 69.78 | Local |
| F1 | 03/24/2010 0:00 | 15 | 49.654908 | -109.467621 | 610603 | 5501390 | 1 | 120.91 | Local |
| F1 | 03/24/2010 0:00 | 18 | 49.655800 | -109.466663 | 610670 | 5501491 | 1 | 146.01 | Local |
| F1 | 03/24/2010 0:00 | 21 | 49.657027 | -109.465942 | 610719 | 5501628 | 1 | 193.37 | Local |
| F1 | 03/25/2010 0:00 | 0 | 49.655344 | -109.466622 | 610674 | 5501440 | 1 | 82.07 | Local |
| F1 | 03/25/2010 0:00 | 3 | 49.656081 | -109.466544 | 610678 | 5501522 | 1 | 17.31 | Local |
| F1 | 03/25/2010 0:00 | 6 | 49.656042 | -109.466776 | 610662 | 5501518 | 1 | 75.70 | Local |
| F1 | 03/25/2010 0:00 | 9 | 49.655364 | -109.466678 | 610670 | 5501442 | 1 | 10.70 | Local |
| F1 | 03/25/2010 0:00 | 12 | 49.655342 | -109.466822 | 610660 | 5501440 | 1 | 21.18 | Local |
| F1 | 03/25/2010 0:00 | 15 | 49.655531 | -109.466853 | 610657 | 5501461 | 1 | 80.20 | Local |
| F1 | 03/25/2010 0:00 | 18 | 49.656224 | -109.466547 | 610678 | 5501538 | 1 | 117.22 | Local |
| F1 | 03/25/2010 0:00 | 21 | 49.657004 | -109.465454 | 610755 | 5501627 | 1 | 134.94 | Local |
| F1 | 03/26/2010 0:00 | 0 | 49.656099 | -109.466699 | 610667 | 5501524 | 1 | 8.15 | Local |
| F1 | 03/26/2010 0:00 | 3 | 49.656074 | -109.466593 | 610675 | 5501521 | 1 | 76.23 | Local |
| F1 | 03/26/2010 0:00 | 6 | 49.655395 | -109.466747 | 610665 | 5501446 | 1 | 758.55 | Local |
| F1 | 03/26/2010 0:00 | 9 | 49.648683 | -109.464875 | 610815 | 5500702 | 1 | 251.24 | Local |
| F1 | 03/26/2010 0:00 | 12 | 49.647600 | -109.467930 | 610597 | 5500577 | 1 | 4.29 | Local |
| F1 | 03/26/2010 0:00 | 15 | 49.647567 | -109.467960 | 610595 | 5500574 | 1 | 358.96 | Local |
| F1 | 03/26/2010 0:00 | 18 | 49.650096 | -109.471051 | 610366 | 5500850 | 1 | 851.18 | Local |
| F1 | 03/26/2010 0:00 | 21 | 49.656974 | -109.465877 | 610724 | 5501623 | 1 | 486.74 | Local |
| F1 | 03/27/2010 0:00 | 0 | 49.656638 | -109.472600 | 610240 | 5501575 | 1 | 1080.11 | Local |
| F1 | 03/27/2010 0:00 | 3 | 49.654228 | -109.487094 | 609199 | 5501286 | 1 | 1180.88 | Local |
| F1 | 03/27/2010 0:00 | 6 | 49.643619 | -109.487821 | 609170 | 5500106 | 1 | 1051.68 | Local |
| F1 | 03/27/2010 0:00 | 9 | 49.634362 | -109.484833 | 609407 | 5499081 | 1 | 21.09 | Local |
| F1 | 03/27/2010 0:00 | 12 | 49.634530 | -109.484967 | 609397 | 5499100 | 0 | 8.63 | Local |
| F1 | 03/27/2010 0:00 | 18 | 49.634588 | -109.484887 | 609402 | 5499106 | 1 | 756.15 | Local |
| F1 | 03/27/2010 0:00 | 21 | 49.627792 | -109.485272 | 609390 | 5498350 | 1 | 953.16 | Local |
| F1 | 03/28/2010 0:00 | 0 | 49.619246 | -109.486295 | 609335 | 5497398 | 1 | 1722.68 | Local |
| F1 | 03/28/2010 0:00 | 3 | 49.625607 | -109.464550 | 610891 | 5498137 | 1 | 1303.93 | Local |
| F1 | 03/28/2010 0:00 | 6 | 49.633717 | -109.477590 | 609931 | 5499020 | 1 | 107.61 | Local |
| F1 | 03/28/2010 0:00 | 9 | 49.632753 | -109.477717 | 609924 | 5498912 | 1 | 4.72 | Local |
| F1 | 03/28/2010 0:00 | 12 | 49.632795 | -109.477726 | 609924 | 5498917 | 1 | 1.05 | Local |
| F1 | 03/28/2010 0:00 | 15 | 49.632789 | -109.477714 | 609924 | 5498917 | 1 | 132.60 | Local |
| F1 | 03/28/2010 0:00 | 18 | 49.633636 | -109.479008 | 609829 | 5499009 | 1 | 20.02 | Local |
| F1 | 03/28/2010 0:00 | 21 | 49.633717 | -109.478760 | 609847 | 5499018 | 1 | 134.31 | Local |
| F1 | 03/29/2010 0:00 | 0 | 49.633818 | -109.476907 | 609980 | 5499032 | 1 | 1864.20 | Local |
| F1 | 03/29/2010 0:00 | 3 | 49.649078 | -109.466216 | 610718 | 5500744 | 1 | 1148.02 | Local |
| F1 | 03/29/2010 0:00 | 6 | 49.649204 | -109.450317 | 611865 | 5500782 | 1 | 489.01 | Local |
| F1 | 03/29/2010 0:00 | 9 | 49.645363 | -109.453615 | 611636 | 5500350 | 1 | 6.98 | Local |
| F1 | 03/29/2010 0:00 | 12 | 49.645307 | -109.453660 | 611633 | 5500344 | 1 | 6.59 | Local |
| F1 | 03/29/2010 0:00 | 15 | 49.645289 | -109.453573 | 611639 | 5500342 | 0 | 490.97 | Local |
| F1 | 03/29/2010 0:00 | 21 | 49.649166 | -109.450318 | 611865 | 5500778 | 1 | 1578.60 | Local |
| F1 | 03/30/2010 0:00 | 0 | 49.657720 | -109.467769 | 610586 | 5501703 | 1 | 2326.93 | Local |

| | | | | | | | | | |
|---|---|---|---|---|---|---|---|---|---|
| F1 | 03/30/2010 0:00 | 3 | 49.672579 | -109.490471 | 608914 | 5503322 | 1 | 1262.79 | Local |
| F1 | 03/30/2010 0:00 | 6 | 49.681982 | -109.480657 | 609601 | 5504381 | 1 | 243.64 | Local |
| F1 | 03/30/2010 0:00 | 9 | 49.684060 | -109.481730 | 609519 | 5504611 | 1 | 6.33 | Local |
| F1 | 03/30/2010 0:00 | 12 | 49.684024 | -109.481662 | 609524 | 5504607 | 1 | 10.98 | Local |
| F1 | 03/30/2010 0:00 | 15 | 49.683987 | -109.481804 | 609514 | 5504602 | 1 | 13.39 | Local |
| F1 | 03/30/2010 0:00 | 18 | 49.684099 | -109.481733 | 609519 | 5504615 | 1 | 14.86 | Local |
| F1 | 03/30/2010 0:00 | 21 | 49.683989 | -109.481851 | 609511 | 5504603 | 1 | 9.89 | Local |
| F1 | 03/31/2010 0:00 | 0 | 49.684044 | -109.481744 | 609518 | 5504609 | 1 | 243.43 | Local |
| F1 | 03/31/2010 0:00 | 3 | 49.682001 | -109.480532 | 609610 | 5504383 | 1 | 1964.28 | Local |
| F1 | 03/31/2010 0:00 | 6 | 49.696380 | -109.464713 | 610719 | 5506005 | 1 | 463.20 | Local |
| F1 | 03/31/2010 0:00 | 9 | 49.692217 | -109.464932 | 610712 | 5505542 | 0 | 20.29 | Local |
| F1 | 03/31/2010 0:00 | 15 | 49.692388 | -109.464834 | 610719 | 5505561 | 1 | 9.44 | Local |
| F1 | 03/31/2010 0:00 | 18 | 49.692326 | -109.464923 | 610713 | 5505554 | 1 | 11.72 | Local |
| F1 | 03/31/2010 0:00 | 21 | 49.692280 | -109.465070 | 610702 | 5505549 | 0 | 14.13 | Local |
| F1 | 04/01/2010 0:00 | 3 | 49.692374 | -109.464938 | 610712 | 5505559 | 1 | 8.19 | Local |
| F1 | 04/01/2010 0:00 | 6 | 49.692302 | -109.464960 | 610710 | 5505551 | 1 | 91.45 | Local |
| F1 | 04/01/2010 0:00 | 9 | 49.691563 | -109.464403 | 610752 | 5505470 | 1 | 6.01 | Local |
| F1 | 04/01/2010 0:00 | 12 | 49.691605 | -109.464456 | 610748 | 5505475 | 0 | 71.16 | Local |
| F1 | 04/01/2010 0:00 | 18 | 49.692019 | -109.465208 | 610693 | 5505520 | 1 | 37.89 | Local |
| F1 | 04/01/2010 0:00 | 21 | 49.692358 | -109.465169 | 610695 | 5505557 | 1 | 3.47 | Local |
| F1 | 04/02/2010 0:00 | 21 | 49.693086 | -109.460892 | 611002 | 5505645 | 1 | 5749.22 | Trans |
| F1 | 04/02/2010 0:00 | 0 | 49.692382 | -109.465201 | 610693 | 5505560 | 1 | 946.00 | Local |
| F1 | 04/02/2010 0:00 | 3 | 49.683989 | -109.467348 | 610557 | 5504624 | 1 | 486.07 | Local |
| F1 | 04/02/2010 0:00 | 6 | 49.679629 | -109.467821 | 610533 | 5504138 | 1 | 144.27 | Local |
| F1 | 04/02/2010 0:00 | 9 | 49.678575 | -109.468989 | 610451 | 5504019 | 1 | 3.65 | Local |
| F1 | 04/02/2010 0:00 | 12 | 49.678605 | -109.469010 | 610449 | 5504023 | 1 | 12.08 | Local |
| F1 | 04/02/2010 0:00 | 15 | 49.678694 | -109.468915 | 610456 | 5504033 | 1 | 277.68 | Local |
| F1 | 04/02/2010 0:00 | 18 | 49.680220 | -109.465869 | 610672 | 5504207 | 1 | 1474.93 | Local |
| F1 | 04/03/2010 0:00 | 0 | 49.691323 | -109.381238 | 616751 | 5505569 | 1 | 5404.37 | Trans |
| F1 | 04/03/2010 0:00 | 3 | 49.713709 | -109.314725 | 621492 | 5508163 | 1 | 5634.22 | Trans |
| F1 | 04/03/2010 0:00 | 6 | 49.753167 | -109.265688 | 624925 | 5512631 | 1 | 2129.56 | Trans |
| F1 | 04/03/2010 0:00 | 9 | 49.748745 | -109.236930 | 627008 | 5512187 | 1 | 909.82 | Trans |
| F1 | 04/03/2010 0:00 | 12 | 49.754051 | -109.227317 | 627687 | 5512793 | 1 | 9.14 | Trans |
| F1 | 04/03/2010 0:00 | 15 | 49.753978 | -109.227258 | 627691 | 5512785 | 1 | 1154.03 | Trans |
| F1 | 04/03/2010 0:00 | 18 | 49.763959 | -109.222867 | 627981 | 5513902 | 1 | 1237.97 | Trans |
| F1 | 04/03/2010 0:00 | 21 | 49.760100 | -109.206747 | 629152 | 5513501 | 1 | 764.54 | Trans |
| F1 | 04/04/2010 0:00 | 0 | 49.766974 | -109.206631 | 629142 | 5514266 | 1 | 723.16 | Trans |
| F1 | 04/04/2010 0:00 | 3 | 49.773173 | -109.209666 | 628907 | 5514949 | 1 | 716.44 | Trans |
| F1 | 04/04/2010 0:00 | 6 | 49.773750 | -109.219574 | 628192 | 5514997 | 1 | 1409.48 | Trans |
| F1 | 04/04/2010 0:00 | 9 | 49.762535 | -109.210457 | 628878 | 5513765 | 1 | 25.69 | Trans |
| F1 | 04/04/2010 0:00 | 12 | 49.762727 | -109.210653 | 628864 | 5513786 | 1 | 36.06 | Trans |
| F1 | 04/04/2010 0:00 | 15 | 49.762416 | -109.210514 | 628875 | 5513752 | 1 | 60.97 | Trans |
| F1 | 04/04/2010 0:00 | 18 | 49.762962 | -109.210438 | 628879 | 5513813 | 1 | 1637.64 | Trans |
| F1 | 04/04/2010 0:00 | 21 | 49.758450 | -109.232078 | 627332 | 5513274 | 1 | 1465.14 | Local |
| F1 | 04/05/2010 0:00 | 0 | 49.745354 | -109.234305 | 627206 | 5511815 | 1 | 68.44 | Local |
| F1 | 04/05/2010 0:00 | 3 | 49.745151 | -109.235202 | 627142 | 5511791 | 1 | 8.94 | Local |
| F1 | 04/05/2010 0:00 | 6 | 49.745192 | -109.235309 | 627134 | 5511795 | 1 | 410.89 | Local |
| F1 | 04/05/2010 0:00 | 9 | 49.748735 | -109.236929 | 627008 | 5512186 | 0 | 173.80 | Local |
| F1 | 04/05/2010 0:00 | 15 | 49.747334 | -109.238000 | 626935 | 5512029 | 1 | 275.17 | Local |
| F1 | 04/05/2010 0:00 | 18 | 49.749790 | -109.237532 | 626962 | 5512302 | 1 | 864.86 | Local |
| F1 | 04/05/2010 0:00 | 21 | 49.756786 | -109.242777 | 626566 | 5513071 | 1 | 181.37 | Local |
| F1 | 04/06/2010 0:00 | 0 | 49.757902 | -109.244613 | 626431 | 5513192 | 1 | 64.28 | Local |
| F1 | 04/06/2010 0:00 | 3 | 49.757939 | -109.243723 | 626495 | 5513198 | 1 | 14.41 | Local |
| F1 | 04/06/2010 0:00 | 6 | 49.757817 | -109.243657 | 626500 | 5513184 | 1 | 4.49 | Local |
| F1 | 04/06/2010 0:00 | 9 | 49.757799 | -109.243602 | 626504 | 5513183 | 1 | 0.53 | Local |

| | | | | | | | | | |
|---|---|---|---|---|---|---|---|---|---|
| F1 | 04/06/2010 0:00 | 12 | 49.757803 | -109.243600 | 626504 | 5513183 | 1 | 2.30 | Local |
| F1 | 04/06/2010 0:00 | 15 | 49.757815 | -109.243627 | 626502 | 5513184 | 1 | 3.94 | Local |
| F1 | 04/06/2010 0:00 | 18 | 49.757850 | -109.243624 | 626502 | 5513188 | 1 | 6.12 | Local |
| F1 | 04/06/2010 0:00 | 21 | 49.757795 | -109.243623 | 626502 | 5513182 | 1 | 7.16 | Local |
| F1 | 04/07/2010 0:00 | 0 | 49.757860 | -109.243620 | 626502 | 5513189 | 1 | 10.08 | Local |
| F1 | 04/07/2010 0:00 | 3 | 49.757798 | -109.243723 | 626495 | 5513182 | 0 | 43.47 | Local |
| F1 | 04/07/2010 0:00 | 9 | 49.757892 | -109.243137 | 626537 | 5513194 | 0 | 34.89 | Local |
| F1 | 04/07/2010 0:00 | 21 | 49.757616 | -109.243369 | 626521 | 5513163 | 1 | 10.18 | Local |
| F1 | 04/08/2010 0:00 | 0 | 49.757568 | -109.243489 | 626513 | 5513157 | 1 | 33.15 | Local |
| F1 | 04/08/2010 0:00 | 3 | 49.757860 | -109.243581 | 626505 | 5513189 | 1 | 6.53 | Local |
| F1 | 04/08/2010 0:00 | 6 | 49.757865 | -109.243671 | 626499 | 5513190 | 1 | 2475.53 | Local |
| F1 | 04/08/2010 0:00 | 9 | 49.763400 | -109.210385 | 628881 | 5513862 | 1 | 52.82 | Local |
| F1 | 04/08/2010 0:00 | 12 | 49.763336 | -109.209658 | 628934 | 5513856 | 0 | 89.45 | Local |
| F1 | 04/08/2010 0:00 | 18 | 49.763962 | -109.208880 | 628988 | 5513927 | 1 | 1707.63 | Local |
| F1 | 04/08/2010 0:00 | 21 | 49.766084 | -109.232360 | 627292 | 5514123 | 1 | 1566.99 | Local |
| F1 | 04/09/2010 0:00 | 0 | 49.765843 | -109.254112 | 625726 | 5514059 | 1 | 2013.71 | Local |
| F1 | 04/09/2010 0:00 | 3 | 49.781770 | -109.240808 | 626643 | 5515852 | 1 | 3262.55 | Local |
| F1 | 04/09/2010 0:00 | 6 | 49.761571 | -109.207954 | 629061 | 5513663 | 1 | 1359.52 | Local |
| F1 | 04/09/2010 0:00 | 9 | 49.773786 | -109.207156 | 629086 | 5515022 | 1 | 48.69 | Local |
| F1 | 04/09/2010 0:00 | 12 | 49.774179 | -109.207453 | 629064 | 5515065 | 1 | 3.60 | Local |
| F1 | 04/09/2010 0:00 | 15 | 49.774176 | -109.207403 | 629067 | 5515065 | 1 | 327.14 | Local |
| F1 | 04/09/2010 0:00 | 18 | 49.771256 | -109.206858 | 629114 | 5514741 | 1 | 2012.03 | Local |
| F1 | 04/09/2010 0:00 | 21 | 49.757629 | -109.188482 | 630474 | 5513258 | 1 | 2677.55 | Local |
| F1 | 04/10/2010 0:00 | 0 | 49.759010 | -109.151376 | 633143 | 5513477 | 1 | 2808.56 | Local |
| F1 | 04/10/2010 0:00 | 3 | 49.749167 | -109.115476 | 635756 | 5512447 | 1 | 597.23 | Local |
| F1 | 04/10/2010 0:00 | 6 | 49.746497 | -109.108285 | 636281 | 5512163 | 1 | 98.00 | Local |
| F1 | 04/10/2010 0:00 | 9 | 49.746483 | -109.109644 | 636183 | 5512159 | 1 | 5.24 | Local |
| F1 | 04/10/2010 0:00 | 12 | 49.746526 | -109.109614 | 636185 | 5512164 | 0 | 214.87 | Local |
| F1 | 04/10/2010 0:00 | 18 | 49.747734 | -109.107287 | 636350 | 5512302 | 1 | 760.84 | Local |
| F1 | 04/10/2010 0:00 | 21 | 49.753603 | -109.112713 | 635942 | 5512945 | 1 | 220.78 | Local |
| F1 | 04/11/2010 0:00 | 0 | 49.755312 | -109.111154 | 636050 | 5513138 | 1 | 4.99 | Local |
| F1 | 04/11/2010 0:00 | 3 | 49.755267 | -109.111154 | 636050 | 5513133 | 1 | 3228.17 | Local |
| F1 | 04/11/2010 0:00 | 6 | 49.771270 | -109.148544 | 633313 | 5514845 | 1 | 2070.09 | Local |
| F1 | 04/11/2010 0:00 | 9 | 49.773157 | -109.177139 | 631249 | 5515004 | 1 | 5.17 | Local |
| F1 | 04/11/2010 0:00 | 12 | 49.773203 | -109.177153 | 631248 | 5515009 | 0 | 18.16 | Local |
| F1 | 04/11/2010 0:00 | 18 | 49.773186 | -109.176903 | 631266 | 5515008 | 1 | 2165.35 | Local |
| F1 | 04/11/2010 0:00 | 21 | 49.771270 | -109.206823 | 629117 | 5514743 | 1 | 2.63 | Local |
| F1 | 04/12/2010 0:00 | 0 | 49.771282 | -109.206854 | 629115 | 5514744 | 1 | 913.09 | Local |
| F1 | 04/12/2010 0:00 | 3 | 49.763187 | -109.208977 | 628983 | 5513841 | 1 | 2657.53 | Local |
| F1 | 04/12/2010 0:00 | 6 | 49.748445 | -109.238010 | 626931 | 5512152 | 1 | 725.13 | Local |
| F1 | 04/12/2010 0:00 | 9 | 49.743558 | -109.231348 | 627424 | 5511620 | 1 | 409.13 | Local |
| F1 | 04/12/2010 0:00 | 12 | 49.741307 | -109.235839 | 627106 | 5511362 | 1 | 18.74 | Local |
| F1 | 04/12/2010 0:00 | 15 | 49.741152 | -109.235739 | 627114 | 5511345 | 1 | 734.07 | Local |
| F1 | 04/12/2010 0:00 | 18 | 49.747740 | -109.235086 | 627143 | 5512079 | 1 | 852.17 | Local |
| F1 | 04/12/2010 0:00 | 21 | 49.740916 | -109.229706 | 627549 | 5511329 | 1 | 13.42 | Local |
| F1 | 04/13/2010 0:00 | 0 | 49.740988 | -109.229556 | 627560 | 5511337 | 1 | 324.18 | Local |
| F1 | 04/13/2010 0:00 | 3 | 49.742192 | -109.233653 | 627261 | 5511464 | 1 | 2007.31 | Local |
| F1 | 04/13/2010 0:00 | 6 | 49.758596 | -109.245280 | 626381 | 5513268 | 1 | 1675.03 | Local |
| F1 | 04/13/2010 0:00 | 9 | 49.773652 | -109.244582 | 626392 | 5514943 | 1 | 2672.45 | Local |
| F1 | 04/13/2010 0:00 | 12 | 49.771394 | -109.207638 | 629058 | 5514755 | 0 | 124.16 | Local |
| F1 | 04/13/2010 0:00 | 18 | 49.770520 | -109.206566 | 629137 | 5514660 | 1 | 1357.81 | Local |
| F1 | 04/13/2010 0:00 | 21 | 49.758358 | -109.208239 | 629049 | 5513305 | 1 | 2411.55 | Local |
| F1 | 04/14/2010 0:00 | 0 | 49.778411 | -109.195494 | 629914 | 5515556 | 1 | 1010.78 | Local |
| F1 | 04/14/2010 0:00 | 3 | 49.787436 | -109.197168 | 629769 | 5516557 | 1 | 19.41 | Local |
| F1 | 04/14/2010 0:00 | 6 | 49.787439 | -109.196898 | 629788 | 5516557 | 0 | 1954.67 | Local |

| | | | | | | | | | |
|---|---|---|---|---|---|---|---|---|---|
| F1 | 04/14/2010 0:00 | 12 | 49.771350 | -109.207829 | 629044 | 5514750 | 0 | 1958.66 | Local |
| F1 | 04/14/2010 0:00 | 21 | 49.787504 | -109.196988 | 629782 | 5516564 | 1 | 1589.96 | Local |
| F1 | 04/15/2010 0:00 | 0 | 49.777690 | -109.213047 | 628652 | 5515446 | 1 | 1615.37 | Local |
| F1 | 04/15/2010 0:00 | 3 | 49.763322 | -109.209749 | 628927 | 5513854 | 1 | 9.96 | Local |
| F1 | 04/15/2010 0:00 | 6 | 49.763360 | -109.209874 | 628918 | 5513858 | 1 | 9.98 | Local |
| F1 | 04/15/2010 0:00 | 9 | 49.763285 | -109.209952 | 628913 | 5513850 | 0 | 166.63 | Local |
| F1 | 04/15/2010 0:00 | 15 | 49.762068 | -109.208603 | 629013 | 5513717 | 1 | 6.36 | Local |
| F1 | 04/15/2010 0:00 | 18 | 49.762110 | -109.208664 | 629009 | 5513721 | 1 | 2825.03 | Local |
| F1 | 04/15/2010 0:00 | 21 | 49.753431 | -109.171809 | 631686 | 5512820 | 1 | 1999.06 | Local |
| F1 | 04/16/2010 0:00 | 0 | 49.753331 | -109.144064 | 633685 | 5512858 | 1 | 2436.37 | Local |
| F1 | 04/16/2010 0:00 | 3 | 49.744814 | -109.112911 | 635953 | 5511968 | 1 | 1248.59 | Local |
| F1 | 04/16/2010 0:00 | 6 | 49.751796 | -109.099340 | 636911 | 5512768 | 1 | 460.30 | Local |
| F1 | 04/16/2010 0:00 | 9 | 49.753607 | -109.093596 | 637319 | 5512980 | 1 | 106.11 | Local |
| F1 | 04/16/2010 0:00 | 12 | 49.753972 | -109.094956 | 637220 | 5513018 | 1 | 14.04 | Local |
| F1 | 04/16/2010 0:00 | 15 | 49.754087 | -109.094877 | 637226 | 5513031 | 1 | 26.30 | Local |
| F1 | 04/16/2010 0:00 | 18 | 49.753861 | -109.094984 | 637219 | 5513006 | 1 | 101.79 | Local |
| F1 | 04/16/2010 0:00 | 21 | 49.754448 | -109.096068 | 637139 | 5513069 | 0 | 825.15 | Local |
| F1 | 04/17/2010 0:00 | 3 | 49.760599 | -109.102476 | 636660 | 5513741 | 1 | 2914.04 | Local |
| F1 | 04/17/2010 0:00 | 6 | 49.765418 | -109.142239 | 633783 | 5514205 | 0 | 1041.55 | Local |
| F1 | 04/17/2010 0:00 | 12 | 49.765546 | -109.156697 | 632742 | 5514194 | 1 | 24.81 | Local |
| F1 | 04/17/2010 0:00 | 15 | 49.765495 | -109.157032 | 632718 | 5514188 | 1 | 26.30 | Local |
| F1 | 04/17/2010 0:00 | 18 | 49.765483 | -109.156667 | 632744 | 5514187 | 1 | 1824.07 | Local |
| F1 | 04/17/2010 0:00 | 21 | 49.753466 | -109.173902 | 631535 | 5512821 | 1 | 2653.64 | Local |
| F1 | 04/18/2010 0:00 | 0 | 49.762066 | -109.208262 | 629038 | 5513717 | 1 | 273.23 | Local |
| F1 | 04/18/2010 0:00 | 3 | 49.760921 | -109.211618 | 628799 | 5513584 | 1 | 175.47 | Local |
| F1 | 04/18/2010 0:00 | 6 | 49.760241 | -109.213817 | 628643 | 5513505 | 1 | 1316.93 | Local |
| F1 | 04/18/2010 0:00 | 9 | 49.771077 | -109.206437 | 629145 | 5514722 | 1 | 6.56 | Local |
| F1 | 04/18/2010 0:00 | 12 | 49.771115 | -109.206368 | 629150 | 5514726 | 1 | 100.96 | Local |
| F1 | 04/18/2010 0:00 | 15 | 49.771527 | -109.207616 | 629059 | 5514770 | 1 | 59.26 | Local |
| F1 | 04/18/2010 0:00 | 18 | 49.771323 | -109.208376 | 629005 | 5514746 | 1 | 1324.43 | Local |
| F1 | 04/18/2010 0:00 | 21 | 49.759938 | -109.213776 | 628646 | 5513471 | 1 | 1231.44 | Local |
| F1 | 04/19/2010 0:00 | 0 | 49.753497 | -109.227681 | 627662 | 5512731 | 1 | 697.13 | Local |
| F1 | 04/19/2010 0:00 | 3 | 49.749060 | -109.234516 | 627181 | 5512226 | 1 | 998.06 | Local |
| F1 | 04/19/2010 0:00 | 6 | 49.756979 | -109.241036 | 626691 | 5513096 | 1 | 256.77 | Local |
| F1 | 04/19/2010 0:00 | 9 | 49.758397 | -109.243849 | 626485 | 5513249 | 1 | 8.07 | Local |
| F1 | 04/19/2010 0:00 | 12 | 49.758341 | -109.243778 | 626490 | 5513242 | 1 | 12.52 | Local |
| F1 | 04/19/2010 0:00 | 15 | 49.758343 | -109.243604 | 626502 | 5513243 | 1 | 439.97 | Local |
| F1 | 04/19/2010 0:00 | 18 | 49.758866 | -109.249658 | 626065 | 5513291 | 1 | 1034.47 | Local |
| F1 | 04/19/2010 0:00 | 21 | 49.767984 | -109.252505 | 625836 | 5514300 | 1 | 897.59 | Local |
| F1 | 04/20/2010 0:00 | 0 | 49.769314 | -109.264797 | 624948 | 5514427 | 1 | 803.74 | Local |
| F1 | 04/20/2010 0:00 | 3 | 49.762977 | -109.270164 | 624578 | 5513714 | 1 | 41.74 | Local |
| F1 | 04/20/2010 0:00 | 6 | 49.762925 | -109.270738 | 624536 | 5513707 | 0 | 1600.14 | Local |
| F1 | 04/20/2010 0:00 | 12 | 49.771964 | -109.253453 | 625758 | 5514741 | 1 | 7.94 | Local |
| F1 | 04/20/2010 0:00 | 15 | 49.772016 | -109.253377 | 625763 | 5514747 | 1 | 203.78 | Local |
| F1 | 04/20/2010 0:00 | 18 | 49.771122 | -109.250908 | 625943 | 5514651 | 1 | 1080.95 | Local |
| F1 | 04/20/2010 0:00 | 21 | 49.763998 | -109.261119 | 625226 | 5513842 | 1 | 687.01 | Local |
| F1 | 04/21/2010 0:00 | 0 | 49.762598 | -109.270408 | 624561 | 5513671 | 1 | 3.51 | Local |
| F1 | 04/21/2010 0:00 | 3 | 49.762570 | -109.270386 | 624563 | 5513668 | 1 | 1218.24 | Local |
| F1 | 04/21/2010 0:00 | 6 | 49.760771 | -109.253704 | 625769 | 5513496 | 1 | 821.25 | Local |
| F1 | 04/21/2010 0:00 | 9 | 49.756781 | -109.244111 | 626470 | 5513069 | 0 | 32.44 | Local |
| F1 | 04/21/2010 0:00 | 15 | 49.756769 | -109.244561 | 626438 | 5513066 | 1 | 12.68 | Local |
| F1 | 04/21/2010 0:00 | 18 | 49.756734 | -109.244728 | 626426 | 5513062 | 1 | 1248.12 | Local |
| F1 | 04/21/2010 0:00 | 21 | 49.762631 | -109.229987 | 627472 | 5513743 | 1 | 1467.25 | Local |
| F1 | 04/22/2010 0:00 | 0 | 49.763497 | -109.209661 | 628933 | 5513874 | 1 | 722.37 | Local |
| F1 | 04/22/2010 0:00 | 3 | 49.769436 | -109.213724 | 628625 | 5514527 | 1 | 2261.06 | Local |

| | | | | | | | | | |
|---|---|---|---|---|---|---|---|---|---|
| F1 | 04/22/2010 0:00 | 6 | 49.772801 | -109.244686 | 626387 | 5514849 | 1 | 140.49 | Local |
| F1 | 04/22/2010 0:00 | 9 | 49.773853 | -109.245767 | 626306 | 5514964 | 1 | 9.17 | Local |
| F1 | 04/22/2010 0:00 | 12 | 49.773882 | -109.245886 | 626298 | 5514967 | 0 | 72.74 | Local |
| F1 | 04/22/2010 0:00 | 18 | 49.774151 | -109.246806 | 626231 | 5514995 | 1 | 1523.60 | Local |
| F1 | 04/22/2010 0:00 | 21 | 49.767200 | -109.265036 | 624936 | 5514192 | 1 | 2707.21 | Local |
| F1 | 04/23/2010 0:00 | 0 | 49.750736 | -109.292720 | 622984 | 5512316 | 1 | 113.94 | Local |
| F1 | 04/23/2010 0:00 | 3 | 49.751731 | -109.293095 | 622955 | 5512426 | 1 | 1002.01 | Local |
| F1 | 04/23/2010 0:00 | 6 | 49.757720 | -109.303487 | 622191 | 5513075 | 1 | 2514.86 | Local |
| F1 | 04/23/2010 0:00 | 9 | 49.746071 | -109.333406 | 620065 | 5511731 | 0 | 56.98 | Local |
| F1 | 04/23/2010 0:00 | 15 | 49.746584 | -109.333425 | 620062 | 5511788 | 1 | 178.04 | Local |
| F1 | 04/23/2010 0:00 | 18 | 49.745948 | -109.331157 | 620227 | 5511721 | 1 | 673.90 | Local |
| F1 | 04/23/2010 0:00 | 21 | 49.749389 | -109.338856 | 619664 | 5512091 | 1 | 18.08 | Local |
| F1 | 04/24/2010 0:00 | 0 | 49.749501 | -109.339038 | 619651 | 5512104 | 1 | 9.58 | Local |
| F1 | 04/24/2010 0:00 | 3 | 49.749569 | -109.338956 | 619657 | 5512111 | 1 | 693.64 | Local |
| F1 | 04/24/2010 0:00 | 6 | 49.752086 | -109.330147 | 620285 | 5512405 | 1 | 351.02 | Local |
| F1 | 04/24/2010 0:00 | 9 | 49.749373 | -109.327657 | 620471 | 5512108 | 1 | 12.17 | Local |
| F1 | 04/24/2010 0:00 | 12 | 49.749340 | -109.327496 | 620483 | 5512104 | 1 | 15.53 | Local |
| F1 | 04/24/2010 0:00 | 15 | 49.749441 | -109.327646 | 620472 | 5512115 | 1 | 245.64 | Local |
| F1 | 04/24/2010 0:00 | 18 | 49.748038 | -109.325012 | 620665 | 5511963 | 1 | 1422.60 | Local |
| F1 | 04/24/2010 0:00 | 21 | 49.754400 | -109.307882 | 621883 | 5512698 | 1 | 1072.42 | Local |
| F1 | 04/25/2010 0:00 | 0 | 49.763483 | -109.302878 | 622220 | 5513716 | 1 | 1716.54 | Local |
| F1 | 04/25/2010 0:00 | 3 | 49.768940 | -109.280585 | 623812 | 5514359 | 1 | 3831.51 | Local |
| F1 | 04/25/2010 0:00 | 6 | 49.784375 | -109.233016 | 627197 | 5516155 | 0 | 2733.46 | Local |
| F1 | 04/25/2010 0:00 | 18 | 49.773216 | -109.199194 | 629661 | 5514972 | 1 | 1816.09 | Local |
| F1 | 04/25/2010 0:00 | 21 | 49.781921 | -109.177856 | 631174 | 5515977 | 1 | 445.41 | Local |
| F1 | 04/26/2010 0:00 | 0 | 49.783564 | -109.172214 | 631576 | 5516170 | 1 | 541.87 | Local |
| F1 | 04/26/2010 0:00 | 3 | 49.779403 | -109.168299 | 631869 | 5515714 | 1 | 577.32 | Local |
| F1 | 04/26/2010 0:00 | 6 | 49.774961 | -109.172450 | 631582 | 5515213 | 0 | 364.81 | Local |
| F1 | 04/26/2010 0:00 | 12 | 49.773055 | -109.176573 | 631290 | 5514994 | 1 | 20.01 | Local |
| F1 | 04/26/2010 0:00 | 15 | 49.772884 | -109.176657 | 631285 | 5514975 | 0 | 1243.00 | Local |
| F1 | 04/26/2010 0:00 | 21 | 49.767649 | -109.161409 | 632397 | 5514419 | 0 | 1953.29 | Local |
| F1 | 04/27/2010 0:00 | 3 | 49.753376 | -109.145606 | 633574 | 5512861 | 0 | 4420.96 | Local |
| F1 | 04/27/2010 0:00 | 9 | 49.762555 | -109.205315 | 629249 | 5513777 | 1 | 93.55 | Local |
| F1 | 04/27/2010 0:00 | 12 | 49.762394 | -109.204040 | 629341 | 5513761 | 1 | 17.39 | Local |
| F1 | 04/27/2010 0:00 | 15 | 49.762475 | -109.204247 | 629326 | 5513769 | 1 | 9.13 | Local |
| F1 | 04/27/2010 0:00 | 18 | 49.762457 | -109.204123 | 629335 | 5513768 | 1 | 313.93 | Local |
| F1 | 04/27/2010 0:00 | 21 | 49.761978 | -109.208418 | 629027 | 5513707 | 1 | 1756.79 | Local |
| F1 | 04/28/2010 0:00 | 0 | 49.767875 | -109.231044 | 627382 | 5514324 | 1 | 877.03 | Local |
| F1 | 04/28/2010 0:00 | 3 | 49.760540 | -109.235518 | 627079 | 5513501 | 1 | 1510.76 | Local |
| F1 | 04/28/2010 0:00 | 6 | 49.747147 | -109.239041 | 626860 | 5512006 | 1 | 168.45 | Local |
| F1 | 04/28/2010 0:00 | 9 | 49.745737 | -109.239892 | 626803 | 5511848 | 1 | 8.80 | Local |
| F1 | 04/28/2010 0:00 | 12 | 49.745806 | -109.239834 | 626807 | 5511856 | 1 | 1.64 | Local |
| F1 | 04/28/2010 0:00 | 15 | 49.745818 | -109.239819 | 626808 | 5511857 | 1 | 68.61 | Local |
| F1 | 04/28/2010 0:00 | 18 | 49.745297 | -109.240329 | 626772 | 5511798 | 1 | 202.62 | Local |
| F1 | 04/28/2010 0:00 | 21 | 49.746193 | -109.237881 | 626946 | 5511902 | 1 | 49.93 | Local |
| F1 | 04/29/2010 0:00 | 0 | 49.746352 | -109.237233 | 626992 | 5511921 | 1 | 1253.30 | Local |
| F1 | 04/29/2010 0:00 | 3 | 49.737250 | -109.226976 | 627755 | 5510926 | 1 | 4580.14 | Local |
| F1 | 04/29/2010 0:00 | 6 | 49.754823 | -109.169490 | 631850 | 5512979 | 1 | 3249.74 | Local |
| F1 | 04/29/2010 0:00 | 9 | 49.755782 | -109.124408 | 635094 | 5513166 | 1 | 1468.62 | Local |
| F1 | 04/29/2010 0:00 | 12 | 49.743646 | -109.116370 | 635707 | 5511831 | 1 | 423.37 | Local |
| F1 | 04/29/2010 0:00 | 15 | 49.742689 | -109.110684 | 636119 | 5511735 | 1 | 2.68 | Local |
| F1 | 04/29/2010 0:00 | 18 | 49.742679 | -109.110717 | 636117 | 5511734 | 1 | 7.01 | Local |
| F1 | 04/29/2010 0:00 | 21 | 49.742711 | -109.110801 | 636111 | 5511738 | 1 | 15.02 | Local |
| F1 | 04/30/2010 0:00 | 0 | 49.742776 | -109.110618 | 636124 | 5511745 | 1 | 9.59 | Local |
| F1 | 04/30/2010 0:00 | 3 | 49.742851 | -109.110553 | 636128 | 5511754 | 1 | 29.90 | Local |

| | | | | | | | | | |
|---|---|---|---|---|---|---|---|---|---|
| F1 | 04/30/2010 0:00 | 6 | 49.742610 | -109.110735 | 636116 | 5511726 | 1 | 6.15 | Local |
| F1 | 04/30/2010 0:00 | 9 | 49.742655 | -109.110784 | 636112 | 5511731 | 1 | 9.09 | Local |
| F1 | 04/30/2010 0:00 | 12 | 49.742717 | -109.110701 | 636118 | 5511738 | 1 | 4.87 | Local |
| F1 | 04/30/2010 0:00 | 15 | 49.742750 | -109.110658 | 636121 | 5511742 | 1 | 7.35 | Local |
| F1 | 04/30/2010 0:00 | 18 | 49.742688 | -109.110692 | 636118 | 5511735 | 0 | 227.44 | Local |
| F1 | 05/02/2010 0:00 | 15 | 49.741220 | -109.112888 | 635964 | 5511568 | 1 | 34.20 | Local |
| F1 | 05/02/2010 0:00 | 18 | 49.741469 | -109.112611 | 635984 | 5511596 | 1 | 728.65 | Local |
| F1 | 05/02/2010 0:00 | 21 | 49.746630 | -109.106381 | 636418 | 5512181 | 1 | 1357.39 | Local |
| F1 | 05/03/2010 0:00 | 0 | 49.745078 | -109.087697 | 637768 | 5512043 | 1 | 1517.90 | Local |
| F1 | 05/03/2010 0:00 | 3 | 49.755063 | -109.102060 | 636706 | 5513127 | 1 | 520.73 | Local |
| F1 | 05/03/2010 0:00 | 6 | 49.755615 | -109.109237 | 636187 | 5513175 | 1 | 225.26 | Local |
| F1 | 05/03/2010 0:00 | 9 | 49.755191 | -109.112295 | 635968 | 5513122 | 0 | 19.68 | Local |
| F1 | 05/03/2010 0:00 | 15 | 49.755364 | -109.112353 | 635963 | 5513141 | 1 | 10.74 | Local |
| F1 | 05/03/2010 0:00 | 18 | 49.755397 | -109.112213 | 635973 | 5513145 | 1 | 309.34 | Local |
| F1 | 05/03/2010 0:00 | 21 | 49.755698 | -109.107945 | 636280 | 5513186 | 1 | 97.75 | Local |
| F1 | 05/04/2010 0:00 | 0 | 49.756533 | -109.108368 | 636247 | 5513279 | 0 | 1758.85 | Local |
| F1 | 05/04/2010 0:00 | 18 | 49.741618 | -109.116491 | 635704 | 5511606 | 1 | 343.35 | Local |
| F1 | 05/04/2010 0:00 | 21 | 49.742638 | -109.120989 | 635377 | 5511711 | 0 | 21.98 | Local |
| F1 | 05/05/2010 0:00 | 6 | 49.742675 | -109.120689 | 635398 | 5511716 | 1 | 872.15 | Local |
| F1 | 05/05/2010 0:00 | 9 | 49.750022 | -109.116453 | 635683 | 5512540 | 0 | 1934.46 | Local |
| F1 | 05/05/2010 0:00 | 15 | 49.756698 | -109.141246 | 633879 | 5513238 | 1 | 807.11 | Local |
| F1 | 05/05/2010 0:00 | 18 | 49.757671 | -109.152348 | 633076 | 5513326 | 1 | 2051.65 | Local |
| F1 | 05/05/2010 0:00 | 21 | 49.753760 | -109.180178 | 631083 | 5512842 | 1 | 1327.68 | Local |
| F1 | 05/06/2010 0:00 | 0 | 49.755446 | -109.198422 | 629764 | 5512998 | 1 | 481.88 | Local |
| F1 | 05/06/2010 0:00 | 3 | 49.758502 | -109.203164 | 629414 | 5513330 | 1 | 679.15 | Local |
| F1 | 05/06/2010 0:00 | 6 | 49.763375 | -109.208847 | 628992 | 5513862 | 1 | 6.21 | Local |
| F1 | 05/06/2010 0:00 | 9 | 49.763325 | -109.208885 | 628990 | 5513856 | 1 | 12.01 | Local |
| F1 | 05/06/2010 0:00 | 12 | 49.763342 | -109.208720 | 629001 | 5513858 | 1 | 96.59 | Local |
| F1 | 05/06/2010 0:00 | 15 | 49.763843 | -109.209815 | 628921 | 5513912 | 1 | 17.97 | Local |
| F1 | 05/06/2010 0:00 | 18 | 49.763920 | -109.209596 | 628937 | 5513921 | 1 | 791.93 | Local |
| F1 | 05/06/2010 0:00 | 21 | 49.764650 | -109.220532 | 628147 | 5513983 | 1 | 1864.30 | Local |
| F1 | 05/07/2010 0:00 | 0 | 49.755702 | -109.242417 | 626595 | 5512951 | 1 | 388.39 | Local |
| F1 | 05/07/2010 0:00 | 3 | 49.753512 | -109.246618 | 626298 | 5512701 | 1 | 8.99 | Local |
| F1 | 05/07/2010 0:00 | 6 | 49.753470 | -109.246512 | 626306 | 5512696 | 1 | 256.72 | Local |
| F1 | 05/07/2010 0:00 | 9 | 49.755031 | -109.243887 | 626491 | 5512874 | 0 | 36.50 | Local |
| F1 | 05/07/2010 0:00 | 15 | 49.755160 | -109.244353 | 626457 | 5512888 | 1 | 236.88 | Local |
| F1 | 05/07/2010 0:00 | 18 | 49.753611 | -109.246610 | 626298 | 5512712 | 1 | 21.95 | Local |
| F1 | 05/07/2010 0:00 | 21 | 49.753438 | -109.246756 | 626288 | 5512692 | 1 | 13.35 | Local |
| F1 | 05/08/2010 0:00 | 0 | 49.753409 | -109.246576 | 626301 | 5512690 | 1 | 17.75 | Local |
| F1 | 05/08/2010 0:00 | 3 | 49.753565 | -109.246523 | 626305 | 5512707 | 1 | 15.15 | Local |
| F1 | 05/08/2010 0:00 | 6 | 49.753445 | -109.246622 | 626298 | 5512693 | 1 | 36.42 | Local |
| F1 | 05/08/2010 0:00 | 9 | 49.753674 | -109.246261 | 626323 | 5512720 | 1 | 27.35 | Local |
| F1 | 05/08/2010 0:00 | 12 | 49.753623 | -109.246632 | 626297 | 5512713 | 0 | 13.69 | Local |
| F1 | 05/08/2010 0:00 | 18 | 49.753509 | -109.246561 | 626302 | 5512701 | 1 | 13.74 | Local |
| F1 | 05/08/2010 0:00 | 21 | 49.753428 | -109.246704 | 626292 | 5512691 | 1 | 9.00 | Local |
| F1 | 05/09/2010 0:00 | 0 | 49.753439 | -109.246580 | 626301 | 5512693 | 1 | 8.90 | Local |
| F1 | 05/09/2010 0:00 | 3 | 49.753495 | -109.246669 | 626294 | 5512699 | 1 | 5.51 | Local |
| F1 | 05/09/2010 0:00 | 6 | 49.753542 | -109.246647 | 626296 | 5512704 | 1 | 231.51 | Local |
| F1 | 05/09/2010 0:00 | 9 | 49.755021 | -109.244384 | 626455 | 5512872 | 1 | 5.90 | Local |
| F1 | 05/09/2010 0:00 | 12 | 49.754995 | -109.244312 | 626460 | 5512870 | 1 | 238.36 | Local |
| F1 | 05/09/2010 0:00 | 15 | 49.753506 | -109.246691 | 626293 | 5512700 | 1 | 9.05 | Local |
| F1 | 05/09/2010 0:00 | 18 | 49.753427 | -109.246661 | 626295 | 5512691 | 1 | 9.43 | Local |
| F1 | 05/09/2010 0:00 | 21 | 49.753503 | -109.246605 | 626299 | 5512700 | 1 | 8.17 | Local |
| F1 | 05/10/2010 0:00 | 0 | 49.753430 | -109.246616 | 626298 | 5512692 | 1 | 1.08 | Local |
| F1 | 05/10/2010 0:00 | 3 | 49.753421 | -109.246620 | 626298 | 5512691 | 1 | 8.61 | Local |

| | | | | | | | | | |
|---|---|---|---|---|---|---|---|---|---|
| F1 | 05/10/2010 0:00 | 6 | 49.753476 | -109.246704 | 626292 | 5512697 | 1 | 251.79 | Local |
| F1 | 05/10/2010 0:00 | 9 | 49.755072 | -109.244224 | 626466 | 5512878 | 0 | 8.32 | Local |
| F1 | 05/10/2010 0:00 | 15 | 49.755143 | -109.244190 | 626468 | 5512886 | 1 | 241.96 | Local |
| F1 | 05/10/2010 0:00 | 18 | 49.753586 | -109.246536 | 626304 | 5512709 | 1 | 22.72 | Local |
| F1 | 05/10/2010 0:00 | 21 | 49.753407 | -109.246688 | 626293 | 5512689 | 1 | 10.28 | Local |
| F1 | 05/11/2010 0:00 | 0 | 49.753450 | -109.246561 | 626302 | 5512694 | 1 | 4.09 | Local |
| F1 | 05/11/2010 0:00 | 3 | 49.753425 | -109.246603 | 626299 | 5512691 | 0 | 253.26 | Local |
| F1 | 05/11/2010 0:00 | 9 | 49.755090 | -109.244205 | 626468 | 5512880 | 0 | 2.41 | Local |
| F1 | 05/11/2010 0:00 | 15 | 49.755099 | -109.244235 | 626465 | 5512881 | 1 | 241.93 | Local |
| F1 | 05/11/2010 0:00 | 18 | 49.753513 | -109.246533 | 626304 | 5512701 | 1 | 13.82 | Local |
| F1 | 05/11/2010 0:00 | 21 | 49.753625 | -109.246614 | 626298 | 5512713 | 1 | 18.32 | Local |
| F1 | 05/12/2010 0:00 | 0 | 49.753478 | -109.246501 | 626306 | 5512697 | 1 | 13.77 | Local |
| F1 | 05/12/2010 0:00 | 3 | 49.753417 | -109.246668 | 626294 | 5512690 | 1 | 21.89 | Local |
| F1 | 05/12/2010 0:00 | 6 | 49.753596 | -109.246544 | 626303 | 5512710 | 1 | 27.71 | Local |
| F1 | 05/12/2010 0:00 | 9 | 49.753426 | -109.246824 | 626283 | 5512691 | 1 | 15.99 | Local |
| F1 | 05/12/2010 0:00 | 12 | 49.753483 | -109.246620 | 626298 | 5512698 | 1 | 8.51 | Local |
| F1 | 05/12/2010 0:00 | 15 | 49.753512 | -109.246511 | 626306 | 5512701 | 1 | 22.31 | Local |
| F1 | 05/12/2010 0:00 | 18 | 49.753417 | -109.246784 | 626286 | 5512690 | 1 | 15.81 | Local |
| F1 | 05/12/2010 0:00 | 21 | 49.753500 | -109.246606 | 626299 | 5512700 | 1 | 10.39 | Local |
| F1 | 05/13/2010 0:00 | 0 | 49.753568 | -109.246507 | 626306 | 5512707 | 1 | 13.27 | Local |
| F1 | 05/13/2010 0:00 | 3 | 49.753451 | -109.246539 | 626304 | 5512694 | 1 | 14.33 | Local |
| F1 | 05/13/2010 0:00 | 6 | 49.753325 | -109.246584 | 626301 | 5512680 | 1 | 268.11 | Local |
| F1 | 05/13/2010 0:00 | 9 | 49.755203 | -109.244250 | 626464 | 5512893 | 1 | 18.83 | Local |
| F1 | 05/13/2010 0:00 | 12 | 49.755074 | -109.244420 | 626452 | 5512878 | 1 | 5.85 | Local |
| F1 | 05/13/2010 0:00 | 15 | 49.755115 | -109.244369 | 626456 | 5512883 | 1 | 189.47 | Local |
| F1 | 05/13/2010 0:00 | 18 | 49.753639 | -109.245683 | 626365 | 5512717 | 1 | 58.86 | Local |
| F1 | 05/13/2010 0:00 | 21 | 49.753267 | -109.246264 | 626324 | 5512674 | 1 | 33.26 | Local |
| F1 | 05/14/2010 0:00 | 0 | 49.753476 | -109.246594 | 626300 | 5512697 | 1 | 5.26 | Local |
| F1 | 05/14/2010 0:00 | 3 | 49.753484 | -109.246666 | 626294 | 5512698 | 1 | 768.32 | Local |
| F1 | 05/14/2010 0:00 | 6 | 49.749740 | -109.237703 | 626950 | 5512297 | 1 | 162.72 | Local |
| F1 | 05/14/2010 0:00 | 9 | 49.749897 | -109.235458 | 627111 | 5512318 | 0 | 203.82 | Local |
| F1 | 05/14/2010 0:00 | 15 | 49.751611 | -109.234457 | 627179 | 5512510 | 1 | 483.94 | Local |
| F1 | 05/14/2010 0:00 | 18 | 49.753007 | -109.228095 | 627633 | 5512676 | 1 | 1350.30 | Local |
| F1 | 05/14/2010 0:00 | 21 | 49.759391 | -109.212152 | 628765 | 5513413 | 1 | 888.57 | Local |
| F1 | 05/15/2010 0:00 | 0 | 49.766673 | -109.207074 | 629111 | 5514231 | 1 | 661.09 | Local |
| F1 | 05/15/2010 0:00 | 3 | 49.772065 | -109.203209 | 629375 | 5514837 | 1 | 354.09 | Local |
| F1 | 05/15/2010 0:00 | 6 | 49.773801 | -109.199087 | 629667 | 5515038 | 1 | 22.02 | Local |
| F1 | 05/15/2010 0:00 | 9 | 49.773860 | -109.198795 | 629688 | 5515045 | 1 | 25.91 | Local |
| F1 | 05/15/2010 0:00 | 12 | 49.773830 | -109.198439 | 629714 | 5515042 | 1 | 321.38 | Local |
| F1 | 05/15/2010 0:00 | 15 | 49.771673 | -109.195469 | 629933 | 5514807 | 1 | 46.62 | Local |
| F1 | 05/15/2010 0:00 | 18 | 49.771433 | -109.196001 | 629896 | 5514780 | 1 | 2325.20 | Local |
| F1 | 05/15/2010 0:00 | 21 | 49.780603 | -109.166983 | 631960 | 5515850 | 1 | 1637.80 | Local |
| F1 | 05/16/2010 0:00 | 0 | 49.774470 | -109.146306 | 633465 | 5515204 | 1 | 1542.21 | Local |
| F1 | 05/16/2010 0:00 | 3 | 49.769009 | -109.126623 | 634898 | 5514633 | 1 | 2964.91 | Local |
| F1 | 05/16/2010 0:00 | 6 | 49.743909 | -109.112746 | 635967 | 5511867 | 1 | 484.49 | Local |
| F1 | 05/16/2010 0:00 | 9 | 49.740275 | -109.109037 | 636244 | 5511470 | 1 | 11.27 | Local |
| F1 | 05/16/2010 0:00 | 12 | 49.740174 | -109.109050 | 636244 | 5511459 | 0 | 11.12 | Local |
| F1 | 05/16/2010 0:00 | 18 | 49.740273 | -109.109077 | 636242 | 5511470 | 1 | 164.41 | Local |
| F1 | 05/16/2010 0:00 | 21 | 49.741504 | -109.110339 | 636147 | 5511604 | 1 | 334.91 | Local |
| F1 | 05/17/2010 0:00 | 0 | 49.738554 | -109.109400 | 636223 | 5511278 | 0 | 402.87 | Local |
| F1 | 05/17/2010 0:00 | 15 | 49.741649 | -109.112307 | 636005 | 5511617 | 1 | 20.73 | Local |
| F1 | 05/17/2010 0:00 | 18 | 49.741544 | -109.112545 | 635988 | 5511605 | 1 | 1148.82 | Local |
| F1 | 05/17/2010 0:00 | 21 | 49.750966 | -109.119082 | 635491 | 5512640 | 1 | 1646.93 | Local |
| F1 | 05/18/2010 0:00 | 0 | 49.757020 | -109.139944 | 633972 | 5513276 | 1 | 1961.19 | Local |
| F1 | 05/18/2010 0:00 | 3 | 49.761010 | -109.166462 | 632051 | 5513672 | 1 | 935.73 | Local |

| | | | | | | | | | |
|---|---|---|---|---|---|---|---|---|---|
| F1 | 05/18/2010 0:00 | 6 | 49.762514 | -109.179242 | 631126 | 5513817 | 1 | 2227.81 | Local |
| F1 | 05/18/2010 0:00 | 9 | 49.770765 | -109.207426 | 629075 | 5514686 | 0 | 76.70 | Local |
| F1 | 05/18/2010 0:00 | 15 | 49.771425 | -109.207735 | 629051 | 5514758 | 1 | 13.09 | Local |
| F1 | 05/18/2010 0:00 | 18 | 49.771311 | -109.207690 | 629054 | 5514746 | 1 | 543.20 | Local |
| F1 | 05/18/2010 0:00 | 21 | 49.770040 | -109.200408 | 629582 | 5514617 | 1 | 695.88 | Local |
| F1 | 05/19/2010 0:00 | 0 | 49.773377 | -109.192234 | 630162 | 5515002 | 1 | 1355.09 | Local |
| F1 | 05/19/2010 0:00 | 3 | 49.783431 | -109.202867 | 629369 | 5516102 | 1 | 136.68 | Local |
| F1 | 05/19/2010 0:00 | 6 | 49.783543 | -109.200976 | 629505 | 5516117 | 1 | 623.15 | Local |
| F1 | 05/19/2010 0:00 | 9 | 49.779863 | -109.207503 | 629045 | 5515697 | 0 | 52.14 | Local |
| F1 | 05/19/2010 0:00 | 18 | 49.780084 | -109.206864 | 629091 | 5515723 | 1 | 2.28 | Local |
| F1 | 05/19/2010 0:00 | 21 | 49.780069 | -109.206885 | 629089 | 5515721 | 1 | 9.44 | Local |
| F1 | 05/20/2010 0:00 | 0 | 49.780122 | -109.206987 | 629082 | 5515727 | 1 | 8.52 | Local |
| F1 | 05/20/2010 0:00 | 3 | 49.780065 | -109.206909 | 629087 | 5515720 | 1 | 763.13 | Local |
| F1 | 05/20/2010 0:00 | 6 | 49.773203 | -109.207032 | 629097 | 5514957 | 1 | 191.20 | Local |
| F1 | 05/20/2010 0:00 | 9 | 49.771485 | -109.206897 | 629111 | 5514767 | 0 | 49.80 | Local |
| F1 | 05/20/2010 0:00 | 18 | 49.771929 | -109.206998 | 629103 | 5514816 | 1 | 563.79 | Local |
| F1 | 05/20/2010 0:00 | 21 | 49.771538 | -109.214803 | 628542 | 5514759 | 1 | 2271.92 | Local |
| F1 | 05/21/2010 0:00 | 0 | 49.783973 | -109.239836 | 626707 | 5516099 | 1 | 791.72 | Local |
| F1 | 05/21/2010 0:00 | 3 | 49.787437 | -109.230229 | 627389 | 5516500 | 1 | 1283.56 | Local |
| F1 | 05/21/2010 0:00 | 6 | 49.779170 | -109.242669 | 626515 | 5515560 | 1 | 1172.46 | Local |
| F1 | 05/21/2010 0:00 | 9 | 49.771411 | -109.253694 | 625742 | 5514679 | 0 | 271.06 | Local |
| F1 | 05/21/2010 0:00 | 15 | 49.768974 | -109.253682 | 625749 | 5514408 | 1 | 9.44 | Local |
| F1 | 05/21/2010 0:00 | 18 | 49.768915 | -109.253777 | 625742 | 5514401 | 1 | 673.58 | Local |
| F1 | 05/21/2010 0:00 | 21 | 49.772098 | -109.261735 | 625161 | 5514742 | 1 | 1534.21 | Local |
| F1 | 05/22/2010 0:00 | 0 | 49.761058 | -109.274511 | 624269 | 5513493 | 1 | 1805.24 | Local |
| F1 | 05/22/2010 0:00 | 3 | 49.753255 | -109.296485 | 622707 | 5512590 | 1 | 1444.94 | Local |
| F1 | 05/22/2010 0:00 | 6 | 49.755809 | -109.316149 | 621284 | 5512842 | 0 | 1732.89 | Local |
| F1 | 05/22/2010 0:00 | 15 | 49.744496 | -109.332690 | 620121 | 5511557 | 1 | 1549.55 | Local |
| F1 | 05/22/2010 0:00 | 18 | 49.748352 | -109.312025 | 621600 | 5512019 | 0 | 518.49 | Local |
| F1 | 05/23/2010 0:00 | 0 | 49.752180 | -109.307915 | 621886 | 5512451 | 1 | 338.23 | Local |
| F1 | 05/23/2010 0:00 | 3 | 49.755205 | -109.307425 | 621914 | 5512788 | 1 | 316.73 | Local |
| F1 | 05/23/2010 0:00 | 6 | 49.755256 | -109.311820 | 621597 | 5512787 | 1 | 24.41 | Local |
| F1 | 05/23/2010 0:00 | 9 | 49.755079 | -109.312021 | 621583 | 5512767 | 0 | 12.25 | Local |
| F1 | 05/23/2010 0:00 | 15 | 49.755170 | -109.311925 | 621590 | 5512777 | 1 | 15.19 | Local |
| F1 | 05/23/2010 0:00 | 18 | 49.755048 | -109.312021 | 621583 | 5512764 | 1 | 6.25 | Local |
| F1 | 05/23/2010 0:00 | 21 | 49.755031 | -109.312104 | 621577 | 5512762 | 1 | 5.75 | Local |
| F1 | 05/24/2010 0:00 | 0 | 49.755075 | -109.312061 | 621580 | 5512767 | 1 | 8.46 | Local |
| F1 | 05/24/2010 0:00 | 3 | 49.754999 | -109.312054 | 621581 | 5512758 | 1 | 5.25 | Local |
| F1 | 05/24/2010 0:00 | 6 | 49.754990 | -109.311983 | 621586 | 5512757 | 1 | 7.97 | Local |
| F1 | 05/24/2010 0:00 | 9 | 49.755047 | -109.311917 | 621591 | 5512764 | 0 | 18.39 | Local |
| F1 | 05/24/2010 0:00 | 15 | 49.754951 | -109.311710 | 621606 | 5512753 | 1 | 51.18 | Local |
| F1 | 05/24/2010 0:00 | 18 | 49.755156 | -109.312346 | 621560 | 5512775 | 1 | 19.67 | Local |
| F1 | 05/24/2010 0:00 | 21 | 49.754984 | -109.312284 | 621564 | 5512756 | 1 | 50.23 | Local |
| F1 | 05/25/2010 0:00 | 0 | 49.755291 | -109.311772 | 621600 | 5512791 | 1 | 10.15 | Local |
| F1 | 05/25/2010 0:00 | 3 | 49.755322 | -109.311640 | 621610 | 5512795 | 1 | 11.55 | Local |
| F1 | 05/25/2010 0:00 | 6 | 49.755423 | -109.311680 | 621607 | 5512806 | 1 | 48.33 | Local |
| F1 | 05/25/2010 0:00 | 9 | 49.755099 | -109.312129 | 621575 | 5512769 | 1 | 19.57 | Local |
| F1 | 05/25/2010 0:00 | 12 | 49.755166 | -109.311877 | 621593 | 5512777 | 0 | 6.29 | Local |
| F1 | 05/25/2010 0:00 | 18 | 49.755161 | -109.311964 | 621587 | 5512776 | 1 | 31.30 | Local |
| F1 | 05/25/2010 0:00 | 21 | 49.755225 | -109.312388 | 621556 | 5512783 | 1 | 9.41 | Local |
| F1 | 05/26/2010 0:00 | 0 | 49.755169 | -109.312289 | 621564 | 5512777 | 0 | 386.51 | Local |
| F1 | 05/26/2010 0:00 | 6 | 49.757018 | -109.307746 | 621886 | 5512990 | 1 | 149.99 | Local |
| F1 | 05/26/2010 0:00 | 9 | 49.757133 | -109.305672 | 622035 | 5513006 | 0 | 3591.77 | Local |
| F1 | 05/26/2010 0:00 | 15 | 49.769270 | -109.259463 | 625332 | 5514431 | 1 | 1690.69 | Local |
| F1 | 05/26/2010 0:00 | 18 | 49.771457 | -109.236232 | 626999 | 5514713 | 1 | 1255.49 | Local |

| | | | | | | | | | | |
|---|---|---|---|---|---|---|---|---|---|---|
| F1 | 05/26/2010 0:00 | 21 | 49.770939 | -109.218818 | 628254 | 5514685 | | 1 | 1088.82 | Local |
| F1 | 05/27/2010 0:00 | 0 | 49.762956 | -109.210066 | 628906 | 5513813 | | 1 | 2178.67 | Local |
| F1 | 05/27/2010 0:00 | 3 | 49.755740 | -109.181949 | 630950 | 5513059 | | 1 | 3016.48 | Local |
| F1 | 05/27/2010 0:00 | 6 | 49.757005 | -109.140125 | 633959 | 5513274 | | 1 | 167.98 | Local |
| F1 | 05/27/2010 0:00 | 9 | 49.757212 | -109.137815 | 634124 | 5513301 | | 1 | 153.57 | Local |
| F1 | 05/27/2010 0:00 | 12 | 49.756973 | -109.139915 | 633974 | 5513271 | | 1 | 539.12 | Local |
| F1 | 05/27/2010 0:00 | 15 | 49.754224 | -109.133751 | 634425 | 5512976 | | 1 | 11.40 | Local |
| F1 | 05/27/2010 0:00 | 18 | 49.754145 | -109.133853 | 634418 | 5512967 | | 1 | 4.08 | Local |
| F1 | 05/27/2010 0:00 | 21 | 49.754181 | -109.133862 | 634417 | 5512971 | | 1 | 3.09 | Local |
| F1 | 05/28/2010 0:00 | 0 | 49.754166 | -109.133898 | 634415 | 5512969 | | 1 | 7.19 | Local |
| F1 | 05/28/2010 0:00 | 3 | 49.754130 | -109.133816 | 634421 | 5512965 | | 1 | 119.43 | Local |
| F1 | 05/28/2010 0:00 | 6 | 49.754210 | -109.132163 | 634540 | 5512977 | | 1 | 1831.83 | Local |
| F1 | 05/28/2010 0:00 | 9 | 49.743727 | -109.112553 | 635981 | 5511847 | | 1 | 41.42 | Local |
| F1 | 05/28/2010 0:00 | 12 | 49.743817 | -109.113111 | 635941 | 5511856 | | 1 | 642.64 | Local |
| F1 | 05/28/2010 0:00 | 15 | 49.745290 | -109.121734 | 635316 | 5512004 | | 1 | 1107.27 | Local |
| F1 | 05/28/2010 0:00 | 18 | 49.754402 | -109.127930 | 634844 | 5513006 | | 1 | 13.18 | Local |
| F1 | 05/28/2010 0:00 | 21 | 49.754305 | -109.128036 | 634837 | 5512995 | | 1 | 425.06 | Local |
| F1 | 05/29/2010 0:00 | 0 | 49.754171 | -109.133932 | 634412 | 5512970 | | 1 | 541.31 | Local |
| F1 | 05/29/2010 0:00 | 3 | 49.757061 | -109.139978 | 633969 | 5513280 | | 1 | 3706.53 | Local |
| F1 | 05/29/2010 0:00 | 6 | 49.768566 | -109.188270 | 630460 | 5514474 | | 0 | 1447.89 | Local |
| F1 | 05/29/2010 0:00 | 12 | 49.771261 | -109.207938 | 629037 | 5514740 | | 1 | 11.66 | Local |
| F1 | 05/29/2010 0:00 | 15 | 49.771201 | -109.207805 | 629046 | 5514733 | | 1 | 625.78 | Local |
| F1 | 05/29/2010 0:00 | 18 | 49.765970 | -109.211006 | 628830 | 5514146 | | 1 | 317.71 | Local |
| F1 | 05/29/2010 0:00 | 21 | 49.766006 | -109.206595 | 629147 | 5514158 | | 1 | 16.09 | Local |
| F1 | 05/30/2010 0:00 | 0 | 49.765937 | -109.206792 | 629133 | 5514150 | | 1 | 11.72 | Local |
| F1 | 05/30/2010 0:00 | 3 | 49.766042 | -109.206796 | 629133 | 5514162 | | 1 | 2.79 | Local |
| F1 | 05/30/2010 0:00 | 6 | 49.766064 | -109.206775 | 629134 | 5514164 | | 1 | 621.07 | Local |
| F1 | 05/30/2010 0:00 | 9 | 49.771595 | -109.207965 | 629034 | 5514777 | | 1 | 29.77 | Local |
| F1 | 05/30/2010 0:00 | 12 | 49.771402 | -109.207679 | 629055 | 5514756 | | 1 | 1352.29 | Local |
| F1 | 05/30/2010 0:00 | 15 | 49.776932 | -109.190955 | 630244 | 5515400 | | 1 | 1672.71 | Local |
| F1 | 05/30/2010 0:00 | 18 | 49.782210 | -109.169203 | 631796 | 5516024 | | 1 | 1675.81 | Local |
| F1 | 05/30/2010 0:00 | 21 | 49.771321 | -109.153117 | 632984 | 5514842 | | 1 | 1601.69 | Local |
| F1 | 05/31/2010 0:00 | 0 | 49.759270 | -109.140939 | 633894 | 5513524 | | 1 | 250.10 | Local |
| F1 | 05/31/2010 0:00 | 3 | 49.757091 | -109.140079 | 633962 | 5513284 | | 1 | 793.06 | Local |
| F1 | 05/31/2010 0:00 | 6 | 49.753830 | -109.130289 | 634676 | 5512938 | | 1 | 1864.82 | Local |
| F1 | 05/31/2010 0:00 | 9 | 49.741641 | -109.112516 | 635990 | 5511615 | | 1 | 58.24 | Local |
| F1 | 05/31/2010 0:00 | 12 | 49.741223 | -109.113004 | 635956 | 5511568 | | 1 | 6.41 | Local |
| F1 | 05/31/2010 0:00 | 15 | 49.741205 | -109.113089 | 635950 | 5511566 | | 1 | 732.70 | Local |
| F1 | 05/31/2010 0:00 | 18 | 49.747022 | -109.108313 | 636278 | 5512221 | | 1 | 1331.31 | Local |
| F1 | 05/31/2010 0:00 | 21 | 49.756976 | -109.118578 | 635510 | 5513309 | | 1 | 2689.47 | Local |
| F1 | 06/01/2010 0:00 | 0 | 49.750134 | -109.154382 | 632951 | 5512485 | | 1 | 4157.35 | Local |
| F1 | 06/01/2010 0:00 | 3 | 49.762666 | -109.208749 | 629001 | 5513783 | | 1 | 37.58 | Local |
| F1 | 06/01/2010 0:00 | 6 | 49.762497 | -109.208297 | 629034 | 5513765 | | 1 | 20.77 | Local |
| F1 | 06/01/2010 0:00 | 9 | 49.762648 | -109.208467 | 629021 | 5513781 | | 1 | 123.42 | Local |
| F1 | 06/01/2010 0:00 | 12 | 49.763608 | -109.209325 | 628957 | 5513887 | | 1 | 18.73 | Local |
| F1 | 06/01/2010 0:00 | 15 | 49.763536 | -109.209560 | 628940 | 5513878 | | 1 | 110.42 | Local |
| F1 | 06/01/2010 0:00 | 18 | 49.762686 | -109.208767 | 629000 | 5513785 | | 1 | 41.91 | Local |
| F1 | 06/01/2010 0:00 | 21 | 49.762577 | -109.208210 | 629040 | 5513774 | | 1 | 8.61 | Local |
| F1 | 06/02/2010 0:00 | 0 | 49.762567 | -109.208328 | 629032 | 5513773 | | 1 | 2.38 | Local |
| F1 | 06/02/2010 0:00 | 3 | 49.762559 | -109.208298 | 629034 | 5513772 | | 1 | 3.71 | Local |
| F1 | 06/02/2010 0:00 | 6 | 49.762570 | -109.208249 | 629037 | 5513773 | | 1 | 26.30 | Local |
| F1 | 06/02/2010 0:00 | 9 | 49.762696 | -109.208559 | 629015 | 5513787 | | 1 | 10.91 | Local |
| F1 | 06/02/2010 0:00 | 12 | 49.762599 | -109.208587 | 629013 | 5513776 | | 1 | 22.56 | Local |
| F1 | 06/02/2010 0:00 | 15 | 49.762529 | -109.208293 | 629034 | 5513769 | | 1 | 4.70 | Local |
| F1 | 06/02/2010 0:00 | 18 | 49.762526 | -109.208358 | 629030 | 5513768 | | 1 | 49.21 | Local |

| | | | | | | | | | |
|---|---|---|---|---|---|---|---|---|---|
| F1 | 06/02/2010 0:00 | 21 | 49.762237 | -109.207840 | 629068 | 5513737 | 1 | 67.08 | Local |
| F1 | 06/03/2010 0:00 | 0 | 49.762521 | -109.208661 | 629008 | 5513767 | 1 | 400.99 | Local |
| F1 | 06/03/2010 0:00 | 3 | 49.766037 | -109.207425 | 629087 | 5514160 | 1 | 306.93 | Local |
| F1 | 06/03/2010 0:00 | 6 | 49.768296 | -109.209876 | 628905 | 5514407 | 1 | 2921.07 | Local |
| F1 | 06/03/2010 0:00 | 9 | 49.748747 | -109.236961 | 627006 | 5512187 | 1 | 13.74 | Local |
| F1 | 06/03/2010 0:00 | 12 | 49.748860 | -109.237037 | 627000 | 5512200 | 1 | 19.53 | Local |
| F1 | 06/03/2010 0:00 | 15 | 49.748685 | -109.237016 | 627002 | 5512180 | 1 | 185.31 | Local |
| F1 | 06/03/2010 0:00 | 18 | 49.750345 | -109.237248 | 626981 | 5512365 | 1 | 2007.12 | Local |
| F1 | 06/03/2010 0:00 | 21 | 49.767661 | -109.245109 | 626370 | 5514276 | 0 | 712.02 | Local |
| F1 | 06/04/2010 0:00 | 3 | 49.771305 | -109.253238 | 625775 | 5514668 | 0 | 535.08 | Local |
| F1 | 06/04/2010 0:00 | 9 | 49.769458 | -109.260098 | 625286 | 5514451 | 1 | 285.73 | Local |
| F1 | 06/04/2010 0:00 | 12 | 49.768841 | -109.263949 | 625010 | 5514376 | 0 | 221.10 | Local |
| F1 | 06/04/2010 0:00 | 18 | 49.768875 | -109.260880 | 625231 | 5514385 | 1 | 485.23 | Local |
| F1 | 06/04/2010 0:00 | 21 | 49.766594 | -109.266623 | 624823 | 5514122 | 1 | 72.60 | Local |
| F1 | 06/05/2010 0:00 | 0 | 49.766041 | -109.267159 | 624786 | 5514059 | 1 | 6.82 | Local |
| F1 | 06/05/2010 0:00 | 3 | 49.766086 | -109.267094 | 624791 | 5514064 | 1 | 170.49 | Local |
| F1 | 06/05/2010 0:00 | 6 | 49.766960 | -109.265150 | 624928 | 5514165 | 1 | 441.02 | Local |
| F1 | 06/05/2010 0:00 | 9 | 49.769286 | -109.260190 | 625280 | 5514432 | 1 | 38.39 | Local |
| F1 | 06/05/2010 0:00 | 12 | 49.769053 | -109.259797 | 625309 | 5514406 | 1 | 9.79 | Local |
| F1 | 06/05/2010 0:00 | 15 | 49.769019 | -109.259922 | 625300 | 5514403 | 1 | 156.57 | Local |
| F1 | 06/05/2010 0:00 | 18 | 49.768365 | -109.261847 | 625163 | 5514327 | 1 | 389.70 | Local |
| F1 | 06/05/2010 0:00 | 21 | 49.766186 | -109.266085 | 624863 | 5514077 | 1 | 83.74 | Local |
| F1 | 06/06/2010 0:00 | 0 | 49.766040 | -109.267225 | 624781 | 5514059 | 1 | 8.24 | Local |
| F1 | 06/06/2010 0:00 | 3 | 49.766033 | -109.267111 | 624790 | 5514059 | 1 | 521.72 | Local |
| F1 | 06/06/2010 0:00 | 6 | 49.769018 | -109.261523 | 625184 | 5514400 | 0 | 126.46 | Local |
| F1 | 06/06/2010 0:00 | 12 | 49.768789 | -109.259803 | 625309 | 5514377 | 1 | 9.20 | Local |
| F1 | 06/06/2010 0:00 | 15 | 49.768708 | -109.259832 | 625307 | 5514368 | 1 | 368.71 | Local |
| F1 | 06/06/2010 0:00 | 18 | 49.768008 | -109.264835 | 624948 | 5514282 | 1 | 267.48 | Local |
| F1 | 06/06/2010 0:00 | 21 | 49.766240 | -109.267353 | 624772 | 5514081 | 0 | 516.31 | Local |
| F1 | 06/07/2010 0:00 | 3 | 49.769114 | -109.261723 | 625170 | 5514410 | 1 | 1774.35 | Local |
| F1 | 06/07/2010 0:00 | 6 | 49.754069 | -109.253517 | 625799 | 5512751 | 1 | 1826.61 | Local |
| F1 | 06/07/2010 0:00 | 9 | 49.744768 | -109.232621 | 627329 | 5511752 | 1 | 616.74 | Local |
| F1 | 06/07/2010 0:00 | 12 | 49.748028 | -109.239546 | 626821 | 5512103 | 1 | 18.90 | Local |
| F1 | 06/07/2010 0:00 | 15 | 49.747872 | -109.239651 | 626814 | 5512086 | 0 | 824.76 | Local |
| F1 | 06/07/2010 0:00 | 21 | 49.754737 | -109.243981 | 626485 | 5512842 | 0 | 18.35 | Local |
| F1 | 06/08/2010 0:00 | 3 | 49.754828 | -109.243768 | 626500 | 5512852 | 1 | 330.99 | Local |
| F1 | 06/08/2010 0:00 | 6 | 49.755904 | -109.239484 | 626805 | 5512979 | 1 | 1881.47 | Local |
| F1 | 06/08/2010 0:00 | 9 | 49.768916 | -109.256179 | 625569 | 5514397 | 1 | 263.71 | Local |
| F1 | 06/08/2010 0:00 | 12 | 49.769036 | -109.259835 | 625306 | 5514405 | 1 | 95.64 | Local |
| F1 | 06/08/2010 0:00 | 15 | 49.768718 | -109.261069 | 625218 | 5514367 | 0 | 1113.15 | Local |
| F1 | 06/08/2010 0:00 | 21 | 49.759056 | -109.257032 | 625533 | 5513300 | 1 | 1041.60 | Local |
| F1 | 06/09/2010 0:00 | 0 | 49.755095 | -109.243930 | 626487 | 5512881 | 0 | 174.27 | Local |
| F1 | 06/09/2010 0:00 | 6 | 49.756437 | -109.242680 | 626574 | 5513033 | 1 | 166.64 | Local |
| F1 | 06/09/2010 0:00 | 9 | 49.756142 | -109.240413 | 626738 | 5513004 | 1 | 2.88 | Local |
| F1 | 06/09/2010 0:00 | 12 | 49.756167 | -109.240407 | 626738 | 5513007 | 1 | 266.32 | Local |
| F1 | 06/09/2010 0:00 | 15 | 49.754888 | -109.243531 | 626517 | 5512859 | 0 | 255.82 | Local |
| F1 | 06/09/2010 0:00 | 21 | 49.756907 | -109.245232 | 626389 | 5513081 | 0 | 213.15 | Local |
| F1 | 06/10/2010 0:00 | 6 | 49.755094 | -109.244274 | 626463 | 5512881 | 1 | 7.40 | Local |
| F1 | 06/10/2010 0:00 | 9 | 49.755045 | -109.244204 | 626468 | 5512875 | 0 | 30.08 | Local |
| F1 | 06/10/2010 0:00 | 18 | 49.755112 | -109.243799 | 626497 | 5512883 | 1 | 1740.11 | Local |
| F1 | 06/10/2010 0:00 | 21 | 49.769198 | -109.254320 | 625702 | 5514432 | 1 | 562.90 | Local |
| F1 | 06/11/2010 0:00 | 0 | 49.768766 | -109.262107 | 625143 | 5514371 | 1 | 883.52 | Local |
| F1 | 06/11/2010 0:00 | 3 | 49.760827 | -109.262588 | 625129 | 5513487 | 1 | 1768.41 | Local |
| F1 | 06/11/2010 0:00 | 6 | 49.746420 | -109.252194 | 625915 | 5511903 | 1 | 1539.60 | Local |
| F1 | 06/11/2010 0:00 | 9 | 49.738657 | -109.234504 | 627209 | 5511070 | 1 | 395.71 | Local |

| | | | | | | | | | |
|---|---|---|---|---|---|---|---|---|---|
| F1 | 06/11/2010 0:00 | 12 | 49.742117 | -109.235784 | 627108 | 5511452 | 1 | 14.07 | Local |
| F1 | 06/11/2010 0:00 | 15 | 49.742162 | -109.235967 | 627095 | 5511457 | 1 | 440.46 | Local |
| F1 | 06/11/2010 0:00 | 18 | 49.744714 | -109.240641 | 626751 | 5511733 | 1 | 1169.45 | Local |
| F1 | 06/11/2010 0:00 | 21 | 49.754947 | -109.244384 | 626455 | 5512864 | 0 | 62.18 | Local |
| F1 | 06/12/2010 0:00 | 12 | 49.754613 | -109.245076 | 626406 | 5512826 | 1 | 248.98 | Local |
| F1 | 06/12/2010 0:00 | 15 | 49.756758 | -109.246065 | 626329 | 5513063 | 0 | 2419.48 | Local |
| F1 | 06/12/2010 0:00 | 21 | 49.761274 | -109.278919 | 623951 | 5513510 | 1 | 58.95 | Local |
| F1 | 06/13/2010 0:00 | 0 | 49.760807 | -109.279304 | 623925 | 5513457 | 1 | 48.18 | Local |
| F1 | 06/13/2010 0:00 | 3 | 49.761240 | -109.279293 | 623925 | 5513505 | 1 | 12.33 | Local |
| F1 | 06/13/2010 0:00 | 6 | 49.761129 | -109.279303 | 623924 | 5513493 | 0 | 29.89 | Local |
| F1 | 06/13/2010 0:00 | 15 | 49.760907 | -109.279537 | 623908 | 5513468 | 1 | 50.79 | Local |
| F1 | 06/13/2010 0:00 | 18 | 49.761204 | -109.279002 | 623946 | 5513502 | 1 | 1311.31 | Local |
| F1 | 06/13/2010 0:00 | 21 | 49.749458 | -109.280601 | 623860 | 5512194 | 1 | 1585.01 | Local |
| F1 | 06/14/2010 0:00 | 0 | 49.750075 | -109.258623 | 625442 | 5512299 | 1 | 1195.01 | Local |
| F1 | 06/14/2010 0:00 | 3 | 49.755077 | -109.243943 | 626486 | 5512879 | 1 | 433.25 | Local |
| F1 | 06/14/2010 0:00 | 6 | 49.751374 | -109.245815 | 626361 | 5512465 | 1 | 620.27 | Local |
| F1 | 06/14/2010 0:00 | 9 | 49.756614 | -109.242864 | 626560 | 5513052 | 1 | 7.06 | Local |
| F1 | 06/14/2010 0:00 | 12 | 49.756600 | -109.242959 | 626553 | 5513050 | 0 | 16.53 | Local |
| F1 | 06/14/2010 0:00 | 18 | 49.756472 | -109.242843 | 626562 | 5513036 | 1 | 1128.97 | Local |
| F1 | 06/14/2010 0:00 | 21 | 49.765124 | -109.251044 | 625949 | 5513984 | 1 | 1308.64 | Local |
| F1 | 06/15/2010 0:00 | 0 | 49.768173 | -109.233496 | 627205 | 5514353 | 1 | 279.79 | Local |
| F1 | 06/15/2010 0:00 | 3 | 49.765962 | -109.231642 | 627344 | 5514110 | 1 | 563.31 | Local |
| F1 | 06/15/2010 0:00 | 6 | 49.760898 | -109.231827 | 627344 | 5513547 | 1 | 1581.90 | Local |
| F1 | 06/15/2010 0:00 | 9 | 49.763581 | -109.210260 | 628890 | 5513882 | 1 | 14.45 | Local |
| F1 | 06/15/2010 0:00 | 12 | 49.763487 | -109.210399 | 628880 | 5513871 | 1 | 10.53 | Local |
| F1 | 06/15/2010 0:00 | 15 | 49.763482 | -109.210253 | 628891 | 5513871 | 1 | 753.57 | Local |
| F1 | 06/15/2010 0:00 | 18 | 49.756877 | -109.207915 | 629077 | 5513141 | 1 | 2508.25 | Local |
| F1 | 06/15/2010 0:00 | 21 | 49.762310 | -109.241708 | 626629 | 5513687 | 1 | 639.38 | Local |
| F1 | 06/16/2010 0:00 | 0 | 49.765578 | -109.249011 | 626094 | 5514038 | 1 | 223.08 | Local |
| F1 | 06/16/2010 0:00 | 3 | 49.766528 | -109.251739 | 625895 | 5514139 | 1 | 931.62 | Local |
| F1 | 06/16/2010 0:00 | 6 | 49.770832 | -109.262836 | 625085 | 5514599 | 1 | 1224.44 | Local |
| F1 | 06/16/2010 0:00 | 9 | 49.767137 | -109.246822 | 626248 | 5514215 | 1 | 438.26 | Local |
| F1 | 06/16/2010 0:00 | 12 | 49.763290 | -109.245497 | 626353 | 5513790 | 1 | 11.46 | Local |
| F1 | 06/16/2010 0:00 | 15 | 49.763194 | -109.245552 | 626349 | 5513779 | 0 | 962.08 | Local |
| F1 | 06/16/2010 0:00 | 21 | 49.754582 | -109.244264 | 626465 | 5512824 | 1 | 167.63 | Local |
| F1 | 06/17/2010 0:00 | 0 | 49.755858 | -109.243025 | 626551 | 5512968 | 1 | 1.43 | Local |
| F1 | 06/17/2010 0:00 | 3 | 49.755846 | -109.243030 | 626550 | 5512966 | 1 | 163.54 | Local |
| F1 | 06/17/2010 0:00 | 6 | 49.755682 | -109.245286 | 626388 | 5512944 | 1 | 29.66 | Local |
| F1 | 06/17/2010 0:00 | 9 | 49.755679 | -109.244874 | 626418 | 5512945 | 0 | 92.23 | Local |
| F1 | 06/17/2010 0:00 | 18 | 49.755744 | -109.243598 | 626510 | 5512954 | 1 | 70.46 | Local |
| F1 | 06/17/2010 0:00 | 21 | 49.755135 | -109.243872 | 626491 | 5512886 | 1 | 1564.65 | Local |
| F1 | 06/18/2010 0:00 | 0 | 49.761622 | -109.224599 | 627862 | 5513640 | 0 | 284.32 | Local |
| F1 | 06/18/2010 0:00 | 6 | 49.762407 | -109.228355 | 627590 | 5513721 | 0 | 1385.08 | Local |
| F1 | 06/18/2010 0:00 | 15 | 49.754825 | -109.243609 | 626511 | 5512852 | 0 | 962.70 | Local |
| F1 | 06/18/2010 0:00 | 21 | 49.759041 | -109.231937 | 627341 | 5513340 | 0 | 490.17 | Local |
| F1 | 06/19/2010 0:00 | 6 | 49.762712 | -109.228171 | 627602 | 5513755 | 0 | 1266.08 | Local |
| F1 | 06/19/2010 0:00 | 12 | 49.756542 | -109.242942 | 626555 | 5513044 | 0 | 192.40 | Local |
| F1 | 06/19/2010 0:00 | 18 | 49.756937 | -109.245543 | 626366 | 5513083 | 1 | 242.76 | Local |
| F1 | 06/19/2010 0:00 | 21 | 49.758829 | -109.247224 | 626240 | 5513291 | 0 | 109.12 | Local |
| F1 | 06/20/2010 0:00 | 3 | 49.759132 | -109.248664 | 626136 | 5513322 | 1 | 603.17 | Local |
| F1 | 06/20/2010 0:00 | 6 | 49.753719 | -109.248133 | 626188 | 5512721 | 1 | 409.58 | Local |
| F1 | 06/20/2010 0:00 | 9 | 49.755998 | -109.243666 | 626504 | 5512982 | 1 | 28.58 | Local |
| F1 | 06/20/2010 0:00 | 12 | 49.755749 | -109.243763 | 626498 | 5512954 | 1 | 3.05 | Local |
| F1 | 06/20/2010 0:00 | 15 | 49.755758 | -109.243803 | 626495 | 5512955 | 1 | 556.33 | Local |
| F1 | 06/20/2010 0:00 | 18 | 49.754905 | -109.251412 | 625949 | 5512848 | 1 | 748.20 | Local |

| | | | | | | | | | |
|---|---|---|---|---|---|---|---|---|---|
| F1 | 06/20/2010 0:00 | 21 | 49.758722 | -109.259964 | 625323 | 5513258 | 0 | 1146.04 | Local |
| F1 | 06/21/2010 0:00 | 3 | 49.769027 | -109.260240 | 625277 | 5514403 | 1 | 1390.11 | Local |
| F1 | 06/21/2010 0:00 | 6 | 49.758899 | -109.248927 | 626118 | 5513296 | 1 | 651.38 | Local |
| F1 | 06/21/2010 0:00 | 9 | 49.762902 | -109.242325 | 626583 | 5513752 | 1 | 881.02 | Local |
| F1 | 06/21/2010 0:00 | 12 | 49.762575 | -109.230105 | 627463 | 5513736 | 1 | 1179.32 | Local |
| F1 | 06/21/2010 0:00 | 15 | 49.758503 | -109.245222 | 626385 | 5513258 | 1 | 545.45 | Local |
| F1 | 06/21/2010 0:00 | 18 | 49.761561 | -109.251142 | 625951 | 5513588 | 1 | 23.23 | Local |
| F1 | 06/21/2010 0:00 | 21 | 49.761423 | -109.251384 | 625934 | 5513572 | 1 | 6.89 | Local |
| F1 | 06/22/2010 0:00 | 0 | 49.761482 | -109.251415 | 625932 | 5513579 | 0 | 304.82 | Local |
| F1 | 06/22/2010 0:00 | 6 | 49.759727 | -109.248165 | 626170 | 5513389 | 1 | 121.64 | Local |
| F1 | 06/22/2010 0:00 | 9 | 49.758834 | -109.249139 | 626102 | 5513288 | 1 | 21.81 | Local |
| F1 | 06/22/2010 0:00 | 12 | 49.759017 | -109.249245 | 626094 | 5513308 | 1 | 7.81 | Local |
| F1 | 06/22/2010 0:00 | 15 | 49.759038 | -109.249141 | 626102 | 5513311 | 0 | 272.28 | Local |
| F1 | 06/22/2010 0:00 | 21 | 49.761481 | -109.248878 | 626114 | 5513583 | 1 | 235.07 | Local |
| F1 | 06/23/2010 0:00 | 0 | 49.759492 | -109.249986 | 626040 | 5513360 | 1 | 13.43 | Local |
| F1 | 06/23/2010 0:00 | 3 | 49.759610 | -109.250027 | 626036 | 5513373 | 1 | 658.44 | Local |
| F1 | 06/23/2010 0:00 | 6 | 49.755720 | -109.243136 | 626543 | 5512952 | 1 | 15.92 | Local |
| F1 | 06/23/2010 0:00 | 9 | 49.755582 | -109.243080 | 626547 | 5512937 | 1 | 357.18 | Local |
| F1 | 06/23/2010 0:00 | 12 | 49.758057 | -109.246240 | 626313 | 5513207 | 1 | 341.62 | Local |
| F1 | 06/23/2010 0:00 | 15 | 49.759947 | -109.249978 | 626039 | 5513411 | 1 | 79.46 | Local |
| F1 | 06/23/2010 0:00 | 18 | 49.759239 | -109.249827 | 626052 | 5513332 | 1 | 226.95 | Local |
| F1 | 06/23/2010 0:00 | 21 | 49.757476 | -109.248240 | 626171 | 5513139 | 1 | 401.94 | Local |
| F4 | 05/06/2011 0:00 | 18 | 49.643777 | -109.612770 | 600150 | 5499949 | 1 | 1547.89 | Trans |
| F4 | 05/06/2011 0:00 | 21 | 49.637807 | -109.632135 | 598764 | 5499260 | 1 | 6302.41 | Trans |
| F4 | 05/07/2011 0:00 | 0 | 49.634592 | -109.719268 | 592479 | 5498792 | 1 | 8582.22 | Trans |
| F4 | 05/07/2011 0:00 | 3 | 49.662627 | -109.830025 | 584433 | 5501778 | 1 | 2510.53 | Trans |
| F4 | 05/07/2011 0:00 | 6 | 49.649000 | -109.857757 | 582455 | 5500233 | 1 | 41.83 | Trans |
| F4 | 05/07/2011 0:00 | 9 | 49.648976 | -109.858335 | 582413 | 5500229 | 1 | 11.92 | Trans |
| F4 | 05/07/2011 0:00 | 12 | 49.648970 | -109.858500 | 582401 | 5500228 | 1 | 14.23 | Trans |
| F4 | 05/07/2011 0:00 | 15 | 49.649097 | -109.858474 | 582403 | 5500243 | 0 | 6621.47 | Trans |
| F4 | 05/08/2011 0:00 | 0 | 49.640617 | -109.949251 | 575863 | 5499204 | 1 | 3660.33 | Trans |
| F4 | 05/08/2011 0:00 | 3 | 49.648525 | -109.998464 | 572298 | 5500035 | 1 | 2168.98 | Trans |
| F4 | 05/08/2011 0:00 | 6 | 49.652569 | -110.027857 | 570171 | 5500457 | 1 | 22.96 | Trans |
| F4 | 05/08/2011 0:00 | 9 | 49.652743 | -110.027686 | 570183 | 5500476 | 1 | 13.82 | Trans |
| F4 | 05/08/2011 0:00 | 12 | 49.652821 | -110.027835 | 570172 | 5500485 | 1 | 8.40 | Trans |
| F4 | 05/08/2011 0:00 | 15 | 49.652765 | -110.027756 | 570178 | 5500479 | 1 | 111.72 | Trans |
| F4 | 05/08/2011 0:00 | 18 | 49.652709 | -110.029301 | 570066 | 5500471 | 1 | 2894.75 | Trans |
| F4 | 05/08/2011 0:00 | 21 | 49.657055 | -110.068841 | 567206 | 5500918 | 0 | 7256.16 | Trans |
| F4 | 05/09/2011 0:00 | 3 | 49.684788 | -110.159869 | 560602 | 5503924 | 1 | 2865.55 | Trans |
| F4 | 05/09/2011 0:00 | 6 | 49.686609 | -110.199493 | 557742 | 5504095 | 1 | 459.94 | Trans |
| F4 | 05/09/2011 0:00 | 9 | 49.684979 | -110.205354 | 557321 | 5503909 | 1 | 93.96 | Trans |
| F4 | 05/09/2011 0:00 | 12 | 49.684516 | -110.204264 | 557400 | 5503859 | 1 | 3.55 | Trans |
| F4 | 05/09/2011 0:00 | 15 | 49.684542 | -110.204294 | 557398 | 5503861 | 1 | 7.85 | Trans |
| F4 | 05/09/2011 0:00 | 18 | 49.684515 | -110.204395 | 557391 | 5503858 | 1 | 1150.38 | Trans |
| F4 | 05/09/2011 0:00 | 21 | 49.685055 | -110.220320 | 556241 | 5503906 | 1 | 4405.46 | Trans |
| F4 | 05/10/2011 0:00 | 0 | 49.670852 | -110.277325 | 552145 | 5502286 | 1 | 1559.36 | Trans |
| F4 | 05/10/2011 0:00 | 3 | 49.658175 | -110.286570 | 551491 | 5500871 | 0 | 1159.37 | Trans |
| F4 | 05/10/2011 0:00 | 9 | 49.655787 | -110.302206 | 550365 | 5500594 | 1 | 16.66 | Trans |
| F4 | 05/10/2011 0:00 | 12 | 49.655670 | -110.302061 | 550375 | 5500582 | 1 | 5.99 | Trans |
| F4 | 05/10/2011 0:00 | 15 | 49.655697 | -110.302133 | 550370 | 5500584 | 1 | 9.82 | Trans |
| F4 | 05/10/2011 0:00 | 18 | 49.655610 | -110.302155 | 550369 | 5500575 | 0 | 2396.97 | Trans |
| F4 | 05/11/2011 0:00 | 0 | 49.650739 | -110.334503 | 548039 | 5500012 | 1 | 5948.81 | Trans |
| F4 | 05/11/2011 0:00 | 3 | 49.635531 | -110.413501 | 542349 | 5498274 | 1 | 3189.72 | Trans |
| F4 | 05/11/2011 0:00 | 6 | 49.630482 | -110.456983 | 539214 | 5497689 | 1 | 1227.48 | Trans |
| F4 | 05/11/2011 0:00 | 9 | 49.629632 | -110.473929 | 537991 | 5497586 | 0 | 52.46 | Trans |

| | | | | | | | | | |
|---|---|---|---|---|---|---|---|---|---|
| F4 | 05/11/2011 0:00 | 15 | 49.630104 | -110.473951 | 537989 | 5497638 | 1 | 30.35 | Trans |
| F4 | 05/11/2011 0:00 | 18 | 49.629833 | -110.473895 | 537993 | 5497608 | 1 | 141.78 | Trans |
| F4 | 05/11/2011 0:00 | 21 | 49.630790 | -110.472597 | 538086 | 5497715 | 1 | 6411.80 | Trans |
| F4 | 05/12/2011 0:00 | 0 | 49.637244 | -110.560832 | 531710 | 5498392 | 1 | 2388.15 | Trans |
| F4 | 05/12/2011 0:00 | 3 | 49.657064 | -110.573583 | 530777 | 5500590 | 1 | 3489.43 | Trans |
| F4 | 05/12/2011 0:00 | 6 | 49.686546 | -110.556997 | 531955 | 5503875 | 1 | 8.69 | Trans |
| F4 | 05/12/2011 0:00 | 9 | 49.686591 | -110.557096 | 531947 | 5503880 | 1 | 6.63 | Trans |
| F4 | 05/12/2011 0:00 | 12 | 49.686639 | -110.557041 | 531951 | 5503885 | 1 | 14.23 | Trans |
| F4 | 05/12/2011 0:00 | 15 | 49.686527 | -110.557138 | 531944 | 5503872 | 1 | 21.96 | Trans |
| F4 | 05/12/2011 0:00 | 18 | 49.686725 | -110.557129 | 531945 | 5503894 | 1 | 17.15 | Trans |
| F4 | 05/12/2011 0:00 | 21 | 49.686571 | -110.557109 | 531947 | 5503877 | 1 | 1162.93 | Trans |
| F4 | 05/13/2011 0:00 | 0 | 49.685130 | -110.541141 | 533099 | 5503724 | 1 | 9844.18 | Trans |
| F4 | 05/13/2011 0:00 | 3 | 49.683598 | -110.404695 | 542943 | 5503623 | 1 | 4179.99 | Trans |
| F4 | 05/13/2011 0:00 | 6 | 49.665595 | -110.353836 | 546629 | 5501652 | 1 | 12.16 | Trans |
| F4 | 05/13/2011 0:00 | 9 | 49.665624 | -110.353674 | 546641 | 5501655 | 1 | 2.68 | Trans |
| F4 | 05/13/2011 0:00 | 12 | 49.665640 | -110.353702 | 546639 | 5501657 | 1 | 6.01 | Trans |
| F4 | 05/13/2011 0:00 | 15 | 49.665638 | -110.353785 | 546633 | 5501656 | 1 | 3.01 | Trans |
| F4 | 05/13/2011 0:00 | 18 | 49.665612 | -110.353798 | 546632 | 5501653 | 1 | 40.61 | Trans |
| F4 | 05/13/2011 0:00 | 21 | 49.665965 | -110.353654 | 546642 | 5501693 | 1 | 2334.65 | Trans |
| F4 | 05/14/2011 0:00 | 0 | 49.656725 | -110.324606 | 548747 | 5500684 | 0 | 1649.90 | Trans |
| F4 | 05/14/2011 0:00 | 9 | 49.655764 | -110.301795 | 550394 | 5500592 | 0 | 492.44 | Trans |
| F4 | 05/15/2011 0:00 | 0 | 49.656317 | -110.295026 | 550882 | 5500658 | 1 | 117.98 | Trans |
| F4 | 05/15/2011 0:00 | 3 | 49.657360 | -110.295330 | 550859 | 5500774 | 1 | 743.86 | Trans |
| F4 | 05/15/2011 0:00 | 6 | 49.650889 | -110.297951 | 550677 | 5500053 | 1 | 100.60 | Trans |
| F4 | 05/15/2011 0:00 | 9 | 49.650135 | -110.297181 | 550733 | 5499969 | 1 | 7.79 | Trans |
| F4 | 05/15/2011 0:00 | 12 | 49.650205 | -110.297181 | 550733 | 5499977 | 1 | 18.54 | Trans |
| F4 | 05/15/2011 0:00 | 15 | 49.650220 | -110.297436 | 550715 | 5499979 | 1 | 482.44 | Trans |
| F4 | 05/15/2011 0:00 | 18 | 49.646230 | -110.300062 | 550529 | 5499533 | 0 | 1748.57 | Trans |
| F4 | 05/16/2011 0:00 | 0 | 49.658229 | -110.284403 | 551647 | 5500878 | 1 | 470.67 | Trans |
| F4 | 05/16/2011 0:00 | 3 | 49.654387 | -110.287140 | 551454 | 5500449 | 1 | 646.06 | Trans |
| F4 | 05/16/2011 0:00 | 6 | 49.649091 | -110.290827 | 551193 | 5499858 | 1 | 25.81 | Trans |
| F4 | 05/16/2011 0:00 | 9 | 49.649320 | -110.290766 | 551197 | 5499883 | 0 | 466.18 | Trans |
| F4 | 05/16/2011 0:00 | 18 | 49.651884 | -110.295877 | 550826 | 5500165 | 1 | 68.37 | Trans |
| F4 | 05/16/2011 0:00 | 21 | 49.652487 | -110.295694 | 550838 | 5500232 | 1 | 1808.46 | Trans |
| F4 | 05/17/2011 0:00 | 0 | 49.661614 | -110.274954 | 552325 | 5501261 | 1 | 1085.31 | Trans |
| F4 | 05/17/2011 0:00 | 3 | 49.664196 | -110.260452 | 553369 | 5501558 | 1 | 203.41 | Trans |
| F4 | 05/17/2011 0:00 | 6 | 49.662718 | -110.258789 | 553491 | 5501395 | 1 | 8.05 | Trans |
| F4 | 05/17/2011 0:00 | 9 | 49.662777 | -110.258853 | 553486 | 5501402 | 1 | 5.88 | Trans |
| F4 | 05/17/2011 0:00 | 12 | 49.662742 | -110.258914 | 553482 | 5501398 | 1 | 11.38 | Trans |
| F4 | 05/17/2011 0:00 | 15 | 49.662649 | -110.258978 | 553477 | 5501387 | 1 | 15.38 | Trans |
| F4 | 05/17/2011 0:00 | 18 | 49.662692 | -110.259181 | 553463 | 5501392 | 0 | 4265.08 | Trans |
| F4 | 05/18/2011 0:00 | 0 | 49.700739 | -110.251639 | 553965 | 5505627 | 1 | 3736.81 | Trans |
| F4 | 05/18/2011 0:00 | 3 | 49.717404 | -110.206631 | 557191 | 5507513 | 1 | 3078.07 | Trans |
| F4 | 05/18/2011 0:00 | 6 | 49.692799 | -110.187065 | 558631 | 5504793 | 1 | 74.93 | Trans |
| F4 | 05/18/2011 0:00 | 9 | 49.692173 | -110.187450 | 558604 | 5504723 | 0 | 11.78 | Trans |
| F4 | 05/18/2011 0:00 | 15 | 49.692278 | -110.187471 | 558602 | 5504735 | 1 | 39.11 | Trans |
| F4 | 05/18/2011 0:00 | 18 | 49.691930 | -110.187389 | 558608 | 5504696 | 1 | 422.03 | Trans |
| F4 | 05/18/2011 0:00 | 21 | 49.693132 | -110.192940 | 558207 | 5504825 | 1 | 5698.73 | Trans |
| F4 | 05/19/2011 0:00 | 0 | 49.669639 | -110.122734 | 563300 | 5502270 | 1 | 3954.79 | Trans |
| F4 | 05/19/2011 0:00 | 3 | 49.670880 | -110.067961 | 567251 | 5502456 | 1 | 6099.67 | Trans |
| F4 | 05/19/2011 0:00 | 6 | 49.660985 | -109.984826 | 573264 | 5501433 | 0 | 862.83 | Trans |
| F4 | 05/19/2011 0:00 | 12 | 49.661761 | -109.972931 | 574122 | 5501531 | 1 | 33.16 | Trans |
| F4 | 05/19/2011 0:00 | 15 | 49.661464 | -109.972971 | 574119 | 5501498 | 1 | 361.54 | Trans |
| F4 | 05/19/2011 0:00 | 18 | 49.661651 | -109.967970 | 574480 | 5501524 | 0 | 10239.63 | Trans |
| F4 | 05/20/2011 0:00 | 12 | 49.641439 | -109.829584 | 584502 | 5499423 | 1 | 146.46 | Trans |

| | | | | | | | | | |
|---|---|---|---|---|---|---|---|---|---|
| F4 | 05/20/2011 0:00 | 15 | 49.640158 | -109.829111 | 584538 | 5499281 | 1 | 11.12 | Trans |
| F4 | 05/20/2011 0:00 | 18 | 49.640130 | -109.828963 | 584549 | 5499278 | 1 | 406.09 | Trans |
| F4 | 05/20/2011 0:00 | 21 | 49.643782 | -109.828870 | 584549 | 5499685 | 1 | 4407.90 | Trans |
| F4 | 05/21/2011 0:00 | 0 | 49.612364 | -109.791651 | 587292 | 5496234 | 1 | 1843.05 | Trans |
| F4 | 05/21/2011 0:00 | 3 | 49.598729 | -109.777145 | 588365 | 5494735 | 1 | 5258.12 | Trans |
| F4 | 05/21/2011 0:00 | 6 | 49.560655 | -109.734009 | 591553 | 5490554 | 1 | 271.98 | Trans |
| F4 | 05/21/2011 0:00 | 9 | 49.560131 | -109.737683 | 591288 | 5490491 | 1 | 8.24 | Trans |
| F4 | 05/21/2011 0:00 | 12 | 49.560186 | -109.737760 | 591282 | 5490497 | 1 | 11.72 | Trans |
| F4 | 05/21/2011 0:00 | 15 | 49.560086 | -109.737709 | 591286 | 5490486 | 1 | 22.85 | Trans |
| F4 | 05/21/2011 0:00 | 18 | 49.560100 | -109.737393 | 591309 | 5490488 | 1 | 41.69 | Trans |
| F4 | 05/21/2011 0:00 | 21 | 49.559996 | -109.737947 | 591269 | 5490476 | 1 | 6493.80 | Trans |
| F4 | 05/22/2011 0:00 | 0 | 49.533450 | -109.657995 | 597104 | 5487625 | 1 | 6781.65 | Trans |
| F4 | 05/22/2011 0:00 | 3 | 49.516930 | -109.567801 | 603665 | 5485909 | 1 | 3574.40 | Trans |
| F4 | 05/22/2011 0:00 | 6 | 49.513551 | -109.518701 | 607226 | 5485602 | 1 | 47.68 | Trans |
| F4 | 05/22/2011 0:00 | 9 | 49.513524 | -109.518044 | 607273 | 5485600 | 1 | 23.67 | Trans |
| F4 | 05/22/2011 0:00 | 12 | 49.513347 | -109.517863 | 607287 | 5485580 | 0 | 32.73 | Trans |
| F4 | 05/22/2011 0:00 | 18 | 49.513616 | -109.518047 | 607273 | 5485610 | 1 | 11.76 | Trans |
| F4 | 05/22/2011 0:00 | 21 | 49.513510 | -109.518057 | 607273 | 5485598 | 0 | 2266.84 | Trans |
| F4 | 05/23/2011 0:00 | 6 | 49.501497 | -109.492762 | 609130 | 5484299 | 1 | 4.35 | Trans |
| F4 | 05/23/2011 0:00 | 9 | 49.501502 | -109.492703 | 609134 | 5484299 | 1 | 4.22 | Trans |
| F4 | 05/23/2011 0:00 | 12 | 49.501512 | -109.492758 | 609130 | 5484301 | 1 | 40.65 | Trans |
| F4 | 05/23/2011 0:00 | 15 | 49.501581 | -109.493310 | 609090 | 5484307 | 1 | 17.04 | Trans |
| F4 | 05/23/2011 0:00 | 18 | 49.501492 | -109.493501 | 609077 | 5484297 | 1 | 246.28 | Trans |
| F4 | 05/23/2011 0:00 | 21 | 49.502454 | -109.496564 | 608853 | 5484400 | 1 | 216.90 | Trans |
| F4 | 05/24/2011 0:00 | 0 | 49.501672 | -109.493821 | 609053 | 5484317 | 1 | 10.73 | Trans |
| F4 | 05/24/2011 0:00 | 3 | 49.501764 | -109.493865 | 609050 | 5484327 | 1 | 3175.96 | Trans |
| F4 | 05/24/2011 0:00 | 6 | 49.500470 | -109.450054 | 612225 | 5484247 | 1 | 13.26 | Trans |
| F4 | 05/24/2011 0:00 | 9 | 49.500352 | -109.450028 | 612227 | 5484234 | 0 | 10.21 | Trans |
| F4 | 05/24/2011 0:00 | 18 | 49.500266 | -109.449978 | 612231 | 5484225 | 1 | 161.51 | Trans |
| F4 | 05/24/2011 0:00 | 21 | 49.500182 | -109.447752 | 612392 | 5484219 | 1 | 233.12 | Trans |
| F4 | 05/25/2011 0:00 | 0 | 49.498314 | -109.446290 | 612502 | 5484013 | 1 | 9.42 | Trans |
| F4 | 05/25/2011 0:00 | 3 | 49.498361 | -109.446399 | 612494 | 5484018 | 1 | 241.46 | Trans |
| F4 | 05/25/2011 0:00 | 6 | 49.499780 | -109.448922 | 612308 | 5484172 | 0 | 10.15 | Trans |
| F4 | 05/25/2011 0:00 | 12 | 49.499781 | -109.449063 | 612298 | 5484172 | 1 | 3.38 | Trans |
| F4 | 05/25/2011 0:00 | 15 | 49.499786 | -109.449017 | 612301 | 5484173 | 1 | 243.85 | Trans |
| F4 | 05/25/2011 0:00 | 18 | 49.498263 | -109.446594 | 612480 | 5484007 | 1 | 94.49 | Trans |
| F4 | 05/25/2011 0:00 | 21 | 49.497586 | -109.445805 | 612539 | 5483933 | 0 | 2451.43 | Trans |
| F4 | 05/26/2011 0:00 | 3 | 49.498514 | -109.411986 | 614985 | 5484087 | 1 | 764.13 | Trans |
| F4 | 05/26/2011 0:00 | 6 | 49.503202 | -109.404271 | 615533 | 5484620 | 1 | 3.16 | Trans |
| F4 | 05/26/2011 0:00 | 9 | 49.503228 | -109.404290 | 615532 | 5484623 | 1 | 60.16 | Trans |
| F4 | 05/26/2011 0:00 | 12 | 49.503290 | -109.403464 | 615591 | 5484631 | 0 | 67.45 | Trans |
| F4 | 05/26/2011 0:00 | 21 | 49.503208 | -109.404387 | 615525 | 5484621 | 1 | 2417.14 | Trans |
| F4 | 05/27/2011 0:00 | 0 | 49.512544 | -109.374240 | 617685 | 5485705 | 1 | 4631.16 | Trans |
| F4 | 05/27/2011 0:00 | 3 | 49.539088 | -109.324936 | 621188 | 5488734 | 0 | 161.79 | Trans |
| F4 | 05/27/2011 0:00 | 12 | 49.540535 | -109.324709 | 621201 | 5488896 | 0 | 40.42 | Trans |
| F4 | 05/27/2011 0:00 | 21 | 49.540898 | -109.324670 | 621203 | 5488936 | 0 | 1367.83 | Trans |
| F4 | 05/28/2011 0:00 | 3 | 49.553041 | -109.321651 | 621391 | 5490291 | 1 | 240.43 | Trans |
| F4 | 05/28/2011 0:00 | 6 | 49.555112 | -109.320698 | 621455 | 5490523 | 1 | 108.78 | Trans |
| F4 | 05/28/2011 0:00 | 9 | 49.555175 | -109.322199 | 621346 | 5490527 | 1 | 22.75 | Trans |
| F4 | 05/28/2011 0:00 | 12 | 49.555380 | -109.322208 | 621345 | 5490550 | 0 | 125.84 | Trans |
| F4 | 05/28/2011 0:00 | 18 | 49.555765 | -109.320572 | 621462 | 5490595 | 1 | 16.92 | Trans |
| F4 | 05/28/2011 0:00 | 21 | 49.555802 | -109.320799 | 621446 | 5490599 | 1 | 16.12 | Trans |
| F4 | 05/29/2011 0:00 | 0 | 49.555775 | -109.320580 | 621462 | 5490597 | 1 | 295.22 | Trans |
| F4 | 05/29/2011 0:00 | 3 | 49.556136 | -109.316537 | 621753 | 5490643 | 1 | 354.69 | Trans |
| F4 | 05/29/2011 0:00 | 6 | 49.558455 | -109.319904 | 621504 | 5490896 | 1 | 15.83 | Trans |

| | | | | | | | | | |
|---|---|---|---|---|---|---|---|---|---|
| F4 | 05/29/2011 0:00 | 9 | 49.558503 | -109.319698 | 621519 | 5490901 | 0 | 1208.89 | Trans |
| F4 | 05/29/2011 0:00 | 15 | 49.548080 | -109.324453 | 621201 | 5489735 | 1 | 8.01 | Trans |
| F4 | 05/29/2011 0:00 | 18 | 49.548151 | -109.324475 | 621199 | 5489743 | 1 | 6.45 | Trans |
| F4 | 05/29/2011 0:00 | 21 | 49.548093 | -109.324482 | 621199 | 5489736 | 1 | 600.33 | Trans |
| F4 | 05/30/2011 0:00 | 0 | 49.553476 | -109.325117 | 621139 | 5490334 | 1 | 9.83 | Trans |
| F4 | 05/30/2011 0:00 | 3 | 49.553562 | -109.325084 | 621142 | 5490343 | 1 | 301.31 | Trans |
| F4 | 05/30/2011 0:00 | 6 | 49.551443 | -109.322487 | 621335 | 5490112 | 1 | 27.15 | Trans |
| F4 | 05/30/2011 0:00 | 9 | 49.551676 | -109.322374 | 621342 | 5490138 | 1 | 277.39 | Trans |
| F4 | 05/30/2011 0:00 | 12 | 49.549403 | -109.323956 | 621233 | 5489883 | 1 | 11.91 | Trans |
| F4 | 05/30/2011 0:00 | 15 | 49.549310 | -109.323877 | 621239 | 5489872 | 1 | 102.24 | Trans |
| F4 | 05/30/2011 0:00 | 18 | 49.549582 | -109.322527 | 621336 | 5489905 | 1 | 1004.29 | Trans |
| F4 | 05/30/2011 0:00 | 21 | 49.540569 | -109.323418 | 621294 | 5488902 | 0 | 811.97 | Trans |
| F4 | 05/31/2011 0:00 | 3 | 49.533339 | -109.324987 | 621199 | 5488095 | 1 | 955.48 | Trans |
| F4 | 05/31/2011 0:00 | 6 | 49.526269 | -109.332491 | 620673 | 5487297 | 0 | 20.75 | Trans |
| F4 | 05/31/2011 0:00 | 18 | 49.526103 | -109.332362 | 620683 | 5487279 | 0 | 12.01 | Trans |
| F4 | 06/01/2011 0:00 | 3 | 49.526205 | -109.332310 | 620686 | 5487290 | 0 | 28.93 | Trans |
| F4 | 06/01/2011 0:00 | 12 | 49.525964 | -109.332161 | 620698 | 5487264 | 1 | 30.40 | Trans |
| F4 | 06/01/2011 0:00 | 15 | 49.526072 | -109.332546 | 620669 | 5487275 | 0 | 24.23 | Trans |
| F4 | 06/01/2011 0:00 | 21 | 49.525935 | -109.332286 | 620689 | 5487260 | 1 | 24.32 | Trans |
| F4 | 06/02/2011 0:00 | 0 | 49.526150 | -109.332352 | 620683 | 5487284 | 0 | 259.94 | Trans |
| F4 | 06/02/2011 0:00 | 6 | 49.523940 | -109.333525 | 620604 | 5487037 | 1 | 249.62 | Trans |
| F4 | 06/02/2011 0:00 | 9 | 49.526094 | -109.332552 | 620669 | 5487278 | 1 | 9.21 | Trans |
| F4 | 06/02/2011 0:00 | 12 | 49.526155 | -109.332638 | 620663 | 5487284 | 1 | 17.59 | Trans |
| F4 | 06/02/2011 0:00 | 15 | 49.526152 | -109.332395 | 620680 | 5487284 | 1 | 34.67 | Trans |
| F4 | 06/02/2011 0:00 | 18 | 49.525840 | -109.332371 | 620683 | 5487250 | 1 | 15.61 | Trans |
| F4 | 06/02/2011 0:00 | 21 | 49.525953 | -109.332243 | 620692 | 5487263 | 1 | 24.33 | Trans |
| F4 | 06/03/2011 0:00 | 0 | 49.526159 | -109.332358 | 620683 | 5487285 | 1 | 8.18 | Trans |
| F4 | 06/03/2011 0:00 | 3 | 49.526089 | -109.332396 | 620680 | 5487277 | 1 | 6.43 | Trans |
| F4 | 06/03/2011 0:00 | 6 | 49.526105 | -109.332482 | 620674 | 5487279 | 1 | 107.04 | Trans |
| F4 | 06/03/2011 0:00 | 9 | 49.525444 | -109.331407 | 620753 | 5487207 | 1 | 96.52 | Trans |
| F4 | 06/03/2011 0:00 | 12 | 49.526180 | -109.332115 | 620700 | 5487288 | 0 | 50.27 | Trans |
| F4 | 06/03/2011 0:00 | 18 | 49.525837 | -109.332567 | 620669 | 5487249 | 1 | 47.18 | Trans |
| F4 | 06/03/2011 0:00 | 21 | 49.526231 | -109.332326 | 620685 | 5487293 | 1 | 20.59 | Trans |
| F4 | 06/04/2011 0:00 | 0 | 49.526088 | -109.332506 | 620672 | 5487277 | 0 | 29.94 | Trans |
| F4 | 06/04/2011 0:00 | 9 | 49.525901 | -109.332804 | 620651 | 5487256 | 0 | 30.63 | Trans |
| F4 | 06/04/2011 0:00 | 15 | 49.526088 | -109.332494 | 620673 | 5487277 | 1 | 8.83 | Trans |
| F4 | 06/04/2011 0:00 | 18 | 49.526026 | -109.332418 | 620679 | 5487270 | 1 | 18.40 | Trans |
| F4 | 06/04/2011 0:00 | 21 | 49.525931 | -109.332626 | 620664 | 5487259 | 0 | 21.46 | Trans |
| F4 | 06/05/2011 0:00 | 3 | 49.526124 | -109.332638 | 620663 | 5487281 | 1 | 300.98 | Trans |
| F4 | 06/05/2011 0:00 | 6 | 49.528720 | -109.331461 | 620741 | 5487571 | 0 | 427.32 | Trans |
| F4 | 06/05/2011 0:00 | 21 | 49.524877 | -109.331502 | 620748 | 5487144 | 1 | 123.71 | Trans |
| F4 | 06/06/2011 0:00 | 0 | 49.525849 | -109.332334 | 620685 | 5487251 | 0 | 29.70 | Trans |
| F4 | 06/06/2011 0:00 | 6 | 49.525943 | -109.332718 | 620657 | 5487261 | 0 | 27.64 | Trans |
| F4 | 06/06/2011 0:00 | 12 | 49.526176 | -109.332588 | 620666 | 5487287 | 1 | 12.55 | Trans |
| F4 | 06/06/2011 0:00 | 15 | 49.526066 | -109.332621 | 620664 | 5487274 | 0 | 12.39 | Trans |
| F4 | 06/06/2011 0:00 | 21 | 49.526017 | -109.332467 | 620675 | 5487269 | 1 | 20.05 | Trans |
| F4 | 06/07/2011 0:00 | 0 | 49.525923 | -109.332705 | 620658 | 5487259 | 0 | 153.87 | Trans |
| F4 | 06/07/2011 0:00 | 6 | 49.527304 | -109.332857 | 620644 | 5487412 | 1 | 1236.51 | Trans |
| F4 | 06/07/2011 0:00 | 9 | 49.537739 | -109.326952 | 621045 | 5488581 | 1 | 200.63 | Trans |
| F4 | 06/07/2011 0:00 | 12 | 49.539319 | -109.328288 | 620945 | 5488755 | 1 | 14.80 | Trans |
| F4 | 06/07/2011 0:00 | 15 | 49.539188 | -109.328321 | 620943 | 5488740 | 1 | 17.64 | Trans |
| F4 | 06/07/2011 0:00 | 18 | 49.539347 | -109.328333 | 620942 | 5488758 | 1 | 1163.72 | Trans |
| F4 | 06/07/2011 0:00 | 21 | 49.548632 | -109.320914 | 621455 | 5489802 | 1 | 5530.26 | Trans |
| F4 | 06/08/2011 0:00 | 0 | 49.595296 | -109.294462 | 623251 | 5495033 | 1 | 5661.47 | Trans |
| F4 | 06/08/2011 0:00 | 3 | 49.624197 | -109.229960 | 627836 | 5498353 | 1 | 3810.05 | Trans |

| | | | | | | | | | |
|---|---|---|---|---|---|---|---|---|---|
| F4 | 06/08/2011 0:00 | 6 | 49.655597 | -109.251074 | 626230 | 5501808 | 1 | 28.91 | Trans |
| F4 | 06/08/2011 0:00 | 9 | 49.655675 | -109.251456 | 626203 | 5501816 | 1 | 17.81 | Trans |
| F4 | 06/08/2011 0:00 | 12 | 49.655615 | -109.251685 | 626186 | 5501809 | 0 | 6213.03 | Trans |
| F4 | 06/09/2011 0:00 | 0 | 49.708034 | -109.281492 | 623902 | 5507587 | 0 | 6550.91 | Trans |
| F4 | 06/09/2011 0:00 | 6 | 49.766891 | -109.285374 | 623472 | 5514124 | 1 | 72.04 | Local |
| F4 | 06/09/2011 0:00 | 9 | 49.766882 | -109.284374 | 623544 | 5514124 | 1 | 66.84 | Local |
| F4 | 06/09/2011 0:00 | 12 | 49.766793 | -109.283457 | 623611 | 5514116 | 1 | 11.47 | Local |
| F4 | 06/09/2011 0:00 | 15 | 49.766795 | -109.283298 | 623622 | 5514117 | 0 | 629.70 | Local |
| F4 | 06/09/2011 0:00 | 21 | 49.771088 | -109.277596 | 624022 | 5514603 | 1 | 1623.58 | Local |
| F4 | 06/10/2011 0:00 | 0 | 49.777788 | -109.257565 | 625447 | 5515381 | 1 | 2265.75 | Local |
| F4 | 06/10/2011 0:00 | 3 | 49.798160 | -109.258079 | 625357 | 5517645 | 1 | 2170.44 | Local |
| F4 | 06/10/2011 0:00 | 6 | 49.778701 | -109.255745 | 625575 | 5515486 | 1 | 83.54 | Local |
| F4 | 06/10/2011 0:00 | 9 | 49.777972 | -109.255465 | 625597 | 5515405 | 1 | 21.11 | Local |
| F4 | 06/10/2011 0:00 | 12 | 49.778155 | -109.255388 | 625602 | 5515426 | 1 | 3.77 | Local |
| F4 | 06/10/2011 0:00 | 15 | 49.778139 | -109.255434 | 625599 | 5515424 | 1 | 9.48 | Local |
| F4 | 06/10/2011 0:00 | 18 | 49.778122 | -109.255305 | 625609 | 5515422 | 1 | 540.66 | Local |
| F4 | 06/10/2011 0:00 | 21 | 49.773338 | -109.253959 | 625718 | 5514893 | 1 | 679.73 | Local |
| F4 | 06/11/2011 0:00 | 0 | 49.774482 | -109.263231 | 625047 | 5515004 | 1 | 50.55 | Local |
| F4 | 06/11/2011 0:00 | 3 | 49.774828 | -109.263686 | 625014 | 5515042 | 1 | 282.95 | Local |
| F4 | 06/11/2011 0:00 | 6 | 49.773623 | -109.267147 | 624768 | 5514902 | 0 | 16.55 | Local |
| F4 | 06/11/2011 0:00 | 18 | 49.773498 | -109.267272 | 624759 | 5514888 | 1 | 57.72 | Local |
| F4 | 06/11/2011 0:00 | 21 | 49.773905 | -109.267770 | 624722 | 5514933 | 1 | 68.99 | Local |
| F4 | 06/12/2011 0:00 | 0 | 49.773486 | -109.267063 | 624774 | 5514887 | 0 | 21.14 | Local |
| F4 | 06/12/2011 0:00 | 6 | 49.773351 | -109.267270 | 624759 | 5514872 | 0 | 24.52 | Local |
| F4 | 06/12/2011 0:00 | 21 | 49.773566 | -109.267195 | 624764 | 5514896 | 1 | 18.28 | Local |
| F4 | 06/13/2011 0:00 | 0 | 49.773480 | -109.267411 | 624749 | 5514886 | 0 | 24.80 | Local |
| F4 | 06/13/2011 0:00 | 18 | 49.773629 | -109.267154 | 624767 | 5514903 | 1 | 8.93 | Local |
| F4 | 06/13/2011 0:00 | 21 | 49.773596 | -109.267267 | 624759 | 5514899 | 1 | 462.59 | Local |
| F4 | 06/14/2011 0:00 | 0 | 49.773953 | -109.273667 | 624297 | 5514928 | 1 | 468.15 | Local |
| F4 | 06/14/2011 0:00 | 3 | 49.773570 | -109.267193 | 624764 | 5514896 | 1 | 8.36 | Local |
| F4 | 06/14/2011 0:00 | 6 | 49.773633 | -109.267257 | 624760 | 5514903 | 1 | 4.76 | Local |
| F4 | 06/14/2011 0:00 | 9 | 49.773597 | -109.267221 | 624762 | 5514899 | 1 | 4.21 | Local |
| F4 | 06/14/2011 0:00 | 12 | 49.773625 | -109.267260 | 624759 | 5514902 | 0 | 86.93 | Local |
| F4 | 06/14/2011 0:00 | 18 | 49.773039 | -109.266460 | 624818 | 5514839 | 1 | 155.32 | Local |
| F4 | 06/14/2011 0:00 | 21 | 49.773930 | -109.268122 | 624697 | 5514935 | 1 | 438.20 | Local |
| F4 | 06/15/2011 0:00 | 0 | 49.775680 | -109.273574 | 624300 | 5515120 | 1 | 213.62 | Local |
| F4 | 06/15/2011 0:00 | 3 | 49.773804 | -109.274208 | 624259 | 5514911 | 1 | 955.84 | Local |
| F4 | 06/15/2011 0:00 | 6 | 49.766828 | -109.281962 | 623718 | 5514122 | 1 | 390.23 | Local |
| F4 | 06/15/2011 0:00 | 9 | 49.766053 | -109.287246 | 623340 | 5514028 | 1 | 405.65 | Local |
| F4 | 06/15/2011 0:00 | 12 | 49.764256 | -109.292147 | 622991 | 5513820 | 1 | 4.84 | Local |
| F4 | 06/15/2011 0:00 | 15 | 49.764213 | -109.292133 | 622992 | 5513815 | 1 | 5.38 | Local |
| F4 | 06/15/2011 0:00 | 18 | 49.764236 | -109.292067 | 622997 | 5513818 | 1 | 185.40 | Local |
| F4 | 06/15/2011 0:00 | 21 | 49.762639 | -109.291328 | 623054 | 5513641 | 1 | 1215.62 | Local |
| F4 | 06/16/2011 0:00 | 0 | 49.757456 | -109.276470 | 624138 | 5513090 | 1 | 1716.41 | Local |
| F4 | 06/16/2011 0:00 | 3 | 49.766324 | -109.256968 | 625519 | 5514108 | 0 | 3582.43 | Local |
| F4 | 06/16/2011 0:00 | 9 | 49.777450 | -109.210286 | 628851 | 5515424 | 1 | 100.21 | Local |
| F4 | 06/16/2011 0:00 | 12 | 49.777457 | -109.208894 | 628951 | 5515427 | 0 | 122.05 | Local |
| F4 | 06/16/2011 0:00 | 18 | 49.777538 | -109.210585 | 628829 | 5515433 | 1 | 20.04 | Local |
| F4 | 06/16/2011 0:00 | 21 | 49.777585 | -109.210316 | 628849 | 5515439 | 1 | 13.96 | Local |
| F4 | 06/17/2011 0:00 | 0 | 49.777460 | -109.210320 | 628849 | 5515425 | 0 | 3026.45 | Local |
| F4 | 06/17/2011 0:00 | 6 | 49.775227 | -109.252205 | 625839 | 5515106 | 1 | 25.26 | Local |
| F4 | 06/17/2011 0:00 | 9 | 49.775031 | -109.252384 | 625827 | 5515084 | 1 | 18.00 | Local |
| F4 | 06/17/2011 0:00 | 12 | 49.775179 | -109.252485 | 625819 | 5515100 | 1 | 26.05 | Local |
| F4 | 06/17/2011 0:00 | 15 | 49.774969 | -109.252643 | 625808 | 5515076 | 1 | 28.70 | Local |
| F4 | 06/17/2011 0:00 | 18 | 49.775181 | -109.252416 | 625824 | 5515100 | 1 | 1778.55 | Local |

| | | | | | | | | | |
|---|---|---|---|---|---|---|---|---|---|
| F4 | 06/18/2011 0:00 | 21 | 49.769674 | -109.275601 | 624169 | 5514449 | 1 | 879.70 | Local |
| F4 | 06/19/2011 0:00 | 0 | 49.764755 | -109.285167 | 623493 | 5513887 | 1 | 447.68 | Local |
| F4 | 06/19/2011 0:00 | 3 | 49.763939 | -109.291253 | 623056 | 5513786 | 0 | 1803.84 | Local |
| F4 | 06/19/2011 0:00 | 9 | 49.751739 | -109.274751 | 624276 | 5512457 | 0 | 966.38 | Local |
| F4 | 06/19/2011 0:00 | 18 | 49.760327 | -109.276807 | 624106 | 5513408 | 1 | 153.27 | Local |
| F4 | 06/19/2011 0:00 | 21 | 49.758981 | -109.276350 | 624142 | 5513259 | 1 | 983.68 | Local |
| F4 | 06/20/2011 0:00 | 0 | 49.767199 | -109.281403 | 623757 | 5514164 | 0 | 727.43 | Local |
| F4 | 06/20/2011 0:00 | 6 | 49.763876 | -109.290103 | 623139 | 5513781 | 1 | 1132.43 | Local |
| F4 | 06/20/2011 0:00 | 9 | 49.763081 | -109.274430 | 624270 | 5513718 | 0 | 93.68 | Local |
| F4 | 06/20/2011 0:00 | 15 | 49.763781 | -109.273705 | 624321 | 5513797 | 0 | 1657.34 | Local |
| F4 | 06/20/2011 0:00 | 21 | 49.773305 | -109.256005 | 625571 | 5514885 | 1 | 334.51 | Local |
| F4 | 06/21/2011 0:00 | 0 | 49.775138 | -109.252322 | 625831 | 5515095 | 1 | 526.98 | Local |
| F4 | 06/21/2011 0:00 | 3 | 49.776124 | -109.259480 | 625313 | 5515193 | 1 | 527.31 | Local |
| F4 | 06/21/2011 0:00 | 6 | 49.775247 | -109.252284 | 625833 | 5515108 | 0 | 12.82 | Local |
| F4 | 06/21/2011 0:00 | 12 | 49.775137 | -109.252235 | 625837 | 5515095 | 1 | 14.20 | Local |
| F4 | 06/21/2011 0:00 | 15 | 49.775072 | -109.252406 | 625825 | 5515088 | 1 | 6.93 | Local |
| F4 | 06/21/2011 0:00 | 18 | 49.775135 | -109.252406 | 625825 | 5515095 | 0 | 2258.20 | Local |
| F4 | 06/22/2011 0:00 | 0 | 49.767262 | -109.281309 | 623764 | 5514172 | 1 | 741.55 | Local |
| F4 | 06/22/2011 0:00 | 3 | 49.763816 | -109.290124 | 623138 | 5513774 | 0 | 480.93 | Local |
| F4 | 06/22/2011 0:00 | 21 | 49.759514 | -109.290809 | 623100 | 5513295 | 0 | 1259.16 | Local |
| F4 | 06/23/2011 0:00 | 3 | 49.754175 | -109.275396 | 624223 | 5512726 | 1 | 1113.16 | Local |
| F4 | 06/23/2011 0:00 | 6 | 49.763522 | -109.280927 | 623801 | 5513756 | 1 | 408.47 | Local |
| F4 | 06/23/2011 0:00 | 9 | 49.767184 | -109.281371 | 623760 | 5514163 | 1 | 19.52 | Local |
| F4 | 06/23/2011 0:00 | 12 | 49.767113 | -109.281619 | 623742 | 5514155 | 0 | 898.90 | Local |
| F4 | 06/23/2011 0:00 | 18 | 49.772281 | -109.272021 | 624420 | 5514745 | 1 | 1437.42 | Local |
| F4 | 06/23/2011 0:00 | 21 | 49.769763 | -109.252445 | 625836 | 5514498 | 1 | 2019.88 | Local |
| F4 | 06/24/2011 0:00 | 0 | 49.767916 | -109.224546 | 627850 | 5514340 | 0 | 179.54 | Local |
| F4 | 06/24/2011 0:00 | 6 | 49.767963 | -109.227038 | 627670 | 5514341 | 1 | 5.12 | Local |
| F4 | 06/24/2011 0:00 | 9 | 49.767935 | -109.226981 | 627674 | 5514338 | 0 | 1.53 | Local |
| F4 | 06/24/2011 0:00 | 15 | 49.767929 | -109.226962 | 627676 | 5514337 | 0 | 2645.64 | Local |
| F4 | 06/24/2011 0:00 | 21 | 49.776091 | -109.261467 | 625170 | 5515186 | 1 | 1925.17 | Local |
| F4 | 06/25/2011 0:00 | 0 | 49.766389 | -109.283606 | 623601 | 5514071 | 0 | 1080.50 | Local |
| F4 | 06/25/2011 0:00 | 9 | 49.761550 | -109.296614 | 622676 | 5513512 | 0 | 1021.23 | Local |
| F4 | 06/25/2011 0:00 | 15 | 49.757024 | -109.284279 | 623576 | 5513029 | 0 | 1147.45 | Local |
| F4 | 06/25/2011 0:00 | 21 | 49.767182 | -109.281473 | 623752 | 5514162 | 1 | 249.14 | Local |
| F4 | 06/26/2011 0:00 | 0 | 49.765720 | -109.278853 | 623945 | 5514004 | 1 | 126.70 | Local |
| F4 | 06/26/2011 0:00 | 3 | 49.766779 | -109.279501 | 623896 | 5514121 | 0 | 1759.99 | Local |
| F4 | 06/26/2011 0:00 | 15 | 49.770710 | -109.255831 | 625590 | 5514597 | 1 | 386.78 | Local |
| F4 | 06/26/2011 0:00 | 18 | 49.767272 | -109.255017 | 625657 | 5514217 | 1 | 455.36 | Local |
| F4 | 06/26/2011 0:00 | 21 | 49.765455 | -109.249352 | 626070 | 5514024 | 1 | 1520.73 | Local |
| F4 | 06/27/2011 0:00 | 0 | 49.752965 | -109.240755 | 626722 | 5512650 | 1 | 712.58 | Local |
| F4 | 06/27/2011 0:00 | 3 | 49.746862 | -109.237741 | 626955 | 5511977 | 1 | 511.87 | Local |
| F4 | 06/27/2011 0:00 | 6 | 49.742703 | -109.234695 | 627185 | 5511519 | 1 | 404.64 | Local |
| F4 | 06/27/2011 0:00 | 9 | 49.745011 | -109.239037 | 626866 | 5511769 | 0 | 20.05 | Local |
| F4 | 06/27/2011 0:00 | 15 | 49.744886 | -109.238837 | 626881 | 5511755 | 0 | 15.20 | Local |
| F4 | 06/28/2011 0:00 | 0 | 49.744978 | -109.238992 | 626869 | 5511765 | 1 | 10.34 | Local |
| F4 | 06/28/2011 0:00 | 3 | 49.745032 | -109.238875 | 626878 | 5511771 | 1 | 10.14 | Local |
| F4 | 06/28/2011 0:00 | 6 | 49.744952 | -109.238943 | 626873 | 5511762 | 1 | 0.95 | Local |
| F4 | 06/28/2011 0:00 | 9 | 49.744946 | -109.238933 | 626874 | 5511762 | 1 | 5.68 | Local |
| F4 | 06/28/2011 0:00 | 12 | 49.744940 | -109.239011 | 626868 | 5511761 | 1 | 21.03 | Local |
| F4 | 06/28/2011 0:00 | 15 | 49.744751 | -109.239012 | 626868 | 5511740 | 0 | 962.48 | Local |
| F4 | 06/28/2011 0:00 | 21 | 49.751677 | -109.247022 | 626273 | 5512496 | 1 | 5.11 | Local |
| F4 | 06/29/2011 0:00 | 0 | 49.751631 | -109.247019 | 626274 | 5512491 | 1 | 7.71 | Local |
| F4 | 06/29/2011 0:00 | 3 | 49.751562 | -109.247031 | 626273 | 5512483 | 1 | 9.05 | Local |
| F4 | 06/29/2011 0:00 | 6 | 49.751644 | -109.247041 | 626272 | 5512492 | 1 | 13.55 | Local |

| | | | | | | | | | | |
|---|---|---|---|---|---|---|---|---|---|---|
| F4 | 06/29/2011 0:00 | 9 | 49.751543 | -109.247147 | 626265 | 5512481 | 1 | 7.97 | Local |
| F4 | 06/29/2011 0:00 | 12 | 49.751488 | -109.247218 | 626260 | 5512475 | 1 | 13.67 | Local |
| F4 | 06/29/2011 0:00 | 15 | 49.751555 | -109.247059 | 626271 | 5512483 | 1 | 32.50 | Local |
| F4 | 06/29/2011 0:00 | 18 | 49.751840 | -109.247156 | 626263 | 5512514 | 1 | 19.31 | Local |
| F4 | 06/29/2011 0:00 | 21 | 49.751698 | -109.247001 | 626275 | 5512499 | 1 | 137.96 | Local |
| F4 | 06/30/2011 0:00 | 0 | 49.750458 | -109.246957 | 626281 | 5512361 | 1 | 133.96 | Local |
| F4 | 06/30/2011 0:00 | 3 | 49.751662 | -109.247038 | 626272 | 5512494 | 0 | 938.58 | Local |
| F4 | 06/30/2011 0:00 | 9 | 49.759893 | -109.244155 | 626459 | 5513414 | 1 | 19.68 | Local |
| F4 | 06/30/2011 0:00 | 12 | 49.759769 | -109.244351 | 626445 | 5513400 | 1 | 16.39 | Local |
| F4 | 06/30/2011 0:00 | 15 | 49.759911 | -109.244410 | 626440 | 5513416 | 1 | 76.83 | Local |
| F4 | 06/30/2011 0:00 | 18 | 49.759272 | -109.244005 | 626471 | 5513346 | 1 | 70.49 | Local |
| F4 | 06/30/2011 0:00 | 21 | 49.759904 | -109.244080 | 626464 | 5513416 | 0 | 6.62 | Local |
| F4 | 07/01/2011 0:00 | 3 | 49.759848 | -109.244111 | 626462 | 5513409 | 0 | 2006.62 | Local |
| F4 | 07/01/2011 0:00 | 12 | 49.750622 | -109.220174 | 628210 | 5512424 | 1 | 778.98 | Local |
| F4 | 07/01/2011 0:00 | 15 | 49.754207 | -109.210886 | 628870 | 5512839 | 0 | 675.89 | Local |
| F4 | 07/01/2011 0:00 | 21 | 49.758901 | -109.204926 | 629286 | 5513371 | 0 | 1707.26 | Local |
| F4 | 07/02/2011 0:00 | 6 | 49.774104 | -109.208226 | 629008 | 5515055 | 1 | 1074.83 | Local |
| F4 | 07/02/2011 0:00 | 9 | 49.777897 | -109.194498 | 629987 | 5515501 | 0 | 8.44 | Local |
| F4 | 07/02/2011 0:00 | 18 | 49.777821 | -109.194492 | 629987 | 5515492 | 0 | 16.77 | Local |
| F4 | 07/03/2011 0:00 | 0 | 49.777952 | -109.194607 | 629979 | 5515507 | 1 | 13.82 | Local |
| F4 | 07/03/2011 0:00 | 3 | 49.777830 | -109.194578 | 629981 | 5515493 | 1 | 281.59 | Local |
| F4 | 07/03/2011 0:00 | 6 | 49.777299 | -109.190755 | 630258 | 5515441 | 1 | 1927.48 | Local |
| F4 | 07/03/2011 0:00 | 9 | 49.764954 | -109.209541 | 628938 | 5514036 | 0 | 1366.31 | Local |
| F4 | 07/03/2011 0:00 | 18 | 49.766309 | -109.190688 | 630292 | 5514219 | 1 | 1308.81 | Local |
| F4 | 07/03/2011 0:00 | 21 | 49.777793 | -109.194664 | 629975 | 5515489 | 0 | 23.26 | Local |
| F4 | 07/04/2011 0:00 | 3 | 49.777989 | -109.194553 | 629982 | 5515511 | 1 | 731.18 | Local |
| F4 | 07/04/2011 0:00 | 6 | 49.782367 | -109.202129 | 629425 | 5515985 | 1 | 1227.29 | Local |
| F4 | 07/04/2011 0:00 | 9 | 49.778720 | -109.218215 | 628277 | 5515551 | 0 | 1488.42 | Local |
| F4 | 07/04/2011 0:00 | 18 | 49.775752 | -109.238369 | 626834 | 5515187 | 1 | 1700.70 | Local |
| F4 | 07/04/2011 0:00 | 21 | 49.760681 | -109.234358 | 627162 | 5513519 | 0 | 2352.47 | Local |
| F4 | 07/05/2011 0:00 | 3 | 49.739787 | -109.229245 | 627585 | 5511204 | 0 | 991.71 | Local |
| F4 | 07/05/2011 0:00 | 15 | 49.738229 | -109.215696 | 628565 | 5511054 | 1 | 13.55 | Local |
| F4 | 07/05/2011 0:00 | 18 | 49.738115 | -109.215761 | 628561 | 5511042 | 1 | 7.27 | Local |
| F4 | 07/05/2011 0:00 | 21 | 49.738097 | -109.215664 | 628568 | 5511040 | 1 | 10.19 | Local |
| F4 | 07/06/2011 0:00 | 0 | 49.738155 | -109.215774 | 628560 | 5511046 | 1 | 688.14 | Local |
| F4 | 07/06/2011 0:00 | 3 | 49.739355 | -109.206407 | 629232 | 5511195 | 0 | 1960.25 | Local |
| F4 | 07/06/2011 0:00 | 15 | 49.756956 | -109.207904 | 629077 | 5513150 | 0 | 1557.64 | Local |
| F4 | 07/07/2011 0:00 | 0 | 49.750949 | -109.227435 | 627686 | 5512448 | 1 | 22.09 | Local |
| F4 | 07/07/2011 0:00 | 3 | 49.750773 | -109.227292 | 627697 | 5512429 | 1 | 20.35 | Local |
| F4 | 07/07/2011 0:00 | 6 | 49.750947 | -109.227382 | 627690 | 5512448 | 0 | 15.76 | Local |
| F4 | 07/07/2011 0:00 | 15 | 49.750871 | -109.227567 | 627677 | 5512440 | 1 | 30.62 | Local |
| F4 | 07/07/2011 0:00 | 18 | 49.751032 | -109.227223 | 627701 | 5512458 | 0 | 1954.62 | Local |
| F4 | 07/08/2011 0:00 | 3 | 49.735057 | -109.238536 | 626928 | 5510663 | 1 | 1764.42 | Local |
| F4 | 07/08/2011 0:00 | 6 | 49.745331 | -109.257193 | 625557 | 5511774 | 1 | 1110.32 | Local |
| F4 | 07/08/2011 0:00 | 9 | 49.753910 | -109.249307 | 626103 | 5512741 | 1 | 38.44 | Local |
| F4 | 07/08/2011 0:00 | 12 | 49.753565 | -109.249351 | 626101 | 5512702 | 1 | 9.72 | Local |
| F4 | 07/08/2011 0:00 | 15 | 49.753647 | -109.249397 | 626097 | 5512711 | 0 | 1233.21 | Local |
| F4 | 07/08/2011 0:00 | 21 | 49.750829 | -109.265952 | 624912 | 5512370 | 0 | 2772.35 | Local |
| F4 | 07/09/2011 0:00 | 21 | 49.750733 | -109.227474 | 627684 | 5512424 | 1 | 385.80 | Local |
| F4 | 07/10/2011 0:00 | 0 | 49.750550 | -109.222127 | 628070 | 5512413 | 1 | 1240.48 | Local |
| F4 | 07/10/2011 0:00 | 3 | 49.759318 | -109.211484 | 628813 | 5513406 | 1 | 831.40 | Local |
| F4 | 07/10/2011 0:00 | 6 | 49.763062 | -109.201494 | 629523 | 5513839 | 1 | 1726.43 | Local |
| F4 | 07/10/2011 0:00 | 9 | 49.778439 | -109.198194 | 629719 | 5515555 | 0 | 796.72 | Local |
| F4 | 07/10/2011 0:00 | 15 | 49.785588 | -109.197465 | 629753 | 5516351 | 1 | 1380.13 | Local |
| F4 | 07/10/2011 0:00 | 18 | 49.797685 | -109.193181 | 630028 | 5517703 | 1 | 800.41 | Local |

| | | | | | | | | | | |
|---|---|---|---|---|---|---|---|---|---|---|
| F4 | 07/10/2011 0:00 | 21 | 49.797408 | -109.182070 | 630829 | 5517691 | 1 | 316.22 | Local |
| F4 | 07/11/2011 0:00 | 0 | 49.796923 | -109.177741 | 631142 | 5517645 | 1 | 493.69 | Local |
| F4 | 07/11/2011 0:00 | 3 | 49.792885 | -109.174892 | 631358 | 5517201 | 0 | 1381.49 | Local |
| F4 | 07/11/2011 0:00 | 9 | 49.781584 | -109.166923 | 631962 | 5515959 | 1 | 4.45 | Local |
| F4 | 07/11/2011 0:00 | 12 | 49.781551 | -109.166959 | 631959 | 5515955 | 1 | 6.70 | Local |
| F4 | 07/11/2011 0:00 | 15 | 49.781600 | -109.167013 | 631955 | 5515960 | 1 | 1501.80 | Local |
| F4 | 07/11/2011 0:00 | 18 | 49.773976 | -109.149800 | 633215 | 5515143 | 0 | 1381.44 | Local |
| F4 | 07/12/2011 0:00 | 0 | 49.762632 | -109.157615 | 632684 | 5513868 | 1 | 11.54 | Local |
| F4 | 07/12/2011 0:00 | 3 | 49.762689 | -109.157749 | 632674 | 5513874 | 0 | 605.18 | Local |
| F4 | 07/12/2011 0:00 | 18 | 49.757452 | -109.160036 | 632523 | 5513288 | 1 | 995.22 | Local |
| F4 | 07/12/2011 0:00 | 21 | 49.752305 | -109.148736 | 633351 | 5512736 | 0 | 2053.06 | Local |
| F4 | 07/13/2011 0:00 | 3 | 49.762902 | -109.172072 | 631642 | 5513873 | 1 | 2107.10 | Local |
| F4 | 07/13/2011 0:00 | 6 | 49.781818 | -109.170362 | 631714 | 5515979 | 1 | 103.92 | Local |
| F4 | 07/13/2011 0:00 | 9 | 49.782279 | -109.171617 | 631622 | 5516028 | 0 | 2.18 | Local |
| F4 | 07/13/2011 0:00 | 15 | 49.782299 | -109.171613 | 631622 | 5516030 | 1 | 6.28 | Local |
| F4 | 07/13/2011 0:00 | 18 | 49.782243 | -109.171598 | 631623 | 5516024 | 1 | 9.52 | Local |
| F4 | 07/13/2011 0:00 | 21 | 49.782325 | -109.171634 | 631621 | 5516033 | 1 | 4.62 | Local |
| F4 | 07/14/2011 0:00 | 0 | 49.782329 | -109.171698 | 631616 | 5516033 | 1 | 9.35 | Local |
| F4 | 07/14/2011 0:00 | 3 | 49.782261 | -109.171622 | 631622 | 5516026 | 0 | 2194.07 | Local |
| F4 | 07/14/2011 0:00 | 15 | 49.773480 | -109.144338 | 633610 | 5515098 | 1 | 640.64 | Local |
| F4 | 07/14/2011 0:00 | 18 | 49.771787 | -109.135835 | 634227 | 5514925 | 1 | 1615.15 | Local |
| F4 | 07/14/2011 0:00 | 21 | 49.757532 | -109.140127 | 633957 | 5513332 | 0 | 3578.11 | Local |
| F4 | 07/15/2011 0:00 | 6 | 49.764615 | -109.091676 | 637426 | 5514208 | 1 | 1196.31 | Local |
| F4 | 07/15/2011 0:00 | 9 | 49.761819 | -109.107712 | 636279 | 5513867 | 1 | 106.74 | Local |
| F4 | 07/15/2011 0:00 | 12 | 49.760903 | -109.107270 | 636314 | 5513766 | 1 | 107.58 | Local |
| F4 | 07/15/2011 0:00 | 15 | 49.760902 | -109.108763 | 636206 | 5513763 | 1 | 605.16 | Local |
| F4 | 07/15/2011 0:00 | 18 | 49.755471 | -109.109310 | 636182 | 5513159 | 1 | 1506.32 | Local |
| F4 | 07/15/2011 0:00 | 21 | 49.744723 | -109.122032 | 635296 | 5511941 | 1 | 1158.72 | Local |
| F4 | 07/16/2011 0:00 | 0 | 49.750270 | -109.135645 | 634300 | 5512533 | 1 | 878.50 | Local |
| F4 | 07/16/2011 0:00 | 3 | 49.757397 | -109.140903 | 633901 | 5513316 | 1 | 3.16 | Local |
| F4 | 07/16/2011 0:00 | 6 | 49.757371 | -109.140885 | 633903 | 5513313 | 1 | 8.72 | Local |
| F4 | 07/16/2011 0:00 | 9 | 49.757380 | -109.140765 | 633911 | 5513314 | 1 | 97.42 | Local |
| F4 | 07/16/2011 0:00 | 12 | 49.757880 | -109.139654 | 633990 | 5513372 | 1 | 17.88 | Local |
| F4 | 07/16/2011 0:00 | 15 | 49.757840 | -109.139895 | 633973 | 5513367 | 1 | 41.95 | Local |
| F4 | 07/16/2011 0:00 | 18 | 49.758118 | -109.140288 | 633944 | 5513397 | 1 | 86.27 | Local |
| F4 | 07/16/2011 0:00 | 21 | 49.757436 | -109.140860 | 633904 | 5513321 | 1 | 27.75 | Local |
| F4 | 07/17/2011 0:00 | 0 | 49.757531 | -109.141216 | 633878 | 5513330 | 1 | 559.66 | Local |
| F4 | 07/17/2011 0:00 | 3 | 49.760979 | -109.146875 | 633461 | 5513704 | 1 | 21.50 | Local |
| F4 | 07/17/2011 0:00 | 6 | 49.761038 | -109.147160 | 633441 | 5513710 | 1 | 89.31 | Local |
| F4 | 07/17/2011 0:00 | 9 | 49.761440 | -109.148232 | 633362 | 5513752 | 1 | 16.35 | Local |
| F4 | 07/17/2011 0:00 | 12 | 49.761445 | -109.148459 | 633346 | 5513753 | 1 | 11.01 | Local |
| F4 | 07/17/2011 0:00 | 15 | 49.761528 | -109.148378 | 633352 | 5513762 | 1 | 20.04 | Local |
| F4 | 07/17/2011 0:00 | 18 | 49.761685 | -109.148516 | 633341 | 5513779 | 1 | 75.84 | Local |
| F4 | 07/17/2011 0:00 | 21 | 49.761071 | -109.148976 | 633310 | 5513710 | 1 | 505.07 | Local |
| F4 | 07/18/2011 0:00 | 0 | 49.764766 | -109.153055 | 633006 | 5514114 | 1 | 321.88 | Local |
| F4 | 07/18/2011 0:00 | 3 | 49.767494 | -109.154546 | 632891 | 5514414 | 1 | 602.00 | Local |
| F4 | 07/18/2011 0:00 | 6 | 49.767759 | -109.146199 | 633492 | 5514459 | 1 | 462.90 | Local |
| F4 | 07/18/2011 0:00 | 9 | 49.770581 | -109.150924 | 633144 | 5514764 | 1 | 51.22 | Local |
| F4 | 07/18/2011 0:00 | 12 | 49.770150 | -109.151175 | 633127 | 5514716 | 1 | 247.20 | Local |
| F4 | 07/18/2011 0:00 | 15 | 49.770052 | -109.147747 | 633374 | 5514711 | 1 | 12.86 | Local |
| F4 | 07/18/2011 0:00 | 18 | 49.769959 | -109.147854 | 633366 | 5514700 | 1 | 343.27 | Local |
| F4 | 07/18/2011 0:00 | 21 | 49.767033 | -109.146334 | 633484 | 5514378 | 1 | 99.31 | Local |
| F4 | 07/19/2011 0:00 | 0 | 49.766863 | -109.144981 | 633582 | 5514361 | 1 | 14.77 | Local |
| F4 | 07/19/2011 0:00 | 3 | 49.766731 | -109.145005 | 633580 | 5514346 | 1 | 10.81 | Local |
| F4 | 07/19/2011 0:00 | 6 | 49.766829 | -109.145005 | 633580 | 5514357 | 1 | 173.22 | Local |

| | | | | | | | | | |
|---|---|---|---|---|---|---|---|---|---|
| F4 | 07/19/2011 0:00 | 9 | 49.766621 | -109.142622 | 633752 | 5514338 | 0 | 79.03 | Local |
| F4 | 07/19/2011 0:00 | 15 | 49.766892 | -109.141608 | 633825 | 5514370 | 1 | 3.28 | Local |
| F4 | 07/19/2011 0:00 | 18 | 49.766914 | -109.141637 | 633822 | 5514373 | 1 | 277.71 | Local |
| F4 | 07/19/2011 0:00 | 21 | 49.764998 | -109.144111 | 633650 | 5514155 | 1 | 11.86 | Local |
| F4 | 07/20/2011 0:00 | 0 | 49.764942 | -109.144251 | 633640 | 5514149 | 1 | 718.37 | Local |
| F4 | 07/20/2011 0:00 | 3 | 49.771312 | -109.145909 | 633503 | 5514854 | 1 | 625.63 | Local |
| F4 | 07/20/2011 0:00 | 6 | 49.776935 | -109.145610 | 633509 | 5515480 | 1 | 392.16 | Local |
| F4 | 07/20/2011 0:00 | 9 | 49.779470 | -109.141823 | 633774 | 5515768 | 0 | 1587.02 | Local |
| F4 | 07/20/2011 0:00 | 18 | 49.779913 | -109.119795 | 635359 | 5515857 | 1 | 282.67 | Local |
| F4 | 07/20/2011 0:00 | 21 | 49.782426 | -109.120384 | 635310 | 5516135 | 1 | 1631.74 | Local |
| F4 | 07/21/2011 0:00 | 0 | 49.795898 | -109.129367 | 634626 | 5517617 | 1 | 22.63 | Local |
| F4 | 07/21/2011 0:00 | 3 | 49.795799 | -109.129641 | 634606 | 5517605 | 0 | 377.42 | Local |
| F4 | 07/21/2011 0:00 | 9 | 49.792532 | -109.131066 | 634513 | 5517240 | 0 | 237.75 | Local |
| F4 | 07/21/2011 0:00 | 21 | 49.793470 | -109.134034 | 634296 | 5517339 | 1 | 842.93 | Local |
| F4 | 07/22/2011 0:00 | 0 | 49.788048 | -109.125852 | 634900 | 5516750 | 1 | 392.92 | Local |
| F4 | 07/22/2011 0:00 | 3 | 49.787110 | -109.120590 | 635282 | 5516656 | 0 | 68.08 | Local |
| F4 | 07/22/2011 0:00 | 9 | 49.787091 | -109.121535 | 635214 | 5516652 | 1 | 26.58 | Local |
| F4 | 07/22/2011 0:00 | 12 | 49.787192 | -109.121870 | 635189 | 5516663 | 1 | 85.06 | Local |
| F4 | 07/22/2011 0:00 | 15 | 49.786847 | -109.120815 | 635266 | 5516626 | 1 | 43.85 | Local |
| F4 | 07/22/2011 0:00 | 18 | 49.787240 | -109.120754 | 635269 | 5516670 | 1 | 45.86 | Local |
| F4 | 07/22/2011 0:00 | 21 | 49.787215 | -109.121390 | 635224 | 5516666 | 1 | 40.32 | Local |
| F4 | 07/23/2011 0:00 | 0 | 49.787335 | -109.121919 | 635185 | 5516678 | 1 | 38.93 | Local |
| F4 | 07/23/2011 0:00 | 3 | 49.786992 | -109.121808 | 635194 | 5516640 | 1 | 1332.37 | Local |
| F4 | 07/23/2011 0:00 | 6 | 49.776792 | -109.131515 | 634524 | 5515489 | 1 | 548.62 | Local |
| F4 | 07/23/2011 0:00 | 9 | 49.772462 | -109.135165 | 634273 | 5515001 | 0 | 263.43 | Local |
| F4 | 07/23/2011 0:00 | 15 | 49.770095 | -109.135329 | 634268 | 5514738 | 0 | 668.21 | Local |
| F4 | 07/23/2011 0:00 | 21 | 49.764856 | -109.139871 | 633955 | 5514147 | 1 | 531.99 | Local |
| F4 | 07/24/2011 0:00 | 0 | 49.764158 | -109.147178 | 633431 | 5514057 | 0 | 2812.28 | Local |
| F4 | 07/24/2011 0:00 | 6 | 49.778691 | -109.179134 | 631091 | 5515616 | 1 | 1657.45 | Local |
| F4 | 07/24/2011 0:00 | 9 | 49.789723 | -109.194613 | 629947 | 5516815 | 1 | 192.01 | Local |
| F4 | 07/24/2011 0:00 | 12 | 49.787998 | -109.194744 | 629942 | 5516623 | 1 | 4.39 | Local |
| F4 | 07/24/2011 0:00 | 15 | 49.788006 | -109.194804 | 629938 | 5516624 | 0 | 4062.34 | Local |
| F4 | 07/24/2011 0:00 | 21 | 49.769986 | -109.243877 | 626452 | 5514537 | 0 | 1012.95 | Local |
| F4 | 07/25/2011 0:00 | 6 | 49.768659 | -109.257791 | 625454 | 5514366 | 1 | 1865.14 | Local |
| F4 | 07/25/2011 0:00 | 9 | 49.766924 | -109.283548 | 623604 | 5514130 | 0 | 531.14 | Local |
| F4 | 07/25/2011 0:00 | 18 | 49.764157 | -109.289558 | 623178 | 5513813 | 0 | 1579.85 | Local |
| F4 | 07/26/2011 0:00 | 0 | 49.750074 | -109.286655 | 623423 | 5512252 | 0 | 2755.76 | Local |
| F4 | 07/26/2011 0:00 | 6 | 49.750839 | -109.248426 | 626175 | 5512401 | 1 | 1216.09 | Local |
| F4 | 07/26/2011 0:00 | 9 | 49.750731 | -109.231548 | 627391 | 5512417 | 1 | 88.82 | Local |
| F4 | 07/26/2011 0:00 | 12 | 49.750186 | -109.230647 | 627457 | 5512358 | 1 | 1.20 | Local |
| F4 | 07/26/2011 0:00 | 15 | 49.750186 | -109.230663 | 627456 | 5512358 | 1 | 748.28 | Local |
| F4 | 07/26/2011 0:00 | 18 | 49.752445 | -109.220880 | 628154 | 5512626 | 1 | 1694.97 | Local |
| F4 | 07/26/2011 0:00 | 21 | 49.761468 | -109.201918 | 629496 | 5513662 | 0 | 19.50 | Local |
| F4 | 07/27/2011 0:00 | 6 | 49.761293 | -109.201907 | 629498 | 5513642 | 1 | 1.49 | Local |
| F4 | 07/27/2011 0:00 | 9 | 49.761305 | -109.201897 | 629498 | 5513643 | 0 | 1.81 | Local |
| F4 | 07/27/2011 0:00 | 18 | 49.761303 | -109.201922 | 629496 | 5513643 | 1 | 941.58 | Local |
| F4 | 07/27/2011 0:00 | 21 | 49.767026 | -109.192288 | 630175 | 5514296 | 1 | 1472.25 | Local |
| F4 | 07/28/2011 0:00 | 0 | 49.776585 | -109.178145 | 631167 | 5515383 | 1 | 15.35 | Local |
| F4 | 07/28/2011 0:00 | 3 | 49.776561 | -109.178355 | 631152 | 5515380 | 1 | 7.91 | Local |
| F4 | 07/28/2011 0:00 | 6 | 49.776566 | -109.178245 | 631160 | 5515381 | 1 | 1093.53 | Local |
| F4 | 07/28/2011 0:00 | 9 | 49.772323 | -109.164548 | 632158 | 5514933 | 1 | 6.29 | Local |
| F4 | 07/28/2011 0:00 | 12 | 49.772330 | -109.164634 | 632152 | 5514934 | 0 | 1069.48 | Local |
| F4 | 07/28/2011 0:00 | 21 | 49.763917 | -109.157439 | 632693 | 5514012 | 1 | 2543.66 | Local |
| F4 | 07/29/2011 0:00 | 0 | 49.746130 | -109.135241 | 634340 | 5512074 | 1 | 6.06 | Local |
| F4 | 07/29/2011 0:00 | 3 | 49.746181 | -109.135209 | 634342 | 5512079 | 1 | 40.51 | Local |

| | | | | | | | | | |
|---|---|---|---|---|---|---|---|---|---|
| F4 | 07/29/2011 0:00 | 6 | 49.745820 | -109.135288 | 634338 | 5512039 | 1 | 35.14 | Local |
| F4 | 07/29/2011 0:00 | 9 | 49.746134 | -109.135241 | 634340 | 5512074 | 0 | 715.88 | Local |
| F4 | 07/29/2011 0:00 | 15 | 49.749613 | -109.143601 | 633729 | 5512446 | 1 | 4.07 | Local |
| F4 | 07/29/2011 0:00 | 18 | 49.749579 | -109.143622 | 633727 | 5512442 | 0 | 777.28 | Local |
| F4 | 07/30/2011 0:00 | 3 | 49.746043 | -109.134317 | 634407 | 5512066 | 1 | 1839.98 | Local |
| F4 | 07/30/2011 0:00 | 6 | 49.743573 | -109.109070 | 636233 | 5511837 | 1 | 992.06 | Local |
| F4 | 07/30/2011 0:00 | 9 | 49.747947 | -109.097071 | 637085 | 5512345 | 1 | 10.80 | Local |
| F4 | 07/30/2011 0:00 | 12 | 49.747850 | -109.097070 | 637085 | 5512334 | 0 | 381.27 | Local |
| F4 | 07/30/2011 0:00 | 18 | 49.749468 | -109.101735 | 636745 | 5512505 | 1 | 2186.12 | Local |
| F4 | 07/30/2011 0:00 | 21 | 49.768263 | -109.110628 | 636051 | 5514578 | 0 | 5119.86 | Local |
| F4 | 07/31/2011 0:00 | 3 | 49.795155 | -109.168341 | 631823 | 5517465 | 0 | 342.04 | Local |
| F4 | 07/31/2011 0:00 | 9 | 49.794202 | -109.172859 | 631500 | 5517351 | 0 | 679.09 | Local |
| F4 | 07/31/2011 0:00 | 21 | 49.799304 | -109.178041 | 631114 | 5517909 | 0 | 1608.09 | Local |
| F4 | 08/01/2011 0:00 | 3 | 49.789672 | -109.194702 | 629940 | 5516809 | 0 | 18.26 | Local |
| F4 | 08/01/2011 0:00 | 18 | 49.789628 | -109.194457 | 629958 | 5516805 | 1 | 43.46 | Local |
| F4 | 08/01/2011 0:00 | 21 | 49.789399 | -109.194946 | 629924 | 5516779 | 1 | 33.84 | Local |
| F4 | 08/02/2011 0:00 | 0 | 49.789641 | -109.194661 | 629943 | 5516806 | 1 | 6.58 | Local |
| F4 | 08/02/2011 0:00 | 3 | 49.789665 | -109.194578 | 629949 | 5516809 | 0 | 38.46 | Local |
| F4 | 08/02/2011 0:00 | 9 | 49.789409 | -109.194937 | 629924 | 5516780 | 0 | 21.39 | Local |
| F4 | 08/02/2011 0:00 | 15 | 49.789563 | -109.195115 | 629911 | 5516797 | 1 | 506.23 | Local |
| F4 | 08/02/2011 0:00 | 18 | 49.788757 | -109.202036 | 629415 | 5516695 | 0 | 619.83 | Local |
| F4 | 08/03/2011 0:00 | 0 | 49.787195 | -109.210300 | 628824 | 5516507 | 1 | 12.37 | Local |
| F4 | 08/03/2011 0:00 | 3 | 49.787106 | -109.210403 | 628817 | 5516497 | 0 | 78.39 | Local |
| F4 | 08/03/2011 0:00 | 9 | 49.787802 | -109.210227 | 628828 | 5516575 | 1 | 860.87 | Local |
| F4 | 08/03/2011 0:00 | 12 | 49.782403 | -109.218796 | 628226 | 5515960 | 0 | 178.19 | Local |
| F4 | 08/03/2011 0:00 | 18 | 49.780942 | -109.219813 | 628156 | 5515796 | 1 | 1946.81 | Local |
| F4 | 08/03/2011 0:00 | 21 | 49.777313 | -109.246261 | 626262 | 5515347 | 1 | 1724.43 | Local |
| F4 | 08/04/2011 0:00 | 0 | 49.783337 | -109.224194 | 627834 | 5516054 | 0 | 1077.06 | Local |
| F4 | 08/04/2011 0:00 | 6 | 49.787061 | -109.210385 | 628819 | 5516492 | 0 | 1260.02 | Local |
| F4 | 08/04/2011 0:00 | 12 | 49.776277 | -109.205015 | 629234 | 5515302 | 1 | 76.32 | Local |
| F4 | 08/04/2011 0:00 | 15 | 49.775605 | -109.204799 | 629251 | 5515228 | 0 | 2865.19 | Local |
| F4 | 08/05/2011 0:00 | 0 | 49.756523 | -109.231528 | 627377 | 5513061 | 0 | 1337.77 | Local |
| F4 | 08/05/2011 0:00 | 6 | 49.755678 | -109.213005 | 628713 | 5512999 | 1 | 61.30 | Local |
| F4 | 08/05/2011 0:00 | 9 | 49.755532 | -109.213825 | 628654 | 5512981 | 0 | 6.91 | Local |
| F4 | 08/05/2011 0:00 | 15 | 49.755476 | -109.213866 | 628652 | 5512975 | 0 | 37.88 | Local |
| F4 | 08/05/2011 0:00 | 21 | 49.755701 | -109.214259 | 628623 | 5512999 | 0 | 16.04 | Local |
| F4 | 08/06/2011 0:00 | 3 | 49.755606 | -109.214426 | 628611 | 5512988 | 1 | 29.77 | Local |
| F4 | 08/06/2011 0:00 | 6 | 49.755769 | -109.214099 | 628634 | 5513007 | 0 | 635.16 | Local |
| F4 | 08/06/2011 0:00 | 21 | 49.753960 | -109.205737 | 629241 | 5512820 | 0 | 635.71 | Local |
| F4 | 08/07/2011 0:00 | 3 | 49.755600 | -109.214190 | 628628 | 5512988 | 1 | 18.88 | Local |
| F4 | 08/07/2011 0:00 | 6 | 49.755764 | -109.214255 | 628623 | 5513006 | 1 | 38.59 | Local |
| F4 | 08/07/2011 0:00 | 9 | 49.755422 | -109.214168 | 628630 | 5512968 | 1 | 32.05 | Local |
| F4 | 08/07/2011 0:00 | 12 | 49.755683 | -109.213978 | 628643 | 5512998 | 0 | 1896.09 | Local |
| F4 | 08/07/2011 0:00 | 21 | 49.761237 | -109.238863 | 626836 | 5513573 | 1 | 843.74 | Local |
| F4 | 08/08/2011 0:00 | 0 | 49.765881 | -109.248126 | 626157 | 5514073 | 0 | 3476.92 | Local |
| F4 | 08/08/2011 0:00 | 6 | 49.777445 | -109.203273 | 629356 | 5515435 | 0 | 1423.28 | Local |
| F4 | 08/09/2011 0:00 | 0 | 49.790208 | -109.204753 | 629216 | 5516852 | 1 | 15.83 | Local |
| F4 | 08/09/2011 0:00 | 3 | 49.790346 | -109.204700 | 629219 | 5516867 | 1 | 5.42 | Local |
| F4 | 08/09/2011 0:00 | 6 | 49.790318 | -109.204639 | 629224 | 5516864 | 0 | 134.76 | Local |
| F4 | 08/09/2011 0:00 | 18 | 49.790799 | -109.202921 | 629346 | 5516921 | 0 | 6402.41 | Local |
| F4 | 08/10/2011 0:00 | 3 | 49.773814 | -109.117963 | 635508 | 5515182 | 1 | 2871.94 | Local |
| F4 | 08/10/2011 0:00 | 6 | 49.749995 | -109.102558 | 636684 | 5512562 | 1 | 323.33 | Local |
| F4 | 08/10/2011 0:00 | 9 | 49.749251 | -109.106896 | 636373 | 5512472 | 1 | 495.13 | Local |
| F4 | 08/10/2011 0:00 | 12 | 49.748123 | -109.113543 | 635898 | 5512334 | 1 | 40.61 | Local |
| F4 | 08/10/2011 0:00 | 15 | 49.747946 | -109.113050 | 635934 | 5512315 | 1 | 11.01 | Local |

| | | | | | | | | | |
|---|---|---|---|---|---|---|---|---|---|
| F4 | 08/10/2011 0:00 | 18 | 49.748010 | -109.113166 | 635925 | 5512322 | 0 | 2009.08 | Local |
| F4 | 08/11/2011 0:00 | 0 | 49.758902 | -109.090918 | 637497 | 5513574 | 1 | 2609.14 | Local |
| F4 | 08/11/2011 0:00 | 3 | 49.781640 | -109.081986 | 638076 | 5516118 | 0 | 2084.00 | Local |
| F4 | 08/11/2011 0:00 | 9 | 49.777554 | -109.110229 | 636054 | 5515612 | 1 | 362.39 | Local |
| F4 | 08/11/2011 0:00 | 12 | 49.774364 | -109.111265 | 635989 | 5515256 | 0 | 292.56 | Local |
| F4 | 08/11/2011 0:00 | 21 | 49.776978 | -109.111735 | 635947 | 5515545 | 1 | 307.21 | Local |
| F4 | 08/12/2011 0:00 | 0 | 49.776089 | -109.115774 | 635659 | 5515439 | 1 | 10.44 | Local |
| F4 | 08/12/2011 0:00 | 3 | 49.776139 | -109.115897 | 635650 | 5515444 | 1 | 1804.43 | Local |
| F4 | 08/12/2011 0:00 | 6 | 49.763839 | -109.132237 | 634508 | 5514048 | 0 | 4863.54 | Local |
| F4 | 08/13/2011 0:00 | 0 | 49.742028 | -109.190747 | 630353 | 5511520 | 0 | 70.73 | Local |
| F4 | 08/13/2011 0:00 | 6 | 49.742121 | -109.191718 | 630283 | 5511528 | 0 | 276.52 | Local |
| F4 | 08/13/2011 0:00 | 12 | 49.743593 | -109.194810 | 630056 | 5511687 | 0 | 6.66 | Local |
| F4 | 08/13/2011 0:00 | 18 | 49.743550 | -109.194875 | 630051 | 5511682 | 0 | 2864.61 | Local |
| F4 | 08/14/2011 0:00 | 3 | 49.742632 | -109.234601 | 627192 | 5511512 | 0 | 142.93 | Local |
| F4 | 08/14/2011 0:00 | 9 | 49.742776 | -109.236572 | 627049 | 5511524 | 0 | 3248.14 | Local |
| F4 | 08/15/2011 0:00 | 0 | 49.767932 | -109.259486 | 625334 | 5514282 | 0 | 2053.87 | Local |
| F4 | 08/15/2011 0:00 | 6 | 49.757293 | -109.282793 | 623683 | 5513061 | 0 | 2748.91 | Local |
| F4 | 08/15/2011 0:00 | 18 | 49.751553 | -109.319906 | 621024 | 5512362 | 0 | 558.73 | Local |
| F4 | 08/16/2011 0:00 | 3 | 49.746530 | -109.320033 | 621027 | 5511804 | 0 | 2343.94 | Local |
| F4 | 08/16/2011 0:00 | 12 | 49.749641 | -109.287859 | 623337 | 5512202 | 0 | 19.60 | Local |
| F4 | 08/16/2011 0:00 | 18 | 49.749792 | -109.287718 | 623347 | 5512219 | 0 | 445.84 | Local |
| F4 | 08/17/2011 0:00 | 0 | 49.747696 | -109.282443 | 623732 | 5511995 | 1 | 19.59 | Local |
| F4 | 08/17/2011 0:00 | 3 | 49.747521 | -109.282414 | 623735 | 5511975 | 0 | 880.11 | Local |
| F4 | 08/17/2011 0:00 | 9 | 49.755134 | -109.285754 | 623475 | 5512816 | 0 | 3644.13 | Local |
| F4 | 08/18/2011 0:00 | 3 | 49.749755 | -109.335647 | 619894 | 5512137 | 0 | 563.73 | Local |
| F4 | 08/18/2011 0:00 | 12 | 49.745863 | -109.330634 | 620265 | 5511713 | 0 | 704.05 | Local |
| F4 | 08/18/2011 0:00 | 18 | 49.745509 | -109.320879 | 620969 | 5511689 | 0 | 3252.08 | Local |
| F4 | 08/19/2011 0:00 | 6 | 49.747237 | -109.275825 | 624210 | 5511954 | 0 | 9.96 | Local |
| F4 | 08/19/2011 0:00 | 18 | 49.747205 | -109.275955 | 624201 | 5511951 | 0 | 27.21 | Local |
| F4 | 08/20/2011 0:00 | 3 | 49.747142 | -109.275590 | 624227 | 5511944 | 1 | 14.62 | Local |
| F4 | 08/20/2011 0:00 | 6 | 49.747233 | -109.275737 | 624217 | 5511954 | 0 | 1823.97 | Local |
| F4 | 08/21/2011 0:00 | 6 | 49.762593 | -109.284618 | 623538 | 5513647 | 0 | 1322.60 | Local |
| F4 | 08/21/2011 0:00 | 18 | 49.772242 | -109.273882 | 624286 | 5514738 | 1 | 1749.55 | Local |
| F4 | 08/21/2011 0:00 | 21 | 49.768512 | -109.250283 | 625995 | 5514362 | 0 | 1044.95 | Local |
| F4 | 08/22/2011 0:00 | 6 | 49.768460 | -109.235775 | 627040 | 5514381 | 1 | 728.91 | Local |
| F4 | 08/22/2011 0:00 | 9 | 49.761944 | -109.234684 | 627135 | 5513658 | 1 | 554.56 | Local |
| F4 | 08/22/2011 0:00 | 12 | 49.756988 | -109.235544 | 627086 | 5513106 | 1 | 24.32 | Local |
| F4 | 08/22/2011 0:00 | 15 | 49.756781 | -109.235655 | 627079 | 5513083 | 1 | 12.72 | Local |
| F4 | 08/22/2011 0:00 | 18 | 49.756891 | -109.235607 | 627082 | 5513095 | 1 | 27.42 | Local |
| F4 | 08/22/2011 0:00 | 21 | 49.757138 | -109.235593 | 627082 | 5513123 | 1 | 260.48 | Local |
| F4 | 08/23/2011 0:00 | 0 | 49.756896 | -109.239190 | 626824 | 5513090 | 1 | 2627.05 | Local |
| F4 | 08/23/2011 0:00 | 3 | 49.747260 | -109.205899 | 629247 | 5512075 | 1 | 1319.39 | Local |
| F4 | 08/23/2011 0:00 | 6 | 49.754106 | -109.190942 | 630306 | 5512862 | 1 | 1193.43 | Local |
| F4 | 08/23/2011 0:00 | 9 | 49.758859 | -109.205794 | 629224 | 5513365 | 0 | 8.88 | Local |
| F4 | 08/23/2011 0:00 | 18 | 49.758938 | -109.205805 | 629223 | 5513374 | 1 | 525.79 | Local |
| F4 | 08/23/2011 0:00 | 21 | 49.757500 | -109.198852 | 629728 | 5513226 | 1 | 231.43 | Local |
| F4 | 08/24/2011 0:00 | 0 | 49.755612 | -109.197502 | 629830 | 5513018 | 0 | 4982.12 | Local |
| F4 | 08/24/2011 0:00 | 6 | 49.766826 | -109.130542 | 634621 | 5514383 | 1 | 1449.35 | Local |
| F4 | 08/24/2011 0:00 | 9 | 49.755865 | -109.119658 | 635436 | 5513184 | 1 | 60.30 | Local |
| F4 | 08/24/2011 0:00 | 12 | 49.756393 | -109.119844 | 635421 | 5513242 | 0 | 303.73 | Local |
| F4 | 08/24/2011 0:00 | 21 | 49.753807 | -109.118491 | 635526 | 5512957 | 1 | 179.56 | Local |
| F4 | 08/25/2011 0:00 | 0 | 49.753093 | -109.120728 | 635366 | 5512874 | 1 | 258.76 | Local |
| F4 | 08/25/2011 0:00 | 3 | 49.755034 | -109.122710 | 635218 | 5513086 | 1 | 1258.46 | Local |
| F4 | 08/25/2011 0:00 | 6 | 49.762477 | -109.135869 | 634250 | 5513890 | 1 | 448.44 | Local |
| F4 | 08/25/2011 0:00 | 9 | 49.762047 | -109.142059 | 633805 | 5513831 | 1 | 19.23 | Local |

| | | | | | | | | | |
|---|---|---|---|---|---|---|---|---|---|
| F4 | 08/25/2011 0:00 | 12 | 49.762219 | -109.142030 | 633807 | 5513850 | 0 | 310.36 | Local |
| F4 | 08/25/2011 0:00 | 18 | 49.761643 | -109.146246 | 633505 | 5513779 | 1 | 1915.77 | Local |
| F4 | 08/25/2011 0:00 | 21 | 49.753687 | -109.169832 | 631828 | 5512852 | 1 | 2146.80 | Local |
| F4 | 08/26/2011 0:00 | 0 | 49.757035 | -109.199179 | 629705 | 5513173 | 0 | 2788.26 | Local |
| F4 | 08/26/2011 0:00 | 9 | 49.770049 | -109.232264 | 627288 | 5514564 | 1 | 351.37 | Local |
| F4 | 08/26/2011 0:00 | 12 | 49.773083 | -109.233629 | 627182 | 5514898 | 0 | 251.61 | Local |
| F4 | 08/27/2011 0:00 | 6 | 49.771031 | -109.235103 | 627081 | 5514668 | 1 | 493.90 | Local |
| F4 | 08/27/2011 0:00 | 9 | 49.771967 | -109.241807 | 626596 | 5514761 | 0 | 487.88 | Local |
| F4 | 08/27/2011 0:00 | 21 | 49.769142 | -109.236624 | 626977 | 5514455 | 1 | 660.17 | Local |
| F4 | 08/28/2011 0:00 | 0 | 49.764869 | -109.230261 | 627446 | 5513991 | 1 | 1514.85 | Local |
| F4 | 08/28/2011 0:00 | 3 | 49.778368 | -109.233090 | 627207 | 5515487 | 1 | 1189.61 | Local |
| F4 | 08/28/2011 0:00 | 6 | 49.779314 | -109.216635 | 628389 | 5515620 | 1 | 1712.23 | Local |
| F4 | 08/28/2011 0:00 | 9 | 49.780378 | -109.240356 | 626679 | 5515698 | 1 | 254.93 | Local |
| F4 | 08/28/2011 0:00 | 12 | 49.781260 | -109.243624 | 626441 | 5515791 | 0 | 1013.22 | Local |
| F4 | 08/28/2011 0:00 | 18 | 49.772770 | -109.238519 | 626831 | 5514855 | 1 | 812.91 | Local |
| F4 | 08/28/2011 0:00 | 21 | 49.766079 | -109.233971 | 627176 | 5514119 | 1 | 1389.27 | Local |
| F4 | 08/29/2011 0:00 | 0 | 49.753759 | -109.230771 | 627439 | 5512755 | 1 | 1851.73 | Local |
| F4 | 08/29/2011 0:00 | 3 | 49.737396 | -109.226007 | 627825 | 5510944 | 1 | 1756.98 | Local |
| F4 | 08/29/2011 0:00 | 6 | 49.738394 | -109.250337 | 626069 | 5511014 | 0 | 35.14 | Local |
| F4 | 08/29/2011 0:00 | 12 | 49.738241 | -109.249910 | 626100 | 5510998 | 0 | 281.09 | Local |
| F4 | 08/29/2011 0:00 | 21 | 49.740684 | -109.248911 | 626166 | 5511271 | 0 | 463.01 | Local |
| F4 | 08/30/2011 0:00 | 3 | 49.737819 | -109.253573 | 625837 | 5510945 | 0 | 1813.20 | Local |
| F4 | 08/31/2011 0:00 | 0 | 49.754124 | -109.253351 | 625811 | 5512758 | 1 | 183.18 | Local |
| F4 | 08/31/2011 0:00 | 3 | 49.755181 | -109.251401 | 625949 | 5512878 | 1 | 1590.32 | Local |
| F4 | 08/31/2011 0:00 | 6 | 49.765929 | -109.236837 | 626970 | 5514098 | 0 | 1996.13 | Local |
| F4 | 08/31/2011 0:00 | 15 | 49.783529 | -109.242286 | 626532 | 5516045 | 0 | 1371.25 | Local |
| F4 | 09/01/2011 0:00 | 6 | 49.792831 | -109.254789 | 625608 | 5517058 | 0 | 3690.37 | Local |
| F4 | 09/01/2011 0:00 | 21 | 49.760421 | -109.243771 | 626485 | 5513474 | 1 | 119.38 | Local |
| F4 | 09/02/2011 0:00 | 0 | 49.760022 | -109.245309 | 626375 | 5513427 | 0 | 4131.82 | Local |
| F4 | 09/02/2011 0:00 | 6 | 49.772437 | -109.191242 | 630236 | 5514899 | 1 | 11.10 | Local |
| F4 | 09/02/2011 0:00 | 9 | 49.772416 | -109.191092 | 630247 | 5514897 | 0 | 469.26 | Local |
| F4 | 09/02/2011 0:00 | 18 | 49.768277 | -109.189821 | 630349 | 5514439 | 1 | 1947.03 | Local |
| F4 | 09/02/2011 0:00 | 21 | 49.761718 | -109.214883 | 628562 | 5513667 | 1 | 2.92 | Local |
| F4 | 09/03/2011 0:00 | 0 | 49.761716 | -109.214843 | 628565 | 5513667 | 1 | 4.56 | Local |
| F4 | 09/03/2011 0:00 | 3 | 49.761676 | -109.214833 | 628566 | 5513662 | 1 | 1110.39 | Local |
| F4 | 09/03/2011 0:00 | 6 | 49.763382 | -109.230021 | 627467 | 5513826 | 1 | 1337.09 | Local |
| F4 | 09/03/2011 0:00 | 9 | 49.761389 | -109.248326 | 626154 | 5513574 | 0 | 28.24 | Local |
| F4 | 09/03/2011 0:00 | 21 | 49.761589 | -109.248085 | 626171 | 5513596 | 1 | 6.57 | Local |
| F4 | 09/04/2011 0:00 | 0 | 49.761531 | -109.248106 | 626170 | 5513590 | 0 | 51.35 | Local |
| F4 | 09/04/2011 0:00 | 12 | 49.761071 | -109.248056 | 626175 | 5513539 | 0 | 24.60 | Local |
| F4 | 09/05/2011 0:00 | 0 | 49.761189 | -109.248344 | 626154 | 5513551 | 0 | 365.60 | Local |
| F4 | 09/05/2011 0:00 | 12 | 49.757934 | -109.247635 | 626213 | 5513191 | 0 | 448.89 | Local |
| F4 | 09/06/2011 0:00 | 12 | 49.761925 | -109.248571 | 626135 | 5513633 | 0 | 63.81 | Local |
| F4 | 09/07/2011 0:00 | 0 | 49.761391 | -109.248245 | 626160 | 5513574 | 0 | 322.99 | Local |
| F4 | 09/07/2011 0:00 | 6 | 49.759618 | -109.244693 | 626421 | 5513383 | 1 | 842.75 | Local |
| F4 | 09/07/2011 0:00 | 9 | 49.754629 | -109.253499 | 625799 | 5512813 | 0 | 741.30 | Local |
| F4 | 09/07/2011 0:00 | 18 | 49.761293 | -109.253264 | 625799 | 5513555 | 1 | 477.63 | Local |
| F4 | 09/07/2011 0:00 | 21 | 49.759865 | -109.247011 | 626253 | 5513406 | 1 | 4.72 | Local |
| F4 | 09/08/2011 0:00 | 0 | 49.759835 | -109.247057 | 626250 | 5513403 | 1 | 1510.26 | Local |
| F4 | 09/08/2011 0:00 | 3 | 49.772452 | -109.239298 | 626776 | 5514819 | 1 | 1438.18 | Local |
| F4 | 09/08/2011 0:00 | 6 | 49.767944 | -109.258015 | 625440 | 5514286 | 0 | 1677.89 | Local |
| F4 | 09/08/2011 0:00 | 21 | 49.753117 | -109.253702 | 625789 | 5512645 | 0 | 171.39 | Local |
| F4 | 09/09/2011 0:00 | 3 | 49.754560 | -109.252866 | 625845 | 5512807 | 0 | 1140.24 | Local |
| F4 | 09/09/2011 0:00 | 12 | 49.764388 | -109.248355 | 626144 | 5513907 | 1 | 174.06 | Local |
| F4 | 09/09/2011 0:00 | 15 | 49.762943 | -109.247427 | 626215 | 5513748 | 1 | 10.50 | Local |

| | | | | | | | | | |
|---|---|---|---|---|---|---|---|---|---|
| F4 | 09/09/2011 0:00 | 18 | 49.762850 | -109.247452 | 626214 | 5513738 | 0 | 1778.25 | Local |
| F4 | 09/10/2011 0:00 | 3 | 49.749761 | -109.261631 | 625226 | 5512259 | 1 | 1391.12 | Local |
| F4 | 09/10/2011 0:00 | 6 | 49.760772 | -109.252468 | 625858 | 5513498 | 1 | 688.82 | Local |
| F4 | 09/10/2011 0:00 | 9 | 49.754642 | -109.251102 | 625972 | 5512819 | 0 | 666.10 | Local |
| F4 | 09/10/2011 0:00 | 18 | 49.760010 | -109.247001 | 626253 | 5513423 | 0 | 786.43 | Local |
| F4 | 09/11/2011 0:00 | 12 | 49.766028 | -109.252736 | 625825 | 5514082 | 0 | 610.51 | Local |
| F4 | 09/12/2011 0:00 | 0 | 49.761337 | -109.248334 | 626154 | 5513568 | 0 | 738.79 | Local |
| F4 | 09/12/2011 0:00 | 21 | 49.755028 | -109.251550 | 625939 | 5512861 | 1 | 985.48 | Local |
| F4 | 09/13/2011 0:00 | 0 | 49.763856 | -109.250352 | 626002 | 5513845 | 0 | 500.63 | Local |
| F4 | 09/13/2011 0:00 | 6 | 49.766965 | -109.255379 | 625632 | 5514182 | 1 | 3.51 | Local |
| F4 | 09/13/2011 0:00 | 9 | 49.766954 | -109.255425 | 625629 | 5514180 | 1 | 189.27 | Local |
| F4 | 09/13/2011 0:00 | 12 | 49.768383 | -109.256854 | 625522 | 5514337 | 1 | 111.76 | Local |
| F4 | 09/13/2011 0:00 | 15 | 49.767576 | -109.257780 | 625458 | 5514246 | 1 | 121.03 | Local |
| F4 | 09/13/2011 0:00 | 18 | 49.766665 | -109.258699 | 625394 | 5514143 | 1 | 48.97 | Local |
| F4 | 09/13/2011 0:00 | 21 | 49.767077 | -109.258458 | 625410 | 5514189 | 0 | 42.34 | Local |
| F4 | 09/14/2011 0:00 | 15 | 49.766815 | -109.258884 | 625380 | 5514159 | 0 | 226.44 | Local |
| F4 | 09/14/2011 0:00 | 21 | 49.768835 | -109.259273 | 625347 | 5514383 | 1 | 3.94 | Local |
| F4 | 09/15/2011 0:00 | 0 | 49.768871 | -109.259270 | 625347 | 5514387 | 0 | 38.76 | Local |
| F4 | 09/15/2011 0:00 | 6 | 49.768903 | -109.259806 | 625308 | 5514390 | 1 | 262.73 | Local |
| F4 | 09/15/2011 0:00 | 9 | 49.770664 | -109.262238 | 625129 | 5514581 | 1 | 474.80 | Local |
| F4 | 09/15/2011 0:00 | 12 | 49.769113 | -109.256096 | 625575 | 5514419 | 1 | 99.23 | Local |
| F4 | 09/15/2011 0:00 | 15 | 49.768546 | -109.257160 | 625500 | 5514355 | 0 | 438.12 | Local |
| F4 | 09/16/2011 0:00 | 9 | 49.771601 | -109.261002 | 625215 | 5514688 | 1 | 1008.48 | Local |
| F4 | 09/16/2011 0:00 | 12 | 49.777040 | -109.249797 | 626008 | 5515311 | 1 | 344.14 | Local |
| F4 | 09/16/2011 0:00 | 15 | 49.773945 | -109.249871 | 626011 | 5514967 | 1 | 670.61 | Local |
| F4 | 09/16/2011 0:00 | 18 | 49.768165 | -109.252526 | 625834 | 5514320 | 1 | 1430.88 | Local |
| F4 | 09/16/2011 0:00 | 21 | 49.777079 | -109.238197 | 626843 | 5515335 | 1 | 1547.13 | Local |
| F4 | 09/17/2011 0:00 | 0 | 49.781847 | -109.218013 | 628283 | 5515899 | 1 | 33.27 | Local |
| F4 | 09/17/2011 0:00 | 3 | 49.782116 | -109.218216 | 628268 | 5515929 | 1 | 58.07 | Local |
| F4 | 09/17/2011 0:00 | 6 | 49.781717 | -109.218736 | 628232 | 5515884 | 0 | 42.24 | Local |
| F4 | 09/17/2011 0:00 | 18 | 49.781957 | -109.218283 | 628264 | 5515911 | 1 | 14.56 | Local |
| F4 | 09/17/2011 0:00 | 21 | 49.781827 | -109.218262 | 628265 | 5515897 | 1 | 9.33 | Local |
| F4 | 09/18/2011 0:00 | 0 | 49.781866 | -109.218377 | 628257 | 5515901 | 1 | 12.85 | Local |
| F4 | 09/18/2011 0:00 | 3 | 49.781971 | -109.218452 | 628251 | 5515912 | 1 | 5.78 | Local |
| F4 | 09/18/2011 0:00 | 6 | 49.782021 | -109.218470 | 628250 | 5515918 | 0 | 1600.35 | Local |
| F4 | 09/18/2011 0:00 | 12 | 49.767980 | -109.213603 | 628637 | 5514365 | 0 | 388.85 | Local |
| F4 | 09/18/2011 0:00 | 18 | 49.764617 | -109.212123 | 628753 | 5513994 | 1 | 2111.56 | Local |
| F4 | 09/18/2011 0:00 | 21 | 49.751489 | -109.190946 | 630313 | 5512571 | 0 | 2877.40 | Local |
| F4 | 09/19/2011 0:00 | 6 | 49.768682 | -109.161096 | 632416 | 5514535 | 1 | 2346.20 | Local |
| F4 | 09/19/2011 0:00 | 9 | 49.777345 | -109.131391 | 634531 | 5515551 | 0 | 94.32 | Local |
| F4 | 09/19/2011 0:00 | 21 | 49.776497 | -109.131365 | 634536 | 5515456 | 1 | 5.68 | Local |
| F4 | 09/20/2011 0:00 | 0 | 49.776466 | -109.131303 | 634540 | 5515453 | 1 | 556.14 | Local |
| F4 | 09/20/2011 0:00 | 3 | 49.771529 | -109.132540 | 634465 | 5514902 | 1 | 1154.84 | Local |
| F4 | 09/20/2011 0:00 | 6 | 49.763847 | -109.143330 | 633709 | 5514029 | 1 | 891.48 | Local |
| F4 | 09/20/2011 0:00 | 9 | 49.759586 | -109.153812 | 632966 | 5513536 | 1 | 1352.00 | Local |
| F4 | 09/20/2011 0:00 | 12 | 49.762605 | -109.171992 | 631648 | 5513840 | 1 | 159.88 | Local |
| F4 | 09/20/2011 0:00 | 15 | 49.762649 | -109.174211 | 631488 | 5513841 | 1 | 1634.33 | Local |
| F4 | 09/20/2011 0:00 | 18 | 49.748064 | -109.171420 | 631729 | 5512225 | 1 | 1332.64 | Local |
| F4 | 09/20/2011 0:00 | 21 | 49.739266 | -109.183975 | 630848 | 5511224 | 1 | 877.55 | Local |
| F4 | 09/21/2011 0:00 | 0 | 49.736900 | -109.172358 | 631691 | 5510982 | 1 | 2949.75 | Local |
| F4 | 09/21/2011 0:00 | 3 | 49.739643 | -109.131649 | 634617 | 5511359 | 1 | 1213.80 | Local |
| F4 | 09/21/2011 0:00 | 6 | 49.743462 | -109.115871 | 635743 | 5511812 | 0 | 1702.27 | Local |
| F4 | 09/21/2011 0:00 | 21 | 49.757197 | -109.105442 | 636456 | 5513358 | 1 | 428.66 | Local |
| F4 | 09/22/2011 0:00 | 0 | 49.760385 | -109.102097 | 636688 | 5513718 | 0 | 3783.07 | Local |
| F4 | 09/22/2011 0:00 | 9 | 49.784392 | -109.139313 | 633942 | 5516320 | 0 | 30.91 | Local |

| | | | | | | | | | |
|---|---|---|---|---|---|---|---|---|---|
| F4 | 09/22/2011 0:00 | 15 | 49.784660 | -109.139431 | 633932 | 5516349 | 1 | 6.01 | Local |
| F4 | 09/22/2011 0:00 | 18 | 49.784624 | -109.139369 | 633937 | 5516346 | 0 | 210.47 | Local |
| F4 | 09/23/2011 0:00 | 0 | 49.785819 | -109.137101 | 634097 | 5516482 | 1 | 40.90 | Local |
| F4 | 09/23/2011 0:00 | 3 | 49.786175 | -109.137243 | 634086 | 5516522 | 1 | 124.15 | Local |
| F4 | 09/23/2011 0:00 | 6 | 49.787249 | -109.136773 | 634116 | 5516642 | 1 | 96.81 | Local |
| F4 | 09/23/2011 0:00 | 9 | 49.786995 | -109.138059 | 634025 | 5516612 | 1 | 7.46 | Local |
| F4 | 09/23/2011 0:00 | 12 | 49.787056 | -109.138016 | 634028 | 5516618 | 1 | 3.90 | Local |
| F4 | 09/23/2011 0:00 | 15 | 49.787042 | -109.138066 | 634024 | 5516617 | 0 | 145.72 | Local |
| F4 | 09/24/2011 0:00 | 3 | 49.785853 | -109.137215 | 634089 | 5516486 | 0 | 24.95 | Local |
| F4 | 09/24/2011 0:00 | 9 | 49.786052 | -109.137375 | 634076 | 5516508 | 0 | 60.42 | Local |
| F4 | 09/24/2011 0:00 | 15 | 49.786579 | -109.137167 | 634090 | 5516567 | 1 | 75.84 | Local |
| F4 | 09/25/2011 0:00 | 18 | 49.785915 | -109.137411 | 634074 | 5516493 | 0 | 7.21 | Local |
| F4 | 09/26/2011 0:00 | 6 | 49.785979 | -109.137430 | 634073 | 5516500 | 1 | 20.33 | Local |
| F4 | 09/26/2011 0:00 | 9 | 49.785978 | -109.137148 | 634093 | 5516500 | 0 | 5911.98 | Local |
| F4 | 09/28/2011 0:00 | 0 | 49.752627 | -109.201070 | 629581 | 5512680 | 1 | 668.12 | Local |
| F4 | 09/28/2011 0:00 | 3 | 49.746750 | -109.202998 | 629458 | 5512023 | 1 | 1852.28 | Local |
| F4 | 09/28/2011 0:00 | 6 | 49.744789 | -109.228524 | 627624 | 5511762 | 1 | 1402.47 | Local |
| F4 | 09/28/2011 0:00 | 9 | 49.743738 | -109.247919 | 626230 | 5511612 | 1 | 21.61 | Local |
| F4 | 09/28/2011 0:00 | 12 | 49.743932 | -109.247913 | 626229 | 5511634 | 0 | 354.84 | Local |
| F4 | 09/28/2011 0:00 | 18 | 49.745569 | -109.252140 | 625921 | 5511809 | 1 | 522.21 | Local |
| F4 | 09/28/2011 0:00 | 21 | 49.744283 | -109.259109 | 625422 | 5511654 | 0 | 2354.87 | Local |
| F4 | 09/29/2011 0:00 | 3 | 49.757930 | -109.284102 | 623587 | 5513130 | 1 | 1316.78 | Local |
| F4 | 09/29/2011 0:00 | 6 | 49.764265 | -109.299545 | 622459 | 5513809 | 1 | 976.97 | Local |
| F4 | 09/29/2011 0:00 | 9 | 49.761944 | -109.286464 | 623406 | 5513572 | 1 | 57.93 | Local |
| F4 | 09/29/2011 0:00 | 12 | 49.762460 | -109.286348 | 623413 | 5513630 | 0 | 2739.39 | Local |
| F4 | 09/29/2011 0:00 | 21 | 49.779588 | -109.259011 | 625338 | 5515579 | 1 | 837.70 | Local |
| F4 | 09/30/2011 0:00 | 0 | 49.783008 | -109.248644 | 626075 | 5515977 | 0 | 3836.37 | Local |
| F4 | 09/30/2011 0:00 | 21 | 49.771184 | -109.198597 | 629709 | 5514747 | 1 | 608.91 | Local |
| F4 | 10/01/2011 0:00 | 0 | 49.768804 | -109.190983 | 630264 | 5514496 | 1 | 2896.00 | Local |
| F4 | 10/02/2011 0:00 | 3 | 49.789853 | -109.214666 | 628503 | 5516795 | 1 | 463.74 | Local |
| F4 | 10/02/2011 0:00 | 6 | 49.787744 | -109.209108 | 628909 | 5516570 | 0 | 3634.38 | Local |
| F4 | 10/04/2011 0:00 | 21 | 49.755074 | -109.207712 | 629096 | 5512941 | 0 | 2532.37 | Local |
| F4 | 10/05/2011 0:00 | 3 | 49.737120 | -109.229331 | 627586 | 5510908 | 1 | 2479.84 | Local |
| F4 | 10/05/2011 0:00 | 6 | 49.750042 | -109.257378 | 625532 | 5512297 | 1 | 927.20 | Local |
| F4 | 10/05/2011 0:00 | 9 | 49.758257 | -109.259584 | 625352 | 5513207 | 0 | 1006.10 | Local |
| F4 | 10/05/2011 0:00 | 21 | 49.755061 | -109.272649 | 624419 | 5512830 | 0 | 727.90 | Local |
| F4 | 10/06/2011 0:00 | 9 | 49.757239 | -109.282177 | 623727 | 5513056 | 1 | 13.84 | Local |
| F4 | 10/06/2011 0:00 | 12 | 49.757116 | -109.282201 | 623726 | 5513042 | 0 | 20.26 | Local |
| F4 | 10/06/2011 0:00 | 18 | 49.756960 | -109.282347 | 623716 | 5513025 | 0 | 22.79 | Local |
| F4 | 10/07/2011 0:00 | 18 | 49.756955 | -109.282031 | 623738 | 5513025 | 0 | 111.57 | Local |
| F4 | 10/08/2011 0:00 | 3 | 49.757946 | -109.282275 | 623718 | 5513134 | 0 | 37.96 | Local |
| F4 | 10/08/2011 0:00 | 9 | 49.757611 | -109.282378 | 623712 | 5513097 | 0 | 2036.49 | Local |
| F4 | 10/09/2011 0:00 | 3 | 49.768792 | -109.259986 | 625296 | 5514377 | 1 | 815.41 | Local |
| F4 | 10/10/2011 0:00 | 6 | 49.764532 | -109.269200 | 624643 | 5513888 | 0 | 3177.76 | Local |
| F4 | 10/10/2011 0:00 | 21 | 49.748254 | -109.232946 | 627296 | 5512139 | 0 | 2185.42 | Local |
| F4 | 10/11/2011 0:00 | 6 | 49.750141 | -109.263137 | 625117 | 5512298 | 1 | 1082.16 | Local |
| F4 | 10/11/2011 0:00 | 9 | 49.759822 | -109.261606 | 625202 | 5513377 | 0 | 11.26 | Local |
| F4 | 10/11/2011 0:00 | 18 | 49.759921 | -109.261572 | 625204 | 5513388 | 0 | 510.20 | Local |
| F4 | 10/13/2011 0:00 | 0 | 49.764348 | -109.263434 | 625059 | 5513877 | 0 | 591.63 | Local |
| F4 | 10/13/2011 0:00 | 12 | 49.769620 | -109.264540 | 624966 | 5514462 | 0 | 2411.12 | Local |
| F4 | 10/14/2011 0:00 | 3 | 49.765972 | -109.297539 | 622599 | 5514002 | 0 | 2330.31 | Local |
| F4 | 10/15/2011 0:00 | 15 | 49.768585 | -109.265438 | 624904 | 5514345 | 0 | 514.55 | Local |
| F4 | 10/16/2011 0:00 | 3 | 49.764515 | -109.262037 | 625159 | 5513898 | 1 | 579.80 | Local |
| F4 | 10/16/2011 0:00 | 6 | 49.769475 | -109.264518 | 624968 | 5514446 | 0 | 219.84 | Local |
| F4 | 10/16/2011 0:00 | 18 | 49.767923 | -109.266407 | 624835 | 5514270 | 1 | 1001.57 | Local |

| | | | | | | | | | |
|---|---|---|---|---|---|---|---|---|---|
| F4 | 10/16/2011 0:00 | 21 | 49.759493 | -109.261510 | 625210 | 5513341 | 1 | 32.94 | Local |
| F4 | 10/17/2011 0:00 | 0 | 49.759780 | -109.261624 | 625201 | 5513373 | 1 | 26.79 | Local |
| F4 | 10/17/2011 0:00 | 3 | 49.759837 | -109.261262 | 625227 | 5513379 | 0 | 973.99 | Local |
| F4 | 10/17/2011 0:00 | 9 | 49.752685 | -109.253458 | 625807 | 5512597 | 0 | 235.92 | Local |
| F4 | 10/18/2011 0:00 | 3 | 49.754680 | -109.254570 | 625722 | 5512817 | 1 | 8.00 | Local |
| F4 | 10/18/2011 0:00 | 6 | 49.754670 | -109.254460 | 625730 | 5512816 | 1 | 857.18 | Local |
| F4 | 10/18/2011 0:00 | 9 | 49.758120 | -109.243820 | 626487 | 5513218 | 1 | 11.92 | Local |
| F4 | 10/18/2011 0:00 | 12 | 49.758030 | -109.243910 | 626481 | 5513208 | 1 | 34.50 | Local |
| F4 | 10/18/2011 0:00 | 15 | 49.757720 | -109.243930 | 626480 | 5513173 | 1 | 424.60 | Local |
| F4 | 10/18/2011 0:00 | 18 | 49.756220 | -109.238510 | 626875 | 5513016 | 1 | 1834.48 | Local |
| F4 | 10/18/2011 0:00 | 21 | 49.772570 | -109.241900 | 626588 | 5514828 | 1 | 1792.23 | Local |
| F4 | 10/19/2011 0:00 | 0 | 49.762240 | -109.261000 | 625239 | 5513647 | 1 | 276.11 | Local |
| F4 | 10/19/2011 0:00 | 3 | 49.759800 | -109.261710 | 625195 | 5513375 | 1 | 6.20 | Local |
| F4 | 10/19/2011 0:00 | 6 | 49.759840 | -109.261650 | 625199 | 5513379 | 1 | 572.79 | Local |
| F4 | 10/19/2011 0:00 | 9 | 49.759750 | -109.253700 | 625772 | 5513382 | 1 | 2.43 | Local |
| F4 | 10/19/2011 0:00 | 12 | 49.759760 | -109.253730 | 625769 | 5513384 | 1 | 5.61 | Local |
| F4 | 10/19/2011 0:00 | 15 | 49.759710 | -109.253740 | 625769 | 5513378 | 1 | 376.86 | Local |
| F4 | 10/19/2011 0:00 | 18 | 49.762880 | -109.255590 | 625627 | 5513727 | 1 | 757.21 | Local |
| F4 | 10/19/2011 0:00 | 21 | 49.761260 | -109.265800 | 624896 | 5513530 | 1 | 339.56 | Local |
| F4 | 10/20/2011 0:00 | 0 | 49.759800 | -109.261660 | 625198 | 5513375 | 1 | 2.34 | Local |
| F4 | 10/20/2011 0:00 | 3 | 49.759820 | -109.261650 | 625199 | 5513377 | 1 | 4.91 | Local |
| F4 | 10/20/2011 0:00 | 6 | 49.759850 | -109.261600 | 625202 | 5513380 | 1 | 1388.78 | Local |
| F4 | 10/20/2011 0:00 | 9 | 49.754740 | -109.244010 | 626482 | 5512842 | 0 | 7.86 | Local |
| F4 | 10/20/2011 0:00 | 15 | 49.754700 | -109.243920 | 626489 | 5512837 | 1 | 961.27 | Local |
| F4 | 10/20/2011 0:00 | 18 | 49.753580 | -109.230690 | 627445 | 5512735 | 1 | 1609.51 | Local |
| F4 | 10/20/2011 0:00 | 21 | 49.761420 | -109.211910 | 628777 | 5513639 | 1 | 519.59 | Local |
| F4 | 10/21/2011 0:00 | 0 | 49.762960 | -109.205100 | 629263 | 5513822 | 1 | 1167.45 | Local |
| F4 | 10/21/2011 0:00 | 3 | 49.773040 | -109.209630 | 628910 | 5514935 | 1 | 1264.55 | Local |
| F4 | 10/21/2011 0:00 | 6 | 49.782730 | -109.218820 | 628223 | 5515996 | 1 | 1252.89 | Local |
| F4 | 10/21/2011 0:00 | 9 | 49.775760 | -109.205150 | 629225 | 5515245 | 0 | 294.83 | Local |
| F4 | 10/21/2011 0:00 | 15 | 49.773520 | -109.207340 | 629074 | 5514992 | 1 | 790.91 | Local |
| F4 | 10/21/2011 0:00 | 18 | 49.766430 | -109.206470 | 629155 | 5514205 | 1 | 2019.52 | Local |
| F4 | 10/21/2011 0:00 | 21 | 49.782760 | -109.218740 | 628229 | 5516000 | 1 | 12.52 | Local |
| F4 | 10/22/2011 0:00 | 0 | 49.782660 | -109.218820 | 628223 | 5515988 | 1 | 10.41 | Local |
| F4 | 10/22/2011 0:00 | 3 | 49.782750 | -109.218780 | 628226 | 5515999 | 1 | 9.43 | Local |
| F4 | 10/22/2011 0:00 | 6 | 49.782740 | -109.218910 | 628216 | 5515997 | 0 | 1653.19 | Local |
| F4 | 10/22/2011 0:00 | 15 | 49.768820 | -109.210850 | 628834 | 5514463 | 1 | 868.42 | Local |
| F4 | 10/22/2011 0:00 | 18 | 49.763380 | -109.202200 | 629471 | 5513874 | 0 | 3255.63 | Local |
| F4 | 10/23/2011 0:00 | 0 | 49.737790 | -109.224150 | 627957 | 5510991 | 1 | 594.79 | Local |
| F4 | 10/23/2011 0:00 | 3 | 49.741010 | -109.230740 | 627474 | 5511338 | 1 | 15.13 | Local |
| F4 | 10/23/2011 0:00 | 6 | 49.740940 | -109.230920 | 627461 | 5511330 | 1 | 14.09 | Local |
| F4 | 10/23/2011 0:00 | 9 | 49.740970 | -109.230730 | 627475 | 5511333 | 1 | 148.87 | Local |
| F4 | 10/23/2011 0:00 | 12 | 49.741480 | -109.232640 | 627336 | 5511387 | 1 | 9.01 | Local |
| F4 | 10/23/2011 0:00 | 15 | 49.741560 | -109.232660 | 627334 | 5511396 | 1 | 159.83 | Local |
| F4 | 10/23/2011 0:00 | 18 | 49.742970 | -109.232230 | 627362 | 5511553 | 1 | 2462.09 | Local |
| F4 | 10/23/2011 0:00 | 21 | 49.764200 | -109.222530 | 628005 | 5513930 | 1 | 2109.20 | Local |
| F4 | 10/24/2011 0:00 | 0 | 49.783000 | -109.218650 | 628234 | 5516027 | 1 | 28.25 | Local |
| F4 | 10/24/2011 0:00 | 3 | 49.782750 | -109.218720 | 628230 | 5515999 | 1 | 209.96 | Local |
| F4 | 10/24/2011 0:00 | 6 | 49.783340 | -109.221490 | 628029 | 5516059 | 1 | 1616.35 | Local |
| F4 | 10/24/2011 0:00 | 9 | 49.783930 | -109.243920 | 626413 | 5516087 | 0 | 10.11 | Local |
| F4 | 10/24/2011 0:00 | 15 | 49.783840 | -109.243940 | 626412 | 5516077 | 1 | 900.09 | Local |
| F4 | 10/24/2011 0:00 | 18 | 49.780990 | -109.255640 | 625577 | 5515740 | 1 | 1970.53 | Local |
| F4 | 10/24/2011 0:00 | 21 | 49.771320 | -109.232710 | 627253 | 5514704 | 1 | 155.26 | Local |
| F4 | 10/25/2011 0:00 | 0 | 49.769970 | -109.232160 | 627296 | 5514555 | 1 | 3.09 | Local |
| F4 | 10/25/2011 0:00 | 3 | 49.769980 | -109.232120 | 627299 | 5514556 | 1 | 11.55 | Local |

| | | | | | | | | | |
|---|---|---|---|---|---|---|---|---|---|
| F4 | 10/25/2011 0:00 | 6 | 49.769890 | -109.232200 | 627293 | 5514546 | 1 | 18.04 | Local |
| F4 | 10/25/2011 0:00 | 9 | 49.769900 | -109.231950 | 627311 | 5514548 | 1 | 117.23 | Local |
| F4 | 10/25/2011 0:00 | 12 | 49.769100 | -109.230890 | 627390 | 5514460 | 1 | 5.61 | Local |
| F4 | 10/25/2011 0:00 | 15 | 49.769050 | -109.230880 | 627391 | 5514455 | 1 | 870.06 | Local |
| F4 | 10/25/2011 0:00 | 18 | 49.768730 | -109.218810 | 628261 | 5514440 | 1 | 971.61 | Local |
| F4 | 10/25/2011 0:00 | 21 | 49.770030 | -109.232150 | 627297 | 5514562 | 1 | 7.04 | Local |
| F4 | 10/26/2011 0:00 | 0 | 49.769980 | -109.232090 | 627301 | 5514556 | 1 | 7.54 | Local |
| F4 | 10/26/2011 0:00 | 3 | 49.769960 | -109.232190 | 627294 | 5514554 | 1 | 5.56 | Local |
| F4 | 10/26/2011 0:00 | 6 | 49.769910 | -109.232190 | 627294 | 5514548 | 1 | 1117.02 | Local |
| F4 | 10/26/2011 0:00 | 9 | 49.762670 | -109.221440 | 628087 | 5513762 | 0 | 587.89 | Local |
| F4 | 10/26/2011 0:00 | 15 | 49.758290 | -109.226010 | 627770 | 5513267 | 1 | 1279.12 | Local |
| F4 | 10/26/2011 0:00 | 18 | 49.747280 | -109.220870 | 628169 | 5512052 | 1 | 1008.15 | Local |
| F4 | 10/26/2011 0:00 | 21 | 49.741000 | -109.230960 | 627458 | 5511336 | 1 | 7.95 | Local |
| F4 | 10/27/2011 0:00 | 0 | 49.740940 | -109.230900 | 627463 | 5511330 | 1 | 0.72 | Local |
| F4 | 10/27/2011 0:00 | 3 | 49.740940 | -109.230910 | 627462 | 5511330 | 1 | 15.77 | Local |
| F4 | 10/27/2011 0:00 | 6 | 49.740980 | -109.230700 | 627477 | 5511335 | 1 | 618.40 | Local |
| F4 | 10/27/2011 0:00 | 9 | 49.746350 | -109.228470 | 627624 | 5511935 | 1 | 186.67 | Local |
| F4 | 10/27/2011 0:00 | 12 | 49.747660 | -109.230090 | 627504 | 5512078 | 1 | 90.96 | Local |
| F4 | 10/27/2011 0:00 | 15 | 49.746880 | -109.229710 | 627533 | 5511992 | 1 | 669.04 | Local |
| F4 | 10/27/2011 0:00 | 18 | 49.747320 | -109.238970 | 626865 | 5512025 | 1 | 1339.23 | Local |
| F4 | 10/27/2011 0:00 | 21 | 49.759180 | -109.235740 | 627067 | 5513349 | 1 | 1964.01 | Local |
| F4 | 10/28/2011 0:00 | 0 | 49.776400 | -109.241800 | 626585 | 5515253 | 1 | 998.66 | Local |
| F4 | 10/28/2011 0:00 | 3 | 49.779310 | -109.254920 | 625633 | 5515555 | 1 | 18.15 | Local |
| F4 | 10/28/2011 0:00 | 6 | 49.779470 | -109.254870 | 625636 | 5515573 | 1 | 1601.59 | Local |
| F4 | 10/28/2011 0:00 | 9 | 49.773630 | -109.234540 | 627115 | 5514958 | 1 | 846.67 | Local |
| F4 | 10/28/2011 0:00 | 12 | 49.766270 | -109.237550 | 626918 | 5514134 | 1 | 58.15 | Local |
| F4 | 10/28/2011 0:00 | 15 | 49.766200 | -109.236750 | 626975 | 5514128 | 1 | 2573.17 | Local |
| F4 | 10/28/2011 0:00 | 18 | 49.748140 | -109.214420 | 628631 | 5512158 | 1 | 1629.85 | Local |
| F4 | 10/28/2011 0:00 | 21 | 49.738320 | -109.231210 | 627447 | 5511038 | 1 | 2189.08 | Local |
| F4 | 10/29/2011 0:00 | 0 | 49.743220 | -109.260630 | 625315 | 5511533 | 1 | 314.30 | Local |
| F4 | 10/29/2011 0:00 | 3 | 49.745920 | -109.261920 | 625215 | 5511831 | 1 | 1740.56 | Local |
| F4 | 10/29/2011 0:00 | 6 | 49.761550 | -109.263210 | 625082 | 5513567 | 1 | 1380.80 | Local |
| F4 | 10/29/2011 0:00 | 9 | 49.772100 | -109.253100 | 625783 | 5514756 | 1 | 175.41 | Local |
| F4 | 10/29/2011 0:00 | 12 | 49.772490 | -109.250740 | 625952 | 5514804 | 1 | 4.45 | Local |
| F4 | 10/29/2011 0:00 | 15 | 49.772450 | -109.250740 | 625952 | 5514799 | 1 | 656.36 | Local |
| F4 | 10/29/2011 0:00 | 18 | 49.773090 | -109.241680 | 626602 | 5514886 | 1 | 2655.70 | Local |
| F4 | 10/29/2011 0:00 | 21 | 49.795650 | -109.229580 | 627414 | 5517414 | 1 | 2576.74 | Local |
| F4 | 10/30/2011 0:00 | 0 | 49.779050 | -109.204610 | 629256 | 5515611 | 1 | 2247.19 | Local |
| F4 | 10/30/2011 0:00 | 3 | 49.758850 | -109.205490 | 629246 | 5513364 | 1 | 16.54 | Local |
| F4 | 10/30/2011 0:00 | 6 | 49.758750 | -109.205320 | 629258 | 5513353 | 1 | 534.18 | Local |
| F4 | 10/30/2011 0:00 | 9 | 49.754310 | -109.208150 | 629066 | 5512855 | 1 | 1118.84 | Local |
| F4 | 10/30/2011 0:00 | 12 | 49.764080 | -109.211860 | 628773 | 5513935 | 1 | 59.23 | Local |
| F4 | 10/30/2011 0:00 | 15 | 49.764530 | -109.212300 | 628740 | 5513984 | 1 | 1021.87 | Local |
| F4 | 10/30/2011 0:00 | 18 | 49.773090 | -109.217460 | 628346 | 5514927 | 1 | 2560.70 | Local |
| F4 | 10/30/2011 0:00 | 21 | 49.795830 | -109.223060 | 627883 | 5517445 | 1 | 965.76 | Local |
| F4 | 10/31/2011 0:00 | 0 | 49.793710 | -109.210050 | 628825 | 5517232 | 1 | 1951.61 | Local |
| F4 | 10/31/2011 0:00 | 3 | 49.793790 | -109.237160 | 626874 | 5517195 | 1 | 903.67 | Local |
| F4 | 10/31/2011 0:00 | 6 | 49.789230 | -109.247550 | 626138 | 5516670 | 1 | 13.01 | Local |
| F4 | 10/31/2011 0:00 | 9 | 49.789220 | -109.247730 | 626125 | 5516669 | 1 | 13.31 | Local |
| F4 | 10/31/2011 0:00 | 12 | 49.789150 | -109.247580 | 626136 | 5516661 | 1 | 10.01 | Local |
| F4 | 10/31/2011 0:00 | 15 | 49.789240 | -109.247580 | 626136 | 5516671 | 1 | 4.45 | Local |
| F4 | 10/31/2011 0:00 | 18 | 49.789200 | -109.247580 | 626136 | 5516667 | 1 | 2.65 | Local |
| F4 | 10/31/2011 0:00 | 21 | 49.789220 | -109.247600 | 626134 | 5516669 | 1 | 7.58 | Local |
| F4 | 11/01/2011 0:00 | 0 | 49.789160 | -109.247650 | 626131 | 5516662 | 0 | 7.04 | Local |
| F4 | 11/01/2011 0:00 | 6 | 49.789210 | -109.247590 | 626135 | 5516668 | 1 | 33.37 | Local |

| | | | | | | | | | |
|---|---|---|---|---|---|---|---|---|---|
| F4 | 11/01/2011 0:00 | 9 | 49.789070 | -109.248000 | 626106 | 5516652 | 1 | 37.08 | Local |
| F4 | 11/01/2011 0:00 | 12 | 49.789280 | -109.247600 | 626134 | 5516676 | 1 | 37.40 | Local |
| F4 | 11/01/2011 0:00 | 15 | 49.788950 | -109.247500 | 626142 | 5516639 | 1 | 46.04 | Local |
| F4 | 11/01/2011 0:00 | 18 | 49.789150 | -109.248060 | 626101 | 5516660 | 0 | 33.91 | Local |
| F4 | 11/02/2011 0:00 | 0 | 49.789240 | -109.247610 | 626134 | 5516671 | 1 | 5.30 | Local |
| F4 | 11/02/2011 0:00 | 3 | 49.789200 | -109.247650 | 626131 | 5516667 | 1 | 28.51 | Local |
| F4 | 11/02/2011 0:00 | 6 | 49.789080 | -109.248000 | 626106 | 5516653 | 1 | 29.68 | Local |
| F4 | 11/02/2011 0:00 | 9 | 49.789210 | -109.247640 | 626132 | 5516668 | 1 | 4.86 | Local |
| F4 | 11/02/2011 0:00 | 12 | 49.789190 | -109.247580 | 626136 | 5516666 | 1 | 32.87 | Local |
| F4 | 11/02/2011 0:00 | 15 | 49.789060 | -109.247990 | 626107 | 5516650 | 1 | 23.02 | Local |
| F4 | 11/02/2011 0:00 | 18 | 49.789160 | -109.247710 | 626127 | 5516662 | 1 | 1018.42 | Local |
| F4 | 11/02/2011 0:00 | 21 | 49.782470 | -109.238050 | 626839 | 5515935 | 1 | 1036.85 | Local |
| F4 | 11/03/2011 0:00 | 0 | 49.789110 | -109.248160 | 626094 | 5516656 | 1 | 12.79 | Local |
| F4 | 11/03/2011 0:00 | 3 | 49.789060 | -109.248000 | 626106 | 5516650 | 1 | 4.94 | Local |
| F4 | 11/03/2011 0:00 | 6 | 49.789100 | -109.248030 | 626104 | 5516655 | 1 | 33.90 | Local |
| F4 | 11/03/2011 0:00 | 9 | 49.789250 | -109.247620 | 626133 | 5516672 | 1 | 15.09 | Local |
| F4 | 11/03/2011 0:00 | 12 | 49.789120 | -109.247680 | 626129 | 5516658 | 1 | 5.61 | Local |
| F4 | 11/03/2011 0:00 | 15 | 49.789070 | -109.247670 | 626130 | 5516652 | 1 | 26.22 | Local |
| F4 | 11/03/2011 0:00 | 18 | 49.789300 | -109.247750 | 626123 | 5516678 | 1 | 25.61 | Local |
| F4 | 11/03/2011 0:00 | 21 | 49.789150 | -109.248020 | 626104 | 5516660 | 1 | 27.64 | Local |
| F4 | 11/04/2011 0:00 | 0 | 49.789320 | -109.247740 | 626124 | 5516680 | 1 | 14.03 | Local |
| F4 | 11/04/2011 0:00 | 3 | 49.789440 | -109.247680 | 626128 | 5516693 | 1 | 20.34 | Local |
| F4 | 11/04/2011 0:00 | 6 | 49.789260 | -109.247730 | 626125 | 5516673 | 1 | 633.51 | Local |
| F4 | 11/04/2011 0:00 | 9 | 49.784380 | -109.243190 | 626464 | 5516138 | 0 | 502.48 | Local |
| F4 | 11/04/2011 0:00 | 15 | 49.780130 | -109.240820 | 626646 | 5515670 | 1 | 1099.16 | Local |
| F4 | 11/04/2011 0:00 | 18 | 49.770270 | -109.239750 | 626749 | 5514575 | 1 | 2189.42 | Local |
| F4 | 11/04/2011 0:00 | 21 | 49.789310 | -109.247490 | 626142 | 5516679 | 1 | 3045.94 | Local |
| F4 | 11/05/2011 0:00 | 0 | 49.779060 | -109.208260 | 628993 | 5515606 | 1 | 847.02 | Local |
| F4 | 11/05/2011 0:00 | 3 | 49.771450 | -109.207760 | 629049 | 5514761 | 1 | 670.55 | Local |
| F4 | 11/05/2011 0:00 | 6 | 49.769500 | -109.216570 | 628420 | 5514529 | 1 | 2210.12 | Local |
| F4 | 11/05/2011 0:00 | 9 | 49.752440 | -109.232310 | 627331 | 5512606 | 1 | 860.88 | Local |
| F4 | 11/05/2011 0:00 | 12 | 49.754780 | -109.243700 | 626505 | 5512847 | 1 | 9.72 | Local |
| F4 | 11/05/2011 0:00 | 15 | 49.754820 | -109.243820 | 626496 | 5512851 | 1 | 1309.71 | Local |
| F4 | 11/05/2011 0:00 | 18 | 49.766050 | -109.238340 | 626861 | 5514109 | 1 | 2248.42 | Local |
| F4 | 11/05/2011 0:00 | 21 | 49.774200 | -109.266910 | 624783 | 5514967 | 1 | 2204.32 | Local |
| F4 | 11/06/2011 0:00 | 0 | 49.757350 | -109.283030 | 623665 | 5513067 | 1 | 9.35 | Local |
| F4 | 11/06/2011 0:00 | 3 | 49.757270 | -109.282990 | 623668 | 5513058 | 1 | 9.15 | Local |
| F4 | 11/06/2011 0:00 | 6 | 49.757350 | -109.283020 | 623666 | 5513067 | 1 | 771.96 | Local |
| F4 | 11/06/2011 0:00 | 9 | 49.750480 | -109.281480 | 623795 | 5512306 | 1 | 22.90 | Local |
| F4 | 11/06/2011 0:00 | 12 | 49.750320 | -109.281280 | 623809 | 5512288 | 1 | 782.02 | Local |
| F4 | 11/06/2011 0:00 | 15 | 49.748900 | -109.291910 | 623047 | 5512113 | 1 | 1931.47 | Local |
| F4 | 11/06/2011 0:00 | 18 | 49.761900 | -109.274130 | 624295 | 5513587 | 1 | 1995.65 | Local |
| F4 | 11/06/2011 0:00 | 21 | 49.746140 | -109.260880 | 625290 | 5511857 | 1 | 2724.00 | Local |
| F4 | 11/07/2011 0:00 | 0 | 49.735110 | -109.227130 | 627750 | 5510688 | 1 | 2385.09 | Local |
| F4 | 11/07/2011 0:00 | 3 | 49.754330 | -109.212440 | 628757 | 5512850 | 1 | 971.56 | Local |
| F4 | 11/07/2011 0:00 | 6 | 49.762350 | -109.207090 | 629121 | 5513751 | 1 | 358.28 | Local |
| F4 | 11/07/2011 0:00 | 9 | 49.765510 | -109.208060 | 629043 | 5514100 | 1 | 277.10 | Local |
| F4 | 11/07/2011 0:00 | 12 | 49.764900 | -109.204330 | 629313 | 5514039 | 1 | 580.85 | Local |
| F4 | 11/07/2011 0:00 | 15 | 49.759680 | -109.204040 | 629348 | 5513459 | 1 | 1434.61 | Local |
| F4 | 11/07/2011 0:00 | 18 | 49.769580 | -109.216810 | 628402 | 5514538 | 1 | 1503.68 | Local |
| F4 | 11/07/2011 0:00 | 21 | 49.783080 | -109.215620 | 628452 | 5516041 | 1 | 7.95 | Local |
| F4 | 11/08/2011 0:00 | 0 | 49.783140 | -109.215680 | 628448 | 5516047 | 1 | 14.20 | Local |
| F4 | 11/08/2011 0:00 | 3 | 49.783050 | -109.215820 | 628438 | 5516037 | 1 | 21.04 | Local |
| F4 | 11/08/2011 0:00 | 6 | 49.783230 | -109.215730 | 628444 | 5516057 | 1 | 588.86 | Local |
| F4 | 11/08/2011 0:00 | 9 | 49.778250 | -109.212950 | 628657 | 5515508 | 1 | 613.68 | Local |

| | | | | | | | | | |
|---|---|---|---|---|---|---|---|---|---|
| F4 | 11/08/2011 0:00 | 12 | 49.773330 | -109.209090 | 628948 | 5514968 | 1 | 27.24 | Local |
| F4 | 11/08/2011 0:00 | 15 | 49.773100 | -109.208960 | 628958 | 5514943 | 1 | 668.41 | Local |
| F4 | 11/08/2011 0:00 | 18 | 49.772260 | -109.218150 | 628299 | 5514833 | 1 | 1095.12 | Local |
| F4 | 11/08/2011 0:00 | 21 | 49.775680 | -109.232410 | 627263 | 5515189 | 1 | 34.95 | Local |
| F4 | 11/09/2011 0:00 | 0 | 49.775990 | -109.232490 | 627257 | 5515224 | 1 | 1842.23 | Local |
| F4 | 11/09/2011 0:00 | 3 | 49.789540 | -109.247210 | 626162 | 5516705 | 1 | 963.98 | Local |
| F4 | 11/09/2011 0:00 | 6 | 49.793280 | -109.259290 | 625282 | 5517101 | 1 | 1492.51 | Local |
| F4 | 11/09/2011 0:00 | 9 | 49.780320 | -109.253900 | 625704 | 5515669 | 1 | 11.12 | Local |
| F4 | 11/09/2011 0:00 | 12 | 49.780420 | -109.253900 | 625704 | 5515680 | 1 | 1.82 | Local |
| F4 | 11/09/2011 0:00 | 15 | 49.780410 | -109.253920 | 625702 | 5515679 | 1 | 280.93 | Local |
| F4 | 11/09/2011 0:00 | 18 | 49.778550 | -109.251280 | 625897 | 5515477 | 1 | 2972.30 | Local |
| F4 | 11/09/2011 0:00 | 21 | 49.785570 | -109.211450 | 628746 | 5516325 | 1 | 2106.27 | Local |
| F4 | 11/10/2011 0:00 | 0 | 49.790840 | -109.183350 | 630754 | 5516959 | 1 | 998.02 | Local |
| F4 | 11/10/2011 0:00 | 3 | 49.784250 | -109.192760 | 630095 | 5516210 | 1 | 7.28 | Local |
| F4 | 11/10/2011 0:00 | 6 | 49.784210 | -109.192840 | 630089 | 5516205 | 1 | 1.44 | Local |
| F4 | 11/10/2011 0:00 | 9 | 49.784210 | -109.192820 | 630091 | 5516206 | 0 | 1.32 | Local |
| F4 | 11/10/2011 0:00 | 15 | 49.784220 | -109.192810 | 630091 | 5516207 | 1 | 3.34 | Local |
| F4 | 11/10/2011 0:00 | 18 | 49.784250 | -109.192810 | 630091 | 5516210 | 1 | 25.13 | Local |
| F4 | 11/10/2011 0:00 | 21 | 49.784080 | -109.193040 | 630075 | 5516191 | 1 | 24.35 | Local |
| F4 | 11/11/2011 0:00 | 0 | 49.784220 | -109.192780 | 630093 | 5516207 | 1 | 559.72 | Local |
| F4 | 11/11/2011 0:00 | 3 | 49.781150 | -109.198940 | 629658 | 5515855 | 1 | 1127.97 | Local |
| F4 | 11/11/2011 0:00 | 6 | 49.771010 | -109.199340 | 629656 | 5514727 | 1 | 759.82 | Local |
| F4 | 11/11/2011 0:00 | 9 | 49.769290 | -109.209550 | 628926 | 5514518 | 1 | 9.15 | Local |
| F4 | 11/11/2011 0:00 | 12 | 49.769370 | -109.209580 | 628923 | 5514527 | 1 | 188.38 | Local |
| F4 | 11/11/2011 0:00 | 15 | 49.768540 | -109.211860 | 628762 | 5514431 | 1 | 151.01 | Local |
| F4 | 11/11/2011 0:00 | 18 | 49.767200 | -109.211520 | 628790 | 5514282 | 1 | 1879.52 | Local |
| F4 | 11/11/2011 0:00 | 21 | 49.768980 | -109.237470 | 626916 | 5514436 | 1 | 742.07 | Local |
| F4 | 11/12/2011 0:00 | 0 | 49.771060 | -109.247260 | 626206 | 5514651 | 1 | 1161.03 | Local |
| F4 | 11/12/2011 0:00 | 3 | 49.778570 | -109.258460 | 625380 | 5515467 | 1 | 2108.38 | Local |
| F4 | 11/12/2011 0:00 | 6 | 49.764510 | -109.238820 | 626831 | 5513937 | 1 | 768.55 | Local |
| F4 | 11/12/2011 0:00 | 9 | 49.758990 | -109.232400 | 627308 | 5513334 | 1 | 7.29 | Local |
| F4 | 11/12/2011 0:00 | 12 | 49.759020 | -109.232490 | 627301 | 5513337 | 1 | 148.70 | Local |
| F4 | 11/12/2011 0:00 | 15 | 49.757700 | -109.232820 | 627281 | 5513190 | 1 | 93.71 | Local |
| F4 | 11/12/2011 0:00 | 18 | 49.757850 | -109.231540 | 627372 | 5513209 | 1 | 2700.15 | Local |
| F4 | 11/12/2011 0:00 | 21 | 49.781990 | -109.235580 | 627018 | 5515885 | 1 | 1286.29 | Local |
| F4 | 11/13/2011 0:00 | 0 | 49.792440 | -109.227920 | 627542 | 5517060 | 1 | 1871.53 | Local |
| F4 | 11/13/2011 0:00 | 3 | 49.782990 | -109.206410 | 629116 | 5516046 | 1 | 2538.90 | Local |
| F4 | 11/13/2011 0:00 | 6 | 49.783560 | -109.241660 | 626577 | 5516050 | 1 | 288.72 | Local |
| F4 | 11/13/2011 0:00 | 9 | 49.780990 | -109.241090 | 626624 | 5515765 | 1 | 6.67 | Local |
| F4 | 11/13/2011 0:00 | 12 | 49.781050 | -109.241090 | 626624 | 5515772 | 1 | 11.91 | Local |
| F4 | 11/13/2011 0:00 | 15 | 49.780970 | -109.240980 | 626632 | 5515763 | 1 | 501.38 | Local |
| F4 | 11/13/2011 0:00 | 18 | 49.777620 | -109.236320 | 626977 | 5515398 | 1 | 2042.30 | Local |
| F4 | 11/13/2011 0:00 | 21 | 49.795480 | -109.229710 | 627406 | 5517395 | 1 | 1652.49 | Local |
| F4 | 11/14/2011 0:00 | 0 | 49.785910 | -109.212150 | 628695 | 5516361 | 1 | 2427.10 | Local |
| F4 | 11/14/2011 0:00 | 3 | 49.764480 | -109.205760 | 629212 | 5513990 | 1 | 176.20 | Local |
| F4 | 11/14/2011 0:00 | 6 | 49.765470 | -109.207670 | 629071 | 5514097 | 1 | 5.16 | Local |
| F4 | 11/14/2011 0:00 | 9 | 49.765480 | -109.207740 | 629066 | 5514098 | 1 | 17.22 | Local |
| F4 | 11/14/2011 0:00 | 12 | 49.765610 | -109.207610 | 629075 | 5514112 | 1 | 8.08 | Local |
| F4 | 11/14/2011 0:00 | 15 | 49.765540 | -109.207640 | 629073 | 5514104 | 1 | 1750.11 | Local |
| F4 | 11/14/2011 0:00 | 18 | 49.764800 | -109.231910 | 627328 | 5513981 | 1 | 3863.85 | Local |
| F4 | 11/14/2011 0:00 | 21 | 49.792570 | -109.264160 | 624934 | 5517014 | 1 | 649.74 | Local |
| F4 | 11/15/2011 0:00 | 0 | 49.787440 | -109.268480 | 624636 | 5516436 | 1 | 13.82 | Local |
| F4 | 11/15/2011 0:00 | 3 | 49.787560 | -109.268430 | 624639 | 5516450 | 1 | 1326.15 | Local |
| F4 | 11/15/2011 0:00 | 6 | 49.779640 | -109.254660 | 625651 | 5515592 | 0 | 997.05 | Local |
| F4 | 11/15/2011 0:00 | 15 | 49.780750 | -109.240920 | 626637 | 5515739 | 0 | 24.13 | Local |

| | | | | | | | | | |
|---|---|---|---|---|---|---|---|---|---|
| F4 | 11/15/2011 0:00 | 21 | 49.780950 | -109.241050 | 626627 | 5515761 | 1 | 11.57 | Local |
| F4 | 11/16/2011 0:00 | 0 | 49.780940 | -109.240890 | 626639 | 5515760 | 1 | 1603.81 | Local |
| F4 | 11/16/2011 0:00 | 3 | 49.767040 | -109.234950 | 627103 | 5514224 | 1 | 4.95 | Local |
| F4 | 11/16/2011 0:00 | 6 | 49.767080 | -109.234980 | 627101 | 5514229 | 1 | 2.65 | Local |
| F4 | 11/16/2011 0:00 | 9 | 49.767060 | -109.235000 | 627099 | 5514227 | 1 | 1393.20 | Local |
| F4 | 11/16/2011 0:00 | 12 | 49.755080 | -109.229340 | 627538 | 5512904 | 1 | 693.04 | Local |
| F4 | 11/16/2011 0:00 | 15 | 49.748850 | -109.229600 | 627536 | 5512211 | 1 | 1331.21 | Local |
| F4 | 11/16/2011 0:00 | 18 | 49.744650 | -109.212300 | 628793 | 5511774 | 1 | 644.13 | Local |
| F4 | 11/16/2011 0:00 | 21 | 49.749380 | -109.217460 | 628409 | 5512291 | 1 | 1165.31 | Local |
| F4 | 11/17/2011 0:00 | 0 | 49.758950 | -109.224050 | 627909 | 5513344 | 1 | 2800.14 | Local |
| F4 | 11/17/2011 0:00 | 3 | 49.782880 | -109.211950 | 628717 | 5516025 | 1 | 107.14 | Local |
| F4 | 11/17/2011 0:00 | 6 | 49.783770 | -109.211380 | 628756 | 5516125 | 1 | 11.01 | Local |
| F4 | 11/17/2011 0:00 | 9 | 49.783690 | -109.211470 | 628749 | 5516116 | 1 | 1684.21 | Local |
| F4 | 11/17/2011 0:00 | 12 | 49.776590 | -109.232130 | 627281 | 5515291 | 1 | 7.51 | Local |
| F4 | 11/17/2011 0:00 | 15 | 49.776540 | -109.232060 | 627286 | 5515286 | 1 | 7.58 | Local |
| F4 | 11/17/2011 0:00 | 18 | 49.776600 | -109.232110 | 627282 | 5515292 | 1 | 49.85 | Local |
| F4 | 11/17/2011 0:00 | 21 | 49.776190 | -109.232390 | 627263 | 5515246 | 1 | 2691.21 | Local |
| F4 | 11/18/2011 0:00 | 0 | 49.800360 | -109.230500 | 627336 | 5517936 | 1 | 1689.98 | Local |
| F4 | 11/18/2011 0:00 | 3 | 49.786810 | -109.219870 | 628137 | 5516448 | 0 | 1519.30 | Local |
| F4 | 11/18/2011 0:00 | 21 | 49.798450 | -109.230920 | 627311 | 5517723 | 1 | 150.37 | Local |
| F4 | 11/19/2011 0:00 | 0 | 49.799800 | -109.231040 | 627299 | 5517873 | 1 | 26.04 | Local |
| F4 | 11/19/2011 0:00 | 3 | 49.800010 | -109.230880 | 627309 | 5517897 | 1 | 15.72 | Local |
| F4 | 11/19/2011 0:00 | 6 | 49.799870 | -109.230910 | 627308 | 5517881 | 1 | 1490.88 | Local |
| F4 | 11/19/2011 0:00 | 9 | 49.787570 | -109.239150 | 626747 | 5516500 | 1 | 736.21 | Local |
| F4 | 11/19/2011 0:00 | 12 | 49.781060 | -109.241010 | 626630 | 5515773 | 0 | 2081.64 | Local |
| F4 | 11/19/2011 0:00 | 21 | 49.794940 | -109.221610 | 627990 | 5517349 | 1 | 2511.34 | Local |
| F4 | 11/20/2011 0:00 | 0 | 49.797980 | -109.187040 | 630470 | 5517746 | 1 | 1495.47 | Local |
| F4 | 11/20/2011 0:00 | 3 | 49.788530 | -109.201820 | 629431 | 5516670 | 1 | 277.00 | Local |
| F4 | 11/20/2011 0:00 | 6 | 49.789190 | -109.205530 | 629162 | 5516737 | 0 | 31.76 | Local |
| F4 | 11/20/2011 0:00 | 12 | 49.789170 | -109.205970 | 629131 | 5516734 | 0 | 13.68 | Local |
| F4 | 11/20/2011 0:00 | 18 | 49.789170 | -109.205780 | 629144 | 5516735 | 1 | 1216.29 | Local |
| F4 | 11/20/2011 0:00 | 21 | 49.797810 | -109.195420 | 629867 | 5517713 | 1 | 766.18 | Local |
| F4 | 11/21/2011 0:00 | 0 | 49.800080 | -109.185370 | 630584 | 5517983 | 1 | 9.42 | Local |
| F4 | 11/21/2011 0:00 | 3 | 49.800070 | -109.185240 | 630593 | 5517982 | 1 | 6.72 | Local |
| F4 | 11/21/2011 0:00 | 6 | 49.800110 | -109.185310 | 630588 | 5517986 | 1 | 6.20 | Local |
| F4 | 11/21/2011 0:00 | 9 | 49.800070 | -109.185370 | 630584 | 5517982 | 1 | 2098.33 | Local |
| F4 | 11/21/2011 0:00 | 12 | 49.790620 | -109.160140 | 632425 | 5516975 | 1 | 4.86 | Local |
| F4 | 11/21/2011 0:00 | 15 | 49.790640 | -109.160200 | 632421 | 5516977 | 1 | 12.09 | Local |
| F4 | 11/21/2011 0:00 | 18 | 49.790580 | -109.160060 | 632431 | 5516971 | 0 | 2639.89 | Local |
| F4 | 11/22/2011 0:00 | 0 | 49.797390 | -109.195190 | 629885 | 5517667 | 0 | 59.13 | Local |
| F4 | 11/22/2011 0:00 | 6 | 49.797020 | -109.195780 | 629843 | 5517624 | 1 | 1125.37 | Local |
| F4 | 11/22/2011 0:00 | 9 | 49.789220 | -109.205740 | 629147 | 5516740 | 1 | 4.46 | Local |
| F4 | 11/22/2011 0:00 | 12 | 49.789230 | -109.205800 | 629143 | 5516741 | 0 | 11.49 | Local |
| F4 | 11/22/2011 0:00 | 18 | 49.789130 | -109.205840 | 629140 | 5516730 | 1 | 12.23 | Local |
| F4 | 11/22/2011 0:00 | 21 | 49.789240 | -109.205840 | 629140 | 5516742 | 1 | 961.72 | Local |
| F4 | 11/23/2011 0:00 | 0 | 49.796810 | -109.212300 | 628655 | 5517573 | 1 | 14.47 | Local |
| F4 | 11/23/2011 0:00 | 3 | 49.796940 | -109.212310 | 628654 | 5517587 | 1 | 14.03 | Local |
| F4 | 11/23/2011 0:00 | 6 | 49.797060 | -109.212370 | 628649 | 5517600 | 1 | 19.52 | Local |
| F4 | 11/23/2011 0:00 | 9 | 49.797010 | -109.212110 | 628668 | 5517595 | 1 | 6.85 | Local |
| F4 | 11/23/2011 0:00 | 12 | 49.797030 | -109.212200 | 628662 | 5517597 | 1 | 7.28 | Local |
| F4 | 11/23/2011 0:00 | 15 | 49.797020 | -109.212300 | 628654 | 5517596 | 0 | 14.19 | Local |
| F4 | 11/23/2011 0:00 | 21 | 49.796910 | -109.212200 | 628662 | 5517584 | 1 | 24.06 | Local |
| F4 | 11/24/2011 0:00 | 0 | 49.797100 | -109.212360 | 628650 | 5517605 | 1 | 7.53 | Local |
| F4 | 11/24/2011 0:00 | 3 | 49.797080 | -109.212260 | 628657 | 5517603 | 0 | 15.63 | Local |
| F4 | 11/24/2011 0:00 | 9 | 49.796940 | -109.212280 | 628656 | 5517587 | 1 | 1357.17 | Local |

| | | | | | | | | | |
|---|---|---|---|---|---|---|---|---|---|
| F4 | 11/24/2011 0:00 | 12 | 49.787950 | -109.199530 | 629598 | 5516610 | 1 | 157.88 | Local |
| F4 | 11/24/2011 0:00 | 15 | 49.787640 | -109.197390 | 629752 | 5516579 | 1 | 486.98 | Local |
| F4 | 11/24/2011 0:00 | 18 | 49.783690 | -109.194470 | 629973 | 5516145 | 1 | 1948.71 | Local |
| F4 | 11/24/2011 0:00 | 21 | 49.796470 | -109.212990 | 628606 | 5517534 | 1 | 74.08 | Local |
| F4 | 11/25/2011 0:00 | 0 | 49.796970 | -109.212310 | 628654 | 5517590 | 1 | 12.23 | Local |
| F4 | 11/25/2011 0:00 | 3 | 49.797080 | -109.212310 | 628653 | 5517603 | 1 | 4.68 | Local |
| F4 | 11/25/2011 0:00 | 6 | 49.797040 | -109.212290 | 628655 | 5517598 | 1 | 1960.71 | Local |
| F4 | 11/25/2011 0:00 | 9 | 49.780120 | -109.204630 | 629251 | 5515730 | 1 | 328.43 | Local |
| F4 | 11/25/2011 0:00 | 12 | 49.777230 | -109.203690 | 629327 | 5515411 | 1 | 25.05 | Local |
| F4 | 11/25/2011 0:00 | 15 | 49.777430 | -109.203850 | 629315 | 5515433 | 1 | 1202.32 | Local |
| F4 | 11/25/2011 0:00 | 18 | 49.766740 | -109.206350 | 629163 | 5514240 | 1 | 702.88 | Local |
| F4 | 11/25/2011 0:00 | 21 | 49.772620 | -109.209930 | 628890 | 5514888 | 1 | 2744.11 | Local |
| F4 | 11/26/2011 0:00 | 0 | 49.797240 | -109.212510 | 628639 | 5517620 | 1 | 402.84 | Local |
| F4 | 11/26/2011 0:00 | 3 | 49.793670 | -109.213460 | 628580 | 5517222 | 1 | 3.97 | Local |
| F4 | 11/26/2011 0:00 | 6 | 49.793700 | -109.213430 | 628582 | 5517225 | 1 | 1740.21 | Local |
| F4 | 11/26/2011 0:00 | 9 | 49.778100 | -109.215340 | 628486 | 5515487 | 1 | 686.70 | Local |
| F4 | 11/26/2011 0:00 | 12 | 49.773540 | -109.208910 | 628961 | 5514992 | 1 | 256.21 | Local |
| F4 | 11/26/2011 0:00 | 15 | 49.771460 | -109.207380 | 629076 | 5514763 | 1 | 905.47 | Local |
| F4 | 11/26/2011 0:00 | 18 | 49.763320 | -109.207070 | 629120 | 5513859 | 1 | 601.13 | Local |
| F4 | 11/26/2011 0:00 | 21 | 49.758590 | -109.203030 | 629424 | 5513340 | 1 | 1778.89 | Local |
| F4 | 11/27/2011 0:00 | 0 | 49.743140 | -109.209430 | 629004 | 5511611 | 1 | 2165.03 | Local |
| F4 | 11/27/2011 0:00 | 3 | 49.737800 | -109.238320 | 626936 | 5510968 | 1 | 1423.43 | Local |
| F4 | 11/27/2011 0:00 | 6 | 49.743610 | -109.255920 | 625653 | 5511584 | 1 | 888.22 | Local |
| F4 | 11/27/2011 0:00 | 9 | 49.748360 | -109.246010 | 626355 | 5512129 | 1 | 24.63 | Local |
| F4 | 11/27/2011 0:00 | 12 | 49.748580 | -109.245970 | 626357 | 5512154 | 1 | 4.91 | Local |
| F4 | 11/27/2011 0:00 | 15 | 49.748550 | -109.245920 | 626361 | 5512150 | 1 | 570.43 | Local |
| F4 | 11/27/2011 0:00 | 18 | 49.748340 | -109.238010 | 626931 | 5512140 | 0 | 3390.82 | Local |
| F4 | 11/28/2011 0:00 | 0 | 49.740970 | -109.192350 | 630240 | 5511399 | 1 | 1149.27 | Local |
| F4 | 11/28/2011 0:00 | 3 | 49.751100 | -109.195510 | 629985 | 5512520 | 1 | 1845.20 | Local |
| F4 | 11/28/2011 0:00 | 6 | 49.748410 | -109.170240 | 631813 | 5512265 | 1 | 834.77 | Local |
| F4 | 11/28/2011 0:00 | 9 | 49.751410 | -109.159620 | 632570 | 5512617 | 1 | 619.02 | Local |
| F4 | 11/28/2011 0:00 | 12 | 49.755250 | -109.153400 | 633007 | 5513055 | 1 | 42.01 | Local |
| F4 | 11/28/2011 0:00 | 15 | 49.755570 | -109.153710 | 632984 | 5513090 | 1 | 10.24 | Local |
| F4 | 11/28/2011 0:00 | 18 | 49.755480 | -109.153680 | 632986 | 5513080 | 0 | 1861.41 | Local |
| F4 | 11/29/2011 0:00 | 0 | 49.763380 | -109.176460 | 631324 | 5513918 | 1 | 3071.63 | Local |
| F4 | 11/29/2011 0:00 | 3 | 49.783020 | -109.206450 | 629113 | 5516050 | 1 | 3844.96 | Local |
| F4 | 11/29/2011 0:00 | 6 | 49.787890 | -109.259320 | 625294 | 5516501 | 1 | 1358.26 | Local |
| F4 | 11/29/2011 0:00 | 9 | 49.778350 | -109.247540 | 626167 | 5515461 | 1 | 4.91 | Local |
| F4 | 11/29/2011 0:00 | 12 | 49.778380 | -109.247490 | 626170 | 5515464 | 1 | 30.22 | Local |
| F4 | 11/29/2011 0:00 | 15 | 49.778530 | -109.247840 | 626145 | 5515480 | 1 | 1512.06 | Local |
| F4 | 11/29/2011 0:00 | 18 | 49.765750 | -109.255010 | 625662 | 5514047 | 1 | 375.98 | Local |
| F4 | 11/29/2011 0:00 | 21 | 49.762560 | -109.253280 | 625795 | 5513696 | 0 | 1719.48 | Local |
| F4 | 11/30/2011 0:00 | 3 | 49.747110 | -109.252310 | 625904 | 5511980 | 1 | 3055.05 | Local |
| F4 | 11/30/2011 0:00 | 6 | 49.739180 | -109.211720 | 628849 | 5511167 | 1 | 1129.73 | Local |
| F4 | 11/30/2011 0:00 | 9 | 49.747020 | -109.201750 | 629547 | 5512056 | 1 | 814.30 | Local |
| F4 | 11/30/2011 0:00 | 12 | 49.745910 | -109.212920 | 628745 | 5511913 | 1 | 7.92 | Local |
| F4 | 11/30/2011 0:00 | 15 | 49.745980 | -109.212900 | 628746 | 5511921 | 1 | 17.90 | Local |
| F4 | 11/30/2011 0:00 | 18 | 49.745830 | -109.212990 | 628740 | 5511904 | 1 | 10.92 | Local |
| F4 | 11/30/2011 0:00 | 21 | 49.745890 | -109.213110 | 628731 | 5511910 | 1 | 20.45 | Local |
| F4 | 12/01/2011 0:00 | 0 | 49.745920 | -109.212830 | 628752 | 5511914 | 1 | 14.62 | Local |
| F4 | 12/01/2011 0:00 | 3 | 49.745790 | -109.212860 | 628750 | 5511900 | 1 | 1069.77 | Local |
| F4 | 12/01/2011 0:00 | 6 | 49.748860 | -109.198790 | 629755 | 5512265 | 1 | 3196.04 | Local |
| F4 | 12/01/2011 0:00 | 9 | 49.755130 | -109.155500 | 632856 | 5513038 | 0 | 479.62 | Local |
| F4 | 12/01/2011 0:00 | 18 | 49.759390 | -109.154460 | 632920 | 5513513 | 1 | 2791.21 | Local |
| F4 | 12/01/2011 0:00 | 21 | 49.779500 | -109.177650 | 631195 | 5515708 | 1 | 17.60 | Local |

| | | | | | | | | | |
|---|---|---|---|---|---|---|---|---|---|
| F4 | 12/02/2011 0:00 | 0 | 49.779470 | -109.177410 | 631213 | 5515705 | 1 | 0.72 | Local |
| F4 | 12/02/2011 0:00 | 3 | 49.779470 | -109.177420 | 631212 | 5515705 | 1 | 6.48 | Local |
| F4 | 12/02/2011 0:00 | 6 | 49.779470 | -109.177510 | 631205 | 5515705 | 1 | 4.46 | Local |
| F4 | 12/02/2011 0:00 | 9 | 49.779480 | -109.177450 | 631210 | 5515706 | 1 | 5.74 | Local |
| F4 | 12/02/2011 0:00 | 12 | 49.779430 | -109.177470 | 631208 | 5515701 | 1 | 6.72 | Local |
| F4 | 12/02/2011 0:00 | 15 | 49.779470 | -109.177400 | 631213 | 5515705 | 1 | 25.45 | Local |
| F4 | 12/02/2011 0:00 | 18 | 49.779360 | -109.177710 | 631191 | 5515693 | 0 | 2286.37 | Local |
| F4 | 12/03/2011 0:00 | 0 | 49.789130 | -109.205650 | 629154 | 5516730 | 1 | 11.73 | Local |
| F4 | 12/03/2011 0:00 | 3 | 49.789150 | -109.205810 | 629142 | 5516732 | 1 | 7.54 | Local |
| F4 | 12/03/2011 0:00 | 6 | 49.789130 | -109.205710 | 629150 | 5516730 | 1 | 1207.28 | Local |
| F4 | 12/03/2011 0:00 | 9 | 49.778390 | -109.203260 | 629355 | 5515540 | 1 | 1777.15 | Local |
| F4 | 12/03/2011 0:00 | 12 | 49.762930 | -109.209510 | 628946 | 5513811 | 1 | 4.32 | Local |
| F4 | 12/03/2011 0:00 | 15 | 49.762930 | -109.209570 | 628941 | 5513811 | 1 | 4.23 | Local |
| F4 | 12/03/2011 0:00 | 18 | 49.762950 | -109.209620 | 628938 | 5513813 | 1 | 514.44 | Local |
| F4 | 12/03/2011 0:00 | 21 | 49.761280 | -109.216280 | 628462 | 5513616 | 1 | 516.99 | Local |
| F4 | 12/04/2011 0:00 | 0 | 49.762980 | -109.209600 | 628939 | 5513816 | 1 | 13.03 | Local |
| F4 | 12/04/2011 0:00 | 3 | 49.763020 | -109.209770 | 628927 | 5513821 | 1 | 4.86 | Local |
| F4 | 12/04/2011 0:00 | 6 | 49.763000 | -109.209830 | 628922 | 5513818 | 1 | 15.44 | Local |
| F4 | 12/04/2011 0:00 | 9 | 49.762950 | -109.209630 | 628937 | 5513813 | 1 | 3.10 | Local |
| F4 | 12/04/2011 0:00 | 12 | 49.762970 | -109.209600 | 628939 | 5513815 | 0 | 1.11 | Local |
| F4 | 12/04/2011 0:00 | 18 | 49.762980 | -109.209600 | 628939 | 5513816 | 1 | 6.72 | Local |
| F4 | 12/04/2011 0:00 | 21 | 49.762940 | -109.209670 | 628934 | 5513812 | 1 | 575.71 | Local |
| F4 | 12/05/2011 0:00 | 0 | 49.767020 | -109.214590 | 628569 | 5514257 | 1 | 241.26 | Local |
| F4 | 12/05/2011 0:00 | 3 | 49.765520 | -109.217010 | 628399 | 5514086 | 1 | 8.08 | Local |
| F4 | 12/05/2011 0:00 | 6 | 49.765450 | -109.217040 | 628397 | 5514078 | 1 | 217.40 | Local |
| F4 | 12/05/2011 0:00 | 9 | 49.765140 | -109.214060 | 628612 | 5514049 | 1 | 404.59 | Local |
| F4 | 12/05/2011 0:00 | 12 | 49.762920 | -109.209610 | 628938 | 5513810 | 1 | 655.54 | Local |
| F4 | 12/05/2011 0:00 | 15 | 49.765620 | -109.217700 | 628349 | 5514096 | 1 | 43.57 | Local |
| F4 | 12/05/2011 0:00 | 18 | 49.765570 | -109.217100 | 628392 | 5514091 | 1 | 1001.62 | Local |
| F4 | 12/05/2011 0:00 | 21 | 49.772160 | -109.226580 | 627692 | 5514808 | 0 | 927.60 | Local |
| F4 | 12/06/2011 0:00 | 3 | 49.778190 | -109.235480 | 627036 | 5515463 | 1 | 21.16 | Local |
| F4 | 12/06/2011 0:00 | 6 | 49.778290 | -109.235730 | 627017 | 5515474 | 1 | 648.59 | Local |
| F4 | 12/06/2011 0:00 | 9 | 49.774620 | -109.228730 | 627531 | 5515078 | 0 | 8.90 | Local |
| F4 | 12/07/2011 0:00 | 0 | 49.774700 | -109.228730 | 627531 | 5515087 | 1 | 399.80 | Local |
| F4 | 12/07/2011 0:00 | 3 | 49.774050 | -109.234190 | 627139 | 5515005 | 0 | 402.43 | Local |
| F4 | 12/07/2011 0:00 | 9 | 49.774690 | -109.228690 | 627534 | 5515086 | 0 | 351.78 | Local |
| F4 | 12/08/2011 0:00 | 3 | 49.773900 | -109.233420 | 627195 | 5514990 | 0 | 396.54 | Local |
| F4 | 12/08/2011 0:00 | 18 | 49.773730 | -109.227920 | 627591 | 5514980 | 1 | 718.30 | Local |
| F4 | 12/08/2011 0:00 | 21 | 49.772600 | -109.218100 | 628301 | 5514871 | 0 | 814.07 | Local |
| F4 | 12/09/2011 0:00 | 9 | 49.774740 | -109.228910 | 627518 | 5515091 | 0 | 16.79 | Local |
| F4 | 12/09/2011 0:00 | 18 | 49.774690 | -109.228690 | 627534 | 5515086 | 0 | 2.16 | Local |
| F4 | 12/10/2011 0:00 | 3 | 49.774690 | -109.228720 | 627531 | 5515086 | 1 | 4.95 | Local |
| F4 | 12/10/2011 0:00 | 6 | 49.774650 | -109.228750 | 627529 | 5515081 | 1 | 12.25 | Local |
| F4 | 12/10/2011 0:00 | 9 | 49.774540 | -109.228760 | 627529 | 5515069 | 1 | 17.85 | Local |
| F4 | 12/10/2011 0:00 | 12 | 49.774700 | -109.228740 | 627530 | 5515087 | 1 | 18.30 | Local |
| F4 | 12/10/2011 0:00 | 15 | 49.774630 | -109.228510 | 627547 | 5515079 | 1 | 47.06 | Local |
| F4 | 12/10/2011 0:00 | 18 | 49.775050 | -109.228430 | 627551 | 5515126 | 1 | 45.15 | Local |
| F4 | 12/10/2011 0:00 | 21 | 49.774690 | -109.228720 | 627531 | 5515086 | 1 | 3.10 | Local |
| F4 | 12/11/2011 0:00 | 0 | 49.774710 | -109.228690 | 627533 | 5515088 | 1 | 4.41 | Local |
| F4 | 12/11/2011 0:00 | 3 | 49.774740 | -109.228650 | 627536 | 5515091 | 1 | 7.27 | Local |
| F4 | 12/11/2011 0:00 | 6 | 49.774680 | -109.228690 | 627534 | 5515084 | 1 | 1.82 | Local |
| F4 | 12/11/2011 0:00 | 9 | 49.774670 | -109.228670 | 627535 | 5515083 | 1 | 86.60 | Local |
| F4 | 12/11/2011 0:00 | 12 | 49.774820 | -109.227490 | 627620 | 5515102 | 1 | 85.85 | Local |
| F4 | 12/11/2011 0:00 | 15 | 49.774710 | -109.228670 | 627535 | 5515088 | 1 | 16.16 | Local |
| F4 | 12/11/2011 0:00 | 18 | 49.774570 | -109.228730 | 627531 | 5515072 | 1 | 1267.32 | Local |

| | | | | | | | | | |
|---|---|---|---|---|---|---|---|---|---|
| F4 | 12/11/2011 0:00 | 21 | 49.766110 | -109.216940 | 628402 | 5514152 | 1 | 38.26 | Local |
| F4 | 12/12/2011 0:00 | 0 | 49.765810 | -109.216680 | 628422 | 5514119 | 1 | 4.41 | Local |
| F4 | 12/12/2011 0:00 | 3 | 49.765780 | -109.216640 | 628425 | 5514116 | 1 | 27.81 | Local |
| F4 | 12/12/2011 0:00 | 6 | 49.765640 | -109.216960 | 628402 | 5514099 | 1 | 27.12 | Local |
| F4 | 12/12/2011 0:00 | 9 | 49.765730 | -109.216610 | 628427 | 5514110 | 1 | 0.00 | Local |
| F4 | 12/12/2011 0:00 | 12 | 49.765730 | -109.216610 | 628427 | 5514110 | 1 | 589.32 | Local |
| F4 | 12/12/2011 0:00 | 15 | 49.763030 | -109.209570 | 628941 | 5513822 | 1 | 594.07 | Local |
| F4 | 12/12/2011 0:00 | 18 | 49.765770 | -109.216650 | 628424 | 5514114 | 0 | 1130.17 | Local |
| F4 | 12/13/2011 0:00 | 18 | 49.774640 | -109.224310 | 627849 | 5515087 | 1 | 2281.63 | Local |
| F4 | 12/13/2011 0:00 | 21 | 49.757770 | -109.242340 | 626595 | 5513181 | 1 | 676.85 | Local |
| F4 | 12/14/2011 0:00 | 0 | 49.758430 | -109.251680 | 625920 | 5513239 | 0 | 2383.52 | Local |
| F4 | 12/14/2011 0:00 | 18 | 49.774790 | -109.230300 | 627417 | 5515094 | 1 | 1020.47 | Local |
| F4 | 12/14/2011 0:00 | 21 | 49.767660 | -109.221380 | 628078 | 5514316 | 1 | 1976.33 | Local |
| F4 | 12/15/2011 0:00 | 0 | 49.750270 | -109.227040 | 627716 | 5512374 | 1 | 2378.08 | Local |
| F4 | 12/15/2011 0:00 | 3 | 49.771510 | -109.230880 | 627384 | 5514728 | 0 | 1318.07 | Local |
| F4 | 12/16/2011 0:00 | 3 | 49.776060 | -109.247780 | 626156 | 5515206 | 1 | 2014.08 | Local |
| F4 | 12/16/2011 0:00 | 6 | 49.772070 | -109.275060 | 624202 | 5514717 | 0 | 1783.55 | Local |
| F4 | 12/17/2011 0:00 | 0 | 49.783420 | -109.257560 | 625432 | 5516007 | 1 | 1251.90 | Local |
| F4 | 12/17/2011 0:00 | 3 | 49.772180 | -109.256580 | 625532 | 5514759 | 0 | 2168.23 | Local |
| F4 | 12/17/2011 0:00 | 15 | 49.756620 | -109.238440 | 626879 | 5513060 | 1 | 1109.62 | Local |
| F4 | 12/17/2011 0:00 | 18 | 49.751300 | -109.225410 | 627831 | 5512491 | 1 | 2076.53 | Local |
| F4 | 12/17/2011 0:00 | 21 | 49.735650 | -109.209690 | 629005 | 5510778 | 1 | 1535.92 | Local |
| F4 | 12/18/2011 0:00 | 0 | 49.743770 | -109.192450 | 630225 | 5511710 | 1 | 1022.46 | Local |
| F4 | 12/18/2011 0:00 | 3 | 49.748060 | -109.179900 | 631118 | 5512209 | 1 | 43.94 | Local |
| F4 | 12/18/2011 0:00 | 6 | 49.748160 | -109.180490 | 631075 | 5512219 | 1 | 204.04 | Local |
| F4 | 12/18/2011 0:00 | 9 | 49.749080 | -109.178040 | 631249 | 5512326 | 1 | 2310.16 | Local |
| F4 | 12/18/2011 0:00 | 12 | 49.759860 | -109.205450 | 629246 | 5513477 | 0 | 516.30 | Local |
| F4 | 12/18/2011 0:00 | 18 | 49.763520 | -109.209860 | 628919 | 5513876 | 0 | 16.68 | Local |
| F4 | 12/19/2011 0:00 | 3 | 49.763370 | -109.209860 | 628919 | 5513859 | 0 | 0.00 | Local |
| F4 | 12/19/2011 0:00 | 9 | 49.763370 | -109.209860 | 628919 | 5513859 | 0 | 10.23 | Local |
| F4 | 12/19/2011 0:00 | 21 | 49.763290 | -109.209930 | 628914 | 5513850 | 0 | 506.19 | Local |
| F4 | 12/20/2011 0:00 | 3 | 49.766040 | -109.215530 | 628504 | 5514146 | 1 | 1391.19 | Local |
| F4 | 12/20/2011 0:00 | 6 | 49.770390 | -109.233640 | 627188 | 5514599 | 1 | 174.92 | Local |
| F4 | 12/20/2011 0:00 | 9 | 49.771070 | -109.235830 | 627029 | 5514671 | 0 | 330.29 | Local |
| F4 | 12/20/2011 0:00 | 18 | 49.772170 | -109.240090 | 626719 | 5514786 | 0 | 1851.33 | Local |
| F4 | 12/21/2011 0:00 | 3 | 49.758870 | -109.255550 | 625641 | 5513282 | 1 | 1777.30 | Local |
| F4 | 12/21/2011 0:00 | 6 | 49.752090 | -109.233210 | 627267 | 5512565 | 0 | 3348.99 | Local |
| F4 | 12/22/2011 0:00 | 0 | 49.781860 | -109.240240 | 626683 | 5515863 | 1 | 876.45 | Local |
| F4 | 12/22/2011 0:00 | 3 | 49.787230 | -109.231330 | 627311 | 5516475 | 1 | 6.04 | Local |
| F4 | 12/22/2011 0:00 | 6 | 49.787200 | -109.231260 | 627316 | 5516472 | 1 | 5.74 | Local |
| F4 | 12/22/2011 0:00 | 9 | 49.787150 | -109.231280 | 627314 | 5516466 | 0 | 20.22 | Local |
| F4 | 12/23/2011 0:00 | 9 | 49.787330 | -109.231240 | 627317 | 5516486 | 0 | 911.06 | Local |
| F4 | 12/24/2011 0:00 | 3 | 49.786730 | -109.218620 | 628227 | 5516441 | 1 | 297.63 | Local |
| F4 | 12/24/2011 0:00 | 6 | 49.789310 | -109.217520 | 628299 | 5516730 | 0 | 591.45 | Local |
| F4 | 12/24/2011 0:00 | 18 | 49.794140 | -109.214080 | 628534 | 5517273 | 1 | 11.49 | Local |
| F4 | 12/24/2011 0:00 | 21 | 49.794040 | -109.214040 | 628537 | 5517262 | 1 | 10.13 | Local |
| F4 | 12/25/2011 0:00 | 0 | 49.794110 | -109.213950 | 628543 | 5517270 | 1 | 7.58 | Local |
| F4 | 12/25/2011 0:00 | 3 | 49.794050 | -109.214000 | 628540 | 5517263 | 1 | 20.10 | Local |
| F4 | 12/25/2011 0:00 | 6 | 49.794210 | -109.213870 | 628549 | 5517281 | 1 | 1423.47 | Local |
| F4 | 12/25/2011 0:00 | 9 | 49.781410 | -109.214070 | 628568 | 5515858 | 0 | 1822.94 | Local |
| F4 | 12/26/2011 0:00 | 0 | 49.774590 | -109.237090 | 626929 | 5515060 | 1 | 1563.07 | Local |
| F4 | 12/26/2011 0:00 | 3 | 49.770620 | -109.257910 | 625440 | 5514584 | 0 | 968.02 | Local |
| F4 | 12/26/2011 0:00 | 9 | 49.761930 | -109.257120 | 625520 | 5513619 | 0 | 709.45 | Local |
| F4 | 12/26/2011 0:00 | 18 | 49.767840 | -109.253410 | 625772 | 5514282 | 1 | 1499.33 | Local |
| F4 | 12/26/2011 0:00 | 21 | 49.777730 | -109.267560 | 624727 | 5515358 | 1 | 801.20 | Local |

| | | | | | | | | | |
|---|---|---|---|---|---|---|---|---|---|
| F4 | 12/27/2011 0:00 | 0 | 49.781820 | -109.258400 | 625376 | 5515828 | 1 | 2374.73 | Local |
| F4 | 12/27/2011 0:00 | 3 | 49.791800 | -109.229240 | 627449 | 5516987 | 1 | 787.25 | Local |
| F4 | 12/27/2011 0:00 | 6 | 49.798670 | -109.226600 | 627621 | 5517755 | 1 | 1384.55 | Local |
| F4 | 12/27/2011 0:00 | 9 | 49.786250 | -109.227950 | 627556 | 5516372 | 0 | 594.13 | Local |
| F4 | 12/28/2011 0:00 | 6 | 49.784750 | -109.220030 | 628130 | 5516219 | 0 | 1122.04 | Local |
| F4 | 12/28/2011 0:00 | 12 | 49.789020 | -109.205910 | 629135 | 5516718 | 0 | 195.39 | Local |
| F4 | 12/28/2011 0:00 | 18 | 49.790480 | -109.207420 | 629023 | 5516877 | 1 | 1729.52 | Local |
| F4 | 12/28/2011 0:00 | 21 | 49.798990 | -109.227530 | 627553 | 5517789 | 1 | 990.93 | Local |
| F4 | 12/29/2011 0:00 | 0 | 49.806900 | -109.233870 | 627076 | 5518658 | 1 | 679.14 | Local |
| F4 | 12/29/2011 0:00 | 3 | 49.800820 | -109.232980 | 627156 | 5517983 | 1 | 2878.21 | Local |
| F4 | 12/29/2011 0:00 | 6 | 49.784380 | -109.202100 | 629422 | 5516208 | 1 | 1459.29 | Local |
| F4 | 12/29/2011 0:00 | 9 | 49.796050 | -109.192830 | 630058 | 5517522 | 0 | 1142.51 | Local |
| F4 | 12/29/2011 0:00 | 15 | 49.789880 | -109.205520 | 629161 | 5516814 | 0 | 1904.90 | Local |
| F4 | 12/30/2011 0:00 | 3 | 49.800600 | -109.226160 | 627648 | 5517970 | 1 | 13.31 | Local |
| F4 | 12/30/2011 0:00 | 6 | 49.800540 | -109.226320 | 627636 | 5517963 | 1 | 348.42 | Local |
| F4 | 12/30/2011 0:00 | 9 | 49.800490 | -109.231160 | 627288 | 5517950 | 0 | 2313.98 | Local |
| F4 | 12/30/2011 0:00 | 15 | 49.780670 | -109.240950 | 626635 | 5515730 | 0 | 40.45 | Local |
| F4 | 12/30/2011 0:00 | 21 | 49.781030 | -109.241030 | 626629 | 5515769 | 0 | 631.11 | Local |
| F4 | 12/31/2011 0:00 | 3 | 49.785480 | -109.235590 | 627009 | 5516273 | 1 | 1643.98 | Local |
| F4 | 12/31/2011 0:00 | 6 | 49.800250 | -109.236570 | 626899 | 5517914 | 1 | 2131.28 | Local |
| F4 | 12/31/2011 0:00 | 9 | 49.782400 | -109.247350 | 626170 | 5515911 | 1 | 1298.42 | Local |
| F4 | 12/31/2011 0:00 | 12 | 49.771100 | -109.242810 | 626526 | 5514663 | 1 | 1.11 | Local |
| F4 | 12/31/2011 0:00 | 15 | 49.771110 | -109.242810 | 626526 | 5514664 | 1 | 193.49 | Local |
| F4 | 12/31/2011 0:00 | 18 | 49.769480 | -109.241870 | 626598 | 5514484 | 0 | 938.51 | Local |
| F4 | 01/01/2012 0:00 | 3 | 49.776400 | -109.234410 | 627117 | 5515266 | 1 | 2276.77 | Local |
| F4 | 01/01/2012 0:00 | 6 | 49.795650 | -109.223640 | 627842 | 5517424 | 1 | 2159.78 | Local |
| F4 | 01/01/2012 0:00 | 9 | 49.791190 | -109.194440 | 629955 | 5516979 | 0 | 794.72 | Local |
| F4 | 01/01/2012 0:00 | 18 | 49.789310 | -109.205090 | 629194 | 5516751 | 1 | 55.90 | Local |
| F4 | 01/01/2012 0:00 | 21 | 49.789180 | -109.205840 | 629140 | 5516736 | 1 | 10.89 | Local |
| F4 | 01/02/2012 0:00 | 0 | 49.789230 | -109.205710 | 629149 | 5516741 | 1 | 488.75 | Local |
| F4 | 01/02/2012 0:00 | 3 | 49.785980 | -109.210280 | 628829 | 5516372 | 1 | 53.53 | Local |
| F4 | 01/02/2012 0:00 | 6 | 49.786100 | -109.211000 | 628777 | 5516384 | 1 | 459.69 | Local |
| F4 | 01/02/2012 0:00 | 9 | 49.782170 | -109.212980 | 628645 | 5515944 | 0 | 548.85 | Local |
| F4 | 01/02/2012 0:00 | 18 | 49.781540 | -109.220540 | 628102 | 5515861 | 1 | 312.45 | Local |
| F4 | 01/02/2012 0:00 | 21 | 49.784320 | -109.219910 | 628140 | 5516171 | 1 | 631.69 | Local |
| F4 | 01/03/2012 0:00 | 0 | 49.788900 | -109.214720 | 628502 | 5516689 | 1 | 1141.26 | Local |
| F4 | 01/03/2012 0:00 | 3 | 49.798370 | -109.220830 | 628037 | 5517732 | 1 | 1562.23 | Local |
| F4 | 01/03/2012 0:00 | 6 | 49.789260 | -109.237350 | 626872 | 5516691 | 1 | 2014.20 | Local |
| F4 | 01/03/2012 0:00 | 9 | 49.792560 | -109.264860 | 624883 | 5517011 | 0 | 1286.03 | Local |
| F4 | 01/03/2012 0:00 | 15 | 49.784670 | -109.251800 | 625844 | 5516156 | 1 | 6.20 | Local |
| F4 | 01/03/2012 0:00 | 18 | 49.784630 | -109.251860 | 625840 | 5516151 | 1 | 738.92 | Local |
| F4 | 01/03/2012 0:00 | 21 | 49.788060 | -109.260650 | 625198 | 5516518 | 1 | 573.53 | Local |
| F4 | 01/04/2012 0:00 | 0 | 49.792600 | -109.264430 | 624914 | 5517017 | 1 | 9.10 | Local |
| F4 | 01/04/2012 0:00 | 3 | 49.792650 | -109.264530 | 624907 | 5517022 | 1 | 30.32 | Local |
| F4 | 01/04/2012 0:00 | 6 | 49.792410 | -109.264730 | 624893 | 5516995 | 1 | 1182.10 | Local |
| F4 | 01/04/2012 0:00 | 9 | 49.785720 | -109.251970 | 625829 | 5516272 | 0 | 462.37 | Local |
| F4 | 01/04/2012 0:00 | 15 | 49.785820 | -109.245550 | 626291 | 5516294 | 1 | 272.21 | Local |
| F4 | 01/04/2012 0:00 | 18 | 49.786220 | -109.241820 | 626558 | 5516345 | 1 | 11.10 | Local |
| F4 | 01/04/2012 0:00 | 21 | 49.786150 | -109.241930 | 626550 | 5516337 | 1 | 225.18 | Local |
| F4 | 01/05/2012 0:00 | 0 | 49.788020 | -109.240730 | 626632 | 5516547 | 1 | 1594.72 | Local |
| F4 | 01/05/2012 0:00 | 3 | 49.784110 | -109.219420 | 628176 | 5516149 | 1 | 878.83 | Local |
| F4 | 01/05/2012 0:00 | 6 | 49.788830 | -109.209630 | 628868 | 5516690 | 1 | 1321.41 | Local |
| F4 | 01/05/2012 0:00 | 9 | 49.776950 | -109.210030 | 628871 | 5515369 | 1 | 7.29 | Local |
| F4 | 01/05/2012 0:00 | 12 | 49.776980 | -109.210120 | 628864 | 5515372 | 1 | 412.67 | Local |
| F4 | 01/05/2012 0:00 | 15 | 49.776400 | -109.215780 | 628458 | 5515298 | 1 | 377.03 | Local |

| | | | | | | | | | |
|---|---|---|---|---|---|---|---|---|---|
| F4 | 01/05/2012 0:00 | 18 | 49.774350 | -109.211610 | 628764 | 5515077 | 1 | 1351.33 | Local |
| F4 | 01/05/2012 0:00 | 21 | 49.785890 | -109.217490 | 628310 | 5516350 | 0 | 1745.69 | Local |
| F4 | 01/06/2012 0:00 | 3 | 49.791250 | -109.194700 | 629936 | 5516985 | 1 | 336.48 | Local |
| F4 | 01/06/2012 0:00 | 6 | 49.788270 | -109.195510 | 629886 | 5516652 | 1 | 320.20 | Local |
| F4 | 01/06/2012 0:00 | 9 | 49.785920 | -109.198080 | 629707 | 5516387 | 1 | 231.22 | Local |
| F4 | 01/06/2012 0:00 | 12 | 49.784160 | -109.199790 | 629589 | 5516188 | 1 | 16.30 | Local |
| F4 | 01/06/2012 0:00 | 15 | 49.784280 | -109.199920 | 629579 | 5516201 | 1 | 744.05 | Local |
| F4 | 01/06/2012 0:00 | 18 | 49.781740 | -109.209480 | 628898 | 5515902 | 1 | 1207.30 | Local |
| F4 | 01/06/2012 0:00 | 21 | 49.788360 | -109.222770 | 627924 | 5516615 | 1 | 1913.46 | Local |
| F4 | 01/07/2012 0:00 | 0 | 49.803820 | -109.234440 | 627043 | 5518314 | 1 | 805.67 | Local |
| F4 | 01/07/2012 0:00 | 3 | 49.808780 | -109.242600 | 626443 | 5518852 | 1 | 638.35 | Local |
| F4 | 01/07/2012 0:00 | 6 | 49.814090 | -109.245970 | 626187 | 5519437 | 1 | 1202.00 | Local |
| F4 | 01/07/2012 0:00 | 9 | 49.805990 | -109.234910 | 627004 | 5518555 | 1 | 1870.05 | Local |
| F4 | 01/07/2012 0:00 | 12 | 49.789240 | -109.237220 | 626881 | 5516689 | 1 | 7.04 | Local |
| F4 | 01/07/2012 0:00 | 15 | 49.789190 | -109.237160 | 626886 | 5516683 | 1 | 569.18 | Local |
| F4 | 01/07/2012 0:00 | 18 | 49.784220 | -109.239050 | 626763 | 5516127 | 1 | 56.97 | Local |
| F4 | 01/07/2012 0:00 | 21 | 49.783910 | -109.238420 | 626809 | 5516094 | 1 | 3.64 | Local |
| F4 | 01/08/2012 0:00 | 0 | 49.783890 | -109.238380 | 626812 | 5516092 | 1 | 763.60 | Local |
| F4 | 01/08/2012 0:00 | 3 | 49.778610 | -109.231600 | 627314 | 5515516 | 1 | 2395.56 | Local |
| F4 | 01/08/2012 0:00 | 6 | 49.758600 | -109.219280 | 628253 | 5513313 | 1 | 1060.02 | Local |
| F4 | 01/08/2012 0:00 | 9 | 49.750380 | -109.226730 | 627739 | 5512386 | 1 | 125.22 | Local |
| F4 | 01/08/2012 0:00 | 12 | 49.751500 | -109.226910 | 627723 | 5512511 | 1 | 10.13 | Local |
| F4 | 01/08/2012 0:00 | 15 | 49.751430 | -109.227000 | 627716 | 5512503 | 1 | 96.81 | Local |
| F4 | 01/08/2012 0:00 | 18 | 49.750560 | -109.227050 | 627715 | 5512406 | 1 | 5.74 | Local |
| F4 | 01/08/2012 0:00 | 21 | 49.750610 | -109.227070 | 627713 | 5512411 | 1 | 100.40 | Local |
| F4 | 01/09/2012 0:00 | 0 | 49.751510 | -109.226960 | 627719 | 5512512 | 1 | 5.72 | Local |
| F4 | 01/09/2012 0:00 | 3 | 49.751470 | -109.227010 | 627715 | 5512507 | 1 | 4.95 | Local |
| F4 | 01/09/2012 0:00 | 6 | 49.751430 | -109.227040 | 627713 | 5512503 | 1 | 128.46 | Local |
| F4 | 01/09/2012 0:00 | 9 | 49.750300 | -109.226670 | 627743 | 5512378 | 1 | 11.91 | Local |
| F4 | 01/09/2012 0:00 | 12 | 49.750380 | -109.226780 | 627735 | 5512386 | 1 | 240.78 | Local |
| F4 | 01/09/2012 0:00 | 15 | 49.748340 | -109.225660 | 627821 | 5512161 | 1 | 1204.05 | Local |
| F4 | 01/09/2012 0:00 | 18 | 49.759060 | -109.228010 | 627624 | 5513349 | 1 | 919.51 | Local |
| F4 | 01/09/2012 0:00 | 21 | 49.759270 | -109.240770 | 626704 | 5513351 | 1 | 1458.70 | Local |
| F4 | 01/10/2012 0:00 | 0 | 49.747450 | -109.249550 | 626102 | 5512022 | 1 | 5.46 | Local |
| F4 | 01/10/2012 0:00 | 3 | 49.747480 | -109.249610 | 626098 | 5512025 | 1 | 823.14 | Local |
| F4 | 01/10/2012 0:00 | 6 | 49.753190 | -109.256880 | 625559 | 5512648 | 1 | 1774.93 | Local |
| F4 | 01/10/2012 0:00 | 9 | 49.768010 | -109.266030 | 624862 | 5514280 | 1 | 760.96 | Local |
| F4 | 01/10/2012 0:00 | 12 | 49.773130 | -109.259020 | 625354 | 5514861 | 1 | 911.61 | Local |
| F4 | 01/10/2012 0:00 | 15 | 49.779520 | -109.251090 | 625908 | 5515585 | 1 | 727.09 | Local |
| F4 | 01/10/2012 0:00 | 18 | 49.784150 | -109.243960 | 626410 | 5516111 | 1 | 2273.04 | Local |
| F4 | 01/10/2012 0:00 | 21 | 49.803790 | -109.235210 | 626988 | 5518310 | 1 | 2719.77 | Local |
| F4 | 01/11/2012 0:00 | 0 | 49.782290 | -109.217200 | 628341 | 5515950 | 1 | 226.67 | Local |
| F4 | 01/11/2012 0:00 | 3 | 49.784190 | -109.218340 | 628254 | 5516159 | 1 | 91.45 | Local |
| F4 | 01/11/2012 0:00 | 6 | 49.784200 | -109.219610 | 628162 | 5516158 | 1 | 1201.09 | Local |
| F4 | 01/11/2012 0:00 | 9 | 49.774040 | -109.213950 | 628596 | 5515038 | 1 | 2091.68 | Local |
| F4 | 01/11/2012 0:00 | 12 | 49.755620 | -109.208070 | 629069 | 5513001 | 1 | 18.90 | Local |
| F4 | 01/11/2012 0:00 | 15 | 49.755790 | -109.208070 | 629068 | 5513020 | 1 | 1729.68 | Local |
| F4 | 01/11/2012 0:00 | 18 | 49.761390 | -109.230470 | 627440 | 5513604 | 1 | 3977.82 | Local |
| F4 | 01/11/2012 0:00 | 21 | 49.795150 | -109.212210 | 628666 | 5517388 | 1 | 3489.49 | Local |
| F4 | 01/12/2012 0:00 | 0 | 49.819110 | -109.243520 | 626350 | 5519999 | 1 | 720.24 | Local |
| F4 | 01/12/2012 0:00 | 3 | 49.812640 | -109.243980 | 626334 | 5519279 | 1 | 10.36 | Local |
| F4 | 01/12/2012 0:00 | 6 | 49.812680 | -109.243850 | 626343 | 5519283 | 1 | 1970.43 | Local |
| F4 | 01/12/2012 0:00 | 9 | 49.796420 | -109.232970 | 627168 | 5517494 | 1 | 858.95 | Local |
| F4 | 01/12/2012 0:00 | 12 | 49.789200 | -109.237210 | 626882 | 5516684 | 1 | 5.72 | Local |
| F4 | 01/12/2012 0:00 | 15 | 49.789240 | -109.237260 | 626879 | 5516689 | 1 | 3.09 | Local |

| | | | | | | | | | |
|---|---|---|---|---|---|---|---|---|---|
| F4 | 01/12/2012 0:00 | 18 | 49.789230 | -109.237300 | 626876 | 5516687 | 1 | 2252.28 | Local |
| F4 | 01/12/2012 0:00 | 21 | 49.793260 | -109.267960 | 624659 | 5517084 | 1 | 798.84 | Local |
| F4 | 01/13/2012 0:00 | 0 | 49.786200 | -109.270010 | 624529 | 5516296 | 1 | 2605.57 | Local |
| F4 | 01/13/2012 0:00 | 3 | 49.762830 | -109.267400 | 624777 | 5513702 | 1 | 127.67 | Local |
| F4 | 01/13/2012 0:00 | 6 | 49.761710 | -109.267010 | 624808 | 5513578 | 1 | 403.76 | Local |
| F4 | 01/13/2012 0:00 | 9 | 49.764460 | -109.263350 | 625064 | 5513890 | 0 | 713.90 | Local |
| F4 | 01/13/2012 0:00 | 18 | 49.768250 | -109.255350 | 625631 | 5514325 | 1 | 1793.21 | Local |
| F4 | 01/13/2012 0:00 | 21 | 49.774890 | -109.232660 | 627247 | 5515101 | 1 | 931.64 | Local |
| F4 | 01/14/2012 0:00 | 0 | 49.781670 | -109.225060 | 627777 | 5515868 | 0 | 14.58 | Local |
| F4 | 01/14/2012 0:00 | 6 | 49.781730 | -109.225240 | 627763 | 5515874 | 1 | 7.50 | Local |
| F4 | 01/14/2012 0:00 | 9 | 49.781680 | -109.225310 | 627758 | 5515868 | 1 | 4.86 | Local |
| F4 | 01/14/2012 0:00 | 12 | 49.781660 | -109.225250 | 627763 | 5515866 | 1 | 4.23 | Local |
| F4 | 01/14/2012 0:00 | 15 | 49.781680 | -109.225200 | 627766 | 5515869 | 1 | 5.87 | Local |
| F4 | 01/14/2012 0:00 | 18 | 49.781690 | -109.225120 | 627772 | 5515870 | 1 | 20.59 | Local |
| F4 | 01/14/2012 0:00 | 21 | 49.781580 | -109.225350 | 627756 | 5515857 | 1 | 11.63 | Local |
| F4 | 01/15/2012 0:00 | 0 | 49.781650 | -109.225230 | 627764 | 5515865 | 1 | 5.87 | Local |
| F4 | 01/15/2012 0:00 | 3 | 49.781640 | -109.225310 | 627759 | 5515864 | 1 | 2.43 | Local |
| F4 | 01/15/2012 0:00 | 6 | 49.781630 | -109.225280 | 627761 | 5515863 | 1 | 6.04 | Local |
| F4 | 01/15/2012 0:00 | 9 | 49.781600 | -109.225210 | 627766 | 5515860 | 1 | 6.83 | Local |
| F4 | 01/15/2012 0:00 | 12 | 49.781660 | -109.225190 | 627767 | 5515866 | 1 | 0.72 | Local |
| F4 | 01/15/2012 0:00 | 15 | 49.781660 | -109.225180 | 627768 | 5515866 | 1 | 733.61 | Local |
| F4 | 01/15/2012 0:00 | 18 | 49.779070 | -109.234550 | 627100 | 5515563 | 1 | 734.05 | Local |
| F4 | 01/15/2012 0:00 | 21 | 49.781670 | -109.225180 | 627768 | 5515868 | 1 | 4.86 | Local |
| F4 | 01/16/2012 0:00 | 0 | 49.781690 | -109.225240 | 627764 | 5515870 | 1 | 6.26 | Local |
| F4 | 01/16/2012 0:00 | 3 | 49.781640 | -109.225200 | 627767 | 5515864 | 1 | 5.30 | Local |
| F4 | 01/16/2012 0:00 | 6 | 49.781680 | -109.225240 | 627764 | 5515869 | 1 | 3.97 | Local |
| F4 | 01/16/2012 0:00 | 9 | 49.781650 | -109.225210 | 627766 | 5515865 | 1 | 3.97 | Local |
| F4 | 01/16/2012 0:00 | 12 | 49.781620 | -109.225180 | 627768 | 5515862 | 1 | 5.51 | Local |
| F4 | 01/16/2012 0:00 | 15 | 49.781640 | -109.225250 | 627763 | 5515864 | 1 | 3.09 | Local |
| F4 | 01/16/2012 0:00 | 18 | 49.781650 | -109.225210 | 627766 | 5515865 | 1 | 4.32 | Local |
| F4 | 01/16/2012 0:00 | 21 | 49.781650 | -109.225150 | 627770 | 5515865 | 1 | 8.00 | Local |
| F4 | 01/17/2012 0:00 | 0 | 49.781660 | -109.225260 | 627762 | 5515866 | 1 | 10.36 | Local |
| F4 | 01/17/2012 0:00 | 3 | 49.781620 | -109.225130 | 627772 | 5515862 | 1 | 6.66 | Local |
| F4 | 01/17/2012 0:00 | 6 | 49.781650 | -109.225210 | 627766 | 5515865 | 1 | 7.94 | Local |
| F4 | 01/17/2012 0:00 | 9 | 49.781680 | -109.225310 | 627758 | 5515868 | 1 | 7.29 | Local |
| F4 | 01/17/2012 0:00 | 12 | 49.781650 | -109.225220 | 627765 | 5515865 | 1 | 2.65 | Local |
| F4 | 01/17/2012 0:00 | 15 | 49.781670 | -109.225200 | 627766 | 5515867 | 1 | 1.11 | Local |
| F4 | 01/17/2012 0:00 | 18 | 49.781660 | -109.225200 | 627766 | 5515866 | 1 | 3.64 | Local |
| F4 | 01/17/2012 0:00 | 21 | 49.781680 | -109.225160 | 627769 | 5515869 | 1 | 3.60 | Local |
| F4 | 01/18/2012 0:00 | 0 | 49.781680 | -109.225210 | 627766 | 5515869 | 1 | 6.04 | Local |
| F4 | 01/18/2012 0:00 | 3 | 49.781710 | -109.225280 | 627761 | 5515872 | 1 | 174.29 | Local |
| F4 | 01/18/2012 0:00 | 6 | 49.781680 | -109.222860 | 627935 | 5515873 | 1 | 164.93 | Local |
| F4 | 01/18/2012 0:00 | 9 | 49.781710 | -109.225150 | 627770 | 5515872 | 1 | 5.46 | Local |
| F4 | 01/18/2012 0:00 | 12 | 49.781680 | -109.225210 | 627766 | 5515869 | 0 | 2961.00 | Local |
| F4 | 01/19/2012 0:00 | 6 | 49.769610 | -109.261860 | 625159 | 5514465 | 1 | 1966.07 | Local |
| F4 | 01/19/2012 0:00 | 9 | 49.773500 | -109.235230 | 627066 | 5514942 | 0 | 649.50 | Local |
| F4 | 01/19/2012 0:00 | 18 | 49.777410 | -109.228530 | 627538 | 5515388 | 1 | 1894.42 | Local |
| F4 | 01/19/2012 0:00 | 21 | 49.794430 | -109.229660 | 627412 | 5517278 | 1 | 1941.33 | Local |
| F4 | 01/20/2012 0:00 | 0 | 49.810620 | -109.239750 | 626644 | 5519061 | 1 | 14.50 | Local |
| F4 | 01/20/2012 0:00 | 3 | 49.810690 | -109.239920 | 626631 | 5519069 | 1 | 2.65 | Local |
| F4 | 01/20/2012 0:00 | 6 | 49.810710 | -109.239900 | 626632 | 5519071 | 1 | 2802.63 | Local |
| F4 | 01/20/2012 0:00 | 9 | 49.785570 | -109.237150 | 626896 | 5516281 | 0 | 2362.09 | Local |
| F4 | 01/21/2012 0:00 | 3 | 49.765850 | -109.224960 | 627825 | 5514109 | 1 | 1086.28 | Local |
| F4 | 01/21/2012 0:00 | 6 | 49.758120 | -109.234180 | 627182 | 5513234 | 1 | 1127.89 | Local |
| F4 | 01/21/2012 0:00 | 9 | 49.765280 | -109.223090 | 627961 | 5514049 | 0 | 3273.38 | Local |

| | | | | | | | | | |
|---|---|---|---|---|---|---|---|---|---|
| F4 | 01/22/2012 0:00 | 0 | 49.783030 | -109.259350 | 625305 | 5515961 | 1 | 226.26 | Local |
| F4 | 01/22/2012 0:00 | 3 | 49.782000 | -109.256640 | 625502 | 5515851 | 0 | 21.35 | Local |
| F4 | 01/22/2012 0:00 | 9 | 49.782040 | -109.256930 | 625481 | 5515855 | 1 | 574.11 | Local |
| F4 | 01/22/2012 0:00 | 12 | 49.776890 | -109.257490 | 625454 | 5515282 | 1 | 30.67 | Local |
| F4 | 01/22/2012 0:00 | 15 | 49.776640 | -109.257310 | 625468 | 5515254 | 1 | 1729.29 | Local |
| F4 | 01/22/2012 0:00 | 18 | 49.763250 | -109.245100 | 626382 | 5513786 | 0 | 1770.15 | Local |
| F4 | 01/23/2012 0:00 | 3 | 49.779150 | -109.243920 | 626425 | 5515556 | 1 | 388.53 | Local |
| F4 | 01/23/2012 0:00 | 6 | 49.779650 | -109.238580 | 626809 | 5515620 | 1 | 2276.54 | Local |
| F4 | 01/23/2012 0:00 | 9 | 49.773060 | -109.208650 | 628981 | 5514939 | 1 | 308.59 | Local |
| F4 | 01/23/2012 0:00 | 12 | 49.770900 | -109.211340 | 628793 | 5514694 | 1 | 7.92 | Local |
| F4 | 01/23/2012 0:00 | 15 | 49.770970 | -109.211320 | 628794 | 5514702 | 1 | 608.03 | Local |
| F4 | 01/23/2012 0:00 | 18 | 49.765510 | -109.210870 | 628841 | 5514095 | 1 | 2329.12 | Local |
| F4 | 01/23/2012 0:00 | 21 | 49.785690 | -109.202210 | 629411 | 5516354 | 1 | 2.43 | Local |
| F4 | 01/24/2012 0:00 | 0 | 49.785700 | -109.202180 | 629413 | 5516355 | 1 | 78.07 | Local |
| F4 | 01/24/2012 0:00 | 3 | 49.786390 | -109.202380 | 629397 | 5516431 | 1 | 2093.62 | Local |
| F4 | 01/24/2012 0:00 | 6 | 49.797310 | -109.178690 | 631072 | 5517686 | 1 | 821.48 | Local |
| F4 | 01/24/2012 0:00 | 9 | 49.791270 | -109.185260 | 630616 | 5517004 | 1 | 1680.93 | Local |
| F4 | 01/24/2012 0:00 | 12 | 49.779660 | -109.200210 | 629571 | 5515687 | 1 | 4.32 | Local |
| F4 | 01/24/2012 0:00 | 15 | 49.779660 | -109.200270 | 629566 | 5515687 | 1 | 16.82 | Local |
| F4 | 01/24/2012 0:00 | 18 | 49.779810 | -109.200300 | 629564 | 5515703 | 1 | 13.25 | Local |
| F4 | 01/24/2012 0:00 | 21 | 49.779710 | -109.200200 | 629571 | 5515692 | 1 | 2395.38 | Local |
| F4 | 01/25/2012 0:00 | 0 | 49.796630 | -109.179610 | 631008 | 5517609 | 0 | 1241.68 | Local |
| F4 | 01/25/2012 0:00 | 6 | 49.790810 | -109.194330 | 629964 | 5516937 | 1 | 779.08 | Local |
| F4 | 01/25/2012 0:00 | 9 | 49.788990 | -109.204780 | 629217 | 5516716 | 1 | 79.76 | Local |
| F4 | 01/25/2012 0:00 | 12 | 49.789150 | -109.205860 | 629139 | 5516732 | 1 | 14.98 | Local |
| F4 | 01/25/2012 0:00 | 15 | 49.789260 | -109.205740 | 629147 | 5516745 | 1 | 211.24 | Local |
| F4 | 01/25/2012 0:00 | 18 | 49.789160 | -109.208670 | 628936 | 5516728 | 1 | 247.02 | Local |
| F4 | 01/25/2012 0:00 | 21 | 49.788680 | -109.212020 | 628697 | 5516669 | 1 | 353.85 | Local |
| F4 | 01/26/2012 0:00 | 0 | 49.785800 | -109.214110 | 628554 | 5516346 | 1 | 3.10 | Local |
| F4 | 01/26/2012 0:00 | 3 | 49.785820 | -109.214080 | 628556 | 5516348 | 1 | 9.89 | Local |
| F4 | 01/26/2012 0:00 | 6 | 49.785900 | -109.214140 | 628551 | 5516357 | 1 | 1451.13 | Local |
| F4 | 01/26/2012 0:00 | 9 | 49.775000 | -109.203060 | 629378 | 5515164 | 1 | 999.05 | Local |
| F4 | 01/26/2012 0:00 | 12 | 49.766230 | -109.206070 | 629185 | 5514184 | 1 | 449.54 | Local |
| F4 | 01/26/2012 0:00 | 15 | 49.762200 | -109.206560 | 629160 | 5513735 | 1 | 26.00 | Local |
| F4 | 01/26/2012 0:00 | 18 | 49.762070 | -109.206260 | 629182 | 5513721 | 1 | 559.08 | Local |
| F4 | 01/26/2012 0:00 | 21 | 49.761980 | -109.198500 | 629741 | 5513724 | 1 | 27.21 | Local |
| F4 | 01/27/2012 0:00 | 0 | 49.761850 | -109.198820 | 629718 | 5513709 | 1 | 1.11 | Local |
| F4 | 01/27/2012 0:00 | 3 | 49.761840 | -109.198820 | 629718 | 5513708 | 0 | 332.50 | Local |
| F4 | 01/27/2012 0:00 | 9 | 49.762560 | -109.203300 | 629394 | 5513781 | 1 | 1944.51 | Local |
| F4 | 01/27/2012 0:00 | 12 | 49.774270 | -109.223350 | 627919 | 5515048 | 0 | 1248.21 | Local |
| F4 | 01/28/2012 0:00 | 9 | 49.763420 | -109.227790 | 627628 | 5513834 | 1 | 467.11 | Local |
| F4 | 01/28/2012 0:00 | 12 | 49.766570 | -109.232080 | 627311 | 5514177 | 0 | 2344.03 | Local |
| F4 | 01/28/2012 0:00 | 21 | 49.783340 | -109.251800 | 625847 | 5516008 | 1 | 1202.65 | Local |
| F4 | 01/29/2012 0:00 | 0 | 49.781580 | -109.268280 | 624666 | 5515785 | 1 | 2224.14 | Local |
| F4 | 01/29/2012 0:00 | 3 | 49.762650 | -109.258310 | 625432 | 5513697 | 1 | 1532.95 | Local |
| F4 | 01/29/2012 0:00 | 6 | 49.751570 | -109.245650 | 626373 | 5512487 | 1 | 913.42 | Local |
| F4 | 01/29/2012 0:00 | 9 | 49.759110 | -109.240620 | 626715 | 5513333 | 0 | 13.42 | Local |
| F4 | 01/29/2012 0:00 | 15 | 49.759230 | -109.240640 | 626713 | 5513347 | 1 | 678.86 | Local |
| F4 | 01/29/2012 0:00 | 18 | 49.757770 | -109.231490 | 627376 | 5513200 | 1 | 2064.64 | Local |
| F4 | 01/29/2012 0:00 | 21 | 49.739890 | -109.223770 | 627979 | 5511225 | 1 | 2041.34 | Local |
| F4 | 01/30/2012 0:00 | 0 | 49.734510 | -109.196690 | 629945 | 5510674 | 1 | 917.45 | Local |
| F4 | 01/30/2012 0:00 | 3 | 49.742130 | -109.191810 | 630276 | 5511529 | 1 | 1251.12 | Local |
| F4 | 01/30/2012 0:00 | 6 | 49.737970 | -109.175680 | 631449 | 5511095 | 1 | 1090.04 | Local |
| F4 | 01/30/2012 0:00 | 9 | 49.747500 | -109.172140 | 631679 | 5512161 | 1 | 59.87 | Local |
| F4 | 01/30/2012 0:00 | 12 | 49.747930 | -109.171640 | 631713 | 5512209 | 1 | 9.35 | Local |

| | | | | | | | | | |
|---|---|---|---|---|---|---|---|---|---|
| F4 | 01/30/2012 0:00 | 15 | 49.748010 | -109.171680 | 631710 | 5512218 | 1 | 441.91 | Local |
| F4 | 01/30/2012 0:00 | 18 | 49.748920 | -109.177650 | 631278 | 5512309 | 1 | 312.93 | Local |
| F4 | 01/30/2012 0:00 | 21 | 49.748030 | -109.173530 | 631577 | 5512217 | 1 | 1167.61 | Local |
| F4 | 01/31/2012 0:00 | 0 | 49.758520 | -109.174230 | 631498 | 5513382 | 1 | 653.89 | Local |
| F4 | 01/31/2012 0:00 | 3 | 49.762550 | -109.180840 | 631011 | 5513818 | 0 | 229.89 | Local |
| F4 | 01/31/2012 0:00 | 9 | 49.761570 | -109.178030 | 631216 | 5513714 | 0 | 11.33 | Local |
| F4 | 01/31/2012 0:00 | 15 | 49.761470 | -109.178000 | 631219 | 5513703 | 1 | 6.83 | Local |
| F4 | 01/31/2012 0:00 | 18 | 49.761530 | -109.178020 | 631217 | 5513710 | 1 | 1440.27 | Local |
| F4 | 01/31/2012 0:00 | 21 | 49.771670 | -109.190460 | 630294 | 5514816 | 1 | 1362.23 | Local |
| F4 | 02/01/2012 0:00 | 0 | 49.783800 | -109.193100 | 630071 | 5516159 | 1 | 2250.26 | Local |
| F4 | 02/01/2012 0:00 | 3 | 49.803950 | -109.195970 | 629811 | 5518395 | 1 | 475.98 | Local |
| F4 | 02/01/2012 0:00 | 6 | 49.802570 | -109.189710 | 630265 | 5518252 | 1 | 76.29 | Local |
| F4 | 02/01/2012 0:00 | 9 | 49.802120 | -109.190510 | 630209 | 5518201 | 1 | 1379.49 | Local |
| F4 | 02/01/2012 0:00 | 12 | 49.790210 | -109.195870 | 629855 | 5516867 | 1 | 2.22 | Local |
| F4 | 02/01/2012 0:00 | 15 | 49.790190 | -109.195870 | 629855 | 5516865 | 1 | 7.92 | Local |
| F4 | 02/01/2012 0:00 | 18 | 49.790260 | -109.195850 | 629856 | 5516873 | 1 | 353.44 | Local |
| F4 | 02/01/2012 0:00 | 21 | 49.792960 | -109.193260 | 630035 | 5517178 | 1 | 603.84 | Local |
| F4 | 02/02/2012 0:00 | 0 | 49.798390 | -109.193300 | 630018 | 5517781 | 1 | 517.71 | Local |
| F4 | 02/02/2012 0:00 | 3 | 49.802860 | -109.191290 | 630151 | 5518282 | 1 | 65.46 | Local |
| F4 | 02/02/2012 0:00 | 6 | 49.803360 | -109.190810 | 630184 | 5518338 | 1 | 800.18 | Local |
| F4 | 02/02/2012 0:00 | 9 | 49.796220 | -109.192190 | 630104 | 5517542 | 1 | 366.28 | Local |
| F4 | 02/02/2012 0:00 | 12 | 49.793010 | -109.193330 | 630030 | 5517183 | 0 | 22.80 | Local |
| F4 | 02/02/2012 0:00 | 18 | 49.792810 | -109.193260 | 630036 | 5517161 | 0 | 388.14 | Local |
| F4 | 02/03/2012 0:00 | 0 | 49.790360 | -109.189420 | 630319 | 5516895 | 1 | 2062.96 | Local |
| F4 | 02/03/2012 0:00 | 3 | 49.772440 | -109.196830 | 629833 | 5514890 | 1 | 988.77 | Local |
| F4 | 02/03/2012 0:00 | 6 | 49.765070 | -109.204510 | 629300 | 5514057 | 1 | 134.44 | Local |
| F4 | 02/03/2012 0:00 | 9 | 49.765170 | -109.206370 | 629166 | 5514065 | 1 | 149.20 | Local |
| F4 | 02/03/2012 0:00 | 12 | 49.766060 | -109.207920 | 629052 | 5514162 | 1 | 15.59 | Local |
| F4 | 02/03/2012 0:00 | 15 | 49.766200 | -109.207930 | 629051 | 5514177 | 1 | 1202.87 | Local |
| F4 | 02/03/2012 0:00 | 18 | 49.755480 | -109.205700 | 629240 | 5512989 | 1 | 2009.78 | Local |
| F4 | 02/03/2012 0:00 | 21 | 49.746960 | -109.230300 | 627490 | 5512000 | 1 | 20.03 | Local |
| F4 | 02/04/2012 0:00 | 0 | 49.746780 | -109.230310 | 627490 | 5511980 | 1 | 1064.17 | Local |
| F4 | 02/04/2012 0:00 | 3 | 49.750690 | -109.243790 | 626509 | 5512392 | 1 | 1374.25 | Local |
| F4 | 02/04/2012 0:00 | 6 | 49.754760 | -109.261800 | 625201 | 5512814 | 1 | 1309.06 | Local |
| F4 | 02/04/2012 0:00 | 9 | 49.766420 | -109.259300 | 625351 | 5514115 | 1 | 661.02 | Local |
| F4 | 02/04/2012 0:00 | 12 | 49.769390 | -109.251350 | 625916 | 5514458 | 0 | 386.95 | Local |
| F4 | 02/04/2012 0:00 | 18 | 49.770630 | -109.246330 | 626274 | 5514604 | 0 | 5085.16 | Local |
| F4 | 02/05/2012 0:00 | 0 | 49.816240 | -109.241240 | 626522 | 5519684 | 1 | 228.69 | Local |
| F4 | 02/05/2012 0:00 | 3 | 49.816840 | -109.244280 | 626301 | 5519745 | 1 | 13.44 | Local |
| F4 | 02/05/2012 0:00 | 6 | 49.816760 | -109.244140 | 626312 | 5519736 | 1 | 18.31 | Local |
| F4 | 02/05/2012 0:00 | 9 | 49.816920 | -109.244080 | 626316 | 5519754 | 1 | 5.46 | Local |
| F4 | 02/05/2012 0:00 | 12 | 49.816890 | -109.244140 | 626311 | 5519751 | 1 | 6.62 | Local |
| F4 | 02/05/2012 0:00 | 15 | 49.816840 | -109.244190 | 626308 | 5519745 | 1 | 5.61 | Local |
| F4 | 02/05/2012 0:00 | 18 | 49.816790 | -109.244180 | 626309 | 5519740 | 1 | 18.70 | Local |
| F4 | 02/05/2012 0:00 | 21 | 49.816950 | -109.244100 | 626314 | 5519758 | 1 | 10.24 | Local |
| F4 | 02/06/2012 0:00 | 0 | 49.816860 | -109.244070 | 626316 | 5519748 | 1 | 2.65 | Local |
| F4 | 02/06/2012 0:00 | 3 | 49.816880 | -109.244050 | 626318 | 5519750 | 1 | 16.30 | Local |
| F4 | 02/06/2012 0:00 | 6 | 49.816760 | -109.244180 | 626309 | 5519736 | 1 | 3.64 | Local |
| F4 | 02/06/2012 0:00 | 9 | 49.816780 | -109.244140 | 626312 | 5519739 | 1 | 3.77 | Local |
| F4 | 02/06/2012 0:00 | 12 | 49.816770 | -109.244190 | 626308 | 5519737 | 1 | 3.34 | Local |
| F4 | 02/06/2012 0:00 | 15 | 49.816800 | -109.244190 | 626308 | 5519741 | 1 | 2.34 | Local |
| F4 | 02/06/2012 0:00 | 18 | 49.816780 | -109.244200 | 626307 | 5519739 | 1 | 71.92 | Local |
| F4 | 02/06/2012 0:00 | 21 | 49.816240 | -109.244750 | 626269 | 5519678 | 0 | 84.13 | Local |
| F4 | 02/07/2012 0:00 | 3 | 49.816860 | -109.244080 | 626316 | 5519748 | 1 | 3.34 | Local |
| F4 | 02/07/2012 0:00 | 6 | 49.816890 | -109.244080 | 626316 | 5519751 | 1 | 6.48 | Local |

| | | | | | | | | | |
|---|---|---|---|---|---|---|---|---|---|
| F4 | 02/07/2012 0:00 | 9 | 49.816890 | -109.244170 | 626309 | 5519751 | 1 | 4.91 | Local |
| F4 | 02/07/2012 0:00 | 12 | 49.816860 | -109.244120 | 626313 | 5519748 | 1 | 17.89 | Local |
| F4 | 02/07/2012 0:00 | 15 | 49.816710 | -109.244030 | 626320 | 5519731 | 1 | 12.40 | Local |
| F4 | 02/07/2012 0:00 | 18 | 49.816790 | -109.244150 | 626311 | 5519740 | 1 | 11.20 | Local |
| F4 | 02/07/2012 0:00 | 21 | 49.816880 | -109.244080 | 626316 | 5519750 | 1 | 10.79 | Local |
| F4 | 02/08/2012 0:00 | 0 | 49.816880 | -109.244230 | 626305 | 5519750 | 1 | 11.10 | Local |
| F4 | 02/08/2012 0:00 | 3 | 49.816810 | -109.244120 | 626313 | 5519742 | 1 | 152.34 | Local |
| F4 | 02/08/2012 0:00 | 6 | 49.815470 | -109.244560 | 626285 | 5519592 | 1 | 3157.42 | Local |
| F4 | 02/08/2012 0:00 | 9 | 49.787180 | -109.240820 | 626628 | 5516454 | 1 | 11.01 | Local |
| F4 | 02/08/2012 0:00 | 12 | 49.787260 | -109.240730 | 626634 | 5516463 | 1 | 410.56 | Local |
| F4 | 02/08/2012 0:00 | 15 | 49.785160 | -109.245420 | 626302 | 5516221 | 1 | 970.59 | Local |
| F4 | 02/08/2012 0:00 | 18 | 49.776860 | -109.249590 | 626023 | 5515291 | 1 | 1635.47 | Local |
| F4 | 02/08/2012 0:00 | 21 | 49.766600 | -109.233320 | 627221 | 5514178 | 1 | 2.43 | Local |
| F4 | 02/09/2012 0:00 | 0 | 49.766590 | -109.233350 | 627219 | 5514177 | 1 | 1134.02 | Local |
| F4 | 02/09/2012 0:00 | 3 | 49.757670 | -109.225720 | 627792 | 5513198 | 1 | 508.01 | Local |
| F4 | 02/09/2012 0:00 | 6 | 49.755530 | -109.231950 | 627349 | 5512950 | 1 | 64.93 | Local |
| F4 | 02/09/2012 0:00 | 9 | 49.755500 | -109.232850 | 627284 | 5512945 | 1 | 3.09 | Local |
| F4 | 02/09/2012 0:00 | 12 | 49.755490 | -109.232810 | 627287 | 5512944 | 0 | 1085.20 | Local |
| F4 | 02/09/2012 0:00 | 18 | 49.746210 | -109.237470 | 626976 | 5511905 | 1 | 1889.35 | Local |
| F4 | 02/09/2012 0:00 | 21 | 49.757490 | -109.257080 | 625534 | 5513126 | 1 | 1128.86 | Local |
| F4 | 02/10/2012 0:00 | 0 | 49.764400 | -109.268560 | 624689 | 5513875 | 1 | 8.93 | Local |
| F4 | 02/10/2012 0:00 | 3 | 49.764380 | -109.268680 | 624681 | 5513872 | 1 | 1.32 | Local |
| F4 | 02/10/2012 0:00 | 6 | 49.764390 | -109.268690 | 624680 | 5513873 | 1 | 1512.40 | Local |
| F4 | 02/10/2012 0:00 | 9 | 49.766470 | -109.247940 | 626169 | 5514139 | 1 | 469.46 | Local |
| F4 | 02/10/2012 0:00 | 12 | 49.769900 | -109.244140 | 626434 | 5514527 | 1 | 518.44 | Local |
| F4 | 02/10/2012 0:00 | 15 | 49.765240 | -109.244360 | 626430 | 5514008 | 1 | 1432.56 | Local |
| F4 | 02/10/2012 0:00 | 18 | 49.760680 | -109.225760 | 627781 | 5513533 | 1 | 3178.92 | Local |
| F4 | 02/10/2012 0:00 | 21 | 49.787110 | -109.208940 | 628922 | 5516500 | 1 | 1670.01 | Local |
| F4 | 02/11/2012 0:00 | 0 | 49.799690 | -109.196270 | 629801 | 5517920 | 1 | 3070.48 | Local |
| F4 | 02/11/2012 0:00 | 3 | 49.786000 | -109.159230 | 632503 | 5516463 | 1 | 20.83 | Local |
| F4 | 02/11/2012 0:00 | 6 | 49.785820 | -109.159150 | 632510 | 5516443 | 1 | 904.29 | Local |
| F4 | 02/11/2012 0:00 | 9 | 49.778460 | -109.153810 | 632914 | 5515635 | 1 | 618.97 | Local |
| F4 | 02/11/2012 0:00 | 12 | 49.774500 | -109.147770 | 633360 | 5515205 | 0 | 1290.61 | Local |
| F4 | 02/11/2012 0:00 | 18 | 49.766780 | -109.134390 | 634345 | 5514371 | 1 | 3.41 | Local |
| F4 | 02/11/2012 0:00 | 21 | 49.766750 | -109.134380 | 634345 | 5514367 | 1 | 3.41 | Local |
| F4 | 02/12/2012 0:00 | 0 | 49.766780 | -109.134370 | 634346 | 5514371 | 1 | 8.58 | Local |
| F4 | 02/12/2012 0:00 | 3 | 49.766710 | -109.134420 | 634343 | 5514363 | 1 | 2.43 | Local |
| F4 | 02/12/2012 0:00 | 6 | 49.766720 | -109.134390 | 634345 | 5514364 | 1 | 3.77 | Local |
| F4 | 02/12/2012 0:00 | 9 | 49.766730 | -109.134440 | 634341 | 5514365 | 0 | 19.36 | Local |
| F4 | 02/12/2012 0:00 | 15 | 49.766830 | -109.134220 | 634357 | 5514377 | 1 | 33.89 | Local |
| F4 | 02/12/2012 0:00 | 18 | 49.766590 | -109.134510 | 634336 | 5514349 | 1 | 12.52 | Local |
| F4 | 02/12/2012 0:00 | 21 | 49.766690 | -109.134430 | 634342 | 5514361 | 1 | 9.43 | Local |
| F4 | 02/13/2012 0:00 | 0 | 49.766700 | -109.134300 | 634351 | 5514362 | 1 | 6.62 | Local |
| F4 | 02/13/2012 0:00 | 3 | 49.766750 | -109.134350 | 634348 | 5514368 | 1 | 14.77 | Local |
| F4 | 02/13/2012 0:00 | 6 | 49.766700 | -109.134540 | 634334 | 5514362 | 1 | 7.95 | Local |
| F4 | 02/13/2012 0:00 | 9 | 49.766640 | -109.134480 | 634338 | 5514355 | 1 | 31.05 | Local |
| F4 | 02/13/2012 0:00 | 12 | 49.766910 | -109.134370 | 634346 | 5514385 | 0 | 21.80 | Local |
| F4 | 02/13/2012 0:00 | 18 | 49.766730 | -109.134490 | 634338 | 5514365 | 1 | 11.45 | Local |
| F4 | 02/13/2012 0:00 | 21 | 49.766650 | -109.134390 | 634345 | 5514356 | 1 | 6.48 | Local |
| F4 | 02/14/2012 0:00 | 0 | 49.766650 | -109.134300 | 634351 | 5514356 | 1 | 10.61 | Local |
| F4 | 02/14/2012 0:00 | 3 | 49.766720 | -109.134400 | 634344 | 5514364 | 1 | 2.16 | Local |
| F4 | 02/14/2012 0:00 | 6 | 49.766720 | -109.134370 | 634346 | 5514364 | 1 | 10.13 | Local |
| F4 | 02/14/2012 0:00 | 9 | 49.766790 | -109.134460 | 634340 | 5514372 | 1 | 4.41 | Local |
| F4 | 02/14/2012 0:00 | 12 | 49.766760 | -109.134420 | 634342 | 5514369 | 1 | 4.51 | Local |
| F4 | 02/14/2012 0:00 | 15 | 49.766720 | -109.134430 | 634342 | 5514364 | 1 | 3.10 | Local |

| | | | | | | | | | |
|---|---|---|---|---|---|---|---|---|---|
| F4 | 02/14/2012 0:00 | 18 | 49.766700 | -109.134460 | 634340 | 5514362 | 1 | 4.46 | Local |
| F4 | 02/14/2012 0:00 | 21 | 49.766690 | -109.134400 | 634344 | 5514361 | 1 | 10.03 | Local |
| F4 | 02/15/2012 0:00 | 0 | 49.766780 | -109.134410 | 634343 | 5514371 | 1 | 3.63 | Local |
| F4 | 02/15/2012 0:00 | 3 | 49.766750 | -109.134390 | 634345 | 5514367 | 1 | 5.61 | Local |
| F4 | 02/15/2012 0:00 | 6 | 49.766700 | -109.134400 | 634344 | 5514362 | 1 | 8.82 | Local |
| F4 | 02/15/2012 0:00 | 9 | 49.766760 | -109.134480 | 634338 | 5514368 | 1 | 3.60 | Local |
| F4 | 02/15/2012 0:00 | 12 | 49.766760 | -109.134430 | 634342 | 5514368 | 1 | 5.72 | Local |
| F4 | 02/15/2012 0:00 | 15 | 49.766800 | -109.134380 | 634345 | 5514373 | 1 | 8.93 | Local |
| F4 | 02/15/2012 0:00 | 18 | 49.766720 | -109.134390 | 634345 | 5514364 | 1 | 7.82 | Local |
| F4 | 02/15/2012 0:00 | 21 | 49.766790 | -109.134380 | 634345 | 5514372 | 1 | 7.58 | Local |
| F4 | 02/16/2012 0:00 | 0 | 49.766730 | -109.134330 | 634349 | 5514365 | 1 | 10.90 | Local |
| F4 | 02/16/2012 0:00 | 3 | 49.766820 | -109.134390 | 634344 | 5514375 | 1 | 5.72 | Local |
| F4 | 02/16/2012 0:00 | 6 | 49.766780 | -109.134440 | 634341 | 5514371 | 1 | 6.83 | Local |
| F4 | 02/16/2012 0:00 | 9 | 49.766720 | -109.134420 | 634343 | 5514364 | 1 | 0.00 | Local |
| F4 | 02/16/2012 0:00 | 12 | 49.766720 | -109.134420 | 634343 | 5514364 | 1 | 8.54 | Local |
| F4 | 02/16/2012 0:00 | 15 | 49.766670 | -109.134510 | 634336 | 5514358 | 1 | 1245.93 | Local |
| F4 | 02/16/2012 0:00 | 18 | 49.760190 | -109.148620 | 633338 | 5513613 | 1 | 2077.95 | Local |
| F4 | 02/16/2012 0:00 | 21 | 49.771680 | -109.171370 | 631668 | 5514850 | 1 | 1824.51 | Local |
| F4 | 02/17/2012 0:00 | 0 | 49.778820 | -109.194180 | 630007 | 5515604 | 1 | 11.01 | Local |
| F4 | 02/17/2012 0:00 | 3 | 49.778900 | -109.194090 | 630013 | 5515613 | 1 | 4.23 | Local |
| F4 | 02/17/2012 0:00 | 6 | 49.778920 | -109.194140 | 630010 | 5515615 | 1 | 1773.64 | Local |
| F4 | 02/17/2012 0:00 | 9 | 49.774580 | -109.217840 | 628315 | 5515092 | 1 | 1275.69 | Local |
| F4 | 02/17/2012 0:00 | 12 | 49.764020 | -109.224760 | 627845 | 5513906 | 1 | 523.83 | Local |
| F4 | 02/17/2012 0:00 | 15 | 49.760470 | -109.229540 | 627510 | 5513503 | 1 | 768.32 | Local |
| F4 | 02/17/2012 0:00 | 18 | 49.755590 | -109.237090 | 626979 | 5512948 | 1 | 1139.22 | Local |
| F4 | 02/17/2012 0:00 | 21 | 49.761840 | -109.249620 | 626060 | 5513622 | 1 | 18.02 | Local |
| F4 | 02/18/2012 0:00 | 0 | 49.761680 | -109.249580 | 626063 | 5513604 | 1 | 761.26 | Local |
| F4 | 02/18/2012 0:00 | 3 | 49.759730 | -109.259710 | 625339 | 5513370 | 0 | 4115.93 | Local |
| F4 | 02/18/2012 0:00 | 9 | 49.751440 | -109.315390 | 621350 | 5512357 | 1 | 3.98 | Local |
| F4 | 02/18/2012 0:00 | 12 | 49.751410 | -109.315420 | 621347 | 5512354 | 1 | 577.58 | Local |
| F4 | 02/18/2012 0:00 | 15 | 49.754140 | -109.308600 | 621832 | 5512668 | 1 | 1706.58 | Local |
| F4 | 02/18/2012 0:00 | 18 | 49.765440 | -109.292570 | 622958 | 5513951 | 1 | 2016.64 | Local |
| F4 | 02/18/2012 0:00 | 21 | 49.768210 | -109.264900 | 624943 | 5514304 | 1 | 1458.04 | Local |
| F4 | 02/19/2012 0:00 | 0 | 49.781230 | -109.262510 | 625082 | 5515756 | 1 | 1257.85 | Local |
| F4 | 02/19/2012 0:00 | 3 | 49.792300 | -109.266100 | 624795 | 5516980 | 1 | 1203.23 | Local |
| F4 | 02/19/2012 0:00 | 6 | 49.781580 | -109.268370 | 624659 | 5515785 | 1 | 1801.93 | Local |
| F4 | 02/19/2012 0:00 | 9 | 49.771920 | -109.248280 | 626130 | 5514745 | 1 | 10.80 | Local |
| F4 | 02/19/2012 0:00 | 12 | 49.771920 | -109.248430 | 626120 | 5514744 | 0 | 603.58 | Local |
| F4 | 02/19/2012 0:00 | 18 | 49.766540 | -109.249540 | 626054 | 5514144 | 1 | 2857.56 | Local |
| F4 | 02/19/2012 0:00 | 21 | 49.788130 | -109.228020 | 627546 | 5516581 | 1 | 436.11 | Local |
| F4 | 02/20/2012 0:00 | 0 | 49.791750 | -109.230350 | 627369 | 5516979 | 1 | 2133.03 | Local |
| F4 | 02/20/2012 0:00 | 3 | 49.809450 | -109.241770 | 626501 | 5518928 | 1 | 839.01 | Local |
| F4 | 02/20/2012 0:00 | 6 | 49.804670 | -109.232750 | 627163 | 5518412 | 1 | 2193.62 | Local |
| F4 | 02/20/2012 0:00 | 9 | 49.790820 | -109.211050 | 628761 | 5516909 | 1 | 73.39 | Local |
| F4 | 02/20/2012 0:00 | 12 | 49.790510 | -109.210150 | 628826 | 5516876 | 1 | 6.67 | Local |
| F4 | 02/20/2012 0:00 | 15 | 49.790450 | -109.210150 | 628827 | 5516869 | 1 | 384.27 | Local |
| F4 | 02/20/2012 0:00 | 18 | 49.793510 | -109.212630 | 628640 | 5517205 | 1 | 1356.69 | Local |
| F4 | 02/20/2012 0:00 | 21 | 49.798750 | -109.195610 | 629851 | 5517817 | 1 | 889.36 | Local |
| F4 | 02/21/2012 0:00 | 0 | 49.793560 | -109.186210 | 630541 | 5517256 | 1 | 508.01 | Local |
| F4 | 02/21/2012 0:00 | 3 | 49.790430 | -109.181070 | 630920 | 5516917 | 1 | 7.27 | Local |
| F4 | 02/21/2012 0:00 | 6 | 49.790370 | -109.181110 | 630917 | 5516911 | 1 | 868.49 | Local |
| F4 | 02/21/2012 0:00 | 9 | 49.789400 | -109.193080 | 630058 | 5516782 | 1 | 1310.11 | Local |
| F4 | 02/21/2012 0:00 | 12 | 49.779890 | -109.203820 | 629310 | 5515706 | 1 | 13.02 | Local |
| F4 | 02/21/2012 0:00 | 15 | 49.779850 | -109.203650 | 629323 | 5515702 | 1 | 390.95 | Local |
| F4 | 02/21/2012 0:00 | 18 | 49.776800 | -109.200950 | 629525 | 5515368 | 1 | 8.36 | Local |

| | | | | | | | | | |
|---|---|---|---|---|---|---|---|---|---|
| F4 | 02/21/2012 0:00 | 21 | 49.776860 | -109.200880 | 629530 | 5515374 | 1 | 25.69 | Local |
| F4 | 02/22/2012 0:00 | 0 | 49.776790 | -109.201220 | 629506 | 5515366 | 1 | 249.41 | Local |
| F4 | 02/22/2012 0:00 | 3 | 49.778110 | -109.198420 | 629704 | 5515518 | 1 | 354.59 | Local |
| F4 | 02/22/2012 0:00 | 6 | 49.780130 | -109.202230 | 629424 | 5515736 | 1 | 1031.05 | Local |
| F4 | 02/22/2012 0:00 | 9 | 49.782330 | -109.216140 | 628417 | 5515956 | 1 | 1069.90 | Local |
| F4 | 02/22/2012 0:00 | 12 | 49.780090 | -109.201690 | 629463 | 5515732 | 1 | 20.10 | Local |
| F4 | 02/22/2012 0:00 | 15 | 49.779930 | -109.201820 | 629454 | 5515714 | 1 | 1346.75 | Local |
| F4 | 02/22/2012 0:00 | 18 | 49.772110 | -109.216100 | 628447 | 5514820 | 1 | 1167.64 | Local |
| F4 | 02/22/2012 0:00 | 21 | 49.782610 | -109.216170 | 628414 | 5515987 | 1 | 1125.45 | Local |
| F4 | 02/23/2012 0:00 | 0 | 49.792540 | -109.213150 | 628605 | 5517097 | 1 | 625.80 | Local |
| F4 | 02/23/2012 0:00 | 3 | 49.798160 | -109.213600 | 628558 | 5517721 | 1 | 810.76 | Local |
| F4 | 02/23/2012 0:00 | 6 | 49.796180 | -109.224440 | 627783 | 5517482 | 1 | 1180.87 | Local |
| F4 | 02/23/2012 0:00 | 9 | 49.790710 | -109.210380 | 628809 | 5516898 | 1 | 35.96 | Local |
| F4 | 02/23/2012 0:00 | 12 | 49.790430 | -109.210130 | 628828 | 5516867 | 1 | 5.30 | Local |
| F4 | 02/23/2012 0:00 | 15 | 49.790470 | -109.210170 | 628825 | 5516872 | 1 | 1.82 | Local |
| F4 | 02/23/2012 0:00 | 18 | 49.790460 | -109.210150 | 628827 | 5516870 | 1 | 806.61 | Local |
| F4 | 02/23/2012 0:00 | 21 | 49.793490 | -109.220330 | 628086 | 5517190 | 1 | 1408.87 | Local |
| F4 | 02/24/2012 0:00 | 0 | 49.788720 | -109.238460 | 626794 | 5516629 | 1 | 1.44 | Local |
| F4 | 02/24/2012 0:00 | 3 | 49.788720 | -109.238440 | 626795 | 5516629 | 1 | 891.98 | Local |
| F4 | 02/24/2012 0:00 | 6 | 49.792830 | -109.249080 | 626018 | 5517068 | 1 | 964.69 | Local |
| F4 | 02/24/2012 0:00 | 9 | 49.784160 | -109.248620 | 626074 | 5516105 | 1 | 428.38 | Local |
| F4 | 02/24/2012 0:00 | 12 | 49.782660 | -109.243140 | 626472 | 5515947 | 1 | 7.95 | Local |
| F4 | 02/24/2012 0:00 | 15 | 49.782720 | -109.243080 | 626477 | 5515954 | 1 | 112.54 | Local |
| F4 | 02/24/2012 0:00 | 18 | 49.781740 | -109.242690 | 626507 | 5515846 | 1 | 1244.04 | Local |
| F4 | 02/24/2012 0:00 | 21 | 49.792330 | -109.248260 | 626079 | 5517014 | 1 | 804.58 | Local |
| F4 | 02/25/2012 0:00 | 0 | 49.794310 | -109.259010 | 625300 | 5517216 | 1 | 1959.07 | Local |
| F4 | 02/25/2012 0:00 | 3 | 49.779100 | -109.272740 | 624351 | 5515502 | 1 | 1807.41 | Local |
| F4 | 02/25/2012 0:00 | 6 | 49.768340 | -109.253930 | 625733 | 5514337 | 1 | 12.32 | Local |
| F4 | 02/25/2012 0:00 | 9 | 49.768450 | -109.253950 | 625731 | 5514349 | 1 | 2.34 | Local |
| F4 | 02/25/2012 0:00 | 12 | 49.768430 | -109.253960 | 625730 | 5514347 | 0 | 2187.63 | Local |
| F4 | 02/26/2012 0:00 | 3 | 49.772000 | -109.224090 | 627872 | 5514794 | 0 | 1475.67 | Local |
| F4 | 02/26/2012 0:00 | 15 | 49.773310 | -109.244480 | 626400 | 5514905 | 1 | 881.72 | Local |
| F4 | 02/26/2012 0:00 | 18 | 49.773490 | -109.256720 | 625519 | 5514905 | 1 | 2658.82 | Local |
| F4 | 02/26/2012 0:00 | 21 | 49.793770 | -109.237160 | 626874 | 5517192 | 1 | 4.86 | Local |
| F4 | 02/27/2012 0:00 | 0 | 49.793750 | -109.237220 | 626870 | 5517190 | 1 | 1004.00 | Local |
| F4 | 02/27/2012 0:00 | 3 | 49.786010 | -109.230040 | 627407 | 5516342 | 1 | 176.82 | Local |
| F4 | 02/27/2012 0:00 | 6 | 49.784630 | -109.228820 | 627498 | 5516190 | 0 | 854.08 | Local |
| F4 | 02/27/2012 0:00 | 18 | 49.782600 | -109.217380 | 628327 | 5515984 | 1 | 1016.99 | Local |
| F4 | 02/27/2012 0:00 | 21 | 49.791500 | -109.214130 | 628537 | 5516979 | 1 | 5.74 | Local |
| F4 | 02/28/2012 0:00 | 0 | 49.791450 | -109.214110 | 628539 | 5516974 | 1 | 99.91 | Local |
| F4 | 02/28/2012 0:00 | 3 | 49.792150 | -109.214980 | 628474 | 5517050 | 1 | 407.22 | Local |
| F4 | 02/28/2012 0:00 | 6 | 49.794980 | -109.218570 | 628209 | 5517358 | 1 | 1757.53 | Local |
| F4 | 02/28/2012 0:00 | 9 | 49.779680 | -109.224690 | 627808 | 5515647 | 0 | 805.97 | Local |
| F4 | 02/29/2012 0:00 | 3 | 49.774920 | -109.233130 | 627213 | 5515104 | 1 | 2828.29 | Local |
| F4 | 02/29/2012 0:00 | 6 | 49.778650 | -109.194280 | 630000 | 5515585 | 0 | 2633.47 | Local |
| F4 | 02/29/2012 0:00 | 12 | 49.786620 | -109.159840 | 632458 | 5516531 | 1 | 1321.53 | Local |
| F4 | 02/29/2012 0:00 | 15 | 49.792220 | -109.143650 | 633608 | 5517182 | 1 | 693.60 | Local |
| F4 | 02/29/2012 0:00 | 18 | 49.786200 | -109.141130 | 633806 | 5516518 | 1 | 1391.13 | Local |
| F4 | 02/29/2012 0:00 | 21 | 49.781260 | -109.158880 | 632542 | 5515937 | 1 | 1353.83 | Local |
| F4 | 03/01/2012 0:00 | 0 | 49.782160 | -109.177630 | 631189 | 5516004 | 1 | 1850.79 | Local |
| F4 | 03/01/2012 0:00 | 3 | 49.779050 | -109.202880 | 629380 | 5515614 | 1 | 3.63 | Local |
| F4 | 03/01/2012 0:00 | 6 | 49.779080 | -109.202900 | 629379 | 5515618 | 1 | 2.22 | Local |
| F4 | 03/01/2012 0:00 | 9 | 49.779100 | -109.202900 | 629379 | 5515620 | 1 | 1300.95 | Local |
| F4 | 03/01/2012 0:00 | 12 | 49.776170 | -109.220390 | 628127 | 5515264 | 0 | 1348.59 | Local |
| F4 | 03/02/2012 0:00 | 9 | 49.766650 | -109.231990 | 627317 | 5514186 | 0 | 793.46 | Local |

| | | | | | | | | | |
|---|---|---|---|---|---|---|---|---|---|
| F4 | 03/03/2012 0:00 | 0 | 49.773590 | -109.234550 | 627115 | 5514953 | 1 | 2370.89 | Local |
| F4 | 03/03/2012 0:00 | 3 | 49.765380 | -109.264930 | 624948 | 5513990 | 1 | 2510.69 | Local |
| F4 | 03/03/2012 0:00 | 6 | 49.763890 | -109.230150 | 627457 | 5513882 | 1 | 2947.39 | Local |
| F4 | 03/03/2012 0:00 | 9 | 49.773310 | -109.191900 | 630186 | 5514995 | 1 | 709.48 | Local |
| F4 | 03/03/2012 0:00 | 12 | 49.778830 | -109.196840 | 629816 | 5515600 | 1 | 2.65 | Local |
| F4 | 03/03/2012 0:00 | 15 | 49.778810 | -109.196860 | 629814 | 5515598 | 1 | 1415.58 | Local |
| F4 | 03/03/2012 0:00 | 18 | 49.779790 | -109.216460 | 628401 | 5515673 | 1 | 1227.94 | Local |
| F4 | 03/03/2012 0:00 | 21 | 49.789950 | -109.209780 | 628854 | 5516814 | 1 | 376.85 | Local |
| F4 | 03/04/2012 0:00 | 0 | 49.792510 | -109.213210 | 628601 | 5517093 | 1 | 1543.83 | Local |
| F4 | 03/04/2012 0:00 | 3 | 49.779270 | -109.219660 | 628172 | 5515610 | 0 | 823.57 | Local |
| F4 | 03/04/2012 0:00 | 18 | 49.775820 | -109.229780 | 627452 | 5515209 | 1 | 2995.66 | Local |
| F4 | 03/04/2012 0:00 | 21 | 49.791170 | -109.263970 | 624951 | 5516858 | 1 | 760.87 | Local |
| F4 | 03/05/2012 0:00 | 0 | 49.797460 | -109.259810 | 625234 | 5517565 | 1 | 665.91 | Local |
| F4 | 03/05/2012 0:00 | 3 | 49.796970 | -109.250590 | 625899 | 5517525 | 1 | 216.64 | Local |
| F4 | 03/05/2012 0:00 | 6 | 49.798860 | -109.249860 | 625947 | 5517737 | 1 | 1223.32 | Local |
| F4 | 03/05/2012 0:00 | 9 | 49.788720 | -109.243270 | 626447 | 5516621 | 1 | 4.94 | Local |
| F4 | 03/05/2012 0:00 | 12 | 49.788680 | -109.243300 | 626445 | 5516616 | 0 | 3.10 | Local |
| F4 | 03/05/2012 0:00 | 18 | 49.788700 | -109.243330 | 626443 | 5516618 | 1 | 4.68 | Local |
| F4 | 03/05/2012 0:00 | 21 | 49.788660 | -109.243310 | 626445 | 5516614 | 1 | 1.32 | Local |
| F4 | 03/06/2012 0:00 | 0 | 49.788670 | -109.243300 | 626445 | 5516615 | 1 | 2980.93 | Local |
| F4 | 03/06/2012 0:00 | 3 | 49.815470 | -109.242400 | 626440 | 5519596 | 1 | 57.75 | Local |
| F4 | 03/06/2012 0:00 | 6 | 49.815040 | -109.242850 | 626409 | 5519547 | 1 | 9.62 | Local |
| F4 | 03/06/2012 0:00 | 9 | 49.815020 | -109.242980 | 626400 | 5519545 | 1 | 13.72 | Local |
| F4 | 03/06/2012 0:00 | 12 | 49.815030 | -109.242790 | 626413 | 5519546 | 1 | 9.89 | Local |
| F4 | 03/06/2012 0:00 | 15 | 49.814950 | -109.242850 | 626409 | 5519537 | 1 | 8.63 | Local |
| F4 | 03/06/2012 0:00 | 18 | 49.814950 | -109.242730 | 626418 | 5519538 | 1 | 9.81 | Local |
| F4 | 03/06/2012 0:00 | 21 | 49.815010 | -109.242830 | 626410 | 5519544 | 1 | 6.67 | Local |
| F4 | 03/07/2012 0:00 | 0 | 49.814950 | -109.242830 | 626411 | 5519537 | 0 | 5.56 | Local |
| F4 | 03/07/2012 0:00 | 6 | 49.815000 | -109.242830 | 626411 | 5519543 | 1 | 3644.87 | Local |
| F4 | 03/07/2012 0:00 | 9 | 49.782250 | -109.240780 | 626643 | 5515906 | 1 | 26.74 | Local |
| F4 | 03/07/2012 0:00 | 12 | 49.782470 | -109.240930 | 626632 | 5515930 | 1 | 2.65 | Local |
| F4 | 03/07/2012 0:00 | 15 | 49.782490 | -109.240950 | 626631 | 5515932 | 1 | 2.43 | Local |
| F4 | 03/07/2012 0:00 | 18 | 49.782500 | -109.240980 | 626628 | 5515933 | 1 | 1514.89 | Local |
| F4 | 03/07/2012 0:00 | 21 | 49.784790 | -109.220240 | 628115 | 5516223 | 1 | 664.59 | Local |
| F4 | 03/08/2012 0:00 | 0 | 49.789580 | -109.214720 | 628500 | 5516765 | 1 | 1521.03 | Local |
| F4 | 03/08/2012 0:00 | 3 | 49.791080 | -109.193720 | 630007 | 5516968 | 1 | 647.90 | Local |
| F4 | 03/08/2012 0:00 | 6 | 49.795150 | -109.200160 | 629533 | 5517409 | 1 | 1097.49 | Local |
| F4 | 03/08/2012 0:00 | 9 | 49.787330 | -109.209460 | 628884 | 5516524 | 1 | 424.86 | Local |
| F4 | 03/08/2012 0:00 | 12 | 49.784120 | -109.212660 | 628663 | 5516161 | 1 | 12.35 | Local |
| F4 | 03/08/2012 0:00 | 15 | 49.784080 | -109.212820 | 628651 | 5516157 | 1 | 10.89 | Local |
| F4 | 03/08/2012 0:00 | 18 | 49.784130 | -109.212690 | 628660 | 5516162 | 1 | 488.95 | Local |
| F4 | 03/08/2012 0:00 | 21 | 49.786510 | -109.218400 | 628243 | 5516417 | 1 | 552.16 | Local |
| F4 | 03/09/2012 0:00 | 0 | 49.790770 | -109.214460 | 628515 | 5516897 | 1 | 1853.85 | Local |
| F4 | 03/09/2012 0:00 | 3 | 49.803230 | -109.231570 | 627251 | 5518254 | 1 | 20.21 | Local |
| F4 | 03/09/2012 0:00 | 6 | 49.803280 | -109.231300 | 627271 | 5518260 | 1 | 124.67 | Local |
| F4 | 03/09/2012 0:00 | 9 | 49.802360 | -109.232290 | 627202 | 5518156 | 1 | 2.88 | Local |
| F4 | 03/09/2012 0:00 | 12 | 49.802360 | -109.232330 | 627199 | 5518156 | 1 | 1.44 | Local |
| F4 | 03/09/2012 0:00 | 15 | 49.802360 | -109.232310 | 627200 | 5518156 | 1 | 5.86 | Local |
| F4 | 03/09/2012 0:00 | 18 | 49.802370 | -109.232230 | 627206 | 5518157 | 1 | 10.36 | Local |
| F4 | 03/09/2012 0:00 | 21 | 49.802410 | -109.232360 | 627197 | 5518161 | 1 | 2.65 | Local |
| F4 | 03/10/2012 0:00 | 0 | 49.802390 | -109.232340 | 627198 | 5518159 | 1 | 6.04 | Local |
| F4 | 03/10/2012 0:00 | 3 | 49.802420 | -109.232270 | 627203 | 5518162 | 1 | 8.71 | Local |
| F4 | 03/10/2012 0:00 | 6 | 49.802430 | -109.232390 | 627194 | 5518163 | 1 | 1334.87 | Local |
| F4 | 03/10/2012 0:00 | 9 | 49.790620 | -109.235710 | 626986 | 5516845 | 1 | 13.42 | Local |
| F4 | 03/10/2012 0:00 | 12 | 49.790500 | -109.235730 | 626985 | 5516831 | 1 | 20.83 | Local |

| | | | | | | | | | |
|---|---|---|---|---|---|---|---|---|---|
| F4 | 03/10/2012 0:00 | 15 | 49.790680 | -109.235650 | 626991 | 5516851 | 1 | 7.58 | Local |
| F4 | 03/10/2012 0:00 | 18 | 49.790740 | -109.235600 | 626994 | 5516858 | 1 | 1109.27 | Local |
| F4 | 03/10/2012 0:00 | 21 | 49.786450 | -109.249510 | 626004 | 5516358 | 1 | 1634.35 | Local |
| F4 | 03/11/2012 0:00 | 0 | 49.786450 | -109.272210 | 624370 | 5516320 | 1 | 644.50 | Local |
| F4 | 03/11/2012 0:00 | 3 | 49.780660 | -109.271810 | 624414 | 5515677 | 1 | 2223.12 | Local |
| F4 | 03/11/2012 0:00 | 6 | 49.761360 | -109.263760 | 625043 | 5513545 | 1 | 2387.92 | Local |
| F4 | 03/11/2012 0:00 | 9 | 49.746230 | -109.240240 | 626776 | 5511902 | 1 | 230.84 | Local |
| F4 | 03/11/2012 0:00 | 12 | 49.747520 | -109.237730 | 626954 | 5512050 | 1 | 23.35 | Local |
| F4 | 03/11/2012 0:00 | 15 | 49.747730 | -109.237730 | 626953 | 5512073 | 1 | 172.48 | Local |
| F4 | 03/11/2012 0:00 | 18 | 49.748520 | -109.239790 | 626803 | 5512157 | 1 | 712.16 | Local |
| F4 | 03/11/2012 0:00 | 21 | 49.743730 | -109.233230 | 627288 | 5511636 | 1 | 1206.57 | Local |
| F4 | 03/12/2012 0:00 | 0 | 49.735950 | -109.221560 | 628149 | 5510791 | 1 | 1506.71 | Local |
| F4 | 03/12/2012 0:00 | 3 | 49.745260 | -109.206370 | 629219 | 5511852 | 1 | 4.46 | Local |
| F4 | 03/12/2012 0:00 | 6 | 49.745250 | -109.206430 | 629214 | 5511851 | 1 | 871.61 | Local |
| F4 | 03/12/2012 0:00 | 9 | 49.747660 | -109.217940 | 628379 | 5512099 | 1 | 5.97 | Local |
| F4 | 03/12/2012 0:00 | 12 | 49.747610 | -109.217970 | 628377 | 5512093 | 1 | 4.86 | Local |
| F4 | 03/12/2012 0:00 | 15 | 49.747590 | -109.217910 | 628381 | 5512091 | 1 | 11.33 | Local |
| F4 | 03/12/2012 0:00 | 18 | 49.747690 | -109.217940 | 628379 | 5512102 | 1 | 1177.33 | Local |
| F4 | 03/12/2012 0:00 | 21 | 49.754070 | -109.230980 | 627423 | 5512789 | 1 | 1870.11 | Local |
| F4 | 03/13/2012 0:00 | 0 | 49.770830 | -109.233120 | 627225 | 5514649 | 1 | 2275.31 | Local |
| F4 | 03/13/2012 0:00 | 3 | 49.786400 | -109.212620 | 628659 | 5516415 | 1 | 2646.84 | Local |
| F4 | 03/13/2012 0:00 | 6 | 49.805760 | -109.234010 | 627069 | 5518531 | 1 | 2.65 | Local |
| F4 | 03/13/2012 0:00 | 9 | 49.805740 | -109.234030 | 627068 | 5518528 | 1 | 1661.45 | Local |
| F4 | 03/13/2012 0:00 | 12 | 49.790830 | -109.235510 | 627000 | 5516868 | 0 | 279.80 | Local |
| F4 | 03/13/2012 0:00 | 21 | 49.790060 | -109.239210 | 626736 | 5516776 | 1 | 2262.24 | Local |
| F4 | 03/14/2012 0:00 | 0 | 49.809230 | -109.249730 | 625929 | 5518890 | 1 | 2031.08 | Local |
| F4 | 03/14/2012 0:00 | 3 | 49.827480 | -109.250860 | 625800 | 5520917 | 1 | 1425.75 | Local |
| F4 | 03/14/2012 0:00 | 6 | 49.816340 | -109.241050 | 626535 | 5519695 | 1 | 2898.73 | Local |
| F4 | 03/14/2012 0:00 | 9 | 49.790510 | -109.235630 | 626993 | 5516833 | 1 | 4.51 | Local |
| F4 | 03/14/2012 0:00 | 12 | 49.790550 | -109.235620 | 626993 | 5516837 | 1 | 16.30 | Local |
| F4 | 03/14/2012 0:00 | 15 | 49.790670 | -109.235750 | 626983 | 5516850 | 1 | 142.36 | Local |
| F4 | 03/14/2012 0:00 | 18 | 49.791290 | -109.237480 | 626857 | 5516916 | 1 | 587.47 | Local |
| F4 | 03/14/2012 0:00 | 21 | 49.792800 | -109.245300 | 626291 | 5517071 | 1 | 1860.99 | Local |
| F4 | 03/15/2012 0:00 | 0 | 49.794610 | -109.271000 | 624436 | 5517229 | 1 | 815.83 | Local |
| F4 | 03/15/2012 0:00 | 3 | 49.795080 | -109.259690 | 625249 | 5517300 | 1 | 2310.50 | Local |
| F4 | 03/15/2012 0:00 | 6 | 49.810550 | -109.238260 | 626751 | 5519056 | 1 | 72.18 | Local |
| F4 | 03/15/2012 0:00 | 9 | 49.810950 | -109.239050 | 626693 | 5519099 | 1 | 38.00 | Local |
| F4 | 03/15/2012 0:00 | 12 | 49.810840 | -109.238550 | 626729 | 5519088 | 1 | 15.56 | Local |
| F4 | 03/15/2012 0:00 | 15 | 49.810710 | -109.238630 | 626724 | 5519073 | 1 | 22.50 | Local |
| F4 | 03/15/2012 0:00 | 18 | 49.810620 | -109.238350 | 626744 | 5519064 | 1 | 33.88 | Local |
| F4 | 03/15/2012 0:00 | 21 | 49.810860 | -109.238640 | 626723 | 5519090 | 1 | 8.93 | Local |
| F4 | 03/16/2012 0:00 | 0 | 49.810940 | -109.238650 | 626722 | 5519099 | 1 | 13.52 | Local |
| F4 | 03/16/2012 0:00 | 3 | 49.810820 | -109.238620 | 626724 | 5519085 | 1 | 14.83 | Local |
| F4 | 03/16/2012 0:00 | 6 | 49.810940 | -109.238710 | 626717 | 5519099 | 1 | 4.23 | Local |
| F4 | 03/16/2012 0:00 | 9 | 49.810920 | -109.238760 | 626714 | 5519096 | 1 | 10.36 | Local |
| F4 | 03/16/2012 0:00 | 12 | 49.810880 | -109.238630 | 626723 | 5519092 | 1 | 17.65 | Local |
| F4 | 03/16/2012 0:00 | 15 | 49.810730 | -109.238550 | 626730 | 5519075 | 1 | 52.46 | Local |
| F4 | 03/16/2012 0:00 | 18 | 49.810560 | -109.237870 | 626779 | 5519058 | 1 | 559.23 | Local |
| F4 | 03/16/2012 0:00 | 21 | 49.815050 | -109.241370 | 626515 | 5519551 | 1 | 511.75 | Local |
| F4 | 03/17/2012 0:00 | 0 | 49.810790 | -109.238680 | 626720 | 5519082 | 1 | 5.74 | Local |
| F4 | 03/17/2012 0:00 | 3 | 49.810840 | -109.238700 | 626718 | 5519087 | 1 | 58.35 | Local |
| F4 | 03/17/2012 0:00 | 6 | 49.810380 | -109.238310 | 626748 | 5519037 | 0 | 49.28 | Local |
| F4 | 03/17/2012 0:00 | 12 | 49.810790 | -109.238570 | 626728 | 5519082 | 0 | 17.23 | Local |
| F4 | 03/17/2012 0:00 | 18 | 49.810940 | -109.238630 | 626723 | 5519099 | 1 | 15.09 | Local |
| F4 | 03/17/2012 0:00 | 21 | 49.810810 | -109.238690 | 626719 | 5519084 | 1 | 3.10 | Local |

| | | | | | | | | | | |
|---|---|---|---|---|---|---|---|---|---|---|
| F4 | 03/18/2012 0:00 | 0 | 49.810830 | -109.238660 | 626721 | 5519086 | 1 | 9.27 | Local |
| F4 | 03/18/2012 0:00 | 3 | 49.810900 | -109.238730 | 626716 | 5519094 | 0 | 7.20 | Local |
| F4 | 03/18/2012 0:00 | 9 | 49.810900 | -109.238630 | 626723 | 5519094 | 0 | 12.76 | Local |
| F4 | 03/18/2012 0:00 | 18 | 49.810990 | -109.238520 | 626731 | 5519104 | 1 | 152.62 | Local |
| F4 | 03/18/2012 0:00 | 21 | 49.811030 | -109.236400 | 626883 | 5519112 | 1 | 160.43 | Local |
| F4 | 03/19/2012 0:00 | 0 | 49.810840 | -109.238610 | 626725 | 5519088 | 1 | 10.85 | Local |
| F4 | 03/19/2012 0:00 | 3 | 49.810850 | -109.238460 | 626736 | 5519089 | 0 | 4.91 | Local |
| F4 | 03/19/2012 0:00 | 9 | 49.810820 | -109.238410 | 626739 | 5519086 | 0 | 7.93 | Local |
| F4 | 03/19/2012 0:00 | 21 | 49.810790 | -109.238510 | 626732 | 5519082 | 1 | 4.94 | Local |
| F4 | 03/20/2012 0:00 | 0 | 49.810830 | -109.238540 | 626730 | 5519087 | 1 | 3.10 | Local |
| F4 | 03/20/2012 0:00 | 3 | 49.810850 | -109.238570 | 626728 | 5519089 | 0 | 2732.02 | Local |
| F4 | 03/20/2012 0:00 | 9 | 49.786760 | -109.231120 | 627327 | 5516423 | 0 | 1018.67 | Local |
| F4 | 03/20/2012 0:00 | 21 | 49.795920 | -109.230980 | 627313 | 5517442 | 0 | 1751.60 | Local |
| F4 | 03/21/2012 0:00 | 3 | 49.810890 | -109.238550 | 626729 | 5519093 | 1 | 111.79 | Local |
| F4 | 03/21/2012 0:00 | 6 | 49.809900 | -109.238820 | 626712 | 5518983 | 1 | 3563.03 | Local |
| F4 | 03/21/2012 0:00 | 9 | 49.777920 | -109.235770 | 627015 | 5515433 | 1 | 42.48 | Local |
| F4 | 03/21/2012 0:00 | 12 | 49.778300 | -109.235710 | 627019 | 5515475 | 0 | 13.15 | Local |
| F4 | 03/21/2012 0:00 | 18 | 49.778320 | -109.235890 | 627006 | 5515477 | 0 | 431.34 | Local |
| F4 | 03/22/2012 0:00 | 18 | 49.774720 | -109.238120 | 626855 | 5515073 | 1 | 1612.57 | Local |
| F4 | 03/22/2012 0:00 | 21 | 49.774080 | -109.260490 | 625246 | 5514964 | 1 | 1913.44 | Local |
| F4 | 03/23/2012 0:00 | 0 | 49.791210 | -109.257980 | 625382 | 5516873 | 1 | 1743.19 | Local |
| F4 | 03/23/2012 0:00 | 3 | 49.805490 | -109.267970 | 624626 | 5518444 | 1 | 2173.94 | Local |
| F4 | 03/23/2012 0:00 | 6 | 49.814840 | -109.241440 | 626511 | 5519528 | 1 | 1056.89 | Local |
| F4 | 03/23/2012 0:00 | 9 | 49.806450 | -109.234540 | 627029 | 5518606 | 1 | 13.14 | Local |
| F4 | 03/23/2012 0:00 | 12 | 49.806430 | -109.234720 | 627016 | 5518604 | 1 | 14.62 | Local |
| F4 | 03/23/2012 0:00 | 15 | 49.806300 | -109.234690 | 627019 | 5518590 | 1 | 13.23 | Local |
| F4 | 03/23/2012 0:00 | 18 | 49.806410 | -109.234760 | 627014 | 5518602 | 1 | 1206.99 | Local |
| F4 | 03/23/2012 0:00 | 21 | 49.816210 | -109.241970 | 626469 | 5519679 | 1 | 466.83 | Local |
| F5 | 04/20/2011 0:00 | 21 | 49.648007 | -109.441003 | 612540 | 5500663 | 1 | 7092.66 | Trans |
| F5 | 04/21/2011 0:00 | 0 | 49.669597 | -109.348547 | 619161 | 5503205 | 1 | 3292.71 | Trans |
| F5 | 04/21/2011 0:00 | 3 | 49.687284 | -109.311949 | 621758 | 5505230 | 1 | 5283.92 | Trans |
| F5 | 04/21/2011 0:00 | 6 | 49.727820 | -109.350182 | 618901 | 5509676 | 1 | 27.94 | Trans |
| F5 | 04/21/2011 0:00 | 9 | 49.727642 | -109.350455 | 618882 | 5509655 | 1 | 10.01 | Trans |
| F5 | 04/21/2011 0:00 | 12 | 49.727628 | -109.350318 | 618892 | 5509654 | 1 | 15.37 | Trans |
| F5 | 04/21/2011 0:00 | 15 | 49.727691 | -109.350508 | 618878 | 5509661 | 1 | 2.20 | Trans |
| F5 | 04/21/2011 0:00 | 18 | 49.727672 | -109.350501 | 618878 | 5509659 | 1 | 18.12 | Trans |
| F5 | 04/21/2011 0:00 | 21 | 49.727511 | -109.350546 | 618876 | 5509641 | 1 | 30.71 | Trans |
| F5 | 04/22/2011 0:00 | 0 | 49.727732 | -109.350289 | 618894 | 5509666 | 1 | 869.25 | Trans |
| F5 | 04/22/2011 0:00 | 3 | 49.735545 | -109.350684 | 618846 | 5510533 | 1 | 1632.85 | Trans |
| F5 | 04/22/2011 0:00 | 6 | 49.743479 | -109.331619 | 620200 | 5511446 | 1 | 8.94 | Trans |
| F5 | 04/22/2011 0:00 | 9 | 49.743415 | -109.331543 | 620206 | 5511439 | 1 | 20.62 | Trans |
| F5 | 04/22/2011 0:00 | 12 | 49.743600 | -109.331566 | 620204 | 5511459 | 1 | 9.30 | Trans |
| F5 | 04/22/2011 0:00 | 15 | 49.743595 | -109.331437 | 620213 | 5511459 | 0 | 3976.54 | Trans |
| F5 | 04/22/2011 0:00 | 21 | 49.764125 | -109.286244 | 623417 | 5513815 | 1 | 2393.44 | Trans |
| F5 | 04/23/2011 0:00 | 0 | 49.771867 | -109.317251 | 621165 | 5514625 | 1 | 2622.98 | Trans |
| F5 | 04/23/2011 0:00 | 3 | 49.795272 | -109.321774 | 620781 | 5517220 | 1 | 3393.84 | Trans |
| F5 | 04/23/2011 0:00 | 6 | 49.825684 | -109.317815 | 620990 | 5520607 | 1 | 684.61 | Trans |
| F5 | 04/23/2011 0:00 | 9 | 49.819690 | -109.319987 | 620848 | 5519937 | 1 | 9.66 | Trans |
| F5 | 04/23/2011 0:00 | 12 | 49.819767 | -109.319924 | 620853 | 5519946 | 1 | 3.93 | Trans |
| F5 | 04/23/2011 0:00 | 15 | 49.819734 | -109.319945 | 620851 | 5519942 | 1 | 3.06 | Trans |
| F5 | 04/23/2011 0:00 | 18 | 49.819745 | -109.319984 | 620849 | 5519943 | 1 | 116.89 | Trans |
| F5 | 04/23/2011 0:00 | 21 | 49.820387 | -109.321270 | 620754 | 5520013 | 1 | 7477.04 | Trans |
| F5 | 04/24/2011 0:00 | 0 | 49.835355 | -109.422604 | 613431 | 5521518 | 1 | 3028.61 | Trans |
| F5 | 04/24/2011 0:00 | 3 | 49.817919 | -109.454948 | 611145 | 5519531 | 1 | 3464.73 | Trans |
| F5 | 04/24/2011 0:00 | 6 | 49.797374 | -109.491145 | 608587 | 5517194 | 1 | 161.74 | Trans |

| | | | | | | | | | |
|---|---|---|---|---|---|---|---|---|---|
| F5 | 04/24/2011 0:00 | 9 | 49.796202 | -109.489815 | 608685 | 5517066 | 1 | 1.40 | Trans |
| F5 | 04/24/2011 0:00 | 12 | 49.796193 | -109.489802 | 608686 | 5517065 | 1 | 8.79 | Trans |
| F5 | 04/24/2011 0:00 | 15 | 49.796258 | -109.489872 | 608681 | 5517072 | 0 | 257.94 | Trans |
| F5 | 04/24/2011 0:00 | 21 | 49.794551 | -109.492299 | 608510 | 5516879 | 1 | 4989.79 | Trans |
| F5 | 04/25/2011 0:00 | 0 | 49.750009 | -109.483888 | 609216 | 5511939 | 1 | 5422.99 | Local |
| F5 | 04/25/2011 0:00 | 3 | 49.703408 | -109.506075 | 607720 | 5506726 | 1 | 2516.35 | Local |
| F5 | 04/25/2011 0:00 | 6 | 49.687317 | -109.481544 | 609525 | 5504973 | 1 | 168.51 | Local |
| F5 | 04/25/2011 0:00 | 9 | 49.685983 | -109.480437 | 609608 | 5504826 | 1 | 4.53 | Local |
| F5 | 04/25/2011 0:00 | 12 | 49.685943 | -109.480444 | 609608 | 5504822 | 1 | 33.25 | Local |
| F5 | 04/25/2011 0:00 | 15 | 49.686007 | -109.479993 | 609640 | 5504830 | 1 | 193.41 | Local |
| F5 | 04/25/2011 0:00 | 18 | 49.685397 | -109.482504 | 609460 | 5504758 | 1 | 1096.45 | Local |
| F5 | 04/25/2011 0:00 | 21 | 49.680157 | -109.495378 | 608544 | 5504157 | 1 | 153.59 | Local |
| F5 | 04/26/2011 0:00 | 0 | 49.678779 | -109.495224 | 608558 | 5504004 | 1 | 52.19 | Local |
| F5 | 04/26/2011 0:00 | 3 | 49.678520 | -109.495827 | 608515 | 5503974 | 1 | 685.07 | Local |
| F5 | 04/26/2011 0:00 | 6 | 49.673199 | -109.491044 | 608872 | 5503390 | 1 | 4.08 | Local |
| F5 | 04/26/2011 0:00 | 9 | 49.673164 | -109.491061 | 608871 | 5503386 | 1 | 4.80 | Local |
| F5 | 04/26/2011 0:00 | 12 | 49.673121 | -109.491066 | 608870 | 5503381 | 1 | 11.68 | Local |
| F5 | 04/26/2011 0:00 | 15 | 49.673108 | -109.491227 | 608859 | 5503379 | 1 | 604.42 | Local |
| F5 | 04/26/2011 0:00 | 18 | 49.672310 | -109.499512 | 608263 | 5503279 | 1 | 391.19 | Local |
| F5 | 04/26/2011 0:00 | 21 | 49.669366 | -109.502479 | 608055 | 5502947 | 1 | 49.62 | Local |
| F5 | 04/27/2011 0:00 | 0 | 49.669812 | -109.502489 | 608053 | 5502997 | 1 | 123.25 | Local |
| F5 | 04/27/2011 0:00 | 3 | 49.669231 | -109.503943 | 607950 | 5502930 | 1 | 86.08 | Local |
| F5 | 04/27/2011 0:00 | 6 | 49.669918 | -109.503393 | 607988 | 5503007 | 1 | 78.36 | Local |
| F5 | 04/27/2011 0:00 | 9 | 49.669901 | -109.502308 | 608066 | 5503007 | 1 | 920.12 | Local |
| F5 | 04/27/2011 0:00 | 12 | 49.673039 | -109.490510 | 608911 | 5503373 | 1 | 17.46 | Local |
| F5 | 04/27/2011 0:00 | 15 | 49.673045 | -109.490752 | 608893 | 5503373 | 1 | 15.08 | Local |
| F5 | 04/27/2011 0:00 | 18 | 49.672967 | -109.490581 | 608906 | 5503364 | 1 | 457.61 | Local |
| F5 | 04/27/2011 0:00 | 21 | 49.671075 | -109.496212 | 608504 | 5503146 | 1 | 79.03 | Local |
| F5 | 04/28/2011 0:00 | 0 | 49.670520 | -109.496897 | 608455 | 5503083 | 1 | 25.62 | Local |
| F5 | 04/28/2011 0:00 | 3 | 49.670683 | -109.496646 | 608473 | 5503102 | 0 | 639.13 | Local |
| F5 | 04/28/2011 0:00 | 9 | 49.673840 | -109.489244 | 609000 | 5503463 | 1 | 7.18 | Local |
| F5 | 04/28/2011 0:00 | 12 | 49.673796 | -109.489318 | 608995 | 5503458 | 0 | 132.03 | Local |
| F5 | 04/28/2011 0:00 | 18 | 49.672722 | -109.490097 | 608941 | 5503338 | 0 | 528.68 | Local |
| F5 | 04/29/2011 0:00 | 0 | 49.670724 | -109.496745 | 608466 | 5503106 | 1 | 6.28 | Local |
| F5 | 04/29/2011 0:00 | 3 | 49.670763 | -109.496681 | 608470 | 5503111 | 0 | 153.77 | Local |
| F5 | 04/29/2011 0:00 | 9 | 49.671447 | -109.494829 | 608603 | 5503189 | 1 | 81.65 | Local |
| F5 | 04/29/2011 0:00 | 12 | 49.672176 | -109.494955 | 608592 | 5503270 | 1 | 18.97 | Local |
| F5 | 04/29/2011 0:00 | 15 | 49.672007 | -109.494926 | 608594 | 5503251 | 1 | 56.79 | Local |
| F5 | 04/29/2011 0:00 | 18 | 49.672207 | -109.495650 | 608542 | 5503273 | 1 | 566.82 | Local |
| F5 | 04/29/2011 0:00 | 21 | 49.669623 | -109.502421 | 608059 | 5502976 | 1 | 58.62 | Local |
| F5 | 04/30/2011 0:00 | 0 | 49.670129 | -109.502191 | 608074 | 5503032 | 1 | 56.96 | Local |
| F5 | 04/30/2011 0:00 | 3 | 49.669695 | -109.501772 | 608105 | 5502985 | 1 | 7.30 | Local |
| F5 | 04/30/2011 0:00 | 6 | 49.669644 | -109.501837 | 608101 | 5502979 | 1 | 1930.29 | Local |
| F5 | 04/30/2011 0:00 | 9 | 49.676977 | -109.477591 | 609834 | 5503829 | 1 | 51.19 | Local |
| F5 | 04/30/2011 0:00 | 12 | 49.677388 | -109.477271 | 609856 | 5503875 | 1 | 29.68 | Local |
| F5 | 04/30/2011 0:00 | 15 | 49.677123 | -109.477316 | 609853 | 5503846 | 1 | 8.40 | Local |
| F5 | 04/30/2011 0:00 | 18 | 49.677058 | -109.477377 | 609849 | 5503839 | 1 | 647.88 | Local |
| F5 | 04/30/2011 0:00 | 21 | 49.676143 | -109.486244 | 609211 | 5503724 | 1 | 967.76 | Local |
| F5 | 05/01/2011 0:00 | 0 | 49.670791 | -109.496820 | 608460 | 5503114 | 1 | 57.01 | Local |
| F5 | 05/01/2011 0:00 | 3 | 49.671270 | -109.496537 | 608480 | 5503167 | 1 | 68.89 | Local |
| F5 | 05/01/2011 0:00 | 6 | 49.671361 | -109.495592 | 608548 | 5503179 | 1 | 17.45 | Local |
| F5 | 05/01/2011 0:00 | 9 | 49.671205 | -109.495571 | 608550 | 5503161 | 0 | 14.08 | Local |
| F5 | 05/01/2011 0:00 | 15 | 49.671320 | -109.495652 | 608543 | 5503174 | 1 | 37.08 | Local |
| F5 | 05/01/2011 0:00 | 18 | 49.671602 | -109.495378 | 608563 | 5503206 | 1 | 29.95 | Local |
| F5 | 05/01/2011 0:00 | 21 | 49.671673 | -109.494978 | 608591 | 5503214 | 1 | 116.73 | Local |

| | | | | | | | | | |
|---|---|---|---|---|---|---|---|---|---|
| F5 | 05/02/2011 0:00 | 0 | 49.671376 | -109.496529 | 608480 | 5503179 | 1 | 28.08 | Local |
| F5 | 05/02/2011 0:00 | 3 | 49.671557 | -109.496258 | 608499 | 5503199 | 0 | 830.66 | Local |
| F5 | 05/02/2011 0:00 | 9 | 49.674806 | -109.485893 | 609240 | 5503576 | 1 | 4.83 | Local |
| F5 | 05/02/2011 0:00 | 12 | 49.674794 | -109.485828 | 609244 | 5503574 | 1 | 9.45 | Local |
| F5 | 05/02/2011 0:00 | 15 | 49.674851 | -109.485925 | 609237 | 5503581 | 1 | 144.79 | Local |
| F5 | 05/02/2011 0:00 | 18 | 49.673730 | -109.486946 | 609166 | 5503455 | 1 | 708.01 | Local |
| F5 | 05/02/2011 0:00 | 21 | 49.671686 | -109.496238 | 608500 | 5503214 | 1 | 73.72 | Local |
| F5 | 05/03/2011 0:00 | 0 | 49.671170 | -109.495596 | 608548 | 5503157 | 1 | 59.91 | Local |
| F5 | 05/03/2011 0:00 | 3 | 49.671487 | -109.496267 | 608499 | 5503192 | 1 | 9.90 | Local |
| F5 | 05/03/2011 0:00 | 6 | 49.671575 | -109.496288 | 608497 | 5503201 | 1 | 44.49 | Local |
| F5 | 05/03/2011 0:00 | 9 | 49.671280 | -109.495871 | 608528 | 5503169 | 1 | 27.17 | Local |
| F5 | 05/03/2011 0:00 | 12 | 49.671282 | -109.495495 | 608555 | 5503170 | 1 | 34.82 | Local |
| F5 | 05/03/2011 0:00 | 15 | 49.671487 | -109.495860 | 608528 | 5503192 | 1 | 26.54 | Local |
| F5 | 05/03/2011 0:00 | 18 | 49.671585 | -109.496195 | 608504 | 5503203 | 1 | 23.52 | Local |
| F5 | 05/03/2011 0:00 | 21 | 49.671379 | -109.496267 | 608499 | 5503180 | 1 | 64.78 | Local |
| F5 | 05/04/2011 0:00 | 0 | 49.671093 | -109.495485 | 608556 | 5503149 | 1 | 66.35 | Local |
| F5 | 05/04/2011 0:00 | 3 | 49.671583 | -109.496011 | 608517 | 5503203 | 1 | 12.57 | Local |
| F5 | 05/04/2011 0:00 | 6 | 49.671661 | -109.496136 | 608508 | 5503211 | 1 | 72.56 | Local |
| F5 | 05/04/2011 0:00 | 9 | 49.671191 | -109.495438 | 608559 | 5503160 | 1 | 5.15 | Local |
| F5 | 05/04/2011 0:00 | 12 | 49.671225 | -109.495487 | 608556 | 5503164 | 1 | 10.19 | Local |
| F5 | 05/04/2011 0:00 | 15 | 49.671257 | -109.495620 | 608546 | 5503167 | 1 | 56.58 | Local |
| F5 | 05/04/2011 0:00 | 18 | 49.671758 | -109.495755 | 608535 | 5503223 | 1 | 40.98 | Local |
| F5 | 05/04/2011 0:00 | 21 | 49.671691 | -109.496313 | 608495 | 5503214 | 1 | 17.01 | Local |
| F5 | 05/05/2011 0:00 | 0 | 49.671613 | -109.496110 | 608510 | 5503206 | 1 | 7.41 | Local |
| F5 | 05/05/2011 0:00 | 3 | 49.671547 | -109.496109 | 608510 | 5503199 | 1 | 5.90 | Local |
| F5 | 05/05/2011 0:00 | 6 | 49.671494 | -109.496104 | 608510 | 5503193 | 1 | 110.62 | Local |
| F5 | 05/05/2011 0:00 | 9 | 49.671340 | -109.494589 | 608620 | 5503178 | 1 | 11.19 | Local |
| F5 | 05/05/2011 0:00 | 12 | 49.671265 | -109.494693 | 608613 | 5503169 | 1 | 4.21 | Local |
| F5 | 05/05/2011 0:00 | 15 | 49.671294 | -109.494655 | 608615 | 5503173 | 1 | 0.70 | Local |
| F5 | 05/05/2011 0:00 | 18 | 49.671289 | -109.494648 | 608616 | 5503172 | 1 | 458.38 | Local |
| F5 | 05/05/2011 0:00 | 21 | 49.667680 | -109.497718 | 608402 | 5502766 | 1 | 24.50 | Local |
| F5 | 05/06/2011 0:00 | 0 | 49.667596 | -109.497405 | 608425 | 5502757 | 1 | 17.49 | Local |
| F5 | 05/06/2011 0:00 | 3 | 49.667555 | -109.497638 | 608408 | 5502753 | 1 | 1379.57 | Local |
| F5 | 05/06/2011 0:00 | 6 | 49.656530 | -109.488871 | 609066 | 5501540 | 1 | 274.67 | Local |
| F5 | 05/06/2011 0:00 | 9 | 49.654130 | -109.487969 | 609136 | 5501274 | 1 | 7.52 | Local |
| F5 | 05/06/2011 0:00 | 12 | 49.654063 | -109.487973 | 609136 | 5501267 | 1 | 28.36 | Local |
| F5 | 05/06/2011 0:00 | 15 | 49.653808 | -109.487938 | 609139 | 5501238 | 1 | 359.26 | Local |
| F5 | 05/06/2011 0:00 | 18 | 49.650611 | -109.488656 | 609094 | 5500882 | 1 | 1006.01 | Local |
| F5 | 05/06/2011 0:00 | 21 | 49.646965 | -109.501408 | 608182 | 5500458 | 1 | 578.42 | Local |
| F5 | 05/07/2011 0:00 | 0 | 49.651570 | -109.505135 | 607903 | 5500965 | 1 | 725.60 | Local |
| F5 | 05/07/2011 0:00 | 3 | 49.657752 | -109.501920 | 608121 | 5501657 | 0 | 2375.08 | Local |
| F5 | 05/07/2011 0:00 | 9 | 49.675793 | -109.484301 | 609352 | 5503688 | 0 | 6.14 | Local |
| F5 | 05/07/2011 0:00 | 15 | 49.675847 | -109.484313 | 609351 | 5503694 | 1 | 19.51 | Local |
| F5 | 05/07/2011 0:00 | 18 | 49.675675 | -109.484361 | 609348 | 5503675 | 1 | 954.07 | Local |
| F5 | 05/07/2011 0:00 | 21 | 49.671552 | -109.495955 | 608521 | 5503199 | 1 | 482.78 | Local |
| F5 | 05/08/2011 0:00 | 0 | 49.669778 | -109.502062 | 608084 | 5502993 | 1 | 28.21 | Local |
| F5 | 05/08/2011 0:00 | 3 | 49.669843 | -109.502439 | 608057 | 5503000 | 1 | 41.68 | Local |
| F5 | 05/08/2011 0:00 | 6 | 49.669649 | -109.501945 | 608093 | 5502979 | 1 | 887.62 | Local |
| F5 | 05/08/2011 0:00 | 9 | 49.672839 | -109.490671 | 608899 | 5503350 | 1 | 296.84 | Local |
| F5 | 05/08/2011 0:00 | 12 | 49.675479 | -109.490060 | 608938 | 5503645 | 1 | 16.03 | Local |
| F5 | 05/08/2011 0:00 | 15 | 49.675622 | -109.490033 | 608939 | 5503660 | 1 | 241.24 | Local |
| F5 | 05/08/2011 0:00 | 18 | 49.677752 | -109.489391 | 608981 | 5503898 | 1 | 486.19 | Local |
| F5 | 05/08/2011 0:00 | 21 | 49.680103 | -109.495072 | 608566 | 5504151 | 1 | 774.79 | Local |
| F5 | 05/09/2011 0:00 | 0 | 49.674588 | -109.501635 | 608105 | 5503529 | 1 | 523.83 | Local |
| F5 | 05/09/2011 0:00 | 3 | 49.669897 | -109.502312 | 608066 | 5503006 | 1 | 41.52 | Local |

| | | | | | | | | | |
|---|---|---|---|---|---|---|---|---|---|
| F5 | 05/09/2011 0:00 | 6 | 49.669718 | -109.501807 | 608103 | 5502987 | 1 | 996.48 | Local |
| F5 | 05/09/2011 0:00 | 9 | 49.674427 | -109.490058 | 608940 | 5503528 | 1 | 120.52 | Local |
| F5 | 05/09/2011 0:00 | 12 | 49.675466 | -109.489582 | 608972 | 5503644 | 1 | 17.72 | Local |
| F5 | 05/09/2011 0:00 | 15 | 49.675599 | -109.489716 | 608962 | 5503658 | 1 | 391.27 | Local |
| F5 | 05/09/2011 0:00 | 18 | 49.678074 | -109.493571 | 608679 | 5503928 | 0 | 944.54 | Local |
| F5 | 05/10/2011 0:00 | 0 | 49.669787 | -109.496444 | 608490 | 5503002 | 1 | 1502.51 | Local |
| F5 | 05/10/2011 0:00 | 3 | 49.656621 | -109.501132 | 608181 | 5501532 | 0 | 944.46 | Local |
| F5 | 05/10/2011 0:00 | 9 | 49.648885 | -109.495730 | 608588 | 5500680 | 1 | 66.45 | Local |
| F5 | 05/10/2011 0:00 | 12 | 49.649410 | -109.496170 | 608555 | 5500737 | 1 | 20.31 | Local |
| F5 | 05/10/2011 0:00 | 15 | 49.649228 | -109.496146 | 608557 | 5500717 | 1 | 321.67 | Local |
| F5 | 05/10/2011 0:00 | 18 | 49.651961 | -109.494687 | 608656 | 5501023 | 1 | 409.62 | Local |
| F5 | 05/10/2011 0:00 | 21 | 49.655451 | -109.492871 | 608779 | 5501414 | 1 | 16.33 | Local |
| F5 | 05/11/2011 0:00 | 0 | 49.655327 | -109.492750 | 608788 | 5501400 | 1 | 4.27 | Local |
| F5 | 05/11/2011 0:00 | 3 | 49.655362 | -109.492774 | 608787 | 5501404 | 0 | 541.37 | Local |
| F5 | 05/11/2011 0:00 | 9 | 49.650869 | -109.489887 | 609005 | 5500909 | 1 | 7.83 | Local |
| F5 | 05/11/2011 0:00 | 12 | 49.650804 | -109.489930 | 609002 | 5500902 | 1 | 5.81 | Local |
| F5 | 05/11/2011 0:00 | 15 | 49.650850 | -109.489892 | 609005 | 5500907 | 1 | 616.49 | Local |
| F5 | 05/11/2011 0:00 | 18 | 49.656384 | -109.490418 | 608954 | 5501521 | 1 | 718.27 | Local |
| F5 | 05/11/2011 0:00 | 21 | 49.662842 | -109.490668 | 608922 | 5502239 | 1 | 1093.01 | Local |
| F5 | 05/12/2011 0:00 | 0 | 49.655652 | -109.500994 | 608193 | 5501424 | 1 | 9.83 | Local |
| F5 | 05/12/2011 0:00 | 3 | 49.655739 | -109.500970 | 608194 | 5501434 | 1 | 632.91 | Local |
| F5 | 05/12/2011 0:00 | 6 | 49.651941 | -109.494441 | 608674 | 5501021 | 1 | 1564.40 | Local |
| F5 | 05/12/2011 0:00 | 9 | 49.664012 | -109.483310 | 609450 | 5502380 | 1 | 18.63 | Local |
| F5 | 05/12/2011 0:00 | 12 | 49.664159 | -109.483434 | 609441 | 5502396 | 0 | 636.43 | Local |
| F5 | 05/12/2011 0:00 | 18 | 49.665117 | -109.492127 | 608812 | 5502490 | 1 | 1030.62 | Local |
| F5 | 05/12/2011 0:00 | 21 | 49.674174 | -109.495165 | 608572 | 5503492 | 0 | 2099.39 | Local |
| F5 | 05/13/2011 0:00 | 3 | 49.655725 | -109.501349 | 608167 | 5501432 | 1 | 651.01 | Local |
| F5 | 05/13/2011 0:00 | 6 | 49.651109 | -109.495802 | 608578 | 5500927 | 1 | 974.18 | Local |
| F5 | 05/13/2011 0:00 | 9 | 49.659200 | -109.490628 | 608933 | 5501834 | 0 | 162.71 | Local |
| F5 | 05/13/2011 0:00 | 15 | 49.659803 | -109.488574 | 609080 | 5501904 | 1 | 695.49 | Local |
| F5 | 05/13/2011 0:00 | 18 | 49.665669 | -109.491919 | 608825 | 5502551 | 1 | 366.06 | Local |
| F5 | 05/13/2011 0:00 | 21 | 49.668582 | -109.489557 | 608989 | 5502878 | 1 | 868.41 | Local |
| F5 | 05/14/2011 0:00 | 0 | 49.670904 | -109.501046 | 608155 | 5503120 | 1 | 3.58 | Local |
| F5 | 05/14/2011 0:00 | 3 | 49.670929 | -109.501077 | 608153 | 5503123 | 1 | 6.00 | Local |
| F5 | 05/14/2011 0:00 | 6 | 49.670967 | -109.501018 | 608157 | 5503127 | 1 | 1160.75 | Local |
| F5 | 05/14/2011 0:00 | 9 | 49.680587 | -109.494772 | 608586 | 5504206 | 1 | 7.19 | Local |
| F5 | 05/14/2011 0:00 | 12 | 49.680530 | -109.494726 | 608590 | 5504199 | 1 | 6.38 | Local |
| F5 | 05/14/2011 0:00 | 15 | 49.680570 | -109.494663 | 608594 | 5504204 | 1 | 3.56 | Local |
| F5 | 05/14/2011 0:00 | 18 | 49.680576 | -109.494711 | 608591 | 5504204 | 1 | 9.74 | Local |
| F5 | 05/14/2011 0:00 | 21 | 49.680584 | -109.494846 | 608581 | 5504205 | 1 | 522.19 | Local |
| F5 | 05/15/2011 0:00 | 0 | 49.676715 | -109.490746 | 608885 | 5503781 | 1 | 140.04 | Local |
| F5 | 05/15/2011 0:00 | 3 | 49.675587 | -109.491611 | 608825 | 5503654 | 1 | 15.65 | Local |
| F5 | 05/15/2011 0:00 | 6 | 49.675449 | -109.491569 | 608829 | 5503639 | 1 | 37.28 | Local |
| F5 | 05/15/2011 0:00 | 9 | 49.675117 | -109.491497 | 608835 | 5503602 | 1 | 13.96 | Local |
| F5 | 05/15/2011 0:00 | 12 | 49.675078 | -109.491680 | 608822 | 5503598 | 1 | 21.01 | Local |
| F5 | 05/15/2011 0:00 | 15 | 49.675091 | -109.491971 | 608801 | 5503599 | 1 | 8.70 | Local |
| F5 | 05/15/2011 0:00 | 18 | 49.675090 | -109.491850 | 608809 | 5503599 | 1 | 7.84 | Local |
| F5 | 05/15/2011 0:00 | 21 | 49.675087 | -109.491742 | 608817 | 5503599 | 1 | 6.19 | Local |
| F5 | 05/16/2011 0:00 | 0 | 49.675125 | -109.491678 | 608822 | 5503603 | 1 | 23.72 | Local |
| F5 | 05/16/2011 0:00 | 3 | 49.674957 | -109.491476 | 608837 | 5503584 | 1 | 11.28 | Local |
| F5 | 05/16/2011 0:00 | 6 | 49.675052 | -109.491529 | 608833 | 5503595 | 1 | 3.24 | Local |
| F5 | 05/16/2011 0:00 | 9 | 49.675023 | -109.491529 | 608833 | 5503592 | 1 | 786.72 | Local |
| F5 | 05/16/2011 0:00 | 12 | 49.680440 | -109.498543 | 608315 | 5504184 | 1 | 377.72 | Local |
| F5 | 05/16/2011 0:00 | 15 | 49.683079 | -109.501839 | 608071 | 5504472 | 1 | 292.33 | Local |
| F5 | 05/16/2011 0:00 | 18 | 49.681349 | -109.504890 | 607855 | 5504276 | 1 | 1092.11 | Local |

| | | | | | | | | | |
|---|---|---|---|---|---|---|---|---|---|
| F5 | 05/16/2011 0:00 | 21 | 49.671687 | -109.502170 | 608072 | 5503205 | 1 | 405.46 | Local |
| F5 | 05/17/2011 0:00 | 0 | 49.674245 | -109.498164 | 608356 | 5503496 | 1 | 14.07 | Local |
| F5 | 05/17/2011 0:00 | 3 | 49.674140 | -109.498274 | 608348 | 5503484 | 1 | 518.40 | Local |
| F5 | 05/17/2011 0:00 | 6 | 49.675758 | -109.491536 | 608831 | 5503673 | 1 | 382.86 | Local |
| F5 | 05/17/2011 0:00 | 9 | 49.678376 | -109.488091 | 609073 | 5503969 | 1 | 5.37 | Local |
| F5 | 05/17/2011 0:00 | 12 | 49.678360 | -109.488161 | 609068 | 5503967 | 1 | 14.77 | Local |
| F5 | 05/17/2011 0:00 | 15 | 49.678322 | -109.488357 | 609054 | 5503963 | 1 | 267.52 | Local |
| F5 | 05/17/2011 0:00 | 18 | 49.677103 | -109.491553 | 608826 | 5503823 | 1 | 59.10 | Local |
| F5 | 05/17/2011 0:00 | 21 | 49.676929 | -109.492327 | 608771 | 5503802 | 1 | 9.18 | Local |
| F5 | 05/18/2011 0:00 | 0 | 49.676941 | -109.492201 | 608780 | 5503804 | 1 | 2.68 | Local |
| F5 | 05/18/2011 0:00 | 3 | 49.676955 | -109.492232 | 608778 | 5503805 | 1 | 10.51 | Local |
| F5 | 05/18/2011 0:00 | 6 | 49.676912 | -109.492102 | 608787 | 5503801 | 1 | 163.41 | Local |
| F5 | 05/18/2011 0:00 | 9 | 49.675622 | -109.493188 | 608712 | 5503656 | 1 | 49.72 | Local |
| F5 | 05/18/2011 0:00 | 12 | 49.676060 | -109.493331 | 608700 | 5503704 | 1 | 3.85 | Local |
| F5 | 05/18/2011 0:00 | 15 | 49.676044 | -109.493379 | 608697 | 5503702 | 1 | 102.04 | Local |
| F5 | 05/18/2011 0:00 | 18 | 49.676769 | -109.494246 | 608633 | 5503782 | 1 | 355.56 | Local |
| F5 | 05/18/2011 0:00 | 21 | 49.679546 | -109.496689 | 608450 | 5504087 | 1 | 109.06 | Local |
| F5 | 05/19/2011 0:00 | 0 | 49.679549 | -109.495178 | 608559 | 5504090 | 1 | 960.51 | Local |
| F5 | 05/19/2011 0:00 | 3 | 49.687816 | -109.491316 | 608819 | 5505014 | 0 | 1297.19 | Local |
| F5 | 05/19/2011 0:00 | 9 | 49.680476 | -109.505292 | 607828 | 5504178 | 1 | 95.79 | Local |
| F5 | 05/19/2011 0:00 | 12 | 49.680964 | -109.504198 | 607905 | 5504234 | 1 | 527.31 | Local |
| F5 | 05/19/2011 0:00 | 15 | 49.682436 | -109.497250 | 608403 | 5504407 | 1 | 588.37 | Local |
| F5 | 05/19/2011 0:00 | 18 | 49.677887 | -109.501417 | 608113 | 5503896 | 1 | 866.18 | Local |
| F5 | 05/19/2011 0:00 | 21 | 49.670335 | -109.498476 | 608342 | 5503060 | 1 | 6.05 | Local |
| F5 | 05/20/2011 0:00 | 0 | 49.670369 | -109.498542 | 608337 | 5503064 | 0 | 2332.06 | Local |
| F5 | 05/20/2011 0:00 | 6 | 49.649624 | -109.493785 | 608726 | 5500765 | 1 | 44.83 | Local |
| F5 | 05/20/2011 0:00 | 9 | 49.649612 | -109.493165 | 608771 | 5500764 | 1 | 99.13 | Local |
| F5 | 05/20/2011 0:00 | 12 | 49.648737 | -109.492905 | 608792 | 5500667 | 1 | 112.44 | Local |
| F5 | 05/20/2011 0:00 | 15 | 49.649709 | -109.493336 | 608759 | 5500775 | 1 | 471.51 | Local |
| F5 | 05/20/2011 0:00 | 18 | 49.645470 | -109.493153 | 608781 | 5500304 | 1 | 977.46 | Local |
| F5 | 05/20/2011 0:00 | 21 | 49.646325 | -109.506626 | 607807 | 5500379 | 1 | 1947.49 | Local |
| F5 | 05/21/2011 0:00 | 0 | 49.660265 | -109.522958 | 606597 | 5501906 | 1 | 953.70 | Local |
| F5 | 05/21/2011 0:00 | 3 | 49.668780 | -109.521379 | 606693 | 5502855 | 1 | 1163.31 | Local |
| F5 | 05/21/2011 0:00 | 6 | 49.675949 | -109.509638 | 607524 | 5503669 | 1 | 6.42 | Local |
| F5 | 05/21/2011 0:00 | 9 | 49.675894 | -109.509664 | 607522 | 5503662 | 0 | 11.99 | Local |
| F5 | 05/21/2011 0:00 | 15 | 49.675918 | -109.509826 | 607511 | 5503665 | 1 | 15.61 | Local |
| F5 | 05/21/2011 0:00 | 18 | 49.676054 | -109.509772 | 607514 | 5503680 | 1 | 658.90 | Local |
| F5 | 05/21/2011 0:00 | 21 | 49.672694 | -109.502251 | 608064 | 5503317 | 1 | 22.65 | Local |
| F5 | 05/22/2011 0:00 | 0 | 49.672515 | -109.502401 | 608054 | 5503297 | 1 | 44.19 | Local |
| F5 | 05/22/2011 0:00 | 3 | 49.672646 | -109.502978 | 608012 | 5503311 | 1 | 194.35 | Local |
| F5 | 05/22/2011 0:00 | 6 | 49.673142 | -109.500396 | 608197 | 5503370 | 1 | 304.94 | Local |
| F5 | 05/22/2011 0:00 | 9 | 49.673799 | -109.496293 | 608492 | 5503449 | 1 | 487.77 | Local |
| F5 | 05/22/2011 0:00 | 12 | 49.673430 | -109.489558 | 608978 | 5503417 | 1 | 4.70 | Local |
| F5 | 05/22/2011 0:00 | 15 | 49.673409 | -109.489501 | 608983 | 5503415 | 1 | 12.44 | Local |
| F5 | 05/22/2011 0:00 | 18 | 49.673301 | -109.489545 | 608980 | 5503403 | 1 | 376.28 | Local |
| F5 | 05/22/2011 0:00 | 21 | 49.671806 | -109.494223 | 608645 | 5503230 | 1 | 1315.33 | Local |
| F5 | 05/23/2011 0:00 | 0 | 49.664898 | -109.509018 | 607593 | 5502441 | 1 | 9.66 | Local |
| F5 | 05/23/2011 0:00 | 3 | 49.664877 | -109.509148 | 607584 | 5502438 | 1 | 1507.11 | Local |
| F5 | 05/23/2011 0:00 | 6 | 49.678414 | -109.510195 | 607478 | 5503942 | 1 | 470.19 | Local |
| F5 | 05/23/2011 0:00 | 9 | 49.682642 | -109.510092 | 607477 | 5504412 | 1 | 36.50 | Local |
| F5 | 05/23/2011 0:00 | 12 | 49.682969 | -109.510051 | 607479 | 5504448 | 1 | 23.89 | Local |
| F5 | 05/23/2011 0:00 | 15 | 49.682873 | -109.510348 | 607458 | 5504437 | 1 | 11.40 | Local |
| F5 | 05/23/2011 0:00 | 18 | 49.682807 | -109.510227 | 607467 | 5504430 | 1 | 1026.94 | Local |
| F5 | 05/23/2011 0:00 | 21 | 49.675468 | -109.518867 | 606859 | 5503602 | 1 | 1365.62 | Local |
| F5 | 05/24/2011 0:00 | 0 | 49.665018 | -109.508927 | 607600 | 5502454 | 1 | 21.17 | Local |

| | | | | | | | | | |
|---|---|---|---|---|---|---|---|---|---|
| F5 | 05/24/2011 0:00 | 3 | 49.664864 | -109.509099 | 607587 | 5502437 | 0 | 1713.78 | Local |
| F5 | 05/24/2011 0:00 | 9 | 49.673237 | -109.489163 | 609007 | 5503397 | 1 | 200.68 | Local |
| F5 | 05/24/2011 0:00 | 12 | 49.674525 | -109.487214 | 609145 | 5503543 | 1 | 18.69 | Local |
| F5 | 05/24/2011 0:00 | 15 | 49.674371 | -109.487316 | 609138 | 5503525 | 1 | 34.56 | Local |
| F5 | 05/24/2011 0:00 | 18 | 49.674080 | -109.487487 | 609126 | 5503493 | 1 | 335.55 | Local |
| F5 | 05/24/2011 0:00 | 21 | 49.672752 | -109.483312 | 609431 | 5503351 | 1 | 9.50 | Local |
| F5 | 05/25/2011 0:00 | 0 | 49.672811 | -109.483216 | 609437 | 5503358 | 0 | 5.34 | Local |
| F5 | 05/25/2011 0:00 | 6 | 49.672764 | -109.483224 | 609437 | 5503353 | 1 | 200.97 | Local |
| F5 | 05/25/2011 0:00 | 9 | 49.674571 | -109.483232 | 609432 | 5503553 | 1 | 19.62 | Local |
| F5 | 05/25/2011 0:00 | 12 | 49.674733 | -109.483126 | 609440 | 5503572 | 1 | 20.01 | Local |
| F5 | 05/25/2011 0:00 | 15 | 49.674554 | -109.483146 | 609438 | 5503552 | 1 | 32.87 | Local |
| F5 | 05/25/2011 0:00 | 18 | 49.674846 | -109.483079 | 609443 | 5503584 | 0 | 1352.89 | Local |
| F5 | 05/26/2011 0:00 | 0 | 49.671287 | -109.465151 | 610744 | 5503215 | 1 | 1305.97 | Local |
| F5 | 05/26/2011 0:00 | 3 | 49.662803 | -109.477665 | 609860 | 5502253 | 1 | 1381.79 | Local |
| F5 | 05/26/2011 0:00 | 6 | 49.672043 | -109.490467 | 608916 | 5503262 | 1 | 595.69 | Local |
| F5 | 05/26/2011 0:00 | 9 | 49.676293 | -109.485441 | 609269 | 5503742 | 1 | 11.13 | Local |
| F5 | 05/26/2011 0:00 | 12 | 49.676220 | -109.485335 | 609277 | 5503734 | 1 | 715.75 | Local |
| F5 | 05/26/2011 0:00 | 15 | 49.678948 | -109.476351 | 609919 | 5504050 | 1 | 437.75 | Local |
| F5 | 05/26/2011 0:00 | 18 | 49.676728 | -109.481361 | 609562 | 5503796 | 1 | 2236.15 | Local |
| F5 | 05/26/2011 0:00 | 21 | 49.693536 | -109.464345 | 610752 | 5505690 | 1 | 1260.50 | Local |
| F5 | 05/27/2011 0:00 | 0 | 49.697384 | -109.480783 | 609558 | 5506093 | 1 | 14.25 | Local |
| F5 | 05/27/2011 0:00 | 3 | 49.697293 | -109.480643 | 609568 | 5506083 | 1 | 29.60 | Local |
| F5 | 05/27/2011 0:00 | 6 | 49.697072 | -109.480871 | 609552 | 5506058 | 1 | 679.65 | Local |
| F5 | 05/27/2011 0:00 | 9 | 49.691544 | -109.484894 | 609274 | 5505438 | 1 | 57.00 | Local |
| F5 | 05/27/2011 0:00 | 12 | 49.691377 | -109.484147 | 609329 | 5505420 | 1 | 6.64 | Local |
| F5 | 05/27/2011 0:00 | 15 | 49.691321 | -109.484178 | 609326 | 5505414 | 1 | 1.29 | Local |
| F5 | 05/27/2011 0:00 | 18 | 49.691319 | -109.484195 | 609325 | 5505414 | 1 | 686.43 | Local |
| F5 | 05/27/2011 0:00 | 21 | 49.696220 | -109.489981 | 608897 | 5505950 | 1 | 520.00 | Local |
| F5 | 05/28/2011 0:00 | 0 | 49.700851 | -109.488973 | 608959 | 5506467 | 1 | 1043.43 | Local |
| F5 | 05/28/2011 0:00 | 3 | 49.708376 | -109.480329 | 609566 | 5507316 | 1 | 2217.18 | Local |
| F5 | 05/28/2011 0:00 | 6 | 49.699154 | -109.507588 | 607621 | 5506251 | 1 | 422.14 | Local |
| F5 | 05/28/2011 0:00 | 9 | 49.701265 | -109.512453 | 607265 | 5506479 | 1 | 7.06 | Local |
| F5 | 05/28/2011 0:00 | 12 | 49.701322 | -109.512410 | 607268 | 5506485 | 1 | 7.84 | Local |
| F5 | 05/28/2011 0:00 | 15 | 49.701361 | -109.512320 | 607275 | 5506490 | 1 | 0.48 | Local |
| F5 | 05/28/2011 0:00 | 18 | 49.701360 | -109.512314 | 607275 | 5506490 | 0 | 1423.55 | Local |
| F5 | 05/29/2011 0:00 | 0 | 49.689925 | -109.521190 | 606660 | 5505206 | 1 | 2884.85 | Local |
| F5 | 05/29/2011 0:00 | 3 | 49.665092 | -109.509616 | 607550 | 5502462 | 1 | 1606.46 | Local |
| F5 | 05/29/2011 0:00 | 6 | 49.674609 | -109.492869 | 608737 | 5503544 | 1 | 33.74 | Local |
| F5 | 05/29/2011 0:00 | 9 | 49.674856 | -109.492597 | 608756 | 5503572 | 0 | 13.74 | Local |
| F5 | 05/29/2011 0:00 | 15 | 49.674746 | -109.492681 | 608750 | 5503559 | 1 | 6.87 | Local |
| F5 | 05/29/2011 0:00 | 18 | 49.674801 | -109.492639 | 608753 | 5503565 | 1 | 704.53 | Local |
| F5 | 05/29/2011 0:00 | 21 | 49.672621 | -109.483472 | 609419 | 5503336 | 1 | 1056.18 | Local |
| F5 | 05/30/2011 0:00 | 0 | 49.665765 | -109.493601 | 608704 | 5502559 | 1 | 744.74 | Local |
| F5 | 05/30/2011 0:00 | 3 | 49.659113 | -109.494796 | 608632 | 5501818 | 1 | 820.06 | Local |
| F5 | 05/30/2011 0:00 | 6 | 49.656564 | -109.484135 | 609407 | 5501550 | 1 | 13.51 | Local |
| F5 | 05/30/2011 0:00 | 9 | 49.656625 | -109.483974 | 609419 | 5501557 | 1 | 1120.15 | Local |
| F5 | 05/30/2011 0:00 | 12 | 49.653138 | -109.469417 | 610477 | 5501191 | 1 | 223.77 | Local |
| F5 | 05/30/2011 0:00 | 15 | 49.651662 | -109.467311 | 610633 | 5501030 | 0 | 2269.72 | Local |
| F5 | 05/30/2011 0:00 | 21 | 49.637445 | -109.489867 | 609037 | 5499416 | 1 | 2246.92 | Local |
| F5 | 05/31/2011 0:00 | 0 | 49.642467 | -109.520006 | 606849 | 5499932 | 1 | 2479.92 | Local |
| F5 | 05/31/2011 0:00 | 3 | 49.664068 | -109.528561 | 606185 | 5502321 | 1 | 2369.16 | Local |
| F5 | 05/31/2011 0:00 | 6 | 49.682166 | -109.545885 | 604896 | 5504308 | 0 | 1439.65 | Local |
| F5 | 05/31/2011 0:00 | 15 | 49.695111 | -109.546298 | 604838 | 5505747 | 1 | 306.85 | Local |
| F5 | 05/31/2011 0:00 | 18 | 49.697175 | -109.549122 | 604630 | 5505973 | 1 | 5.77 | Local |
| F5 | 05/31/2011 0:00 | 21 | 49.697222 | -109.549087 | 604632 | 5505978 | 1 | 420.19 | Local |

| | | | | | | | | | |
|---|---|---|---|---|---|---|---|---|---|
| F5 | 06/01/2011 0:00 | 0 | 49.699204 | -109.544127 | 604986 | 5506205 | 1 | 3242.60 | Local |
| F5 | 06/01/2011 0:00 | 3 | 49.706678 | -109.500666 | 608103 | 5507098 | 1 | 863.38 | Local |
| F5 | 06/01/2011 0:00 | 6 | 49.699715 | -109.495367 | 608501 | 5506331 | 1 | 19.32 | Local |
| F5 | 06/01/2011 0:00 | 9 | 49.699596 | -109.495561 | 608487 | 5506318 | 0 | 14.55 | Local |
| F5 | 06/01/2011 0:00 | 15 | 49.699705 | -109.495451 | 608495 | 5506330 | 1 | 31.37 | Local |
| F5 | 06/01/2011 0:00 | 18 | 49.699930 | -109.495188 | 608513 | 5506355 | 1 | 230.95 | Local |
| F5 | 06/01/2011 0:00 | 21 | 49.700704 | -109.498160 | 608297 | 5506437 | 1 | 570.24 | Local |
| F5 | 06/02/2011 0:00 | 0 | 49.700137 | -109.490302 | 608865 | 5506385 | 1 | 1314.34 | Local |
| F5 | 06/02/2011 0:00 | 3 | 49.705853 | -109.474350 | 610002 | 5507044 | 1 | 24.31 | Local |
| F5 | 06/02/2011 0:00 | 6 | 49.705660 | -109.474507 | 609992 | 5507022 | 1 | 13.62 | Local |
| F5 | 06/02/2011 0:00 | 9 | 49.705539 | -109.474540 | 609989 | 5507009 | 0 | 11.33 | Local |
| F5 | 06/02/2011 0:00 | 15 | 49.705627 | -109.474620 | 609983 | 5507019 | 1 | 7.35 | Local |
| F5 | 06/02/2011 0:00 | 18 | 49.705601 | -109.474526 | 609990 | 5507016 | 1 | 11.99 | Local |
| F5 | 06/02/2011 0:00 | 21 | 49.705658 | -109.474668 | 609980 | 5507022 | 1 | 243.13 | Local |
| F5 | 06/03/2011 0:00 | 0 | 49.703472 | -109.474698 | 609983 | 5506779 | 0 | 862.58 | Local |
| F5 | 06/03/2011 0:00 | 6 | 49.702059 | -109.462937 | 610834 | 5506639 | 0 | 1003.34 | Local |
| F5 | 06/03/2011 0:00 | 12 | 49.693087 | -109.464427 | 610747 | 5505639 | 1 | 23.25 | Local |
| F5 | 06/03/2011 0:00 | 15 | 49.692918 | -109.464237 | 610761 | 5505621 | 1 | 26.95 | Local |
| F5 | 06/03/2011 0:00 | 18 | 49.692717 | -109.464446 | 610746 | 5505598 | 1 | 458.33 | Local |
| F5 | 06/03/2011 0:00 | 21 | 49.689117 | -109.461352 | 610978 | 5505203 | 1 | 353.93 | Local |
| F5 | 06/04/2011 0:00 | 0 | 49.686107 | -109.459758 | 611100 | 5504870 | 1 | 829.51 | Local |
| F5 | 06/04/2011 0:00 | 3 | 49.693075 | -109.463867 | 610787 | 5505639 | 1 | 5.62 | Local |
| F5 | 06/04/2011 0:00 | 6 | 49.693044 | -109.463928 | 610783 | 5505635 | 1 | 11.26 | Local |
| F5 | 06/04/2011 0:00 | 9 | 49.692948 | -109.463878 | 610787 | 5505625 | 0 | 83.62 | Local |
| F5 | 06/04/2011 0:00 | 15 | 49.692832 | -109.465024 | 610704 | 5505610 | 1 | 34.41 | Local |
| F5 | 06/04/2011 0:00 | 18 | 49.692844 | -109.464547 | 610739 | 5505612 | 1 | 51.14 | Local |
| F5 | 06/04/2011 0:00 | 21 | 49.692972 | -109.463866 | 610788 | 5505627 | 1 | 1135.91 | Local |
| F5 | 06/05/2011 0:00 | 0 | 49.697570 | -109.477928 | 609763 | 5506118 | 0 | 2731.85 | Local |
| F5 | 06/05/2011 0:00 | 6 | 49.705068 | -109.514000 | 607145 | 5506899 | 1 | 410.13 | Local |
| F5 | 06/05/2011 0:00 | 9 | 49.701382 | -109.513798 | 607168 | 5506490 | 1 | 59.04 | Local |
| F5 | 06/05/2011 0:00 | 12 | 49.701643 | -109.513086 | 607219 | 5506520 | 1 | 25.43 | Local |
| F5 | 06/05/2011 0:00 | 15 | 49.701417 | -109.513032 | 607223 | 5506495 | 1 | 80.63 | Local |
| F5 | 06/05/2011 0:00 | 18 | 49.702138 | -109.513149 | 607213 | 5506575 | 1 | 8.11 | Local |
| F5 | 06/05/2011 0:00 | 21 | 49.702120 | -109.513258 | 607205 | 5506573 | 1 | 24.66 | Local |
| F5 | 06/06/2011 0:00 | 0 | 49.702171 | -109.513591 | 607181 | 5506578 | 1 | 139.77 | Local |
| F5 | 06/06/2011 0:00 | 3 | 49.702332 | -109.515513 | 607042 | 5506593 | 1 | 150.08 | Local |
| F5 | 06/06/2011 0:00 | 6 | 49.701537 | -109.513831 | 607165 | 5506507 | 1 | 368.87 | Local |
| F5 | 06/06/2011 0:00 | 9 | 49.698939 | -109.517011 | 606942 | 5506214 | 1 | 207.13 | Local |
| F5 | 06/06/2011 0:00 | 12 | 49.697726 | -109.514832 | 607102 | 5506082 | 1 | 15.38 | Local |
| F5 | 06/06/2011 0:00 | 15 | 49.697588 | -109.514834 | 607102 | 5506067 | 1 | 95.50 | Local |
| F5 | 06/06/2011 0:00 | 18 | 49.698251 | -109.513993 | 607161 | 5506142 | 1 | 356.47 | Local |
| F5 | 06/06/2011 0:00 | 21 | 49.701456 | -109.513841 | 607165 | 5506498 | 1 | 13.33 | Local |
| F5 | 06/07/2011 0:00 | 0 | 49.701495 | -109.513666 | 607177 | 5506503 | 1 | 6.45 | Local |
| F5 | 06/07/2011 0:00 | 3 | 49.701512 | -109.513751 | 607171 | 5506505 | 1 | 90.13 | Local |
| F5 | 06/07/2011 0:00 | 6 | 49.701282 | -109.514950 | 607085 | 5506477 | 1 | 91.79 | Local |
| F5 | 06/07/2011 0:00 | 9 | 49.701555 | -109.513749 | 607171 | 5506509 | 1 | 7.01 | Local |
| F5 | 06/07/2011 0:00 | 12 | 49.701545 | -109.513845 | 607164 | 5506508 | 1 | 19.40 | Local |
| F5 | 06/07/2011 0:00 | 15 | 49.701385 | -109.513738 | 607172 | 5506490 | 1 | 14.84 | Local |
| F5 | 06/07/2011 0:00 | 18 | 49.701515 | -109.513785 | 607169 | 5506505 | 1 | 3.31 | Local |
| F5 | 06/07/2011 0:00 | 21 | 49.701518 | -109.513739 | 607172 | 5506505 | 1 | 6.47 | Local |
| F5 | 06/08/2011 0:00 | 0 | 49.701479 | -109.513673 | 607177 | 5506501 | 1 | 13.00 | Local |
| F5 | 06/08/2011 0:00 | 3 | 49.701472 | -109.513853 | 607164 | 5506500 | 1 | 308.72 | Local |
| F5 | 06/08/2011 0:00 | 6 | 49.702154 | -109.518002 | 606863 | 5506570 | 1 | 1294.59 | Local |
| F5 | 06/08/2011 0:00 | 9 | 49.691153 | -109.512126 | 607311 | 5505355 | 1 | 770.25 | Local |
| F5 | 06/08/2011 0:00 | 12 | 49.698053 | -109.513077 | 607227 | 5506121 | 1 | 27.83 | Local |

| | | | | | | | | | |
|---|---|---|---|---|---|---|---|---|---|
| F5 | 06/08/2011 0:00 | 15 | 49.698229 | -109.512803 | 607247 | 5506141 | 1 | 52.03 | Local |
| F5 | 06/08/2011 0:00 | 18 | 49.698063 | -109.512128 | 607296 | 5506123 | 1 | 136.74 | Local |
| F5 | 06/08/2011 0:00 | 21 | 49.699084 | -109.511069 | 607370 | 5506238 | 1 | 131.02 | Local |
| F5 | 06/09/2011 0:00 | 0 | 49.698191 | -109.512255 | 607286 | 5506137 | 1 | 11.54 | Local |
| F5 | 06/09/2011 0:00 | 3 | 49.698284 | -109.512183 | 607291 | 5506148 | 1 | 30.72 | Local |
| F5 | 06/09/2011 0:00 | 6 | 49.698069 | -109.511915 | 607311 | 5506124 | 1 | 241.86 | Local |
| F5 | 06/09/2011 0:00 | 9 | 49.700236 | -109.512204 | 607286 | 5506365 | 1 | 9.39 | Local |
| F5 | 06/09/2011 0:00 | 12 | 49.700255 | -109.512077 | 607295 | 5506367 | 1 | 282.58 | Local |
| F5 | 06/09/2011 0:00 | 15 | 49.700306 | -109.508160 | 607577 | 5506378 | 1 | 694.27 | Local |
| F5 | 06/09/2011 0:00 | 18 | 49.694063 | -109.508247 | 607585 | 5505684 | 1 | 1189.49 | Local |
| F5 | 06/09/2011 0:00 | 21 | 49.688040 | -109.494618 | 608581 | 5505034 | 1 | 716.43 | Local |
| F5 | 06/10/2011 0:00 | 0 | 49.688206 | -109.504546 | 607864 | 5505038 | 1 | 8.19 | Local |
| F5 | 06/10/2011 0:00 | 3 | 49.688261 | -109.504621 | 607859 | 5505045 | 1 | 54.05 | Local |
| F5 | 06/10/2011 0:00 | 6 | 49.688415 | -109.503910 | 607910 | 5505063 | 1 | 30.61 | Local |
| F5 | 06/10/2011 0:00 | 9 | 49.688306 | -109.504300 | 607882 | 5505050 | 1 | 26.44 | Local |
| F5 | 06/10/2011 0:00 | 12 | 49.688435 | -109.504607 | 607859 | 5505064 | 1 | 314.27 | Local |
| F5 | 06/10/2011 0:00 | 15 | 49.685811 | -109.506224 | 607749 | 5504770 | 1 | 10.58 | Local |
| F5 | 06/10/2011 0:00 | 18 | 49.685817 | -109.506370 | 607738 | 5504770 | 1 | 454.20 | Local |
| F5 | 06/10/2011 0:00 | 21 | 49.682958 | -109.501874 | 608069 | 5504459 | 1 | 618.25 | Local |
| F5 | 06/11/2011 0:00 | 0 | 49.688260 | -109.504454 | 607871 | 5505045 | 0 | 13.67 | Local |
| F5 | 06/11/2011 0:00 | 6 | 49.688274 | -109.504643 | 607857 | 5505046 | 1 | 146.52 | Local |
| F5 | 06/11/2011 0:00 | 9 | 49.687491 | -109.506277 | 607741 | 5504957 | 1 | 29.00 | Local |
| F5 | 06/11/2011 0:00 | 12 | 49.687231 | -109.506302 | 607740 | 5504928 | 1 | 147.96 | Local |
| F5 | 06/11/2011 0:00 | 15 | 49.685901 | -109.506351 | 607739 | 5504780 | 1 | 348.79 | Local |
| F5 | 06/11/2011 0:00 | 18 | 49.685080 | -109.511017 | 607405 | 5504682 | 0 | 1792.48 | Local |
| F5 | 06/12/2011 0:00 | 0 | 49.671572 | -109.497460 | 608412 | 5503199 | 1 | 443.90 | Local |
| F5 | 06/12/2011 0:00 | 3 | 49.667580 | -109.497421 | 608424 | 5502756 | 0 | 2616.56 | Local |
| F5 | 06/12/2011 0:00 | 12 | 49.690492 | -109.505681 | 607777 | 5505291 | 0 | 10.53 | Local |
| F5 | 06/12/2011 0:00 | 18 | 49.690585 | -109.505650 | 607779 | 5505301 | 1 | 320.78 | Local |
| F5 | 06/12/2011 0:00 | 21 | 49.688008 | -109.503652 | 607929 | 5505018 | 1 | 17.57 | Local |
| F5 | 06/13/2011 0:00 | 0 | 49.687914 | -109.503456 | 607944 | 5505008 | 1 | 20.26 | Local |
| F5 | 06/13/2011 0:00 | 3 | 49.688001 | -109.503703 | 607926 | 5505017 | 1 | 79.81 | Local |
| F5 | 06/13/2011 0:00 | 6 | 49.688432 | -109.504588 | 607861 | 5505063 | 1 | 97.95 | Local |
| F5 | 06/13/2011 0:00 | 9 | 49.688593 | -109.503253 | 607957 | 5505083 | 1 | 12.57 | Local |
| F5 | 06/13/2011 0:00 | 12 | 49.688583 | -109.503426 | 607944 | 5505082 | 1 | 61.76 | Local |
| F5 | 06/13/2011 0:00 | 15 | 49.688344 | -109.504199 | 607889 | 5505054 | 1 | 15.08 | Local |
| F5 | 06/13/2011 0:00 | 18 | 49.688236 | -109.504326 | 607880 | 5505042 | 1 | 22.80 | Local |
| F5 | 06/13/2011 0:00 | 21 | 49.688437 | -109.504265 | 607884 | 5505065 | 1 | 10.27 | Local |
| F5 | 06/14/2011 0:00 | 0 | 49.688447 | -109.504406 | 607874 | 5505065 | 1 | 10.72 | Local |
| F5 | 06/14/2011 0:00 | 3 | 49.688378 | -109.504510 | 607867 | 5505058 | 1 | 22.48 | Local |
| F5 | 06/14/2011 0:00 | 6 | 49.688557 | -109.504363 | 607877 | 5505078 | 1 | 4.35 | Local |
| F5 | 06/14/2011 0:00 | 9 | 49.688518 | -109.504362 | 607877 | 5505073 | 1 | 10.73 | Local |
| F5 | 06/14/2011 0:00 | 12 | 49.688441 | -109.504452 | 607871 | 5505065 | 0 | 46.06 | Local |
| F5 | 06/14/2011 0:00 | 21 | 49.688045 | -109.504265 | 607885 | 5505021 | 1 | 39.57 | Local |
| F5 | 06/15/2011 0:00 | 0 | 49.687968 | -109.503729 | 607924 | 5505013 | 1 | 74.66 | Local |
| F5 | 06/15/2011 0:00 | 3 | 49.688444 | -109.504460 | 607870 | 5505065 | 1 | 16.31 | Local |
| F5 | 06/15/2011 0:00 | 6 | 49.688299 | -109.504423 | 607873 | 5505049 | 1 | 253.47 | Local |
| F5 | 06/15/2011 0:00 | 9 | 49.690412 | -109.505741 | 607773 | 5505282 | 0 | 207.69 | Local |
| F5 | 06/15/2011 0:00 | 18 | 49.688634 | -109.504857 | 607841 | 5505086 | 1 | 38.89 | Local |
| F5 | 06/15/2011 0:00 | 21 | 49.688553 | -109.504333 | 607879 | 5505077 | 1 | 24.46 | Local |
| F5 | 06/16/2011 0:00 | 0 | 49.688336 | -109.504391 | 607875 | 5505053 | 1 | 59.16 | Local |
| F5 | 06/16/2011 0:00 | 3 | 49.688061 | -109.503690 | 607926 | 5505024 | 1 | 94.92 | Local |
| F5 | 06/16/2011 0:00 | 6 | 49.688794 | -109.503016 | 607973 | 5505106 | 1 | 1544.31 | Local |
| F5 | 06/16/2011 0:00 | 9 | 49.688296 | -109.524409 | 606432 | 5505020 | 1 | 852.73 | Local |
| F5 | 06/16/2011 0:00 | 12 | 49.690844 | -109.535559 | 605622 | 5505288 | 1 | 1.82 | Local |

| | | | | | | | | | |
|---|---|---|---|---|---|---|---|---|---|
| F5 | 06/16/2011 0:00 | 15 | 49.690860 | -109.535556 | 605622 | 5505289 | 1 | 12.24 | Local |
| F5 | 06/16/2011 0:00 | 18 | 49.690810 | -109.535708 | 605611 | 5505284 | 1 | 75.16 | Local |
| F5 | 06/16/2011 0:00 | 21 | 49.691483 | -109.535812 | 605602 | 5505358 | 1 | 33.86 | Local |
| F5 | 06/17/2011 0:00 | 0 | 49.691764 | -109.535994 | 605588 | 5505389 | 1 | 71.84 | Local |
| F5 | 06/17/2011 0:00 | 3 | 49.691129 | -109.535804 | 605603 | 5505319 | 1 | 131.28 | Local |
| F5 | 06/17/2011 0:00 | 6 | 49.690411 | -109.534360 | 605709 | 5505241 | 0 | 131.44 | Local |
| F5 | 06/17/2011 0:00 | 12 | 49.691476 | -109.533569 | 605764 | 5505361 | 1 | 3.44 | Local |
| F5 | 06/17/2011 0:00 | 15 | 49.691446 | -109.533561 | 605765 | 5505357 | 1 | 166.50 | Local |
| F5 | 06/17/2011 0:00 | 18 | 49.690932 | -109.535729 | 605609 | 5505297 | 1 | 59.04 | Local |
| F5 | 06/17/2011 0:00 | 21 | 49.691360 | -109.535245 | 605643 | 5505345 | 1 | 492.43 | Local |
| F5 | 06/18/2011 0:00 | 0 | 49.687754 | -109.539208 | 605365 | 5504939 | 1 | 33.58 | Local |
| F5 | 06/18/2011 0:00 | 3 | 49.687463 | -109.539333 | 605357 | 5504907 | 0 | 1012.34 | Local |
| F5 | 06/18/2011 0:00 | 9 | 49.688047 | -109.525329 | 606366 | 5504991 | 1 | 1161.80 | Local |
| F5 | 06/18/2011 0:00 | 12 | 49.696907 | -109.516792 | 606962 | 5505988 | 1 | 886.33 | Local |
| F5 | 06/18/2011 0:00 | 15 | 49.702588 | -109.508172 | 607571 | 5506632 | 1 | 470.05 | Local |
| F5 | 06/18/2011 0:00 | 18 | 49.698539 | -109.506303 | 607715 | 5506185 | 1 | 1200.06 | Local |
| F5 | 06/18/2011 0:00 | 21 | 49.687905 | -109.503462 | 607943 | 5505007 | 1 | 26.15 | Local |
| F5 | 06/19/2011 0:00 | 0 | 49.688085 | -109.503694 | 607926 | 5505026 | 1 | 14.70 | Local |
| F5 | 06/19/2011 0:00 | 3 | 49.687956 | -109.503736 | 607923 | 5505012 | 1 | 10.27 | Local |
| F5 | 06/19/2011 0:00 | 6 | 49.688047 | -109.503713 | 607925 | 5505022 | 1 | 57.96 | Local |
| F5 | 06/19/2011 0:00 | 9 | 49.688549 | -109.503496 | 607939 | 5505078 | 1 | 3.95 | Local |
| F5 | 06/19/2011 0:00 | 12 | 49.688551 | -109.503441 | 607943 | 5505078 | 1 | 1.29 | Local |
| F5 | 06/19/2011 0:00 | 15 | 49.688539 | -109.503441 | 607943 | 5505077 | 1 | 63.82 | Local |
| F5 | 06/19/2011 0:00 | 18 | 49.688241 | -109.504197 | 607889 | 5505043 | 1 | 68.11 | Local |
| F5 | 06/19/2011 0:00 | 21 | 49.688531 | -109.503366 | 607949 | 5505076 | 1 | 4.84 | Local |
| F5 | 06/20/2011 0:00 | 0 | 49.688573 | -109.503379 | 607948 | 5505081 | 1 | 43.28 | Local |
| F5 | 06/20/2011 0:00 | 3 | 49.688316 | -109.503829 | 607916 | 5505052 | 0 | 1224.57 | Local |
| F5 | 06/20/2011 0:00 | 9 | 49.695900 | -109.491520 | 608787 | 5505913 | 1 | 151.00 | Local |
| F5 | 06/20/2011 0:00 | 12 | 49.696303 | -109.493519 | 608642 | 5505955 | 1 | 902.66 | Local |
| F5 | 06/20/2011 0:00 | 15 | 49.688981 | -109.488117 | 609048 | 5505148 | 1 | 1155.01 | Local |
| F5 | 06/20/2011 0:00 | 18 | 49.680526 | -109.478817 | 609737 | 5504222 | 1 | 1622.82 | Local |
| F5 | 06/20/2011 0:00 | 21 | 49.666018 | -109.481257 | 609594 | 5502605 | 1 | 269.82 | Local |
| F5 | 06/21/2011 0:00 | 0 | 49.664954 | -109.484618 | 609354 | 5502482 | 1 | 3.25 | Local |
| F5 | 06/21/2011 0:00 | 3 | 49.664958 | -109.484662 | 609351 | 5502483 | 1 | 440.51 | Local |
| F5 | 06/21/2011 0:00 | 6 | 49.661994 | -109.480613 | 609649 | 5502159 | 1 | 996.89 | Local |
| F5 | 06/21/2011 0:00 | 9 | 49.655621 | -109.470898 | 610365 | 5501465 | 1 | 119.13 | Local |
| F5 | 06/21/2011 0:00 | 12 | 49.655220 | -109.469368 | 610476 | 5501422 | 1 | 16.97 | Local |
| F5 | 06/21/2011 0:00 | 15 | 49.655170 | -109.469590 | 610460 | 5501417 | 1 | 447.91 | Local |
| F5 | 06/21/2011 0:00 | 18 | 49.651466 | -109.467152 | 610645 | 5501008 | 1 | 1264.68 | Local |
| F5 | 06/21/2011 0:00 | 21 | 49.655702 | -109.483409 | 609462 | 5501456 | 0 | 2621.32 | Local |
| F5 | 06/22/2011 0:00 | 9 | 49.632565 | -109.490365 | 609011 | 5498873 | 1 | 9.90 | Local |
| F5 | 06/22/2011 0:00 | 12 | 49.632508 | -109.490259 | 609019 | 5498867 | 1 | 5.08 | Local |
| F5 | 06/22/2011 0:00 | 15 | 49.632503 | -109.490329 | 609014 | 5498866 | 1 | 5.44 | Local |
| F5 | 06/22/2011 0:00 | 18 | 49.632537 | -109.490383 | 609010 | 5498870 | 1 | 577.81 | Local |
| F5 | 06/22/2011 0:00 | 21 | 49.634917 | -109.497495 | 608491 | 5499124 | 1 | 1222.98 | Local |
| F5 | 06/23/2011 0:00 | 0 | 49.645771 | -109.500229 | 608270 | 5500327 | 1 | 276.73 | Local |
| F5 | 06/23/2011 0:00 | 3 | 49.648246 | -109.499829 | 608293 | 5500603 | 1 | 2833.27 | Local |
| F5 | 06/23/2011 0:00 | 6 | 49.664506 | -109.530049 | 606077 | 5502367 | 1 | 2606.11 | Local |
| F5 | 06/23/2011 0:00 | 9 | 49.682753 | -109.552715 | 604402 | 5504364 | 1 | 282.37 | Local |
| F5 | 06/23/2011 0:00 | 12 | 49.682336 | -109.556575 | 604124 | 5504313 | 1 | 505.44 | Local |
| F5 | 06/23/2011 0:00 | 15 | 49.680966 | -109.563255 | 603645 | 5504151 | 1 | 225.51 | Local |
| F5 | 06/23/2011 0:00 | 18 | 49.679191 | -109.561742 | 603758 | 5503956 | 0 | 468.18 | Local |
| F5 | 06/24/2011 0:00 | 0 | 49.681289 | -109.556115 | 604160 | 5504197 | 1 | 13.70 | Local |
| F5 | 06/24/2011 0:00 | 3 | 49.681166 | -109.556134 | 604159 | 5504183 | 1 | 1054.57 | Local |
| F5 | 06/24/2011 0:00 | 6 | 49.690632 | -109.557034 | 604073 | 5505234 | 1 | 374.05 | Local |

| | | | | | | | | | |
|---|---|---|---|---|---|---|---|---|---|
| F5 | 06/24/2011 0:00 | 9 | 49.692667 | -109.552905 | 604367 | 5505466 | 1 | 737.50 | Local |
| F5 | 06/24/2011 0:00 | 12 | 49.693920 | -109.542865 | 605088 | 5505619 | 1 | 18.00 | Local |
| F5 | 06/24/2011 0:00 | 15 | 49.694038 | -109.542693 | 605100 | 5505633 | 1 | 28.61 | Local |
| F5 | 06/24/2011 0:00 | 18 | 49.693831 | -109.542928 | 605084 | 5505609 | 1 | 44.22 | Local |
| F5 | 06/24/2011 0:00 | 21 | 49.694184 | -109.542647 | 605103 | 5505649 | 1 | 21.54 | Local |
| F5 | 06/25/2011 0:00 | 0 | 49.693995 | -109.542712 | 605099 | 5505628 | 1 | 1.27 | Local |
| F5 | 06/25/2011 0:00 | 3 | 49.694006 | -109.542715 | 605099 | 5505629 | 1 | 20.06 | Local |
| F5 | 06/25/2011 0:00 | 6 | 49.693883 | -109.542918 | 605085 | 5505615 | 1 | 28.16 | Local |
| F5 | 06/25/2011 0:00 | 9 | 49.693775 | -109.542565 | 605110 | 5505604 | 0 | 33.46 | Local |
| F5 | 06/25/2011 0:00 | 15 | 49.694035 | -109.542798 | 605093 | 5505632 | 1 | 69.53 | Local |
| F5 | 06/25/2011 0:00 | 18 | 49.694455 | -109.543512 | 605040 | 5505678 | 1 | 70.48 | Local |
| F5 | 06/25/2011 0:00 | 21 | 49.694030 | -109.542788 | 605094 | 5505632 | 1 | 43.55 | Local |
| F5 | 06/26/2011 0:00 | 0 | 49.694323 | -109.543188 | 605064 | 5505664 | 1 | 5.79 | Local |
| F5 | 06/26/2011 0:00 | 3 | 49.694290 | -109.543251 | 605060 | 5505660 | 1 | 539.01 | Local |
| F5 | 06/26/2011 0:00 | 6 | 49.692305 | -109.536434 | 605556 | 5505449 | 1 | 43.19 | Local |
| F5 | 06/26/2011 0:00 | 9 | 49.692618 | -109.536080 | 605580 | 5505484 | 1 | 721.37 | Local |
| F5 | 06/26/2011 0:00 | 12 | 49.695736 | -109.527310 | 606206 | 5505843 | 0 | 1008.23 | Local |
| F5 | 06/26/2011 0:00 | 21 | 49.692384 | -109.514323 | 607150 | 5505489 | 1 | 8.36 | Local |
| F5 | 06/27/2011 0:00 | 0 | 49.692459 | -109.514325 | 607150 | 5505497 | 1 | 32.45 | Local |
| F5 | 06/27/2011 0:00 | 3 | 49.692530 | -109.513889 | 607181 | 5505506 | 1 | 15.81 | Local |
| F5 | 06/27/2011 0:00 | 6 | 49.692670 | -109.513928 | 607178 | 5505521 | 1 | 19.94 | Local |
| F5 | 06/27/2011 0:00 | 9 | 49.692604 | -109.514186 | 607159 | 5505514 | 0 | 256.63 | Local |
| F5 | 06/27/2011 0:00 | 21 | 49.692444 | -109.510636 | 607416 | 5505501 | 1 | 244.25 | Local |
| F5 | 06/28/2011 0:00 | 0 | 49.692763 | -109.513987 | 607173 | 5505532 | 1 | 29.58 | Local |
| F5 | 06/28/2011 0:00 | 3 | 49.692509 | -109.514109 | 607165 | 5505503 | 1 | 171.97 | Local |
| F5 | 06/28/2011 0:00 | 6 | 49.692713 | -109.511745 | 607335 | 5505529 | 0 | 225.26 | Local |
| F5 | 06/28/2011 0:00 | 12 | 49.694705 | -109.511176 | 607372 | 5505751 | 1 | 5.81 | Local |
| F5 | 06/28/2011 0:00 | 15 | 49.694724 | -109.511101 | 607377 | 5505754 | 0 | 380.03 | Local |
| F5 | 06/28/2011 0:00 | 21 | 49.697817 | -109.513343 | 607209 | 5506094 | 1 | 562.32 | Local |
| F5 | 06/29/2011 0:00 | 0 | 49.692785 | -109.514117 | 607164 | 5505534 | 0 | 1687.77 | Local |
| F5 | 06/29/2011 0:00 | 9 | 49.677649 | -109.512370 | 607323 | 5503854 | 0 | 2012.76 | Local |
| F5 | 06/29/2011 0:00 | 21 | 49.678821 | -109.484534 | 609329 | 5504024 | 1 | 291.01 | Local |
| F5 | 06/30/2011 0:00 | 0 | 49.680015 | -109.480945 | 609585 | 5504162 | 1 | 132.20 | Local |
| F5 | 06/30/2011 0:00 | 3 | 49.679522 | -109.479278 | 609706 | 5504110 | 1 | 547.74 | Local |
| F5 | 06/30/2011 0:00 | 6 | 49.682615 | -109.473369 | 610126 | 5504462 | 1 | 45.52 | Local |
| F5 | 06/30/2011 0:00 | 9 | 49.683024 | -109.473382 | 610124 | 5504508 | 0 | 51.44 | Local |
| F5 | 06/30/2011 0:00 | 18 | 49.682580 | -109.473583 | 610110 | 5504458 | 1 | 10.10 | Local |
| F5 | 06/30/2011 0:00 | 21 | 49.682490 | -109.473594 | 610110 | 5504448 | 1 | 49.75 | Local |
| F5 | 07/01/2011 0:00 | 0 | 49.682933 | -109.473502 | 610115 | 5504497 | 1 | 18.26 | Local |
| F5 | 07/01/2011 0:00 | 3 | 49.682771 | -109.473541 | 610113 | 5504479 | 1 | 62.10 | Local |
| F5 | 07/01/2011 0:00 | 6 | 49.682393 | -109.472906 | 610160 | 5504438 | 1 | 131.89 | Local |
| F5 | 07/01/2011 0:00 | 9 | 49.681391 | -109.473883 | 610091 | 5504325 | 1 | 7.29 | Local |
| F5 | 07/01/2011 0:00 | 12 | 49.681326 | -109.473902 | 610090 | 5504318 | 1 | 164.40 | Local |
| F5 | 07/01/2011 0:00 | 15 | 49.682789 | -109.474236 | 610063 | 5504480 | 0 | 52.65 | Local |
| F5 | 07/01/2011 0:00 | 21 | 49.682538 | -109.473617 | 610108 | 5504453 | 1 | 18.90 | Local |
| F5 | 07/02/2011 0:00 | 0 | 49.682708 | -109.473628 | 610107 | 5504472 | 1 | 9.46 | Local |
| F5 | 07/02/2011 0:00 | 3 | 49.682625 | -109.473659 | 610105 | 5504463 | 0 | 314.72 | Local |
| F5 | 07/02/2011 0:00 | 12 | 49.685451 | -109.473895 | 610081 | 5504777 | 0 | 281.19 | Local |
| F5 | 07/02/2011 0:00 | 18 | 49.682922 | -109.473904 | 610086 | 5504496 | 1 | 34.70 | Local |
| F5 | 07/02/2011 0:00 | 21 | 49.682643 | -109.473691 | 610102 | 5504465 | 1 | 2.17 | Local |
| F5 | 07/03/2011 0:00 | 0 | 49.682659 | -109.473708 | 610101 | 5504466 | 1 | 8.79 | Local |
| F5 | 07/03/2011 0:00 | 3 | 49.682622 | -109.473600 | 610109 | 5504463 | 1 | 76.92 | Local |
| F5 | 07/03/2011 0:00 | 6 | 49.682075 | -109.472949 | 610157 | 5504403 | 1 | 222.60 | Local |
| F5 | 07/03/2011 0:00 | 9 | 49.683967 | -109.471941 | 610226 | 5504614 | 1 | 33.47 | Local |
| F5 | 07/03/2011 0:00 | 12 | 49.683719 | -109.471677 | 610245 | 5504587 | 1 | 7.87 | Local |

| | | | | | | | | | |
|---|---|---|---|---|---|---|---|---|---|
| F5 | 07/03/2011 0:00 | 15 | 49.683719 | -109.471568 | 610253 | 5504588 | 1 | 108.75 | Local |
| F5 | 07/03/2011 0:00 | 18 | 49.684679 | -109.471860 | 610230 | 5504694 | 1 | 937.30 | Local |
| F5 | 07/03/2011 0:00 | 21 | 49.677055 | -109.466319 | 610647 | 5503854 | 1 | 705.50 | Local |
| F5 | 07/04/2011 0:00 | 0 | 49.670840 | -109.464350 | 610803 | 5503166 | 1 | 707.56 | Local |
| F5 | 07/04/2011 0:00 | 3 | 49.670398 | -109.474131 | 610098 | 5503103 | 1 | 986.65 | Local |
| F5 | 07/04/2011 0:00 | 6 | 49.664884 | -109.484841 | 609338 | 5502474 | 1 | 237.47 | Local |
| F5 | 07/04/2011 0:00 | 9 | 49.662760 | -109.485175 | 609319 | 5502238 | 1 | 194.26 | Local |
| F5 | 07/04/2011 0:00 | 12 | 49.663688 | -109.487455 | 609152 | 5502337 | 1 | 31.05 | Local |
| F5 | 07/04/2011 0:00 | 15 | 49.663417 | -109.487562 | 609145 | 5502307 | 1 | 262.33 | Local |
| F5 | 07/04/2011 0:00 | 18 | 49.662261 | -109.484395 | 609376 | 5502183 | 1 | 832.97 | Local |
| F5 | 07/04/2011 0:00 | 21 | 49.658658 | -109.494512 | 608654 | 5501768 | 1 | 1327.72 | Local |
| F5 | 07/05/2011 0:00 | 0 | 49.670357 | -109.498194 | 608362 | 5503063 | 1 | 995.99 | Local |
| F5 | 07/05/2011 0:00 | 3 | 49.673916 | -109.510859 | 607441 | 5503441 | 0 | 3162.00 | Local |
| F5 | 07/05/2011 0:00 | 9 | 49.682460 | -109.552657 | 604407 | 5504332 | 1 | 25.04 | Local |
| F5 | 07/05/2011 0:00 | 12 | 49.682279 | -109.552862 | 604392 | 5504311 | 1 | 24.37 | Local |
| F5 | 07/05/2011 0:00 | 15 | 49.682497 | -109.552824 | 604395 | 5504336 | 1 | 59.15 | Local |
| F5 | 07/05/2011 0:00 | 18 | 49.681966 | -109.552763 | 604400 | 5504277 | 1 | 36.52 | Local |
| F5 | 07/05/2011 0:00 | 21 | 49.682079 | -109.552288 | 604434 | 5504290 | 1 | 29.27 | Local |
| F5 | 07/06/2011 0:00 | 0 | 49.681874 | -109.552542 | 604416 | 5504267 | 1 | 41.29 | Local |
| F5 | 07/06/2011 0:00 | 3 | 49.682054 | -109.553043 | 604380 | 5504286 | 1 | 28.54 | Local |
| F5 | 07/06/2011 0:00 | 6 | 49.682241 | -109.552773 | 604399 | 5504307 | 1 | 82.80 | Local |
| F5 | 07/06/2011 0:00 | 9 | 49.682975 | -109.552965 | 604383 | 5504389 | 1 | 27.14 | Local |
| F5 | 07/06/2011 0:00 | 12 | 49.682751 | -109.552814 | 604395 | 5504364 | 1 | 5.18 | Local |
| F5 | 07/06/2011 0:00 | 15 | 49.682734 | -109.552881 | 604390 | 5504362 | 1 | 75.56 | Local |
| F5 | 07/06/2011 0:00 | 18 | 49.682056 | -109.552807 | 604397 | 5504287 | 0 | 741.65 | Local |
| F5 | 07/07/2011 0:00 | 0 | 49.679421 | -109.543364 | 605084 | 5504007 | 0 | 2033.52 | Local |
| F5 | 07/07/2011 0:00 | 6 | 49.661170 | -109.541557 | 605253 | 5501980 | 0 | 2805.39 | Local |
| F5 | 07/07/2011 0:00 | 12 | 49.678103 | -109.512739 | 607296 | 5503904 | 1 | 41.87 | Local |
| F5 | 07/07/2011 0:00 | 15 | 49.678478 | -109.512789 | 607291 | 5503945 | 1 | 673.27 | Local |
| F5 | 07/07/2011 0:00 | 18 | 49.684358 | -109.510562 | 607439 | 5504602 | 1 | 1866.42 | Local |
| F5 | 07/07/2011 0:00 | 21 | 49.699243 | -109.522521 | 606544 | 5506240 | 1 | 558.25 | Local |
| F5 | 07/08/2011 0:00 | 0 | 49.696657 | -109.529156 | 606071 | 5505943 | 1 | 152.67 | Local |
| F5 | 07/08/2011 0:00 | 3 | 49.695289 | -109.529329 | 606061 | 5505791 | 1 | 6.62 | Local |
| F5 | 07/08/2011 0:00 | 6 | 49.695285 | -109.529421 | 606055 | 5505790 | 1 | 744.35 | Local |
| F5 | 07/08/2011 0:00 | 9 | 49.698986 | -109.520821 | 606667 | 5506214 | 1 | 1010.16 | Local |
| F5 | 07/08/2011 0:00 | 12 | 49.705331 | -109.510797 | 607376 | 5506933 | 1 | 24.56 | Local |
| F5 | 07/08/2011 0:00 | 15 | 49.705166 | -109.510570 | 607392 | 5506915 | 1 | 15.62 | Local |
| F5 | 07/08/2011 0:00 | 18 | 49.705053 | -109.510699 | 607383 | 5506903 | 0 | 1338.20 | Local |
| F5 | 07/09/2011 0:00 | 0 | 49.697753 | -109.525452 | 606336 | 5506070 | 1 | 1315.37 | Local |
| F5 | 07/09/2011 0:00 | 3 | 49.687514 | -109.534584 | 605699 | 5504919 | 1 | 1178.20 | Local |
| F5 | 07/09/2011 0:00 | 6 | 49.683576 | -109.549745 | 604614 | 5504460 | 1 | 638.06 | Local |
| F5 | 07/09/2011 0:00 | 9 | 49.686860 | -109.556998 | 604084 | 5504815 | 1 | 7.50 | Local |
| F5 | 07/09/2011 0:00 | 12 | 49.686927 | -109.556995 | 604084 | 5504822 | 1 | 7.03 | Local |
| F5 | 07/09/2011 0:00 | 15 | 49.686959 | -109.556911 | 604090 | 5504826 | 1 | 11.12 | Local |
| F5 | 07/09/2011 0:00 | 18 | 49.686916 | -109.557050 | 604080 | 5504821 | 1 | 956.24 | Local |
| F5 | 07/09/2011 0:00 | 21 | 49.695418 | -109.555054 | 604206 | 5505769 | 0 | 797.53 | Local |
| F5 | 07/10/2011 0:00 | 3 | 49.694765 | -109.544043 | 605001 | 5505712 | 1 | 22.62 | Local |
| F5 | 07/10/2011 0:00 | 6 | 49.694968 | -109.544063 | 605000 | 5505734 | 1 | 9.51 | Local |
| F5 | 07/10/2011 0:00 | 9 | 49.694914 | -109.544165 | 604992 | 5505728 | 1 | 5.52 | Local |
| F5 | 07/10/2011 0:00 | 12 | 49.694875 | -109.544213 | 604989 | 5505724 | 1 | 10.37 | Local |
| F5 | 07/10/2011 0:00 | 15 | 49.694877 | -109.544069 | 604999 | 5505724 | 1 | 16.13 | Local |
| F5 | 07/10/2011 0:00 | 18 | 49.694785 | -109.544243 | 604987 | 5505714 | 1 | 13.76 | Local |
| F5 | 07/10/2011 0:00 | 21 | 49.694855 | -109.544085 | 604998 | 5505722 | 1 | 16.13 | Local |
| F5 | 07/11/2011 0:00 | 0 | 49.694971 | -109.544219 | 604988 | 5505734 | 1 | 7.32 | Local |
| F5 | 07/11/2011 0:00 | 3 | 49.694950 | -109.544315 | 604981 | 5505732 | 1 | 33.29 | Local |

| | | | | | | | | | |
|---|---|---|---|---|---|---|---|---|---|
| F5 | 07/11/2011 0:00 | 6 | 49.694980 | -109.543856 | 605015 | 5505736 | 1 | 92.50 | Local |
| F5 | 07/11/2011 0:00 | 9 | 49.694856 | -109.545124 | 604923 | 5505720 | 1 | 58.95 | Local |
| F5 | 07/11/2011 0:00 | 12 | 49.694924 | -109.544314 | 604982 | 5505729 | 1 | 8.68 | Local |
| F5 | 07/11/2011 0:00 | 15 | 49.694943 | -109.544197 | 604990 | 5505731 | 1 | 9.05 | Local |
| F5 | 07/11/2011 0:00 | 18 | 49.694864 | -109.544169 | 604992 | 5505722 | 1 | 10.65 | Local |
| F5 | 07/11/2011 0:00 | 21 | 49.694942 | -109.544255 | 604986 | 5505731 | 1 | 389.36 | Local |
| F5 | 07/12/2011 0:00 | 0 | 49.691460 | -109.544833 | 604952 | 5505343 | 1 | 2.49 | Local |
| F5 | 07/12/2011 0:00 | 3 | 49.691442 | -109.544814 | 604953 | 5505341 | 1 | 1201.10 | Local |
| F5 | 07/12/2011 0:00 | 6 | 49.690425 | -109.528237 | 606151 | 5505251 | 1 | 1459.52 | Local |
| F5 | 07/12/2011 0:00 | 9 | 49.689923 | -109.508019 | 607610 | 5505224 | 0 | 428.17 | Local |
| F5 | 07/12/2011 0:00 | 15 | 49.692305 | -109.503355 | 607941 | 5505496 | 1 | 439.22 | Local |
| F5 | 07/12/2011 0:00 | 18 | 49.693380 | -109.497496 | 608361 | 5505624 | 1 | 1444.92 | Local |
| F5 | 07/12/2011 0:00 | 21 | 49.685180 | -109.481958 | 609500 | 5504735 | 1 | 1261.47 | Local |
| F5 | 07/13/2011 0:00 | 0 | 49.675307 | -109.490570 | 608901 | 5503625 | 1 | 1243.96 | Local |
| F5 | 07/13/2011 0:00 | 3 | 49.667227 | -109.502493 | 608059 | 5502709 | 1 | 2673.22 | Local |
| F5 | 07/13/2011 0:00 | 6 | 49.643309 | -109.498752 | 608382 | 5500056 | 1 | 1540.03 | Local |
| F5 | 07/13/2011 0:00 | 9 | 49.632705 | -109.485035 | 609396 | 5498896 | 1 | 1669.70 | Local |
| F5 | 07/13/2011 0:00 | 12 | 49.617692 | -109.484541 | 609465 | 5497228 | 1 | 31.76 | Local |
| F5 | 07/13/2011 0:00 | 15 | 49.617411 | -109.484622 | 609460 | 5497197 | 1 | 399.65 | Local |
| F5 | 07/13/2011 0:00 | 18 | 49.613903 | -109.485824 | 609381 | 5496805 | 0 | 1107.81 | Local |
| F5 | 07/14/2011 0:00 | 0 | 49.622589 | -109.478313 | 609904 | 5497782 | 1 | 40.99 | Local |
| F5 | 07/14/2011 0:00 | 3 | 49.622234 | -109.478161 | 609916 | 5497742 | 1 | 2438.61 | Local |
| F5 | 07/14/2011 0:00 | 6 | 49.644110 | -109.475781 | 610038 | 5500178 | 1 | 1035.52 | Local |
| F5 | 07/14/2011 0:00 | 9 | 49.650893 | -109.465954 | 610733 | 5500946 | 1 | 228.60 | Local |
| F5 | 07/14/2011 0:00 | 12 | 49.652417 | -109.468079 | 610576 | 5501113 | 1 | 848.99 | Local |
| F5 | 07/14/2011 0:00 | 15 | 49.656003 | -109.478462 | 609818 | 5501496 | 1 | 4.94 | Local |
| F5 | 07/14/2011 0:00 | 18 | 49.656047 | -109.478467 | 609818 | 5501501 | 1 | 7.06 | Local |
| F5 | 07/14/2011 0:00 | 21 | 49.656107 | -109.478500 | 609815 | 5501508 | 0 | 8.84 | Local |
| F5 | 07/15/2011 0:00 | 3 | 49.656032 | -109.478543 | 609812 | 5501499 | 1 | 64.83 | Local |
| F5 | 07/15/2011 0:00 | 6 | 49.656548 | -109.478964 | 609781 | 5501556 | 1 | 55.41 | Local |
| F5 | 07/15/2011 0:00 | 9 | 49.656155 | -109.478491 | 609816 | 5501513 | 1 | 6.11 | Local |
| F5 | 07/15/2011 0:00 | 12 | 49.656198 | -109.478543 | 609812 | 5501518 | 1 | 15.52 | Local |
| F5 | 07/15/2011 0:00 | 15 | 49.656112 | -109.478712 | 609800 | 5501508 | 1 | 31.55 | Local |
| F5 | 07/15/2011 0:00 | 18 | 49.656361 | -109.478503 | 609814 | 5501536 | 1 | 39.77 | Local |
| F5 | 07/15/2011 0:00 | 21 | 49.656697 | -109.478689 | 609800 | 5501573 | 0 | 86.94 | Local |
| F5 | 07/16/2011 0:00 | 3 | 49.655931 | -109.478447 | 609819 | 5501488 | 1 | 26.34 | Local |
| F5 | 07/16/2011 0:00 | 6 | 49.656126 | -109.478655 | 609804 | 5501510 | 1 | 29.43 | Local |
| F5 | 07/16/2011 0:00 | 9 | 49.656388 | -109.478590 | 609808 | 5501539 | 1 | 12.04 | Local |
| F5 | 07/16/2011 0:00 | 12 | 49.656282 | -109.478554 | 609811 | 5501527 | 1 | 67.18 | Local |
| F5 | 07/16/2011 0:00 | 15 | 49.655826 | -109.477943 | 609856 | 5501477 | 1 | 92.92 | Local |
| F5 | 07/16/2011 0:00 | 18 | 49.656250 | -109.479053 | 609775 | 5501523 | 1 | 9.27 | Local |
| F5 | 07/16/2011 0:00 | 21 | 49.656173 | -109.479004 | 609779 | 5501514 | 1 | 6.24 | Local |
| F5 | 07/17/2011 0:00 | 0 | 49.656227 | -109.479028 | 609777 | 5501520 | 1 | 1.78 | Local |
| F5 | 07/17/2011 0:00 | 3 | 49.656233 | -109.479006 | 609778 | 5501521 | 1 | 5.76 | Local |
| F5 | 07/17/2011 0:00 | 6 | 49.656182 | -109.479010 | 609778 | 5501515 | 1 | 53.05 | Local |
| F5 | 07/17/2011 0:00 | 9 | 49.656134 | -109.478279 | 609831 | 5501511 | 1 | 8.30 | Local |
| F5 | 07/17/2011 0:00 | 12 | 49.656148 | -109.478166 | 609839 | 5501513 | 1 | 30.47 | Local |
| F5 | 07/17/2011 0:00 | 15 | 49.656034 | -109.478550 | 609812 | 5501499 | 0 | 17.44 | Local |
| F5 | 07/17/2011 0:00 | 21 | 49.655984 | -109.478321 | 609828 | 5501494 | 1 | 20.82 | Local |
| F5 | 07/18/2011 0:00 | 0 | 49.656162 | -109.478233 | 609834 | 5501514 | 1 | 29.54 | Local |
| F5 | 07/18/2011 0:00 | 3 | 49.656018 | -109.478576 | 609810 | 5501498 | 1 | 23.34 | Local |
| F5 | 07/18/2011 0:00 | 6 | 49.655892 | -109.478318 | 609829 | 5501484 | 1 | 16.72 | Local |
| F5 | 07/18/2011 0:00 | 9 | 49.656038 | -109.478369 | 609825 | 5501500 | 1 | 32.42 | Local |
| F5 | 07/18/2011 0:00 | 12 | 49.655991 | -109.477926 | 609857 | 5501496 | 0 | 24.01 | Local |
| F5 | 07/18/2011 0:00 | 18 | 49.656206 | -109.477900 | 609858 | 5501520 | 1 | 40.79 | Local |

| | | | | | | | | | |
|---|---|---|---|---|---|---|---|---|---|
| F5 | 07/18/2011 0:00 | 21 | 49.656060 | -109.478418 | 609821 | 5501503 | 1 | 17.40 | Local |
| F5 | 07/19/2011 0:00 | 0 | 49.655904 | -109.478415 | 609822 | 5501485 | 1 | 20.39 | Local |
| F5 | 07/19/2011 0:00 | 3 | 49.656086 | -109.478446 | 609819 | 5501505 | 0 | 21.76 | Local |
| F5 | 07/19/2011 0:00 | 12 | 49.656062 | -109.478146 | 609841 | 5501503 | 1 | 15.07 | Local |
| F5 | 07/19/2011 0:00 | 15 | 49.656184 | -109.478053 | 609847 | 5501517 | 1 | 34.55 | Local |
| F5 | 07/19/2011 0:00 | 18 | 49.655947 | -109.478364 | 609825 | 5501490 | 1 | 22.08 | Local |
| F5 | 07/19/2011 0:00 | 21 | 49.655930 | -109.478668 | 609803 | 5501488 | 1 | 41.02 | Local |
| F5 | 07/20/2011 0:00 | 0 | 49.656002 | -109.478111 | 609844 | 5501497 | 1 | 11.75 | Local |
| F5 | 07/20/2011 0:00 | 3 | 49.655897 | -109.478135 | 609842 | 5501485 | 1 | 45.22 | Local |
| F5 | 07/20/2011 0:00 | 6 | 49.655925 | -109.478760 | 609797 | 5501487 | 1 | 74.48 | Local |
| F5 | 07/20/2011 0:00 | 9 | 49.655734 | -109.477770 | 609869 | 5501467 | 0 | 8.71 | Local |
| F5 | 07/20/2011 0:00 | 15 | 49.655662 | -109.477817 | 609865 | 5501459 | 1 | 63.36 | Local |
| F5 | 07/20/2011 0:00 | 18 | 49.655938 | -109.478585 | 609809 | 5501489 | 1 | 46.87 | Local |
| F5 | 07/20/2011 0:00 | 21 | 49.656154 | -109.479142 | 609769 | 5501512 | 1 | 43.33 | Local |
| F5 | 07/21/2011 0:00 | 0 | 49.656283 | -109.478576 | 609809 | 5501527 | 0 | 847.05 | Local |
| F5 | 07/21/2011 0:00 | 6 | 49.663700 | -109.481246 | 609600 | 5502348 | 1 | 18.94 | Local |
| F5 | 07/21/2011 0:00 | 9 | 49.663704 | -109.481508 | 609581 | 5502348 | 1 | 31.30 | Local |
| F5 | 07/21/2011 0:00 | 12 | 49.663465 | -109.481280 | 609598 | 5502322 | 1 | 14.26 | Local |
| F5 | 07/21/2011 0:00 | 15 | 49.663590 | -109.481319 | 609595 | 5502335 | 1 | 14.19 | Local |
| F5 | 07/21/2011 0:00 | 18 | 49.663695 | -109.481207 | 609603 | 5502347 | 1 | 477.47 | Local |
| F5 | 07/21/2011 0:00 | 21 | 49.665974 | -109.486815 | 609193 | 5502592 | 1 | 545.54 | Local |
| F5 | 07/22/2011 0:00 | 0 | 49.664210 | -109.493869 | 608688 | 5502386 | 0 | 3422.56 | Local |
| F5 | 07/22/2011 0:00 | 12 | 49.694983 | -109.494906 | 608545 | 5505806 | 1 | 319.70 | Local |
| F5 | 07/22/2011 0:00 | 15 | 49.695170 | -109.490483 | 608863 | 5505833 | 1 | 1163.66 | Local |
| F5 | 07/22/2011 0:00 | 18 | 49.700409 | -109.504449 | 607844 | 5506395 | 1 | 877.55 | Local |
| F5 | 07/22/2011 0:00 | 21 | 49.703703 | -109.515507 | 607040 | 5506746 | 1 | 1191.53 | Local |
| F5 | 07/23/2011 0:00 | 0 | 49.695904 | -109.526836 | 606240 | 5505862 | 1 | 92.56 | Local |
| F5 | 07/23/2011 0:00 | 3 | 49.695140 | -109.527345 | 606205 | 5505777 | 0 | 2438.25 | Local |
| F5 | 07/23/2011 0:00 | 9 | 49.681084 | -109.553288 | 604364 | 5504178 | 1 | 87.70 | Local |
| F5 | 07/23/2011 0:00 | 12 | 49.680335 | -109.553669 | 604338 | 5504094 | 1 | 14.67 | Local |
| F5 | 07/23/2011 0:00 | 15 | 49.680218 | -109.553763 | 604332 | 5504081 | 0 | 149.18 | Local |
| F5 | 07/23/2011 0:00 | 21 | 49.680617 | -109.551789 | 604473 | 5504128 | 1 | 7.96 | Local |
| F5 | 07/24/2011 0:00 | 0 | 49.680686 | -109.551822 | 604471 | 5504136 | 0 | 36.92 | Local |
| F5 | 07/24/2011 0:00 | 6 | 49.680967 | -109.552095 | 604450 | 5504166 | 1 | 46.72 | Local |
| F5 | 07/24/2011 0:00 | 9 | 49.681377 | -109.552235 | 604439 | 5504212 | 1 | 16.56 | Local |
| F5 | 07/24/2011 0:00 | 12 | 49.681232 | -109.552183 | 604443 | 5504196 | 1 | 26.37 | Local |
| F5 | 07/24/2011 0:00 | 15 | 49.681040 | -109.552398 | 604428 | 5504174 | 1 | 17.66 | Local |
| F5 | 07/24/2011 0:00 | 18 | 49.680882 | -109.552369 | 604431 | 5504157 | 1 | 281.72 | Local |
| F5 | 07/24/2011 0:00 | 21 | 49.678355 | -109.552092 | 604456 | 5503876 | 1 | 355.85 | Local |
| F5 | 07/25/2011 0:00 | 0 | 49.680244 | -109.556072 | 604165 | 5504081 | 1 | 64.04 | Local |
| F5 | 07/25/2011 0:00 | 3 | 49.679687 | -109.556297 | 604150 | 5504018 | 1 | 729.34 | Local |
| F5 | 07/25/2011 0:00 | 6 | 49.684451 | -109.549348 | 604641 | 5504558 | 1 | 1830.39 | Local |
| F5 | 07/25/2011 0:00 | 9 | 49.695548 | -109.530606 | 605969 | 5505818 | 0 | 641.51 | Local |
| F5 | 07/25/2011 0:00 | 18 | 49.700065 | -109.525074 | 606358 | 5506328 | 1 | 873.02 | Local |
| F5 | 07/25/2011 0:00 | 21 | 49.692959 | -109.519929 | 606744 | 5505545 | 1 | 380.89 | Local |
| F5 | 07/26/2011 0:00 | 0 | 49.689661 | -109.521359 | 606649 | 5505176 | 1 | 966.95 | Local |
| F5 | 07/26/2011 0:00 | 3 | 49.684319 | -109.531935 | 605897 | 5504567 | 1 | 659.56 | Local |
| F5 | 07/26/2011 0:00 | 6 | 49.689404 | -109.527228 | 606226 | 5505139 | 1 | 1021.47 | Local |
| F5 | 07/26/2011 0:00 | 9 | 49.695165 | -109.516199 | 607009 | 5505795 | 1 | 756.85 | Local |
| F5 | 07/26/2011 0:00 | 12 | 49.699693 | -109.508363 | 607564 | 5506310 | 1 | 17.43 | Local |
| F5 | 07/26/2011 0:00 | 15 | 49.699763 | -109.508579 | 607548 | 5506317 | 1 | 59.03 | Local |
| F5 | 07/26/2011 0:00 | 18 | 49.699571 | -109.509342 | 607493 | 5506295 | 1 | 1493.72 | Local |
| F5 | 07/26/2011 0:00 | 21 | 49.705113 | -109.528209 | 606121 | 5506884 | 1 | 1040.02 | Local |
| F5 | 07/27/2011 0:00 | 0 | 49.700982 | -109.541147 | 605197 | 5506407 | 1 | 1162.16 | Local |
| F5 | 07/27/2011 0:00 | 3 | 49.695855 | -109.555189 | 604195 | 5505817 | 1 | 741.02 | Local |

| | | | | | | | | | |
|---|---|---|---|---|---|---|---|---|---|
| F5 | 07/27/2011 0:00 | 6 | 49.691565 | -109.547328 | 604771 | 5505351 | 1 | 19.05 | Local |
| F5 | 07/27/2011 0:00 | 9 | 49.691719 | -109.547441 | 604763 | 5505368 | 1 | 30.17 | Local |
| F5 | 07/27/2011 0:00 | 12 | 49.691704 | -109.547023 | 604793 | 5505367 | 1 | 10.08 | Local |
| F5 | 07/27/2011 0:00 | 15 | 49.691708 | -109.547163 | 604783 | 5505368 | 1 | 20.80 | Local |
| F5 | 07/27/2011 0:00 | 18 | 49.691668 | -109.546882 | 604803 | 5505363 | 1 | 24.24 | Local |
| F5 | 07/27/2011 0:00 | 21 | 49.691622 | -109.547210 | 604780 | 5505358 | 0 | 35.77 | Local |
| F5 | 07/28/2011 0:00 | 3 | 49.691929 | -109.547358 | 604769 | 5505392 | 1 | 50.28 | Local |
| F5 | 07/28/2011 0:00 | 6 | 49.691489 | -109.547194 | 604781 | 5505343 | 1 | 18.12 | Local |
| F5 | 07/28/2011 0:00 | 9 | 49.691648 | -109.547136 | 604785 | 5505361 | 1 | 3.19 | Local |
| F5 | 07/28/2011 0:00 | 12 | 49.691641 | -109.547093 | 604788 | 5505360 | 1 | 48.16 | Local |
| F5 | 07/28/2011 0:00 | 15 | 49.692022 | -109.546777 | 604810 | 5505403 | 1 | 12.10 | Local |
| F5 | 07/28/2011 0:00 | 18 | 49.692102 | -109.546890 | 604802 | 5505412 | 1 | 540.08 | Local |
| F5 | 07/28/2011 0:00 | 21 | 49.689445 | -109.553158 | 604356 | 5505108 | 1 | 231.83 | Local |
| F5 | 07/29/2011 0:00 | 0 | 49.689087 | -109.556324 | 604128 | 5505063 | 1 | 170.49 | Local |
| F5 | 07/29/2011 0:00 | 3 | 49.689838 | -109.558385 | 603978 | 5505144 | 1 | 590.28 | Local |
| F5 | 07/29/2011 0:00 | 6 | 49.684859 | -109.555544 | 604193 | 5504594 | 1 | 35.41 | Local |
| F5 | 07/29/2011 0:00 | 9 | 49.685130 | -109.555286 | 604211 | 5504625 | 1 | 7.15 | Local |
| F5 | 07/29/2011 0:00 | 12 | 49.685068 | -109.555310 | 604210 | 5504618 | 1 | 4.79 | Local |
| F5 | 07/29/2011 0:00 | 15 | 49.685041 | -109.555362 | 604206 | 5504615 | 1 | 11.63 | Local |
| F5 | 07/29/2011 0:00 | 18 | 49.685090 | -109.555505 | 604196 | 5504620 | 1 | 640.82 | Local |
| F5 | 07/29/2011 0:00 | 21 | 49.680597 | -109.549942 | 604606 | 5504128 | 1 | 1005.60 | Local |
| F5 | 07/30/2011 0:00 | 0 | 49.675654 | -109.538271 | 605459 | 5503595 | 1 | 1203.15 | Local |
| F5 | 07/30/2011 0:00 | 3 | 49.665517 | -109.532443 | 605902 | 5502476 | 0 | 3528.10 | Local |
| F5 | 07/30/2011 0:00 | 9 | 49.682850 | -109.491491 | 608818 | 5504462 | 1 | 1171.21 | Local |
| F5 | 07/30/2011 0:00 | 12 | 49.686958 | -109.476542 | 609887 | 5504940 | 1 | 477.84 | Local |
| F5 | 07/30/2011 0:00 | 15 | 49.684612 | -109.470993 | 610293 | 5504688 | 0 | 1300.46 | Local |
| F5 | 07/30/2011 0:00 | 21 | 49.696169 | -109.468225 | 610466 | 5505976 | 1 | 1935.30 | Local |
| F5 | 07/31/2011 0:00 | 0 | 49.698994 | -109.494701 | 608550 | 5506252 | 1 | 1370.68 | Local |
| F5 | 07/31/2011 0:00 | 3 | 49.694597 | -109.512455 | 607280 | 5505738 | 1 | 1577.82 | Local |
| F5 | 07/31/2011 0:00 | 6 | 49.693541 | -109.534268 | 605709 | 5505589 | 1 | 1297.12 | Local |
| F5 | 07/31/2011 0:00 | 9 | 49.687176 | -109.549337 | 604636 | 5504861 | 1 | 224.29 | Local |
| F5 | 07/31/2011 0:00 | 12 | 49.688117 | -109.552087 | 604436 | 5504961 | 1 | 15.20 | Local |
| F5 | 07/31/2011 0:00 | 15 | 49.688012 | -109.552222 | 604426 | 5504949 | 1 | 6.94 | Local |
| F5 | 07/31/2011 0:00 | 18 | 49.688027 | -109.552315 | 604419 | 5504951 | 1 | 761.04 | Local |
| F5 | 07/31/2011 0:00 | 21 | 49.693957 | -109.557584 | 604027 | 5505603 | 1 | 689.78 | Local |
| F5 | 08/01/2011 0:00 | 0 | 49.700073 | -109.559181 | 603898 | 5506281 | 1 | 597.63 | Local |
| F5 | 08/01/2011 0:00 | 3 | 49.705322 | -109.557396 | 604016 | 5506867 | 1 | 44.17 | Local |
| F5 | 08/01/2011 0:00 | 6 | 49.705445 | -109.557979 | 603974 | 5506880 | 0 | 709.22 | Local |
| F5 | 08/01/2011 0:00 | 15 | 49.699068 | -109.558203 | 603971 | 5506170 | 1 | 19.99 | Local |
| F5 | 08/01/2011 0:00 | 18 | 49.698895 | -109.558125 | 603977 | 5506151 | 1 | 247.01 | Local |
| F5 | 08/01/2011 0:00 | 21 | 49.696878 | -109.556690 | 604085 | 5505929 | 1 | 322.95 | Local |
| F5 | 08/02/2011 0:00 | 0 | 49.694905 | -109.559976 | 603852 | 5505705 | 1 | 56.79 | Local |
| F5 | 08/02/2011 0:00 | 3 | 49.694766 | -109.559218 | 603907 | 5505691 | 1 | 134.29 | Local |
| F5 | 08/02/2011 0:00 | 6 | 49.695386 | -109.557620 | 604021 | 5505762 | 1 | 13.05 | Local |
| F5 | 08/02/2011 0:00 | 9 | 49.695484 | -109.557520 | 604028 | 5505773 | 1 | 16.22 | Local |
| F5 | 08/02/2011 0:00 | 12 | 49.695356 | -109.557630 | 604020 | 5505759 | 1 | 134.36 | Local |
| F5 | 08/02/2011 0:00 | 15 | 49.694582 | -109.559060 | 603919 | 5505670 | 1 | 49.06 | Local |
| F5 | 08/02/2011 0:00 | 18 | 49.694959 | -109.558708 | 603944 | 5505713 | 1 | 91.48 | Local |
| F5 | 08/02/2011 0:00 | 21 | 49.694247 | -109.559342 | 603899 | 5505633 | 1 | 77.27 | Local |
| F5 | 08/03/2011 0:00 | 0 | 49.694806 | -109.558707 | 603944 | 5505696 | 1 | 27.07 | Local |
| F5 | 08/03/2011 0:00 | 3 | 49.694779 | -109.559080 | 603917 | 5505692 | 1 | 53.03 | Local |
| F5 | 08/03/2011 0:00 | 6 | 49.695206 | -109.559408 | 603892 | 5505739 | 1 | 128.37 | Local |
| F5 | 08/03/2011 0:00 | 9 | 49.695245 | -109.557630 | 604021 | 5505746 | 1 | 24.35 | Local |
| F5 | 08/03/2011 0:00 | 12 | 49.695349 | -109.557926 | 603999 | 5505757 | 0 | 82.42 | Local |
| F5 | 08/03/2011 0:00 | 18 | 49.694846 | -109.558764 | 603940 | 5505700 | 1 | 3.89 | Local |

| | | | | | | | | | |
|---|---|---|---|---|---|---|---|---|---|
| F5 | 08/03/2011 0:00 | 21 | 49.694813 | -109.558780 | 603939 | 5505696 | 1 | 2.46 | Local |
| F5 | 08/04/2011 0:00 | 0 | 49.694791 | -109.558782 | 603939 | 5505694 | 1 | 17.66 | Local |
| F5 | 08/04/2011 0:00 | 3 | 49.694949 | -109.558799 | 603937 | 5505712 | 1 | 29.17 | Local |
| F5 | 08/04/2011 0:00 | 6 | 49.694749 | -109.559061 | 603919 | 5505689 | 1 | 144.60 | Local |
| F5 | 08/04/2011 0:00 | 9 | 49.694586 | -109.557072 | 604062 | 5505674 | 1 | 1349.15 | Local |
| F5 | 08/04/2011 0:00 | 12 | 49.691870 | -109.538842 | 605383 | 5505397 | 1 | 10.75 | Local |
| F5 | 08/04/2011 0:00 | 15 | 49.691961 | -109.538790 | 605386 | 5505407 | 1 | 12.58 | Local |
| F5 | 08/04/2011 0:00 | 18 | 49.691901 | -109.538938 | 605376 | 5505400 | 1 | 1277.03 | Local |
| F5 | 08/04/2011 0:00 | 21 | 49.683821 | -109.526359 | 606301 | 5504520 | 1 | 842.52 | Local |
| F5 | 08/05/2011 0:00 | 0 | 49.677260 | -109.520518 | 606736 | 5503799 | 1 | 1744.67 | Local |
| F5 | 08/05/2011 0:00 | 3 | 49.661589 | -109.521734 | 606683 | 5502055 | 1 | 526.83 | Local |
| F5 | 08/05/2011 0:00 | 6 | 49.657319 | -109.518573 | 606920 | 5501585 | 0 | 2741.73 | Local |
| F5 | 08/05/2011 0:00 | 12 | 49.675513 | -109.492933 | 608730 | 5503644 | 0 | 14.43 | Local |
| F5 | 08/05/2011 0:00 | 18 | 49.675385 | -109.492898 | 608733 | 5503630 | 1 | 915.69 | Local |
| F5 | 08/05/2011 0:00 | 21 | 49.672451 | -109.504755 | 607884 | 5503287 | 1 | 1957.14 | Local |
| F5 | 08/06/2011 0:00 | 0 | 49.657373 | -109.518744 | 606908 | 5501590 | 0 | 116.49 | Local |
| F5 | 08/06/2011 0:00 | 6 | 49.658288 | -109.517960 | 606962 | 5501693 | 1 | 2502.16 | Local |
| F5 | 08/06/2011 0:00 | 9 | 49.672063 | -109.490546 | 608910 | 5503264 | 1 | 13.58 | Local |
| F5 | 08/06/2011 0:00 | 12 | 49.672154 | -109.490419 | 608919 | 5503274 | 1 | 9.74 | Local |
| F5 | 08/06/2011 0:00 | 15 | 49.672099 | -109.490524 | 608912 | 5503268 | 1 | 8.43 | Local |
| F5 | 08/06/2011 0:00 | 18 | 49.672079 | -109.490411 | 608920 | 5503266 | 1 | 207.43 | Local |
| F5 | 08/06/2011 0:00 | 21 | 49.673791 | -109.489267 | 608999 | 5503458 | 1 | 2120.50 | Local |
| F5 | 08/07/2011 0:00 | 0 | 49.665559 | -109.515772 | 607104 | 5502505 | 1 | 147.36 | Local |
| F5 | 08/07/2011 0:00 | 3 | 49.666881 | -109.515923 | 607091 | 5502651 | 1 | 903.97 | Local |
| F5 | 08/07/2011 0:00 | 6 | 49.674742 | -109.512731 | 607304 | 5503530 | 1 | 473.19 | Local |
| F5 | 08/07/2011 0:00 | 9 | 49.678831 | -109.510912 | 607426 | 5503987 | 0 | 220.38 | Local |
| F5 | 08/07/2011 0:00 | 15 | 49.680220 | -109.508734 | 607580 | 5504145 | 1 | 9.24 | Local |
| F5 | 08/07/2011 0:00 | 18 | 49.680282 | -109.508648 | 607586 | 5504152 | 1 | 1347.95 | Local |
| F5 | 08/07/2011 0:00 | 21 | 49.671127 | -109.520893 | 606723 | 5503116 | 1 | 1475.44 | Local |
| F5 | 08/08/2011 0:00 | 0 | 49.657978 | -109.518146 | 606950 | 5501659 | 1 | 51.39 | Local |
| F5 | 08/08/2011 0:00 | 3 | 49.657591 | -109.518536 | 606922 | 5501615 | 0 | 2624.03 | Local |
| F5 | 08/08/2011 0:00 | 9 | 49.680338 | -109.508857 | 607571 | 5504158 | 1 | 37.38 | Local |
| F5 | 08/08/2011 0:00 | 12 | 49.680326 | -109.508339 | 607608 | 5504157 | 0 | 180.01 | Local |
| F5 | 08/08/2011 0:00 | 18 | 49.681786 | -109.509416 | 607527 | 5504318 | 1 | 357.25 | Local |
| F5 | 08/08/2011 0:00 | 21 | 49.679381 | -109.512699 | 607296 | 5504046 | 1 | 2388.78 | Local |
| F5 | 08/09/2011 0:00 | 0 | 49.658158 | -109.517832 | 606972 | 5501679 | 1 | 88.67 | Local |
| F5 | 08/09/2011 0:00 | 3 | 49.657499 | -109.518523 | 606924 | 5501605 | 1 | 2129.26 | Local |
| F5 | 08/09/2011 0:00 | 6 | 49.674254 | -109.504241 | 607917 | 5503488 | 1 | 1021.02 | Local |
| F5 | 08/09/2011 0:00 | 9 | 49.681414 | -109.495381 | 608541 | 5504297 | 1 | 17.19 | Local |
| F5 | 08/09/2011 0:00 | 12 | 49.681521 | -109.495209 | 608553 | 5504309 | 1 | 4.41 | Local |
| F5 | 08/09/2011 0:00 | 15 | 49.681543 | -109.495261 | 608549 | 5504311 | 1 | 11.02 | Local |
| F5 | 08/09/2011 0:00 | 18 | 49.681483 | -109.495383 | 608540 | 5504304 | 1 | 556.64 | Local |
| F5 | 08/09/2011 0:00 | 21 | 49.683571 | -109.488371 | 609041 | 5504546 | 1 | 785.02 | Local |
| F5 | 08/10/2011 0:00 | 0 | 49.680198 | -109.478812 | 609738 | 5504185 | 1 | 3.42 | Local |
| F5 | 08/10/2011 0:00 | 3 | 49.680185 | -109.478769 | 609742 | 5504184 | 1 | 1399.13 | Local |
| F5 | 08/10/2011 0:00 | 6 | 49.690754 | -109.489294 | 608959 | 5505344 | 1 | 894.97 | Local |
| F5 | 08/10/2011 0:00 | 9 | 49.687314 | -109.500510 | 608157 | 5504945 | 1 | 1475.33 | Local |
| F5 | 08/10/2011 0:00 | 12 | 49.700109 | -109.495098 | 608519 | 5506375 | 1 | 8.08 | Local |
| F5 | 08/10/2011 0:00 | 15 | 49.700123 | -109.494988 | 608527 | 5506377 | 1 | 13.08 | Local |
| F5 | 08/10/2011 0:00 | 18 | 49.700080 | -109.495156 | 608515 | 5506372 | 1 | 1419.74 | Local |
| F5 | 08/10/2011 0:00 | 21 | 49.706229 | -109.512409 | 607258 | 5507031 | 1 | 1757.88 | Local |
| F5 | 08/11/2011 0:00 | 0 | 49.702596 | -109.536132 | 605555 | 5506593 | 1 | 887.25 | Local |
| F5 | 08/11/2011 0:00 | 3 | 49.695074 | -109.532027 | 605867 | 5505763 | 1 | 972.26 | Local |
| F5 | 08/11/2011 0:00 | 6 | 49.688044 | -109.524014 | 606461 | 5504993 | 1 | 1040.31 | Local |
| F5 | 08/11/2011 0:00 | 9 | 49.689102 | -109.538342 | 605425 | 5505090 | 1 | 302.10 | Local |

| | | | | | | | | | |
|---|---|---|---|---|---|---|---|---|---|
| F5 | 08/11/2011 0:00 | 12 | 49.691469 | -109.536288 | 605568 | 5505356 | 1 | 373.59 | Local |
| F5 | 08/11/2011 0:00 | 15 | 49.692223 | -109.541335 | 605202 | 5505433 | 1 | 4.39 | Local |
| F5 | 08/11/2011 0:00 | 18 | 49.692260 | -109.541356 | 605201 | 5505437 | 1 | 1138.99 | Local |
| F5 | 08/11/2011 0:00 | 21 | 49.682323 | -109.537520 | 605499 | 5504338 | 1 | 690.75 | Local |
| F5 | 08/12/2011 0:00 | 0 | 49.676577 | -109.533881 | 605774 | 5503704 | 1 | 688.45 | Local |
| F5 | 08/12/2011 0:00 | 3 | 49.671696 | -109.539750 | 605361 | 5503153 | 1 | 1509.48 | Local |
| F5 | 08/12/2011 0:00 | 6 | 49.659168 | -109.547806 | 604807 | 5501749 | 1 | 1572.54 | Local |
| F5 | 08/12/2011 0:00 | 9 | 49.671246 | -109.559141 | 603963 | 5503076 | 1 | 107.24 | Local |
| F5 | 08/12/2011 0:00 | 12 | 49.671831 | -109.560322 | 603876 | 5503139 | 1 | 14.90 | Local |
| F5 | 08/12/2011 0:00 | 15 | 49.671931 | -109.560461 | 603866 | 5503150 | 1 | 529.34 | Local |
| F5 | 08/12/2011 0:00 | 18 | 49.667404 | -109.562734 | 603712 | 5502644 | 1 | 1068.85 | Local |
| F5 | 08/12/2011 0:00 | 21 | 49.658648 | -109.568842 | 603290 | 5501662 | 1 | 1093.48 | Local |
| F5 | 08/13/2011 0:00 | 0 | 49.648850 | -109.570142 | 603216 | 5500571 | 1 | 1149.30 | Local |
| F5 | 08/13/2011 0:00 | 3 | 49.649613 | -109.554266 | 604361 | 5500678 | 0 | 794.57 | Local |
| F5 | 08/13/2011 0:00 | 9 | 49.651588 | -109.564843 | 603593 | 5500883 | 1 | 3.32 | Local |
| F5 | 08/13/2011 0:00 | 12 | 49.651570 | -109.564807 | 603596 | 5500881 | 1 | 245.91 | Local |
| F5 | 08/13/2011 0:00 | 15 | 49.651821 | -109.568191 | 603351 | 5500904 | 1 | 394.12 | Local |
| F5 | 08/13/2011 0:00 | 18 | 49.655241 | -109.566754 | 603447 | 5501286 | 1 | 1171.44 | Local |
| F5 | 08/13/2011 0:00 | 21 | 49.653066 | -109.582632 | 602306 | 5501023 | 1 | 2937.23 | Local |
| F5 | 08/14/2011 0:00 | 0 | 49.650603 | -109.623141 | 599387 | 5500695 | 1 | 506.83 | Local |
| F5 | 08/14/2011 0:00 | 3 | 49.654869 | -109.620666 | 599557 | 5501172 | 1 | 2663.19 | Local |
| F5 | 08/14/2011 0:00 | 6 | 49.668247 | -109.590061 | 601738 | 5502700 | 1 | 881.91 | Local |
| F5 | 08/14/2011 0:00 | 9 | 49.667664 | -109.577874 | 602619 | 5502652 | 1 | 709.15 | Local |
| F5 | 08/14/2011 0:00 | 12 | 49.665531 | -109.568614 | 603291 | 5502428 | 1 | 8.94 | Local |
| F5 | 08/14/2011 0:00 | 15 | 49.665595 | -109.568689 | 603286 | 5502435 | 1 | 73.24 | Local |
| F5 | 08/14/2011 0:00 | 18 | 49.665388 | -109.569652 | 603217 | 5502410 | 1 | 627.08 | Local |
| F5 | 08/14/2011 0:00 | 21 | 49.660842 | -109.564510 | 603598 | 5501912 | 1 | 617.88 | Local |
| F5 | 08/15/2011 0:00 | 0 | 49.656624 | -109.558938 | 604009 | 5501451 | 1 | 1695.99 | Local |
| F5 | 08/15/2011 0:00 | 3 | 49.670673 | -109.549790 | 604639 | 5503025 | 0 | 1520.17 | Local |
| F5 | 08/15/2011 0:00 | 9 | 49.684333 | -109.548935 | 604671 | 5504545 | 1 | 286.98 | Local |
| F5 | 08/15/2011 0:00 | 12 | 49.686905 | -109.548601 | 604690 | 5504831 | 1 | 355.97 | Local |
| F5 | 08/15/2011 0:00 | 15 | 49.689571 | -109.551332 | 604487 | 5505124 | 1 | 338.89 | Local |
| F5 | 08/15/2011 0:00 | 18 | 49.690548 | -109.555782 | 604164 | 5505227 | 1 | 294.00 | Local |
| F5 | 08/15/2011 0:00 | 21 | 49.692681 | -109.553375 | 604333 | 5505467 | 1 | 1949.78 | Local |
| F5 | 08/16/2011 0:00 | 0 | 49.709744 | -109.559611 | 603847 | 5507355 | 1 | 43.40 | Local |
| F5 | 08/16/2011 0:00 | 3 | 49.709353 | -109.559591 | 603849 | 5507312 | 1 | 14.56 | Local |
| F5 | 08/16/2011 0:00 | 6 | 49.709463 | -109.559481 | 603857 | 5507324 | 0 | 16.47 | Local |
| F5 | 08/16/2011 0:00 | 12 | 49.709316 | -109.559459 | 603859 | 5507308 | 1 | 17.39 | Local |
| F5 | 08/16/2011 0:00 | 15 | 49.709463 | -109.559542 | 603852 | 5507324 | 1 | 5.95 | Local |
| F5 | 08/16/2011 0:00 | 18 | 49.709410 | -109.559527 | 603854 | 5507318 | 1 | 7.18 | Local |
| F5 | 08/16/2011 0:00 | 21 | 49.709473 | -109.559549 | 603852 | 5507325 | 1 | 8.16 | Local |
| F5 | 08/17/2011 0:00 | 0 | 49.709408 | -109.559496 | 603856 | 5507318 | 0 | 17.05 | Local |
| F5 | 08/17/2011 0:00 | 6 | 49.709288 | -109.559643 | 603846 | 5507305 | 1 | 1545.62 | Local |
| F5 | 08/17/2011 0:00 | 9 | 49.697754 | -109.547681 | 604733 | 5506039 | 1 | 10.77 | Local |
| F5 | 08/17/2011 0:00 | 12 | 49.697792 | -109.547818 | 604723 | 5506043 | 1 | 7.01 | Local |
| F5 | 08/17/2011 0:00 | 15 | 49.697761 | -109.547734 | 604729 | 5506040 | 1 | 20.60 | Local |
| F5 | 08/17/2011 0:00 | 18 | 49.697628 | -109.547534 | 604744 | 5506025 | 1 | 923.93 | Local |
| F5 | 08/17/2011 0:00 | 21 | 49.691157 | -109.539499 | 605337 | 5505317 | 1 | 1191.87 | Local |
| F5 | 08/18/2011 0:00 | 0 | 49.682261 | -109.530283 | 606021 | 5504341 | 1 | 980.23 | Local |
| F5 | 08/18/2011 0:00 | 3 | 49.674115 | -109.525091 | 606413 | 5503443 | 1 | 2972.67 | Local |
| F5 | 08/18/2011 0:00 | 6 | 49.651421 | -109.503320 | 608034 | 5500951 | 1 | 709.06 | Local |
| F5 | 08/18/2011 0:00 | 9 | 49.652253 | -109.493582 | 608735 | 5501057 | 1 | 789.69 | Local |
| F5 | 08/18/2011 0:00 | 12 | 49.656999 | -109.485445 | 609312 | 5501597 | 1 | 158.54 | Local |
| F5 | 08/18/2011 0:00 | 15 | 49.656728 | -109.483289 | 609468 | 5501570 | 1 | 265.48 | Local |
| F5 | 08/18/2011 0:00 | 18 | 49.656158 | -109.479718 | 609727 | 5501512 | 1 | 1284.78 | Local |

| | | | | | | | | | |
|---|---|---|---|---|---|---|---|---|---|
| F5 | 08/18/2011 0:00 | 21 | 49.657008 | -109.461968 | 611006 | 5501632 | 1 | 1124.81 | Local |
| F5 | 08/19/2011 0:00 | 0 | 49.661026 | -109.447667 | 612029 | 5502100 | 1 | 6.38 | Local |
| F5 | 08/19/2011 0:00 | 3 | 49.660994 | -109.447741 | 612024 | 5502096 | 1 | 3.08 | Local |
| F5 | 08/19/2011 0:00 | 6 | 49.661014 | -109.447711 | 612026 | 5502099 | 1 | 1220.05 | Local |
| F5 | 08/19/2011 0:00 | 9 | 49.652990 | -109.459238 | 611213 | 5501189 | 1 | 8.88 | Local |
| F5 | 08/19/2011 0:00 | 12 | 49.653051 | -109.459160 | 611218 | 5501196 | 1 | 2.05 | Local |
| F5 | 08/19/2011 0:00 | 15 | 49.653033 | -109.459164 | 611218 | 5501194 | 1 | 80.40 | Local |
| F5 | 08/19/2011 0:00 | 18 | 49.652679 | -109.460135 | 611148 | 5501154 | 0 | 1767.75 | Local |
| F5 | 08/20/2011 0:00 | 0 | 49.663482 | -109.478101 | 609827 | 5502328 | 1 | 1373.25 | Local |
| F5 | 08/20/2011 0:00 | 3 | 49.661355 | -109.496843 | 608480 | 5502065 | 1 | 1982.67 | Local |
| F5 | 08/20/2011 0:00 | 6 | 49.675932 | -109.512666 | 607306 | 5503662 | 1 | 1881.25 | Local |
| F5 | 08/20/2011 0:00 | 9 | 49.692408 | -109.518587 | 606842 | 5505486 | 1 | 816.51 | Local |
| F5 | 08/20/2011 0:00 | 12 | 49.699652 | -109.520446 | 606692 | 5506288 | 1 | 28.16 | Local |
| F5 | 08/20/2011 0:00 | 15 | 49.699889 | -109.520307 | 606702 | 5506315 | 0 | 3389.10 | Local |
| F5 | 10/17/2011 0:00 | 3 | 49.683550 | -109.559970 | 603877 | 5504443 | 1 | 2371.39 | Local |
| F5 | 10/17/2011 0:00 | 6 | 49.678600 | -109.528000 | 606194 | 5503937 | 1 | 2209.86 | Local |
| F5 | 10/17/2011 0:00 | 9 | 49.690750 | -109.503760 | 607915 | 5505322 | 1 | 790.89 | Local |
| F5 | 10/17/2011 0:00 | 12 | 49.690940 | -109.492800 | 608705 | 5505359 | 1 | 12.41 | Local |
| F5 | 10/17/2011 0:00 | 15 | 49.690860 | -109.492680 | 608714 | 5505351 | 1 | 328.90 | Local |
| F5 | 10/17/2011 0:00 | 18 | 49.690670 | -109.488130 | 609043 | 5505336 | 1 | 602.30 | Local |
| F5 | 10/17/2011 0:00 | 21 | 49.685350 | -109.489700 | 608942 | 5504742 | 1 | 4.33 | Local |
| F5 | 10/18/2011 0:00 | 0 | 49.685350 | -109.489760 | 608937 | 5504742 | 1 | 1685.06 | Local |
| F5 | 10/18/2011 0:00 | 3 | 49.671880 | -109.500460 | 608195 | 5503229 | 1 | 2824.36 | Local |
| F5 | 10/18/2011 0:00 | 6 | 49.666300 | -109.538640 | 605453 | 5502555 | 1 | 2750.88 | Local |
| F5 | 10/18/2011 0:00 | 9 | 49.682760 | -109.510180 | 607470 | 5504425 | 1 | 79.29 | Local |
| F5 | 10/18/2011 0:00 | 12 | 49.683150 | -109.509260 | 607536 | 5504470 | 1 | 19.03 | Local |
| F5 | 10/18/2011 0:00 | 15 | 49.682980 | -109.509290 | 607534 | 5504451 | 1 | 3.98 | Local |
| F5 | 10/18/2011 0:00 | 18 | 49.682950 | -109.509260 | 607536 | 5504447 | 1 | 2812.52 | Local |
| F5 | 10/18/2011 0:00 | 21 | 49.665510 | -109.537490 | 605537 | 5502469 | 1 | 45.07 | Local |
| F5 | 10/19/2011 0:00 | 0 | 49.665170 | -109.537830 | 605514 | 5502430 | 1 | 19.82 | Local |
| F5 | 10/19/2011 0:00 | 3 | 49.665310 | -109.537660 | 605526 | 5502446 | 1 | 242.22 | Local |
| F5 | 10/19/2011 0:00 | 6 | 49.665670 | -109.540970 | 605286 | 5502481 | 1 | 3001.80 | Local |
| F5 | 10/19/2011 0:00 | 9 | 49.682390 | -109.508310 | 607606 | 5504386 | 1 | 265.70 | Local |
| F5 | 10/19/2011 0:00 | 12 | 49.684660 | -109.509460 | 607518 | 5504637 | 0 | 15.31 | Local |
| F5 | 10/19/2011 0:00 | 18 | 49.684790 | -109.509390 | 607523 | 5504652 | 1 | 198.10 | Local |
| F5 | 10/19/2011 0:00 | 21 | 49.686530 | -109.508800 | 607561 | 5504846 | 1 | 3162.81 | Local |
| F5 | 10/20/2011 0:00 | 0 | 49.665190 | -109.537780 | 605517 | 5502433 | 1 | 45.59 | Local |
| F5 | 10/20/2011 0:00 | 3 | 49.665600 | -109.537780 | 605516 | 5502478 | 0 | 3206.31 | Local |
| F5 | 10/20/2011 0:00 | 9 | 49.687210 | -109.508360 | 607591 | 5504922 | 0 | 12.21 | Local |
| F5 | 10/20/2011 0:00 | 15 | 49.687110 | -109.508290 | 607597 | 5504911 | 1 | 48.06 | Local |
| F5 | 10/20/2011 0:00 | 18 | 49.686990 | -109.507650 | 607643 | 5504899 | 1 | 1997.43 | Local |
| F5 | 10/20/2011 0:00 | 21 | 49.669650 | -109.514880 | 607160 | 5502961 | 1 | 876.83 | Local |
| F5 | 10/21/2011 0:00 | 0 | 49.669970 | -109.502740 | 608035 | 5503014 | 1 | 2111.11 | Local |
| F5 | 10/21/2011 0:00 | 3 | 49.651670 | -109.494950 | 608638 | 5500991 | 1 | 816.67 | Local |
| F5 | 10/21/2011 0:00 | 6 | 49.658500 | -109.490790 | 608923 | 5501756 | 1 | 2002.67 | Local |
| F5 | 10/21/2011 0:00 | 9 | 49.676000 | -109.484230 | 609357 | 5503711 | 1 | 123.26 | Local |
| F5 | 10/21/2011 0:00 | 12 | 49.676310 | -109.485870 | 609238 | 5503743 | 1 | 22.46 | Local |
| F5 | 10/21/2011 0:00 | 15 | 49.676470 | -109.485680 | 609251 | 5503761 | 1 | 311.42 | Local |
| F5 | 10/21/2011 0:00 | 18 | 49.676710 | -109.489980 | 608941 | 5503781 | 1 | 2342.79 | Local |
| F5 | 10/21/2011 0:00 | 21 | 49.666260 | -109.518170 | 606930 | 5502579 | 1 | 1461.39 | Local |
| F5 | 10/22/2011 0:00 | 0 | 49.665450 | -109.538380 | 605473 | 5502461 | 1 | 58.70 | Local |
| F5 | 10/22/2011 0:00 | 3 | 49.665280 | -109.537610 | 605529 | 5502443 | 1 | 17.18 | Local |
| F5 | 10/22/2011 0:00 | 6 | 49.665160 | -109.537760 | 605519 | 5502429 | 1 | 2785.32 | Local |
| F5 | 10/22/2011 0:00 | 9 | 49.682250 | -109.509540 | 607517 | 5504369 | 1 | 10.37 | Local |
| F5 | 10/22/2011 0:00 | 12 | 49.682310 | -109.509650 | 607509 | 5504376 | 1 | 3.10 | Local |

| | | | | | | | | | |
|---|---|---|---|---|---|---|---|---|---|
| F5 | 10/22/2011 0:00 | 15 | 49.682290 | -109.509620 | 607511 | 5504373 | 1 | 298.32 | Local |
| F5 | 10/22/2011 0:00 | 18 | 49.682420 | -109.513750 | 607213 | 5504382 | 1 | 2384.53 | Local |
| F5 | 10/22/2011 0:00 | 21 | 49.667590 | -109.537620 | 605523 | 5502700 | 1 | 275.07 | Local |
| F5 | 10/23/2011 0:00 | 0 | 49.665180 | -109.538480 | 605467 | 5502430 | 1 | 0.72 | Local |
| F5 | 10/23/2011 0:00 | 3 | 49.665180 | -109.538490 | 605466 | 5502430 | 1 | 63.26 | Local |
| F5 | 10/23/2011 0:00 | 6 | 49.665290 | -109.537630 | 605528 | 5502444 | 1 | 2589.02 | Local |
| F5 | 10/23/2011 0:00 | 9 | 49.681780 | -109.512300 | 607319 | 5504313 | 0 | 94.40 | Local |
| F5 | 10/23/2011 0:00 | 15 | 49.682620 | -109.512490 | 607304 | 5504406 | 1 | 437.26 | Local |
| F5 | 10/23/2011 0:00 | 18 | 49.684200 | -109.518040 | 606900 | 5504574 | 1 | 619.51 | Local |
| F5 | 10/23/2011 0:00 | 21 | 49.685500 | -109.526390 | 606295 | 5504706 | 1 | 903.45 | Local |
| F5 | 10/24/2011 0:00 | 0 | 49.677720 | -109.530000 | 606051 | 5503836 | 1 | 1496.26 | Local |
| F5 | 10/24/2011 0:00 | 3 | 49.665260 | -109.537830 | 605513 | 5502440 | 1 | 8.82 | Local |
| F5 | 10/24/2011 0:00 | 6 | 49.665200 | -109.537750 | 605519 | 5502434 | 1 | 1480.12 | Local |
| F5 | 10/24/2011 0:00 | 9 | 49.678140 | -109.532940 | 605838 | 5503879 | 1 | 1465.27 | Local |
| F5 | 10/24/2011 0:00 | 12 | 49.683460 | -109.514360 | 607167 | 5504497 | 1 | 23.89 | Local |
| F5 | 10/24/2011 0:00 | 15 | 49.683250 | -109.514290 | 607172 | 5504474 | 1 | 610.92 | Local |
| F5 | 10/24/2011 0:00 | 18 | 49.688610 | -109.512430 | 607295 | 5505072 | 1 | 432.81 | Local |
| F5 | 10/24/2011 0:00 | 21 | 49.686560 | -109.507330 | 607667 | 5504851 | 1 | 87.01 | Local |
| F5 | 10/25/2011 0:00 | 0 | 49.686750 | -109.508500 | 607582 | 5504871 | 1 | 221.94 | Local |
| F5 | 10/25/2011 0:00 | 3 | 49.685410 | -109.506220 | 607750 | 5504725 | 1 | 286.75 | Local |
| F5 | 10/25/2011 0:00 | 6 | 49.686620 | -109.502710 | 608000 | 5504865 | 1 | 1112.32 | Local |
| F5 | 10/25/2011 0:00 | 9 | 49.695520 | -109.509750 | 607473 | 5505844 | 1 | 520.46 | Local |
| F5 | 10/25/2011 0:00 | 12 | 49.699550 | -109.506080 | 607729 | 5506297 | 1 | 202.05 | Local |
| F5 | 10/25/2011 0:00 | 15 | 49.698730 | -109.503580 | 607911 | 5506210 | 1 | 717.36 | Local |
| F5 | 10/25/2011 0:00 | 18 | 49.705070 | -109.501740 | 608029 | 5506917 | 1 | 2327.86 | Local |
| F5 | 10/25/2011 0:00 | 21 | 49.707400 | -109.469660 | 610337 | 5507223 | 1 | 11.14 | Local |
| F5 | 10/26/2011 0:00 | 0 | 49.707300 | -109.469650 | 610338 | 5507212 | 1 | 2448.43 | Local |
| F5 | 10/26/2011 0:00 | 3 | 49.686060 | -109.460700 | 611032 | 5504864 | 1 | 2.43 | Local |
| F5 | 10/26/2011 0:00 | 6 | 49.686050 | -109.460730 | 611030 | 5504863 | 1 | 1006.10 | Local |
| F5 | 10/26/2011 0:00 | 9 | 49.678090 | -109.467360 | 610569 | 5503968 | 1 | 5.97 | Local |
| F5 | 10/26/2011 0:00 | 12 | 49.678040 | -109.467390 | 610567 | 5503962 | 1 | 2.34 | Local |
| F5 | 10/26/2011 0:00 | 15 | 49.678060 | -109.467400 | 610567 | 5503965 | 1 | 845.91 | Local |
| F5 | 10/26/2011 0:00 | 18 | 49.670870 | -109.463570 | 610859 | 5503171 | 1 | 706.68 | Local |
| F5 | 10/26/2011 0:00 | 21 | 49.677210 | -109.464250 | 610796 | 5503875 | 1 | 276.10 | Local |
| F5 | 10/27/2011 0:00 | 0 | 49.679550 | -109.462970 | 610883 | 5504137 | 1 | 723.79 | Local |
| F5 | 10/27/2011 0:00 | 3 | 49.685980 | -109.461410 | 610981 | 5504854 | 1 | 210.36 | Local |
| F5 | 10/27/2011 0:00 | 6 | 49.687710 | -109.462590 | 610892 | 5505044 | 1 | 259.07 | Local |
| F5 | 10/27/2011 0:00 | 9 | 49.689600 | -109.464690 | 610736 | 5505251 | 1 | 9.28 | Local |
| F5 | 10/27/2011 0:00 | 12 | 49.689570 | -109.464570 | 610745 | 5505248 | 1 | 39.26 | Local |
| F5 | 10/27/2011 0:00 | 15 | 49.689890 | -109.464800 | 610727 | 5505284 | 1 | 667.41 | Local |
| F5 | 10/27/2011 0:00 | 18 | 49.692420 | -109.473190 | 610116 | 5505552 | 1 | 482.44 | Local |
| F5 | 10/27/2011 0:00 | 21 | 49.694110 | -109.479350 | 609668 | 5505731 | 1 | 420.99 | Local |
| F5 | 10/28/2011 0:00 | 0 | 49.695220 | -109.484930 | 609263 | 5505847 | 0 | 6.63 | Local |
| F5 | 10/28/2011 0:00 | 6 | 49.695270 | -109.484980 | 609260 | 5505852 | 1 | 1013.40 | Local |
| F5 | 10/28/2011 0:00 | 9 | 49.686870 | -109.490430 | 608885 | 5504910 | 1 | 214.29 | Local |
| F5 | 10/28/2011 0:00 | 12 | 49.685060 | -109.489410 | 608963 | 5504711 | 1 | 14.46 | Local |
| F5 | 10/28/2011 0:00 | 15 | 49.684930 | -109.489410 | 608963 | 5504696 | 1 | 1026.66 | Local |
| F5 | 10/28/2011 0:00 | 18 | 49.679510 | -109.500930 | 608144 | 5504077 | 1 | 962.55 | Local |
| F5 | 10/28/2011 0:00 | 21 | 49.686950 | -109.507750 | 607636 | 5504894 | 1 | 2586.57 | Local |
| F5 | 10/29/2011 0:00 | 0 | 49.709440 | -109.498590 | 608247 | 5507408 | 1 | 1450.74 | Local |
| F5 | 10/29/2011 0:00 | 3 | 49.722330 | -109.495480 | 608442 | 5508845 | 1 | 1289.88 | Local |
| F5 | 10/29/2011 0:00 | 6 | 49.711330 | -109.501160 | 608057 | 5507614 | 1 | 9.63 | Local |
| F5 | 10/29/2011 0:00 | 9 | 49.711310 | -109.501290 | 608048 | 5507612 | 1 | 118.16 | Local |
| F5 | 10/29/2011 0:00 | 12 | 49.710390 | -109.502110 | 607991 | 5507508 | 1 | 480.76 | Local |
| F5 | 10/29/2011 0:00 | 15 | 49.707810 | -109.507460 | 607611 | 5507214 | 1 | 1543.95 | Local |

| | | | | | | | | | |
|---|---|---|---|---|---|---|---|---|---|
| F5 | 10/29/2011 0:00 | 18 | 49.704440 | -109.528230 | 606121 | 5506810 | 1 | 1632.43 | Local |
| F5 | 10/29/2011 0:00 | 21 | 49.700880 | -109.550190 | 604545 | 5506383 | 1 | 566.31 | Local |
| F5 | 10/30/2011 0:00 | 0 | 49.702110 | -109.557810 | 603993 | 5506509 | 1 | 687.98 | Local |
| F5 | 10/30/2011 0:00 | 3 | 49.695930 | -109.557350 | 604039 | 5505823 | 1 | 1713.59 | Local |
| F5 | 10/30/2011 0:00 | 6 | 49.680520 | -109.557610 | 604053 | 5504109 | 1 | 163.93 | Local |
| F5 | 10/30/2011 0:00 | 9 | 49.681810 | -109.556510 | 604130 | 5504254 | 1 | 13.72 | Local |
| F5 | 10/30/2011 0:00 | 12 | 49.681720 | -109.556640 | 604121 | 5504244 | 1 | 8.55 | Local |
| F5 | 10/30/2011 0:00 | 15 | 49.681670 | -109.556730 | 604115 | 5504238 | 1 | 1557.56 | Local |
| F5 | 10/30/2011 0:00 | 18 | 49.677070 | -109.577120 | 602653 | 5503699 | 1 | 1111.49 | Local |
| F5 | 10/30/2011 0:00 | 21 | 49.676160 | -109.592460 | 601549 | 5503577 | 1 | 1445.90 | Local |
| F5 | 10/31/2011 0:00 | 0 | 49.665830 | -109.604630 | 600692 | 5502412 | 1 | 846.28 | Local |
| F5 | 10/31/2011 0:00 | 3 | 49.658810 | -109.609160 | 600379 | 5501625 | 1 | 3.63 | Local |
| F5 | 10/31/2011 0:00 | 6 | 49.658780 | -109.609180 | 600378 | 5501622 | 1 | 5.88 | Local |
| F5 | 10/31/2011 0:00 | 9 | 49.658790 | -109.609260 | 600372 | 5501623 | 1 | 7.27 | Local |
| F5 | 10/31/2011 0:00 | 12 | 49.658850 | -109.609220 | 600375 | 5501630 | 1 | 286.65 | Local |
| F5 | 10/31/2011 0:00 | 15 | 49.657790 | -109.612840 | 600116 | 5501507 | 1 | 351.25 | Local |
| F5 | 10/31/2011 0:00 | 18 | 49.656130 | -109.616980 | 599821 | 5501317 | 1 | 619.54 | Local |
| F5 | 10/31/2011 0:00 | 21 | 49.650790 | -109.619430 | 599655 | 5500720 | 1 | 342.97 | Local |
| F5 | 11/01/2011 0:00 | 0 | 49.651870 | -109.614980 | 599974 | 5500846 | 1 | 211.15 | Local |
| F5 | 11/01/2011 0:00 | 3 | 49.651140 | -109.612280 | 600170 | 5500769 | 1 | 24.08 | Local |
| F5 | 11/01/2011 0:00 | 6 | 49.650950 | -109.612120 | 600182 | 5500748 | 1 | 26.93 | Local |
| F5 | 11/01/2011 0:00 | 9 | 49.650790 | -109.612400 | 600162 | 5500730 | 1 | 7.95 | Local |
| F5 | 11/01/2011 0:00 | 12 | 49.650820 | -109.612300 | 600169 | 5500733 | 1 | 346.89 | Local |
| F5 | 11/01/2011 0:00 | 15 | 49.653360 | -109.609510 | 600365 | 5501019 | 1 | 12.72 | Local |
| F5 | 11/01/2011 0:00 | 18 | 49.653390 | -109.609680 | 600353 | 5501022 | 1 | 3.10 | Local |
| F5 | 11/01/2011 0:00 | 21 | 49.653370 | -109.609710 | 600351 | 5501020 | 1 | 11.12 | Local |
| F5 | 11/02/2011 0:00 | 0 | 49.653470 | -109.609710 | 600351 | 5501031 | 1 | 6.83 | Local |
| F5 | 11/02/2011 0:00 | 3 | 49.653410 | -109.609690 | 600352 | 5501024 | 1 | 2.34 | Local |
| F5 | 11/02/2011 0:00 | 6 | 49.653430 | -109.609680 | 600353 | 5501027 | 1 | 4.95 | Local |
| F5 | 11/02/2011 0:00 | 9 | 49.653390 | -109.609710 | 600351 | 5501022 | 1 | 465.19 | Local |
| F5 | 11/02/2011 0:00 | 12 | 49.652250 | -109.603510 | 600801 | 5500904 | 1 | 3.61 | Local |
| F5 | 11/02/2011 0:00 | 15 | 49.652250 | -109.603560 | 600797 | 5500904 | 1 | 62.32 | Local |
| F5 | 11/02/2011 0:00 | 18 | 49.652640 | -109.602940 | 600841 | 5500948 | 1 | 56.35 | Local |
| F5 | 11/02/2011 0:00 | 21 | 49.652660 | -109.602160 | 600897 | 5500951 | 0 | 7.59 | Local |
| F5 | 11/03/2011 0:00 | 3 | 49.652600 | -109.602210 | 600894 | 5500944 | 1 | 100.28 | Local |
| F5 | 11/03/2011 0:00 | 6 | 49.652300 | -109.603520 | 600800 | 5500909 | 1 | 0.72 | Local |
| F5 | 11/03/2011 0:00 | 9 | 49.652300 | -109.603530 | 600799 | 5500909 | 1 | 6.63 | Local |
| F5 | 11/03/2011 0:00 | 12 | 49.652250 | -109.603580 | 600796 | 5500904 | 1 | 33.65 | Local |
| F5 | 11/03/2011 0:00 | 15 | 49.652030 | -109.603900 | 600773 | 5500879 | 1 | 68.48 | Local |
| F5 | 11/03/2011 0:00 | 18 | 49.651800 | -109.604780 | 600710 | 5500852 | 1 | 58.19 | Local |
| F5 | 11/03/2011 0:00 | 21 | 49.652170 | -109.604210 | 600750 | 5500894 | 1 | 4.95 | Local |
| F5 | 11/04/2011 0:00 | 0 | 49.652130 | -109.604240 | 600748 | 5500889 | 0 | 15.11 | Local |
| F5 | 11/04/2011 0:00 | 6 | 49.652090 | -109.604040 | 600763 | 5500885 | 1 | 161.92 | Local |
| F5 | 11/04/2011 0:00 | 9 | 49.652950 | -109.605850 | 600630 | 5500978 | 1 | 2.43 | Local |
| F5 | 11/04/2011 0:00 | 12 | 49.652960 | -109.605880 | 600628 | 5500979 | 1 | 12.02 | Local |
| F5 | 11/04/2011 0:00 | 15 | 49.652930 | -109.605720 | 600640 | 5500976 | 1 | 144.49 | Local |
| F5 | 11/04/2011 0:00 | 18 | 49.653410 | -109.607580 | 600505 | 5501027 | 1 | 132.85 | Local |
| F5 | 11/04/2011 0:00 | 21 | 49.653430 | -109.609420 | 600372 | 5501027 | 1 | 11.02 | Local |
| F5 | 11/05/2011 0:00 | 0 | 49.653350 | -109.609510 | 600365 | 5501018 | 1 | 8.08 | Local |
| F5 | 11/05/2011 0:00 | 3 | 49.653420 | -109.609480 | 600367 | 5501026 | 1 | 19.79 | Local |
| F5 | 11/05/2011 0:00 | 6 | 49.653260 | -109.609600 | 600359 | 5501008 | 1 | 19.77 | Local |
| F5 | 11/05/2011 0:00 | 9 | 49.653430 | -109.609680 | 600353 | 5501027 | 1 | 3.09 | Local |
| F5 | 11/05/2011 0:00 | 12 | 49.653420 | -109.609640 | 600356 | 5501026 | 1 | 4.87 | Local |
| F5 | 11/05/2011 0:00 | 15 | 49.653400 | -109.609580 | 600360 | 5501023 | 1 | 2.65 | Local |
| F5 | 11/05/2011 0:00 | 18 | 49.653380 | -109.609600 | 600359 | 5501021 | 1 | 3.64 | Local |

| | | | | | | | | | |
|---|---|---|---|---|---|---|---|---|---|
| F5 | 11/05/2011 0:00 | 21 | 49.653360 | -109.609640 | 600356 | 5501019 | 1 | 7.51 | Local |
| F5 | 11/06/2011 0:00 | 0 | 49.653410 | -109.609570 | 600361 | 5501025 | 1 | 11.93 | Local |
| F5 | 11/06/2011 0:00 | 3 | 49.653320 | -109.609660 | 600355 | 5501014 | 1 | 10.61 | Local |
| F5 | 11/06/2011 0:00 | 6 | 49.653400 | -109.609580 | 600360 | 5501023 | 1 | 6.21 | Local |
| F5 | 11/06/2011 0:00 | 9 | 49.653360 | -109.609640 | 600356 | 5501019 | 1 | 1.11 | Local |
| F5 | 11/06/2011 0:00 | 12 | 49.653350 | -109.609640 | 600356 | 5501018 | 1 | 3.41 | Local |
| F5 | 11/06/2011 0:00 | 15 | 49.653380 | -109.609650 | 600355 | 5501021 | 1 | 3.98 | Local |
| F5 | 11/06/2011 0:00 | 18 | 49.653410 | -109.609620 | 600357 | 5501025 | 1 | 14.53 | Local |
| F5 | 11/06/2011 0:00 | 21 | 49.653280 | -109.609600 | 600359 | 5501010 | 1 | 13.36 | Local |
| F5 | 11/07/2011 0:00 | 0 | 49.653400 | -109.609590 | 600360 | 5501023 | 1 | 3.64 | Local |
| F5 | 11/07/2011 0:00 | 3 | 49.653380 | -109.609550 | 600363 | 5501021 | 1 | 6.87 | Local |
| F5 | 11/07/2011 0:00 | 6 | 49.653400 | -109.609640 | 600356 | 5501023 | 1 | 618.18 | Local |
| F5 | 11/07/2011 0:00 | 9 | 49.655260 | -109.601570 | 600935 | 5501241 | 1 | 614.59 | Local |
| F5 | 11/07/2011 0:00 | 12 | 49.658330 | -109.594490 | 601439 | 5501592 | 1 | 7.30 | Local |
| F5 | 11/07/2011 0:00 | 15 | 49.658320 | -109.594390 | 601446 | 5501591 | 1 | 1487.96 | Local |
| F5 | 11/07/2011 0:00 | 18 | 49.671320 | -109.589500 | 601772 | 5503043 | 1 | 132.81 | Local |
| F5 | 11/07/2011 0:00 | 21 | 49.670610 | -109.588020 | 601881 | 5502966 | 1 | 6.67 | Local |
| F5 | 11/08/2011 0:00 | 0 | 49.670640 | -109.587940 | 601886 | 5502969 | 1 | 1667.89 | Local |
| F5 | 11/08/2011 0:00 | 3 | 49.684210 | -109.578090 | 602568 | 5504491 | 1 | 882.07 | Local |
| F5 | 11/08/2011 0:00 | 6 | 49.680050 | -109.567680 | 603328 | 5504043 | 1 | 71.83 | Local |
| F5 | 11/08/2011 0:00 | 9 | 49.680680 | -109.567460 | 603343 | 5504113 | 1 | 6.73 | Local |
| F5 | 11/08/2011 0:00 | 12 | 49.680720 | -109.567390 | 603348 | 5504118 | 1 | 1551.78 | Local |
| F5 | 11/08/2011 0:00 | 15 | 49.689100 | -109.550190 | 604570 | 5505073 | 1 | 1365.32 | Local |
| F5 | 11/08/2011 0:00 | 18 | 49.700910 | -109.545010 | 604919 | 5506393 | 1 | 694.38 | Local |
| F5 | 11/08/2011 0:00 | 21 | 49.702950 | -109.535910 | 605570 | 5506633 | 1 | 218.71 | Local |
| F5 | 11/09/2011 0:00 | 0 | 49.704770 | -109.537060 | 605483 | 5506834 | 1 | 736.71 | Local |
| F5 | 11/09/2011 0:00 | 3 | 49.707410 | -109.527690 | 606153 | 5507140 | 1 | 16.33 | Local |
| F5 | 11/09/2011 0:00 | 6 | 49.707300 | -109.527540 | 606164 | 5507128 | 1 | 15.58 | Local |
| F5 | 11/09/2011 0:00 | 9 | 49.707160 | -109.527550 | 606164 | 5507113 | 1 | 38.63 | Local |
| F5 | 11/09/2011 0:00 | 12 | 49.706820 | -109.527660 | 606157 | 5507075 | 1 | 52.97 | Local |
| F5 | 11/09/2011 0:00 | 15 | 49.707290 | -109.527540 | 606164 | 5507127 | 1 | 385.20 | Local |
| F5 | 11/09/2011 0:00 | 18 | 49.703940 | -109.526180 | 606270 | 5506757 | 1 | 24.98 | Local |
| F5 | 11/09/2011 0:00 | 21 | 49.704160 | -109.526250 | 606264 | 5506781 | 1 | 7.30 | Local |
| F5 | 11/10/2011 0:00 | 0 | 49.704150 | -109.526150 | 606271 | 5506780 | 1 | 454.01 | Local |
| F5 | 11/10/2011 0:00 | 3 | 49.700370 | -109.523770 | 606451 | 5506363 | 1 | 1493.82 | Local |
| F5 | 11/10/2011 0:00 | 6 | 49.688570 | -109.513870 | 607191 | 5505066 | 1 | 319.15 | Local |
| F5 | 11/10/2011 0:00 | 9 | 49.686030 | -109.511810 | 607345 | 5504786 | 1 | 22.32 | Local |
| F5 | 11/10/2011 0:00 | 12 | 49.686100 | -109.512100 | 607324 | 5504794 | 1 | 1.44 | Local |
| F5 | 11/10/2011 0:00 | 15 | 49.686100 | -109.512080 | 607326 | 5504794 | 1 | 49.82 | Local |
| F5 | 11/10/2011 0:00 | 18 | 49.685890 | -109.512690 | 607282 | 5504769 | 0 | 434.99 | Local |
| F5 | 11/11/2011 0:00 | 0 | 49.682030 | -109.513670 | 607220 | 5504339 | 1 | 178.36 | Local |
| F5 | 11/11/2011 0:00 | 3 | 49.680650 | -109.514930 | 607132 | 5504184 | 1 | 2070.35 | Local |
| F5 | 11/11/2011 0:00 | 6 | 49.662600 | -109.521970 | 606664 | 5502167 | 1 | 1917.82 | Local |
| F5 | 11/11/2011 0:00 | 9 | 49.671990 | -109.499680 | 608251 | 5503243 | 1 | 341.74 | Local |
| F5 | 11/11/2011 0:00 | 12 | 49.673850 | -109.495910 | 608519 | 5503455 | 1 | 20.64 | Local |
| F5 | 11/11/2011 0:00 | 15 | 49.674030 | -109.495980 | 608514 | 5503475 | 1 | 865.37 | Local |
| F5 | 11/11/2011 0:00 | 18 | 49.678780 | -109.486480 | 609188 | 5504017 | 1 | 1624.18 | Local |
| F5 | 11/11/2011 0:00 | 21 | 49.669290 | -109.469370 | 610444 | 5502987 | 1 | 266.65 | Local |
| F5 | 11/12/2011 0:00 | 0 | 49.669830 | -109.465770 | 610703 | 5503052 | 1 | 66.38 | Local |
| F5 | 11/12/2011 0:00 | 3 | 49.670420 | -109.465910 | 610691 | 5503117 | 1 | 817.00 | Local |
| F5 | 11/12/2011 0:00 | 6 | 49.675520 | -109.474060 | 610092 | 5503672 | 1 | 258.50 | Local |
| F5 | 11/12/2011 0:00 | 9 | 49.677070 | -109.476730 | 609896 | 5503841 | 1 | 3.98 | Local |
| F5 | 11/12/2011 0:00 | 12 | 49.677040 | -109.476700 | 609898 | 5503837 | 1 | 677.57 | Local |
| F5 | 11/12/2011 0:00 | 15 | 49.682910 | -109.474180 | 610066 | 5504494 | 1 | 1431.51 | Local |
| F5 | 11/12/2011 0:00 | 18 | 49.693590 | -109.463100 | 610841 | 5505697 | 1 | 2349.63 | Local |

| | | | | | | | | | |
|---|---|---|---|---|---|---|---|---|---|
| F5 | 11/12/2011 0:00 | 21 | 49.713740 | -109.453290 | 611503 | 5507952 | 1 | 1176.86 | Local |
| F5 | 11/13/2011 0:00 | 0 | 49.707670 | -109.466660 | 610553 | 5507257 | 1 | 618.09 | Local |
| F5 | 11/13/2011 0:00 | 3 | 49.705100 | -109.474260 | 610011 | 5506961 | 1 | 16.39 | Local |
| F5 | 11/13/2011 0:00 | 6 | 49.705030 | -109.474060 | 610025 | 5506953 | 1 | 22.73 | Local |
| F5 | 11/13/2011 0:00 | 9 | 49.705110 | -109.474350 | 610004 | 5506962 | 0 | 138.25 | Local |
| F5 | 11/13/2011 0:00 | 15 | 49.703870 | -109.474490 | 609997 | 5506823 | 1 | 19.50 | Local |
| F5 | 11/13/2011 0:00 | 18 | 49.704020 | -109.474630 | 609986 | 5506840 | 1 | 672.39 | Local |
| F5 | 11/13/2011 0:00 | 21 | 49.709630 | -109.471150 | 610225 | 5507469 | 1 | 473.63 | Local |
| F5 | 11/14/2011 0:00 | 0 | 49.708860 | -109.464690 | 610692 | 5507393 | 1 | 7.92 | Local |
| F5 | 11/14/2011 0:00 | 3 | 49.708790 | -109.464670 | 610694 | 5507385 | 1 | 15.13 | Local |
| F5 | 11/14/2011 0:00 | 6 | 49.708860 | -109.464850 | 610681 | 5507392 | 1 | 467.64 | Local |
| F5 | 11/14/2011 0:00 | 9 | 49.709230 | -109.471310 | 610214 | 5507424 | 1 | 548.62 | Local |
| F5 | 11/14/2011 0:00 | 12 | 49.704300 | -109.471610 | 610203 | 5506875 | 1 | 14.47 | Local |
| F5 | 11/14/2011 0:00 | 15 | 49.704430 | -109.471600 | 610204 | 5506890 | 1 | 4.68 | Local |
| F5 | 11/14/2011 0:00 | 18 | 49.704390 | -109.471580 | 610205 | 5506886 | 1 | 703.52 | Local |
| F5 | 11/14/2011 0:00 | 21 | 49.708830 | -109.464630 | 610696 | 5507389 | 1 | 18.16 | Local |
| F5 | 11/15/2011 0:00 | 0 | 49.708850 | -109.464880 | 610678 | 5507391 | 1 | 4.47 | Local |
| F5 | 11/15/2011 0:00 | 3 | 49.708840 | -109.464820 | 610683 | 5507390 | 1 | 4.95 | Local |
| F5 | 11/15/2011 0:00 | 6 | 49.708880 | -109.464790 | 610685 | 5507395 | 1 | 413.98 | Local |
| F5 | 11/15/2011 0:00 | 9 | 49.708220 | -109.470440 | 610279 | 5507313 | 1 | 442.42 | Local |
| F5 | 11/15/2011 0:00 | 12 | 49.705220 | -109.474470 | 609995 | 5506974 | 1 | 2.88 | Local |
| F5 | 11/15/2011 0:00 | 15 | 49.705220 | -109.474430 | 609998 | 5506974 | 1 | 356.40 | Local |
| F5 | 11/15/2011 0:00 | 18 | 49.706800 | -109.470130 | 610305 | 5507156 | 1 | 440.94 | Local |
| F5 | 11/15/2011 0:00 | 21 | 49.708800 | -109.464850 | 610681 | 5507386 | 1 | 630.63 | Local |
| F5 | 11/16/2011 0:00 | 0 | 49.707200 | -109.456460 | 611289 | 5507220 | 1 | 14.03 | Local |
| F5 | 11/16/2011 0:00 | 3 | 49.707080 | -109.456520 | 611285 | 5507207 | 1 | 8.58 | Local |
| F5 | 11/16/2011 0:00 | 6 | 49.707150 | -109.456470 | 611289 | 5507215 | 0 | 2095.88 | Local |
| F5 | 11/16/2011 0:00 | 12 | 49.689110 | -109.464890 | 610723 | 5505197 | 1 | 35.05 | Local |
| F5 | 11/16/2011 0:00 | 15 | 49.689030 | -109.464420 | 610757 | 5505188 | 1 | 15.52 | Local |
| F5 | 11/16/2011 0:00 | 18 | 49.688930 | -109.464270 | 610768 | 5505178 | 1 | 8.82 | Local |
| F5 | 11/16/2011 0:00 | 21 | 49.688990 | -109.464350 | 610762 | 5505184 | 0 | 1454.79 | Local |
| F5 | 11/17/2011 0:00 | 3 | 49.675990 | -109.462080 | 610955 | 5503742 | 1 | 452.93 | Local |
| F5 | 11/17/2011 0:00 | 6 | 49.672180 | -109.464300 | 610804 | 5503315 | 1 | 12.53 | Local |
| F5 | 11/17/2011 0:00 | 9 | 49.672080 | -109.464220 | 610810 | 5503304 | 1 | 1135.89 | Local |
| F5 | 11/17/2011 0:00 | 12 | 49.682090 | -109.467360 | 610560 | 5504413 | 1 | 15.58 | Local |
| F5 | 11/17/2011 0:00 | 15 | 49.681950 | -109.467370 | 610560 | 5504397 | 1 | 1410.88 | Local |
| F5 | 11/17/2011 0:00 | 18 | 49.693820 | -109.474280 | 610035 | 5505706 | 1 | 2407.86 | Local |
| F5 | 11/17/2011 0:00 | 21 | 49.708880 | -109.498270 | 608271 | 5507346 | 1 | 26.39 | Local |
| F5 | 11/18/2011 0:00 | 0 | 49.709110 | -109.498360 | 608264 | 5507371 | 1 | 2817.27 | Local |
| F5 | 11/18/2011 0:00 | 3 | 49.702660 | -109.536140 | 605554 | 5506600 | 0 | 3461.61 | Local |
| F5 | 11/18/2011 0:00 | 15 | 49.682230 | -109.572350 | 602987 | 5504279 | 1 | 1688.26 | Local |
| F5 | 11/18/2011 0:00 | 18 | 49.676090 | -109.593750 | 601456 | 5503567 | 1 | 17.81 | Local |
| F5 | 11/18/2011 0:00 | 21 | 49.675930 | -109.593760 | 601455 | 5503549 | 1 | 8.94 | Local |
| F5 | 11/19/2011 0:00 | 0 | 49.675910 | -109.593640 | 601464 | 5503547 | 1 | 6.05 | Local |
| F5 | 11/19/2011 0:00 | 3 | 49.675940 | -109.593710 | 601459 | 5503551 | 1 | 8.30 | Local |
| F5 | 11/19/2011 0:00 | 6 | 49.676010 | -109.593670 | 601462 | 5503558 | 1 | 537.34 | Local |
| F5 | 11/19/2011 0:00 | 9 | 49.676490 | -109.601080 | 600926 | 5503602 | 0 | 1077.19 | Local |
| F5 | 11/19/2011 0:00 | 15 | 49.667820 | -109.594420 | 601425 | 5502647 | 1 | 1435.20 | Local |
| F5 | 11/19/2011 0:00 | 18 | 49.679480 | -109.602950 | 600785 | 5503932 | 1 | 1420.05 | Local |
| F5 | 11/19/2011 0:00 | 21 | 49.675440 | -109.584280 | 602140 | 5503508 | 0 | 438.85 | Local |
| F5 | 11/20/2011 0:00 | 3 | 49.677010 | -109.578700 | 602539 | 5503690 | 0 | 128.25 | Local |
| F5 | 11/20/2011 0:00 | 9 | 49.675870 | -109.578970 | 602522 | 5503563 | 1 | 571.56 | Local |
| F5 | 11/20/2011 0:00 | 12 | 49.677970 | -109.571740 | 603040 | 5503806 | 1 | 7.29 | Local |
| F5 | 11/20/2011 0:00 | 15 | 49.678010 | -109.571660 | 603045 | 5503811 | 1 | 1090.16 | Local |
| F5 | 11/20/2011 0:00 | 18 | 49.680810 | -109.557180 | 604084 | 5504142 | 1 | 2572.17 | Local |

| | | | | | | | | | |
|---|---|---|---|---|---|---|---|---|---|
| F5 | 11/20/2011 0:00 | 21 | 49.667730 | -109.527780 | 606233 | 5502729 | 1 | 1745.05 | Local |
| F5 | 11/21/2011 0:00 | 0 | 49.662910 | -109.504770 | 607904 | 5502226 | 1 | 957.63 | Local |
| F5 | 11/21/2011 0:00 | 3 | 49.668250 | -109.494360 | 608643 | 5502835 | 1 | 931.23 | Local |
| F5 | 11/21/2011 0:00 | 6 | 49.665600 | -109.482120 | 609533 | 5502558 | 1 | 71.60 | Local |
| F5 | 11/21/2011 0:00 | 9 | 49.665000 | -109.481760 | 609560 | 5502492 | 1 | 1436.77 | Local |
| F5 | 11/21/2011 0:00 | 12 | 49.655410 | -109.468420 | 610544 | 5501445 | 1 | 138.31 | Local |
| F5 | 11/21/2011 0:00 | 15 | 49.655250 | -109.466520 | 610682 | 5501430 | 1 | 962.92 | Local |
| F5 | 11/21/2011 0:00 | 18 | 49.648380 | -109.458400 | 611283 | 5500678 | 1 | 569.76 | Local |
| F5 | 11/21/2011 0:00 | 21 | 49.653500 | -109.458710 | 611249 | 5501247 | 1 | 1568.28 | Local |
| F5 | 11/22/2011 0:00 | 0 | 49.664420 | -109.472460 | 610232 | 5502441 | 1 | 3171.95 | Local |
| F5 | 11/22/2011 0:00 | 3 | 49.691890 | -109.460610 | 611025 | 5505512 | 1 | 22.54 | Local |
| F5 | 11/22/2011 0:00 | 6 | 49.691980 | -109.460890 | 611004 | 5505522 | 1 | 14.60 | Local |
| F5 | 11/22/2011 0:00 | 9 | 49.691920 | -109.460710 | 611018 | 5505515 | 1 | 9.89 | Local |
| F5 | 11/22/2011 0:00 | 12 | 49.692000 | -109.460770 | 611013 | 5505524 | 1 | 7.30 | Local |
| F5 | 11/22/2011 0:00 | 15 | 49.692030 | -109.460860 | 611007 | 5505527 | 1 | 4.87 | Local |
| F5 | 11/22/2011 0:00 | 18 | 49.692050 | -109.460800 | 611011 | 5505530 | 0 | 28.13 | Local |
| F5 | 11/23/2011 0:00 | 0 | 49.691800 | -109.460740 | 611016 | 5505502 | 1 | 9.28 | Local |
| F5 | 11/23/2011 0:00 | 3 | 49.691870 | -109.460810 | 611010 | 5505510 | 1 | 8.58 | Local |
| F5 | 11/23/2011 0:00 | 6 | 49.691940 | -109.460760 | 611014 | 5505517 | 1 | 15.19 | Local |
| F5 | 11/23/2011 0:00 | 9 | 49.691950 | -109.460550 | 611029 | 5505519 | 1 | 16.02 | Local |
| F5 | 11/23/2011 0:00 | 12 | 49.691930 | -109.460770 | 611013 | 5505516 | 1 | 1.33 | Local |
| F5 | 11/23/2011 0:00 | 15 | 49.691920 | -109.460760 | 611014 | 5505515 | 1 | 7.01 | Local |
| F5 | 11/23/2011 0:00 | 18 | 49.691980 | -109.460790 | 611012 | 5505522 | 0 | 0.00 | Local |
| F5 | 11/24/2011 0:00 | 0 | 49.691980 | -109.460790 | 611012 | 5505522 | 0 | 9.16 | Local |
| F5 | 11/24/2011 0:00 | 6 | 49.691900 | -109.460820 | 611010 | 5505513 | 1 | 14.41 | Local |
| F5 | 11/24/2011 0:00 | 9 | 49.691860 | -109.460630 | 611023 | 5505509 | 1 | 260.84 | Local |
| F5 | 11/24/2011 0:00 | 12 | 49.691530 | -109.464210 | 610766 | 5505467 | 1 | 261.36 | Local |
| F5 | 11/24/2011 0:00 | 15 | 49.692000 | -109.460660 | 611021 | 5505524 | 1 | 19.79 | Local |
| F5 | 11/24/2011 0:00 | 18 | 49.691840 | -109.460540 | 611030 | 5505507 | 1 | 7.30 | Local |
| F5 | 11/24/2011 0:00 | 21 | 49.691850 | -109.460640 | 611023 | 5505508 | 1 | 11.12 | Local |
| F5 | 11/25/2011 0:00 | 0 | 49.691950 | -109.460640 | 611023 | 5505519 | 1 | 10.16 | Local |
| F5 | 11/25/2011 0:00 | 3 | 49.691940 | -109.460780 | 611012 | 5505517 | 1 | 11.70 | Local |
| F5 | 11/25/2011 0:00 | 6 | 49.691900 | -109.460630 | 611023 | 5505513 | 1 | 4.41 | Local |
| F5 | 11/25/2011 0:00 | 9 | 49.691870 | -109.460670 | 611021 | 5505510 | 1 | 6.05 | Local |
| F5 | 11/25/2011 0:00 | 12 | 49.691900 | -109.460600 | 611026 | 5505513 | 1 | 5.61 | Local |
| F5 | 11/25/2011 0:00 | 15 | 49.691950 | -109.460590 | 611026 | 5505519 | 1 | 6.26 | Local |
| F5 | 11/25/2011 0:00 | 18 | 49.691900 | -109.460630 | 611023 | 5505513 | 1 | 4.68 | Local |
| F5 | 11/25/2011 0:00 | 21 | 49.691860 | -109.460650 | 611022 | 5505509 | 1 | 10.24 | Local |
| F5 | 11/26/2011 0:00 | 0 | 49.691950 | -109.460680 | 611020 | 5505519 | 1 | 12.53 | Local |
| F5 | 11/26/2011 0:00 | 3 | 49.691850 | -109.460600 | 611026 | 5505508 | 1 | 2.16 | Local |
| F5 | 11/26/2011 0:00 | 6 | 49.691850 | -109.460630 | 611024 | 5505508 | 1 | 13.52 | Local |
| F5 | 11/26/2011 0:00 | 9 | 49.691960 | -109.460710 | 611017 | 5505520 | 1 | 15.15 | Local |
| F5 | 11/26/2011 0:00 | 12 | 49.691880 | -109.460540 | 611030 | 5505511 | 1 | 24.07 | Local |
| F5 | 11/26/2011 0:00 | 15 | 49.692070 | -109.460700 | 611018 | 5505532 | 1 | 17.47 | Local |
| F5 | 11/26/2011 0:00 | 18 | 49.691930 | -109.460590 | 611026 | 5505517 | 1 | 2.34 | Local |
| F5 | 11/26/2011 0:00 | 21 | 49.691950 | -109.460600 | 611025 | 5505519 | 1 | 4.41 | Local |
| F5 | 11/27/2011 0:00 | 0 | 49.691920 | -109.460640 | 611023 | 5505515 | 1 | 5.52 | Local |
| F5 | 11/27/2011 0:00 | 3 | 49.691900 | -109.460570 | 611028 | 5505513 | 1 | 7.55 | Local |
| F5 | 11/27/2011 0:00 | 6 | 49.691920 | -109.460670 | 611020 | 5505515 | 1 | 9.64 | Local |
| F5 | 11/27/2011 0:00 | 9 | 49.691940 | -109.460800 | 611011 | 5505517 | 1 | 18.56 | Local |
| F5 | 11/27/2011 0:00 | 12 | 49.691800 | -109.460660 | 611021 | 5505502 | 1 | 16.16 | Local |
| F5 | 11/27/2011 0:00 | 15 | 49.691930 | -109.460760 | 611014 | 5505516 | 1 | 11.33 | Local |
| F5 | 11/27/2011 0:00 | 18 | 49.691830 | -109.460730 | 611016 | 5505505 | 1 | 18.49 | Local |
| F5 | 11/27/2011 0:00 | 21 | 49.691990 | -109.460800 | 611011 | 5505523 | 1 | 7.92 | Local |
| F5 | 11/28/2011 0:00 | 0 | 49.691920 | -109.460820 | 611010 | 5505515 | 1 | 7.30 | Local |

| | | | | | | | | | |
|---|---|---|---|---|---|---|---|---|---|
| F5 | 11/28/2011 0:00 | 3 | 49.691890 | -109.460730 | 611016 | 5505512 | 1 | 2.89 | Local |
| F5 | 11/28/2011 0:00 | 6 | 49.691890 | -109.460770 | 611013 | 5505512 | 1 | 3.64 | Local |
| F5 | 11/28/2011 0:00 | 9 | 49.691870 | -109.460810 | 611010 | 5505510 | 1 | 8.01 | Local |
| F5 | 11/28/2011 0:00 | 12 | 49.691820 | -109.460730 | 611016 | 5505504 | 1 | 54.21 | Local |
| F5 | 11/28/2011 0:00 | 15 | 49.692130 | -109.461310 | 610974 | 5505538 | 1 | 22.10 | Local |
| F5 | 11/28/2011 0:00 | 18 | 49.691950 | -109.461180 | 610984 | 5505518 | 1 | 27.59 | Local |
| F5 | 11/28/2011 0:00 | 21 | 49.691850 | -109.460830 | 611009 | 5505507 | 1 | 1.33 | Local |
| F5 | 11/29/2011 0:00 | 0 | 49.691860 | -109.460820 | 611010 | 5505508 | 1 | 5.46 | Local |
| F5 | 11/29/2011 0:00 | 3 | 49.691890 | -109.460880 | 611005 | 5505512 | 1 | 36.37 | Local |
| F5 | 11/29/2011 0:00 | 6 | 49.691790 | -109.460400 | 611040 | 5505501 | 1 | 332.09 | Local |
| F5 | 11/29/2011 0:00 | 9 | 49.689100 | -109.462400 | 610902 | 5505199 | 0 | 135.56 | Local |
| F5 | 11/29/2011 0:00 | 15 | 49.688750 | -109.464200 | 610773 | 5505158 | 1 | 237.55 | Local |
| F5 | 11/29/2011 0:00 | 18 | 49.688220 | -109.461010 | 611004 | 5505103 | 1 | 110.55 | Local |
| F5 | 11/29/2011 0:00 | 21 | 49.687610 | -109.459800 | 611093 | 5505037 | 1 | 120.73 | Local |
| F5 | 11/30/2011 0:00 | 0 | 49.688360 | -109.461010 | 611004 | 5505119 | 1 | 18.02 | Local |
| F5 | 11/30/2011 0:00 | 3 | 49.688200 | -109.461050 | 611002 | 5505101 | 1 | 680.05 | Local |
| F5 | 11/30/2011 0:00 | 6 | 49.683510 | -109.467100 | 610576 | 5504571 | 0 | 953.63 | Local |
| F5 | 11/30/2011 0:00 | 12 | 49.685520 | -109.479950 | 609644 | 5504775 | 1 | 1597.89 | Local |
| F5 | 11/30/2011 0:00 | 15 | 49.696310 | -109.494580 | 608565 | 5505954 | 1 | 644.11 | Local |
| F5 | 11/30/2011 0:00 | 18 | 49.690530 | -109.495170 | 608535 | 5505310 | 1 | 12.34 | Local |
| F5 | 11/30/2011 0:00 | 21 | 49.690440 | -109.495070 | 608543 | 5505300 | 0 | 1157.08 | Local |
| F5 | 12/01/2011 0:00 | 9 | 49.681280 | -109.502680 | 608014 | 5504271 | 1 | 481.63 | Local |
| F5 | 12/01/2011 0:00 | 12 | 49.682010 | -109.496100 | 608487 | 5504362 | 1 | 10.60 | Local |
| F5 | 12/01/2011 0:00 | 15 | 49.681930 | -109.496020 | 608493 | 5504353 | 1 | 537.61 | Local |
| F5 | 12/01/2011 0:00 | 18 | 49.677180 | -109.497410 | 608404 | 5503823 | 1 | 1805.97 | Local |
| F5 | 12/01/2011 0:00 | 21 | 49.660980 | -109.495620 | 608569 | 5502025 | 1 | 1365.29 | Local |
| F5 | 12/02/2011 0:00 | 0 | 49.650820 | -109.506240 | 607825 | 5500880 | 0 | 2432.18 | Local |
| F5 | 12/02/2011 0:00 | 15 | 49.633950 | -109.484800 | 609410 | 5499035 | 1 | 824.07 | Local |
| F5 | 12/02/2011 0:00 | 18 | 49.626560 | -109.483940 | 609489 | 5498215 | 1 | 3181.77 | Local |
| F5 | 12/02/2011 0:00 | 21 | 49.654570 | -109.492950 | 608776 | 5501316 | 0 | 3036.00 | Local |
| F5 | 12/03/2011 0:00 | 9 | 49.679950 | -109.508460 | 607600 | 5504115 | 1 | 434.34 | Local |
| F5 | 12/03/2011 0:00 | 12 | 49.681350 | -109.502840 | 608003 | 5504279 | 1 | 4.87 | Local |
| F5 | 12/03/2011 0:00 | 15 | 49.681370 | -109.502780 | 608007 | 5504281 | 1 | 6.05 | Local |
| F5 | 12/03/2011 0:00 | 18 | 49.681400 | -109.502710 | 608012 | 5504284 | 1 | 15.14 | Local |
| F5 | 12/03/2011 0:00 | 21 | 49.681330 | -109.502890 | 607999 | 5504276 | 1 | 10.11 | Local |
| F5 | 12/04/2011 0:00 | 0 | 49.681420 | -109.502910 | 607997 | 5504286 | 1 | 3156.14 | Local |
| F5 | 12/04/2011 0:00 | 3 | 49.657860 | -109.478520 | 609810 | 5501703 | 1 | 247.23 | Local |
| F5 | 12/04/2011 0:00 | 6 | 49.657340 | -109.475190 | 610051 | 5501650 | 1 | 15.52 | Local |
| F5 | 12/04/2011 0:00 | 9 | 49.657370 | -109.474980 | 610066 | 5501653 | 1 | 12.42 | Local |
| F5 | 12/04/2011 0:00 | 12 | 49.657260 | -109.475010 | 610064 | 5501641 | 1 | 6.63 | Local |
| F5 | 12/04/2011 0:00 | 15 | 49.657310 | -109.474960 | 610068 | 5501647 | 1 | 2.34 | Local |
| F5 | 12/04/2011 0:00 | 18 | 49.657330 | -109.474970 | 610067 | 5501649 | 1 | 1164.26 | Local |
| F5 | 12/04/2011 0:00 | 21 | 49.663020 | -109.488510 | 609077 | 5502262 | 1 | 44.53 | Local |
| F5 | 12/05/2011 0:00 | 0 | 49.663080 | -109.489120 | 609033 | 5502267 | 0 | 1218.73 | Local |
| F5 | 12/05/2011 0:00 | 9 | 49.671820 | -109.499310 | 608278 | 5503224 | 1 | 12.75 | Local |
| F5 | 12/05/2011 0:00 | 12 | 49.671710 | -109.499360 | 608275 | 5503212 | 1 | 4.41 | Local |
| F5 | 12/05/2011 0:00 | 15 | 49.671740 | -109.499320 | 608278 | 5503215 | 1 | 45.73 | Local |
| F5 | 12/05/2011 0:00 | 18 | 49.671890 | -109.498730 | 608320 | 5503233 | 0 | 2645.85 | Local |
| F5 | 12/06/2011 0:00 | 3 | 49.678090 | -109.463330 | 610860 | 5503974 | 1 | 296.78 | Local |
| F5 | 12/06/2011 0:00 | 6 | 49.680660 | -109.464440 | 610774 | 5504258 | 0 | 6.21 | Local |
| F5 | 12/06/2011 0:00 | 15 | 49.680620 | -109.464500 | 610770 | 5504253 | 1 | 14.60 | Local |
| F5 | 12/06/2011 0:00 | 18 | 49.680640 | -109.464700 | 610755 | 5504255 | 1 | 15.31 | Local |
| F5 | 12/06/2011 0:00 | 21 | 49.680660 | -109.464490 | 610771 | 5504258 | 1 | 5.47 | Local |
| F5 | 12/07/2011 0:00 | 0 | 49.680630 | -109.464430 | 610775 | 5504255 | 1 | 2.34 | Local |
| F5 | 12/07/2011 0:00 | 3 | 49.680650 | -109.464440 | 610774 | 5504257 | 1 | 4.47 | Local |

| | | | | | | | | | |
|---|---|---|---|---|---|---|---|---|---|
| F5 | 12/07/2011 0:00 | 6 | 49.680660 | -109.464500 | 610770 | 5504258 | 1 | 152.09 | Local |
| F5 | 12/07/2011 0:00 | 9 | 49.679400 | -109.465320 | 610714 | 5504117 | 1 | 153.67 | Local |
| F5 | 12/07/2011 0:00 | 12 | 49.680670 | -109.464480 | 610771 | 5504259 | 1 | 4.24 | Local |
| F5 | 12/07/2011 0:00 | 15 | 49.680650 | -109.464430 | 610775 | 5504257 | 1 | 1.82 | Local |
| F5 | 12/07/2011 0:00 | 18 | 49.680660 | -109.464450 | 610773 | 5504258 | 1 | 0.72 | Local |
| F5 | 12/07/2011 0:00 | 21 | 49.680660 | -109.464460 | 610773 | 5504258 | 1 | 10.14 | Local |
| F5 | 12/08/2011 0:00 | 0 | 49.680590 | -109.464370 | 610779 | 5504250 | 1 | 17.03 | Local |
| F5 | 12/08/2011 0:00 | 3 | 49.680660 | -109.464580 | 610764 | 5504258 | 1 | 10.38 | Local |
| F5 | 12/08/2011 0:00 | 6 | 49.680620 | -109.464450 | 610774 | 5504253 | 1 | 4.41 | Local |
| F5 | 12/08/2011 0:00 | 9 | 49.680650 | -109.464490 | 610771 | 5504257 | 1 | 6.59 | Local |
| F5 | 12/08/2011 0:00 | 12 | 49.680640 | -109.464400 | 610777 | 5504256 | 1 | 3.64 | Local |
| F5 | 12/08/2011 0:00 | 15 | 49.680660 | -109.464440 | 610774 | 5504258 | 1 | 1.82 | Local |
| F5 | 12/08/2011 0:00 | 18 | 49.680650 | -109.464460 | 610773 | 5504257 | 0 | 154.42 | Local |
| F5 | 12/09/2011 0:00 | 0 | 49.679370 | -109.465290 | 610716 | 5504113 | 1 | 135.77 | Local |
| F5 | 12/09/2011 0:00 | 3 | 49.680200 | -109.463910 | 610813 | 5504208 | 1 | 60.36 | Local |
| F5 | 12/09/2011 0:00 | 6 | 49.680620 | -109.464440 | 610774 | 5504253 | 1 | 3.41 | Local |
| F5 | 12/09/2011 0:00 | 9 | 49.680650 | -109.464450 | 610773 | 5504257 | 1 | 3.09 | Local |
| F5 | 12/09/2011 0:00 | 12 | 49.680640 | -109.464490 | 610771 | 5504256 | 1 | 8.94 | Local |
| F5 | 12/09/2011 0:00 | 15 | 49.680660 | -109.464370 | 610779 | 5504258 | 1 | 13.33 | Local |
| F5 | 12/09/2011 0:00 | 18 | 49.680590 | -109.464520 | 610769 | 5504250 | 1 | 2.65 | Local |
| F5 | 12/09/2011 0:00 | 21 | 49.680610 | -109.464500 | 610770 | 5504252 | 1 | 13.65 | Local |
| F5 | 12/10/2011 0:00 | 0 | 49.680730 | -109.464540 | 610767 | 5504266 | 1 | 9.28 | Local |
| F5 | 12/10/2011 0:00 | 3 | 49.680660 | -109.464470 | 610772 | 5504258 | 1 | 3.63 | Local |
| F5 | 12/10/2011 0:00 | 6 | 49.680690 | -109.464450 | 610773 | 5504261 | 1 | 27.70 | Local |
| F5 | 12/10/2011 0:00 | 9 | 49.680470 | -109.464630 | 610761 | 5504237 | 1 | 15.57 | Local |
| F5 | 12/10/2011 0:00 | 12 | 49.680600 | -109.464550 | 610766 | 5504251 | 1 | 7.27 | Local |
| F5 | 12/10/2011 0:00 | 15 | 49.680660 | -109.464510 | 610769 | 5504258 | 1 | 60.26 | Local |
| F5 | 12/10/2011 0:00 | 18 | 49.681200 | -109.464440 | 610773 | 5504318 | 1 | 58.02 | Local |
| F5 | 12/10/2011 0:00 | 21 | 49.680690 | -109.464610 | 610762 | 5504261 | 1 | 12.88 | Local |
| F5 | 12/11/2011 0:00 | 0 | 49.680590 | -109.464520 | 610769 | 5504250 | 1 | 9.16 | Local |
| F5 | 12/11/2011 0:00 | 3 | 49.680670 | -109.464490 | 610771 | 5504259 | 1 | 3.61 | Local |
| F5 | 12/11/2011 0:00 | 6 | 49.680670 | -109.464440 | 610774 | 5504259 | 1 | 5.97 | Local |
| F5 | 12/11/2011 0:00 | 9 | 49.680620 | -109.464470 | 610772 | 5504253 | 1 | 3.34 | Local |
| F5 | 12/11/2011 0:00 | 12 | 49.680650 | -109.464470 | 610772 | 5504257 | 1 | 2.16 | Local |
| F5 | 12/11/2011 0:00 | 15 | 49.680650 | -109.464440 | 610774 | 5504257 | 1 | 6.67 | Local |
| F5 | 12/11/2011 0:00 | 18 | 49.680680 | -109.464520 | 610768 | 5504260 | 1 | 8.08 | Local |
| F5 | 12/11/2011 0:00 | 21 | 49.680610 | -109.464490 | 610771 | 5504252 | 1 | 9.31 | Local |
| F5 | 12/12/2011 0:00 | 0 | 49.680550 | -109.464400 | 610777 | 5504246 | 1 | 7.95 | Local |
| F5 | 12/12/2011 0:00 | 3 | 49.680610 | -109.464460 | 610773 | 5504252 | 1 | 2.22 | Local |
| F5 | 12/12/2011 0:00 | 6 | 49.680630 | -109.464460 | 610773 | 5504255 | 1 | 1.82 | Local |
| F5 | 12/12/2011 0:00 | 9 | 49.680640 | -109.464480 | 610771 | 5504256 | 1 | 1.33 | Local |
| F5 | 12/12/2011 0:00 | 12 | 49.680650 | -109.464470 | 610772 | 5504257 | 1 | 1.82 | Local |
| F5 | 12/12/2011 0:00 | 15 | 49.680640 | -109.464450 | 610773 | 5504256 | 1 | 2.16 | Local |
| F5 | 12/12/2011 0:00 | 18 | 49.680640 | -109.464480 | 610771 | 5504256 | 1 | 1.82 | Local |
| F5 | 12/12/2011 0:00 | 21 | 49.680650 | -109.464500 | 610770 | 5504257 | 1 | 2.16 | Local |
| F5 | 12/13/2011 0:00 | 0 | 49.680650 | -109.464470 | 610772 | 5504257 | 1 | 2.43 | Local |
| F5 | 12/13/2011 0:00 | 3 | 49.680640 | -109.464440 | 610774 | 5504256 | 1 | 1.11 | Local |
| F5 | 12/13/2011 0:00 | 6 | 49.680650 | -109.464440 | 610774 | 5504257 | 1 | 4.87 | Local |
| F5 | 12/13/2011 0:00 | 9 | 49.680630 | -109.464500 | 610770 | 5504254 | 1 | 4.47 | Local |
| F5 | 12/13/2011 0:00 | 12 | 49.680640 | -109.464440 | 610774 | 5504256 | 1 | 1.44 | Local |
| F5 | 12/13/2011 0:00 | 15 | 49.680640 | -109.464460 | 610773 | 5504256 | 1 | 0.72 | Local |
| F5 | 12/13/2011 0:00 | 18 | 49.680640 | -109.464470 | 610772 | 5504256 | 1 | 2.34 | Local |
| F5 | 12/13/2011 0:00 | 21 | 49.680660 | -109.464460 | 610773 | 5504258 | 1 | 7.29 | Local |
| F5 | 12/14/2011 0:00 | 0 | 49.680700 | -109.464380 | 610778 | 5504262 | 1 | 11.70 | Local |
| F5 | 12/14/2011 0:00 | 3 | 49.680660 | -109.464530 | 610768 | 5504258 | 1 | 741.09 | Local |

| | | | | | | | | | |
|---|---|---|---|---|---|---|---|---|---|
| F5 | 12/14/2011 0:00 | 6 | 49.674000 | -109.464920 | 610755 | 5503517 | 1 | 730.81 | Local |
| F5 | 12/14/2011 0:00 | 9 | 49.680240 | -109.468100 | 610511 | 5504206 | 1 | 227.13 | Local |
| F5 | 12/14/2011 0:00 | 12 | 49.682280 | -109.467940 | 610518 | 5504433 | 1 | 9.01 | Local |
| F5 | 12/14/2011 0:00 | 15 | 49.682360 | -109.467960 | 610516 | 5504442 | 1 | 2.43 | Local |
| F5 | 12/14/2011 0:00 | 18 | 49.682370 | -109.467930 | 610519 | 5504443 | 1 | 12.47 | Local |
| F5 | 12/14/2011 0:00 | 21 | 49.682350 | -109.468100 | 610506 | 5504440 | 1 | 6.86 | Local |
| F5 | 12/15/2011 0:00 | 0 | 49.682330 | -109.468010 | 610513 | 5504438 | 0 | 4.24 | Local |
| F5 | 12/15/2011 0:00 | 6 | 49.682310 | -109.467960 | 610517 | 5504436 | 1 | 1.33 | Local |
| F5 | 12/15/2011 0:00 | 9 | 49.682300 | -109.467970 | 610516 | 5504435 | 1 | 1.11 | Local |
| F5 | 12/15/2011 0:00 | 12 | 49.682310 | -109.467970 | 610516 | 5504436 | 1 | 124.40 | Local |
| F5 | 12/15/2011 0:00 | 15 | 49.682800 | -109.466420 | 610626 | 5504493 | 1 | 43.33 | Local |
| F5 | 12/15/2011 0:00 | 18 | 49.682440 | -109.466190 | 610644 | 5504453 | 1 | 132.60 | Local |
| F5 | 12/15/2011 0:00 | 21 | 49.682330 | -109.468020 | 610512 | 5504438 | 1 | 2.34 | Local |
| F5 | 12/16/2011 0:00 | 0 | 49.682350 | -109.468030 | 610511 | 5504441 | 1 | 6.05 | Local |
| F5 | 12/16/2011 0:00 | 3 | 49.682380 | -109.467960 | 610516 | 5504444 | 1 | 10.03 | Local |
| F5 | 12/16/2011 0:00 | 6 | 49.682290 | -109.467950 | 610517 | 5504434 | 1 | 103.56 | Local |
| F5 | 12/16/2011 0:00 | 9 | 49.682210 | -109.466520 | 610621 | 5504427 | 1 | 105.60 | Local |
| F5 | 12/16/2011 0:00 | 12 | 49.682380 | -109.467960 | 610516 | 5504444 | 1 | 11.21 | Local |
| F5 | 12/16/2011 0:00 | 15 | 49.682280 | -109.467980 | 610515 | 5504433 | 1 | 471.82 | Local |
| F5 | 12/16/2011 0:00 | 18 | 49.679350 | -109.463250 | 610863 | 5504114 | 1 | 587.15 | Local |
| F5 | 12/16/2011 0:00 | 21 | 49.684620 | -109.463760 | 610814 | 5504699 | 1 | 956.55 | Local |
| F5 | 12/17/2011 0:00 | 0 | 49.693190 | -109.462610 | 610878 | 5505654 | 1 | 1944.41 | Local |
| F5 | 12/17/2011 0:00 | 3 | 49.686900 | -109.487760 | 609078 | 5504918 | 1 | 87.25 | Local |
| F5 | 12/17/2011 0:00 | 6 | 49.686560 | -109.486670 | 609157 | 5504881 | 0 | 1.11 | Local |
| F5 | 12/17/2011 0:00 | 15 | 49.686550 | -109.486670 | 609157 | 5504880 | 1 | 1581.11 | Local |
| F5 | 12/17/2011 0:00 | 18 | 49.676370 | -109.501970 | 608076 | 5503726 | 1 | 1347.86 | Local |
| F5 | 12/17/2011 0:00 | 21 | 49.664280 | -109.503320 | 608006 | 5502380 | 1 | 691.16 | Local |
| F5 | 12/18/2011 0:00 | 0 | 49.670440 | -109.502040 | 608084 | 5503067 | 1 | 199.17 | Local |
| F5 | 12/18/2011 0:00 | 3 | 49.672020 | -109.500740 | 608175 | 5503244 | 1 | 27.93 | Local |
| F5 | 12/18/2011 0:00 | 6 | 49.671840 | -109.500470 | 608195 | 5503225 | 1 | 305.66 | Local |
| F5 | 12/18/2011 0:00 | 9 | 49.674580 | -109.500130 | 608213 | 5503530 | 1 | 3.09 | Local |
| F5 | 12/18/2011 0:00 | 12 | 49.674570 | -109.500170 | 608210 | 5503529 | 1 | 256.24 | Local |
| F5 | 12/18/2011 0:00 | 15 | 49.676870 | -109.499950 | 608221 | 5503785 | 1 | 190.83 | Local |
| F5 | 12/18/2011 0:00 | 18 | 49.677500 | -109.502410 | 608042 | 5503851 | 1 | 363.33 | Local |
| F5 | 12/18/2011 0:00 | 21 | 49.674590 | -109.500120 | 608214 | 5503531 | 1 | 2.65 | Local |
| F5 | 12/19/2011 0:00 | 0 | 49.674610 | -109.500100 | 608215 | 5503533 | 1 | 4.51 | Local |
| F5 | 12/19/2011 0:00 | 3 | 49.674570 | -109.500110 | 608215 | 5503529 | 1 | 9.01 | Local |
| F5 | 12/19/2011 0:00 | 6 | 49.674490 | -109.500090 | 608216 | 5503520 | 1 | 146.16 | Local |
| F5 | 12/19/2011 0:00 | 9 | 49.675790 | -109.500390 | 608192 | 5503664 | 1 | 1084.61 | Local |
| F5 | 12/19/2011 0:00 | 12 | 49.682410 | -109.511430 | 607381 | 5504384 | 1 | 720.29 | Local |
| F5 | 12/19/2011 0:00 | 15 | 49.680960 | -109.521160 | 606682 | 5504209 | 1 | 2102.51 | Local |
| F5 | 12/19/2011 0:00 | 18 | 49.695770 | -109.539280 | 605343 | 5505830 | 1 | 1646.83 | Local |
| F5 | 12/19/2011 0:00 | 21 | 49.701600 | -109.560270 | 603817 | 5506449 | 1 | 637.28 | Local |
| F5 | 12/20/2011 0:00 | 0 | 49.696310 | -109.556870 | 604073 | 5505866 | 1 | 1143.25 | Local |
| F5 | 12/20/2011 0:00 | 3 | 49.686030 | -109.556590 | 604115 | 5504723 | 1 | 722.67 | Local |
| F5 | 12/20/2011 0:00 | 6 | 49.682440 | -109.564940 | 603521 | 5504312 | 1 | 23.28 | Local |
| F5 | 12/20/2011 0:00 | 9 | 49.682580 | -109.564700 | 603538 | 5504328 | 1 | 14.54 | Local |
| F5 | 12/20/2011 0:00 | 12 | 49.682700 | -109.564780 | 603532 | 5504342 | 1 | 352.63 | Local |
| F5 | 12/20/2011 0:00 | 15 | 49.681980 | -109.569540 | 603190 | 5504255 | 1 | 2017.79 | Local |
| F5 | 12/20/2011 0:00 | 18 | 49.673720 | -109.594440 | 601411 | 5503303 | 1 | 445.53 | Local |
| F5 | 12/20/2011 0:00 | 21 | 49.675860 | -109.599660 | 601030 | 5503534 | 1 | 849.24 | Local |
| F5 | 12/21/2011 0:00 | 0 | 49.668570 | -109.603170 | 600792 | 5502719 | 1 | 226.86 | Local |
| F5 | 12/21/2011 0:00 | 3 | 49.670400 | -109.604560 | 600688 | 5502920 | 1 | 554.55 | Local |
| F5 | 12/21/2011 0:00 | 6 | 49.668140 | -109.597710 | 601186 | 5502678 | 1 | 771.71 | Local |
| F5 | 12/21/2011 0:00 | 9 | 49.674750 | -109.600970 | 600938 | 5503409 | 1 | 32.21 | Local |

| | | | | | | | | | |
|---|---|---|---|---|---|---|---|---|---|
| F5 | 12/21/2011 0:00 | 12 | 49.674990 | -109.600720 | 600955 | 5503436 | 1 | 11.12 | Local |
| F5 | 12/21/2011 0:00 | 15 | 49.675060 | -109.600610 | 600963 | 5503443 | 1 | 10.11 | Local |
| F5 | 12/21/2011 0:00 | 18 | 49.674970 | -109.600630 | 600962 | 5503433 | 1 | 6.59 | Local |
| F5 | 12/21/2011 0:00 | 21 | 49.674960 | -109.600720 | 600955 | 5503432 | 1 | 2.22 | Local |
| F5 | 12/22/2011 0:00 | 0 | 49.674980 | -109.600720 | 600955 | 5503434 | 1 | 4.33 | Local |
| F5 | 12/22/2011 0:00 | 3 | 49.674980 | -109.600660 | 600959 | 5503435 | 1 | 6.05 | Local |
| F5 | 12/22/2011 0:00 | 6 | 49.674950 | -109.600730 | 600954 | 5503431 | 1 | 3.98 | Local |
| F5 | 12/22/2011 0:00 | 9 | 49.674920 | -109.600700 | 600957 | 5503428 | 1 | 8.92 | Local |
| F5 | 12/22/2011 0:00 | 12 | 49.675000 | -109.600710 | 600956 | 5503437 | 1 | 3.34 | Local |
| F5 | 12/22/2011 0:00 | 15 | 49.674970 | -109.600710 | 600956 | 5503433 | 1 | 6.05 | Local |
| F5 | 12/22/2011 0:00 | 18 | 49.674940 | -109.600640 | 600961 | 5503430 | 1 | 8.91 | Local |
| F5 | 12/22/2011 0:00 | 21 | 49.675010 | -109.600700 | 600957 | 5503438 | 1 | 3.34 | Local |
| F5 | 12/23/2011 0:00 | 0 | 49.674980 | -109.600700 | 600957 | 5503434 | 1 | 6.63 | Local |
| F5 | 12/23/2011 0:00 | 3 | 49.675030 | -109.600650 | 600960 | 5503440 | 1 | 10.03 | Local |
| F5 | 12/23/2011 0:00 | 6 | 49.674940 | -109.600660 | 600960 | 5503430 | 1 | 7.27 | Local |
| F5 | 12/23/2011 0:00 | 9 | 49.675000 | -109.600700 | 600957 | 5503437 | 1 | 2.16 | Local |
| F5 | 12/23/2011 0:00 | 12 | 49.675000 | -109.600670 | 600959 | 5503437 | 1 | 10.93 | Local |
| F5 | 12/23/2011 0:00 | 15 | 49.674940 | -109.600790 | 600950 | 5503430 | 1 | 9.11 | Local |
| F5 | 12/23/2011 0:00 | 18 | 49.674890 | -109.600690 | 600957 | 5503424 | 1 | 9.35 | Local |
| F5 | 12/23/2011 0:00 | 21 | 49.674970 | -109.600650 | 600960 | 5503433 | 0 | 1.33 | Local |
| F5 | 12/24/2011 0:00 | 6 | 49.674960 | -109.600660 | 600959 | 5503432 | 1 | 11.21 | Local |
| F5 | 12/24/2011 0:00 | 9 | 49.675060 | -109.600680 | 600958 | 5503443 | 1 | 11.33 | Local |
| F5 | 12/24/2011 0:00 | 12 | 49.674960 | -109.600650 | 600960 | 5503432 | 1 | 2.34 | Local |
| F5 | 12/24/2011 0:00 | 15 | 49.674940 | -109.600660 | 600960 | 5503430 | 1 | 14.60 | Local |
| F5 | 12/24/2011 0:00 | 18 | 49.674960 | -109.600860 | 600945 | 5503432 | 1 | 14.57 | Local |
| F5 | 12/24/2011 0:00 | 21 | 49.674880 | -109.600700 | 600957 | 5503423 | 1 | 3.10 | Local |
| F5 | 12/25/2011 0:00 | 0 | 49.674860 | -109.600670 | 600959 | 5503421 | 1 | 3.98 | Local |
| F5 | 12/25/2011 0:00 | 3 | 49.674890 | -109.600640 | 600961 | 5503425 | 1 | 5.17 | Local |
| F5 | 12/25/2011 0:00 | 6 | 49.674880 | -109.600710 | 600956 | 5503423 | 1 | 12.23 | Local |
| F5 | 12/25/2011 0:00 | 9 | 49.674990 | -109.600710 | 600956 | 5503436 | 1 | 8.91 | Local |
| F5 | 12/25/2011 0:00 | 12 | 49.675060 | -109.600650 | 600960 | 5503443 | 1 | 15.91 | Local |
| F5 | 12/25/2011 0:00 | 15 | 49.675070 | -109.600870 | 600944 | 5503444 | 1 | 67.92 | Local |
| F5 | 12/25/2011 0:00 | 18 | 49.675370 | -109.600050 | 601003 | 5503479 | 1 | 32.00 | Local |
| F5 | 12/25/2011 0:00 | 21 | 49.675110 | -109.600240 | 600989 | 5503450 | 1 | 38.92 | Local |
| F5 | 12/26/2011 0:00 | 0 | 49.675460 | -109.600230 | 600989 | 5503488 | 1 | 177.04 | Local |
| F5 | 12/26/2011 0:00 | 3 | 49.675240 | -109.597800 | 601165 | 5503467 | 1 | 89.13 | Local |
| F5 | 12/26/2011 0:00 | 6 | 49.675760 | -109.596860 | 601232 | 5503526 | 1 | 8.91 | Local |
| F5 | 12/26/2011 0:00 | 9 | 49.675830 | -109.596800 | 601236 | 5503534 | 1 | 167.06 | Local |
| F5 | 12/26/2011 0:00 | 12 | 49.676640 | -109.594850 | 601375 | 5503627 | 1 | 468.66 | Local |
| F5 | 12/26/2011 0:00 | 15 | 49.672530 | -109.593410 | 601488 | 5503172 | 1 | 518.10 | Local |
| F5 | 12/26/2011 0:00 | 18 | 49.674590 | -109.586970 | 601948 | 5503410 | 1 | 51.01 | Local |
| F5 | 12/26/2011 0:00 | 21 | 49.674870 | -109.587530 | 601907 | 5503440 | 1 | 66.60 | Local |
| F5 | 12/27/2011 0:00 | 0 | 49.674670 | -109.586660 | 601970 | 5503419 | 1 | 734.98 | Local |
| F5 | 12/27/2011 0:00 | 3 | 49.675360 | -109.576530 | 602700 | 5503509 | 1 | 37.93 | Local |
| F5 | 12/27/2011 0:00 | 6 | 49.675310 | -109.576010 | 602737 | 5503505 | 1 | 21.80 | Local |
| F5 | 12/27/2011 0:00 | 9 | 49.675200 | -109.576260 | 602719 | 5503492 | 1 | 222.57 | Local |
| F5 | 12/27/2011 0:00 | 12 | 49.676660 | -109.574150 | 602868 | 5503657 | 1 | 9.35 | Local |
| F5 | 12/27/2011 0:00 | 15 | 49.676580 | -109.574190 | 602866 | 5503648 | 1 | 868.01 | Local |
| F5 | 12/27/2011 0:00 | 18 | 49.682770 | -109.566860 | 603381 | 5504347 | 1 | 1123.93 | Local |
| F5 | 12/27/2011 0:00 | 21 | 49.683100 | -109.551290 | 604504 | 5504405 | 1 | 1276.54 | Local |
| F5 | 12/28/2011 0:00 | 0 | 49.676340 | -109.536990 | 605550 | 5503673 | 1 | 49.05 | Local |
| F5 | 12/28/2011 0:00 | 3 | 49.676090 | -109.536430 | 605591 | 5503646 | 1 | 17.79 | Local |
| F5 | 12/28/2011 0:00 | 6 | 49.676250 | -109.536430 | 605591 | 5503664 | 1 | 335.22 | Local |
| F5 | 12/28/2011 0:00 | 9 | 49.678210 | -109.532900 | 605841 | 5503887 | 1 | 1230.93 | Local |
| F5 | 12/28/2011 0:00 | 12 | 49.681720 | -109.516720 | 607001 | 5504300 | 1 | 19.57 | Local |

| | | | | | | | | | |
|---|---|---|---|---|---|---|---|---|---|
| F5 | 12/28/2011 0:00 | 15 | 49.681890 | -109.516790 | 606995 | 5504319 | 1 | 6.83 | Local |
| F5 | 12/28/2011 0:00 | 18 | 49.681830 | -109.516770 | 606997 | 5504312 | 0 | 2298.76 | Local |
| F5 | 12/29/2011 0:00 | 0 | 49.667830 | -109.493330 | 608719 | 5502789 | 1 | 209.39 | Local |
| F5 | 12/29/2011 0:00 | 3 | 49.667270 | -109.490560 | 608920 | 5502731 | 1 | 116.83 | Local |
| F5 | 12/29/2011 0:00 | 6 | 49.666790 | -109.492000 | 608817 | 5502676 | 1 | 20.07 | Local |
| F5 | 12/29/2011 0:00 | 9 | 49.666970 | -109.492020 | 608815 | 5502696 | 0 | 1218.20 | Local |
| F5 | 12/29/2011 0:00 | 15 | 49.677750 | -109.489010 | 609008 | 5503898 | 1 | 631.22 | Local |
| F5 | 12/29/2011 0:00 | 18 | 49.683380 | -109.487890 | 609076 | 5504526 | 0 | 57.89 | Local |
| F5 | 12/30/2011 0:00 | 0 | 49.683340 | -109.487090 | 609134 | 5504523 | 0 | 555.17 | Local |
| F5 | 12/30/2011 0:00 | 9 | 49.683160 | -109.479400 | 609689 | 5504514 | 1 | 419.00 | Local |
| F5 | 12/30/2011 0:00 | 12 | 49.684110 | -109.473780 | 610093 | 5504628 | 1 | 269.26 | Local |
| F5 | 12/30/2011 0:00 | 15 | 49.685610 | -109.470850 | 610301 | 5504799 | 1 | 908.08 | Local |
| F5 | 12/30/2011 0:00 | 18 | 49.678810 | -109.463880 | 610819 | 5504053 | 1 | 625.31 | Local |
| F5 | 12/30/2011 0:00 | 21 | 49.683980 | -109.460470 | 611053 | 5504633 | 1 | 234.87 | Local |
| F5 | 12/31/2011 0:00 | 0 | 49.685890 | -109.461860 | 610948 | 5504843 | 1 | 99.79 | Local |
| F5 | 12/31/2011 0:00 | 3 | 49.685270 | -109.462860 | 610878 | 5504773 | 1 | 98.26 | Local |
| F5 | 12/31/2011 0:00 | 6 | 49.685870 | -109.461860 | 610948 | 5504841 | 1 | 6.67 | Local |
| F5 | 12/31/2011 0:00 | 9 | 49.685930 | -109.461860 | 610948 | 5504848 | 0 | 379.52 | Local |
| F5 | 12/31/2011 0:00 | 15 | 49.688940 | -109.464340 | 610763 | 5505179 | 1 | 342.71 | Local |
| F5 | 12/31/2011 0:00 | 18 | 49.690700 | -109.460440 | 611040 | 5505380 | 1 | 603.82 | Local |
| F5 | 12/31/2011 0:00 | 21 | 49.695910 | -109.462800 | 610858 | 5505956 | 1 | 3.98 | Local |
| F5 | 01/01/2012 0:00 | 0 | 49.695940 | -109.462770 | 610860 | 5505959 | 1 | 13.42 | Local |
| F5 | 01/01/2012 0:00 | 3 | 49.695820 | -109.462790 | 610859 | 5505946 | 1 | 12.57 | Local |
| F5 | 01/01/2012 0:00 | 6 | 49.695930 | -109.462750 | 610861 | 5505958 | 1 | 278.30 | Local |
| F5 | 01/01/2012 0:00 | 9 | 49.693940 | -109.465090 | 610697 | 5505733 | 1 | 90.25 | Local |
| F5 | 01/01/2012 0:00 | 12 | 49.694120 | -109.466310 | 610609 | 5505752 | 1 | 13.71 | Local |
| F5 | 01/01/2012 0:00 | 15 | 49.694030 | -109.466180 | 610618 | 5505742 | 1 | 283.95 | Local |
| F5 | 01/01/2012 0:00 | 18 | 49.696210 | -109.464130 | 610761 | 5505987 | 1 | 102.04 | Local |
| F5 | 01/01/2012 0:00 | 21 | 49.695880 | -109.462810 | 610857 | 5505952 | 1 | 10.24 | Local |
| F5 | 01/02/2012 0:00 | 0 | 49.695970 | -109.462840 | 610855 | 5505962 | 1 | 6.83 | Local |
| F5 | 01/02/2012 0:00 | 3 | 49.695910 | -109.462820 | 610856 | 5505956 | 1 | 5.46 | Local |
| F5 | 01/02/2012 0:00 | 6 | 49.695880 | -109.462880 | 610852 | 5505952 | 1 | 565.38 | Local |
| F5 | 01/02/2012 0:00 | 9 | 49.691210 | -109.465980 | 610639 | 5505429 | 1 | 128.91 | Local |
| F5 | 01/02/2012 0:00 | 12 | 49.691050 | -109.464210 | 610767 | 5505413 | 1 | 16.16 | Local |
| F5 | 01/02/2012 0:00 | 15 | 49.691180 | -109.464310 | 610760 | 5505428 | 1 | 257.89 | Local |
| F5 | 01/02/2012 0:00 | 18 | 49.692390 | -109.461260 | 610977 | 5505567 | 1 | 476.48 | Local |
| F5 | 01/02/2012 0:00 | 21 | 49.696160 | -109.464400 | 610742 | 5505981 | 1 | 27.43 | Local |
| F5 | 01/03/2012 0:00 | 0 | 49.696340 | -109.464660 | 610723 | 5506001 | 1 | 141.34 | Local |
| F5 | 01/03/2012 0:00 | 3 | 49.695940 | -109.462800 | 610858 | 5505959 | 0 | 6.83 | Local |
| F5 | 01/03/2012 0:00 | 18 | 49.695880 | -109.462820 | 610856 | 5505952 | 0 | 9.60 | Local |
| F5 | 01/04/2012 0:00 | 3 | 49.695960 | -109.462870 | 610853 | 5505961 | 1 | 11.12 | Local |
| F5 | 01/04/2012 0:00 | 6 | 49.695860 | -109.462870 | 610853 | 5505950 | 1 | 360.88 | Local |
| F5 | 01/04/2012 0:00 | 9 | 49.692770 | -109.464400 | 610750 | 5505604 | 0 | 124.54 | Local |
| F5 | 01/04/2012 0:00 | 15 | 49.691650 | -109.464400 | 610752 | 5505480 | 1 | 392.25 | Local |
| F5 | 01/04/2012 0:00 | 18 | 49.688220 | -109.463130 | 610851 | 5505100 | 1 | 847.44 | Local |
| F5 | 01/04/2012 0:00 | 21 | 49.695840 | -109.462920 | 610849 | 5505948 | 1 | 9.69 | Local |
| F5 | 01/05/2012 0:00 | 0 | 49.695890 | -109.462810 | 610857 | 5505953 | 1 | 5.52 | Local |
| F5 | 01/05/2012 0:00 | 3 | 49.695870 | -109.462880 | 610852 | 5505951 | 1 | 4.47 | Local |
| F5 | 01/05/2012 0:00 | 6 | 49.695860 | -109.462820 | 610856 | 5505950 | 1 | 210.51 | Local |
| F5 | 01/05/2012 0:00 | 9 | 49.694260 | -109.464380 | 610748 | 5505770 | 1 | 350.30 | Local |
| F5 | 01/05/2012 0:00 | 12 | 49.691110 | -109.464310 | 610760 | 5505420 | 1 | 10.01 | Local |
| F5 | 01/05/2012 0:00 | 15 | 49.691200 | -109.464310 | 610760 | 5505430 | 1 | 229.24 | Local |
| F5 | 01/05/2012 0:00 | 18 | 49.692890 | -109.462490 | 610887 | 5505620 | 1 | 339.62 | Local |
| F5 | 01/05/2012 0:00 | 21 | 49.695800 | -109.463920 | 610777 | 5505942 | 0 | 86.52 | Local |
| F5 | 01/06/2012 0:00 | 6 | 49.695940 | -109.462740 | 610862 | 5505959 | 1 | 341.81 | Local |

| | | | | | | | | | | |
|---|---|---|---|---|---|---|---|---|---|---|
| F5 | 01/06/2012 0:00 | 9 | 49.692950 | -109.463840 | 610790 | 5505625 | 1 | 182.29 | Local |
| F5 | 01/06/2012 0:00 | 12 | 49.691320 | -109.464110 | 610774 | 5505443 | 1 | 25.44 | Local |
| F5 | 01/06/2012 0:00 | 15 | 49.691110 | -109.464250 | 610764 | 5505420 | 1 | 244.41 | Local |
| F5 | 01/06/2012 0:00 | 18 | 49.689360 | -109.466300 | 610620 | 5505222 | 1 | 633.42 | Local |
| F5 | 01/06/2012 0:00 | 21 | 49.693190 | -109.472800 | 610143 | 5505639 | 1 | 12.81 | Local |
| F5 | 01/07/2012 0:00 | 0 | 49.693140 | -109.472640 | 610154 | 5505633 | 1 | 1183.53 | Local |
| F5 | 01/07/2012 0:00 | 3 | 49.684490 | -109.482200 | 609484 | 5504658 | 1 | 62.92 | Local |
| F5 | 01/07/2012 0:00 | 6 | 49.684530 | -109.481330 | 609547 | 5504663 | 1 | 11.14 | Local |
| F5 | 01/07/2012 0:00 | 9 | 49.684430 | -109.481320 | 609548 | 5504652 | 1 | 12.25 | Local |
| F5 | 01/07/2012 0:00 | 12 | 49.684540 | -109.481330 | 609547 | 5504664 | 1 | 569.82 | Local |
| F5 | 01/07/2012 0:00 | 15 | 49.685460 | -109.489100 | 608985 | 5504755 | 1 | 1122.38 | Local |
| F5 | 01/07/2012 0:00 | 18 | 49.682390 | -109.503920 | 607922 | 5504393 | 1 | 437.78 | Local |
| F5 | 01/07/2012 0:00 | 21 | 49.683740 | -109.509620 | 607508 | 5504535 | 1 | 1872.27 | Local |
| F5 | 01/08/2012 0:00 | 0 | 49.669240 | -109.522810 | 606589 | 5502904 | 1 | 704.82 | Local |
| F5 | 01/08/2012 0:00 | 3 | 49.662910 | -109.523320 | 606566 | 5502199 | 1 | 1878.35 | Local |
| F5 | 01/08/2012 0:00 | 6 | 49.649950 | -109.506630 | 607799 | 5500782 | 1 | 407.49 | Local |
| F5 | 01/08/2012 0:00 | 9 | 49.646490 | -109.504770 | 607940 | 5500400 | 1 | 460.07 | Local |
| F5 | 01/08/2012 0:00 | 12 | 49.643390 | -109.500550 | 608252 | 5500062 | 1 | 21.79 | Local |
| F5 | 01/08/2012 0:00 | 15 | 49.643560 | -109.500400 | 608262 | 5500081 | 1 | 769.08 | Local |
| F5 | 01/08/2012 0:00 | 18 | 49.638950 | -109.492460 | 608846 | 5499580 | 1 | 1259.74 | Local |
| F5 | 01/08/2012 0:00 | 21 | 49.636710 | -109.509560 | 607616 | 5499306 | 1 | 1362.70 | Local |
| F5 | 01/09/2012 0:00 | 0 | 49.648730 | -109.505880 | 607855 | 5500648 | 1 | 805.42 | Local |
| F5 | 01/09/2012 0:00 | 3 | 49.655970 | -109.505540 | 607864 | 5501453 | 1 | 1.33 | Local |
| F5 | 01/09/2012 0:00 | 6 | 49.655960 | -109.505530 | 607865 | 5501452 | 1 | 494.04 | Local |
| F5 | 01/09/2012 0:00 | 9 | 49.652300 | -109.501650 | 608153 | 5501051 | 1 | 1.82 | Local |
| F5 | 01/09/2012 0:00 | 12 | 49.652310 | -109.501670 | 608151 | 5501052 | 1 | 23.11 | Local |
| F5 | 01/09/2012 0:00 | 15 | 49.652490 | -109.501510 | 608163 | 5501072 | 1 | 173.03 | Local |
| F5 | 01/09/2012 0:00 | 18 | 49.653940 | -109.502380 | 608097 | 5501232 | 1 | 215.14 | Local |
| F5 | 01/09/2012 0:00 | 21 | 49.654850 | -109.505010 | 607905 | 5501330 | 1 | 24.56 | Local |
| F5 | 01/10/2012 0:00 | 0 | 49.655070 | -109.505040 | 607902 | 5501354 | 1 | 103.25 | Local |
| F5 | 01/10/2012 0:00 | 3 | 49.655940 | -109.505540 | 607864 | 5501450 | 1 | 119.46 | Local |
| F5 | 01/10/2012 0:00 | 6 | 49.654890 | -109.505190 | 607892 | 5501334 | 1 | 1229.95 | Local |
| F5 | 01/10/2012 0:00 | 9 | 49.644300 | -109.500270 | 608270 | 5500163 | 1 | 488.06 | Local |
| F5 | 01/10/2012 0:00 | 12 | 49.642170 | -109.494360 | 608702 | 5499935 | 1 | 11.46 | Local |
| F5 | 01/10/2012 0:00 | 15 | 49.642250 | -109.494460 | 608694 | 5499944 | 1 | 779.38 | Local |
| F5 | 01/10/2012 0:00 | 18 | 49.648630 | -109.498930 | 608357 | 5500647 | 1 | 374.16 | Local |
| F5 | 01/10/2012 0:00 | 21 | 49.651990 | -109.499210 | 608330 | 5501020 | 1 | 184.82 | Local |
| F5 | 01/11/2012 0:00 | 0 | 49.653650 | -109.499340 | 608317 | 5501204 | 1 | 280.19 | Local |
| F5 | 01/11/2012 0:00 | 3 | 49.652160 | -109.502470 | 608094 | 5501034 | 1 | 150.49 | Local |
| F5 | 01/11/2012 0:00 | 6 | 49.652320 | -109.504540 | 607944 | 5501049 | 1 | 933.48 | Local |
| F5 | 01/11/2012 0:00 | 9 | 49.644860 | -109.498610 | 608389 | 5500228 | 1 | 412.76 | Local |
| F5 | 01/11/2012 0:00 | 12 | 49.642280 | -109.494500 | 608691 | 5499947 | 1 | 5.74 | Local |
| F5 | 01/11/2012 0:00 | 15 | 49.642230 | -109.494480 | 608693 | 5499942 | 1 | 6.63 | Local |
| F5 | 01/11/2012 0:00 | 18 | 49.642180 | -109.494530 | 608689 | 5499936 | 1 | 7.95 | Local |
| F5 | 01/11/2012 0:00 | 21 | 49.642210 | -109.494430 | 608696 | 5499940 | 1 | 1726.26 | Local |
| F5 | 01/12/2012 0:00 | 0 | 49.655940 | -109.505590 | 607860 | 5501450 | 1 | 94.52 | Local |
| F5 | 01/12/2012 0:00 | 3 | 49.655600 | -109.504390 | 607948 | 5501414 | 1 | 129.13 | Local |
| F5 | 01/12/2012 0:00 | 6 | 49.654920 | -109.505840 | 607845 | 5501336 | 1 | 689.07 | Local |
| F5 | 01/12/2012 0:00 | 9 | 49.649530 | -109.501130 | 608197 | 5500744 | 1 | 209.82 | Local |
| F5 | 01/12/2012 0:00 | 12 | 49.650470 | -109.498610 | 608376 | 5500852 | 1 | 110.15 | Local |
| F5 | 01/12/2012 0:00 | 15 | 49.651090 | -109.499800 | 608289 | 5500919 | 1 | 506.71 | Local |
| F5 | 01/12/2012 0:00 | 18 | 49.653740 | -109.505510 | 607871 | 5501205 | 1 | 311.23 | Local |
| F5 | 01/12/2012 0:00 | 21 | 49.652200 | -109.501910 | 608134 | 5501039 | 1 | 13.53 | Local |
| F5 | 01/13/2012 0:00 | 0 | 49.652310 | -109.501830 | 608140 | 5501052 | 1 | 4.47 | Local |
| F5 | 01/13/2012 0:00 | 3 | 49.652300 | -109.501890 | 608136 | 5501051 | 1 | 5.78 | Local |

| | | | | | | | | | | |
|---|---|---|---|---|---|---|---|---|---|---|
| F5 | 01/13/2012 0:00 | 6 | 49.652300 | -109.501810 | 608141 | 5501051 | 0 | 695.61 | Local |
| F5 | 01/13/2012 0:00 | 12 | 49.647550 | -109.495540 | 608604 | 5500532 | 1 | 19.29 | Local |
| F5 | 01/13/2012 0:00 | 15 | 49.647590 | -109.495280 | 608623 | 5500536 | 1 | 160.05 | Local |
| F5 | 01/13/2012 0:00 | 18 | 49.648970 | -109.494650 | 608665 | 5500691 | 1 | 1346.54 | Local |
| F5 | 01/13/2012 0:00 | 21 | 49.661070 | -109.493900 | 608693 | 5502037 | 1 | 981.55 | Local |
| F5 | 01/14/2012 0:00 | 0 | 49.669760 | -109.496290 | 608501 | 5503000 | 1 | 1218.79 | Local |
| F5 | 01/14/2012 0:00 | 3 | 49.664650 | -109.511230 | 607434 | 5502410 | 1 | 798.56 | Local |
| F5 | 01/14/2012 0:00 | 6 | 49.671270 | -109.515520 | 607110 | 5503140 | 1 | 924.58 | Local |
| F5 | 01/14/2012 0:00 | 9 | 49.676740 | -109.505870 | 607794 | 5503762 | 1 | 1489.48 | Local |
| F5 | 01/14/2012 0:00 | 12 | 49.685260 | -109.489940 | 608924 | 5504732 | 1 | 799.03 | Local |
| F5 | 01/14/2012 0:00 | 15 | 49.684610 | -109.478910 | 609721 | 5504676 | 1 | 1293.19 | Local |
| F5 | 01/14/2012 0:00 | 18 | 49.678050 | -109.464110 | 610804 | 5503968 | 1 | 529.52 | Local |
| F5 | 01/14/2012 0:00 | 21 | 49.682310 | -109.460830 | 611031 | 5504447 | 0 | 1677.25 | Local |
| F5 | 01/15/2012 0:00 | 3 | 49.667710 | -109.466670 | 610643 | 5502815 | 1 | 3.10 | Local |
| F5 | 01/15/2012 0:00 | 6 | 49.667690 | -109.466700 | 610641 | 5502813 | 1 | 399.70 | Local |
| F5 | 01/15/2012 0:00 | 9 | 49.671240 | -109.467570 | 610570 | 5503206 | 1 | 81.56 | Local |
| F5 | 01/15/2012 0:00 | 12 | 49.671970 | -109.467680 | 610560 | 5503287 | 1 | 9.60 | Local |
| F5 | 01/15/2012 0:00 | 15 | 49.672050 | -109.467630 | 610564 | 5503296 | 1 | 468.64 | Local |
| F5 | 01/15/2012 0:00 | 18 | 49.673880 | -109.461780 | 610981 | 5503508 | 1 | 3162.08 | Local |
| F5 | 01/15/2012 0:00 | 21 | 49.665110 | -109.503460 | 607994 | 5502472 | 1 | 1541.93 | Local |
| F5 | 01/16/2012 0:00 | 0 | 49.669900 | -109.523510 | 606537 | 5502976 | 0 | 1640.94 | Local |
| F5 | 01/16/2012 0:00 | 6 | 49.667910 | -109.500980 | 608167 | 5502787 | 1 | 76.60 | Local |
| F5 | 01/16/2012 0:00 | 9 | 49.668370 | -109.501770 | 608109 | 5502837 | 1 | 698.03 | Local |
| F5 | 01/16/2012 0:00 | 12 | 49.674560 | -109.500160 | 608211 | 5503528 | 1 | 0.00 | Local |
| F5 | 01/16/2012 0:00 | 15 | 49.674560 | -109.500160 | 608211 | 5503528 | 1 | 3.34 | Local |
| F5 | 01/16/2012 0:00 | 18 | 49.674530 | -109.500160 | 608211 | 5503524 | 1 | 7.95 | Local |
| F5 | 01/16/2012 0:00 | 21 | 49.674560 | -109.500060 | 608218 | 5503528 | 1 | 995.40 | Local |
| F5 | 01/17/2012 0:00 | 0 | 49.667520 | -109.508580 | 607619 | 5502733 | 1 | 1728.32 | Local |
| F5 | 01/17/2012 0:00 | 3 | 49.651990 | -109.507590 | 607725 | 5501008 | 0 | 1247.39 | Local |
| F5 | 01/17/2012 0:00 | 12 | 49.642540 | -109.498280 | 608418 | 5499971 | 1 | 4.47 | Local |
| F5 | 01/17/2012 0:00 | 15 | 49.642550 | -109.498220 | 608422 | 5499972 | 1 | 460.17 | Local |
| F5 | 01/17/2012 0:00 | 18 | 49.642970 | -109.491880 | 608879 | 5500028 | 1 | 1594.64 | Local |
| F5 | 01/17/2012 0:00 | 21 | 49.656160 | -109.500550 | 608224 | 5501482 | 1 | 206.36 | Local |
| F5 | 01/18/2012 0:00 | 0 | 49.657440 | -109.498480 | 608370 | 5501627 | 1 | 25.16 | Local |
| F5 | 01/18/2012 0:00 | 3 | 49.657270 | -109.498710 | 608354 | 5501608 | 0 | 858.22 | Local |
| F5 | 01/18/2012 0:00 | 9 | 49.649730 | -109.496170 | 608554 | 5500773 | 1 | 410.66 | Local |
| F5 | 01/18/2012 0:00 | 12 | 49.652770 | -109.492940 | 608780 | 5501116 | 1 | 1547.33 | Local |
| F5 | 01/18/2012 0:00 | 15 | 49.655770 | -109.472010 | 610284 | 5501480 | 1 | 272.32 | Local |
| F5 | 01/18/2012 0:00 | 18 | 49.657280 | -109.474980 | 610067 | 5501643 | 1 | 1532.29 | Local |
| F5 | 01/18/2012 0:00 | 21 | 49.664130 | -109.493400 | 608722 | 5502378 | 1 | 10.35 | Local |
| F5 | 01/19/2012 0:00 | 0 | 49.664110 | -109.493260 | 608732 | 5502376 | 1 | 929.81 | Local |
| F5 | 01/19/2012 0:00 | 3 | 49.657470 | -109.501090 | 608182 | 5501626 | 0 | 2362.27 | Local |
| F5 | 01/19/2012 0:00 | 12 | 49.678710 | -109.500410 | 608184 | 5503989 | 1 | 40.32 | Local |
| F5 | 01/19/2012 0:00 | 15 | 49.678400 | -109.500120 | 608205 | 5503955 | 1 | 5.17 | Local |
| F5 | 01/19/2012 0:00 | 18 | 49.678410 | -109.500050 | 608210 | 5503956 | 1 | 14.22 | Local |
| F5 | 01/19/2012 0:00 | 21 | 49.678500 | -109.500190 | 608200 | 5503966 | 1 | 10.60 | Local |
| F5 | 01/20/2012 0:00 | 0 | 49.678420 | -109.500270 | 608194 | 5503957 | 1 | 12.71 | Local |
| F5 | 01/20/2012 0:00 | 3 | 49.678450 | -109.500100 | 608207 | 5503960 | 1 | 17.57 | Local |
| F5 | 01/20/2012 0:00 | 6 | 49.678370 | -109.499890 | 608222 | 5503952 | 1 | 39.56 | Local |
| F5 | 01/20/2012 0:00 | 9 | 49.678620 | -109.500280 | 608193 | 5503979 | 1 | 67.26 | Local |
| F5 | 01/20/2012 0:00 | 12 | 49.678080 | -109.499860 | 608225 | 5503919 | 1 | 4.87 | Local |
| F5 | 01/20/2012 0:00 | 15 | 49.678060 | -109.499920 | 608221 | 5503917 | 1 | 68.50 | Local |
| F5 | 01/20/2012 0:00 | 18 | 49.678630 | -109.500280 | 608193 | 5503980 | 1 | 722.03 | Local |
| F5 | 01/20/2012 0:00 | 21 | 49.677600 | -109.510160 | 607483 | 5503851 | 1 | 13.52 | Local |
| F5 | 01/21/2012 0:00 | 0 | 49.677480 | -109.510130 | 607485 | 5503838 | 1 | 692.55 | Local |

| | | | | | | | | | |
|---|---|---|---|---|---|---|---|---|---|
| F5 | 01/21/2012 0:00 | 3 | 49.678570 | -109.500680 | 608165 | 5503973 | 1 | 27.81 | Local |
| F5 | 01/21/2012 0:00 | 6 | 49.678500 | -109.501050 | 608138 | 5503964 | 1 | 367.61 | Local |
| F5 | 01/21/2012 0:00 | 9 | 49.681700 | -109.499770 | 608223 | 5504322 | 1 | 217.28 | Local |
| F5 | 01/21/2012 0:00 | 12 | 49.679760 | -109.500130 | 608202 | 5504106 | 1 | 52.26 | Local |
| F5 | 01/21/2012 0:00 | 15 | 49.679420 | -109.500630 | 608166 | 5504067 | 1 | 91.46 | Local |
| F5 | 01/21/2012 0:00 | 18 | 49.678600 | -109.500530 | 608175 | 5503976 | 0 | 84.31 | Local |
| F5 | 01/22/2012 0:00 | 3 | 49.677890 | -109.500940 | 608147 | 5503897 | 1 | 399.96 | Local |
| F5 | 01/22/2012 0:00 | 6 | 49.676680 | -109.506160 | 607773 | 5503755 | 1 | 703.23 | Local |
| F5 | 01/22/2012 0:00 | 9 | 49.681140 | -109.499250 | 608262 | 5504261 | 1 | 15.41 | Local |
| F5 | 01/22/2012 0:00 | 12 | 49.681250 | -109.499380 | 608252 | 5504273 | 1 | 13.36 | Local |
| F5 | 01/22/2012 0:00 | 15 | 49.681370 | -109.499390 | 608251 | 5504286 | 1 | 19.65 | Local |
| F5 | 01/22/2012 0:00 | 18 | 49.681300 | -109.499640 | 608234 | 5504278 | 1 | 28.06 | Local |
| F5 | 01/22/2012 0:00 | 21 | 49.681190 | -109.499290 | 608259 | 5504266 | 1 | 1479.29 | Local |
| F5 | 01/23/2012 0:00 | 0 | 49.686940 | -109.517780 | 606913 | 5504879 | 1 | 2197.25 | Local |
| F5 | 01/23/2012 0:00 | 3 | 49.667580 | -109.523880 | 606515 | 5502718 | 1 | 2755.49 | Local |
| F5 | 01/23/2012 0:00 | 6 | 49.646680 | -109.503370 | 608041 | 5500424 | 1 | 596.33 | Local |
| F5 | 01/23/2012 0:00 | 9 | 49.647550 | -109.495220 | 608628 | 5500532 | 1 | 1002.78 | Local |
| F5 | 01/23/2012 0:00 | 12 | 49.654490 | -109.486350 | 609252 | 5501316 | 1 | 9.64 | Local |
| F5 | 01/23/2012 0:00 | 15 | 49.654470 | -109.486220 | 609262 | 5501314 | 1 | 184.04 | Local |
| F5 | 01/23/2012 0:00 | 18 | 49.654880 | -109.488690 | 609083 | 5501356 | 1 | 919.27 | Local |
| F5 | 01/23/2012 0:00 | 21 | 49.662250 | -109.494460 | 608650 | 5502167 | 1 | 553.70 | Local |
| F5 | 01/24/2012 0:00 | 0 | 49.666960 | -109.491970 | 608819 | 5502695 | 0 | 651.19 | Local |
| F5 | 01/24/2012 0:00 | 6 | 49.669730 | -109.499920 | 608239 | 5502991 | 1 | 1284.75 | Local |
| F5 | 01/24/2012 0:00 | 9 | 49.676370 | -109.485350 | 609275 | 5503750 | 0 | 77.57 | Local |
| F5 | 01/24/2012 0:00 | 15 | 49.676720 | -109.484420 | 609342 | 5503791 | 1 | 1599.24 | Local |
| F5 | 01/24/2012 0:00 | 18 | 49.685420 | -109.466770 | 610595 | 5504784 | 1 | 2675.31 | Local |
| F5 | 01/24/2012 0:00 | 21 | 49.709380 | -109.463400 | 610784 | 5507452 | 1 | 3646.31 | Local |
| F5 | 01/25/2012 0:00 | 0 | 49.706980 | -109.513830 | 607153 | 5507112 | 1 | 2634.58 | Local |
| F5 | 01/25/2012 0:00 | 3 | 49.698330 | -109.547840 | 604720 | 5506103 | 1 | 8.08 | Local |
| F5 | 01/25/2012 0:00 | 6 | 49.698400 | -109.547810 | 604722 | 5506111 | 1 | 349.85 | Local |
| F5 | 01/25/2012 0:00 | 9 | 49.695410 | -109.546300 | 604837 | 5505780 | 1 | 32.25 | Local |
| F5 | 01/25/2012 0:00 | 12 | 49.695120 | -109.546300 | 604838 | 5505748 | 1 | 5.17 | Local |
| F5 | 01/25/2012 0:00 | 15 | 49.695130 | -109.546370 | 604833 | 5505749 | 1 | 2.43 | Local |
| F5 | 01/25/2012 0:00 | 18 | 49.695120 | -109.546400 | 604831 | 5505748 | 1 | 385.38 | Local |
| F5 | 01/25/2012 0:00 | 21 | 49.698470 | -109.547770 | 604725 | 5506118 | 1 | 33.94 | Local |
| F5 | 01/26/2012 0:00 | 0 | 49.698220 | -109.547500 | 604745 | 5506091 | 1 | 28.19 | Local |
| F5 | 01/26/2012 0:00 | 3 | 49.698390 | -109.547790 | 604723 | 5506109 | 1 | 23.86 | Local |
| F5 | 01/26/2012 0:00 | 6 | 49.698190 | -109.547670 | 604733 | 5506087 | 1 | 60.08 | Local |
| F5 | 01/26/2012 0:00 | 9 | 49.697790 | -109.547110 | 604774 | 5506044 | 1 | 300.45 | Local |
| F5 | 01/26/2012 0:00 | 12 | 49.695120 | -109.546470 | 604826 | 5505748 | 1 | 374.96 | Local |
| F5 | 01/26/2012 0:00 | 15 | 49.698390 | -109.547740 | 604727 | 5506110 | 1 | 574.12 | Local |
| F5 | 01/26/2012 0:00 | 18 | 49.694740 | -109.542110 | 605141 | 5505712 | 1 | 829.11 | Local |
| F5 | 01/26/2012 0:00 | 21 | 49.696390 | -109.553320 | 604329 | 5505879 | 1 | 283.79 | Local |
| F5 | 01/27/2012 0:00 | 0 | 49.696170 | -109.557240 | 604047 | 5505850 | 1 | 2021.59 | Local |
| F5 | 01/27/2012 0:00 | 3 | 49.680850 | -109.572330 | 602991 | 5504126 | 1 | 334.00 | Local |
| F5 | 01/27/2012 0:00 | 6 | 49.680460 | -109.576920 | 602661 | 5504076 | 1 | 477.63 | Local |
| F5 | 01/27/2012 0:00 | 9 | 49.677340 | -109.581470 | 602339 | 5503723 | 1 | 461.34 | Local |
| F5 | 01/27/2012 0:00 | 12 | 49.675160 | -109.586910 | 601951 | 5503473 | 1 | 3.64 | Local |
| F5 | 01/27/2012 0:00 | 15 | 49.675180 | -109.586870 | 601954 | 5503475 | 0 | 1119.72 | Local |
| F5 | 01/27/2012 0:00 | 21 | 49.668470 | -109.598440 | 601133 | 5502714 | 1 | 2232.05 | Local |
| F5 | 01/28/2012 0:00 | 0 | 49.662740 | -109.568800 | 603284 | 5502117 | 1 | 2062.81 | Local |
| F5 | 01/28/2012 0:00 | 3 | 49.681110 | -109.572790 | 602957 | 5504154 | 1 | 32.54 | Local |
| F5 | 01/28/2012 0:00 | 6 | 49.681130 | -109.572340 | 602990 | 5504157 | 1 | 9.69 | Local |
| F5 | 01/28/2012 0:00 | 9 | 49.681200 | -109.572420 | 602984 | 5504164 | 1 | 4.41 | Local |
| F5 | 01/28/2012 0:00 | 12 | 49.681170 | -109.572380 | 602987 | 5504161 | 1 | 3.41 | Local |

| | | | | | | | | | |
|---|---|---|---|---|---|---|---|---|---|
| F5 | 01/28/2012 0:00 | 15 | 49.681200 | -109.572390 | 602986 | 5504164 | 1 | 318.98 | Local |
| F5 | 01/28/2012 0:00 | 18 | 49.683010 | -109.568960 | 603229 | 5504370 | 1 | 3.77 | Local |
| F5 | 01/28/2012 0:00 | 21 | 49.683020 | -109.569010 | 603226 | 5504371 | 1 | 1784.95 | Local |
| F5 | 01/29/2012 0:00 | 0 | 49.696960 | -109.556740 | 604081 | 5505938 | 1 | 865.15 | Local |
| F5 | 01/29/2012 0:00 | 3 | 49.704490 | -109.553720 | 604283 | 5506779 | 1 | 479.88 | Local |
| F5 | 01/29/2012 0:00 | 6 | 49.700380 | -109.555750 | 604145 | 5506320 | 1 | 498.64 | Local |
| F5 | 01/29/2012 0:00 | 9 | 49.700230 | -109.548840 | 604644 | 5506313 | 1 | 622.44 | Local |
| F5 | 01/29/2012 0:00 | 12 | 49.694960 | -109.545930 | 604865 | 5505731 | 1 | 39.94 | Local |
| F5 | 01/29/2012 0:00 | 15 | 49.695160 | -109.546390 | 604831 | 5505752 | 1 | 8.58 | Local |
| F5 | 01/29/2012 0:00 | 18 | 49.695090 | -109.546340 | 604835 | 5505745 | 1 | 819.00 | Local |
| F5 | 01/29/2012 0:00 | 21 | 49.698300 | -109.556560 | 604091 | 5506087 | 1 | 671.31 | Local |
| F5 | 01/30/2012 0:00 | 0 | 49.701440 | -109.548610 | 604658 | 5506447 | 1 | 818.11 | Local |
| F5 | 01/30/2012 0:00 | 3 | 49.695180 | -109.542650 | 605101 | 5505760 | 1 | 26.00 | Local |
| F5 | 01/30/2012 0:00 | 6 | 49.695000 | -109.542880 | 605085 | 5505739 | 1 | 18.15 | Local |
| F5 | 01/30/2012 0:00 | 9 | 49.695160 | -109.542930 | 605081 | 5505757 | 1 | 176.18 | Local |
| F5 | 01/30/2012 0:00 | 12 | 49.693980 | -109.541300 | 605201 | 5505628 | 1 | 81.08 | Local |
| F5 | 01/30/2012 0:00 | 15 | 49.693420 | -109.542020 | 605150 | 5505565 | 1 | 359.38 | Local |
| F5 | 01/30/2012 0:00 | 18 | 49.690200 | -109.541590 | 605188 | 5505208 | 1 | 1386.42 | Local |
| F5 | 01/30/2012 0:00 | 21 | 49.679040 | -109.533020 | 605831 | 5503979 | 1 | 1190.27 | Local |
| F5 | 01/31/2012 0:00 | 0 | 49.672170 | -109.520370 | 606758 | 5503233 | 1 | 1572.26 | Local |
| F5 | 01/31/2012 0:00 | 3 | 49.663990 | -109.502600 | 608058 | 5502349 | 1 | 1035.95 | Local |
| F5 | 01/31/2012 0:00 | 6 | 49.660520 | -109.515920 | 607105 | 5501944 | 1 | 1908.26 | Local |
| F5 | 01/31/2012 0:00 | 9 | 49.677570 | -109.512910 | 607285 | 5503844 | 1 | 5.97 | Local |
| F5 | 01/31/2012 0:00 | 12 | 49.677520 | -109.512880 | 607287 | 5503839 | 1 | 11.55 | Local |
| F5 | 01/31/2012 0:00 | 15 | 49.677610 | -109.512960 | 607281 | 5503848 | 1 | 0.72 | Local |
| F5 | 01/31/2012 0:00 | 18 | 49.677610 | -109.512950 | 607282 | 5503848 | 1 | 46.95 | Local |
| F5 | 01/31/2012 0:00 | 21 | 49.677630 | -109.512300 | 607328 | 5503852 | 0 | 2448.93 | Local |
| F5 | 02/01/2012 0:00 | 6 | 49.658960 | -109.494300 | 608669 | 5501802 | 1 | 535.61 | Local |
| F5 | 02/01/2012 0:00 | 9 | 49.662760 | -109.489740 | 608989 | 5502231 | 1 | 8.30 | Local |
| F5 | 02/01/2012 0:00 | 12 | 49.662830 | -109.489700 | 608992 | 5502239 | 1 | 10.11 | Local |
| F5 | 02/01/2012 0:00 | 15 | 49.662740 | -109.489680 | 608993 | 5502229 | 1 | 1334.64 | Local |
| F5 | 02/01/2012 0:00 | 18 | 49.657480 | -109.473060 | 610205 | 5501668 | 1 | 1781.28 | Local |
| F5 | 02/01/2012 0:00 | 21 | 49.646220 | -109.455510 | 611497 | 5500442 | 1 | 621.68 | Local |
| F5 | 02/02/2012 0:00 | 0 | 49.645560 | -109.446960 | 612116 | 5500382 | 1 | 1941.66 | Local |
| F5 | 02/02/2012 0:00 | 3 | 49.654370 | -109.470180 | 610420 | 5501327 | 1 | 591.01 | Local |
| F5 | 02/02/2012 0:00 | 6 | 49.658160 | -109.475920 | 609997 | 5501740 | 1 | 154.67 | Local |
| F5 | 02/02/2012 0:00 | 9 | 49.656910 | -109.474980 | 610067 | 5501602 | 1 | 3.98 | Local |
| F5 | 02/02/2012 0:00 | 12 | 49.656940 | -109.475010 | 610065 | 5501605 | 1 | 4.51 | Local |
| F5 | 02/02/2012 0:00 | 15 | 49.656900 | -109.475000 | 610066 | 5501601 | 1 | 108.32 | Local |
| F5 | 02/02/2012 0:00 | 18 | 49.657720 | -109.475810 | 610006 | 5501691 | 0 | 99.30 | Local |
| F5 | 02/03/2012 0:00 | 0 | 49.656980 | -109.475040 | 610063 | 5501610 | 1 | 95.45 | Local |
| F5 | 02/03/2012 0:00 | 3 | 49.657700 | -109.475760 | 610009 | 5501689 | 1 | 9.28 | Local |
| F5 | 02/03/2012 0:00 | 6 | 49.657770 | -109.475830 | 610004 | 5501696 | 1 | 199.75 | Local |
| F5 | 02/03/2012 0:00 | 9 | 49.656440 | -109.473970 | 610141 | 5501551 | 1 | 32.16 | Local |
| F5 | 02/03/2012 0:00 | 12 | 49.656670 | -109.474240 | 610121 | 5501576 | 1 | 13.42 | Local |
| F5 | 02/03/2012 0:00 | 15 | 49.656790 | -109.474260 | 610120 | 5501590 | 1 | 142.12 | Local |
| F5 | 02/03/2012 0:00 | 18 | 49.657850 | -109.475360 | 610038 | 5501706 | 1 | 41.82 | Local |
| F5 | 02/03/2012 0:00 | 21 | 49.657660 | -109.475860 | 610002 | 5501684 | 1 | 5.61 | Local |
| F5 | 02/04/2012 0:00 | 0 | 49.657710 | -109.475870 | 610001 | 5501690 | 1 | 7.59 | Local |
| F5 | 02/04/2012 0:00 | 3 | 49.657770 | -109.475820 | 610005 | 5501696 | 0 | 106.25 | Local |
| F5 | 02/04/2012 0:00 | 9 | 49.657150 | -109.474700 | 610087 | 5501629 | 1 | 113.91 | Local |
| F5 | 02/04/2012 0:00 | 12 | 49.657740 | -109.475990 | 609993 | 5501693 | 1 | 3.34 | Local |
| F5 | 02/04/2012 0:00 | 15 | 49.657770 | -109.475990 | 609993 | 5501696 | 1 | 7.95 | Local |
| F5 | 02/04/2012 0:00 | 18 | 49.657800 | -109.476090 | 609985 | 5501699 | 1 | 15.52 | Local |
| F5 | 02/04/2012 0:00 | 21 | 49.657700 | -109.475940 | 609996 | 5501689 | 1 | 23.63 | Local |

| | | | | | | | | | |
|---|---|---|---|---|---|---|---|---|---|
| F5 | 02/05/2012 0:00 | 0 | 49.657910 | -109.475990 | 609992 | 5501712 | 1 | 2.65 | Local |
| F5 | 02/05/2012 0:00 | 3 | 49.657930 | -109.476010 | 609991 | 5501714 | 1 | 116.99 | Local |
| F5 | 02/05/2012 0:00 | 6 | 49.657400 | -109.474610 | 610093 | 5501657 | 1 | 109.70 | Local |
| F5 | 02/05/2012 0:00 | 9 | 49.657900 | -109.475920 | 609997 | 5501711 | 1 | 444.47 | Local |
| F5 | 02/05/2012 0:00 | 12 | 49.655910 | -109.470580 | 610387 | 5501497 | 1 | 10.39 | Local |
| F5 | 02/05/2012 0:00 | 15 | 49.655870 | -109.470450 | 610397 | 5501493 | 1 | 462.71 | Local |
| F5 | 02/05/2012 0:00 | 18 | 49.658050 | -109.475910 | 609998 | 5501727 | 1 | 16.74 | Local |
| F5 | 02/05/2012 0:00 | 21 | 49.657930 | -109.475770 | 610008 | 5501714 | 1 | 22.33 | Local |
| F5 | 02/06/2012 0:00 | 0 | 49.657740 | -109.475870 | 610001 | 5501693 | 1 | 6.67 | Local |
| F5 | 02/06/2012 0:00 | 3 | 49.657680 | -109.475870 | 610001 | 5501686 | 1 | 258.35 | Local |
| F5 | 02/06/2012 0:00 | 6 | 49.657260 | -109.472350 | 610256 | 5501645 | 1 | 7.30 | Local |
| F5 | 02/06/2012 0:00 | 9 | 49.657250 | -109.472250 | 610264 | 5501644 | 1 | 931.16 | Local |
| F5 | 02/06/2012 0:00 | 12 | 49.663140 | -109.481420 | 609589 | 5502285 | 1 | 8.91 | Local |
| F5 | 02/06/2012 0:00 | 15 | 49.663070 | -109.481360 | 609593 | 5502278 | 1 | 541.79 | Local |
| F5 | 02/06/2012 0:00 | 18 | 49.659090 | -109.477030 | 609915 | 5501841 | 1 | 1846.87 | Local |
| F5 | 02/06/2012 0:00 | 21 | 49.656040 | -109.502180 | 608106 | 5501466 | 1 | 52.95 | Local |
| F5 | 02/07/2012 0:00 | 0 | 49.656490 | -109.502420 | 608088 | 5501516 | 1 | 633.83 | Local |
| F5 | 02/07/2012 0:00 | 3 | 49.662190 | -109.502500 | 608070 | 5502149 | 1 | 625.43 | Local |
| F5 | 02/07/2012 0:00 | 6 | 49.665880 | -109.509040 | 607589 | 5502550 | 1 | 1325.81 | Local |
| F5 | 02/07/2012 0:00 | 9 | 49.677540 | -109.512880 | 607287 | 5503841 | 1 | 432.54 | Local |
| F5 | 02/07/2012 0:00 | 12 | 49.680930 | -109.509940 | 607491 | 5504222 | 1 | 149.70 | Local |
| F5 | 02/07/2012 0:00 | 15 | 49.682270 | -109.509740 | 607503 | 5504371 | 1 | 455.23 | Local |
| F5 | 02/07/2012 0:00 | 18 | 49.683030 | -109.515940 | 607054 | 5504447 | 1 | 2450.76 | Local |
| F5 | 02/07/2012 0:00 | 21 | 49.661300 | -109.521620 | 606692 | 5502023 | 1 | 1255.11 | Local |
| F5 | 02/08/2012 0:00 | 0 | 49.662870 | -109.504400 | 607931 | 5502222 | 1 | 783.92 | Local |
| F5 | 02/08/2012 0:00 | 3 | 49.664080 | -109.493700 | 608700 | 5502372 | 1 | 1460.68 | Local |
| F5 | 02/08/2012 0:00 | 6 | 49.657940 | -109.475810 | 610005 | 5501715 | 1 | 700.87 | Local |
| F5 | 02/08/2012 0:00 | 9 | 49.663080 | -109.481430 | 609588 | 5502279 | 1 | 3.78 | Local |
| F5 | 02/08/2012 0:00 | 12 | 49.663070 | -109.481380 | 609592 | 5502278 | 1 | 5.74 | Local |
| F5 | 02/08/2012 0:00 | 15 | 49.663120 | -109.481400 | 609590 | 5502283 | 1 | 3.61 | Local |
| F5 | 02/08/2012 0:00 | 18 | 49.663120 | -109.481350 | 609594 | 5502283 | 1 | 3.09 | Local |
| F5 | 02/08/2012 0:00 | 21 | 49.663130 | -109.481390 | 609591 | 5502284 | 1 | 1143.92 | Local |
| F5 | 02/09/2012 0:00 | 0 | 49.666960 | -109.496100 | 608521 | 5502689 | 1 | 1401.36 | Local |
| F5 | 02/09/2012 0:00 | 3 | 49.672340 | -109.513660 | 607242 | 5503262 | 1 | 2149.66 | Local |
| F5 | 02/09/2012 0:00 | 6 | 49.691050 | -109.521160 | 606660 | 5505331 | 1 | 792.28 | Local |
| F5 | 02/09/2012 0:00 | 9 | 49.692740 | -109.510490 | 607426 | 5505534 | 1 | 49.84 | Local |
| F5 | 02/09/2012 0:00 | 12 | 49.693110 | -109.510880 | 607397 | 5505575 | 1 | 21.32 | Local |
| F5 | 02/09/2012 0:00 | 15 | 49.693300 | -109.510920 | 607393 | 5505596 | 1 | 1330.98 | Local |
| F5 | 02/09/2012 0:00 | 18 | 49.705060 | -109.507480 | 607616 | 5506908 | 1 | 738.96 | Local |
| F5 | 02/09/2012 0:00 | 21 | 49.710600 | -109.513140 | 607195 | 5507516 | 1 | 1425.30 | Local |
| F5 | 02/10/2012 0:00 | 0 | 49.714640 | -109.531900 | 605834 | 5507938 | 1 | 1081.07 | Local |
| F5 | 02/10/2012 0:00 | 3 | 49.705100 | -109.529010 | 606063 | 5506882 | 1 | 42.47 | Local |
| F5 | 02/10/2012 0:00 | 6 | 49.705480 | -109.528950 | 606067 | 5506924 | 1 | 9.15 | Local |
| F5 | 02/10/2012 0:00 | 9 | 49.705400 | -109.528920 | 606069 | 5506915 | 0 | 325.01 | Local |
| F5 | 02/10/2012 0:00 | 15 | 49.703120 | -109.531740 | 605871 | 5506658 | 1 | 712.68 | Local |
| F5 | 02/10/2012 0:00 | 18 | 49.696780 | -109.533190 | 605780 | 5505951 | 1 | 1408.72 | Local |
| F5 | 02/10/2012 0:00 | 21 | 49.684730 | -109.527160 | 606241 | 5504620 | 1 | 2499.74 | Local |
| F5 | 02/11/2012 0:00 | 0 | 49.666220 | -109.507500 | 607700 | 5502590 | 1 | 687.68 | Local |
| F5 | 02/11/2012 0:00 | 3 | 49.668950 | -109.498950 | 608311 | 5502906 | 1 | 3067.74 | Local |
| F5 | 02/11/2012 0:00 | 6 | 49.641420 | -109.496170 | 608573 | 5499849 | 1 | 492.79 | Local |
| F5 | 02/11/2012 0:00 | 9 | 49.641260 | -109.489350 | 609065 | 5499841 | 1 | 1.11 | Local |
| F5 | 02/11/2012 0:00 | 12 | 49.641250 | -109.489350 | 609065 | 5499840 | 1 | 1.44 | Local |
| F5 | 02/11/2012 0:00 | 15 | 49.641250 | -109.489370 | 609064 | 5499840 | 1 | 3.10 | Local |
| F5 | 02/11/2012 0:00 | 18 | 49.641230 | -109.489400 | 609062 | 5499838 | 1 | 715.98 | Local |
| F5 | 02/11/2012 0:00 | 21 | 49.644480 | -109.497960 | 608437 | 5500187 | 1 | 2170.70 | Local |

| | | | | | | | | | |
|---|---|---|---|---|---|---|---|---|---|
| F5 | 02/12/2012 0:00 | 0 | 49.663260 | -109.506170 | 607802 | 5502263 | 0 | 1466.93 | Local |
| F5 | 02/12/2012 0:00 | 6 | 49.676260 | -109.502710 | 608023 | 5503713 | 1 | 1189.63 | Local |
| F5 | 02/12/2012 0:00 | 9 | 49.681890 | -109.516730 | 606999 | 5504319 | 1 | 14.27 | Local |
| F5 | 02/12/2012 0:00 | 12 | 49.681770 | -109.516660 | 607005 | 5504306 | 1 | 14.57 | Local |
| F5 | 02/12/2012 0:00 | 15 | 49.681850 | -109.516820 | 606993 | 5504314 | 1 | 199.07 | Local |
| F5 | 02/12/2012 0:00 | 18 | 49.680420 | -109.518480 | 606876 | 5504153 | 1 | 769.61 | Local |
| F5 | 02/12/2012 0:00 | 21 | 49.677780 | -109.528340 | 606171 | 5503845 | 1 | 2293.60 | Local |
| F5 | 02/13/2012 0:00 | 0 | 49.684040 | -109.558630 | 603972 | 5504499 | 1 | 784.25 | Local |
| F5 | 02/13/2012 0:00 | 3 | 49.685890 | -109.569120 | 603212 | 5504690 | 1 | 1077.38 | Local |
| F5 | 02/13/2012 0:00 | 6 | 49.680910 | -109.581930 | 602298 | 5504119 | 1 | 371.12 | Local |
| F5 | 02/13/2012 0:00 | 9 | 49.677890 | -109.584120 | 602147 | 5503780 | 1 | 36.76 | Local |
| F5 | 02/13/2012 0:00 | 12 | 49.677560 | -109.584150 | 602145 | 5503744 | 0 | 57.27 | Local |
| F5 | 02/13/2012 0:00 | 18 | 49.677610 | -109.583360 | 602202 | 5503750 | 0 | 66.08 | Local |
| F5 | 02/14/2012 0:00 | 0 | 49.677720 | -109.584260 | 602137 | 5503761 | 1 | 14.46 | Local |
| F5 | 02/14/2012 0:00 | 3 | 49.677590 | -109.584260 | 602137 | 5503747 | 1 | 1.11 | Local |
| F5 | 02/14/2012 0:00 | 6 | 49.677600 | -109.584260 | 602137 | 5503748 | 1 | 72.68 | Local |
| F5 | 02/14/2012 0:00 | 9 | 49.677130 | -109.583560 | 602189 | 5503697 | 1 | 42.80 | Local |
| F5 | 02/14/2012 0:00 | 12 | 49.677420 | -109.583950 | 602160 | 5503728 | 1 | 1.44 | Local |
| F5 | 02/14/2012 0:00 | 15 | 49.677420 | -109.583970 | 602158 | 5503728 | 1 | 10.14 | Local |
| F5 | 02/14/2012 0:00 | 18 | 49.677490 | -109.584060 | 602152 | 5503736 | 1 | 27.32 | Local |
| F5 | 02/14/2012 0:00 | 21 | 49.677690 | -109.584280 | 602136 | 5503758 | 1 | 41.49 | Local |
| F5 | 02/15/2012 0:00 | 0 | 49.677380 | -109.583960 | 602159 | 5503724 | 1 | 6.73 | Local |
| F5 | 02/15/2012 0:00 | 3 | 49.677420 | -109.584030 | 602154 | 5503728 | 1 | 5.05 | Local |
| F5 | 02/15/2012 0:00 | 6 | 49.677420 | -109.583960 | 602159 | 5503728 | 1 | 4.68 | Local |
| F5 | 02/15/2012 0:00 | 9 | 49.677460 | -109.583980 | 602158 | 5503733 | 1 | 7.82 | Local |
| F5 | 02/15/2012 0:00 | 12 | 49.677390 | -109.583970 | 602159 | 5503725 | 1 | 12.53 | Local |
| F5 | 02/15/2012 0:00 | 15 | 49.677490 | -109.583890 | 602164 | 5503736 | 1 | 8.37 | Local |
| F5 | 02/15/2012 0:00 | 18 | 49.677430 | -109.583960 | 602159 | 5503729 | 1 | 7.05 | Local |
| F5 | 02/15/2012 0:00 | 21 | 49.677380 | -109.584020 | 602155 | 5503724 | 0 | 11.76 | Local |
| F5 | 02/16/2012 0:00 | 3 | 49.677400 | -109.583860 | 602166 | 5503726 | 1 | 8.48 | Local |
| F5 | 02/16/2012 0:00 | 6 | 49.677440 | -109.583960 | 602159 | 5503731 | 1 | 60.78 | Local |
| F5 | 02/16/2012 0:00 | 9 | 49.677910 | -109.584390 | 602127 | 5503782 | 0 | 3.64 | Local |
| F5 | 02/16/2012 0:00 | 18 | 49.677930 | -109.584350 | 602130 | 5503785 | 1 | 3.10 | Local |
| F5 | 02/16/2012 0:00 | 21 | 49.677950 | -109.584320 | 602132 | 5503787 | 1 | 4.45 | Local |
| F5 | 02/17/2012 0:00 | 0 | 49.677910 | -109.584320 | 602132 | 5503782 | 1 | 1.44 | Local |
| F5 | 02/17/2012 0:00 | 3 | 49.677910 | -109.584300 | 602134 | 5503782 | 1 | 2.16 | Local |
| F5 | 02/17/2012 0:00 | 6 | 49.677910 | -109.584330 | 602131 | 5503782 | 1 | 60.06 | Local |
| F5 | 02/17/2012 0:00 | 9 | 49.677370 | -109.584350 | 602131 | 5503722 | 0 | 51.35 | Local |
| F5 | 02/17/2012 0:00 | 15 | 49.677820 | -109.584510 | 602119 | 5503772 | 1 | 11.64 | Local |
| F5 | 02/17/2012 0:00 | 18 | 49.677890 | -109.584390 | 602127 | 5503780 | 1 | 2.65 | Local |
| F5 | 02/17/2012 0:00 | 21 | 49.677870 | -109.584370 | 602129 | 5503778 | 0 | 1.82 | Local |
| F5 | 02/18/2012 0:00 | 6 | 49.677880 | -109.584390 | 602127 | 5503779 | 1 | 275.21 | Local |
| F5 | 02/18/2012 0:00 | 9 | 49.676280 | -109.581480 | 602340 | 5503605 | 1 | 520.82 | Local |
| F5 | 02/18/2012 0:00 | 12 | 49.676500 | -109.574270 | 602860 | 5503639 | 1 | 6.86 | Local |
| F5 | 02/18/2012 0:00 | 15 | 49.676480 | -109.574180 | 602867 | 5503637 | 1 | 1625.85 | Local |
| F5 | 02/18/2012 0:00 | 18 | 49.683340 | -109.554280 | 604288 | 5504427 | 1 | 1950.05 | Local |
| F5 | 02/18/2012 0:00 | 21 | 49.685120 | -109.527390 | 606223 | 5504663 | 1 | 728.97 | Local |
| F5 | 02/18/2012 0:00 | 21 | 49.685120 | -109.527390 | 606223 | 5504663 | 1 | 728.97 | Local |
| F5 | 02/19/2012 0:00 | 0 | 49.680800 | -109.519790 | 606781 | 5504193 | 1 | 963.99 | Local |
| F5 | 02/19/2012 0:00 | 3 | 49.673670 | -109.512190 | 607345 | 5503412 | 1 | 1200.14 | Local |
| F5 | 02/19/2012 0:00 | 6 | 49.671660 | -109.495850 | 608528 | 5503212 | 1 | 724.60 | Local |
| F5 | 02/19/2012 0:00 | 9 | 49.678170 | -109.495400 | 608546 | 5503936 | 1 | 63.18 | Local |
| F5 | 02/19/2012 0:00 | 12 | 49.678720 | -109.495620 | 608529 | 5503997 | 1 | 316.11 | Local |
| F5 | 02/19/2012 0:00 | 15 | 49.680430 | -109.499120 | 608273 | 5504182 | 1 | 733.63 | Local |
| F5 | 02/19/2012 0:00 | 18 | 49.682930 | -109.508530 | 607589 | 5504446 | 1 | 299.09 | Local |

| | | | | | | | | | |
|---|---|---|---|---|---|---|---|---|---|
| F5 | 02/19/2012 0:00 | 21 | 49.684640 | -109.511730 | 607354 | 5504632 | 1 | 5.73 | Local |
| F5 | 02/19/2012 0:00 | 0 | 49.680800 | -109.519790 | 606781 | 5504193 | 1 | 963.99 | Local |
| F5 | 02/19/2012 0:00 | 3 | 49.673670 | -109.512190 | 607345 | 5503412 | 1 | 1200.14 | Local |
| F5 | 02/19/2012 0:00 | 6 | 49.671660 | -109.495850 | 608528 | 5503212 | 1 | 724.60 | Local |
| F5 | 02/19/2012 0:00 | 9 | 49.678170 | -109.495400 | 608546 | 5503936 | 1 | 63.18 | Local |
| F5 | 02/19/2012 0:00 | 12 | 49.678720 | -109.495620 | 608529 | 5503997 | 1 | 316.11 | Local |
| F5 | 02/19/2012 0:00 | 15 | 49.680430 | -109.499120 | 608273 | 5504182 | 1 | 733.63 | Local |
| F5 | 02/19/2012 0:00 | 18 | 49.682930 | -109.508530 | 607589 | 5504446 | 1 | 299.09 | Local |
| F5 | 02/19/2012 0:00 | 21 | 49.684640 | -109.511730 | 607354 | 5504632 | 1 | 5.73 | Local |
| F5 | 02/20/2012 0:00 | 0 | 49.684600 | -109.511780 | 607351 | 5504627 | 1 | 1612.95 | Local |
| F5 | 02/20/2012 0:00 | 3 | 49.671260 | -109.503000 | 608013 | 5503157 | 1 | 1411.57 | Local |
| F5 | 02/20/2012 0:00 | 6 | 49.660110 | -109.493650 | 608713 | 5501931 | 1 | 1695.44 | Local |
| F5 | 02/20/2012 0:00 | 9 | 49.675350 | -109.492910 | 608732 | 5503626 | 1 | 201.64 | Local |
| F5 | 02/20/2012 0:00 | 12 | 49.677060 | -109.493840 | 608661 | 5503815 | 1 | 82.62 | Local |
| F5 | 02/20/2012 0:00 | 15 | 49.676710 | -109.492830 | 608735 | 5503777 | 1 | 546.54 | Local |
| F5 | 02/20/2012 0:00 | 18 | 49.680590 | -109.497480 | 608391 | 5504202 | 1 | 466.25 | Local |
| F5 | 02/20/2012 0:00 | 21 | 49.676710 | -109.499930 | 608223 | 5503767 | 1 | 25.86 | Local |
| F5 | 02/20/2012 0:00 | 0 | 49.684600 | -109.511780 | 607351 | 5504627 | 1 | 1612.95 | Local |
| F5 | 02/20/2012 0:00 | 3 | 49.671260 | -109.503000 | 608013 | 5503157 | 1 | 1411.57 | Local |
| F5 | 02/20/2012 0:00 | 6 | 49.660110 | -109.493650 | 608713 | 5501931 | 1 | 1695.44 | Local |
| F5 | 02/20/2012 0:00 | 9 | 49.675350 | -109.492910 | 608732 | 5503626 | 1 | 201.64 | Local |
| F5 | 02/20/2012 0:00 | 12 | 49.677060 | -109.493840 | 608661 | 5503815 | 1 | 82.62 | Local |
| F5 | 02/20/2012 0:00 | 15 | 49.676710 | -109.492830 | 608735 | 5503777 | 1 | 546.54 | Local |
| F5 | 02/20/2012 0:00 | 18 | 49.680590 | -109.497480 | 608391 | 5504202 | 1 | 466.25 | Local |
| F5 | 02/20/2012 0:00 | 21 | 49.676710 | -109.499930 | 608223 | 5503767 | 1 | 25.86 | Local |
| F5 | 02/21/2012 0:00 | 0 | 49.676760 | -109.500280 | 608197 | 5503772 | 1 | 676.28 | Local |
| F5 | 02/21/2012 0:00 | 3 | 49.671070 | -109.496970 | 608449 | 5503144 | 1 | 1379.31 | Local |
| F5 | 02/21/2012 0:00 | 6 | 49.678600 | -109.512160 | 607336 | 5503960 | 1 | 1023.10 | Local |
| F5 | 02/21/2012 0:00 | 9 | 49.681860 | -109.498900 | 608286 | 5504341 | 1 | 19.65 | Local |
| F5 | 02/21/2012 0:00 | 12 | 49.681980 | -109.499100 | 608271 | 5504354 | 1 | 50.27 | Local |
| F5 | 02/21/2012 0:00 | 15 | 49.682240 | -109.498530 | 608312 | 5504384 | 1 | 7.27 | Local |
| F5 | 02/21/2012 0:00 | 18 | 49.682180 | -109.498490 | 608315 | 5504377 | 1 | 33.37 | Local |
| F5 | 02/21/2012 0:00 | 21 | 49.682360 | -109.498120 | 608341 | 5504398 | 1 | 61.80 | Local |
| F5 | 02/21/2012 0:00 | 0 | 49.676760 | -109.500280 | 608197 | 5503772 | 1 | 676.28 | Local |
| F5 | 02/21/2012 0:00 | 3 | 49.671070 | -109.496970 | 608449 | 5503144 | 1 | 1379.31 | Local |
| F5 | 02/21/2012 0:00 | 6 | 49.678600 | -109.512160 | 607336 | 5503960 | 1 | 1023.10 | Local |
| F5 | 02/21/2012 0:00 | 9 | 49.681860 | -109.498900 | 608286 | 5504341 | 1 | 19.65 | Local |
| F5 | 02/21/2012 0:00 | 12 | 49.681980 | -109.499100 | 608271 | 5504354 | 1 | 50.27 | Local |
| F5 | 02/21/2012 0:00 | 15 | 49.682240 | -109.498530 | 608312 | 5504384 | 1 | 7.27 | Local |
| F5 | 02/21/2012 0:00 | 18 | 49.682180 | -109.498490 | 608315 | 5504377 | 1 | 33.37 | Local |
| F5 | 02/21/2012 0:00 | 21 | 49.682360 | -109.498120 | 608341 | 5504398 | 1 | 61.80 | Local |
| F5 | 02/22/2012 0:00 | 0 | 49.682640 | -109.497380 | 608394 | 5504430 | 1 | 72.65 | Local |
| F5 | 02/22/2012 0:00 | 3 | 49.682270 | -109.498210 | 608335 | 5504388 | 1 | 77.30 | Local |
| F5 | 02/22/2012 0:00 | 6 | 49.681780 | -109.497450 | 608390 | 5504334 | 1 | 316.01 | Local |
| F5 | 02/22/2012 0:00 | 9 | 49.684580 | -109.498200 | 608330 | 5504644 | 1 | 816.63 | Local |
| F5 | 02/22/2012 0:00 | 12 | 49.691400 | -109.502400 | 608012 | 5505397 | 1 | 288.43 | Local |
| F5 | 02/22/2012 0:00 | 15 | 49.693990 | -109.502180 | 608022 | 5505685 | 1 | 218.80 | Local |
| F5 | 02/22/2012 0:00 | 18 | 49.695850 | -109.501190 | 608089 | 5505893 | 1 | 697.74 | Local |
| F5 | 02/22/2012 0:00 | 21 | 49.689610 | -109.500170 | 608177 | 5505201 | 1 | 906.19 | Local |
| F5 | 02/22/2012 0:00 | 0 | 49.682640 | -109.497380 | 608394 | 5504430 | 1 | 72.65 | Local |
| F5 | 02/22/2012 0:00 | 3 | 49.682270 | -109.498210 | 608335 | 5504388 | 1 | 77.30 | Local |
| F5 | 02/22/2012 0:00 | 6 | 49.681780 | -109.497450 | 608390 | 5504334 | 1 | 316.01 | Local |
| F5 | 02/22/2012 0:00 | 9 | 49.684580 | -109.498200 | 608330 | 5504644 | 1 | 816.63 | Local |
| F5 | 02/22/2012 0:00 | 12 | 49.691400 | -109.502400 | 608012 | 5505397 | 1 | 288.43 | Local |
| F5 | 02/22/2012 0:00 | 15 | 49.693990 | -109.502180 | 608022 | 5505685 | 1 | 218.80 | Local |

| | | | | | | | | | | |
|---|---|---|---|---|---|---|---|---|---|---|
| F5 | 02/22/2012 0:00 | 18 | 49.695850 | -109.501190 | 608089 | 5505893 | 1 | 697.74 | Local |
| F5 | 02/22/2012 0:00 | 21 | 49.689610 | -109.500170 | 608177 | 5505201 | 1 | 906.19 | Local |
| F5 | 02/23/2012 0:00 | 0 | 49.694950 | -109.509660 | 607481 | 5505781 | 1 | 24.92 | Local |
| F5 | 02/23/2012 0:00 | 3 | 49.694990 | -109.510000 | 607456 | 5505785 | 1 | 752.93 | Local |
| F5 | 02/23/2012 0:00 | 6 | 49.691840 | -109.500760 | 608129 | 5505448 | 1 | 821.51 | Local |
| F5 | 02/23/2012 0:00 | 9 | 49.698240 | -109.506450 | 607705 | 5506151 | 1 | 596.05 | Local |
| F5 | 02/23/2012 0:00 | 12 | 49.695530 | -109.499320 | 608225 | 5505860 | 1 | 694.27 | Local |
| F5 | 02/23/2012 0:00 | 15 | 49.693060 | -109.490480 | 608868 | 5505598 | 1 | 310.46 | Local |
| F5 | 02/23/2012 0:00 | 18 | 49.690300 | -109.489830 | 608921 | 5505292 | 1 | 826.59 | Local |
| F5 | 02/23/2012 0:00 | 21 | 49.692220 | -109.500900 | 608118 | 5505490 | 1 | 366.62 | Local |
| F5 | 02/23/2012 0:00 | 0 | 49.694950 | -109.509660 | 607481 | 5505781 | 1 | 24.92 | Local |
| F5 | 02/23/2012 0:00 | 3 | 49.694990 | -109.510000 | 607456 | 5505785 | 1 | 752.93 | Local |
| F5 | 02/23/2012 0:00 | 6 | 49.691840 | -109.500760 | 608129 | 5505448 | 1 | 821.51 | Local |
| F5 | 02/23/2012 0:00 | 9 | 49.698240 | -109.506450 | 607705 | 5506151 | 1 | 596.05 | Local |
| F5 | 02/23/2012 0:00 | 12 | 49.695530 | -109.499320 | 608225 | 5505860 | 1 | 694.27 | Local |
| F5 | 02/23/2012 0:00 | 15 | 49.693060 | -109.490480 | 608868 | 5505598 | 1 | 310.46 | Local |
| F5 | 02/23/2012 0:00 | 18 | 49.690300 | -109.489830 | 608921 | 5505292 | 1 | 826.59 | Local |
| F5 | 02/23/2012 0:00 | 21 | 49.692220 | -109.500900 | 608118 | 5505490 | 1 | 366.62 | Local |
| F5 | 02/24/2012 0:00 | 0 | 49.695170 | -109.498630 | 608276 | 5505821 | 1 | 407.64 | Local |
| F5 | 02/24/2012 0:00 | 3 | 49.692180 | -109.495360 | 608518 | 5505493 | 1 | 378.14 | Local |
| F5 | 02/24/2012 0:00 | 6 | 49.695410 | -109.493720 | 608629 | 5505855 | 1 | 480.13 | Local |
| F5 | 02/24/2012 0:00 | 9 | 49.693180 | -109.499420 | 608223 | 5505599 | 1 | 382.78 | Local |
| F5 | 02/24/2012 0:00 | 12 | 49.692350 | -109.494270 | 608596 | 5505514 | 1 | 235.07 | Local |
| F5 | 02/24/2012 0:00 | 15 | 49.693950 | -109.492140 | 608746 | 5505695 | 1 | 1128.32 | Local |
| F5 | 02/24/2012 0:00 | 18 | 49.687030 | -109.503580 | 607937 | 5504909 | 1 | 68.75 | Local |
| F5 | 02/24/2012 0:00 | 21 | 49.686430 | -109.503350 | 607955 | 5504843 | 1 | 609.76 | Local |
| F5 | 02/24/2012 0:00 | 0 | 49.695170 | -109.498630 | 608276 | 5505821 | 1 | 407.64 | Local |
| F5 | 02/24/2012 0:00 | 3 | 49.692180 | -109.495360 | 608518 | 5505493 | 1 | 378.14 | Local |
| F5 | 02/24/2012 0:00 | 6 | 49.695410 | -109.493720 | 608629 | 5505855 | 1 | 480.13 | Local |
| F5 | 02/24/2012 0:00 | 9 | 49.693180 | -109.499420 | 608223 | 5505599 | 1 | 382.78 | Local |
| F5 | 02/24/2012 0:00 | 12 | 49.692350 | -109.494270 | 608596 | 5505514 | 1 | 235.07 | Local |
| F5 | 02/24/2012 0:00 | 15 | 49.693950 | -109.492140 | 608746 | 5505695 | 1 | 1128.32 | Local |
| F5 | 02/24/2012 0:00 | 18 | 49.687030 | -109.503580 | 607937 | 5504909 | 1 | 68.75 | Local |
| F5 | 02/24/2012 0:00 | 21 | 49.686430 | -109.503350 | 607955 | 5504843 | 1 | 609.76 | Local |
| F5 | 02/25/2012 0:00 | 0 | 49.681110 | -109.505400 | 607818 | 5504248 | 1 | 135.13 | Local |
| F5 | 02/25/2012 0:00 | 3 | 49.679920 | -109.505020 | 607849 | 5504117 | 1 | 3.10 | Local |
| F5 | 02/25/2012 0:00 | 6 | 49.679900 | -109.505050 | 607846 | 5504114 | 1 | 3.34 | Local |
| F5 | 02/25/2012 0:00 | 9 | 49.679930 | -109.505050 | 607846 | 5504118 | 1 | 729.95 | Local |
| F5 | 02/25/2012 0:00 | 12 | 49.684820 | -109.498300 | 608322 | 5504671 | 1 | 33.35 | Local |
| F5 | 02/25/2012 0:00 | 15 | 49.684790 | -109.497840 | 608356 | 5504668 | 1 | 730.84 | Local |
| F5 | 02/25/2012 0:00 | 18 | 49.678370 | -109.500010 | 608213 | 5503951 | 0 | 8.61 | Local |
| F5 | 02/25/2012 0:00 | 0 | 49.681110 | -109.505400 | 607818 | 5504248 | 1 | 135.13 | Local |
| F5 | 02/25/2012 0:00 | 3 | 49.679920 | -109.505020 | 607849 | 5504117 | 1 | 3.10 | Local |
| F5 | 02/25/2012 0:00 | 6 | 49.679900 | -109.505050 | 607846 | 5504114 | 1 | 3.34 | Local |
| F5 | 02/25/2012 0:00 | 9 | 49.679930 | -109.505050 | 607846 | 5504118 | 1 | 729.95 | Local |
| F5 | 02/25/2012 0:00 | 12 | 49.684820 | -109.498300 | 608322 | 5504671 | 1 | 33.35 | Local |
| F5 | 02/25/2012 0:00 | 15 | 49.684790 | -109.497840 | 608356 | 5504668 | 1 | 730.84 | Local |
| F5 | 02/25/2012 0:00 | 18 | 49.678370 | -109.500010 | 608213 | 5503951 | 0 | 8.61 | Local |
| F5 | 02/26/2012 0:00 | 0 | 49.678400 | -109.500120 | 608205 | 5503955 | 1 | 531.57 | Local |
| F5 | 02/26/2012 0:00 | 3 | 49.673790 | -109.502070 | 608075 | 5503439 | 1 | 935.18 | Local |
| F5 | 02/26/2012 0:00 | 6 | 49.682170 | -109.500970 | 608136 | 5504373 | 0 | 27.25 | Local |
| F5 | 02/26/2012 0:00 | 12 | 49.682030 | -109.501280 | 608114 | 5504357 | 0 | 1545.09 | Local |
| F5 | 02/26/2012 0:00 | 21 | 49.678100 | -109.480740 | 609604 | 5503949 | 0 | 1205.96 | Local |
| F5 | 02/26/2012 0:00 | 0 | 49.678400 | -109.500120 | 608205 | 5503955 | 1 | 531.57 | Local |
| F5 | 02/26/2012 0:00 | 3 | 49.673790 | -109.502070 | 608075 | 5503439 | 1 | 935.18 | Local |

| | | | | | | | | | |
|---|---|---|---|---|---|---|---|---|---|
| F5 | 02/26/2012 0:00 | 6 | 49.682170 | -109.500970 | 608136 | 5504373 | 0 | 27.25 | Local |
| F5 | 02/26/2012 0:00 | 12 | 49.682030 | -109.501280 | 608114 | 5504357 | 0 | 1545.09 | Local |
| F5 | 02/26/2012 0:00 | 21 | 49.678100 | -109.480740 | 609604 | 5503949 | 0 | 1205.96 | Local |
| F5 | 02/27/2012 0:00 | 9 | 49.682300 | -109.465330 | 610706 | 5504439 | 1 | 2772.83 | Local |
| F5 | 02/27/2012 0:00 | 12 | 49.697510 | -109.495790 | 608475 | 5506085 | 1 | 649.08 | Local |
| F5 | 02/27/2012 0:00 | 15 | 49.701400 | -109.502500 | 607983 | 5506508 | 1 | 42.90 | Local |
| F5 | 02/27/2012 0:00 | 18 | 49.701530 | -109.501940 | 608023 | 5506523 | 1 | 0.72 | Local |
| F5 | 02/27/2012 0:00 | 21 | 49.701530 | -109.501930 | 608023 | 5506523 | 1 | 1.82 | Local |
| F5 | 02/27/2012 0:00 | 9 | 49.682300 | -109.465330 | 610706 | 5504439 | 1 | 2772.83 | Local |
| F5 | 02/27/2012 0:00 | 12 | 49.697510 | -109.495790 | 608475 | 5506085 | 1 | 649.08 | Local |
| F5 | 02/27/2012 0:00 | 15 | 49.701400 | -109.502500 | 607983 | 5506508 | 1 | 42.90 | Local |
| F5 | 02/27/2012 0:00 | 18 | 49.701530 | -109.501940 | 608023 | 5506523 | 1 | 0.72 | Local |
| F5 | 02/27/2012 0:00 | 21 | 49.701530 | -109.501930 | 608023 | 5506523 | 1 | 1.82 | Local |
| F5 | 02/28/2012 0:00 | 0 | 49.701520 | -109.501950 | 608022 | 5506522 | 1 | 15.83 | Local |
| F5 | 02/28/2012 0:00 | 3 | 49.701430 | -109.502120 | 608010 | 5506512 | 1 | 16.95 | Local |
| F5 | 02/28/2012 0:00 | 6 | 49.701510 | -109.501920 | 608024 | 5506521 | 1 | 33.28 | Local |
| F5 | 02/28/2012 0:00 | 9 | 49.701350 | -109.502310 | 607996 | 5506503 | 1 | 3.34 | Local |
| F5 | 02/28/2012 0:00 | 12 | 49.701380 | -109.502310 | 607996 | 5506506 | 1 | 3.34 | Local |
| F5 | 02/28/2012 0:00 | 15 | 49.701410 | -109.502310 | 607996 | 5506510 | 1 | 32.67 | Local |
| F5 | 02/28/2012 0:00 | 18 | 49.701520 | -109.501890 | 608026 | 5506522 | 1 | 2590.12 | Local |
| F5 | 02/28/2012 0:00 | 0 | 49.701520 | -109.501950 | 608022 | 5506522 | 1 | 15.83 | Local |
| F5 | 02/28/2012 0:00 | 3 | 49.701430 | -109.502120 | 608010 | 5506512 | 1 | 16.95 | Local |
| F5 | 02/28/2012 0:00 | 6 | 49.701510 | -109.501920 | 608024 | 5506521 | 1 | 33.28 | Local |
| F5 | 02/28/2012 0:00 | 9 | 49.701350 | -109.502310 | 607996 | 5506503 | 1 | 3.34 | Local |
| F5 | 02/28/2012 0:00 | 12 | 49.701380 | -109.502310 | 607996 | 5506506 | 1 | 3.34 | Local |
| F5 | 02/28/2012 0:00 | 15 | 49.701410 | -109.502310 | 607996 | 5506510 | 1 | 32.67 | Local |
| F5 | 02/28/2012 0:00 | 18 | 49.701520 | -109.501890 | 608026 | 5506522 | 1 | 38.81 | Local |
| F5 | 02/28/2012 0:00 | 21 | 49.701430 | -109.502410 | 607989 | 5506512 | 1 | 37.74 | Local |
| F5 | 02/29/2012 0:00 | 0 | 49.701530 | -109.501910 | 608025 | 5506524 | 1 | 1359.36 | Local |
| F5 | 02/29/2012 0:00 | 3 | 49.700490 | -109.520690 | 606673 | 5506381 | 1 | 1376.19 | Local |
| F5 | 02/29/2012 0:00 | 6 | 49.688240 | -109.517970 | 606896 | 5505023 | 1 | 727.29 | Local |
| F5 | 02/29/2012 0:00 | 9 | 49.682750 | -109.512490 | 607303 | 5504421 | 1 | 200.83 | Local |
| F5 | 02/29/2012 0:00 | 12 | 49.682470 | -109.509740 | 607502 | 5504393 | 1 | 509.29 | Local |
| F5 | 02/29/2012 0:00 | 15 | 49.681130 | -109.502990 | 607992 | 5504254 | 0 | 373.48 | Local |
| F5 | 02/29/2012 0:00 | 21 | 49.678370 | -109.500040 | 608211 | 5503951 | 0 | 2071.97 | Local |
| F5 | 03/01/2012 0:00 | 6 | 49.664120 | -109.481540 | 609578 | 5502394 | 1 | 1135.01 | Local |
| F5 | 03/01/2012 0:00 | 9 | 49.672180 | -109.491190 | 608863 | 5503276 | 1 | 1.33 | Local |
| F5 | 03/01/2012 0:00 | 12 | 49.672170 | -109.491180 | 608864 | 5503275 | 1 | 2.43 | Local |
| F5 | 03/01/2012 0:00 | 15 | 49.672180 | -109.491210 | 608862 | 5503276 | 1 | 371.49 | Local |
| F5 | 03/01/2012 0:00 | 18 | 49.669050 | -109.493010 | 608739 | 5502925 | 1 | 1079.44 | Local |
| F5 | 03/01/2012 0:00 | 21 | 49.659480 | -109.490500 | 608942 | 5501865 | 1 | 1093.84 | Local |
| F5 | 03/02/2012 0:00 | 0 | 49.649990 | -109.494490 | 608675 | 5500804 | 1 | 490.03 | Local |
| F5 | 03/02/2012 0:00 | 3 | 49.645810 | -109.492340 | 608839 | 5500343 | 1 | 13.85 | Local |
| F5 | 03/02/2012 0:00 | 6 | 49.645920 | -109.492430 | 608833 | 5500355 | 0 | 9.16 | Local |
| F5 | 03/02/2012 0:00 | 12 | 49.645840 | -109.492400 | 608835 | 5500346 | 1 | 222.38 | Local |
| F5 | 03/02/2012 0:00 | 15 | 49.646320 | -109.495390 | 608618 | 5500395 | 1 | 42.61 | Local |
| F5 | 03/02/2012 0:00 | 18 | 49.646650 | -109.495690 | 608596 | 5500431 | 1 | 64.60 | Local |
| F5 | 03/02/2012 0:00 | 21 | 49.646110 | -109.495360 | 608621 | 5500372 | 1 | 18.50 | Local |
| F5 | 03/03/2012 0:00 | 0 | 49.646240 | -109.495200 | 608632 | 5500386 | 1 | 2.22 | Local |
| F5 | 03/03/2012 0:00 | 3 | 49.646220 | -109.495200 | 608632 | 5500384 | 1 | 2.34 | Local |
| F5 | 03/03/2012 0:00 | 6 | 49.646200 | -109.495190 | 608633 | 5500382 | 1 | 6.27 | Local |
| F5 | 03/03/2012 0:00 | 9 | 49.646250 | -109.495230 | 608630 | 5500388 | 1 | 33.36 | Local |
| F5 | 03/03/2012 0:00 | 12 | 49.646460 | -109.495560 | 608605 | 5500410 | 1 | 44.52 | Local |
| F5 | 03/03/2012 0:00 | 15 | 49.646100 | -109.495290 | 608626 | 5500371 | 1 | 18.15 | Local |
| F5 | 03/03/2012 0:00 | 18 | 49.646260 | -109.495240 | 608629 | 5500389 | 1 | 103.48 | Local |

| | | | | | | | | | |
|---|---|---|---|---|---|---|---|---|---|
| F5 | 03/03/2012 0:00 | 21 | 49.645350 | -109.494940 | 608653 | 5500288 | 1 | 45.06 | Local |
| F5 | 03/04/2012 0:00 | 0 | 49.644950 | -109.495040 | 608646 | 5500243 | 0 | 45.78 | Local |
| F5 | 03/04/2012 0:00 | 6 | 49.645350 | -109.494890 | 608656 | 5500288 | 1 | 2.17 | Local |
| F5 | 03/04/2012 0:00 | 9 | 49.645350 | -109.494920 | 608654 | 5500288 | 1 | 3.41 | Local |
| F5 | 03/04/2012 0:00 | 12 | 49.645320 | -109.494910 | 608655 | 5500285 | 0 | 98.22 | Local |
| F5 | 03/04/2012 0:00 | 18 | 49.646170 | -109.495280 | 608626 | 5500379 | 0 | 14.14 | Local |
| F5 | 03/05/2012 0:00 | 0 | 49.646220 | -109.495100 | 608639 | 5500384 | 1 | 95.96 | Local |
| F5 | 03/05/2012 0:00 | 3 | 49.645370 | -109.494870 | 608658 | 5500290 | 1 | 88.87 | Local |
| F5 | 03/05/2012 0:00 | 6 | 49.646140 | -109.495200 | 608632 | 5500375 | 1 | 83.94 | Local |
| F5 | 03/05/2012 0:00 | 9 | 49.645400 | -109.494970 | 608650 | 5500293 | 1 | 3.10 | Local |
| F5 | 03/05/2012 0:00 | 12 | 49.645380 | -109.494940 | 608653 | 5500291 | 1 | 88.00 | Local |
| F5 | 03/05/2012 0:00 | 15 | 49.646140 | -109.495280 | 608626 | 5500375 | 1 | 8.55 | Local |
| F5 | 03/05/2012 0:00 | 18 | 49.646190 | -109.495190 | 608633 | 5500381 | 1 | 128.63 | Local |
| F5 | 03/05/2012 0:00 | 21 | 49.645270 | -109.494110 | 608713 | 5500280 | 1 | 7.92 | Local |
| F5 | 03/06/2012 0:00 | 0 | 49.645200 | -109.494090 | 608714 | 5500272 | 1 | 101.43 | Local |
| F5 | 03/06/2012 0:00 | 3 | 49.646080 | -109.494460 | 608686 | 5500370 | 1 | 57.05 | Local |
| F5 | 03/06/2012 0:00 | 6 | 49.646090 | -109.495250 | 608629 | 5500370 | 1 | 85.45 | Local |
| F5 | 03/06/2012 0:00 | 9 | 49.645360 | -109.494880 | 608657 | 5500289 | 1 | 93.17 | Local |
| F5 | 03/06/2012 0:00 | 12 | 49.646170 | -109.495210 | 608631 | 5500379 | 1 | 9.69 | Local |
| F5 | 03/06/2012 0:00 | 15 | 49.646120 | -109.495320 | 608623 | 5500373 | 1 | 10.62 | Local |
| F5 | 03/06/2012 0:00 | 18 | 49.646190 | -109.495220 | 608631 | 5500381 | 1 | 243.30 | Local |
| F5 | 03/06/2012 0:00 | 21 | 49.646600 | -109.498530 | 608391 | 5500422 | 1 | 498.15 | Local |
| F5 | 03/07/2012 0:00 | 0 | 49.651080 | -109.498540 | 608380 | 5500920 | 1 | 766.89 | Local |
| F5 | 03/07/2012 0:00 | 3 | 49.657740 | -109.495780 | 608564 | 5501664 | 1 | 1204.32 | Local |
| F5 | 03/07/2012 0:00 | 6 | 49.660250 | -109.479550 | 609730 | 5501967 | 1 | 870.28 | Local |
| F5 | 03/07/2012 0:00 | 9 | 49.656450 | -109.469010 | 610499 | 5501560 | 1 | 19.06 | Local |
| F5 | 03/07/2012 0:00 | 12 | 49.656480 | -109.468750 | 610518 | 5501563 | 1 | 22.44 | Local |
| F5 | 03/07/2012 0:00 | 15 | 49.656360 | -109.469000 | 610500 | 5501550 | 1 | 805.09 | Local |
| F5 | 03/07/2012 0:00 | 18 | 49.652230 | -109.459840 | 611171 | 5501104 | 1 | 842.54 | Local |
| F5 | 03/07/2012 0:00 | 21 | 49.648260 | -109.449900 | 611897 | 5500677 | 1 | 2215.28 | Local |
| F5 | 03/08/2012 0:00 | 0 | 49.650250 | -109.480430 | 609689 | 5500854 | 1 | 1193.14 | Local |
| F5 | 03/08/2012 0:00 | 3 | 49.658350 | -109.491270 | 608889 | 5501738 | 1 | 1289.45 | Local |
| F5 | 03/08/2012 0:00 | 6 | 49.664390 | -109.506520 | 607775 | 5502388 | 1 | 1282.54 | Local |
| F5 | 03/08/2012 0:00 | 9 | 49.675120 | -109.513040 | 607281 | 5503571 | 1 | 75.86 | Local |
| F5 | 03/08/2012 0:00 | 12 | 49.675220 | -109.512000 | 607355 | 5503584 | 1 | 4.87 | Local |
| F5 | 03/08/2012 0:00 | 15 | 49.675200 | -109.511940 | 607360 | 5503582 | 1 | 16.49 | Local |
| F5 | 03/08/2012 0:00 | 18 | 49.675240 | -109.512160 | 607344 | 5503586 | 1 | 2.43 | Local |
| F5 | 03/08/2012 0:00 | 21 | 49.675230 | -109.512190 | 607342 | 5503585 | 1 | 710.85 | Local |
| F5 | 03/09/2012 0:00 | 0 | 49.679250 | -109.519850 | 606780 | 5504021 | 1 | 862.18 | Local |
| F5 | 03/09/2012 0:00 | 3 | 49.675190 | -109.530030 | 606055 | 5503555 | 1 | 1162.18 | Local |
| F5 | 03/09/2012 0:00 | 6 | 49.664760 | -109.528990 | 606152 | 5502397 | 1 | 1576.81 | Local |
| F5 | 03/09/2012 0:00 | 9 | 49.678840 | -109.526390 | 606309 | 5503966 | 1 | 5.61 | Local |
| F5 | 03/09/2012 0:00 | 12 | 49.678890 | -109.526380 | 606310 | 5503972 | 1 | 6.71 | Local |
| F5 | 03/09/2012 0:00 | 15 | 49.678830 | -109.526390 | 606309 | 5503965 | 1 | 16.37 | Local |
| F5 | 03/09/2012 0:00 | 18 | 49.678970 | -109.526320 | 606314 | 5503981 | 1 | 9.35 | Local |
| F5 | 03/09/2012 0:00 | 21 | 49.678890 | -109.526360 | 606311 | 5503972 | 1 | 14.53 | Local |
| F5 | 03/10/2012 0:00 | 0 | 49.678760 | -109.526340 | 606313 | 5503957 | 1 | 16.40 | Local |
| F5 | 03/10/2012 0:00 | 3 | 49.678830 | -109.526140 | 606327 | 5503965 | 1 | 11.64 | Local |
| F5 | 03/10/2012 0:00 | 6 | 49.678900 | -109.526260 | 606319 | 5503973 | 1 | 66.98 | Local |
| F5 | 03/10/2012 0:00 | 9 | 49.678410 | -109.526800 | 606281 | 5503918 | 1 | 11.49 | Local |
| F5 | 03/10/2012 0:00 | 12 | 49.678310 | -109.526840 | 606278 | 5503907 | 1 | 14.03 | Local |
| F5 | 03/10/2012 0:00 | 15 | 49.678430 | -109.526900 | 606273 | 5503920 | 1 | 72.42 | Local |
| F5 | 03/10/2012 0:00 | 18 | 49.678870 | -109.526160 | 606326 | 5503970 | 0 | 4.95 | Local |
| F5 | 03/11/2012 0:00 | 0 | 49.678830 | -109.526130 | 606328 | 5503965 | 1 | 24.14 | Local |
| F5 | 03/11/2012 0:00 | 3 | 49.679030 | -109.526260 | 606318 | 5503987 | 1 | 178.75 | Local |

| | | | | | | | | | |
|---|---|---|---|---|---|---|---|---|---|
| F5 | 03/11/2012 0:00 | 6 | 49.677540 | -109.527190 | 606254 | 5503820 | 1 | 1330.72 | Local |
| F5 | 03/11/2012 0:00 | 9 | 49.685130 | -109.541450 | 605209 | 5504644 | 1 | 736.86 | Local |
| F5 | 03/11/2012 0:00 | 12 | 49.691050 | -109.536860 | 605528 | 5505309 | 0 | 174.19 | Local |
| F5 | 03/11/2012 0:00 | 18 | 49.691710 | -109.539050 | 605368 | 5505379 | 1 | 952.61 | Local |
| F5 | 03/11/2012 0:00 | 21 | 49.696490 | -109.550010 | 604567 | 5505895 | 1 | 1083.63 | Local |
| F5 | 03/12/2012 0:00 | 0 | 49.705120 | -109.556990 | 604046 | 5506845 | 1 | 2409.77 | Local |
| F5 | 03/12/2012 0:00 | 3 | 49.683660 | -109.552330 | 604428 | 5504466 | 1 | 2393.26 | Local |
| F5 | 03/12/2012 0:00 | 6 | 49.670150 | -109.526510 | 606320 | 5503000 | 1 | 1455.83 | Local |
| F5 | 03/12/2012 0:00 | 9 | 49.681580 | -109.516670 | 607004 | 5504284 | 1 | 23.11 | Local |
| F5 | 03/12/2012 0:00 | 12 | 49.681760 | -109.516830 | 606993 | 5504304 | 1 | 24.67 | Local |
| F5 | 03/12/2012 0:00 | 15 | 49.681940 | -109.517030 | 606978 | 5504324 | 1 | 9.64 | Local |
| F5 | 03/12/2012 0:00 | 18 | 49.681960 | -109.516900 | 606987 | 5504326 | 1 | 1010.80 | Local |
| F5 | 03/12/2012 0:00 | 21 | 49.672940 | -109.515160 | 607132 | 5503326 | 1 | 1065.32 | Local |
| F5 | 03/13/2012 0:00 | 0 | 49.663590 | -109.511940 | 607385 | 5502291 | 1 | 1481.52 | Local |
| F5 | 03/13/2012 0:00 | 3 | 49.650770 | -109.506350 | 607817 | 5500874 | 1 | 2015.85 | Local |
| F5 | 03/13/2012 0:00 | 6 | 49.668790 | -109.509410 | 607556 | 5502873 | 1 | 1139.04 | Local |
| F5 | 03/13/2012 0:00 | 9 | 49.677160 | -109.500310 | 608194 | 5503817 | 1 | 36.64 | Local |
| F5 | 03/13/2012 0:00 | 12 | 49.676860 | -109.500100 | 608210 | 5503783 | 1 | 40.57 | Local |
| F5 | 03/13/2012 0:00 | 15 | 49.676670 | -109.500580 | 608176 | 5503762 | 1 | 33.36 | Local |
| F5 | 03/13/2012 0:00 | 18 | 49.676970 | -109.500580 | 608175 | 5503795 | 0 | 1561.61 | Local |
| F5 | 03/14/2012 0:00 | 0 | 49.663240 | -109.496030 | 608534 | 5502275 | 1 | 1154.18 | Local |
| F5 | 03/14/2012 0:00 | 3 | 49.653590 | -109.501920 | 608130 | 5501194 | 1 | 710.41 | Local |
| F5 | 03/14/2012 0:00 | 6 | 49.647520 | -109.498850 | 608366 | 5500524 | 1 | 289.47 | Local |
| F5 | 03/14/2012 0:00 | 9 | 49.649290 | -109.495910 | 608574 | 5500725 | 1 | 6.06 | Local |
| F5 | 03/14/2012 0:00 | 12 | 49.649260 | -109.495840 | 608579 | 5500721 | 1 | 408.73 | Local |
| F5 | 03/14/2012 0:00 | 15 | 49.651280 | -109.491110 | 608916 | 5500953 | 1 | 312.66 | Local |
| F5 | 03/14/2012 0:00 | 18 | 49.653810 | -109.493000 | 608774 | 5501231 | 1 | 728.43 | Local |
| F5 | 03/14/2012 0:00 | 21 | 49.660240 | -109.491070 | 608899 | 5501949 | 1 | 2278.38 | Local |
| F5 | 03/15/2012 0:00 | 0 | 49.676440 | -109.471740 | 610257 | 5503778 | 1 | 2101.09 | Local |
| F5 | 03/15/2012 0:00 | 3 | 49.670480 | -109.499370 | 608277 | 5503075 | 0 | 1785.46 | Local |
| F5 | 03/15/2012 0:00 | 9 | 49.681890 | -109.516780 | 606996 | 5504319 | 1 | 51.19 | Local |
| F5 | 03/15/2012 0:00 | 12 | 49.682210 | -109.516270 | 607032 | 5504355 | 1 | 546.64 | Local |
| F5 | 03/15/2012 0:00 | 15 | 49.685350 | -109.510440 | 607446 | 5504713 | 1 | 65.61 | Local |
| F5 | 03/15/2012 0:00 | 18 | 49.684760 | -109.510450 | 607446 | 5504647 | 1 | 1424.86 | Local |
| F5 | 03/15/2012 0:00 | 21 | 49.693510 | -109.524880 | 606386 | 5505599 | 1 | 2664.58 | Local |
| F5 | 03/16/2012 0:00 | 0 | 49.717070 | -109.531630 | 605848 | 5508209 | 1 | 242.18 | Local |
| F5 | 03/16/2012 0:00 | 3 | 49.717230 | -109.534980 | 605606 | 5508222 | 1 | 3.10 | Local |
| F5 | 03/16/2012 0:00 | 6 | 49.717250 | -109.535010 | 605604 | 5508224 | 1 | 1414.32 | Local |
| F5 | 03/16/2012 0:00 | 9 | 49.705130 | -109.529060 | 606059 | 5506885 | 1 | 20.11 | Local |
| F5 | 03/16/2012 0:00 | 12 | 49.704970 | -109.528930 | 606069 | 5506867 | 1 | 4.68 | Local |
| F5 | 03/16/2012 0:00 | 15 | 49.705010 | -109.528950 | 606068 | 5506872 | 1 | 6.71 | Local |
| F5 | 03/16/2012 0:00 | 18 | 49.705070 | -109.528960 | 606067 | 5506879 | 1 | 2.22 | Local |
| F5 | 03/16/2012 0:00 | 21 | 49.705090 | -109.528960 | 606067 | 5506881 | 1 | 1058.38 | Local |
| F5 | 03/17/2012 0:00 | 0 | 49.713310 | -109.521560 | 606582 | 5507805 | 1 | 48.44 | Local |
| F5 | 03/17/2012 0:00 | 3 | 49.713420 | -109.522210 | 606535 | 5507816 | 1 | 7.82 | Local |
| F5 | 03/17/2012 0:00 | 6 | 49.713350 | -109.522200 | 606536 | 5507809 | 1 | 1023.23 | Local |
| F5 | 03/17/2012 0:00 | 9 | 49.704920 | -109.516510 | 606965 | 5506879 | 1 | 15.78 | Local |
| F5 | 03/17/2012 0:00 | 12 | 49.704880 | -109.516300 | 606980 | 5506875 | 1 | 7.58 | Local |
| F5 | 03/17/2012 0:00 | 15 | 49.704940 | -109.516250 | 606983 | 5506882 | 1 | 3.10 | Local |
| F5 | 03/17/2012 0:00 | 18 | 49.704920 | -109.516280 | 606981 | 5506880 | 1 | 981.42 | Local |
| F5 | 03/17/2012 0:00 | 21 | 49.713270 | -109.520690 | 606645 | 5507802 | 1 | 103.16 | Local |
| F5 | 03/18/2012 0:00 | 0 | 49.713490 | -109.522080 | 606544 | 5507824 | 1 | 172.50 | Local |
| F5 | 03/18/2012 0:00 | 3 | 49.715040 | -109.521980 | 606548 | 5507997 | 1 | 897.66 | Local |
| F5 | 03/18/2012 0:00 | 6 | 49.713010 | -109.534030 | 605684 | 5507754 | 1 | 928.17 | Local |
| F5 | 03/18/2012 0:00 | 9 | 49.705310 | -109.529060 | 606059 | 5506905 | 0 | 19.12 | Local |

| | | | | | | | | | |
|---|---|---|---|---|---|---|---|---|---|
| F5 | 03/18/2012 0:00 | 18 | 49.705140 | -109.529100 | 606057 | 5506886 | 1 | 920.91 | Local |
| F5 | 03/18/2012 0:00 | 21 | 49.712820 | -109.533880 | 605695 | 5507733 | 1 | 27.42 | Local |
| F5 | 03/19/2012 0:00 | 0 | 49.713000 | -109.534140 | 605676 | 5507753 | 0 | 937.51 | Local |
| F5 | 03/19/2012 0:00 | 6 | 49.705650 | -109.527770 | 606151 | 5506945 | 1 | 112.45 | Local |
| F5 | 03/19/2012 0:00 | 9 | 49.704960 | -109.528910 | 606071 | 5506866 | 1 | 18.90 | Local |
| F5 | 03/19/2012 0:00 | 12 | 49.705130 | -109.528910 | 606070 | 5506885 | 1 | 10.03 | Local |
| F5 | 03/19/2012 0:00 | 15 | 49.705040 | -109.528920 | 606070 | 5506875 | 1 | 17.16 | Local |
| F5 | 03/19/2012 0:00 | 18 | 49.705180 | -109.529020 | 606062 | 5506891 | 1 | 12.41 | Local |
| F5 | 03/19/2012 0:00 | 21 | 49.705100 | -109.528900 | 606071 | 5506882 | 1 | 4.41 | Local |
| F5 | 03/20/2012 0:00 | 0 | 49.705070 | -109.528940 | 606068 | 5506879 | 1 | 1214.04 | Local |
| F5 | 03/20/2012 0:00 | 3 | 49.696620 | -109.518280 | 606855 | 5505954 | 0 | 1922.01 | Local |
| F5 | 03/20/2012 0:00 | 18 | 49.680410 | -109.509030 | 607558 | 5504165 | 0 | 1227.56 | Local |
| F5 | 03/21/2012 0:00 | 9 | 49.680640 | -109.492020 | 608785 | 5504215 | 0 | 1213.15 | Local |
| F5 | 03/21/2012 0:00 | 21 | 49.669730 | -109.491930 | 608816 | 5503003 | 1 | 650.54 | Local |
| F5 | 03/22/2012 0:00 | 0 | 49.663960 | -109.490440 | 608936 | 5502363 | 0 | 513.58 | Local |
| F5 | 03/22/2012 0:00 | 6 | 49.664980 | -109.483500 | 609434 | 5502487 | 1 | 217.50 | Local |
| F5 | 03/22/2012 0:00 | 9 | 49.665980 | -109.486090 | 609245 | 5502594 | 1 | 19.14 | Local |
| F5 | 03/22/2012 0:00 | 12 | 49.665830 | -109.486220 | 609236 | 5502577 | 1 | 13.93 | Local |
| F5 | 03/22/2012 0:00 | 15 | 49.665900 | -109.486060 | 609248 | 5502585 | 1 | 99.67 | Local |
| F5 | 03/22/2012 0:00 | 18 | 49.665480 | -109.487280 | 609161 | 5502537 | 1 | 365.87 | Local |
| F5 | 03/22/2012 0:00 | 21 | 49.663190 | -109.483640 | 609428 | 5502288 | 0 | 2677.47 | Local |
| F5 | 03/23/2012 0:00 | 3 | 49.682420 | -109.461310 | 610996 | 5504458 | 0 | 1602.23 | Local |
| F5 | 03/23/2012 0:00 | 18 | 49.696480 | -109.466170 | 610613 | 5506014 | 0 | NA | Local |
| M2 | 04/12/2011 0:00 | 6 | 49.703607 | -109.413655 | 614384 | 5506885 | 1 | 1801.01 | Trans |
| M2 | 04/12/2011 0:00 | 9 | 49.707950 | -109.389595 | 616108 | 5507405 | 0 | 41.09 | Trans |
| M2 | 04/12/2011 0:00 | 15 | 49.708252 | -109.389268 | 616131 | 5507439 | 1 | 46.04 | Trans |
| M2 | 04/12/2011 0:00 | 18 | 49.707951 | -109.389705 | 616100 | 5507405 | 1 | 719.82 | Trans |
| M2 | 04/12/2011 0:00 | 21 | 49.707688 | -109.379732 | 616820 | 5507391 | 1 | 2180.44 | Trans |
| M2 | 04/13/2011 0:00 | 0 | 49.688524 | -109.373329 | 617328 | 5505270 | 1 | 1931.58 | Trans |
| M2 | 04/13/2011 0:00 | 3 | 49.676538 | -109.353951 | 618754 | 5503968 | 1 | 2565.38 | Trans |
| M2 | 04/13/2011 0:00 | 6 | 49.658606 | -109.331589 | 620412 | 5502010 | 1 | 101.38 | Trans |
| M2 | 04/13/2011 0:00 | 9 | 49.657729 | -109.331205 | 620442 | 5501914 | 1 | 7.86 | Trans |
| M2 | 04/13/2011 0:00 | 12 | 49.657663 | -109.331164 | 620445 | 5501906 | 1 | 43.12 | Trans |
| M2 | 04/13/2011 0:00 | 15 | 49.657874 | -109.331666 | 620408 | 5501929 | 1 | 181.15 | Trans |
| M2 | 04/13/2011 0:00 | 18 | 49.658173 | -109.329199 | 620586 | 5501966 | 1 | 147.75 | Trans |
| M2 | 04/13/2011 0:00 | 21 | 49.657235 | -109.330649 | 620483 | 5501860 | 1 | 1838.46 | Trans |
| M2 | 04/14/2011 0:00 | 0 | 49.642562 | -109.342383 | 619672 | 5500210 | 1 | 3942.97 | Trans |
| M2 | 04/14/2011 0:00 | 3 | 49.621276 | -109.386046 | 616571 | 5497775 | 0 | 7861.19 | Trans |
| M2 | 04/20/2011 0:00 | 21 | 49.672334 | -109.593996 | 601446 | 5503149 | 1 | 2237.72 | Trans |
| M2 | 04/21/2011 0:00 | 0 | 49.670327 | -109.624850 | 599224 | 5502885 | 1 | 1446.08 | Trans |
| M2 | 04/21/2011 0:00 | 3 | 49.682027 | -109.633601 | 598569 | 5504174 | 1 | 1670.33 | Trans |
| M2 | 04/21/2011 0:00 | 6 | 49.690648 | -109.652563 | 597184 | 5505108 | 1 | 186.40 | Trans |
| M2 | 04/21/2011 0:00 | 9 | 49.689028 | -109.651896 | 597235 | 5504929 | 1 | 105.02 | Trans |
| M2 | 04/21/2011 0:00 | 12 | 49.689646 | -109.652997 | 597154 | 5504996 | 1 | 15.63 | Trans |
| M2 | 04/21/2011 0:00 | 15 | 49.689785 | -109.653023 | 597152 | 5505011 | 1 | 114.44 | Trans |
| M2 | 04/21/2011 0:00 | 18 | 49.690778 | -109.652602 | 597181 | 5505122 | 1 | 842.56 | Trans |
| M2 | 04/21/2011 0:00 | 21 | 49.695291 | -109.661985 | 596495 | 5505612 | 1 | 60.81 | Trans |
| M2 | 04/22/2011 0:00 | 0 | 49.695717 | -109.662513 | 596456 | 5505659 | 1 | 2892.72 | Trans |
| M2 | 04/22/2011 0:00 | 3 | 49.698920 | -109.702316 | 593579 | 5505964 | 1 | 2678.23 | Trans |
| M2 | 04/22/2011 0:00 | 6 | 49.682548 | -109.729550 | 591646 | 5504111 | 1 | 434.35 | Trans |
| M2 | 04/22/2011 0:00 | 9 | 49.683470 | -109.723699 | 592067 | 5504220 | 1 | 13.59 | Trans |
| M2 | 04/22/2011 0:00 | 12 | 49.683403 | -109.723856 | 592055 | 5504213 | 1 | 43.26 | Trans |
| M2 | 04/22/2011 0:00 | 15 | 49.683710 | -109.723488 | 592081 | 5504247 | 1 | 37.51 | Trans |
| M2 | 04/22/2011 0:00 | 18 | 49.683421 | -109.723756 | 592063 | 5504215 | 1 | 20.71 | Trans |
| M2 | 04/22/2011 0:00 | 21 | 49.683447 | -109.723472 | 592083 | 5504218 | 1 | 339.34 | Trans |

| | | | | | | | | | |
|---|---|---|---|---|---|---|---|---|---|
| M2 | 04/23/2011 0:00 | 0 | 49.682092 | -109.727687 | 591782 | 5504062 | 1 | 446.22 | Trans |
| M2 | 04/23/2011 0:00 | 3 | 49.685866 | -109.725581 | 591926 | 5504484 | 0 | 1542.46 | Trans |
| M2 | 04/23/2011 0:00 | 9 | 49.693534 | -109.743400 | 590627 | 5505315 | 1 | 12.99 | Trans |
| M2 | 04/23/2011 0:00 | 12 | 49.693427 | -109.743472 | 590622 | 5505303 | 1 | 9.00 | Trans |
| M2 | 04/23/2011 0:00 | 15 | 49.693506 | -109.743450 | 590623 | 5505312 | 1 | 5.12 | Trans |
| M2 | 04/23/2011 0:00 | 18 | 49.693462 | -109.743428 | 590625 | 5505307 | 1 | 124.07 | Trans |
| M2 | 04/23/2011 0:00 | 21 | 49.694428 | -109.742568 | 590685 | 5505416 | 1 | 257.83 | Trans |
| M2 | 04/24/2011 0:00 | 0 | 49.694972 | -109.746043 | 590434 | 5505472 | 1 | 3459.94 | Trans |
| M2 | 04/24/2011 0:00 | 3 | 49.715398 | -109.782238 | 587786 | 5507700 | 1 | 371.05 | Trans |
| M2 | 04/24/2011 0:00 | 6 | 49.715639 | -109.787372 | 587416 | 5507721 | 1 | 5.90 | Trans |
| M2 | 04/24/2011 0:00 | 9 | 49.715594 | -109.787327 | 587419 | 5507716 | 1 | 8.30 | Trans |
| M2 | 04/24/2011 0:00 | 12 | 49.715521 | -109.787347 | 587418 | 5507708 | 1 | 15.00 | Trans |
| M2 | 04/24/2011 0:00 | 15 | 49.715645 | -109.787427 | 587412 | 5507721 | 1 | 8.38 | Trans |
| M2 | 04/24/2011 0:00 | 18 | 49.715645 | -109.787311 | 587420 | 5507721 | 1 | 34.20 | Trans |
| M2 | 04/24/2011 0:00 | 21 | 49.715641 | -109.786837 | 587455 | 5507721 | 1 | 1816.64 | Trans |
| M2 | 04/25/2011 0:00 | 0 | 49.712401 | -109.811533 | 585680 | 5507333 | 1 | 2705.27 | Trans |
| M2 | 04/25/2011 0:00 | 3 | 49.718957 | -109.847669 | 583064 | 5508021 | 1 | 5663.21 | Trans |
| M2 | 04/25/2011 0:00 | 6 | 49.762445 | -109.888583 | 580043 | 5512811 | 1 | 2111.12 | Trans |
| M2 | 04/25/2011 0:00 | 9 | 49.745996 | -109.873946 | 581125 | 5510998 | 1 | 4.30 | Trans |
| M2 | 04/25/2011 0:00 | 12 | 49.745960 | -109.873968 | 581123 | 5510994 | 1 | 4.69 | Trans |
| M2 | 04/25/2011 0:00 | 15 | 49.746000 | -109.873991 | 581121 | 5510999 | 1 | 9.69 | Trans |
| M2 | 04/25/2011 0:00 | 18 | 49.745917 | -109.874031 | 581119 | 5510990 | 1 | 1077.04 | Trans |
| M2 | 04/25/2011 0:00 | 21 | 49.736860 | -109.879334 | 580752 | 5509977 | 1 | 5851.63 | Trans |
| M2 | 04/26/2011 0:00 | 0 | 49.702124 | -109.940315 | 576412 | 5506051 | 1 | 447.40 | Trans |
| M2 | 04/26/2011 0:00 | 3 | 49.698242 | -109.938683 | 576536 | 5505621 | 1 | 2767.92 | Trans |
| M2 | 04/26/2011 0:00 | 6 | 49.677616 | -109.960169 | 575018 | 5503307 | 1 | 146.79 | Trans |
| M2 | 04/26/2011 0:00 | 9 | 49.677498 | -109.962195 | 574872 | 5503292 | 1 | 81.70 | Trans |
| M2 | 04/26/2011 0:00 | 12 | 49.676931 | -109.962915 | 574821 | 5503228 | 1 | 14.72 | Trans |
| M2 | 04/26/2011 0:00 | 15 | 49.676940 | -109.963118 | 574806 | 5503228 | 1 | 5.06 | Trans |
| M2 | 04/26/2011 0:00 | 18 | 49.676960 | -109.963056 | 574811 | 5503231 | 1 | 107.75 | Trans |
| M2 | 04/26/2011 0:00 | 21 | 49.675991 | -109.962997 | 574817 | 5503123 | 1 | 999.90 | Trans |
| M2 | 04/27/2011 0:00 | 0 | 49.667975 | -109.969278 | 574376 | 5502226 | 1 | 2419.59 | Trans |
| M2 | 04/27/2011 0:00 | 3 | 49.667678 | -110.002803 | 571957 | 5502160 | 1 | 1539.60 | Trans |
| M2 | 04/27/2011 0:00 | 6 | 49.659437 | -110.019946 | 570732 | 5501228 | 1 | 160.04 | Trans |
| M2 | 04/27/2011 0:00 | 9 | 49.660188 | -110.021837 | 570594 | 5501309 | 1 | 43.94 | Trans |
| M2 | 04/27/2011 0:00 | 12 | 49.659794 | -110.021882 | 570592 | 5501265 | 1 | 14.60 | Trans |
| M2 | 04/27/2011 0:00 | 15 | 49.659925 | -110.021900 | 570590 | 5501280 | 1 | 6.71 | Trans |
| M2 | 04/27/2011 0:00 | 18 | 49.659869 | -110.021935 | 570588 | 5501274 | 1 | 220.02 | Trans |
| M2 | 04/27/2011 0:00 | 21 | 49.659986 | -110.024978 | 570368 | 5501284 | 1 | 1308.89 | Trans |
| M2 | 04/28/2011 0:00 | 0 | 49.661472 | -110.042968 | 569068 | 5501432 | 1 | 2015.44 | Trans |
| M2 | 04/28/2011 0:00 | 3 | 49.663736 | -110.070675 | 567065 | 5501659 | 1 | 1362.39 | Trans |
| M2 | 04/28/2011 0:00 | 6 | 49.661652 | -110.089278 | 565725 | 5501411 | 1 | 154.81 | Trans |
| M2 | 04/28/2011 0:00 | 9 | 49.662911 | -110.090193 | 565658 | 5501550 | 1 | 224.14 | Trans |
| M2 | 04/28/2011 0:00 | 12 | 49.664552 | -110.091996 | 565525 | 5501731 | 1 | 121.11 | Trans |
| M2 | 04/28/2011 0:00 | 15 | 49.665618 | -110.092348 | 565498 | 5501849 | 1 | 12.60 | Trans |
| M2 | 04/28/2011 0:00 | 18 | 49.665516 | -110.092426 | 565493 | 5501838 | 0 | 1610.80 | Trans |
| M2 | 04/29/2011 0:00 | 0 | 49.664862 | -110.070129 | 567103 | 5501785 | 1 | 554.12 | Trans |
| M2 | 04/29/2011 0:00 | 3 | 49.660362 | -110.073428 | 566871 | 5501281 | 1 | 1642.86 | Trans |
| M2 | 04/29/2011 0:00 | 6 | 49.664401 | -110.051532 | 568445 | 5501750 | 1 | 198.21 | Trans |
| M2 | 04/29/2011 0:00 | 9 | 49.664597 | -110.048802 | 568642 | 5501774 | 1 | 377.16 | Trans |
| M2 | 04/29/2011 0:00 | 12 | 49.664201 | -110.043612 | 569017 | 5501735 | 1 | 1241.84 | Trans |
| M2 | 04/29/2011 0:00 | 15 | 49.661596 | -110.026880 | 570228 | 5501461 | 1 | 673.99 | Trans |
| M2 | 04/29/2011 0:00 | 18 | 49.659399 | -110.018176 | 570860 | 5501225 | 1 | 25.91 | Trans |
| M2 | 04/29/2011 0:00 | 21 | 49.659302 | -110.017850 | 570883 | 5501215 | 1 | 3111.58 | Trans |
| M2 | 04/30/2011 0:00 | 0 | 49.677790 | -110.050220 | 568521 | 5503240 | 1 | 12.82 | Trans |

| | | | | | | | | | |
|---|---|---|---|---|---|---|---|---|---|
| M2 | 04/30/2011 0:00 | 3 | 49.677678 | -110.050180 | 568524 | 5503227 | 1 | 1819.05 | Trans |
| M2 | 04/30/2011 0:00 | 6 | 49.677704 | -110.075392 | 566705 | 5503208 | 1 | 317.34 | Trans |
| M2 | 04/30/2011 0:00 | 9 | 49.677570 | -110.079785 | 566389 | 5503189 | 1 | 4.26 | Trans |
| M2 | 04/30/2011 0:00 | 12 | 49.677569 | -110.079726 | 566393 | 5503189 | 0 | 5.61 | Trans |
| M2 | 04/30/2011 0:00 | 18 | 49.677543 | -110.079793 | 566388 | 5503186 | 1 | 125.18 | Trans |
| M2 | 04/30/2011 0:00 | 21 | 49.677545 | -110.078059 | 566513 | 5503188 | 1 | 1239.66 | Trans |
| M2 | 05/01/2011 0:00 | 0 | 49.684813 | -110.091089 | 565563 | 5503984 | 1 | 2828.48 | Trans |
| M2 | 05/01/2011 0:00 | 3 | 49.680470 | -110.129720 | 562782 | 5503468 | 1 | 1142.68 | Trans |
| M2 | 05/01/2011 0:00 | 6 | 49.677780 | -110.145006 | 561683 | 5503157 | 1 | 16.27 | Trans |
| M2 | 05/01/2011 0:00 | 9 | 49.677923 | -110.145051 | 561680 | 5503173 | 1 | 109.99 | Trans |
| M2 | 05/01/2011 0:00 | 12 | 49.677197 | -110.146085 | 561606 | 5503091 | 1 | 241.36 | Trans |
| M2 | 05/01/2011 0:00 | 15 | 49.675385 | -110.147927 | 561475 | 5502888 | 1 | 14.37 | Trans |
| M2 | 05/01/2011 0:00 | 18 | 49.675421 | -110.147736 | 561489 | 5502892 | 1 | 703.91 | Trans |
| M2 | 05/01/2011 0:00 | 21 | 49.673671 | -110.157112 | 560815 | 5502690 | 1 | 865.54 | Trans |
| M2 | 05/02/2011 0:00 | 0 | 49.678353 | -110.166696 | 560118 | 5503203 | 1 | 2117.25 | Trans |
| M2 | 05/02/2011 0:00 | 3 | 49.694260 | -110.182832 | 558934 | 5504959 | 1 | 1151.13 | Trans |
| M2 | 05/02/2011 0:00 | 6 | 49.690354 | -110.197612 | 557873 | 5504513 | 1 | 157.28 | Trans |
| M2 | 05/02/2011 0:00 | 9 | 49.689096 | -110.196615 | 557946 | 5504374 | 1 | 22.18 | Trans |
| M2 | 05/02/2011 0:00 | 12 | 49.688929 | -110.196782 | 557935 | 5504355 | 1 | 10.29 | Trans |
| M2 | 05/02/2011 0:00 | 15 | 49.689011 | -110.196845 | 557930 | 5504364 | 1 | 3.93 | Trans |
| M2 | 05/02/2011 0:00 | 18 | 49.689047 | -110.196839 | 557930 | 5504368 | 1 | 160.01 | Trans |
| M2 | 05/02/2011 0:00 | 21 | 49.689164 | -110.199050 | 557771 | 5504379 | 1 | 3143.70 | Trans |
| M2 | 05/03/2011 0:00 | 0 | 49.681798 | -110.241125 | 554744 | 5503529 | 1 | 1202.12 | Trans |
| M2 | 05/03/2011 0:00 | 3 | 49.681635 | -110.257786 | 553542 | 5503499 | 1 | 1118.27 | Trans |
| M2 | 05/03/2011 0:00 | 6 | 49.672353 | -110.263756 | 553122 | 5502463 | 1 | 12.44 | Trans |
| M2 | 05/03/2011 0:00 | 9 | 49.672243 | -110.263789 | 553120 | 5502450 | 1 | 66.57 | Trans |
| M2 | 05/03/2011 0:00 | 12 | 49.671750 | -110.264312 | 553083 | 5502395 | 1 | 90.88 | Trans |
| M2 | 05/03/2011 0:00 | 15 | 49.672520 | -110.264734 | 553051 | 5502481 | 1 | 25.14 | Trans |
| M2 | 05/03/2011 0:00 | 18 | 49.672376 | -110.265002 | 553032 | 5502464 | 1 | 10.91 | Trans |
| M2 | 05/03/2011 0:00 | 21 | 49.672443 | -110.264892 | 553040 | 5502472 | 1 | 502.59 | Trans |
| M2 | 05/04/2011 0:00 | 0 | 49.668179 | -110.267204 | 552878 | 5501996 | 1 | 7.86 | Trans |
| M2 | 05/04/2011 0:00 | 3 | 49.668113 | -110.267162 | 552881 | 5501989 | 1 | 25.19 | Trans |
| M2 | 05/04/2011 0:00 | 6 | 49.668255 | -110.267435 | 552861 | 5502004 | 1 | 7.15 | Trans |
| M2 | 05/04/2011 0:00 | 9 | 49.668211 | -110.267362 | 552866 | 5502000 | 1 | 10.01 | Trans |
| M2 | 05/04/2011 0:00 | 12 | 49.668186 | -110.267229 | 552876 | 5501997 | 1 | 12.94 | Trans |
| M2 | 05/04/2011 0:00 | 15 | 49.668274 | -110.267347 | 552867 | 5502007 | 1 | 8.75 | Trans |
| M2 | 05/04/2011 0:00 | 18 | 49.668197 | -110.267322 | 552869 | 5501998 | 1 | 21.57 | Trans |
| M2 | 05/04/2011 0:00 | 21 | 49.668334 | -110.267534 | 552854 | 5502013 | 1 | 11.09 | Trans |
| M2 | 05/05/2011 0:00 | 0 | 49.668295 | -110.267392 | 552864 | 5502009 | 1 | 5.96 | Trans |
| M2 | 05/05/2011 0:00 | 3 | 49.668256 | -110.267449 | 552860 | 5502005 | 1 | 13.53 | Trans |
| M2 | 05/05/2011 0:00 | 6 | 49.668161 | -110.267333 | 552868 | 5501994 | 1 | 24.68 | Trans |
| M2 | 05/05/2011 0:00 | 9 | 49.668341 | -110.267533 | 552854 | 5502014 | 1 | 34.61 | Trans |
| M2 | 05/05/2011 0:00 | 12 | 49.668600 | -110.267800 | 552834 | 5502043 | 1 | 39.99 | Trans |
| M2 | 05/05/2011 0:00 | 15 | 49.668321 | -110.267449 | 552860 | 5502012 | 1 | 7.54 | Trans |
| M2 | 05/05/2011 0:00 | 18 | 49.668267 | -110.267387 | 552864 | 5502006 | 1 | 14.91 | Trans |
| M2 | 05/05/2011 0:00 | 21 | 49.668179 | -110.267230 | 552876 | 5501996 | 1 | 126.17 | Trans |
| M2 | 05/06/2011 0:00 | 0 | 49.667686 | -110.268805 | 552763 | 5501940 | 1 | 129.76 | Trans |
| M2 | 05/06/2011 0:00 | 3 | 49.668218 | -110.267205 | 552878 | 5502001 | 1 | 143.32 | Trans |
| M2 | 05/06/2011 0:00 | 6 | 49.667799 | -110.269083 | 552743 | 5501953 | 1 | 923.64 | Trans |
| M2 | 05/06/2011 0:00 | 9 | 49.675277 | -110.263508 | 553137 | 5502788 | 1 | 11.54 | Trans |
| M2 | 05/06/2011 0:00 | 12 | 49.675176 | -110.263466 | 553140 | 5502777 | 1 | 21.27 | Trans |
| M2 | 05/06/2011 0:00 | 15 | 49.675352 | -110.263583 | 553131 | 5502796 | 1 | 10.64 | Trans |
| M2 | 05/06/2011 0:00 | 18 | 49.675324 | -110.263442 | 553141 | 5502793 | 1 | 450.76 | Trans |
| M2 | 05/06/2011 0:00 | 21 | 49.671280 | -110.263001 | 553178 | 5502344 | 1 | 451.17 | Trans |
| M2 | 05/07/2011 0:00 | 0 | 49.668381 | -110.267377 | 552865 | 5502019 | 1 | 1.25 | Trans |

| | | | | | | | | | |
|---|---|---|---|---|---|---|---|---|---|
| M2 | 05/07/2011 0:00 | 3 | 49.668389 | -110.267364 | 552866 | 5502019 | 1 | 821.15 | Trans |
| M2 | 05/07/2011 0:00 | 6 | 49.675360 | -110.263604 | 553130 | 5502797 | 1 | 17.48 | Trans |
| M2 | 05/07/2011 0:00 | 9 | 49.675262 | -110.263414 | 553143 | 5502786 | 0 | 4.10 | Trans |
| M2 | 05/07/2011 0:00 | 15 | 49.675293 | -110.263444 | 553141 | 5502790 | 0 | 392.04 | Trans |
| M2 | 05/07/2011 0:00 | 21 | 49.671871 | -110.264759 | 553050 | 5502408 | 1 | 427.09 | Trans |
| M2 | 05/08/2011 0:00 | 0 | 49.668322 | -110.267022 | 552891 | 5502012 | 1 | 16.74 | Trans |
| M2 | 05/08/2011 0:00 | 3 | 49.668181 | -110.267104 | 552885 | 5501996 | 1 | 1039.05 | Trans |
| M2 | 05/08/2011 0:00 | 6 | 49.661427 | -110.257154 | 553610 | 5501253 | 1 | 23.84 | Trans |
| M2 | 05/08/2011 0:00 | 9 | 49.661641 | -110.257150 | 553610 | 5501276 | 1 | 12.16 | Trans |
| M2 | 05/08/2011 0:00 | 12 | 49.661537 | -110.257101 | 553614 | 5501265 | 1 | 32.26 | Trans |
| M2 | 05/08/2011 0:00 | 15 | 49.661311 | -110.256820 | 553634 | 5501240 | 1 | 225.99 | Trans |
| M2 | 05/08/2011 0:00 | 18 | 49.659333 | -110.257543 | 553585 | 5501020 | 1 | 337.60 | Trans |
| M2 | 05/08/2011 0:00 | 21 | 49.662232 | -110.258935 | 553481 | 5501341 | 1 | 906.15 | Trans |
| M2 | 05/09/2011 0:00 | 0 | 49.668306 | -110.267306 | 552870 | 5502010 | 0 | 599.48 | Trans |
| M2 | 05/09/2011 0:00 | 6 | 49.665021 | -110.260720 | 553349 | 5501650 | 1 | 252.76 | Trans |
| M2 | 05/09/2011 0:00 | 9 | 49.663524 | -110.258083 | 553541 | 5501485 | 1 | 7.41 | Trans |
| M2 | 05/09/2011 0:00 | 12 | 49.663482 | -110.258004 | 553547 | 5501480 | 1 | 86.87 | Trans |
| M2 | 05/09/2011 0:00 | 15 | 49.664179 | -110.258548 | 553507 | 5501558 | 1 | 85.78 | Trans |
| M2 | 05/09/2011 0:00 | 18 | 49.663522 | -110.257924 | 553552 | 5501485 | 1 | 437.36 | Trans |
| M2 | 05/09/2011 0:00 | 21 | 49.666593 | -110.261712 | 553276 | 5501824 | 1 | 454.64 | Trans |
| M2 | 05/10/2011 0:00 | 0 | 49.668377 | -110.267380 | 552865 | 5502018 | 1 | 8.87 | Trans |
| M2 | 05/10/2011 0:00 | 3 | 49.668305 | -110.267329 | 552869 | 5502010 | 1 | 741.73 | Trans |
| M2 | 05/10/2011 0:00 | 6 | 49.665263 | -110.258182 | 553532 | 5501678 | 1 | 200.57 | Trans |
| M2 | 05/10/2011 0:00 | 9 | 49.663615 | -110.257050 | 553615 | 5501496 | 1 | 23.80 | Trans |
| M2 | 05/10/2011 0:00 | 12 | 49.663654 | -110.257375 | 553592 | 5501500 | 1 | 16.34 | Trans |
| M2 | 05/10/2011 0:00 | 15 | 49.663572 | -110.257187 | 553606 | 5501491 | 1 | 22.46 | Trans |
| M2 | 05/10/2011 0:00 | 18 | 49.663415 | -110.257383 | 553592 | 5501473 | 1 | 449.81 | Trans |
| M2 | 05/10/2011 0:00 | 21 | 49.665678 | -110.252216 | 553962 | 5501729 | 1 | 218.18 | Trans |
| M2 | 05/11/2011 0:00 | 0 | 49.667096 | -110.254305 | 553810 | 5501885 | 1 | 11.65 | Trans |
| M2 | 05/11/2011 0:00 | 3 | 49.667065 | -110.254151 | 553821 | 5501882 | 1 | 459.16 | Trans |
| M2 | 05/11/2011 0:00 | 6 | 49.663484 | -110.257323 | 553596 | 5501481 | 1 | 8.34 | Trans |
| M2 | 05/11/2011 0:00 | 9 | 49.663518 | -110.257426 | 553588 | 5501485 | 1 | 22.99 | Trans |
| M2 | 05/11/2011 0:00 | 12 | 49.663422 | -110.257144 | 553609 | 5501474 | 1 | 21.49 | Trans |
| M2 | 05/11/2011 0:00 | 15 | 49.663575 | -110.257325 | 553596 | 5501491 | 1 | 8.66 | Trans |
| M2 | 05/11/2011 0:00 | 18 | 49.663554 | -110.257209 | 553604 | 5501489 | 1 | 429.24 | Trans |
| M2 | 05/11/2011 0:00 | 21 | 49.665654 | -110.252217 | 553962 | 5501726 | 1 | 224.82 | Trans |
| M2 | 05/12/2011 0:00 | 0 | 49.666973 | -110.254578 | 553790 | 5501871 | 1 | 55.91 | Trans |
| M2 | 05/12/2011 0:00 | 3 | 49.666835 | -110.253833 | 553844 | 5501856 | 1 | 299.44 | Trans |
| M2 | 05/12/2011 0:00 | 6 | 49.669467 | -110.254717 | 553777 | 5502148 | 1 | 14.33 | Trans |
| M2 | 05/12/2011 0:00 | 9 | 49.669596 | -110.254721 | 553777 | 5502163 | 1 | 190.07 | Trans |
| M2 | 05/12/2011 0:00 | 12 | 49.671207 | -110.253843 | 553838 | 5502342 | 1 | 412.16 | Trans |
| M2 | 05/12/2011 0:00 | 15 | 49.674213 | -110.250499 | 554076 | 5502679 | 0 | 851.14 | Trans |
| M2 | 05/12/2011 0:00 | 21 | 49.666938 | -110.254172 | 553819 | 5501867 | 0 | 3430.07 | Trans |
| M2 | 05/13/2011 0:00 | 3 | 49.659158 | -110.300165 | 550509 | 5500971 | 1 | 2092.40 | Trans |
| M2 | 05/13/2011 0:00 | 6 | 49.653034 | -110.327576 | 548536 | 5500272 | 1 | 1090.59 | Trans |
| M2 | 05/13/2011 0:00 | 9 | 49.650260 | -110.342067 | 547493 | 5499954 | 1 | 14.63 | Trans |
| M2 | 05/13/2011 0:00 | 12 | 49.650171 | -110.341918 | 547504 | 5499944 | 1 | 13.68 | Trans |
| M2 | 05/13/2011 0:00 | 15 | 49.650277 | -110.342014 | 547497 | 5499956 | 1 | 392.88 | Trans |
| M2 | 05/13/2011 0:00 | 18 | 49.649133 | -110.347163 | 547126 | 5499825 | 1 | 1603.62 | Trans |
| M2 | 05/13/2011 0:00 | 21 | 49.642839 | -110.367149 | 545690 | 5499113 | 1 | 1452.25 | Trans |
| M2 | 05/14/2011 0:00 | 0 | 49.639912 | -110.386752 | 544277 | 5498776 | 1 | 1860.67 | Trans |
| M2 | 05/14/2011 0:00 | 3 | 49.627027 | -110.403196 | 543101 | 5497334 | 1 | 188.59 | Trans |
| M2 | 05/14/2011 0:00 | 6 | 49.627414 | -110.400653 | 543284 | 5497379 | 1 | 8.31 | Trans |
| M2 | 05/14/2011 0:00 | 9 | 49.627469 | -110.400575 | 543290 | 5497385 | 1 | 15.85 | Trans |
| M2 | 05/14/2011 0:00 | 12 | 49.627583 | -110.400707 | 543280 | 5497398 | 1 | 37.63 | Trans |

| | | | | | | | | | |
|---|---|---|---|---|---|---|---|---|---|
| M2 | 05/14/2011 0:00 | 15 | 49.627359 | -110.400317 | 543309 | 5497373 | 0 | 129.38 | Trans |
| M2 | 05/14/2011 0:00 | 21 | 49.626886 | -110.401954 | 543191 | 5497319 | 1 | 11.63 | Trans |
| M2 | 05/15/2011 0:00 | 0 | 49.626796 | -110.402037 | 543185 | 5497309 | 1 | 12.77 | Trans |
| M2 | 05/15/2011 0:00 | 3 | 49.626836 | -110.401871 | 543197 | 5497314 | 1 | 109.60 | Trans |
| M2 | 05/15/2011 0:00 | 6 | 49.627415 | -110.400642 | 543285 | 5497379 | 0 | 10.84 | Trans |
| M2 | 05/15/2011 0:00 | 12 | 49.627381 | -110.400501 | 543295 | 5497375 | 0 | 12.72 | Trans |
| M2 | 05/15/2011 0:00 | 18 | 49.627429 | -110.400661 | 543284 | 5497380 | 1 | 215.15 | Trans |
| M2 | 05/15/2011 0:00 | 21 | 49.627484 | -110.397683 | 543499 | 5497388 | 1 | 297.58 | Trans |
| M2 | 05/16/2011 0:00 | 0 | 49.627059 | -110.401751 | 543205 | 5497339 | 1 | 16.41 | Trans |
| M2 | 05/16/2011 0:00 | 3 | 49.626950 | -110.401905 | 543194 | 5497326 | 1 | 4.43 | Trans |
| M2 | 05/16/2011 0:00 | 6 | 49.626929 | -110.401957 | 543191 | 5497324 | 1 | 14.80 | Trans |
| M2 | 05/16/2011 0:00 | 9 | 49.626799 | -110.401910 | 543194 | 5497310 | 1 | 9.22 | Trans |
| M2 | 05/16/2011 0:00 | 12 | 49.626717 | -110.401917 | 543194 | 5497300 | 1 | 17.66 | Trans |
| M2 | 05/16/2011 0:00 | 15 | 49.626844 | -110.402062 | 543183 | 5497315 | 1 | 1.87 | Trans |
| M2 | 05/16/2011 0:00 | 18 | 49.626828 | -110.402067 | 543183 | 5497313 | 1 | 12.83 | Trans |
| M2 | 05/16/2011 0:00 | 21 | 49.626828 | -110.401889 | 543196 | 5497313 | 1 | 30.18 | Trans |
| M2 | 05/17/2011 0:00 | 0 | 49.626567 | -110.402002 | 543188 | 5497284 | 1 | 16.74 | Trans |
| M2 | 05/17/2011 0:00 | 3 | 49.626430 | -110.402101 | 543181 | 5497269 | 1 | 57.99 | Trans |
| M2 | 05/17/2011 0:00 | 6 | 49.626945 | -110.401974 | 543189 | 5497326 | 0 | 1043.77 | Trans |
| M2 | 05/17/2011 0:00 | 12 | 49.625975 | -110.387600 | 544228 | 5497226 | 1 | 143.08 | Trans |
| M2 | 05/17/2011 0:00 | 15 | 49.624827 | -110.388495 | 544165 | 5497098 | 1 | 422.92 | Trans |
| M2 | 05/17/2011 0:00 | 18 | 49.626933 | -110.383619 | 544515 | 5497335 | 1 | 722.96 | Trans |
| M2 | 05/17/2011 0:00 | 21 | 49.620699 | -110.380772 | 544726 | 5496644 | 1 | 1262.62 | Trans |
| M2 | 05/18/2011 0:00 | 0 | 49.609349 | -110.380209 | 544777 | 5495382 | 1 | 1173.44 | Trans |
| M2 | 05/18/2011 0:00 | 3 | 49.600054 | -110.387905 | 544230 | 5494344 | 1 | 1551.56 | Trans |
| M2 | 05/18/2011 0:00 | 6 | 49.613007 | -110.395897 | 543641 | 5495780 | 1 | 244.97 | Trans |
| M2 | 05/18/2011 0:00 | 9 | 49.615172 | -110.396531 | 543593 | 5496020 | 1 | 10.47 | Trans |
| M2 | 05/18/2011 0:00 | 12 | 49.615078 | -110.396527 | 543593 | 5496010 | 1 | 11.55 | Trans |
| M2 | 05/18/2011 0:00 | 15 | 49.615181 | -110.396544 | 543592 | 5496021 | 1 | 22.47 | Trans |
| M2 | 05/18/2011 0:00 | 18 | 49.615252 | -110.396253 | 543613 | 5496029 | 1 | 1380.72 | Trans |
| M2 | 05/18/2011 0:00 | 21 | 49.627020 | -110.402357 | 543162 | 5497334 | 1 | 41.69 | Trans |
| M2 | 05/19/2011 0:00 | 0 | 49.626787 | -110.401906 | 543194 | 5497308 | 1 | 14.04 | Trans |
| M2 | 05/19/2011 0:00 | 3 | 49.626875 | -110.402045 | 543184 | 5497318 | 1 | 108.21 | Trans |
| M2 | 05/19/2011 0:00 | 6 | 49.627573 | -110.401001 | 543259 | 5497396 | 1 | 179.38 | Trans |
| M2 | 05/19/2011 0:00 | 9 | 49.627676 | -110.398522 | 543438 | 5497409 | 1 | 7.18 | Trans |
| M2 | 05/19/2011 0:00 | 12 | 49.627674 | -110.398621 | 543431 | 5497409 | 1 | 8.29 | Trans |
| M2 | 05/19/2011 0:00 | 15 | 49.627605 | -110.398668 | 543428 | 5497401 | 1 | 16.61 | Trans |
| M2 | 05/19/2011 0:00 | 18 | 49.627742 | -110.398574 | 543434 | 5497416 | 1 | 261.16 | Trans |
| M2 | 05/19/2011 0:00 | 21 | 49.626887 | -110.401942 | 543192 | 5497319 | 1 | 1824.85 | Trans |
| M2 | 05/20/2011 0:00 | 0 | 49.642021 | -110.411725 | 542472 | 5498996 | 1 | 1853.54 | Trans |
| M2 | 05/20/2011 0:00 | 3 | 49.655072 | -110.395749 | 543614 | 5500456 | 1 | 1618.87 | Trans |
| M2 | 05/20/2011 0:00 | 6 | 49.653145 | -110.417980 | 542011 | 5500230 | 1 | 20.29 | Trans |
| M2 | 05/20/2011 0:00 | 9 | 49.653292 | -110.418145 | 541999 | 5500246 | 1 | 12.29 | Trans |
| M2 | 05/20/2011 0:00 | 12 | 49.653394 | -110.418212 | 541994 | 5500257 | 1 | 68.81 | Trans |
| M2 | 05/20/2011 0:00 | 15 | 49.652856 | -110.418683 | 541960 | 5500197 | 1 | 9.65 | Trans |
| M2 | 05/20/2011 0:00 | 18 | 49.652893 | -110.418562 | 541969 | 5500201 | 1 | 711.13 | Trans |
| M2 | 05/20/2011 0:00 | 21 | 49.649389 | -110.426803 | 541377 | 5499807 | 1 | 3743.94 | Trans |
| M2 | 05/21/2011 0:00 | 0 | 49.635066 | -110.473735 | 538001 | 5498190 | 1 | 7579.93 | Trans |
| M2 | 05/21/2011 0:00 | 3 | 49.632891 | -110.578651 | 530426 | 5497900 | 1 | 7139.92 | Trans |
| M2 | 05/21/2011 0:00 | 6 | 49.631571 | -110.479799 | 537565 | 5497798 | 1 | 820.33 | Trans |
| M2 | 05/21/2011 0:00 | 9 | 49.629880 | -110.468742 | 538365 | 5497616 | 1 | 38.78 | Trans |
| M2 | 05/21/2011 0:00 | 12 | 49.629557 | -110.468540 | 538380 | 5497580 | 1 | 2.02 | Trans |
| M2 | 05/21/2011 0:00 | 15 | 49.629545 | -110.468519 | 538381 | 5497579 | 1 | 10.33 | Trans |
| M2 | 05/21/2011 0:00 | 18 | 49.629470 | -110.468604 | 538375 | 5497570 | 1 | 78.65 | Trans |
| M2 | 05/21/2011 0:00 | 21 | 49.628823 | -110.468163 | 538408 | 5497499 | 1 | 29.59 | Trans |

| | | | | | | | | | |
|---|---|---|---|---|---|---|---|---|---|
| M2 | 05/22/2011 0:00 | 0 | 49.628854 | -110.468570 | 538378 | 5497502 | 1 | 39.53 | Trans |
| M2 | 05/22/2011 0:00 | 3 | 49.628705 | -110.468072 | 538414 | 5497486 | 1 | 11.44 | Trans |
| M2 | 05/22/2011 0:00 | 6 | 49.628781 | -110.468181 | 538407 | 5497494 | 1 | 11.76 | Trans |
| M2 | 05/22/2011 0:00 | 9 | 49.628770 | -110.468019 | 538418 | 5497493 | 1 | 19.43 | Trans |
| M2 | 05/22/2011 0:00 | 12 | 49.628635 | -110.467847 | 538431 | 5497478 | 1 | 31.75 | Trans |
| M2 | 05/22/2011 0:00 | 15 | 49.628848 | -110.468140 | 538409 | 5497501 | 1 | 17.39 | Trans |
| M2 | 05/22/2011 0:00 | 18 | 49.628743 | -110.468318 | 538397 | 5497490 | 1 | 31.67 | Trans |
| M2 | 05/22/2011 0:00 | 21 | 49.628990 | -110.468101 | 538412 | 5497517 | 1 | 34.69 | Trans |
| M2 | 05/23/2011 0:00 | 0 | 49.628687 | -110.468218 | 538404 | 5497484 | 1 | 60.61 | Trans |
| M2 | 05/23/2011 0:00 | 3 | 49.628316 | -110.467603 | 538449 | 5497443 | 1 | 63.30 | Trans |
| M2 | 05/23/2011 0:00 | 6 | 49.628803 | -110.468058 | 538415 | 5497497 | 1 | 10.46 | Trans |
| M2 | 05/23/2011 0:00 | 9 | 49.628768 | -110.468192 | 538406 | 5497493 | 0 | 17.26 | Trans |
| M2 | 05/23/2011 0:00 | 15 | 49.628644 | -110.468048 | 538416 | 5497479 | 1 | 22.38 | Trans |
| M2 | 05/23/2011 0:00 | 18 | 49.628659 | -110.468357 | 538394 | 5497480 | 1 | 19.42 | Trans |
| M2 | 05/23/2011 0:00 | 21 | 49.628721 | -110.468106 | 538412 | 5497487 | 0 | 3.05 | Trans |
| M2 | 05/24/2011 0:00 | 6 | 49.628749 | -110.468108 | 538412 | 5497490 | 1 | 11.95 | Trans |
| M2 | 05/24/2011 0:00 | 9 | 49.628823 | -110.468227 | 538403 | 5497499 | 1 | 34.03 | Trans |
| M2 | 05/24/2011 0:00 | 12 | 49.629018 | -110.467864 | 538429 | 5497521 | 1 | 36.04 | Trans |
| M2 | 05/24/2011 0:00 | 15 | 49.628772 | -110.468189 | 538406 | 5497493 | 1 | 5.97 | Trans |
| M2 | 05/24/2011 0:00 | 18 | 49.628774 | -110.468106 | 538412 | 5497493 | 0 | 71.57 | Trans |
| M2 | 05/25/2011 0:00 | 0 | 49.628355 | -110.468858 | 538358 | 5497446 | 0 | 68.12 | Trans |
| M2 | 05/25/2011 0:00 | 6 | 49.628746 | -110.468132 | 538410 | 5497490 | 0 | 17.09 | Trans |
| M2 | 05/25/2011 0:00 | 12 | 49.628881 | -110.468244 | 538402 | 5497505 | 1 | 27.89 | Trans |
| M2 | 05/25/2011 0:00 | 15 | 49.628631 | -110.468265 | 538401 | 5497477 | 1 | 59.29 | Trans |
| M2 | 05/25/2011 0:00 | 18 | 49.629134 | -110.467994 | 538420 | 5497533 | 1 | 62.11 | Trans |
| M2 | 05/25/2011 0:00 | 21 | 49.628587 | -110.468166 | 538408 | 5497472 | 1 | 6.05 | Trans |
| M2 | 05/26/2011 0:00 | 0 | 49.628641 | -110.468169 | 538407 | 5497478 | 1 | 13.34 | Trans |
| M2 | 05/26/2011 0:00 | 3 | 49.628760 | -110.468200 | 538405 | 5497492 | 1 | 13.43 | Trans |
| M2 | 05/26/2011 0:00 | 6 | 49.628878 | -110.468166 | 538408 | 5497505 | 1 | 10.00 | Trans |
| M2 | 05/26/2011 0:00 | 9 | 49.628923 | -110.468285 | 538399 | 5497510 | 1 | 17.54 | Trans |
| M2 | 05/26/2011 0:00 | 12 | 49.628804 | -110.468127 | 538410 | 5497497 | 1 | 19.98 | Trans |
| M2 | 05/26/2011 0:00 | 15 | 49.628865 | -110.467866 | 538429 | 5497503 | 1 | 58.83 | Trans |
| M2 | 05/26/2011 0:00 | 18 | 49.628464 | -110.468398 | 538391 | 5497459 | 1 | 26.87 | Trans |
| M2 | 05/26/2011 0:00 | 21 | 49.628677 | -110.468223 | 538404 | 5497482 | 1 | 318.34 | Trans |
| M2 | 05/27/2011 0:00 | 0 | 49.626741 | -110.464975 | 538640 | 5497269 | 1 | 16.00 | Trans |
| M2 | 05/27/2011 0:00 | 3 | 49.626665 | -110.464787 | 538653 | 5497260 | 1 | 753.85 | Trans |
| M2 | 05/27/2011 0:00 | 6 | 49.621802 | -110.457514 | 539182 | 5496724 | 1 | 879.50 | Trans |
| M2 | 05/27/2011 0:00 | 9 | 49.616970 | -110.447873 | 539883 | 5496191 | 0 | 369.07 | Trans |
| M2 | 05/27/2011 0:00 | 15 | 49.616069 | -110.442956 | 540239 | 5496094 | 0 | 1515.91 | Trans |
| M2 | 05/27/2011 0:00 | 21 | 49.602461 | -110.441634 | 540345 | 5494582 | 1 | 3721.46 | Trans |
| M2 | 05/28/2011 0:00 | 0 | 49.617249 | -110.395423 | 543671 | 5496252 | 1 | 1368.31 | Trans |
| M2 | 05/28/2011 0:00 | 3 | 49.625647 | -110.381575 | 544664 | 5497193 | 1 | 1363.56 | Trans |
| M2 | 05/28/2011 0:00 | 6 | 49.634119 | -110.367923 | 545642 | 5498144 | 1 | 216.50 | Trans |
| M2 | 05/28/2011 0:00 | 9 | 49.635749 | -110.369563 | 545522 | 5498324 | 1 | 1349.89 | Trans |
| M2 | 05/28/2011 0:00 | 12 | 49.632034 | -110.351767 | 546810 | 5497922 | 0 | 3193.64 | Trans |
| M2 | 05/28/2011 0:00 | 21 | 49.655899 | -110.327149 | 548564 | 5500590 | 1 | 1048.76 | Trans |
| M2 | 05/29/2011 0:00 | 0 | 49.654302 | -110.312829 | 549600 | 5500422 | 1 | 690.78 | Trans |
| M2 | 05/29/2011 0:00 | 3 | 49.658687 | -110.306050 | 550084 | 5500914 | 1 | 412.05 | Trans |
| M2 | 05/29/2011 0:00 | 6 | 49.656137 | -110.301908 | 550386 | 5500634 | 1 | 57.10 | Trans |
| M2 | 05/29/2011 0:00 | 9 | 49.655945 | -110.302641 | 550333 | 5500612 | 1 | 15.37 | Trans |
| M2 | 05/29/2011 0:00 | 12 | 49.656082 | -110.302616 | 550335 | 5500627 | 1 | 15.53 | Trans |
| M2 | 05/29/2011 0:00 | 15 | 49.655992 | -110.302781 | 550323 | 5500617 | 1 | 10.54 | Trans |
| M2 | 05/29/2011 0:00 | 18 | 49.656062 | -110.302683 | 550330 | 5500625 | 1 | 26.20 | Trans |
| M2 | 05/29/2011 0:00 | 21 | 49.656126 | -110.302333 | 550355 | 5500632 | 1 | 25.41 | Trans |
| M2 | 05/30/2011 0:00 | 0 | 49.656058 | -110.301997 | 550380 | 5500625 | 1 | 35.10 | Trans |

| | | | | | | | | | |
|---|---|---|---|---|---|---|---|---|---|
| M2 | 05/30/2011 0:00 | 3 | 49.655909 | -110.302426 | 550349 | 5500608 | 1 | 40.52 | Trans |
| M2 | 05/30/2011 0:00 | 6 | 49.656114 | -110.301961 | 550382 | 5500631 | 1 | 95.29 | Trans |
| M2 | 05/30/2011 0:00 | 9 | 49.655570 | -110.300942 | 550456 | 5500571 | 0 | 118.99 | Trans |
| M2 | 05/30/2011 0:00 | 15 | 49.656047 | -110.302417 | 550349 | 5500623 | 1 | 9.29 | Trans |
| M2 | 05/30/2011 0:00 | 18 | 49.655976 | -110.302348 | 550354 | 5500615 | 1 | 31.69 | Trans |
| M2 | 05/30/2011 0:00 | 21 | 49.656104 | -110.301955 | 550383 | 5500630 | 1 | 3.80 | Trans |
| M2 | 05/31/2011 0:00 | 0 | 49.656070 | -110.301948 | 550383 | 5500626 | 1 | 12.95 | Trans |
| M2 | 05/31/2011 0:00 | 3 | 49.656127 | -110.301793 | 550394 | 5500633 | 1 | 51.25 | Trans |
| M2 | 05/31/2011 0:00 | 6 | 49.656001 | -110.302476 | 550345 | 5500618 | 1 | 3.39 | Trans |
| M2 | 05/31/2011 0:00 | 9 | 49.656009 | -110.302430 | 550348 | 5500619 | 1 | 9.26 | Trans |
| M2 | 05/31/2011 0:00 | 12 | 49.655945 | -110.302512 | 550343 | 5500612 | 0 | 21.35 | Trans |
| M2 | 05/31/2011 0:00 | 18 | 49.656126 | -110.302414 | 550349 | 5500632 | 1 | 552.99 | Trans |
| M2 | 05/31/2011 0:00 | 21 | 49.652801 | -110.296716 | 550764 | 5500266 | 1 | 2323.47 | Trans |
| M2 | 06/01/2011 0:00 | 0 | 49.642926 | -110.268351 | 552822 | 5499188 | 1 | 1224.26 | Trans |
| M2 | 06/01/2011 0:00 | 3 | 49.637560 | -110.253545 | 553897 | 5498602 | 1 | 2607.85 | Trans |
| M2 | 06/01/2011 0:00 | 6 | 49.658225 | -110.236456 | 555108 | 5500912 | 1 | 1002.63 | Trans |
| M2 | 06/01/2011 0:00 | 9 | 49.662957 | -110.224630 | 555956 | 5501446 | 1 | 49.49 | Trans |
| M2 | 06/01/2011 0:00 | 12 | 49.662867 | -110.225302 | 555907 | 5501436 | 0 | 1425.44 | Trans |
| M2 | 06/01/2011 0:00 | 18 | 49.668015 | -110.207212 | 557207 | 5502022 | 1 | 693.83 | Trans |
| M2 | 06/01/2011 0:00 | 21 | 49.665227 | -110.198610 | 557831 | 5501718 | 1 | 282.31 | Trans |
| M2 | 06/02/2011 0:00 | 0 | 49.664561 | -110.202385 | 557559 | 5501642 | 0 | 288.57 | Trans |
| M2 | 06/02/2011 0:00 | 6 | 49.664868 | -110.198415 | 557845 | 5501679 | 1 | 109.33 | Trans |
| M2 | 06/02/2011 0:00 | 9 | 49.665263 | -110.197027 | 557945 | 5501724 | 0 | 31.91 | Trans |
| M2 | 06/02/2011 0:00 | 15 | 49.665089 | -110.197379 | 557920 | 5501704 | 0 | 89.19 | Trans |
| M2 | 06/02/2011 0:00 | 21 | 49.665553 | -110.198387 | 557846 | 5501755 | 1 | 733.08 | Trans |
| M2 | 06/03/2011 0:00 | 0 | 49.661152 | -110.190824 | 558398 | 5501271 | 1 | 13.27 | Trans |
| M2 | 06/03/2011 0:00 | 3 | 49.661267 | -110.190776 | 558401 | 5501284 | 1 | 887.42 | Trans |
| M2 | 06/03/2011 0:00 | 6 | 49.664556 | -110.201980 | 557588 | 5501641 | 0 | 3231.04 | Trans |
| M2 | 06/03/2011 0:00 | 12 | 49.647622 | -110.165601 | 560235 | 5499787 | 0 | 1707.27 | Trans |
| M2 | 06/03/2011 0:00 | 18 | 49.642038 | -110.143573 | 561832 | 5499184 | 0 | 3150.59 | Trans |
| M2 | 06/04/2011 0:00 | 0 | 49.629050 | -110.104795 | 564649 | 5497773 | 1 | 34.29 | Local |
| M2 | 06/04/2011 0:00 | 3 | 49.629358 | -110.104783 | 564649 | 5497807 | 1 | 17.65 | Local |
| M2 | 06/04/2011 0:00 | 6 | 49.629282 | -110.104569 | 564665 | 5497799 | 1 | 90.29 | Local |
| M2 | 06/04/2011 0:00 | 9 | 49.628782 | -110.105553 | 564594 | 5497742 | 1 | 42.32 | Local |
| M2 | 06/04/2011 0:00 | 12 | 49.629038 | -110.105120 | 564625 | 5497771 | 1 | 52.31 | Local |
| M2 | 06/04/2011 0:00 | 15 | 49.629308 | -110.104527 | 564668 | 5497802 | 1 | 168.59 | Local |
| M2 | 06/04/2011 0:00 | 18 | 49.630823 | -110.104619 | 564659 | 5497970 | 1 | 162.55 | Local |
| M2 | 06/04/2011 0:00 | 21 | 49.629372 | -110.104355 | 564680 | 5497809 | 1 | 50.81 | Local |
| M2 | 06/05/2011 0:00 | 0 | 49.629063 | -110.104874 | 564643 | 5497774 | 1 | 51.39 | Local |
| M2 | 06/05/2011 0:00 | 3 | 49.629503 | -110.104653 | 564658 | 5497823 | 1 | 185.09 | Local |
| M2 | 06/05/2011 0:00 | 6 | 49.629119 | -110.102159 | 564839 | 5497783 | 1 | 202.18 | Local |
| M2 | 06/05/2011 0:00 | 9 | 49.628178 | -110.099764 | 565013 | 5497680 | 0 | 370.54 | Local |
| M2 | 06/05/2011 0:00 | 15 | 49.629464 | -110.104497 | 564670 | 5497819 | 1 | 101.25 | Local |
| M2 | 06/05/2011 0:00 | 18 | 49.628651 | -110.105131 | 564625 | 5497728 | 1 | 548.50 | Local |
| M2 | 06/05/2011 0:00 | 21 | 49.626202 | -110.098538 | 565104 | 5497462 | 1 | 564.32 | Local |
| M2 | 06/06/2011 0:00 | 0 | 49.629404 | -110.104601 | 564662 | 5497812 | 1 | 7.99 | Local |
| M2 | 06/06/2011 0:00 | 3 | 49.629337 | -110.104560 | 564665 | 5497805 | 0 | 216.38 | Local |
| M2 | 06/06/2011 0:00 | 9 | 49.629218 | -110.101570 | 564881 | 5497794 | 1 | 117.80 | Local |
| M2 | 06/06/2011 0:00 | 12 | 49.629700 | -110.100117 | 564986 | 5497849 | 1 | 28.93 | Local |
| M2 | 06/06/2011 0:00 | 15 | 49.629784 | -110.100496 | 564958 | 5497858 | 1 | 5.89 | Local |
| M2 | 06/06/2011 0:00 | 18 | 49.629815 | -110.100429 | 564963 | 5497862 | 1 | 299.90 | Local |
| M2 | 06/06/2011 0:00 | 21 | 49.629415 | -110.104536 | 564667 | 5497814 | 1 | 7.92 | Local |
| M2 | 06/07/2011 0:00 | 0 | 49.629343 | -110.104543 | 564666 | 5497806 | 1 | 2.69 | Local |
| M2 | 06/07/2011 0:00 | 3 | 49.629355 | -110.104576 | 564664 | 5497807 | 1 | 21.41 | Local |
| M2 | 06/07/2011 0:00 | 6 | 49.629178 | -110.104692 | 564656 | 5497787 | 0 | 131.29 | Local |

| | | | | | | | | | |
|---|---|---|---|---|---|---|---|---|---|
| M2 | 06/07/2011 0:00 | 12 | 49.628157 | -110.103779 | 564723 | 5497674 | 1 | 12.02 | Local |
| M2 | 06/07/2011 0:00 | 15 | 49.628255 | -110.103849 | 564718 | 5497685 | 1 | 845.25 | Local |
| M2 | 06/07/2011 0:00 | 18 | 49.622182 | -110.096809 | 565234 | 5497016 | 1 | 800.55 | Local |
| M2 | 06/07/2011 0:00 | 21 | 49.619804 | -110.086348 | 565993 | 5496761 | 1 | 12.93 | Local |
| M2 | 06/08/2011 0:00 | 0 | 49.619716 | -110.086464 | 565985 | 5496751 | 0 | 1647.39 | Local |
| M2 | 06/08/2011 0:00 | 6 | 49.632278 | -110.074369 | 566841 | 5498158 | 1 | 53.46 | Local |
| M2 | 06/08/2011 0:00 | 9 | 49.632412 | -110.075080 | 566790 | 5498173 | 0 | 40.34 | Local |
| M2 | 06/08/2011 0:00 | 15 | 49.632070 | -110.075269 | 566777 | 5498135 | 0 | 984.06 | Local |
| M2 | 06/08/2011 0:00 | 21 | 49.635466 | -110.087853 | 565863 | 5498501 | 0 | 1386.66 | Local |
| M2 | 06/09/2011 0:00 | 3 | 49.629364 | -110.104600 | 564662 | 5497808 | 1 | 6.56 | Local |
| M2 | 06/09/2011 0:00 | 6 | 49.629358 | -110.104509 | 564669 | 5497807 | 1 | 164.77 | Local |
| M2 | 06/09/2011 0:00 | 9 | 49.628218 | -110.103052 | 564776 | 5497682 | 1 | 958.28 | Local |
| M2 | 06/09/2011 0:00 | 12 | 49.620976 | -110.095857 | 565305 | 5496883 | 1 | 361.47 | Local |
| M2 | 06/09/2011 0:00 | 15 | 49.620321 | -110.100758 | 564952 | 5496806 | 1 | 7.17 | Local |
| M2 | 06/09/2011 0:00 | 18 | 49.620383 | -110.100788 | 564949 | 5496813 | 1 | 813.16 | Local |
| M2 | 06/09/2011 0:00 | 21 | 49.613141 | -110.102366 | 564845 | 5496006 | 0 | 269.88 | Local |
| M2 | 06/10/2011 0:00 | 3 | 49.612984 | -110.098638 | 565115 | 5495992 | 0 | 1848.79 | Local |
| M2 | 06/10/2011 0:00 | 9 | 49.622000 | -110.077134 | 566656 | 5497013 | 1 | 32.09 | Local |
| M2 | 06/10/2011 0:00 | 12 | 49.622128 | -110.076736 | 566684 | 5497028 | 1 | 19.14 | Local |
| M2 | 06/10/2011 0:00 | 15 | 49.621969 | -110.076836 | 566677 | 5497010 | 1 | 15.60 | Local |
| M2 | 06/10/2011 0:00 | 18 | 49.622102 | -110.076901 | 566672 | 5497025 | 1 | 16.31 | Local |
| M2 | 06/10/2011 0:00 | 21 | 49.621958 | -110.076859 | 566676 | 5497009 | 0 | 5.91 | Local |
| M2 | 06/11/2011 0:00 | 3 | 49.621992 | -110.076795 | 566680 | 5497013 | 0 | 1182.89 | Local |
| M2 | 06/11/2011 0:00 | 9 | 49.626967 | -110.091272 | 565628 | 5497553 | 1 | 23.74 | Local |
| M2 | 06/11/2011 0:00 | 12 | 49.626856 | -110.091553 | 565608 | 5497540 | 1 | 8.44 | Local |
| M2 | 06/11/2011 0:00 | 15 | 49.626782 | -110.091580 | 565606 | 5497532 | 1 | 232.87 | Local |
| M2 | 06/11/2011 0:00 | 18 | 49.628685 | -110.092929 | 565506 | 5497743 | 1 | 1842.53 | Local |
| M2 | 06/11/2011 0:00 | 21 | 49.644533 | -110.085471 | 566023 | 5499511 | 1 | 589.11 | Local |
| M2 | 06/12/2011 0:00 | 0 | 49.639850 | -110.089288 | 565754 | 5498987 | 1 | 44.64 | Local |
| M2 | 06/12/2011 0:00 | 3 | 49.639465 | -110.089113 | 565767 | 5498944 | 1 | 3524.47 | Local |
| M2 | 06/12/2011 0:00 | 6 | 49.610576 | -110.109202 | 564355 | 5495715 | 1 | 815.48 | Local |
| M2 | 06/12/2011 0:00 | 9 | 49.615465 | -110.100787 | 564956 | 5496266 | 0 | 727.70 | Local |
| M2 | 06/12/2011 0:00 | 21 | 49.622009 | -110.100960 | 564935 | 5496993 | 1 | 754.55 | Local |
| M2 | 06/13/2011 0:00 | 0 | 49.627251 | -110.094325 | 565407 | 5497582 | 1 | 2300.77 | Local |
| M2 | 06/13/2011 0:00 | 3 | 49.640603 | -110.118666 | 563632 | 5499046 | 0 | 4397.50 | Local |
| M2 | 06/13/2011 0:00 | 9 | 49.639679 | -110.057779 | 568029 | 5498996 | 0 | 716.61 | Local |
| M2 | 06/13/2011 0:00 | 15 | 49.633266 | -110.056792 | 568109 | 5498284 | 1 | 2.65 | Local |
| M2 | 06/13/2011 0:00 | 18 | 49.633288 | -110.056777 | 568110 | 5498286 | 1 | 938.46 | Local |
| M2 | 06/13/2011 0:00 | 21 | 49.633038 | -110.069767 | 567173 | 5498247 | 1 | 1752.24 | Local |
| M2 | 06/14/2011 0:00 | 0 | 49.620376 | -110.055323 | 568233 | 5496852 | 1 | 416.29 | Local |
| M2 | 06/14/2011 0:00 | 3 | 49.618797 | -110.050097 | 568613 | 5496682 | 1 | 363.68 | Local |
| M2 | 06/14/2011 0:00 | 6 | 49.615823 | -110.048003 | 568768 | 5496353 | 1 | 610.91 | Local |
| M2 | 06/14/2011 0:00 | 9 | 49.610328 | -110.048060 | 568772 | 5495742 | 1 | 753.08 | Local |
| M2 | 06/14/2011 0:00 | 12 | 49.616831 | -110.045140 | 568974 | 5496467 | 1 | 45.71 | Local |
| M2 | 06/14/2011 0:00 | 15 | 49.616470 | -110.044836 | 568996 | 5496428 | 1 | 18.14 | Local |
| M2 | 06/14/2011 0:00 | 18 | 49.616308 | -110.044863 | 568995 | 5496410 | 1 | 224.21 | Local |
| M2 | 06/14/2011 0:00 | 21 | 49.615456 | -110.047676 | 568793 | 5496312 | 1 | 1523.40 | Local |
| M2 | 06/15/2011 0:00 | 0 | 49.611780 | -110.027363 | 570265 | 5495922 | 1 | 2732.81 | Local |
| M2 | 06/15/2011 0:00 | 3 | 49.603255 | -109.991888 | 572841 | 5495008 | 1 | 903.04 | Local |
| M2 | 06/15/2011 0:00 | 6 | 49.595145 | -109.991220 | 572901 | 5494107 | 1 | 860.51 | Local |
| M2 | 06/15/2011 0:00 | 9 | 49.598885 | -110.001645 | 572142 | 5494513 | 1 | 2134.52 | Local |
| M2 | 06/15/2011 0:00 | 12 | 49.613706 | -109.982869 | 573477 | 5496179 | 1 | 447.60 | Local |
| M2 | 06/15/2011 0:00 | 15 | 49.617728 | -109.982581 | 573491 | 5496626 | 1 | 1201.63 | Local |
| M2 | 06/15/2011 0:00 | 18 | 49.617381 | -109.999206 | 572291 | 5496572 | 1 | 3296.51 | Local |
| M2 | 06/15/2011 0:00 | 21 | 49.616283 | -110.044808 | 568999 | 5496407 | 1 | 231.17 | Local |

| | | | | | | | | | |
|---|---|---|---|---|---|---|---|---|---|
| M2 | 06/16/2011 0:00 | 0 | 49.615709 | -110.047883 | 568777 | 5496340 | 1 | 30.22 | Local |
| M2 | 06/16/2011 0:00 | 3 | 49.615439 | -110.047839 | 568781 | 5496310 | 1 | 108.06 | Local |
| M2 | 06/16/2011 0:00 | 6 | 49.615512 | -110.049331 | 568673 | 5496317 | 1 | 1949.65 | Local |
| M2 | 06/16/2011 0:00 | 9 | 49.628095 | -110.030533 | 570013 | 5497733 | 1 | 1268.31 | Local |
| M2 | 06/16/2011 0:00 | 12 | 49.636526 | -110.018702 | 570855 | 5498682 | 1 | 464.38 | Local |
| M2 | 06/16/2011 0:00 | 15 | 49.640620 | -110.017426 | 570941 | 5499138 | 1 | 168.98 | Local |
| M2 | 06/16/2011 0:00 | 18 | 49.639211 | -110.016546 | 571007 | 5498982 | 1 | 564.83 | Local |
| M2 | 06/16/2011 0:00 | 21 | 49.634946 | -110.020796 | 570706 | 5498504 | 1 | 23.50 | Local |
| M2 | 06/17/2011 0:00 | 0 | 49.634861 | -110.020497 | 570728 | 5498495 | 1 | 10.72 | Local |
| M2 | 06/17/2011 0:00 | 3 | 49.634892 | -110.020638 | 570718 | 5498498 | 1 | 6.00 | Local |
| M2 | 06/17/2011 0:00 | 6 | 49.634841 | -110.020664 | 570716 | 5498492 | 1 | 12.46 | Local |
| M2 | 06/17/2011 0:00 | 9 | 49.634940 | -110.020744 | 570710 | 5498503 | 1 | 2.18 | Local |
| M2 | 06/17/2011 0:00 | 12 | 49.634947 | -110.020716 | 570712 | 5498504 | 1 | 32.00 | Local |
| M2 | 06/17/2011 0:00 | 15 | 49.634858 | -110.020295 | 570742 | 5498495 | 1 | 25.04 | Local |
| M2 | 06/17/2011 0:00 | 18 | 49.634858 | -110.020642 | 570717 | 5498494 | 1 | 8.26 | Local |
| M2 | 06/17/2011 0:00 | 21 | 49.634895 | -110.020542 | 570724 | 5498499 | 1 | 660.75 | Local |
| M2 | 06/18/2011 0:00 | 0 | 49.631107 | -110.027592 | 570221 | 5498071 | 1 | 632.10 | Local |
| M2 | 06/18/2011 0:00 | 3 | 49.627750 | -110.034655 | 569716 | 5497691 | 1 | 1364.98 | Local |
| M2 | 06/18/2011 0:00 | 6 | 49.617102 | -110.044063 | 569051 | 5496499 | 1 | 957.20 | Local |
| M2 | 06/18/2011 0:00 | 9 | 49.618027 | -110.057236 | 568098 | 5496589 | 1 | 53.85 | Local |
| M2 | 06/18/2011 0:00 | 12 | 49.618147 | -110.056514 | 568150 | 5496603 | 1 | 82.86 | Local |
| M2 | 06/18/2011 0:00 | 15 | 49.617632 | -110.055685 | 568211 | 5496547 | 1 | 2.07 | Local |
| M2 | 06/18/2011 0:00 | 18 | 49.617630 | -110.055713 | 568209 | 5496547 | 1 | 121.72 | Local |
| M2 | 06/18/2011 0:00 | 21 | 49.618049 | -110.057271 | 568096 | 5496592 | 1 | 3.83 | Local |
| M2 | 06/19/2011 0:00 | 0 | 49.618074 | -110.057306 | 568093 | 5496595 | 1 | 4.10 | Local |
| M2 | 06/19/2011 0:00 | 3 | 49.618043 | -110.057336 | 568091 | 5496591 | 1 | 198.77 | Local |
| M2 | 06/19/2011 0:00 | 6 | 49.618272 | -110.054607 | 568288 | 5496619 | 1 | 164.12 | Local |
| M2 | 06/19/2011 0:00 | 9 | 49.617174 | -110.056126 | 568180 | 5496496 | 1 | 128.27 | Local |
| M2 | 06/19/2011 0:00 | 12 | 49.617921 | -110.054773 | 568277 | 5496580 | 1 | 6.24 | Local |
| M2 | 06/19/2011 0:00 | 15 | 49.617896 | -110.054850 | 568271 | 5496577 | 1 | 167.54 | Local |
| M2 | 06/19/2011 0:00 | 18 | 49.618047 | -110.057157 | 568104 | 5496592 | 1 | 22.59 | Local |
| M2 | 06/19/2011 0:00 | 21 | 49.617865 | -110.057295 | 568094 | 5496571 | 1 | 24.85 | Local |
| M2 | 06/20/2011 0:00 | 0 | 49.618057 | -110.057119 | 568107 | 5496593 | 1 | 17.86 | Local |
| M2 | 06/20/2011 0:00 | 3 | 49.617901 | -110.057182 | 568103 | 5496575 | 1 | 4.44 | Local |
| M2 | 06/20/2011 0:00 | 6 | 49.617941 | -110.057188 | 568102 | 5496580 | 0 | 7.73 | Local |
| M2 | 06/20/2011 0:00 | 12 | 49.618005 | -110.057229 | 568099 | 5496587 | 1 | 32.93 | Local |
| M2 | 06/20/2011 0:00 | 15 | 49.617728 | -110.057068 | 568111 | 5496556 | 1 | 49.96 | Local |
| M2 | 06/20/2011 0:00 | 18 | 49.618158 | -110.057272 | 568096 | 5496604 | 0 | 24.66 | Local |
| M2 | 06/21/2011 0:00 | 0 | 49.617944 | -110.057181 | 568103 | 5496580 | 1 | 8.18 | Local |
| M2 | 06/21/2011 0:00 | 3 | 49.617870 | -110.057184 | 568102 | 5496572 | 1 | 16.42 | Local |
| M2 | 06/21/2011 0:00 | 6 | 49.618014 | -110.057237 | 568098 | 5496588 | 0 | 625.01 | Local |
| M2 | 06/21/2011 0:00 | 12 | 49.615457 | -110.049532 | 568659 | 5496311 | 1 | 9.67 | Local |
| M2 | 06/21/2011 0:00 | 15 | 49.615505 | -110.049643 | 568650 | 5496316 | 1 | 18.17 | Local |
| M2 | 06/21/2011 0:00 | 18 | 49.615535 | -110.049396 | 568668 | 5496319 | 1 | 615.25 | Local |
| M2 | 06/21/2011 0:00 | 21 | 49.617854 | -110.057129 | 568106 | 5496570 | 1 | 11.34 | Local |
| M2 | 06/22/2011 0:00 | 0 | 49.617865 | -110.057285 | 568095 | 5496571 | 1 | 190.96 | Local |
| M2 | 06/22/2011 0:00 | 3 | 49.618724 | -110.059574 | 567929 | 5496665 | 1 | 1277.51 | Local |
| M2 | 06/22/2011 0:00 | 6 | 49.610157 | -110.047790 | 568792 | 5495723 | 0 | 1892.69 | Local |
| M2 | 06/22/2011 0:00 | 18 | 49.625137 | -110.035344 | 569670 | 5497400 | 1 | 1047.71 | Local |
| M2 | 06/22/2011 0:00 | 21 | 49.626455 | -110.049708 | 568630 | 5497533 | 1 | 166.09 | Local |
| M2 | 06/23/2011 0:00 | 0 | 49.625772 | -110.051753 | 568484 | 5497455 | 1 | 228.63 | Local |
| M2 | 06/23/2011 0:00 | 3 | 49.626939 | -110.054359 | 568294 | 5497583 | 1 | 342.32 | Local |
| M2 | 06/23/2011 0:00 | 6 | 49.628073 | -110.058766 | 567974 | 5497705 | 0 | 1345.77 | Local |
| M2 | 06/23/2011 0:00 | 15 | 49.632991 | -110.041739 | 569197 | 5498267 | 0 | 1027.28 | Local |
| M2 | 06/23/2011 0:00 | 21 | 49.625882 | -110.050824 | 568551 | 5497468 | 1 | 2333.32 | Local |

| | | | | | | | | | |
|---|---|---|---|---|---|---|---|---|---|
| M2 | 06/24/2011 0:00 | 0 | 49.621282 | -110.082343 | 566280 | 5496929 | 1 | 1153.21 | Local |
| M2 | 06/24/2011 0:00 | 3 | 49.615012 | -110.095060 | 565370 | 5496221 | 1 | 1674.40 | Local |
| M2 | 06/24/2011 0:00 | 6 | 49.609639 | -110.116711 | 563813 | 5495605 | 1 | 1167.68 | Local |
| M2 | 06/24/2011 0:00 | 9 | 49.617063 | -110.105280 | 564629 | 5496440 | 1 | 1358.77 | Local |
| M2 | 06/24/2011 0:00 | 12 | 49.628054 | -110.097054 | 565209 | 5497669 | 1 | 23.01 | Local |
| M2 | 06/24/2011 0:00 | 15 | 49.628260 | -110.097083 | 565207 | 5497692 | 0 | 3111.24 | Local |
| M2 | 06/24/2011 0:00 | 21 | 49.644238 | -110.061712 | 567739 | 5499499 | 1 | 897.53 | Local |
| M2 | 06/25/2011 0:00 | 0 | 49.650103 | -110.053169 | 568347 | 5500159 | 0 | 2823.04 | Local |
| M2 | 06/25/2011 0:00 | 6 | 49.624736 | -110.051487 | 568504 | 5497340 | 1 | 598.97 | Local |
| M2 | 06/25/2011 0:00 | 9 | 49.629143 | -110.046718 | 568843 | 5497835 | 1 | 64.96 | Local |
| M2 | 06/25/2011 0:00 | 12 | 49.629703 | -110.046458 | 568861 | 5497897 | 1 | 73.54 | Local |
| M2 | 06/25/2011 0:00 | 15 | 49.629107 | -110.046901 | 568829 | 5497831 | 1 | 11.96 | Local |
| M2 | 06/25/2011 0:00 | 18 | 49.629206 | -110.046965 | 568825 | 5497842 | 1 | 1654.97 | Local |
| M2 | 06/25/2011 0:00 | 21 | 49.641830 | -110.034822 | 569684 | 5499256 | 1 | 2268.79 | Local |
| M2 | 06/26/2011 0:00 | 0 | 49.659175 | -110.018265 | 570854 | 5501200 | 1 | 613.81 | Local |
| M2 | 06/26/2011 0:00 | 3 | 49.655705 | -110.011651 | 571336 | 5500821 | 1 | 1051.95 | Local |
| M2 | 06/26/2011 0:00 | 6 | 49.650871 | -109.999124 | 572247 | 5500295 | 1 | 1774.98 | Local |
| M2 | 06/26/2011 0:00 | 9 | 49.664663 | -110.011509 | 571333 | 5501817 | 1 | 546.96 | Local |
| M2 | 06/26/2011 0:00 | 12 | 49.659984 | -110.009170 | 571509 | 5501299 | 1 | 8.77 | Local |
| M2 | 06/26/2011 0:00 | 15 | 49.660062 | -110.009156 | 571510 | 5501307 | 1 | 1016.67 | Local |
| M2 | 06/26/2011 0:00 | 18 | 49.655469 | -109.996976 | 572396 | 5500808 | 1 | 69.45 | Local |
| M2 | 06/26/2011 0:00 | 21 | 49.655029 | -109.997658 | 572347 | 5500759 | 1 | 77.01 | Local |
| M2 | 06/27/2011 0:00 | 0 | 49.655519 | -109.996903 | 572401 | 5500814 | 1 | 10.87 | Local |
| M2 | 06/27/2011 0:00 | 3 | 49.655435 | -109.996825 | 572407 | 5500805 | 1 | 62.12 | Local |
| M2 | 06/27/2011 0:00 | 6 | 49.655524 | -109.997675 | 572345 | 5500814 | 1 | 140.23 | Local |
| M2 | 06/27/2011 0:00 | 9 | 49.655364 | -109.999602 | 572206 | 5500794 | 0 | 555.42 | Local |
| M2 | 06/27/2011 0:00 | 18 | 49.657525 | -110.006540 | 571702 | 5501028 | 1 | 130.47 | Local |
| M2 | 06/27/2011 0:00 | 21 | 49.657107 | -110.008229 | 571581 | 5500980 | 0 | 1098.75 | Local |
| M2 | 06/28/2011 0:00 | 6 | 49.653959 | -109.993800 | 572627 | 5500644 | 1 | 967.18 | Local |
| M2 | 06/28/2011 0:00 | 9 | 49.657911 | -110.005737 | 571760 | 5501071 | 1 | 35.98 | Local |
| M2 | 06/28/2011 0:00 | 12 | 49.657605 | -110.005577 | 571772 | 5501038 | 1 | 10.53 | Local |
| M2 | 06/28/2011 0:00 | 15 | 49.657663 | -110.005462 | 571780 | 5501044 | 1 | 15.61 | Local |
| M2 | 06/28/2011 0:00 | 18 | 49.657618 | -110.005667 | 571765 | 5501039 | 1 | 100.88 | Local |
| M2 | 06/28/2011 0:00 | 21 | 49.658442 | -110.006252 | 571722 | 5501130 | 1 | 1515.88 | Local |
| M2 | 06/29/2011 0:00 | 0 | 49.663530 | -110.025737 | 570308 | 5501677 | 1 | 661.03 | Local |
| M2 | 06/29/2011 0:00 | 3 | 49.667999 | -110.031779 | 569866 | 5502168 | 1 | 1151.69 | Local |
| M2 | 06/29/2011 0:00 | 6 | 49.659993 | -110.021654 | 570608 | 5501288 | 1 | 1638.32 | Local |
| M2 | 06/29/2011 0:00 | 9 | 49.665760 | -110.042543 | 569092 | 5501910 | 1 | 474.89 | Local |
| M2 | 06/29/2011 0:00 | 12 | 49.667179 | -110.048750 | 568642 | 5502062 | 0 | 241.96 | Local |
| M2 | 06/29/2011 0:00 | 18 | 49.665009 | -110.049011 | 568626 | 5501820 | 1 | 199.63 | Local |
| M2 | 06/29/2011 0:00 | 21 | 49.663227 | -110.049339 | 568605 | 5501622 | 1 | 1485.51 | Local |
| M2 | 06/30/2011 0:00 | 0 | 49.666634 | -110.029435 | 570037 | 5502019 | 1 | 378.19 | Local |
| M2 | 06/30/2011 0:00 | 3 | 49.669891 | -110.030944 | 569923 | 5502380 | 1 | 0.65 | Local |
| M2 | 06/30/2011 0:00 | 6 | 49.669897 | -110.030944 | 569923 | 5502380 | 1 | 14.23 | Local |
| M2 | 06/30/2011 0:00 | 9 | 49.669946 | -110.030762 | 569936 | 5502386 | 1 | 22.38 | Local |
| M2 | 06/30/2011 0:00 | 12 | 49.670049 | -110.031028 | 569917 | 5502397 | 1 | 2.16 | Local |
| M2 | 06/30/2011 0:00 | 15 | 49.670046 | -110.030999 | 569919 | 5502397 | 1 | 4.23 | Local |
| M2 | 06/30/2011 0:00 | 18 | 49.670082 | -110.031020 | 569917 | 5502401 | 1 | 22.75 | Local |
| M2 | 06/30/2011 0:00 | 21 | 49.669880 | -110.030965 | 569922 | 5502378 | 1 | 294.27 | Local |
| M2 | 07/01/2011 0:00 | 0 | 49.672047 | -110.028624 | 570088 | 5502621 | 1 | 1036.83 | Local |
| M2 | 07/01/2011 0:00 | 3 | 49.664357 | -110.020498 | 570685 | 5501774 | 0 | 47.36 | Local |
| M2 | 07/01/2011 0:00 | 9 | 49.664783 | -110.020507 | 570684 | 5501821 | 1 | 28.98 | Local |
| M2 | 07/01/2011 0:00 | 12 | 49.664549 | -110.020684 | 570671 | 5501795 | 1 | 8.24 | Local |
| M2 | 07/01/2011 0:00 | 15 | 49.664496 | -110.020603 | 570677 | 5501789 | 1 | 52.33 | Local |
| M2 | 07/01/2011 0:00 | 18 | 49.664692 | -110.021262 | 570629 | 5501811 | 1 | 46.34 | Local |

| | | | | | | | | | | |
|---|---|---|---|---|---|---|---|---|---|---|
| M2 | 07/01/2011 0:00 | 21 | 49.664863 | -110.020676 | 570671 | 5501830 | 0 | 14.07 | Local |
| M2 | 07/02/2011 0:00 | 6 | 49.664748 | -110.020595 | 570677 | 5501817 | 1 | 4.07 | Local |
| M2 | 07/02/2011 0:00 | 9 | 49.664784 | -110.020604 | 570677 | 5501821 | 1 | 23.28 | Local |
| M2 | 07/02/2011 0:00 | 12 | 49.664594 | -110.020739 | 570667 | 5501800 | 1 | 67.13 | Local |
| M2 | 07/02/2011 0:00 | 15 | 49.664983 | -110.021450 | 570615 | 5501843 | 0 | 64.21 | Local |
| M2 | 07/02/2011 0:00 | 21 | 49.664893 | -110.020571 | 570679 | 5501834 | 1 | 38.65 | Local |
| M2 | 07/03/2011 0:00 | 0 | 49.665110 | -110.020153 | 570709 | 5501858 | 1 | 384.85 | Local |
| M2 | 07/03/2011 0:00 | 3 | 49.664004 | -110.015100 | 571075 | 5501740 | 1 | 294.03 | Local |
| M2 | 07/03/2011 0:00 | 6 | 49.664376 | -110.019133 | 570783 | 5501777 | 1 | 17.95 | Local |
| M2 | 07/03/2011 0:00 | 9 | 49.664242 | -110.018994 | 570794 | 5501763 | 1 | 597.56 | Local |
| M2 | 07/03/2011 0:00 | 12 | 49.660563 | -110.025029 | 570363 | 5501348 | 0 | 10.50 | Local |
| M2 | 07/03/2011 0:00 | 18 | 49.660651 | -110.024974 | 570367 | 5501358 | 0 | 17.68 | Local |
| M2 | 07/04/2011 0:00 | 0 | 49.660768 | -110.025139 | 570355 | 5501371 | 1 | 5.03 | Local |
| M2 | 07/04/2011 0:00 | 3 | 49.660743 | -110.025196 | 570351 | 5501368 | 1 | 198.63 | Local |
| M2 | 07/04/2011 0:00 | 6 | 49.662319 | -110.026492 | 570255 | 5501542 | 1 | 60.86 | Local |
| M2 | 07/04/2011 0:00 | 9 | 49.662861 | -110.026617 | 570246 | 5501602 | 1 | 15.16 | Local |
| M2 | 07/04/2011 0:00 | 12 | 49.662736 | -110.026702 | 570240 | 5501588 | 1 | 16.00 | Local |
| M2 | 07/04/2011 0:00 | 15 | 49.662686 | -110.026494 | 570255 | 5501583 | 0 | 10.73 | Local |
| M2 | 07/04/2011 0:00 | 21 | 49.662712 | -110.026351 | 570265 | 5501586 | 1 | 492.18 | Local |
| M2 | 07/05/2011 0:00 | 0 | 49.665110 | -110.020618 | 570675 | 5501858 | 1 | 0.46 | Local |
| M2 | 07/05/2011 0:00 | 3 | 49.665113 | -110.020614 | 570676 | 5501858 | 1 | 485.75 | Local |
| M2 | 07/05/2011 0:00 | 6 | 49.663422 | -110.014408 | 571126 | 5501676 | 1 | 503.75 | Local |
| M2 | 07/05/2011 0:00 | 9 | 49.660210 | -110.009486 | 571486 | 5501323 | 1 | 54.26 | Local |
| M2 | 07/05/2011 0:00 | 12 | 49.659754 | -110.009216 | 571506 | 5501273 | 1 | 16.85 | Local |
| M2 | 07/05/2011 0:00 | 15 | 49.659903 | -110.009169 | 571509 | 5501290 | 1 | 4.69 | Local |
| M2 | 07/05/2011 0:00 | 18 | 49.659861 | -110.009180 | 571508 | 5501285 | 1 | 93.88 | Local |
| M2 | 07/05/2011 0:00 | 21 | 49.659237 | -110.010057 | 571446 | 5501215 | 1 | 655.29 | Local |
| M2 | 07/06/2011 0:00 | 0 | 49.663017 | -110.017023 | 570938 | 5501628 | 1 | 849.76 | Local |
| M2 | 07/06/2011 0:00 | 3 | 49.670552 | -110.015056 | 571069 | 5502468 | 1 | 1890.52 | Local |
| M2 | 07/06/2011 0:00 | 6 | 49.660407 | -109.994032 | 572601 | 5501360 | 1 | 71.17 | Local |
| M2 | 07/06/2011 0:00 | 9 | 49.661009 | -109.994370 | 572575 | 5501427 | 1 | 236.23 | Local |
| M2 | 07/06/2011 0:00 | 12 | 49.660887 | -109.991102 | 572811 | 5501416 | 1 | 7.81 | Local |
| M2 | 07/06/2011 0:00 | 15 | 49.660942 | -109.991169 | 572807 | 5501422 | 1 | 39.39 | Local |
| M2 | 07/06/2011 0:00 | 18 | 49.660852 | -109.991697 | 572769 | 5501412 | 1 | 259.84 | Local |
| M2 | 07/06/2011 0:00 | 21 | 49.659338 | -109.994440 | 572573 | 5501241 | 1 | 2946.68 | Local |
| M2 | 07/07/2011 0:00 | 0 | 49.675384 | -110.026938 | 570204 | 5502994 | 1 | 1801.65 | Local |
| M2 | 07/07/2011 0:00 | 3 | 49.682600 | -110.004579 | 571807 | 5503817 | 1 | 3274.80 | Local |
| M2 | 07/07/2011 0:00 | 6 | 49.666455 | -110.042540 | 569091 | 5501987 | 1 | 279.78 | Local |
| M2 | 07/07/2011 0:00 | 9 | 49.665051 | -110.045757 | 568861 | 5501828 | 1 | 116.09 | Local |
| M2 | 07/07/2011 0:00 | 12 | 49.664239 | -110.046767 | 568790 | 5501736 | 1 | 1.02 | Local |
| M2 | 07/07/2011 0:00 | 15 | 49.664244 | -110.046755 | 568790 | 5501737 | 1 | 77.30 | Local |
| M2 | 07/07/2011 0:00 | 18 | 49.663713 | -110.046064 | 568841 | 5501679 | 0 | 1769.73 | Local |
| M2 | 07/08/2011 0:00 | 0 | 49.679113 | -110.052261 | 568372 | 5503385 | 1 | 3980.42 | Local |
| M2 | 07/08/2011 0:00 | 3 | 49.667989 | -109.999827 | 572171 | 5502198 | 1 | 5071.51 | Local |
| M2 | 07/08/2011 0:00 | 6 | 49.670027 | -109.929619 | 577234 | 5502494 | 1 | 1287.01 | Local |
| M2 | 07/08/2011 0:00 | 9 | 49.670628 | -109.911808 | 578518 | 5502579 | 1 | 3.93 | Local |
| M2 | 07/08/2011 0:00 | 12 | 49.670595 | -109.911828 | 578517 | 5502575 | 1 | 37.96 | Local |
| M2 | 07/08/2011 0:00 | 15 | 49.670612 | -109.912353 | 578479 | 5502577 | 1 | 220.11 | Local |
| M2 | 07/08/2011 0:00 | 18 | 49.671422 | -109.909570 | 578678 | 5502670 | 1 | 1530.10 | Local |
| M2 | 07/08/2011 0:00 | 21 | 49.685125 | -109.907626 | 578797 | 5504195 | 1 | 1936.07 | Local |
| M2 | 07/09/2011 0:00 | 0 | 49.696273 | -109.887006 | 580266 | 5505456 | 1 | 24.88 | Local |
| M2 | 07/09/2011 0:00 | 3 | 49.696132 | -109.887274 | 580247 | 5505440 | 1 | 2437.45 | Local |
| M2 | 07/09/2011 0:00 | 6 | 49.682489 | -109.913724 | 578361 | 5503896 | 1 | 8.21 | Local |
| M2 | 07/09/2011 0:00 | 9 | 49.682563 | -109.913729 | 578361 | 5503904 | 1 | 13.38 | Local |
| M2 | 07/09/2011 0:00 | 12 | 49.682450 | -109.913791 | 578356 | 5503891 | 1 | 6.73 | Local |

| | | | | | | | | | |
|---|---|---|---|---|---|---|---|---|---|
| M2 | 07/09/2011 0:00 | 15 | 49.682508 | -109.913766 | 578358 | 5503898 | 1 | 3.63 | Local |
| M2 | 07/09/2011 0:00 | 18 | 49.682476 | -109.913769 | 578358 | 5503894 | 1 | 484.38 | Local |
| M2 | 07/09/2011 0:00 | 21 | 49.684104 | -109.919997 | 577906 | 5504069 | 1 | 2614.64 | Local |
| M2 | 07/10/2011 0:00 | 0 | 49.695618 | -109.888392 | 580167 | 5505382 | 1 | 442.71 | Local |
| M2 | 07/10/2011 0:00 | 3 | 49.698728 | -109.884558 | 580438 | 5505732 | 1 | 3283.61 | Local |
| M2 | 07/10/2011 0:00 | 6 | 49.674791 | -109.911217 | 578554 | 5503043 | 0 | 206.79 | Local |
| M2 | 07/10/2011 0:00 | 12 | 49.676276 | -109.912942 | 578427 | 5503206 | 0 | 63.36 | Local |
| M2 | 07/10/2011 0:00 | 18 | 49.675822 | -109.912411 | 578466 | 5503156 | 1 | 327.02 | Local |
| M2 | 07/10/2011 0:00 | 21 | 49.676800 | -109.916686 | 578156 | 5503260 | 1 | 954.96 | Local |
| M2 | 07/11/2011 0:00 | 0 | 49.669636 | -109.909386 | 578695 | 5502471 | 1 | 2448.70 | Local |
| M2 | 07/11/2011 0:00 | 3 | 49.660393 | -109.878589 | 580932 | 5501476 | 1 | 3667.17 | Local |
| M2 | 07/11/2011 0:00 | 6 | 49.681540 | -109.917587 | 578084 | 5503786 | 1 | 293.25 | Local |
| M2 | 07/11/2011 0:00 | 9 | 49.684147 | -109.918202 | 578035 | 5504075 | 1 | 27.86 | Local |
| M2 | 07/11/2011 0:00 | 12 | 49.684326 | -109.918473 | 578015 | 5504095 | 1 | 293.54 | Local |
| M2 | 07/11/2011 0:00 | 15 | 49.684505 | -109.922533 | 577722 | 5504111 | 1 | 122.53 | Local |
| M2 | 07/11/2011 0:00 | 18 | 49.685166 | -109.923892 | 577623 | 5504183 | 1 | 6.83 | Local |
| M2 | 07/11/2011 0:00 | 21 | 49.685106 | -109.923911 | 577622 | 5504176 | 1 | 415.05 | Local |
| M2 | 07/12/2011 0:00 | 0 | 49.685994 | -109.918323 | 578024 | 5504281 | 1 | 16.15 | Local |
| M2 | 07/12/2011 0:00 | 3 | 49.685895 | -109.918486 | 578012 | 5504269 | 1 | 224.18 | Local |
| M2 | 07/12/2011 0:00 | 6 | 49.685495 | -109.921532 | 577793 | 5504222 | 1 | 8.45 | Local |
| M2 | 07/12/2011 0:00 | 9 | 49.685571 | -109.921531 | 577793 | 5504230 | 0 | 187.42 | Local |
| M2 | 07/12/2011 0:00 | 15 | 49.685114 | -109.924031 | 577613 | 5504177 | 1 | 39.92 | Local |
| M2 | 07/12/2011 0:00 | 18 | 49.684771 | -109.924196 | 577602 | 5504139 | 1 | 125.35 | Local |
| M2 | 07/12/2011 0:00 | 21 | 49.685845 | -109.923670 | 577638 | 5504259 | 1 | 1421.71 | Local |
| M2 | 07/13/2011 0:00 | 0 | 49.695539 | -109.910816 | 578550 | 5505350 | 1 | 414.64 | Local |
| M2 | 07/13/2011 0:00 | 3 | 49.694555 | -109.905270 | 578951 | 5505246 | 1 | 1297.67 | Local |
| M2 | 07/13/2011 0:00 | 6 | 49.683265 | -109.909834 | 578640 | 5503986 | 0 | 313.58 | Local |
| M2 | 07/13/2011 0:00 | 12 | 49.682947 | -109.914153 | 578329 | 5503946 | 0 | 646.76 | Local |
| M2 | 07/13/2011 0:00 | 18 | 49.684236 | -109.922895 | 577697 | 5504080 | 1 | 284.54 | Local |
| M2 | 07/13/2011 0:00 | 21 | 49.682881 | -109.919550 | 577940 | 5503933 | 0 | 1485.51 | Local |
| M2 | 07/14/2011 0:00 | 3 | 49.674991 | -109.936166 | 576754 | 5503039 | 1 | 3095.53 | Local |
| M2 | 07/14/2011 0:00 | 6 | 49.664038 | -109.975604 | 573925 | 5501782 | 1 | 959.70 | Local |
| M2 | 07/14/2011 0:00 | 9 | 49.661233 | -109.988180 | 573022 | 5501458 | 1 | 1333.74 | Local |
| M2 | 07/14/2011 0:00 | 12 | 49.657983 | -110.005968 | 571743 | 5501079 | 1 | 6.76 | Local |
| M2 | 07/14/2011 0:00 | 15 | 49.658023 | -110.006039 | 571738 | 5501084 | 1 | 4.21 | Local |
| M2 | 07/14/2011 0:00 | 18 | 49.658047 | -110.005994 | 571741 | 5501086 | 1 | 579.00 | Local |
| M2 | 07/14/2011 0:00 | 21 | 49.661803 | -110.011551 | 571334 | 5501499 | 1 | 1271.55 | Local |
| M2 | 07/15/2011 0:00 | 0 | 49.672016 | -110.003624 | 571891 | 5502642 | 1 | 1642.00 | Local |
| M2 | 07/15/2011 0:00 | 3 | 49.659550 | -109.991426 | 572790 | 5501267 | 1 | 1504.20 | Local |
| M2 | 07/15/2011 0:00 | 6 | 49.665612 | -109.972794 | 574126 | 5501960 | 1 | 21.38 | Local |
| M2 | 07/15/2011 0:00 | 9 | 49.665719 | -109.972548 | 574143 | 5501972 | 1 | 49.04 | Local |
| M2 | 07/15/2011 0:00 | 12 | 49.666134 | -109.972315 | 574159 | 5502018 | 1 | 68.01 | Local |
| M2 | 07/15/2011 0:00 | 15 | 49.665544 | -109.972563 | 574142 | 5501952 | 1 | 204.39 | Local |
| M2 | 07/15/2011 0:00 | 18 | 49.665362 | -109.969745 | 574346 | 5501935 | 1 | 188.19 | Local |
| M2 | 07/15/2011 0:00 | 21 | 49.665276 | -109.972349 | 574158 | 5501923 | 1 | 43.59 | Local |
| M2 | 07/16/2011 0:00 | 0 | 49.665659 | -109.972478 | 574148 | 5501965 | 0 | 68.59 | Local |
| M2 | 07/16/2011 0:00 | 6 | 49.665994 | -109.973276 | 574090 | 5502001 | 0 | 93.33 | Local |
| M2 | 07/16/2011 0:00 | 15 | 49.665619 | -109.972119 | 574174 | 5501961 | 1 | 8.64 | Local |
| M2 | 07/16/2011 0:00 | 18 | 49.665691 | -109.972074 | 574177 | 5501969 | 0 | 727.21 | Local |
| M2 | 07/17/2011 0:00 | 0 | 49.672087 | -109.974182 | 574016 | 5502678 | 1 | 1933.02 | Local |
| M2 | 07/17/2011 0:00 | 3 | 49.688892 | -109.967316 | 574485 | 5504553 | 0 | 260.87 | Local |
| M2 | 07/17/2011 0:00 | 9 | 49.687919 | -109.964025 | 574724 | 5504448 | 1 | 27.37 | Local |
| M2 | 07/17/2011 0:00 | 12 | 49.687820 | -109.963678 | 574749 | 5504438 | 0 | 127.65 | Local |
| M2 | 07/17/2011 0:00 | 21 | 49.686789 | -109.964456 | 574695 | 5504322 | 1 | 199.31 | Local |
| M2 | 07/18/2011 0:00 | 0 | 49.685902 | -109.966857 | 574523 | 5504221 | 1 | 1164.30 | Local |

| | | | | | | | | | |
|---|---|---|---|---|---|---|---|---|---|
| M2 | 07/18/2011 0:00 | 3 | 49.675636 | -109.970041 | 574309 | 5503077 | 1 | 4144.78 | Local |
| M2 | 07/18/2011 0:00 | 6 | 49.661466 | -110.023166 | 570497 | 5501450 | 1 | 92.41 | Local |
| M2 | 07/18/2011 0:00 | 9 | 49.661934 | -110.024224 | 570420 | 5501501 | 1 | 97.60 | Local |
| M2 | 07/18/2011 0:00 | 12 | 49.661272 | -110.025113 | 570356 | 5501427 | 1 | 219.78 | Local |
| M2 | 07/18/2011 0:00 | 15 | 49.660021 | -110.027471 | 570188 | 5501286 | 1 | 129.60 | Local |
| M2 | 07/18/2011 0:00 | 18 | 49.661063 | -110.026665 | 570245 | 5501402 | 1 | 535.87 | Local |
| M2 | 07/18/2011 0:00 | 21 | 49.665564 | -110.029321 | 570047 | 5501900 | 1 | 3008.55 | Local |
| M2 | 07/19/2011 0:00 | 0 | 49.692464 | -110.024802 | 570334 | 5504895 | 1 | 1695.15 | Local |
| M2 | 07/19/2011 0:00 | 3 | 49.706522 | -110.015704 | 570970 | 5506466 | 1 | 128.27 | Local |
| M2 | 07/19/2011 0:00 | 6 | 49.705461 | -110.016405 | 570921 | 5506348 | 1 | 32.86 | Local |
| M2 | 07/19/2011 0:00 | 9 | 49.705756 | -110.016375 | 570922 | 5506381 | 1 | 75.69 | Local |
| M2 | 07/19/2011 0:00 | 12 | 49.706255 | -110.015660 | 570973 | 5506437 | 1 | 53.22 | Local |
| M2 | 07/19/2011 0:00 | 15 | 49.705974 | -110.016258 | 570930 | 5506405 | 1 | 6.86 | Local |
| M2 | 07/19/2011 0:00 | 18 | 49.705991 | -110.016349 | 570924 | 5506407 | 1 | 45.17 | Local |
| M2 | 07/19/2011 0:00 | 21 | 49.706014 | -110.015724 | 570969 | 5506410 | 1 | 157.89 | Local |
| M2 | 07/20/2011 0:00 | 0 | 49.707238 | -110.014614 | 571047 | 5506547 | 1 | 5.92 | Local |
| M2 | 07/20/2011 0:00 | 3 | 49.707187 | -110.014591 | 571049 | 5506541 | 1 | 15.95 | Local |
| M2 | 07/20/2011 0:00 | 6 | 49.707065 | -110.014706 | 571041 | 5506528 | 1 | 13.82 | Local |
| M2 | 07/20/2011 0:00 | 9 | 49.707176 | -110.014790 | 571034 | 5506540 | 1 | 102.99 | Local |
| M2 | 07/20/2011 0:00 | 12 | 49.708101 | -110.014882 | 571026 | 5506643 | 0 | 56.84 | Local |
| M2 | 07/20/2011 0:00 | 21 | 49.707684 | -110.015338 | 570994 | 5506596 | 1 | 90.34 | Local |
| M2 | 07/21/2011 0:00 | 0 | 49.707115 | -110.014443 | 571060 | 5506533 | 1 | 25.13 | Local |
| M2 | 07/21/2011 0:00 | 3 | 49.707161 | -110.014784 | 571035 | 5506538 | 1 | 8.57 | Local |
| M2 | 07/21/2011 0:00 | 6 | 49.707089 | -110.014743 | 571038 | 5506530 | 0 | 38.82 | Local |
| M2 | 07/21/2011 0:00 | 12 | 49.707437 | -110.014709 | 571040 | 5506569 | 1 | 22.34 | Local |
| M2 | 07/21/2011 0:00 | 15 | 49.707620 | -110.014581 | 571049 | 5506589 | 1 | 56.68 | Local |
| M2 | 07/21/2011 0:00 | 18 | 49.707128 | -110.014788 | 571035 | 5506535 | 1 | 7.54 | Local |
| M2 | 07/21/2011 0:00 | 21 | 49.707144 | -110.014686 | 571042 | 5506536 | 1 | 10.47 | Local |
| M2 | 07/22/2011 0:00 | 0 | 49.707096 | -110.014811 | 571033 | 5506531 | 1 | 21.34 | Local |
| M2 | 07/22/2011 0:00 | 3 | 49.707126 | -110.014519 | 571054 | 5506535 | 1 | 38.08 | Local |
| M2 | 07/22/2011 0:00 | 6 | 49.707458 | -110.014646 | 571044 | 5506571 | 1 | 22.20 | Local |
| M2 | 07/22/2011 0:00 | 9 | 49.707636 | -110.014506 | 571054 | 5506591 | 1 | 536.85 | Local |
| M2 | 07/22/2011 0:00 | 12 | 49.702850 | -110.015483 | 570991 | 5506058 | 1 | 452.40 | Local |
| M2 | 07/22/2011 0:00 | 15 | 49.706891 | -110.014750 | 571038 | 5506508 | 1 | 3.81 | Local |
| M2 | 07/22/2011 0:00 | 18 | 49.706924 | -110.014740 | 571038 | 5506512 | 1 | 27.11 | Local |
| M2 | 07/22/2011 0:00 | 21 | 49.707168 | -110.014723 | 571039 | 5506539 | 1 | 18.00 | Local |
| M2 | 07/23/2011 0:00 | 0 | 49.707054 | -110.014900 | 571027 | 5506526 | 1 | 22.03 | Local |
| M2 | 07/23/2011 0:00 | 3 | 49.707024 | -110.014598 | 571049 | 5506523 | 1 | 4100.78 | Local |
| M2 | 07/23/2011 0:00 | 6 | 49.670471 | -110.022176 | 570555 | 5502452 | 1 | 946.89 | Local |
| M2 | 07/23/2011 0:00 | 9 | 49.664197 | -110.031049 | 569924 | 5501746 | 1 | 380.07 | Local |
| M2 | 07/23/2011 0:00 | 12 | 49.661905 | -110.027143 | 570209 | 5501495 | 0 | 4.08 | Local |
| M2 | 07/23/2011 0:00 | 18 | 49.661917 | -110.027196 | 570205 | 5501497 | 1 | 83.70 | Local |
| M2 | 07/23/2011 0:00 | 21 | 49.661329 | -110.027918 | 570154 | 5501430 | 1 | 1664.02 | Local |
| M2 | 07/24/2011 0:00 | 0 | 49.674130 | -110.015973 | 570997 | 5502865 | 1 | 1581.86 | Local |
| M2 | 07/24/2011 0:00 | 3 | 49.673549 | -110.037877 | 569418 | 5502780 | 1 | 1752.32 | Local |
| M2 | 07/24/2011 0:00 | 6 | 49.674875 | -110.062077 | 567670 | 5502905 | 1 | 11.28 | Local |
| M2 | 07/24/2011 0:00 | 9 | 49.674972 | -110.062031 | 567673 | 5502916 | 1 | 191.05 | Local |
| M2 | 07/24/2011 0:00 | 12 | 49.675540 | -110.064530 | 567492 | 5502977 | 1 | 18.65 | Local |
| M2 | 07/24/2011 0:00 | 15 | 49.675702 | -110.064464 | 567497 | 5502995 | 0 | 220.34 | Local |
| M2 | 07/24/2011 0:00 | 21 | 49.677607 | -110.065305 | 567433 | 5503206 | 1 | 808.23 | Local |
| M2 | 07/25/2011 0:00 | 0 | 49.679562 | -110.054515 | 568209 | 5503433 | 1 | 242.27 | Local |
| M2 | 07/25/2011 0:00 | 3 | 49.677385 | -110.054646 | 568203 | 5503191 | 1 | 153.13 | Local |
| M2 | 07/25/2011 0:00 | 6 | 49.677155 | -110.052553 | 568354 | 5503167 | 1 | 57.69 | Local |
| M2 | 07/25/2011 0:00 | 9 | 49.676637 | -110.052519 | 568357 | 5503110 | 0 | 76.98 | Local |
| M2 | 07/25/2011 0:00 | 18 | 49.676052 | -110.051948 | 568399 | 5503045 | 1 | 9.16 | Local |

| | | | | | | | | | |
|---|---|---|---|---|---|---|---|---|---|
| M2 | 07/25/2011 0:00 | 21 | 49.676062 | -110.051822 | 568408 | 5503046 | 1 | 2035.97 | Local |
| M2 | 07/26/2011 0:00 | 0 | 49.680524 | -110.024453 | 570376 | 5503568 | 1 | 2195.52 | Local |
| M2 | 07/26/2011 0:00 | 3 | 49.698523 | -110.011937 | 571253 | 5505581 | 0 | 809.38 | Local |
| M2 | 07/26/2011 0:00 | 9 | 49.705233 | -110.016289 | 570929 | 5506322 | 1 | 9.89 | Local |
| M2 | 07/26/2011 0:00 | 12 | 49.705170 | -110.016385 | 570922 | 5506315 | 1 | 9.75 | Local |
| M2 | 07/26/2011 0:00 | 15 | 49.705257 | -110.016384 | 570922 | 5506325 | 1 | 5.44 | Local |
| M2 | 07/26/2011 0:00 | 18 | 49.705214 | -110.016349 | 570925 | 5506320 | 1 | 16.48 | Local |
| M2 | 07/26/2011 0:00 | 21 | 49.705085 | -110.016460 | 570917 | 5506306 | 1 | 575.02 | Local |
| M2 | 07/27/2011 0:00 | 0 | 49.699992 | -110.015068 | 571025 | 5505741 | 1 | 1953.65 | Local |
| M2 | 07/27/2011 0:00 | 3 | 49.685145 | -110.000583 | 572091 | 5504104 | 0 | 2355.86 | Local |
| M2 | 07/27/2011 0:00 | 15 | 49.664177 | -109.995889 | 572461 | 5501777 | 1 | 72.48 | Local |
| M2 | 07/27/2011 0:00 | 18 | 49.664734 | -109.995367 | 572498 | 5501840 | 1 | 78.94 | Local |
| M2 | 07/27/2011 0:00 | 21 | 49.664074 | -109.995771 | 572470 | 5501766 | 1 | 1504.09 | Local |
| M2 | 07/28/2011 0:00 | 0 | 49.669438 | -110.014905 | 571081 | 5502344 | 1 | 4371.51 | Local |
| M2 | 07/28/2011 0:00 | 3 | 49.686341 | -110.069610 | 567111 | 5504173 | 1 | 2665.72 | Local |
| M2 | 07/28/2011 0:00 | 6 | 49.674021 | -110.101307 | 564841 | 5502776 | 1 | 19.02 | Local |
| M2 | 07/28/2011 0:00 | 9 | 49.674191 | -110.101278 | 564843 | 5502794 | 1 | 115.38 | Local |
| M2 | 07/28/2011 0:00 | 12 | 49.673626 | -110.099936 | 564940 | 5502733 | 1 | 2.83 | Local |
| M2 | 07/28/2011 0:00 | 15 | 49.673646 | -110.099912 | 564942 | 5502735 | 1 | 5.17 | Local |
| M2 | 07/28/2011 0:00 | 18 | 49.673633 | -110.099844 | 564947 | 5502734 | 1 | 72.56 | Local |
| M2 | 07/28/2011 0:00 | 21 | 49.673695 | -110.100845 | 564874 | 5502740 | 1 | 77.97 | Local |
| M2 | 07/29/2011 0:00 | 0 | 49.674391 | -110.100978 | 564864 | 5502817 | 1 | 42.44 | Local |
| M2 | 07/29/2011 0:00 | 3 | 49.674068 | -110.101291 | 564842 | 5502781 | 1 | 659.59 | Local |
| M2 | 07/29/2011 0:00 | 6 | 49.669592 | -110.107291 | 564415 | 5502278 | 1 | 11.42 | Local |
| M2 | 07/29/2011 0:00 | 9 | 49.669690 | -110.107340 | 564411 | 5502289 | 1 | 4.60 | Local |
| M2 | 07/29/2011 0:00 | 12 | 49.669658 | -110.107300 | 564414 | 5502285 | 1 | 2.82 | Local |
| M2 | 07/29/2011 0:00 | 15 | 49.669657 | -110.107339 | 564411 | 5502285 | 1 | 31.21 | Local |
| M2 | 07/29/2011 0:00 | 18 | 49.669618 | -110.106910 | 564442 | 5502281 | 1 | 170.97 | Local |
| M2 | 07/29/2011 0:00 | 21 | 49.669431 | -110.104559 | 564612 | 5502262 | 1 | 1220.47 | Local |
| M2 | 07/30/2011 0:00 | 0 | 49.661212 | -110.093349 | 565432 | 5501358 | 1 | 2797.20 | Local |
| M2 | 07/30/2011 0:00 | 3 | 49.650034 | -110.128067 | 562941 | 5500086 | 1 | 1856.46 | Local |
| M2 | 07/30/2011 0:00 | 6 | 49.641665 | -110.150318 | 561345 | 5499137 | 1 | 734.79 | Local |
| M2 | 07/30/2011 0:00 | 9 | 49.639789 | -110.160076 | 560643 | 5498921 | 1 | 12.09 | Local |
| M2 | 07/30/2011 0:00 | 12 | 49.639720 | -110.159945 | 560653 | 5498913 | 1 | 6.72 | Local |
| M2 | 07/30/2011 0:00 | 15 | 49.639779 | -110.159966 | 560651 | 5498920 | 1 | 17.68 | Local |
| M2 | 07/30/2011 0:00 | 18 | 49.639935 | -110.160015 | 560647 | 5498937 | 1 | 938.04 | Local |
| M2 | 07/30/2011 0:00 | 21 | 49.643015 | -110.172110 | 559770 | 5499270 | 1 | 3788.72 | Local |
| M2 | 07/31/2011 0:00 | 0 | 49.668350 | -110.207213 | 557206 | 5502059 | 1 | 2022.77 | Local |
| M2 | 07/31/2011 0:00 | 3 | 49.686141 | -110.201354 | 557608 | 5504042 | 1 | 1384.26 | Local |
| M2 | 07/31/2011 0:00 | 6 | 49.691554 | -110.184071 | 558848 | 5504657 | 1 | 172.99 | Local |
| M2 | 07/31/2011 0:00 | 9 | 49.691696 | -110.181683 | 559020 | 5504674 | 1 | 21.51 | Local |
| M2 | 07/31/2011 0:00 | 12 | 49.691713 | -110.181386 | 559042 | 5504677 | 1 | 225.76 | Local |
| M2 | 07/31/2011 0:00 | 15 | 49.689762 | -110.182250 | 558982 | 5504459 | 1 | 14.36 | Local |
| M2 | 07/31/2011 0:00 | 18 | 49.689882 | -110.182176 | 558987 | 5504472 | 1 | 8.67 | Local |
| M2 | 07/31/2011 0:00 | 21 | 49.689812 | -110.182230 | 558983 | 5504464 | 1 | 1685.89 | Local |
| M2 | 08/01/2011 0:00 | 0 | 49.704058 | -110.174225 | 559543 | 5506055 | 1 | 3206.66 | Local |
| M2 | 08/01/2011 0:00 | 3 | 49.697058 | -110.131088 | 562662 | 5505312 | 1 | 1536.27 | Local |
| M2 | 08/01/2011 0:00 | 6 | 49.685805 | -110.118730 | 563568 | 5504071 | 1 | 2143.31 | Local |
| M2 | 08/01/2011 0:00 | 9 | 49.667545 | -110.128252 | 562905 | 5502033 | 1 | 28.73 | Local |
| M2 | 08/01/2011 0:00 | 12 | 49.667343 | -110.128500 | 562887 | 5502010 | 1 | 18.73 | Local |
| M2 | 08/01/2011 0:00 | 15 | 49.667430 | -110.128278 | 562903 | 5502020 | 1 | 44.08 | Local |
| M2 | 08/01/2011 0:00 | 18 | 49.667396 | -110.128887 | 562859 | 5502016 | 1 | 18.04 | Local |
| M2 | 08/01/2011 0:00 | 21 | 49.667543 | -110.128993 | 562852 | 5502032 | 1 | 5479.86 | Local |
| M2 | 08/02/2011 0:00 | 0 | 49.663541 | -110.053310 | 568318 | 5501653 | 1 | 2672.76 | Local |
| M2 | 08/02/2011 0:00 | 3 | 49.678195 | -110.023948 | 570416 | 5503309 | 1 | 1192.87 | Local |

| | | | | | | | | | |
|---|---|---|---|---|---|---|---|---|---|
| M2 | 08/02/2011 0:00 | 6 | 49.668292 | -110.030311 | 569971 | 5502202 | 0 | 28.35 | Local |
| M2 | 08/02/2011 0:00 | 12 | 49.668173 | -110.029964 | 569996 | 5502189 | 1 | 5.86 | Local |
| M2 | 08/02/2011 0:00 | 15 | 49.668152 | -110.030038 | 569991 | 5502187 | 1 | 13.34 | Local |
| M2 | 08/02/2011 0:00 | 18 | 49.668113 | -110.029863 | 570004 | 5502183 | 1 | 7.31 | Local |
| M2 | 08/02/2011 0:00 | 21 | 49.668148 | -110.029777 | 570010 | 5502187 | 1 | 1198.51 | Local |
| M2 | 08/03/2011 0:00 | 0 | 49.678312 | -110.024244 | 570395 | 5503322 | 1 | 25.35 | Local |
| M2 | 08/03/2011 0:00 | 3 | 49.678198 | -110.023940 | 570417 | 5503310 | 1 | 1195.86 | Local |
| M2 | 08/03/2011 0:00 | 6 | 49.668206 | -110.030075 | 569988 | 5502193 | 0 | 6.85 | Local |
| M2 | 08/03/2011 0:00 | 15 | 49.668163 | -110.030007 | 569993 | 5502188 | 1 | 10.11 | Local |
| M2 | 08/03/2011 0:00 | 18 | 49.668196 | -110.030138 | 569984 | 5502192 | 1 | 106.56 | Local |
| M2 | 08/03/2011 0:00 | 21 | 49.668131 | -110.028664 | 570090 | 5502186 | 1 | 1168.08 | Local |
| M2 | 08/04/2011 0:00 | 0 | 49.678170 | -110.023891 | 570420 | 5503306 | 1 | 6.71 | Local |
| M2 | 08/04/2011 0:00 | 3 | 49.678132 | -110.023819 | 570425 | 5503302 | 1 | 1203.50 | Local |
| M2 | 08/04/2011 0:00 | 6 | 49.668101 | -110.030087 | 569988 | 5502181 | 1 | 14.57 | Local |
| M2 | 08/04/2011 0:00 | 9 | 49.668228 | -110.030037 | 569991 | 5502195 | 1 | 25.79 | Local |
| M2 | 08/04/2011 0:00 | 12 | 49.668356 | -110.029739 | 570012 | 5502210 | 0 | 32.11 | Local |
| M2 | 08/04/2011 0:00 | 18 | 49.668124 | -110.030005 | 569994 | 5502184 | 1 | 63.36 | Local |
| M2 | 08/04/2011 0:00 | 21 | 49.668685 | -110.030160 | 569982 | 5502246 | 1 | 1222.17 | Local |
| M2 | 08/05/2011 0:00 | 0 | 49.670916 | -110.013575 | 571175 | 5502510 | 1 | 0.35 | Local |
| M2 | 08/05/2011 0:00 | 3 | 49.670918 | -110.013579 | 571175 | 5502510 | 1 | 581.50 | Local |
| M2 | 08/05/2011 0:00 | 6 | 49.665789 | -110.012003 | 571296 | 5501941 | 0 | 155.43 | Local |
| M2 | 08/05/2011 0:00 | 12 | 49.664810 | -110.013541 | 571186 | 5501831 | 1 | 3.00 | Local |
| M2 | 08/05/2011 0:00 | 15 | 49.664822 | -110.013503 | 571189 | 5501832 | 1 | 31.87 | Local |
| M2 | 08/05/2011 0:00 | 18 | 49.665070 | -110.013282 | 571205 | 5501860 | 1 | 60.24 | Local |
| M2 | 08/05/2011 0:00 | 21 | 49.665534 | -110.012852 | 571235 | 5501912 | 1 | 750.45 | Local |
| M2 | 08/06/2011 0:00 | 0 | 49.672281 | -110.012578 | 571245 | 5502663 | 1 | 4.56 | Local |
| M2 | 08/06/2011 0:00 | 3 | 49.672320 | -110.012601 | 571243 | 5502667 | 1 | 937.28 | Local |
| M2 | 08/06/2011 0:00 | 6 | 49.665640 | -110.020526 | 570681 | 5501917 | 1 | 8.35 | Local |
| M2 | 08/06/2011 0:00 | 9 | 49.665715 | -110.020542 | 570680 | 5501925 | 0 | 3.59 | Local |
| M2 | 08/06/2011 0:00 | 15 | 49.665746 | -110.020557 | 570679 | 5501928 | 1 | 8.54 | Local |
| M2 | 08/06/2011 0:00 | 18 | 49.665669 | -110.020572 | 570678 | 5501920 | 1 | 57.94 | Local |
| M2 | 08/06/2011 0:00 | 21 | 49.665998 | -110.021195 | 570632 | 5501956 | 1 | 771.92 | Local |
| M2 | 08/07/2011 0:00 | 0 | 49.670888 | -110.013601 | 571173 | 5502507 | 1 | 3.42 | Local |
| M2 | 08/07/2011 0:00 | 3 | 49.670918 | -110.013590 | 571174 | 5502510 | 1 | 725.84 | Local |
| M2 | 08/07/2011 0:00 | 6 | 49.665507 | -110.019218 | 570776 | 5501903 | 1 | 74.50 | Local |
| M2 | 08/07/2011 0:00 | 9 | 49.665709 | -110.020203 | 570704 | 5501925 | 1 | 23.13 | Local |
| M2 | 08/07/2011 0:00 | 12 | 49.665532 | -110.020373 | 570692 | 5501905 | 0 | 327.31 | Local |
| M2 | 08/07/2011 0:00 | 18 | 49.662621 | -110.019707 | 570745 | 5501582 | 1 | 214.81 | Local |
| M2 | 08/07/2011 0:00 | 21 | 49.664228 | -110.018055 | 570861 | 5501762 | 1 | 806.61 | Local |
| M2 | 08/08/2011 0:00 | 0 | 49.670879 | -110.013593 | 571174 | 5502506 | 1 | 8.94 | Local |
| M2 | 08/08/2011 0:00 | 3 | 49.670840 | -110.013701 | 571166 | 5502501 | 1 | 950.81 | Local |
| M2 | 08/08/2011 0:00 | 6 | 49.662290 | -110.013430 | 571198 | 5501551 | 0 | 18.91 | Local |
| M2 | 08/08/2011 0:00 | 12 | 49.662442 | -110.013549 | 571189 | 5501568 | 1 | 1.27 | Local |
| M2 | 08/08/2011 0:00 | 15 | 49.662430 | -110.013553 | 571189 | 5501566 | 1 | 3.46 | Local |
| M2 | 08/08/2011 0:00 | 18 | 49.662401 | -110.013570 | 571188 | 5501563 | 1 | 172.28 | Local |
| M2 | 08/08/2011 0:00 | 21 | 49.663946 | -110.013751 | 571172 | 5501735 | 1 | 561.88 | Local |
| M2 | 08/09/2011 0:00 | 0 | 49.668796 | -110.015938 | 571008 | 5502272 | 1 | 290.90 | Local |
| M2 | 08/09/2011 0:00 | 3 | 49.670905 | -110.013553 | 571177 | 5502509 | 1 | 688.15 | Local |
| M2 | 08/09/2011 0:00 | 6 | 49.664716 | -110.013465 | 571192 | 5501821 | 1 | 16.19 | Local |
| M2 | 08/09/2011 0:00 | 9 | 49.664858 | -110.013518 | 571188 | 5501836 | 1 | 5.97 | Local |
| M2 | 08/09/2011 0:00 | 12 | 49.664805 | -110.013530 | 571187 | 5501830 | 0 | 4.07 | Local |
| M2 | 08/09/2011 0:00 | 18 | 49.664840 | -110.013545 | 571186 | 5501834 | 1 | 2.61 | Local |
| M2 | 08/09/2011 0:00 | 21 | 49.664856 | -110.013519 | 571188 | 5501836 | 1 | 699.41 | Local |
| M2 | 08/10/2011 0:00 | 0 | 49.668542 | -110.005666 | 571749 | 5502253 | 1 | 736.88 | Local |
| M2 | 08/10/2011 0:00 | 3 | 49.668214 | -109.995467 | 572486 | 5502227 | 1 | 1730.58 | Local |

| | | | | | | | | | |
|---|---|---|---|---|---|---|---|---|---|
| M2 | 08/10/2011 0:00 | 6 | 49.663341 | -109.972692 | 574136 | 5501707 | 1 | 221.81 | Local |
| M2 | 08/10/2011 0:00 | 9 | 49.661476 | -109.971604 | 574218 | 5501501 | 1 | 95.27 | Local |
| M2 | 08/10/2011 0:00 | 12 | 49.662214 | -109.970936 | 574265 | 5501584 | 0 | 0.63 | Local |
| M2 | 08/10/2011 0:00 | 18 | 49.662213 | -109.970944 | 574264 | 5501583 | 1 | 4.99 | Local |
| M2 | 08/10/2011 0:00 | 21 | 49.662237 | -109.971002 | 574260 | 5501586 | 1 | 2939.24 | Local |
| M2 | 08/11/2011 0:00 | 0 | 49.688589 | -109.974234 | 573987 | 5504513 | 1 | 1478.13 | Local |
| M2 | 08/11/2011 0:00 | 3 | 49.688580 | -109.953743 | 575465 | 5504532 | 1 | 781.33 | Local |
| M2 | 08/11/2011 0:00 | 6 | 49.689365 | -109.964507 | 574687 | 5504608 | 1 | 229.31 | Local |
| M2 | 08/11/2011 0:00 | 9 | 49.687819 | -109.966611 | 574538 | 5504435 | 1 | 51.38 | Local |
| M2 | 08/11/2011 0:00 | 12 | 49.687988 | -109.965948 | 574585 | 5504454 | 1 | 274.68 | Local |
| M2 | 08/11/2011 0:00 | 15 | 49.689103 | -109.969346 | 574339 | 5504575 | 1 | 14.75 | Local |
| M2 | 08/11/2011 0:00 | 18 | 49.689059 | -109.969153 | 574353 | 5504570 | 1 | 130.18 | Local |
| M2 | 08/11/2011 0:00 | 21 | 49.689089 | -109.970957 | 574222 | 5504571 | 1 | 1804.21 | Local |
| M2 | 08/12/2011 0:00 | 0 | 49.685148 | -109.995219 | 572478 | 5504110 | 1 | 1688.16 | Local |
| M2 | 08/12/2011 0:00 | 3 | 49.697112 | -110.009629 | 571421 | 5505426 | 1 | 1066.13 | Local |
| M2 | 08/12/2011 0:00 | 6 | 49.705608 | -110.016483 | 570915 | 5506364 | 1 | 3.07 | Local |
| M2 | 08/12/2011 0:00 | 9 | 49.705599 | -110.016442 | 570918 | 5506363 | 0 | 50.04 | Local |
| M2 | 08/12/2011 0:00 | 15 | 49.705951 | -110.016875 | 570886 | 5506402 | 1 | 31.49 | Local |
| M2 | 08/12/2011 0:00 | 18 | 49.705718 | -110.016627 | 570904 | 5506376 | 1 | 41.44 | Local |
| M2 | 08/12/2011 0:00 | 21 | 49.706091 | -110.016604 | 570905 | 5506417 | 1 | 11.45 | Local |
| M2 | 08/13/2011 0:00 | 0 | 49.706094 | -110.016762 | 570894 | 5506418 | 1 | 179.53 | Local |
| M2 | 08/13/2011 0:00 | 3 | 49.706942 | -110.014643 | 571045 | 5506514 | 1 | 95.34 | Local |
| M2 | 08/13/2011 0:00 | 6 | 49.706288 | -110.015499 | 570985 | 5506440 | 0 | 101.79 | Local |
| M2 | 08/13/2011 0:00 | 18 | 49.705623 | -110.016469 | 570916 | 5506366 | 1 | 15.62 | Local |
| M2 | 08/13/2011 0:00 | 21 | 49.705747 | -110.016568 | 570908 | 5506379 | 1 | 118.09 | Local |
| M2 | 08/14/2011 0:00 | 0 | 49.706483 | -110.015387 | 570992 | 5506462 | 1 | 18.08 | Local |
| M2 | 08/14/2011 0:00 | 3 | 49.706645 | -110.015375 | 570993 | 5506480 | 1 | 72.77 | Local |
| M2 | 08/14/2011 0:00 | 6 | 49.706981 | -110.014508 | 571055 | 5506518 | 0 | 203.51 | Local |
| M2 | 08/14/2011 0:00 | 18 | 49.705754 | -110.016604 | 570906 | 5506380 | 1 | 70.06 | Local |
| M2 | 08/14/2011 0:00 | 21 | 49.705792 | -110.015634 | 570976 | 5506385 | 1 | 179.35 | Local |
| M2 | 08/15/2011 0:00 | 0 | 49.707397 | -110.015385 | 570991 | 5506564 | 1 | 111.33 | Local |
| M2 | 08/15/2011 0:00 | 3 | 49.706421 | -110.015731 | 570968 | 5506455 | 1 | 79.43 | Local |
| M2 | 08/15/2011 0:00 | 6 | 49.706065 | -110.016687 | 570899 | 5506415 | 1 | 102.79 | Local |
| M2 | 08/15/2011 0:00 | 9 | 49.705166 | -110.016357 | 570924 | 5506315 | 0 | 3.98 | Local |
| M2 | 08/15/2011 0:00 | 15 | 49.705185 | -110.016403 | 570921 | 5506317 | 1 | 2.17 | Local |
| M2 | 08/15/2011 0:00 | 18 | 49.705166 | -110.016405 | 570921 | 5506315 | 1 | 135.26 | Local |
| M2 | 08/15/2011 0:00 | 21 | 49.706334 | -110.015878 | 570957 | 5506445 | 1 | 312.19 | Local |
| M2 | 08/16/2011 0:00 | 0 | 49.709057 | -110.014824 | 571029 | 5506749 | 1 | 115.29 | Local |
| M2 | 08/16/2011 0:00 | 3 | 49.708048 | -110.014456 | 571057 | 5506637 | 1 | 335.55 | Local |
| M2 | 08/16/2011 0:00 | 6 | 49.705281 | -110.016313 | 570927 | 5506328 | 1 | 27.62 | Local |
| M2 | 08/16/2011 0:00 | 9 | 49.705051 | -110.016460 | 570917 | 5506302 | 1 | 14.70 | Local |
| M2 | 08/16/2011 0:00 | 12 | 49.705177 | -110.016399 | 570921 | 5506316 | 0 | 3.85 | Local |
| M2 | 08/16/2011 0:00 | 18 | 49.705147 | -110.016375 | 570923 | 5506313 | 1 | 333.45 | Local |
| M2 | 08/16/2011 0:00 | 21 | 49.702168 | -110.015835 | 570966 | 5505982 | 1 | 1732.72 | Local |
| M2 | 08/17/2011 0:00 | 0 | 49.688346 | -110.004739 | 571787 | 5504456 | 1 | 1548.27 | Local |
| M2 | 08/17/2011 0:00 | 3 | 49.683195 | -109.984799 | 573233 | 5503903 | 1 | 3066.65 | Local |
| M2 | 08/17/2011 0:00 | 6 | 49.660989 | -110.010008 | 571447 | 5501410 | 1 | 318.24 | Local |
| M2 | 08/17/2011 0:00 | 9 | 49.658254 | -110.008706 | 571545 | 5501107 | 1 | 121.42 | Local |
| M2 | 08/17/2011 0:00 | 12 | 49.659344 | -110.008820 | 571535 | 5501228 | 0 | 268.93 | Local |
| M2 | 08/17/2011 0:00 | 18 | 49.657326 | -110.006766 | 571686 | 5501005 | 1 | 1242.26 | Local |
| M2 | 08/17/2011 0:00 | 21 | 49.646532 | -110.011210 | 571381 | 5499801 | 1 | 646.75 | Local |
| M2 | 08/18/2011 0:00 | 0 | 49.651369 | -110.016188 | 571015 | 5500334 | 1 | 563.03 | Local |
| M2 | 08/18/2011 0:00 | 3 | 49.646367 | -110.014963 | 571111 | 5499779 | 1 | 91.97 | Local |
| M2 | 08/18/2011 0:00 | 6 | 49.646759 | -110.013841 | 571191 | 5499824 | 1 | 82.23 | Local |
| M2 | 08/18/2011 0:00 | 9 | 49.646405 | -110.014841 | 571119 | 5499784 | 1 | 1.54 | Local |

| | | | | | | | | | |
|---|---|---|---|---|---|---|---|---|---|
| M2 | 08/18/2011 0:00 | 12 | 49.646405 | -110.014820 | 571121 | 5499784 | 0 | 113.44 | Local |
| M2 | 08/18/2011 0:00 | 18 | 49.645771 | -110.013588 | 571211 | 5499714 | 1 | 220.97 | Local |
| M2 | 08/18/2011 0:00 | 21 | 49.644914 | -110.010827 | 571411 | 5499622 | 1 | 799.22 | Local |
| M2 | 08/19/2011 0:00 | 0 | 49.651114 | -110.016429 | 570998 | 5500306 | 1 | 1535.57 | Local |
| M2 | 08/19/2011 0:00 | 3 | 49.663980 | -110.024160 | 570421 | 5501729 | 1 | 1338.71 | Local |
| M2 | 08/19/2011 0:00 | 6 | 49.654102 | -110.034764 | 569670 | 5500621 | 1 | 1825.49 | Local |
| M2 | 08/19/2011 0:00 | 9 | 49.647110 | -110.057644 | 568028 | 5499822 | 1 | 27.59 | Local |
| M2 | 08/19/2011 0:00 | 12 | 49.647132 | -110.058025 | 568001 | 5499824 | 1 | 9.66 | Local |
| M2 | 08/19/2011 0:00 | 15 | 49.647098 | -110.057902 | 568010 | 5499821 | 1 | 502.59 | Local |
| M2 | 08/19/2011 0:00 | 18 | 49.645321 | -110.064302 | 567550 | 5499617 | 0 | 6490.77 | Local |
| M2 | 08/20/2011 0:00 | 3 | 49.687991 | -110.002921 | 571919 | 5504418 | 1 | 2088.56 | Local |
| M2 | 08/20/2011 0:00 | 6 | 49.704746 | -110.016014 | 570950 | 5506269 | 1 | 47.78 | Local |
| M2 | 08/20/2011 0:00 | 9 | 49.705114 | -110.016357 | 570925 | 5506309 | 0 | 3.64 | Local |
| M2 | 08/20/2011 0:00 | 18 | 49.705146 | -110.016353 | 570925 | 5506313 | 1 | 61.17 | Local |
| M2 | 08/20/2011 0:00 | 21 | 49.704896 | -110.015598 | 570980 | 5506286 | 1 | 209.18 | Local |
| M2 | 08/21/2011 0:00 | 0 | 49.706755 | -110.015148 | 571009 | 5506493 | 1 | 5.25 | Local |
| M2 | 08/21/2011 0:00 | 3 | 49.706802 | -110.015152 | 571009 | 5506498 | 1 | 108.32 | Local |
| M2 | 08/21/2011 0:00 | 6 | 49.705846 | -110.015438 | 570990 | 5506391 | 1 | 17.85 | Local |
| M2 | 08/21/2011 0:00 | 9 | 49.705707 | -110.015563 | 570981 | 5506376 | 1 | 63.31 | Local |
| M2 | 08/21/2011 0:00 | 12 | 49.705574 | -110.016416 | 570920 | 5506360 | 1 | 9.77 | Local |
| M2 | 08/21/2011 0:00 | 15 | 49.705662 | -110.016421 | 570919 | 5506370 | 1 | 7.66 | Local |
| M2 | 08/21/2011 0:00 | 18 | 49.705593 | -110.016415 | 570920 | 5506362 | 1 | 75.00 | Local |
| M2 | 08/21/2011 0:00 | 21 | 49.705102 | -110.015702 | 570972 | 5506308 | 1 | 1637.47 | Local |
| M2 | 08/22/2011 0:00 | 0 | 49.697193 | -110.034856 | 569602 | 5505411 | 1 | 1786.55 | Local |
| M2 | 08/22/2011 0:00 | 3 | 49.684019 | -110.049038 | 568598 | 5503934 | 1 | 730.02 | Local |
| M2 | 08/22/2011 0:00 | 6 | 49.677454 | -110.049087 | 568604 | 5503204 | 1 | 254.23 | Local |
| M2 | 08/22/2011 0:00 | 9 | 49.676068 | -110.051890 | 568403 | 5503047 | 1 | 1.13 | Local |
| M2 | 08/22/2011 0:00 | 12 | 49.676077 | -110.051898 | 568403 | 5503048 | 1 | 2.48 | Local |
| M2 | 08/22/2011 0:00 | 15 | 49.676099 | -110.051894 | 568403 | 5503050 | 1 | 7.26 | Local |
| M2 | 08/22/2011 0:00 | 18 | 49.676060 | -110.051975 | 568397 | 5503046 | 1 | 58.96 | Local |
| M2 | 08/22/2011 0:00 | 21 | 49.676350 | -110.052658 | 568347 | 5503078 | 1 | 771.91 | Local |
| M2 | 08/23/2011 0:00 | 0 | 49.677833 | -110.042206 | 569099 | 5503252 | 1 | 1361.11 | Local |
| M2 | 08/23/2011 0:00 | 3 | 49.667740 | -110.031531 | 569884 | 5502140 | 1 | 1534.08 | Local |
| M2 | 08/23/2011 0:00 | 6 | 49.653950 | -110.032249 | 569852 | 5500606 | 1 | 1090.13 | Local |
| M2 | 08/23/2011 0:00 | 9 | 49.646367 | -110.041821 | 569172 | 5499754 | 1 | 55.71 | Local |
| M2 | 08/23/2011 0:00 | 12 | 49.646850 | -110.041616 | 569186 | 5499808 | 0 | 12.48 | Local |
| M2 | 08/23/2011 0:00 | 18 | 49.646748 | -110.041544 | 569191 | 5499797 | 1 | 786.04 | Local |
| M2 | 08/23/2011 0:00 | 21 | 49.643009 | -110.032304 | 569864 | 5499390 | 1 | 1636.42 | Local |
| M2 | 08/24/2011 0:00 | 0 | 49.635265 | -110.051577 | 568483 | 5498511 | 0 | 1588.71 | Local |
| M2 | 08/24/2011 0:00 | 9 | 49.634574 | -110.073552 | 566897 | 5498414 | 1 | 193.67 | Local |
| M2 | 08/24/2011 0:00 | 12 | 49.636299 | -110.073181 | 566922 | 5498606 | 0 | 9.97 | Local |
| M2 | 08/24/2011 0:00 | 18 | 49.636216 | -110.073232 | 566918 | 5498597 | 1 | 188.56 | Local |
| M2 | 08/24/2011 0:00 | 21 | 49.637637 | -110.074657 | 566813 | 5498754 | 1 | 1164.20 | Local |
| M2 | 08/25/2011 0:00 | 0 | 49.647511 | -110.069293 | 567187 | 5499856 | 1 | 2438.15 | Local |
| M2 | 08/25/2011 0:00 | 3 | 49.662949 | -110.045304 | 568897 | 5501594 | 1 | 5051.31 | Local |
| M2 | 08/25/2011 0:00 | 6 | 49.704029 | -110.015399 | 570995 | 5506189 | 1 | 132.57 | Local |
| M2 | 08/25/2011 0:00 | 9 | 49.705006 | -110.016453 | 570918 | 5506297 | 0 | 32.92 | Local |
| M2 | 08/25/2011 0:00 | 15 | 49.705283 | -110.016291 | 570929 | 5506328 | 1 | 13.92 | Local |
| M2 | 08/25/2011 0:00 | 18 | 49.705170 | -110.016375 | 570923 | 5506315 | 1 | 40.22 | Local |
| M2 | 08/25/2011 0:00 | 21 | 49.705170 | -110.015817 | 570963 | 5506316 | 1 | 1009.87 | Local |
| M2 | 08/26/2011 0:00 | 0 | 49.714210 | -110.014460 | 571048 | 5507322 | 1 | 164.55 | Local |
| M2 | 08/26/2011 0:00 | 3 | 49.715396 | -110.013094 | 571145 | 5507455 | 1 | 827.71 | Local |
| M2 | 08/26/2011 0:00 | 6 | 49.708003 | -110.014442 | 571058 | 5506632 | 0 | 242.28 | Local |
| M2 | 08/26/2011 0:00 | 12 | 49.706088 | -110.016046 | 570945 | 5506418 | 0 | 163.74 | Local |
| M2 | 08/26/2011 0:00 | 18 | 49.707120 | -110.014426 | 571061 | 5506534 | 1 | 78.31 | Local |

| | | | | | | | | | |
|---|---|---|---|---|---|---|---|---|---|
| M2 | 08/26/2011 0:00 | 21 | 49.707824 | -110.014389 | 571062 | 5506612 | 1 | 3741.92 | Local |
| M2 | 08/27/2011 0:00 | 0 | 49.740909 | -110.023895 | 570329 | 5510282 | 1 | 3674.96 | Local |
| M2 | 08/27/2011 0:00 | 3 | 49.730876 | -110.072486 | 566842 | 5509122 | 1 | 535.43 | Local |
| M2 | 08/27/2011 0:00 | 6 | 49.728522 | -110.078966 | 566378 | 5508854 | 1 | 4097.35 | Local |
| M2 | 08/27/2011 0:00 | 9 | 49.695327 | -110.103647 | 564644 | 5505142 | 1 | 11.39 | Local |
| M2 | 08/27/2011 0:00 | 12 | 49.695425 | -110.103690 | 564640 | 5505153 | 0 | 15.70 | Local |
| M2 | 08/27/2011 0:00 | 18 | 49.695287 | -110.103732 | 564638 | 5505138 | 1 | 165.44 | Local |
| M2 | 08/27/2011 0:00 | 21 | 49.694277 | -110.105416 | 564517 | 5505024 | 1 | 2507.29 | Local |
| M2 | 08/28/2011 0:00 | 0 | 49.708607 | -110.078570 | 566434 | 5506641 | 1 | 3413.01 | Local |
| M2 | 08/28/2011 0:00 | 3 | 49.734772 | -110.053810 | 568182 | 5509572 | 1 | 2704.63 | Local |
| M2 | 08/28/2011 0:00 | 6 | 49.728058 | -110.089881 | 565592 | 5508793 | 0 | 35.71 | Local |
| M2 | 08/28/2011 0:00 | 18 | 49.728377 | -110.089936 | 565588 | 5508829 | 1 | 53.92 | Local |
| M2 | 08/28/2011 0:00 | 21 | 49.728811 | -110.090271 | 565563 | 5508876 | 1 | 3193.22 | Local |
| M2 | 08/29/2011 0:00 | 0 | 49.706883 | -110.118876 | 563530 | 5506414 | 1 | 2523.23 | Local |
| M2 | 08/29/2011 0:00 | 3 | 49.684212 | -110.120482 | 563444 | 5503892 | 1 | 1660.67 | Local |
| M2 | 08/29/2011 0:00 | 6 | 49.677251 | -110.140848 | 561984 | 5503101 | 1 | 2655.49 | Local |
| M2 | 08/29/2011 0:00 | 9 | 49.653773 | -110.147596 | 561527 | 5500486 | 1 | 286.93 | Local |
| M2 | 08/29/2011 0:00 | 12 | 49.651445 | -110.149313 | 561406 | 5500225 | 0 | 4093.45 | Local |
| M2 | 08/30/2011 0:00 | 0 | 49.687952 | -110.141962 | 561890 | 5504290 | 1 | 796.00 | Local |
| M2 | 08/30/2011 0:00 | 3 | 49.694186 | -110.136535 | 562273 | 5504988 | 1 | 84.40 | Local |
| M2 | 08/30/2011 0:00 | 6 | 49.693430 | -110.136431 | 562282 | 5504904 | 1 | 96.11 | Local |
| M2 | 08/30/2011 0:00 | 9 | 49.692565 | -110.136438 | 562282 | 5504808 | 1 | 11.03 | Local |
| M2 | 08/30/2011 0:00 | 12 | 49.692656 | -110.136499 | 562278 | 5504818 | 0 | 14.24 | Local |
| M2 | 08/30/2011 0:00 | 18 | 49.692536 | -110.136434 | 562283 | 5504804 | 1 | 195.03 | Local |
| M2 | 08/30/2011 0:00 | 21 | 49.693949 | -110.134833 | 562396 | 5504963 | 1 | 130.62 | Local |
| M2 | 08/31/2011 0:00 | 0 | 49.694387 | -110.136513 | 562275 | 5505010 | 1 | 15.03 | Local |
| M2 | 08/31/2011 0:00 | 3 | 49.694252 | -110.136526 | 562274 | 5504995 | 1 | 351.67 | Local |
| M2 | 08/31/2011 0:00 | 6 | 49.691093 | -110.136284 | 562295 | 5504644 | 1 | 359.26 | Local |
| M2 | 08/31/2011 0:00 | 9 | 49.688225 | -110.138578 | 562134 | 5504323 | 1 | 194.03 | Local |
| M2 | 08/31/2011 0:00 | 12 | 49.689943 | -110.139047 | 562098 | 5504514 | 1 | 18.15 | Local |
| M2 | 08/31/2011 0:00 | 15 | 49.690089 | -110.138933 | 562106 | 5504530 | 1 | 148.49 | Local |
| M2 | 08/31/2011 0:00 | 18 | 49.691250 | -110.137915 | 562178 | 5504660 | 1 | 337.63 | Local |
| M2 | 08/31/2011 0:00 | 21 | 49.694158 | -110.136566 | 562271 | 5504984 | 1 | 35.44 | Local |
| M2 | 09/01/2011 0:00 | 0 | 49.693921 | -110.136236 | 562295 | 5504958 | 1 | 31.68 | Local |
| M2 | 09/01/2011 0:00 | 3 | 49.694134 | -110.136529 | 562274 | 5504982 | 1 | 27.13 | Local |
| M2 | 09/01/2011 0:00 | 6 | 49.693970 | -110.136250 | 562294 | 5504964 | 1 | 55.81 | Local |
| M2 | 09/01/2011 0:00 | 9 | 49.694107 | -110.135506 | 562348 | 5504980 | 1 | 3.01 | Local |
| M2 | 09/01/2011 0:00 | 12 | 49.694080 | -110.135504 | 562348 | 5504977 | 1 | 4.98 | Local |
| M2 | 09/01/2011 0:00 | 15 | 49.694116 | -110.135461 | 562351 | 5504981 | 1 | 15.39 | Local |
| M2 | 09/01/2011 0:00 | 18 | 49.694081 | -110.135254 | 562366 | 5504977 | 1 | 92.24 | Local |
| M2 | 09/01/2011 0:00 | 21 | 49.694269 | -110.136500 | 562276 | 5504997 | 1 | 51.85 | Local |
| M2 | 09/02/2011 0:00 | 0 | 49.694708 | -110.136258 | 562293 | 5505046 | 1 | 68.10 | Local |
| M2 | 09/02/2011 0:00 | 3 | 49.694124 | -110.136543 | 562273 | 5504981 | 1 | 288.75 | Local |
| M2 | 09/02/2011 0:00 | 6 | 49.691872 | -110.138538 | 562132 | 5504729 | 1 | 641.38 | Local |
| M2 | 09/02/2011 0:00 | 9 | 49.686813 | -110.142810 | 561830 | 5504163 | 1 | 20.38 | Local |
| M2 | 09/02/2011 0:00 | 12 | 49.686708 | -110.142579 | 561847 | 5504151 | 1 | 102.11 | Local |
| M2 | 09/02/2011 0:00 | 15 | 49.686616 | -110.143987 | 561745 | 5504140 | 1 | 10.08 | Local |
| M2 | 09/02/2011 0:00 | 18 | 49.686617 | -110.143848 | 561755 | 5504140 | 1 | 258.88 | Local |
| M2 | 09/02/2011 0:00 | 21 | 49.688908 | -110.144490 | 561706 | 5504394 | 1 | 1589.42 | Local |
| M2 | 09/03/2011 0:00 | 0 | 49.700534 | -110.131666 | 562616 | 5505697 | 1 | 832.54 | Local |
| M2 | 09/03/2011 0:00 | 3 | 49.693482 | -110.127785 | 562905 | 5504917 | 1 | 1214.06 | Local |
| M2 | 09/03/2011 0:00 | 6 | 49.686731 | -110.141013 | 561960 | 5504155 | 0 | 2735.07 | Local |
| M2 | 09/03/2011 0:00 | 12 | 49.672692 | -110.172145 | 559731 | 5502569 | 1 | 818.92 | Local |
| M2 | 09/03/2011 0:00 | 15 | 49.679117 | -110.166593 | 560124 | 5503288 | 1 | 15.80 | Local |
| M2 | 09/03/2011 0:00 | 18 | 49.678982 | -110.166525 | 560129 | 5503273 | 1 | 529.49 | Local |

| | | | | | | | | | | |
|---|---|---|---|---|---|---|---|---|---|---|
| M2 | 09/03/2011 0:00 | 21 | 49.677201 | -110.159719 | 560622 | 5503080 | | 1 | 912.47 | Local |
| M2 | 09/04/2011 0:00 | 0 | 49.674162 | -110.171466 | 559779 | 5502733 | | 1 | 35.71 | Local |
| M2 | 09/04/2011 0:00 | 3 | 49.674311 | -110.171905 | 559747 | 5502749 | | 1 | 12.39 | Local |
| M2 | 09/04/2011 0:00 | 6 | 49.674201 | -110.171877 | 559749 | 5502737 | | 1 | 29.70 | Local |
| M2 | 09/04/2011 0:00 | 9 | 49.674463 | -110.171958 | 559743 | 5502766 | | 1 | 11.62 | Local |
| M2 | 09/04/2011 0:00 | 12 | 49.674391 | -110.171841 | 559751 | 5502758 | | 1 | 6.08 | Local |
| M2 | 09/04/2011 0:00 | 15 | 49.674368 | -110.171917 | 559746 | 5502756 | | 1 | 8.01 | Local |
| M2 | 09/04/2011 0:00 | 18 | 49.674296 | -110.171913 | 559746 | 5502748 | | 1 | 441.46 | Local |
| M2 | 09/04/2011 0:00 | 21 | 49.677044 | -110.176330 | 559424 | 5503050 | | 1 | 660.76 | Local |
| M2 | 09/05/2011 0:00 | 0 | 49.671512 | -110.179677 | 559189 | 5502432 | | 1 | 5.21 | Local |
| M2 | 09/05/2011 0:00 | 3 | 49.671469 | -110.179646 | 559192 | 5502427 | | 0 | 809.77 | Local |
| M2 | 09/05/2011 0:00 | 9 | 49.664364 | -110.182112 | 559022 | 5501635 | | 1 | 62.93 | Local |
| M2 | 09/05/2011 0:00 | 12 | 49.664909 | -110.181879 | 559039 | 5501696 | | 1 | 50.31 | Local |
| M2 | 09/05/2011 0:00 | 15 | 49.664475 | -110.182074 | 559025 | 5501648 | | 1 | 9.05 | Local |
| M2 | 09/05/2011 0:00 | 18 | 49.664434 | -110.181966 | 559033 | 5501643 | | 1 | 443.88 | Local |
| M2 | 09/05/2011 0:00 | 21 | 49.668123 | -110.179613 | 559198 | 5502055 | | 1 | 400.27 | Local |
| M2 | 09/06/2011 0:00 | 0 | 49.671722 | -110.179751 | 559184 | 5502455 | | 1 | 15.33 | Local |
| M2 | 09/06/2011 0:00 | 3 | 49.671590 | -110.179814 | 559179 | 5502441 | | 1 | 1260.83 | Local |
| M2 | 09/06/2011 0:00 | 6 | 49.664467 | -110.193409 | 558207 | 5501638 | | 1 | 3300.48 | Local |
| M2 | 09/06/2011 0:00 | 9 | 49.641253 | -110.164911 | 560292 | 5499080 | | 0 | 5.03 | Local |
| M2 | 09/06/2011 0:00 | 15 | 49.641252 | -110.164841 | 560297 | 5499080 | | 1 | 9.54 | Local |
| M2 | 09/06/2011 0:00 | 18 | 49.641266 | -110.164711 | 560307 | 5499081 | | 1 | 1166.04 | Local |
| M2 | 09/06/2011 0:00 | 21 | 49.630802 | -110.165804 | 560241 | 5497917 | | 1 | 2968.56 | Local |
| M2 | 09/07/2011 0:00 | 0 | 49.628077 | -110.124914 | 563197 | 5497648 | | 1 | 207.00 | Local |
| M2 | 09/07/2011 0:00 | 3 | 49.629406 | -110.122908 | 563340 | 5497797 | | 1 | 2173.51 | Local |
| M2 | 09/07/2011 0:00 | 6 | 49.620122 | -110.096426 | 565265 | 5496788 | | 1 | 2459.78 | Local |
| M2 | 09/07/2011 0:00 | 9 | 49.636300 | -110.073196 | 566921 | 5498607 | | 1 | 8.61 | Local |
| M2 | 09/07/2011 0:00 | 12 | 49.636343 | -110.073097 | 566928 | 5498611 | | 1 | 3.42 | Local |
| M2 | 09/07/2011 0:00 | 15 | 49.636345 | -110.073144 | 566924 | 5498612 | | 1 | 32.03 | Local |
| M2 | 09/07/2011 0:00 | 18 | 49.636322 | -110.073587 | 566892 | 5498609 | | 1 | 36.96 | Local |
| M2 | 09/07/2011 0:00 | 21 | 49.636612 | -110.073337 | 566910 | 5498641 | | 0 | 31.53 | Local |
| M2 | 09/08/2011 0:00 | 3 | 49.636354 | -110.073519 | 566897 | 5498612 | | 1 | 22.37 | Local |
| M2 | 09/08/2011 0:00 | 6 | 49.636352 | -110.073829 | 566875 | 5498612 | | 1 | 26.99 | Local |
| M2 | 09/08/2011 0:00 | 9 | 49.636445 | -110.073484 | 566900 | 5498622 | | 0 | 55.82 | Local |
| M2 | 09/08/2011 0:00 | 18 | 49.636670 | -110.074175 | 566849 | 5498647 | | 1 | 45.37 | Local |
| M2 | 09/08/2011 0:00 | 21 | 49.636477 | -110.073621 | 566890 | 5498626 | | 1 | 8.94 | Local |
| M2 | 09/09/2011 0:00 | 0 | 49.636402 | -110.073578 | 566893 | 5498618 | | 1 | 5.72 | Local |
| M2 | 09/09/2011 0:00 | 3 | 49.636452 | -110.073564 | 566894 | 5498623 | | 1 | 9.67 | Local |
| M2 | 09/09/2011 0:00 | 6 | 49.636408 | -110.073449 | 566902 | 5498618 | | 1 | 7.94 | Local |
| M2 | 09/09/2011 0:00 | 9 | 49.636340 | -110.073485 | 566900 | 5498611 | | 0 | 2.19 | Local |
| M2 | 09/09/2011 0:00 | 15 | 49.636359 | -110.073494 | 566899 | 5498613 | | 1 | 8.30 | Local |
| M2 | 09/09/2011 0:00 | 18 | 49.636424 | -110.073439 | 566903 | 5498620 | | 0 | 14.65 | Local |
| M2 | 09/10/2011 0:00 | 0 | 49.636354 | -110.073610 | 566891 | 5498612 | | 1 | 10.09 | Local |
| M2 | 09/10/2011 0:00 | 3 | 49.636431 | -110.073685 | 566885 | 5498621 | | 1 | 24.04 | Local |
| M2 | 09/10/2011 0:00 | 6 | 49.636584 | -110.073450 | 566902 | 5498638 | | 1 | 136.19 | Local |
| M2 | 09/10/2011 0:00 | 9 | 49.635907 | -110.075021 | 566789 | 5498561 | | 1 | 121.55 | Local |
| M2 | 09/10/2011 0:00 | 12 | 49.636397 | -110.073517 | 566897 | 5498617 | | 1 | 39.21 | Local |
| M2 | 09/10/2011 0:00 | 15 | 49.636364 | -110.072976 | 566936 | 5498614 | | 1 | 9.47 | Local |
| M2 | 09/10/2011 0:00 | 18 | 49.636408 | -110.073088 | 566928 | 5498619 | | 1 | 59.34 | Local |
| M2 | 09/10/2011 0:00 | 21 | 49.636646 | -110.073824 | 566875 | 5498644 | | 0 | 28.34 | Local |
| M2 | 09/11/2011 0:00 | 3 | 49.636391 | -110.073823 | 566875 | 5498616 | | 1 | 14.80 | Local |
| M2 | 09/11/2011 0:00 | 6 | 49.636511 | -110.073734 | 566881 | 5498630 | | 1 | 28.02 | Local |
| M2 | 09/11/2011 0:00 | 9 | 49.636405 | -110.073382 | 566907 | 5498618 | | 1 | 6.78 | Local |
| M2 | 09/11/2011 0:00 | 12 | 49.636421 | -110.073472 | 566900 | 5498620 | | 0 | 16.38 | Local |
| M2 | 09/11/2011 0:00 | 18 | 49.636568 | -110.073464 | 566901 | 5498636 | | 1 | 18.12 | Local |

| | | | | | | | | | |
|---|---|---|---|---|---|---|---|---|---|
| M2 | 09/11/2011 0:00 | 21 | 49.636408 | -110.073506 | 566898 | 5498618 | 1 | 4.61 | Local |
| M2 | 09/12/2011 0:00 | 0 | 49.636383 | -110.073557 | 566894 | 5498615 | 1 | 40.49 | Local |
| M2 | 09/12/2011 0:00 | 3 | 49.636540 | -110.074062 | 566858 | 5498633 | 1 | 29.28 | Local |
| M2 | 09/12/2011 0:00 | 6 | 49.636282 | -110.073981 | 566864 | 5498604 | 1 | 29.45 | Local |
| M2 | 09/12/2011 0:00 | 9 | 49.636366 | -110.073594 | 566892 | 5498614 | 1 | 13.98 | Local |
| M2 | 09/12/2011 0:00 | 12 | 49.636451 | -110.073452 | 566902 | 5498623 | 1 | 14.16 | Local |
| M2 | 09/12/2011 0:00 | 15 | 49.636338 | -110.073362 | 566909 | 5498611 | 1 | 20.28 | Local |
| M2 | 09/12/2011 0:00 | 18 | 49.636326 | -110.073082 | 566929 | 5498610 | 1 | 46.15 | Local |
| M2 | 09/12/2011 0:00 | 21 | 49.636433 | -110.073699 | 566884 | 5498621 | 0 | 1234.90 | Local |
| M2 | 09/13/2011 0:00 | 3 | 49.641898 | -110.088588 | 565802 | 5499215 | 1 | 806.08 | Local |
| M2 | 09/13/2011 0:00 | 6 | 49.649064 | -110.086901 | 565914 | 5500014 | 1 | 1176.53 | Local |
| M2 | 09/13/2011 0:00 | 9 | 49.650790 | -110.102980 | 564751 | 5500191 | 1 | 1100.90 | Local |
| M2 | 09/13/2011 0:00 | 12 | 49.640893 | -110.103471 | 564729 | 5499091 | 1 | 8.55 | Local |
| M2 | 09/13/2011 0:00 | 15 | 49.640820 | -110.103506 | 564726 | 5499083 | 1 | 13.82 | Local |
| M2 | 09/13/2011 0:00 | 18 | 49.640925 | -110.103607 | 564719 | 5499094 | 1 | 654.78 | Local |
| M2 | 09/13/2011 0:00 | 21 | 49.642090 | -110.094718 | 565359 | 5499231 | 1 | 161.42 | Local |
| M2 | 09/14/2011 0:00 | 0 | 49.641396 | -110.092754 | 565502 | 5499156 | 1 | 96.76 | Local |
| M2 | 09/14/2011 0:00 | 3 | 49.642080 | -110.093583 | 565441 | 5499231 | 0 | 212.75 | Local |
| M2 | 09/14/2011 0:00 | 9 | 49.641693 | -110.096469 | 565233 | 5499186 | 1 | 5.69 | Local |
| M2 | 09/14/2011 0:00 | 12 | 49.641730 | -110.096414 | 565237 | 5499190 | 1 | 1192.93 | Local |
| M2 | 09/14/2011 0:00 | 15 | 49.631054 | -110.098069 | 565132 | 5498002 | 1 | 6.92 | Local |
| M2 | 09/14/2011 0:00 | 18 | 49.631037 | -110.097977 | 565138 | 5498000 | 1 | 2432.62 | Local |
| M2 | 09/14/2011 0:00 | 21 | 49.613231 | -110.117547 | 563748 | 5496003 | 1 | 945.68 | Local |
| M2 | 09/15/2011 0:00 | 0 | 49.609693 | -110.129451 | 562893 | 5495600 | 1 | 1038.73 | Local |
| M2 | 09/15/2011 0:00 | 3 | 49.615688 | -110.140478 | 562089 | 5496257 | 1 | 1042.28 | Local |
| M2 | 09/15/2011 0:00 | 6 | 49.615077 | -110.126081 | 563129 | 5496201 | 1 | 1780.48 | Local |
| M2 | 09/15/2011 0:00 | 9 | 49.612127 | -110.101857 | 564883 | 5495894 | 1 | 4.74 | Local |
| M2 | 09/15/2011 0:00 | 12 | 49.612120 | -110.101793 | 564888 | 5495893 | 1 | 77.10 | Local |
| M2 | 09/15/2011 0:00 | 15 | 49.612513 | -110.102672 | 564824 | 5495936 | 1 | 49.37 | Local |
| M2 | 09/15/2011 0:00 | 18 | 49.612773 | -110.102117 | 564864 | 5495966 | 1 | 1258.06 | Local |
| M2 | 09/15/2011 0:00 | 21 | 49.617105 | -110.086029 | 566020 | 5496461 | 1 | 2461.24 | Local |
| M2 | 09/16/2011 0:00 | 0 | 49.620290 | -110.052312 | 568451 | 5496845 | 1 | 689.52 | Local |
| M2 | 09/16/2011 0:00 | 3 | 49.619003 | -110.061649 | 567778 | 5496694 | 1 | 927.03 | Local |
| M2 | 09/16/2011 0:00 | 6 | 49.610929 | -110.064848 | 567558 | 5495793 | 1 | 1448.17 | Local |
| M2 | 09/16/2011 0:00 | 9 | 49.611788 | -110.044847 | 569002 | 5495907 | 0 | 722.14 | Local |
| M2 | 09/16/2011 0:00 | 15 | 49.612230 | -110.034874 | 569722 | 5495965 | 1 | 26.55 | Local |
| M2 | 09/16/2011 0:00 | 18 | 49.612469 | -110.034860 | 569723 | 5495992 | 1 | 1205.31 | Local |
| M2 | 09/16/2011 0:00 | 21 | 49.623246 | -110.033058 | 569837 | 5497192 | 1 | 2148.49 | Local |
| M2 | 09/17/2011 0:00 | 0 | 49.641296 | -110.022435 | 570579 | 5499208 | 1 | 2756.22 | Local |
| M2 | 09/17/2011 0:00 | 3 | 49.665949 | -110.026436 | 570254 | 5501945 | 1 | 4.27 | Local |
| M2 | 09/17/2011 0:00 | 6 | 49.665978 | -110.026474 | 570251 | 5501949 | 1 | 1121.66 | Local |
| M2 | 09/17/2011 0:00 | 9 | 49.666979 | -110.041940 | 569134 | 5502046 | 1 | 220.10 | Local |
| M2 | 09/17/2011 0:00 | 12 | 49.668947 | -110.042280 | 569107 | 5502264 | 1 | 9.74 | Local |
| M2 | 09/17/2011 0:00 | 15 | 49.669033 | -110.042302 | 569105 | 5502274 | 1 | 1.22 | Local |
| M2 | 09/17/2011 0:00 | 18 | 49.669044 | -110.042304 | 569105 | 5502275 | 1 | 971.19 | Local |
| M2 | 09/17/2011 0:00 | 21 | 49.667834 | -110.028975 | 570068 | 5502153 | 1 | 1912.33 | Local |
| M2 | 09/18/2011 0:00 | 0 | 49.677783 | -110.050594 | 568494 | 5503239 | 1 | 1449.24 | Local |
| M2 | 09/18/2011 0:00 | 3 | 49.683475 | -110.068666 | 567183 | 5503855 | 0 | 1534.42 | Local |
| M2 | 09/18/2011 0:00 | 9 | 49.678233 | -110.088340 | 565771 | 5503255 | 1 | 334.27 | Local |
| M2 | 09/18/2011 0:00 | 12 | 49.675964 | -110.085299 | 565993 | 5503006 | 0 | 52.65 | Local |
| M2 | 09/18/2011 0:00 | 18 | 49.676438 | -110.085306 | 565992 | 5503058 | 1 | 1683.80 | Local |
| M2 | 09/18/2011 0:00 | 21 | 49.688108 | -110.070431 | 567049 | 5504369 | 1 | 2264.26 | Local |
| M2 | 09/19/2011 0:00 | 0 | 49.697125 | -110.098579 | 565007 | 5505347 | 1 | 1710.12 | Local |
| M2 | 09/19/2011 0:00 | 3 | 49.682929 | -110.107706 | 564367 | 5503760 | 1 | 1358.13 | Local |
| M2 | 09/19/2011 0:00 | 6 | 49.694560 | -110.113458 | 563937 | 5505049 | 1 | 503.71 | Local |

| | | | | | | | | | |
|---|---|---|---|---|---|---|---|---|---|
| M2 | 09/19/2011 0:00 | 9 | 49.693754 | -110.120331 | 563442 | 5504953 | 1 | 0.94 | Local |
| M2 | 09/19/2011 0:00 | 12 | 49.693746 | -110.120328 | 563443 | 5504952 | 1 | 4.07 | Local |
| M2 | 09/19/2011 0:00 | 15 | 49.693715 | -110.120297 | 563445 | 5504949 | 1 | 5.41 | Local |
| M2 | 09/19/2011 0:00 | 18 | 49.693758 | -110.120262 | 563447 | 5504954 | 1 | 1675.74 | Local |
| M2 | 09/19/2011 0:00 | 21 | 49.696032 | -110.097293 | 565101 | 5505226 | 1 | 1082.22 | Local |
| M2 | 09/20/2011 0:00 | 0 | 49.702805 | -110.086515 | 565869 | 5505989 | 1 | 14.41 | Local |
| M2 | 09/20/2011 0:00 | 3 | 49.702817 | -110.086714 | 565855 | 5505990 | 1 | 12.48 | Local |
| M2 | 09/20/2011 0:00 | 6 | 49.702818 | -110.086541 | 565867 | 5505990 | 1 | 107.28 | Local |
| M2 | 09/20/2011 0:00 | 9 | 49.703615 | -110.087379 | 565806 | 5506078 | 1 | 19.97 | Local |
| M2 | 09/20/2011 0:00 | 12 | 49.703792 | -110.087423 | 565802 | 5506098 | 1 | 6.72 | Local |
| M2 | 09/20/2011 0:00 | 15 | 49.703733 | -110.087403 | 565804 | 5506091 | 1 | 430.28 | Local |
| M2 | 09/20/2011 0:00 | 18 | 49.703980 | -110.093358 | 565374 | 5506113 | 1 | 438.29 | Local |
| M2 | 09/20/2011 0:00 | 21 | 49.701386 | -110.088782 | 565707 | 5505829 | 1 | 1641.54 | Local |
| M2 | 09/21/2011 0:00 | 0 | 49.691927 | -110.071305 | 566981 | 5504793 | 1 | 1305.98 | Local |
| M2 | 09/21/2011 0:00 | 3 | 49.686161 | -110.055531 | 568126 | 5504166 | 1 | 11.90 | Local |
| M2 | 09/21/2011 0:00 | 6 | 49.686266 | -110.055498 | 568129 | 5504177 | 1 | 1427.99 | Local |
| M2 | 09/21/2011 0:00 | 9 | 49.675342 | -110.065907 | 567393 | 5502954 | 1 | 31.70 | Local |
| M2 | 09/21/2011 0:00 | 12 | 49.675430 | -110.065489 | 567423 | 5502964 | 1 | 17.54 | Local |
| M2 | 09/21/2011 0:00 | 15 | 49.675566 | -110.065612 | 567414 | 5502979 | 1 | 18.73 | Local |
| M2 | 09/21/2011 0:00 | 18 | 49.675455 | -110.065417 | 567428 | 5502967 | 1 | 991.53 | Local |
| M2 | 09/21/2011 0:00 | 21 | 49.678975 | -110.052790 | 568334 | 5503369 | 1 | 829.94 | Local |
| M2 | 09/22/2011 0:00 | 0 | 49.686216 | -110.055582 | 568123 | 5504172 | 1 | 961.77 | Local |
| M2 | 09/22/2011 0:00 | 3 | 49.677645 | -110.053775 | 568265 | 5503221 | 1 | 151.08 | Local |
| M2 | 09/22/2011 0:00 | 6 | 49.676664 | -110.052327 | 568371 | 5503113 | 0 | 86.55 | Local |
| M2 | 09/22/2011 0:00 | 12 | 49.676000 | -110.051700 | 568417 | 5503040 | 1 | 579.78 | Local |
| M2 | 09/22/2011 0:00 | 15 | 49.671528 | -110.055833 | 568125 | 5502539 | 1 | 3.36 | Local |
| M2 | 09/22/2011 0:00 | 18 | 49.671518 | -110.055789 | 568128 | 5502538 | 1 | 94.34 | Local |
| M2 | 09/22/2011 0:00 | 21 | 49.670694 | -110.056098 | 568107 | 5502446 | 1 | 672.24 | Local |
| M2 | 09/23/2011 0:00 | 0 | 49.665467 | -110.051416 | 568452 | 5501869 | 1 | 1562.60 | Local |
| M2 | 09/23/2011 0:00 | 3 | 49.651927 | -110.057216 | 568053 | 5500358 | 1 | 1185.01 | Local |
| M2 | 09/23/2011 0:00 | 6 | 49.644339 | -110.068744 | 567231 | 5499504 | 1 | 1606.90 | Local |
| M2 | 09/23/2011 0:00 | 9 | 49.632365 | -110.056282 | 568147 | 5498184 | 0 | 272.16 | Local |
| M2 | 09/23/2011 0:00 | 15 | 49.633706 | -110.053128 | 568373 | 5498336 | 1 | 5.81 | Local |
| M2 | 09/23/2011 0:00 | 18 | 49.633754 | -110.053160 | 568371 | 5498341 | 0 | 5113.37 | Local |
| M2 | 09/24/2011 0:00 | 0 | 49.588329 | -110.042095 | 569234 | 5493302 | 1 | 1895.66 | Local |
| M2 | 09/24/2011 0:00 | 3 | 49.586494 | -110.068169 | 567352 | 5493074 | 0 | 1348.26 | Local |
| M2 | 09/24/2011 0:00 | 9 | 49.585645 | -110.049562 | 568698 | 5492996 | 1 | 15.23 | Local |
| M2 | 09/24/2011 0:00 | 12 | 49.585586 | -110.049753 | 568685 | 5492990 | 1 | 1.88 | Local |
| M2 | 09/24/2011 0:00 | 15 | 49.585601 | -110.049739 | 568686 | 5492991 | 1 | 7.29 | Local |
| M2 | 09/24/2011 0:00 | 18 | 49.585651 | -110.049675 | 568690 | 5492997 | 1 | 659.41 | Local |
| M2 | 09/24/2011 0:00 | 21 | 49.581927 | -110.042575 | 569208 | 5492589 | 1 | 3511.11 | Local |
| M2 | 09/25/2011 0:00 | 0 | 49.567137 | -109.999669 | 572332 | 5490986 | 1 | 3418.10 | Local |
| M2 | 09/25/2011 0:00 | 3 | 49.570077 | -109.952616 | 575730 | 5491359 | 0 | 2903.32 | Local |
| M2 | 09/25/2011 0:00 | 9 | 49.559516 | -109.915899 | 578401 | 5490222 | 1 | 154.74 | Local |
| M2 | 09/25/2011 0:00 | 12 | 49.559394 | -109.918030 | 578247 | 5490206 | 0 | 18.00 | Local |
| M2 | 09/25/2011 0:00 | 18 | 49.559514 | -109.917864 | 578259 | 5490220 | 1 | 2992.63 | Local |
| M2 | 09/25/2011 0:00 | 21 | 49.561366 | -109.876584 | 581241 | 5490470 | 1 | 5549.54 | Local |
| M2 | 09/26/2011 0:00 | 0 | 49.601176 | -109.830280 | 584521 | 5494946 | 1 | 6304.91 | Local |
| M2 | 09/26/2011 0:00 | 3 | 49.655008 | -109.802843 | 586408 | 5500962 | 1 | 1994.36 | Local |
| M2 | 09/26/2011 0:00 | 6 | 49.656526 | -109.830373 | 584418 | 5501100 | 1 | 2591.08 | Local |
| M2 | 09/26/2011 0:00 | 9 | 49.650241 | -109.864937 | 581934 | 5500363 | 1 | 779.04 | Local |
| M2 | 09/26/2011 0:00 | 12 | 49.649798 | -109.875706 | 581158 | 5500302 | 1 | 24.11 | Local |
| M2 | 09/26/2011 0:00 | 15 | 49.649599 | -109.875573 | 581168 | 5500280 | 1 | 10.96 | Local |
| M2 | 09/26/2011 0:00 | 18 | 49.649530 | -109.875465 | 581176 | 5500272 | 1 | 3544.82 | Local |
| M2 | 09/26/2011 0:00 | 21 | 49.680092 | -109.889450 | 580116 | 5503655 | 1 | 2030.81 | Local |

| | | | | | | | | | |
|---|---|---|---|---|---|---|---|---|---|
| M2 | 09/27/2011 0:00 | 0 | 49.690173 | -109.912924 | 578406 | 5504751 | 1 | 3972.44 | Local |
| M2 | 09/27/2011 0:00 | 3 | 49.721496 | -109.939422 | 576446 | 5508206 | 1 | 1080.91 | Local |
| M2 | 09/27/2011 0:00 | 6 | 49.718193 | -109.953524 | 575435 | 5507824 | 1 | 4.52 | Local |
| M2 | 09/27/2011 0:00 | 9 | 49.718214 | -109.953471 | 575438 | 5507827 | 0 | 73.08 | Local |
| M2 | 09/27/2011 0:00 | 18 | 49.718093 | -109.954467 | 575367 | 5507812 | 1 | 311.47 | Local |
| M2 | 09/27/2011 0:00 | 21 | 49.720441 | -109.956823 | 575193 | 5508071 | 1 | 847.33 | Local |
| M2 | 09/28/2011 0:00 | 0 | 49.714794 | -109.948931 | 575771 | 5507451 | 1 | 2.72 | Local |
| M2 | 09/28/2011 0:00 | 3 | 49.714811 | -109.948958 | 575769 | 5507453 | 1 | 1289.34 | Local |
| M2 | 09/28/2011 0:00 | 6 | 49.725720 | -109.955022 | 575315 | 5508660 | 1 | 674.33 | Local |
| M2 | 09/28/2011 0:00 | 9 | 49.720705 | -109.960282 | 574944 | 5508097 | 0 | 7.38 | Local |
| M2 | 09/28/2011 0:00 | 15 | 49.720689 | -109.960182 | 574951 | 5508095 | 1 | 7.03 | Local |
| M2 | 09/28/2011 0:00 | 18 | 49.720746 | -109.960226 | 574948 | 5508102 | 1 | 1211.16 | Local |
| M2 | 09/28/2011 0:00 | 21 | 49.720321 | -109.943437 | 576158 | 5508071 | 1 | 843.72 | Local |
| M2 | 09/29/2011 0:00 | 0 | 49.714050 | -109.950029 | 575693 | 5507367 | 1 | 1338.97 | Local |
| M2 | 09/29/2011 0:00 | 3 | 49.703391 | -109.941388 | 576333 | 5506191 | 1 | 263.79 | Local |
| M2 | 09/29/2011 0:00 | 6 | 49.701127 | -109.940295 | 576415 | 5505940 | 1 | 1068.99 | Local |
| M2 | 09/29/2011 0:00 | 9 | 49.692073 | -109.945280 | 576070 | 5504929 | 1 | 23.21 | Local |
| M2 | 09/29/2011 0:00 | 12 | 49.691910 | -109.945482 | 576055 | 5504911 | 1 | 16.56 | Local |
| M2 | 09/29/2011 0:00 | 15 | 49.692058 | -109.945458 | 576057 | 5504927 | 1 | 25.54 | Local |
| M2 | 09/29/2011 0:00 | 18 | 49.692244 | -109.945666 | 576042 | 5504947 | 1 | 2015.13 | Local |
| M2 | 09/29/2011 0:00 | 21 | 49.685477 | -109.971582 | 574183 | 5504169 | 1 | 1528.55 | Local |
| M2 | 09/30/2011 0:00 | 0 | 49.682560 | -109.992288 | 572694 | 5503825 | 1 | 1716.52 | Local |
| M2 | 09/30/2011 0:00 | 3 | 49.670733 | -110.007579 | 571608 | 5502495 | 1 | 1955.35 | Local |
| M2 | 09/30/2011 0:00 | 6 | 49.655010 | -110.019715 | 570755 | 5500736 | 1 | 1982.29 | Local |
| M2 | 09/30/2011 0:00 | 9 | 49.637963 | -110.027760 | 570199 | 5498833 | 1 | 55.37 | Local |
| M2 | 09/30/2011 0:00 | 12 | 49.637465 | -110.027741 | 570201 | 5498778 | 0 | 71.33 | Local |
| M2 | 09/30/2011 0:00 | 18 | 49.638043 | -110.028170 | 570169 | 5498841 | 1 | 1525.54 | Local |
| M2 | 09/30/2011 0:00 | 21 | 49.627409 | -110.041519 | 569220 | 5497647 | 1 | 2299.71 | Local |
| M2 | 10/01/2011 0:00 | 0 | 49.619123 | -110.070691 | 567125 | 5496699 | 1 | 2673.90 | Local |
| M2 | 10/01/2011 0:00 | 3 | 49.602008 | -110.044691 | 569027 | 5494820 | 1 | 2409.19 | Local |
| M2 | 10/01/2011 0:00 | 6 | 49.596441 | -110.076910 | 566707 | 5494172 | 1 | 2540.23 | Local |
| M2 | 10/01/2011 0:00 | 9 | 49.618872 | -110.083586 | 566194 | 5496660 | 1 | 6.56 | Local |
| M2 | 10/01/2011 0:00 | 12 | 49.618841 | -110.083663 | 566188 | 5496656 | 1 | 379.05 | Local |
| M2 | 10/01/2011 0:00 | 15 | 49.620204 | -110.088473 | 565839 | 5496804 | 1 | 24.21 | Local |
| M2 | 10/01/2011 0:00 | 18 | 49.620395 | -110.088634 | 565827 | 5496825 | 0 | 4838.69 | Local |
| M2 | 10/02/2011 0:00 | 0 | 49.647765 | -110.036539 | 569551 | 5499914 | 1 | 2028.15 | Local |
| M2 | 10/02/2011 0:00 | 3 | 49.664724 | -110.026190 | 570274 | 5501809 | 1 | 3493.81 | Local |
| M2 | 10/02/2011 0:00 | 6 | 49.693993 | -110.008570 | 571502 | 5505080 | 1 | 1275.22 | Local |
| M2 | 10/02/2011 0:00 | 9 | 49.704402 | -110.015995 | 570952 | 5506230 | 0 | 8.19 | Local |
| M2 | 10/02/2011 0:00 | 15 | 49.704470 | -110.016040 | 570948 | 5506238 | 1 | 6.38 | Local |
| M2 | 10/02/2011 0:00 | 18 | 49.704502 | -110.015967 | 570954 | 5506241 | 1 | 235.97 | Local |
| M2 | 10/02/2011 0:00 | 21 | 49.706471 | -110.014744 | 571039 | 5506461 | 1 | 7.69 | Local |
| M2 | 10/03/2011 0:00 | 0 | 49.706507 | -110.014835 | 571032 | 5506465 | 1 | 8.32 | Local |
| M2 | 10/03/2011 0:00 | 3 | 49.706467 | -110.014737 | 571039 | 5506461 | 1 | 69.50 | Local |
| M2 | 10/03/2011 0:00 | 6 | 49.705861 | -110.014505 | 571057 | 5506394 | 1 | 152.19 | Local |
| M2 | 10/03/2011 0:00 | 9 | 49.705193 | -110.016347 | 570925 | 5506318 | 0 | 64.56 | Local |
| M2 | 10/03/2011 0:00 | 18 | 49.705766 | -110.016491 | 570914 | 5506381 | 1 | 151.12 | Local |
| M2 | 10/03/2011 0:00 | 21 | 49.706491 | -110.014719 | 571041 | 5506464 | 1 | 4.07 | Local |
| M2 | 10/04/2011 0:00 | 0 | 49.706527 | -110.014728 | 571040 | 5506468 | 1 | 2910.81 | Local |
| M2 | 10/04/2011 0:00 | 3 | 49.685511 | -109.990664 | 572806 | 5504154 | 1 | 1097.87 | Local |
| M2 | 10/04/2011 0:00 | 6 | 49.683973 | -109.975630 | 573893 | 5503998 | 1 | 859.20 | Local |
| M2 | 10/04/2011 0:00 | 9 | 49.688704 | -109.966214 | 574565 | 5504533 | 1 | 21.18 | Local |
| M2 | 10/04/2011 0:00 | 12 | 49.688656 | -109.965930 | 574586 | 5504528 | 1 | 31.96 | Local |
| M2 | 10/04/2011 0:00 | 15 | 49.688688 | -109.966370 | 574554 | 5504531 | 1 | 22.09 | Local |
| M2 | 10/04/2011 0:00 | 18 | 49.688722 | -109.966068 | 574576 | 5504535 | 1 | 370.54 | Local |

| | | | | | | | | | |
|---|---|---|---|---|---|---|---|---|---|
| M2 | 10/04/2011 0:00 | 21 | 49.690721 | -109.961958 | 574869 | 5504762 | 1 | 1671.49 | Local |
| M2 | 10/05/2011 0:00 | 0 | 49.680370 | -109.978762 | 573673 | 5503594 | 1 | 1821.95 | Local |
| M2 | 10/05/2011 0:00 | 3 | 49.678635 | -109.953650 | 575487 | 5503426 | 1 | 830.11 | Local |
| M2 | 10/05/2011 0:00 | 6 | 49.683454 | -109.962438 | 574845 | 5503953 | 1 | 594.57 | Local |
| M2 | 10/05/2011 0:00 | 9 | 49.688357 | -109.965730 | 574601 | 5504495 | 1 | 51.32 | Local |
| M2 | 10/05/2011 0:00 | 12 | 49.688587 | -109.966347 | 574556 | 5504520 | 1 | 10.10 | Local |
| M2 | 10/05/2011 0:00 | 15 | 49.688678 | -109.966343 | 574556 | 5504530 | 1 | 4.39 | Local |
| M2 | 10/05/2011 0:00 | 18 | 49.688706 | -109.966300 | 574559 | 5504533 | 1 | 560.46 | Local |
| M2 | 10/05/2011 0:00 | 21 | 49.688647 | -109.974070 | 573999 | 5504519 | 1 | 2633.88 | Local |
| M2 | 10/06/2011 0:00 | 0 | 49.696098 | -110.008733 | 571487 | 5505314 | 1 | 2190.50 | Local |
| M2 | 10/06/2011 0:00 | 3 | 49.715613 | -110.012907 | 571158 | 5507480 | 1 | 1711.19 | Local |
| M2 | 10/06/2011 0:00 | 6 | 49.705608 | -109.994873 | 572473 | 5506385 | 1 | 2727.94 | Local |
| M2 | 10/06/2011 0:00 | 9 | 49.686275 | -109.971585 | 574181 | 5504258 | 1 | 241.20 | Local |
| M2 | 10/06/2011 0:00 | 12 | 49.687780 | -109.969178 | 574353 | 5504428 | 1 | 211.87 | Local |
| M2 | 10/06/2011 0:00 | 15 | 49.686772 | -109.971671 | 574174 | 5504313 | 1 | 136.74 | Local |
| M2 | 10/06/2011 0:00 | 18 | 49.686985 | -109.969804 | 574309 | 5504339 | 1 | 1919.00 | Local |
| M2 | 10/06/2011 0:00 | 21 | 49.688788 | -109.943346 | 576214 | 5504566 | 1 | 2295.44 | Local |
| M2 | 10/07/2011 0:00 | 0 | 49.709431 | -109.943854 | 576145 | 5506860 | 1 | 2822.45 | Local |
| M2 | 10/07/2011 0:00 | 3 | 49.725110 | -109.974644 | 573902 | 5508572 | 1 | 10.65 | Local |
| M2 | 10/07/2011 0:00 | 6 | 49.725020 | -109.974593 | 573906 | 5508562 | 1 | 175.74 | Local |
| M2 | 10/07/2011 0:00 | 9 | 49.724580 | -109.972251 | 574075 | 5508516 | 1 | 5.47 | Local |
| M2 | 10/07/2011 0:00 | 12 | 49.724531 | -109.972249 | 574075 | 5508510 | 1 | 12.50 | Local |
| M2 | 10/07/2011 0:00 | 15 | 49.724419 | -109.972257 | 574075 | 5508498 | 1 | 5.17 | Local |
| M2 | 10/07/2011 0:00 | 18 | 49.724412 | -109.972328 | 574070 | 5508497 | 1 | 111.85 | Local |
| M2 | 10/07/2011 0:00 | 21 | 49.723462 | -109.971819 | 574108 | 5508392 | 1 | 397.68 | Local |
| M2 | 10/08/2011 0:00 | 0 | 49.726310 | -109.968481 | 574344 | 5508712 | 1 | 629.98 | Local |
| M2 | 10/08/2011 0:00 | 3 | 49.725733 | -109.977176 | 573718 | 5508639 | 1 | 580.38 | Local |
| M2 | 10/08/2011 0:00 | 6 | 49.720573 | -109.978388 | 573639 | 5508064 | 1 | 589.28 | Local |
| M2 | 10/08/2011 0:00 | 9 | 49.722883 | -109.971031 | 574166 | 5508328 | 1 | 181.13 | Local |
| M2 | 10/08/2011 0:00 | 12 | 49.724165 | -109.972582 | 574052 | 5508469 | 1 | 4.38 | Local |
| M2 | 10/08/2011 0:00 | 15 | 49.724137 | -109.972624 | 574049 | 5508466 | 1 | 9.35 | Local |
| M2 | 10/08/2011 0:00 | 18 | 49.724172 | -109.972743 | 574040 | 5508470 | 1 | 387.27 | Local |
| M2 | 10/08/2011 0:00 | 21 | 49.722207 | -109.968307 | 574363 | 5508256 | 1 | 917.91 | Local |
| M2 | 10/09/2011 0:00 | 0 | 49.729566 | -109.962536 | 574768 | 5509080 | 1 | 1712.45 | Local |
| M2 | 10/09/2011 0:00 | 3 | 49.738927 | -109.943667 | 576113 | 5510140 | 1 | 1405.51 | Local |
| M2 | 10/09/2011 0:00 | 6 | 49.731395 | -109.928005 | 577253 | 5509318 | 1 | 17.38 | Local |
| M2 | 10/09/2011 0:00 | 9 | 49.731471 | -109.928215 | 577238 | 5509326 | 1 | 8.46 | Local |
| M2 | 10/09/2011 0:00 | 12 | 49.731497 | -109.928106 | 577246 | 5509329 | 1 | 27.88 | Local |
| M2 | 10/09/2011 0:00 | 15 | 49.731461 | -109.927723 | 577273 | 5509326 | 1 | 43.84 | Local |
| M2 | 10/09/2011 0:00 | 18 | 49.731130 | -109.928055 | 577250 | 5509289 | 1 | 37.61 | Local |
| M2 | 10/09/2011 0:00 | 21 | 49.731468 | -109.928062 | 577249 | 5509326 | 1 | 35.06 | Local |
| M2 | 10/10/2011 0:00 | 0 | 49.731617 | -109.928491 | 577218 | 5509342 | 1 | 34.52 | Local |
| M2 | 10/10/2011 0:00 | 3 | 49.731505 | -109.928044 | 577250 | 5509330 | 1 | 20.13 | Local |
| M2 | 10/10/2011 0:00 | 6 | 49.731519 | -109.927765 | 577270 | 5509332 | 1 | 9.67 | Local |
| M2 | 10/10/2011 0:00 | 9 | 49.731542 | -109.927894 | 577261 | 5509335 | 1 | 12.88 | Local |
| M2 | 10/10/2011 0:00 | 12 | 49.731572 | -109.927722 | 577273 | 5509338 | 0 | 23.10 | Local |
| M2 | 10/10/2011 0:00 | 18 | 49.731507 | -109.928026 | 577251 | 5509331 | 0 | 11.91 | Local |
| M2 | 10/11/2011 0:00 | 0 | 49.731419 | -109.928120 | 577245 | 5509321 | 1 | 11.00 | Local |
| M2 | 10/11/2011 0:00 | 3 | 49.731497 | -109.928213 | 577238 | 5509329 | 1 | 15.34 | Local |
| M2 | 10/11/2011 0:00 | 6 | 49.731431 | -109.928026 | 577252 | 5509322 | 1 | 3.74 | Local |
| M2 | 10/11/2011 0:00 | 9 | 49.731449 | -109.927982 | 577255 | 5509324 | 1 | 19.16 | Local |
| M2 | 10/11/2011 0:00 | 12 | 49.731537 | -109.927753 | 577271 | 5509334 | 1 | 27.05 | Local |
| M2 | 10/11/2011 0:00 | 15 | 49.731378 | -109.928038 | 577251 | 5509316 | 1 | 6.39 | Local |
| M2 | 10/11/2011 0:00 | 18 | 49.731434 | -109.928018 | 577252 | 5509322 | 1 | 1.97 | Local |
| M2 | 10/11/2011 0:00 | 21 | 49.731445 | -109.927997 | 577254 | 5509324 | 1 | 5.97 | Local |

| | | | | | | | | | |
|---|---|---|---|---|---|---|---|---|---|
| M2 | 10/12/2011 0:00 | 0 | 49.731405 | -109.928051 | 577250 | 5509319 | 1 | 9.54 | Local |
| M2 | 10/12/2011 0:00 | 3 | 49.731486 | -109.928094 | 577247 | 5509328 | 1 | 1.34 | Local |
| M2 | 10/12/2011 0:00 | 6 | 49.731478 | -109.928079 | 577248 | 5509327 | 1 | 5.62 | Local |
| M2 | 10/12/2011 0:00 | 9 | 49.731429 | -109.928061 | 577249 | 5509322 | 1 | 10.90 | Local |
| M2 | 10/12/2011 0:00 | 12 | 49.731525 | -109.928025 | 577252 | 5509333 | 1 | 1.08 | Local |
| M2 | 10/12/2011 0:00 | 15 | 49.731529 | -109.928038 | 577251 | 5509333 | 1 | 12.24 | Local |
| M2 | 10/12/2011 0:00 | 18 | 49.731456 | -109.927911 | 577260 | 5509325 | 1 | 11.20 | Local |
| M2 | 10/12/2011 0:00 | 21 | 49.731491 | -109.928057 | 577249 | 5509329 | 1 | 2.33 | Local |
| M2 | 10/13/2011 0:00 | 0 | 49.731502 | -109.928084 | 577247 | 5509330 | 1 | 0.18 | Local |
| M2 | 10/13/2011 0:00 | 3 | 49.731501 | -109.928085 | 577247 | 5509330 | 1 | 11.41 | Local |
| M2 | 10/13/2011 0:00 | 6 | 49.731559 | -109.927955 | 577257 | 5509336 | 1 | 309.57 | Local |
| M2 | 10/13/2011 0:00 | 9 | 49.733870 | -109.930350 | 577080 | 5509591 | 0 | 7.60 | Local |
| M2 | 10/13/2011 0:00 | 15 | 49.733808 | -109.930307 | 577084 | 5509584 | 1 | 13.74 | Local |
| M2 | 10/13/2011 0:00 | 18 | 49.733926 | -109.930364 | 577079 | 5509597 | 1 | 2190.03 | Local |
| M2 | 10/13/2011 0:00 | 21 | 49.729650 | -109.960026 | 574948 | 5509092 | 1 | 2517.63 | Local |
| M2 | 10/14/2011 0:00 | 0 | 49.709554 | -109.943935 | 576139 | 5506874 | 1 | 874.08 | Local |
| M2 | 10/14/2011 0:00 | 3 | 49.701899 | -109.941177 | 576350 | 5506025 | 1 | 7.68 | Local |
| M2 | 10/14/2011 0:00 | 6 | 49.701897 | -109.941070 | 576358 | 5506025 | 1 | 3083.94 | Local |
| M2 | 10/14/2011 0:00 | 9 | 49.677876 | -109.962446 | 574853 | 5503333 | 1 | 0.74 | Local |
| M2 | 10/14/2011 0:00 | 12 | 49.677878 | -109.962455 | 574853 | 5503334 | 1 | 83.72 | Local |
| M2 | 10/14/2011 0:00 | 15 | 49.677170 | -109.962849 | 574825 | 5503254 | 1 | 13.34 | Local |
| M2 | 10/14/2011 0:00 | 18 | 49.677241 | -109.962998 | 574815 | 5503262 | 1 | 137.77 | Local |
| M2 | 10/14/2011 0:00 | 21 | 49.676118 | -109.963803 | 574758 | 5503136 | 1 | 522.16 | Local |
| M2 | 10/15/2011 0:00 | 0 | 49.672118 | -109.960009 | 575038 | 5502696 | 1 | 1361.25 | Local |
| M2 | 10/15/2011 0:00 | 3 | 49.659888 | -109.959168 | 575118 | 5501337 | 1 | 2037.30 | Local |
| M2 | 10/15/2011 0:00 | 6 | 49.641733 | -109.962989 | 574870 | 5499314 | 1 | 641.23 | Local |
| M2 | 10/15/2011 0:00 | 9 | 49.640876 | -109.971771 | 574237 | 5499211 | 1 | 208.22 | Local |
| M2 | 10/15/2011 0:00 | 12 | 49.639384 | -109.973515 | 574113 | 5499043 | 1 | 20.13 | Local |
| M2 | 10/15/2011 0:00 | 15 | 49.639499 | -109.973731 | 574098 | 5499055 | 1 | 506.28 | Local |
| M2 | 10/15/2011 0:00 | 18 | 49.643442 | -109.977236 | 573839 | 5499490 | 1 | 2463.16 | Local |
| M2 | 10/15/2011 0:00 | 21 | 49.665476 | -109.973692 | 574061 | 5501944 | 1 | 3182.31 | Local |
| M2 | 10/16/2011 0:00 | 0 | 49.686538 | -109.943826 | 576183 | 5504315 | 1 | 964.69 | Local |
| M2 | 10/16/2011 0:00 | 3 | 49.695071 | -109.941413 | 576344 | 5505266 | 1 | 2354.01 | Local |
| M2 | 10/16/2011 0:00 | 6 | 49.675971 | -109.955490 | 575358 | 5503128 | 1 | 410.67 | Local |
| M2 | 10/16/2011 0:00 | 9 | 49.677682 | -109.960534 | 574992 | 5503314 | 0 | 22.63 | Local |
| M2 | 10/16/2011 0:00 | 15 | 49.677879 | -109.960457 | 574997 | 5503336 | 1 | 9.54 | Local |
| M2 | 10/16/2011 0:00 | 18 | 49.677919 | -109.960340 | 575005 | 5503340 | 1 | 1571.80 | Local |
| M2 | 10/16/2011 0:00 | 21 | 49.691770 | -109.964701 | 574669 | 5504876 | 1 | 3626.53 | Local |
| M2 | 10/17/2011 0:00 | 0 | 49.722187 | -109.946548 | 575931 | 5508276 | 1 | 1638.43 | Local |
| M2 | 10/17/2011 0:00 | 3 | 49.729520 | -109.926831 | 577341 | 5509111 | 1 | 3026.55 | Local |
| M2 | 10/17/2011 0:00 | 6 | 49.704074 | -109.941741 | 576306 | 5506267 | 1 | 2528.17 | Local |
| M2 | 10/17/2011 0:00 | 9 | 49.688607 | -109.967435 | 574477 | 5504521 | 1 | 132.20 | Local |
| M2 | 10/17/2011 0:00 | 12 | 49.688935 | -109.965674 | 574604 | 5504560 | 1 | 60.36 | Local |
| M2 | 10/17/2011 0:00 | 15 | 49.688601 | -109.966334 | 574557 | 5504522 | 0 | 2206.69 | Local |
| M2 | 10/17/2011 0:00 | 21 | 49.692410 | -109.996358 | 572385 | 5504916 | 1 | 4078.89 | Local |
| M2 | 10/18/2011 0:00 | 0 | 49.683247 | -110.051112 | 568449 | 5503846 | 1 | 1396.55 | Local |
| M2 | 10/18/2011 0:00 | 3 | 49.683538 | -110.070465 | 567053 | 5503861 | 0 | 893.49 | Local |
| M2 | 10/19/2011 0:00 | 0 | 49.675510 | -110.071030 | 567023 | 5502968 | 1 | 9.73 | Local |
| M2 | 10/19/2011 0:00 | 3 | 49.675470 | -110.071150 | 567015 | 5502963 | 1 | 413.54 | Local |
| M2 | 10/19/2011 0:00 | 6 | 49.672800 | -110.067160 | 567306 | 5502670 | 1 | 873.35 | Local |
| M2 | 10/19/2011 0:00 | 9 | 49.669760 | -110.056000 | 568116 | 5502342 | 1 | 374.93 | Local |
| M2 | 10/19/2011 0:00 | 12 | 49.671400 | -110.051460 | 568441 | 5502528 | 1 | 33.00 | Local |
| M2 | 10/19/2011 0:00 | 15 | 49.671130 | -110.051650 | 568427 | 5502498 | 0 | 16.15 | Local |
| M2 | 10/19/2011 0:00 | 21 | 49.671000 | -110.051750 | 568420 | 5502484 | 1 | 6.26 | Local |
| M2 | 10/20/2011 0:00 | 0 | 49.671050 | -110.051710 | 568423 | 5502489 | 1 | 8.66 | Local |

| | | | | | | | | | |
|---|---|---|---|---|---|---|---|---|---|
| M2 | 10/20/2011 0:00 | 3 | 49.671050 | -110.051830 | 568415 | 5502489 | 1 | 1865.65 | Local |
| M2 | 10/20/2011 0:00 | 6 | 49.663300 | -110.028900 | 570080 | 5501649 | 1 | 495.81 | Local |
| M2 | 10/20/2011 0:00 | 9 | 49.659870 | -110.024510 | 570402 | 5501271 | 1 | 21.78 | Local |
| M2 | 10/20/2011 0:00 | 12 | 49.660040 | -110.024360 | 570413 | 5501290 | 1 | 16.49 | Local |
| M2 | 10/20/2011 0:00 | 15 | 49.660170 | -110.024250 | 570420 | 5501305 | 1 | 11.64 | Local |
| M2 | 10/20/2011 0:00 | 18 | 49.660100 | -110.024370 | 570412 | 5501297 | 1 | 5.30 | Local |
| M2 | 10/20/2011 0:00 | 21 | 49.660140 | -110.024330 | 570415 | 5501302 | 1 | 1448.62 | Local |
| M2 | 10/21/2011 0:00 | 0 | 49.667260 | -110.007520 | 571617 | 5502109 | 0 | 2069.66 | Local |
| M2 | 10/21/2011 0:00 | 6 | 49.665430 | -109.978980 | 573679 | 5501933 | 1 | 1120.06 | Local |
| M2 | 10/21/2011 0:00 | 9 | 49.674520 | -109.972290 | 574148 | 5502950 | 0 | 27.05 | Local |
| M2 | 10/21/2011 0:00 | 15 | 49.674740 | -109.972450 | 574136 | 5502975 | 1 | 16.16 | Local |
| M2 | 10/21/2011 0:00 | 18 | 49.674600 | -109.972510 | 574132 | 5502959 | 1 | 788.82 | Local |
| M2 | 10/21/2011 0:00 | 21 | 49.675470 | -109.961660 | 574914 | 5503067 | 1 | 209.08 | Local |
| M2 | 10/22/2011 0:00 | 0 | 49.677120 | -109.963050 | 574811 | 5503249 | 1 | 21.39 | Local |
| M2 | 10/22/2011 0:00 | 3 | 49.677080 | -109.962760 | 574832 | 5503244 | 1 | 261.87 | Local |
| M2 | 10/22/2011 0:00 | 6 | 49.675430 | -109.965350 | 574648 | 5503058 | 1 | 299.37 | Local |
| M2 | 10/22/2011 0:00 | 9 | 49.677120 | -109.962120 | 574878 | 5503250 | 1 | 18.96 | Local |
| M2 | 10/22/2011 0:00 | 12 | 49.677290 | -109.962140 | 574876 | 5503268 | 0 | 8.91 | Local |
| M2 | 10/22/2011 0:00 | 18 | 49.677220 | -109.962080 | 574881 | 5503261 | 1 | 7.27 | Local |
| M2 | 10/22/2011 0:00 | 21 | 49.677160 | -109.962120 | 574878 | 5503254 | 0 | 63.91 | Local |
| M2 | 10/23/2011 0:00 | 3 | 49.676690 | -109.961610 | 574916 | 5503202 | 1 | 811.59 | Local |
| M2 | 10/23/2011 0:00 | 6 | 49.683890 | -109.959760 | 575038 | 5504005 | 1 | 689.02 | Local |
| M2 | 10/23/2011 0:00 | 9 | 49.688680 | -109.965820 | 574594 | 5504531 | 1 | 47.10 | Local |
| M2 | 10/23/2011 0:00 | 12 | 49.688640 | -109.966470 | 574547 | 5504526 | 0 | 16.92 | Local |
| M2 | 10/23/2011 0:00 | 18 | 49.688670 | -109.966240 | 574563 | 5504529 | 0 | 4.87 | Local |
| M2 | 10/24/2011 0:00 | 0 | 49.688650 | -109.966300 | 574559 | 5504527 | 1 | 7.05 | Local |
| M2 | 10/24/2011 0:00 | 3 | 49.688700 | -109.966240 | 574563 | 5504533 | 1 | 7.05 | Local |
| M2 | 10/24/2011 0:00 | 6 | 49.688750 | -109.966300 | 574559 | 5504538 | 1 | 94.54 | Local |
| M2 | 10/24/2011 0:00 | 9 | 49.688600 | -109.967590 | 574466 | 5504520 | 1 | 4.47 | Local |
| M2 | 10/24/2011 0:00 | 12 | 49.688590 | -109.967530 | 574470 | 5504519 | 1 | 88.85 | Local |
| M2 | 10/24/2011 0:00 | 15 | 49.688700 | -109.966310 | 574558 | 5504533 | 1 | 305.02 | Local |
| M2 | 10/24/2011 0:00 | 18 | 49.686230 | -109.964470 | 574695 | 5504260 | 1 | 160.86 | Local |
| M2 | 10/24/2011 0:00 | 21 | 49.686920 | -109.962510 | 574835 | 5504339 | 1 | 372.79 | Local |
| M2 | 10/25/2011 0:00 | 0 | 49.686420 | -109.957400 | 575204 | 5504288 | 1 | 281.02 | Local |
| M2 | 10/25/2011 0:00 | 3 | 49.683900 | -109.957100 | 575230 | 5504008 | 1 | 37.92 | Local |
| M2 | 10/25/2011 0:00 | 6 | 49.683850 | -109.956580 | 575267 | 5504003 | 1 | 799.68 | Local |
| M2 | 10/25/2011 0:00 | 9 | 49.688040 | -109.965590 | 574611 | 5504460 | 1 | 100.40 | Local |
| M2 | 10/25/2011 0:00 | 12 | 49.688750 | -109.966450 | 574548 | 5504538 | 1 | 9.31 | Local |
| M2 | 10/25/2011 0:00 | 15 | 49.688690 | -109.966360 | 574555 | 5504532 | 1 | 320.13 | Local |
| M2 | 10/25/2011 0:00 | 18 | 49.690040 | -109.970280 | 574270 | 5504678 | 1 | 1882.89 | Local |
| M2 | 10/25/2011 0:00 | 21 | 49.675450 | -109.957030 | 575248 | 5503069 | 1 | 2567.92 | Local |
| M2 | 10/26/2011 0:00 | 0 | 49.697980 | -109.949200 | 575778 | 5505582 | 1 | 3.61 | Local |
| M2 | 10/26/2011 0:00 | 3 | 49.697980 | -109.949150 | 575781 | 5505582 | 1 | 3662.48 | Local |
| M2 | 10/26/2011 0:00 | 6 | 49.727760 | -109.927440 | 577300 | 5508915 | 1 | 1.82 | Local |
| M2 | 10/26/2011 0:00 | 9 | 49.727750 | -109.927420 | 577301 | 5508913 | 1 | 4.51 | Local |
| M2 | 10/26/2011 0:00 | 12 | 49.727790 | -109.927430 | 577300 | 5508918 | 1 | 4.86 | Local |
| M2 | 10/26/2011 0:00 | 15 | 49.727770 | -109.927370 | 577305 | 5508916 | 1 | 2.43 | Local |
| M2 | 10/26/2011 0:00 | 18 | 49.727760 | -109.927400 | 577303 | 5508915 | 1 | 2.88 | Local |
| M2 | 10/26/2011 0:00 | 21 | 49.727760 | -109.927440 | 577300 | 5508915 | 1 | 8.92 | Local |
| M2 | 10/27/2011 0:00 | 0 | 49.727840 | -109.927450 | 577299 | 5508923 | 1 | 3.41 | Local |
| M2 | 10/27/2011 0:00 | 3 | 49.727810 | -109.927440 | 577300 | 5508920 | 1 | 4.51 | Local |
| M2 | 10/27/2011 0:00 | 6 | 49.727770 | -109.927430 | 577300 | 5508916 | 1 | 1.33 | Local |
| M2 | 10/27/2011 0:00 | 9 | 49.727780 | -109.927420 | 577301 | 5508917 | 1 | 1.82 | Local |
| M2 | 10/27/2011 0:00 | 12 | 49.727790 | -109.927400 | 577303 | 5508918 | 1 | 3.60 | Local |
| M2 | 10/27/2011 0:00 | 15 | 49.727790 | -109.927450 | 577299 | 5508918 | 1 | 2.34 | Local |

| | | | | | | | | | |
|---|---|---|---|---|---|---|---|---|---|
| M2 | 10/27/2011 0:00 | 18 | 49.727770 | -109.927440 | 577300 | 5508916 | 1 | 31.05 | Local |
| M2 | 10/27/2011 0:00 | 21 | 49.728040 | -109.927330 | 577307 | 5508946 | 1 | 92.53 | Local |
| M2 | 10/28/2011 0:00 | 0 | 49.728720 | -109.926590 | 577359 | 5509022 | 1 | 27.03 | Local |
| M2 | 10/28/2011 0:00 | 3 | 49.728480 | -109.926650 | 577355 | 5508995 | 1 | 3.97 | Local |
| M2 | 10/28/2011 0:00 | 6 | 49.728450 | -109.926680 | 577353 | 5508992 | 1 | 6.49 | Local |
| M2 | 10/28/2011 0:00 | 9 | 49.728450 | -109.926590 | 577360 | 5508992 | 1 | 5.17 | Local |
| M2 | 10/28/2011 0:00 | 12 | 49.728440 | -109.926660 | 577355 | 5508991 | 1 | 5.17 | Local |
| M2 | 10/28/2011 0:00 | 15 | 49.728450 | -109.926590 | 577360 | 5508992 | 1 | 3.41 | Local |
| M2 | 10/28/2011 0:00 | 18 | 49.728480 | -109.926600 | 577359 | 5508995 | 1 | 0.00 | Local |
| M2 | 10/28/2011 0:00 | 21 | 49.728480 | -109.926600 | 577359 | 5508995 | 1 | 145.90 | Local |
| M2 | 10/29/2011 0:00 | 0 | 49.728010 | -109.924710 | 577496 | 5508945 | 1 | 407.20 | Local |
| M2 | 10/29/2011 0:00 | 3 | 49.731310 | -109.927160 | 577314 | 5509310 | 1 | 4.32 | Local |
| M2 | 10/29/2011 0:00 | 6 | 49.731310 | -109.927220 | 577310 | 5509309 | 1 | 3.63 | Local |
| M2 | 10/29/2011 0:00 | 9 | 49.731280 | -109.927240 | 577309 | 5509306 | 1 | 38.36 | Local |
| M2 | 10/29/2011 0:00 | 12 | 49.731500 | -109.927650 | 577279 | 5509330 | 1 | 22.59 | Local |
| M2 | 10/29/2011 0:00 | 15 | 49.731470 | -109.927340 | 577301 | 5509327 | 1 | 21.88 | Local |
| M2 | 10/29/2011 0:00 | 18 | 49.731500 | -109.927640 | 577279 | 5509330 | 1 | 36.32 | Local |
| M2 | 10/29/2011 0:00 | 21 | 49.731310 | -109.927230 | 577309 | 5509309 | 1 | 4.23 | Local |
| M2 | 10/30/2011 0:00 | 0 | 49.731290 | -109.927180 | 577313 | 5509307 | 1 | 2.22 | Local |
| M2 | 10/30/2011 0:00 | 3 | 49.731310 | -109.927180 | 577313 | 5509310 | 1 | 2.16 | Local |
| M2 | 10/30/2011 0:00 | 6 | 49.731310 | -109.927210 | 577311 | 5509309 | 1 | 39.92 | Local |
| M2 | 10/30/2011 0:00 | 9 | 49.731500 | -109.927680 | 577276 | 5509330 | 1 | 16.72 | Local |
| M2 | 10/30/2011 0:00 | 12 | 49.731480 | -109.927450 | 577293 | 5509328 | 1 | 14.46 | Local |
| M2 | 10/30/2011 0:00 | 15 | 49.731490 | -109.927650 | 577279 | 5509329 | 1 | 9.10 | Local |
| M2 | 10/30/2011 0:00 | 18 | 49.731440 | -109.927550 | 577286 | 5509324 | 1 | 29.64 | Local |
| M2 | 10/30/2011 0:00 | 21 | 49.731290 | -109.927210 | 577311 | 5509307 | 1 | 204.93 | Local |
| M2 | 10/31/2011 0:00 | 0 | 49.731380 | -109.930050 | 577106 | 5509314 | 1 | 175.01 | Local |
| M2 | 10/31/2011 0:00 | 3 | 49.731510 | -109.927630 | 577280 | 5509331 | 1 | 41.87 | Local |
| M2 | 10/31/2011 0:00 | 6 | 49.731280 | -109.927170 | 577314 | 5509306 | 1 | 76.43 | Local |
| M2 | 10/31/2011 0:00 | 9 | 49.731300 | -109.928230 | 577237 | 5509307 | 0 | 44.61 | Local |
| M2 | 10/31/2011 0:00 | 15 | 49.731440 | -109.927650 | 577279 | 5509323 | 1 | 9.60 | Local |
| M2 | 10/31/2011 0:00 | 18 | 49.731520 | -109.927700 | 577275 | 5509332 | 1 | 7.01 | Local |
| M2 | 10/31/2011 0:00 | 21 | 49.731580 | -109.927730 | 577273 | 5509339 | 0 | 50.92 | Local |
| M2 | 11/01/2011 0:00 | 3 | 49.731270 | -109.927210 | 577311 | 5509305 | 0 | 104.16 | Local |
| M2 | 11/01/2011 0:00 | 9 | 49.731350 | -109.928650 | 577207 | 5509312 | 0 | 2.16 | Local |
| M2 | 11/01/2011 0:00 | 15 | 49.731350 | -109.928680 | 577205 | 5509312 | 1 | 4.51 | Local |
| M2 | 11/01/2011 0:00 | 18 | 49.731390 | -109.928670 | 577205 | 5509317 | 1 | 2.34 | Local |
| M2 | 11/01/2011 0:00 | 21 | 49.731370 | -109.928680 | 577205 | 5509315 | 1 | 14.46 | Local |
| M2 | 11/02/2011 0:00 | 0 | 49.731380 | -109.928480 | 577219 | 5509316 | 1 | 12.25 | Local |
| M2 | 11/02/2011 0:00 | 3 | 49.731380 | -109.928650 | 577207 | 5509316 | 1 | 2060.32 | Local |
| M2 | 11/02/2011 0:00 | 6 | 49.718760 | -109.949580 | 575718 | 5507891 | 1 | 3561.54 | Local |
| M2 | 11/02/2011 0:00 | 9 | 49.688690 | -109.966600 | 574537 | 5504531 | 0 | 187.32 | Local |
| M2 | 11/02/2011 0:00 | 15 | 49.687600 | -109.964620 | 574682 | 5504412 | 1 | 129.63 | Local |
| M2 | 11/02/2011 0:00 | 18 | 49.686750 | -109.965850 | 574594 | 5504316 | 0 | 199.34 | Local |
| M2 | 11/03/2011 0:00 | 0 | 49.685640 | -109.968020 | 574439 | 5504191 | 1 | 918.68 | Local |
| M2 | 11/03/2011 0:00 | 3 | 49.677590 | -109.970890 | 574245 | 5503293 | 1 | 79.68 | Local |
| M2 | 11/03/2011 0:00 | 6 | 49.678110 | -109.971650 | 574189 | 5503350 | 1 | 623.32 | Local |
| M2 | 11/03/2011 0:00 | 9 | 49.672860 | -109.968620 | 574416 | 5502769 | 1 | 23.35 | Local |
| M2 | 11/03/2011 0:00 | 12 | 49.672690 | -109.968430 | 574430 | 5502751 | 0 | 21.59 | Local |
| M2 | 11/03/2011 0:00 | 18 | 49.672840 | -109.968620 | 574416 | 5502767 | 1 | 963.22 | Local |
| M2 | 11/03/2011 0:00 | 21 | 49.664290 | -109.966470 | 574584 | 5501819 | 1 | 1186.75 | Local |
| M2 | 11/04/2011 0:00 | 0 | 49.664520 | -109.950030 | 575770 | 5501861 | 1 | 2115.21 | Local |
| M2 | 11/04/2011 0:00 | 3 | 49.672180 | -109.923200 | 577694 | 5502740 | 1 | 469.07 | Local |
| M2 | 11/04/2011 0:00 | 6 | 49.673140 | -109.916870 | 578149 | 5502853 | 1 | 205.96 | Local |
| M2 | 11/04/2011 0:00 | 9 | 49.671300 | -109.916540 | 578176 | 5502649 | 1 | 93.17 | Local |

| | | | | | | | | | |
|---|---|---|---|---|---|---|---|---|---|
| M2 | 11/04/2011 0:00 | 12 | 49.671090 | -109.917790 | 578086 | 5502624 | 1 | 95.67 | Local |
| M2 | 11/04/2011 0:00 | 15 | 49.671170 | -109.916470 | 578181 | 5502635 | 1 | 130.95 | Local |
| M2 | 11/04/2011 0:00 | 18 | 49.671320 | -109.914670 | 578311 | 5502653 | 1 | 3026.03 | Local |
| M2 | 11/04/2011 0:00 | 21 | 49.695330 | -109.894920 | 579696 | 5505343 | 1 | 3210.76 | Local |
| M2 | 11/05/2011 0:00 | 0 | 49.714630 | -109.861800 | 582052 | 5507524 | 1 | 17.64 | Local |
| M2 | 11/05/2011 0:00 | 3 | 49.714750 | -109.861640 | 582064 | 5507538 | 1 | 11.52 | Local |
| M2 | 11/05/2011 0:00 | 6 | 49.714800 | -109.861780 | 582054 | 5507543 | 1 | 2.22 | Local |
| M2 | 11/05/2011 0:00 | 9 | 49.714820 | -109.861780 | 582054 | 5507546 | 1 | 4.41 | Local |
| M2 | 11/05/2011 0:00 | 12 | 49.714790 | -109.861740 | 582056 | 5507542 | 1 | 34.65 | Local |
| M2 | 11/05/2011 0:00 | 15 | 49.714490 | -109.861870 | 582048 | 5507509 | 1 | 34.65 | Local |
| M2 | 11/05/2011 0:00 | 18 | 49.714790 | -109.861740 | 582056 | 5507542 | 1 | 3.64 | Local |
| M2 | 11/05/2011 0:00 | 21 | 49.714770 | -109.861780 | 582054 | 5507540 | 1 | 403.57 | Local |
| M2 | 11/06/2011 0:00 | 0 | 49.716440 | -109.856810 | 582409 | 5507731 | 1 | 491.37 | Local |
| M2 | 11/06/2011 0:00 | 3 | 49.716620 | -109.850000 | 582900 | 5507759 | 1 | 355.93 | Local |
| M2 | 11/06/2011 0:00 | 6 | 49.715620 | -109.854690 | 582563 | 5507642 | 1 | 5715.35 | Local |
| M2 | 11/06/2011 0:00 | 9 | 49.682350 | -109.915100 | 578262 | 5503879 | 1 | 51.09 | Local |
| M2 | 11/06/2011 0:00 | 12 | 49.681980 | -109.914680 | 578293 | 5503838 | 1 | 13.66 | Local |
| M2 | 11/06/2011 0:00 | 15 | 49.681880 | -109.914570 | 578301 | 5503827 | 1 | 88.67 | Local |
| M2 | 11/06/2011 0:00 | 18 | 49.681330 | -109.915460 | 578238 | 5503765 | 1 | 5.30 | Local |
| M2 | 11/06/2011 0:00 | 21 | 49.681290 | -109.915500 | 578235 | 5503761 | 1 | 3.98 | Local |
| M2 | 11/07/2011 0:00 | 0 | 49.681320 | -109.915470 | 578237 | 5503764 | 1 | 1364.60 | Local |
| M2 | 11/07/2011 0:00 | 3 | 49.684110 | -109.897050 | 579561 | 5504094 | 1 | 795.40 | Local |
| M2 | 11/07/2011 0:00 | 6 | 49.684890 | -109.908010 | 578769 | 5504169 | 1 | 816.97 | Local |
| M2 | 11/07/2011 0:00 | 9 | 49.683680 | -109.919180 | 577965 | 5504022 | 1 | 1.11 | Local |
| M2 | 11/07/2011 0:00 | 12 | 49.683670 | -109.919180 | 577966 | 5504021 | 1 | 102.79 | Local |
| M2 | 11/07/2011 0:00 | 15 | 49.682880 | -109.918440 | 578020 | 5503934 | 0 | 3610.52 | Local |
| M2 | 11/07/2011 0:00 | 21 | 49.698610 | -109.874650 | 581153 | 5505730 | 1 | 771.60 | Local |
| M2 | 11/08/2011 0:00 | 0 | 49.703790 | -109.867530 | 581658 | 5506313 | 0 | 345.63 | Local |
| M2 | 11/08/2011 0:00 | 6 | 49.705150 | -109.863220 | 581966 | 5506469 | 1 | 4613.17 | Local |
| M2 | 11/08/2011 0:00 | 9 | 49.681360 | -109.915620 | 578226 | 5503768 | 1 | 13.46 | Local |
| M2 | 11/08/2011 0:00 | 12 | 49.681280 | -109.915480 | 578236 | 5503760 | 0 | 294.45 | Local |
| M2 | 11/09/2011 0:00 | 0 | 49.683150 | -109.918370 | 578025 | 5503964 | 1 | 8.01 | Local |
| M2 | 11/09/2011 0:00 | 3 | 49.683100 | -109.918290 | 578031 | 5503959 | 1 | 358.02 | Local |
| M2 | 11/09/2011 0:00 | 6 | 49.680850 | -109.914740 | 578290 | 5503712 | 1 | 80.02 | Local |
| M2 | 11/09/2011 0:00 | 9 | 49.681320 | -109.915580 | 578229 | 5503764 | 1 | 6.86 | Local |
| M2 | 11/09/2011 0:00 | 12 | 49.681340 | -109.915670 | 578222 | 5503766 | 1 | 286.58 | Local |
| M2 | 11/09/2011 0:00 | 15 | 49.683200 | -109.918420 | 578021 | 5503970 | 0 | 4305.67 | Local |
| M2 | 11/09/2011 0:00 | 21 | 49.703860 | -109.867930 | 581629 | 5506320 | 1 | 1416.01 | Local |
| M2 | 11/10/2011 0:00 | 0 | 49.715570 | -109.860210 | 582165 | 5507631 | 1 | 391.08 | Local |
| M2 | 11/10/2011 0:00 | 3 | 49.716020 | -109.854830 | 582553 | 5507687 | 1 | 1417.30 | Local |
| M2 | 11/10/2011 0:00 | 6 | 49.704850 | -109.864300 | 581889 | 5506434 | 1 | 2172.10 | Local |
| M2 | 11/10/2011 0:00 | 9 | 49.698530 | -109.892800 | 579844 | 5505701 | 0 | 64.82 | Local |
| M2 | 11/10/2011 0:00 | 18 | 49.697970 | -109.893050 | 579827 | 5505639 | 1 | 2188.38 | Local |
| M2 | 11/10/2011 0:00 | 21 | 49.705040 | -109.864730 | 581857 | 5506455 | 1 | 335.52 | Local |
| M2 | 11/11/2011 0:00 | 0 | 49.702980 | -109.868130 | 581616 | 5506222 | 1 | 48.51 | Local |
| M2 | 11/11/2011 0:00 | 3 | 49.702570 | -109.867900 | 581633 | 5506177 | 0 | 4287.94 | Local |
| M2 | 11/11/2011 0:00 | 9 | 49.681780 | -109.917970 | 578056 | 5503813 | 0 | 68.37 | Local |
| M2 | 11/11/2011 0:00 | 15 | 49.682390 | -109.917850 | 578063 | 5503880 | 0 | 3375.28 | Local |
| M2 | 11/11/2011 0:00 | 21 | 49.697550 | -109.877310 | 580963 | 5505609 | 1 | 1009.83 | Local |
| M2 | 11/12/2011 0:00 | 0 | 49.703960 | -109.867390 | 581667 | 5506332 | 1 | 16.49 | Local |
| M2 | 11/12/2011 0:00 | 3 | 49.703830 | -109.867500 | 581660 | 5506318 | 1 | 1.11 | Local |
| M2 | 11/12/2011 0:00 | 6 | 49.703840 | -109.867500 | 581660 | 5506319 | 1 | 4264.00 | Local |
| M2 | 11/12/2011 0:00 | 9 | 49.681700 | -109.915770 | 578215 | 5503806 | 1 | 41.02 | Local |
| M2 | 11/12/2011 0:00 | 12 | 49.681350 | -109.915590 | 578228 | 5503767 | 1 | 8.30 | Local |
| M2 | 11/12/2011 0:00 | 15 | 49.681280 | -109.915550 | 578231 | 5503759 | 1 | 25.57 | Local |

| | | | | | | | | | |
|---|---|---|---|---|---|---|---|---|---|
| M2 | 11/12/2011 0:00 | 18 | 49.681510 | -109.915550 | 578231 | 5503785 | 1 | 3507.07 | Local |
| M2 | 11/12/2011 0:00 | 21 | 49.698330 | -109.874420 | 581170 | 5505699 | 1 | 743.30 | Local |
| M2 | 11/13/2011 0:00 | 0 | 49.703010 | -109.867060 | 581693 | 5506227 | 1 | 90.52 | Local |
| M2 | 11/13/2011 0:00 | 3 | 49.703770 | -109.867510 | 581659 | 5506311 | 1 | 336.95 | Local |
| M2 | 11/13/2011 0:00 | 6 | 49.701520 | -109.870640 | 581437 | 5506057 | 1 | 4019.30 | Local |
| M2 | 11/13/2011 0:00 | 9 | 49.680290 | -109.915740 | 578219 | 5503649 | 1 | 162.69 | Local |
| M2 | 11/13/2011 0:00 | 12 | 49.681620 | -109.916680 | 578149 | 5503796 | 1 | 92.10 | Local |
| M2 | 11/13/2011 0:00 | 15 | 49.681320 | -109.915490 | 578235 | 5503764 | 1 | 9.31 | Local |
| M2 | 11/13/2011 0:00 | 18 | 49.681260 | -109.915580 | 578229 | 5503757 | 1 | 1536.31 | Local |
| M2 | 11/13/2011 0:00 | 21 | 49.693780 | -109.906570 | 578859 | 5505159 | 1 | 1938.04 | Local |
| M2 | 11/14/2011 0:00 | 0 | 49.676980 | -109.913730 | 578369 | 5503283 | 1 | 2080.12 | Local |
| M2 | 11/14/2011 0:00 | 3 | 49.660160 | -109.901110 | 579307 | 5501427 | 1 | 3.34 | Local |
| M2 | 11/14/2011 0:00 | 6 | 49.660130 | -109.901110 | 579307 | 5501423 | 1 | 737.42 | Local |
| M2 | 11/14/2011 0:00 | 9 | 49.654030 | -109.905120 | 579028 | 5500741 | 1 | 1091.34 | Local |
| M2 | 11/14/2011 0:00 | 12 | 49.645190 | -109.898550 | 579516 | 5499765 | 1 | 629.79 | Local |
| M2 | 11/14/2011 0:00 | 15 | 49.647700 | -109.890730 | 580077 | 5500052 | 1 | 446.77 | Local |
| M2 | 11/14/2011 0:00 | 18 | 49.651200 | -109.887690 | 580290 | 5500445 | 1 | 3743.95 | Local |
| M2 | 11/14/2011 0:00 | 21 | 49.652400 | -109.835860 | 584030 | 5500635 | 1 | 1637.84 | Local |
| M2 | 11/15/2011 0:00 | 0 | 49.638180 | -109.829940 | 584481 | 5499060 | 1 | 2129.88 | Local |
| M2 | 11/15/2011 0:00 | 3 | 49.621940 | -109.814300 | 585639 | 5497273 | 1 | 1404.79 | Local |
| M2 | 11/15/2011 0:00 | 6 | 49.615770 | -109.797330 | 586876 | 5496606 | 1 | 16.04 | Local |
| M2 | 11/15/2011 0:00 | 9 | 49.615670 | -109.797170 | 586888 | 5496595 | 1 | 803.54 | Local |
| M2 | 11/15/2011 0:00 | 12 | 49.616550 | -109.808210 | 586089 | 5496680 | 1 | 7.31 | Local |
| M2 | 11/15/2011 0:00 | 15 | 49.616580 | -109.808120 | 586095 | 5496684 | 1 | 3.11 | Local |
| M2 | 11/15/2011 0:00 | 18 | 49.616600 | -109.808150 | 586093 | 5496686 | 1 | 3094.08 | Local |
| M2 | 11/15/2011 0:00 | 21 | 49.601420 | -109.772260 | 588713 | 5495040 | 1 | 1653.10 | Local |
| M2 | 11/16/2011 0:00 | 0 | 49.587120 | -109.766000 | 589191 | 5493458 | 1 | 4253.21 | Local |
| M2 | 11/16/2011 0:00 | 3 | 49.613710 | -109.723690 | 592199 | 5496465 | 1 | 6813.66 | Local |
| M2 | 11/16/2011 0:00 | 6 | 49.641410 | -109.807840 | 586071 | 5499445 | 1 | 910.25 | Local |
| M2 | 11/16/2011 0:00 | 9 | 49.641150 | -109.820440 | 585162 | 5499401 | 1 | 38.92 | Local |
| M2 | 11/16/2011 0:00 | 12 | 49.641500 | -109.820440 | 585162 | 5499440 | 1 | 24.66 | Local |
| M2 | 11/16/2011 0:00 | 15 | 49.641710 | -109.820330 | 585169 | 5499464 | 1 | 12.75 | Local |
| M2 | 11/16/2011 0:00 | 18 | 49.641600 | -109.820380 | 585166 | 5499451 | 1 | 4212.56 | Local |
| M2 | 11/16/2011 0:00 | 21 | 49.678930 | -109.830350 | 584381 | 5503590 | 1 | 3368.24 | Local |
| M2 | 11/17/2011 0:00 | 0 | 49.706550 | -109.849530 | 582951 | 5506640 | 1 | 153.68 | Local |
| M2 | 11/17/2011 0:00 | 3 | 49.707630 | -109.848200 | 583045 | 5506761 | 1 | 755.63 | Local |
| M2 | 11/17/2011 0:00 | 6 | 49.710280 | -109.838550 | 583736 | 5507067 | 1 | 6620.67 | Local |
| M2 | 11/17/2011 0:00 | 9 | 49.681510 | -109.918920 | 577988 | 5503782 | 1 | 117.47 | Local |
| M2 | 11/17/2011 0:00 | 12 | 49.682470 | -109.918240 | 578035 | 5503889 | 1 | 60.56 | Local |
| M2 | 11/17/2011 0:00 | 15 | 49.683010 | -109.918350 | 578026 | 5503949 | 0 | 9.28 | Local |
| M2 | 11/17/2011 0:00 | 21 | 49.683040 | -109.918470 | 578018 | 5503952 | 1 | 5.46 | Local |
| M2 | 11/18/2011 0:00 | 0 | 49.683010 | -109.918410 | 578022 | 5503949 | 1 | 10.90 | Local |
| M2 | 11/18/2011 0:00 | 3 | 49.682960 | -109.918540 | 578013 | 5503943 | 1 | 22.10 | Local |
| M2 | 11/18/2011 0:00 | 6 | 49.683150 | -109.918450 | 578019 | 5503964 | 1 | 22.25 | Local |
| M2 | 11/18/2011 0:00 | 9 | 49.682950 | -109.918440 | 578020 | 5503942 | 1 | 23.42 | Local |
| M2 | 11/18/2011 0:00 | 12 | 49.683140 | -109.918300 | 578030 | 5503963 | 1 | 21.81 | Local |
| M2 | 11/18/2011 0:00 | 15 | 49.682960 | -109.918420 | 578021 | 5503943 | 1 | 345.60 | Local |
| M2 | 11/18/2011 0:00 | 18 | 49.685130 | -109.921850 | 577771 | 5504181 | 1 | 2285.75 | Local |
| M2 | 11/18/2011 0:00 | 21 | 49.689100 | -109.890760 | 580007 | 5504655 | 1 | 3169.57 | Local |
| M2 | 11/19/2011 0:00 | 0 | 49.708480 | -109.858530 | 582299 | 5506844 | 1 | 197.18 | Local |
| M2 | 11/19/2011 0:00 | 3 | 49.709780 | -109.860390 | 582162 | 5506987 | 1 | 546.71 | Local |
| M2 | 11/19/2011 0:00 | 6 | 49.714610 | -109.861810 | 582052 | 5507522 | 1 | 2725.90 | Local |
| M2 | 11/19/2011 0:00 | 9 | 49.698860 | -109.890780 | 579989 | 5505740 | 0 | 2694.36 | Local |
| M2 | 11/19/2011 0:00 | 15 | 49.682510 | -109.918350 | 578027 | 5503893 | 1 | 65.92 | Local |
| M2 | 11/19/2011 0:00 | 18 | 49.683100 | -109.918440 | 578020 | 5503959 | 1 | 15.57 | Local |

| | | | | | | | | | |
|---|---|---|---|---|---|---|---|---|---|
| M2 | 11/19/2011 0:00 | 21 | 49.682960 | -109.918440 | 578020 | 5503943 | 1 | 2.43 | Local |
| M2 | 11/20/2011 0:00 | 0 | 49.682970 | -109.918470 | 578018 | 5503944 | 1 | 5552.54 | Local |
| M2 | 11/20/2011 0:00 | 3 | 49.715700 | -109.860320 | 582157 | 5507645 | 1 | 199.43 | Local |
| M2 | 11/20/2011 0:00 | 6 | 49.717190 | -109.861860 | 582044 | 5507809 | 1 | 5732.14 | Local |
| M2 | 11/20/2011 0:00 | 9 | 49.681550 | -109.919290 | 577961 | 5503786 | 1 | 170.15 | Local |
| M2 | 11/20/2011 0:00 | 12 | 49.682980 | -109.918450 | 578019 | 5503945 | 1 | 3.10 | Local |
| M2 | 11/20/2011 0:00 | 15 | 49.683000 | -109.918480 | 578017 | 5503948 | 1 | 12.25 | Local |
| M2 | 11/20/2011 0:00 | 18 | 49.683110 | -109.918490 | 578016 | 5503960 | 1 | 24.73 | Local |
| M2 | 11/20/2011 0:00 | 21 | 49.682890 | -109.918540 | 578013 | 5503935 | 1 | 926.47 | Local |
| M2 | 11/21/2011 0:00 | 0 | 49.682140 | -109.931330 | 577091 | 5503839 | 1 | 1609.69 | Local |
| M2 | 11/21/2011 0:00 | 3 | 49.669440 | -109.920620 | 577884 | 5502438 | 1 | 365.12 | Local |
| M2 | 11/21/2011 0:00 | 6 | 49.668730 | -109.915680 | 578242 | 5502364 | 1 | 14.03 | Local |
| M2 | 11/21/2011 0:00 | 9 | 49.668850 | -109.915620 | 578246 | 5502377 | 0 | 1714.17 | Local |
| M2 | 11/21/2011 0:00 | 21 | 49.677920 | -109.896410 | 579617 | 5503406 | 1 | 4878.53 | Local |
| M2 | 11/22/2011 0:00 | 0 | 49.716210 | -109.863380 | 581936 | 5507698 | 1 | 197.40 | Local |
| M2 | 11/22/2011 0:00 | 3 | 49.717970 | -109.863740 | 581907 | 5507894 | 1 | 525.78 | Local |
| M2 | 11/22/2011 0:00 | 6 | 49.713430 | -109.861700 | 582062 | 5507391 | 1 | 5383.37 | Local |
| M2 | 11/22/2011 0:00 | 9 | 49.680690 | -109.916690 | 578150 | 5503693 | 1 | 119.85 | Local |
| M2 | 11/22/2011 0:00 | 12 | 49.681110 | -109.915160 | 578260 | 5503741 | 1 | 5.97 | Local |
| M2 | 11/22/2011 0:00 | 15 | 49.681060 | -109.915130 | 578262 | 5503735 | 1 | 15.41 | Local |
| M2 | 11/22/2011 0:00 | 18 | 49.680950 | -109.915000 | 578271 | 5503723 | 0 | 59.94 | Local |
| M2 | 11/23/2011 0:00 | 0 | 49.680510 | -109.914520 | 578307 | 5503675 | 1 | 2563.06 | Local |
| M2 | 11/23/2011 0:00 | 3 | 49.690660 | -109.882620 | 580591 | 5504837 | 1 | 2079.70 | Local |
| M2 | 11/23/2011 0:00 | 6 | 49.675400 | -109.899290 | 579414 | 5503123 | 1 | 712.85 | Local |
| M2 | 11/23/2011 0:00 | 9 | 49.669390 | -109.902730 | 579175 | 5502451 | 1 | 14.09 | Local |
| M2 | 11/23/2011 0:00 | 12 | 49.669490 | -109.902610 | 579184 | 5502462 | 1 | 59.84 | Local |
| M2 | 11/23/2011 0:00 | 15 | 49.669570 | -109.903430 | 579124 | 5502470 | 1 | 2.65 | Local |
| M2 | 11/23/2011 0:00 | 18 | 49.669590 | -109.903450 | 579123 | 5502472 | 1 | 4821.89 | Local |
| M2 | 11/23/2011 0:00 | 21 | 49.704820 | -109.864470 | 581876 | 5506431 | 1 | 1423.35 | Local |
| M2 | 11/24/2011 0:00 | 0 | 49.715780 | -109.854270 | 582593 | 5507661 | 1 | 16.62 | Local |
| M2 | 11/24/2011 0:00 | 3 | 49.715790 | -109.854500 | 582577 | 5507661 | 1 | 15.09 | Local |
| M2 | 11/24/2011 0:00 | 6 | 49.715750 | -109.854300 | 582591 | 5507657 | 0 | 2599.78 | Local |
| M2 | 11/24/2011 0:00 | 12 | 49.697760 | -109.877330 | 580961 | 5505632 | 1 | 38.67 | Local |
| M2 | 11/24/2011 0:00 | 15 | 49.698080 | -109.877120 | 580976 | 5505668 | 1 | 40.67 | Local |
| M2 | 11/24/2011 0:00 | 18 | 49.697720 | -109.877220 | 580969 | 5505628 | 1 | 1152.79 | Local |
| M2 | 11/24/2011 0:00 | 21 | 49.704540 | -109.865180 | 581826 | 5506399 | 1 | 1597.47 | Local |
| M2 | 11/25/2011 0:00 | 0 | 49.716050 | -109.851920 | 582762 | 5507693 | 1 | 1828.64 | Local |
| M2 | 11/25/2011 0:00 | 3 | 49.709960 | -109.828360 | 584471 | 5507042 | 1 | 766.21 | Local |
| M2 | 11/25/2011 0:00 | 6 | 49.703280 | -109.825750 | 584671 | 5506303 | 1 | 406.62 | Local |
| M2 | 11/25/2011 0:00 | 9 | 49.699630 | -109.825400 | 584702 | 5505897 | 1 | 11.69 | Local |
| M2 | 11/25/2011 0:00 | 12 | 49.699730 | -109.825350 | 584706 | 5505908 | 1 | 13.66 | Local |
| M2 | 11/25/2011 0:00 | 15 | 49.699630 | -109.825460 | 584698 | 5505897 | 1 | 195.11 | Local |
| M2 | 11/25/2011 0:00 | 18 | 49.701380 | -109.825260 | 584710 | 5506092 | 1 | 195.94 | Local |
| M2 | 11/25/2011 0:00 | 21 | 49.703120 | -109.825690 | 584676 | 5506285 | 0 | 219.59 | Local |
| M2 | 11/26/2011 0:00 | 6 | 49.701180 | -109.826260 | 584638 | 5506069 | 1 | 6377.60 | Local |
| M2 | 11/26/2011 0:00 | 9 | 49.684270 | -109.910750 | 578573 | 5504097 | 1 | 1022.45 | Local |
| M2 | 11/26/2011 0:00 | 12 | 49.675930 | -109.916720 | 578155 | 5503163 | 1 | 4.95 | Local |
| M2 | 11/26/2011 0:00 | 15 | 49.675970 | -109.916750 | 578153 | 5503168 | 1 | 10.23 | Local |
| M2 | 11/26/2011 0:00 | 18 | 49.675890 | -109.916680 | 578158 | 5503159 | 1 | 2600.93 | Local |
| M2 | 11/26/2011 0:00 | 21 | 49.662340 | -109.887300 | 580300 | 5501684 | 1 | 700.85 | Local |
| M2 | 11/27/2011 0:00 | 0 | 49.657050 | -109.882020 | 580690 | 5501101 | 1 | 13.97 | Local |
| M2 | 11/27/2011 0:00 | 3 | 49.657110 | -109.882190 | 580678 | 5501108 | 1 | 9.45 | Local |
| M2 | 11/27/2011 0:00 | 6 | 49.657100 | -109.882060 | 580687 | 5501107 | 1 | 591.46 | Local |
| M2 | 11/27/2011 0:00 | 9 | 49.652030 | -109.884540 | 580516 | 5500540 | 1 | 21.18 | Local |
| M2 | 11/27/2011 0:00 | 12 | 49.652220 | -109.884520 | 580518 | 5500561 | 1 | 11.69 | Local |

| | | | | | | | | | |
|---|---|---|---|---|---|---|---|---|---|
| M2 | 11/27/2011 0:00 | 15 | 49.652120 | -109.884470 | 580521 | 5500550 | 1 | 6.05 | Local |
| M2 | 11/27/2011 0:00 | 18 | 49.652090 | -109.884540 | 580516 | 5500547 | 1 | 756.21 | Local |
| M2 | 11/27/2011 0:00 | 21 | 49.649740 | -109.874710 | 581230 | 5500296 | 0 | 3398.14 | Local |
| M2 | 11/28/2011 0:00 | 6 | 49.649600 | -109.827640 | 584628 | 5500333 | 1 | 784.21 | Local |
| M2 | 11/28/2011 0:00 | 9 | 49.642910 | -109.831080 | 584391 | 5499585 | 1 | 225.36 | Local |
| M2 | 11/28/2011 0:00 | 12 | 49.641050 | -109.832320 | 584305 | 5499377 | 1 | 100.08 | Local |
| M2 | 11/28/2011 0:00 | 15 | 49.640820 | -109.830980 | 584402 | 5499353 | 1 | 13.53 | Local |
| M2 | 11/28/2011 0:00 | 18 | 49.640930 | -109.830900 | 584407 | 5499365 | 1 | 2569.91 | Local |
| M2 | 11/28/2011 0:00 | 21 | 49.650400 | -109.798430 | 586735 | 5500455 | 1 | 5037.58 | Local |
| M2 | 11/29/2011 0:00 | 0 | 49.680940 | -109.746870 | 590400 | 5503911 | 1 | 275.29 | Local |
| M2 | 11/29/2011 0:00 | 3 | 49.683390 | -109.746320 | 590435 | 5504184 | 1 | 1214.89 | Local |
| M2 | 11/29/2011 0:00 | 6 | 49.682640 | -109.763120 | 589225 | 5504081 | 1 | 7267.00 | Local |
| M2 | 11/29/2011 0:00 | 9 | 49.656940 | -109.855710 | 582589 | 5501117 | 1 | 151.92 | Local |
| M2 | 11/29/2011 0:00 | 12 | 49.657220 | -109.857770 | 582440 | 5501146 | 0 | 246.17 | Local |
| M2 | 11/29/2011 0:00 | 18 | 49.659080 | -109.855920 | 582570 | 5501355 | 1 | 68.66 | Local |
| M2 | 11/29/2011 0:00 | 21 | 49.658900 | -109.855010 | 582636 | 5501336 | 1 | 69.65 | Local |
| M2 | 11/30/2011 0:00 | 0 | 49.658790 | -109.855960 | 582568 | 5501323 | 1 | 1336.99 | Local |
| M2 | 11/30/2011 0:00 | 3 | 49.664760 | -109.839880 | 583718 | 5502004 | 1 | 4553.28 | Local |
| M2 | 11/30/2011 0:00 | 6 | 49.691840 | -109.792540 | 587086 | 5505069 | 1 | 537.69 | Local |
| M2 | 11/30/2011 0:00 | 9 | 49.696120 | -109.789070 | 587329 | 5505549 | 1 | 5.56 | Local |
| M2 | 11/30/2011 0:00 | 12 | 49.696170 | -109.789070 | 587328 | 5505554 | 0 | 351.06 | Local |
| M2 | 11/30/2011 0:00 | 21 | 49.699260 | -109.788070 | 587395 | 5505899 | 1 | 7.82 | Local |
| M2 | 12/01/2011 0:00 | 0 | 49.699190 | -109.788060 | 587396 | 5505891 | 1 | 2.34 | Local |
| M2 | 12/01/2011 0:00 | 3 | 49.699170 | -109.788070 | 587395 | 5505889 | 1 | 374.17 | Local |
| M2 | 12/01/2011 0:00 | 6 | 49.695890 | -109.789230 | 587317 | 5505523 | 0 | 788.40 | Local |
| M2 | 12/01/2011 0:00 | 21 | 49.691530 | -109.797850 | 586704 | 5505028 | 1 | 5262.85 | Local |
| M2 | 12/02/2011 0:00 | 0 | 49.654730 | -109.843720 | 583458 | 5500885 | 1 | 66.60 | Local |
| M2 | 12/02/2011 0:00 | 3 | 49.655060 | -109.844490 | 583402 | 5500921 | 1 | 168.00 | Local |
| M2 | 12/02/2011 0:00 | 6 | 49.653560 | -109.844770 | 583384 | 5500754 | 1 | 629.77 | Local |
| M2 | 12/02/2011 0:00 | 9 | 49.648960 | -109.849860 | 583025 | 5500237 | 0 | 22.56 | Local |
| M2 | 12/02/2011 0:00 | 15 | 49.648870 | -109.849580 | 583045 | 5500227 | 1 | 843.42 | Local |
| M2 | 12/02/2011 0:00 | 18 | 49.641320 | -109.848450 | 583140 | 5499389 | 1 | 564.58 | Local |
| M2 | 12/02/2011 0:00 | 21 | 49.640600 | -109.840710 | 583700 | 5499317 | 1 | 10.64 | Local |
| M2 | 12/03/2011 0:00 | 0 | 49.640510 | -109.840660 | 583703 | 5499308 | 1 | 11.49 | Local |
| M2 | 12/03/2011 0:00 | 3 | 49.640610 | -109.840700 | 583700 | 5499319 | 1 | 15.17 | Local |
| M2 | 12/03/2011 0:00 | 6 | 49.640490 | -109.840800 | 583693 | 5499305 | 1 | 21.43 | Local |
| M2 | 12/03/2011 0:00 | 9 | 49.640680 | -109.840850 | 583689 | 5499326 | 1 | 23.13 | Local |
| M2 | 12/03/2011 0:00 | 12 | 49.640550 | -109.840600 | 583708 | 5499312 | 1 | 5.61 | Local |
| M2 | 12/03/2011 0:00 | 15 | 49.640600 | -109.840610 | 583707 | 5499318 | 1 | 2.17 | Local |
| M2 | 12/03/2011 0:00 | 18 | 49.640600 | -109.840640 | 583705 | 5499318 | 1 | 2.17 | Local |
| M2 | 12/03/2011 0:00 | 21 | 49.640600 | -109.840610 | 583707 | 5499318 | 1 | 2.65 | Local |
| M2 | 12/04/2011 0:00 | 0 | 49.640620 | -109.840590 | 583708 | 5499320 | 0 | 5.73 | Local |
| M2 | 12/04/2011 0:00 | 9 | 49.640580 | -109.840640 | 583705 | 5499315 | 1 | 3.34 | Local |
| M2 | 12/04/2011 0:00 | 12 | 49.640610 | -109.840640 | 583705 | 5499319 | 1 | 3.63 | Local |
| M2 | 12/04/2011 0:00 | 15 | 49.640580 | -109.840620 | 583706 | 5499315 | 1 | 6.19 | Local |
| M2 | 12/04/2011 0:00 | 18 | 49.640560 | -109.840700 | 583701 | 5499313 | 1 | 7.30 | Local |
| M2 | 12/04/2011 0:00 | 21 | 49.640590 | -109.840610 | 583707 | 5499316 | 1 | 1.44 | Local |
| M2 | 12/05/2011 0:00 | 0 | 49.640590 | -109.840630 | 583706 | 5499316 | 1 | 3.78 | Local |
| M2 | 12/05/2011 0:00 | 3 | 49.640600 | -109.840680 | 583702 | 5499318 | 0 | 11.33 | Local |
| M2 | 12/05/2011 0:00 | 9 | 49.640570 | -109.840530 | 583713 | 5499314 | 1 | 11.21 | Local |
| M2 | 12/05/2011 0:00 | 12 | 49.640470 | -109.840550 | 583711 | 5499303 | 1 | 14.27 | Local |
| M2 | 12/05/2011 0:00 | 15 | 49.640590 | -109.840620 | 583706 | 5499316 | 1 | 9.35 | Local |
| M2 | 12/05/2011 0:00 | 18 | 49.640670 | -109.840660 | 583703 | 5499325 | 0 | 4.45 | Local |
| M2 | 12/06/2011 0:00 | 0 | 49.640630 | -109.840660 | 583703 | 5499321 | 0 | 10.61 | Local |
| M2 | 12/06/2011 0:00 | 6 | 49.640550 | -109.840740 | 583698 | 5499312 | 1 | 12.38 | Local |

| | | | | | | | | | | |
|---|---|---|---|---|---|---|---|---|---|---|
| M2 | 12/06/2011 0:00 | 9 | 49.640590 | -109.840580 | 583709 | 5499317 | 1 | 4.51 | Local |
| M2 | 12/06/2011 0:00 | 12 | 49.640550 | -109.840590 | 583708 | 5499312 | 0 | 7.31 | Local |
| M2 | 12/06/2011 0:00 | 18 | 49.640560 | -109.840490 | 583716 | 5499313 | 0 | 15.33 | Local |
| M2 | 12/07/2011 0:00 | 0 | 49.640580 | -109.840700 | 583700 | 5499315 | 0 | 12.38 | Local |
| M2 | 12/07/2011 0:00 | 6 | 49.640540 | -109.840540 | 583712 | 5499311 | 1 | 14.61 | Local |
| M2 | 12/07/2011 0:00 | 9 | 49.640600 | -109.840720 | 583699 | 5499317 | 1 | 7.94 | Local |
| M2 | 12/07/2011 0:00 | 12 | 49.640600 | -109.840610 | 583707 | 5499318 | 1 | 2.43 | Local |
| M2 | 12/07/2011 0:00 | 15 | 49.640610 | -109.840640 | 583705 | 5499319 | 1 | 7.29 | Local |
| M2 | 12/07/2011 0:00 | 18 | 49.640570 | -109.840720 | 583699 | 5499314 | 1 | 9.65 | Local |
| M2 | 12/07/2011 0:00 | 21 | 49.640590 | -109.840590 | 583708 | 5499317 | 1 | 2.43 | Local |
| M2 | 12/08/2011 0:00 | 0 | 49.640600 | -109.840620 | 583706 | 5499318 | 1 | 6.06 | Local |
| M2 | 12/08/2011 0:00 | 3 | 49.640570 | -109.840690 | 583701 | 5499314 | 1 | 3.10 | Local |
| M2 | 12/08/2011 0:00 | 6 | 49.640590 | -109.840660 | 583703 | 5499316 | 1 | 5.47 | Local |
| M2 | 12/08/2011 0:00 | 9 | 49.640620 | -109.840600 | 583708 | 5499320 | 1 | 1.82 | Local |
| M2 | 12/08/2011 0:00 | 12 | 49.640610 | -109.840620 | 583706 | 5499319 | 1 | 3.65 | Local |
| M2 | 12/08/2011 0:00 | 15 | 49.640590 | -109.840580 | 583709 | 5499317 | 1 | 5.88 | Local |
| M2 | 12/08/2011 0:00 | 18 | 49.640580 | -109.840660 | 583703 | 5499315 | 1 | 262.07 | Local |
| M2 | 12/08/2011 0:00 | 21 | 49.640280 | -109.844260 | 583444 | 5499278 | 1 | 174.65 | Local |
| M2 | 12/09/2011 0:00 | 0 | 49.640560 | -109.846640 | 583272 | 5499306 | 1 | 483.91 | Local |
| M2 | 12/09/2011 0:00 | 3 | 49.640460 | -109.839940 | 583756 | 5499303 | 1 | 49.55 | Local |
| M2 | 12/09/2011 0:00 | 6 | 49.640520 | -109.840620 | 583706 | 5499309 | 1 | 7.95 | Local |
| M2 | 12/09/2011 0:00 | 9 | 49.640580 | -109.840560 | 583711 | 5499315 | 1 | 228.14 | Local |
| M2 | 12/09/2011 0:00 | 12 | 49.642570 | -109.841330 | 583652 | 5499536 | 1 | 8.48 | Local |
| M2 | 12/09/2011 0:00 | 15 | 49.642610 | -109.841230 | 583659 | 5499540 | 1 | 235.29 | Local |
| M2 | 12/09/2011 0:00 | 18 | 49.640530 | -109.840630 | 583706 | 5499310 | 1 | 3.98 | Local |
| M2 | 12/09/2011 0:00 | 21 | 49.640560 | -109.840660 | 583703 | 5499313 | 1 | 8.37 | Local |
| M2 | 12/10/2011 0:00 | 0 | 49.640620 | -109.840590 | 583708 | 5499320 | 1 | 14.53 | Local |
| M2 | 12/10/2011 0:00 | 3 | 49.640490 | -109.840570 | 583710 | 5499305 | 1 | 20.34 | Local |
| M2 | 12/10/2011 0:00 | 6 | 49.640670 | -109.840620 | 583706 | 5499325 | 1 | 14.84 | Local |
| M2 | 12/10/2011 0:00 | 9 | 49.640550 | -109.840530 | 583713 | 5499312 | 1 | 9.83 | Local |
| M2 | 12/10/2011 0:00 | 12 | 49.640490 | -109.840430 | 583720 | 5499306 | 1 | 19.93 | Local |
| M2 | 12/10/2011 0:00 | 15 | 49.640620 | -109.840620 | 583706 | 5499320 | 1 | 8.66 | Local |
| M2 | 12/10/2011 0:00 | 18 | 49.640620 | -109.840740 | 583698 | 5499320 | 0 | 9.74 | Local |
| M2 | 12/11/2011 0:00 | 0 | 49.640580 | -109.840620 | 583706 | 5499315 | 0 | 4.47 | Local |
| M2 | 12/11/2011 0:00 | 9 | 49.640570 | -109.840560 | 583711 | 5499314 | 1 | 2.89 | Local |
| M2 | 12/11/2011 0:00 | 12 | 49.640570 | -109.840600 | 583708 | 5499314 | 1 | 2.34 | Local |
| M2 | 12/11/2011 0:00 | 15 | 49.640590 | -109.840590 | 583708 | 5499317 | 1 | 11.04 | Local |
| M2 | 12/11/2011 0:00 | 18 | 49.640550 | -109.840730 | 583698 | 5499312 | 1 | 9.69 | Local |
| M2 | 12/11/2011 0:00 | 21 | 49.640600 | -109.840620 | 583706 | 5499318 | 1 | 3.61 | Local |
| M2 | 12/12/2011 0:00 | 0 | 49.640600 | -109.840570 | 583710 | 5499318 | 1 | 3.78 | Local |
| M2 | 12/12/2011 0:00 | 3 | 49.640610 | -109.840620 | 583706 | 5499319 | 1 | 4.95 | Local |
| M2 | 12/12/2011 0:00 | 6 | 49.640570 | -109.840590 | 583708 | 5499314 | 1 | 4.41 | Local |
| M2 | 12/12/2011 0:00 | 9 | 49.640600 | -109.840630 | 583705 | 5499318 | 1 | 3.78 | Local |
| M2 | 12/12/2011 0:00 | 12 | 49.640590 | -109.840580 | 583709 | 5499317 | 1 | 1.33 | Local |
| M2 | 12/12/2011 0:00 | 15 | 49.640580 | -109.840590 | 583708 | 5499315 | 1 | 13.19 | Local |
| M2 | 12/12/2011 0:00 | 18 | 49.640560 | -109.840770 | 583695 | 5499313 | 1 | 11.33 | Local |
| M2 | 12/12/2011 0:00 | 21 | 49.640590 | -109.840620 | 583706 | 5499316 | 1 | 3.61 | Local |
| M2 | 12/13/2011 0:00 | 0 | 49.640590 | -109.840670 | 583703 | 5499316 | 1 | 2.65 | Local |
| M2 | 12/13/2011 0:00 | 3 | 49.640570 | -109.840690 | 583701 | 5499314 | 1 | 9.39 | Local |
| M2 | 12/13/2011 0:00 | 6 | 49.640570 | -109.840560 | 583711 | 5499314 | 1 | 9.65 | Local |
| M2 | 12/13/2011 0:00 | 9 | 49.640590 | -109.840690 | 583701 | 5499316 | 1 | 11.04 | Local |
| M2 | 12/13/2011 0:00 | 12 | 49.640550 | -109.840550 | 583711 | 5499312 | 0 | 1075.11 | Local |
| M2 | 12/13/2011 0:00 | 21 | 49.647060 | -109.829540 | 584495 | 5500048 | 1 | 55.69 | Local |
| M2 | 12/14/2011 0:00 | 0 | 49.647550 | -109.829380 | 584506 | 5500103 | 1 | 14.58 | Local |
| M2 | 12/14/2011 0:00 | 3 | 49.647440 | -109.829490 | 584498 | 5500090 | 1 | 23.75 | Local |

| | | | | | | | | | |
|---|---|---|---|---|---|---|---|---|---|
| M2 | 12/14/2011 0:00 | 6 | 49.647650 | -109.829550 | 584493 | 5500114 | 1 | 3.10 | Local |
| M2 | 12/14/2011 0:00 | 9 | 49.647630 | -109.829580 | 584491 | 5500111 | 1 | 11.54 | Local |
| M2 | 12/14/2011 0:00 | 12 | 49.647580 | -109.829440 | 584501 | 5500106 | 1 | 10.62 | Local |
| M2 | 12/14/2011 0:00 | 15 | 49.647650 | -109.829540 | 584494 | 5500114 | 1 | 1.33 | Local |
| M2 | 12/14/2011 0:00 | 18 | 49.647660 | -109.829530 | 584495 | 5500115 | 1 | 4.68 | Local |
| M2 | 12/14/2011 0:00 | 21 | 49.647620 | -109.829550 | 584493 | 5500110 | 1 | 0.00 | Local |
| M2 | 12/15/2011 0:00 | 0 | 49.647620 | -109.829550 | 584493 | 5500110 | 1 | 3.34 | Local |
| M2 | 12/15/2011 0:00 | 3 | 49.647590 | -109.829550 | 584493 | 5500107 | 1 | 10.90 | Local |
| M2 | 12/15/2011 0:00 | 6 | 49.647680 | -109.829490 | 584498 | 5500117 | 1 | 5.97 | Local |
| M2 | 12/15/2011 0:00 | 9 | 49.647630 | -109.829520 | 584495 | 5500112 | 1 | 3.41 | Local |
| M2 | 12/15/2011 0:00 | 12 | 49.647660 | -109.829510 | 584496 | 5500115 | 1 | 4.51 | Local |
| M2 | 12/15/2011 0:00 | 15 | 49.647620 | -109.829520 | 584495 | 5500110 | 1 | 3.64 | Local |
| M2 | 12/15/2011 0:00 | 18 | 49.647590 | -109.829540 | 584494 | 5500107 | 1 | 4.68 | Local |
| M2 | 12/15/2011 0:00 | 21 | 49.647630 | -109.829520 | 584495 | 5500112 | 0 | 3.34 | Local |
| M2 | 12/16/2011 0:00 | 3 | 49.647660 | -109.829520 | 584495 | 5500115 | 1 | 1.11 | Local |
| M2 | 12/16/2011 0:00 | 6 | 49.647650 | -109.829520 | 584495 | 5500114 | 1 | 0.00 | Local |
| M2 | 12/16/2011 0:00 | 9 | 49.647650 | -109.829520 | 584495 | 5500114 | 1 | 3.34 | Local |
| M2 | 12/16/2011 0:00 | 12 | 49.647620 | -109.829520 | 584495 | 5500110 | 1 | 7.82 | Local |
| M2 | 12/16/2011 0:00 | 15 | 49.647550 | -109.829510 | 584496 | 5500103 | 0 | 8.08 | Local |
| M2 | 12/17/2011 0:00 | 0 | 49.647480 | -109.829480 | 584499 | 5500095 | 1 | 12.23 | Local |
| M2 | 12/17/2011 0:00 | 3 | 49.647590 | -109.829480 | 584498 | 5500107 | 1 | 11.14 | Local |
| M2 | 12/17/2011 0:00 | 6 | 49.647690 | -109.829470 | 584499 | 5500118 | 0 | 15.52 | Local |
| M2 | 12/17/2011 0:00 | 12 | 49.647590 | -109.829620 | 584488 | 5500107 | 1 | 4.92 | Local |
| M2 | 12/17/2011 0:00 | 15 | 49.647620 | -109.829570 | 584492 | 5500110 | 1 | 2.34 | Local |
| M2 | 12/17/2011 0:00 | 18 | 49.647600 | -109.829580 | 584491 | 5500108 | 1 | 10.42 | Local |
| M2 | 12/17/2011 0:00 | 21 | 49.647690 | -109.829540 | 584494 | 5500118 | 1 | 3.64 | Local |
| M2 | 12/18/2011 0:00 | 0 | 49.647710 | -109.829580 | 584491 | 5500120 | 1 | 7.94 | Local |
| M2 | 12/18/2011 0:00 | 3 | 49.647710 | -109.829470 | 584499 | 5500121 | 0 | 6.26 | Local |
| M2 | 12/18/2011 0:00 | 9 | 49.647660 | -109.829510 | 584496 | 5500115 | 1 | 11.12 | Local |
| M2 | 12/18/2011 0:00 | 12 | 49.647730 | -109.829620 | 584488 | 5500123 | 1 | 3.78 | Local |
| M2 | 12/18/2011 0:00 | 15 | 49.647720 | -109.829570 | 584492 | 5500122 | 1 | 4.95 | Local |
| M2 | 12/18/2011 0:00 | 18 | 49.647760 | -109.829600 | 584489 | 5500126 | 1 | 3.78 | Local |
| M2 | 12/18/2011 0:00 | 21 | 49.647770 | -109.829550 | 584493 | 5500127 | 1 | 9.15 | Local |
| M2 | 12/19/2011 0:00 | 0 | 49.647690 | -109.829580 | 584491 | 5500118 | 1 | 13.65 | Local |
| M2 | 12/19/2011 0:00 | 3 | 49.647810 | -109.829540 | 584494 | 5500132 | 1 | 12.57 | Local |
| M2 | 12/19/2011 0:00 | 6 | 49.647700 | -109.829580 | 584491 | 5500119 | 1 | 2.65 | Local |
| M2 | 12/19/2011 0:00 | 9 | 49.647720 | -109.829560 | 584492 | 5500122 | 1 | 1.44 | Local |
| M2 | 12/19/2011 0:00 | 12 | 49.647720 | -109.829580 | 584491 | 5500121 | 1 | 6.83 | Local |
| M2 | 12/19/2011 0:00 | 15 | 49.647780 | -109.829560 | 584492 | 5500128 | 1 | 3.98 | Local |
| M2 | 12/19/2011 0:00 | 18 | 49.647750 | -109.829530 | 584495 | 5500125 | 0 | 8.30 | Local |
| M2 | 12/20/2011 0:00 | 3 | 49.647680 | -109.829490 | 584498 | 5500117 | 1 | 17.85 | Local |
| M2 | 12/20/2011 0:00 | 6 | 49.647840 | -109.829510 | 584496 | 5500135 | 1 | 17.92 | Local |
| M2 | 12/20/2011 0:00 | 9 | 49.647680 | -109.829540 | 584494 | 5500117 | 1 | 2.34 | Local |
| M2 | 12/20/2011 0:00 | 12 | 49.647700 | -109.829550 | 584493 | 5500119 | 0 | 0.72 | Local |
| M2 | 12/20/2011 0:00 | 18 | 49.647700 | -109.829560 | 584492 | 5500119 | 1 | 0.72 | Local |
| M2 | 12/20/2011 0:00 | 21 | 49.647700 | -109.829550 | 584493 | 5500119 | 1 | 310.06 | Local |
| M2 | 12/21/2011 0:00 | 0 | 49.650280 | -109.831180 | 584371 | 5500404 | 1 | 330.49 | Local |
| M2 | 12/21/2011 0:00 | 3 | 49.647430 | -109.829880 | 584470 | 5500089 | 0 | 31.82 | Local |
| M2 | 12/21/2011 0:00 | 9 | 49.647670 | -109.829640 | 584487 | 5500116 | 1 | 12.57 | Local |
| M2 | 12/21/2011 0:00 | 12 | 49.647780 | -109.829600 | 584489 | 5500128 | 1 | 16.49 | Local |
| M2 | 12/21/2011 0:00 | 15 | 49.647740 | -109.829820 | 584474 | 5500123 | 1 | 19.62 | Local |
| M2 | 12/21/2011 0:00 | 18 | 49.647720 | -109.829550 | 584493 | 5500122 | 0 | 6.05 | Local |
| M2 | 12/22/2011 0:00 | 3 | 49.647750 | -109.829620 | 584488 | 5500125 | 1 | 9.11 | Local |
| M2 | 12/22/2011 0:00 | 6 | 49.647700 | -109.829520 | 584495 | 5500119 | 0 | 5.47 | Local |
| M2 | 12/22/2011 0:00 | 12 | 49.647730 | -109.829580 | 584491 | 5500123 | 1 | 0.00 | Local |

| | | | | | | | | | |
|---|---|---|---|---|---|---|---|---|---|
| M2 | 12/22/2011 0:00 | 15 | 49.647730 | -109.829580 | 584491 | 5500123 | 1 | 4.45 | Local |
| M2 | 12/22/2011 0:00 | 18 | 49.647690 | -109.829580 | 584491 | 5500118 | 1 | 5029.92 | Local |
| M2 | 12/22/2011 0:00 | 21 | 49.692770 | -109.823760 | 584833 | 5505136 | 1 | 260.76 | Local |
| M2 | 12/23/2011 0:00 | 0 | 49.694980 | -109.822550 | 584916 | 5505384 | 1 | 1.33 | Local |
| M2 | 12/23/2011 0:00 | 3 | 49.694970 | -109.822560 | 584915 | 5505382 | 1 | 565.49 | Local |
| M2 | 12/23/2011 0:00 | 6 | 49.699840 | -109.824820 | 584744 | 5505921 | 1 | 332.15 | Local |
| M2 | 12/23/2011 0:00 | 9 | 49.702820 | -109.825140 | 584716 | 5506252 | 1 | 131.56 | Local |
| M2 | 12/23/2011 0:00 | 12 | 49.703860 | -109.826010 | 584651 | 5506367 | 1 | 3.63 | Local |
| M2 | 12/23/2011 0:00 | 15 | 49.703890 | -109.825990 | 584653 | 5506370 | 1 | 9.01 | Local |
| M2 | 12/23/2011 0:00 | 18 | 49.703810 | -109.826010 | 584651 | 5506361 | 1 | 140.76 | Local |
| M2 | 12/23/2011 0:00 | 21 | 49.703000 | -109.824510 | 584761 | 5506273 | 1 | 3.09 | Local |
| M2 | 12/24/2011 0:00 | 0 | 49.702990 | -109.824470 | 584764 | 5506272 | 1 | 294.14 | Local |
| M2 | 12/24/2011 0:00 | 3 | 49.705530 | -109.825610 | 584677 | 5506553 | 1 | 21.75 | Local |
| M2 | 12/24/2011 0:00 | 6 | 49.705550 | -109.825310 | 584699 | 5506556 | 0 | 15.74 | Local |
| M2 | 12/24/2011 0:00 | 18 | 49.705470 | -109.825490 | 584686 | 5506546 | 0 | 46.78 | Local |
| M2 | 12/25/2011 0:00 | 0 | 49.705730 | -109.824980 | 584722 | 5506576 | 0 | 2.43 | Local |
| M2 | 12/25/2011 0:00 | 6 | 49.705720 | -109.824950 | 584724 | 5506575 | 1 | 7.51 | Local |
| M2 | 12/25/2011 0:00 | 9 | 49.705670 | -109.824880 | 584729 | 5506569 | 0 | 47.67 | Local |
| M2 | 12/25/2011 0:00 | 18 | 49.705490 | -109.825480 | 584687 | 5506549 | 1 | 380.19 | Local |
| M2 | 12/25/2011 0:00 | 21 | 49.708720 | -109.823750 | 584806 | 5506910 | 1 | 109.50 | Local |
| M2 | 12/26/2011 0:00 | 0 | 49.707740 | -109.823900 | 584797 | 5506801 | 0 | 11.45 | Local |
| M2 | 12/26/2011 0:00 | 6 | 49.707820 | -109.824000 | 584789 | 5506809 | 1 | 3.10 | Local |
| M2 | 12/26/2011 0:00 | 9 | 49.707800 | -109.823970 | 584791 | 5506807 | 1 | 8.82 | Local |
| M2 | 12/26/2011 0:00 | 12 | 49.707860 | -109.824050 | 584786 | 5506814 | 1 | 11.45 | Local |
| M2 | 12/26/2011 0:00 | 15 | 49.707780 | -109.823950 | 584793 | 5506805 | 1 | 10.36 | Local |
| M2 | 12/26/2011 0:00 | 18 | 49.707840 | -109.823840 | 584801 | 5506812 | 1 | 9.44 | Local |
| M2 | 12/26/2011 0:00 | 21 | 49.707830 | -109.823970 | 584791 | 5506811 | 1 | 7.04 | Local |
| M2 | 12/27/2011 0:00 | 0 | 49.707780 | -109.824030 | 584787 | 5506805 | 1 | 93.80 | Local |
| M2 | 12/27/2011 0:00 | 3 | 49.707810 | -109.822730 | 584881 | 5506810 | 1 | 128.67 | Local |
| M2 | 12/27/2011 0:00 | 6 | 49.706760 | -109.823480 | 584829 | 5506692 | 0 | 158.52 | Local |
| M2 | 12/27/2011 0:00 | 18 | 49.705730 | -109.825000 | 584721 | 5506576 | 1 | 5.73 | Local |
| M2 | 12/27/2011 0:00 | 21 | 49.705690 | -109.824950 | 584724 | 5506571 | 1 | 379.29 | Local |
| M2 | 12/28/2011 0:00 | 0 | 49.709070 | -109.825660 | 584667 | 5506946 | 1 | 14.74 | Local |
| M2 | 12/28/2011 0:00 | 3 | 49.708940 | -109.825700 | 584665 | 5506932 | 1 | 9.60 | Local |
| M2 | 12/28/2011 0:00 | 6 | 49.709020 | -109.825650 | 584668 | 5506941 | 1 | 288.50 | Local |
| M2 | 12/28/2011 0:00 | 9 | 49.706920 | -109.823300 | 584841 | 5506710 | 0 | 22.57 | Local |
| M2 | 12/28/2011 0:00 | 15 | 49.706730 | -109.823410 | 584834 | 5506689 | 0 | 524.34 | Local |
| M2 | 12/28/2011 0:00 | 21 | 49.711440 | -109.823050 | 584851 | 5507213 | 1 | 747.70 | Local |
| M2 | 12/29/2011 0:00 | 0 | 49.716210 | -109.830360 | 584316 | 5507735 | 1 | 255.14 | Local |
| M2 | 12/29/2011 0:00 | 3 | 49.716890 | -109.826980 | 584559 | 5507814 | 1 | 3.63 | Local |
| M2 | 12/29/2011 0:00 | 6 | 49.716920 | -109.827000 | 584557 | 5507818 | 1 | 2.65 | Local |
| M2 | 12/29/2011 0:00 | 9 | 49.716900 | -109.827020 | 584556 | 5507815 | 1 | 3.77 | Local |
| M2 | 12/29/2011 0:00 | 12 | 49.716890 | -109.826970 | 584559 | 5507814 | 1 | 2.34 | Local |
| M2 | 12/29/2011 0:00 | 15 | 49.716870 | -109.826980 | 584559 | 5507812 | 1 | 5.74 | Local |
| M2 | 12/29/2011 0:00 | 18 | 49.716920 | -109.827000 | 584557 | 5507818 | 1 | 146.03 | Local |
| M2 | 12/29/2011 0:00 | 21 | 49.716400 | -109.828860 | 584424 | 5507758 | 1 | 6.63 | Local |
| M2 | 12/30/2011 0:00 | 0 | 49.716350 | -109.828910 | 584420 | 5507752 | 1 | 137.74 | Local |
| M2 | 12/30/2011 0:00 | 3 | 49.716940 | -109.827230 | 584540 | 5507820 | 1 | 166.00 | Local |
| M2 | 12/30/2011 0:00 | 6 | 49.716690 | -109.829500 | 584377 | 5507789 | 1 | 0.72 | Local |
| M2 | 12/30/2011 0:00 | 9 | 49.716690 | -109.829510 | 584377 | 5507789 | 1 | 2.22 | Local |
| M2 | 12/30/2011 0:00 | 12 | 49.716710 | -109.829510 | 584377 | 5507792 | 1 | 1.82 | Local |
| M2 | 12/30/2011 0:00 | 15 | 49.716720 | -109.829530 | 584375 | 5507793 | 1 | 1.82 | Local |
| M2 | 12/30/2011 0:00 | 18 | 49.716710 | -109.829510 | 584377 | 5507792 | 1 | 2358.45 | Local |
| M2 | 12/30/2011 0:00 | 21 | 49.715110 | -109.862130 | 582028 | 5507577 | 1 | 11.01 | Local |
| M2 | 12/31/2011 0:00 | 0 | 49.715190 | -109.862220 | 582021 | 5507586 | 0 | 3.63 | Local |

| | | | | | | | | | |
|---|---|---|---|---|---|---|---|---|---|
| M2 | 12/31/2011 0:00 | 6 | 49.715220 | -109.862200 | 582023 | 5507590 | 1 | 4.95 | Local |
| M2 | 12/31/2011 0:00 | 9 | 49.715260 | -109.862230 | 582020 | 5507594 | 1 | 5.61 | Local |
| M2 | 12/31/2011 0:00 | 12 | 49.715210 | -109.862220 | 582021 | 5507589 | 1 | 4.45 | Local |
| M2 | 12/31/2011 0:00 | 15 | 49.715250 | -109.862220 | 582021 | 5507593 | 1 | 7.78 | Local |
| M2 | 12/31/2011 0:00 | 18 | 49.715320 | -109.862220 | 582021 | 5507601 | 1 | 11.12 | Local |
| M2 | 12/31/2011 0:00 | 21 | 49.715220 | -109.862220 | 582021 | 5507590 | 1 | 1.33 | Local |
| M2 | 01/01/2012 0:00 | 0 | 49.715230 | -109.862210 | 582022 | 5507591 | 1 | 1.11 | Local |
| M2 | 01/01/2012 0:00 | 3 | 49.715240 | -109.862210 | 582022 | 5507592 | 1 | 2.88 | Local |
| M2 | 01/01/2012 0:00 | 6 | 49.715240 | -109.862250 | 582019 | 5507592 | 1 | 4.51 | Local |
| M2 | 01/01/2012 0:00 | 9 | 49.715200 | -109.862240 | 582020 | 5507587 | 1 | 5.56 | Local |
| M2 | 01/01/2012 0:00 | 12 | 49.715250 | -109.862240 | 582020 | 5507593 | 1 | 1.44 | Local |
| M2 | 01/01/2012 0:00 | 15 | 49.715250 | -109.862220 | 582021 | 5507593 | 1 | 7.78 | Local |
| M2 | 01/01/2012 0:00 | 18 | 49.715180 | -109.862220 | 582021 | 5507585 | 1 | 1169.72 | Local |
| M2 | 01/01/2012 0:00 | 21 | 49.704750 | -109.864340 | 581886 | 5506423 | 1 | 2.34 | Local |
| M2 | 01/02/2012 0:00 | 0 | 49.704770 | -109.864330 | 581887 | 5506426 | 1 | 3.64 | Local |
| M2 | 01/02/2012 0:00 | 3 | 49.704750 | -109.864290 | 581890 | 5506423 | 1 | 3.09 | Local |
| M2 | 01/02/2012 0:00 | 6 | 49.704760 | -109.864330 | 581887 | 5506424 | 1 | 350.65 | Local |
| M2 | 01/02/2012 0:00 | 9 | 49.702640 | -109.867930 | 581631 | 5506185 | 1 | 15.14 | Local |
| M2 | 01/02/2012 0:00 | 12 | 49.702640 | -109.868140 | 581615 | 5506185 | 1 | 2.43 | Local |
| M2 | 01/02/2012 0:00 | 15 | 49.702630 | -109.868110 | 581618 | 5506184 | 1 | 2.43 | Local |
| M2 | 01/02/2012 0:00 | 18 | 49.702620 | -109.868080 | 581620 | 5506182 | 1 | 1.11 | Local |
| M2 | 01/02/2012 0:00 | 21 | 49.702610 | -109.868080 | 581620 | 5506181 | 1 | 11.12 | Local |
| M2 | 01/03/2012 0:00 | 0 | 49.702510 | -109.868080 | 581620 | 5506170 | 1 | 19.56 | Local |
| M2 | 01/03/2012 0:00 | 3 | 49.702680 | -109.868010 | 581625 | 5506189 | 1 | 12.18 | Local |
| M2 | 01/03/2012 0:00 | 6 | 49.702610 | -109.868140 | 581616 | 5506181 | 1 | 7.55 | Local |
| M2 | 01/03/2012 0:00 | 9 | 49.702630 | -109.868040 | 581623 | 5506184 | 1 | 4912.01 | Local |
| M3 | 06/16/2011 0:00 | 21 | 49.638254 | -109.683980 | 595020 | 5499243 | 1 | 2045.50 | Trans |
| M3 | 06/17/2011 0:00 | 0 | 49.634899 | -109.711831 | 593015 | 5498835 | 0 | 8600.11 | Trans |
| M3 | 06/17/2011 0:00 | 6 | 49.618708 | -109.828263 | 584636 | 5496898 | 1 | 12.22 | Trans |
| M3 | 06/17/2011 0:00 | 9 | 49.618720 | -109.828431 | 584624 | 5496899 | 0 | 1058.72 | Trans |
| M3 | 06/17/2011 0:00 | 15 | 49.617106 | -109.842874 | 583584 | 5496703 | 1 | 13.30 | Trans |
| M3 | 06/17/2011 0:00 | 18 | 49.616986 | -109.842883 | 583583 | 5496690 | 1 | 2181.32 | Trans |
| M3 | 06/17/2011 0:00 | 21 | 49.612055 | -109.872107 | 581481 | 5496110 | 0 | 8824.33 | Trans |
| M3 | 06/18/2011 0:00 | 3 | 49.549517 | -109.947264 | 576148 | 5489078 | 1 | 2097.20 | Trans |
| M3 | 06/18/2011 0:00 | 6 | 49.530787 | -109.943842 | 576425 | 5486999 | 1 | 1030.93 | Trans |
| M3 | 06/18/2011 0:00 | 9 | 49.539953 | -109.941690 | 576567 | 5488021 | 1 | 293.89 | Trans |
| M3 | 06/18/2011 0:00 | 12 | 49.539511 | -109.945694 | 576278 | 5487967 | 1 | 11.86 | Trans |
| M3 | 06/18/2011 0:00 | 15 | 49.539414 | -109.945627 | 576283 | 5487957 | 1 | 8.34 | Trans |
| M3 | 06/18/2011 0:00 | 18 | 49.539483 | -109.945671 | 576279 | 5487964 | 1 | 1.98 | Trans |
| M3 | 06/18/2011 0:00 | 21 | 49.539467 | -109.945681 | 576279 | 5487963 | 1 | 4501.26 | Trans |
| M3 | 06/19/2011 0:00 | 0 | 49.499454 | -109.936206 | 577027 | 5483524 | 1 | 2.14 | Trans |
| M3 | 06/19/2011 0:00 | 3 | 49.499435 | -109.936213 | 577026 | 5483522 | 1 | 2976.80 | Trans |
| M3 | 06/19/2011 0:00 | 6 | 49.526207 | -109.936697 | 576949 | 5486498 | 1 | 2605.05 | Trans |
| M3 | 06/19/2011 0:00 | 9 | 49.549500 | -109.940593 | 576631 | 5489083 | 1 | 92.53 | Trans |
| M3 | 06/19/2011 0:00 | 12 | 49.550329 | -109.940711 | 576621 | 5489175 | 1 | 11.47 | Trans |
| M3 | 06/19/2011 0:00 | 15 | 49.550262 | -109.940590 | 576630 | 5489168 | 1 | 438.24 | Trans |
| M3 | 06/19/2011 0:00 | 18 | 49.554166 | -109.939758 | 576684 | 5489603 | 1 | 4.52 | Trans |
| M3 | 06/19/2011 0:00 | 21 | 49.554206 | -109.939746 | 576685 | 5489607 | 1 | 77.13 | Trans |
| M3 | 06/20/2011 0:00 | 0 | 49.553541 | -109.939441 | 576708 | 5489534 | 1 | 15.49 | Trans |
| M3 | 06/20/2011 0:00 | 3 | 49.553668 | -109.939528 | 576702 | 5489548 | 1 | 14.62 | Trans |
| M3 | 06/20/2011 0:00 | 6 | 49.553546 | -109.939453 | 576707 | 5489534 | 0 | 94.97 | Trans |
| M3 | 06/20/2011 0:00 | 12 | 49.553029 | -109.940498 | 576632 | 5489476 | 1 | 24.28 | Trans |
| M3 | 06/20/2011 0:00 | 15 | 49.553106 | -109.940184 | 576655 | 5489484 | 1 | 13.32 | Trans |
| M3 | 06/20/2011 0:00 | 18 | 49.553183 | -109.940042 | 576665 | 5489493 | 0 | 13.57 | Trans |
| M3 | 06/21/2011 0:00 | 0 | 49.553061 | -109.940048 | 576665 | 5489480 | 1 | 223.52 | Trans |

| | | | | | | | | | |
|---|---|---|---|---|---|---|---|---|---|
| M3 | 06/21/2011 0:00 | 3 | 49.552102 | -109.937332 | 576863 | 5489376 | 1 | 240.47 | Trans |
| M3 | 06/21/2011 0:00 | 6 | 49.553061 | -109.940311 | 576646 | 5489479 | 1 | 13.26 | Trans |
| M3 | 06/21/2011 0:00 | 9 | 49.552960 | -109.940407 | 576639 | 5489468 | 1 | 874.30 | Trans |
| M3 | 06/21/2011 0:00 | 12 | 49.553333 | -109.928334 | 577512 | 5489522 | 1 | 35.83 | Trans |
| M3 | 06/21/2011 0:00 | 15 | 49.553292 | -109.927843 | 577547 | 5489518 | 1 | 1476.48 | Trans |
| M3 | 06/21/2011 0:00 | 18 | 49.543553 | -109.913968 | 578566 | 5488449 | 1 | 143.90 | Trans |
| M3 | 06/21/2011 0:00 | 21 | 49.544446 | -109.915407 | 578461 | 5488547 | 1 | 6593.49 | Trans |
| M3 | 06/22/2011 0:00 | 0 | 49.568523 | -109.998715 | 572399 | 5491140 | 1 | 6369.17 | Trans |
| M3 | 06/22/2011 0:00 | 3 | 49.592702 | -110.078585 | 566591 | 5493755 | 1 | 4100.67 | Trans |
| M3 | 06/22/2011 0:00 | 6 | 49.612278 | -110.126680 | 563090 | 5495890 | 1 | 8.79 | Trans |
| M3 | 06/22/2011 0:00 | 9 | 49.612281 | -110.126802 | 563081 | 5495890 | 1 | 603.22 | Trans |
| M3 | 06/22/2011 0:00 | 12 | 49.615578 | -110.133433 | 562598 | 5496251 | 1 | 468.17 | Trans |
| M3 | 06/22/2011 0:00 | 15 | 49.615459 | -110.139911 | 562130 | 5496232 | 1 | 332.60 | Trans |
| M3 | 06/22/2011 0:00 | 18 | 49.615488 | -110.144515 | 561797 | 5496232 | 1 | 236.46 | Trans |
| M3 | 06/22/2011 0:00 | 21 | 49.613429 | -110.145333 | 561741 | 5496002 | 1 | 6643.87 | Trans |
| M3 | 06/23/2011 0:00 | 0 | 49.617107 | -110.237128 | 555106 | 5496340 | 1 | 5299.59 | Trans |
| M3 | 06/23/2011 0:00 | 3 | 49.601157 | -110.306251 | 550129 | 5494518 | 1 | 180.47 | Trans |
| M3 | 06/23/2011 0:00 | 6 | 49.602600 | -110.307395 | 550045 | 5494678 | 1 | 100.30 | Trans |
| M3 | 06/23/2011 0:00 | 9 | 49.603502 | -110.307399 | 550044 | 5494778 | 1 | 63.66 | Trans |
| M3 | 06/23/2011 0:00 | 12 | 49.603157 | -110.308101 | 549993 | 5494739 | 1 | 332.02 | Trans |
| M3 | 06/23/2011 0:00 | 15 | 49.600543 | -110.305878 | 550156 | 5494450 | 1 | 356.14 | Trans |
| M3 | 06/23/2011 0:00 | 18 | 49.602208 | -110.310089 | 549851 | 5494633 | 1 | 492.47 | Trans |
| M3 | 06/23/2011 0:00 | 21 | 49.601478 | -110.316811 | 549366 | 5494547 | 1 | 886.22 | Trans |
| M3 | 06/24/2011 0:00 | 0 | 49.595309 | -110.309044 | 549933 | 5493866 | 1 | 2130.04 | Trans |
| M3 | 06/24/2011 0:00 | 3 | 49.594955 | -110.338513 | 547804 | 5493808 | 1 | 576.02 | Trans |
| M3 | 06/24/2011 0:00 | 6 | 49.591988 | -110.345047 | 547334 | 5493474 | 1 | 30.88 | Trans |
| M3 | 06/24/2011 0:00 | 9 | 49.591720 | -110.344934 | 547343 | 5493444 | 1 | 158.07 | Trans |
| M3 | 06/24/2011 0:00 | 12 | 49.592321 | -110.342952 | 547486 | 5493512 | 1 | 93.32 | Trans |
| M3 | 06/24/2011 0:00 | 15 | 49.593148 | -110.342730 | 547501 | 5493604 | 1 | 249.26 | Trans |
| M3 | 06/24/2011 0:00 | 18 | 49.591132 | -110.344236 | 547394 | 5493379 | 1 | 51.18 | Trans |
| M3 | 06/24/2011 0:00 | 21 | 49.591587 | -110.344337 | 547386 | 5493430 | 1 | 800.36 | Trans |
| M3 | 06/25/2011 0:00 | 0 | 49.584747 | -110.347788 | 547143 | 5492667 | 1 | 9.05 | Trans |
| M3 | 06/25/2011 0:00 | 3 | 49.584726 | -110.347909 | 547135 | 5492665 | 1 | 635.66 | Trans |
| M3 | 06/25/2011 0:00 | 6 | 49.590166 | -110.345200 | 547325 | 5493271 | 1 | 2.50 | Trans |
| M3 | 06/25/2011 0:00 | 9 | 49.590147 | -110.345218 | 547324 | 5493269 | 0 | 155.63 | Trans |
| M3 | 06/25/2011 0:00 | 15 | 49.591428 | -110.344350 | 547385 | 5493412 | 1 | 33.70 | Trans |
| M3 | 06/25/2011 0:00 | 18 | 49.591153 | -110.344152 | 547400 | 5493381 | 1 | 59.04 | Trans |
| M3 | 06/25/2011 0:00 | 21 | 49.591184 | -110.344967 | 547341 | 5493384 | 1 | 453.79 | Trans |
| M3 | 06/26/2011 0:00 | 0 | 49.594216 | -110.349171 | 547034 | 5493719 | 1 | 342.07 | Trans |
| M3 | 06/26/2011 0:00 | 3 | 49.592113 | -110.345716 | 547286 | 5493487 | 1 | 361.34 | Trans |
| M3 | 06/26/2011 0:00 | 6 | 49.589111 | -110.347629 | 547151 | 5493152 | 1 | 17.57 | Trans |
| M3 | 06/26/2011 0:00 | 9 | 49.589245 | -110.347758 | 547141 | 5493167 | 1 | 5.94 | Trans |
| M3 | 06/26/2011 0:00 | 12 | 49.589243 | -110.347840 | 547135 | 5493167 | 1 | 483.67 | Trans |
| M3 | 06/26/2011 0:00 | 15 | 49.586250 | -110.352696 | 546787 | 5492831 | 1 | 675.88 | Trans |
| M3 | 06/26/2011 0:00 | 18 | 49.581687 | -110.358875 | 546345 | 5492320 | 1 | 105.59 | Trans |
| M3 | 06/26/2011 0:00 | 21 | 49.582184 | -110.360120 | 546254 | 5492374 | 1 | 78.99 | Trans |
| M3 | 06/27/2011 0:00 | 0 | 49.582101 | -110.359034 | 546333 | 5492366 | 1 | 201.06 | Trans |
| M3 | 06/27/2011 0:00 | 3 | 49.581617 | -110.361714 | 546140 | 5492310 | 1 | 103.95 | Trans |
| M3 | 06/27/2011 0:00 | 6 | 49.582482 | -110.361167 | 546178 | 5492407 | 1 | 2.21 | Trans |
| M3 | 06/27/2011 0:00 | 9 | 49.582497 | -110.361148 | 546180 | 5492408 | 1 | 99.60 | Trans |
| M3 | 06/27/2011 0:00 | 12 | 49.582895 | -110.362382 | 546090 | 5492452 | 1 | 139.19 | Trans |
| M3 | 06/27/2011 0:00 | 15 | 49.583980 | -110.363341 | 546020 | 5492572 | 0 | 2739.72 | Trans |
| M3 | 06/27/2011 0:00 | 21 | 49.601422 | -110.390121 | 544068 | 5494495 | 1 | 3477.51 | Trans |
| M3 | 06/28/2011 0:00 | 0 | 49.594096 | -110.436904 | 540694 | 5493654 | 1 | 3799.04 | Trans |
| M3 | 06/28/2011 0:00 | 3 | 49.588984 | -110.488877 | 536942 | 5493059 | 1 | 1683.22 | Trans |

| | | | | | | | | | |
|---|---|---|---|---|---|---|---|---|---|
| M3 | 06/28/2011 0:00 | 6 | 49.590159 | -110.512096 | 535263 | 5493179 | 1 | 18.22 | Trans |
| M3 | 06/28/2011 0:00 | 9 | 49.590017 | -110.511968 | 535272 | 5493163 | 0 | 8.01 | Trans |
| M3 | 06/28/2011 0:00 | 15 | 49.590052 | -110.512065 | 535265 | 5493167 | 1 | 8.51 | Trans |
| M3 | 06/28/2011 0:00 | 18 | 49.590116 | -110.512130 | 535261 | 5493174 | 1 | 74.74 | Trans |
| M3 | 06/28/2011 0:00 | 21 | 49.590702 | -110.511622 | 535297 | 5493239 | 1 | 453.79 | Trans |
| M3 | 06/29/2011 0:00 | 0 | 49.593263 | -110.516511 | 534942 | 5493522 | 1 | 5.44 | Trans |
| M3 | 06/29/2011 0:00 | 3 | 49.593256 | -110.516437 | 534947 | 5493521 | 1 | 4371.75 | Trans |
| M3 | 06/29/2011 0:00 | 6 | 49.616122 | -110.565661 | 531375 | 5496041 | 1 | 4279.77 | Trans |
| M3 | 06/29/2011 0:00 | 9 | 49.631198 | -110.511139 | 535302 | 5497742 | 1 | 13.88 | Trans |
| M3 | 06/29/2011 0:00 | 12 | 49.631144 | -110.511312 | 535290 | 5497736 | 1 | 17.75 | Trans |
| M3 | 06/29/2011 0:00 | 15 | 49.631261 | -110.511145 | 535302 | 5497749 | 1 | 4.79 | Trans |
| M3 | 06/29/2011 0:00 | 18 | 49.631243 | -110.511205 | 535298 | 5497747 | 1 | 117.22 | Trans |
| M3 | 06/29/2011 0:00 | 21 | 49.630820 | -110.509719 | 535405 | 5497700 | 1 | 4169.85 | Trans |
| M3 | 06/30/2011 0:00 | 0 | 49.593495 | -110.515390 | 535023 | 5493548 | 1 | 131.69 | Trans |
| M3 | 06/30/2011 0:00 | 3 | 49.592888 | -110.516954 | 534910 | 5493480 | 1 | 3169.49 | Trans |
| M3 | 06/30/2011 0:00 | 6 | 49.613068 | -110.485971 | 537134 | 5495738 | 1 | 1516.97 | Trans |
| M3 | 06/30/2011 0:00 | 9 | 49.621967 | -110.470052 | 538277 | 5496736 | 1 | 75.29 | Trans |
| M3 | 06/30/2011 0:00 | 12 | 49.621351 | -110.470486 | 538246 | 5496667 | 1 | 231.02 | Trans |
| M3 | 06/30/2011 0:00 | 15 | 49.622352 | -110.473289 | 538043 | 5496777 | 1 | 7.49 | Trans |
| M3 | 06/30/2011 0:00 | 18 | 49.622397 | -110.473366 | 538037 | 5496782 | 1 | 17.75 | Trans |
| M3 | 06/30/2011 0:00 | 21 | 49.622239 | -110.473340 | 538039 | 5496764 | 1 | 4583.44 | Trans |
| M3 | 07/01/2011 0:00 | 0 | 49.589149 | -110.511177 | 535330 | 5493067 | 1 | 165.04 | Trans |
| M3 | 07/01/2011 0:00 | 3 | 49.588029 | -110.509678 | 535439 | 5492943 | 1 | 7.25 | Trans |
| M3 | 07/01/2011 0:00 | 6 | 49.587965 | -110.509689 | 535438 | 5492936 | 1 | 281.06 | Trans |
| M3 | 07/01/2011 0:00 | 9 | 49.589996 | -110.512004 | 535270 | 5493161 | 0 | 7.97 | Trans |
| M3 | 07/01/2011 0:00 | 15 | 49.590064 | -110.512037 | 535267 | 5493168 | 1 | 5.30 | Trans |
| M3 | 07/01/2011 0:00 | 18 | 49.590027 | -110.512083 | 535264 | 5493164 | 1 | 284.29 | Trans |
| M3 | 07/01/2011 0:00 | 21 | 49.588001 | -110.509683 | 535439 | 5492940 | 1 | 2190.77 | Trans |
| M3 | 07/02/2011 0:00 | 0 | 49.574789 | -110.532167 | 533823 | 5491461 | 1 | 5100.06 | Trans |
| M3 | 07/02/2011 0:00 | 3 | 49.572091 | -110.602585 | 528733 | 5491132 | 1 | 6231.49 | Trans |
| M3 | 07/02/2011 0:00 | 6 | 49.570098 | -110.688716 | 522507 | 5490881 | 1 | 14.02 | Trans |
| M3 | 07/02/2011 0:00 | 9 | 49.569997 | -110.688600 | 522516 | 5490869 | 1 | 2.02 | Trans |
| M3 | 07/02/2011 0:00 | 12 | 49.569989 | -110.688624 | 522514 | 5490868 | 1 | 3.92 | Trans |
| M3 | 07/02/2011 0:00 | 15 | 49.569995 | -110.688678 | 522510 | 5490869 | 1 | 3.90 | Trans |
| M3 | 07/02/2011 0:00 | 18 | 49.569962 | -110.688660 | 522511 | 5490865 | 1 | 4.84 | Trans |
| M3 | 07/02/2011 0:00 | 21 | 49.570004 | -110.688675 | 522510 | 5490870 | 1 | 3832.59 | Trans |
| M3 | 07/03/2011 0:00 | 0 | 49.564867 | -110.741086 | 518723 | 5490285 | 1 | 6632.54 | Trans |
| M3 | 07/03/2011 0:00 | 3 | 49.577911 | -110.830600 | 512246 | 5491716 | 1 | 6255.33 | Trans |
| M3 | 07/03/2011 0:00 | 6 | 49.575918 | -110.917072 | 505995 | 5491484 | 1 | 112.02 | Trans |
| M3 | 07/03/2011 0:00 | 9 | 49.576355 | -110.918468 | 505894 | 5491533 | 0 | 58.49 | Trans |
| M3 | 07/03/2011 0:00 | 15 | 49.576425 | -110.917667 | 505952 | 5491541 | 1 | 7.30 | Trans |
| M3 | 07/03/2011 0:00 | 18 | 49.576390 | -110.917752 | 505946 | 5491537 | 1 | 360.77 | Trans |
| M3 | 07/03/2011 0:00 | 21 | 49.575922 | -110.922690 | 505589 | 5491484 | 1 | 424.69 | Trans |
| M3 | 07/04/2011 0:00 | 0 | 49.576494 | -110.928498 | 505169 | 5491548 | 1 | 5476.30 | Trans |
| M3 | 07/04/2011 0:00 | 3 | 49.587565 | -111.002318 | 499832 | 5492776 | 0 | 9084.09 | Trans |
| M3 | 07/04/2011 0:00 | 15 | 49.581922 | -111.127693 | 490770 | 5492156 | 0 | 33.00 | Trans |
| M3 | 07/04/2011 0:00 | 21 | 49.582219 | -111.127705 | 490769 | 5492189 | 1 | 83.42 | Trans |
| M3 | 07/05/2011 0:00 | 0 | 49.582964 | -111.127568 | 490779 | 5492272 | 1 | 7510.49 | Trans |
| M3 | 07/05/2011 0:00 | 3 | 49.596127 | -111.229490 | 483416 | 5493753 | 1 | 2912.28 | Trans |
| M3 | 07/05/2011 0:00 | 6 | 49.611920 | -111.261647 | 481098 | 5495517 | 1 | 27.41 | Trans |
| M3 | 07/05/2011 0:00 | 9 | 49.612167 | -111.261644 | 481098 | 5495544 | 0 | 5117.73 | Trans |
| M3 | 07/06/2011 0:00 | 0 | 49.643717 | -111.313245 | 477385 | 5499066 | 1 | 8023.12 | Trans |
| M3 | 07/06/2011 0:00 | 3 | 49.684948 | -111.404488 | 470823 | 5503681 | 1 | 810.08 | Trans |
| M3 | 07/06/2011 0:00 | 6 | 49.690312 | -111.412090 | 470277 | 5504281 | 1 | 55.76 | Trans |
| M3 | 07/06/2011 0:00 | 9 | 49.690810 | -111.412173 | 470272 | 5504336 | 1 | 370.58 | Trans |

| | | | | | | | | | |
|---|---|---|---|---|---|---|---|---|---|
| M3 | 07/06/2011 0:00 | 12 | 49.692338 | -111.416739 | 469943 | 5504508 | 1 | 5.75 | Trans |
| M3 | 07/06/2011 0:00 | 15 | 49.692287 | -111.416753 | 469942 | 5504502 | 1 | 10.45 | Trans |
| M3 | 07/06/2011 0:00 | 18 | 49.692372 | -111.416816 | 469938 | 5504511 | 1 | 3.17 | Trans |
| M3 | 07/06/2011 0:00 | 21 | 49.692377 | -111.416773 | 469941 | 5504512 | 1 | 3482.44 | Trans |
| M3 | 07/07/2011 0:00 | 0 | 49.722993 | -111.426970 | 469225 | 5507920 | 1 | 7399.77 | Trans |
| M3 | 07/07/2011 0:00 | 3 | 49.789492 | -111.422710 | 469574 | 5515312 | 1 | 4316.49 | Trans |
| M3 | 07/07/2011 0:00 | 6 | 49.818959 | -111.383653 | 472402 | 5518573 | 1 | 124.69 | Trans |
| M3 | 07/07/2011 0:00 | 9 | 49.818312 | -111.385070 | 472299 | 5518501 | 1 | 14.35 | Trans |
| M3 | 07/07/2011 0:00 | 12 | 49.818274 | -111.384879 | 472313 | 5518497 | 1 | 7.41 | Trans |
| M3 | 07/07/2011 0:00 | 15 | 49.818341 | -111.384883 | 472313 | 5518504 | 1 | 2.44 | Trans |
| M3 | 07/07/2011 0:00 | 18 | 49.818325 | -111.384860 | 472314 | 5518503 | 1 | 1.84 | Trans |
| M3 | 07/07/2011 0:00 | 21 | 49.818318 | -111.384883 | 472313 | 5518502 | 1 | 2530.04 | Trans |
| M3 | 07/08/2011 0:00 | 0 | 49.828540 | -111.353458 | 474579 | 5519627 | 1 | 6538.18 | Trans |
| M3 | 07/08/2011 0:00 | 3 | 49.867780 | -111.285723 | 479467 | 5523969 | 1 | 4339.53 | Trans |
| M3 | 07/08/2011 0:00 | 6 | 49.864215 | -111.225592 | 483787 | 5523558 | 1 | 42.48 | Trans |
| M3 | 07/08/2011 0:00 | 9 | 49.864281 | -111.226174 | 483745 | 5523566 | 1 | 14.20 | Trans |
| M3 | 07/08/2011 0:00 | 12 | 49.864154 | -111.226190 | 483744 | 5523551 | 1 | 2.34 | Trans |
| M3 | 07/08/2011 0:00 | 15 | 49.864156 | -111.226223 | 483742 | 5523552 | 1 | 6.40 | Trans |
| M3 | 07/08/2011 0:00 | 18 | 49.864104 | -111.226259 | 483739 | 5523546 | 1 | 127.74 | Trans |
| M3 | 07/08/2011 0:00 | 21 | 49.864066 | -111.224483 | 483867 | 5523541 | 1 | 3162.57 | Trans |
| M3 | 07/09/2011 0:00 | 0 | 49.840379 | -111.200126 | 485610 | 5520903 | 1 | 5697.48 | Trans |
| M3 | 07/09/2011 0:00 | 3 | 49.811101 | -111.135115 | 490279 | 5517637 | 1 | 228.95 | Trans |
| M3 | 07/09/2011 0:00 | 6 | 49.810151 | -111.137938 | 490075 | 5517532 | 1 | 435.16 | Trans |
| M3 | 07/09/2011 0:00 | 9 | 49.806251 | -111.137435 | 490111 | 5517098 | 0 | 129.19 | Trans |
| M3 | 07/09/2011 0:00 | 15 | 49.806095 | -111.135655 | 490239 | 5517081 | 1 | 5.31 | Trans |
| M3 | 07/09/2011 0:00 | 18 | 49.806081 | -111.135726 | 490234 | 5517079 | 1 | 3.76 | Trans |
| M3 | 07/09/2011 0:00 | 21 | 49.806089 | -111.135675 | 490238 | 5517080 | 1 | 1400.14 | Trans |
| M3 | 07/10/2011 0:00 | 0 | 49.807325 | -111.116310 | 491631 | 5517215 | 1 | 5323.89 | Local |
| M3 | 07/10/2011 0:00 | 3 | 49.780011 | -111.055553 | 496001 | 5514173 | 1 | 810.52 | Local |
| M3 | 07/10/2011 0:00 | 6 | 49.777370 | -111.045060 | 496756 | 5513879 | 1 | 15.97 | Local |
| M3 | 07/10/2011 0:00 | 9 | 49.777514 | -111.045054 | 496756 | 5513895 | 1 | 9.10 | Local |
| M3 | 07/10/2011 0:00 | 12 | 49.777450 | -111.044975 | 496762 | 5513888 | 1 | 6.60 | Local |
| M3 | 07/10/2011 0:00 | 15 | 49.777450 | -111.045067 | 496755 | 5513888 | 1 | 27.63 | Local |
| M3 | 07/10/2011 0:00 | 18 | 49.777674 | -111.044899 | 496767 | 5513913 | 1 | 3.72 | Local |
| M3 | 07/10/2011 0:00 | 21 | 49.777706 | -111.044914 | 496766 | 5513916 | 1 | 1594.47 | Local |
| M3 | 07/11/2011 0:00 | 0 | 49.763605 | -111.048960 | 496474 | 5512349 | 1 | 87.06 | Local |
| M3 | 07/11/2011 0:00 | 3 | 49.764327 | -111.048491 | 496508 | 5512429 | 1 | 23.78 | Local |
| M3 | 07/11/2011 0:00 | 6 | 49.764476 | -111.048253 | 496525 | 5512446 | 1 | 112.88 | Local |
| M3 | 07/11/2011 0:00 | 9 | 49.763541 | -111.047642 | 496569 | 5512342 | 1 | 12.78 | Local |
| M3 | 07/11/2011 0:00 | 12 | 49.763459 | -111.047518 | 496578 | 5512333 | 1 | 95.41 | Local |
| M3 | 07/11/2011 0:00 | 15 | 49.764175 | -111.048249 | 496525 | 5512412 | 1 | 346.36 | Local |
| M3 | 07/11/2011 0:00 | 18 | 49.767080 | -111.049983 | 496401 | 5512735 | 1 | 26.27 | Local |
| M3 | 07/11/2011 0:00 | 21 | 49.766896 | -111.050210 | 496384 | 5512715 | 1 | 380.72 | Local |
| M3 | 07/12/2011 0:00 | 0 | 49.764981 | -111.054593 | 496068 | 5512502 | 1 | 9.59 | Local |
| M3 | 07/12/2011 0:00 | 3 | 49.764894 | -111.054590 | 496069 | 5512493 | 1 | 6.12 | Local |
| M3 | 07/12/2011 0:00 | 6 | 49.764942 | -111.054633 | 496066 | 5512498 | 1 | 4.08 | Local |
| M3 | 07/12/2011 0:00 | 9 | 49.764977 | -111.054617 | 496067 | 5512502 | 1 | 23.71 | Local |
| M3 | 07/12/2011 0:00 | 12 | 49.764817 | -111.054833 | 496051 | 5512484 | 1 | 4.35 | Local |
| M3 | 07/12/2011 0:00 | 15 | 49.764777 | -111.054831 | 496051 | 5512480 | 1 | 2.43 | Local |
| M3 | 07/12/2011 0:00 | 18 | 49.764761 | -111.054808 | 496053 | 5512478 | 1 | 57.32 | Local |
| M3 | 07/12/2011 0:00 | 21 | 49.765240 | -111.054512 | 496074 | 5512531 | 0 | 138.85 | Local |
| M3 | 07/13/2011 0:00 | 3 | 49.765360 | -111.052593 | 496212 | 5512544 | 1 | 326.54 | Local |
| M3 | 07/13/2011 0:00 | 6 | 49.764424 | -111.048295 | 496522 | 5512440 | 1 | 12.76 | Local |
| M3 | 07/13/2011 0:00 | 9 | 49.764526 | -111.048214 | 496528 | 5512451 | 1 | 4.74 | Local |
| M3 | 07/13/2011 0:00 | 12 | 49.764555 | -111.048262 | 496524 | 5512454 | 1 | 5.38 | Local |

| | | | | | | | | | |
|---|---|---|---|---|---|---|---|---|---|
| M3 | 07/13/2011 0:00 | 15 | 49.764598 | -111.048230 | 496527 | 5512459 | 1 | 4.08 | Local |
| M3 | 07/13/2011 0:00 | 18 | 49.764563 | -111.048245 | 496526 | 5512455 | 1 | 868.68 | Local |
| M3 | 07/13/2011 0:00 | 21 | 49.756773 | -111.049165 | 496459 | 5511589 | 1 | 860.64 | Local |
| M3 | 07/14/2011 0:00 | 0 | 49.749136 | -111.047207 | 496599 | 5510740 | 1 | 38.85 | Local |
| M3 | 07/14/2011 0:00 | 3 | 49.748790 | -111.047135 | 496604 | 5510702 | 1 | 1122.83 | Local |
| M3 | 07/14/2011 0:00 | 6 | 49.758751 | -111.049706 | 496420 | 5511809 | 1 | 12.19 | Local |
| M3 | 07/14/2011 0:00 | 9 | 49.758849 | -111.049783 | 496414 | 5511820 | 1 | 15.57 | Local |
| M3 | 07/14/2011 0:00 | 12 | 49.758709 | -111.049766 | 496416 | 5511805 | 1 | 6.88 | Local |
| M3 | 07/14/2011 0:00 | 15 | 49.758665 | -111.049699 | 496420 | 5511800 | 1 | 59.81 | Local |
| M3 | 07/14/2011 0:00 | 18 | 49.758194 | -111.050100 | 496391 | 5511747 | 1 | 5.93 | Local |
| M3 | 07/14/2011 0:00 | 21 | 49.758148 | -111.050142 | 496388 | 5511742 | 1 | 167.93 | Local |
| M3 | 07/15/2011 0:00 | 0 | 49.759080 | -111.051977 | 496256 | 5511846 | 1 | 101.05 | Local |
| M3 | 07/15/2011 0:00 | 3 | 49.758698 | -111.050704 | 496348 | 5511803 | 1 | 22.62 | Local |
| M3 | 07/15/2011 0:00 | 6 | 49.758674 | -111.051016 | 496326 | 5511801 | 1 | 161.72 | Local |
| M3 | 07/15/2011 0:00 | 9 | 49.759429 | -111.049096 | 496464 | 5511885 | 1 | 3.19 | Local |
| M3 | 07/15/2011 0:00 | 12 | 49.759422 | -111.049053 | 496467 | 5511884 | 1 | 180.65 | Local |
| M3 | 07/15/2011 0:00 | 15 | 49.758020 | -111.050322 | 496375 | 5511728 | 1 | 35.30 | Local |
| M3 | 07/15/2011 0:00 | 18 | 49.757708 | -111.050230 | 496382 | 5511693 | 1 | 37.30 | Local |
| M3 | 07/15/2011 0:00 | 21 | 49.758022 | -111.050412 | 496369 | 5511728 | 1 | 4.81 | Local |
| M3 | 07/16/2011 0:00 | 0 | 49.758025 | -111.050345 | 496374 | 5511729 | 1 | 6.12 | Local |
| M3 | 07/16/2011 0:00 | 3 | 49.757987 | -111.050283 | 496378 | 5511724 | 0 | 182.91 | Local |
| M3 | 07/16/2011 0:00 | 9 | 49.759434 | -111.049074 | 496465 | 5511885 | 1 | 7.64 | Local |
| M3 | 07/16/2011 0:00 | 12 | 49.759367 | -111.049049 | 496467 | 5511878 | 1 | 1.48 | Local |
| M3 | 07/16/2011 0:00 | 15 | 49.759377 | -111.049063 | 496466 | 5511879 | 1 | 4.98 | Local |
| M3 | 07/16/2011 0:00 | 18 | 49.759334 | -111.049085 | 496465 | 5511874 | 1 | 143.61 | Local |
| M3 | 07/16/2011 0:00 | 21 | 49.760429 | -111.048026 | 496541 | 5511996 | 1 | 386.94 | Local |
| M3 | 07/17/2011 0:00 | 0 | 49.763609 | -111.050207 | 496384 | 5512349 | 1 | 251.51 | Local |
| M3 | 07/17/2011 0:00 | 3 | 49.765818 | -111.049454 | 496439 | 5512595 | 1 | 3.42 | Local |
| M3 | 07/17/2011 0:00 | 6 | 49.765792 | -111.049478 | 496437 | 5512592 | 1 | 77.90 | Local |
| M3 | 07/17/2011 0:00 | 9 | 49.766247 | -111.048656 | 496496 | 5512643 | 1 | 57.04 | Local |
| M3 | 07/17/2011 0:00 | 12 | 49.766166 | -111.049439 | 496440 | 5512634 | 1 | 2.65 | Local |
| M3 | 07/17/2011 0:00 | 15 | 49.766188 | -111.049425 | 496441 | 5512636 | 1 | 4.48 | Local |
| M3 | 07/17/2011 0:00 | 18 | 49.766228 | -111.049436 | 496440 | 5512641 | 1 | 117.56 | Local |
| M3 | 07/17/2011 0:00 | 21 | 49.765241 | -111.048849 | 496482 | 5512531 | 1 | 107.02 | Local |
| M3 | 07/18/2011 0:00 | 0 | 49.765699 | -111.050157 | 496388 | 5512582 | 1 | 9.98 | Local |
| M3 | 07/18/2011 0:00 | 3 | 49.765612 | -111.050190 | 496386 | 5512572 | 1 | 123.29 | Local |
| M3 | 07/18/2011 0:00 | 6 | 49.766193 | -111.048732 | 496491 | 5512637 | 1 | 141.53 | Local |
| M3 | 07/18/2011 0:00 | 9 | 49.764928 | -111.048513 | 496506 | 5512496 | 1 | 18.87 | Local |
| M3 | 07/18/2011 0:00 | 12 | 49.764796 | -111.048349 | 496518 | 5512481 | 1 | 34.24 | Local |
| M3 | 07/18/2011 0:00 | 15 | 49.765046 | -111.048626 | 496498 | 5512509 | 0 | 7.47 | Local |
| M3 | 07/18/2011 0:00 | 21 | 49.765096 | -111.048557 | 496503 | 5512515 | 1 | 122.79 | Local |
| M3 | 07/19/2011 0:00 | 0 | 49.766192 | -111.048775 | 496488 | 5512637 | 1 | 114.70 | Local |
| M3 | 07/19/2011 0:00 | 3 | 49.765165 | -111.048926 | 496477 | 5512522 | 1 | 89.36 | Local |
| M3 | 07/19/2011 0:00 | 6 | 49.764497 | -111.048235 | 496526 | 5512448 | 1 | 555.84 | Local |
| M3 | 07/19/2011 0:00 | 9 | 49.759527 | -111.049062 | 496466 | 5511896 | 1 | 17.73 | Local |
| M3 | 07/19/2011 0:00 | 12 | 49.759367 | -111.049062 | 496466 | 5511878 | 1 | 12.95 | Local |
| M3 | 07/19/2011 0:00 | 15 | 49.759456 | -111.048945 | 496475 | 5511888 | 1 | 10.07 | Local |
| M3 | 07/19/2011 0:00 | 18 | 49.759411 | -111.049066 | 496466 | 5511883 | 1 | 168.56 | Local |
| M3 | 07/19/2011 0:00 | 21 | 49.758084 | -111.050199 | 496384 | 5511735 | 1 | 1855.08 | Local |
| M3 | 07/20/2011 0:00 | 0 | 49.743694 | -111.037162 | 497323 | 5510135 | 1 | 525.96 | Local |
| M3 | 07/20/2011 0:00 | 3 | 49.745631 | -111.043823 | 496843 | 5510350 | 1 | 237.19 | Local |
| M3 | 07/20/2011 0:00 | 6 | 49.744721 | -111.040846 | 497057 | 5510249 | 1 | 12.36 | Local |
| M3 | 07/20/2011 0:00 | 9 | 49.744780 | -111.040991 | 497047 | 5510256 | 1 | 7.76 | Local |
| M3 | 07/20/2011 0:00 | 12 | 49.744710 | -111.040979 | 497048 | 5510248 | 1 | 2.80 | Local |
| M3 | 07/20/2011 0:00 | 15 | 49.744734 | -111.040968 | 497048 | 5510251 | 1 | 1.13 | Local |

| | | | | | | | | | |
|---|---|---|---|---|---|---|---|---|---|
| M3 | 07/20/2011 0:00 | 18 | 49.744743 | -111.040959 | 497049 | 5510251 | 1 | 148.54 | Local |
| M3 | 07/20/2011 0:00 | 21 | 49.743762 | -111.039560 | 497150 | 5510142 | 1 | 1112.22 | Local |
| M3 | 07/21/2011 0:00 | 0 | 49.753176 | -111.044779 | 496774 | 5511189 | 1 | 318.05 | Local |
| M3 | 07/21/2011 0:00 | 3 | 49.755331 | -111.047684 | 496565 | 5511429 | 1 | 17.11 | Local |
| M3 | 07/21/2011 0:00 | 6 | 49.755473 | -111.047774 | 496559 | 5511445 | 1 | 14.09 | Local |
| M3 | 07/21/2011 0:00 | 9 | 49.755599 | -111.047751 | 496560 | 5511459 | 1 | 7.07 | Local |
| M3 | 07/21/2011 0:00 | 12 | 49.755544 | -111.047801 | 496557 | 5511453 | 1 | 6.64 | Local |
| M3 | 07/21/2011 0:00 | 15 | 49.755514 | -111.047721 | 496563 | 5511449 | 1 | 5.13 | Local |
| M3 | 07/21/2011 0:00 | 18 | 49.755469 | -111.047735 | 496562 | 5511444 | 1 | 25.91 | Local |
| M3 | 07/21/2011 0:00 | 21 | 49.755689 | -111.047615 | 496570 | 5511469 | 1 | 164.81 | Local |
| M3 | 07/22/2011 0:00 | 0 | 49.756891 | -111.048954 | 496474 | 5511602 | 1 | 12.93 | Local |
| M3 | 07/22/2011 0:00 | 3 | 49.756960 | -111.048811 | 496484 | 5511610 | 1 | 267.99 | Local |
| M3 | 07/22/2011 0:00 | 6 | 49.759368 | -111.048990 | 496472 | 5511878 | 1 | 71.00 | Local |
| M3 | 07/22/2011 0:00 | 9 | 49.759959 | -111.048614 | 496499 | 5511943 | 1 | 125.04 | Local |
| M3 | 07/22/2011 0:00 | 12 | 49.761034 | -111.048103 | 496536 | 5512063 | 1 | 12.44 | Local |
| M3 | 07/22/2011 0:00 | 15 | 49.761107 | -111.047973 | 496545 | 5512071 | 1 | 4.07 | Local |
| M3 | 07/22/2011 0:00 | 18 | 49.761140 | -111.047996 | 496543 | 5512075 | 1 | 17.69 | Local |
| M3 | 07/22/2011 0:00 | 21 | 49.761020 | -111.047834 | 496555 | 5512062 | 1 | 301.57 | Local |
| M3 | 07/23/2011 0:00 | 0 | 49.761229 | -111.052009 | 496254 | 5512085 | 1 | 235.76 | Local |
| M3 | 07/23/2011 0:00 | 3 | 49.762184 | -111.049086 | 496465 | 5512191 | 1 | 8.45 | Local |
| M3 | 07/23/2011 0:00 | 6 | 49.762259 | -111.049069 | 496466 | 5512199 | 1 | 67.27 | Local |
| M3 | 07/23/2011 0:00 | 9 | 49.762410 | -111.049974 | 496401 | 5512216 | 1 | 2.99 | Local |
| M3 | 07/23/2011 0:00 | 12 | 49.762431 | -111.050000 | 496399 | 5512218 | 1 | 18.97 | Local |
| M3 | 07/23/2011 0:00 | 15 | 49.762548 | -111.050191 | 496385 | 5512231 | 1 | 9.26 | Local |
| M3 | 07/23/2011 0:00 | 18 | 49.762480 | -111.050118 | 496391 | 5512224 | 1 | 185.52 | Local |
| M3 | 07/23/2011 0:00 | 21 | 49.761732 | -111.047815 | 496556 | 5512141 | 1 | 1134.62 | Local |
| M3 | 07/24/2011 0:00 | 0 | 49.751654 | -111.050298 | 496377 | 5511020 | 1 | 1020.65 | Local |
| M3 | 07/24/2011 0:00 | 3 | 49.744770 | -111.040924 | 497052 | 5510255 | 1 | 6.39 | Local |
| M3 | 07/24/2011 0:00 | 6 | 49.744718 | -111.040961 | 497049 | 5510249 | 1 | 6.00 | Local |
| M3 | 07/24/2011 0:00 | 9 | 49.744772 | -111.040955 | 497049 | 5510255 | 1 | 3.30 | Local |
| M3 | 07/24/2011 0:00 | 12 | 49.744756 | -111.040916 | 497052 | 5510253 | 1 | 173.48 | Local |
| M3 | 07/24/2011 0:00 | 15 | 49.743999 | -111.038811 | 497204 | 5510169 | 1 | 6.17 | Local |
| M3 | 07/24/2011 0:00 | 18 | 49.743999 | -111.038725 | 497210 | 5510169 | 1 | 55.36 | Local |
| M3 | 07/24/2011 0:00 | 21 | 49.743502 | -111.038722 | 497210 | 5510113 | 1 | 420.55 | Local |
| M3 | 07/25/2011 0:00 | 0 | 49.742485 | -111.033100 | 497615 | 5510000 | 1 | 3793.88 | Local |
| M3 | 07/25/2011 0:00 | 3 | 49.774377 | -111.014365 | 498966 | 5513545 | 1 | 120.04 | Local |
| M3 | 07/25/2011 0:00 | 6 | 49.774884 | -111.015836 | 498860 | 5513602 | 1 | 115.49 | Local |
| M3 | 07/25/2011 0:00 | 9 | 49.774962 | -111.017436 | 498745 | 5513611 | 1 | 38.02 | Local |
| M3 | 07/25/2011 0:00 | 12 | 49.774882 | -111.016923 | 498782 | 5513602 | 0 | 7.63 | Local |
| M3 | 07/25/2011 0:00 | 18 | 49.774814 | -111.016938 | 498780 | 5513594 | 1 | 200.26 | Local |
| M3 | 07/25/2011 0:00 | 21 | 49.774720 | -111.014160 | 498980 | 5513584 | 1 | 75.54 | Local |
| M3 | 07/26/2011 0:00 | 0 | 49.774807 | -111.013120 | 499055 | 5513593 | 1 | 17.96 | Local |
| M3 | 07/26/2011 0:00 | 3 | 49.774687 | -111.013287 | 499043 | 5513580 | 1 | 51.70 | Local |
| M3 | 07/26/2011 0:00 | 6 | 49.774826 | -111.013973 | 498994 | 5513595 | 1 | 11.62 | Local |
| M3 | 07/26/2011 0:00 | 9 | 49.774915 | -111.013890 | 499000 | 5513605 | 1 | 7.80 | Local |
| M3 | 07/26/2011 0:00 | 12 | 49.774858 | -111.013953 | 498995 | 5513599 | 1 | 16.66 | Local |
| M3 | 07/26/2011 0:00 | 15 | 49.774853 | -111.014184 | 498979 | 5513598 | 1 | 4.54 | Local |
| M3 | 07/26/2011 0:00 | 18 | 49.774818 | -111.014151 | 498981 | 5513595 | 1 | 202.53 | Local |
| M3 | 07/26/2011 0:00 | 21 | 49.776314 | -111.012547 | 499097 | 5513761 | 1 | 256.38 | Local |
| M3 | 07/27/2011 0:00 | 0 | 49.776716 | -111.009040 | 499349 | 5513805 | 1 | 22.57 | Local |
| M3 | 07/27/2011 0:00 | 3 | 49.776845 | -111.009281 | 499332 | 5513820 | 1 | 178.14 | Local |
| M3 | 07/27/2011 0:00 | 6 | 49.777581 | -111.011480 | 499174 | 5513902 | 1 | 14.06 | Local |
| M3 | 07/27/2011 0:00 | 9 | 49.777705 | -111.011447 | 499176 | 5513915 | 1 | 14.49 | Local |
| M3 | 07/27/2011 0:00 | 12 | 49.777579 | -111.011498 | 499172 | 5513901 | 1 | 4.71 | Local |
| M3 | 07/27/2011 0:00 | 15 | 49.777622 | -111.011492 | 499173 | 5513906 | 1 | 24.49 | Local |

| | | | | | | | | | |
|---|---|---|---|---|---|---|---|---|---|
| M3 | 07/27/2011 0:00 | 18 | 49.777841 | -111.011512 | 499171 | 5513931 | 1 | 352.80 | Local |
| M3 | 07/27/2011 0:00 | 21 | 49.776427 | -111.007126 | 499487 | 5513773 | 1 | 469.73 | Local |
| M3 | 07/28/2011 0:00 | 0 | 49.780037 | -111.010516 | 499243 | 5514175 | 1 | 1489.52 | Local |
| M3 | 07/28/2011 0:00 | 3 | 49.778645 | -110.989939 | 500724 | 5514020 | 1 | 1492.49 | Local |
| M3 | 07/28/2011 0:00 | 6 | 49.780182 | -111.010533 | 499242 | 5514191 | 1 | 425.80 | Local |
| M3 | 07/28/2011 0:00 | 9 | 49.776652 | -111.012829 | 499076 | 5513798 | 0 | 6.99 | Local |
| M3 | 07/28/2011 0:00 | 15 | 49.776611 | -111.012901 | 499071 | 5513794 | 1 | 0.98 | Local |
| M3 | 07/28/2011 0:00 | 18 | 49.776613 | -111.012888 | 499072 | 5513794 | 1 | 197.12 | Local |
| M3 | 07/28/2011 0:00 | 21 | 49.774991 | -111.013993 | 498992 | 5513614 | 0 | 230.25 | Local |
| M3 | 07/29/2011 0:00 | 6 | 49.775774 | -111.016954 | 498779 | 5513701 | 1 | 2.62 | Local |
| M3 | 07/29/2011 0:00 | 9 | 49.775757 | -111.016980 | 498777 | 5513699 | 1 | 26.88 | Local |
| M3 | 07/29/2011 0:00 | 12 | 49.775526 | -111.016874 | 498785 | 5513673 | 1 | 5.53 | Local |
| M3 | 07/29/2011 0:00 | 15 | 49.775525 | -111.016950 | 498780 | 5513673 | 1 | 6.55 | Local |
| M3 | 07/29/2011 0:00 | 18 | 49.775483 | -111.016887 | 498784 | 5513668 | 1 | 59.78 | Local |
| M3 | 07/29/2011 0:00 | 21 | 49.775130 | -111.017514 | 498739 | 5513629 | 1 | 214.62 | Local |
| M3 | 07/30/2011 0:00 | 0 | 49.775353 | -111.020474 | 498526 | 5513654 | 1 | 7.40 | Local |
| M3 | 07/30/2011 0:00 | 3 | 49.775315 | -111.020391 | 498532 | 5513650 | 1 | 7.98 | Local |
| M3 | 07/30/2011 0:00 | 6 | 49.775325 | -111.020500 | 498524 | 5513651 | 1 | 11.56 | Local |
| M3 | 07/30/2011 0:00 | 9 | 49.775380 | -111.020364 | 498534 | 5513657 | 1 | 221.94 | Local |
| M3 | 07/30/2011 0:00 | 12 | 49.775293 | -111.017284 | 498756 | 5513647 | 1 | 10.87 | Local |
| M3 | 07/30/2011 0:00 | 15 | 49.775196 | -111.017277 | 498756 | 5513637 | 1 | 1.31 | Local |
| M3 | 07/30/2011 0:00 | 18 | 49.775202 | -111.017262 | 498757 | 5513637 | 1 | 328.50 | Local |
| M3 | 07/30/2011 0:00 | 21 | 49.775260 | -111.012701 | 499086 | 5513644 | 1 | 527.67 | Local |
| M3 | 07/31/2011 0:00 | 0 | 49.778696 | -111.007645 | 499450 | 5514026 | 1 | 174.93 | Local |
| M3 | 07/31/2011 0:00 | 3 | 49.780268 | -111.007727 | 499444 | 5514200 | 1 | 16.74 | Local |
| M3 | 07/31/2011 0:00 | 6 | 49.780118 | -111.007740 | 499443 | 5514184 | 1 | 16.44 | Local |
| M3 | 07/31/2011 0:00 | 9 | 49.780251 | -111.007839 | 499436 | 5514199 | 1 | 28.98 | Local |
| M3 | 07/31/2011 0:00 | 12 | 49.780445 | -111.008109 | 499416 | 5514220 | 1 | 6.11 | Local |
| M3 | 07/31/2011 0:00 | 15 | 49.780481 | -111.008173 | 499412 | 5514224 | 1 | 63.85 | Local |
| M3 | 07/31/2011 0:00 | 18 | 49.781028 | -111.008443 | 499392 | 5514285 | 1 | 495.63 | Local |
| M3 | 07/31/2011 0:00 | 21 | 49.780789 | -111.001568 | 499887 | 5514258 | 1 | 598.08 | Local |
| M3 | 08/01/2011 0:00 | 0 | 49.781348 | -110.993306 | 500482 | 5514320 | 1 | 433.22 | Local |
| M3 | 08/01/2011 0:00 | 3 | 49.781078 | -110.987302 | 500914 | 5514290 | 1 | 402.75 | Local |
| M3 | 08/01/2011 0:00 | 6 | 49.781382 | -110.992877 | 500513 | 5514324 | 1 | 13.58 | Local |
| M3 | 08/01/2011 0:00 | 9 | 49.781503 | -110.992851 | 500515 | 5514338 | 1 | 4.83 | Local |
| M3 | 08/01/2011 0:00 | 12 | 49.781460 | -110.992860 | 500514 | 5514333 | 1 | 34.82 | Local |
| M3 | 08/01/2011 0:00 | 15 | 49.781640 | -110.993255 | 500486 | 5514353 | 1 | 6.59 | Local |
| M3 | 08/01/2011 0:00 | 18 | 49.781582 | -110.993239 | 500487 | 5514346 | 1 | 35.49 | Local |
| M3 | 08/01/2011 0:00 | 21 | 49.781743 | -110.992813 | 500517 | 5514364 | 1 | 342.34 | Local |
| M3 | 08/02/2011 0:00 | 0 | 49.780643 | -110.988371 | 500837 | 5514242 | 1 | 319.96 | Local |
| M3 | 08/02/2011 0:00 | 3 | 49.782811 | -110.985449 | 501047 | 5514483 | 1 | 6.79 | Local |
| M3 | 08/02/2011 0:00 | 6 | 49.782775 | -110.985525 | 501042 | 5514479 | 1 | 8.30 | Local |
| M3 | 08/02/2011 0:00 | 9 | 49.782848 | -110.985501 | 501044 | 5514487 | 1 | 5.86 | Local |
| M3 | 08/02/2011 0:00 | 12 | 49.782837 | -110.985581 | 501038 | 5514486 | 1 | 4.99 | Local |
| M3 | 08/02/2011 0:00 | 15 | 49.782858 | -110.985643 | 501034 | 5514488 | 1 | 2.92 | Local |
| M3 | 08/02/2011 0:00 | 18 | 49.782865 | -110.985604 | 501036 | 5514489 | 1 | 213.46 | Local |
| M3 | 08/02/2011 0:00 | 21 | 49.780960 | -110.985972 | 501010 | 5514277 | 1 | 208.97 | Local |
| M3 | 08/03/2011 0:00 | 0 | 49.782815 | -110.985499 | 501044 | 5514484 | 1 | 7.37 | Local |
| M3 | 08/03/2011 0:00 | 3 | 49.782875 | -110.985540 | 501041 | 5514490 | 1 | 564.37 | Local |
| M3 | 08/03/2011 0:00 | 6 | 49.781761 | -110.993188 | 500490 | 5514366 | 1 | 191.47 | Local |
| M3 | 08/03/2011 0:00 | 9 | 49.781695 | -110.995846 | 500299 | 5514359 | 1 | 5.57 | Local |
| M3 | 08/03/2011 0:00 | 12 | 49.781669 | -110.995780 | 500304 | 5514356 | 1 | 10.16 | Local |
| M3 | 08/03/2011 0:00 | 15 | 49.781592 | -110.995856 | 500298 | 5514348 | 1 | 274.31 | Local |
| M3 | 08/03/2011 0:00 | 18 | 49.780579 | -110.999331 | 500048 | 5514235 | 1 | 167.76 | Local |
| M3 | 08/03/2011 0:00 | 21 | 49.780695 | -111.001654 | 499881 | 5514248 | 1 | 827.94 | Local |

| | | | | | | | | | |
|---|---|---|---|---|---|---|---|---|---|
| M3 | 08/04/2011 0:00 | 0 | 49.774900 | -111.008876 | 499361 | 5513604 | 1 | 377.37 | Local |
| M3 | 08/04/2011 0:00 | 3 | 49.774810 | -111.014115 | 498984 | 5513594 | 1 | 22.16 | Local |
| M3 | 08/04/2011 0:00 | 6 | 49.774890 | -111.014397 | 498963 | 5513603 | 1 | 5.94 | Local |
| M3 | 08/04/2011 0:00 | 9 | 49.774943 | -111.014390 | 498964 | 5513608 | 0 | 13.88 | Local |
| M3 | 08/04/2011 0:00 | 15 | 49.774821 | -111.014352 | 498967 | 5513595 | 1 | 8.93 | Local |
| M3 | 08/04/2011 0:00 | 18 | 49.774887 | -111.014282 | 498972 | 5513602 | 1 | 268.20 | Local |
| M3 | 08/04/2011 0:00 | 21 | 49.775107 | -111.017991 | 498705 | 5513627 | 1 | 181.91 | Local |
| M3 | 08/05/2011 0:00 | 0 | 49.775099 | -111.020517 | 498523 | 5513626 | 1 | 1.82 | Local |
| M3 | 08/05/2011 0:00 | 3 | 49.775085 | -111.020530 | 498522 | 5513624 | 1 | 9.47 | Local |
| M3 | 08/05/2011 0:00 | 6 | 49.775088 | -111.020662 | 498512 | 5513625 | 1 | 6.86 | Local |
| M3 | 08/05/2011 0:00 | 9 | 49.775122 | -111.020582 | 498518 | 5513628 | 1 | 5.06 | Local |
| M3 | 08/05/2011 0:00 | 12 | 49.775112 | -111.020651 | 498513 | 5513627 | 1 | 1.83 | Local |
| M3 | 08/05/2011 0:00 | 15 | 49.775105 | -111.020674 | 498511 | 5513627 | 1 | 13.83 | Local |
| M3 | 08/05/2011 0:00 | 18 | 49.775222 | -111.020741 | 498507 | 5513640 | 1 | 176.05 | Local |
| M3 | 08/05/2011 0:00 | 21 | 49.773807 | -111.019643 | 498586 | 5513482 | 0 | 112.91 | Local |
| M3 | 08/06/2011 0:00 | 3 | 49.774796 | -111.019285 | 498611 | 5513592 | 1 | 146.04 | Local |
| M3 | 08/06/2011 0:00 | 6 | 49.775082 | -111.017305 | 498754 | 5513624 | 1 | 9.99 | Local |
| M3 | 08/06/2011 0:00 | 9 | 49.775168 | -111.017345 | 498751 | 5513633 | 1 | 2.13 | Local |
| M3 | 08/06/2011 0:00 | 12 | 49.775162 | -111.017373 | 498749 | 5513633 | 1 | 3.42 | Local |
| M3 | 08/06/2011 0:00 | 15 | 49.775131 | -111.017379 | 498749 | 5513629 | 1 | 108.42 | Local |
| M3 | 08/06/2011 0:00 | 18 | 49.774817 | -111.018804 | 498646 | 5513594 | 1 | 53.47 | Local |
| M3 | 08/06/2011 0:00 | 21 | 49.774344 | -111.018665 | 498656 | 5513542 | 1 | 332.25 | Local |
| M3 | 08/07/2011 0:00 | 0 | 49.772204 | -111.021886 | 498424 | 5513304 | 1 | 579.86 | Local |
| M3 | 08/07/2011 0:00 | 3 | 49.770139 | -111.029281 | 497892 | 5513075 | 1 | 814.01 | Local |
| M3 | 08/07/2011 0:00 | 6 | 49.775993 | -111.036071 | 497403 | 5513726 | 1 | 88.84 | Local |
| M3 | 08/07/2011 0:00 | 9 | 49.776026 | -111.034838 | 497492 | 5513729 | 1 | 3.82 | Local |
| M3 | 08/07/2011 0:00 | 12 | 49.776010 | -111.034885 | 497488 | 5513727 | 1 | 3.31 | Local |
| M3 | 08/07/2011 0:00 | 15 | 49.775980 | -111.034880 | 497489 | 5513724 | 0 | 11.77 | Local |
| M3 | 08/07/2011 0:00 | 21 | 49.776008 | -111.034722 | 497500 | 5513727 | 1 | 12.51 | Local |
| M3 | 08/08/2011 0:00 | 0 | 49.776022 | -111.034895 | 497488 | 5513729 | 1 | 4.25 | Local |
| M3 | 08/08/2011 0:00 | 3 | 49.776060 | -111.034887 | 497488 | 5513733 | 1 | 105.07 | Local |
| M3 | 08/08/2011 0:00 | 6 | 49.775498 | -111.036060 | 497404 | 5513671 | 1 | 48.09 | Local |
| M3 | 08/08/2011 0:00 | 9 | 49.775918 | -111.035898 | 497415 | 5513717 | 1 | 32.92 | Local |
| M3 | 08/08/2011 0:00 | 12 | 49.776189 | -111.036082 | 497402 | 5513747 | 1 | 12.51 | Local |
| M3 | 08/08/2011 0:00 | 15 | 49.776097 | -111.036181 | 497395 | 5513737 | 1 | 74.70 | Local |
| M3 | 08/08/2011 0:00 | 18 | 49.775519 | -111.036710 | 497357 | 5513673 | 1 | 461.65 | Local |
| M3 | 08/08/2011 0:00 | 21 | 49.771941 | -111.039964 | 497122 | 5513275 | 1 | 790.22 | Local |
| M3 | 08/09/2011 0:00 | 0 | 49.767832 | -111.048918 | 496477 | 5512819 | 1 | 362.55 | Local |
| M3 | 08/09/2011 0:00 | 3 | 49.764596 | -111.048288 | 496522 | 5512459 | 1 | 446.73 | Local |
| M3 | 08/09/2011 0:00 | 6 | 49.760581 | -111.048062 | 496538 | 5512013 | 1 | 12.93 | Local |
| M3 | 08/09/2011 0:00 | 9 | 49.760599 | -111.048240 | 496526 | 5512015 | 1 | 11.92 | Local |
| M3 | 08/09/2011 0:00 | 12 | 49.760499 | -111.048299 | 496521 | 5512004 | 1 | 1.36 | Local |
| M3 | 08/09/2011 0:00 | 15 | 49.760503 | -111.048317 | 496520 | 5512004 | 1 | 8.54 | Local |
| M3 | 08/09/2011 0:00 | 18 | 49.760451 | -111.048231 | 496526 | 5511998 | 1 | 469.03 | Local |
| M3 | 08/09/2011 0:00 | 21 | 49.756289 | -111.049298 | 496449 | 5511536 | 1 | 219.89 | Local |
| M3 | 08/10/2011 0:00 | 0 | 49.754325 | -111.048942 | 496475 | 5511317 | 1 | 45.58 | Local |
| M3 | 08/10/2011 0:00 | 3 | 49.754020 | -111.049364 | 496444 | 5511283 | 1 | 4.37 | Local |
| M3 | 08/10/2011 0:00 | 6 | 49.753987 | -111.049330 | 496447 | 5511280 | 1 | 29.97 | Local |
| M3 | 08/10/2011 0:00 | 9 | 49.754035 | -111.048920 | 496476 | 5511285 | 1 | 6.53 | Local |
| M3 | 08/10/2011 0:00 | 12 | 49.753987 | -111.048869 | 496480 | 5511280 | 1 | 15.92 | Local |
| M3 | 08/10/2011 0:00 | 15 | 49.754126 | -111.048919 | 496476 | 5511295 | 1 | 20.88 | Local |
| M3 | 08/10/2011 0:00 | 18 | 49.754036 | -111.049173 | 496458 | 5511285 | 1 | 11.41 | Local |
| M3 | 08/10/2011 0:00 | 21 | 49.753936 | -111.049212 | 496455 | 5511274 | 1 | 10.17 | Local |
| M3 | 08/11/2011 0:00 | 0 | 49.753960 | -111.049349 | 496445 | 5511277 | 1 | 1207.40 | Local |
| M3 | 08/11/2011 0:00 | 3 | 49.761873 | -111.037869 | 497273 | 5512156 | 1 | 1140.17 | Local |

| | | | | | | | | | |
|---|---|---|---|---|---|---|---|---|---|
| M3 | 08/11/2011 0:00 | 6 | 49.772017 | -111.040192 | 497106 | 5513284 | 1 | 19.32 | Local |
| M3 | 08/11/2011 0:00 | 9 | 49.771926 | -111.040420 | 497090 | 5513274 | 1 | 11.44 | Local |
| M3 | 08/11/2011 0:00 | 12 | 49.771868 | -111.040289 | 497099 | 5513267 | 0 | 441.56 | Local |
| M3 | 08/11/2011 0:00 | 18 | 49.775060 | -111.036639 | 497362 | 5513622 | 1 | 128.26 | Local |
| M3 | 08/11/2011 0:00 | 21 | 49.775653 | -111.035111 | 497472 | 5513688 | 1 | 1105.66 | Local |
| M3 | 08/12/2011 0:00 | 0 | 49.769641 | -111.047343 | 496591 | 5513020 | 1 | 928.63 | Local |
| M3 | 08/12/2011 0:00 | 3 | 49.777845 | -111.044923 | 496766 | 5513932 | 1 | 43.92 | Local |
| M3 | 08/12/2011 0:00 | 6 | 49.777469 | -111.045110 | 496752 | 5513890 | 1 | 8.75 | Local |
| M3 | 08/12/2011 0:00 | 9 | 49.777420 | -111.045015 | 496759 | 5513885 | 1 | 4.31 | Local |
| M3 | 08/12/2011 0:00 | 12 | 49.777435 | -111.045070 | 496755 | 5513886 | 0 | 5.54 | Local |
| M3 | 08/12/2011 0:00 | 18 | 49.777396 | -111.045117 | 496752 | 5513882 | 1 | 27.52 | Local |
| M3 | 08/12/2011 0:00 | 21 | 49.777637 | -111.045031 | 496758 | 5513909 | 1 | 686.55 | Local |
| M3 | 08/13/2011 0:00 | 0 | 49.773129 | -111.038515 | 497227 | 5513407 | 1 | 8.04 | Local |
| M3 | 08/13/2011 0:00 | 3 | 49.773165 | -111.038611 | 497220 | 5513411 | 1 | 369.34 | Local |
| M3 | 08/13/2011 0:00 | 6 | 49.775692 | -111.035282 | 497460 | 5513692 | 1 | 73.53 | Local |
| M3 | 08/13/2011 0:00 | 9 | 49.775955 | -111.036218 | 497392 | 5513721 | 1 | 36.87 | Local |
| M3 | 08/13/2011 0:00 | 12 | 49.776285 | -111.036267 | 497389 | 5513758 | 1 | 54.67 | Local |
| M3 | 08/13/2011 0:00 | 15 | 49.776029 | -111.035619 | 497435 | 5513730 | 1 | 25.21 | Local |
| M3 | 08/13/2011 0:00 | 18 | 49.775977 | -111.035279 | 497460 | 5513724 | 0 | 5.37 | Local |
| M3 | 08/14/2011 0:00 | 0 | 49.775964 | -111.035350 | 497455 | 5513722 | 1 | 4.25 | Local |
| M3 | 08/14/2011 0:00 | 3 | 49.775941 | -111.035302 | 497458 | 5513720 | 1 | 110.89 | Local |
| M3 | 08/14/2011 0:00 | 6 | 49.775689 | -111.033813 | 497566 | 5513692 | 1 | 1009.32 | Local |
| M3 | 08/14/2011 0:00 | 9 | 49.773306 | -111.020286 | 498539 | 5513427 | 1 | 7.76 | Local |
| M3 | 08/14/2011 0:00 | 12 | 49.773237 | -111.020274 | 498540 | 5513419 | 1 | 12.96 | Local |
| M3 | 08/14/2011 0:00 | 15 | 49.773352 | -111.020298 | 498538 | 5513432 | 1 | 6.61 | Local |
| M3 | 08/14/2011 0:00 | 18 | 49.773293 | -111.020309 | 498538 | 5513425 | 1 | 100.20 | Local |
| M3 | 08/14/2011 0:00 | 21 | 49.773274 | -111.021700 | 498438 | 5513423 | 1 | 71.96 | Local |
| M3 | 08/15/2011 0:00 | 0 | 49.772630 | -111.021803 | 498430 | 5513351 | 1 | 264.20 | Local |
| M3 | 08/15/2011 0:00 | 3 | 49.774281 | -111.019163 | 498620 | 5513535 | 1 | 610.24 | Local |
| M3 | 08/15/2011 0:00 | 6 | 49.776580 | -111.011467 | 499174 | 5513790 | 1 | 95.69 | Local |
| M3 | 08/15/2011 0:00 | 9 | 49.777440 | -111.011495 | 499172 | 5513886 | 1 | 14.28 | Local |
| M3 | 08/15/2011 0:00 | 12 | 49.777492 | -111.011313 | 499185 | 5513892 | 1 | 11.62 | Local |
| M3 | 08/15/2011 0:00 | 15 | 49.777481 | -111.011474 | 499174 | 5513891 | 1 | 5.35 | Local |
| M3 | 08/15/2011 0:00 | 18 | 49.777434 | -111.011495 | 499172 | 5513885 | 1 | 5.33 | Local |
| M3 | 08/15/2011 0:00 | 21 | 49.777481 | -111.011512 | 499171 | 5513891 | 1 | 230.79 | Local |
| M3 | 08/16/2011 0:00 | 0 | 49.778763 | -111.008990 | 499353 | 5514033 | 1 | 121.47 | Local |
| M3 | 08/16/2011 0:00 | 3 | 49.778942 | -111.010655 | 499233 | 5514053 | 1 | 128.01 | Local |
| M3 | 08/16/2011 0:00 | 6 | 49.779469 | -111.009073 | 499347 | 5514112 | 0 | 9.42 | Local |
| M3 | 08/16/2011 0:00 | 12 | 49.779436 | -111.008953 | 499355 | 5514108 | 1 | 94.70 | Local |
| M3 | 08/16/2011 0:00 | 15 | 49.779365 | -111.010264 | 499261 | 5514100 | 1 | 281.29 | Local |
| M3 | 08/16/2011 0:00 | 18 | 49.777908 | -111.013458 | 499031 | 5513938 | 1 | 147.22 | Local |
| M3 | 08/16/2011 0:00 | 21 | 49.776644 | -111.014067 | 498987 | 5513798 | 1 | 67.26 | Local |
| M3 | 08/17/2011 0:00 | 0 | 49.776041 | -111.014129 | 498983 | 5513730 | 1 | 749.55 | Local |
| M3 | 08/17/2011 0:00 | 3 | 49.772377 | -111.022867 | 498353 | 5513323 | 1 | 3.19 | Local |
| M3 | 08/17/2011 0:00 | 6 | 49.772406 | -111.022862 | 498354 | 5513326 | 1 | 180.77 | Local |
| M3 | 08/17/2011 0:00 | 9 | 49.773004 | -111.020528 | 498522 | 5513393 | 1 | 13.53 | Local |
| M3 | 08/17/2011 0:00 | 12 | 49.772923 | -111.020668 | 498512 | 5513384 | 1 | 9.55 | Local |
| M3 | 08/17/2011 0:00 | 15 | 49.772854 | -111.020746 | 498506 | 5513376 | 1 | 10.25 | Local |
| M3 | 08/17/2011 0:00 | 18 | 49.772916 | -111.020642 | 498514 | 5513383 | 1 | 362.52 | Local |
| M3 | 08/17/2011 0:00 | 21 | 49.774162 | -111.015989 | 498849 | 5513522 | 1 | 487.74 | Local |
| M3 | 08/18/2011 0:00 | 0 | 49.777425 | -111.011461 | 499175 | 5513884 | 1 | 5.48 | Local |
| M3 | 08/18/2011 0:00 | 3 | 49.777376 | -111.011457 | 499175 | 5513879 | 1 | 232.34 | Local |
| M3 | 08/18/2011 0:00 | 6 | 49.778825 | -111.009132 | 499343 | 5514040 | 1 | 226.41 | Local |
| M3 | 08/18/2011 0:00 | 9 | 49.778803 | -111.005987 | 499569 | 5514038 | 1 | 35.26 | Local |
| M3 | 08/18/2011 0:00 | 12 | 49.778905 | -111.005524 | 499602 | 5514049 | 1 | 677.76 | Local |

| | | | | | | | | | |
|---|---|---|---|---|---|---|---|---|---|
| M3 | 08/18/2011 0:00 | 15 | 49.778818 | -110.996111 | 500280 | 5514039 | 1 | 248.84 | Local |
| M3 | 08/18/2011 0:00 | 18 | 49.776585 | -110.995877 | 500297 | 5513791 | 1 | 757.19 | Local |
| M3 | 08/18/2011 0:00 | 21 | 49.780616 | -110.987400 | 500907 | 5514239 | 1 | 1580.28 | Local |
| M3 | 08/19/2011 0:00 | 0 | 49.781094 | -111.009339 | 499328 | 5514292 | 1 | 661.71 | Local |
| M3 | 08/19/2011 0:00 | 3 | 49.776026 | -111.014157 | 498981 | 5513729 | 0 | 925.03 | Local |
| M3 | 08/19/2011 0:00 | 9 | 49.771351 | -111.024784 | 498215 | 5513209 | 1 | 41.68 | Local |
| M3 | 08/19/2011 0:00 | 12 | 49.771052 | -111.025134 | 498190 | 5513176 | 1 | 139.73 | Local |
| M3 | 08/19/2011 0:00 | 15 | 49.770291 | -111.026678 | 498079 | 5513091 | 1 | 515.18 | Local |
| M3 | 08/19/2011 0:00 | 18 | 49.773700 | -111.031522 | 497730 | 5513471 | 1 | 394.77 | Local |
| M3 | 08/19/2011 0:00 | 21 | 49.774605 | -111.036824 | 497349 | 5513571 | 1 | 930.64 | Local |
| M3 | 08/20/2011 0:00 | 0 | 49.769682 | -111.047278 | 496596 | 5513025 | 1 | 2278.95 | Local |
| M3 | 08/20/2011 0:00 | 3 | 49.749190 | -111.048049 | 496539 | 5510746 | 1 | 715.94 | Local |
| M3 | 08/20/2011 0:00 | 6 | 49.744694 | -111.040935 | 497051 | 5510246 | 1 | 13.27 | Local |
| M3 | 08/20/2011 0:00 | 9 | 49.744807 | -111.040993 | 497047 | 5510259 | 0 | 107.00 | Local |
| M3 | 08/20/2011 0:00 | 15 | 49.744858 | -111.042476 | 496940 | 5510264 | 1 | 60.83 | Local |
| M3 | 08/20/2011 0:00 | 18 | 49.745386 | -111.042698 | 496924 | 5510323 | 1 | 539.10 | Local |
| M3 | 08/20/2011 0:00 | 21 | 49.749004 | -111.047681 | 496565 | 5510726 | 1 | 31.97 | Local |
| M3 | 08/21/2011 0:00 | 0 | 49.749119 | -111.048088 | 496536 | 5510738 | 1 | 177.59 | Local |
| M3 | 08/21/2011 0:00 | 3 | 49.747525 | -111.047944 | 496546 | 5510561 | 1 | 357.37 | Local |
| M3 | 08/21/2011 0:00 | 6 | 49.750737 | -111.048121 | 496533 | 5510918 | 1 | 347.25 | Local |
| M3 | 08/21/2011 0:00 | 9 | 49.747778 | -111.046579 | 496644 | 5510589 | 1 | 13.19 | Local |
| M3 | 08/21/2011 0:00 | 12 | 49.747894 | -111.046613 | 496642 | 5510602 | 1 | 142.99 | Local |
| M3 | 08/21/2011 0:00 | 15 | 49.746616 | -111.046832 | 496626 | 5510460 | 0 | 180.15 | Local |
| M3 | 08/21/2011 0:00 | 21 | 49.748212 | -111.047269 | 496595 | 5510637 | 1 | 119.09 | Local |
| M3 | 08/22/2011 0:00 | 0 | 49.749175 | -111.047993 | 496543 | 5510744 | 1 | 103.06 | Local |
| M3 | 08/22/2011 0:00 | 3 | 49.749352 | -111.046589 | 496644 | 5510764 | 1 | 88.90 | Local |
| M3 | 08/22/2011 0:00 | 6 | 49.749212 | -111.047804 | 496556 | 5510749 | 0 | 476.86 | Local |
| M3 | 08/22/2011 0:00 | 21 | 49.745516 | -111.044446 | 496798 | 5510338 | 0 | 293.22 | Local |
| M3 | 08/23/2011 0:00 | 3 | 49.744761 | -111.040546 | 497079 | 5510254 | 1 | 491.68 | Local |
| M3 | 08/23/2011 0:00 | 6 | 49.741125 | -111.036661 | 497358 | 5509849 | 1 | 415.35 | Local |
| M3 | 08/23/2011 0:00 | 9 | 49.743994 | -111.040355 | 497093 | 5510168 | 1 | 80.35 | Local |
| M3 | 08/23/2011 0:00 | 12 | 49.744619 | -111.040915 | 497052 | 5510238 | 0 | 337.76 | Local |
| M3 | 08/23/2011 0:00 | 21 | 49.747122 | -111.043570 | 496861 | 5510516 | 1 | 610.02 | Local |
| M3 | 08/24/2011 0:00 | 0 | 49.743423 | -111.037317 | 497311 | 5510105 | 1 | 62.87 | Local |
| M3 | 08/24/2011 0:00 | 3 | 49.743744 | -111.038035 | 497260 | 5510140 | 1 | 121.24 | Local |
| M3 | 08/24/2011 0:00 | 6 | 49.744424 | -111.039352 | 497165 | 5510216 | 1 | 203.74 | Local |
| M3 | 08/24/2011 0:00 | 9 | 49.745288 | -111.041845 | 496985 | 5510312 | 0 | 190.75 | Local |
| M3 | 08/24/2011 0:00 | 18 | 49.745271 | -111.039197 | 497176 | 5510310 | 1 | 138.97 | Local |
| M3 | 08/24/2011 0:00 | 21 | 49.744235 | -111.038119 | 497254 | 5510195 | 1 | 27.84 | Local |
| M3 | 08/25/2011 0:00 | 0 | 49.744051 | -111.037857 | 497273 | 5510174 | 1 | 142.69 | Local |
| M3 | 08/25/2011 0:00 | 3 | 49.743033 | -111.036650 | 497359 | 5510061 | 0 | 460.64 | Local |
| M3 | 08/25/2011 0:00 | 9 | 49.744841 | -111.042403 | 496945 | 5510262 | 0 | 187.81 | Local |
| M3 | 08/25/2011 0:00 | 21 | 49.743422 | -111.040987 | 497047 | 5510105 | 1 | 1010.35 | Local |
| M3 | 08/26/2011 0:00 | 0 | 49.752184 | -111.037265 | 497316 | 5511079 | 0 | 2955.01 | Local |
| M3 | 08/26/2011 0:00 | 6 | 49.778446 | -111.030956 | 497771 | 5513998 | 0 | 1004.66 | Local |
| M3 | 08/26/2011 0:00 | 21 | 49.772763 | -111.041805 | 496990 | 5513367 | 1 | 239.48 | Local |
| M3 | 08/27/2011 0:00 | 0 | 49.771216 | -111.039490 | 497156 | 5513195 | 1 | 196.22 | Local |
| M3 | 08/27/2011 0:00 | 3 | 49.772866 | -111.038524 | 497226 | 5513378 | 1 | 175.18 | Local |
| M3 | 08/27/2011 0:00 | 6 | 49.771962 | -111.040516 | 497083 | 5513278 | 1 | 13.85 | Local |
| M3 | 08/27/2011 0:00 | 9 | 49.771899 | -111.040349 | 497095 | 5513271 | 1 | 17.00 | Local |
| M3 | 08/27/2011 0:00 | 12 | 49.771761 | -111.040449 | 497087 | 5513255 | 0 | 295.80 | Local |
| M3 | 08/27/2011 0:00 | 21 | 49.774017 | -111.038274 | 497244 | 5513506 | 1 | 272.62 | Local |
| M3 | 08/28/2011 0:00 | 0 | 49.774689 | -111.034632 | 497506 | 5513581 | 1 | 55.92 | Local |
| M3 | 08/28/2011 0:00 | 3 | 49.774564 | -111.033879 | 497561 | 5513567 | 0 | 396.72 | Local |
| M3 | 08/28/2011 0:00 | 9 | 49.774722 | -111.039384 | 497164 | 5513584 | 0 | 209.72 | Local |

| | | | | | | | | | |
|---|---|---|---|---|---|---|---|---|---|
| M3 | 08/28/2011 0:00 | 18 | 49.774229 | -111.036572 | 497367 | 5513530 | 1 | 175.39 | Local |
| M3 | 08/28/2011 0:00 | 21 | 49.773938 | -111.034178 | 497539 | 5513497 | 1 | 451.07 | Local |
| M3 | 08/29/2011 0:00 | 0 | 49.771908 | -111.028754 | 497930 | 5513271 | 1 | 388.72 | Local |
| M3 | 08/29/2011 0:00 | 3 | 49.770936 | -111.023569 | 498303 | 5513163 | 0 | 151.16 | Local |
| M3 | 08/29/2011 0:00 | 9 | 49.772149 | -111.024516 | 498235 | 5513298 | 1 | 95.83 | Local |
| M3 | 08/29/2011 0:00 | 12 | 49.771302 | -111.024764 | 498217 | 5513204 | 0 | 65.39 | Local |
| M3 | 08/29/2011 0:00 | 18 | 49.771884 | -111.024635 | 498226 | 5513269 | 1 | 437.06 | Local |
| M3 | 08/29/2011 0:00 | 21 | 49.775425 | -111.021999 | 498416 | 5513662 | 1 | 570.79 | Local |
| M3 | 08/30/2011 0:00 | 0 | 49.776054 | -111.014132 | 498983 | 5513732 | 1 | 315.75 | Local |
| M3 | 08/30/2011 0:00 | 3 | 49.778492 | -111.011882 | 499145 | 5514003 | 0 | 241.97 | Local |
| M3 | 08/30/2011 0:00 | 9 | 49.776318 | -111.012040 | 499133 | 5513761 | 1 | 102.84 | Local |
| M3 | 08/30/2011 0:00 | 12 | 49.777130 | -111.011355 | 499182 | 5513852 | 0 | 134.80 | Local |
| M3 | 08/30/2011 0:00 | 18 | 49.777285 | -111.009498 | 499316 | 5513869 | 1 | 660.25 | Local |
| M3 | 08/30/2011 0:00 | 21 | 49.776376 | -111.000436 | 499969 | 5513768 | 1 | 352.84 | Local |
| M3 | 08/31/2011 0:00 | 0 | 49.774386 | -110.996618 | 500244 | 5513546 | 1 | 103.42 | Local |
| M3 | 08/31/2011 0:00 | 3 | 49.775048 | -110.995608 | 500316 | 5513620 | 0 | 195.74 | Local |
| M3 | 08/31/2011 0:00 | 9 | 49.774566 | -110.998223 | 500128 | 5513566 | 1 | 110.87 | Local |
| M3 | 08/31/2011 0:00 | 12 | 49.773736 | -110.997370 | 500189 | 5513474 | 0 | 254.85 | Local |
| M3 | 08/31/2011 0:00 | 18 | 49.771965 | -110.999619 | 500027 | 5513277 | 0 | 538.70 | Local |
| M3 | 09/01/2011 0:00 | 0 | 49.776123 | -110.995778 | 500304 | 5513740 | 1 | 127.04 | Local |
| M3 | 09/01/2011 0:00 | 3 | 49.777228 | -110.996227 | 500272 | 5513862 | 1 | 868.58 | Local |
| M3 | 09/01/2011 0:00 | 6 | 49.775429 | -111.007966 | 499426 | 5513662 | 1 | 325.22 | Local |
| M3 | 09/01/2011 0:00 | 9 | 49.777885 | -111.005512 | 499603 | 5513935 | 0 | 613.74 | Local |
| M3 | 09/01/2011 0:00 | 18 | 49.775037 | -111.012814 | 499077 | 5513619 | 1 | 681.52 | Local |
| M3 | 09/01/2011 0:00 | 21 | 49.774758 | -111.022270 | 498397 | 5513588 | 1 | 623.05 | Local |
| M3 | 09/02/2011 0:00 | 0 | 49.774285 | -111.030892 | 497776 | 5513536 | 1 | 957.65 | Local |
| M3 | 09/02/2011 0:00 | 3 | 49.781227 | -111.038767 | 497209 | 5514308 | 1 | 67.71 | Local |
| M3 | 09/02/2011 0:00 | 6 | 49.781722 | -111.039315 | 497170 | 5514363 | 0 | 649.80 | Local |
| M3 | 09/02/2011 0:00 | 12 | 49.776257 | -111.036117 | 497400 | 5513755 | 0 | 59.95 | Local |
| M3 | 09/02/2011 0:00 | 18 | 49.776091 | -111.036909 | 497343 | 5513737 | 1 | 585.96 | Local |
| M3 | 09/02/2011 0:00 | 21 | 49.781216 | -111.038805 | 497206 | 5514307 | 1 | 54.56 | Local |
| M3 | 09/03/2011 0:00 | 0 | 49.781372 | -111.039524 | 497155 | 5514324 | 1 | 1007.15 | Local |
| M3 | 09/03/2011 0:00 | 3 | 49.786082 | -111.027572 | 498015 | 5514847 | 1 | 1265.94 | Local |
| M3 | 09/03/2011 0:00 | 6 | 49.774968 | -111.023744 | 498290 | 5513611 | 1 | 252.98 | Local |
| M3 | 09/03/2011 0:00 | 9 | 49.775639 | -111.020387 | 498532 | 5513686 | 0 | 230.79 | Local |
| M3 | 09/03/2011 0:00 | 18 | 49.773623 | -111.021149 | 498477 | 5513462 | 1 | 256.16 | Local |
| M3 | 09/03/2011 0:00 | 21 | 49.775136 | -111.018467 | 498670 | 5513630 | 1 | 108.85 | Local |
| M3 | 09/04/2011 0:00 | 0 | 49.774170 | -111.018228 | 498688 | 5513522 | 1 | 450.96 | Local |
| M3 | 09/04/2011 0:00 | 3 | 49.772811 | -111.012327 | 499112 | 5513371 | 1 | 366.47 | Local |
| M3 | 09/04/2011 0:00 | 6 | 49.774506 | -111.016692 | 498798 | 5513560 | 1 | 106.55 | Local |
| M3 | 09/04/2011 0:00 | 9 | 49.775080 | -111.015507 | 498883 | 5513624 | 0 | 203.52 | Local |
| M3 | 09/04/2011 0:00 | 18 | 49.774379 | -111.018118 | 498695 | 5513546 | 1 | 82.63 | Local |
| M3 | 09/04/2011 0:00 | 21 | 49.773843 | -111.018912 | 498638 | 5513486 | 1 | 159.80 | Local |
| M3 | 09/05/2011 0:00 | 0 | 49.775170 | -111.018062 | 498700 | 5513634 | 1 | 494.60 | Local |
| M3 | 09/05/2011 0:00 | 3 | 49.778007 | -111.023354 | 498319 | 5513949 | 1 | 355.31 | Local |
| M3 | 09/05/2011 0:00 | 6 | 49.775367 | -111.026136 | 498118 | 5513656 | 1 | 519.68 | Local |
| M3 | 09/05/2011 0:00 | 9 | 49.775112 | -111.018929 | 498637 | 5513627 | 0 | 586.99 | Local |
| M3 | 09/05/2011 0:00 | 18 | 49.771268 | -111.013340 | 499039 | 5513200 | 1 | 679.86 | Local |
| M3 | 09/05/2011 0:00 | 21 | 49.776141 | -111.007635 | 499450 | 5513742 | 1 | 1123.48 | Local |
| M3 | 09/06/2011 0:00 | 0 | 49.781293 | -110.994211 | 500417 | 5514314 | 1 | 113.60 | Local |
| M3 | 09/06/2011 0:00 | 3 | 49.781921 | -110.995455 | 500327 | 5514384 | 1 | 98.07 | Local |
| M3 | 09/06/2011 0:00 | 6 | 49.781270 | -110.994536 | 500393 | 5514312 | 1 | 78.61 | Local |
| M3 | 09/06/2011 0:00 | 9 | 49.780570 | -110.994379 | 500405 | 5514234 | 0 | 131.91 | Local |
| M3 | 09/06/2011 0:00 | 18 | 49.781414 | -110.993091 | 500497 | 5514328 | 1 | 112.29 | Local |
| M3 | 09/06/2011 0:00 | 21 | 49.782404 | -110.993398 | 500475 | 5514438 | 1 | 145.21 | Local |

| | | | | | | | | | |
|---|---|---|---|---|---|---|---|---|---|
| M3 | 09/07/2011 0:00 | 0 | 49.781512 | -110.994870 | 500369 | 5514339 | 0 | 15.03 | Local |
| M3 | 09/07/2011 0:00 | 6 | 49.781611 | -110.995012 | 500359 | 5514350 | 1 | 94.22 | Local |
| M3 | 09/07/2011 0:00 | 9 | 49.781404 | -110.996281 | 500268 | 5514327 | 1 | 268.56 | Local |
| M3 | 09/07/2011 0:00 | 12 | 49.780680 | -110.992722 | 500524 | 5514246 | 0 | 225.33 | Local |
| M3 | 09/08/2011 0:00 | 3 | 49.781968 | -110.995139 | 500350 | 5514389 | 1 | 177.76 | Local |
| M3 | 09/08/2011 0:00 | 6 | 49.780794 | -110.996814 | 500229 | 5514259 | 1 | 762.28 | Local |
| M3 | 09/08/2011 0:00 | 9 | 49.781167 | -111.007387 | 499468 | 5514300 | 1 | 147.55 | Local |
| M3 | 09/08/2011 0:00 | 12 | 49.780552 | -111.005571 | 499599 | 5514232 | 0 | 43.66 | Local |
| M3 | 09/08/2011 0:00 | 18 | 49.780322 | -111.005079 | 499634 | 5514206 | 1 | 331.77 | Local |
| M3 | 09/08/2011 0:00 | 21 | 49.777543 | -111.006756 | 499514 | 5513897 | 1 | 1294.55 | Local |
| M3 | 09/09/2011 0:00 | 0 | 49.773851 | -111.023808 | 498286 | 5513487 | 1 | 648.74 | Local |
| M3 | 09/09/2011 0:00 | 3 | 49.769846 | -111.030361 | 497814 | 5513042 | 1 | 201.36 | Local |
| M3 | 09/09/2011 0:00 | 6 | 49.770827 | -111.028010 | 497983 | 5513151 | 1 | 287.27 | Local |
| M3 | 09/09/2011 0:00 | 9 | 49.772881 | -111.030430 | 497809 | 5513380 | 1 | 447.96 | Local |
| M3 | 09/09/2011 0:00 | 12 | 49.769999 | -111.026083 | 498122 | 5513059 | 1 | 303.75 | Local |
| M3 | 09/09/2011 0:00 | 15 | 49.769864 | -111.030296 | 497818 | 5513044 | 0 | 739.94 | Local |
| M3 | 09/09/2011 0:00 | 21 | 49.773313 | -111.021507 | 498451 | 5513427 | 1 | 858.64 | Local |
| M3 | 09/10/2011 0:00 | 0 | 49.769134 | -111.011479 | 499173 | 5512963 | 1 | 1086.76 | Local |
| M3 | 09/10/2011 0:00 | 3 | 49.763562 | -110.999080 | 500066 | 5512343 | 1 | 1696.47 | Local |
| M3 | 09/10/2011 0:00 | 6 | 49.776332 | -111.011977 | 499138 | 5513763 | 1 | 44.91 | Local |
| M3 | 09/10/2011 0:00 | 9 | 49.776735 | -111.011951 | 499140 | 5513808 | 1 | 183.35 | Local |
| M3 | 09/10/2011 0:00 | 12 | 49.775352 | -111.013338 | 499040 | 5513654 | 0 | 38.27 | Local |
| M3 | 09/10/2011 0:00 | 18 | 49.775683 | -111.013484 | 499029 | 5513691 | 1 | 543.76 | Local |
| M3 | 09/10/2011 0:00 | 21 | 49.771599 | -111.009328 | 499328 | 5513237 | 1 | 226.75 | Local |
| M3 | 09/11/2011 0:00 | 0 | 49.770278 | -111.006929 | 499501 | 5513090 | 1 | 313.31 | Local |
| M3 | 09/11/2011 0:00 | 3 | 49.771545 | -111.003043 | 499781 | 5513231 | 1 | 10.86 | Local |
| M3 | 09/11/2011 0:00 | 6 | 49.771636 | -111.002986 | 499785 | 5513241 | 0 | 181.42 | Local |
| M3 | 09/11/2011 0:00 | 18 | 49.770138 | -111.001987 | 499857 | 5513074 | 1 | 465.74 | Local |
| M3 | 09/11/2011 0:00 | 21 | 49.770341 | -110.995527 | 500322 | 5513097 | 0 | 293.55 | Local |
| M3 | 09/12/2011 0:00 | 3 | 49.768473 | -110.992646 | 500530 | 5512889 | 1 | 7.11 | Local |
| M3 | 09/12/2011 0:00 | 6 | 49.768537 | -110.992642 | 500530 | 5512896 | 1 | 840.94 | Local |
| M3 | 09/12/2011 0:00 | 9 | 49.774658 | -110.999503 | 500036 | 5513577 | 0 | 51.16 | Local |
| M3 | 09/12/2011 0:00 | 18 | 49.775118 | -110.999482 | 500037 | 5513628 | 1 | 1098.16 | Local |
| M3 | 09/12/2011 0:00 | 21 | 49.766058 | -110.993406 | 500475 | 5512621 | 1 | 615.51 | Local |
| M3 | 09/13/2011 0:00 | 0 | 49.765513 | -110.984901 | 501087 | 5512560 | 1 | 977.00 | Local |
| M3 | 09/13/2011 0:00 | 3 | 49.773309 | -110.991164 | 500636 | 5513427 | 1 | 243.37 | Local |
| M3 | 09/13/2011 0:00 | 6 | 49.771594 | -110.993265 | 500485 | 5513236 | 1 | 191.70 | Local |
| M3 | 09/13/2011 0:00 | 9 | 49.771701 | -110.995922 | 500294 | 5513248 | 0 | 122.50 | Local |
| M3 | 09/13/2011 0:00 | 18 | 49.771347 | -110.994310 | 500410 | 5513209 | 1 | 42.18 | Local |
| M3 | 09/13/2011 0:00 | 21 | 49.771709 | -110.994132 | 500423 | 5513249 | 1 | 1036.39 | Local |
| M3 | 09/14/2011 0:00 | 0 | 49.780942 | -110.992155 | 500565 | 5514275 | 1 | 416.73 | Local |
| M3 | 09/14/2011 0:00 | 3 | 49.781474 | -110.986425 | 500977 | 5514335 | 1 | 603.24 | Local |
| M3 | 09/14/2011 0:00 | 6 | 49.782394 | -110.994683 | 500383 | 5514437 | 1 | 62.56 | Local |
| M3 | 09/14/2011 0:00 | 9 | 49.781871 | -110.995001 | 500360 | 5514379 | 0 | 517.49 | Local |
| M3 | 09/14/2011 0:00 | 18 | 49.780870 | -111.002021 | 499855 | 5514267 | 1 | 1184.38 | Local |
| M3 | 09/14/2011 0:00 | 21 | 49.780887 | -111.018472 | 498670 | 5514269 | 1 | 1579.59 | Local |
| M3 | 09/15/2011 0:00 | 0 | 49.773147 | -111.036869 | 497345 | 5513409 | 1 | 431.37 | Local |
| M3 | 09/15/2011 0:00 | 3 | 49.772364 | -111.031001 | 497768 | 5513322 | 1 | 1206.36 | Local |
| M3 | 09/15/2011 0:00 | 6 | 49.774911 | -111.014715 | 498940 | 5513605 | 1 | 6.33 | Local |
| M3 | 09/15/2011 0:00 | 9 | 49.774902 | -111.014628 | 498947 | 5513604 | 1 | 5.61 | Local |
| M3 | 09/15/2011 0:00 | 12 | 49.774899 | -111.014706 | 498941 | 5513604 | 0 | 97.77 | Local |
| M3 | 09/15/2011 0:00 | 18 | 49.775017 | -111.016052 | 498844 | 5513617 | 1 | 787.50 | Local |
| M3 | 09/15/2011 0:00 | 21 | 49.772276 | -111.005967 | 499570 | 5513312 | 1 | 2095.03 | Local |
| M3 | 09/16/2011 0:00 | 0 | 49.764428 | -110.979517 | 501475 | 5512439 | 1 | 854.97 | Local |
| M3 | 09/16/2011 0:00 | 3 | 49.760380 | -110.969424 | 502202 | 5511990 | 1 | 682.27 | Local |

| | | | | | | | | | |
|---|---|---|---|---|---|---|---|---|---|
| M3 | 09/16/2011 0:00 | 6 | 49.763854 | -110.977232 | 501640 | 5512376 | 0 | 1024.74 | Local |
| M3 | 09/16/2011 0:00 | 12 | 49.768581 | -110.989449 | 500760 | 5512901 | 0 | 145.14 | Local |
| M3 | 09/16/2011 0:00 | 18 | 49.767322 | -110.989982 | 500721 | 5512761 | 1 | 368.97 | Local |
| M3 | 09/16/2011 0:00 | 21 | 49.766974 | -110.984887 | 501088 | 5512722 | 1 | 282.17 | Local |
| M3 | 09/17/2011 0:00 | 0 | 49.766934 | -110.988805 | 500806 | 5512718 | 1 | 1.53 | Local |
| M3 | 09/17/2011 0:00 | 3 | 49.766925 | -110.988820 | 500805 | 5512717 | 1 | 1141.16 | Local |
| M3 | 09/17/2011 0:00 | 6 | 49.773867 | -111.000492 | 499965 | 5513489 | 1 | 118.48 | Local |
| M3 | 09/17/2011 0:00 | 9 | 49.774723 | -110.999513 | 500035 | 5513584 | 0 | 966.85 | Local |
| M3 | 09/17/2011 0:00 | 18 | 49.778648 | -111.011496 | 499172 | 5514020 | 1 | 937.49 | Local |
| M3 | 09/17/2011 0:00 | 21 | 49.774721 | -111.023019 | 498343 | 5513584 | 1 | 3707.73 | Local |
| M3 | 09/18/2011 0:00 | 0 | 49.741695 | -111.015864 | 498857 | 5509912 | 1 | 3755.05 | Local |
| M3 | 09/18/2011 0:00 | 3 | 49.763755 | -110.976390 | 501700 | 5512365 | 1 | 2065.81 | Local |
| M3 | 09/18/2011 0:00 | 6 | 49.778027 | -110.994760 | 500377 | 5513951 | 1 | 193.93 | Local |
| M3 | 09/18/2011 0:00 | 9 | 49.779505 | -110.996192 | 500274 | 5514116 | 0 | 1370.12 | Local |
| M3 | 09/18/2011 0:00 | 21 | 49.791781 | -110.997851 | 500155 | 5515480 | 1 | 1788.38 | Local |
| M3 | 09/19/2011 0:00 | 0 | 49.801275 | -110.977791 | 501598 | 5516536 | 1 | 5.34 | Local |
| M3 | 09/19/2011 0:00 | 3 | 49.801227 | -110.977781 | 501599 | 5516531 | 1 | 1250.43 | Local |
| M3 | 09/19/2011 0:00 | 6 | 49.795289 | -110.992537 | 500537 | 5515870 | 1 | 1858.84 | Local |
| M3 | 09/19/2011 0:00 | 9 | 49.780210 | -111.003693 | 499734 | 5514194 | 1 | 354.65 | Local |
| M3 | 09/19/2011 0:00 | 12 | 49.777020 | -111.003692 | 499734 | 5513839 | 0 | 613.29 | Local |
| M3 | 09/19/2011 0:00 | 18 | 49.777996 | -111.012075 | 499131 | 5513948 | 1 | 1423.29 | Local |
| M3 | 09/19/2011 0:00 | 21 | 49.783678 | -110.994359 | 500406 | 5514579 | 1 | 1814.67 | Local |
| M3 | 09/20/2011 0:00 | 0 | 49.799116 | -110.986174 | 500995 | 5516296 | 1 | 14.89 | Local |
| M3 | 09/20/2011 0:00 | 3 | 49.799247 | -110.986134 | 500998 | 5516311 | 1 | 1646.47 | Local |
| M3 | 09/20/2011 0:00 | 6 | 49.787961 | -111.000945 | 499932 | 5515056 | 0 | 6.26 | Local |
| M3 | 09/20/2011 0:00 | 21 | 49.787928 | -111.000874 | 499937 | 5515052 | 0 | 802.02 | Local |
| M3 | 09/21/2011 0:00 | 3 | 49.780902 | -111.003396 | 499756 | 5514271 | 1 | 199.37 | Local |
| M3 | 09/21/2011 0:00 | 6 | 49.780904 | -111.006165 | 499556 | 5514271 | 1 | 845.66 | Local |
| M3 | 09/21/2011 0:00 | 9 | 49.775306 | -111.014117 | 498984 | 5513649 | 0 | 1485.93 | Local |
| M3 | 09/21/2011 0:00 | 21 | 49.779738 | -111.033588 | 497582 | 5514142 | 1 | 3619.81 | Local |
| M3 | 09/22/2011 0:00 | 0 | 49.808311 | -111.009480 | 499318 | 5517318 | 1 | 3118.26 | Local |
| M3 | 09/22/2011 0:00 | 3 | 49.825444 | -110.975162 | 501787 | 5519223 | 1 | 2652.94 | Local |
| M3 | 09/22/2011 0:00 | 6 | 49.801685 | -110.978562 | 501543 | 5516582 | 1 | 2823.77 | Local |
| M3 | 09/22/2011 0:00 | 9 | 49.781940 | -111.003240 | 499767 | 5514386 | 0 | 176.70 | Local |
| M3 | 09/22/2011 0:00 | 15 | 49.780730 | -111.004829 | 499652 | 5514252 | 1 | 24.00 | Local |
| M3 | 09/22/2011 0:00 | 18 | 49.780647 | -111.004522 | 499674 | 5514242 | 1 | 1139.49 | Local |
| M3 | 09/22/2011 0:00 | 21 | 49.780457 | -110.988696 | 500814 | 5514221 | 1 | 20.12 | Local |
| M3 | 09/23/2011 0:00 | 0 | 49.780600 | -110.988524 | 500826 | 5514237 | 1 | 40.96 | Local |
| M3 | 09/23/2011 0:00 | 3 | 49.780327 | -110.988906 | 500799 | 5514207 | 1 | 18.86 | Local |
| M3 | 09/23/2011 0:00 | 6 | 49.780416 | -110.988683 | 500815 | 5514217 | 1 | 25.61 | Local |
| M3 | 09/23/2011 0:00 | 9 | 49.780312 | -110.988367 | 500838 | 5514205 | 0 | 35.70 | Local |
| M3 | 09/23/2011 0:00 | 18 | 49.780237 | -110.988849 | 500803 | 5514197 | 1 | 137.44 | Local |
| M3 | 09/23/2011 0:00 | 21 | 49.780402 | -110.986957 | 500939 | 5514215 | 0 | 682.85 | Local |
| M3 | 09/24/2011 0:00 | 3 | 49.780570 | -110.977476 | 501622 | 5514234 | 1 | 1319.27 | Local |
| M3 | 09/24/2011 0:00 | 6 | 49.782749 | -110.995489 | 500325 | 5514476 | 1 | 808.34 | Local |
| M3 | 09/24/2011 0:00 | 9 | 49.777046 | -111.002454 | 499823 | 5513842 | 0 | 17.96 | Local |
| M3 | 09/24/2011 0:00 | 15 | 49.777195 | -111.002356 | 499830 | 5513859 | 1 | 224.45 | Local |
| M3 | 09/24/2011 0:00 | 18 | 49.778544 | -111.000036 | 499997 | 5514009 | 1 | 256.33 | Local |
| M3 | 09/24/2011 0:00 | 21 | 49.780495 | -111.001933 | 499861 | 5514226 | 1 | 78.58 | Local |
| M3 | 09/25/2011 0:00 | 0 | 49.779824 | -111.001588 | 499886 | 5514151 | 0 | 847.08 | Local |
| M3 | 09/25/2011 0:00 | 6 | 49.776624 | -111.012265 | 499117 | 5513795 | 0 | 800.18 | Local |
| M3 | 09/25/2011 0:00 | 21 | 49.772142 | -111.020961 | 498491 | 5513297 | 1 | 440.77 | Local |
| M3 | 09/26/2011 0:00 | 0 | 49.773924 | -111.015493 | 498884 | 5513495 | 1 | 138.80 | Local |
| M3 | 09/26/2011 0:00 | 3 | 49.774621 | -111.017092 | 498769 | 5513573 | 1 | 1244.17 | Local |
| M3 | 09/26/2011 0:00 | 6 | 49.774753 | -110.999814 | 500013 | 5513587 | 1 | 81.17 | Local |

| | | | | | | | | | |
|---|---|---|---|---|---|---|---|---|---|
| M3 | 09/26/2011 0:00 | 9 | 49.774265 | -110.998975 | 500074 | 5513533 | 0 | 276.88 | Local |
| M3 | 09/26/2011 0:00 | 15 | 49.776673 | -110.999951 | 500004 | 5513801 | 1 | 88.51 | Local |
| M3 | 09/26/2011 0:00 | 18 | 49.776880 | -111.001138 | 499918 | 5513824 | 1 | 30.82 | Local |
| M3 | 09/26/2011 0:00 | 21 | 49.776924 | -111.000716 | 499948 | 5513829 | 0 | 231.04 | Local |
| M3 | 09/27/2011 0:00 | 3 | 49.778672 | -110.998980 | 500073 | 5514023 | 1 | 239.15 | Local |
| M3 | 09/27/2011 0:00 | 6 | 49.776846 | -111.000738 | 499947 | 5513820 | 0 | 335.57 | Local |
| M3 | 09/27/2011 0:00 | 15 | 49.778604 | -111.004527 | 499674 | 5514015 | 0 | 190.81 | Local |
| M3 | 09/27/2011 0:00 | 21 | 49.778244 | -111.001935 | 499861 | 5513975 | 1 | 171.59 | Local |
| M3 | 09/28/2011 0:00 | 0 | 49.776872 | -111.000844 | 499939 | 5513823 | 1 | 11.31 | Local |
| M3 | 09/28/2011 0:00 | 3 | 49.776780 | -111.000911 | 499934 | 5513813 | 1 | 4.40 | Local |
| M3 | 09/28/2011 0:00 | 6 | 49.776764 | -111.000856 | 499938 | 5513811 | 1 | 330.57 | Local |
| M3 | 09/28/2011 0:00 | 9 | 49.778474 | -111.004612 | 499668 | 5514001 | 0 | 116.38 | Local |
| M3 | 09/28/2011 0:00 | 18 | 49.777510 | -111.003981 | 499713 | 5513894 | 1 | 209.12 | Local |
| M3 | 09/28/2011 0:00 | 21 | 49.776939 | -111.001214 | 499913 | 5513830 | 1 | 28.32 | Local |
| M3 | 09/29/2011 0:00 | 0 | 49.776812 | -111.000873 | 499937 | 5513816 | 1 | 143.93 | Local |
| M3 | 09/29/2011 0:00 | 3 | 49.775795 | -111.002111 | 499848 | 5513703 | 1 | 135.87 | Local |
| M3 | 09/29/2011 0:00 | 6 | 49.776713 | -111.000865 | 499938 | 5513805 | 1 | 488.74 | Local |
| M3 | 09/29/2011 0:00 | 9 | 49.779048 | -111.006616 | 499524 | 5514065 | 0 | 268.85 | Local |
| M3 | 09/30/2011 0:00 | 0 | 49.779189 | -111.002888 | 499792 | 5514080 | 1 | 467.45 | Local |
| M3 | 09/30/2011 0:00 | 3 | 49.775054 | -111.001707 | 499877 | 5513621 | 1 | 361.65 | Local |
| M3 | 09/30/2011 0:00 | 6 | 49.778042 | -110.999720 | 500020 | 5513953 | 1 | 921.65 | Local |
| M3 | 09/30/2011 0:00 | 9 | 49.778361 | -111.012512 | 499099 | 5513988 | 0 | 892.29 | Local |
| M3 | 09/30/2011 0:00 | 18 | 49.770881 | -111.008020 | 499423 | 5513157 | 1 | 713.23 | Local |
| M3 | 09/30/2011 0:00 | 21 | 49.775335 | -111.000891 | 499936 | 5513652 | 1 | 198.40 | Local |
| M3 | 10/01/2011 0:00 | 0 | 49.777096 | -111.000450 | 499968 | 5513848 | 1 | 44.06 | Local |
| M3 | 10/01/2011 0:00 | 3 | 49.776859 | -111.000941 | 499932 | 5513821 | 1 | 201.33 | Local |
| M3 | 10/01/2011 0:00 | 6 | 49.778665 | -111.001150 | 499917 | 5514022 | 1 | 240.53 | Local |
| M3 | 10/01/2011 0:00 | 9 | 49.777857 | -111.004249 | 499694 | 5513932 | 0 | 803.42 | Local |
| M3 | 10/01/2011 0:00 | 15 | 49.784950 | -111.006388 | 499540 | 5514721 | 1 | 910.44 | Local |
| M3 | 10/01/2011 0:00 | 18 | 49.777547 | -111.000983 | 499929 | 5513898 | 1 | 253.70 | Local |
| M3 | 10/01/2011 0:00 | 21 | 49.775284 | -111.000523 | 499962 | 5513646 | 1 | 681.60 | Local |
| M3 | 10/02/2011 0:00 | 0 | 49.778666 | -111.008420 | 499394 | 5514022 | 1 | 15.89 | Local |
| M3 | 10/02/2011 0:00 | 3 | 49.778781 | -111.008551 | 499384 | 5514035 | 1 | 252.12 | Local |
| M3 | 10/02/2011 0:00 | 6 | 49.778068 | -111.011875 | 499145 | 5513956 | 0 | 483.33 | Local |
| M3 | 10/02/2011 0:00 | 18 | 49.774540 | -111.015796 | 498863 | 5513564 | 1 | 108.76 | Local |
| M3 | 10/02/2011 0:00 | 21 | 49.775135 | -111.014598 | 498949 | 5513630 | 1 | 1494.67 | Local |
| M3 | 10/03/2011 0:00 | 0 | 49.775031 | -111.035356 | 497454 | 5513619 | 0 | 1462.86 | Local |
| M3 | 10/03/2011 0:00 | 6 | 49.765055 | -111.048602 | 496500 | 5512510 | 1 | 2644.01 | Local |
| M3 | 10/03/2011 0:00 | 9 | 49.775019 | -111.015262 | 498901 | 5513617 | 0 | 38.80 | Local |
| M3 | 10/03/2011 0:00 | 15 | 49.774798 | -111.015680 | 498871 | 5513592 | 1 | 62.01 | Local |
| M3 | 10/03/2011 0:00 | 18 | 49.775336 | -111.015448 | 498888 | 5513652 | 1 | 1346.42 | Local |
| M3 | 10/03/2011 0:00 | 21 | 49.781362 | -110.999227 | 500056 | 5514322 | 1 | 194.73 | Local |
| M3 | 10/04/2011 0:00 | 0 | 49.782167 | -111.001630 | 499883 | 5514411 | 1 | 1202.38 | Local |
| M3 | 10/04/2011 0:00 | 3 | 49.781622 | -111.018311 | 498682 | 5514351 | 1 | 764.82 | Local |
| M3 | 10/04/2011 0:00 | 6 | 49.774941 | -111.015776 | 498864 | 5513608 | 1 | 406.40 | Local |
| M3 | 10/04/2011 0:00 | 9 | 49.778498 | -111.017077 | 498771 | 5514004 | 0 | 330.00 | Local |
| M3 | 10/04/2011 0:00 | 15 | 49.778327 | -111.012501 | 499100 | 5513985 | 1 | 417.22 | Local |
| M3 | 10/04/2011 0:00 | 18 | 49.774858 | -111.014712 | 498941 | 5513599 | 1 | 254.17 | Local |
| M3 | 10/04/2011 0:00 | 21 | 49.773473 | -111.017520 | 498738 | 5513445 | 1 | 971.67 | Local |
| M3 | 10/05/2011 0:00 | 0 | 49.771171 | -111.030538 | 497801 | 5513189 | 1 | 1007.09 | Local |
| M3 | 10/05/2011 0:00 | 3 | 49.769978 | -111.044402 | 496803 | 5513057 | 1 | 313.16 | Local |
| M3 | 10/05/2011 0:00 | 6 | 49.768432 | -111.048038 | 496541 | 5512886 | 1 | 1279.72 | Local |
| M3 | 10/05/2011 0:00 | 9 | 49.774614 | -111.033046 | 497621 | 5513572 | 0 | 612.85 | Local |
| M3 | 10/05/2011 0:00 | 15 | 49.770505 | -111.027372 | 498029 | 5513115 | 1 | 620.83 | Local |
| M3 | 10/05/2011 0:00 | 18 | 49.773641 | -111.020239 | 498543 | 5513464 | 1 | 3370.71 | Local |

| | | | | | | | | | |
|---|---|---|---|---|---|---|---|---|---|
| M3 | 10/05/2011 0:00 | 21 | 49.762652 | -110.976614 | 501684 | 5512242 | 1 | 2947.79 | Local |
| M3 | 10/06/2011 0:00 | 0 | 49.739753 | -110.955985 | 503171 | 5509697 | 1 | 5542.01 | Local |
| M3 | 10/06/2011 0:00 | 3 | 49.770575 | -111.016454 | 498815 | 5513123 | 0 | 1937.49 | Local |
| M3 | 10/06/2011 0:00 | 21 | 49.779479 | -110.993322 | 500481 | 5514113 | 0 | 313.49 | Local |
| M3 | 10/07/2011 0:00 | 3 | 49.781167 | -110.989834 | 500732 | 5514300 | 1 | 1267.84 | Local |
| M3 | 10/07/2011 0:00 | 6 | 49.776343 | -111.005791 | 499583 | 5513764 | 1 | 369.43 | Local |
| M3 | 10/07/2011 0:00 | 9 | 49.776541 | -111.010913 | 499214 | 5513786 | 0 | 463.97 | Local |
| M3 | 10/07/2011 0:00 | 18 | 49.780622 | -111.009566 | 499311 | 5514240 | 1 | 95.04 | Local |
| M3 | 10/07/2011 0:00 | 21 | 49.780639 | -111.008246 | 499406 | 5514242 | 1 | 147.06 | Local |
| M3 | 10/08/2011 0:00 | 0 | 49.781959 | -111.008128 | 499415 | 5514388 | 1 | 407.39 | Local |
| M3 | 10/08/2011 0:00 | 3 | 49.778360 | -111.009185 | 499339 | 5513988 | 0 | 249.53 | Local |
| M3 | 10/08/2011 0:00 | 9 | 49.780006 | -111.011540 | 499169 | 5514171 | 1 | 202.83 | Local |
| M3 | 10/08/2011 0:00 | 12 | 49.781746 | -111.010692 | 499230 | 5514365 | 1 | 162.55 | Local |
| M3 | 10/08/2011 0:00 | 15 | 49.780476 | -111.009572 | 499311 | 5514224 | 1 | 75.99 | Local |
| M3 | 10/08/2011 0:00 | 18 | 49.781137 | -111.009302 | 499330 | 5514297 | 1 | 73.70 | Local |
| M3 | 10/08/2011 0:00 | 21 | 49.780493 | -111.009547 | 499313 | 5514225 | 1 | 167.64 | Local |
| M3 | 10/09/2011 0:00 | 0 | 49.781519 | -111.007841 | 499436 | 5514340 | 1 | 167.92 | Local |
| M3 | 10/09/2011 0:00 | 3 | 49.780528 | -111.009601 | 499309 | 5514229 | 1 | 593.28 | Local |
| M3 | 10/09/2011 0:00 | 6 | 49.775508 | -111.012395 | 499108 | 5513671 | 1 | 71.42 | Local |
| M3 | 10/09/2011 0:00 | 9 | 49.775260 | -111.013310 | 499042 | 5513644 | 1 | 232.01 | Local |
| M3 | 10/09/2011 0:00 | 12 | 49.776643 | -111.010896 | 499216 | 5513797 | 0 | 1307.16 | Local |
| M3 | 10/10/2011 0:00 | 0 | 49.772223 | -111.027719 | 498004 | 5513306 | 0 | 983.50 | Local |
| M3 | 10/10/2011 0:00 | 6 | 49.777281 | -111.016513 | 498811 | 5513868 | 0 | 455.57 | Local |
| M3 | 10/10/2011 0:00 | 15 | 49.780471 | -111.012541 | 499097 | 5514223 | 1 | 918.46 | Local |
| M3 | 10/10/2011 0:00 | 18 | 49.785629 | -111.022506 | 498380 | 5514797 | 1 | 1050.45 | Local |
| M3 | 10/10/2011 0:00 | 21 | 49.778168 | -111.013554 | 499024 | 5513967 | 0 | 900.62 | Local |
| M3 | 10/11/2011 0:00 | 6 | 49.779766 | -111.001291 | 499907 | 5514145 | 1 | 111.21 | Local |
| M3 | 10/11/2011 0:00 | 9 | 49.779057 | -111.002379 | 499829 | 5514066 | 0 | 462.86 | Local |
| M3 | 10/11/2011 0:00 | 15 | 49.783155 | -111.001247 | 499910 | 5514521 | 1 | 466.43 | Local |
| M3 | 10/11/2011 0:00 | 18 | 49.780848 | -111.006658 | 499521 | 5514265 | 1 | 219.20 | Local |
| M3 | 10/11/2011 0:00 | 21 | 49.779291 | -111.008527 | 499386 | 5514092 | 1 | 71.35 | Local |
| M3 | 10/12/2011 0:00 | 0 | 49.779920 | -111.008329 | 499400 | 5514162 | 1 | 216.19 | Local |
| M3 | 10/12/2011 0:00 | 3 | 49.778892 | -111.005780 | 499584 | 5514047 | 1 | 139.11 | Local |
| M3 | 10/12/2011 0:00 | 6 | 49.777651 | -111.005535 | 499601 | 5513909 | 1 | 105.05 | Local |
| M3 | 10/12/2011 0:00 | 9 | 49.778580 | -111.005795 | 499583 | 5514013 | 0 | 19.83 | Local |
| M3 | 10/12/2011 0:00 | 15 | 49.778758 | -111.005809 | 499582 | 5514033 | 1 | 3.00 | Local |
| M3 | 10/12/2011 0:00 | 18 | 49.778784 | -111.005796 | 499583 | 5514035 | 1 | 69.35 | Local |
| M3 | 10/12/2011 0:00 | 21 | 49.778244 | -111.005314 | 499617 | 5513975 | 0 | 26.32 | Local |
| M3 | 10/13/2011 0:00 | 3 | 49.778070 | -111.005562 | 499600 | 5513956 | 1 | 97.07 | Local |
| M3 | 10/13/2011 0:00 | 6 | 49.778900 | -111.005977 | 499570 | 5514048 | 1 | 11.04 | Local |
| M3 | 10/13/2011 0:00 | 9 | 49.778801 | -111.005976 | 499570 | 5514037 | 1 | 1.31 | Local |
| M3 | 10/13/2011 0:00 | 12 | 49.778809 | -111.005963 | 499571 | 5514038 | 1 | 12.68 | Local |
| M3 | 10/13/2011 0:00 | 15 | 49.778723 | -111.005848 | 499579 | 5514029 | 1 | 20.96 | Local |
| M3 | 10/13/2011 0:00 | 18 | 49.778900 | -111.005751 | 499586 | 5514048 | 1 | 7.31 | Local |
| M3 | 10/13/2011 0:00 | 21 | 49.778841 | -111.005794 | 499583 | 5514042 | 1 | 8.78 | Local |
| M3 | 10/14/2011 0:00 | 0 | 49.778762 | -111.005785 | 499584 | 5514033 | 1 | 5.35 | Local |
| M3 | 10/14/2011 0:00 | 3 | 49.778719 | -111.005819 | 499581 | 5514028 | 1 | 35.76 | Local |
| M3 | 10/14/2011 0:00 | 6 | 49.778840 | -111.006279 | 499548 | 5514042 | 1 | 28.66 | Local |
| M3 | 10/14/2011 0:00 | 9 | 49.778909 | -111.005895 | 499576 | 5514049 | 0 | 12.94 | Local |
| M3 | 10/14/2011 0:00 | 18 | 49.778795 | -111.005861 | 499578 | 5514037 | 1 | 10.63 | Local |
| M3 | 10/14/2011 0:00 | 21 | 49.778882 | -111.005799 | 499583 | 5514046 | 1 | 104.06 | Local |
| M3 | 10/15/2011 0:00 | 0 | 49.779790 | -111.006150 | 499557 | 5514147 | 1 | 60.10 | Local |
| M3 | 10/15/2011 0:00 | 3 | 49.779254 | -111.006262 | 499549 | 5514088 | 1 | 71.60 | Local |
| M3 | 10/15/2011 0:00 | 6 | 49.778678 | -111.005818 | 499581 | 5514024 | 1 | 251.99 | Local |
| M3 | 10/15/2011 0:00 | 9 | 49.778380 | -111.009287 | 499331 | 5513990 | 1 | 161.92 | Local |

| | | | | | | | | | |
|---|---|---|---|---|---|---|---|---|---|
| M3 | 10/15/2011 0:00 | 12 | 49.778406 | -111.007039 | 499493 | 5513993 | 1 | 88.09 | Local |
| M3 | 10/15/2011 0:00 | 15 | 49.778318 | -111.005823 | 499581 | 5513984 | 1 | 61.84 | Local |
| M3 | 10/15/2011 0:00 | 18 | 49.778875 | -111.005842 | 499579 | 5514045 | 1 | 4.17 | Local |
| M3 | 10/15/2011 0:00 | 21 | 49.778874 | -111.005784 | 499584 | 5514045 | 1 | 15.76 | Local |
| M3 | 10/16/2011 0:00 | 0 | 49.778739 | -111.005852 | 499579 | 5514030 | 1 | 24.37 | Local |
| M3 | 10/16/2011 0:00 | 3 | 49.778949 | -111.005755 | 499586 | 5514054 | 1 | 7.07 | Local |
| M3 | 10/16/2011 0:00 | 6 | 49.778886 | -111.005751 | 499586 | 5514047 | 1 | 20.09 | Local |
| M3 | 10/16/2011 0:00 | 9 | 49.778745 | -111.005925 | 499573 | 5514031 | 0 | 115.94 | Local |
| M3 | 10/16/2011 0:00 | 18 | 49.779726 | -111.006471 | 499534 | 5514140 | 1 | 106.07 | Local |
| M3 | 10/16/2011 0:00 | 21 | 49.778897 | -111.005742 | 499587 | 5514048 | 1 | 332.96 | Local |
| M3 | 10/17/2011 0:00 | 0 | 49.778011 | -111.010160 | 499269 | 5513950 | 1 | 331.18 | Local |
| M3 | 10/17/2011 0:00 | 3 | 49.778961 | -111.005800 | 499582 | 5514055 | 1 | 11.72 | Local |
| M3 | 10/17/2011 0:00 | 6 | 49.778856 | -111.005783 | 499584 | 5514043 | 0 | 56.13 | Local |
| M3 | 10/17/2011 0:00 | 15 | 49.778605 | -111.005107 | 499632 | 5514015 | 1 | 50.67 | Local |
| M3 | 10/17/2011 0:00 | 18 | 49.778790 | -111.005750 | 499586 | 5514036 | 1 | 13.25 | Local |
| M3 | 10/17/2011 0:00 | 21 | 49.778903 | -111.005805 | 499582 | 5514049 | 1 | 18.43 | Local |
| M3 | 10/18/2011 0:00 | 0 | 49.778755 | -111.005692 | 499590 | 5514032 | 0 | 17.54 | Local |
| M3 | 10/18/2011 0:00 | 6 | 49.778847 | -111.005889 | 499576 | 5514042 | 1 | 5.91 | Local |
| M3 | 10/18/2011 0:00 | 9 | 49.778853 | -111.005971 | 499570 | 5514043 | 0 | 158.50 | Local |
| M3 | 10/18/2011 0:00 | 15 | 49.780161 | -111.005097 | 499633 | 5514188 | 0 | 150.35 | Local |
| M3 | 10/18/2011 0:00 | 21 | 49.778887 | -111.005798 | 499583 | 5514047 | 1 | 11.58 | Local |
| M3 | 10/19/2011 0:00 | 0 | 49.778803 | -111.005703 | 499589 | 5514037 | 1 | 14.53 | Local |
| M3 | 10/19/2011 0:00 | 3 | 49.778931 | -111.005745 | 499586 | 5514052 | 1 | 22.67 | Local |
| M3 | 10/19/2011 0:00 | 6 | 49.778774 | -111.005946 | 499572 | 5514034 | 1 | 102.96 | Local |
| M3 | 10/19/2011 0:00 | 9 | 49.778350 | -111.007217 | 499480 | 5513987 | 0 | 2517.14 | Local |
| M3 | 10/21/2011 0:00 | 0 | 49.771860 | -111.040710 | 497069 | 5513266 | 1 | 1.32 | Local |
| M3 | 10/21/2011 0:00 | 3 | 49.771850 | -111.040700 | 497069 | 5513265 | 1 | 1217.50 | Local |
| M3 | 10/21/2011 0:00 | 6 | 49.762270 | -111.048890 | 496479 | 5512200 | 1 | 2372.28 | Local |
| M3 | 10/21/2011 0:00 | 9 | 49.742420 | -111.036810 | 497348 | 5509993 | 1 | 3.10 | Local |
| M3 | 10/21/2011 0:00 | 12 | 49.742400 | -111.036840 | 497346 | 5509991 | 0 | 3.41 | Local |
| M3 | 10/21/2011 0:00 | 18 | 49.742430 | -111.036830 | 497346 | 5509994 | 1 | 3835.23 | Local |
| M3 | 10/21/2011 0:00 | 21 | 49.774400 | -111.016830 | 498788 | 5513548 | 1 | 527.15 | Local |
| M3 | 10/22/2011 0:00 | 3 | 49.759530 | -110.963400 | 502636 | 5511895 | 1 | 3710.09 | Trans |
| M3 | 10/22/2011 0:00 | 6 | 49.737910 | -110.924170 | 505464 | 5509494 | 1 | 673.52 | Trans |
| M3 | 10/22/2011 0:00 | 9 | 49.731910 | -110.922880 | 505558 | 5508827 | 1 | 6.66 | Trans |
| M3 | 10/22/2011 0:00 | 12 | 49.731880 | -110.922960 | 505552 | 5508823 | 0 | 1.11 | Trans |
| M3 | 10/22/2011 0:00 | 18 | 49.731890 | -110.922960 | 505552 | 5508825 | 1 | 3326.20 | Trans |
| M3 | 10/22/2011 0:00 | 21 | 49.731120 | -110.876820 | 508877 | 5508743 | 1 | 5989.64 | Trans |
| M3 | 10/22/2011 0:00 | 0 | 49.778350 | -111.012780 | 499080 | 5513987 | 1 | 4125.80 | Local |
| M3 | 10/23/2011 0:00 | 0 | 49.733700 | -110.793800 | 514859 | 5509043 | 1 | 2835.43 | Trans |
| M3 | 10/23/2011 0:00 | 3 | 49.746120 | -110.759430 | 517332 | 5510432 | 0 | 2438.79 | Trans |
| M3 | 10/23/2011 0:00 | 9 | 49.759040 | -110.732070 | 519298 | 5511875 | 1 | 2.43 | Trans |
| M3 | 10/23/2011 0:00 | 12 | 49.759050 | -110.732040 | 519300 | 5511876 | 1 | 0.72 | Trans |
| M3 | 10/23/2011 0:00 | 15 | 49.759050 | -110.732030 | 519300 | 5511876 | 0 | 4768.99 | Trans |
| M3 | 10/23/2011 0:00 | 21 | 49.765390 | -110.666540 | 524014 | 5512600 | 1 | 5835.56 | Trans |
| M3 | 10/24/2011 0:00 | 0 | 49.780890 | -110.589110 | 529581 | 5514351 | 1 | 8932.00 | Trans |
| M3 | 10/24/2011 0:00 | 3 | 49.798620 | -110.468080 | 538280 | 5516376 | 1 | 5708.86 | Trans |
| M3 | 10/24/2011 0:00 | 6 | 49.804020 | -110.389190 | 543953 | 5517020 | 1 | 43.87 | Trans |
| M3 | 10/24/2011 0:00 | 9 | 49.803950 | -110.388590 | 543996 | 5517013 | 1 | 1.44 | Trans |
| M3 | 10/24/2011 0:00 | 12 | 49.803950 | -110.388610 | 543994 | 5517013 | 1 | 3.41 | Trans |
| M3 | 10/24/2011 0:00 | 15 | 49.803920 | -110.388620 | 543994 | 5517009 | 1 | 14.45 | Trans |
| M3 | 10/24/2011 0:00 | 18 | 49.804050 | -110.388620 | 543994 | 5517024 | 1 | 5629.38 | Trans |
| M3 | 10/24/2011 0:00 | 21 | 49.778400 | -110.321190 | 548871 | 5514214 | 1 | 2719.16 | Trans |
| M3 | 10/25/2011 0:00 | 0 | 49.767100 | -110.287700 | 551294 | 5512980 | 1 | 4.23 | Trans |
| M3 | 10/25/2011 0:00 | 3 | 49.767120 | -110.287750 | 551291 | 5512982 | 1 | 2.65 | Trans |

| | | | | | | | | | | |
|---|---|---|---|---|---|---|---|---|---|---|
| M3 | 10/25/2011 0:00 | 6 | 49.767100 | -110.287730 | 551292 | 5512980 | 1 | 8957.12 | Trans |
| M3 | 10/25/2011 0:00 | 9 | 49.687380 | -110.269810 | 552669 | 5504129 | 1 | 1155.78 | Trans |
| M3 | 10/25/2011 0:00 | 12 | 49.677350 | -110.265600 | 552983 | 5503017 | 1 | 340.04 | Trans |
| M3 | 10/25/2011 0:00 | 15 | 49.674300 | -110.265250 | 553012 | 5502678 | 1 | 14.74 | Trans |
| M3 | 10/25/2011 0:00 | 18 | 49.674170 | -110.265210 | 553015 | 5502664 | 1 | 343.85 | Trans |
| M3 | 10/25/2011 0:00 | 21 | 49.676450 | -110.261990 | 553245 | 5502919 | 1 | 229.99 | Trans |
| M3 | 10/26/2011 0:00 | 0 | 49.677590 | -110.264650 | 553052 | 5503044 | 1 | 321.37 | Trans |
| M3 | 10/26/2011 0:00 | 3 | 49.678150 | -110.269020 | 552736 | 5503103 | 1 | 5.52 | Trans |
| M3 | 10/26/2011 0:00 | 6 | 49.678130 | -110.268950 | 552741 | 5503101 | 1 | 260.25 | Trans |
| M3 | 10/26/2011 0:00 | 9 | 49.677330 | -110.265560 | 552986 | 5503015 | 1 | 431.36 | Trans |
| M3 | 10/26/2011 0:00 | 12 | 49.675510 | -110.260280 | 553369 | 5502816 | 1 | 34.29 | Trans |
| M3 | 10/26/2011 0:00 | 15 | 49.675210 | -110.260390 | 553362 | 5502783 | 1 | 114.46 | Trans |
| M3 | 10/26/2011 0:00 | 18 | 49.675800 | -110.259090 | 553455 | 5502849 | 1 | 1687.31 | Trans |
| M3 | 10/26/2011 0:00 | 21 | 49.690690 | -110.263610 | 553112 | 5504501 | 1 | 2078.65 | Trans |
| M3 | 10/27/2011 0:00 | 0 | 49.672000 | -110.264330 | 553081 | 5502423 | 1 | 756.07 | Trans |
| M3 | 10/27/2011 0:00 | 3 | 49.678110 | -110.268930 | 552742 | 5503099 | 0 | 5.73 | Trans |
| M3 | 10/27/2011 0:00 | 9 | 49.678150 | -110.268980 | 552739 | 5503103 | 1 | 118.77 | Trans |
| M3 | 10/27/2011 0:00 | 12 | 49.678510 | -110.267430 | 552850 | 5503145 | 1 | 197.51 | Trans |
| M3 | 10/27/2011 0:00 | 15 | 49.677290 | -110.265440 | 552995 | 5503010 | 1 | 49.00 | Trans |
| M3 | 10/27/2011 0:00 | 18 | 49.677110 | -110.266060 | 552951 | 5502990 | 1 | 971.47 | Trans |
| M3 | 10/27/2011 0:00 | 21 | 49.684410 | -110.258660 | 553476 | 5503807 | 1 | 1022.10 | Trans |
| M3 | 10/28/2011 0:00 | 0 | 49.678050 | -110.268890 | 552745 | 5503092 | 0 | 13.71 | Trans |
| M3 | 10/28/2011 0:00 | 6 | 49.678140 | -110.269020 | 552736 | 5503102 | 1 | 12.53 | Trans |
| M3 | 10/28/2011 0:00 | 9 | 49.678240 | -110.268940 | 552742 | 5503113 | 1 | 274.04 | Trans |
| M3 | 10/28/2011 0:00 | 12 | 49.677330 | -110.265410 | 552997 | 5503015 | 1 | 487.37 | Trans |
| M3 | 10/28/2011 0:00 | 15 | 49.675420 | -110.259330 | 553438 | 5502807 | 1 | 389.51 | Trans |
| M3 | 10/28/2011 0:00 | 18 | 49.677780 | -110.255340 | 553723 | 5503072 | 1 | 2849.38 | Trans |
| M3 | 10/28/2011 0:00 | 21 | 49.673870 | -110.294370 | 550911 | 5502610 | 1 | 1558.09 | Trans |
| M3 | 10/29/2011 0:00 | 0 | 49.666730 | -110.312950 | 549578 | 5501804 | 1 | 2658.38 | Trans |
| M3 | 10/29/2011 0:00 | 3 | 49.665510 | -110.349740 | 546925 | 5501645 | 1 | 86.32 | Trans |
| M3 | 10/29/2011 0:00 | 6 | 49.664890 | -110.350460 | 546873 | 5501575 | 1 | 285.56 | Trans |
| M3 | 10/29/2011 0:00 | 9 | 49.665560 | -110.354280 | 546597 | 5501647 | 1 | 10.11 | Trans |
| M3 | 10/29/2011 0:00 | 12 | 49.665470 | -110.354300 | 546596 | 5501637 | 0 | 287.06 | Trans |
| M3 | 10/29/2011 0:00 | 18 | 49.664750 | -110.350480 | 546872 | 5501560 | 0 | 1259.44 | Trans |
| M3 | 10/30/2011 0:00 | 0 | 49.663260 | -110.367780 | 545625 | 5501383 | 1 | 2705.20 | Trans |
| M3 | 10/30/2011 0:00 | 3 | 49.661130 | -110.405120 | 542932 | 5501125 | 1 | 4457.16 | Trans |
| M3 | 10/30/2011 0:00 | 6 | 49.646750 | -110.462760 | 538784 | 5499495 | 1 | 338.34 | Trans |
| M3 | 10/30/2011 0:00 | 9 | 49.647990 | -110.467040 | 538474 | 5499630 | 1 | 110.11 | Trans |
| M3 | 10/30/2011 0:00 | 12 | 49.647010 | -110.466820 | 538490 | 5499521 | 1 | 0.72 | Trans |
| M3 | 10/30/2011 0:00 | 15 | 49.647010 | -110.466810 | 538491 | 5499521 | 1 | 3.09 | Trans |
| M3 | 10/30/2011 0:00 | 18 | 49.647000 | -110.466770 | 538494 | 5499520 | 1 | 5136.80 | Trans |
| M3 | 10/30/2011 0:00 | 21 | 49.649760 | -110.537800 | 533364 | 5499793 | 1 | 4789.82 | Trans |
| M3 | 10/31/2011 0:00 | 0 | 49.664280 | -110.600280 | 528846 | 5501382 | 1 | 6084.26 | Trans |
| M3 | 10/31/2011 0:00 | 3 | 49.678270 | -110.681800 | 522956 | 5502909 | 1 | 4727.72 | Trans |
| M3 | 10/31/2011 0:00 | 6 | 49.695340 | -110.741830 | 518619 | 5504790 | 1 | 58.09 | Trans |
| M3 | 10/31/2011 0:00 | 9 | 49.695060 | -110.741150 | 518668 | 5504759 | 1 | 7.92 | Trans |
| M3 | 10/31/2011 0:00 | 12 | 49.694990 | -110.741170 | 518667 | 5504751 | 1 | 4.68 | Trans |
| M3 | 10/31/2011 0:00 | 15 | 49.695030 | -110.741190 | 518665 | 5504756 | 1 | 2.65 | Trans |
| M3 | 10/31/2011 0:00 | 18 | 49.695050 | -110.741210 | 518664 | 5504758 | 1 | 408.69 | Trans |
| M3 | 10/31/2011 0:00 | 21 | 49.697250 | -110.745750 | 518336 | 5505001 | 1 | 4.68 | Trans |
| M3 | 11/01/2011 0:00 | 0 | 49.697290 | -110.745770 | 518334 | 5505006 | 1 | 7.82 | Trans |
| M3 | 11/01/2011 0:00 | 3 | 49.697220 | -110.745760 | 518335 | 5504998 | 1 | 3.77 | Trans |
| M3 | 11/01/2011 0:00 | 6 | 49.697230 | -110.745710 | 518338 | 5504999 | 1 | 407.63 | Trans |
| M3 | 11/01/2011 0:00 | 9 | 49.695020 | -110.741200 | 518664 | 5504755 | 0 | 1.44 | Trans |
| M3 | 11/01/2011 0:00 | 15 | 49.695020 | -110.741220 | 518663 | 5504755 | 1 | 408.93 | Trans |

| | | | | | | | | | |
|---|---|---|---|---|---|---|---|---|---|
| M3 | 11/01/2011 0:00 | 18 | 49.697410 | -110.745530 | 518351 | 5505019 | 1 | 634.63 | Trans |
| M3 | 11/01/2011 0:00 | 21 | 49.700920 | -110.752470 | 517850 | 5505408 | 0 | 1189.37 | Trans |
| M3 | 11/02/2011 0:00 | 3 | 49.710890 | -110.758450 | 517415 | 5506515 | 0 | 15.78 | Trans |
| M3 | 11/02/2011 0:00 | 9 | 49.710930 | -110.758240 | 517430 | 5506519 | 1 | 2.16 | Trans |
| M3 | 11/02/2011 0:00 | 12 | 49.710930 | -110.758270 | 517428 | 5506519 | 1 | 4.24 | Trans |
| M3 | 11/02/2011 0:00 | 15 | 49.710950 | -110.758320 | 517424 | 5506522 | 1 | 2.22 | Trans |
| M3 | 11/02/2011 0:00 | 18 | 49.710970 | -110.758320 | 517424 | 5506524 | 1 | 21.28 | Trans |
| M3 | 11/02/2011 0:00 | 21 | 49.711090 | -110.758090 | 517441 | 5506537 | 1 | 10.34 | Trans |
| M3 | 11/03/2011 0:00 | 0 | 49.711070 | -110.758230 | 517431 | 5506535 | 1 | 18.70 | Trans |
| M3 | 11/03/2011 0:00 | 3 | 49.710910 | -110.758310 | 517425 | 5506517 | 0 | 14.57 | Trans |
| M3 | 11/03/2011 0:00 | 9 | 49.710990 | -110.758470 | 517413 | 5506526 | 1 | 8.47 | Trans |
| M3 | 11/03/2011 0:00 | 12 | 49.710950 | -110.758370 | 517421 | 5506522 | 1 | 0.72 | Trans |
| M3 | 11/03/2011 0:00 | 15 | 49.710950 | -110.758380 | 517420 | 5506522 | 1 | 3.10 | Trans |
| M3 | 11/03/2011 0:00 | 18 | 49.710970 | -110.758350 | 517422 | 5506524 | 1 | 1618.39 | Trans |
| M3 | 11/03/2011 0:00 | 21 | 49.724150 | -110.767880 | 516730 | 5507987 | 1 | 1789.34 | Trans |
| M3 | 11/04/2011 0:00 | 0 | 49.739440 | -110.775630 | 516167 | 5509685 | 1 | 485.51 | Trans |
| M3 | 11/04/2011 0:00 | 3 | 49.735390 | -110.773110 | 516350 | 5509236 | 1 | 1450.77 | Trans |
| M3 | 11/04/2011 0:00 | 6 | 49.722860 | -110.767490 | 516759 | 5507844 | 1 | 18.55 | Trans |
| M3 | 11/04/2011 0:00 | 9 | 49.722720 | -110.767350 | 516769 | 5507828 | 1 | 3.41 | Trans |
| M3 | 11/04/2011 0:00 | 12 | 49.722690 | -110.767340 | 516770 | 5507825 | 1 | 2.34 | Trans |
| M3 | 11/04/2011 0:00 | 15 | 49.722710 | -110.767350 | 516769 | 5507827 | 1 | 79.70 | Trans |
| M3 | 11/04/2011 0:00 | 18 | 49.722020 | -110.767050 | 516791 | 5507750 | 1 | 482.24 | Trans |
| M3 | 11/04/2011 0:00 | 21 | 49.725310 | -110.762690 | 517104 | 5508117 | 1 | 583.59 | Trans |
| M3 | 11/05/2011 0:00 | 0 | 49.728360 | -110.769280 | 516628 | 5508455 | 1 | 143.88 | Trans |
| M3 | 11/05/2011 0:00 | 3 | 49.729650 | -110.769120 | 516639 | 5508598 | 1 | 772.42 | Trans |
| M3 | 11/05/2011 0:00 | 6 | 49.722770 | -110.767630 | 516749 | 5507834 | 1 | 9.73 | Trans |
| M3 | 11/05/2011 0:00 | 9 | 49.722810 | -110.767510 | 516757 | 5507838 | 1 | 14.52 | Trans |
| M3 | 11/05/2011 0:00 | 12 | 49.722740 | -110.767340 | 516770 | 5507830 | 1 | 8.47 | Trans |
| M3 | 11/05/2011 0:00 | 15 | 49.722780 | -110.767440 | 516763 | 5507835 | 1 | 10.41 | Trans |
| M3 | 11/05/2011 0:00 | 18 | 49.722870 | -110.767400 | 516765 | 5507845 | 1 | 3944.21 | Trans |
| M3 | 11/05/2011 0:00 | 21 | 49.702930 | -110.722150 | 520035 | 5505639 | 1 | 8769.69 | Trans |
| M3 | 11/06/2011 0:00 | 0 | 49.639530 | -110.649840 | 525282 | 5498612 | 1 | 9017.37 | Trans |
| M3 | 11/06/2011 0:00 | 3 | 49.593480 | -110.547080 | 532732 | 5493532 | 1 | 6524.93 | Trans |
| M3 | 11/06/2011 0:00 | 6 | 49.618500 | -110.465390 | 538616 | 5496352 | 1 | 243.11 | Local |
| M3 | 11/06/2011 0:00 | 9 | 49.618710 | -110.462040 | 538858 | 5496378 | 1 | 1.11 | Local |
| M3 | 11/06/2011 0:00 | 12 | 49.618700 | -110.462040 | 538858 | 5496376 | 1 | 42.63 | Local |
| M3 | 11/06/2011 0:00 | 15 | 49.618710 | -110.461450 | 538901 | 5496378 | 1 | 83.52 | Local |
| M3 | 11/06/2011 0:00 | 18 | 49.619430 | -110.461120 | 538924 | 5496458 | 1 | 872.55 | Local |
| M3 | 11/06/2011 0:00 | 21 | 49.611620 | -110.459930 | 539016 | 5495590 | 1 | 974.46 | Local |
| M3 | 11/07/2011 0:00 | 0 | 49.618030 | -110.469130 | 538346 | 5496298 | 1 | 2156.64 | Local |
| M3 | 11/07/2011 0:00 | 3 | 49.634610 | -110.484630 | 537214 | 5498134 | 1 | 1584.33 | Local |
| M3 | 11/07/2011 0:00 | 6 | 49.623480 | -110.470930 | 538212 | 5496903 | 1 | 842.11 | Local |
| M3 | 11/07/2011 0:00 | 9 | 49.619490 | -110.461020 | 538931 | 5496465 | 1 | 3.65 | Local |
| M3 | 11/07/2011 0:00 | 12 | 49.619470 | -110.460980 | 538934 | 5496463 | 1 | 5.30 | Local |
| M3 | 11/07/2011 0:00 | 15 | 49.619510 | -110.460940 | 538937 | 5496467 | 1 | 7.96 | Local |
| M3 | 11/07/2011 0:00 | 18 | 49.619480 | -110.461040 | 538930 | 5496464 | 1 | 3077.21 | Local |
| M3 | 11/07/2011 0:00 | 21 | 49.605020 | -110.497360 | 536317 | 5494838 | 1 | 420.15 | Local |
| M3 | 11/08/2011 0:00 | 0 | 49.605940 | -110.503000 | 535909 | 5494937 | 1 | 1704.95 | Local |
| M3 | 11/08/2011 0:00 | 3 | 49.621170 | -110.505760 | 535698 | 5496629 | 1 | 640.16 | Local |
| M3 | 11/08/2011 0:00 | 6 | 49.622200 | -110.514480 | 535068 | 5496740 | 1 | 3296.99 | Local |
| M3 | 11/08/2011 0:00 | 9 | 49.629200 | -110.470120 | 538266 | 5497540 | 1 | 64.42 | Local |
| M3 | 11/08/2011 0:00 | 12 | 49.628630 | -110.470280 | 538255 | 5497476 | 1 | 13.48 | Local |
| M3 | 11/08/2011 0:00 | 15 | 49.628680 | -110.470450 | 538243 | 5497482 | 1 | 351.62 | Local |
| M3 | 11/08/2011 0:00 | 18 | 49.631140 | -110.467390 | 538462 | 5497757 | 1 | 3324.29 | Local |
| M3 | 11/08/2011 0:00 | 21 | 49.623570 | -110.511920 | 535252 | 5496893 | 1 | 2258.28 | Local |

| | | | | | | | | | |
|---|---|---|---|---|---|---|---|---|---|
| M3 | 11/09/2011 0:00 | 0 | 49.643870 | -110.510840 | 535315 | 5499151 | 1 | 2192.97 | Local |
| M3 | 11/09/2011 0:00 | 3 | 49.625850 | -110.498490 | 536220 | 5497153 | 1 | 1424.20 | Local |
| M3 | 11/09/2011 0:00 | 6 | 49.613040 | -110.498520 | 536227 | 5495729 | 1 | 2018.85 | Local |
| M3 | 11/09/2011 0:00 | 9 | 49.621730 | -110.473980 | 537993 | 5496707 | 1 | 220.31 | Local |
| M3 | 11/09/2011 0:00 | 12 | 49.621710 | -110.470930 | 538213 | 5496706 | 1 | 6.21 | Local |
| M3 | 11/09/2011 0:00 | 15 | 49.621670 | -110.470870 | 538218 | 5496702 | 1 | 3.78 | Local |
| M3 | 11/09/2011 0:00 | 18 | 49.621660 | -110.470920 | 538214 | 5496701 | 1 | 2943.82 | Local |
| M3 | 11/09/2011 0:00 | 21 | 49.607630 | -110.505480 | 535728 | 5495124 | 1 | 140.54 | Local |
| M3 | 11/10/2011 0:00 | 0 | 49.608630 | -110.504290 | 535814 | 5495236 | 1 | 1988.57 | Local |
| M3 | 11/10/2011 0:00 | 3 | 49.594110 | -110.520360 | 534663 | 5493614 | 1 | 50.64 | Local |
| M3 | 11/10/2011 0:00 | 6 | 49.594000 | -110.521040 | 534614 | 5493602 | 0 | 5223.12 | Local |
| M3 | 11/10/2011 0:00 | 12 | 49.620350 | -110.461190 | 538918 | 5496560 | 0 | 102.59 | Local |
| M3 | 11/10/2011 0:00 | 18 | 49.619430 | -110.461080 | 538927 | 5496458 | 1 | 3473.73 | Local |
| M3 | 11/10/2011 0:00 | 21 | 49.588620 | -110.469070 | 538374 | 5493029 | 1 | 494.47 | Local |
| M3 | 11/11/2011 0:00 | 0 | 49.585800 | -110.474360 | 537994 | 5492712 | 1 | 2358.22 | Local |
| M3 | 11/11/2011 0:00 | 3 | 49.567360 | -110.458240 | 539174 | 5490671 | 1 | 583.87 | Local |
| M3 | 11/11/2011 0:00 | 6 | 49.569060 | -110.465880 | 538620 | 5490856 | 1 | 2843.34 | Local |
| M3 | 11/11/2011 0:00 | 9 | 49.594320 | -110.459730 | 539044 | 5493667 | 1 | 16.04 | Local |
| M3 | 11/11/2011 0:00 | 12 | 49.594220 | -110.459570 | 539056 | 5493656 | 1 | 106.32 | Local |
| M3 | 11/11/2011 0:00 | 15 | 49.594470 | -110.458150 | 539158 | 5493685 | 1 | 7.96 | Local |
| M3 | 11/11/2011 0:00 | 18 | 49.594530 | -110.458210 | 539154 | 5493691 | 1 | 1880.71 | Local |
| M3 | 11/11/2011 0:00 | 21 | 49.579090 | -110.468840 | 538398 | 5491969 | 1 | 3029.89 | Local |
| M3 | 11/12/2011 0:00 | 0 | 49.557000 | -110.493380 | 536640 | 5489501 | 1 | 4393.88 | Local |
| M3 | 11/12/2011 0:00 | 3 | 49.523680 | -110.526040 | 534302 | 5485781 | 1 | 5534.15 | Local |
| M3 | 11/12/2011 0:00 | 6 | 49.571990 | -110.507600 | 535601 | 5491161 | 1 | 4221.04 | Local |
| M3 | 11/12/2011 0:00 | 9 | 49.593620 | -110.459610 | 539054 | 5493589 | 1 | 70.37 | Local |
| M3 | 11/12/2011 0:00 | 12 | 49.594230 | -110.459350 | 539072 | 5493657 | 1 | 3.64 | Local |
| M3 | 11/12/2011 0:00 | 15 | 49.594200 | -110.459370 | 539070 | 5493654 | 1 | 4.48 | Local |
| M3 | 11/12/2011 0:00 | 18 | 49.594190 | -110.459430 | 539066 | 5493653 | 1 | 4384.73 | Local |
| M3 | 11/12/2011 0:00 | 21 | 49.593150 | -110.520080 | 534684 | 5493507 | 1 | 726.32 | Local |
| M3 | 11/13/2011 0:00 | 0 | 49.590690 | -110.510770 | 535358 | 5493238 | 1 | 22.25 | Local |
| M3 | 11/13/2011 0:00 | 3 | 49.590490 | -110.510780 | 535358 | 5493216 | 1 | 512.00 | Local |
| M3 | 11/13/2011 0:00 | 6 | 49.593180 | -110.505030 | 535771 | 5493518 | 1 | 3645.53 | Local |
| M3 | 11/13/2011 0:00 | 9 | 49.616810 | -110.470050 | 538281 | 5496162 | 1 | 720.69 | Local |
| M3 | 11/13/2011 0:00 | 12 | 49.620140 | -110.461490 | 538897 | 5496537 | 1 | 75.52 | Local |
| M3 | 11/13/2011 0:00 | 15 | 49.619530 | -110.461030 | 538930 | 5496469 | 1 | 2.89 | Local |
| M3 | 11/13/2011 0:00 | 18 | 49.619530 | -110.460990 | 538933 | 5496469 | 1 | 1458.77 | Local |
| M3 | 11/13/2011 0:00 | 21 | 49.620310 | -110.481150 | 537476 | 5496546 | 1 | 3.98 | Local |
| M3 | 11/14/2011 0:00 | 0 | 49.620340 | -110.481120 | 537479 | 5496549 | 1 | 5.74 | Local |
| M3 | 11/14/2011 0:00 | 3 | 49.620390 | -110.481140 | 537477 | 5496555 | 1 | 1.82 | Local |
| M3 | 11/14/2011 0:00 | 6 | 49.620380 | -110.481160 | 537476 | 5496553 | 1 | 372.77 | Local |
| M3 | 11/14/2011 0:00 | 9 | 49.622100 | -110.476730 | 537794 | 5496747 | 1 | 23.54 | Local |
| M3 | 11/14/2011 0:00 | 12 | 49.621950 | -110.476500 | 537811 | 5496730 | 1 | 2.44 | Local |
| M3 | 11/14/2011 0:00 | 15 | 49.621940 | -110.476530 | 537809 | 5496729 | 1 | 377.52 | Local |
| M3 | 11/14/2011 0:00 | 18 | 49.620340 | -110.481140 | 537477 | 5496549 | 1 | 4.47 | Local |
| M3 | 11/14/2011 0:00 | 21 | 49.620330 | -110.481200 | 537473 | 5496548 | 1 | 3.63 | Local |
| M3 | 11/15/2011 0:00 | 0 | 49.620360 | -110.481220 | 537471 | 5496551 | 1 | 234.73 | Local |
| M3 | 11/15/2011 0:00 | 3 | 49.622450 | -110.481680 | 537436 | 5496783 | 1 | 237.69 | Local |
| M3 | 11/15/2011 0:00 | 6 | 49.620340 | -110.481150 | 537476 | 5496549 | 1 | 11.65 | Local |
| M3 | 11/15/2011 0:00 | 9 | 49.620410 | -110.481030 | 537485 | 5496557 | 1 | 6.87 | Local |
| M3 | 11/15/2011 0:00 | 12 | 49.620390 | -110.481120 | 537479 | 5496555 | 1 | 7.92 | Local |
| M3 | 11/15/2011 0:00 | 15 | 49.620320 | -110.481100 | 537480 | 5496547 | 1 | 70.98 | Local |
| M3 | 11/15/2011 0:00 | 18 | 49.620810 | -110.480470 | 537525 | 5496602 | 1 | 79.15 | Local |
| M3 | 11/15/2011 0:00 | 21 | 49.620310 | -110.481250 | 537469 | 5496546 | 1 | 9.29 | Local |
| M3 | 11/16/2011 0:00 | 0 | 49.620340 | -110.481130 | 537478 | 5496549 | 1 | 2.34 | Local |

| | | | | | | | | | |
|---|---|---|---|---|---|---|---|---|---|
| M3 | 11/16/2011 0:00 | 3 | 49.620360 | -110.481140 | 537477 | 5496551 | 1 | 2.17 | Local |
| M3 | 11/16/2011 0:00 | 6 | 49.620360 | -110.481110 | 537479 | 5496551 | 1 | 2.44 | Local |
| M3 | 11/16/2011 0:00 | 9 | 49.620350 | -110.481140 | 537477 | 5496550 | 1 | 1.11 | Local |
| M3 | 11/16/2011 0:00 | 12 | 49.620360 | -110.481140 | 537477 | 5496551 | 1 | 8.62 | Local |
| M3 | 11/16/2011 0:00 | 15 | 49.620330 | -110.481030 | 537485 | 5496548 | 1 | 6.60 | Local |
| M3 | 11/16/2011 0:00 | 18 | 49.620340 | -110.481120 | 537479 | 5496549 | 1 | 6.26 | Local |
| M3 | 11/16/2011 0:00 | 21 | 49.620390 | -110.481160 | 537476 | 5496555 | 1 | 2.44 | Local |
| M3 | 11/17/2011 0:00 | 0 | 49.620380 | -110.481130 | 537478 | 5496553 | 1 | 6.83 | Local |
| M3 | 11/17/2011 0:00 | 3 | 49.620320 | -110.481110 | 537479 | 5496547 | 1 | 8.92 | Local |
| M3 | 11/17/2011 0:00 | 6 | 49.620400 | -110.481100 | 537480 | 5496556 | 1 | 5.30 | Local |
| M3 | 11/17/2011 0:00 | 9 | 49.620360 | -110.481140 | 537477 | 5496551 | 1 | 2.22 | Local |
| M3 | 11/17/2011 0:00 | 12 | 49.620340 | -110.481140 | 537477 | 5496549 | 1 | 2.44 | Local |
| M3 | 11/17/2011 0:00 | 15 | 49.620350 | -110.481110 | 537479 | 5496550 | 1 | 11.77 | Local |
| M3 | 11/17/2011 0:00 | 18 | 49.620370 | -110.480950 | 537491 | 5496552 | 1 | 13.77 | Local |
| M3 | 11/17/2011 0:00 | 21 | 49.620360 | -110.481140 | 537477 | 5496551 | 1 | 3.63 | Local |
| M3 | 11/18/2011 0:00 | 0 | 49.620390 | -110.481120 | 537479 | 5496555 | 1 | 6.71 | Local |
| M3 | 11/18/2011 0:00 | 3 | 49.620330 | -110.481130 | 537478 | 5496548 | 1 | 11.65 | Local |
| M3 | 11/18/2011 0:00 | 6 | 49.620400 | -110.481010 | 537486 | 5496556 | 1 | 1373.99 | Local |
| M3 | 11/18/2011 0:00 | 9 | 49.620590 | -110.461990 | 538860 | 5496587 | 1 | 143.15 | Local |
| M3 | 11/18/2011 0:00 | 12 | 49.619430 | -110.461130 | 538923 | 5496458 | 1 | 13.19 | Local |
| M3 | 11/18/2011 0:00 | 15 | 49.619450 | -110.460950 | 538936 | 5496460 | 1 | 11.21 | Local |
| M3 | 11/18/2011 0:00 | 18 | 49.619540 | -110.461020 | 538931 | 5496470 | 1 | 1497.46 | Local |
| M3 | 11/18/2011 0:00 | 21 | 49.619680 | -110.481750 | 537434 | 5496475 | 1 | 11.12 | Local |
| M3 | 11/19/2011 0:00 | 0 | 49.619780 | -110.481750 | 537433 | 5496486 | 1 | 9.83 | Local |
| M3 | 11/19/2011 0:00 | 3 | 49.619720 | -110.481650 | 537441 | 5496480 | 1 | 5.78 | Local |
| M3 | 11/19/2011 0:00 | 6 | 49.619720 | -110.481730 | 537435 | 5496480 | 1 | 773.05 | Local |
| M3 | 11/19/2011 0:00 | 9 | 49.621490 | -110.471380 | 538181 | 5496682 | 1 | 789.47 | Local |
| M3 | 11/19/2011 0:00 | 12 | 49.619170 | -110.461050 | 538929 | 5496429 | 1 | 41.65 | Local |
| M3 | 11/19/2011 0:00 | 15 | 49.619540 | -110.460960 | 538935 | 5496470 | 1 | 4.68 | Local |
| M3 | 11/19/2011 0:00 | 18 | 49.619580 | -110.460980 | 538934 | 5496475 | 1 | 3195.94 | Local |
| M3 | 11/19/2011 0:00 | 21 | 49.607150 | -110.500870 | 536062 | 5495073 | 1 | 481.19 | Local |
| M3 | 11/20/2011 0:00 | 0 | 49.604590 | -110.506240 | 535676 | 5494786 | 1 | 24.77 | Local |
| M3 | 11/20/2011 0:00 | 3 | 49.604510 | -110.506560 | 535653 | 5494777 | 1 | 24.40 | Local |
| M3 | 11/20/2011 0:00 | 6 | 49.604580 | -110.506240 | 535676 | 5494785 | 1 | 3140.18 | Local |
| M3 | 11/20/2011 0:00 | 9 | 49.621770 | -110.471750 | 538154 | 5496713 | 1 | 177.80 | Local |
| M3 | 11/20/2011 0:00 | 12 | 49.622340 | -110.469450 | 538320 | 5496777 | 1 | 12.23 | Local |
| M3 | 11/20/2011 0:00 | 15 | 49.622450 | -110.469450 | 538320 | 5496789 | 1 | 1.82 | Local |
| M3 | 11/20/2011 0:00 | 18 | 49.622460 | -110.469470 | 538318 | 5496791 | 1 | 694.04 | Local |
| M3 | 11/20/2011 0:00 | 21 | 49.628130 | -110.465450 | 538604 | 5497423 | 1 | 1114.29 | Local |
| M3 | 11/21/2011 0:00 | 0 | 49.632960 | -110.478970 | 537624 | 5497953 | 1 | 24.22 | Local |
| M3 | 11/21/2011 0:00 | 3 | 49.632840 | -110.478690 | 537644 | 5497940 | 1 | 19.39 | Local |
| M3 | 11/21/2011 0:00 | 6 | 49.633010 | -110.478630 | 537649 | 5497959 | 1 | 5.74 | Local |
| M3 | 11/21/2011 0:00 | 9 | 49.633060 | -110.478610 | 537650 | 5497964 | 0 | 1244.25 | Local |
| M3 | 11/21/2011 0:00 | 18 | 49.635460 | -110.495440 | 536433 | 5498223 | 1 | 2065.96 | Local |
| M3 | 11/21/2011 0:00 | 21 | 49.625780 | -110.519860 | 534677 | 5497135 | 1 | 1365.37 | Local |
| M3 | 11/22/2011 0:00 | 0 | 49.620800 | -110.502580 | 535928 | 5496590 | 1 | 18.56 | Local |
| M3 | 11/22/2011 0:00 | 3 | 49.620940 | -110.502440 | 535938 | 5496605 | 1 | 21.24 | Local |
| M3 | 11/22/2011 0:00 | 6 | 49.620800 | -110.502640 | 535924 | 5496590 | 1 | 457.99 | Local |
| M3 | 11/22/2011 0:00 | 9 | 49.624150 | -110.498950 | 536188 | 5496964 | 1 | 1.82 | Local |
| M3 | 11/22/2011 0:00 | 12 | 49.624160 | -110.498930 | 536189 | 5496965 | 1 | 3.98 | Local |
| M3 | 11/22/2011 0:00 | 15 | 49.624190 | -110.498900 | 536191 | 5496968 | 1 | 5.56 | Local |
| M3 | 11/22/2011 0:00 | 18 | 49.624140 | -110.498900 | 536191 | 5496963 | 1 | 454.86 | Local |
| M3 | 11/22/2011 0:00 | 21 | 49.620980 | -110.502900 | 535905 | 5496610 | 1 | 27.16 | Local |
| M3 | 11/23/2011 0:00 | 0 | 49.620810 | -110.502630 | 535925 | 5496591 | 1 | 203.52 | Local |
| M3 | 11/23/2011 0:00 | 3 | 49.619800 | -110.500280 | 536095 | 5496480 | 1 | 2.34 | Local |

| | | | | | | | | | |
|---|---|---|---|---|---|---|---|---|---|
| M3 | 11/23/2011 0:00 | 6 | 49.619780 | -110.500270 | 536096 | 5496477 | 0 | 2347.49 | Local |
| M3 | 11/23/2011 0:00 | 15 | 49.628530 | -110.470690 | 538225 | 5497465 | 1 | 143.43 | Local |
| M3 | 11/23/2011 0:00 | 18 | 49.629820 | -110.470710 | 538223 | 5497608 | 1 | 1641.91 | Local |
| M3 | 11/23/2011 0:00 | 21 | 49.638240 | -110.489390 | 536868 | 5498535 | 1 | 890.02 | Local |
| M3 | 11/24/2011 0:00 | 0 | 49.639240 | -110.501620 | 535984 | 5498640 | 1 | 1101.09 | Local |
| M3 | 11/24/2011 0:00 | 3 | 49.631250 | -110.510630 | 535339 | 5497748 | 1 | 873.73 | Local |
| M3 | 11/24/2011 0:00 | 6 | 49.626510 | -110.500980 | 536040 | 5497225 | 0 | 2216.63 | Local |
| M3 | 11/24/2011 0:00 | 18 | 49.629690 | -110.470680 | 538225 | 5497594 | 1 | 2368.18 | Local |
| M3 | 11/24/2011 0:00 | 21 | 49.611410 | -110.487510 | 537024 | 5495553 | 1 | 2242.52 | Local |
| M3 | 11/25/2011 0:00 | 0 | 49.591960 | -110.495730 | 536444 | 5493387 | 1 | 2922.57 | Local |
| M3 | 11/25/2011 0:00 | 3 | 49.566730 | -110.484380 | 537284 | 5490587 | 1 | 1581.99 | Local |
| M3 | 11/25/2011 0:00 | 6 | 49.554880 | -110.472270 | 538169 | 5489276 | 1 | 4313.13 | Local |
| M3 | 11/25/2011 0:00 | 9 | 49.583730 | -110.432390 | 541029 | 5492504 | 1 | 17.48 | Local |
| M3 | 11/25/2011 0:00 | 12 | 49.583870 | -110.432500 | 541021 | 5492520 | 1 | 10.91 | Local |
| M3 | 11/25/2011 0:00 | 15 | 49.583780 | -110.432440 | 541025 | 5492510 | 1 | 21.29 | Local |
| M3 | 11/25/2011 0:00 | 18 | 49.583870 | -110.432180 | 541044 | 5492520 | 1 | 1740.25 | Local |
| M3 | 11/25/2011 0:00 | 21 | 49.575120 | -110.412220 | 542494 | 5491558 | 1 | 3585.14 | Local |
| M3 | 11/26/2011 0:00 | 0 | 49.543360 | -110.403640 | 543143 | 5488032 | 1 | 496.49 | Local |
| M3 | 11/26/2011 0:00 | 3 | 49.546830 | -110.399320 | 543452 | 5488420 | 1 | 522.02 | Local |
| M3 | 11/26/2011 0:00 | 6 | 49.551390 | -110.397600 | 543573 | 5488928 | 1 | 603.95 | Local |
| M3 | 11/26/2011 0:00 | 9 | 49.556380 | -110.394300 | 543807 | 5489485 | 1 | 4.68 | Local |
| M3 | 11/26/2011 0:00 | 12 | 49.556420 | -110.394280 | 543808 | 5489490 | 1 | 8.08 | Local |
| M3 | 11/26/2011 0:00 | 15 | 49.556350 | -110.394250 | 543810 | 5489482 | 1 | 4.68 | Local |
| M3 | 11/26/2011 0:00 | 18 | 49.556390 | -110.394270 | 543809 | 5489486 | 1 | 985.60 | Local |
| M3 | 11/26/2011 0:00 | 21 | 49.551350 | -110.383060 | 544624 | 5488933 | 1 | 1730.05 | Local |
| M3 | 11/27/2011 0:00 | 0 | 49.541060 | -110.365120 | 545932 | 5487799 | 1 | 2358.28 | Local |
| M3 | 11/27/2011 0:00 | 3 | 49.520880 | -110.375160 | 545224 | 5485550 | 1 | 487.17 | Local |
| M3 | 11/27/2011 0:00 | 6 | 49.519190 | -110.381370 | 544776 | 5485358 | 1 | 3078.31 | Local |
| M3 | 11/27/2011 0:00 | 9 | 49.546110 | -110.371420 | 545471 | 5488357 | 1 | 1097.27 | Local |
| M3 | 11/27/2011 0:00 | 12 | 49.552940 | -110.382370 | 544673 | 5489110 | 1 | 1.82 | Local |
| M3 | 11/27/2011 0:00 | 15 | 49.552930 | -110.382350 | 544674 | 5489109 | 1 | 5.56 | Local |
| M3 | 11/27/2011 0:00 | 18 | 49.552880 | -110.382350 | 544674 | 5489103 | 1 | 320.86 | Local |
| M3 | 11/27/2011 0:00 | 21 | 49.553030 | -110.386780 | 544354 | 5489117 | 1 | 522.71 | Local |
| M3 | 11/28/2011 0:00 | 0 | 49.557580 | -110.388600 | 544218 | 5489622 | 1 | 2726.53 | Local |
| M3 | 11/28/2011 0:00 | 3 | 49.533060 | -110.387930 | 544289 | 5486896 | 1 | 1585.41 | Local |
| M3 | 11/28/2011 0:00 | 6 | 49.519420 | -110.381540 | 544763 | 5485384 | 0 | 341.05 | Local |
| M3 | 11/28/2011 0:00 | 12 | 49.521170 | -110.385410 | 544482 | 5485576 | 0 | 8.92 | Local |
| M3 | 11/28/2011 0:00 | 18 | 49.521250 | -110.385420 | 544481 | 5485585 | 1 | 638.51 | Local |
| M3 | 11/28/2011 0:00 | 21 | 49.520090 | -110.376780 | 545107 | 5485461 | 1 | 1282.97 | Local |
| M3 | 11/29/2011 0:00 | 0 | 49.513810 | -110.391650 | 544037 | 5484754 | 1 | 1935.53 | Local |
| M3 | 11/29/2011 0:00 | 3 | 49.498240 | -110.403610 | 543185 | 5483016 | 1 | 2099.25 | Local |
| M3 | 11/29/2011 0:00 | 6 | 49.514580 | -110.389080 | 544222 | 5484841 | 1 | 80.83 | Local |
| M3 | 11/29/2011 0:00 | 9 | 49.513870 | -110.389320 | 544205 | 5484762 | 1 | 6.67 | Local |
| M3 | 11/29/2011 0:00 | 12 | 49.513930 | -110.389320 | 544205 | 5484769 | 0 | 37.83 | Local |
| M3 | 11/29/2011 0:00 | 18 | 49.514270 | -110.389300 | 544206 | 5484806 | 1 | 1313.05 | Local |
| M3 | 11/29/2011 0:00 | 21 | 49.521410 | -110.403750 | 543154 | 5485592 | 1 | 1935.54 | Local |
| M3 | 11/30/2011 0:00 | 0 | 49.519820 | -110.430380 | 541228 | 5485400 | 1 | 1123.49 | Local |
| M3 | 11/30/2011 0:00 | 3 | 49.513910 | -110.442970 | 540322 | 5484736 | 1 | 847.96 | Local |
| M3 | 11/30/2011 0:00 | 6 | 49.515580 | -110.431540 | 541148 | 5484928 | 0 | 3067.55 | Local |
| M3 | 11/30/2011 0:00 | 12 | 49.513920 | -110.389240 | 544211 | 5484768 | 1 | 5.74 | Local |
| M3 | 11/30/2011 0:00 | 15 | 49.513870 | -110.389220 | 544212 | 5484762 | 1 | 658.63 | Local |
| M3 | 11/30/2011 0:00 | 18 | 49.519030 | -110.393690 | 543884 | 5485333 | 1 | 669.02 | Local |
| M3 | 11/30/2011 0:00 | 21 | 49.524200 | -110.398420 | 543537 | 5485905 | 1 | 2152.41 | Local |
| M3 | 12/01/2011 0:00 | 0 | 49.519770 | -110.427370 | 541446 | 5485396 | 1 | 1109.75 | Local |
| M3 | 12/01/2011 0:00 | 3 | 49.516120 | -110.441640 | 540416 | 5484983 | 1 | 3.10 | Local |

| | | | | | | | | | |
|---|---|---|---|---|---|---|---|---|---|
| M3 | 12/01/2011 0:00 | 6 | 49.516130 | -110.441680 | 540413 | 5484984 | 1 | 3517.97 | Local |
| M3 | 12/01/2011 0:00 | 9 | 49.510600 | -110.393830 | 543882 | 5484396 | 1 | 491.21 | Local |
| M3 | 12/01/2011 0:00 | 12 | 49.513860 | -110.389250 | 544210 | 5484761 | 0 | 1613.91 | Local |
| M3 | 12/01/2011 0:00 | 18 | 49.527750 | -110.382770 | 544667 | 5486309 | 1 | 2793.99 | Local |
| M3 | 12/01/2011 0:00 | 21 | 49.552760 | -110.386550 | 544371 | 5489087 | 1 | 35.92 | Local |
| M3 | 12/02/2011 0:00 | 0 | 49.552560 | -110.386160 | 544399 | 5489065 | 1 | 2385.02 | Local |
| M3 | 12/02/2011 0:00 | 3 | 49.573130 | -110.395520 | 543704 | 5491347 | 0 | 36.58 | Local |
| M3 | 12/02/2011 0:00 | 9 | 49.572870 | -110.395210 | 543726 | 5491318 | 1 | 22.42 | Local |
| M3 | 12/02/2011 0:00 | 12 | 49.573070 | -110.395250 | 543723 | 5491340 | 1 | 2.89 | Local |
| M3 | 12/02/2011 0:00 | 15 | 49.573070 | -110.395210 | 543726 | 5491340 | 1 | 386.04 | Local |
| M3 | 12/02/2011 0:00 | 18 | 49.575370 | -110.399210 | 543435 | 5491593 | 0 | 3375.21 | Local |
| M3 | 12/03/2011 0:00 | 3 | 49.596350 | -110.432960 | 540977 | 5493907 | 0 | 625.61 | Local |
| M3 | 12/03/2011 0:00 | 9 | 49.590780 | -110.431730 | 541071 | 5493288 | 1 | 258.24 | Local |
| M3 | 12/03/2011 0:00 | 12 | 49.589760 | -110.428520 | 541304 | 5493177 | 1 | 20.48 | Local |
| M3 | 12/03/2011 0:00 | 15 | 49.589940 | -110.428460 | 541308 | 5493197 | 1 | 184.87 | Local |
| M3 | 12/03/2011 0:00 | 18 | 49.590960 | -110.430480 | 541161 | 5493309 | 1 | 3430.38 | Local |
| M3 | 12/03/2011 0:00 | 21 | 49.617170 | -110.455530 | 539329 | 5496210 | 1 | 482.84 | Local |
| M3 | 12/04/2011 0:00 | 0 | 49.619600 | -110.461070 | 538927 | 5496477 | 1 | 1958.79 | Local |
| M3 | 12/04/2011 0:00 | 3 | 49.633060 | -110.478570 | 537653 | 5497964 | 1 | 4.33 | Local |
| M3 | 12/04/2011 0:00 | 6 | 49.633060 | -110.478510 | 537657 | 5497965 | 1 | 6.50 | Local |
| M3 | 12/04/2011 0:00 | 9 | 49.633060 | -110.478600 | 537651 | 5497964 | 1 | 6.06 | Local |
| M3 | 12/04/2011 0:00 | 12 | 49.633030 | -110.478530 | 537656 | 5497961 | 0 | 3.61 | Local |
| M3 | 12/04/2011 0:00 | 18 | 49.633030 | -110.478580 | 537652 | 5497961 | 1 | 1813.45 | Local |
| M3 | 12/04/2011 0:00 | 21 | 49.638060 | -110.502470 | 535923 | 5498509 | 1 | 2034.44 | Local |
| M3 | 12/05/2011 0:00 | 0 | 49.620060 | -110.507540 | 535570 | 5496505 | 1 | 1113.51 | Local |
| M3 | 12/05/2011 0:00 | 3 | 49.625180 | -110.494290 | 536524 | 5497081 | 1 | 1832.10 | Local |
| M3 | 12/05/2011 0:00 | 6 | 49.622390 | -110.469290 | 538331 | 5496783 | 1 | 81.46 | Local |
| M3 | 12/05/2011 0:00 | 9 | 49.623070 | -110.468870 | 538361 | 5496859 | 1 | 5.74 | Local |
| M3 | 12/05/2011 0:00 | 12 | 49.623120 | -110.468850 | 538363 | 5496864 | 1 | 11.55 | Local |
| M3 | 12/05/2011 0:00 | 15 | 49.623030 | -110.468770 | 538368 | 5496854 | 1 | 6.67 | Local |
| M3 | 12/05/2011 0:00 | 18 | 49.623060 | -110.468850 | 538363 | 5496858 | 1 | 2020.97 | Local |
| M3 | 12/05/2011 0:00 | 21 | 49.610270 | -110.488730 | 536936 | 5495426 | 1 | 1.11 | Local |
| M3 | 12/06/2011 0:00 | 0 | 49.610260 | -110.488730 | 536937 | 5495425 | 1 | 7.95 | Local |
| M3 | 12/06/2011 0:00 | 3 | 49.610260 | -110.488620 | 536944 | 5495425 | 1 | 8.02 | Local |
| M3 | 12/06/2011 0:00 | 6 | 49.610250 | -110.488730 | 536937 | 5495424 | 1 | 2385.93 | Local |
| M3 | 12/06/2011 0:00 | 9 | 49.624350 | -110.463830 | 538724 | 5497004 | 1 | 376.28 | Local |
| M3 | 12/06/2011 0:00 | 12 | 49.626990 | -110.460570 | 538958 | 5497299 | 1 | 63.02 | Local |
| M3 | 12/06/2011 0:00 | 15 | 49.626550 | -110.461120 | 538918 | 5497250 | 1 | 5.18 | Local |
| M3 | 12/06/2011 0:00 | 18 | 49.626560 | -110.461190 | 538913 | 5497251 | 1 | 2688.02 | Local |
| M3 | 12/06/2011 0:00 | 21 | 49.610300 | -110.488730 | 536936 | 5495429 | 1 | 2014.20 | Local |
| M3 | 12/07/2011 0:00 | 0 | 49.618680 | -110.464010 | 538716 | 5496373 | 1 | 5.47 | Local |
| M3 | 12/07/2011 0:00 | 3 | 49.618650 | -110.463950 | 538720 | 5496370 | 1 | 3.10 | Local |
| M3 | 12/07/2011 0:00 | 6 | 49.618660 | -110.463990 | 538717 | 5496371 | 1 | 1.33 | Local |
| M3 | 12/07/2011 0:00 | 9 | 49.618670 | -110.464000 | 538716 | 5496372 | 1 | 1.33 | Local |
| M3 | 12/07/2011 0:00 | 12 | 49.618660 | -110.463990 | 538717 | 5496371 | 1 | 2.34 | Local |
| M3 | 12/07/2011 0:00 | 15 | 49.618640 | -110.463980 | 538718 | 5496369 | 1 | 817.77 | Local |
| M3 | 12/07/2011 0:00 | 18 | 49.624980 | -110.458240 | 539127 | 5497077 | 1 | 1724.30 | Local |
| M3 | 12/07/2011 0:00 | 21 | 49.633060 | -110.478620 | 537649 | 5497964 | 1 | 4.92 | Local |
| M3 | 12/08/2011 0:00 | 0 | 49.633030 | -110.478570 | 537653 | 5497961 | 1 | 0.72 | Local |
| M3 | 12/08/2011 0:00 | 3 | 49.633030 | -110.478560 | 537654 | 5497961 | 1 | 10.24 | Local |
| M3 | 12/08/2011 0:00 | 6 | 49.633120 | -110.478530 | 537656 | 5497971 | 1 | 14.03 | Local |
| M3 | 12/08/2011 0:00 | 9 | 49.633000 | -110.478590 | 537652 | 5497958 | 1 | 4.68 | Local |
| M3 | 12/08/2011 0:00 | 12 | 49.633040 | -110.478570 | 537653 | 5497962 | 1 | 3.10 | Local |
| M3 | 12/08/2011 0:00 | 15 | 49.633030 | -110.478530 | 537656 | 5497961 | 1 | 3.61 | Local |
| M3 | 12/08/2011 0:00 | 18 | 49.633030 | -110.478580 | 537652 | 5497961 | 1 | 2376.23 | Local |

| | | | | | | | | | |
|---|---|---|---|---|---|---|---|---|---|
| M3 | 12/08/2011 0:00 | 21 | 49.624900 | -110.509010 | 535461 | 5497042 | 1 | 1975.71 | Local |
| M3 | 12/09/2011 0:00 | 0 | 49.618470 | -110.534510 | 533624 | 5496316 | 1 | 2511.45 | Local |
| M3 | 12/09/2011 0:00 | 3 | 49.617740 | -110.499760 | 536134 | 5496251 | 1 | 1244.13 | Local |
| M3 | 12/09/2011 0:00 | 6 | 49.620580 | -110.483100 | 537335 | 5496575 | 1 | 1289.85 | Local |
| M3 | 12/09/2011 0:00 | 9 | 49.629760 | -110.472180 | 538117 | 5497601 | 1 | 7.92 | Local |
| M3 | 12/09/2011 0:00 | 12 | 49.629830 | -110.472160 | 538118 | 5497609 | 1 | 3.98 | Local |
| M3 | 12/09/2011 0:00 | 15 | 49.629800 | -110.472190 | 538116 | 5497605 | 1 | 310.31 | Local |
| M3 | 12/09/2011 0:00 | 18 | 49.631410 | -110.475700 | 537861 | 5497782 | 1 | 4921.35 | Local |
| M3 | 12/09/2011 0:00 | 21 | 49.658400 | -110.529730 | 533941 | 5500757 | 1 | 1243.10 | Local |
| M3 | 12/10/2011 0:00 | 0 | 49.654150 | -110.545660 | 532794 | 5500278 | 1 | 1217.85 | Local |
| M3 | 12/10/2011 0:00 | 3 | 49.665090 | -110.546510 | 532725 | 5501494 | 1 | 5789.27 | Local |
| M3 | 12/10/2011 0:00 | 6 | 49.632470 | -110.484000 | 537261 | 5497896 | 1 | 74.22 | Local |
| M3 | 12/10/2011 0:00 | 9 | 49.632200 | -110.483060 | 537329 | 5497867 | 1 | 339.68 | Local |
| M3 | 12/10/2011 0:00 | 12 | 49.632950 | -110.478500 | 537658 | 5497952 | 1 | 4.51 | Local |
| M3 | 12/10/2011 0:00 | 15 | 49.632990 | -110.478510 | 537657 | 5497957 | 1 | 4.41 | Local |
| M3 | 12/10/2011 0:00 | 18 | 49.633020 | -110.478470 | 537660 | 5497960 | 0 | 6.59 | Local |
| M3 | 12/11/2011 0:00 | 3 | 49.633030 | -110.478560 | 537654 | 5497961 | 1 | 7.01 | Local |
| M3 | 12/11/2011 0:00 | 6 | 49.633090 | -110.478590 | 537651 | 5497968 | 1 | 3.65 | Local |
| M3 | 12/11/2011 0:00 | 9 | 49.633070 | -110.478550 | 537654 | 5497966 | 1 | 3.61 | Local |
| M3 | 12/11/2011 0:00 | 12 | 49.633070 | -110.478600 | 537651 | 5497966 | 0 | 366.13 | Local |
| M3 | 12/11/2011 0:00 | 18 | 49.632200 | -110.483490 | 537298 | 5497866 | 1 | 2998.81 | Local |
| M3 | 12/11/2011 0:00 | 21 | 49.608730 | -110.503950 | 535838 | 5495247 | 1 | 1014.79 | Local |
| M3 | 12/12/2011 0:00 | 0 | 49.600680 | -110.510570 | 535366 | 5494349 | 1 | 879.55 | Local |
| M3 | 12/12/2011 0:00 | 3 | 49.595390 | -110.501520 | 536023 | 5493765 | 0 | 3959.44 | Local |
| M3 | 12/12/2011 0:00 | 9 | 49.619460 | -110.461130 | 538923 | 5496461 | 1 | 9.70 | Local |
| M3 | 12/12/2011 0:00 | 12 | 49.619510 | -110.461020 | 538931 | 5496467 | 1 | 12.97 | Local |
| M3 | 12/12/2011 0:00 | 15 | 49.619620 | -110.461080 | 538927 | 5496479 | 1 | 3.98 | Local |
| M3 | 12/12/2011 0:00 | 18 | 49.619590 | -110.461110 | 538924 | 5496476 | 1 | 3593.25 | Local |
| M3 | 12/12/2011 0:00 | 21 | 49.641090 | -110.498260 | 536225 | 5498848 | 1 | 430.35 | Local |
| M3 | 12/13/2011 0:00 | 0 | 49.637380 | -110.499960 | 536105 | 5498434 | 1 | 10.41 | Local |
| M3 | 12/13/2011 0:00 | 3 | 49.637470 | -110.499920 | 536108 | 5498444 | 1 | 194.73 | Local |
| M3 | 12/13/2011 0:00 | 6 | 49.636240 | -110.498000 | 536248 | 5498308 | 1 | 1445.77 | Local |
| M3 | 12/13/2011 0:00 | 9 | 49.633050 | -110.478590 | 537651 | 5497963 | 1 | 1.33 | Local |
| M3 | 12/13/2011 0:00 | 12 | 49.633060 | -110.478600 | 537651 | 5497964 | 1 | 11.71 | Local |
| M3 | 12/13/2011 0:00 | 15 | 49.633020 | -110.478450 | 537662 | 5497960 | 1 | 10.83 | Local |
| M3 | 12/13/2011 0:00 | 18 | 49.633020 | -110.478600 | 537651 | 5497960 | 1 | 1065.30 | Local |
| M3 | 12/13/2011 0:00 | 21 | 49.634820 | -110.493090 | 536603 | 5498153 | 1 | 572.10 | Local |
| M3 | 12/14/2011 0:00 | 0 | 49.637450 | -110.499900 | 536109 | 5498442 | 1 | 1.44 | Local |
| M3 | 12/14/2011 0:00 | 3 | 49.637450 | -110.499920 | 536108 | 5498442 | 1 | 0.00 | Local |
| M3 | 12/14/2011 0:00 | 6 | 49.637450 | -110.499920 | 536108 | 5498442 | 1 | 1615.69 | Local |
| M3 | 12/14/2011 0:00 | 9 | 49.633040 | -110.478600 | 537651 | 5497962 | 1 | 3.78 | Local |
| M3 | 12/14/2011 0:00 | 12 | 49.633030 | -110.478550 | 537654 | 5497961 | 1 | 1.33 | Local |
| M3 | 12/14/2011 0:00 | 15 | 49.633040 | -110.478560 | 537654 | 5497962 | 1 | 2.22 | Local |
| M3 | 12/14/2011 0:00 | 18 | 49.633020 | -110.478560 | 537654 | 5497960 | 1 | 3.65 | Local |
| M3 | 12/14/2011 0:00 | 21 | 49.633040 | -110.478600 | 537651 | 5497962 | 1 | 3.65 | Local |
| M3 | 12/15/2011 0:00 | 0 | 49.633020 | -110.478640 | 537648 | 5497960 | 1 | 11.12 | Local |
| M3 | 12/15/2011 0:00 | 3 | 49.633090 | -110.478750 | 537640 | 5497968 | 1 | 11.54 | Local |
| M3 | 12/15/2011 0:00 | 6 | 49.633040 | -110.478610 | 537650 | 5497962 | 1 | 3.34 | Local |
| M3 | 12/15/2011 0:00 | 9 | 49.633070 | -110.478610 | 537650 | 5497966 | 1 | 1.33 | Local |
| M3 | 12/15/2011 0:00 | 12 | 49.633060 | -110.478600 | 537651 | 5497964 | 1 | 16.50 | Local |
| M3 | 12/15/2011 0:00 | 15 | 49.633020 | -110.478820 | 537635 | 5497960 | 1 | 406.53 | Local |
| M3 | 12/15/2011 0:00 | 18 | 49.632240 | -110.484320 | 537238 | 5497870 | 1 | 2660.30 | Local |
| M3 | 12/15/2011 0:00 | 21 | 49.626710 | -110.520160 | 534654 | 5497239 | 1 | 3682.94 | Local |
| M3 | 12/16/2011 0:00 | 0 | 49.655530 | -110.545310 | 532818 | 5500431 | 1 | 19.57 | Local |
| M3 | 12/16/2011 0:00 | 3 | 49.655580 | -110.545570 | 532800 | 5500437 | 1 | 4333.86 | Local |

| | | | | | | | | | |
|---|---|---|---|---|---|---|---|---|---|
| M3 | 12/16/2011 0:00 | 6 | 49.638180 | -110.491850 | 536690 | 5498527 | 0 | 1161.74 | Local |
| M3 | 12/16/2011 0:00 | 12 | 49.632830 | -110.478030 | 537692 | 5497939 | 1 | 42.55 | Local |
| M3 | 12/16/2011 0:00 | 15 | 49.633010 | -110.478550 | 537654 | 5497959 | 1 | 5.61 | Local |
| M3 | 12/16/2011 0:00 | 18 | 49.633060 | -110.478560 | 537654 | 5497964 | 1 | 16.50 | Local |
| M3 | 12/16/2011 0:00 | 21 | 49.633020 | -110.478340 | 537670 | 5497960 | 1 | 1834.25 | Local |
| M3 | 12/17/2011 0:00 | 0 | 49.644530 | -110.460140 | 538975 | 5499249 | 1 | 2033.82 | Local |
| M3 | 12/17/2011 0:00 | 3 | 49.654650 | -110.483610 | 537273 | 5500362 | 1 | 1254.56 | Local |
| M3 | 12/17/2011 0:00 | 6 | 49.644550 | -110.475860 | 537840 | 5499243 | 1 | 1292.68 | Local |
| M3 | 12/17/2011 0:00 | 9 | 49.633060 | -110.478600 | 537651 | 5497964 | 1 | 3.41 | Local |
| M3 | 12/17/2011 0:00 | 12 | 49.633030 | -110.478590 | 537652 | 5497961 | 1 | 4.33 | Local |
| M3 | 12/17/2011 0:00 | 15 | 49.633030 | -110.478530 | 537656 | 5497961 | 1 | 61.29 | Local |
| M3 | 12/17/2011 0:00 | 18 | 49.632760 | -110.479270 | 537603 | 5497931 | 1 | 3279.23 | Local |
| M3 | 12/17/2011 0:00 | 21 | 49.653820 | -110.511070 | 535291 | 5500257 | 1 | 1414.74 | Local |
| M3 | 12/18/2011 0:00 | 0 | 49.657930 | -110.492520 | 536627 | 5500723 | 1 | 23.84 | Local |
| M3 | 12/18/2011 0:00 | 3 | 49.657940 | -110.492190 | 536651 | 5500724 | 1 | 19.77 | Local |
| M3 | 12/18/2011 0:00 | 6 | 49.657910 | -110.492460 | 536631 | 5500720 | 1 | 3385.81 | Local |
| M3 | 12/18/2011 0:00 | 9 | 49.630420 | -110.472280 | 538109 | 5497674 | 1 | 82.50 | Local |
| M3 | 12/18/2011 0:00 | 12 | 49.630160 | -110.471210 | 538187 | 5497646 | 1 | 4.24 | Local |
| M3 | 12/18/2011 0:00 | 15 | 49.630140 | -110.471260 | 538183 | 5497644 | 1 | 3.65 | Local |
| M3 | 12/18/2011 0:00 | 18 | 49.630160 | -110.471300 | 538180 | 5497646 | 1 | 2.34 | Local |
| M3 | 12/18/2011 0:00 | 21 | 49.630140 | -110.471290 | 538181 | 5497644 | 1 | 4.45 | Local |
| M3 | 12/19/2011 0:00 | 0 | 49.630180 | -110.471290 | 538181 | 5497648 | 1 | 5.30 | Local |
| M3 | 12/19/2011 0:00 | 3 | 49.630140 | -110.471250 | 538184 | 5497644 | 1 | 621.43 | Local |
| M3 | 12/19/2011 0:00 | 6 | 49.633110 | -110.478540 | 537655 | 5497970 | 0 | 12.25 | Local |
| M3 | 12/19/2011 0:00 | 12 | 49.633000 | -110.478550 | 537654 | 5497958 | 1 | 11.71 | Local |
| M3 | 12/19/2011 0:00 | 15 | 49.633040 | -110.478700 | 537644 | 5497962 | 1 | 371.76 | Local |
| M3 | 12/19/2011 0:00 | 18 | 49.631720 | -110.483430 | 537303 | 5497813 | 1 | 2953.17 | Local |
| M3 | 12/19/2011 0:00 | 21 | 49.608810 | -110.504120 | 535826 | 5495256 | 1 | 2080.26 | Local |
| M3 | 12/20/2011 0:00 | 0 | 49.594490 | -110.522650 | 534497 | 5493655 | 1 | 1929.20 | Local |
| M3 | 12/20/2011 0:00 | 3 | 49.585330 | -110.499980 | 536142 | 5492648 | 1 | 3258.66 | Local |
| M3 | 12/20/2011 0:00 | 6 | 49.579700 | -110.455740 | 539344 | 5492044 | 0 | 60.59 | Local |
| M3 | 12/20/2011 0:00 | 12 | 49.579840 | -110.456550 | 539286 | 5492059 | 1 | 45.06 | Local |
| M3 | 12/20/2011 0:00 | 15 | 49.579440 | -110.456650 | 539279 | 5492014 | 1 | 130.01 | Local |
| M3 | 12/20/2011 0:00 | 18 | 49.579840 | -110.454960 | 539401 | 5492060 | 1 | 133.72 | Local |
| M3 | 12/20/2011 0:00 | 21 | 49.579470 | -110.456720 | 539274 | 5492018 | 1 | 5.18 | Local |
| M3 | 12/21/2011 0:00 | 0 | 49.579460 | -110.456650 | 539279 | 5492017 | 1 | 1078.91 | Local |
| M3 | 12/21/2011 0:00 | 3 | 49.580120 | -110.441760 | 540355 | 5492098 | 1 | 1780.67 | Local |
| M3 | 12/21/2011 0:00 | 6 | 49.593610 | -110.428480 | 541303 | 5493605 | 1 | 313.65 | Local |
| M3 | 12/21/2011 0:00 | 9 | 49.590970 | -110.430010 | 541195 | 5493311 | 1 | 199.41 | Local |
| M3 | 12/21/2011 0:00 | 12 | 49.591980 | -110.427730 | 541359 | 5493424 | 1 | 7.27 | Local |
| M3 | 12/21/2011 0:00 | 15 | 49.591920 | -110.427690 | 541362 | 5493417 | 1 | 77.65 | Local |
| M3 | 12/21/2011 0:00 | 18 | 49.592560 | -110.428120 | 541330 | 5493488 | 0 | 117.03 | Local |
| M3 | 12/22/2011 0:00 | 0 | 49.593580 | -110.428520 | 541300 | 5493602 | 1 | 4.51 | Local |
| M3 | 12/22/2011 0:00 | 3 | 49.593620 | -110.428510 | 541301 | 5493606 | 1 | 3.41 | Local |
| M3 | 12/22/2011 0:00 | 6 | 49.593590 | -110.428520 | 541300 | 5493603 | 1 | 82.69 | Local |
| M3 | 12/22/2011 0:00 | 9 | 49.593900 | -110.427480 | 541375 | 5493638 | 0 | 83.07 | Local |
| M3 | 12/22/2011 0:00 | 15 | 49.593470 | -110.428420 | 541308 | 5493589 | 1 | 22.53 | Local |
| M3 | 12/22/2011 0:00 | 18 | 49.593670 | -110.428470 | 541304 | 5493612 | 1 | 401.64 | Local |
| M3 | 12/22/2011 0:00 | 21 | 49.595790 | -110.432970 | 540977 | 5493845 | 0 | 785.09 | Local |
| M3 | 12/23/2011 0:00 | 9 | 49.590770 | -110.425330 | 541533 | 5493291 | 1 | 110.00 | Local |
| M3 | 12/23/2011 0:00 | 12 | 49.590060 | -110.424270 | 541611 | 5493213 | 1 | 10.64 | Local |
| M3 | 12/23/2011 0:00 | 15 | 49.590150 | -110.424220 | 541614 | 5493223 | 1 | 527.47 | Local |
| M3 | 12/23/2011 0:00 | 18 | 49.594720 | -110.422260 | 541752 | 5493732 | 1 | 1492.05 | Local |
| M3 | 12/23/2011 0:00 | 21 | 49.583650 | -110.410590 | 542605 | 5492508 | 1 | 589.50 | Local |
| M3 | 12/24/2011 0:00 | 0 | 49.587680 | -110.405290 | 542984 | 5492959 | 1 | 2120.92 | Local |

| | | | | | | | | | |
|---|---|---|---|---|---|---|---|---|---|
| M3 | 12/24/2011 0:00 | 3 | 49.594250 | -110.432840 | 540988 | 5493674 | 1 | 8.74 | Local |
| M3 | 12/24/2011 0:00 | 6 | 49.594240 | -110.432960 | 540979 | 5493672 | 1 | 147.91 | Local |
| M3 | 12/24/2011 0:00 | 9 | 49.592910 | -110.432910 | 540984 | 5493525 | 1 | 410.40 | Local |
| M3 | 12/24/2011 0:00 | 12 | 49.589920 | -110.429580 | 541227 | 5493194 | 1 | 64.34 | Local |
| M3 | 12/24/2011 0:00 | 15 | 49.590460 | -110.429900 | 541203 | 5493254 | 1 | 14.54 | Local |
| M3 | 12/24/2011 0:00 | 18 | 49.590580 | -110.429980 | 541197 | 5493267 | 1 | 305.79 | Local |
| M3 | 12/24/2011 0:00 | 21 | 49.593090 | -110.431710 | 541070 | 5493545 | 1 | 384.69 | Local |
| M3 | 12/25/2011 0:00 | 0 | 49.596430 | -110.433100 | 540967 | 5493916 | 0 | 319.91 | Local |
| M3 | 12/25/2011 0:00 | 9 | 49.593610 | -110.433980 | 540906 | 5493602 | 1 | 451.83 | Local |
| M3 | 12/25/2011 0:00 | 12 | 49.590460 | -110.430030 | 541194 | 5493254 | 1 | 8.95 | Local |
| M3 | 12/25/2011 0:00 | 15 | 49.590480 | -110.430150 | 541185 | 5493256 | 1 | 688.91 | Local |
| M3 | 12/25/2011 0:00 | 18 | 49.584980 | -110.425760 | 541507 | 5492647 | 1 | 1237.08 | Local |
| M3 | 12/25/2011 0:00 | 21 | 49.573880 | -110.424570 | 541603 | 5491414 | 1 | 679.90 | Local |
| M3 | 12/26/2011 0:00 | 0 | 49.573290 | -110.415210 | 542280 | 5491353 | 1 | 1409.33 | Local |
| M3 | 12/26/2011 0:00 | 3 | 49.585710 | -110.411310 | 542551 | 5492736 | 0 | 1966.91 | Local |
| M3 | 12/26/2011 0:00 | 9 | 49.594840 | -110.434620 | 540859 | 5493738 | 0 | 591.77 | Local |
| M3 | 12/26/2011 0:00 | 15 | 49.590450 | -110.429990 | 541197 | 5493253 | 1 | 4.51 | Local |
| M3 | 12/26/2011 0:00 | 18 | 49.590490 | -110.429980 | 541198 | 5493257 | 1 | 1316.54 | Local |
| M3 | 12/26/2011 0:00 | 21 | 49.580320 | -110.439310 | 540532 | 5492121 | 1 | 1857.55 | Local |
| M3 | 12/27/2011 0:00 | 0 | 49.575950 | -110.464110 | 538742 | 5491623 | 1 | 1911.99 | Local |
| M3 | 12/27/2011 0:00 | 3 | 49.584830 | -110.441460 | 540372 | 5492622 | 1 | 478.91 | Local |
| M3 | 12/27/2011 0:00 | 6 | 49.586840 | -110.435600 | 540794 | 5492848 | 1 | 632.65 | Local |
| M3 | 12/27/2011 0:00 | 9 | 49.589940 | -110.428260 | 541322 | 5493197 | 1 | 132.05 | Local |
| M3 | 12/27/2011 0:00 | 12 | 49.590450 | -110.429910 | 541203 | 5493253 | 1 | 3.98 | Local |
| M3 | 12/27/2011 0:00 | 15 | 49.590480 | -110.429940 | 541200 | 5493256 | 1 | 9.01 | Local |
| M3 | 12/27/2011 0:00 | 18 | 49.590400 | -110.429960 | 541199 | 5493247 | 1 | 759.03 | Local |
| M3 | 12/27/2011 0:00 | 21 | 49.596980 | -110.432760 | 540991 | 5493977 | 0 | 2010.82 | Local |
| M3 | 12/28/2011 0:00 | 6 | 49.589190 | -110.407650 | 542813 | 5493125 | 1 | 654.13 | Local |
| M3 | 12/28/2011 0:00 | 9 | 49.588710 | -110.416670 | 542161 | 5493067 | 1 | 122.59 | Local |
| M3 | 12/28/2011 0:00 | 12 | 49.588160 | -110.418140 | 542055 | 5493005 | 1 | 2.44 | Local |
| M3 | 12/28/2011 0:00 | 15 | 49.588170 | -110.418170 | 542053 | 5493006 | 1 | 9.70 | Local |
| M3 | 12/28/2011 0:00 | 18 | 49.588100 | -110.418090 | 542059 | 5492998 | 1 | 1933.06 | Local |
| M3 | 12/28/2011 0:00 | 21 | 49.573180 | -110.431820 | 541079 | 5491332 | 1 | 200.91 | Local |
| M3 | 12/29/2011 0:00 | 0 | 49.571410 | -110.431260 | 541121 | 5491135 | 1 | 954.77 | Local |
| M3 | 12/29/2011 0:00 | 3 | 49.572680 | -110.444320 | 540176 | 5491269 | 1 | 1187.85 | Local |
| M3 | 12/29/2011 0:00 | 6 | 49.579950 | -110.456360 | 539299 | 5492071 | 1 | 2208.95 | Local |
| M3 | 12/29/2011 0:00 | 9 | 49.590680 | -110.430640 | 541150 | 5493278 | 1 | 57.64 | Local |
| M3 | 12/29/2011 0:00 | 12 | 49.590420 | -110.429950 | 541200 | 5493249 | 1 | 1.33 | Local |
| M3 | 12/29/2011 0:00 | 15 | 49.590430 | -110.429960 | 541199 | 5493251 | 1 | 1.33 | Local |
| M3 | 12/29/2011 0:00 | 18 | 49.590440 | -110.429970 | 541198 | 5493252 | 0 | 2287.61 | Local |
| M3 | 12/30/2011 0:00 | 3 | 49.593710 | -110.461220 | 538937 | 5493599 | 1 | 378.47 | Local |
| M3 | 12/30/2011 0:00 | 6 | 49.596650 | -110.458580 | 539126 | 5493927 | 1 | 22.46 | Local |
| M3 | 12/30/2011 0:00 | 9 | 49.596750 | -110.458310 | 539145 | 5493938 | 1 | 9.35 | Local |
| M3 | 12/30/2011 0:00 | 12 | 49.596830 | -110.458350 | 539142 | 5493947 | 1 | 17.21 | Local |
| M3 | 12/30/2011 0:00 | 15 | 49.596870 | -110.458120 | 539159 | 5493951 | 1 | 8.03 | Local |
| M3 | 12/30/2011 0:00 | 18 | 49.596860 | -110.458230 | 539151 | 5493950 | 1 | 1283.67 | Local |
| M3 | 12/30/2011 0:00 | 21 | 49.587920 | -110.469470 | 538345 | 5492951 | 1 | 3755.16 | Local |
| M3 | 12/31/2011 0:00 | 0 | 49.619970 | -110.453070 | 539505 | 5496522 | 1 | 2215.94 | Local |
| M3 | 12/31/2011 0:00 | 3 | 49.629880 | -110.479690 | 537574 | 5497610 | 1 | 1281.36 | Local |
| M3 | 12/31/2011 0:00 | 6 | 49.635360 | -110.495300 | 536443 | 5498212 | 0 | 1239.82 | Local |
| M3 | 12/31/2011 0:00 | 12 | 49.633090 | -110.478490 | 537659 | 5497968 | 1 | 9.70 | Local |
| M3 | 12/31/2011 0:00 | 15 | 49.633040 | -110.478380 | 537667 | 5497962 | 1 | 10.11 | Local |
| M3 | 12/31/2011 0:00 | 18 | 49.633040 | -110.478520 | 537657 | 5497962 | 1 | 3.65 | Local |
| M3 | 12/31/2011 0:00 | 21 | 49.633060 | -110.478560 | 537654 | 5497964 | 1 | 2398.36 | Local |
| M3 | 01/01/2012 0:00 | 0 | 49.654630 | -110.478110 | 537670 | 5500363 | 1 | 1779.82 | Local |

| | | | | | | | | | |
|---|---|---|---|---|---|---|---|---|---|
| M3 | 01/01/2012 0:00 | 3 | 49.655520 | -110.502730 | 535892 | 5500450 | 1 | 3320.28 | Local |
| M3 | 01/01/2012 0:00 | 6 | 49.655360 | -110.548730 | 532572 | 5500411 | 1 | 4695.67 | Local |
| M3 | 01/01/2012 0:00 | 9 | 49.634540 | -110.492140 | 536672 | 5498122 | 1 | 2024.77 | Local |
| M3 | 01/01/2012 0:00 | 12 | 49.628370 | -110.465760 | 538582 | 5497450 | 1 | 36.35 | Local |
| M3 | 01/01/2012 0:00 | 15 | 49.628090 | -110.465500 | 538601 | 5497419 | 1 | 4.95 | Local |
| M3 | 01/01/2012 0:00 | 18 | 49.628050 | -110.465530 | 538599 | 5497414 | 1 | 3.10 | Local |
| M3 | 01/01/2012 0:00 | 21 | 49.628040 | -110.465490 | 538601 | 5497413 | 1 | 5.88 | Local |
| M3 | 01/02/2012 0:00 | 0 | 49.628030 | -110.465570 | 538596 | 5497412 | 1 | 1090.48 | Local |
| M3 | 01/02/2012 0:00 | 3 | 49.633020 | -110.478570 | 537653 | 5497960 | 1 | 2700.32 | Local |
| M3 | 01/02/2012 0:00 | 6 | 49.633390 | -110.515960 | 534953 | 5497983 | 0 | 2707.87 | Local |
| M3 | 01/02/2012 0:00 | 15 | 49.633930 | -110.478470 | 537659 | 5498061 | 1 | 104.76 | Local |
| M3 | 01/02/2012 0:00 | 18 | 49.632990 | -110.478570 | 537653 | 5497957 | 1 | 2925.03 | Local |
| M3 | 01/02/2012 0:00 | 21 | 49.630690 | -110.518920 | 534741 | 5497682 | 1 | 4225.34 | Local |
| M3 | 01/03/2012 0:00 | 0 | 49.657050 | -110.561080 | 531679 | 5500594 | 1 | 1245.42 | Local |
| M3 | 01/03/2012 0:00 | 3 | 49.659870 | -110.577780 | 530472 | 5500900 | 1 | 335.85 | Local |
| M3 | 01/03/2012 0:00 | 6 | 49.657980 | -110.574150 | 530735 | 5500692 | 1 | 7430.57 | Local |
| M3 | 01/03/2012 0:00 | 9 | 49.632170 | -110.479210 | 537607 | 5497865 | 0 | 109.83 | Local |
| M3 | 01/03/2012 0:00 | 15 | 49.633060 | -110.478550 | 537654 | 5497964 | 1 | 815.11 | Local |
| M3 | 01/03/2012 0:00 | 18 | 49.630180 | -110.468170 | 538406 | 5497650 | 1 | 5.74 | Local |
| M3 | 01/03/2012 0:00 | 21 | 49.630230 | -110.468190 | 538405 | 5497655 | 1 | 5.74 | Local |
| M3 | 01/04/2012 0:00 | 0 | 49.630180 | -110.468170 | 538406 | 5497650 | 1 | 123.18 | Local |
| M3 | 01/04/2012 0:00 | 3 | 49.630980 | -110.466990 | 538491 | 5497739 | 1 | 314.52 | Local |
| M3 | 01/04/2012 0:00 | 6 | 49.633310 | -110.464520 | 538667 | 5497999 | 1 | 26.68 | Local |
| M3 | 01/04/2012 0:00 | 9 | 49.633550 | -110.464520 | 538667 | 5498026 | 1 | 11.49 | Local |
| M3 | 01/04/2012 0:00 | 12 | 49.633450 | -110.464560 | 538664 | 5498015 | 1 | 3.41 | Local |
| M3 | 01/04/2012 0:00 | 15 | 49.633480 | -110.464550 | 538665 | 5498018 | 0 | 2578.97 | Local |
| M3 | 01/04/2012 0:00 | 21 | 49.656490 | -110.469070 | 538321 | 5500574 | 1 | 2319.04 | Local |
| M3 | 01/05/2012 0:00 | 0 | 49.647460 | -110.498030 | 536237 | 5499556 | 1 | 2146.55 | Local |
| M3 | 01/05/2012 0:00 | 3 | 49.632830 | -110.478630 | 537649 | 5497939 | 1 | 106.11 | Local |
| M3 | 01/05/2012 0:00 | 6 | 49.633020 | -110.477190 | 537753 | 5497961 | 0 | 690.57 | Local |
| M3 | 01/05/2012 0:00 | 12 | 49.628740 | -110.470260 | 538256 | 5497488 | 1 | 170.96 | Local |
| M3 | 01/05/2012 0:00 | 15 | 49.629980 | -110.468860 | 538357 | 5497627 | 1 | 14.58 | Local |
| M3 | 01/05/2012 0:00 | 18 | 49.630090 | -110.468750 | 538364 | 5497639 | 1 | 5616.95 | Local |
| M3 | 01/05/2012 0:00 | 21 | 49.645830 | -110.542670 | 533016 | 5499354 | 1 | 5003.89 | Local |
| M3 | 01/06/2012 0:00 | 0 | 49.660940 | -110.607970 | 528293 | 5501007 | 1 | 8182.73 | Local |
| M3 | 01/06/2012 0:00 | 3 | 49.689200 | -110.712690 | 520723 | 5504115 | 1 | 71.65 | Local |
| M3 | 01/06/2012 0:00 | 6 | 49.688770 | -110.713430 | 520670 | 5504067 | 1 | 3094.83 | Local |
| M3 | 01/06/2012 0:00 | 9 | 49.700770 | -110.752150 | 517873 | 5505391 | 1 | 58.17 | Local |
| M3 | 01/06/2012 0:00 | 12 | 49.700510 | -110.751450 | 517923 | 5505363 | 1 | 8.08 | Local |
| M3 | 01/06/2012 0:00 | 15 | 49.700580 | -110.751480 | 517921 | 5505370 | 1 | 4.86 | Local |
| M3 | 01/06/2012 0:00 | 18 | 49.700560 | -110.751540 | 517917 | 5505368 | 1 | 21.12 | Local |
| M3 | 01/06/2012 0:00 | 21 | 49.700750 | -110.751540 | 517917 | 5505389 | 1 | 10.60 | Local |
| M3 | 01/07/2012 0:00 | 0 | 49.700830 | -110.751620 | 517911 | 5505398 | 1 | 25.73 | Local |
| M3 | 01/07/2012 0:00 | 3 | 49.701040 | -110.751770 | 517900 | 5505421 | 1 | 601.33 | Local |
| M3 | 01/07/2012 0:00 | 6 | 49.704920 | -110.757580 | 517480 | 5505851 | 0 | 232.33 | Local |
| M3 | 01/07/2012 0:00 | 12 | 49.706900 | -110.756550 | 517553 | 5506072 | 0 | 9.35 | Local |
| M3 | 01/07/2012 0:00 | 18 | 49.706820 | -110.756590 | 517550 | 5506063 | 1 | 1160.40 | Local |
| M3 | 01/07/2012 0:00 | 21 | 49.717230 | -110.757750 | 517463 | 5507220 | 1 | 466.60 | Local |
| M3 | 01/08/2012 0:00 | 0 | 49.719630 | -110.763060 | 517079 | 5507486 | 1 | 9.28 | Local |
| M3 | 01/08/2012 0:00 | 3 | 49.719560 | -110.763130 | 517074 | 5507478 | 1 | 421.68 | Local |
| M3 | 01/08/2012 0:00 | 6 | 49.722410 | -110.766990 | 516795 | 5507794 | 0 | 6.26 | Local |
| M3 | 01/08/2012 0:00 | 18 | 49.722360 | -110.766950 | 516798 | 5507788 | 1 | 466.63 | Local |
| M3 | 01/08/2012 0:00 | 21 | 49.725260 | -110.771630 | 516460 | 5508110 | 1 | 1492.82 | Local |
| M3 | 01/09/2012 0:00 | 0 | 49.738470 | -110.775340 | 516188 | 5509577 | 1 | 2219.19 | Local |
| M3 | 01/09/2012 0:00 | 3 | 49.739470 | -110.744580 | 518404 | 5509696 | 1 | 1175.87 | Local |

| | | | | | | | | | |
|---|---|---|---|---|---|---|---|---|---|
| M3 | 01/09/2012 0:00 | 6 | 49.729560 | -110.750280 | 517997 | 5508593 | 0 | 1765.17 | Local |
| M3 | 01/09/2012 0:00 | 21 | 49.717810 | -110.766750 | 516814 | 5507282 | 1 | 23.01 | Local |
| M3 | 01/10/2012 0:00 | 0 | 49.717690 | -110.766490 | 516833 | 5507269 | 1 | 32.58 | Local |
| M3 | 01/10/2012 0:00 | 3 | 49.717920 | -110.766770 | 516812 | 5507295 | 1 | 288.58 | Local |
| M3 | 01/10/2012 0:00 | 6 | 49.720430 | -110.765750 | 516885 | 5507574 | 1 | 225.90 | Local |
| M3 | 01/10/2012 0:00 | 9 | 49.722360 | -110.766730 | 516814 | 5507788 | 0 | 13.72 | Local |
| M3 | 01/10/2012 0:00 | 15 | 49.722400 | -110.766910 | 516801 | 5507793 | 1 | 179.12 | Local |
| M3 | 01/10/2012 0:00 | 18 | 49.724010 | -110.767000 | 516794 | 5507972 | 1 | 1178.19 | Local |
| M3 | 01/10/2012 0:00 | 21 | 49.734600 | -110.767600 | 516747 | 5509149 | 1 | 583.83 | Local |
| M3 | 01/11/2012 0:00 | 0 | 49.736970 | -110.774830 | 516225 | 5509411 | 1 | 30.51 | Local |
| M3 | 01/11/2012 0:00 | 3 | 49.737060 | -110.775230 | 516196 | 5509421 | 1 | 146.56 | Local |
| M3 | 01/11/2012 0:00 | 6 | 49.736820 | -110.773230 | 516340 | 5509394 | 1 | 1485.45 | Local |
| M3 | 01/11/2012 0:00 | 9 | 49.724080 | -110.767020 | 516792 | 5507979 | 1 | 8.89 | Local |
| M3 | 01/11/2012 0:00 | 12 | 49.724000 | -110.767020 | 516792 | 5507971 | 1 | 2.43 | Local |
| M3 | 01/11/2012 0:00 | 15 | 49.723990 | -110.767050 | 516790 | 5507969 | 1 | 3.41 | Local |
| M3 | 01/11/2012 0:00 | 18 | 49.724020 | -110.767040 | 516791 | 5507973 | 1 | 472.29 | Local |
| M3 | 01/11/2012 0:00 | 21 | 49.727490 | -110.770820 | 516517 | 5508358 | 1 | 1990.13 | Local |
| M3 | 01/12/2012 0:00 | 0 | 49.718350 | -110.747080 | 518232 | 5507347 | 1 | 31.02 | Local |
| M3 | 01/12/2012 0:00 | 3 | 49.718360 | -110.746650 | 518263 | 5507348 | 1 | 321.99 | Local |
| M3 | 01/12/2012 0:00 | 6 | 49.715960 | -110.749150 | 518083 | 5507081 | 1 | 1277.80 | Local |
| M3 | 01/12/2012 0:00 | 9 | 49.720290 | -110.765570 | 516898 | 5507558 | 0 | 12.52 | Local |
| M3 | 01/12/2012 0:00 | 15 | 49.720390 | -110.765490 | 516904 | 5507570 | 1 | 11.04 | Local |
| M3 | 01/12/2012 0:00 | 18 | 49.720370 | -110.765640 | 516893 | 5507567 | 1 | 1415.93 | Local |
| M3 | 01/12/2012 0:00 | 21 | 49.707680 | -110.767300 | 516778 | 5506156 | 1 | 1895.43 | Local |
| M3 | 01/13/2012 0:00 | 0 | 49.691570 | -110.758700 | 517404 | 5504367 | 1 | 1073.49 | Local |
| M3 | 01/13/2012 0:00 | 3 | 49.700790 | -110.754280 | 517719 | 5505393 | 1 | 846.82 | Local |
| M3 | 01/13/2012 0:00 | 6 | 49.708040 | -110.757880 | 517457 | 5506198 | 1 | 233.89 | Local |
| M3 | 01/13/2012 0:00 | 9 | 49.710130 | -110.757510 | 517483 | 5506431 | 0 | 10.41 | Local |
| M3 | 01/13/2012 0:00 | 15 | 49.710040 | -110.757550 | 517480 | 5506421 | 1 | 22.10 | Local |
| M3 | 01/13/2012 0:00 | 18 | 49.710220 | -110.757680 | 517471 | 5506441 | 1 | 725.53 | Local |
| M3 | 01/13/2012 0:00 | 21 | 49.711460 | -110.767560 | 516758 | 5506576 | 1 | 135.93 | Local |
| M3 | 01/14/2012 0:00 | 0 | 49.712300 | -110.768930 | 516659 | 5506669 | 1 | 194.18 | Local |
| M3 | 01/14/2012 0:00 | 3 | 49.710570 | -110.768560 | 516686 | 5506477 | 1 | 603.65 | Local |
| M3 | 01/14/2012 0:00 | 6 | 49.715980 | -110.767850 | 516735 | 5507079 | 1 | 519.21 | Local |
| M3 | 01/14/2012 0:00 | 9 | 49.720390 | -110.765480 | 516905 | 5507570 | 1 | 252.96 | Local |
| M3 | 01/14/2012 0:00 | 12 | 49.722450 | -110.766970 | 516797 | 5507798 | 1 | 9.89 | Local |
| M3 | 01/14/2012 0:00 | 15 | 49.722370 | -110.766910 | 516801 | 5507789 | 1 | 51.15 | Local |
| M3 | 01/14/2012 0:00 | 18 | 49.722750 | -110.767310 | 516772 | 5507832 | 1 | 1283.37 | Local |
| M3 | 01/14/2012 0:00 | 21 | 49.733980 | -110.771430 | 516471 | 5509079 | 1 | 777.05 | Local |
| M3 | 01/15/2012 0:00 | 0 | 49.740870 | -110.773240 | 516338 | 5509845 | 1 | 876.83 | Local |
| M3 | 01/15/2012 0:00 | 3 | 49.745650 | -110.782920 | 515639 | 5510374 | 1 | 1708.28 | Local |
| M3 | 01/15/2012 0:00 | 6 | 49.732210 | -110.771430 | 516472 | 5508882 | 1 | 747.72 | Local |
| M3 | 01/15/2012 0:00 | 9 | 49.725770 | -110.768440 | 516689 | 5508167 | 0 | 7.58 | Local |
| M3 | 01/15/2012 0:00 | 18 | 49.725830 | -110.768490 | 516686 | 5508174 | 1 | 1021.67 | Local |
| M3 | 01/15/2012 0:00 | 21 | 49.735000 | -110.767570 | 516749 | 5509193 | 0 | 23.31 | Local |
| M3 | 01/16/2012 0:00 | 3 | 49.735060 | -110.767880 | 516727 | 5509200 | 1 | 1024.94 | Local |
| M3 | 01/16/2012 0:00 | 6 | 49.725850 | -110.768500 | 516685 | 5508176 | 1 | 400.32 | Local |
| M3 | 01/16/2012 0:00 | 9 | 49.722400 | -110.766910 | 516801 | 5507793 | 1 | 0.72 | Local |
| M3 | 01/16/2012 0:00 | 12 | 49.722400 | -110.766920 | 516800 | 5507793 | 1 | 11.59 | Local |
| M3 | 01/16/2012 0:00 | 15 | 49.722390 | -110.766760 | 516812 | 5507792 | 1 | 11.59 | Local |
| M3 | 01/16/2012 0:00 | 18 | 49.722380 | -110.766920 | 516800 | 5507790 | 1 | 802.01 | Local |
| M3 | 01/16/2012 0:00 | 21 | 49.729190 | -110.770590 | 516533 | 5508547 | 0 | 401.35 | Local |
| M3 | 01/17/2012 0:00 | 6 | 49.725860 | -110.768440 | 516689 | 5508177 | 1 | 6.73 | Local |
| M3 | 01/17/2012 0:00 | 9 | 49.725900 | -110.768510 | 516684 | 5508181 | 1 | 7.51 | Local |
| M3 | 01/17/2012 0:00 | 12 | 49.725850 | -110.768440 | 516689 | 5508176 | 0 | 2094.95 | Local |

| | | | | | | | | | |
|---|---|---|---|---|---|---|---|---|---|
| M3 | 01/17/2012 0:00 | 21 | 49.711890 | -110.748920 | 518101 | 5506628 | 0 | 1279.61 | Local |
| M3 | 01/18/2012 0:00 | 15 | 49.700530 | -110.751770 | 517900 | 5505365 | 1 | 10.87 | Local |
| M3 | 01/18/2012 0:00 | 18 | 49.700540 | -110.751620 | 517911 | 5505366 | 1 | 1229.66 | Local |
| M3 | 01/18/2012 0:00 | 21 | 49.711500 | -110.753910 | 517742 | 5506584 | 1 | 218.23 | Local |
| M3 | 01/19/2012 0:00 | 0 | 49.710600 | -110.751220 | 517936 | 5506484 | 1 | 4.95 | Local |
| M3 | 01/19/2012 0:00 | 3 | 49.710560 | -110.751190 | 517938 | 5506480 | 1 | 3.63 | Local |
| M3 | 01/19/2012 0:00 | 6 | 49.710590 | -110.751210 | 517937 | 5506483 | 1 | 6.05 | Local |
| M3 | 01/19/2012 0:00 | 9 | 49.710560 | -110.751280 | 517932 | 5506480 | 1 | 3.63 | Local |
| M3 | 01/19/2012 0:00 | 12 | 49.710590 | -110.751260 | 517933 | 5506483 | 1 | 3.10 | Local |
| M3 | 01/19/2012 0:00 | 15 | 49.710570 | -110.751230 | 517935 | 5506481 | 1 | 2.43 | Local |
| M3 | 01/19/2012 0:00 | 18 | 49.710560 | -110.751260 | 517933 | 5506480 | 1 | 438.95 | Local |
| M3 | 01/19/2012 0:00 | 21 | 49.714130 | -110.753860 | 517745 | 5506876 | 1 | 1.44 | Local |
| M3 | 01/20/2012 0:00 | 0 | 49.714130 | -110.753840 | 517746 | 5506876 | 1 | 2.16 | Local |
| M3 | 01/20/2012 0:00 | 3 | 49.714130 | -110.753870 | 517744 | 5506876 | 1 | 1124.14 | Local |
| M3 | 01/20/2012 0:00 | 6 | 49.720800 | -110.765590 | 516897 | 5507615 | 1 | 202.38 | Local |
| M3 | 01/20/2012 0:00 | 9 | 49.722410 | -110.766900 | 516802 | 5507794 | 1 | 1.32 | Local |
| M3 | 01/20/2012 0:00 | 12 | 49.722400 | -110.766890 | 516802 | 5507793 | 1 | 3.10 | Local |
| M3 | 01/20/2012 0:00 | 15 | 49.722380 | -110.766860 | 516804 | 5507790 | 1 | 3.77 | Local |
| M3 | 01/20/2012 0:00 | 18 | 49.722390 | -110.766910 | 516801 | 5507792 | 1 | 2387.79 | Local |
| M3 | 01/20/2012 0:00 | 21 | 49.742340 | -110.779180 | 515910 | 5510007 | 1 | 2332.94 | Local |
| M3 | 01/21/2012 0:00 | 0 | 49.748140 | -110.748060 | 518150 | 5510659 | 1 | 1157.40 | Local |
| M3 | 01/21/2012 0:00 | 3 | 49.739750 | -110.757570 | 517468 | 5509724 | 0 | 2219.79 | Local |
| M3 | 01/21/2012 0:00 | 21 | 49.722240 | -110.772370 | 516407 | 5507774 | 1 | 991.22 | Local |
| M3 | 01/22/2012 0:00 | 0 | 49.713350 | -110.771330 | 516485 | 5506786 | 1 | 1313.04 | Local |
| M3 | 01/22/2012 0:00 | 3 | 49.701940 | -110.766630 | 516828 | 5505518 | 1 | 42.61 | Local |
| M3 | 01/22/2012 0:00 | 6 | 49.701800 | -110.767180 | 516788 | 5505502 | 0 | 713.28 | Local |
| M3 | 01/22/2012 0:00 | 21 | 49.708130 | -110.768790 | 516670 | 5506206 | 1 | 794.74 | Local |
| M3 | 01/23/2012 0:00 | 0 | 49.715270 | -110.769320 | 516630 | 5506999 | 1 | 724.65 | Local |
| M3 | 01/23/2012 0:00 | 3 | 49.721440 | -110.766080 | 516861 | 5507686 | 1 | 2.16 | Local |
| M3 | 01/23/2012 0:00 | 6 | 49.721440 | -110.766050 | 516863 | 5507686 | 1 | 158.72 | Local |
| M3 | 01/23/2012 0:00 | 9 | 49.720140 | -110.765140 | 516929 | 5507542 | 0 | 469.85 | Local |
| M3 | 01/23/2012 0:00 | 21 | 49.717680 | -110.759840 | 517312 | 5507270 | 1 | 126.08 | Local |
| M3 | 01/24/2012 0:00 | 0 | 49.718540 | -110.760980 | 517230 | 5507365 | 1 | 349.03 | Local |
| M3 | 01/24/2012 0:00 | 3 | 49.720510 | -110.764750 | 516957 | 5507583 | 0 | 20.24 | Local |
| M3 | 01/24/2012 0:00 | 9 | 49.720460 | -110.765020 | 516938 | 5507577 | 0 | 18.91 | Local |
| M3 | 01/24/2012 0:00 | 18 | 49.720570 | -110.765220 | 516923 | 5507590 | 1 | 1012.13 | Local |
| M3 | 01/24/2012 0:00 | 21 | 49.729230 | -110.769550 | 516608 | 5508551 | 1 | 80.14 | Local |
| M3 | 01/25/2012 0:00 | 0 | 49.729900 | -110.769140 | 516638 | 5508626 | 1 | 1.32 | Local |
| M3 | 01/25/2012 0:00 | 3 | 49.729890 | -110.769130 | 516638 | 5508625 | 1 | 2.16 | Local |
| M3 | 01/25/2012 0:00 | 6 | 49.729890 | -110.769160 | 516636 | 5508625 | 1 | 69.18 | Local |
| M3 | 01/25/2012 0:00 | 9 | 49.729380 | -110.769710 | 516597 | 5508568 | 1 | 3.63 | Local |
| M3 | 01/25/2012 0:00 | 12 | 49.729350 | -110.769730 | 516595 | 5508565 | 1 | 6.71 | Local |
| M3 | 01/25/2012 0:00 | 15 | 49.729290 | -110.769720 | 516596 | 5508558 | 1 | 4.51 | Local |
| M3 | 01/25/2012 0:00 | 18 | 49.729330 | -110.769710 | 516597 | 5508563 | 1 | 4.23 | Local |
| M3 | 01/25/2012 0:00 | 21 | 49.729350 | -110.769660 | 516600 | 5508565 | 1 | 1.32 | Local |
| M3 | 01/26/2012 0:00 | 0 | 49.729340 | -110.769670 | 516600 | 5508564 | 1 | 13.65 | Local |
| M3 | 01/26/2012 0:00 | 3 | 49.729220 | -110.769710 | 516597 | 5508550 | 1 | 86.74 | Local |
| M3 | 01/26/2012 0:00 | 6 | 49.729900 | -110.769120 | 516639 | 5508626 | 1 | 71.55 | Local |
| M3 | 01/26/2012 0:00 | 9 | 49.729360 | -110.769660 | 516600 | 5508566 | 1 | 2.43 | Local |
| M3 | 01/26/2012 0:00 | 12 | 49.729370 | -110.769690 | 516598 | 5508567 | 1 | 0.72 | Local |
| M3 | 01/26/2012 0:00 | 15 | 49.729370 | -110.769680 | 516599 | 5508567 | 1 | 4.51 | Local |
| M3 | 01/26/2012 0:00 | 18 | 49.729330 | -110.769670 | 516600 | 5508563 | 1 | 4.41 | Local |
| M3 | 01/26/2012 0:00 | 21 | 49.729360 | -110.769630 | 516602 | 5508566 | 1 | 7.86 | Local |
| M3 | 01/27/2012 0:00 | 0 | 49.729320 | -110.769720 | 516596 | 5508561 | 1 | 0.72 | Local |
| M3 | 01/27/2012 0:00 | 3 | 49.729320 | -110.769730 | 516595 | 5508561 | 1 | 8.36 | Local |

| | | | | | | | | | |
|---|---|---|---|---|---|---|---|---|---|
| M3 | 01/27/2012 0:00 | 6 | 49.729380 | -110.769660 | 516600 | 5508568 | 1 | 3.41 | Local |
| M3 | 01/27/2012 0:00 | 9 | 49.729350 | -110.769670 | 516600 | 5508565 | 1 | 5.46 | Local |
| M3 | 01/27/2012 0:00 | 12 | 49.729320 | -110.769610 | 516604 | 5508561 | 1 | 6.20 | Local |
| M3 | 01/27/2012 0:00 | 15 | 49.729360 | -110.769670 | 516600 | 5508566 | 1 | 2.34 | Local |
| M3 | 01/27/2012 0:00 | 18 | 49.729380 | -110.769680 | 516599 | 5508568 | 1 | 513.91 | Local |
| M3 | 01/27/2012 0:00 | 21 | 49.729810 | -110.762580 | 517110 | 5508617 | 1 | 1972.06 | Local |
| M3 | 01/28/2012 0:00 | 0 | 49.721460 | -110.738440 | 518853 | 5507695 | 0 | 1490.33 | Local |
| M3 | 01/28/2012 0:00 | 6 | 49.711280 | -110.751890 | 517888 | 5506560 | 0 | 19.48 | Local |
| M3 | 01/28/2012 0:00 | 12 | 49.711120 | -110.752000 | 517880 | 5506542 | 0 | 1101.54 | Local |
| M3 | 01/28/2012 0:00 | 21 | 49.701290 | -110.753910 | 517746 | 5505449 | 1 | 3911.12 | Local |
| M3 | 01/29/2012 0:00 | 0 | 49.680720 | -110.709920 | 520927 | 5503173 | 1 | 5981.77 | Local |
| M3 | 01/29/2012 0:00 | 3 | 49.654610 | -110.637440 | 526169 | 5500293 | 1 | 9295.07 | Local |
| M3 | 01/29/2012 0:00 | 6 | 49.641680 | -110.510230 | 535360 | 5498907 | 1 | 2487.83 | Local |
| M3 | 01/29/2012 0:00 | 9 | 49.633240 | -110.478320 | 537671 | 5497985 | 1 | 25.74 | Local |
| M3 | 01/29/2012 0:00 | 12 | 49.633030 | -110.478470 | 537660 | 5497961 | 1 | 7.56 | Local |
| M3 | 01/29/2012 0:00 | 15 | 49.633010 | -110.478570 | 537653 | 5497959 | 0 | 7.92 | Local |
| M3 | 01/29/2012 0:00 | 21 | 49.633080 | -110.478590 | 537651 | 5497967 | 1 | 656.91 | Local |
| M3 | 01/30/2012 0:00 | 0 | 49.628330 | -110.473180 | 538046 | 5497441 | 1 | 16.93 | Local |
| M3 | 01/30/2012 0:00 | 3 | 49.628480 | -110.473140 | 538049 | 5497458 | 1 | 219.82 | Local |
| M3 | 01/30/2012 0:00 | 6 | 49.630040 | -110.471270 | 538182 | 5497632 | 1 | 108.52 | Local |
| M3 | 01/30/2012 0:00 | 9 | 49.629300 | -110.470290 | 538254 | 5497551 | 1 | 133.53 | Local |
| M3 | 01/30/2012 0:00 | 12 | 49.630020 | -110.468810 | 538360 | 5497631 | 0 | 164.77 | Local |
| M3 | 01/30/2012 0:00 | 18 | 49.631210 | -110.467450 | 538457 | 5497764 | 1 | 1927.94 | Local |
| M3 | 01/30/2012 0:00 | 21 | 49.648360 | -110.463500 | 538729 | 5499673 | 1 | 991.02 | Local |
| M3 | 01/31/2012 0:00 | 0 | 49.656510 | -110.469060 | 538321 | 5500576 | 1 | 981.26 | Local |
| M3 | 01/31/2012 0:00 | 3 | 49.664770 | -110.473850 | 537969 | 5501492 | 1 | 533.61 | Local |
| M3 | 01/31/2012 0:00 | 6 | 49.661860 | -110.467970 | 538396 | 5501172 | 1 | 1589.17 | Local |
| M3 | 01/31/2012 0:00 | 9 | 49.647720 | -110.471190 | 538174 | 5499598 | 1 | 58.21 | Local |
| M3 | 01/31/2012 0:00 | 12 | 49.648150 | -110.471650 | 538141 | 5499646 | 1 | 9.28 | Local |
| M3 | 01/31/2012 0:00 | 15 | 49.648220 | -110.471580 | 538146 | 5499653 | 0 | 1672.70 | Local |
| M3 | 01/31/2012 0:00 | 21 | 49.651920 | -110.494040 | 536522 | 5500054 | 1 | 3845.69 | Local |
| M3 | 02/01/2012 0:00 | 0 | 49.644920 | -110.546210 | 532761 | 5499251 | 1 | 1818.59 | Local |
| M3 | 02/01/2012 0:00 | 3 | 49.637290 | -110.523930 | 534374 | 5498413 | 1 | 1652.80 | Local |
| M3 | 02/01/2012 0:00 | 6 | 49.631750 | -110.502690 | 535912 | 5497807 | 1 | 2209.60 | Local |
| M3 | 02/01/2012 0:00 | 9 | 49.629870 | -110.472230 | 538113 | 5497613 | 1 | 75.36 | Local |
| M3 | 02/01/2012 0:00 | 12 | 49.630120 | -110.471260 | 538183 | 5497641 | 1 | 5.61 | Local |
| M3 | 02/01/2012 0:00 | 15 | 49.630170 | -110.471250 | 538184 | 5497647 | 1 | 4.51 | Local |
| M3 | 02/01/2012 0:00 | 18 | 49.630130 | -110.471260 | 538183 | 5497642 | 1 | 1171.58 | Local |
| M3 | 02/01/2012 0:00 | 21 | 49.624840 | -110.485290 | 537174 | 5497047 | 1 | 1150.67 | Local |
| M3 | 02/02/2012 0:00 | 0 | 49.618490 | -110.497870 | 536270 | 5496335 | 1 | 2937.20 | Local |
| M3 | 02/02/2012 0:00 | 3 | 49.635840 | -110.528540 | 534043 | 5498250 | 1 | 1507.19 | Local |
| M3 | 02/02/2012 0:00 | 6 | 49.643180 | -110.546090 | 532770 | 5499058 | 0 | 5000.59 | Local |
| M3 | 02/02/2012 0:00 | 12 | 49.633060 | -110.478610 | 537650 | 5497964 | 0 | 1.44 | Local |
| M3 | 02/02/2012 0:00 | 18 | 49.633060 | -110.478590 | 537651 | 5497964 | 1 | 1981.65 | Local |
| M3 | 02/02/2012 0:00 | 21 | 49.638860 | -110.504540 | 535773 | 5498597 | 1 | 5312.51 | Local |
| M3 | 02/03/2012 0:00 | 0 | 49.655800 | -110.573350 | 530794 | 5500450 | 1 | 2177.76 | Local |
| M3 | 02/03/2012 0:00 | 3 | 49.667800 | -110.597200 | 529066 | 5501774 | 1 | 5452.34 | Local |
| M3 | 02/03/2012 0:00 | 6 | 49.662670 | -110.522060 | 534492 | 5501236 | 1 | 5155.39 | Local |
| M3 | 02/03/2012 0:00 | 9 | 49.630100 | -110.471230 | 538185 | 5497639 | 1 | 6.26 | Local |
| M3 | 02/03/2012 0:00 | 12 | 49.630150 | -110.471270 | 538182 | 5497645 | 1 | 4.45 | Local |
| M3 | 02/03/2012 0:00 | 15 | 49.630110 | -110.471270 | 538182 | 5497640 | 1 | 4.95 | Local |
| M3 | 02/03/2012 0:00 | 18 | 49.630150 | -110.471240 | 538185 | 5497645 | 0 | 17.06 | Local |
| M3 | 02/04/2012 0:00 | 0 | 49.630000 | -110.471290 | 538181 | 5497628 | 1 | 1195.51 | Local |
| M3 | 02/04/2012 0:00 | 3 | 49.626710 | -110.487050 | 537045 | 5497254 | 1 | 18.89 | Local |
| M3 | 02/04/2012 0:00 | 6 | 49.626760 | -110.487300 | 537027 | 5497260 | 1 | 1020.87 | Local |

| | | | | | | | | | |
|---|---|---|---|---|---|---|---|---|---|
| M3 | 02/04/2012 0:00 | 9 | 49.628970 | -110.473580 | 538016 | 5497512 | 1 | 273.19 | Local |
| M3 | 02/04/2012 0:00 | 12 | 49.629870 | -110.470060 | 538270 | 5497614 | 0 | 67.16 | Local |
| M3 | 02/04/2012 0:00 | 18 | 49.629290 | -110.470320 | 538252 | 5497550 | 0 | 1374.14 | Local |
| M3 | 02/05/2012 0:00 | 0 | 49.626070 | -110.488690 | 536927 | 5497182 | 1 | 138.70 | Local |
| M3 | 02/05/2012 0:00 | 3 | 49.626760 | -110.487090 | 537042 | 5497260 | 1 | 8.67 | Local |
| M3 | 02/05/2012 0:00 | 6 | 49.626760 | -110.487210 | 537034 | 5497260 | 1 | 995.23 | Local |
| M3 | 02/05/2012 0:00 | 9 | 49.628980 | -110.473860 | 537996 | 5497513 | 1 | 77.93 | Local |
| M3 | 02/05/2012 0:00 | 12 | 49.628540 | -110.473020 | 538057 | 5497465 | 0 | 48.09 | Local |
| M3 | 02/05/2012 0:00 | 18 | 49.628680 | -110.473650 | 538012 | 5497480 | 1 | 1204.72 | Local |
| M3 | 02/05/2012 0:00 | 21 | 49.626150 | -110.489870 | 536842 | 5497191 | 0 | 40.10 | Local |
| M3 | 02/06/2012 0:00 | 3 | 49.626430 | -110.489520 | 536867 | 5497222 | 1 | 50.16 | Local |
| M3 | 02/06/2012 0:00 | 6 | 49.626240 | -110.490150 | 536822 | 5497201 | 1 | 29.42 | Local |
| M3 | 02/06/2012 0:00 | 9 | 49.626190 | -110.489750 | 536851 | 5497195 | 1 | 4.51 | Local |
| M3 | 02/06/2012 0:00 | 12 | 49.626230 | -110.489760 | 536850 | 5497200 | 1 | 5.61 | Local |
| M3 | 02/06/2012 0:00 | 15 | 49.626180 | -110.489750 | 536851 | 5497194 | 1 | 193.40 | Local |
| M3 | 02/06/2012 0:00 | 18 | 49.626750 | -110.487220 | 537033 | 5497259 | 1 | 108.58 | Local |
| M3 | 02/06/2012 0:00 | 21 | 49.626880 | -110.488710 | 536925 | 5497272 | 1 | 105.71 | Local |
| M3 | 02/07/2012 0:00 | 0 | 49.626750 | -110.487260 | 537030 | 5497259 | 1 | 1.82 | Local |
| M3 | 02/07/2012 0:00 | 3 | 49.626760 | -110.487280 | 537029 | 5497260 | 1 | 331.99 | Local |
| M3 | 02/07/2012 0:00 | 6 | 49.625940 | -110.491700 | 536710 | 5497166 | 1 | 13.05 | Local |
| M3 | 02/07/2012 0:00 | 9 | 49.625930 | -110.491880 | 536697 | 5497165 | 1 | 347.88 | Local |
| M3 | 02/07/2012 0:00 | 12 | 49.626770 | -110.487240 | 537032 | 5497261 | 1 | 1.33 | Local |
| M3 | 02/07/2012 0:00 | 15 | 49.626780 | -110.487250 | 537031 | 5497262 | 1 | 6.06 | Local |
| M3 | 02/07/2012 0:00 | 18 | 49.626750 | -110.487180 | 537036 | 5497259 | 1 | 335.46 | Local |
| M3 | 02/07/2012 0:00 | 21 | 49.625930 | -110.491650 | 536714 | 5497165 | 0 | 335.95 | Local |
| M3 | 02/08/2012 0:00 | 3 | 49.626670 | -110.487140 | 537039 | 5497250 | 1 | 336.65 | Local |
| M3 | 02/08/2012 0:00 | 6 | 49.625930 | -110.491660 | 536713 | 5497165 | 1 | 335.29 | Local |
| M3 | 02/08/2012 0:00 | 9 | 49.626560 | -110.487120 | 537040 | 5497238 | 1 | 12.57 | Local |
| M3 | 02/08/2012 0:00 | 12 | 49.626670 | -110.487160 | 537037 | 5497250 | 1 | 10.64 | Local |
| M3 | 02/08/2012 0:00 | 15 | 49.626760 | -110.487210 | 537034 | 5497260 | 1 | 2.89 | Local |
| M3 | 02/08/2012 0:00 | 18 | 49.626760 | -110.487250 | 537031 | 5497260 | 0 | 322.28 | Local |
| M3 | 02/09/2012 0:00 | 0 | 49.626240 | -110.491640 | 536714 | 5497200 | 1 | 44.69 | Local |
| M3 | 02/09/2012 0:00 | 3 | 49.625850 | -110.491790 | 536704 | 5497156 | 1 | 128.87 | Local |
| M3 | 02/09/2012 0:00 | 6 | 49.626810 | -110.490790 | 536775 | 5497264 | 1 | 1461.58 | Local |
| M3 | 02/09/2012 0:00 | 9 | 49.630160 | -110.471220 | 538186 | 5497646 | 1 | 0.00 | Local |
| M3 | 02/09/2012 0:00 | 12 | 49.630160 | -110.471220 | 538186 | 5497646 | 1 | 7.01 | Local |
| M3 | 02/09/2012 0:00 | 15 | 49.630100 | -110.471190 | 538188 | 5497639 | 1 | 5.52 | Local |
| M3 | 02/09/2012 0:00 | 18 | 49.630120 | -110.471260 | 538183 | 5497641 | 1 | 2372.71 | Local |
| M3 | 02/09/2012 0:00 | 21 | 49.635260 | -110.503150 | 535876 | 5498197 | 1 | 2749.19 | Local |
| M3 | 02/10/2012 0:00 | 0 | 49.655290 | -110.480820 | 537473 | 5500435 | 1 | 1290.78 | Local |
| M3 | 02/10/2012 0:00 | 3 | 49.648970 | -110.465820 | 538561 | 5499740 | 1 | 1934.52 | Local |
| M3 | 02/10/2012 0:00 | 6 | 49.633940 | -110.479320 | 537598 | 5498062 | 1 | 111.21 | Local |
| M3 | 02/10/2012 0:00 | 9 | 49.633070 | -110.478560 | 537654 | 5497966 | 1 | 604.93 | Local |
| M3 | 02/10/2012 0:00 | 12 | 49.630300 | -110.471350 | 538176 | 5497661 | 1 | 202.48 | Local |
| M3 | 02/10/2012 0:00 | 15 | 49.630120 | -110.468560 | 538378 | 5497643 | 1 | 11.93 | Local |
| M3 | 02/10/2012 0:00 | 18 | 49.630020 | -110.468500 | 538382 | 5497632 | 1 | 1658.79 | Local |
| M3 | 02/10/2012 0:00 | 21 | 49.644220 | -110.461450 | 538880 | 5499214 | 1 | 2132.77 | Local |
| M3 | 02/11/2012 0:00 | 0 | 49.663390 | -110.460360 | 538944 | 5501346 | 0 | 3661.80 | Local |
| M3 | 02/11/2012 0:00 | 6 | 49.632610 | -110.478410 | 537665 | 5497915 | 0 | 3074.90 | Local |
| M3 | 02/11/2012 0:00 | 21 | 49.638430 | -110.520040 | 534655 | 5498542 | 1 | 3316.98 | Local |
| M3 | 02/12/2012 0:00 | 0 | 49.662180 | -110.547850 | 532631 | 5501170 | 1 | 3300.78 | Local |
| M3 | 02/12/2012 0:00 | 3 | 49.649680 | -110.506370 | 535633 | 5499799 | 1 | 923.47 | Local |
| M3 | 02/12/2012 0:00 | 6 | 49.650050 | -110.493590 | 536556 | 5499846 | 1 | 1598.70 | Local |
| M3 | 02/12/2012 0:00 | 9 | 49.648240 | -110.471620 | 538143 | 5499656 | 1 | 10.03 | Local |
| M3 | 02/12/2012 0:00 | 12 | 49.648150 | -110.471630 | 538142 | 5499646 | 1 | 2.34 | Local |

| | | | | | | | | | |
|---|---|---|---|---|---|---|---|---|---|
| M3 | 02/12/2012 0:00 | 15 | 49.648170 | -110.471640 | 538142 | 5499648 | 0 | 1055.50 | Local |
| M3 | 02/12/2012 0:00 | 21 | 49.649740 | -110.486060 | 537099 | 5499815 | 1 | 810.66 | Local |
| M3 | 02/13/2012 0:00 | 0 | 49.652690 | -110.496330 | 536356 | 5500138 | 1 | 2865.55 | Local |
| M3 | 02/13/2012 0:00 | 3 | 49.658690 | -110.534940 | 533565 | 5500787 | 1 | 3944.13 | Local |
| M3 | 02/13/2012 0:00 | 6 | 49.636830 | -110.491910 | 536687 | 5498377 | 1 | 1146.22 | Local |
| M3 | 02/13/2012 0:00 | 9 | 49.632120 | -110.477790 | 537710 | 5497860 | 1 | 685.96 | Local |
| M3 | 02/13/2012 0:00 | 12 | 49.630090 | -110.468820 | 538359 | 5497639 | 1 | 41.75 | Local |
| M3 | 02/13/2012 0:00 | 15 | 49.629940 | -110.468290 | 538398 | 5497623 | 1 | 4.33 | Local |
| M3 | 02/13/2012 0:00 | 18 | 49.629940 | -110.468350 | 538393 | 5497623 | 1 | 2800.60 | Local |
| M3 | 02/13/2012 0:00 | 21 | 49.641760 | -110.502600 | 535911 | 5498920 | 0 | 7300.73 | Local |
| M3 | 02/14/2012 0:00 | 3 | 49.664420 | -110.597530 | 529044 | 5501398 | 1 | 402.52 | Local |
| M3 | 02/14/2012 0:00 | 6 | 49.667300 | -110.600910 | 528798 | 5501717 | 0 | 70.64 | Local |
| M3 | 02/14/2012 0:00 | 15 | 49.667550 | -110.600010 | 528863 | 5501745 | 1 | 10.41 | Local |
| M3 | 02/14/2012 0:00 | 18 | 49.667640 | -110.600050 | 528860 | 5501755 | 1 | 7.58 | Local |
| M3 | 02/14/2012 0:00 | 21 | 49.667580 | -110.600000 | 528864 | 5501749 | 1 | 6.21 | Local |
| M3 | 02/15/2012 0:00 | 0 | 49.667540 | -110.599940 | 528868 | 5501744 | 1 | 6.59 | Local |
| M3 | 02/15/2012 0:00 | 3 | 49.667530 | -110.600030 | 528862 | 5501743 | 1 | 6.26 | Local |
| M3 | 02/15/2012 0:00 | 6 | 49.667580 | -110.599990 | 528865 | 5501749 | 1 | 0.72 | Local |
| M3 | 02/15/2012 0:00 | 9 | 49.667580 | -110.600000 | 528864 | 5501749 | 1 | 1.33 | Local |
| M3 | 02/15/2012 0:00 | 12 | 49.667570 | -110.599990 | 528865 | 5501747 | 1 | 1.11 | Local |
| M3 | 02/15/2012 0:00 | 15 | 49.667560 | -110.599990 | 528865 | 5501746 | 1 | 3.10 | Local |
| M3 | 02/15/2012 0:00 | 18 | 49.667540 | -110.600020 | 528863 | 5501744 | 1 | 3.63 | Local |
| M3 | 02/15/2012 0:00 | 21 | 49.667570 | -110.600040 | 528861 | 5501747 | 1 | 1.33 | Local |
| M3 | 02/16/2012 0:00 | 0 | 49.667560 | -110.600030 | 528862 | 5501746 | 1 | 3.64 | Local |
| M3 | 02/16/2012 0:00 | 3 | 49.667540 | -110.600070 | 528859 | 5501744 | 1 | 2.89 | Local |
| M3 | 02/16/2012 0:00 | 6 | 49.667540 | -110.600030 | 528862 | 5501744 | 1 | 3.98 | Local |
| M3 | 02/16/2012 0:00 | 9 | 49.667570 | -110.600000 | 528864 | 5501747 | 0 | 9.01 | Local |
| M3 | 02/16/2012 0:00 | 18 | 49.667490 | -110.600020 | 528863 | 5501739 | 1 | 2.65 | Local |
| M3 | 02/16/2012 0:00 | 21 | 49.667510 | -110.600000 | 528864 | 5501741 | 1 | 1.33 | Local |
| M3 | 02/17/2012 0:00 | 0 | 49.667500 | -110.600010 | 528863 | 5501740 | 1 | 3.61 | Local |
| M3 | 02/17/2012 0:00 | 3 | 49.667500 | -110.599960 | 528867 | 5501740 | 1 | 1.82 | Local |
| M3 | 02/17/2012 0:00 | 6 | 49.667510 | -110.599980 | 528865 | 5501741 | 0 | 6133.94 | Local |
| M3 | 02/17/2012 0:00 | 12 | 49.644430 | -110.522790 | 534452 | 5499207 | 1 | 3462.21 | Local |
| M3 | 02/17/2012 0:00 | 15 | 49.632450 | -110.478530 | 537656 | 5497897 | 1 | 66.85 | Local |
| M3 | 02/17/2012 0:00 | 18 | 49.633050 | -110.478470 | 537660 | 5497963 | 1 | 11.55 | Local |
| M3 | 02/17/2012 0:00 | 21 | 49.633050 | -110.478630 | 537649 | 5497963 | 1 | 15.85 | Local |
| M3 | 02/18/2012 0:00 | 0 | 49.632920 | -110.478720 | 537642 | 5497949 | 1 | 12.88 | Local |
| M3 | 02/18/2012 0:00 | 3 | 49.633020 | -110.478630 | 537649 | 5497960 | 1 | 5.73 | Local |
| M3 | 02/18/2012 0:00 | 6 | 49.632980 | -110.478580 | 537652 | 5497956 | 1 | 842.70 | Local |
| M3 | 02/18/2012 0:00 | 9 | 49.628480 | -110.469190 | 538334 | 5497460 | 1 | 194.56 | Local |
| M3 | 02/18/2012 0:00 | 12 | 49.630050 | -110.468000 | 538419 | 5497635 | 1 | 341.65 | Local |
| M3 | 02/18/2012 0:00 | 15 | 49.632930 | -110.466350 | 538535 | 5497956 | 1 | 414.12 | Local |
| M3 | 02/18/2012 0:00 | 18 | 49.633940 | -110.460830 | 538933 | 5498071 | 1 | 1874.11 | Local |
| M3 | 02/18/2012 0:00 | 21 | 49.649660 | -110.470200 | 538244 | 5499814 | 1 | 763.06 | Local |
| M3 | 02/19/2012 0:00 | 0 | 49.656490 | -110.469160 | 538314 | 5500574 | 1 | 275.38 | Local |
| M3 | 02/19/2012 0:00 | 3 | 49.656360 | -110.465350 | 538589 | 5500562 | 1 | 2867.92 | Local |
| M3 | 02/19/2012 0:00 | 6 | 49.632690 | -110.481140 | 537468 | 5497922 | 0 | 970.53 | Local |
| M3 | 02/19/2012 0:00 | 12 | 49.629930 | -110.468390 | 538390 | 5497622 | 1 | 11.21 | Local |
| M3 | 02/19/2012 0:00 | 15 | 49.630020 | -110.468320 | 538395 | 5497632 | 1 | 34.95 | Local |
| M3 | 02/19/2012 0:00 | 18 | 49.629740 | -110.468540 | 538380 | 5497600 | 1 | 1293.56 | Local |
| M3 | 02/19/2012 0:00 | 21 | 49.619520 | -110.477100 | 537770 | 5496460 | 1 | 1961.21 | Local |
| M3 | 02/20/2012 0:00 | 0 | 49.618000 | -110.504150 | 535817 | 5496278 | 1 | 1959.27 | Local |
| M3 | 02/20/2012 0:00 | 3 | 49.608180 | -110.526670 | 534197 | 5495175 | 1 | 3429.23 | Local |
| M3 | 02/20/2012 0:00 | 6 | 49.634850 | -110.502820 | 535901 | 5498152 | 1 | 1773.77 | Local |
| M3 | 02/20/2012 0:00 | 9 | 49.632800 | -110.478460 | 537661 | 5497936 | 1 | 33.21 | Local |

| | | | | | | | | | | |
|---|---|---|---|---|---|---|---|---|---|---|
| M3 | 02/20/2012 0:00 | 12 | 49.633090 | -110.478570 | 537653 | 5497968 | | 1 | 1.33 | Local |
| M3 | 02/20/2012 0:00 | 15 | 49.633100 | -110.478560 | 537654 | 5497969 | | 1 | 6.67 | Local |
| M3 | 02/20/2012 0:00 | 18 | 49.633040 | -110.478560 | 537654 | 5497962 | | 1 | 2889.66 | Local |
| M3 | 02/20/2012 0:00 | 21 | 49.644800 | -110.514250 | 535068 | 5499252 | | 1 | 5023.28 | Local |
| M3 | 02/21/2012 0:00 | 0 | 49.662300 | -110.578410 | 530425 | 5501170 | | 1 | 1948.13 | Local |
| M3 | 02/21/2012 0:00 | 3 | 49.669920 | -110.602720 | 528666 | 5502008 | | 1 | 555.54 | Local |
| M3 | 02/21/2012 0:00 | 6 | 49.673410 | -110.608230 | 528267 | 5502394 | | 1 | 8010.29 | Local |
| M3 | 02/21/2012 0:00 | 9 | 49.642800 | -110.507760 | 535538 | 5499033 | | 1 | 2988.39 | Local |
| M3 | 02/21/2012 0:00 | 12 | 49.630130 | -110.471260 | 538183 | 5497642 | | 1 | 0.00 | Local |
| M3 | 02/21/2012 0:00 | 15 | 49.630130 | -110.471260 | 538183 | 5497642 | | 1 | 2.44 | Local |
| M3 | 02/21/2012 0:00 | 18 | 49.630120 | -110.471230 | 538185 | 5497641 | | 1 | 76.45 | Local |
| M3 | 02/21/2012 0:00 | 21 | 49.629860 | -110.472210 | 538115 | 5497612 | | 1 | 7.82 | Local |
| M3 | 02/22/2012 0:00 | 0 | 49.629790 | -110.472220 | 538114 | 5497604 | | 1 | 0.00 | Local |
| M3 | 02/22/2012 0:00 | 3 | 49.629790 | -110.472220 | 538114 | 5497604 | | 1 | 11.55 | Local |
| M3 | 02/22/2012 0:00 | 6 | 49.629790 | -110.472060 | 538126 | 5497604 | | 1 | 262.11 | Local |
| M3 | 02/22/2012 0:00 | 9 | 49.629960 | -110.468440 | 538387 | 5497625 | | 1 | 16.82 | Local |
| M3 | 02/22/2012 0:00 | 12 | 49.629810 | -110.468470 | 538385 | 5497608 | | 1 | 11.69 | Local |
| M3 | 02/22/2012 0:00 | 15 | 49.629910 | -110.468420 | 538388 | 5497619 | | 1 | 978.73 | Local |
| M3 | 02/22/2012 0:00 | 18 | 49.634320 | -110.456690 | 539232 | 5498116 | | 1 | 2140.34 | Local |
| M3 | 02/22/2012 0:00 | 21 | 49.650400 | -110.472990 | 538042 | 5499895 | | 1 | 3872.62 | Local |
| M3 | 02/23/2012 0:00 | 0 | 49.676820 | -110.438020 | 540545 | 5502851 | | 1 | 3921.38 | Local |
| M3 | 02/23/2012 0:00 | 3 | 49.647230 | -110.408450 | 542704 | 5499577 | | 1 | 3398.81 | Local |
| M3 | 02/23/2012 0:00 | 6 | 49.636060 | -110.452270 | 539550 | 5498312 | | 1 | 465.23 | Local |
| M3 | 02/23/2012 0:00 | 9 | 49.633520 | -110.457390 | 539182 | 5498026 | | 1 | 1.82 | Local |
| M3 | 02/23/2012 0:00 | 12 | 49.633510 | -110.457410 | 539181 | 5498025 | | 1 | 1.44 | Local |
| M3 | 02/23/2012 0:00 | 15 | 49.633510 | -110.457430 | 539179 | 5498025 | | 1 | 4.41 | Local |
| M3 | 02/23/2012 0:00 | 18 | 49.633540 | -110.457390 | 539182 | 5498029 | | 1 | 2627.43 | Local |
| M3 | 02/23/2012 0:00 | 21 | 49.642310 | -110.491180 | 536735 | 5498987 | | 1 | 2390.15 | Local |
| M3 | 02/24/2012 0:00 | 0 | 49.655670 | -110.517120 | 534853 | 5500460 | | 1 | 3.98 | Local |
| M3 | 02/24/2012 0:00 | 3 | 49.655640 | -110.517090 | 534855 | 5500456 | | 1 | 3036.63 | Local |
| M3 | 02/24/2012 0:00 | 6 | 49.634790 | -110.489920 | 536832 | 5498151 | | 0 | 842.19 | Local |
| M3 | 02/24/2012 0:00 | 15 | 49.633020 | -110.478580 | 537652 | 5497960 | | 1 | 5.05 | Local |
| M3 | 02/24/2012 0:00 | 18 | 49.633020 | -110.478650 | 537647 | 5497960 | | 1 | 8.67 | Local |
| M3 | 02/24/2012 0:00 | 21 | 49.633020 | -110.478530 | 537656 | 5497960 | | 1 | 2320.49 | Local |
| M3 | 02/25/2012 0:00 | 0 | 49.626000 | -110.508790 | 535476 | 5497165 | | 1 | 9.89 | Local |
| M3 | 02/25/2012 0:00 | 3 | 49.626080 | -110.508730 | 535480 | 5497174 | | 1 | 22.53 | Local |
| M3 | 02/25/2012 0:00 | 6 | 49.625880 | -110.508780 | 535477 | 5497152 | | 1 | 2940.29 | Local |
| M3 | 02/25/2012 0:00 | 9 | 49.630340 | -110.468650 | 538371 | 5497667 | | 1 | 113.17 | Local |
| M3 | 02/25/2012 0:00 | 12 | 49.630120 | -110.467120 | 538482 | 5497643 | | 1 | 781.01 | Local |
| M3 | 02/25/2012 0:00 | 15 | 49.633330 | -110.457500 | 539174 | 5498005 | | 0 | 646.39 | Local |
| M3 | 02/26/2012 0:00 | 0 | 49.631090 | -110.465760 | 538580 | 5497752 | | 1 | 950.12 | Local |
| M3 | 02/26/2012 0:00 | 3 | 49.633040 | -110.478570 | 537653 | 5497962 | | 1 | 2.65 | Local |
| M3 | 02/26/2012 0:00 | 6 | 49.633020 | -110.478590 | 537652 | 5497960 | | 1 | 4.45 | Local |
| M3 | 02/26/2012 0:00 | 9 | 49.633060 | -110.478590 | 537651 | 5497964 | | 1 | 0.72 | Local |
| M3 | 02/26/2012 0:00 | 12 | 49.633060 | -110.478600 | 537651 | 5497964 | | 1 | 12.88 | Local |
| M3 | 02/26/2012 0:00 | 15 | 49.632960 | -110.478510 | 537657 | 5497953 | | 1 | 9.89 | Local |
| M3 | 02/26/2012 0:00 | 18 | 49.633040 | -110.478570 | 537653 | 5497962 | | 1 | 2307.43 | Local |
| M3 | 02/26/2012 0:00 | 21 | 49.626320 | -110.508800 | 535475 | 5497200 | | 1 | 80.25 | Local |
| M3 | 02/27/2012 0:00 | 0 | 49.625610 | -110.508600 | 535490 | 5497122 | | 1 | 12.75 | Local |
| M3 | 02/27/2012 0:00 | 3 | 49.625720 | -110.508650 | 535486 | 5497134 | | 1 | 1451.63 | Local |
| M3 | 02/27/2012 0:00 | 6 | 49.625150 | -110.488570 | 536937 | 5497080 | | 1 | 1545.56 | Local |
| M3 | 02/27/2012 0:00 | 9 | 49.629940 | -110.468480 | 538384 | 5497623 | | 1 | 9.29 | Local |
| M3 | 02/27/2012 0:00 | 12 | 49.629910 | -110.468360 | 538393 | 5497619 | | 1 | 734.45 | Local |
| M3 | 02/27/2012 0:00 | 15 | 49.629890 | -110.458190 | 539127 | 5497622 | | 0 | 204.90 | Local |
| M3 | 02/27/2012 0:00 | 21 | 49.631210 | -110.456210 | 539269 | 5497770 | | 1 | 1098.34 | Local |

| | | | | | | | | | |
|---|---|---|---|---|---|---|---|---|---|
| M3 | 02/28/2012 0:00 | 0 | 49.641030 | -110.457870 | 539141 | 5498861 | 0 | 2678.14 | Local |
| M3 | 02/28/2012 0:00 | 21 | 49.661150 | -110.478270 | 537653 | 5501088 | 1 | 2491.63 | Local |
| M3 | 02/29/2012 0:00 | 0 | 49.657120 | -110.512230 | 535205 | 5500623 | 1 | 304.64 | Local |
| M3 | 02/29/2012 0:00 | 3 | 49.655850 | -110.515970 | 534936 | 5500480 | 1 | 1.82 | Local |
| M3 | 02/29/2012 0:00 | 6 | 49.655840 | -110.515990 | 534935 | 5500479 | 0 | 3709.75 | Local |
| M3 | 02/29/2012 0:00 | 12 | 49.633000 | -110.478530 | 537656 | 5497958 | 1 | 4.92 | Local |
| M3 | 02/29/2012 0:00 | 15 | 49.633030 | -110.478580 | 537652 | 5497961 | 1 | 5.47 | Local |
| M3 | 02/29/2012 0:00 | 18 | 49.633060 | -110.478640 | 537648 | 5497964 | 1 | 6.19 | Local |
| M3 | 02/29/2012 0:00 | 21 | 49.633080 | -110.478560 | 537654 | 5497967 | 0 | 15.16 | Local |
| M3 | 03/01/2012 0:00 | 3 | 49.633000 | -110.478730 | 537641 | 5497958 | 1 | 1333.31 | Local |
| M3 | 03/01/2012 0:00 | 6 | 49.632540 | -110.460280 | 538974 | 5497916 | 1 | 326.44 | Local |
| M3 | 03/01/2012 0:00 | 9 | 49.631160 | -110.456290 | 539263 | 5497765 | 1 | 272.96 | Local |
| M3 | 03/01/2012 0:00 | 12 | 49.629570 | -110.453410 | 539473 | 5497589 | 1 | 275.18 | Local |
| M3 | 03/01/2012 0:00 | 15 | 49.631290 | -110.456150 | 539273 | 5497779 | 1 | 20.28 | Local |
| M3 | 03/01/2012 0:00 | 18 | 49.631240 | -110.456420 | 539254 | 5497773 | 1 | 1688.02 | Local |
| M3 | 03/01/2012 0:00 | 21 | 49.645130 | -110.465860 | 538561 | 5499313 | 1 | 5493.07 | Local |
| M3 | 03/02/2012 0:00 | 0 | 49.643610 | -110.541910 | 533072 | 5499108 | 0 | 4916.09 | Local |
| M3 | 03/02/2012 0:00 | 18 | 49.666320 | -110.600350 | 528839 | 5501608 | 1 | 763.71 | Local |
| M3 | 03/02/2012 0:00 | 21 | 49.663280 | -110.609840 | 528156 | 5501267 | 1 | 1023.98 | Local |
| M3 | 03/03/2012 0:00 | 0 | 49.659590 | -110.596840 | 529097 | 5500862 | 1 | 353.06 | Local |
| M3 | 03/03/2012 0:00 | 3 | 49.659290 | -110.591970 | 529448 | 5500830 | 1 | 4131.58 | Local |
| M3 | 03/03/2012 0:00 | 6 | 49.646340 | -110.538320 | 533329 | 5499413 | 1 | 4486.83 | Local |
| M3 | 03/03/2012 0:00 | 9 | 49.633020 | -110.479660 | 537574 | 5497959 | 0 | 80.88 | Local |
| M3 | 03/03/2012 0:00 | 15 | 49.633020 | -110.478540 | 537655 | 5497960 | 1 | 24.38 | Local |
| M3 | 03/03/2012 0:00 | 18 | 49.632950 | -110.478860 | 537632 | 5497952 | 1 | 25.51 | Local |
| M3 | 03/03/2012 0:00 | 21 | 49.633060 | -110.478550 | 537654 | 5497964 | 0 | 11.93 | Local |
| M3 | 03/04/2012 0:00 | 3 | 49.632980 | -110.478660 | 537646 | 5497956 | 1 | 1942.19 | Local |
| M3 | 03/04/2012 0:00 | 6 | 49.650340 | -110.475660 | 537850 | 5499887 | 1 | 1938.31 | Local |
| M3 | 03/04/2012 0:00 | 9 | 49.637990 | -110.456710 | 539227 | 5498524 | 1 | 2.43 | Local |
| M3 | 03/04/2012 0:00 | 12 | 49.638000 | -110.456680 | 539230 | 5498525 | 1 | 6.06 | Local |
| M3 | 03/04/2012 0:00 | 15 | 49.638030 | -110.456750 | 539225 | 5498528 | 1 | 4.95 | Local |
| M3 | 03/04/2012 0:00 | 18 | 49.637990 | -110.456720 | 539227 | 5498524 | 1 | 1942.65 | Local |
| M3 | 03/04/2012 0:00 | 21 | 49.652000 | -110.472800 | 538055 | 5500073 | 1 | 3131.62 | Local |
| M3 | 03/05/2012 0:00 | 0 | 49.654800 | -110.515970 | 534937 | 5500363 | 1 | 3285.04 | Local |
| M3 | 03/05/2012 0:00 | 3 | 49.671810 | -110.553190 | 532239 | 5502238 | 1 | 824.98 | Local |
| M3 | 03/05/2012 0:00 | 6 | 49.666440 | -110.545300 | 532812 | 5501644 | 1 | 6831.57 | Local |
| M3 | 03/05/2012 0:00 | 9 | 49.630180 | -110.468900 | 538353 | 5497649 | 1 | 33.45 | Local |
| M3 | 03/05/2012 0:00 | 12 | 49.630040 | -110.468490 | 538383 | 5497634 | 1 | 5.06 | Local |
| M3 | 03/05/2012 0:00 | 15 | 49.630040 | -110.468560 | 538378 | 5497634 | 1 | 9.60 | Local |
| M3 | 03/05/2012 0:00 | 18 | 49.629960 | -110.468610 | 538375 | 5497625 | 1 | 18.21 | Local |
| M3 | 03/05/2012 0:00 | 21 | 49.630040 | -110.468390 | 538390 | 5497634 | 1 | 239.09 | Local |
| M3 | 03/06/2012 0:00 | 0 | 49.630450 | -110.471640 | 538155 | 5497678 | 1 | 6644.33 | Local |
| M3 | 03/06/2012 0:00 | 3 | 49.666670 | -110.544850 | 532844 | 5501670 | 1 | 2904.53 | Local |
| M3 | 03/06/2012 0:00 | 6 | 49.656560 | -110.507740 | 535529 | 5500563 | 1 | 4042.21 | Local |
| M3 | 03/06/2012 0:00 | 9 | 49.632190 | -110.466190 | 538548 | 5497874 | 1 | 3.78 | Local |
| M3 | 03/06/2012 0:00 | 12 | 49.632200 | -110.466240 | 538544 | 5497875 | 1 | 21.23 | Local |
| M3 | 03/06/2012 0:00 | 15 | 49.632010 | -110.466270 | 538542 | 5497854 | 1 | 17.23 | Local |
| M3 | 03/06/2012 0:00 | 18 | 49.632160 | -110.466210 | 538546 | 5497871 | 1 | 0.00 | Local |
| M3 | 03/06/2012 0:00 | 21 | 49.632160 | -110.466210 | 538546 | 5497871 | 1 | 6.67 | Local |
| M3 | 03/07/2012 0:00 | 0 | 49.632220 | -110.466210 | 538546 | 5497877 | 1 | 3.41 | Local |
| M3 | 03/07/2012 0:00 | 3 | 49.632190 | -110.466220 | 538545 | 5497874 | 0 | 781.94 | Local |
| M3 | 03/07/2012 0:00 | 9 | 49.634980 | -110.456280 | 539261 | 5498189 | 1 | 338.51 | Local |
| M3 | 03/07/2012 0:00 | 12 | 49.638010 | -110.456740 | 539225 | 5498526 | 1 | 0.72 | Local |
| M3 | 03/07/2012 0:00 | 15 | 49.638010 | -110.456730 | 539226 | 5498526 | 1 | 4.51 | Local |
| M3 | 03/07/2012 0:00 | 18 | 49.638050 | -110.456740 | 539225 | 5498530 | 1 | 282.75 | Local |

| | | | | | | | | | |
|---|---|---|---|---|---|---|---|---|---|
| M3 | 03/07/2012 0:00 | 21 | 49.638690 | -110.452950 | 539498 | 5498604 | 1 | 1474.57 | Local |
| M3 | 03/08/2012 0:00 | 0 | 49.648310 | -110.467010 | 538476 | 5499666 | 1 | 1569.87 | Local |
| M3 | 03/08/2012 0:00 | 3 | 49.660030 | -110.479140 | 537591 | 5500963 | 1 | 370.13 | Local |
| M3 | 03/08/2012 0:00 | 6 | 49.656970 | -110.481160 | 537448 | 5500621 | 1 | 2984.64 | Local |
| M3 | 03/08/2012 0:00 | 9 | 49.631770 | -110.466910 | 538496 | 5497827 | 1 | 150.44 | Local |
| M3 | 03/08/2012 0:00 | 12 | 49.632210 | -110.464940 | 538638 | 5497877 | 0 | 77.62 | Local |
| M3 | 03/08/2012 0:00 | 18 | 49.631800 | -110.464070 | 538701 | 5497832 | 1 | 1907.37 | Local |
| M3 | 03/08/2012 0:00 | 21 | 49.635000 | -110.490020 | 536825 | 5498174 | 1 | 3529.85 | Local |
| M3 | 03/09/2012 0:00 | 0 | 49.658680 | -110.522590 | 534456 | 5500792 | 1 | 4712.96 | Local |
| M3 | 03/09/2012 0:00 | 3 | 49.673410 | -110.583830 | 530027 | 5502403 | 1 | 4493.15 | Local |
| M3 | 03/09/2012 0:00 | 6 | 49.656430 | -110.527330 | 534116 | 5500539 | 0 | 5107.67 | Local |
| M3 | 03/09/2012 0:00 | 12 | 49.631830 | -110.467580 | 538447 | 5497833 | 1 | 47.07 | Local |
| M3 | 03/09/2012 0:00 | 15 | 49.631910 | -110.466940 | 538494 | 5497843 | 1 | 12.72 | Local |
| M3 | 03/09/2012 0:00 | 18 | 49.631970 | -110.467090 | 538483 | 5497849 | 1 | 20.75 | Local |
| M3 | 03/09/2012 0:00 | 21 | 49.631850 | -110.466870 | 538499 | 5497836 | 1 | 11.49 | Local |
| M3 | 03/10/2012 0:00 | 0 | 49.631950 | -110.466910 | 538496 | 5497847 | 1 | 7.92 | Local |
| M3 | 03/10/2012 0:00 | 3 | 49.631880 | -110.466890 | 538497 | 5497839 | 0 | 12.97 | Local |
| M3 | 03/10/2012 0:00 | 9 | 49.631990 | -110.466830 | 538502 | 5497851 | 1 | 608.66 | Local |
| M3 | 03/10/2012 0:00 | 12 | 49.627870 | -110.461280 | 538906 | 5497396 | 1 | 120.58 | Local |
| M3 | 03/10/2012 0:00 | 15 | 49.628650 | -110.460120 | 538989 | 5497484 | 1 | 600.64 | Local |
| M3 | 03/10/2012 0:00 | 18 | 49.627710 | -110.451930 | 539581 | 5497383 | 1 | 3048.82 | Local |
| M3 | 03/10/2012 0:00 | 21 | 49.648390 | -110.479660 | 537562 | 5499668 | 1 | 3142.14 | Local |
| M3 | 03/11/2012 0:00 | 0 | 49.657720 | -110.520750 | 534590 | 5500686 | 1 | 6472.31 | Local |
| M3 | 03/11/2012 0:00 | 3 | 49.673800 | -110.606950 | 528359 | 5502437 | 1 | 4076.25 | Local |
| M3 | 03/11/2012 0:00 | 6 | 49.653880 | -110.559530 | 531793 | 5500242 | 1 | 4357.78 | Local |
| M3 | 03/11/2012 0:00 | 9 | 49.641510 | -110.502250 | 535937 | 5498892 | 1 | 114.32 | Local |
| M3 | 03/11/2012 0:00 | 12 | 49.641300 | -110.500700 | 536049 | 5498870 | 1 | 45.44 | Local |
| M3 | 03/11/2012 0:00 | 15 | 49.641090 | -110.500160 | 536088 | 5498847 | 1 | 8.94 | Local |
| M3 | 03/11/2012 0:00 | 18 | 49.641070 | -110.500040 | 536097 | 5498844 | 1 | 1041.35 | Local |
| M3 | 03/11/2012 0:00 | 21 | 49.639290 | -110.514200 | 535076 | 5498640 | 1 | 5736.42 | Local |
| M3 | 03/12/2012 0:00 | 0 | 49.662100 | -110.585480 | 529915 | 5501145 | 1 | 5.97 | Local |
| M3 | 03/12/2012 0:00 | 3 | 49.662050 | -110.585510 | 529913 | 5501139 | 1 | 4854.23 | Local |
| M3 | 03/12/2012 0:00 | 6 | 49.651600 | -110.520210 | 534633 | 5500006 | 1 | 4247.54 | Local |
| M3 | 03/12/2012 0:00 | 9 | 49.631690 | -110.470000 | 538273 | 5497816 | 1 | 200.45 | Local |
| M3 | 03/12/2012 0:00 | 12 | 49.630190 | -110.468460 | 538385 | 5497651 | 0 | 3.10 | Local |
| M3 | 03/12/2012 0:00 | 18 | 49.630200 | -110.468420 | 538388 | 5497652 | 1 | 221.75 | Local |
| M3 | 03/12/2012 0:00 | 21 | 49.632010 | -110.467130 | 538480 | 5497854 | 1 | 1001.17 | Local |
| M3 | 03/13/2012 0:00 | 0 | 49.639490 | -110.459410 | 539031 | 5498689 | 1 | 10.93 | Local |
| M3 | 03/13/2012 0:00 | 3 | 49.639430 | -110.459290 | 539040 | 5498683 | 1 | 13.34 | Local |
| M3 | 03/13/2012 0:00 | 6 | 49.639360 | -110.459440 | 539029 | 5498675 | 1 | 648.32 | Local |
| M3 | 03/13/2012 0:00 | 9 | 49.635700 | -110.452450 | 539537 | 5498271 | 1 | 1.82 | Local |
| M3 | 03/13/2012 0:00 | 12 | 49.635710 | -110.452470 | 539535 | 5498272 | 1 | 654.32 | Local |
| M3 | 03/13/2012 0:00 | 15 | 49.631480 | -110.446170 | 539994 | 5497806 | 1 | 267.97 | Local |
| M3 | 03/13/2012 0:00 | 18 | 49.629070 | -110.446220 | 539992 | 5497538 | 1 | 3725.97 | Local |
| M3 | 03/13/2012 0:00 | 21 | 49.605740 | -110.483250 | 537336 | 5494925 | 1 | 3929.26 | Local |
| M3 | 03/14/2012 0:00 | 0 | 49.570400 | -110.483810 | 537322 | 5490996 | 1 | 3445.33 | Local |
| M3 | 03/14/2012 0:00 | 3 | 49.553210 | -110.444170 | 540203 | 5489105 | 1 | 978.24 | Local |
| M3 | 03/14/2012 0:00 | 6 | 49.552680 | -110.457670 | 539227 | 5489039 | 1 | 4612.88 | Local |
| M3 | 03/14/2012 0:00 | 9 | 49.589610 | -110.428590 | 541299 | 5493160 | 0 | 15.34 | Local |
| M3 | 03/14/2012 0:00 | 15 | 49.589590 | -110.428380 | 541314 | 5493158 | 1 | 18.15 | Local |
| M3 | 03/14/2012 0:00 | 18 | 49.589750 | -110.428330 | 541317 | 5493176 | 1 | 1067.99 | Local |
| M3 | 03/14/2012 0:00 | 21 | 49.593350 | -110.442030 | 540324 | 5493569 | 1 | 2891.64 | Local |
| M3 | 03/15/2012 0:00 | 0 | 49.573930 | -110.468640 | 538416 | 5491396 | 1 | 3255.49 | Local |
| M3 | 03/15/2012 0:00 | 3 | 49.545510 | -110.457800 | 539223 | 5488242 | 1 | 3100.82 | Local |
| M3 | 03/15/2012 0:00 | 6 | 49.569610 | -110.436220 | 540764 | 5490932 | 1 | 1856.94 | Local |

| | | | | | | | | | |
|---|---|---|---|---|---|---|---|---|---|
| M3 | 03/15/2012 0:00 | 9 | 49.584590 | -110.424860 | 541573 | 5492604 | 1 | 35.47 | Local |
| M3 | 03/15/2012 0:00 | 12 | 49.584820 | -110.425200 | 541548 | 5492629 | 1 | 5.06 | Local |
| M3 | 03/15/2012 0:00 | 15 | 49.584820 | -110.425270 | 541543 | 5492629 | 1 | 3.34 | Local |
| M3 | 03/15/2012 0:00 | 18 | 49.584790 | -110.425270 | 541543 | 5492626 | 1 | 2030.21 | Local |
| M3 | 03/15/2012 0:00 | 21 | 49.601140 | -110.437780 | 540625 | 5494437 | 1 | 2781.07 | Local |
| M3 | 03/16/2012 0:00 | 0 | 49.624590 | -110.451180 | 539638 | 5497037 | 1 | 3648.70 | Local |
| M3 | 03/16/2012 0:00 | 3 | 49.639840 | -110.495920 | 536395 | 5498710 | 1 | 69.39 | Local |
| M3 | 03/16/2012 0:00 | 6 | 49.639220 | -110.496030 | 536388 | 5498641 | 1 | 3.64 | Local |
| M3 | 03/16/2012 0:00 | 9 | 49.639240 | -110.496070 | 536385 | 5498643 | 1 | 3.41 | Local |
| M3 | 03/16/2012 0:00 | 12 | 49.639210 | -110.496080 | 536384 | 5498640 | 1 | 3.10 | Local |
| M3 | 03/16/2012 0:00 | 15 | 49.639190 | -110.496110 | 536382 | 5498637 | 1 | 3.09 | Local |
| M3 | 03/16/2012 0:00 | 18 | 49.639180 | -110.496150 | 536379 | 5498636 | 1 | 5.73 | Local |
| M3 | 03/16/2012 0:00 | 21 | 49.639220 | -110.496100 | 536382 | 5498641 | 1 | 6.87 | Local |
| M3 | 03/17/2012 0:00 | 0 | 49.639240 | -110.496010 | 536389 | 5498643 | 1 | 2.22 | Local |
| M3 | 03/17/2012 0:00 | 3 | 49.639220 | -110.496010 | 536389 | 5498641 | 1 | 5.30 | Local |
| M3 | 03/17/2012 0:00 | 6 | 49.639180 | -110.495970 | 536392 | 5498636 | 1 | 28.51 | Local |
| M3 | 03/17/2012 0:00 | 9 | 49.639140 | -110.496360 | 536364 | 5498632 | 1 | 0.00 | Local |
| M3 | 03/17/2012 0:00 | 12 | 49.639140 | -110.496360 | 536364 | 5498632 | 1 | 2.17 | Local |
| M3 | 03/17/2012 0:00 | 15 | 49.639140 | -110.496390 | 536362 | 5498632 | 1 | 22.09 | Local |
| M3 | 03/17/2012 0:00 | 18 | 49.639220 | -110.496110 | 536382 | 5498641 | 1 | 2.34 | Local |
| M3 | 03/17/2012 0:00 | 21 | 49.639240 | -110.496100 | 536382 | 5498643 | 1 | 7.55 | Local |
| M3 | 03/18/2012 0:00 | 0 | 49.639220 | -110.496000 | 536390 | 5498641 | 1 | 0.00 | Local |
| M3 | 03/18/2012 0:00 | 3 | 49.639220 | -110.496000 | 536390 | 5498641 | 1 | 1.11 | Local |
| M3 | 03/18/2012 0:00 | 6 | 49.639210 | -110.496000 | 536390 | 5498640 | 1 | 22.36 | Local |
| M3 | 03/18/2012 0:00 | 9 | 49.639160 | -110.496300 | 536368 | 5498634 | 1 | 9.15 | Local |
| M3 | 03/18/2012 0:00 | 12 | 49.639080 | -110.496330 | 536366 | 5498625 | 1 | 19.39 | Local |
| M3 | 03/18/2012 0:00 | 15 | 49.639170 | -110.496100 | 536383 | 5498635 | 1 | 17.33 | Local |
| M3 | 03/18/2012 0:00 | 18 | 49.639170 | -110.496340 | 536365 | 5498635 | 1 | 25.07 | Local |
| M3 | 03/18/2012 0:00 | 21 | 49.639240 | -110.496010 | 536389 | 5498643 | 1 | 12.47 | Local |
| M3 | 03/19/2012 0:00 | 0 | 49.639220 | -110.495840 | 536401 | 5498641 | 1 | 9.39 | Local |
| M3 | 03/19/2012 0:00 | 3 | 49.639220 | -110.495970 | 536392 | 5498641 | 1 | 7.87 | Local |
| M3 | 03/19/2012 0:00 | 6 | 49.639180 | -110.496060 | 536385 | 5498636 | 1 | 3.64 | Local |
| M3 | 03/19/2012 0:00 | 9 | 49.639200 | -110.496100 | 536383 | 5498638 | 1 | 3.10 | Local |
| M3 | 03/19/2012 0:00 | 12 | 49.639220 | -110.496070 | 536385 | 5498641 | 1 | 3.64 | Local |
| M3 | 03/19/2012 0:00 | 15 | 49.639200 | -110.496110 | 536382 | 5498638 | 1 | 66.29 | Local |
| M3 | 03/19/2012 0:00 | 18 | 49.639030 | -110.496990 | 536318 | 5498619 | 1 | 66.98 | Local |
| M3 | 03/19/2012 0:00 | 21 | 49.639200 | -110.496100 | 536383 | 5498638 | 1 | 1.33 | Local |
| M3 | 03/20/2012 0:00 | 0 | 49.639190 | -110.496090 | 536383 | 5498637 | 1 | 5.74 | Local |
| M3 | 03/20/2012 0:00 | 3 | 49.639240 | -110.496070 | 536385 | 5498643 | 1 | 2.43 | Local |
| M3 | 03/20/2012 0:00 | 6 | 49.639230 | -110.496100 | 536382 | 5498642 | 1 | 2552.22 | Local |
| M3 | 03/20/2012 0:00 | 9 | 49.630470 | -110.463430 | 538748 | 5497684 | 0 | 966.73 | Local |
| M3 | 03/21/2012 0:00 | 0 | 49.637960 | -110.456630 | 539233 | 5498520 | 1 | 263.89 | Local |
| M3 | 03/21/2012 0:00 | 3 | 49.639020 | -110.459900 | 538996 | 5498637 | 1 | 896.47 | Local |
| M3 | 03/21/2012 0:00 | 6 | 49.631280 | -110.456420 | 539254 | 5497778 | 1 | 20.23 | Local |
| M3 | 03/21/2012 0:00 | 9 | 49.631110 | -110.456320 | 539261 | 5497759 | 0 | 3011.67 | Local |
| M3 | 03/22/2012 0:00 | 0 | 49.639250 | -110.496100 | 536382 | 5498644 | 1 | 62.91 | Local |
| M3 | 03/22/2012 0:00 | 3 | 49.639280 | -110.495230 | 536445 | 5498648 | 1 | 63.06 | Local |
| M3 | 03/22/2012 0:00 | 6 | 49.639230 | -110.496100 | 536382 | 5498642 | 1 | 1469.92 | Local |
| M3 | 03/22/2012 0:00 | 9 | 49.632780 | -110.478330 | 537670 | 5497933 | 0 | 38.58 | Local |
| M3 | 03/22/2012 0:00 | 15 | 49.633090 | -110.478570 | 537653 | 5497968 | 1 | 15.83 | Local |
| M3 | 03/22/2012 0:00 | 18 | 49.632950 | -110.478530 | 537656 | 5497952 | 1 | 83.72 | Local |
| M3 | 03/22/2012 0:00 | 21 | 49.632660 | -110.479600 | 537579 | 5497919 | 1 | 1389.39 | Local |
| M3 | 03/23/2012 0:00 | 0 | 49.639280 | -110.495920 | 536395 | 5498647 | 1 | 13.74 | Local |
| M3 | 03/23/2012 0:00 | 3 | 49.639240 | -110.496100 | 536382 | 5498643 | 1 | 7.95 | Local |
| M3 | 03/23/2012 0:00 | 6 | 49.639270 | -110.496200 | 536375 | 5498646 | 0 | 2126.20 | Local |

| | | | | | | | | | |
|---|---|---|---|---|---|---|---|---|---|
| M3 | 03/23/2012 0:00 | 12 | 49.631730 | -110.469140 | 538335 | 5497821 | 1 | 18.96 | Local |
| M3 | 03/23/2012 0:00 | 15 | 49.631560 | -110.469160 | 538334 | 5497802 | 1 | 19.20 | Local |
| M3 | 03/23/2012 0:00 | 18 | 49.631720 | -110.469260 | 538326 | 5497820 | 1 | 2106.81 | Local |
| M3 | 03/23/2012 0:00 | 21 | 49.639120 | -110.496120 | 536381 | 5498630 | 1 | 36.14 | Local |
| M3 | 03/24/2012 0:00 | 0 | 49.639370 | -110.495800 | 536404 | 5498658 | 1 | 25.55 | Local |
| M3 | 03/24/2012 0:00 | 3 | 49.639190 | -110.496020 | 536388 | 5498637 | 1 | 7.29 | Local |
| M3 | 03/24/2012 0:00 | 6 | 49.639230 | -110.496100 | 536382 | 5498642 | 0 | 2125.99 | Local |
| M3 | 03/24/2012 0:00 | 15 | 49.631560 | -110.469130 | 538336 | 5497802 | 0 | 2128.39 | Local |
| M3 | 03/24/2012 0:00 | 21 | 49.639600 | -110.495880 | 536398 | 5498683 | 1 | 36.94 | Local |
| M3 | 03/25/2012 0:00 | 0 | 49.639300 | -110.496100 | 536382 | 5498650 | 1 | 10.03 | Local |
| M3 | 03/25/2012 0:00 | 3 | 49.639210 | -110.496110 | 536382 | 5498640 | 1 | 2.43 | Local |
| M3 | 03/25/2012 0:00 | 6 | 49.639220 | -110.496080 | 536384 | 5498641 | 1 | 2.17 | Local |
| M3 | 03/25/2012 0:00 | 9 | 49.639220 | -110.496110 | 536382 | 5498641 | 1 | 2310.10 | Local |
| M3 | 03/25/2012 0:00 | 12 | 49.632020 | -110.466100 | 538554 | 5497855 | 1 | 124.33 | Local |
| M3 | 03/25/2012 0:00 | 15 | 49.631890 | -110.464390 | 538678 | 5497842 | 1 | 12.48 | Local |
| M3 | 03/25/2012 0:00 | 18 | 49.631870 | -110.464560 | 538666 | 5497839 | 1 | 2413.96 | Local |
| M3 | 03/25/2012 0:00 | 21 | 49.639250 | -110.496000 | 536390 | 5498644 | 1 | 6.06 | Local |
| M3 | 03/26/2012 0:00 | 0 | 49.639220 | -110.496070 | 536385 | 5498641 | 1 | 3.34 | Local |
| M3 | 03/26/2012 0:00 | 3 | 49.639250 | -110.496070 | 536385 | 5498644 | 1 | 1.33 | Local |
| M3 | 03/26/2012 0:00 | 6 | 49.639240 | -110.496080 | 536384 | 5498643 | 1 | 2130.14 | Local |
| M3 | 03/26/2012 0:00 | 9 | 49.631390 | -110.469170 | 538333 | 5497784 | 1 | 144.74 | Local |
| M3 | 03/26/2012 0:00 | 12 | 49.631150 | -110.467200 | 538475 | 5497758 | 1 | 9.74 | Local |
| M3 | 03/26/2012 0:00 | 15 | 49.631110 | -110.467320 | 538467 | 5497753 | 1 | 2.17 | Local |
| M3 | 03/26/2012 0:00 | 18 | 49.631110 | -110.467290 | 538469 | 5497753 | 1 | 1317.96 | Local |
| M3 | 03/26/2012 0:00 | 21 | 49.636730 | -110.483360 | 537304 | 5498370 | 1 | 958.59 | Local |
| M3 | 03/27/2012 0:00 | 0 | 49.638970 | -110.496180 | 536377 | 5498613 | 1 | 30.57 | Local |
| M3 | 03/27/2012 0:00 | 3 | 49.639240 | -110.496100 | 536382 | 5498643 | 1 | 2.22 | Local |
| M3 | 03/27/2012 0:00 | 6 | 49.639220 | -110.496100 | 536382 | 5498641 | 0 | 2122.21 | Local |
| M3 | 03/27/2012 0:00 | 12 | 49.631650 | -110.469120 | 538336 | 5497812 | 1 | 385.26 | Local |
| M3 | 03/27/2012 0:00 | 15 | 49.631800 | -110.463790 | 538721 | 5497832 | 1 | 16.04 | Local |
| M3 | 03/27/2012 0:00 | 18 | 49.631820 | -110.464010 | 538705 | 5497834 | 1 | 2467.62 | Local |
| M3 | 03/27/2012 0:00 | 21 | 49.639380 | -110.496140 | 536379 | 5498658 | 1 | 15.09 | Local |
| M3 | 03/28/2012 0:00 | 0 | 49.639250 | -110.496080 | 536384 | 5498644 | 1 | 1.33 | Local |
| M3 | 03/28/2012 0:00 | 3 | 49.639240 | -110.496070 | 536385 | 5498643 | 1 | 8.82 | Local |
| M3 | 03/28/2012 0:00 | 6 | 49.639180 | -110.495990 | 536390 | 5498636 | 1 | 2110.73 | Local |
| M3 | 03/28/2012 0:00 | 9 | 49.631660 | -110.469150 | 538334 | 5497814 | 1 | 20.79 | Local |
| M3 | 03/28/2012 0:00 | 12 | 49.631830 | -110.469030 | 538343 | 5497833 | 1 | 33.98 | Local |
| M3 | 03/28/2012 0:00 | 15 | 49.631530 | -110.469120 | 538337 | 5497799 | 1 | 18.17 | Local |
| M3 | 03/28/2012 0:00 | 18 | 49.631680 | -110.469020 | 538344 | 5497816 | 1 | 1686.28 | Local |
| M3 | 03/28/2012 0:00 | 21 | 49.631540 | -110.445670 | 540030 | 5497812 | 1 | 2687.22 | Local |
| M3 | 03/29/2012 0:00 | 0 | 49.608220 | -110.435890 | 540756 | 5495225 | 1 | 3398.18 | Local |
| M3 | 03/29/2012 0:00 | 3 | 49.579560 | -110.419550 | 541961 | 5492048 | 1 | 2905.60 | Local |
| M3 | 03/29/2012 0:00 | 6 | 49.560770 | -110.391620 | 543997 | 5489975 | 1 | 11.35 | Local |
| M3 | 03/29/2012 0:00 | 9 | 49.560740 | -110.391770 | 543986 | 5489971 | 1 | 10.23 | Local |
| M3 | 03/29/2012 0:00 | 12 | 49.560820 | -110.391700 | 543991 | 5489980 | 1 | 173.66 | Local |
| M3 | 03/29/2012 0:00 | 15 | 49.560350 | -110.389410 | 544157 | 5489929 | 1 | 4.45 | Local |
| M3 | 03/29/2012 0:00 | 18 | 49.560390 | -110.389410 | 544157 | 5489934 | 1 | 970.61 | Local |
| M3 | 03/29/2012 0:00 | 21 | 49.553120 | -110.381980 | 544701 | 5489130 | 1 | 3654.73 | Local |
| M3 | 03/30/2012 0:00 | 0 | 49.520450 | -110.387580 | 544325 | 5485495 | 1 | 3055.02 | Local |
| M3 | 03/30/2012 0:00 | 3 | 49.511920 | -110.427700 | 541429 | 5484523 | 1 | 963.17 | Local |
| M3 | 03/30/2012 0:00 | 6 | 49.520580 | -110.428070 | 541395 | 5485486 | 1 | 2529.59 | Local |
| M3 | 03/30/2012 0:00 | 9 | 49.518630 | -110.393250 | 543916 | 5485289 | 1 | 5.53 | Local |
| M3 | 03/30/2012 0:00 | 12 | 49.518650 | -110.393320 | 543911 | 5485291 | 1 | 1.33 | Local |
| M3 | 03/30/2012 0:00 | 15 | 49.518660 | -110.393310 | 543912 | 5485292 | 1 | 6.71 | Local |
| M3 | 03/30/2012 0:00 | 18 | 49.518720 | -110.393300 | 543913 | 5485299 | 1 | 1240.76 | Local |

| | | | | | | | | | |
|---|---|---|---|---|---|---|---|---|---|
| M3 | 03/30/2012 0:00 | 21 | 49.529880 | -110.393210 | 543909 | 5486540 | 1 | 552.36 | Local |
| M3 | 03/31/2012 0:00 | 0 | 49.534020 | -110.397430 | 543600 | 5486997 | 1 | 1495.62 | Local |
| M3 | 03/31/2012 0:00 | 3 | 49.542640 | -110.413300 | 542444 | 5487947 | 1 | 1539.26 | Local |
| M3 | 03/31/2012 0:00 | 6 | 49.554460 | -110.402220 | 543236 | 5489267 | 1 | 1061.25 | Local |
| M3 | 03/31/2012 0:00 | 9 | 49.562770 | -110.395000 | 543750 | 5490195 | 0 | 409.04 | Local |
| M3 | 03/31/2012 0:00 | 21 | 49.564920 | -110.390410 | 544080 | 5490437 | 1 | 3009.74 | Local |
| M3 | 04/01/2012 0:00 | 0 | 49.572520 | -110.430360 | 541185 | 5491259 | 1 | 3943.36 | Local |
| M3 | 04/01/2012 0:00 | 3 | 49.540590 | -110.454100 | 539495 | 5487697 | 1 | 885.07 | Local |
| M3 | 04/01/2012 0:00 | 6 | 49.533360 | -110.459220 | 539130 | 5486890 | 1 | 1258.48 | Local |
| M3 | 04/01/2012 0:00 | 9 | 49.544180 | -110.454110 | 539491 | 5488096 | 1 | 3.65 | Local |
| M3 | 04/01/2012 0:00 | 12 | 49.544200 | -110.454150 | 539488 | 5488098 | 1 | 2.34 | Local |
| M3 | 04/01/2012 0:00 | 15 | 49.544180 | -110.454160 | 539487 | 5488096 | 1 | 7.57 | Local |
| M3 | 04/01/2012 0:00 | 18 | 49.544160 | -110.454060 | 539495 | 5488094 | 1 | 6.27 | Local |
| M3 | 04/01/2012 0:00 | 21 | 49.544210 | -110.454100 | 539492 | 5488099 | 1 | 521.15 | Local |
| M3 | 04/02/2012 0:00 | 0 | 49.539560 | -110.453190 | 539561 | 5487583 | 1 | 659.91 | Local |
| M3 | 04/02/2012 0:00 | 3 | 49.543770 | -110.446760 | 540023 | 5488054 | 1 | 1879.08 | Local |
| M3 | 04/02/2012 0:00 | 6 | 49.550360 | -110.422840 | 541748 | 5488800 | 1 | 2902.04 | Local |
| M3 | 04/02/2012 0:00 | 9 | 49.575110 | -110.410090 | 542648 | 5491558 | 1 | 52.79 | Local |
| M3 | 04/02/2012 0:00 | 12 | 49.575350 | -110.409460 | 542694 | 5491585 | 0 | 7.30 | Local |
| M3 | 04/02/2012 0:00 | 18 | 49.575390 | -110.409540 | 542688 | 5491590 | 1 | 532.69 | Local |
| M3 | 04/02/2012 0:00 | 21 | 49.579920 | -110.407140 | 542857 | 5492095 | 1 | 3143.54 | Local |
| M3 | 04/03/2012 0:00 | 0 | 49.600090 | -110.437620 | 540637 | 5494320 | 1 | 4039.22 | Local |
| M3 | 04/03/2012 0:00 | 3 | 49.635080 | -110.452670 | 539522 | 5498202 | 1 | 4744.93 | Local |
| M3 | 04/03/2012 0:00 | 6 | 49.640710 | -110.387530 | 544220 | 5498865 | 1 | 809.43 | Local |
| M3 | 04/03/2012 0:00 | 9 | 49.633590 | -110.389870 | 544058 | 5498072 | 1 | 6.71 | Local |
| M3 | 04/03/2012 0:00 | 12 | 49.633650 | -110.389880 | 544057 | 5498078 | 1 | 3.78 | Local |
| M3 | 04/03/2012 0:00 | 15 | 49.633660 | -110.389830 | 544060 | 5498079 | 1 | 159.41 | Local |
| M3 | 04/03/2012 0:00 | 18 | 49.634490 | -110.391630 | 543930 | 5498171 | 1 | 878.32 | Local |
| M3 | 04/03/2012 0:00 | 21 | 49.626590 | -110.391650 | 543935 | 5497292 | 1 | 1399.18 | Local |
| M3 | 04/04/2012 0:00 | 0 | 49.614020 | -110.392590 | 543879 | 5495894 | 1 | 226.42 | Local |
| M3 | 04/04/2012 0:00 | 3 | 49.611990 | -110.392840 | 543862 | 5495668 | 1 | 166.26 | Local |
| M3 | 04/04/2012 0:00 | 6 | 49.613470 | -110.392510 | 543885 | 5495833 | 1 | 48.29 | Local |
| M3 | 04/04/2012 0:00 | 9 | 49.613840 | -110.392860 | 543859 | 5495874 | 1 | 9.35 | Local |
| M3 | 04/04/2012 0:00 | 12 | 49.613920 | -110.392820 | 543862 | 5495883 | 1 | 32.89 | Local |
| M3 | 04/04/2012 0:00 | 15 | 49.613630 | -110.392910 | 543856 | 5495851 | 0 | 42.93 | Local |
| M3 | 04/04/2012 0:00 | 21 | 49.613260 | -110.393080 | 543844 | 5495810 | 0 | 71.11 | Local |
| M3 | 04/05/2012 0:00 | 3 | 49.613850 | -110.392700 | 543871 | 5495875 | 1 | 140.14 | Local |
| M3 | 04/05/2012 0:00 | 6 | 49.612730 | -110.391810 | 543936 | 5495751 | 1 | 178.74 | Local |
| M3 | 04/05/2012 0:00 | 9 | 49.614150 | -110.392970 | 543851 | 5495909 | 1 | 37.88 | Local |
| M3 | 04/05/2012 0:00 | 12 | 49.613830 | -110.392790 | 543864 | 5495873 | 1 | 55.11 | Local |
| M3 | 04/05/2012 0:00 | 15 | 49.613380 | -110.392470 | 543888 | 5495823 | 1 | 54.00 | Local |
| M3 | 04/05/2012 0:00 | 18 | 49.613840 | -110.392710 | 543870 | 5495874 | 1 | 7.95 | Local |
| M3 | 04/05/2012 0:00 | 21 | 49.613840 | -110.392820 | 543862 | 5495874 | 0 | 3.11 | Local |
| M3 | 04/06/2012 0:00 | 3 | 49.613860 | -110.392790 | 543864 | 5495876 | 1 | 3.10 | Local |
| M3 | 04/06/2012 0:00 | 6 | 49.613870 | -110.392750 | 543867 | 5495878 | 1 | 18.70 | Local |
| M3 | 04/06/2012 0:00 | 9 | 49.613710 | -110.392830 | 543862 | 5495860 | 1 | 10.64 | Local |
| M3 | 04/06/2012 0:00 | 12 | 49.613620 | -110.392780 | 543865 | 5495850 | 1 | 23.45 | Local |
| M3 | 04/06/2012 0:00 | 15 | 49.613830 | -110.392750 | 543867 | 5495873 | 1 | 1.33 | Local |
| M3 | 04/06/2012 0:00 | 18 | 49.613820 | -110.392740 | 543868 | 5495872 | 1 | 6.63 | Local |
| M3 | 04/06/2012 0:00 | 21 | 49.613770 | -110.392690 | 543872 | 5495866 | 0 | 6.06 | Local |
| M3 | 04/07/2012 0:00 | 3 | 49.613800 | -110.392760 | 543867 | 5495870 | 1 | 2.22 | Local |
| M3 | 04/07/2012 0:00 | 6 | 49.613820 | -110.392760 | 543867 | 5495872 | 0 | 10.42 | Local |
| M3 | 04/07/2012 0:00 | 12 | 49.613730 | -110.392800 | 543864 | 5495862 | 1 | 16.32 | Local |
| M3 | 04/07/2012 0:00 | 15 | 49.613850 | -110.392670 | 543873 | 5495875 | 1 | 12.12 | Local |
| M3 | 04/07/2012 0:00 | 18 | 49.613910 | -110.392810 | 543863 | 5495882 | 1 | 10.61 | Local |

| | | | | | | | | | |
|---|---|---|---|---|---|---|---|---|---|
| M3 | 04/07/2012 0:00 | 21 | 49.613830 | -110.392730 | 543869 | 5495873 | 1 | 3.34 | Local |
| M3 | 04/08/2012 0:00 | 0 | 49.613800 | -110.392730 | 543869 | 5495870 | 1 | 32.32 | Local |
| M3 | 04/08/2012 0:00 | 3 | 49.614060 | -110.392530 | 543883 | 5495899 | 1 | 33.55 | Local |
| M3 | 04/08/2012 0:00 | 6 | 49.613810 | -110.392790 | 543864 | 5495871 | 0 | 2.22 | Local |
| M3 | 04/08/2012 0:00 | 12 | 49.613830 | -110.392790 | 543864 | 5495873 | 1 | 3.10 | Local |
| M3 | 04/08/2012 0:00 | 15 | 49.613840 | -110.392750 | 543867 | 5495874 | 0 | 21.83 | Local |
| M3 | 04/08/2012 0:00 | 21 | 49.613940 | -110.392490 | 543886 | 5495885 | 1 | 19.49 | Local |
| M3 | 04/09/2012 0:00 | 0 | 49.613860 | -110.392730 | 543869 | 5495876 | 1 | 21.24 | Local |
| M3 | 04/09/2012 0:00 | 3 | 49.613670 | -110.392760 | 543867 | 5495855 | 1 | 50.66 | Local |
| M3 | 04/09/2012 0:00 | 6 | 49.614120 | -110.392870 | 543858 | 5495905 | 1 | 18.21 | Local |
| M3 | 04/09/2012 0:00 | 9 | 49.614070 | -110.392630 | 543876 | 5495900 | 1 | 16.74 | Local |
| M3 | 04/09/2012 0:00 | 12 | 49.613950 | -110.392770 | 543866 | 5495886 | 0 | 29.24 | Local |
| M3 | 04/09/2012 0:00 | 18 | 49.613990 | -110.392370 | 543895 | 5495891 | 1 | 38.18 | Local |
| M3 | 04/09/2012 0:00 | 21 | 49.614080 | -110.392880 | 543858 | 5495901 | 1 | 31.86 | Local |
| M3 | 04/10/2012 0:00 | 0 | 49.614060 | -110.392440 | 543889 | 5495899 | 1 | 4.68 | Local |
| M3 | 04/10/2012 0:00 | 3 | 49.614020 | -110.392420 | 543891 | 5495894 | 1 | 392.96 | Local |
| M3 | 04/10/2012 0:00 | 6 | 49.610530 | -110.393280 | 543832 | 5495506 | 0 | 382.67 | Local |
| M3 | 04/10/2012 0:00 | 12 | 49.613940 | -110.392560 | 543881 | 5495885 | 1 | 20.26 | Local |
| M3 | 04/10/2012 0:00 | 15 | 49.613950 | -110.392280 | 543901 | 5495887 | 1 | 15.81 | Local |
| M3 | 04/10/2012 0:00 | 18 | 49.613990 | -110.392490 | 543886 | 5495891 | 1 | 10.65 | Local |
| M3 | 04/10/2012 0:00 | 21 | 49.614020 | -110.392630 | 543876 | 5495894 | 1 | 2782.64 | Local |
| M3 | 04/11/2012 0:00 | 0 | 49.639030 | -110.394100 | 543747 | 5498674 | 1 | 1499.84 | Local |
| M3 | 04/11/2012 0:00 | 3 | 49.652520 | -110.393990 | 543743 | 5500174 | 1 | 2773.46 | Local |
| M3 | 04/11/2012 0:00 | 6 | 49.627790 | -110.388950 | 544129 | 5497427 | 1 | 1557.52 | Local |
| M3 | 04/11/2012 0:00 | 9 | 49.613970 | -110.392480 | 543887 | 5495889 | 1 | 7.05 | Local |
| M3 | 04/11/2012 0:00 | 12 | 49.614020 | -110.392420 | 543891 | 5495894 | 1 | 14.23 | Local |
| M3 | 04/11/2012 0:00 | 15 | 49.613930 | -110.392280 | 543901 | 5495884 | 1 | 1.33 | Local |
| M3 | 04/11/2012 0:00 | 18 | 49.613920 | -110.392290 | 543900 | 5495883 | 1 | 23.08 | Local |
| M3 | 04/11/2012 0:00 | 21 | 49.614020 | -110.392570 | 543880 | 5495894 | 1 | 3585.27 | Local |
| M3 | 04/12/2012 0:00 | 0 | 49.619230 | -110.441550 | 540338 | 5496446 | 1 | 2168.19 | Local |
| M3 | 04/12/2012 0:00 | 3 | 49.638130 | -110.448950 | 539788 | 5498543 | 1 | 574.46 | Local |
| M3 | 04/12/2012 0:00 | 6 | 49.639180 | -110.456740 | 539224 | 5498656 | 1 | 885.06 | Local |
| M3 | 04/12/2012 0:00 | 9 | 49.631280 | -110.458250 | 539122 | 5497777 | 0 | 1224.95 | Local |
| M3 | 04/12/2012 0:00 | 15 | 49.621440 | -110.450620 | 539681 | 5496687 | 1 | 1042.33 | Local |
| M3 | 04/12/2012 0:00 | 18 | 49.617200 | -110.437750 | 540614 | 5496222 | 1 | 1372.37 | Local |
| M3 | 04/12/2012 0:00 | 21 | 49.626190 | -110.424730 | 541547 | 5497229 | 1 | 3175.44 | Local |
| M3 | 04/13/2012 0:00 | 0 | 49.654600 | -110.420210 | 541849 | 5500390 | 1 | 3709.11 | Local |
| M3 | 04/13/2012 0:00 | 3 | 49.646360 | -110.370420 | 545450 | 5499503 | 1 | 2310.57 | Local |
| M3 | 04/13/2012 0:00 | 6 | 49.627330 | -110.383280 | 544539 | 5497380 | 1 | 83.05 | Local |
| M3 | 04/13/2012 0:00 | 9 | 49.627710 | -110.384270 | 544467 | 5497421 | 1 | 1492.91 | Local |
| M3 | 04/13/2012 0:00 | 12 | 49.614940 | -110.390660 | 544017 | 5495998 | 0 | 906.16 | Local |
| M3 | 04/13/2012 0:00 | 21 | 49.607440 | -110.385750 | 544379 | 5495167 | 1 | 3768.19 | Local |
| M3 | 04/14/2012 0:00 | 0 | 49.573870 | -110.392930 | 543890 | 5491430 | 1 | 4598.29 | Local |
| M3 | 04/14/2012 0:00 | 3 | 49.533940 | -110.409500 | 542727 | 5486982 | 1 | 2678.90 | Local |
| M3 | 04/14/2012 0:00 | 6 | 49.552350 | -110.385610 | 544439 | 5489042 | 1 | 757.16 | Local |
| M3 | 04/14/2012 0:00 | 9 | 49.558890 | -110.388530 | 544222 | 5489768 | 1 | 14.76 | Local |
| M3 | 04/14/2012 0:00 | 12 | 49.558800 | -110.388380 | 544233 | 5489758 | 1 | 9.98 | Local |
| M3 | 04/14/2012 0:00 | 15 | 49.558770 | -110.388510 | 544223 | 5489754 | 1 | 4.68 | Local |
| M3 | 04/14/2012 0:00 | 18 | 49.558810 | -110.388490 | 544225 | 5489759 | 1 | 2556.64 | Local |
| M3 | 04/14/2012 0:00 | 21 | 49.570820 | -110.418640 | 542034 | 5491077 | 1 | 5393.52 | Local |
| M3 | 04/15/2012 0:00 | 0 | 49.535730 | -110.470130 | 538339 | 5487148 | 1 | 2892.67 | Local |
| M3 | 04/15/2012 0:00 | 3 | 49.525850 | -110.433150 | 541023 | 5486069 | 1 | 2867.71 | Local |
| M3 | 04/15/2012 0:00 | 6 | 49.525440 | -110.393530 | 543890 | 5486046 | 1 | 98.80 | Local |
| M3 | 04/15/2012 0:00 | 9 | 49.525270 | -110.394870 | 543793 | 5486026 | 1 | 3.79 | Local |
| M3 | 04/15/2012 0:00 | 12 | 49.525260 | -110.394820 | 543797 | 5486025 | 1 | 8.89 | Local |

| | | | | | | | | | |
|---|---|---|---|---|---|---|---|---|---|
| M3 | 04/15/2012 0:00 | 15 | 49.525340 | -110.394820 | 543797 | 5486034 | 1 | 12.75 | Local |
| M3 | 04/15/2012 0:00 | 18 | 49.525230 | -110.394870 | 543793 | 5486022 | 1 | 983.58 | Local |
| M3 | 04/15/2012 0:00 | 21 | 49.531950 | -110.403710 | 543148 | 5486764 | 1 | 4320.84 | Local |
| M3 | 04/16/2012 0:00 | 0 | 49.511120 | -110.454110 | 539518 | 5484420 | 1 | 5118.98 | Local |
| M3 | 04/16/2012 0:00 | 3 | 49.477710 | -110.502750 | 536021 | 5480682 | 1 | 1751.15 | Local |
| M3 | 04/16/2012 0:00 | 6 | 49.488460 | -110.485080 | 537293 | 5481885 | 0 | 7362.24 | Local |
| M3 | 04/16/2012 0:00 | 12 | 49.532100 | -110.408590 | 542794 | 5486778 | 0 | 3.10 | Local |
| M3 | 04/16/2012 0:00 | 18 | 49.532110 | -110.408630 | 542792 | 5486779 | 1 | 464.27 | Local |
| M3 | 04/16/2012 0:00 | 21 | 49.533300 | -110.402480 | 543235 | 5486914 | 1 | 3332.85 | Local |
| M3 | 04/17/2012 0:00 | 0 | 49.553940 | -110.369070 | 545634 | 5489229 | 1 | 4797.81 | Local |
| M3 | 04/17/2012 0:00 | 3 | 49.588990 | -110.407780 | 542803 | 5493103 | 1 | 3904.66 | Local |
| M3 | 04/17/2012 0:00 | 6 | 49.614810 | -110.444410 | 540135 | 5495953 | 1 | 828.51 | Local |
| M3 | 04/17/2012 0:00 | 9 | 49.621360 | -110.449880 | 539734 | 5496678 | 1 | 656.55 | Local |
| M3 | 04/17/2012 0:00 | 12 | 49.625880 | -110.455730 | 539308 | 5497178 | 1 | 1302.33 | Local |
| M3 | 04/17/2012 0:00 | 15 | 49.637520 | -110.453710 | 539444 | 5498473 | 1 | 10.62 | Local |
| M3 | 04/17/2012 0:00 | 18 | 49.637450 | -110.453810 | 539437 | 5498465 | 1 | 1282.58 | Local |
| M3 | 04/17/2012 0:00 | 21 | 49.646760 | -110.464300 | 538673 | 5499495 | 1 | 1550.84 | Local |
| M3 | 04/18/2012 0:00 | 0 | 49.655170 | -110.481440 | 537429 | 5500421 | 1 | 3079.37 | Local |
| M3 | 04/18/2012 0:00 | 3 | 49.638740 | -110.515780 | 534962 | 5498578 | 1 | 3315.19 | Local |
| M3 | 04/18/2012 0:00 | 6 | 49.628720 | -110.472540 | 538092 | 5497485 | 1 | 984.15 | Local |
| M3 | 04/18/2012 0:00 | 9 | 49.631980 | -110.459870 | 539004 | 5497854 | 1 | 339.26 | Local |
| M3 | 04/18/2012 0:00 | 12 | 49.630600 | -110.455680 | 539308 | 5497703 | 1 | 218.69 | Local |
| M3 | 04/18/2012 0:00 | 15 | 49.631300 | -110.458510 | 539103 | 5497779 | 1 | 7.22 | Local |
| M3 | 04/18/2012 0:00 | 18 | 49.631300 | -110.458410 | 539110 | 5497779 | 1 | 1684.10 | Local |
| M3 | 04/18/2012 0:00 | 21 | 49.632300 | -110.481680 | 537429 | 5497878 | 1 | 3479.65 | Local |
| M3 | 04/19/2012 0:00 | 0 | 49.607260 | -110.452780 | 539536 | 5495109 | 1 | 3256.80 | Local |
| M3 | 04/19/2012 0:00 | 3 | 49.592400 | -110.413940 | 542355 | 5493478 | 1 | 1128.06 | Local |
| M3 | 04/19/2012 0:00 | 6 | 49.583230 | -110.407260 | 542846 | 5492463 | 0 | 160.31 | Local |
| M3 | 04/19/2012 0:00 | 12 | 49.582680 | -110.409310 | 542698 | 5492400 | 1 | 6.83 | Local |
| M3 | 04/19/2012 0:00 | 15 | 49.582740 | -110.409290 | 542700 | 5492407 | 1 | 0.00 | Local |
| M3 | 04/19/2012 0:00 | 18 | 49.582740 | -110.409290 | 542700 | 5492407 | 1 | 1733.80 | Local |
| M3 | 04/19/2012 0:00 | 21 | 49.571380 | -110.392860 | 543897 | 5491154 | 1 | 2122.86 | Local |
| M3 | 04/20/2012 0:00 | 0 | 49.553160 | -110.384080 | 544549 | 5489133 | 1 | 3079.36 | Local |
| M3 | 04/20/2012 0:00 | 3 | 49.579070 | -110.399130 | 543437 | 5492005 | 1 | 1890.25 | Local |
| M3 | 04/20/2012 0:00 | 6 | 49.562200 | -110.395880 | 543687 | 5490131 | 1 | 479.79 | Local |
| M3 | 04/20/2012 0:00 | 9 | 49.565430 | -110.391480 | 544003 | 5490493 | 1 | 364.46 | Local |
| M3 | 04/20/2012 0:00 | 12 | 49.562530 | -110.389130 | 544175 | 5490172 | 0 | 10.37 | Local |
| M3 | 04/20/2012 0:00 | 18 | 49.562550 | -110.389270 | 544165 | 5490174 | 1 | 534.19 | Local |
| M3 | 04/20/2012 0:00 | 21 | 49.566390 | -110.393710 | 543840 | 5490598 | 1 | 3852.75 | Local |
| M3 | 04/21/2012 0:00 | 0 | 49.594680 | -110.424490 | 541591 | 5493726 | 0 | 3464.74 | Local |
| M3 | 04/21/2012 0:00 | 6 | 49.620160 | -110.396880 | 543563 | 5496574 | 1 | 602.89 | Local |
| M3 | 04/21/2012 0:00 | 9 | 49.624850 | -110.392690 | 543862 | 5497098 | 1 | 704.96 | Local |
| M3 | 04/21/2012 0:00 | 12 | 49.631160 | -110.391730 | 543925 | 5497800 | 1 | 2.34 | Local |
| M3 | 04/21/2012 0:00 | 15 | 49.631140 | -110.391720 | 543926 | 5497798 | 1 | 834.69 | Local |
| M3 | 04/21/2012 0:00 | 18 | 49.623720 | -110.393480 | 543806 | 5496972 | 1 | 731.09 | Local |
| M3 | 04/21/2012 0:00 | 21 | 49.627620 | -110.385330 | 544391 | 5497411 | 1 | 4816.35 | Local |
| M3 | 04/22/2012 0:00 | 0 | 49.637850 | -110.450140 | 539702 | 5498512 | 1 | 408.35 | Local |
| M3 | 04/22/2012 0:00 | 3 | 49.635660 | -110.454680 | 539376 | 5498266 | 1 | 1067.42 | Local |
| M3 | 04/22/2012 0:00 | 6 | 49.632840 | -110.468810 | 538358 | 5497945 | 1 | 649.99 | Local |
| M3 | 04/22/2012 0:00 | 9 | 49.629420 | -110.461510 | 538888 | 5497568 | 1 | 18.18 | Local |
| M3 | 04/22/2012 0:00 | 12 | 49.629560 | -110.461640 | 538878 | 5497584 | 1 | 5.06 | Local |
| M3 | 04/22/2012 0:00 | 15 | 49.629560 | -110.461710 | 538873 | 5497584 | 0 | 424.27 | Local |
| M3 | 04/22/2012 0:00 | 21 | 49.631950 | -110.457130 | 539202 | 5497852 | 1 | 480.40 | Local |
| M3 | 04/23/2012 0:00 | 0 | 49.635460 | -110.461010 | 538919 | 5498240 | 1 | 2088.80 | Local |
| M3 | 04/23/2012 0:00 | 3 | 49.628080 | -110.434410 | 540846 | 5497434 | 1 | 505.98 | Local |

| | | | | | | | | | |
|---|---|---|---|---|---|---|---|---|---|
| M3 | 04/23/2012 0:00 | 6 | 49.624240 | -110.438170 | 540578 | 5497005 | 0 | 755.52 | Local |
| M3 | 04/23/2012 0:00 | 15 | 49.623510 | -110.448570 | 539827 | 5496918 | 0 | 1125.97 | Local |
| M3 | 04/23/2012 0:00 | 21 | 49.632360 | -110.456150 | 539272 | 5497898 | 1 | 1224.68 | Local |
| M3 | 04/24/2012 0:00 | 0 | 49.638190 | -110.441760 | 540307 | 5498554 | 1 | 902.22 | Local |
| M3 | 04/24/2012 0:00 | 3 | 49.630100 | -110.442740 | 540243 | 5497654 | 1 | 418.05 | Local |
| M3 | 04/24/2012 0:00 | 6 | 49.633060 | -110.446310 | 539982 | 5497981 | 1 | 739.21 | Local |
| M3 | 04/24/2012 0:00 | 9 | 49.633300 | -110.456540 | 539244 | 5498002 | 1 | 152.95 | Local |
| M3 | 04/24/2012 0:00 | 12 | 49.634420 | -110.457770 | 539154 | 5498126 | 1 | 926.21 | Local |
| M3 | 04/24/2012 0:00 | 15 | 49.631770 | -110.469930 | 538278 | 5497825 | 1 | 275.74 | Local |
| M3 | 04/24/2012 0:00 | 18 | 49.630580 | -110.473280 | 538037 | 5497691 | 1 | 91.90 | Local |
| M3 | 04/24/2012 0:00 | 21 | 49.631130 | -110.474230 | 537968 | 5497752 | 1 | 2522.69 | Local |
| M3 | 04/25/2012 0:00 | 0 | 49.640250 | -110.506220 | 535651 | 5498750 | 1 | 2925.75 | Local |
| M3 | 04/25/2012 0:00 | 3 | 49.653770 | -110.540990 | 533131 | 5500238 | 1 | 966.51 | Local |
| M3 | 04/25/2012 0:00 | 6 | 49.662450 | -110.540250 | 533179 | 5501203 | 1 | 3596.27 | Local |
| M3 | 04/25/2012 0:00 | 9 | 49.641470 | -110.502330 | 535931 | 5498888 | 1 | 3.61 | Local |
| M3 | 04/25/2012 0:00 | 12 | 49.641470 | -110.502280 | 535935 | 5498888 | 1 | 2.17 | Local |
| M3 | 04/25/2012 0:00 | 15 | 49.641470 | -110.502310 | 535932 | 5498888 | 1 | 3.63 | Local |
| M3 | 04/25/2012 0:00 | 18 | 49.641440 | -110.502290 | 535934 | 5498885 | 1 | 21.92 | Local |
| M3 | 04/25/2012 0:00 | 21 | 49.641470 | -110.502590 | 535912 | 5498888 | 1 | 1683.23 | Local |
| M3 | 04/26/2012 0:00 | 0 | 49.634570 | -110.481840 | 537416 | 5498131 | 1 | 657.16 | Local |
| M3 | 04/26/2012 0:00 | 3 | 49.631940 | -110.473690 | 538006 | 5497842 | 1 | 34.29 | Local |
| M3 | 04/26/2012 0:00 | 6 | 49.631640 | -110.473800 | 537998 | 5497809 | 1 | 238.27 | Local |
| M3 | 04/26/2012 0:00 | 9 | 49.630720 | -110.470820 | 538214 | 5497708 | 1 | 14.61 | Local |
| M3 | 04/26/2012 0:00 | 12 | 49.630850 | -110.470850 | 538212 | 5497723 | 1 | 21.78 | Local |
| M3 | 04/26/2012 0:00 | 15 | 49.630870 | -110.470550 | 538234 | 5497725 | 1 | 78.06 | Local |
| M3 | 04/26/2012 0:00 | 18 | 49.630900 | -110.469470 | 538312 | 5497729 | 1 | 17.06 | Local |
| M3 | 04/26/2012 0:00 | 21 | 49.630750 | -110.469420 | 538315 | 5497712 | 1 | 5148.85 | Local |
| M3 | 04/27/2012 0:00 | 0 | 49.591400 | -110.507000 | 535630 | 5493319 | 1 | 6134.89 | Local |
| M3 | 04/27/2012 0:00 | 3 | 49.541960 | -110.469320 | 538392 | 5487841 | 1 | 3828.41 | Local |
| M3 | 04/27/2012 0:00 | 6 | 49.519400 | -110.429350 | 541303 | 5485354 | 1 | 582.59 | Local |
| M3 | 04/27/2012 0:00 | 9 | 49.514160 | -110.429410 | 541303 | 5484771 | 1 | 15.80 | Local |
| M3 | 04/27/2012 0:00 | 12 | 49.514230 | -110.429220 | 541317 | 5484779 | 1 | 6.27 | Local |
| M3 | 04/27/2012 0:00 | 15 | 49.514280 | -110.429260 | 541314 | 5484785 | 1 | 98.83 | Local |
| M3 | 04/27/2012 0:00 | 18 | 49.513410 | -110.429540 | 541294 | 5484688 | 1 | 222.01 | Local |
| M3 | 04/27/2012 0:00 | 21 | 49.514320 | -110.426810 | 541491 | 5484791 | 1 | 3002.78 | Local |
| M3 | 04/28/2012 0:00 | 0 | 49.490620 | -110.446700 | 540071 | 5482145 | 1 | 73.85 | Local |
| M3 | 04/28/2012 0:00 | 3 | 49.491040 | -110.445910 | 540128 | 5482192 | 1 | 4.25 | Local |
| M3 | 04/28/2012 0:00 | 6 | 49.491060 | -110.445960 | 540124 | 5482194 | 1 | 3.62 | Local |
| M3 | 04/28/2012 0:00 | 9 | 49.491060 | -110.445910 | 540128 | 5482194 | 1 | 7.92 | Local |
| M3 | 04/28/2012 0:00 | 12 | 49.490990 | -110.445890 | 540129 | 5482187 | 1 | 8.38 | Local |
| M3 | 04/28/2012 0:00 | 15 | 49.491050 | -110.445960 | 540124 | 5482193 | 1 | 4.68 | Local |
| M3 | 04/28/2012 0:00 | 18 | 49.491010 | -110.445980 | 540122 | 5482189 | 1 | 150.69 | Local |
| M3 | 04/28/2012 0:00 | 21 | 49.491500 | -110.447920 | 539982 | 5482242 | 1 | 2149.58 | Local |
| M3 | 04/29/2012 0:00 | 0 | 49.510820 | -110.446760 | 540050 | 5484391 | 1 | 3837.05 | Local |
| M3 | 04/29/2012 0:00 | 3 | 49.539510 | -110.417290 | 542159 | 5487596 | 1 | 3343.16 | Local |
| M3 | 04/29/2012 0:00 | 6 | 49.563850 | -110.390150 | 544100 | 5490318 | 1 | 12.42 | Local |
| M3 | 04/29/2012 0:00 | 9 | 49.563740 | -110.390120 | 544102 | 5490306 | 1 | 1194.00 | Local |
| M3 | 04/29/2012 0:00 | 12 | 49.573640 | -110.396520 | 543631 | 5491403 | 1 | 70.18 | Local |
| M3 | 04/29/2012 0:00 | 15 | 49.573360 | -110.395650 | 543694 | 5491372 | 1 | 1.11 | Local |
| M3 | 04/29/2012 0:00 | 18 | 49.573370 | -110.395650 | 543694 | 5491373 | 0 | 5541.82 | Local |
| M3 | 04/30/2012 0:00 | 0 | 49.606330 | -110.453170 | 539509 | 5495006 | 1 | 6657.63 | Local |
| M3 | 04/30/2012 0:00 | 3 | 49.646090 | -110.522100 | 534500 | 5499392 | 1 | 810.18 | Local |
| M3 | 04/30/2012 0:00 | 6 | 49.644110 | -110.511300 | 535281 | 5499177 | 1 | 2590.31 | Local |
| M3 | 04/30/2012 0:00 | 9 | 49.632610 | -110.480100 | 537543 | 5497914 | 1 | 37.81 | Local |
| M3 | 04/30/2012 0:00 | 12 | 49.632650 | -110.479580 | 537580 | 5497918 | 1 | 5.30 | Local |

| | | | | | | | | | |
|---|---|---|---|---|---|---|---|---|---|
| M3 | 04/30/2012 0:00 | 15 | 49.632610 | -110.479620 | 537577 | 5497914 | 1 | 4.68 | Local |
| M3 | 04/30/2012 0:00 | 18 | 49.632650 | -110.479640 | 537576 | 5497918 | 1 | 623.45 | Local |
| M3 | 04/30/2012 0:00 | 21 | 49.628280 | -110.474230 | 537970 | 5497435 | 1 | 8088.94 | Local |
| M3 | 05/01/2012 0:00 | 0 | 49.654730 | -110.578600 | 530416 | 5500328 | 1 | 2871.50 | Local |
| M3 | 05/01/2012 0:00 | 3 | 49.673240 | -110.606350 | 528402 | 5502375 | 1 | 853.46 | Local |
| M3 | 05/01/2012 0:00 | 6 | 49.666640 | -110.600310 | 528842 | 5501644 | 0 | 20.58 | Local |
| M3 | 05/01/2012 0:00 | 15 | 49.666740 | -110.600070 | 528859 | 5501655 | 1 | 1.11 | Local |
| M3 | 05/01/2012 0:00 | 18 | 49.666730 | -110.600070 | 528859 | 5501654 | 1 | 1160.20 | Local |
| M3 | 05/01/2012 0:00 | 21 | 49.657390 | -110.592900 | 529382 | 5500618 | 1 | 5965.60 | Local |
| M3 | 05/02/2012 0:00 | 0 | 49.646300 | -110.673760 | 523552 | 5499357 | 1 | 5025.82 | Local |
| M3 | 05/02/2012 0:00 | 3 | 49.672240 | -110.730790 | 519424 | 5502225 | 1 | 2056.55 | Local |
| M3 | 05/02/2012 0:00 | 6 | 49.686740 | -110.748490 | 518142 | 5503832 | 1 | 39.97 | Local |
| M3 | 05/02/2012 0:00 | 9 | 49.687080 | -110.748670 | 518129 | 5503870 | 1 | 1.33 | Local |
| M3 | 05/02/2012 0:00 | 12 | 49.687070 | -110.748660 | 518129 | 5503869 | 0 | 2.65 | Local |
| M3 | 05/02/2012 0:00 | 18 | 49.687050 | -110.748640 | 518131 | 5503867 | 1 | 7.95 | Local |
| M3 | 05/02/2012 0:00 | 21 | 49.687080 | -110.748540 | 518138 | 5503870 | 1 | 1081.09 | Local |
| M3 | 05/03/2012 0:00 | 0 | 49.696790 | -110.747740 | 518192 | 5504950 | 1 | 1109.20 | Local |
| M3 | 05/03/2012 0:00 | 3 | 49.704800 | -110.756910 | 517528 | 5505838 | 1 | 328.25 | Local |
| M3 | 05/03/2012 0:00 | 6 | 49.707730 | -110.756350 | 517567 | 5506164 | 1 | 23.10 | Local |
| M3 | 05/03/2012 0:00 | 9 | 49.707860 | -110.756100 | 517585 | 5506179 | 1 | 25.26 | Local |
| M3 | 05/03/2012 0:00 | 12 | 49.707850 | -110.756450 | 517560 | 5506177 | 1 | 3.61 | Local |
| M3 | 05/03/2012 0:00 | 15 | 49.707850 | -110.756500 | 517556 | 5506177 | 1 | 7.94 | Local |
| M3 | 05/03/2012 0:00 | 18 | 49.707880 | -110.756400 | 517564 | 5506181 | 1 | 419.96 | Local |
| M3 | 05/03/2012 0:00 | 21 | 49.705530 | -110.751840 | 517893 | 5505921 | 1 | 365.46 | Local |
| M3 | 05/04/2012 0:00 | 0 | 49.708790 | -110.751190 | 517939 | 5506283 | 1 | 13.33 | Local |
| M3 | 05/04/2012 0:00 | 3 | 49.708850 | -110.751350 | 517927 | 5506290 | 1 | 274.55 | Local |
| M3 | 05/04/2012 0:00 | 6 | 49.711200 | -110.752520 | 517842 | 5506551 | 0 | 23.36 | Local |
| M3 | 05/04/2012 0:00 | 12 | 49.711360 | -110.752310 | 517857 | 5506569 | 1 | 46.22 | Local |
| M3 | 05/04/2012 0:00 | 15 | 49.711030 | -110.752700 | 517829 | 5506532 | 0 | 5433.34 | Local |
| M3 | 05/05/2012 0:00 | 0 | 49.681880 | -110.692230 | 522202 | 5503307 | 1 | 8612.65 | Local |
| M3 | 05/05/2012 0:00 | 3 | 49.661910 | -110.576900 | 530534 | 5501127 | 1 | 8225.38 | Local |
| M3 | 05/05/2012 0:00 | 6 | 49.640950 | -110.467620 | 538437 | 5498847 | 0 | 1437.38 | Local |
| M3 | 05/05/2012 0:00 | 12 | 49.631000 | -110.454910 | 539363 | 5497748 | 1 | 9.60 | Local |
| M3 | 05/05/2012 0:00 | 15 | 49.630920 | -110.454960 | 539360 | 5497739 | 1 | 31.80 | Local |
| M3 | 05/05/2012 0:00 | 18 | 49.631200 | -110.454870 | 539366 | 5497770 | 1 | 78.12 | Local |
| M3 | 05/05/2012 0:00 | 21 | 49.630720 | -110.454080 | 539423 | 5497717 | 1 | 15.77 | Local |
| M3 | 05/06/2012 0:00 | 0 | 49.630790 | -110.454270 | 539409 | 5497725 | 1 | 45.27 | Local |
| M3 | 05/06/2012 0:00 | 3 | 49.630400 | -110.454450 | 539397 | 5497681 | 0 | 64.01 | Local |
| M3 | 05/06/2012 0:00 | 12 | 49.630510 | -110.455320 | 539334 | 5497693 | 1 | 15.57 | Local |
| M3 | 05/06/2012 0:00 | 15 | 49.630640 | -110.455240 | 539340 | 5497707 | 1 | 53.86 | Local |
| M3 | 05/06/2012 0:00 | 18 | 49.630960 | -110.454680 | 539380 | 5497743 | 1 | 13.36 | Local |
| M3 | 05/06/2012 0:00 | 21 | 49.630840 | -110.454670 | 539381 | 5497730 | 1 | 9.74 | Local |
| M3 | 05/07/2012 0:00 | 0 | 49.630800 | -110.454550 | 539389 | 5497726 | 1 | 39.43 | Local |
| M3 | 05/07/2012 0:00 | 3 | 49.630470 | -110.454350 | 539404 | 5497689 | 1 | 32.63 | Local |
| M3 | 05/07/2012 0:00 | 6 | 49.630710 | -110.454610 | 539385 | 5497715 | 0 | 69.47 | Local |
| M3 | 05/07/2012 0:00 | 12 | 49.630670 | -110.453650 | 539454 | 5497712 | 1 | 26.92 | Local |
| M3 | 05/07/2012 0:00 | 15 | 49.630470 | -110.453860 | 539439 | 5497689 | 1 | 1377.51 | Local |
| M3 | 05/07/2012 0:00 | 18 | 49.628230 | -110.472620 | 538086 | 5497430 | 1 | 707.72 | Local |
| M3 | 05/07/2012 0:00 | 21 | 49.626110 | -110.463380 | 538755 | 5497200 | 1 | 2721.56 | Local |
| M3 | 05/08/2012 0:00 | 0 | 49.640300 | -110.432670 | 540961 | 5498793 | 1 | 2966.50 | Local |
| M3 | 05/08/2012 0:00 | 3 | 49.613680 | -110.435470 | 540781 | 5495832 | 1 | 73.71 | Local |
| M3 | 05/08/2012 0:00 | 6 | 49.614180 | -110.434800 | 540829 | 5495888 | 0 | 4.87 | Local |
| M3 | 05/08/2012 0:00 | 12 | 49.614200 | -110.434860 | 540825 | 5495890 | 1 | 18.09 | Local |
| M3 | 05/08/2012 0:00 | 15 | 49.614210 | -110.435110 | 540807 | 5495891 | 0 | 46.84 | Local |
| M3 | 05/08/2012 0:00 | 21 | 49.614580 | -110.434800 | 540829 | 5495933 | 1 | 2612.82 | Local |

| | | | | | | | | | |
|---|---|---|---|---|---|---|---|---|---|
| M3 | 05/09/2012 0:00 | 0 | 49.599160 | -110.462090 | 538870 | 5494204 | 1 | 3190.05 | Local |
| M3 | 05/09/2012 0:00 | 3 | 49.583200 | -110.498770 | 536231 | 5492411 | 1 | 0.72 | Local |
| M3 | 05/09/2012 0:00 | 6 | 49.583200 | -110.498780 | 536230 | 5492411 | 0 | 2914.11 | Local |
| M3 | 05/09/2012 0:00 | 18 | 49.593260 | -110.461550 | 538914 | 5493548 | 1 | 129.16 | Local |
| M3 | 05/09/2012 0:00 | 21 | 49.593930 | -110.460090 | 539019 | 5493624 | 1 | 2600.03 | Local |
| M3 | 05/10/2012 0:00 | 0 | 49.616690 | -110.451820 | 539598 | 5496158 | 1 | 2685.61 | Local |
| M3 | 05/10/2012 0:00 | 3 | 49.632870 | -110.479430 | 537591 | 5497943 | 1 | 1724.59 | Local |
| M3 | 05/10/2012 0:00 | 6 | 49.624160 | -110.499190 | 536171 | 5496965 | 1 | 28.94 | Local |
| M3 | 05/10/2012 0:00 | 9 | 49.623900 | -110.499170 | 536172 | 5496936 | 1 | 2.34 | Local |
| M3 | 05/10/2012 0:00 | 12 | 49.623920 | -110.499160 | 536173 | 5496938 | 1 | 3.41 | Local |
| M3 | 05/10/2012 0:00 | 15 | 49.623890 | -110.499170 | 536172 | 5496935 | 1 | 1.33 | Local |
| M3 | 05/10/2012 0:00 | 18 | 49.623900 | -110.499180 | 536171 | 5496936 | 1 | 5.61 | Local |
| M3 | 05/10/2012 0:00 | 21 | 49.623950 | -110.499170 | 536172 | 5496942 | 1 | 3605.56 | Local |
| M3 | 05/11/2012 0:00 | 0 | 49.644370 | -110.460380 | 538957 | 5499231 | 1 | 4524.36 | Local |
| M3 | 05/11/2012 0:00 | 3 | 49.658670 | -110.519060 | 534711 | 5500792 | 1 | 2053.34 | Local |
| M3 | 05/11/2012 0:00 | 6 | 49.642290 | -110.505920 | 535671 | 5498977 | 1 | 279.15 | Local |
| M3 | 05/11/2012 0:00 | 9 | 49.641500 | -110.502250 | 535937 | 5498891 | 1 | 20.74 | Local |
| M3 | 05/11/2012 0:00 | 12 | 49.641380 | -110.502470 | 535921 | 5498878 | 0 | 159.18 | Local |
| M3 | 05/11/2012 0:00 | 18 | 49.639950 | -110.502360 | 535930 | 5498719 | 1 | 993.69 | Local |
| M3 | 05/11/2012 0:00 | 21 | 49.633410 | -110.492980 | 536612 | 5497996 | 0 | 4909.05 | Local |
| M3 | 05/12/2012 0:00 | 3 | 49.655540 | -110.551820 | 532349 | 5500430 | 1 | 3064.27 | Local |
| M3 | 05/12/2012 0:00 | 6 | 49.663120 | -110.592640 | 529398 | 5501256 | 1 | 36.15 | Local |
| M3 | 05/12/2012 0:00 | 9 | 49.663140 | -110.592140 | 529434 | 5501258 | 1 | 41.16 | Local |
| M3 | 05/12/2012 0:00 | 12 | 49.662940 | -110.592620 | 529399 | 5501236 | 1 | 21.23 | Local |
| M3 | 05/12/2012 0:00 | 15 | 49.663080 | -110.592420 | 529414 | 5501251 | 1 | 45.12 | Local |
| M3 | 05/12/2012 0:00 | 18 | 49.662820 | -110.592900 | 529379 | 5501222 | 1 | 543.38 | Local |
| M3 | 05/12/2012 0:00 | 21 | 49.667480 | -110.595170 | 529213 | 5501739 | 1 | 3507.71 | Local |
| M3 | 05/13/2012 0:00 | 0 | 49.659340 | -110.642130 | 525828 | 5500817 | 1 | 3624.00 | Local |
| M3 | 05/13/2012 0:00 | 3 | 49.659010 | -110.591920 | 529452 | 5500799 | 1 | 7828.21 | Local |
| M3 | 05/13/2012 0:00 | 6 | 49.634650 | -110.490180 | 536813 | 5498135 | 1 | 731.65 | Local |
| M3 | 05/13/2012 0:00 | 9 | 49.632860 | -110.480430 | 537519 | 5497941 | 1 | 14.10 | Local |
| M3 | 05/13/2012 0:00 | 12 | 49.632760 | -110.480550 | 537510 | 5497930 | 1 | 8.25 | Local |
| M3 | 05/13/2012 0:00 | 15 | 49.632780 | -110.480660 | 537502 | 5497932 | 1 | 10.41 | Local |
| M3 | 05/13/2012 0:00 | 18 | 49.632690 | -110.480620 | 537505 | 5497922 | 1 | 437.89 | Local |
| M3 | 05/13/2012 0:00 | 21 | 49.630950 | -110.475180 | 537899 | 5497732 | 1 | 3450.97 | Local |
| M3 | 05/14/2012 0:00 | 0 | 49.614840 | -110.434340 | 540862 | 5495962 | 1 | 5725.54 | Local |
| M3 | 05/14/2012 0:00 | 3 | 49.583330 | -110.497010 | 536358 | 5492427 | 1 | 3240.41 | Local |
| M3 | 05/14/2012 0:00 | 6 | 49.612370 | -110.500830 | 536061 | 5495653 | 1 | 621.23 | Local |
| M3 | 05/14/2012 0:00 | 9 | 49.617770 | -110.503040 | 535897 | 5496253 | 1 | 17.48 | Local |
| M3 | 05/14/2012 0:00 | 12 | 49.617790 | -110.503280 | 535880 | 5496255 | 0 | 3.10 | Local |
| M3 | 05/14/2012 0:00 | 18 | 49.617800 | -110.503320 | 535877 | 5496256 | 1 | 2166.71 | Local |
| M3 | 05/14/2012 0:00 | 21 | 49.627430 | -110.477240 | 537753 | 5497339 | 1 | 3765.43 | Local |
| M3 | 05/15/2012 0:00 | 0 | 49.658660 | -110.497420 | 536273 | 5500801 | 1 | 9098.21 | Local |
| M3 | 05/15/2012 0:00 | 3 | 49.674150 | -110.621220 | 527329 | 5502471 | 1 | 8226.11 | Local |
| M3 | 05/15/2012 0:00 | 6 | 49.690160 | -110.732550 | 519290 | 5504217 | 1 | 87.16 | Local |
| M3 | 05/15/2012 0:00 | 9 | 49.690470 | -110.733660 | 519210 | 5504251 | 0 | 918.37 | Local |
| M3 | 05/15/2012 0:00 | 15 | 49.696320 | -110.742650 | 518559 | 5504899 | 0 | 1.82 | Local |
| M3 | 05/15/2012 0:00 | 21 | 49.696330 | -110.742630 | 518561 | 5504900 | 1 | 14.12 | Local |
| M3 | 05/16/2012 0:00 | 0 | 49.696380 | -110.742450 | 518574 | 5504906 | 1 | 297.06 | Local |
| M3 | 05/16/2012 0:00 | 3 | 49.694070 | -110.740380 | 518724 | 5504649 | 1 | 1061.48 | Local |
| M3 | 05/16/2012 0:00 | 6 | 49.700280 | -110.751560 | 517915 | 5505337 | 1 | 17.23 | Local |
| M3 | 05/16/2012 0:00 | 9 | 49.700430 | -110.751620 | 517911 | 5505354 | 1 | 1.44 | Local |
| M3 | 05/16/2012 0:00 | 12 | 49.700430 | -110.751600 | 517912 | 5505354 | 1 | 6.26 | Local |
| M3 | 05/16/2012 0:00 | 15 | 49.700380 | -110.751640 | 517910 | 5505348 | 0 | 269.33 | Local |
| M3 | 05/16/2012 0:00 | 21 | 49.701640 | -110.754830 | 517679 | 5505487 | 1 | 227.39 | Local |

| | | | | | | | | | |
|---|---|---|---|---|---|---|---|---|---|
| M3 | 05/17/2012 0:00 | 0 | 49.703350 | -110.756560 | 517554 | 5505677 | 1 | 241.75 | Local |
| M3 | 05/17/2012 0:00 | 3 | 49.703830 | -110.759830 | 517318 | 5505730 | 1 | 209.42 | Local |
| M3 | 05/17/2012 0:00 | 6 | 49.705220 | -110.757870 | 517459 | 5505885 | 0 | 29.34 | Local |
| M3 | 05/17/2012 0:00 | 15 | 49.705480 | -110.757940 | 517453 | 5505914 | 1 | 3.10 | Local |
| M3 | 05/17/2012 0:00 | 18 | 49.705460 | -110.757910 | 517456 | 5505911 | 1 | 300.08 | Local |
| M3 | 05/17/2012 0:00 | 21 | 49.702840 | -110.756910 | 517529 | 5505620 | 1 | 279.97 | Local |
| M3 | 05/18/2012 0:00 | 0 | 49.700380 | -110.756080 | 517589 | 5505347 | 1 | 4804.18 | Local |
| M3 | 05/18/2012 0:00 | 3 | 49.687870 | -110.692320 | 522193 | 5503973 | 1 | 11816.64 | Local |
| M3 | 05/18/2012 0:00 | 6 | 49.649660 | -110.539510 | 533241 | 5499781 | 1 | 4838.12 | Local |
| M3 | 05/18/2012 0:00 | 9 | 49.633150 | -110.477510 | 537729 | 5497975 | 1 | 446.41 | Local |
| M3 | 05/18/2012 0:00 | 12 | 49.630180 | -110.473350 | 538032 | 5497647 | 0 | 1807.10 | Local |
| M3 | 05/18/2012 0:00 | 21 | 49.643220 | -110.458410 | 539101 | 5499104 | 1 | 1511.04 | Local |
| M3 | 05/19/2012 0:00 | 0 | 49.656800 | -110.457570 | 539150 | 5500615 | 1 | 557.75 | Local |
| M3 | 05/19/2012 0:00 | 3 | 49.653500 | -110.463390 | 538733 | 5500245 | 1 | 52.77 | Local |
| M3 | 05/19/2012 0:00 | 6 | 49.653690 | -110.464060 | 538684 | 5500265 | 0 | 394.23 | Local |
| M3 | 05/19/2012 0:00 | 12 | 49.650850 | -110.467330 | 538451 | 5499948 | 1 | 2.22 | Local |
| M3 | 05/19/2012 0:00 | 15 | 49.650830 | -110.467330 | 538451 | 5499946 | 1 | 3.78 | Local |
| M3 | 05/19/2012 0:00 | 18 | 49.650840 | -110.467280 | 538454 | 5499947 | 0 | 328.55 | Local |
| M3 | 05/20/2012 0:00 | 0 | 49.653270 | -110.464690 | 538639 | 5500218 | 1 | 70.84 | Local |
| M3 | 05/20/2012 0:00 | 3 | 49.653610 | -110.463860 | 538699 | 5500257 | 1 | 400.79 | Local |
| M3 | 05/20/2012 0:00 | 6 | 49.650760 | -110.467260 | 538456 | 5499938 | 0 | 6.83 | Local |
| M3 | 05/20/2012 0:00 | 15 | 49.650700 | -110.467240 | 538457 | 5499931 | 1 | 16.60 | Local |
| M3 | 05/20/2012 0:00 | 18 | 49.650840 | -110.467320 | 538451 | 5499947 | 1 | 24.83 | Local |
| M3 | 05/20/2012 0:00 | 21 | 49.651000 | -110.467560 | 538434 | 5499965 | 1 | 1994.77 | Local |
| M3 | 05/21/2012 0:00 | 0 | 49.633380 | -110.462350 | 538824 | 5498008 | 1 | 2667.39 | Local |
| M3 | 05/21/2012 0:00 | 3 | 49.618320 | -110.433600 | 540913 | 5496349 | 1 | 396.01 | Local |
| M3 | 05/21/2012 0:00 | 6 | 49.614760 | -110.433420 | 540929 | 5495954 | 0 | 3024.96 | Local |
| M3 | 05/21/2012 0:00 | 18 | 49.621580 | -110.473960 | 537995 | 5496690 | 1 | 2377.35 | Local |
| M3 | 05/21/2012 0:00 | 21 | 49.607040 | -110.498090 | 536263 | 5495062 | 1 | 444.25 | Local |
| M3 | 05/22/2012 0:00 | 0 | 49.604770 | -110.503150 | 535899 | 5494807 | 1 | 5.73 | Local |
| M3 | 05/22/2012 0:00 | 3 | 49.604730 | -110.503100 | 535902 | 5494803 | 1 | 7.31 | Local |
| M3 | 05/22/2012 0:00 | 6 | 49.604760 | -110.503010 | 535909 | 5494806 | 1 | 8.95 | Local |
| M3 | 05/22/2012 0:00 | 9 | 49.604780 | -110.503130 | 535900 | 5494808 | 0 | 7.59 | Local |
| M3 | 05/22/2012 0:00 | 15 | 49.604720 | -110.503180 | 535897 | 5494802 | 1 | 11.54 | Local |
| M3 | 05/22/2012 0:00 | 18 | 49.604770 | -110.503320 | 535886 | 5494807 | 1 | 632.72 | Local |
| M3 | 05/22/2012 0:00 | 21 | 49.609530 | -110.508120 | 535536 | 5495334 | 1 | 3057.23 | Local |
| M3 | 05/23/2012 0:00 | 0 | 49.631790 | -110.483270 | 537315 | 5497821 | 1 | 4482.64 | Local |
| M3 | 05/23/2012 0:00 | 3 | 49.593100 | -110.465810 | 538606 | 5493528 | 1 | 3015.88 | Local |
| M3 | 05/23/2012 0:00 | 6 | 49.571120 | -110.441360 | 540391 | 5491098 | 0 | 1445.55 | Local |
| M3 | 05/23/2012 0:00 | 12 | 49.572500 | -110.421480 | 541827 | 5491262 | 1 | 2854.72 | Local |
| M3 | 05/23/2012 0:00 | 15 | 49.559020 | -110.387880 | 544269 | 5489782 | 1 | 114.87 | Local |
| M3 | 05/23/2012 0:00 | 18 | 49.558520 | -110.389270 | 544169 | 5489726 | 1 | 702.81 | Local |
| M3 | 05/23/2012 0:00 | 21 | 49.552730 | -110.385370 | 544456 | 5489085 | 1 | 3723.90 | Local |
| M3 | 05/24/2012 0:00 | 0 | 49.519300 | -110.382170 | 544718 | 5485370 | 1 | 1208.79 | Local |
| M3 | 05/24/2012 0:00 | 3 | 49.508480 | -110.380530 | 544846 | 5484168 | 1 | 1453.53 | Local |
| M3 | 05/24/2012 0:00 | 6 | 49.518580 | -110.393280 | 543914 | 5485283 | 0 | 7.57 | Local |
| M3 | 05/24/2012 0:00 | 12 | 49.518560 | -110.393380 | 543907 | 5485281 | 1 | 53.57 | Local |
| M3 | 05/24/2012 0:00 | 15 | 49.518570 | -110.392640 | 543961 | 5485283 | 0 | 44.38 | Local |
| M3 | 05/24/2012 0:00 | 21 | 49.518610 | -110.393250 | 543917 | 5485287 | 1 | 1.33 | Local |
| M3 | 05/25/2012 0:00 | 0 | 49.518620 | -110.393260 | 543916 | 5485288 | 1 | 1.33 | Local |
| M3 | 05/25/2012 0:00 | 3 | 49.518630 | -110.393250 | 543916 | 5485289 | 1 | 5.07 | Local |
| M3 | 05/25/2012 0:00 | 6 | 49.518630 | -110.393320 | 543911 | 5485289 | 0 | 56.18 | Local |
| M3 | 05/25/2012 0:00 | 12 | 49.518500 | -110.392570 | 543966 | 5485275 | 1 | 11.22 | Local |
| M3 | 05/25/2012 0:00 | 15 | 49.518590 | -110.392640 | 543961 | 5485285 | 1 | 10.15 | Local |
| M3 | 05/25/2012 0:00 | 18 | 49.518660 | -110.392730 | 543954 | 5485293 | 1 | 35.48 | Local |

| | | | | | | | | | |
|---|---|---|---|---|---|---|---|---|---|
| M3 | 05/25/2012 0:00 | 21 | 49.518650 | -110.393220 | 543919 | 5485291 | 1 | 621.21 | Local |
| M3 | 05/26/2012 0:00 | 0 | 49.518950 | -110.384650 | 544539 | 5485330 | 0 | 639.46 | Local |
| M3 | 05/26/2012 0:00 | 6 | 49.518620 | -110.393470 | 543901 | 5485288 | 0 | 19.11 | Local |
| M3 | 05/26/2012 0:00 | 18 | 49.518650 | -110.393210 | 543919 | 5485291 | 1 | 9.35 | Local |
| M3 | 05/26/2012 0:00 | 21 | 49.518730 | -110.393250 | 543916 | 5485300 | 1 | 527.43 | Local |
| M3 | 05/27/2012 0:00 | 0 | 49.520330 | -110.386390 | 544411 | 5485482 | 1 | 4.95 | Local |
| M3 | 05/27/2012 0:00 | 3 | 49.520370 | -110.386360 | 544414 | 5485486 | 1 | 3817.04 | Local |
| M3 | 05/27/2012 0:00 | 6 | 49.554690 | -110.387790 | 544279 | 5489301 | 1 | 335.27 | Local |
| M3 | 05/27/2012 0:00 | 9 | 49.557270 | -110.390190 | 544103 | 5489586 | 0 | 5.53 | Local |
| M3 | 05/27/2012 0:00 | 15 | 49.557250 | -110.390120 | 544108 | 5489584 | 1 | 7.32 | Local |
| M3 | 05/27/2012 0:00 | 18 | 49.557240 | -110.390220 | 544101 | 5489583 | 1 | 2.44 | Local |
| M3 | 05/27/2012 0:00 | 21 | 49.557250 | -110.390190 | 544103 | 5489584 | 1 | 25.34 | Local |
| M3 | 05/28/2012 0:00 | 0 | 49.557240 | -110.390540 | 544078 | 5489583 | 1 | 23.25 | Local |
| M3 | 05/28/2012 0:00 | 3 | 49.557260 | -110.390220 | 544101 | 5489585 | 1 | 43.65 | Local |
| M3 | 05/28/2012 0:00 | 6 | 49.557050 | -110.390730 | 544064 | 5489562 | 0 | 46.19 | Local |
| M3 | 05/28/2012 0:00 | 12 | 49.557300 | -110.390220 | 544101 | 5489590 | 1 | 22.15 | Local |
| M3 | 05/28/2012 0:00 | 15 | 49.557260 | -110.390520 | 544079 | 5489585 | 1 | 22.67 | Local |
| M3 | 05/28/2012 0:00 | 18 | 49.557290 | -110.390210 | 544102 | 5489589 | 1 | 33.03 | Local |
| M3 | 05/28/2012 0:00 | 21 | 49.557190 | -110.390640 | 544071 | 5489577 | 1 | 422.73 | Local |
| M3 | 05/29/2012 0:00 | 0 | 49.560880 | -110.392050 | 543965 | 5489987 | 1 | 443.40 | Local |
| M3 | 05/29/2012 0:00 | 3 | 49.557110 | -110.390050 | 544113 | 5489569 | 1 | 17.20 | Local |
| M3 | 05/29/2012 0:00 | 6 | 49.557230 | -110.390200 | 544102 | 5489582 | 1 | 3.78 | Local |
| M3 | 05/29/2012 0:00 | 9 | 49.557220 | -110.390250 | 544099 | 5489581 | 0 | 8.89 | Local |
| M3 | 05/29/2012 0:00 | 15 | 49.557300 | -110.390250 | 544099 | 5489590 | 0 | 44.17 | Local |
| M3 | 05/29/2012 0:00 | 21 | 49.557280 | -110.390860 | 544055 | 5489587 | 1 | 1202.89 | Local |
| M3 | 05/30/2012 0:00 | 0 | 49.547620 | -110.383370 | 544605 | 5488518 | 1 | 1817.41 | Local |
| M3 | 05/30/2012 0:00 | 3 | 49.536380 | -110.401610 | 543296 | 5487257 | 1 | 2579.33 | Local |
| M3 | 05/30/2012 0:00 | 6 | 49.557090 | -110.385540 | 544440 | 5489569 | 0 | 263.01 | Local |
| M3 | 05/30/2012 0:00 | 12 | 49.558850 | -110.383110 | 544614 | 5489766 | 1 | 2890.53 | Local |
| M3 | 05/30/2012 0:00 | 15 | 49.573320 | -110.416320 | 542200 | 5491356 | 0 | 5129.25 | Local |
| M3 | 05/30/2012 0:00 | 21 | 49.610750 | -110.457810 | 539170 | 5495495 | 1 | 2777.52 | Local |
| M3 | 05/31/2012 0:00 | 0 | 49.635730 | -110.457290 | 539187 | 5498272 | 1 | 1828.23 | Local |
| M3 | 05/31/2012 0:00 | 3 | 49.631250 | -110.481650 | 537432 | 5497762 | 1 | 198.34 | Local |
| M3 | 05/31/2012 0:00 | 6 | 49.632700 | -110.480050 | 537546 | 5497924 | 0 | 427.50 | Local |
| M3 | 05/31/2012 0:00 | 12 | 49.631110 | -110.485440 | 537158 | 5497744 | 1 | 31.30 | Local |
| M3 | 05/31/2012 0:00 | 15 | 49.631340 | -110.485190 | 537176 | 5497770 | 1 | 1990.22 | Local |
| M3 | 05/31/2012 0:00 | 18 | 49.624350 | -110.459820 | 539014 | 5497006 | 1 | 4354.96 | Local |
| M3 | 05/31/2012 0:00 | 21 | 49.585180 | -110.459450 | 539072 | 5492651 | 1 | 2350.68 | Local |
| M3 | 06/01/2012 0:00 | 0 | 49.573150 | -110.432710 | 541015 | 5491328 | 1 | 6612.52 | Local |
| M3 | 06/01/2012 0:00 | 3 | 49.526140 | -110.376710 | 545107 | 5486134 | 0 | 1314.63 | Local |
| M3 | 06/01/2012 0:00 | 12 | 49.525220 | -110.394820 | 543797 | 5486021 | 1 | 113.16 | Local |
| M3 | 06/01/2012 0:00 | 15 | 49.525660 | -110.393410 | 543899 | 5486070 | 1 | 1556.27 | Local |
| M3 | 06/01/2012 0:00 | 18 | 49.533300 | -110.411430 | 542588 | 5486909 | 1 | 27.50 | Local |
| M3 | 06/01/2012 0:00 | 21 | 49.533530 | -110.411290 | 542598 | 5486935 | 1 | 4.42 | Local |
| M3 | 06/02/2012 0:00 | 0 | 49.533500 | -110.411250 | 542601 | 5486932 | 0 | 3.62 | Local |
| M3 | 06/02/2012 0:00 | 6 | 49.533500 | -110.411300 | 542597 | 5486932 | 0 | 25.20 | Local |
| M3 | 06/02/2012 0:00 | 15 | 49.533310 | -110.411490 | 542584 | 5486910 | 1 | 27.35 | Local |
| M3 | 06/02/2012 0:00 | 18 | 49.533510 | -110.411270 | 542599 | 5486933 | 1 | 17.51 | Local |
| M3 | 06/02/2012 0:00 | 21 | 49.533530 | -110.411510 | 542582 | 5486935 | 1 | 17.93 | Local |
| M3 | 06/03/2012 0:00 | 0 | 49.533490 | -110.411270 | 542599 | 5486931 | 1 | 742.29 | Local |
| M3 | 06/03/2012 0:00 | 3 | 49.531790 | -110.421190 | 541883 | 5486736 | 1 | 717.08 | Local |
| M3 | 06/03/2012 0:00 | 6 | 49.533390 | -110.411590 | 542576 | 5486919 | 1 | 13.36 | Local |
| M3 | 06/03/2012 0:00 | 9 | 49.533320 | -110.411440 | 542587 | 5486912 | 0 | 23.36 | Local |
| M3 | 06/03/2012 0:00 | 15 | 49.533530 | -110.411430 | 542588 | 5486935 | 1 | 18.25 | Local |
| M3 | 06/03/2012 0:00 | 18 | 49.533430 | -110.411230 | 542602 | 5486924 | 0 | 8.58 | Local |

| | | | | | | | | | |
|---|---|---|---|---|---|---|---|---|---|
| M3 | 06/04/2012 0:00 | 0 | 49.533500 | -110.411280 | 542599 | 5486932 | 1 | 2.89 | Local |
| M3 | 06/04/2012 0:00 | 3 | 49.533500 | -110.411240 | 542601 | 5486932 | 1 | 46.43 | Local |
| M3 | 06/04/2012 0:00 | 6 | 49.533470 | -110.410600 | 542648 | 5486929 | 0 | 3228.59 | Local |
| M3 | 06/04/2012 0:00 | 12 | 49.556960 | -110.384360 | 544525 | 5489555 | 1 | 2.22 | Local |
| M3 | 06/04/2012 0:00 | 15 | 49.556940 | -110.384360 | 544525 | 5489553 | 1 | 320.88 | Local |
| M3 | 06/04/2012 0:00 | 18 | 49.559090 | -110.387320 | 544309 | 5489790 | 1 | 2166.36 | Local |
| M3 | 06/04/2012 0:00 | 21 | 49.539620 | -110.388510 | 544241 | 5487625 | 1 | 1590.97 | Local |
| M3 | 06/05/2012 0:00 | 0 | 49.532440 | -110.407530 | 542871 | 5486816 | 0 | 1231.74 | Local |
| M3 | 06/05/2012 0:00 | 6 | 49.525130 | -110.394740 | 543803 | 5486011 | 0 | 143.75 | Local |
| M3 | 06/05/2012 0:00 | 18 | 49.524430 | -110.393070 | 543924 | 5485934 | 1 | 1347.83 | Local |
| M3 | 06/05/2012 0:00 | 21 | 49.532290 | -110.407250 | 542891 | 5486799 | 1 | 28.48 | Local |
| M3 | 06/06/2012 0:00 | 0 | 49.532470 | -110.407530 | 542871 | 5486819 | 0 | 97.39 | Local |
| M3 | 06/06/2012 0:00 | 9 | 49.532100 | -110.406310 | 542959 | 5486779 | 0 | 5.56 | Local |
| M3 | 06/06/2012 0:00 | 15 | 49.532150 | -110.406310 | 542959 | 5486784 | 1 | 102.14 | Local |
| M3 | 06/06/2012 0:00 | 18 | 49.532310 | -110.407700 | 542859 | 5486801 | 0 | 23.43 | Local |
| M3 | 06/07/2012 0:00 | 3 | 49.532500 | -110.407560 | 542869 | 5486823 | 1 | 1373.72 | Local |
| M3 | 06/07/2012 0:00 | 6 | 49.542000 | -110.395420 | 543739 | 5487886 | 1 | 1280.15 | Local |
| M3 | 06/07/2012 0:00 | 9 | 49.551840 | -110.386230 | 544395 | 5488985 | 1 | 58.24 | Local |
| M3 | 06/07/2012 0:00 | 12 | 49.552340 | -110.385990 | 544411 | 5489041 | 1 | 56.75 | Local |
| M3 | 06/07/2012 0:00 | 15 | 49.552830 | -110.385770 | 544427 | 5489095 | 1 | 427.12 | Local |
| M3 | 06/07/2012 0:00 | 18 | 49.556570 | -110.384420 | 544521 | 5489512 | 1 | 2897.50 | Local |
| M3 | 06/07/2012 0:00 | 21 | 49.579470 | -110.403550 | 543117 | 5492047 | 1 | 4489.08 | Local |
| M3 | 06/08/2012 0:00 | 0 | 49.605510 | -110.451020 | 539665 | 5494916 | 1 | 3318.31 | Local |
| M3 | 06/08/2012 0:00 | 3 | 49.631640 | -110.473220 | 538040 | 5497809 | 1 | 1097.54 | Local |
| M3 | 06/08/2012 0:00 | 6 | 49.637830 | -110.461380 | 538890 | 5498504 | 1 | 1681.26 | Local |
| M3 | 06/08/2012 0:00 | 9 | 49.624330 | -110.450890 | 539659 | 5497008 | 0 | 1826.61 | Local |
| M3 | 06/08/2012 0:00 | 15 | 49.610780 | -110.436590 | 540703 | 5495509 | 0 | 781.51 | Local |
| M3 | 06/08/2012 0:00 | 21 | 49.606910 | -110.445620 | 540054 | 5495074 | 1 | 1514.88 | Local |
| M3 | 06/09/2012 0:00 | 0 | 49.596880 | -110.431430 | 541087 | 5493967 | 1 | 89.18 | Local |
| M3 | 06/09/2012 0:00 | 3 | 49.596080 | -110.431340 | 541095 | 5493878 | 1 | 670.03 | Local |
| M3 | 06/09/2012 0:00 | 6 | 49.590870 | -110.426680 | 541436 | 5493301 | 1 | 553.67 | Local |
| M3 | 06/09/2012 0:00 | 9 | 49.585890 | -110.426680 | 541440 | 5492748 | 1 | 498.92 | Local |
| M3 | 06/09/2012 0:00 | 12 | 49.581630 | -110.428850 | 541287 | 5492273 | 1 | 93.49 | Local |
| M3 | 06/09/2012 0:00 | 15 | 49.581570 | -110.430140 | 541193 | 5492265 | 1 | 161.55 | Local |
| M3 | 06/09/2012 0:00 | 18 | 49.581890 | -110.427960 | 541351 | 5492302 | 1 | 982.94 | Local |
| M3 | 06/09/2012 0:00 | 21 | 49.573100 | -110.426500 | 541464 | 5491326 | 1 | 1057.73 | Local |
| M3 | 06/10/2012 0:00 | 0 | 49.570710 | -110.440660 | 540442 | 5491052 | 1 | 1384.57 | Local |
| M3 | 06/10/2012 0:00 | 3 | 49.580060 | -110.453310 | 539520 | 5492085 | 1 | 1809.66 | Local |
| M3 | 06/10/2012 0:00 | 6 | 49.596240 | -110.450580 | 539704 | 5493885 | 0 | 744.82 | Local |
| M3 | 06/10/2012 0:00 | 12 | 49.595540 | -110.440330 | 540445 | 5493813 | 0 | 219.51 | Local |
| M3 | 06/10/2012 0:00 | 18 | 49.594400 | -110.442810 | 540267 | 5493685 | 1 | 873.21 | Local |
| M3 | 06/10/2012 0:00 | 21 | 49.601400 | -110.437330 | 540657 | 5494466 | 0 | 760.81 | Local |
| M3 | 06/11/2012 0:00 | 9 | 49.606300 | -110.444680 | 540122 | 5495007 | 1 | 249.31 | Local |
| M3 | 06/11/2012 0:00 | 12 | 49.608150 | -110.446630 | 539980 | 5495212 | 1 | 71.93 | Local |
| M3 | 06/11/2012 0:00 | 15 | 49.608610 | -110.447330 | 539929 | 5495262 | 1 | 147.10 | Local |
| M3 | 06/11/2012 0:00 | 18 | 49.609190 | -110.445500 | 540060 | 5495328 | 1 | 528.99 | Local |
| M3 | 06/11/2012 0:00 | 21 | 49.609900 | -110.438260 | 540583 | 5495411 | 0 | 1587.38 | Local |
| M3 | 06/12/2012 0:00 | 3 | 49.596420 | -110.431020 | 541117 | 5493916 | 1 | 2227.30 | Local |
| M3 | 06/12/2012 0:00 | 6 | 49.576610 | -110.426430 | 541466 | 5491716 | 1 | 48.14 | Local |
| M3 | 06/12/2012 0:00 | 9 | 49.576250 | -110.426060 | 541493 | 5491676 | 1 | 208.09 | Local |
| M3 | 06/12/2012 0:00 | 12 | 49.574970 | -110.423960 | 541646 | 5491535 | 1 | 1720.82 | Local |
| M3 | 06/12/2012 0:00 | 15 | 49.563760 | -110.407550 | 542842 | 5490298 | 1 | 374.24 | Local |
| M3 | 06/12/2012 0:00 | 18 | 49.563610 | -110.402380 | 543216 | 5490284 | 1 | 61.19 | Local |
| M3 | 06/12/2012 0:00 | 21 | 49.563250 | -110.401740 | 543263 | 5490245 | 1 | 1427.54 | Local |
| M3 | 06/13/2012 0:00 | 0 | 49.550410 | -110.401790 | 543270 | 5488817 | 1 | 3068.11 | Local |

| | | | | | | | | | |
|---|---|---|---|---|---|---|---|---|---|
| M3 | 06/13/2012 0:00 | 3 | 49.528390 | -110.427350 | 541440 | 5486355 | 1 | 1861.45 | Local |
| M3 | 06/13/2012 0:00 | 6 | 49.511650 | -110.427840 | 541419 | 5484493 | 1 | 207.85 | Local |
| M3 | 06/13/2012 0:00 | 9 | 49.513180 | -110.429490 | 541298 | 5484662 | 1 | 212.16 | Local |
| M3 | 06/13/2012 0:00 | 12 | 49.514860 | -110.428100 | 541397 | 5484850 | 1 | 2822.39 | Local |
| M3 | 06/13/2012 0:00 | 15 | 49.514800 | -110.389110 | 544220 | 5484866 | 1 | 542.57 | Local |
| M3 | 06/13/2012 0:00 | 18 | 49.517810 | -110.383210 | 544644 | 5485204 | 1 | 7.32 | Local |
| M3 | 06/13/2012 0:00 | 21 | 49.517840 | -110.383300 | 544637 | 5485207 | 1 | 15.80 | Local |
| M3 | 06/14/2012 0:00 | 0 | 49.517770 | -110.383490 | 544624 | 5485199 | 1 | 14.15 | Local |
| M3 | 06/14/2012 0:00 | 3 | 49.517740 | -110.383300 | 544637 | 5485196 | 1 | 6.51 | Local |
| M3 | 06/14/2012 0:00 | 6 | 49.517740 | -110.383210 | 544644 | 5485196 | 1 | 5.48 | Local |
| M3 | 06/14/2012 0:00 | 9 | 49.517710 | -110.383270 | 544640 | 5485192 | 1 | 15.09 | Local |
| M3 | 06/14/2012 0:00 | 12 | 49.517840 | -110.383330 | 544635 | 5485207 | 0 | 7.82 | Local |
| M3 | 06/14/2012 0:00 | 18 | 49.517770 | -110.383320 | 544636 | 5485199 | 1 | 54.48 | Local |
| M3 | 06/14/2012 0:00 | 21 | 49.518090 | -110.382750 | 544677 | 5485235 | 1 | 5.56 | Local |
| M3 | 06/15/2012 0:00 | 0 | 49.518040 | -110.382750 | 544677 | 5485229 | 0 | 51.02 | Local |
| M3 | 06/15/2012 0:00 | 6 | 49.517770 | -110.383320 | 544636 | 5485199 | 1 | 4.51 | Local |
| M3 | 06/15/2012 0:00 | 9 | 49.517730 | -110.383310 | 544637 | 5485195 | 0 | 404.17 | Local |
| M3 | 06/15/2012 0:00 | 15 | 49.521060 | -110.385550 | 544472 | 5485564 | 0 | 399.25 | Local |
| M3 | 06/16/2012 0:00 | 0 | 49.517790 | -110.383270 | 544640 | 5485201 | 0 | 361.53 | Local |
| M3 | 06/16/2012 0:00 | 6 | 49.517160 | -110.388170 | 544286 | 5485128 | 0 | 341.07 | Local |
| M3 | 06/16/2012 0:00 | 12 | 49.518400 | -110.392480 | 543972 | 5485264 | 1 | 15.92 | Local |
| M3 | 06/16/2012 0:00 | 15 | 49.518520 | -110.392600 | 543964 | 5485277 | 1 | 0.00 | Local |
| M3 | 06/16/2012 0:00 | 18 | 49.518520 | -110.392600 | 543964 | 5485277 | 1 | 510.78 | Local |
| M3 | 06/16/2012 0:00 | 21 | 49.517620 | -110.385680 | 544465 | 5485181 | 0 | 173.70 | Local |
| M3 | 06/17/2012 0:00 | 21 | 49.517820 | -110.383300 | 544637 | 5485205 | 0 | 3.11 | Local |
| M3 | 06/18/2012 0:00 | 3 | 49.517800 | -110.383270 | 544640 | 5485202 | 1 | 3836.29 | Local |
| M3 | 06/18/2012 0:00 | 6 | 49.552260 | -110.386000 | 544411 | 5489032 | 0 | 1.33 | Local |
| M3 | 06/18/2012 0:00 | 12 | 49.552270 | -110.385990 | 544411 | 5489033 | 1 | 12.31 | Local |
| M3 | 06/18/2012 0:00 | 15 | 49.552160 | -110.385970 | 544413 | 5489021 | 1 | 995.54 | Local |
| M3 | 06/18/2012 0:00 | 18 | 49.560710 | -110.390060 | 544109 | 5489969 | 0 | 18.94 | Local |
| M3 | 06/19/2012 0:00 | 3 | 49.560600 | -110.390260 | 544095 | 5489957 | 1 | 445.01 | Local |
| M3 | 06/19/2012 0:00 | 6 | 49.558460 | -110.395460 | 543721 | 5489716 | 1 | 511.07 | Local |
| M3 | 06/19/2012 0:00 | 9 | 49.560940 | -110.389510 | 544149 | 5489995 | 1 | 63.85 | Local |
| M3 | 06/19/2012 0:00 | 12 | 49.560410 | -110.389850 | 544125 | 5489936 | 1 | 17.25 | Local |
| M3 | 06/19/2012 0:00 | 15 | 49.560470 | -110.390070 | 544109 | 5489942 | 1 | 20.84 | Local |
| M3 | 06/19/2012 0:00 | 18 | 49.560630 | -110.390220 | 544098 | 5489960 | 1 | 12.23 | Local |
| M3 | 06/19/2012 0:00 | 21 | 49.560520 | -110.390220 | 544098 | 5489948 | 0 | 8.08 | Local |
| M3 | 06/20/2012 0:00 | 3 | 49.560450 | -110.390250 | 544096 | 5489940 | 0 | 5.61 | Local |
| M3 | 06/20/2012 0:00 | 9 | 49.560500 | -110.390240 | 544097 | 5489946 | 1 | 7.01 | Local |
| M3 | 06/20/2012 0:00 | 12 | 49.560560 | -110.390210 | 544099 | 5489952 | 0 | 11.94 | Local |
| M3 | 06/20/2012 0:00 | 18 | 49.560470 | -110.390120 | 544105 | 5489942 | 1 | 12.49 | Local |
| M3 | 06/20/2012 0:00 | 21 | 49.560450 | -110.390290 | 544093 | 5489940 | 0 | 3.10 | Local |
| M3 | 06/21/2012 0:00 | 3 | 49.560460 | -110.390250 | 544096 | 5489941 | 1 | 6.83 | Local |
| M3 | 06/21/2012 0:00 | 6 | 49.560520 | -110.390230 | 544097 | 5489948 | 1 | 4.45 | Local |
| M3 | 06/21/2012 0:00 | 9 | 49.560480 | -110.390230 | 544097 | 5489943 | 1 | 12.49 | Local |
| M3 | 06/21/2012 0:00 | 12 | 49.560500 | -110.390060 | 544110 | 5489946 | 0 | 20.22 | Local |
| M3 | 06/21/2012 0:00 | 18 | 49.560680 | -110.390100 | 544107 | 5489966 | 0 | 339.41 | Local |
| M3 | 06/22/2012 0:00 | 6 | 49.557880 | -110.391970 | 543974 | 5489653 | 1 | 25.58 | Local |
| M3 | 06/22/2012 0:00 | 9 | 49.558110 | -110.391960 | 543974 | 5489679 | 0 | 731.47 | Local |
| M3 | 06/22/2012 0:00 | 15 | 49.562890 | -110.398910 | 543468 | 5490206 | 1 | 2069.36 | Local |
| M3 | 06/22/2012 0:00 | 18 | 49.575010 | -110.420630 | 541887 | 5491541 | 1 | 4.34 | Local |
| M3 | 06/22/2012 0:00 | 21 | 49.575010 | -110.420570 | 541891 | 5491541 | 1 | 26.82 | Local |
| M3 | 06/23/2012 0:00 | 0 | 49.575090 | -110.420220 | 541916 | 5491550 | 1 | 161.61 | Local |
| M3 | 06/23/2012 0:00 | 3 | 49.574990 | -110.422450 | 541755 | 5491538 | 1 | 406.85 | Local |
| M3 | 06/23/2012 0:00 | 6 | 49.573080 | -110.427250 | 541410 | 5491323 | 0 | 1617.61 | Local |

| | | | | | | | | | |
|---|---|---|---|---|---|---|---|---|---|
| M3 | 06/23/2012 0:00 | 12 | 49.586700 | -110.435120 | 540829 | 5492833 | 1 | 158.29 | Local |
| M3 | 06/23/2012 0:00 | 15 | 49.587970 | -110.434130 | 540900 | 5492975 | 1 | 1440.39 | Local |
| M3 | 06/23/2012 0:00 | 18 | 49.600270 | -110.440390 | 540437 | 5494339 | 1 | 3307.80 | Local |
| M3 | 06/23/2012 0:00 | 21 | 49.624880 | -110.466120 | 538558 | 5497061 | 1 | 2540.96 | Local |
| M3 | 06/24/2012 0:00 | 0 | 49.647680 | -110.468550 | 538365 | 5499595 | 1 | 266.69 | Local |
| M3 | 06/24/2012 0:00 | 3 | 49.647470 | -110.464870 | 538631 | 5499573 | 1 | 275.53 | Local |
| M3 | 06/24/2012 0:00 | 6 | 49.646370 | -110.461450 | 538879 | 5499453 | 1 | 17.64 | Local |
| M3 | 06/24/2012 0:00 | 9 | 49.646240 | -110.461310 | 538889 | 5499439 | 1 | 6.87 | Local |
| M3 | 06/24/2012 0:00 | 12 | 49.646260 | -110.461400 | 538882 | 5499441 | 1 | 2.17 | Local |
| M3 | 06/24/2012 0:00 | 15 | 49.646260 | -110.461430 | 538880 | 5499441 | 1 | 5.74 | Local |
| M3 | 06/24/2012 0:00 | 18 | 49.646210 | -110.461410 | 538882 | 5499435 | 1 | 9.15 | Local |
| M3 | 06/24/2012 0:00 | 21 | 49.646290 | -110.461380 | 538884 | 5499444 | 1 | 2094.61 | Local |
| M3 | 06/25/2012 0:00 | 0 | 49.640060 | -110.434000 | 540865 | 5498766 | 1 | 2378.58 | Local |
| M3 | 06/25/2012 0:00 | 3 | 49.656050 | -110.412110 | 542432 | 5500556 | 1 | 1459.85 | Local |
| M3 | 06/25/2012 0:00 | 6 | 49.650730 | -110.393620 | 543771 | 5499975 | 1 | 254.26 | Local |
| M3 | 06/25/2012 0:00 | 9 | 49.652740 | -110.395300 | 543648 | 5500197 | 1 | 136.13 | Local |
| M3 | 06/25/2012 0:00 | 12 | 49.653960 | -110.395140 | 543659 | 5500333 | 0 | 438.14 | Local |
| M3 | 06/25/2012 0:00 | 18 | 49.650020 | -110.395020 | 543671 | 5499895 | 1 | 406.37 | Local |
| M3 | 06/25/2012 0:00 | 21 | 49.653570 | -110.396360 | 543571 | 5500289 | 1 | 1867.99 | Local |
| M3 | 06/26/2012 0:00 | 0 | 49.642870 | -110.376410 | 545021 | 5499111 | 1 | 4454.91 | Local |
| M3 | 06/26/2012 0:00 | 3 | 49.645320 | -110.438000 | 540572 | 5499349 | 0 | 1725.07 | Local |
| M3 | 06/26/2012 0:00 | 12 | 49.646160 | -110.461860 | 538849 | 5499429 | 1 | 34.81 | Local |
| M3 | 06/26/2012 0:00 | 15 | 49.646130 | -110.461380 | 538884 | 5499426 | 0 | 220.20 | Local |
| M3 | 06/27/2012 0:00 | 0 | 49.645810 | -110.464390 | 538667 | 5499389 | 0 | 253.03 | Local |
| M3 | 06/27/2012 0:00 | 6 | 49.645100 | -110.461060 | 538908 | 5499312 | 0 | 129.71 | Local |
| M3 | 06/27/2012 0:00 | 12 | 49.644510 | -110.459510 | 539020 | 5499247 | 0 | 716.36 | Local |
| M3 | 06/27/2012 0:00 | 18 | 49.638150 | -110.457920 | 539140 | 5498541 | 1 | 958.47 | Local |
| M3 | 06/27/2012 0:00 | 21 | 49.630490 | -110.464010 | 538706 | 5497686 | 1 | 2201.59 | Local |
| M3 | 06/28/2012 0:00 | 0 | 49.650180 | -110.467250 | 538457 | 5499874 | 1 | 599.28 | Local |
| M3 | 06/28/2012 0:00 | 3 | 49.646330 | -110.461440 | 538879 | 5499449 | 0 | 48.69 | Local |
| M3 | 06/28/2012 0:00 | 12 | 49.646130 | -110.462040 | 538836 | 5499426 | 1 | 5.17 | Local |
| M3 | 06/28/2012 0:00 | 15 | 49.646120 | -110.462110 | 538831 | 5499425 | 1 | 54.85 | Local |
| M3 | 06/28/2012 0:00 | 18 | 49.646340 | -110.461430 | 538880 | 5499450 | 1 | 19.24 | Local |
| M3 | 06/28/2012 0:00 | 21 | 49.646170 | -110.461380 | 538884 | 5499431 | 1 | 2327.91 | Local |
| M3 | 06/29/2012 0:00 | 0 | 49.650500 | -110.429830 | 541158 | 5499929 | 1 | 1068.76 | Local |
| M3 | 06/29/2012 0:00 | 3 | 49.654790 | -110.416580 | 542111 | 5500413 | 1 | 46.04 | Local |
| M3 | 06/29/2012 0:00 | 6 | 49.655200 | -110.416490 | 542117 | 5500459 | 0 | 21.40 | Local |
| M3 | 06/29/2012 0:00 | 18 | 49.655240 | -110.416200 | 542138 | 5500463 | 1 | 26.50 | Local |
| M3 | 06/29/2012 0:00 | 21 | 49.655150 | -110.415860 | 542162 | 5500454 | 1 | 2012.38 | Local |
| M3 | 06/30/2012 0:00 | 0 | 49.656340 | -110.388040 | 544169 | 5500602 | 1 | 2436.90 | Local |
| M3 | 06/30/2012 0:00 | 3 | 49.658130 | -110.354390 | 546596 | 5500821 | 1 | 5186.07 | Local |
| M3 | 06/30/2012 0:00 | 6 | 49.640450 | -110.420870 | 541813 | 5498817 | 0 | 1781.66 | Local |
| M3 | 06/30/2012 0:00 | 12 | 49.632530 | -110.442320 | 540271 | 5497924 | 1 | 637.62 | Local |
| M3 | 06/30/2012 0:00 | 15 | 49.630610 | -110.450640 | 539672 | 5497706 | 0 | 2599.68 | Local |
| M3 | 06/30/2012 0:00 | 21 | 49.607330 | -110.454010 | 539447 | 5495116 | 1 | 3724.63 | Local |
| M3 | 07/01/2012 0:00 | 0 | 49.574150 | -110.446890 | 539989 | 5491431 | 1 | 1653.14 | Local |
| M3 | 07/01/2012 0:00 | 3 | 49.573120 | -110.424080 | 541639 | 5491329 | 0 | 3017.19 | Local |
| M3 | 07/01/2012 0:00 | 12 | 49.558050 | -110.389380 | 544161 | 5489674 | 0 | 20.01 | Local |
| M3 | 07/01/2012 0:00 | 21 | 49.557870 | -110.389380 | 544161 | 5489654 | 1 | 12.42 | Local |
| M3 | 07/02/2012 0:00 | 0 | 49.557980 | -110.389350 | 544163 | 5489666 | 0 | 18.84 | Local |
| M3 | 07/02/2012 0:00 | 6 | 49.557990 | -110.389610 | 544144 | 5489667 | 0 | 52.77 | Local |
| M3 | 07/02/2012 0:00 | 12 | 49.558410 | -110.389270 | 544169 | 5489714 | 0 | 10.61 | Local |
| M3 | 07/02/2012 0:00 | 18 | 49.558330 | -110.389190 | 544175 | 5489705 | 1 | 37.33 | Local |
| M3 | 07/02/2012 0:00 | 21 | 49.558110 | -110.389580 | 544147 | 5489680 | 0 | 26.41 | Local |
| M3 | 07/03/2012 0:00 | 21 | 49.558270 | -110.389310 | 544166 | 5489698 | 1 | 62.74 | Local |

| | | | | | | | | | |
|---|---|---|---|---|---|---|---|---|---|
| M3 | 07/04/2012 0:00 | 0 | 49.558010 | -110.390080 | 544110 | 5489669 | 1 | 18.94 | Local |
| M3 | 07/04/2012 0:00 | 3 | 49.558030 | -110.389820 | 544129 | 5489671 | 0 | 661.90 | Local |
| M3 | 07/04/2012 0:00 | 15 | 49.558150 | -110.380670 | 544791 | 5489690 | 1 | 70.27 | Local |
| M3 | 07/04/2012 0:00 | 18 | 49.557990 | -110.379730 | 544859 | 5489673 | 1 | 335.32 | Local |
| M3 | 07/04/2012 0:00 | 21 | 49.560160 | -110.382950 | 544624 | 5489912 | 1 | 761.74 | Local |
| M3 | 07/05/2012 0:00 | 0 | 49.556150 | -110.391490 | 544010 | 5489461 | 1 | 1800.68 | Local |
| M3 | 07/05/2012 0:00 | 3 | 49.565380 | -110.411950 | 542522 | 5490476 | 1 | 2211.43 | Local |
| M3 | 07/05/2012 0:00 | 6 | 49.581770 | -110.429280 | 541255 | 5492288 | 1 | 130.97 | Local |
| M3 | 07/05/2012 0:00 | 9 | 49.582380 | -110.430830 | 541143 | 5492355 | 0 | 578.44 | Local |
| M3 | 07/05/2012 0:00 | 18 | 49.587000 | -110.434510 | 540873 | 5492867 | 1 | 1632.08 | Local |
| M3 | 07/05/2012 0:00 | 21 | 49.600500 | -110.443380 | 540221 | 5494363 | 0 | 4989.74 | Local |
| M3 | 07/06/2012 0:00 | 3 | 49.567660 | -110.396330 | 543650 | 5490738 | 0 | 2968.41 | Local |
| M3 | 07/06/2012 0:00 | 9 | 49.582550 | -110.430410 | 541173 | 5492374 | 0 | 5.56 | Local |
| M3 | 07/06/2012 0:00 | 15 | 49.582500 | -110.430410 | 541173 | 5492369 | 0 | 72.76 | Local |
| M3 | 07/06/2012 0:00 | 21 | 49.582160 | -110.429550 | 541236 | 5492331 | 1 | 4256.99 | Local |
| M3 | 07/07/2012 0:00 | 0 | 49.557120 | -110.385010 | 544478 | 5489573 | 1 | 1746.86 | Local |
| M3 | 07/07/2012 0:00 | 3 | 49.541720 | -110.389800 | 544145 | 5487858 | 1 | 4168.71 | Local |
| M3 | 07/07/2012 0:00 | 6 | 49.573260 | -110.420970 | 541863 | 5491347 | 1 | 2283.18 | Local |
| M3 | 07/07/2012 0:00 | 9 | 49.590450 | -110.438250 | 540600 | 5493248 | 1 | 1192.08 | Local |
| M3 | 07/07/2012 0:00 | 12 | 49.600370 | -110.444510 | 540139 | 5494348 | 1 | 510.55 | Local |
| M3 | 07/07/2012 0:00 | 15 | 49.603850 | -110.439900 | 540469 | 5494737 | 1 | 1628.39 | Local |
| M3 | 07/07/2012 0:00 | 18 | 49.613080 | -110.457400 | 539198 | 5495754 | 1 | 2322.91 | Local |
| M3 | 07/07/2012 0:00 | 21 | 49.632790 | -110.468070 | 538411 | 5497940 | 1 | 1707.79 | Local |
| M3 | 07/08/2012 0:00 | 0 | 49.648150 | -110.467860 | 538414 | 5499648 | 1 | 1584.47 | Local |
| M3 | 07/08/2012 0:00 | 3 | 49.658130 | -110.452190 | 539538 | 5500765 | 0 | 2191.63 | Local |
| M3 | 07/08/2012 0:00 | 15 | 49.638690 | -110.457220 | 539190 | 5498601 | 1 | 30.30 | Local |
| M3 | 07/08/2012 0:00 | 18 | 49.638830 | -110.456860 | 539216 | 5498617 | 1 | 546.22 | Local |
| M3 | 07/08/2012 0:00 | 21 | 49.636890 | -110.449910 | 539719 | 5498405 | 1 | 676.33 | Local |
| M3 | 07/09/2012 0:00 | 0 | 49.632900 | -110.442840 | 540233 | 5497965 | 1 | 1860.51 | Local |
| M3 | 07/09/2012 0:00 | 3 | 49.617900 | -110.431420 | 541070 | 5496304 | 1 | 1616.18 | Local |
| M3 | 07/09/2012 0:00 | 6 | 49.603510 | -110.434590 | 540853 | 5494702 | 1 | 1778.86 | Local |
| M3 | 07/09/2012 0:00 | 9 | 49.587530 | -110.433360 | 540956 | 5492926 | 1 | 438.01 | Local |
| M3 | 07/09/2012 0:00 | 12 | 49.584420 | -110.437080 | 540689 | 5492578 | 0 | 7.01 | Local |
| M3 | 07/09/2012 0:00 | 18 | 49.584360 | -110.437110 | 540687 | 5492572 | 1 | 52.81 | Local |
| M3 | 07/09/2012 0:00 | 21 | 49.584380 | -110.436380 | 540740 | 5492574 | 0 | 50.90 | Local |
| M3 | 07/10/2012 0:00 | 3 | 49.584430 | -110.437080 | 540689 | 5492580 | 1 | 5.30 | Local |
| M3 | 07/10/2012 0:00 | 6 | 49.584390 | -110.437040 | 540692 | 5492575 | 1 | 607.77 | Local |
| M3 | 07/10/2012 0:00 | 9 | 49.579090 | -110.434980 | 540846 | 5491987 | 0 | 1035.25 | Local |
| M3 | 07/11/2012 0:00 | 0 | 49.572880 | -110.445650 | 540079 | 5491291 | 1 | 7.30 | Local |
| M3 | 07/11/2012 0:00 | 3 | 49.572920 | -110.445730 | 540074 | 5491295 | 1 | 22.53 | Local |
| M3 | 07/11/2012 0:00 | 6 | 49.572720 | -110.445680 | 540077 | 5491273 | 0 | 456.73 | Local |
| M3 | 07/11/2012 0:00 | 21 | 49.570610 | -110.440260 | 540471 | 5491041 | 1 | 2184.45 | Local |
| M3 | 07/12/2012 0:00 | 0 | 49.575110 | -110.410850 | 542593 | 5491558 | 1 | 2516.88 | Local |
| M3 | 07/12/2012 0:00 | 3 | 49.557960 | -110.388130 | 544252 | 5489664 | 0 | 1572.32 | Local |
| M3 | 07/12/2012 0:00 | 9 | 49.543920 | -110.385520 | 544453 | 5488105 | 0 | 1034.60 | Local |
| M3 | 07/12/2012 0:00 | 18 | 49.534720 | -110.387670 | 544306 | 5487081 | 1 | 1188.61 | Local |
| M3 | 07/12/2012 0:00 | 21 | 49.525130 | -110.394930 | 543789 | 5486011 | 1 | 1295.50 | Local |
| M3 | 07/13/2012 0:00 | 0 | 49.519510 | -110.379250 | 544929 | 5485395 | 1 | 2106.07 | Local |
| M3 | 07/13/2012 0:00 | 3 | 49.533920 | -110.398140 | 543549 | 5486986 | 0 | 373.03 | Local |
| M3 | 07/13/2012 0:00 | 18 | 49.535700 | -110.402510 | 543231 | 5487181 | 1 | 77.56 | Local |
| M3 | 07/13/2012 0:00 | 21 | 49.536310 | -110.401990 | 543268 | 5487249 | 1 | 82.46 | Local |
| M3 | 07/14/2012 0:00 | 0 | 49.535650 | -110.402510 | 543231 | 5487176 | 1 | 6.67 | Local |
| M3 | 07/14/2012 0:00 | 3 | 49.535710 | -110.402510 | 543231 | 5487182 | 1 | 6.71 | Local |
| M3 | 07/14/2012 0:00 | 6 | 49.535650 | -110.402500 | 543232 | 5487176 | 1 | 6.63 | Local |
| M3 | 07/14/2012 0:00 | 9 | 49.535700 | -110.402450 | 543236 | 5487181 | 1 | 5.97 | Local |

| | | | | | | | | | |
|---|---|---|---|---|---|---|---|---|---|
| M3 | 07/14/2012 0:00 | 12 | 49.535650 | -110.402420 | 543238 | 5487176 | 0 | 220.92 | Local |
| M3 | 07/14/2012 0:00 | 18 | 49.537140 | -110.400400 | 543383 | 5487343 | 1 | 48.19 | Local |
| M3 | 07/14/2012 0:00 | 21 | 49.537020 | -110.401040 | 543336 | 5487329 | 1 | 3971.81 | Local |
| M3 | 07/15/2012 0:00 | 0 | 49.570550 | -110.419990 | 541937 | 5491046 | 1 | 3113.43 | Local |
| M3 | 07/15/2012 0:00 | 3 | 49.585150 | -110.456740 | 539268 | 5492649 | 1 | 371.08 | Local |
| M3 | 07/15/2012 0:00 | 6 | 49.581840 | -110.457400 | 539223 | 5492281 | 1 | 4.92 | Local |
| M3 | 07/15/2012 0:00 | 9 | 49.581870 | -110.457450 | 539219 | 5492284 | 1 | 8.49 | Local |
| M3 | 07/15/2012 0:00 | 12 | 49.581910 | -110.457350 | 539226 | 5492289 | 0 | 6.88 | Local |
| M3 | 07/15/2012 0:00 | 21 | 49.581930 | -110.457260 | 539233 | 5492291 | 0 | 3.41 | Local |
| M3 | 07/16/2012 0:00 | 3 | 49.581900 | -110.457270 | 539232 | 5492288 | 0 | 1367.26 | Local |
| M3 | 07/16/2012 0:00 | 9 | 49.594090 | -110.459770 | 539042 | 5493642 | 1 | 3.61 | Local |
| M3 | 07/16/2012 0:00 | 12 | 49.594090 | -110.459820 | 539038 | 5493641 | 1 | 303.63 | Local |
| M3 | 07/16/2012 0:00 | 15 | 49.596800 | -110.459300 | 539073 | 5493943 | 1 | 3533.22 | Local |
| M3 | 07/16/2012 0:00 | 18 | 49.626790 | -110.475480 | 537881 | 5497269 | 0 | 2665.77 | Local |
| M3 | 07/17/2012 0:00 | 0 | 49.650670 | -110.472160 | 538102 | 5499926 | 1 | 474.28 | Local |
| M3 | 07/17/2012 0:00 | 3 | 49.653490 | -110.467230 | 538456 | 5500242 | 1 | 314.10 | Local |
| M3 | 07/17/2012 0:00 | 6 | 49.650680 | -110.466780 | 538490 | 5499929 | 1 | 2732.23 | Local |
| M3 | 07/17/2012 0:00 | 9 | 49.636640 | -110.497840 | 536259 | 5498353 | 1 | 30.05 | Local |
| M3 | 07/17/2012 0:00 | 12 | 49.636440 | -110.498120 | 536239 | 5498331 | 1 | 3.10 | Local |
| M3 | 07/17/2012 0:00 | 15 | 49.636420 | -110.498090 | 536241 | 5498328 | 0 | 1381.32 | Local |
| M3 | 07/17/2012 0:00 | 21 | 49.624000 | -110.497590 | 536286 | 5496948 | 1 | 1215.54 | Local |
| M3 | 07/18/2012 0:00 | 0 | 49.613940 | -110.504180 | 535818 | 5495826 | 1 | 1412.28 | Local |
| M3 | 07/18/2012 0:00 | 3 | 49.601310 | -110.506270 | 535676 | 5494421 | 1 | 788.63 | Local |
| M3 | 07/18/2012 0:00 | 6 | 49.606500 | -110.498830 | 536210 | 5495002 | 0 | 57.52 | Local |
| M3 | 07/18/2012 0:00 | 12 | 49.606840 | -110.498230 | 536253 | 5495040 | 1 | 3.10 | Local |
| M3 | 07/18/2012 0:00 | 15 | 49.606850 | -110.498270 | 536250 | 5495041 | 0 | 53.03 | Local |
| M3 | 07/18/2012 0:00 | 21 | 49.606520 | -110.498800 | 536212 | 5495004 | 0 | 5.61 | Local |
| M3 | 07/19/2012 0:00 | 3 | 49.606570 | -110.498790 | 536212 | 5495009 | 0 | 50.06 | Local |
| M3 | 07/19/2012 0:00 | 12 | 49.606860 | -110.498260 | 536251 | 5495042 | 0 | 534.03 | Local |
| M3 | 07/19/2012 0:00 | 18 | 49.604480 | -110.504680 | 535788 | 5494774 | 1 | 1904.36 | Local |
| M3 | 07/19/2012 0:00 | 21 | 49.620140 | -110.494000 | 536548 | 5496520 | 1 | 1074.09 | Local |
| M3 | 07/20/2012 0:00 | 0 | 49.629720 | -110.492080 | 536680 | 5497586 | 1 | 2897.90 | Local |
| M3 | 07/20/2012 0:00 | 3 | 49.649510 | -110.465960 | 538551 | 5499800 | 0 | 1908.62 | Local |
| M3 | 07/20/2012 0:00 | 12 | 49.643650 | -110.441110 | 540349 | 5499161 | 0 | 3470.83 | Local |
| M3 | 07/20/2012 0:00 | 21 | 49.656280 | -110.397140 | 543512 | 5500590 | 1 | 1115.69 | Local |
| M3 | 07/21/2012 0:00 | 0 | 49.660840 | -110.383370 | 544502 | 5501105 | 1 | 1062.55 | Local |
| M3 | 07/21/2012 0:00 | 3 | 49.667650 | -110.373040 | 545241 | 5501868 | 0 | 4.51 | Local |
| M3 | 07/21/2012 0:00 | 9 | 49.667610 | -110.373030 | 545242 | 5501864 | 0 | 2.34 | Local |
| M3 | 07/22/2012 0:00 | 0 | 49.667630 | -110.373020 | 545243 | 5501866 | 1 | 1.82 | Local |
| M3 | 07/22/2012 0:00 | 3 | 49.667620 | -110.373040 | 545241 | 5501865 | 0 | 2274.05 | Local |
| M3 | 07/22/2012 0:00 | 21 | 49.655650 | -110.398590 | 543408 | 5500519 | 1 | 1273.84 | Local |
| M3 | 07/23/2012 0:00 | 0 | 49.659450 | -110.415240 | 542203 | 5500932 | 1 | 3558.93 | Local |
| M3 | 07/23/2012 0:00 | 3 | 49.646240 | -110.460150 | 538973 | 5499439 | 1 | 2176.93 | Local |
| M3 | 07/23/2012 0:00 | 6 | 49.632010 | -110.480860 | 537488 | 5497847 | 0 | 87.31 | Local |
| M3 | 07/23/2012 0:00 | 12 | 49.632790 | -110.480720 | 537498 | 5497933 | 1 | 3.98 | Local |
| M3 | 07/23/2012 0:00 | 15 | 49.632820 | -110.480690 | 537500 | 5497937 | 1 | 3.10 | Local |
| M3 | 07/23/2012 0:00 | 18 | 49.632800 | -110.480720 | 537498 | 5497934 | 1 | 238.15 | Local |
| M3 | 07/23/2012 0:00 | 21 | 49.631780 | -110.483620 | 537289 | 5497820 | 1 | 1920.98 | Local |
| M3 | 07/24/2012 0:00 | 0 | 49.615450 | -110.492310 | 536674 | 5496000 | 1 | 3529.27 | Local |
| M3 | 07/24/2012 0:00 | 3 | 49.589490 | -110.464200 | 538725 | 5493128 | 1 | 1285.89 | Local |
| M3 | 07/24/2012 0:00 | 6 | 49.579920 | -110.454210 | 539455 | 5492069 | 0 | 1254.27 | Local |
| M3 | 07/24/2012 0:00 | 18 | 49.574930 | -110.438650 | 540584 | 5491523 | 1 | 474.33 | Local |
| M3 | 07/24/2012 0:00 | 21 | 49.570820 | -110.440410 | 540460 | 5491065 | 1 | 1045.36 | Local |
| M3 | 07/25/2012 0:00 | 0 | 49.562770 | -110.432940 | 541007 | 5490174 | 1 | 2504.51 | Local |
| M3 | 07/25/2012 0:00 | 3 | 49.541310 | -110.422410 | 541787 | 5487794 | 1 | 1161.22 | Local |

| | | | | | | | | | |
|---|---|---|---|---|---|---|---|---|---|
| M3 | 07/25/2012 0:00 | 6 | 49.533590 | -110.411600 | 542575 | 5486942 | 0 | 253.78 | Local |
| M3 | 07/25/2012 0:00 | 18 | 49.532190 | -110.408830 | 542777 | 5486787 | 1 | 691.13 | Local |
| M3 | 07/25/2012 0:00 | 21 | 49.532100 | -110.418380 | 542086 | 5486772 | 1 | 1125.07 | Local |
| M3 | 07/26/2012 0:00 | 0 | 49.541740 | -110.423110 | 541736 | 5487841 | 1 | 61.78 | Local |
| M3 | 07/26/2012 0:00 | 3 | 49.541640 | -110.422270 | 541796 | 5487831 | 0 | 683.50 | Local |
| M3 | 07/26/2012 0:00 | 18 | 49.540110 | -110.413120 | 542460 | 5487666 | 1 | 167.24 | Local |
| M3 | 07/26/2012 0:00 | 21 | 49.538780 | -110.412040 | 542539 | 5487518 | 1 | 691.99 | Local |
| M3 | 07/27/2012 0:00 | 0 | 49.541380 | -110.420730 | 541908 | 5487802 | 0 | 1176.61 | Local |
| M3 | 07/27/2012 0:00 | 15 | 49.530810 | -110.421540 | 541858 | 5486627 | 1 | 5.73 | Local |
| M3 | 07/27/2012 0:00 | 18 | 49.530850 | -110.421590 | 541855 | 5486631 | 1 | 352.80 | Local |
| M3 | 07/27/2012 0:00 | 21 | 49.531000 | -110.416720 | 542207 | 5486651 | 1 | 1254.55 | Local |
| M3 | 07/28/2012 0:00 | 0 | 49.541440 | -110.423300 | 541722 | 5487808 | 1 | 59.49 | Local |
| M3 | 07/28/2012 0:00 | 3 | 49.541480 | -110.422480 | 541781 | 5487813 | 0 | 696.28 | Local |
| M3 | 07/28/2012 0:00 | 12 | 49.539970 | -110.413140 | 542458 | 5487650 | 1 | 15.31 | Local |
| M3 | 07/28/2012 0:00 | 15 | 49.540100 | -110.413070 | 542463 | 5487664 | 1 | 4.51 | Local |
| M3 | 07/28/2012 0:00 | 18 | 49.540060 | -110.413080 | 542463 | 5487660 | 1 | 150.42 | Local |
| M3 | 07/28/2012 0:00 | 21 | 49.539740 | -110.415100 | 542317 | 5487623 | 1 | 565.41 | Local |
| M3 | 07/29/2012 0:00 | 0 | 49.541450 | -110.422460 | 541783 | 5487809 | 1 | 8.58 | Local |
| M3 | 07/29/2012 0:00 | 3 | 49.541380 | -110.422410 | 541787 | 5487802 | 1 | 1523.06 | Local |
| M3 | 07/29/2012 0:00 | 6 | 49.531220 | -110.408290 | 542817 | 5486680 | 0 | 112.65 | Local |
| M3 | 07/29/2012 0:00 | 15 | 49.532190 | -110.408740 | 542783 | 5486788 | 0 | 576.34 | Local |
| M3 | 07/29/2012 0:00 | 21 | 49.535500 | -110.402610 | 543224 | 5487159 | 1 | 2565.89 | Local |
| M3 | 07/30/2012 0:00 | 0 | 49.554560 | -110.382610 | 544654 | 5489290 | 1 | 1165.21 | Local |
| M3 | 07/30/2012 0:00 | 3 | 49.561140 | -110.395150 | 543741 | 5490014 | 0 | 3279.13 | Local |
| M3 | 07/30/2012 0:00 | 9 | 49.569880 | -110.438460 | 540602 | 5490961 | 1 | 19.12 | Local |
| M3 | 07/30/2012 0:00 | 12 | 49.569710 | -110.438500 | 540599 | 5490942 | 0 | 164.17 | Local |
| M3 | 07/30/2012 0:00 | 18 | 49.570610 | -110.440300 | 540468 | 5491041 | 1 | 321.46 | Local |
| M3 | 07/30/2012 0:00 | 21 | 49.572010 | -110.444190 | 540186 | 5491195 | 1 | 2631.47 | Local |
| M3 | 07/31/2012 0:00 | 0 | 49.593620 | -110.459040 | 539095 | 5493590 | 1 | 2940.03 | Local |
| M3 | 07/31/2012 0:00 | 3 | 49.619680 | -110.465950 | 538575 | 5496483 | 1 | 2001.41 | Local |
| M3 | 07/31/2012 0:00 | 6 | 49.626570 | -110.491550 | 536721 | 5497237 | 1 | 65.63 | Local |
| M3 | 07/31/2012 0:00 | 9 | 49.625980 | -110.491580 | 536719 | 5497171 | 1 | 25.55 | Local |
| M3 | 07/31/2012 0:00 | 12 | 49.626160 | -110.491360 | 536735 | 5497191 | 0 | NA | Local |
| M6 | 02/01/2012 0:00 | 18 | 49.710260 | -109.417570 | 614086 | 5507619 | 1 | 2711.46 | Trans |
| M6 | 02/01/2012 0:00 | 21 | 49.694170 | -109.445820 | 612086 | 5505787 | 1 | 2657.61 | Trans |
| M6 | 02/02/2012 0:00 | 0 | 49.674570 | -109.466900 | 610611 | 5503577 | 1 | 1944.22 | Trans |
| M6 | 02/02/2012 0:00 | 3 | 49.657260 | -109.470700 | 610376 | 5501647 | 1 | 1147.59 | Trans |
| M6 | 02/02/2012 0:00 | 6 | 49.646940 | -109.470870 | 610387 | 5500500 | 1 | 1987.64 | Trans |
| M6 | 02/02/2012 0:00 | 9 | 49.629110 | -109.468910 | 610568 | 5498520 | 1 | 86.40 | Trans |
| M6 | 02/02/2012 0:00 | 12 | 49.629880 | -109.468750 | 610578 | 5498606 | 0 | 724.91 | Trans |
| M6 | 02/02/2012 0:00 | 18 | 49.623400 | -109.469850 | 610514 | 5497884 | 1 | 2710.53 | Trans |
| M6 | 02/02/2012 0:00 | 21 | 49.603410 | -109.491320 | 609007 | 5495631 | 0 | 6645.42 | Trans |
| M6 | 02/03/2012 0:00 | 3 | 49.604300 | -109.583270 | 602362 | 5495600 | 1 | 7636.80 | Trans |
| M6 | 02/03/2012 0:00 | 6 | 49.614590 | -109.687770 | 594792 | 5496607 | 1 | 2074.09 | Trans |
| M6 | 02/03/2012 0:00 | 9 | 49.633120 | -109.684470 | 594994 | 5498672 | 1 | 4.87 | Trans |
| M6 | 02/03/2012 0:00 | 12 | 49.633140 | -109.684530 | 594990 | 5498674 | 1 | 3.98 | Trans |
| M6 | 02/03/2012 0:00 | 15 | 49.633110 | -109.684560 | 594988 | 5498670 | 0 | 2147.79 | Trans |
| M6 | 02/03/2012 0:00 | 21 | 49.652210 | -109.689000 | 594630 | 5500788 | 1 | 4203.12 | Trans |
| M6 | 02/04/2012 0:00 | 0 | 49.683790 | -109.721010 | 592260 | 5504259 | 1 | 771.94 | Trans |
| M6 | 02/04/2012 0:00 | 3 | 49.686830 | -109.730630 | 591560 | 5504585 | 1 | 4859.91 | Trans |
| M6 | 02/04/2012 0:00 | 6 | 49.697180 | -109.796090 | 586820 | 5505658 | 1 | 9053.49 | Trans |
| M6 | 02/04/2012 0:00 | 9 | 49.683170 | -109.919730 | 577927 | 5503965 | 0 | 327.09 | Trans |
| M6 | 02/04/2012 0:00 | 15 | 49.682940 | -109.915210 | 578253 | 5503944 | 0 | 1776.57 | Trans |
| M6 | 02/04/2012 0:00 | 21 | 49.683290 | -109.939830 | 576477 | 5503958 | 0 | 2498.54 | Trans |
| M6 | 02/05/2012 0:00 | 3 | 49.674590 | -109.971760 | 574187 | 5502959 | 1 | 2382.21 | Trans |

| | | | | | | | | | |
|---|---|---|---|---|---|---|---|---|---|
| M6 | 02/05/2012 0:00 | 6 | 49.661480 | -109.997870 | 572322 | 5501476 | 1 | 28.50 | Trans |
| M6 | 02/05/2012 0:00 | 9 | 49.661520 | -109.998260 | 572294 | 5501480 | 0 | 592.92 | Trans |
| M6 | 02/05/2012 0:00 | 15 | 49.659610 | -109.990590 | 572850 | 5501275 | 1 | 17.06 | Trans |
| M6 | 02/05/2012 0:00 | 18 | 49.659460 | -109.990540 | 572854 | 5501258 | 1 | 557.94 | Trans |
| M6 | 02/05/2012 0:00 | 21 | 49.662600 | -109.996570 | 572414 | 5501601 | 1 | 2468.47 | Trans |
| M6 | 02/06/2012 0:00 | 0 | 49.658850 | -110.030280 | 569987 | 5501153 | 1 | 1151.23 | Trans |
| M6 | 02/06/2012 0:00 | 3 | 49.664720 | -110.043420 | 569030 | 5501793 | 1 | 379.55 | Trans |
| M6 | 02/06/2012 0:00 | 6 | 49.666570 | -110.047840 | 568709 | 5501995 | 1 | 1.82 | Trans |
| M6 | 02/06/2012 0:00 | 9 | 49.666560 | -110.047820 | 568710 | 5501994 | 1 | 342.89 | Trans |
| M6 | 02/06/2012 0:00 | 12 | 49.669320 | -110.049940 | 568553 | 5502299 | 1 | 96.52 | Trans |
| M6 | 02/06/2012 0:00 | 15 | 49.669460 | -110.051260 | 568458 | 5502313 | 1 | 69.61 | Trans |
| M6 | 02/06/2012 0:00 | 18 | 49.669800 | -110.052070 | 568399 | 5502350 | 1 | 585.39 | Trans |
| M6 | 02/06/2012 0:00 | 21 | 49.667230 | -110.059150 | 567892 | 5502058 | 1 | 2939.30 | Trans |
| M6 | 02/07/2012 0:00 | 0 | 49.682130 | -110.092800 | 565444 | 5503684 | 1 | 1551.16 | Trans |
| M6 | 02/07/2012 0:00 | 3 | 49.673280 | -110.109420 | 564256 | 5502686 | 1 | 1241.12 | Trans |
| M6 | 02/07/2012 0:00 | 6 | 49.667560 | -110.124190 | 563198 | 5502038 | 0 | 1356.12 | Trans |
| M6 | 02/07/2012 0:00 | 15 | 49.669390 | -110.142770 | 561855 | 5502226 | 1 | 112.28 | Trans |
| M6 | 02/07/2012 0:00 | 18 | 49.670260 | -110.143560 | 561797 | 5502322 | 1 | 4625.06 | Trans |
| M6 | 02/07/2012 0:00 | 21 | 49.699350 | -110.097730 | 565065 | 5505595 | 1 | 3683.04 | Trans |
| M6 | 02/08/2012 0:00 | 0 | 49.676200 | -110.061210 | 567731 | 5503053 | 1 | 684.80 | Trans |
| M6 | 02/08/2012 0:00 | 3 | 49.675790 | -110.051740 | 568414 | 5503016 | 1 | 7266.64 | Trans |
| M6 | 02/08/2012 0:00 | 6 | 49.711570 | -109.967430 | 574442 | 5507074 | 1 | 3656.93 | Trans |
| M6 | 02/08/2012 0:00 | 9 | 49.727060 | -109.922680 | 577644 | 5508842 | 1 | 6.71 | Trans |
| M6 | 02/08/2012 0:00 | 12 | 49.727000 | -109.922670 | 577645 | 5508835 | 0 | 4.23 | Trans |
| M6 | 02/08/2012 0:00 | 18 | 49.727020 | -109.922720 | 577641 | 5508837 | 1 | 4.86 | Trans |
| M6 | 02/08/2012 0:00 | 21 | 49.727040 | -109.922660 | 577645 | 5508839 | 1 | 0.72 | Trans |
| M6 | 02/09/2012 0:00 | 0 | 49.727040 | -109.922670 | 577645 | 5508839 | 1 | 1.82 | Trans |
| M6 | 02/09/2012 0:00 | 3 | 49.727030 | -109.922690 | 577643 | 5508838 | 1 | 10.60 | Trans |
| M6 | 02/09/2012 0:00 | 6 | 49.727110 | -109.922610 | 577649 | 5508847 | 1 | 26.71 | Trans |
| M6 | 02/09/2012 0:00 | 9 | 49.726900 | -109.922790 | 577636 | 5508824 | 0 | 20.28 | Trans |
| M6 | 02/09/2012 0:00 | 15 | 49.727050 | -109.922630 | 577647 | 5508841 | 1 | 39.29 | Trans |
| M6 | 02/09/2012 0:00 | 18 | 49.726810 | -109.923030 | 577619 | 5508814 | 1 | 39.02 | Trans |
| M6 | 02/09/2012 0:00 | 21 | 49.727060 | -109.922650 | 577646 | 5508842 | 1 | 4.51 | Trans |
| M6 | 02/10/2012 0:00 | 0 | 49.727100 | -109.922660 | 577645 | 5508846 | 1 | 7.29 | Trans |
| M6 | 02/10/2012 0:00 | 3 | 49.727090 | -109.922760 | 577638 | 5508845 | 1 | 9.82 | Trans |
| M6 | 02/10/2012 0:00 | 6 | 49.727030 | -109.922660 | 577645 | 5508838 | 1 | 35.27 | Trans |
| M6 | 02/10/2012 0:00 | 9 | 49.726790 | -109.922980 | 577623 | 5508811 | 1 | 35.27 | Trans |
| M6 | 02/10/2012 0:00 | 12 | 49.727030 | -109.922660 | 577645 | 5508838 | 0 | 26.45 | Trans |
| M6 | 02/10/2012 0:00 | 18 | 49.726850 | -109.922900 | 577628 | 5508818 | 0 | 29.82 | Trans |
| M6 | 02/11/2012 0:00 | 6 | 49.726730 | -109.923270 | 577602 | 5508804 | 1 | 14.56 | Trans |
| M6 | 02/11/2012 0:00 | 9 | 49.726810 | -109.923110 | 577613 | 5508813 | 0 | 39.97 | Trans |
| M6 | 02/11/2012 0:00 | 15 | 49.727020 | -109.922660 | 577645 | 5508837 | 1 | 41.10 | Trans |
| M6 | 02/11/2012 0:00 | 18 | 49.726770 | -109.923080 | 577615 | 5508809 | 1 | 41.13 | Trans |
| M6 | 02/11/2012 0:00 | 21 | 49.727040 | -109.922690 | 577643 | 5508839 | 1 | 5.72 | Trans |
| M6 | 02/12/2012 0:00 | 0 | 49.727000 | -109.922640 | 577647 | 5508835 | 1 | 4.91 | Trans |
| M6 | 02/12/2012 0:00 | 3 | 49.727030 | -109.922690 | 577643 | 5508838 | 1 | 33.62 | Trans |
| M6 | 02/12/2012 0:00 | 6 | 49.726810 | -109.922370 | 577667 | 5508814 | 1 | 30.29 | Trans |
| M6 | 02/12/2012 0:00 | 9 | 49.726820 | -109.922790 | 577636 | 5508815 | 1 | 179.70 | Trans |
| M6 | 02/12/2012 0:00 | 12 | 49.725980 | -109.924920 | 577484 | 5508719 | 1 | 197.20 | Trans |
| M6 | 02/12/2012 0:00 | 15 | 49.726970 | -109.922650 | 577646 | 5508832 | 1 | 40.06 | Trans |
| M6 | 02/12/2012 0:00 | 18 | 49.726800 | -109.923140 | 577611 | 5508812 | 1 | 51.19 | Trans |
| M6 | 02/12/2012 0:00 | 21 | 49.726790 | -109.922430 | 577662 | 5508812 | 0 | 3764.01 | Trans |
| M6 | 02/13/2012 0:00 | 6 | 49.693050 | -109.918170 | 578023 | 5505065 | 0 | 1429.64 | Local |
| M6 | 02/13/2012 0:00 | 12 | 49.680400 | -109.914620 | 578300 | 5503663 | 1 | 111.52 | Local |
| M6 | 02/13/2012 0:00 | 15 | 49.679900 | -109.913280 | 578397 | 5503608 | 1 | 5.88 | Local |

| | | | | | | | | | |
|---|---|---|---|---|---|---|---|---|---|
| M6 | 02/13/2012 0:00 | 18 | 49.679890 | -109.913360 | 578391 | 5503607 | 1 | 844.05 | Local |
| M6 | 02/13/2012 0:00 | 21 | 49.673250 | -109.907690 | 578811 | 5502875 | 1 | 1100.52 | Local |
| M6 | 02/14/2012 0:00 | 0 | 49.682940 | -109.910800 | 578571 | 5503949 | 1 | 275.76 | Local |
| M6 | 02/14/2012 0:00 | 3 | 49.682740 | -109.914610 | 578297 | 5503923 | 1 | 27.64 | Local |
| M6 | 02/14/2012 0:00 | 6 | 49.682500 | -109.914710 | 578290 | 5503896 | 1 | 587.61 | Local |
| M6 | 02/14/2012 0:00 | 9 | 49.677220 | -109.914360 | 578324 | 5503309 | 1 | 3127.77 | Local |
| M6 | 02/14/2012 0:00 | 12 | 49.652530 | -109.935130 | 576864 | 5500543 | 1 | 70.38 | Local |
| M6 | 02/14/2012 0:00 | 15 | 49.652220 | -109.934280 | 576926 | 5500509 | 1 | 20.46 | Local |
| M6 | 02/14/2012 0:00 | 18 | 49.652380 | -109.934420 | 576916 | 5500527 | 1 | 2649.48 | Local |
| M6 | 02/14/2012 0:00 | 21 | 49.640100 | -109.965870 | 574664 | 5499130 | 1 | 3194.78 | Local |
| M6 | 02/15/2012 0:00 | 0 | 49.658280 | -110.000140 | 572163 | 5501118 | 1 | 641.75 | Local |
| M6 | 02/15/2012 0:00 | 3 | 49.662340 | -110.006460 | 571701 | 5501563 | 1 | 2273.72 | Local |
| M6 | 02/15/2012 0:00 | 6 | 49.658810 | -109.975430 | 573946 | 5501201 | 1 | 683.92 | Local |
| M6 | 02/15/2012 0:00 | 9 | 49.658360 | -109.965980 | 574628 | 5501160 | 0 | 44.48 | Local |
| M6 | 02/15/2012 0:00 | 15 | 49.657960 | -109.965990 | 574628 | 5501116 | 1 | 335.10 | Local |
| M6 | 02/15/2012 0:00 | 18 | 49.659910 | -109.962450 | 574881 | 5501336 | 1 | 7837.22 | Local |
| M6 | 02/15/2012 0:00 | 21 | 49.686370 | -109.861780 | 582101 | 5504383 | 1 | 4975.79 | Local |
| M6 | 02/16/2012 0:00 | 0 | 49.697690 | -109.795040 | 586895 | 5505716 | 1 | 429.38 | Local |
| M6 | 02/16/2012 0:00 | 3 | 49.697430 | -109.789100 | 587324 | 5505694 | 1 | 1785.05 | Local |
| M6 | 02/16/2012 0:00 | 6 | 49.699710 | -109.813600 | 585553 | 5505920 | 1 | 7201.32 | Local |
| M6 | 02/16/2012 0:00 | 9 | 49.684890 | -109.910790 | 578569 | 5504166 | 1 | 585.85 | Local |
| M6 | 02/16/2012 0:00 | 12 | 49.680020 | -109.913890 | 578353 | 5503621 | 0 | 29.89 | Local |
| M6 | 02/16/2012 0:00 | 18 | 49.679950 | -109.913490 | 578382 | 5503614 | 1 | 9.64 | Local |
| M6 | 02/16/2012 0:00 | 21 | 49.679930 | -109.913620 | 578373 | 5503611 | 1 | 29.86 | Local |
| M6 | 02/17/2012 0:00 | 0 | 49.680020 | -109.914010 | 578344 | 5503621 | 1 | 12.32 | Local |
| M6 | 02/17/2012 0:00 | 3 | 49.680030 | -109.913840 | 578357 | 5503622 | 1 | 281.59 | Local |
| M6 | 02/17/2012 0:00 | 6 | 49.682540 | -109.914360 | 578315 | 5503901 | 0 | 1034.97 | Local |
| M6 | 02/17/2012 0:00 | 12 | 49.673500 | -109.910940 | 578576 | 5502899 | 1 | 11.05 | Local |
| M6 | 02/17/2012 0:00 | 15 | 49.673480 | -109.911090 | 578565 | 5502897 | 1 | 13.71 | Local |
| M6 | 02/17/2012 0:00 | 18 | 49.673480 | -109.910900 | 578579 | 5502897 | 1 | 4338.43 | Local |
| M6 | 02/17/2012 0:00 | 21 | 49.670050 | -109.851010 | 582906 | 5502580 | 1 | 6014.49 | Local |
| M6 | 02/18/2012 0:00 | 0 | 49.705020 | -109.787400 | 587433 | 5506540 | 1 | 1028.22 | Local |
| M6 | 02/18/2012 0:00 | 3 | 49.713900 | -109.783420 | 587704 | 5507532 | 1 | 22.34 | Local |
| M6 | 02/18/2012 0:00 | 6 | 49.713700 | -109.783390 | 587706 | 5507510 | 1 | 423.79 | Local |
| M6 | 02/18/2012 0:00 | 9 | 49.717420 | -109.782110 | 587792 | 5507925 | 1 | 242.65 | Local |
| M6 | 02/18/2012 0:00 | 12 | 49.719340 | -109.780510 | 587904 | 5508140 | 0 | 30.38 | Local |
| M6 | 02/18/2012 0:00 | 18 | 49.719090 | -109.780680 | 587892 | 5508112 | 1 | 55.20 | Local |
| M6 | 02/18/2012 0:00 | 21 | 49.719190 | -109.781430 | 587838 | 5508122 | 1 | 267.51 | Local |
| M6 | 02/19/2012 0:00 | 0 | 49.720430 | -109.778250 | 588065 | 5508264 | 1 | 913.43 | Local |
| M6 | 02/19/2012 0:00 | 3 | 49.714140 | -109.786400 | 587489 | 5507555 | 1 | 3946.05 | Local |
| M6 | 02/19/2012 0:00 | 6 | 49.697580 | -109.834800 | 584028 | 5505659 | 0 | 5991.31 | Local |
| M6 | 02/19/2012 0:00 | 12 | 49.682610 | -109.914590 | 578298 | 5503908 | 1 | 280.80 | Local |
| M6 | 02/19/2012 0:00 | 15 | 49.680120 | -109.913940 | 578349 | 5503632 | 1 | 27.90 | Local |
| M6 | 02/19/2012 0:00 | 18 | 49.679970 | -109.913630 | 578372 | 5503616 | 0 | 304.89 | Local |
| M6 | 02/20/2012 0:00 | 0 | 49.682660 | -109.914450 | 578308 | 5503914 | 1 | 15.85 | Local |
| M6 | 02/20/2012 0:00 | 3 | 49.682790 | -109.914540 | 578302 | 5503928 | 1 | 21.56 | Local |
| M6 | 02/20/2012 0:00 | 6 | 49.682600 | -109.914600 | 578298 | 5503907 | 0 | 1032.02 | Local |
| M6 | 02/20/2012 0:00 | 12 | 49.673440 | -109.912290 | 578479 | 5502891 | 1 | 22.25 | Local |
| M6 | 02/20/2012 0:00 | 15 | 49.673640 | -109.912280 | 578479 | 5502913 | 1 | 4.95 | Local |
| M6 | 02/20/2012 0:00 | 18 | 49.673680 | -109.912250 | 578481 | 5502918 | 1 | 2001.69 | Local |
| M6 | 02/20/2012 0:00 | 21 | 49.687330 | -109.894160 | 579764 | 5504455 | 1 | 5330.69 | Local |
| M6 | 02/21/2012 0:00 | 0 | 49.704930 | -109.825410 | 584693 | 5506486 | 1 | 1901.62 | Local |
| M6 | 02/21/2012 0:00 | 3 | 49.720340 | -109.813970 | 585490 | 5508213 | 1 | 229.02 | Local |
| M6 | 02/21/2012 0:00 | 6 | 49.722080 | -109.812270 | 585610 | 5508408 | 1 | 17.67 | Local |
| M6 | 02/21/2012 0:00 | 9 | 49.722010 | -109.812490 | 585594 | 5508400 | 1 | 3.60 | Local |

| | | | | | | | | | |
|---|---|---|---|---|---|---|---|---|---|
| M6 | 02/21/2012 0:00 | 12 | 49.722010 | -109.812440 | 585598 | 5508400 | 1 | 7.78 | Local |
| M6 | 02/21/2012 0:00 | 15 | 49.722080 | -109.812440 | 585598 | 5508408 | 1 | 10.24 | Local |
| M6 | 02/21/2012 0:00 | 18 | 49.721990 | -109.812470 | 585596 | 5508398 | 1 | 48.15 | Local |
| M6 | 02/21/2012 0:00 | 21 | 49.722310 | -109.812020 | 585627 | 5508434 | 1 | 26.33 | Local |
| M6 | 02/22/2012 0:00 | 0 | 49.722350 | -109.812380 | 585601 | 5508438 | 0 | 15.73 | Local |
| M6 | 02/22/2012 0:00 | 6 | 49.722270 | -109.812560 | 585589 | 5508429 | 1 | 8642.28 | Local |
| M6 | 02/22/2012 0:00 | 9 | 49.682570 | -109.915590 | 578226 | 5503903 | 1 | 61.57 | Local |
| M6 | 02/22/2012 0:00 | 12 | 49.682520 | -109.914740 | 578288 | 5503898 | 1 | 23.10 | Local |
| M6 | 02/22/2012 0:00 | 15 | 49.682700 | -109.914580 | 578299 | 5503918 | 0 | 47.23 | Local |
| M6 | 02/22/2012 0:00 | 21 | 49.682920 | -109.914020 | 578339 | 5503943 | 0 | 1802.84 | Local |
| M6 | 02/23/2012 0:00 | 3 | 49.671340 | -109.931510 | 577096 | 5502638 | 1 | 430.36 | Local |
| M6 | 02/23/2012 0:00 | 6 | 49.667630 | -109.933210 | 576979 | 5502224 | 1 | 5.74 | Local |
| M6 | 02/23/2012 0:00 | 9 | 49.667680 | -109.933190 | 576980 | 5502229 | 1 | 7.95 | Local |
| M6 | 02/23/2012 0:00 | 12 | 49.667620 | -109.933250 | 576976 | 5502223 | 0 | 11.12 | Local |
| M6 | 02/23/2012 0:00 | 18 | 49.667720 | -109.933250 | 576976 | 5502234 | 1 | 2036.62 | Local |
| M6 | 02/23/2012 0:00 | 21 | 49.671950 | -109.905790 | 578950 | 5502732 | 1 | 6696.52 | Local |
| M6 | 02/24/2012 0:00 | 0 | 49.701590 | -109.824980 | 584729 | 5506116 | 1 | 1123.08 | Local |
| M6 | 02/24/2012 0:00 | 3 | 49.711570 | -109.822580 | 584885 | 5507228 | 1 | 37.70 | Local |
| M6 | 02/24/2012 0:00 | 6 | 49.711900 | -109.822460 | 584893 | 5507265 | 1 | 6377.44 | Local |
| M6 | 02/24/2012 0:00 | 9 | 49.682310 | -109.898210 | 579480 | 5503892 | 1 | 1339.70 | Local |
| M6 | 02/24/2012 0:00 | 12 | 49.673520 | -109.910910 | 578578 | 5502902 | 1 | 25.50 | Local |
| M6 | 02/24/2012 0:00 | 15 | 49.673740 | -109.910810 | 578585 | 5502926 | 1 | 21.72 | Local |
| M6 | 02/24/2012 0:00 | 18 | 49.673550 | -109.910880 | 578581 | 5502905 | 1 | 615.02 | Local |
| M6 | 02/24/2012 0:00 | 21 | 49.677290 | -109.917160 | 578121 | 5503314 | 1 | 3674.19 | Local |
| M6 | 02/25/2012 0:00 | 0 | 49.704220 | -109.887640 | 580207 | 5506339 | 1 | 4618.34 | Local |
| M6 | 02/25/2012 0:00 | 3 | 49.716350 | -109.826380 | 584603 | 5507755 | 1 | 898.37 | Local |
| M6 | 02/25/2012 0:00 | 6 | 49.714150 | -109.814390 | 585471 | 5507524 | 1 | 215.93 | Local |
| M6 | 02/25/2012 0:00 | 9 | 49.716070 | -109.814840 | 585435 | 5507737 | 1 | 3.60 | Local |
| M6 | 02/25/2012 0:00 | 12 | 49.716070 | -109.814890 | 585432 | 5507737 | 1 | 2.43 | Local |
| M6 | 02/25/2012 0:00 | 15 | 49.716060 | -109.814860 | 585434 | 5507736 | 1 | 8.01 | Local |
| M6 | 02/25/2012 0:00 | 18 | 49.716010 | -109.814940 | 585428 | 5507730 | 1 | 1588.50 | Local |
| M6 | 02/25/2012 0:00 | 21 | 49.703300 | -109.825000 | 584725 | 5506306 | 1 | 7130.34 | Local |
| M6 | 02/26/2012 0:00 | 0 | 49.676270 | -109.914640 | 578305 | 5503203 | 1 | 370.15 | Local |
| M6 | 02/26/2012 0:00 | 3 | 49.673830 | -109.911150 | 578561 | 5502936 | 1 | 35.94 | Local |
| M6 | 02/26/2012 0:00 | 6 | 49.673540 | -109.910930 | 578577 | 5502904 | 1 | 7.58 | Local |
| M6 | 02/26/2012 0:00 | 9 | 49.673480 | -109.910880 | 578581 | 5502897 | 1 | 868.92 | Local |
| M6 | 02/26/2012 0:00 | 12 | 49.669870 | -109.921560 | 577816 | 5502485 | 1 | 1394.68 | Local |
| M6 | 02/26/2012 0:00 | 15 | 49.659160 | -109.931620 | 577107 | 5501284 | 1 | 91.52 | Local |
| M6 | 02/26/2012 0:00 | 18 | 49.658960 | -109.930390 | 577196 | 5501263 | 1 | 414.83 | Local |
| M6 | 02/26/2012 0:00 | 21 | 49.660910 | -109.925490 | 577547 | 5501485 | 1 | 2132.09 | Local |
| M6 | 02/27/2012 0:00 | 0 | 49.665320 | -109.954240 | 575465 | 5501946 | 0 | 380.94 | Local |
| M6 | 02/27/2012 0:00 | 6 | 49.663370 | -109.949900 | 575781 | 5501733 | 1 | 9.60 | Local |
| M6 | 02/27/2012 0:00 | 9 | 49.663450 | -109.949850 | 575785 | 5501742 | 1 | 7.92 | Local |
| M6 | 02/27/2012 0:00 | 12 | 49.663380 | -109.949870 | 575783 | 5501734 | 1 | 3.63 | Local |
| M6 | 02/27/2012 0:00 | 15 | 49.663410 | -109.949850 | 575785 | 5501738 | 1 | 3.63 | Local |
| M6 | 02/27/2012 0:00 | 18 | 49.663440 | -109.949830 | 575786 | 5501741 | 1 | 2713.47 | Local |
| M6 | 02/27/2012 0:00 | 21 | 49.667520 | -109.912760 | 578455 | 5502233 | 1 | 2070.30 | Local |
| M6 | 02/28/2012 0:00 | 0 | 49.659880 | -109.886600 | 580355 | 5501411 | 1 | 8.91 | Local |
| M6 | 02/28/2012 0:00 | 3 | 49.659950 | -109.886660 | 580350 | 5501419 | 1 | 1.33 | Local |
| M6 | 02/28/2012 0:00 | 6 | 49.659960 | -109.886650 | 580351 | 5501420 | 1 | 5.97 | Local |
| M6 | 02/28/2012 0:00 | 9 | 49.660010 | -109.886680 | 580349 | 5501425 | 0 | 2256.01 | Local |
| M6 | 02/28/2012 0:00 | 21 | 49.663470 | -109.917480 | 578120 | 5501777 | 0 | 19.06 | Local |
| M6 | 02/29/2012 0:00 | 6 | 49.663500 | -109.917220 | 578139 | 5501781 | 0 | 14.60 | Local |
| M6 | 02/29/2012 0:00 | 12 | 49.663480 | -109.917020 | 578154 | 5501779 | 0 | 14.62 | Local |
| M6 | 02/29/2012 0:00 | 18 | 49.663350 | -109.917050 | 578152 | 5501765 | 0 | 13.42 | Local |

| | | | | | | | | | |
|---|---|---|---|---|---|---|---|---|---|
| M6 | 03/01/2012 0:00 | 3 | 49.663470 | -109.917030 | 578153 | 5501778 | 0 | 24.24 | Local |
| M6 | 03/01/2012 0:00 | 9 | 49.663260 | -109.916940 | 578160 | 5501755 | 1 | 33.04 | Local |
| M6 | 03/01/2012 0:00 | 12 | 49.663500 | -109.917210 | 578140 | 5501781 | 0 | 34.14 | Local |
| M6 | 03/01/2012 0:00 | 18 | 49.663280 | -109.916880 | 578164 | 5501757 | 1 | 27.08 | Local |
| M6 | 03/01/2012 0:00 | 21 | 49.663490 | -109.917070 | 578150 | 5501780 | 0 | 3.98 | Local |
| M6 | 03/02/2012 0:00 | 6 | 49.663460 | -109.917040 | 578152 | 5501777 | 1 | 5.47 | Local |
| M6 | 03/02/2012 0:00 | 9 | 49.663490 | -109.917100 | 578148 | 5501780 | 1 | 1.44 | Local |
| M6 | 03/02/2012 0:00 | 12 | 49.663490 | -109.917120 | 578146 | 5501780 | 0 | 21.61 | Local |
| M6 | 03/03/2012 0:00 | 0 | 49.663330 | -109.916950 | 578159 | 5501762 | 0 | 12.42 | Local |
| M6 | 03/03/2012 0:00 | 6 | 49.663440 | -109.916980 | 578157 | 5501775 | 0 | 12.93 | Local |
| M6 | 03/03/2012 0:00 | 18 | 49.663520 | -109.917110 | 578147 | 5501783 | 0 | 13.52 | Local |
| M6 | 03/04/2012 0:00 | 3 | 49.663400 | -109.917080 | 578149 | 5501770 | 1 | 10.38 | Local |
| M6 | 03/04/2012 0:00 | 6 | 49.663440 | -109.916950 | 578159 | 5501775 | 0 | 9.28 | Local |
| M6 | 03/04/2012 0:00 | 12 | 49.663510 | -109.916880 | 578164 | 5501782 | 0 | 23.75 | Local |
| M6 | 03/04/2012 0:00 | 18 | 49.663560 | -109.917200 | 578141 | 5501788 | 0 | 25.19 | Local |
| M6 | 03/05/2012 0:00 | 12 | 49.663370 | -109.917010 | 578155 | 5501767 | 0 | 13.66 | Local |
| M6 | 03/05/2012 0:00 | 18 | 49.663470 | -109.917120 | 578146 | 5501778 | 1 | 16.98 | Local |
| M6 | 03/05/2012 0:00 | 21 | 49.663380 | -109.916930 | 578160 | 5501768 | 0 | 19.28 | Local |
| M6 | 03/06/2012 0:00 | 6 | 49.663420 | -109.916670 | 578179 | 5501773 | 1 | 27.78 | Local |
| M6 | 03/06/2012 0:00 | 9 | 49.663460 | -109.917050 | 578152 | 5501777 | 0 | 10.17 | Local |
| M6 | 03/06/2012 0:00 | 15 | 49.663470 | -109.916910 | 578162 | 5501778 | 0 | 33.29 | Local |
| M6 | 03/07/2012 0:00 | 3 | 49.663560 | -109.917350 | 578130 | 5501788 | 1 | 120.73 | Local |
| M6 | 03/07/2012 0:00 | 6 | 49.662720 | -109.916290 | 578208 | 5501695 | 0 | 136.40 | Local |
| M6 | 03/07/2012 0:00 | 12 | 49.662290 | -109.918060 | 578081 | 5501646 | 1 | 140.28 | Local |
| M6 | 03/07/2012 0:00 | 15 | 49.663360 | -109.917030 | 578153 | 5501766 | 0 | 9.11 | Local |
| M6 | 03/07/2012 0:00 | 21 | 49.663410 | -109.917130 | 578146 | 5501771 | 1 | 14.81 | Local |
| M6 | 03/08/2012 0:00 | 15 | 49.650080 | -109.937330 | 576709 | 5500268 | 0 | 2638.78 | Trans |
| M6 | 03/08/2012 0:00 | 21 | 49.632210 | -109.961380 | 575000 | 5498257 | 1 | 2323.38 | Trans |
| M6 | 03/08/2012 0:00 | 0 | 49.663440 | -109.916930 | 578160 | 5501775 | 1 | 20.27 | Local |
| M6 | 03/08/2012 0:00 | 3 | 49.663300 | -109.917110 | 578147 | 5501759 | 0 | 28.05 | Local |
| M6 | 03/08/2012 0:00 | 9 | 49.663540 | -109.916990 | 578156 | 5501786 | 1 | 949.75 | Local |
| M6 | 03/08/2012 0:00 | 12 | 49.658400 | -109.927500 | 577405 | 5501203 | 1 | 1165.87 | Local |
| M6 | 03/09/2012 0:00 | 0 | 49.623220 | -109.990420 | 572917 | 5497229 | 0 | 779.47 | Trans |
| M6 | 03/09/2012 0:00 | 6 | 49.627860 | -109.998510 | 572326 | 5497737 | 0 | 91.09 | Trans |
| M6 | 03/09/2012 0:00 | 18 | 49.627170 | -109.999190 | 572278 | 5497660 | 0 | 16.35 | Trans |
| M6 | 03/10/2012 0:00 | 3 | 49.627250 | -109.999000 | 572291 | 5497669 | 1 | 49.36 | Trans |
| M6 | 03/10/2012 0:00 | 6 | 49.626820 | -109.998830 | 572304 | 5497621 | 1 | 47.15 | Trans |
| M6 | 03/10/2012 0:00 | 9 | 49.627150 | -109.999240 | 572274 | 5497658 | 0 | 40.97 | Trans |
| M6 | 03/10/2012 0:00 | 18 | 49.627440 | -109.998890 | 572299 | 5497690 | 0 | 5115.27 | Trans |
| M6 | 03/11/2012 0:00 | 0 | 49.648030 | -110.062240 | 567695 | 5499920 | 1 | 3783.94 | Trans |
| M6 | 03/11/2012 0:00 | 3 | 49.673240 | -110.097460 | 565119 | 5502692 | 1 | 640.65 | Trans |
| M6 | 03/11/2012 0:00 | 6 | 49.669180 | -110.103760 | 564670 | 5502235 | 1 | 5.17 | Trans |
| M6 | 03/11/2012 0:00 | 9 | 49.669170 | -110.103830 | 564665 | 5502234 | 0 | 73.94 | Trans |
| M6 | 03/11/2012 0:00 | 15 | 49.669630 | -110.103090 | 564718 | 5502286 | 0 | 583.93 | Trans |
| M6 | 03/11/2012 0:00 | 21 | 49.669750 | -110.111180 | 564134 | 5502292 | 1 | 5125.38 | Trans |
| M6 | 03/12/2012 0:00 | 0 | 49.689030 | -110.175710 | 559454 | 5504383 | 0 | 366.73 | Trans |
| M6 | 03/12/2012 0:00 | 12 | 49.689910 | -110.180610 | 559100 | 5504477 | 0 | 244.73 | Trans |
| M6 | 03/12/2012 0:00 | 18 | 49.689300 | -110.177350 | 559336 | 5504411 | 1 | 7.95 | Trans |
| M6 | 03/12/2012 0:00 | 21 | 49.689270 | -110.177450 | 559328 | 5504408 | 1 | 6.86 | Trans |
| M6 | 03/13/2012 0:00 | 0 | 49.689290 | -110.177360 | 559335 | 5504410 | 0 | 13.23 | Trans |
| M6 | 03/13/2012 0:00 | 6 | 49.689200 | -110.177240 | 559344 | 5504400 | 1 | 372.55 | Trans |
| M6 | 03/13/2012 0:00 | 9 | 49.690890 | -110.181700 | 559020 | 5504585 | 0 | 260.38 | Trans |
| M6 | 03/13/2012 0:00 | 21 | 49.691380 | -110.178170 | 559274 | 5504642 | 1 | 4641.94 | Trans |
| M6 | 03/14/2012 0:00 | 0 | 49.684970 | -110.241760 | 554695 | 5503881 | 1 | 1350.85 | Trans |
| M6 | 03/14/2012 0:00 | 3 | 49.680630 | -110.259250 | 553438 | 5503386 | 1 | 2522.91 | Trans |

| | | | | | | | | | |
|---|---|---|---|---|---|---|---|---|---|
| M6 | 03/14/2012 0:00 | 6 | 49.659560 | -110.272230 | 552524 | 5501034 | 1 | 1109.83 | Trans |
| M6 | 03/14/2012 0:00 | 9 | 49.652280 | -110.282750 | 551773 | 5500218 | 0 | 33.85 | Trans |
| M6 | 03/14/2012 0:00 | 18 | 49.651980 | -110.282670 | 551779 | 5500184 | 0 | 5206.32 | Trans |
| M6 | 03/15/2012 0:00 | 0 | 49.659230 | -110.353930 | 546628 | 5500944 | 1 | 3059.43 | Trans |
| M6 | 03/15/2012 0:00 | 3 | 49.654330 | -110.395640 | 543622 | 5500374 | 1 | 698.43 | Trans |
| M6 | 03/15/2012 0:00 | 6 | 49.648420 | -110.398920 | 543391 | 5499715 | 0 | 724.14 | Trans |
| M6 | 03/15/2012 0:00 | 12 | 49.643620 | -110.405700 | 542906 | 5499178 | 1 | 39.84 | Trans |
| M6 | 03/15/2012 0:00 | 15 | 49.643740 | -110.406220 | 542868 | 5499191 | 1 | 11.93 | Trans |
| M6 | 03/15/2012 0:00 | 18 | 49.643640 | -110.406160 | 542872 | 5499179 | 1 | 2325.09 | Trans |
| M6 | 03/15/2012 0:00 | 21 | 49.645330 | -110.438260 | 540554 | 5499350 | 1 | 2149.96 | Trans |
| M6 | 03/16/2012 0:00 | 0 | 49.647140 | -110.467910 | 538412 | 5499535 | 1 | 2402.36 | Trans |
| M6 | 03/16/2012 0:00 | 3 | 49.650830 | -110.500700 | 536042 | 5499929 | 1 | 1435.66 | Trans |
| M6 | 03/16/2012 0:00 | 6 | 49.654710 | -110.481730 | 537408 | 5500370 | 0 | 1046.96 | Trans |
| M6 | 03/16/2012 0:00 | 12 | 49.648880 | -110.470340 | 538235 | 5499727 | 0 | 2473.10 | Trans |
| M6 | 03/16/2012 0:00 | 21 | 49.670640 | -110.477450 | 537705 | 5502143 | 1 | 9218.30 | Trans |
| M6 | 03/17/2012 0:00 | 0 | 49.750450 | -110.512100 | 535147 | 5510999 | 1 | 2905.09 | Trans |
| M6 | 03/17/2012 0:00 | 3 | 49.772210 | -110.534430 | 533523 | 5513408 | 1 | 7805.46 | Trans |
| M6 | 03/17/2012 0:00 | 6 | 49.823200 | -110.608980 | 528126 | 5519047 | 1 | 4657.43 | Trans |
| M6 | 03/17/2012 0:00 | 9 | 49.782660 | -110.625280 | 526976 | 5514534 | 0 | 54.49 | Trans |
| M6 | 03/17/2012 0:00 | 15 | 49.782240 | -110.625670 | 526948 | 5514487 | 1 | 3.09 | Trans |
| M6 | 03/17/2012 0:00 | 18 | 49.782230 | -110.625710 | 526945 | 5514486 | 1 | 2850.90 | Trans |
| M6 | 03/17/2012 0:00 | 21 | 49.756680 | -110.622360 | 527201 | 5511646 | 1 | 1937.14 | Trans |
| M6 | 03/18/2012 0:00 | 0 | 49.739690 | -110.616400 | 527640 | 5509760 | 1 | 2522.13 | Trans |
| M6 | 03/18/2012 0:00 | 3 | 49.724790 | -110.590010 | 529550 | 5508113 | 1 | 9406.43 | Trans |
| M6 | 03/18/2012 0:00 | 6 | 49.678000 | -110.481330 | 537419 | 5502959 | 1 | 2566.80 | Trans |
| M6 | 03/18/2012 0:00 | 9 | 49.656290 | -110.469230 | 538309 | 5500552 | 1 | 26.72 | Trans |
| M6 | 03/18/2012 0:00 | 12 | 49.656530 | -110.469250 | 538308 | 5500579 | 1 | 15.52 | Trans |
| M6 | 03/18/2012 0:00 | 15 | 49.656500 | -110.469460 | 538292 | 5500575 | 1 | 2.65 | Trans |
| M6 | 03/18/2012 0:00 | 18 | 49.656480 | -110.469440 | 538294 | 5500573 | 1 | 977.10 | Trans |
| M6 | 03/18/2012 0:00 | 21 | 49.647960 | -110.466120 | 538540 | 5499627 | 1 | 5882.14 | Trans |
| M6 | 03/19/2012 0:00 | 0 | 49.629010 | -110.390060 | 544048 | 5497562 | 1 | 2414.65 | Trans |
| M6 | 03/19/2012 0:00 | 3 | 49.617850 | -110.361380 | 546130 | 5496339 | 1 | 712.84 | Trans |
| M6 | 03/19/2012 0:00 | 6 | 49.612800 | -110.355300 | 546574 | 5495781 | 1 | 2190.56 | Trans |
| M6 | 03/19/2012 0:00 | 9 | 49.601420 | -110.330550 | 548373 | 5494532 | 0 | 378.04 | Trans |
| M6 | 03/20/2012 0:00 | 3 | 49.598020 | -110.330610 | 548372 | 5494153 | 1 | 953.88 | Trans |
| M6 | 03/20/2012 0:00 | 6 | 49.606190 | -110.326580 | 548655 | 5495064 | 1 | 1127.76 | Trans |
| M6 | 03/20/2012 0:00 | 9 | 49.600030 | -110.338980 | 547765 | 5494372 | 1 | 47.19 | Trans |
| M6 | 03/20/2012 0:00 | 12 | 49.599660 | -110.339300 | 547742 | 5494330 | 0 | 810.86 | Trans |
| M6 | 03/21/2012 0:00 | 0 | 49.600420 | -110.328140 | 548548 | 5494422 | 1 | 2162.82 | Trans |
| M6 | 03/21/2012 0:00 | 3 | 49.612150 | -110.304260 | 550261 | 5495742 | 0 | 7698.06 | Trans |
| M6 | 03/22/2012 0:00 | 18 | 49.669700 | -110.244980 | 554480 | 5502181 | 1 | 2315.07 | Trans |
| M6 | 03/22/2012 0:00 | 21 | 49.682440 | -110.219600 | 556296 | 5503616 | 1 | 5159.05 | Trans |
| M6 | 03/23/2012 0:00 | 0 | 49.702740 | -110.155280 | 560911 | 5505923 | 1 | 2254.41 | Trans |
| M6 | 03/23/2012 0:00 | 3 | 49.683810 | -110.144080 | 561742 | 5503828 | 1 | 486.38 | Trans |
| M6 | 03/23/2012 0:00 | 6 | 49.681010 | -110.138900 | 562119 | 5503521 | 1 | 1259.95 | Trans |
| M6 | 03/23/2012 0:00 | 9 | 49.669780 | -110.141240 | 561965 | 5502270 | 1 | 634.46 | Trans |
| M6 | 03/23/2012 0:00 | 12 | 49.666760 | -110.133780 | 562507 | 5501941 | 0 | 245.17 | Trans |
| M6 | 03/23/2012 0:00 | 18 | 49.666210 | -110.130490 | 562745 | 5501882 | 1 | 1190.86 | Trans |
| M6 | 03/23/2012 0:00 | 21 | 49.671700 | -110.116320 | 563761 | 5502505 | 1 | 4996.60 | Trans |
| M6 | 03/24/2012 0:00 | 0 | 49.677650 | -110.047680 | 568705 | 5503227 | 1 | 5684.24 | Trans |
| M6 | 03/24/2012 0:00 | 3 | 49.656160 | -109.976210 | 573893 | 5500905 | 1 | 58.50 | Trans |
| M6 | 03/24/2012 0:00 | 6 | 49.656610 | -109.976630 | 573862 | 5500955 | 1 | 1233.04 | Trans |
| M6 | 03/24/2012 0:00 | 9 | 49.659950 | -109.960340 | 575033 | 5501342 | 1 | 1.11 | Trans |
| M6 | 03/24/2012 0:00 | 12 | 49.659960 | -109.960340 | 575033 | 5501344 | 0 | 3.64 | Trans |
| M6 | 03/24/2012 0:00 | 18 | 49.659980 | -109.960300 | 575036 | 5501346 | 1 | 3455.13 | Trans |

| | | | | | | | | | |
|---|---|---|---|---|---|---|---|---|---|
| M6 | 03/24/2012 0:00 | 21 | 49.670570 | -109.915290 | 578267 | 5502569 | 1 | 6554.33 | Local |
| M6 | 03/25/2012 0:00 | 0 | 49.704100 | -109.840560 | 583602 | 5506377 | 1 | 1118.43 | Local |
| M6 | 03/25/2012 0:00 | 3 | 49.702340 | -109.825290 | 584706 | 5506199 | 1 | 33.34 | Local |
| M6 | 03/25/2012 0:00 | 6 | 49.702310 | -109.824830 | 584739 | 5506196 | 0 | 1600.84 | Local |
| M6 | 03/25/2012 0:00 | 18 | 49.715200 | -109.814940 | 585429 | 5507640 | 1 | 41.66 | Local |
| M6 | 03/25/2012 0:00 | 21 | 49.715560 | -109.814780 | 585440 | 5507680 | 0 | 41.58 | Local |
| M6 | 03/26/2012 0:00 | 3 | 49.715900 | -109.814540 | 585457 | 5507718 | 1 | 937.22 | Local |
| M6 | 03/26/2012 0:00 | 6 | 49.709900 | -109.823670 | 584809 | 5507041 | 1 | 7198.81 | Local |
| M6 | 03/26/2012 0:00 | 9 | 49.680980 | -109.912970 | 578418 | 5503729 | 0 | 270.18 | Local |
| M6 | 03/26/2012 0:00 | 15 | 49.678550 | -109.912970 | 578422 | 5503459 | 1 | 333.98 | Local |
| M6 | 03/26/2012 0:00 | 18 | 49.681240 | -109.915030 | 578269 | 5503756 | 0 | 83.37 | Local |
| M6 | 03/27/2012 0:00 | 9 | 49.680620 | -109.914380 | 578317 | 5503687 | 0 | 1798.46 | Local |
| M6 | 03/27/2012 0:00 | 21 | 49.694660 | -109.902000 | 579187 | 5505261 | 1 | 5071.77 | Local |
| M6 | 03/28/2012 0:00 | 0 | 49.710240 | -109.835900 | 583927 | 5507065 | 1 | 1027.38 | Local |
| M6 | 03/28/2012 0:00 | 3 | 49.711740 | -109.821840 | 584938 | 5507248 | 1 | 700.29 | Local |
| M6 | 03/28/2012 0:00 | 6 | 49.716010 | -109.814700 | 585445 | 5507730 | 1 | 5468.58 | Local |
| M6 | 03/28/2012 0:00 | 9 | 49.679840 | -109.866080 | 581802 | 5503652 | 0 | 3377.05 | Local |
| M6 | 03/29/2012 0:00 | 0 | 49.682090 | -109.912760 | 578431 | 5503852 | 1 | 751.00 | Local |
| M6 | 03/29/2012 0:00 | 3 | 49.680710 | -109.902570 | 579168 | 5503710 | 0 | 2377.70 | Local |
| M6 | 03/29/2012 0:00 | 15 | 49.660670 | -109.914070 | 578371 | 5501470 | 0 | 5144.29 | Local |
| M6 | 03/30/2012 0:00 | 3 | 49.653550 | -109.843650 | 583465 | 5500754 | 1 | 1474.26 | Local |
| M6 | 03/30/2012 0:00 | 6 | 49.660860 | -109.860690 | 582223 | 5501548 | 0 | 5676.87 | Local |
| M6 | 03/31/2012 0:00 | 3 | 49.705830 | -109.823430 | 584834 | 5506589 | 1 | 310.02 | Local |
| M6 | 03/31/2012 0:00 | 6 | 49.703310 | -109.825270 | 584705 | 5506307 | 0 | 6882.90 | Local |
| M6 | 03/31/2012 0:00 | 21 | 49.683580 | -109.915720 | 578215 | 5504015 | 0 | 2386.82 | Local |
| M6 | 04/01/2012 0:00 | 3 | 49.696470 | -109.889260 | 580103 | 5505476 | 1 | 1171.47 | Local |
| M6 | 04/01/2012 0:00 | 6 | 49.686920 | -109.882400 | 580613 | 5504422 | 0 | 2429.47 | Local |
| M6 | 04/01/2012 0:00 | 21 | 49.684120 | -109.915800 | 578209 | 5504075 | 0 | 1402.70 | Local |
| M6 | 04/02/2012 0:00 | 15 | 49.672270 | -109.909130 | 578709 | 5502764 | 0 | 677.30 | Local |
| M6 | 04/02/2012 0:00 | 21 | 49.671400 | -109.899840 | 579381 | 5502678 | 0 | 5020.98 | Local |
| M6 | 04/03/2012 0:00 | 3 | 49.700340 | -109.846410 | 583186 | 5505953 | 1 | 3843.47 | Local |
| M6 | 04/03/2012 0:00 | 6 | 49.724940 | -109.808960 | 585843 | 5508730 | 0 | 363.10 | Local |
| M6 | 04/03/2012 0:00 | 21 | 49.722120 | -109.811500 | 585665 | 5508413 | 0 | 114.17 | Local |
| M6 | 04/04/2012 0:00 | 6 | 49.721340 | -109.812530 | 585592 | 5508325 | 0 | 791.96 | Local |
| M6 | 04/04/2012 0:00 | 21 | 49.714890 | -109.817190 | 585268 | 5507603 | 1 | 7110.94 | Local |
| M6 | 04/05/2012 0:00 | 0 | 49.681150 | -109.900950 | 579285 | 5503760 | 0 | 3142.63 | Local |
| M6 | 04/05/2012 0:00 | 9 | 49.660180 | -109.930150 | 577211 | 5501399 | 0 | 1152.03 | Local |
| M6 | 04/05/2012 0:00 | 15 | 49.649820 | -109.929900 | 577246 | 5500247 | 0 | 14296.92 | Local |
| M6 | 04/08/2012 0:00 | 6 | 49.586430 | -109.757710 | 589792 | 5493391 | 0 | 125.40 | Local |
| M6 | 04/09/2012 0:00 | 0 | 49.586620 | -109.759420 | 589668 | 5493410 | 0 | 315.11 | Local |
| M6 | 04/09/2012 0:00 | 6 | 49.583860 | -109.758430 | 589744 | 5493104 | 0 | 699.19 | Local |
| M6 | 04/09/2012 0:00 | 21 | 49.585760 | -109.767650 | 589074 | 5493305 | 0 | 155.77 | Local |
| M6 | 04/10/2012 0:00 | 3 | 49.584360 | -109.767730 | 589071 | 5493149 | 0 | 4684.48 | Local |
| M6 | 04/11/2012 0:00 | 0 | 49.620410 | -109.801280 | 586582 | 5497118 | 1 | 26.68 | Local |
| M6 | 04/11/2012 0:00 | 3 | 49.620550 | -109.801580 | 586560 | 5497133 | 1 | 222.83 | Local |
| M6 | 04/11/2012 0:00 | 6 | 49.620250 | -109.798530 | 586781 | 5497103 | 0 | 341.90 | Local |
| M6 | 04/11/2012 0:00 | 21 | 49.620140 | -109.803260 | 586440 | 5497085 | 0 | 3241.00 | Local |
| M6 | 04/12/2012 0:00 | 3 | 49.633640 | -109.843030 | 583544 | 5498541 | 0 | 7718.18 | Local |
| M6 | 04/13/2012 0:00 | 21 | 49.683830 | -109.916900 | 578130 | 5504042 | 1 | 2468.97 | Local |
| M6 | 04/14/2012 0:00 | 0 | 49.701310 | -109.895790 | 579624 | 5506007 | 1 | 2439.52 | Local |
| M6 | 04/14/2012 0:00 | 3 | 49.723060 | -109.891340 | 579909 | 5508430 | 1 | 71.82 | Local |
| M6 | 04/14/2012 0:00 | 6 | 49.722430 | -109.891120 | 579926 | 5508360 | 1 | 4744.69 | Local |
| M6 | 04/14/2012 0:00 | 9 | 49.682800 | -109.915520 | 578231 | 5503928 | 0 | 6473.88 | Local |
| M6 | 04/16/2012 0:00 | 3 | 49.735220 | -109.876440 | 580963 | 5509798 | 1 | 2088.62 | Local |
| M6 | 04/16/2012 0:00 | 6 | 49.717540 | -109.886230 | 580286 | 5507822 | 0 | 4585.62 | Local |

| | | | | | | | | | |
|---|---|---|---|---|---|---|---|---|---|
| M6 | 04/17/2012 0:00 | 6 | 49.684050 | -109.923340 | 577665 | 5504059 | 0 | 1624.78 | Local |
| M6 | 04/17/2012 0:00 | 21 | 49.684260 | -109.900820 | 579289 | 5504106 | 1 | 4562.53 | Local |
| M6 | 04/18/2012 0:00 | 0 | 49.706870 | -109.848030 | 583058 | 5506677 | 1 | 2355.25 | Local |
| M6 | 04/18/2012 0:00 | 3 | 49.714160 | -109.878700 | 580835 | 5507454 | 1 | 4378.00 | Local |
| M6 | 04/18/2012 0:00 | 6 | 49.683100 | -109.916010 | 578195 | 5503961 | 0 | 90.14 | Local |
| M6 | 04/18/2012 0:00 | 21 | 49.683200 | -109.914770 | 578284 | 5503974 | 1 | 4110.84 | Local |
| M6 | 04/19/2012 0:00 | 0 | 49.713840 | -109.882870 | 580535 | 5507414 | 0 | 3954.90 | Local |
| M6 | 04/19/2012 0:00 | 6 | 49.686630 | -109.918190 | 578032 | 5504351 | 0 | 789.62 | Local |
| M6 | 04/19/2012 0:00 | 21 | 49.680270 | -109.913320 | 578394 | 5503649 | 0 | 1230.31 | Local |
| M6 | 04/20/2012 0:00 | 3 | 49.671750 | -109.902440 | 579192 | 5502714 | 0 | 1832.84 | Local |
| M6 | 04/20/2012 0:00 | 15 | 49.669540 | -109.927610 | 577380 | 5502442 | 0 | 3089.73 | Local |
| M6 | 04/21/2012 0:00 | 0 | 49.695750 | -109.941840 | 576312 | 5505341 | 1 | 593.48 | Local |
| M6 | 04/21/2012 0:00 | 3 | 49.697700 | -109.949500 | 575757 | 5505550 | 0 | 3305.53 | Local |
| M6 | 04/22/2012 0:00 | 0 | 49.678200 | -109.984090 | 573291 | 5503348 | 1 | 5087.56 | Local |
| M6 | 04/23/2012 0:00 | 3 | 49.679140 | -109.913590 | 578376 | 5503524 | 1 | 1136.63 | Local |
| M6 | 04/23/2012 0:00 | 6 | 49.670870 | -109.922850 | 577721 | 5502595 | 0 | 4436.34 | Local |
| M6 | 04/24/2012 0:00 | 0 | 49.710740 | -109.920470 | 577829 | 5507030 | 0 | 53.84 | Local |
| M6 | 04/24/2012 0:00 | 6 | 49.710940 | -109.919790 | 577878 | 5507053 | 0 | 1869.97 | Local |
| M6 | 04/25/2012 0:00 | 0 | 49.701300 | -109.941040 | 576361 | 5505959 | 1 | 2333.45 | Local |
| M6 | 04/25/2012 0:00 | 3 | 49.719910 | -109.956000 | 575254 | 5508013 | 1 | 589.40 | Local |
| M6 | 04/25/2012 0:00 | 6 | 49.715480 | -109.951510 | 575584 | 5507525 | 0 | 2255.80 | Local |
| M6 | 04/25/2012 0:00 | 21 | 49.697540 | -109.936900 | 576665 | 5505545 | 1 | 1076.00 | Local |
| M6 | 04/26/2012 0:00 | 0 | 49.688780 | -109.943240 | 576222 | 5504565 | 1 | 4883.27 | Local |
| M6 | 04/26/2012 0:00 | 3 | 49.663060 | -109.888380 | 580221 | 5501762 | 1 | 1411.19 | Local |
| M6 | 04/26/2012 0:00 | 6 | 49.670600 | -109.904110 | 579074 | 5502584 | 1 | 731.80 | Local |
| M6 | 04/26/2012 0:00 | 9 | 49.674170 | -109.912630 | 578453 | 5502972 | 0 | 389.76 | Local |
| M6 | 04/26/2012 0:00 | 18 | 49.676970 | -109.915880 | 578214 | 5503280 | 1 | 372.50 | Local |
| M6 | 04/26/2012 0:00 | 21 | 49.676860 | -109.910720 | 578587 | 5503273 | 0 | 5749.94 | Local |
| M6 | 04/27/2012 0:00 | 6 | 49.722870 | -109.874320 | 581136 | 5508427 | 0 | 948.14 | Local |
| M6 | 04/28/2012 0:00 | 6 | 49.717400 | -109.884410 | 580418 | 5507808 | 0 | 4605.94 | Local |
| M6 | 04/28/2012 0:00 | 12 | 49.681240 | -109.915570 | 578230 | 5503755 | 0 | 4.51 | Local |
| M6 | 04/28/2012 0:00 | 18 | 49.681280 | -109.915580 | 578229 | 5503759 | 0 | 1513.38 | Local |
| M6 | 04/29/2012 0:00 | 0 | 49.691010 | -109.900910 | 579271 | 5504857 | 1 | 3658.69 | Local |
| M6 | 04/29/2012 0:00 | 3 | 49.719850 | -109.876480 | 580985 | 5508089 | 0 | NA | Local |
| M7 | 02/12/2012 0:00 | 21 | 49.777991 | -108.975024 | 645786 | 5515915 | 1 | 2772.96 | Trans |
| M7 | 02/13/2012 0:00 | 0 | 49.801444 | -108.961949 | 646657 | 5518547 | 1 | 166.77 | Trans |
| M7 | 02/13/2012 0:00 | 3 | 49.801877 | -108.959730 | 646815 | 5518600 | 1 | 63.03 | Trans |
| M7 | 02/13/2012 0:00 | 6 | 49.801412 | -108.960230 | 646780 | 5518547 | 1 | 475.30 | Trans |
| M7 | 02/13/2012 0:00 | 9 | 49.805002 | -108.963815 | 646512 | 5518939 | 1 | 2.23 | Trans |
| M7 | 02/13/2012 0:00 | 12 | 49.805004 | -108.963845 | 646509 | 5518939 | 1 | 15.65 | Trans |
| M7 | 02/13/2012 0:00 | 15 | 49.805130 | -108.963750 | 646516 | 5518954 | 1 | 32.33 | Trans |
| M7 | 02/13/2012 0:00 | 18 | 49.804951 | -108.964104 | 646491 | 5518933 | 1 | 589.76 | Trans |
| M7 | 02/13/2012 0:00 | 21 | 49.806027 | -108.956081 | 647065 | 5519068 | 0 | 6420.67 | Trans |
| M7 | 02/14/2012 0:00 | 3 | 49.836126 | -108.879932 | 652449 | 5522567 | 1 | 7246.73 | Trans |
| M7 | 02/14/2012 0:00 | 6 | 49.855054 | -108.783513 | 659320 | 5524871 | 1 | 2046.69 | Trans |
| M7 | 02/14/2012 0:00 | 9 | 49.861475 | -108.756836 | 661216 | 5525642 | 1 | 6.49 | Trans |
| M7 | 02/14/2012 0:00 | 12 | 49.861528 | -108.756872 | 661213 | 5525648 | 1 | 14.52 | Trans |
| M7 | 02/14/2012 0:00 | 15 | 49.861402 | -108.756924 | 661209 | 5525634 | 1 | 12.84 | Trans |
| M7 | 02/14/2012 0:00 | 18 | 49.861499 | -108.757021 | 661202 | 5525644 | 1 | 3423.33 | Trans |
| M7 | 02/14/2012 0:00 | 21 | 49.881976 | -108.721465 | 663688 | 5527998 | 1 | 1187.73 | Trans |
| M7 | 02/15/2012 0:00 | 0 | 49.883667 | -108.705146 | 664855 | 5528222 | 1 | 685.76 | Trans |
| M7 | 02/15/2012 0:00 | 3 | 49.883004 | -108.714633 | 664175 | 5528127 | 1 | 16.76 | Trans |
| M7 | 02/15/2012 0:00 | 6 | 49.882854 | -108.714632 | 664176 | 5528111 | 1 | 151.94 | Trans |
| M7 | 02/15/2012 0:00 | 9 | 49.881569 | -108.715351 | 664129 | 5527966 | 1 | 91.02 | Trans |
| M7 | 02/15/2012 0:00 | 12 | 49.881749 | -108.714115 | 664217 | 5527989 | 1 | 11.57 | Trans |

| | | | | | | | | | |
|---|---|---|---|---|---|---|---|---|---|
| M7 | 02/15/2012 0:00 | 15 | 49.881849 | -108.714068 | 664220 | 5528000 | 1 | 302.70 | Trans |
| M7 | 02/15/2012 0:00 | 18 | 49.883308 | -108.710512 | 664470 | 5528170 | 1 | 508.43 | Trans |
| M7 | 02/15/2012 0:00 | 21 | 49.881242 | -108.716823 | 664024 | 5527926 | 1 | 2367.06 | Trans |
| M7 | 02/16/2012 0:00 | 0 | 49.874094 | -108.685802 | 666277 | 5527200 | 1 | 1383.11 | Trans |
| M7 | 02/16/2012 0:00 | 3 | 49.863749 | -108.696480 | 665545 | 5526027 | 1 | 702.58 | Trans |
| M7 | 02/16/2012 0:00 | 6 | 49.857452 | -108.697246 | 665512 | 5525325 | 1 | 339.89 | Trans |
| M7 | 02/16/2012 0:00 | 9 | 49.859655 | -108.700521 | 665269 | 5525563 | 1 | 46.57 | Trans |
| M7 | 02/16/2012 0:00 | 12 | 49.859911 | -108.700008 | 665305 | 5525592 | 1 | 309.30 | Trans |
| M7 | 02/16/2012 0:00 | 15 | 49.857278 | -108.701392 | 665214 | 5525296 | 1 | 158.52 | Trans |
| M7 | 02/16/2012 0:00 | 18 | 49.855866 | -108.701692 | 665197 | 5525139 | 1 | 639.35 | Trans |
| M7 | 02/16/2012 0:00 | 21 | 49.851850 | -108.695330 | 665669 | 5524706 | 1 | 922.10 | Trans |
| M7 | 02/17/2012 0:00 | 0 | 49.859399 | -108.690027 | 666024 | 5525557 | 1 | 672.14 | Trans |
| M7 | 02/17/2012 0:00 | 3 | 49.865267 | -108.687792 | 666164 | 5526215 | 1 | 1271.98 | Trans |
| M7 | 02/17/2012 0:00 | 6 | 49.857735 | -108.701105 | 665233 | 5525348 | 1 | 297.50 | Trans |
| M7 | 02/17/2012 0:00 | 9 | 49.860244 | -108.699671 | 665328 | 5525630 | 0 | 11.91 | Trans |
| M7 | 02/17/2012 0:00 | 15 | 49.860326 | -108.699778 | 665320 | 5525639 | 1 | 401.53 | Trans |
| M7 | 02/17/2012 0:00 | 18 | 49.856838 | -108.701214 | 665229 | 5525248 | 1 | 2128.33 | Trans |
| M7 | 02/17/2012 0:00 | 21 | 49.872354 | -108.683888 | 666420 | 5527011 | 1 | 739.18 | Trans |
| M7 | 02/18/2012 0:00 | 0 | 49.878909 | -108.682192 | 666520 | 5527744 | 1 | 2253.53 | Trans |
| M7 | 02/18/2012 0:00 | 3 | 49.860553 | -108.695464 | 665629 | 5525674 | 1 | 739.05 | Trans |
| M7 | 02/18/2012 0:00 | 6 | 49.855559 | -108.702245 | 665159 | 5525103 | 1 | 1052.22 | Trans |
| M7 | 02/18/2012 0:00 | 9 | 49.864975 | -108.700832 | 665228 | 5526153 | 1 | 75.46 | Trans |
| M7 | 02/18/2012 0:00 | 12 | 49.864314 | -108.700595 | 665247 | 5526080 | 1 | 14.49 | Trans |
| M7 | 02/18/2012 0:00 | 15 | 49.864366 | -108.700780 | 665234 | 5526086 | 1 | 14.75 | Trans |
| M7 | 02/18/2012 0:00 | 18 | 49.864233 | -108.700784 | 665234 | 5526071 | 1 | 500.03 | Trans |
| M7 | 02/18/2012 0:00 | 21 | 49.868612 | -108.702361 | 665106 | 5526554 | 1 | 89.89 | Trans |
| M7 | 02/19/2012 0:00 | 0 | 49.869228 | -108.701551 | 665162 | 5526625 | 1 | 88.29 | Trans |
| M7 | 02/19/2012 0:00 | 3 | 49.868617 | -108.702335 | 665108 | 5526555 | 1 | 231.25 | Trans |
| M7 | 02/19/2012 0:00 | 6 | 49.869921 | -108.699830 | 665283 | 5526705 | 1 | 509.09 | Trans |
| M7 | 02/19/2012 0:00 | 9 | 49.874479 | -108.700497 | 665220 | 5527211 | 1 | 8.75 | Trans |
| M7 | 02/19/2012 0:00 | 12 | 49.874402 | -108.700523 | 665218 | 5527202 | 1 | 9.64 | Trans |
| M7 | 02/19/2012 0:00 | 15 | 49.874375 | -108.700395 | 665228 | 5527199 | 1 | 71.24 | Trans |
| M7 | 02/19/2012 0:00 | 18 | 49.874990 | -108.700672 | 665206 | 5527267 | 1 | 165.85 | Trans |
| M7 | 02/19/2012 0:00 | 21 | 49.876454 | -108.701111 | 665169 | 5527429 | 1 | 492.48 | Trans |
| M7 | 02/20/2012 0:00 | 0 | 49.880261 | -108.697612 | 665407 | 5527860 | 1 | 10.76 | Trans |
| M7 | 02/20/2012 0:00 | 3 | 49.880340 | -108.697524 | 665413 | 5527869 | 1 | 6.32 | Trans |
| M7 | 02/20/2012 0:00 | 6 | 49.880315 | -108.697603 | 665408 | 5527866 | 1 | 3.75 | Trans |
| M7 | 02/20/2012 0:00 | 9 | 49.880344 | -108.697630 | 665406 | 5527869 | 1 | 6.99 | Trans |
| M7 | 02/20/2012 0:00 | 12 | 49.880388 | -108.697560 | 665411 | 5527874 | 0 | 5.92 | Trans |
| M7 | 02/20/2012 0:00 | 18 | 49.880373 | -108.697480 | 665416 | 5527873 | 1 | 79.77 | Trans |
| M7 | 02/20/2012 0:00 | 21 | 49.879799 | -108.698146 | 665371 | 5527807 | 1 | 2.95 | Trans |
| M7 | 02/21/2012 0:00 | 0 | 49.879777 | -108.698124 | 665372 | 5527805 | 1 | 78.87 | Trans |
| M7 | 02/21/2012 0:00 | 3 | 49.880392 | -108.697577 | 665409 | 5527874 | 1 | 5.08 | Trans |
| M7 | 02/21/2012 0:00 | 6 | 49.880346 | -108.697573 | 665410 | 5527869 | 1 | 31.37 | Trans |
| M7 | 02/21/2012 0:00 | 9 | 49.880207 | -108.697194 | 665438 | 5527855 | 1 | 85.44 | Trans |
| M7 | 02/21/2012 0:00 | 12 | 49.879440 | -108.697269 | 665435 | 5527769 | 1 | 108.11 | Trans |
| M7 | 02/21/2012 0:00 | 15 | 49.880387 | -108.697604 | 665408 | 5527874 | 1 | 4.11 | Trans |
| M7 | 02/21/2012 0:00 | 18 | 49.880419 | -108.697573 | 665410 | 5527877 | 1 | 97.12 | Trans |
| M7 | 02/21/2012 0:00 | 21 | 49.879620 | -108.698119 | 665373 | 5527787 | 0 | 100.01 | Trans |
| M7 | 02/22/2012 0:00 | 9 | 49.880343 | -108.697291 | 665430 | 5527870 | 1 | 9.62 | Trans |
| M7 | 02/22/2012 0:00 | 12 | 49.880419 | -108.697353 | 665425 | 5527878 | 1 | 18.81 | Trans |
| M7 | 02/22/2012 0:00 | 15 | 49.880315 | -108.697559 | 665411 | 5527866 | 1 | 9.26 | Trans |
| M7 | 02/22/2012 0:00 | 18 | 49.880290 | -108.697682 | 665402 | 5527863 | 1 | 64.47 | Trans |
| M7 | 02/22/2012 0:00 | 21 | 49.879775 | -108.698093 | 665374 | 5527805 | 1 | 2.23 | Trans |
| M7 | 02/23/2012 0:00 | 0 | 49.879777 | -108.698124 | 665372 | 5527805 | 1 | 90.36 | Trans |

| | | | | | | | | | | |
|---|---|---|---|---|---|---|---|---|---|---|
| M7 | 02/23/2012 0:00 | 3 | 49.880388 | -108.697296 | 665430 | 5527875 | 1 | 9.21 | Trans |
| M7 | 02/23/2012 0:00 | 6 | 49.880468 | -108.697327 | 665427 | 5527883 | 1 | 24.44 | Trans |
| M7 | 02/23/2012 0:00 | 9 | 49.880319 | -108.697577 | 665410 | 5527866 | 1 | 3.00 | Trans |
| M7 | 02/23/2012 0:00 | 12 | 49.880346 | -108.697573 | 665410 | 5527869 | 1 | 19.74 | Trans |
| M7 | 02/23/2012 0:00 | 15 | 49.880367 | -108.697300 | 665429 | 5527872 | 1 | 103.63 | Trans |
| M7 | 02/23/2012 0:00 | 18 | 49.879548 | -108.697986 | 665383 | 5527780 | 0 | 106.84 | Trans |
| M7 | 02/24/2012 0:00 | 6 | 49.880444 | -108.697450 | 665418 | 5527880 | 1 | 6.70 | Trans |
| M7 | 02/24/2012 0:00 | 9 | 49.880417 | -108.697366 | 665424 | 5527878 | 1 | 5.39 | Trans |
| M7 | 02/24/2012 0:00 | 12 | 49.880415 | -108.697291 | 665430 | 5527878 | 0 | 12.41 | Trans |
| M7 | 02/25/2012 0:00 | 0 | 49.880527 | -108.697292 | 665429 | 5527890 | 1 | 12.37 | Trans |
| M7 | 02/25/2012 0:00 | 3 | 49.880417 | -108.697322 | 665428 | 5527878 | 1 | 24.24 | Trans |
| M7 | 02/25/2012 0:00 | 6 | 49.880445 | -108.697657 | 665404 | 5527880 | 1 | 497.18 | Trans |
| M7 | 02/25/2012 0:00 | 9 | 49.876062 | -108.699014 | 665321 | 5527390 | 1 | 19.82 | Trans |
| M7 | 02/25/2012 0:00 | 12 | 49.876121 | -108.699274 | 665302 | 5527396 | 0 | 1012.52 | Trans |
| M7 | 02/25/2012 0:00 | 18 | 49.883182 | -108.690381 | 665917 | 5528200 | 1 | 2311.70 | Trans |
| M7 | 02/25/2012 0:00 | 21 | 49.886383 | -108.658596 | 668189 | 5528627 | 1 | 2945.42 | Trans |
| M7 | 02/26/2012 0:00 | 0 | 49.871840 | -108.692846 | 665779 | 5526934 | 1 | 538.47 | Trans |
| M7 | 02/26/2012 0:00 | 3 | 49.867775 | -108.696915 | 665500 | 5526473 | 1 | 179.17 | Trans |
| M7 | 02/26/2012 0:00 | 6 | 49.867951 | -108.699393 | 665321 | 5526487 | 1 | 894.96 | Trans |
| M7 | 02/26/2012 0:00 | 9 | 49.859911 | -108.698878 | 665386 | 5525595 | 1 | 513.19 | Trans |
| M7 | 02/26/2012 0:00 | 12 | 49.864364 | -108.700750 | 665236 | 5526086 | 0 | 1580.53 | Trans |
| M7 | 02/26/2012 0:00 | 21 | 49.850523 | -108.695767 | 665642 | 5524558 | 1 | 1923.45 | Trans |
| M7 | 02/27/2012 0:00 | 0 | 49.867804 | -108.696810 | 665508 | 5526477 | 1 | 5.11 | Trans |
| M7 | 02/27/2012 0:00 | 3 | 49.867845 | -108.696841 | 665505 | 5526481 | 1 | 383.32 | Trans |
| M7 | 02/27/2012 0:00 | 6 | 49.867262 | -108.691586 | 665885 | 5526428 | 1 | 635.17 | Trans |
| M7 | 02/27/2012 0:00 | 9 | 49.862546 | -108.696570 | 665543 | 5525893 | 1 | 5.73 | Trans |
| M7 | 02/27/2012 0:00 | 12 | 49.862598 | -108.696575 | 665542 | 5525898 | 1 | 10.02 | Trans |
| M7 | 02/27/2012 0:00 | 15 | 49.862522 | -108.696649 | 665537 | 5525890 | 1 | 615.10 | Trans |
| M7 | 02/27/2012 0:00 | 18 | 49.857834 | -108.701189 | 665227 | 5525359 | 1 | 1960.65 | Trans |
| M7 | 02/27/2012 0:00 | 21 | 49.872377 | -108.685775 | 666285 | 5527009 | 1 | 41.28 | Trans |
| M7 | 02/28/2012 0:00 | 0 | 49.872409 | -108.686347 | 666244 | 5527012 | 1 | 10.20 | Trans |
| M7 | 02/28/2012 0:00 | 3 | 49.872327 | -108.686285 | 666248 | 5527003 | 1 | 11.03 | Trans |
| M7 | 02/28/2012 0:00 | 6 | 49.872252 | -108.686386 | 666241 | 5526994 | 1 | 1041.60 | Trans |
| M7 | 02/28/2012 0:00 | 9 | 49.875988 | -108.699674 | 665274 | 5527380 | 1 | 103.40 | Trans |
| M7 | 02/28/2012 0:00 | 12 | 49.876886 | -108.699301 | 665298 | 5527481 | 1 | 6.47 | Trans |
| M7 | 02/28/2012 0:00 | 15 | 49.876832 | -108.699265 | 665300 | 5527475 | 1 | 248.07 | Trans |
| M7 | 02/28/2012 0:00 | 18 | 49.879038 | -108.698754 | 665330 | 5527721 | 1 | 1028.71 | Trans |
| M7 | 02/28/2012 0:00 | 21 | 49.874943 | -108.685920 | 666266 | 5527294 | 1 | 1109.98 | Trans |
| M7 | 02/29/2012 0:00 | 0 | 49.867901 | -108.696863 | 665503 | 5526487 | 1 | 12.57 | Trans |
| M7 | 02/29/2012 0:00 | 3 | 49.867802 | -108.696779 | 665510 | 5526477 | 1 | 5.48 | Trans |
| M7 | 02/29/2012 0:00 | 6 | 49.867773 | -108.696840 | 665505 | 5526473 | 1 | 3.00 | Trans |
| M7 | 02/29/2012 0:00 | 9 | 49.867800 | -108.696836 | 665506 | 5526476 | 1 | 257.00 | Trans |
| M7 | 02/29/2012 0:00 | 12 | 49.866158 | -108.699352 | 665331 | 5526288 | 1 | 35.33 | Trans |
| M7 | 02/29/2012 0:00 | 15 | 49.865903 | -108.699645 | 665310 | 5526259 | 1 | 207.30 | Trans |
| M7 | 02/29/2012 0:00 | 18 | 49.864933 | -108.702107 | 665137 | 5526146 | 1 | 2666.91 | Trans |
| M7 | 02/29/2012 0:00 | 21 | 49.841017 | -108.699421 | 665411 | 5523493 | 1 | 1019.63 | Trans |
| M7 | 03/01/2012 0:00 | 0 | 49.832109 | -108.702771 | 665201 | 5522495 | 1 | 3446.74 | Trans |
| M7 | 03/01/2012 0:00 | 3 | 49.801316 | -108.697373 | 665694 | 5519084 | 1 | 1226.95 | Trans |
| M7 | 03/01/2012 0:00 | 6 | 49.790381 | -108.695118 | 665894 | 5517874 | 1 | 51.63 | Trans |
| M7 | 03/01/2012 0:00 | 9 | 49.790790 | -108.695459 | 665868 | 5517918 | 1 | 5.38 | Trans |
| M7 | 03/01/2012 0:00 | 12 | 49.790788 | -108.695384 | 665873 | 5517918 | 1 | 5.83 | Trans |
| M7 | 03/01/2012 0:00 | 15 | 49.790742 | -108.695423 | 665871 | 5517913 | 1 | 8.59 | Trans |
| M7 | 03/01/2012 0:00 | 18 | 49.790816 | -108.695455 | 665868 | 5517921 | 1 | 28.53 | Trans |
| M7 | 03/01/2012 0:00 | 21 | 49.790560 | -108.695453 | 665869 | 5517893 | 0 | 23.45 | Trans |
| M7 | 03/02/2012 0:00 | 9 | 49.790771 | -108.695450 | 665869 | 5517916 | 1 | 8.59 | Trans |

| | | | | | | | | | |
|---|---|---|---|---|---|---|---|---|---|
| M7 | 03/02/2012 0:00 | 12 | 49.790845 | -108.695481 | 665866 | 5517924 | 1 | 14.75 | Trans |
| M7 | 03/02/2012 0:00 | 15 | 49.790713 | -108.695485 | 665866 | 5517910 | 1 | 11.77 | Trans |
| M7 | 03/02/2012 0:00 | 18 | 49.790818 | -108.695485 | 665866 | 5517921 | 1 | 34.95 | Trans |
| M7 | 03/02/2012 0:00 | 21 | 49.790509 | -108.695405 | 665873 | 5517887 | 1 | 46.40 | Trans |
| M7 | 03/03/2012 0:00 | 0 | 49.790200 | -108.695838 | 665843 | 5517852 | 1 | 4.68 | Trans |
| M7 | 03/03/2012 0:00 | 3 | 49.790175 | -108.695785 | 665847 | 5517849 | 1 | 11.03 | Trans |
| M7 | 03/03/2012 0:00 | 6 | 49.790100 | -108.695885 | 665840 | 5517841 | 1 | 87.42 | Trans |
| M7 | 03/03/2012 0:00 | 9 | 49.790872 | -108.695652 | 665854 | 5517927 | 1 | 96.22 | Trans |
| M7 | 03/03/2012 0:00 | 12 | 49.790019 | -108.695428 | 665873 | 5517833 | 1 | 2.45 | Trans |
| M7 | 03/03/2012 0:00 | 15 | 49.790002 | -108.695406 | 665874 | 5517831 | 1 | 163.12 | Trans |
| M7 | 03/03/2012 0:00 | 18 | 49.791410 | -108.696042 | 665824 | 5517986 | 1 | 2554.86 | Trans |
| M7 | 03/03/2012 0:00 | 21 | 49.813624 | -108.687005 | 666398 | 5520475 | 1 | 1119.09 | Trans |
| M7 | 03/04/2012 0:00 | 0 | 49.820385 | -108.698522 | 665546 | 5521201 | 1 | 4234.52 | Trans |
| M7 | 03/04/2012 0:00 | 3 | 49.858455 | -108.699292 | 665361 | 5525432 | 0 | 2050.05 | Trans |
| M7 | 03/04/2012 0:00 | 18 | 49.876888 | -108.699375 | 665292 | 5527481 | 1 | 905.74 | Trans |
| M7 | 03/04/2012 0:00 | 21 | 49.881647 | -108.689149 | 666011 | 5528033 | 1 | 49.11 | Trans |
| M7 | 03/05/2012 0:00 | 0 | 49.882059 | -108.688901 | 666027 | 5528079 | 1 | 46.82 | Trans |
| M7 | 03/05/2012 0:00 | 3 | 49.881672 | -108.689158 | 666010 | 5528035 | 1 | 56.78 | Trans |
| M7 | 03/05/2012 0:00 | 6 | 49.882035 | -108.689715 | 665969 | 5528074 | 1 | 9.49 | Trans |
| M7 | 03/05/2012 0:00 | 9 | 49.882034 | -108.689847 | 665959 | 5528074 | 1 | 58.53 | Trans |
| M7 | 03/05/2012 0:00 | 12 | 49.881670 | -108.689260 | 666003 | 5528035 | 1 | 1.95 | Trans |
| M7 | 03/05/2012 0:00 | 15 | 49.881674 | -108.689233 | 666004 | 5528035 | 1 | 2.23 | Trans |
| M7 | 03/05/2012 0:00 | 18 | 49.881672 | -108.689202 | 666007 | 5528035 | 1 | 26.26 | Trans |
| M7 | 03/05/2012 0:00 | 21 | 49.881853 | -108.689437 | 665989 | 5528055 | 1 | 16.31 | Trans |
| M7 | 03/06/2012 0:00 | 0 | 49.881906 | -108.689648 | 665974 | 5528060 | 1 | 43.79 | Trans |
| M7 | 03/06/2012 0:00 | 3 | 49.881672 | -108.689158 | 666010 | 5528035 | 1 | 56.00 | Trans |
| M7 | 03/06/2012 0:00 | 6 | 49.882003 | -108.689745 | 665966 | 5528071 | 1 | 17.28 | Trans |
| M7 | 03/06/2012 0:00 | 9 | 49.881882 | -108.689595 | 665978 | 5528058 | 1 | 23.04 | Trans |
| M7 | 03/06/2012 0:00 | 12 | 49.882032 | -108.689816 | 665961 | 5528074 | 1 | 3.00 | Trans |
| M7 | 03/06/2012 0:00 | 15 | 49.882005 | -108.689820 | 665961 | 5528071 | 1 | 6.70 | Trans |
| M7 | 03/06/2012 0:00 | 18 | 49.882032 | -108.689904 | 665955 | 5528074 | 1 | 10.05 | Trans |
| M7 | 03/06/2012 0:00 | 21 | 49.881976 | -108.689793 | 665963 | 5528068 | 1 | 16.54 | Trans |
| M7 | 03/07/2012 0:00 | 0 | 49.881878 | -108.689621 | 665976 | 5528057 | 1 | 8.74 | Trans |
| M7 | 03/07/2012 0:00 | 3 | 49.881954 | -108.689595 | 665977 | 5528066 | 1 | 8.05 | Trans |
| M7 | 03/07/2012 0:00 | 6 | 49.881923 | -108.689494 | 665985 | 5528062 | 1 | 178.32 | Trans |
| M7 | 03/07/2012 0:00 | 9 | 49.883083 | -108.691207 | 665858 | 5528188 | 1 | 159.58 | Trans |
| M7 | 03/07/2012 0:00 | 12 | 49.882003 | -108.689745 | 665966 | 5528071 | 1 | 1.96 | Trans |
| M7 | 03/07/2012 0:00 | 15 | 49.882008 | -108.689719 | 665968 | 5528071 | 1 | 9.49 | Trans |
| M7 | 03/07/2012 0:00 | 18 | 49.881950 | -108.689622 | 665976 | 5528065 | 1 | 3203.43 | Trans |
| M7 | 03/07/2012 0:00 | 21 | 49.891533 | -108.647583 | 668962 | 5529224 | 1 | 67.67 | Trans |
| M7 | 03/08/2012 0:00 | 0 | 49.891535 | -108.648524 | 668894 | 5529223 | 1 | 1503.33 | Trans |
| M7 | 03/08/2012 0:00 | 3 | 49.883921 | -108.665810 | 667679 | 5528337 | 1 | 2872.50 | Trans |
| M7 | 03/08/2012 0:00 | 6 | 49.867620 | -108.696809 | 665508 | 5526456 | 1 | 277.15 | Trans |
| M7 | 03/08/2012 0:00 | 9 | 49.865850 | -108.699522 | 665319 | 5526253 | 0 | 6479.05 | Trans |
| M7 | 03/09/2012 0:00 | 0 | 49.808026 | -108.688583 | 666304 | 5519850 | 1 | 3871.66 | Trans |
| M7 | 03/09/2012 0:00 | 3 | 49.774150 | -108.676202 | 667311 | 5516111 | 0 | 3687.89 | Trans |
| M7 | 03/09/2012 0:00 | 9 | 49.742968 | -108.658792 | 668673 | 5512684 | 1 | 7.81 | Trans |
| M7 | 03/09/2012 0:00 | 12 | 49.743024 | -108.658726 | 668677 | 5512690 | 1 | 5.00 | Trans |
| M7 | 03/09/2012 0:00 | 15 | 49.743068 | -108.658744 | 668676 | 5512695 | 1 | 14.21 | Trans |
| M7 | 03/09/2012 0:00 | 18 | 49.742944 | -108.658695 | 668680 | 5512681 | 1 | 617.48 | Trans |
| M7 | 03/09/2012 0:00 | 21 | 49.737570 | -108.656543 | 668853 | 5512089 | 1 | 356.08 | Trans |
| M7 | 03/10/2012 0:00 | 0 | 49.734496 | -108.655160 | 668964 | 5511750 | 1 | 1423.15 | Trans |
| M7 | 03/10/2012 0:00 | 3 | 49.722135 | -108.660262 | 668639 | 5510365 | 1 | 1088.50 | Trans |
| M7 | 03/10/2012 0:00 | 6 | 49.714428 | -108.669566 | 667995 | 5509487 | 1 | 26.26 | Trans |
| M7 | 03/10/2012 0:00 | 9 | 49.714197 | -108.669490 | 668001 | 5509462 | 1 | 35.05 | Trans |

| | | | | | | | | | |
|---|---|---|---|---|---|---|---|---|---|
| M7 | 03/10/2012 0:00 | 12 | 49.714299 | -108.669030 | 668034 | 5509474 | 1 | 13.56 | Trans |
| M7 | 03/10/2012 0:00 | 15 | 49.714275 | -108.668846 | 668047 | 5509472 | 1 | 6.71 | Trans |
| M7 | 03/10/2012 0:00 | 18 | 49.714301 | -108.668929 | 668041 | 5509474 | 1 | 1146.17 | Trans |
| M7 | 03/10/2012 0:00 | 21 | 49.704138 | -108.671563 | 667887 | 5508339 | 1 | 3170.09 | Trans |
| M7 | 03/11/2012 0:00 | 0 | 49.676050 | -108.679042 | 667444 | 5505200 | 1 | 4680.83 | Trans |
| M7 | 03/11/2012 0:00 | 3 | 49.640804 | -108.714474 | 665007 | 5501203 | 1 | 2260.66 | Trans |
| M7 | 03/11/2012 0:00 | 6 | 49.620963 | -108.721275 | 664583 | 5498983 | 0 | 996.68 | Trans |
| M7 | 03/11/2012 0:00 | 12 | 49.625085 | -108.733523 | 663685 | 5499414 | 1 | 15.78 | Trans |
| M7 | 03/11/2012 0:00 | 15 | 49.625059 | -108.733309 | 663700 | 5499412 | 1 | 14.55 | Trans |
| M7 | 03/11/2012 0:00 | 18 | 49.625005 | -108.733492 | 663687 | 5499405 | 1 | 2492.02 | Trans |
| M7 | 03/11/2012 0:00 | 21 | 49.603015 | -108.740121 | 663282 | 5496947 | 1 | 3868.72 | Trans |
| M7 | 03/12/2012 0:00 | 0 | 49.577567 | -108.776605 | 660730 | 5494039 | 1 | 2391.73 | Trans |
| M7 | 03/12/2012 0:00 | 3 | 49.573041 | -108.808936 | 658407 | 5493467 | 1 | 1372.87 | Trans |
| M7 | 03/12/2012 0:00 | 6 | 49.568534 | -108.826607 | 657144 | 5492929 | 1 | 426.36 | Trans |
| M7 | 03/12/2012 0:00 | 9 | 49.569940 | -108.821123 | 657536 | 5493097 | 1 | 5.73 | Trans |
| M7 | 03/12/2012 0:00 | 12 | 49.569991 | -108.821128 | 657536 | 5493103 | 0 | 5.73 | Trans |
| M7 | 03/12/2012 0:00 | 18 | 49.569940 | -108.821123 | 657536 | 5493097 | 1 | 1678.94 | Trans |
| M7 | 03/12/2012 0:00 | 21 | 49.555760 | -108.829088 | 657006 | 5491504 | 1 | 3363.61 | Trans |
| M7 | 03/13/2012 0:00 | 0 | 49.526086 | -108.838088 | 656450 | 5488187 | 1 | 3802.00 | Trans |
| M7 | 03/13/2012 0:00 | 3 | 49.521277 | -108.890084 | 652702 | 5487545 | 1 | 5039.21 | Trans |
| M7 | 03/13/2012 0:00 | 6 | 49.475996 | -108.892728 | 652652 | 5482506 | 1 | 1003.48 | Trans |
| M7 | 03/13/2012 0:00 | 9 | 49.472943 | -108.905759 | 651717 | 5482141 | 1 | 3.74 | Trans |
| M7 | 03/13/2012 0:00 | 12 | 49.472972 | -108.905785 | 651715 | 5482144 | 1 | 5.38 | Trans |
| M7 | 03/13/2012 0:00 | 15 | 49.472941 | -108.905728 | 651720 | 5482141 | 1 | 0.00 | Trans |
| M7 | 03/13/2012 0:00 | 18 | 49.472941 | -108.905728 | 651720 | 5482141 | 1 | 2503.82 | Trans |
| M7 | 03/13/2012 0:00 | 21 | 49.454509 | -108.885889 | 653214 | 5480132 | 1 | 10853.39 | Trans |
| M7 | 03/14/2012 0:00 | 0 | 49.371950 | -108.806115 | 659262 | 5471120 | 1 | 11266.74 | Trans |
| M7 | 03/14/2012 0:00 | 3 | 49.277540 | -108.749882 | 663657 | 5460745 | 1 | 8171.80 | Trans |
| M7 | 03/14/2012 0:00 | 6 | 49.211901 | -108.699426 | 667548 | 5453560 | 1 | 4650.29 | Trans |
| M7 | 03/14/2012 0:00 | 9 | 49.250429 | -108.674608 | 669224 | 5457898 | 1 | 33.84 | Trans |
| M7 | 03/14/2012 0:00 | 12 | 49.250430 | -108.675073 | 669190 | 5457897 | 0 | 4.10 | Trans |
| M7 | 03/14/2012 0:00 | 18 | 49.250399 | -108.675103 | 669188 | 5457893 | 1 | 3859.87 | Trans |
| M7 | 03/14/2012 0:00 | 21 | 49.261771 | -108.625005 | 672794 | 5459271 | 1 | 8619.64 | Trans |
| M7 | 03/15/2012 0:00 | 0 | 49.276769 | -108.508802 | 681192 | 5461210 | 1 | 4856.96 | Trans |
| M7 | 03/15/2012 0:00 | 3 | 49.268703 | -108.443209 | 685993 | 5460473 | 1 | 6232.87 | Trans |
| M7 | 03/15/2012 0:00 | 6 | 49.238598 | -108.370997 | 691362 | 5457307 | 1 | 4.68 | Trans |
| M7 | 03/15/2012 0:00 | 9 | 49.238622 | -108.371049 | 691358 | 5457309 | 1 | 5.00 | Trans |
| M7 | 03/15/2012 0:00 | 12 | 49.238577 | -108.371049 | 691358 | 5457304 | 1 | 0.00 | Trans |
| M7 | 03/15/2012 0:00 | 15 | 49.238577 | -108.371049 | 691358 | 5457304 | 1 | 7.33 | Trans |
| M7 | 03/15/2012 0:00 | 18 | 49.238544 | -108.371136 | 691352 | 5457300 | 1 | 2024.29 | Trans |
| M7 | 03/15/2012 0:00 | 21 | 49.242005 | -108.343846 | 693325 | 5457754 | 1 | 8533.96 | Trans |
| M7 | 03/16/2012 0:00 | 0 | 49.224500 | -108.229767 | 701698 | 5456107 | 1 | 6599.81 | Trans |
| M7 | 03/16/2012 0:00 | 3 | 49.174656 | -108.180641 | 705481 | 5450699 | 1 | 6788.85 | Trans |
| M7 | 03/16/2012 0:00 | 6 | 49.125705 | -108.125060 | 709738 | 5445410 | 0 | 8009.97 | Trans |
| M7 | 03/16/2012 0:00 | 15 | 49.086740 | -108.032820 | 716636 | 5441339 | 1 | 18.80 | Trans |
| M7 | 03/16/2012 0:00 | 18 | 49.086718 | -108.032565 | 716655 | 5441337 | 1 | 1416.51 | Trans |
| M7 | 03/16/2012 0:00 | 21 | 49.077444 | -108.019277 | 717666 | 5440345 | 1 | 7932.81 | Trans |
| M7 | 03/17/2012 0:00 | 0 | 49.017980 | -107.959379 | 722304 | 5433910 | 1 | 5754.74 | Trans |
| M7 | 03/17/2012 0:00 | 3 | 48.967320 | -107.943431 | 723697 | 5428326 | 1 | 10631.20 | Trans |
| M7 | 03/17/2012 0:00 | 6 | 48.880171 | -107.883885 | 728452 | 5418817 | 1 | 5338.77 | Trans |
| M7 | 03/17/2012 0:00 | 9 | 48.910764 | -107.939974 | 724203 | 5422050 | 1 | 4.11 | Trans |
| M7 | 03/17/2012 0:00 | 12 | 48.910795 | -107.939944 | 724205 | 5422054 | 1 | 2.22 | Trans |
| M7 | 03/17/2012 0:00 | 15 | 48.910798 | -107.939974 | 724203 | 5422054 | 1 | 4.69 | Trans |
| M7 | 03/17/2012 0:00 | 18 | 48.910823 | -107.940025 | 724199 | 5422057 | 1 | 3355.20 | Trans |
| M7 | 03/17/2012 0:00 | 21 | 48.880992 | -107.946804 | 723836 | 5418721 | 1 | 5094.61 | Trans |

| | | | | | | | | | |
|---|---|---|---|---|---|---|---|---|---|
| M7 | 03/18/2012 0:00 | 0 | 48.835297 | -107.951569 | 723690 | 5413629 | 1 | 6916.16 | Trans |
| M7 | 03/18/2012 0:00 | 3 | 48.773422 | -107.942277 | 724648 | 5406779 | 1 | 4385.24 | Trans |
| M7 | 03/18/2012 0:00 | 6 | 48.734310 | -107.949774 | 724272 | 5402410 | 1 | 6.06 | Trans |
| M7 | 03/18/2012 0:00 | 9 | 48.734304 | -107.949856 | 724266 | 5402409 | 0 | 2288.62 | Trans |
| M7 | 03/18/2012 0:00 | 21 | 48.716721 | -107.933702 | 725532 | 5400503 | 1 | 5027.24 | Trans |
| M7 | 03/19/2012 0:00 | 0 | 48.672175 | -107.922161 | 726581 | 5395586 | 1 | 191.04 | Trans |
| M7 | 03/19/2012 0:00 | 3 | 48.670481 | -107.922594 | 726557 | 5395397 | 1 | 1317.77 | Trans |
| M7 | 03/19/2012 0:00 | 6 | 48.658788 | -107.919721 | 726821 | 5394106 | 1 | 2.97 | Trans |
| M7 | 03/19/2012 0:00 | 9 | 48.658765 | -107.919742 | 726819 | 5394103 | 1 | 2.82 | Trans |
| M7 | 03/19/2012 0:00 | 12 | 48.658790 | -107.919751 | 726818 | 5394106 | 1 | 4.69 | Trans |
| M7 | 03/19/2012 0:00 | 15 | 48.658765 | -107.919699 | 726822 | 5394103 | 1 | 10.49 | Trans |
| M7 | 03/19/2012 0:00 | 18 | 48.658683 | -107.919769 | 726817 | 5394094 | 1 | 7.46 | Trans |
| M7 | 03/19/2012 0:00 | 21 | 48.658732 | -107.919700 | 726822 | 5394099 | 1 | 6.71 | Trans |
| M7 | 03/20/2012 0:00 | 0 | 48.658759 | -107.919781 | 726816 | 5394102 | 1 | 1282.92 | Trans |
| M7 | 03/20/2012 0:00 | 3 | 48.670025 | -107.923513 | 726491 | 5395343 | 1 | 162.98 | Trans |
| M7 | 03/20/2012 0:00 | 6 | 48.670665 | -107.925503 | 726342 | 5395409 | 1 | 7.07 | Trans |
| M7 | 03/20/2012 0:00 | 9 | 48.670612 | -107.925555 | 726338 | 5395402 | 1 | 4.69 | Trans |
| M7 | 03/20/2012 0:00 | 12 | 48.670637 | -107.925607 | 726334 | 5395405 | 1 | 105.86 | Trans |
| M7 | 03/20/2012 0:00 | 15 | 48.671508 | -107.925027 | 726373 | 5395504 | 1 | 11.70 | Trans |
| M7 | 03/20/2012 0:00 | 18 | 48.671510 | -107.925186 | 726361 | 5395503 | 1 | 490.84 | Trans |
| M7 | 03/20/2012 0:00 | 21 | 48.667309 | -107.927224 | 726230 | 5395030 | 1 | 5118.56 | Trans |
| M7 | 03/21/2012 0:00 | 0 | 48.638177 | -107.873460 | 730321 | 5391954 | 1 | 5642.04 | Trans |
| M7 | 03/21/2012 0:00 | 3 | 48.601007 | -107.821402 | 734327 | 5387981 | 1 | 4427.49 | Trans |
| M7 | 03/21/2012 0:00 | 6 | 48.577175 | -107.773347 | 737981 | 5385481 | 0 | 862.10 | Trans |
| M7 | 03/21/2012 0:00 | 12 | 48.572562 | -107.763962 | 738695 | 5384998 | 1 | 18.25 | Trans |
| M7 | 03/21/2012 0:00 | 15 | 48.572415 | -107.763852 | 738704 | 5384982 | 1 | 18.50 | Trans |
| M7 | 03/21/2012 0:00 | 18 | 48.572486 | -107.764078 | 738687 | 5384989 | 1 | 441.12 | Trans |
| M7 | 03/21/2012 0:00 | 21 | 48.570008 | -107.768744 | 738354 | 5384699 | 1 | 4723.59 | Trans |
| M7 | 03/22/2012 0:00 | 0 | 48.543966 | -107.718215 | 742206 | 5381964 | 1 | 6995.29 | Trans |
| M7 | 03/22/2012 0:00 | 3 | 48.529635 | -107.626014 | 749080 | 5380668 | 1 | 6351.30 | Trans |
| M7 | 03/22/2012 0:00 | 6 | 48.534091 | -107.540312 | 755383 | 5381446 | 1 | 565.93 | Trans |
| M7 | 03/22/2012 0:00 | 9 | 48.539129 | -107.541384 | 755279 | 5382002 | 1 | 42.72 | Trans |
| M7 | 03/22/2012 0:00 | 12 | 48.538750 | -107.541476 | 755274 | 5381960 | 1 | 17.03 | Trans |
| M7 | 03/22/2012 0:00 | 15 | 48.538899 | -107.541529 | 755269 | 5381976 | 1 | 18.50 | Trans |
| M7 | 03/22/2012 0:00 | 18 | 48.538746 | -107.541631 | 755262 | 5381959 | 1 | 189.87 | Trans |
| M7 | 03/22/2012 0:00 | 21 | 48.539957 | -107.539819 | 755390 | 5382100 | 1 | 2521.56 | Trans |
| M7 | 03/23/2012 0:00 | 0 | 48.538436 | -107.505763 | 757911 | 5382045 | 1 | 70.24 | Trans |
| M7 | 03/23/2012 0:00 | 3 | 48.538057 | -107.505003 | 757969 | 5382005 | 1 | 54.95 | Trans |
| M7 | 03/23/2012 0:00 | 6 | 48.538519 | -107.504742 | 757986 | 5382058 | 1 | 259.86 | Trans |
| M7 | 03/23/2012 0:00 | 9 | 48.536696 | -107.502544 | 758157 | 5381863 | 1 | 1.96 | Trans |
| M7 | 03/23/2012 0:00 | 12 | 48.536699 | -107.502518 | 758159 | 5381863 | 1 | 9.65 | Trans |
| M7 | 03/23/2012 0:00 | 15 | 48.536672 | -107.502395 | 758169 | 5381860 | 1 | 76.86 | Trans |
| M7 | 03/23/2012 0:00 | 18 | 48.537079 | -107.503235 | 758104 | 5381903 | 1 | 169.15 | Trans |
| M7 | 03/23/2012 0:00 | 21 | 48.538108 | -107.504921 | 757975 | 5382011 | 1 | 14.33 | Trans |
| M7 | 03/24/2012 0:00 | 0 | 48.537980 | -107.504949 | 757973 | 5381997 | 1 | 3086.38 | Trans |
| M7 | 03/24/2012 0:00 | 3 | 48.528099 | -107.465915 | 760905 | 5381032 | 1 | 2052.61 | Trans |
| M7 | 03/24/2012 0:00 | 6 | 48.533116 | -107.439183 | 762852 | 5381681 | 1 | 52.04 | Trans |
| M7 | 03/24/2012 0:00 | 9 | 48.533342 | -107.438566 | 762896 | 5381708 | 1 | 7.28 | Trans |
| M7 | 03/24/2012 0:00 | 12 | 48.533343 | -107.438467 | 762904 | 5381709 | 1 | 3.46 | Trans |
| M7 | 03/24/2012 0:00 | 15 | 48.533372 | -107.438450 | 762905 | 5381712 | 1 | 9.50 | Trans |
| M7 | 03/24/2012 0:00 | 18 | 48.533371 | -107.438321 | 762914 | 5381712 | 1 | 34.61 | Trans |
| M7 | 03/24/2012 0:00 | 21 | 48.533298 | -107.438777 | 762881 | 5381702 | 1 | 39.94 | Trans |
| M7 | 03/25/2012 0:00 | 0 | 48.533343 | -107.438240 | 762920 | 5381709 | 1 | 269.82 | Trans |
| M7 | 03/25/2012 0:00 | 3 | 48.535749 | -107.437773 | 762942 | 5381978 | 1 | 8.72 | Trans |
| M7 | 03/25/2012 0:00 | 6 | 48.535827 | -107.437772 | 762942 | 5381987 | 1 | 283.17 | Trans |

| | | | | | | | | | |
|---|---|---|---|---|---|---|---|---|---|
| M7 | 03/25/2012 0:00 | 9 | 48.533327 | -107.438489 | 762902 | 5381707 | 1 | 8.76 | Trans |
| M7 | 03/25/2012 0:00 | 12 | 48.533374 | -107.438394 | 762909 | 5381712 | 0 | 60.09 | Trans |
| M7 | 03/25/2012 0:00 | 18 | 48.533881 | -107.438671 | 762886 | 5381768 | 1 | 260.87 | Trans |
| M7 | 03/25/2012 0:00 | 21 | 48.536110 | -107.437574 | 762955 | 5382019 | 1 | 1600.63 | Trans |
| M7 | 03/26/2012 0:00 | 0 | 48.542079 | -107.417860 | 764379 | 5382750 | 1 | 3719.73 | Trans |
| M7 | 03/26/2012 0:00 | 3 | 48.541099 | -107.367531 | 768098 | 5382817 | 1 | 5846.24 | Trans |
| M7 | 03/26/2012 0:00 | 6 | 48.528587 | -107.290683 | 773837 | 5381699 | 1 | 2882.49 | Trans |
| M7 | 03/26/2012 0:00 | 9 | 48.515196 | -107.257296 | 776374 | 5380331 | 1 | 205.27 | Trans |
| M7 | 03/26/2012 0:00 | 12 | 48.515609 | -107.254589 | 776571 | 5380387 | 1 | 9.88 | Trans |
| M7 | 03/26/2012 0:00 | 15 | 48.515684 | -107.254660 | 776566 | 5380395 | 1 | 6.47 | Trans |
| M7 | 03/26/2012 0:00 | 18 | 48.515626 | -107.254653 | 776567 | 5380389 | 1 | 530.14 | Trans |
| M7 | 03/26/2012 0:00 | 21 | 48.515808 | -107.247486 | 777095 | 5380435 | 1 | 5848.61 | Trans |
| M7 | 03/27/2012 0:00 | 0 | 48.506000 | -107.169763 | 782888 | 5379630 | 1 | 5096.56 | Trans |
| M7 | 03/27/2012 0:00 | 3 | 48.536646 | -107.118517 | 786499 | 5383227 | 1 | 5726.47 | Trans |
| M7 | 03/27/2012 0:00 | 6 | 48.587163 | -107.103704 | 787305 | 5388896 | 1 | 324.38 | Trans |
| M7 | 03/27/2012 0:00 | 9 | 48.586928 | -107.099324 | 787630 | 5388886 | 1 | 4.69 | Trans |
| M7 | 03/27/2012 0:00 | 12 | 48.586954 | -107.099375 | 787626 | 5388889 | 1 | 14.54 | Trans |
| M7 | 03/27/2012 0:00 | 15 | 48.587079 | -107.099320 | 787629 | 5388903 | 1 | 11.58 | Trans |
| M7 | 03/27/2012 0:00 | 18 | 48.586980 | -107.099369 | 787626 | 5388892 | 1 | 384.62 | Trans |
| M7 | 03/27/2012 0:00 | 21 | 48.583980 | -107.096782 | 787834 | 5388568 | 1 | 881.45 | Trans |
| M7 | 03/28/2012 0:00 | 0 | 48.580295 | -107.086213 | 788634 | 5388199 | 1 | 1562.95 | Trans |
| M7 | 03/28/2012 0:00 | 3 | 48.584336 | -107.065938 | 790106 | 5388725 | 1 | 4460.99 | Trans |
| M7 | 03/28/2012 0:00 | 6 | 48.594885 | -107.007636 | 794343 | 5390120 | 1 | 210.25 | Trans |
| M7 | 03/28/2012 0:00 | 9 | 48.593045 | -107.006985 | 794402 | 5389918 | 1 | 11.45 | Trans |
| M7 | 03/28/2012 0:00 | 12 | 48.593091 | -107.006846 | 794411 | 5389924 | 1 | 18.64 | Trans |
| M7 | 03/28/2012 0:00 | 15 | 48.592997 | -107.007054 | 794397 | 5389913 | 1 | 12.89 | Trans |
| M7 | 03/28/2012 0:00 | 18 | 48.592917 | -107.006928 | 794406 | 5389904 | 1 | 1428.26 | Trans |
| M7 | 03/28/2012 0:00 | 21 | 48.604639 | -106.999044 | 794919 | 5391237 | 1 | 3914.56 | Trans |
| M7 | 03/29/2012 0:00 | 0 | 48.635980 | -106.974948 | 796511 | 5394814 | 1 | 1831.92 | Trans |
| M7 | 03/29/2012 0:00 | 3 | 48.650210 | -106.962457 | 797347 | 5396443 | 1 | 8071.32 | Trans |
| M7 | 03/29/2012 0:00 | 6 | 48.701128 | -106.884462 | 802784 | 5402409 | 1 | 2603.22 | Trans |
| M7 | 03/29/2012 0:00 | 9 | 48.694679 | -106.918431 | 800324 | 5401558 | 1 | 188.35 | Trans |
| M7 | 03/29/2012 0:00 | 12 | 48.695420 | -106.920729 | 800151 | 5401631 | 1 | 3.47 | Trans |
| M7 | 03/29/2012 0:00 | 15 | 48.695449 | -106.920711 | 800152 | 5401634 | 0 | 195.89 | Trans |
| M7 | 03/29/2012 0:00 | 21 | 48.695244 | -106.918070 | 800347 | 5401622 | 1 | 7149.07 | Trans |
| M7 | 03/30/2012 0:00 | 0 | 48.720277 | -106.828679 | 806771 | 5404760 | 1 | 5882.89 | Trans |
| M7 | 03/30/2012 0:00 | 3 | 48.704511 | -106.752437 | 812474 | 5403318 | 1 | 19.53 | Trans |
| M7 | 03/30/2012 0:00 | 6 | 48.704686 | -106.752440 | 812473 | 5403338 | 1 | 54.45 | Trans |
| M7 | 03/30/2012 0:00 | 9 | 48.704308 | -106.751971 | 812510 | 5403297 | 1 | 16.42 | Trans |
| M7 | 03/30/2012 0:00 | 12 | 48.704350 | -106.752185 | 812494 | 5403301 | 1 | 13.58 | Trans |
| M7 | 03/30/2012 0:00 | 15 | 48.704323 | -106.752005 | 812507 | 5403299 | 1 | 46.17 | Trans |
| M7 | 03/30/2012 0:00 | 18 | 48.704535 | -106.752544 | 812466 | 5403320 | 1 | 4.11 | Trans |
| M7 | 03/30/2012 0:00 | 21 | 48.704566 | -106.752513 | 812468 | 5403324 | 1 | 2.44 | Trans |
| M7 | 03/31/2012 0:00 | 0 | 48.704583 | -106.752534 | 812467 | 5403326 | 1 | 3.16 | Trans |
| M7 | 03/31/2012 0:00 | 3 | 48.704583 | -106.752577 | 812464 | 5403326 | 1 | 7.08 | Trans |
| M7 | 03/31/2012 0:00 | 6 | 48.704636 | -106.752523 | 812467 | 5403332 | 1 | 51.68 | Trans |
| M7 | 03/31/2012 0:00 | 9 | 48.704323 | -106.752005 | 812507 | 5403299 | 1 | 5.92 | Trans |
| M7 | 03/31/2012 0:00 | 12 | 48.704307 | -106.751928 | 812513 | 5403298 | 1 | 8.65 | Trans |
| M7 | 03/31/2012 0:00 | 15 | 48.704380 | -106.751969 | 812510 | 5403306 | 1 | 6.00 | Trans |
| M7 | 03/31/2012 0:00 | 18 | 48.704327 | -106.751979 | 812509 | 5403300 | 1 | 47.45 | Trans |
| M7 | 03/31/2012 0:00 | 21 | 48.704566 | -106.752513 | 812468 | 5403324 | 1 | 13.36 | Trans |
| M7 | 04/01/2012 0:00 | 0 | 48.704685 | -106.752496 | 812469 | 5403337 | 1 | 46.00 | Trans |
| M7 | 04/01/2012 0:00 | 3 | 48.704969 | -106.752951 | 812434 | 5403367 | 1 | 4696.37 | Trans |
| M7 | 04/01/2012 0:00 | 6 | 48.696696 | -106.690438 | 817083 | 5402706 | 1 | 1190.31 | Trans |
| M7 | 04/01/2012 0:00 | 9 | 48.686133 | -106.687907 | 817336 | 5401543 | 1 | 3.59 | Trans |

| | | | | | | | | | | |
|---|---|---|---|---|---|---|---|---|---|---|
| M7 | 04/01/2012 0:00 | 12 | 48.686102 | -106.687896 | 817337 | 5401539 | 1 | 7.29 | Trans |
| M7 | 04/01/2012 0:00 | 15 | 48.686153 | -106.687958 | 817332 | 5401545 | 1 | 2.22 | Trans |
| M7 | 04/01/2012 0:00 | 18 | 48.686150 | -106.687928 | 817334 | 5401545 | 1 | 8.83 | Trans |
| M7 | 04/01/2012 0:00 | 21 | 48.686079 | -106.687875 | 817339 | 5401537 | 1 | 19.65 | Trans |
| M7 | 04/02/2012 0:00 | 0 | 48.686128 | -106.688131 | 817320 | 5401541 | 1 | 110.65 | Trans |
| M7 | 04/02/2012 0:00 | 3 | 48.685796 | -106.686716 | 817426 | 5401510 | 1 | 5006.87 | Trans |
| M7 | 04/02/2012 0:00 | 6 | 48.705688 | -106.625766 | 821783 | 5403977 | 1 | 326.20 | Trans |
| M7 | 04/02/2012 0:00 | 9 | 48.704352 | -106.621824 | 822082 | 5403845 | 1 | 3.00 | Trans |
| M7 | 04/02/2012 0:00 | 12 | 48.704378 | -106.621819 | 822082 | 5403848 | 1 | 5.75 | Trans |
| M7 | 04/02/2012 0:00 | 15 | 48.704430 | -106.621822 | 822081 | 5403853 | 1 | 6.40 | Trans |
| M7 | 04/02/2012 0:00 | 18 | 48.704379 | -106.621862 | 822079 | 5403848 | 1 | 2279.25 | Trans |
| M7 | 04/02/2012 0:00 | 21 | 48.721070 | -106.603934 | 823290 | 5405778 | 1 | 4876.29 | Trans |
| M7 | 04/03/2012 0:00 | 0 | 48.740446 | -106.544537 | 827531 | 5408185 | 1 | 5352.25 | Trans |
| M7 | 04/03/2012 0:00 | 3 | 48.766590 | -106.483505 | 831844 | 5411355 | 0 | 1910.32 | Trans |
| M7 | 04/03/2012 0:00 | 12 | 48.781055 | -106.469533 | 832774 | 5413023 | 1 | 5.72 | Trans |
| M7 | 04/03/2012 0:00 | 15 | 48.781103 | -106.469562 | 832772 | 5413028 | 0 | 238.67 | Trans |
| M7 | 04/03/2012 0:00 | 21 | 48.781794 | -106.466490 | 832993 | 5413119 | 1 | 7100.00 | Trans |
| M7 | 04/04/2012 0:00 | 0 | 48.806447 | -106.377454 | 839364 | 5416252 | 1 | 4795.44 | Trans |
| M7 | 04/04/2012 0:00 | 3 | 48.819608 | -106.315347 | 843832 | 5417993 | 1 | 1440.45 | Trans |
| M7 | 04/04/2012 0:00 | 6 | 48.827213 | -106.299494 | 844943 | 5418910 | 1 | 36.61 | Trans |
| M7 | 04/04/2012 0:00 | 9 | 48.826900 | -106.299645 | 844934 | 5418875 | 1 | 4.58 | Trans |
| M7 | 04/04/2012 0:00 | 12 | 48.826926 | -106.299597 | 844938 | 5418878 | 0 | 4.13 | Trans |
| M7 | 04/04/2012 0:00 | 18 | 48.826954 | -106.299634 | 844935 | 5418881 | 1 | 1171.84 | Trans |
| M7 | 04/04/2012 0:00 | 21 | 48.831100 | -106.284980 | 845981 | 5419408 | 1 | 2117.55 | Trans |
| M7 | 04/05/2012 0:00 | 0 | 48.827309 | -106.256749 | 848078 | 5419116 | 1 | 741.68 | Trans |
| M7 | 04/05/2012 0:00 | 3 | 48.823622 | -106.248345 | 848720 | 5418745 | 1 | 3006.25 | Trans |
| M7 | 04/05/2012 0:00 | 6 | 48.822885 | -106.207466 | 851725 | 5418851 | 1 | 2.23 | Trans |
| M7 | 04/05/2012 0:00 | 9 | 48.822883 | -106.207436 | 851727 | 5418851 | 1 | 4.69 | Trans |
| M7 | 04/05/2012 0:00 | 12 | 48.822857 | -106.207385 | 851731 | 5418848 | 1 | 9.49 | Trans |
| M7 | 04/05/2012 0:00 | 15 | 48.822860 | -106.207514 | 851721 | 5418848 | 1 | 6.32 | Trans |
| M7 | 04/05/2012 0:00 | 18 | 48.822883 | -106.207436 | 851727 | 5418851 | 1 | 265.96 | Trans |
| M7 | 04/05/2012 0:00 | 21 | 48.821933 | -106.204116 | 851977 | 5418761 | 1 | 1638.52 | Trans |
| M7 | 04/06/2012 0:00 | 0 | 48.819424 | -106.182155 | 853606 | 5418584 | 1 | 3023.32 | Trans |
| M7 | 04/06/2012 0:00 | 3 | 48.822447 | -106.141287 | 856583 | 5419111 | 1 | 9894.39 | Trans |
| M7 | 04/06/2012 0:00 | 6 | 48.801992 | -106.010346 | 866339 | 5417461 | 1 | 794.68 | Trans |
| M7 | 04/06/2012 0:00 | 9 | 48.801940 | -106.021150 | 865547 | 5417403 | 1 | 11.36 | Trans |
| M7 | 04/06/2012 0:00 | 12 | 48.801839 | -106.021173 | 865546 | 5417392 | 1 | 15.05 | Trans |
| M7 | 04/06/2012 0:00 | 15 | 48.801966 | -106.021102 | 865550 | 5417407 | 1 | 2.22 | Trans |
| M7 | 04/06/2012 0:00 | 18 | 48.801969 | -106.021132 | 865548 | 5417407 | 1 | 3256.84 | Trans |
| M7 | 04/06/2012 0:00 | 21 | 48.787806 | -105.982395 | 868495 | 5416020 | 1 | 5158.81 | Trans |
| M7 | 04/07/2012 0:00 | 0 | 48.800380 | -105.914904 | 873356 | 5417747 | 1 | 3674.92 | Trans |
| M7 | 04/07/2012 0:00 | 3 | 48.825413 | -105.882342 | 875558 | 5420689 | 1 | 4052.99 | Trans |
| M7 | 04/07/2012 0:00 | 6 | 48.851916 | -105.844551 | 878131 | 5423821 | 1 | 14.89 | Trans |
| M7 | 04/07/2012 0:00 | 9 | 48.851808 | -105.844431 | 878140 | 5423809 | 1 | 9.39 | Trans |
| M7 | 04/07/2012 0:00 | 12 | 48.851860 | -105.844532 | 878133 | 5423815 | 1 | 12.57 | Trans |
| M7 | 04/07/2012 0:00 | 15 | 48.851961 | -105.844609 | 878126 | 5423825 | 0 | 2194.21 | Trans |
| M7 | 04/07/2012 0:00 | 21 | 48.870804 | -105.835878 | 878624 | 5425962 | 1 | 10363.82 | Trans |
| M7 | 04/08/2012 0:00 | 0 | 48.901247 | -105.702525 | 888161 | 5430019 | 1 | 7473.88 | Trans |
| M7 | 04/08/2012 0:00 | 3 | 48.954184 | -105.639931 | 892330 | 5436222 | 1 | 658.85 | Trans |
| M7 | 04/08/2012 0:00 | 6 | 48.960099 | -105.639942 | 892282 | 5436879 | 0 | 5.88 | Trans |
| M7 | 04/08/2012 0:00 | 12 | 48.960148 | -105.639974 | 892280 | 5436884 | 1 | 3.16 | Trans |
| M7 | 04/08/2012 0:00 | 15 | 48.960147 | -105.639931 | 892283 | 5436884 | 1 | 8.63 | Trans |
| M7 | 04/08/2012 0:00 | 18 | 48.960070 | -105.639918 | 892284 | 5436876 | 1 | 1507.28 | Trans |
| M7 | 04/08/2012 0:00 | 21 | 48.969618 | -105.625351 | 893275 | 5438012 | 1 | 1117.11 | Trans |
| M7 | 04/09/2012 0:00 | 0 | 48.978785 | -105.619170 | 893654 | 5439063 | 1 | 275.24 | Trans |

| | | | | | | | | | |
|---|---|---|---|---|---|---|---|---|---|
| M7 | 04/09/2012 0:00 | 3 | 48.979042 | -105.615436 | 893925 | 5439111 | 1 | 864.90 | Trans |
| M7 | 04/09/2012 0:00 | 6 | 48.982168 | -105.604636 | 894690 | 5439514 | 1 | 19.44 | Trans |
| M7 | 04/09/2012 0:00 | 9 | 48.982034 | -105.604466 | 894704 | 5439500 | 0 | 8.26 | Trans |
| M7 | 04/09/2012 0:00 | 15 | 48.982090 | -105.604541 | 894698 | 5439506 | 1 | 7.09 | Trans |
| M7 | 04/09/2012 0:00 | 18 | 48.982037 | -105.604596 | 894694 | 5439500 | 1 | 7.29 | Trans |
| M7 | 04/09/2012 0:00 | 21 | 48.982062 | -105.604504 | 894701 | 5439503 | 1 | 7535.25 | Trans |
| M7 | 04/10/2012 0:00 | 0 | 49.045421 | -105.568442 | 896833 | 5446730 | 1 | 5651.32 | Trans |
| M7 | 04/10/2012 0:00 | 3 | 49.088946 | -105.528758 | 899381 | 5451775 | 0 | 1249.88 | Trans |
| M7 | 04/10/2012 0:00 | 12 | 49.096567 | -105.516216 | 900235 | 5452688 | 0 | 13.80 | Trans |
| M7 | 04/10/2012 0:00 | 21 | 49.096443 | -105.516219 | 900235 | 5452674 | 1 | 6098.18 | Trans |
| M7 | 04/11/2012 0:00 | 0 | 49.119971 | -105.440924 | 905536 | 5455689 | 1 | 6076.17 | Trans |
| M7 | 04/11/2012 0:00 | 3 | 49.119820 | -105.357822 | 911597 | 5456122 | 1 | 3831.59 | Trans |
| M7 | 04/11/2012 0:00 | 6 | 49.125881 | -105.306237 | 915307 | 5457077 | 1 | 2.97 | Trans |
| M7 | 04/11/2012 0:00 | 9 | 49.125857 | -105.306217 | 915309 | 5457075 | 1 | 4.69 | Trans |
| M7 | 04/11/2012 0:00 | 12 | 49.125831 | -105.306166 | 915313 | 5457072 | 1 | 7.54 | Trans |
| M7 | 04/11/2012 0:00 | 15 | 49.125881 | -105.306237 | 915307 | 5457077 | 1 | 5.64 | Trans |
| M7 | 04/11/2012 0:00 | 18 | 49.125831 | -105.306223 | 915309 | 5457072 | 1 | 1071.55 | Trans |
| M7 | 04/11/2012 0:00 | 21 | 49.135354 | -105.304169 | 915379 | 5458141 | 1 | 628.13 | Trans |
| M7 | 04/12/2012 0:00 | 0 | 49.139581 | -105.298482 | 915758 | 5458642 | 1 | 30.66 | Trans |
| M7 | 04/12/2012 0:00 | 3 | 49.139831 | -105.298305 | 915769 | 5458670 | 1 | 184.00 | Trans |
| M7 | 04/12/2012 0:00 | 6 | 49.141472 | -105.298596 | 915734 | 5458851 | 1 | 2.19 | Trans |
| M7 | 04/12/2012 0:00 | 9 | 49.141452 | -105.298589 | 915734 | 5458849 | 1 | 14.51 | Trans |
| M7 | 04/12/2012 0:00 | 12 | 49.141342 | -105.298483 | 915743 | 5458837 | 1 | 16.61 | Trans |
| M7 | 04/12/2012 0:00 | 15 | 49.141472 | -105.298596 | 915734 | 5458851 | 1 | 75.79 | Trans |
| M7 | 04/12/2012 0:00 | 18 | 49.141728 | -105.299557 | 915661 | 5458874 | 1 | 117.01 | Trans |
| M7 | 04/12/2012 0:00 | 21 | 49.142674 | -105.300253 | 915603 | 5458975 | 1 | 2.82 | Trans |
| M7 | 04/13/2012 0:00 | 0 | 49.142649 | -105.300245 | 915604 | 5458973 | 1 | 13.62 | Trans |
| M7 | 04/13/2012 0:00 | 3 | 49.142527 | -105.300236 | 915605 | 5458959 | 1 | 157.53 | Trans |
| M7 | 04/13/2012 0:00 | 6 | 49.141496 | -105.298760 | 915721 | 5458853 | 1 | 53.48 | Trans |
| M7 | 04/13/2012 0:00 | 9 | 49.141728 | -105.299401 | 915673 | 5458875 | 1 | 75.23 | Trans |
| M7 | 04/13/2012 0:00 | 12 | 49.141294 | -105.298612 | 915734 | 5458831 | 1 | 19.70 | Trans |
| M7 | 04/13/2012 0:00 | 15 | 49.141469 | -105.298566 | 915736 | 5458851 | 1 | 9.95 | Trans |
| M7 | 04/13/2012 0:00 | 18 | 49.141394 | -105.298641 | 915731 | 5458842 | 1 | 339.39 | Trans |
| M7 | 04/13/2012 0:00 | 21 | 49.144369 | -105.299638 | 915633 | 5459167 | 1 | 5242.45 | Trans |
| M7 | 04/14/2012 0:00 | 0 | 49.189420 | -105.278906 | 916765 | 5464286 | 1 | 1705.63 | Trans |
| M7 | 04/14/2012 0:00 | 3 | 49.201171 | -105.293881 | 915576 | 5465509 | 0 | 1156.11 | Trans |
| M7 | 04/14/2012 0:00 | 9 | 49.192495 | -105.285193 | 916281 | 5464593 | 1 | 15.80 | Trans |
| M7 | 04/14/2012 0:00 | 12 | 49.192390 | -105.285048 | 916293 | 5464582 | 1 | 19.00 | Trans |
| M7 | 04/14/2012 0:00 | 15 | 49.192526 | -105.285204 | 916280 | 5464596 | 1 | 3.59 | Trans |
| M7 | 04/14/2012 0:00 | 18 | 49.192495 | -105.285193 | 916281 | 5464593 | 1 | 27.53 | Trans |
| M7 | 04/14/2012 0:00 | 21 | 49.192702 | -105.284989 | 916294 | 5464617 | 1 | 302.90 | Trans |
| M7 | 04/15/2012 0:00 | 0 | 49.193466 | -105.281008 | 916578 | 5464724 | 1 | 1374.35 | Trans |
| M7 | 04/15/2012 0:00 | 3 | 49.181130 | -105.281124 | 916673 | 5463353 | 1 | 1587.17 | Trans |
| M7 | 04/15/2012 0:00 | 6 | 49.188247 | -105.299952 | 915242 | 5464040 | 1 | 10.06 | Trans |
| M7 | 04/15/2012 0:00 | 9 | 49.188299 | -105.299841 | 915250 | 5464046 | 1 | 39.14 | Trans |
| M7 | 04/15/2012 0:00 | 12 | 49.188094 | -105.299406 | 915283 | 5464026 | 1 | 41.22 | Trans |
| M7 | 04/15/2012 0:00 | 15 | 49.188349 | -105.299815 | 915251 | 5464052 | 1 | 215.11 | Trans |
| M7 | 04/15/2012 0:00 | 18 | 49.189265 | -105.302408 | 915055 | 5464139 | 1 | 890.97 | Trans |
| M7 | 04/15/2012 0:00 | 21 | 49.192496 | -105.313570 | 914215 | 5464437 | 1 | 9.50 | Trans |
| M7 | 04/16/2012 0:00 | 0 | 49.192493 | -105.313440 | 914225 | 5464437 | 1 | 8.46 | Trans |
| M7 | 04/16/2012 0:00 | 3 | 49.192418 | -105.313418 | 914227 | 5464429 | 1 | 6.72 | Trans |
| M7 | 04/16/2012 0:00 | 6 | 49.192389 | -105.313338 | 914233 | 5464426 | 1 | 12.90 | Trans |
| M7 | 04/16/2012 0:00 | 9 | 49.192471 | -105.313463 | 914223 | 5464435 | 1 | 225.49 | Trans |
| M7 | 04/16/2012 0:00 | 12 | 49.191417 | -105.310827 | 914424 | 5464332 | 1 | 5.08 | Trans |
| M7 | 04/16/2012 0:00 | 15 | 49.191371 | -105.310825 | 914424 | 5464327 | 1 | 48.01 | Trans |

| | | | | | | | | | |
|---|---|---|---|---|---|---|---|---|---|
| M7 | 04/16/2012 0:00 | 18 | 49.191522 | -105.311442 | 914378 | 5464340 | 1 | 994.23 | Trans |
| M7 | 04/16/2012 0:00 | 21 | 49.193675 | -105.298227 | 915322 | 5464652 | 1 | 5497.79 | Trans |
| M7 | 04/17/2012 0:00 | 0 | 49.206168 | -105.225374 | 920520 | 5466443 | 1 | 2550.26 | Trans |
| M7 | 04/17/2012 0:00 | 3 | 49.210155 | -105.190971 | 922990 | 5467079 | 1 | 14.06 | Trans |
| M7 | 04/17/2012 0:00 | 6 | 49.210029 | -105.190988 | 922990 | 5467065 | 1 | 3.59 | Trans |
| M7 | 04/17/2012 0:00 | 9 | 49.210060 | -105.190999 | 922989 | 5467068 | 1 | 15.02 | Trans |
| M7 | 04/17/2012 0:00 | 12 | 49.209926 | -105.190986 | 922991 | 5467053 | 1 | 51.03 | Trans |
| M7 | 04/17/2012 0:00 | 15 | 49.209855 | -105.190295 | 923042 | 5467049 | 1 | 54.93 | Trans |
| M7 | 04/17/2012 0:00 | 18 | 49.210007 | -105.191011 | 922988 | 5467062 | 1 | 5.75 | Trans |
| M7 | 04/17/2012 0:00 | 21 | 49.210059 | -105.191012 | 922988 | 5467068 | 1 | 4.13 | Trans |
| M7 | 04/18/2012 0:00 | 0 | 49.210031 | -105.190975 | 922991 | 5467065 | 1 | 0.99 | Trans |
| M7 | 04/18/2012 0:00 | 3 | 49.210029 | -105.190988 | 922990 | 5467065 | 1 | 123.01 | Trans |
| M7 | 04/18/2012 0:00 | 6 | 49.210444 | -105.189426 | 923100 | 5467119 | 0 | 10.25 | Trans |
| M7 | 04/18/2012 0:00 | 12 | 49.210518 | -105.189508 | 923093 | 5467127 | 0 | 27.03 | Trans |
| M7 | 04/18/2012 0:00 | 18 | 49.210465 | -105.189147 | 923120 | 5467123 | 1 | 1600.03 | Trans |
| M7 | 04/18/2012 0:00 | 21 | 49.220173 | -105.172993 | 924213 | 5468292 | 1 | 7664.90 | Trans |
| M7 | 04/19/2012 0:00 | 0 | 49.252300 | -105.080096 | 930692 | 5472388 | 1 | 5114.54 | Trans |
| M7 | 04/19/2012 0:00 | 3 | 49.252677 | -105.009979 | 935787 | 5472833 | 0 | 9901.61 | Trans |
| M7 | 04/19/2012 0:00 | 9 | 49.245184 | -104.874733 | 945688 | 5472791 | 0 | 70.89 | Trans |
| M7 | 04/19/2012 0:00 | 15 | 49.245716 | -104.875265 | 945645 | 5472847 | 1 | 9.27 | Trans |
| M7 | 04/19/2012 0:00 | 18 | 49.245639 | -104.875313 | 945642 | 5472838 | 1 | 3125.06 | Trans |
| M7 | 04/19/2012 0:00 | 21 | 49.241422 | -104.832971 | 948760 | 5472621 | 1 | 8462.96 | Trans |
| M7 | 04/20/2012 0:00 | 0 | 49.256750 | -104.719365 | 956880 | 5475006 | 1 | 2293.09 | Trans |
| M7 | 04/20/2012 0:00 | 3 | 49.258698 | -104.688073 | 959136 | 5475412 | 1 | 4522.25 | Trans |
| M7 | 04/20/2012 0:00 | 6 | 49.265049 | -104.626845 | 963528 | 5476492 | 1 | 4.70 | Trans |
| M7 | 04/20/2012 0:00 | 9 | 49.265075 | -104.626895 | 963524 | 5476495 | 1 | 9.39 | Trans |
| M7 | 04/20/2012 0:00 | 12 | 49.264994 | -104.626928 | 963522 | 5476486 | 1 | 9.30 | Trans |
| M7 | 04/20/2012 0:00 | 15 | 49.265077 | -104.626939 | 963521 | 5476495 | 1 | 6.42 | Trans |
| M7 | 04/20/2012 0:00 | 18 | 49.265020 | -104.626921 | 963522 | 5476489 | 1 | 12.71 | Trans |
| M7 | 04/20/2012 0:00 | 21 | 49.265071 | -104.626765 | 963533 | 5476495 | 1 | 9187.95 | Trans |
| M7 | 04/21/2012 0:00 | 0 | 49.251685 | -104.502505 | 972693 | 5475781 | 1 | 7956.26 | Trans |
| M7 | 04/21/2012 0:00 | 3 | 49.248019 | -104.393628 | 980645 | 5476062 | 1 | 2747.11 | Trans |
| M7 | 04/21/2012 0:00 | 6 | 49.253681 | -104.356994 | 983253 | 5476925 | 1 | 3.75 | Trans |
| M7 | 04/21/2012 0:00 | 9 | 49.253651 | -104.356971 | 983255 | 5476922 | 1 | 17.10 | Trans |
| M7 | 04/21/2012 0:00 | 12 | 49.253499 | -104.356940 | 983258 | 5476906 | 1 | 20.67 | Trans |
| M7 | 04/21/2012 0:00 | 15 | 49.253681 | -104.356994 | 983253 | 5476925 | 1 | 9.41 | Trans |
| M7 | 04/21/2012 0:00 | 18 | 49.253628 | -104.356894 | 983260 | 5476920 | 1 | 1325.63 | Trans |
| M7 | 04/21/2012 0:00 | 21 | 49.261284 | -104.342995 | 984196 | 5477860 | 1 | 6793.76 | Trans |
| M7 | 04/22/2012 0:00 | 0 | 49.249893 | -104.251546 | 990956 | 5477186 | 1 | 4613.80 | Trans |
| M7 | 04/22/2012 0:00 | 3 | 49.262334 | -104.191255 | 995214 | 5478963 | 0 | 5453.43 | Trans |
| M7 | 04/22/2012 0:00 | 21 | 49.293411 | -104.133537 | 999094 | 5482795 | 1 | 1962.21 | Trans |
| M7 | 04/23/2012 0:00 | 0 | 49.308108 | -104.118740 | 1000019 | 5484525 | 1 | 4419.91 | Trans |
| M7 | 04/23/2012 0:00 | 3 | 49.303445 | -104.058547 | 1004438 | 5484409 | 0 | 4012.33 | Trans |
| M7 | 04/23/2012 0:00 | 21 | 49.283761 | -104.012502 | 1007984 | 5482533 | 1 | 8470.31 | Trans |
| M7 | 04/24/2012 0:00 | 0 | 49.297097 | -103.898196 | 1016148 | 5484792 | 1 | 10633.47 | Trans |
| M7 | 04/24/2012 0:00 | 3 | 49.274903 | -103.756468 | 1026678 | 5483309 | 1 | 5372.60 | Trans |
| M7 | 04/24/2012 0:00 | 6 | 49.275668 | -103.682869 | 1032016 | 5483912 | 1 | 806.56 | Trans |
| M7 | 04/24/2012 0:00 | 9 | 49.278302 | -103.693160 | 1031240 | 5484132 | 1 | 5.73 | Trans |
| M7 | 04/24/2012 0:00 | 12 | 49.278254 | -103.693134 | 1031243 | 5484126 | 1 | 7.09 | Trans |
| M7 | 04/24/2012 0:00 | 15 | 49.278306 | -103.693190 | 1031238 | 5484132 | 1 | 2.22 | Trans |
| M7 | 04/24/2012 0:00 | 18 | 49.278302 | -103.693160 | 1031240 | 5484132 | 1 | 2.83 | Trans |
| M7 | 04/24/2012 0:00 | 21 | 49.278277 | -103.693154 | 1031241 | 5484129 | 1 | 8197.77 | Trans |
| M7 | 04/25/2012 0:00 | 0 | 49.277407 | -103.580850 | 1039409 | 5484831 | 1 | 8309.63 | Trans |
| M7 | 04/25/2012 0:00 | 3 | 49.283654 | -103.467412 | 1047579 | 5486344 | 1 | 3792.85 | Trans |
| M7 | 04/25/2012 0:00 | 6 | 49.258749 | -103.432059 | 1050426 | 5483837 | 1 | 3.17 | Trans |

| | | | | | | | | | |
|---|---|---|---|---|---|---|---|---|---|
| M7 | 04/25/2012 0:00 | 9 | 49.258751 | -103.432102 | 1050422 | 5483837 | 1 | 12.24 | Trans |
| M7 | 04/25/2012 0:00 | 12 | 49.258642 | -103.432100 | 1050424 | 5483825 | 1 | 12.40 | Trans |
| M7 | 04/25/2012 0:00 | 15 | 49.258749 | -103.432059 | 1050426 | 5483837 | 1 | 4.13 | Trans |
| M7 | 04/25/2012 0:00 | 18 | 49.258720 | -103.432093 | 1050423 | 5483834 | 1 | 1720.58 | Trans |
| M7 | 04/25/2012 0:00 | 21 | 49.253240 | -103.410074 | 1052085 | 5483387 | 1 | 2717.88 | Trans |
| M7 | 04/26/2012 0:00 | 0 | 49.243259 | -103.376136 | 1054664 | 5482528 | 1 | 27.04 | Trans |
| M7 | 04/26/2012 0:00 | 3 | 49.243439 | -103.376384 | 1054644 | 5482546 | 1 | 517.13 | Trans |
| M7 | 04/26/2012 0:00 | 6 | 49.240009 | -103.381143 | 1054336 | 5482130 | 1 | 187.64 | Trans |
| M7 | 04/26/2012 0:00 | 9 | 49.240007 | -103.383711 | 1054150 | 5482111 | 1 | 4.13 | Trans |
| M7 | 04/26/2012 0:00 | 12 | 49.239978 | -103.383674 | 1054153 | 5482108 | 1 | 0.00 | Trans |
| M7 | 04/26/2012 0:00 | 15 | 49.239978 | -103.383674 | 1054153 | 5482108 | 1 | 5.17 | Trans |
| M7 | 04/26/2012 0:00 | 18 | 49.239958 | -103.383738 | 1054148 | 5482106 | 1 | 1183.43 | Trans |
| M7 | 04/26/2012 0:00 | 21 | 49.244028 | -103.368781 | 1055190 | 5482668 | 1 | 3307.21 | Trans |
| M7 | 04/27/2012 0:00 | 0 | 49.227692 | -103.331018 | 1058119 | 5481134 | 1 | 3350.93 | Trans |
| M7 | 04/27/2012 0:00 | 3 | 49.240239 | -103.372679 | 1054949 | 5482218 | 1 | 806.67 | Trans |
| M7 | 04/27/2012 0:00 | 6 | 49.239961 | -103.383711 | 1054150 | 5482106 | 1 | 5.09 | Trans |
| M7 | 04/27/2012 0:00 | 9 | 49.240007 | -103.383711 | 1054150 | 5482111 | 1 | 8.08 | Trans |
| M7 | 04/27/2012 0:00 | 12 | 49.239934 | -103.383718 | 1054150 | 5482103 | 1 | 5.38 | Trans |
| M7 | 04/27/2012 0:00 | 15 | 49.239982 | -103.383704 | 1054150 | 5482109 | 1 | 8.86 | Trans |
| M7 | 04/27/2012 0:00 | 18 | 49.239909 | -103.383655 | 1054155 | 5482101 | 1 | 1938.92 | Trans |
| M7 | 04/27/2012 0:00 | 21 | 49.257107 | -103.387444 | 1053686 | 5483982 | 1 | 7422.47 | Trans |
| M7 | 04/28/2012 0:00 | 0 | 49.292562 | -103.473462 | 1047041 | 5487289 | 1 | 606.29 | Trans |
| M7 | 04/28/2012 0:00 | 3 | 49.297890 | -103.475095 | 1046863 | 5487869 | 1 | 120.51 | Trans |
| M7 | 04/28/2012 0:00 | 6 | 49.296918 | -103.475816 | 1046822 | 5487756 | 1 | 6.43 | Trans |
| M7 | 04/28/2012 0:00 | 9 | 49.296864 | -103.475844 | 1046820 | 5487750 | 1 | 11.39 | Trans |
| M7 | 04/28/2012 0:00 | 12 | 49.296763 | -103.475872 | 1046819 | 5487738 | 1 | 23.10 | Trans |
| M7 | 04/28/2012 0:00 | 15 | 49.296970 | -103.475873 | 1046817 | 5487761 | 1 | 183.69 | Trans |
| M7 | 04/28/2012 0:00 | 18 | 49.297249 | -103.473392 | 1046994 | 5487810 | 1 | 5.76 | Trans |
| M7 | 04/28/2012 0:00 | 21 | 49.297300 | -103.473391 | 1046993 | 5487816 | 1 | 5.76 | Trans |
| M7 | 04/29/2012 0:00 | 0 | 49.297249 | -103.473392 | 1046994 | 5487810 | 1 | 7.08 | Trans |
| M7 | 04/29/2012 0:00 | 3 | 49.297300 | -103.473335 | 1046998 | 5487816 | 1 | 25.10 | Trans |
| M7 | 04/29/2012 0:00 | 6 | 49.297149 | -103.473590 | 1046981 | 5487798 | 1 | 28.27 | Trans |
| M7 | 04/29/2012 0:00 | 9 | 49.297355 | -103.473364 | 1046995 | 5487822 | 1 | 5.21 | Trans |
| M7 | 04/29/2012 0:00 | 12 | 49.297397 | -103.473334 | 1046997 | 5487827 | 1 | 10.71 | Trans |
| M7 | 04/29/2012 0:00 | 15 | 49.297304 | -103.473365 | 1046995 | 5487817 | 1 | 4.13 | Trans |
| M7 | 04/29/2012 0:00 | 18 | 49.297275 | -103.473328 | 1046998 | 5487814 | 1 | 4.13 | Trans |
| M7 | 04/29/2012 0:00 | 21 | 49.297304 | -103.473365 | 1046995 | 5487817 | 1 | 2.21 | Trans |
| M7 | 04/30/2012 0:00 | 0 | 49.297300 | -103.473335 | 1046998 | 5487816 | 1 | 113.14 | Trans |
| M7 | 04/30/2012 0:00 | 3 | 49.297536 | -103.474842 | 1046885 | 5487832 | 1 | 114.28 | Trans |
| M7 | 04/30/2012 0:00 | 6 | 49.297275 | -103.473328 | 1046998 | 5487814 | 1 | 186.31 | Trans |
| M7 | 04/30/2012 0:00 | 9 | 49.296842 | -103.475794 | 1046824 | 5487748 | 1 | 20.91 | Trans |
| M7 | 04/30/2012 0:00 | 12 | 49.296947 | -103.475556 | 1046840 | 5487761 | 1 | 20.80 | Trans |
| M7 | 04/30/2012 0:00 | 15 | 49.296762 | -103.475588 | 1046840 | 5487740 | 1 | 180.03 | Trans |
| M7 | 04/30/2012 0:00 | 18 | 49.297247 | -103.473235 | 1047005 | 5487811 | 1 | 986.01 | Trans |
| M7 | 04/30/2012 0:00 | 21 | 49.293304 | -103.461143 | 1047927 | 5487462 | 1 | 4772.28 | Trans |
| M7 | 05/01/2012 0:00 | 0 | 49.267352 | -103.409182 | 1051992 | 5484960 | 1 | 5219.31 | Trans |
| M7 | 05/01/2012 0:00 | 3 | 49.240543 | -103.350631 | 1056548 | 5482415 | 1 | 5075.83 | Trans |
| M7 | 05/01/2012 0:00 | 6 | 49.207321 | -103.303201 | 1060374 | 5479079 | 1 | 11.62 | Trans |
| M7 | 05/01/2012 0:00 | 9 | 49.207418 | -103.303143 | 1060377 | 5479090 | 1 | 8.08 | Trans |
| M7 | 05/01/2012 0:00 | 12 | 49.207346 | -103.303150 | 1060377 | 5479082 | 1 | 2.98 | Trans |
| M7 | 05/01/2012 0:00 | 15 | 49.207341 | -103.303190 | 1060374 | 5479081 | 1 | 7.57 | Trans |
| M7 | 05/01/2012 0:00 | 18 | 49.207291 | -103.303121 | 1060380 | 5479076 | 1 | 886.33 | Trans |
| M7 | 05/01/2012 0:00 | 21 | 49.203326 | -103.292619 | 1061189 | 5478714 | 1 | 3194.30 | Trans |
| M7 | 05/02/2012 0:00 | 0 | 49.190192 | -103.253813 | 1064162 | 5477546 | 1 | 5475.18 | Trans |
| M7 | 05/02/2012 0:00 | 3 | 49.176059 | -103.182149 | 1069539 | 5476516 | 1 | 679.73 | Trans |

| | | | | | | | | | |
|---|---|---|---|---|---|---|---|---|---|
| M7 | 05/02/2012 0:00 | 6 | 49.174550 | -103.173150 | 1070212 | 5476417 | 1 | 6.41 | Trans |
| M7 | 05/02/2012 0:00 | 9 | 49.174599 | -103.173106 | 1070214 | 5476423 | 1 | 17.79 | Trans |
| M7 | 05/02/2012 0:00 | 12 | 49.174473 | -103.173255 | 1070205 | 5476407 | 1 | 12.76 | Trans |
| M7 | 05/02/2012 0:00 | 15 | 49.174573 | -103.173170 | 1070210 | 5476419 | 1 | 2.98 | Trans |
| M7 | 05/02/2012 0:00 | 18 | 49.174550 | -103.173150 | 1070212 | 5476417 | 1 | 832.74 | Trans |
| M7 | 05/02/2012 0:00 | 21 | 49.168358 | -103.166802 | 1070745 | 5475777 | 1 | 6118.32 | Trans |
| M7 | 05/03/2012 0:00 | 0 | 49.142262 | -103.093308 | 1076400 | 5473440 | 1 | 4102.30 | Trans |
| M7 | 05/03/2012 0:00 | 3 | 49.122075 | -103.046508 | 1080045 | 5471558 | 1 | 1389.92 | Trans |
| M7 | 05/03/2012 0:00 | 6 | 49.126295 | -103.064358 | 1078695 | 5471889 | 1 | 2.84 | Trans |
| M7 | 05/03/2012 0:00 | 9 | 49.126269 | -103.064351 | 1078696 | 5471887 | 1 | 8.78 | Trans |
| M7 | 05/03/2012 0:00 | 12 | 49.126344 | -103.064314 | 1078697 | 5471895 | 1 | 8.78 | Trans |
| M7 | 05/03/2012 0:00 | 15 | 49.126269 | -103.064351 | 1078696 | 5471887 | 1 | 4.13 | Trans |
| M7 | 05/03/2012 0:00 | 18 | 49.126241 | -103.064315 | 1078699 | 5471884 | 1 | 7.09 | Trans |
| M7 | 05/03/2012 0:00 | 21 | 49.126293 | -103.064371 | 1078694 | 5471889 | 1 | 1738.10 | Trans |
| M7 | 05/04/2012 0:00 | 0 | 49.120175 | -103.042555 | 1080355 | 5471378 | 1 | 4376.59 | Trans |
| M7 | 05/04/2012 0:00 | 3 | 49.110273 | -102.984765 | 1084683 | 5470725 | 1 | 1725.90 | Trans |
| M7 | 05/04/2012 0:00 | 6 | 49.115972 | -102.962873 | 1086211 | 5471528 | 1 | 13.08 | Trans |
| M7 | 05/04/2012 0:00 | 9 | 49.116052 | -102.962742 | 1086219 | 5471538 | 1 | 6.56 | Trans |
| M7 | 05/04/2012 0:00 | 12 | 49.116006 | -102.962687 | 1086224 | 5471533 | 1 | 10.23 | Trans |
| M7 | 05/04/2012 0:00 | 15 | 49.116004 | -102.962826 | 1086214 | 5471532 | 1 | 3.48 | Trans |
| M7 | 05/04/2012 0:00 | 18 | 49.115975 | -102.962847 | 1086213 | 5471528 | 1 | 266.18 | Trans |
| M7 | 05/04/2012 0:00 | 21 | 49.113596 | -102.962609 | 1086258 | 5471266 | 1 | 391.87 | Trans |
| M7 | 05/05/2012 0:00 | 0 | 49.111650 | -102.958159 | 1086605 | 5471085 | 1 | 4093.97 | Trans |
| M7 | 05/05/2012 0:00 | 3 | 49.088689 | -102.914615 | 1090052 | 5468876 | 1 | 566.17 | Trans |
| M7 | 05/05/2012 0:00 | 6 | 49.092421 | -102.919842 | 1089627 | 5469249 | 1 | 75.82 | Trans |
| M7 | 05/05/2012 0:00 | 9 | 49.092090 | -102.918939 | 1089696 | 5469220 | 1 | 112.55 | Trans |
| M7 | 05/05/2012 0:00 | 12 | 49.091200 | -102.919660 | 1089655 | 5469115 | 1 | 15.08 | Trans |
| M7 | 05/05/2012 0:00 | 15 | 49.091069 | -102.919610 | 1089660 | 5469101 | 1 | 110.76 | Trans |
| M7 | 05/05/2012 0:00 | 18 | 49.090376 | -102.918529 | 1089747 | 5469032 | 1 | 825.39 | Trans |
| M7 | 05/05/2012 0:00 | 21 | 49.086075 | -102.909373 | 1090466 | 5468627 | 1 | 542.31 | Trans |
| M7 | 05/06/2012 0:00 | 0 | 49.084030 | -102.916081 | 1090001 | 5468347 | 1 | 592.82 | Trans |
| M7 | 05/06/2012 0:00 | 3 | 49.089198 | -102.917938 | 1089804 | 5468906 | 1 | 395.49 | Trans |
| M7 | 05/06/2012 0:00 | 6 | 49.092479 | -102.919970 | 1089617 | 5469255 | 1 | 141.71 | Trans |
| M7 | 05/06/2012 0:00 | 9 | 49.091762 | -102.918375 | 1089741 | 5469188 | 1 | 10.85 | Trans |
| M7 | 05/06/2012 0:00 | 12 | 49.091859 | -102.918373 | 1089741 | 5469198 | 1 | 9.31 | Trans |
| M7 | 05/06/2012 0:00 | 15 | 49.091787 | -102.918438 | 1089737 | 5469190 | 1 | 2.83 | Trans |
| M7 | 05/06/2012 0:00 | 18 | 49.091762 | -102.918431 | 1089737 | 5469187 | 1 | 599.90 | Trans |
| M7 | 05/06/2012 0:00 | 21 | 49.086409 | -102.917721 | 1089853 | 5468598 | 1 | 1657.38 | Trans |
| M7 | 05/07/2012 0:00 | 0 | 49.080450 | -102.897022 | 1091433 | 5468099 | 1 | 10.29 | Trans |
| M7 | 05/07/2012 0:00 | 3 | 49.080518 | -102.896927 | 1091439 | 5468107 | 1 | 54.53 | Trans |
| M7 | 05/07/2012 0:00 | 6 | 49.080087 | -102.897276 | 1091419 | 5468057 | 1 | 20.16 | Trans |
| M7 | 05/07/2012 0:00 | 9 | 49.080267 | -102.897282 | 1091417 | 5468077 | 1 | 28.34 | Trans |
| M7 | 05/07/2012 0:00 | 12 | 49.080243 | -102.896897 | 1091445 | 5468077 | 1 | 21.27 | Trans |
| M7 | 05/07/2012 0:00 | 15 | 49.080213 | -102.897183 | 1091424 | 5468072 | 1 | 8.78 | Trans |
| M7 | 05/07/2012 0:00 | 18 | 49.080288 | -102.897148 | 1091426 | 5468080 | 1 | 620.09 | Trans |
| M7 | 05/07/2012 0:00 | 21 | 49.078371 | -102.889213 | 1092028 | 5467930 | 1 | 357.61 | Trans |
| M7 | 05/08/2012 0:00 | 0 | 49.076631 | -102.885119 | 1092347 | 5467769 | 1 | 205.52 | Trans |
| M7 | 05/08/2012 0:00 | 3 | 49.076321 | -102.882357 | 1092552 | 5467756 | 0 | 425.67 | Trans |
| M7 | 05/08/2012 0:00 | 21 | 49.077758 | -102.887733 | 1092143 | 5467873 | 1 | 311.99 | Trans |
| M7 | 05/09/2012 0:00 | 0 | 49.075121 | -102.886323 | 1092277 | 5467592 | 1 | 265.02 | Trans |
| M7 | 05/09/2012 0:00 | 3 | 49.077470 | -102.886843 | 1092211 | 5467848 | 0 | 368.88 | Trans |
| M7 | 05/09/2012 0:00 | 21 | 49.078984 | -102.891314 | 1091867 | 5467981 | 1 | 6025.82 | Trans |
| M7 | 05/10/2012 0:00 | 0 | 49.049723 | -102.822299 | 1097252 | 5465277 | 1 | 4041.80 | Trans |
| M7 | 05/10/2012 0:00 | 3 | 49.044782 | -102.767746 | 1101292 | 5465162 | 1 | 329.02 | Trans |
| M7 | 05/10/2012 0:00 | 6 | 49.047443 | -102.769672 | 1101119 | 5465442 | 1 | 135.62 | Trans |

| | | | | | | | | | |
|---|---|---|---|---|---|---|---|---|---|
| M7 | 05/10/2012 0:00 | 9 | 49.048009 | -102.771307 | 1100993 | 5465492 | 1 | 75.17 | Trans |
| M7 | 05/10/2012 0:00 | 12 | 49.048293 | -102.772236 | 1100922 | 5465516 | 1 | 14.38 | Trans |
| M7 | 05/10/2012 0:00 | 15 | 49.048291 | -102.772432 | 1100908 | 5465515 | 1 | 2.44 | Trans |
| M7 | 05/10/2012 0:00 | 18 | 49.048309 | -102.772451 | 1100906 | 5465516 | 1 | 408.01 | Trans |
| M7 | 05/10/2012 0:00 | 21 | 49.051948 | -102.771941 | 1100899 | 5465924 | 1 | 2189.45 | Trans |
| M7 | 05/11/2012 0:00 | 0 | 49.046343 | -102.743355 | 1103053 | 5465530 | 1 | 5469.11 | Trans |
| M7 | 05/11/2012 0:00 | 3 | 49.044042 | -102.668932 | 1108511 | 5465873 | 1 | 164.39 | Trans |
| M7 | 05/11/2012 0:00 | 6 | 49.044303 | -102.666728 | 1108669 | 5465920 | 1 | 56.35 | Trans |
| M7 | 05/11/2012 0:00 | 9 | 49.043808 | -102.666573 | 1108686 | 5465866 | 1 | 12.85 | Trans |
| M7 | 05/11/2012 0:00 | 12 | 49.043885 | -102.666703 | 1108676 | 5465873 | 1 | 9.04 | Trans |
| M7 | 05/11/2012 0:00 | 15 | 49.043805 | -102.666725 | 1108675 | 5465864 | 1 | 3.59 | Trans |
| M7 | 05/11/2012 0:00 | 18 | 49.043837 | -102.666734 | 1108674 | 5465868 | 1 | 197.30 | Trans |
| M7 | 05/11/2012 0:00 | 21 | 49.045604 | -102.666699 | 1108655 | 5466064 | 1 | 4312.10 | Trans |
| M7 | 05/12/2012 0:00 | 0 | 49.031298 | -102.612148 | 1112812 | 5464918 | 1 | 8647.41 | Trans |
| M7 | 05/12/2012 0:00 | 3 | 49.052850 | -102.499027 | 1120798 | 5468236 | 1 | 3180.05 | Trans |
| M7 | 05/12/2012 0:00 | 6 | 49.064540 | -102.459526 | 1123532 | 5469859 | 1 | 20.51 | Trans |
| M7 | 05/12/2012 0:00 | 9 | 49.064692 | -102.459368 | 1123542 | 5469877 | 1 | 4.24 | Trans |
| M7 | 05/12/2012 0:00 | 12 | 49.064674 | -102.459419 | 1123538 | 5469875 | 1 | 7.09 | Trans |
| M7 | 05/12/2012 0:00 | 15 | 49.064623 | -102.459476 | 1123535 | 5469869 | 1 | 8.09 | Trans |
| M7 | 05/12/2012 0:00 | 18 | 49.064695 | -102.459468 | 1123535 | 5469877 | 1 | 2461.27 | Trans |
| M7 | 05/12/2012 0:00 | 21 | 49.078879 | -102.433801 | 1125227 | 5471664 | 1 | 3364.01 | Trans |
| M7 | 05/13/2012 0:00 | 0 | 49.071433 | -102.389384 | 1128560 | 5471207 | 1 | 4715.34 | Trans |
| M7 | 05/13/2012 0:00 | 3 | 49.063293 | -102.326350 | 1133260 | 5470831 | 1 | 2809.83 | Trans |
| M7 | 05/13/2012 0:00 | 6 | 49.069437 | -102.289232 | 1135888 | 5471826 | 1 | 11.22 | Trans |
| M7 | 05/13/2012 0:00 | 9 | 49.069511 | -102.289336 | 1135880 | 5471833 | 0 | 4.59 | Trans |
| M7 | 05/13/2012 0:00 | 18 | 49.069536 | -102.289285 | 1135883 | 5471836 | 1 | 43.00 | Trans |
| M7 | 05/13/2012 0:00 | 21 | 49.069920 | -102.289311 | 1135876 | 5471879 | 1 | 734.62 | Trans |
| M7 | 05/14/2012 0:00 | 0 | 49.066999 | -102.280345 | 1136568 | 5471630 | 1 | 7442.96 | Trans |
| M7 | 05/14/2012 0:00 | 3 | 49.107117 | -102.199371 | 1141951 | 5476770 | 1 | 1669.99 | Trans |
| M7 | 05/14/2012 0:00 | 6 | 49.120277 | -102.188577 | 1142567 | 5478322 | 0 | 60.60 | Trans |
| M7 | 05/14/2012 0:00 | 15 | 49.120811 | -102.188723 | 1142549 | 5478380 | 1 | 4.14 | Trans |
| M7 | 05/14/2012 0:00 | 18 | 49.120812 | -102.188779 | 1142545 | 5478380 | 1 | 49.53 | Trans |
| M7 | 05/14/2012 0:00 | 21 | 49.120384 | -102.188603 | 1142563 | 5478334 | 1 | 3390.70 | Trans |
| M7 | 05/15/2012 0:00 | 0 | 49.138531 | -102.151539 | 1145026 | 5480665 | 1 | 1082.06 | Trans |
| M7 | 05/15/2012 0:00 | 3 | 49.147566 | -102.156851 | 1144521 | 5481622 | 1 | 163.56 | Trans |
| M7 | 05/15/2012 0:00 | 6 | 49.147622 | -102.159081 | 1144358 | 5481609 | 1 | 11.45 | Trans |
| M7 | 05/15/2012 0:00 | 9 | 49.147618 | -102.158925 | 1144370 | 5481610 | 0 | 12.77 | Trans |
| M7 | 05/15/2012 0:00 | 15 | 49.147515 | -102.158998 | 1144366 | 5481598 | 0 | 177.61 | Trans |
| M7 | 05/15/2012 0:00 | 21 | 49.148007 | -102.156695 | 1144527 | 5481672 | 1 | 1521.81 | Trans |
| M7 | 05/16/2012 0:00 | 0 | 49.160315 | -102.165588 | 1143719 | 5482962 | 1 | 36.32 | Trans |
| M7 | 05/16/2012 0:00 | 3 | 49.160109 | -102.165971 | 1143694 | 5482936 | 1 | 167.98 | Trans |
| M7 | 05/16/2012 0:00 | 6 | 49.161595 | -102.165626 | 1143699 | 5483104 | 1 | 82.12 | Trans |
| M7 | 05/16/2012 0:00 | 9 | 49.161317 | -102.164588 | 1143779 | 5483082 | 1 | 11.63 | Trans |
| M7 | 05/16/2012 0:00 | 12 | 49.161413 | -102.164528 | 1143782 | 5483093 | 0 | 15.37 | Trans |
| M7 | 05/16/2012 0:00 | 18 | 49.161412 | -102.164738 | 1143766 | 5483091 | 1 | 168.55 | Trans |
| M7 | 05/16/2012 0:00 | 21 | 49.160135 | -102.165964 | 1143694 | 5482939 | 1 | 2601.03 | Trans |
| M7 | 05/17/2012 0:00 | 0 | 49.183408 | -102.165147 | 1143450 | 5485528 | 1 | 45.20 | Trans |
| M7 | 05/17/2012 0:00 | 3 | 49.183022 | -102.165331 | 1143441 | 5485484 | 1 | 1221.03 | Trans |
| M7 | 05/17/2012 0:00 | 6 | 49.172275 | -102.168352 | 1143362 | 5484265 | 0 | 128.40 | Trans |
| M7 | 05/17/2012 0:00 | 12 | 49.171634 | -102.169807 | 1143264 | 5484182 | 1 | 32.98 | Trans |
| M7 | 05/17/2012 0:00 | 15 | 49.171917 | -102.169680 | 1143270 | 5484214 | 0 | 2568.64 | Trans |
| M7 | 05/18/2012 0:00 | 0 | 49.186862 | -102.143036 | 1145013 | 5486101 | 1 | 7469.80 | Trans |
| M7 | 05/18/2012 0:00 | 3 | 49.253573 | -102.136428 | 1144619 | 5493560 | 0 | 3927.54 | Trans |
| M7 | 05/18/2012 0:00 | 9 | 49.288492 | -102.130280 | 1144608 | 5497488 | 1 | 4.72 | Trans |
| M7 | 05/18/2012 0:00 | 12 | 49.288519 | -102.130329 | 1144604 | 5497491 | 1 | 3.01 | Trans |

| | | | | | | | | | |
|---|---|---|---|---|---|---|---|---|---|
| M7 | 05/18/2012 0:00 | 15 | 49.288493 | -102.130336 | 1144603 | 5497488 | 1 | 1.99 | Trans |
| M7 | 05/18/2012 0:00 | 18 | 49.288495 | -102.130309 | 1144605 | 5497488 | 1 | 5.12 | Trans |
| M7 | 05/18/2012 0:00 | 21 | 49.288495 | -102.130379 | 1144600 | 5497487 | 1 | 7923.17 | Trans |
| M7 | 05/19/2012 0:00 | 0 | 49.358022 | -102.151687 | 1142143 | 5505020 | 1 | 3313.58 | Trans |
| M7 | 05/19/2012 0:00 | 3 | 49.387668 | -102.152853 | 1141670 | 5508300 | 1 | 88.11 | Trans |
| M7 | 05/19/2012 0:00 | 6 | 49.388333 | -102.152203 | 1141708 | 5508379 | 0 | 10.44 | Trans |
| M7 | 05/19/2012 0:00 | 15 | 49.388416 | -102.152139 | 1141712 | 5508389 | 1 | 9.26 | Trans |
| M7 | 05/19/2012 0:00 | 18 | 49.388335 | -102.152119 | 1141714 | 5508380 | 1 | 51.81 | Trans |
| M7 | 05/19/2012 0:00 | 21 | 49.388105 | -102.152736 | 1141672 | 5508349 | 1 | 3877.97 | Trans |
| M7 | 05/20/2012 0:00 | 0 | 49.422230 | -102.162434 | 1140523 | 5512053 | 0 | 8977.71 | Trans |
| M7 | 05/20/2012 0:00 | 6 | 49.499950 | -102.193714 | 1137243 | 5520410 | 1 | 58.76 | Trans |
| M7 | 05/20/2012 0:00 | 9 | 49.500055 | -102.192923 | 1137299 | 5520428 | 1 | 4.14 | Trans |
| M7 | 05/20/2012 0:00 | 12 | 49.500056 | -102.192980 | 1137295 | 5520428 | 1 | 3.49 | Trans |
| M7 | 05/20/2012 0:00 | 15 | 49.500028 | -102.193001 | 1137294 | 5520425 | 1 | 6.57 | Trans |
| M7 | 05/20/2012 0:00 | 18 | 49.499981 | -102.192946 | 1137298 | 5520420 | 1 | 4.13 | Trans |
| M7 | 05/20/2012 0:00 | 21 | 49.499952 | -102.192981 | 1137296 | 5520416 | 1 | 3908.38 | Trans |
| M7 | 05/21/2012 0:00 | 0 | 49.534279 | -102.203319 | 1136101 | 5524137 | 1 | 7948.35 | Trans |
| M7 | 05/21/2012 0:00 | 3 | 49.604322 | -102.184187 | 1136565 | 5532072 | 1 | 5308.96 | Trans |
| M7 | 05/21/2012 0:00 | 6 | 49.647714 | -102.213994 | 1133848 | 5536634 | 1 | 5.75 | Trans |
| M7 | 05/21/2012 0:00 | 9 | 49.647662 | -102.213996 | 1133849 | 5536628 | 1 | 5.75 | Trans |
| M7 | 05/21/2012 0:00 | 12 | 49.647714 | -102.213994 | 1133848 | 5536634 | 1 | 47.56 | Trans |
| M7 | 05/21/2012 0:00 | 15 | 49.647383 | -102.214406 | 1133823 | 5536593 | 0 | 7475.43 | Trans |
| M7 | 05/22/2012 0:00 | 6 | 49.713658 | -102.200262 | 1133974 | 5544067 | 1 | 2.97 | Trans |
| M7 | 05/22/2012 0:00 | 9 | 49.713684 | -102.200271 | 1133973 | 5544070 | 0 | 139.23 | Trans |
| M7 | 05/22/2012 0:00 | 18 | 49.714887 | -102.200775 | 1133921 | 5544199 | 1 | 718.39 | Trans |
| M7 | 05/22/2012 0:00 | 21 | 49.721132 | -102.203130 | 1133670 | 5544872 | 1 | 2706.01 | Trans |
| M7 | 05/23/2012 0:00 | 0 | 49.742768 | -102.219926 | 1132179 | 5547130 | 1 | 1603.64 | Trans |
| M7 | 05/23/2012 0:00 | 3 | 49.745765 | -102.241587 | 1130582 | 5547280 | 1 | 23.12 | Trans |
| M7 | 05/23/2012 0:00 | 6 | 49.745559 | -102.241591 | 1130584 | 5547257 | 1 | 21.66 | Local |
| M7 | 05/23/2012 0:00 | 9 | 49.745374 | -102.241686 | 1130580 | 5547235 | 1 | 22.68 | Local |
| M7 | 05/23/2012 0:00 | 12 | 49.745530 | -102.241485 | 1130593 | 5547254 | 1 | 42.96 | Local |
| M7 | 05/23/2012 0:00 | 15 | 49.745891 | -102.241690 | 1130573 | 5547293 | 1 | 14.76 | Local |
| M7 | 05/23/2012 0:00 | 18 | 49.745834 | -102.241874 | 1130561 | 5547285 | 1 | 24.04 | Local |
| M7 | 05/23/2012 0:00 | 21 | 49.745683 | -102.241637 | 1130580 | 5547270 | 1 | 638.50 | Local |
| M7 | 05/24/2012 0:00 | 0 | 49.746349 | -102.250397 | 1129941 | 5547270 | 1 | 378.77 | Local |
| M7 | 05/24/2012 0:00 | 3 | 49.743281 | -102.252623 | 1129821 | 5546911 | 1 | 320.13 | Local |
| M7 | 05/24/2012 0:00 | 6 | 49.742332 | -102.256796 | 1129533 | 5546770 | 1 | 9.26 | Local |
| M7 | 05/24/2012 0:00 | 9 | 49.742250 | -102.256775 | 1129536 | 5546761 | 1 | 152.45 | Local |
| M7 | 05/24/2012 0:00 | 12 | 49.743306 | -102.255440 | 1129618 | 5546890 | 1 | 17.67 | Local |
| M7 | 05/24/2012 0:00 | 15 | 49.743203 | -102.255626 | 1129606 | 5546877 | 0 | 22.32 | Local |
| M7 | 05/25/2012 0:00 | 0 | 49.743175 | -102.255321 | 1129628 | 5546876 | 1 | 7.51 | Local |
| M7 | 05/25/2012 0:00 | 3 | 49.743205 | -102.255414 | 1129621 | 5546879 | 1 | 2.19 | Local |
| M7 | 05/25/2012 0:00 | 6 | 49.743224 | -102.255420 | 1129621 | 5546881 | 1 | 6.44 | Local |
| M7 | 05/25/2012 0:00 | 9 | 49.743279 | -102.255391 | 1129622 | 5546887 | 1 | 5.73 | Local |
| M7 | 05/25/2012 0:00 | 12 | 49.743327 | -102.255416 | 1129620 | 5546892 | 1 | 8.56 | Local |
| M7 | 05/25/2012 0:00 | 15 | 49.743251 | -102.255412 | 1129621 | 5546884 | 0 | 1.99 | Local |
| M7 | 05/25/2012 0:00 | 21 | 49.743248 | -102.255439 | 1129619 | 5546883 | 1 | 22.95 | Local |
| M7 | 05/26/2012 0:00 | 0 | 49.743070 | -102.255281 | 1129633 | 5546865 | 1 | 70.14 | Local |
| M7 | 05/26/2012 0:00 | 3 | 49.742484 | -102.254932 | 1129665 | 5546803 | 0 | 90.37 | Local |
| M7 | 05/26/2012 0:00 | 9 | 49.743254 | -102.255314 | 1129628 | 5546885 | 1 | 0.00 | Local |
| M7 | 05/26/2012 0:00 | 12 | 49.743254 | -102.255314 | 1129628 | 5546885 | 1 | 8.34 | Local |
| M7 | 05/26/2012 0:00 | 15 | 49.743224 | -102.255420 | 1129621 | 5546881 | 1 | 5.12 | Local |
| M7 | 05/26/2012 0:00 | 18 | 49.743224 | -102.255490 | 1129616 | 5546880 | 1 | 31.73 | Local |
| M7 | 05/26/2012 0:00 | 21 | 49.743406 | -102.255154 | 1129637 | 5546903 | 0 | 23.19 | Local |
| M7 | 05/27/2012 0:00 | 12 | 49.743204 | -102.255229 | 1129635 | 5546880 | 1 | 65.02 | Local |

| | | | | | | | | | |
|---|---|---|---|---|---|---|---|---|---|
| M7 | 05/27/2012 0:00 | 15 | 49.742661 | -102.254905 | 1129665 | 5546823 | 1 | 87.96 | Local |
| M7 | 05/27/2012 0:00 | 18 | 49.743278 | -102.255660 | 1129603 | 5546885 | 1 | 43.81 | Local |
| M7 | 05/27/2012 0:00 | 21 | 49.743305 | -102.255057 | 1129646 | 5546893 | 1 | 28.78 | Local |
| M7 | 05/28/2012 0:00 | 0 | 49.743223 | -102.255433 | 1129620 | 5546880 | 0 | 6.81 | Local |
| M7 | 05/28/2012 0:00 | 6 | 49.743206 | -102.255343 | 1129626 | 5546879 | 1 | 9.01 | Local |
| M7 | 05/28/2012 0:00 | 9 | 49.743226 | -102.255463 | 1129617 | 5546881 | 1 | 9.47 | Local |
| M7 | 05/28/2012 0:00 | 12 | 49.743305 | -102.255511 | 1129613 | 5546889 | 1 | 20.80 | Local |
| M7 | 05/28/2012 0:00 | 15 | 49.743125 | -102.255437 | 1129621 | 5546870 | 1 | 16.56 | Local |
| M7 | 05/28/2012 0:00 | 18 | 49.743122 | -102.255208 | 1129637 | 5546871 | 1 | 17.80 | Local |
| M7 | 05/28/2012 0:00 | 21 | 49.743250 | -102.255355 | 1129625 | 5546884 | 1 | 3.00 | Local |
| M7 | 05/29/2012 0:00 | 0 | 49.743223 | -102.255363 | 1129625 | 5546881 | 1 | 13.13 | Local |
| M7 | 05/29/2012 0:00 | 3 | 49.743205 | -102.255542 | 1129612 | 5546878 | 1 | 13.66 | Local |
| M7 | 05/29/2012 0:00 | 6 | 49.743279 | -102.255391 | 1129622 | 5546887 | 1 | 4.14 | Local |
| M7 | 05/29/2012 0:00 | 9 | 49.743250 | -102.255355 | 1129625 | 5546884 | 1 | 8.81 | Local |
| M7 | 05/29/2012 0:00 | 12 | 49.743306 | -102.255440 | 1129618 | 5546890 | 1 | 17.57 | Local |
| M7 | 05/29/2012 0:00 | 15 | 49.743251 | -102.255668 | 1129602 | 5546882 | 1 | 22.33 | Local |
| M7 | 05/29/2012 0:00 | 18 | 49.743223 | -102.255363 | 1129625 | 5546881 | 1 | 129.59 | Local |
| M7 | 05/29/2012 0:00 | 21 | 49.742916 | -102.253637 | 1129753 | 5546862 | 1 | 2266.83 | Local |
| M7 | 05/30/2012 0:00 | 0 | 49.762963 | -102.248810 | 1129839 | 5549127 | 1 | 491.44 | Local |
| M7 | 05/30/2012 0:00 | 3 | 49.763915 | -102.242180 | 1130303 | 5549288 | 1 | 783.70 | Local |
| M7 | 05/30/2012 0:00 | 6 | 49.767701 | -102.233064 | 1130908 | 5549786 | 0 | 747.79 | Local |
| M7 | 05/30/2012 0:00 | 12 | 49.773561 | -102.228069 | 1131191 | 5550478 | 1 | 114.06 | Local |
| M7 | 05/30/2012 0:00 | 15 | 49.772541 | -102.227993 | 1131210 | 5550366 | 1 | 558.71 | Local |
| M7 | 05/30/2012 0:00 | 18 | 49.774482 | -102.235109 | 1130673 | 5550521 | 1 | 1539.42 | Local |
| M7 | 05/30/2012 0:00 | 21 | 49.785926 | -102.223259 | 1131375 | 5551891 | 1 | 46.39 | Local |
| M7 | 05/31/2012 0:00 | 0 | 49.786232 | -102.223692 | 1131340 | 5551921 | 1 | 352.58 | Local |
| M7 | 05/31/2012 0:00 | 3 | 49.786108 | -102.228562 | 1130991 | 5551866 | 1 | 21.52 | Local |
| M7 | 05/31/2012 0:00 | 6 | 49.786183 | -102.228836 | 1130971 | 5551872 | 1 | 354.80 | Local |
| M7 | 05/31/2012 0:00 | 9 | 49.787496 | -102.224369 | 1131274 | 5552056 | 1 | 49.73 | Local |
| M7 | 05/31/2012 0:00 | 12 | 49.787820 | -102.223899 | 1131304 | 5552096 | 1 | 18.22 | Local |
| M7 | 05/31/2012 0:00 | 15 | 49.787793 | -102.223651 | 1131322 | 5552095 | 1 | 389.74 | Local |
| M7 | 05/31/2012 0:00 | 18 | 49.786311 | -102.228528 | 1130991 | 5551889 | 1 | 584.61 | Local |
| M7 | 05/31/2012 0:00 | 21 | 49.791431 | -102.230194 | 1130804 | 5552443 | 1 | 2048.27 | Local |
| M7 | 06/01/2012 0:00 | 0 | 49.793559 | -102.258323 | 1128756 | 5552441 | 1 | 1841.78 | Local |
| M7 | 06/01/2012 0:00 | 3 | 49.790205 | -102.233390 | 1130591 | 5552280 | 1 | 263.76 | Local |
| M7 | 06/01/2012 0:00 | 6 | 49.791459 | -102.230301 | 1130796 | 5552445 | 0 | 5.82 | Local |
| M7 | 06/01/2012 0:00 | 12 | 49.791411 | -102.230330 | 1130795 | 5552440 | 1 | 1150.20 | Local |
| M7 | 06/01/2012 0:00 | 15 | 49.797804 | -102.242795 | 1129816 | 5553043 | 0 | 3510.53 | Local |
| M7 | 06/01/2012 0:00 | 21 | 49.779682 | -102.282445 | 1127204 | 5550698 | 0 | 4072.40 | Local |
| M7 | 06/02/2012 0:00 | 3 | 49.786800 | -102.227233 | 1131078 | 5551954 | 1 | 260.52 | Local |
| M7 | 06/02/2012 0:00 | 6 | 49.786566 | -102.223649 | 1131338 | 5551958 | 1 | 738.69 | Local |
| M7 | 06/02/2012 0:00 | 9 | 49.791581 | -102.230303 | 1130795 | 5552459 | 0 | 35.68 | Local |
| M7 | 06/02/2012 0:00 | 15 | 49.791893 | -102.230410 | 1130783 | 5552492 | 1 | 631.19 | Local |
| M7 | 06/02/2012 0:00 | 18 | 49.795606 | -102.236988 | 1130262 | 5552849 | 1 | 461.56 | Local |
| M7 | 06/02/2012 0:00 | 21 | 49.796191 | -102.243306 | 1129800 | 5552860 | 1 | 2827.92 | Local |
| M7 | 06/03/2012 0:00 | 0 | 49.814854 | -102.269727 | 1127660 | 5554708 | 0 | 6135.49 | Local |
| M7 | 06/03/2012 0:00 | 6 | 49.869769 | -102.268774 | 1127013 | 5560809 | 0 | 603.44 | Local |
| M7 | 06/03/2012 0:00 | 12 | 49.868797 | -102.260554 | 1127615 | 5560771 | 0 | 2113.98 | Local |
| M7 | 06/03/2012 0:00 | 21 | 49.862293 | -102.233064 | 1129672 | 5560281 | 0 | 3715.68 | Local |
| M7 | 06/04/2012 0:00 | 6 | 49.845940 | -102.188275 | 1133099 | 5558846 | 1 | 9.31 | Local |
| M7 | 06/04/2012 0:00 | 9 | 49.845856 | -102.188268 | 1133101 | 5558837 | 0 | 7.56 | Local |
| M7 | 06/04/2012 0:00 | 15 | 49.845907 | -102.188337 | 1133095 | 5558842 | 1 | 2.95 | Local |
| M7 | 06/04/2012 0:00 | 18 | 49.845886 | -102.188361 | 1133094 | 5558840 | 1 | 248.61 | Local |
| M7 | 06/04/2012 0:00 | 21 | 49.847545 | -102.186068 | 1133237 | 5559043 | 1 | 74.54 | Local |
| M7 | 06/05/2012 0:00 | 0 | 49.848114 | -102.185530 | 1133268 | 5559111 | 1 | 33.90 | Local |

| | | | | | | | | | |
|---|---|---|---|---|---|---|---|---|---|
| M7 | 06/05/2012 0:00 | 3 | 49.847827 | -102.185683 | 1133260 | 5559078 | 1 | 615.37 | Local |
| M7 | 06/05/2012 0:00 | 6 | 49.844582 | -102.192564 | 1132809 | 5558659 | 1 | 6.74 | Local |
| M7 | 06/05/2012 0:00 | 9 | 49.844528 | -102.192522 | 1132813 | 5558654 | 1 | 17.80 | Local |
| M7 | 06/05/2012 0:00 | 12 | 49.844656 | -102.192669 | 1132801 | 5558667 | 1 | 1024.13 | Local |
| M7 | 06/05/2012 0:00 | 15 | 49.837051 | -102.200582 | 1132333 | 5557756 | 1 | 1149.09 | Local |
| M7 | 06/05/2012 0:00 | 18 | 49.832056 | -102.214482 | 1131401 | 5557084 | 1 | 1762.54 | Local |
| M7 | 06/05/2012 0:00 | 21 | 49.828272 | -102.238158 | 1129751 | 5556463 | 1 | 2727.25 | Local |
| M7 | 06/06/2012 0:00 | 0 | 49.807069 | -102.256854 | 1128686 | 5553953 | 0 | 1740.36 | Local |
| M7 | 06/06/2012 0:00 | 6 | 49.796861 | -102.238674 | 1130124 | 5552974 | 1 | 65.55 | Local |
| M7 | 06/06/2012 0:00 | 9 | 49.797322 | -102.239233 | 1130078 | 5553020 | 1 | 15.46 | Local |
| M7 | 06/06/2012 0:00 | 12 | 49.797246 | -102.239412 | 1130066 | 5553010 | 1 | 39.69 | Local |
| M7 | 06/06/2012 0:00 | 15 | 49.797601 | -102.239418 | 1130061 | 5553050 | 1 | 203.16 | Local |
| M7 | 06/06/2012 0:00 | 18 | 49.795809 | -102.239896 | 1130050 | 5552847 | 1 | 194.53 | Local |
| M7 | 06/06/2012 0:00 | 21 | 49.797093 | -102.238079 | 1130164 | 5553004 | 1 | 243.03 | Local |
| M7 | 06/07/2012 0:00 | 0 | 49.796246 | -102.241174 | 1129953 | 5552884 | 1 | 41.46 | Local |
| M7 | 06/07/2012 0:00 | 3 | 49.795883 | -102.241053 | 1129966 | 5552845 | 1 | 11.97 | Local |
| M7 | 06/07/2012 0:00 | 6 | 49.795964 | -102.240945 | 1129973 | 5552855 | 1 | 50.12 | Local |
| M7 | 06/07/2012 0:00 | 9 | 49.796273 | -102.240442 | 1130005 | 5552893 | 0 | 81.92 | Local |
| M7 | 06/07/2012 0:00 | 18 | 49.795561 | -102.240714 | 1129995 | 5552812 | 0 | 24.33 | Local |
| M7 | 06/08/2012 0:00 | 18 | 49.795348 | -102.240645 | 1130003 | 5552789 | 1 | 67.61 | Local |
| M7 | 06/08/2012 0:00 | 21 | 49.795907 | -102.241001 | 1129970 | 5552848 | 1 | 26.16 | Local |
| M7 | 06/09/2012 0:00 | 0 | 49.795714 | -102.240796 | 1129987 | 5552829 | 1 | 15.24 | Local |
| M7 | 06/09/2012 0:00 | 3 | 49.795809 | -102.240948 | 1129975 | 5552838 | 1 | 21.94 | Local |
| M7 | 06/09/2012 0:00 | 6 | 49.795655 | -102.241135 | 1129963 | 5552819 | 0 | 342.83 | Local |
| M7 | 06/09/2012 0:00 | 12 | 49.798139 | -102.243918 | 1129731 | 5553071 | 1 | 1059.72 | Local |
| M7 | 06/09/2012 0:00 | 15 | 49.806790 | -102.249930 | 1129186 | 5553980 | 1 | 561.51 | Local |
| M7 | 06/09/2012 0:00 | 18 | 49.810453 | -102.244613 | 1129520 | 5554432 | 1 | 557.25 | Local |
| M7 | 06/09/2012 0:00 | 21 | 49.815415 | -102.243823 | 1129512 | 5554989 | 0 | 524.86 | Local |
| M7 | 06/10/2012 0:00 | 3 | 49.820108 | -102.243518 | 1129473 | 5555512 | 1 | 5.40 | Local |
| M7 | 06/10/2012 0:00 | 6 | 49.820082 | -102.243455 | 1129478 | 5555510 | 1 | 4.72 | Local |
| M7 | 06/10/2012 0:00 | 9 | 49.820055 | -102.243405 | 1129482 | 5555507 | 1 | 6.18 | Local |
| M7 | 06/10/2012 0:00 | 12 | 49.820080 | -102.243482 | 1129476 | 5555509 | 1 | 886.37 | Local |
| M7 | 06/10/2012 0:00 | 15 | 49.827813 | -102.246218 | 1129179 | 5556344 | 1 | 2130.59 | Local |
| M7 | 06/10/2012 0:00 | 18 | 49.846749 | -102.242725 | 1129182 | 5558475 | 1 | 1877.25 | Local |
| M7 | 06/10/2012 0:00 | 21 | 49.861551 | -102.230426 | 1129871 | 5560221 | 1 | 2938.09 | Local |
| M7 | 06/11/2012 0:00 | 0 | 49.874454 | -102.265879 | 1127160 | 5561354 | 1 | 2556.92 | Local |
| M7 | 06/11/2012 0:00 | 3 | 49.852388 | -102.275276 | 1126773 | 5558826 | 1 | 3446.28 | Local |
| M7 | 06/11/2012 0:00 | 6 | 49.823915 | -102.256923 | 1128461 | 5555821 | 1 | 7.84 | Local |
| M7 | 06/11/2012 0:00 | 9 | 49.823968 | -102.256851 | 1128466 | 5555828 | 1 | 9.42 | Local |
| M7 | 06/11/2012 0:00 | 12 | 49.824049 | -102.256814 | 1128467 | 5555837 | 1 | 267.10 | Local |
| M7 | 06/11/2012 0:00 | 15 | 49.821895 | -102.255210 | 1128610 | 5555612 | 1 | 854.85 | Local |
| M7 | 06/11/2012 0:00 | 18 | 49.820236 | -102.243665 | 1129461 | 5555525 | 1 | 13.52 | Local |
| M7 | 06/11/2012 0:00 | 21 | 49.820153 | -102.243530 | 1129472 | 5555517 | 1 | 2.84 | Local |
| M7 | 06/12/2012 0:00 | 0 | 49.820178 | -102.243536 | 1129471 | 5555520 | 0 | 4.71 | Local |
| M7 | 06/12/2012 0:00 | 6 | 49.820150 | -102.243486 | 1129475 | 5555517 | 1 | 9.90 | Local |
| M7 | 06/12/2012 0:00 | 9 | 49.820233 | -102.243436 | 1129477 | 5555527 | 0 | 2931.60 | Local |
| M7 | 06/12/2012 0:00 | 18 | 49.794323 | -102.237029 | 1130276 | 5552706 | 1 | 1336.27 | Local |
| M7 | 06/12/2012 0:00 | 21 | 49.782778 | -102.232201 | 1130773 | 5551466 | 1 | 1476.84 | Local |
| M7 | 06/13/2012 0:00 | 0 | 49.778785 | -102.212741 | 1132224 | 5551188 | 0 | 3971.38 | Local |
| M7 | 06/13/2012 0:00 | 6 | 49.791046 | -102.264272 | 1128362 | 5552112 | 1 | 272.57 | Local |
| M7 | 06/13/2012 0:00 | 9 | 49.788613 | -102.263990 | 1128414 | 5551845 | 1 | 295.97 | Local |
| M7 | 06/13/2012 0:00 | 12 | 49.786359 | -102.266141 | 1128288 | 5551577 | 1 | 1386.42 | Local |
| M7 | 06/13/2012 0:00 | 15 | 49.775995 | -102.276682 | 1127666 | 5550338 | 1 | 322.93 | Local |
| M7 | 06/13/2012 0:00 | 18 | 49.773231 | -102.275378 | 1127795 | 5550042 | 1 | 187.22 | Local |
| M7 | 06/13/2012 0:00 | 21 | 49.774869 | -102.275925 | 1127735 | 5550219 | 1 | 8.80 | Local |

| | | | | | | | | | |
|---|---|---|---|---|---|---|---|---|---|
| M7 | 06/14/2012 0:00 | 0 | 49.774944 | -102.275888 | 1127737 | 5550228 | 1 | 771.58 | Local |
| M7 | 06/14/2012 0:00 | 3 | 49.780242 | -102.282731 | 1127176 | 5550758 | 1 | 1679.28 | Local |
| M7 | 06/14/2012 0:00 | 6 | 49.789562 | -102.264518 | 1128363 | 5551946 | 1 | 119.80 | Local |
| M7 | 06/14/2012 0:00 | 9 | 49.790562 | -102.265115 | 1128307 | 5552052 | 0 | 1639.18 | Local |
| M7 | 06/14/2012 0:00 | 15 | 49.799030 | -102.246605 | 1129526 | 5553147 | 1 | 1227.69 | Local |
| M7 | 06/14/2012 0:00 | 18 | 49.807253 | -102.257868 | 1128610 | 5553965 | 1 | 1891.45 | Local |
| M7 | 06/14/2012 0:00 | 21 | 49.822184 | -102.270202 | 1127530 | 5555517 | 1 | 126.88 | Local |
| M7 | 06/15/2012 0:00 | 0 | 49.822153 | -102.271957 | 1127405 | 5555499 | 1 | 1.33 | Local |
| M7 | 06/15/2012 0:00 | 3 | 49.822158 | -102.271973 | 1127404 | 5555500 | 0 | 9.47 | Local |
| M7 | 06/15/2012 0:00 | 12 | 49.822073 | -102.271980 | 1127404 | 5555490 | 1 | 20.41 | Local |
| M7 | 06/15/2012 0:00 | 15 | 49.822179 | -102.271750 | 1127419 | 5555504 | 0 | 3556.05 | Local |
| M7 | 06/17/2012 0:00 | 9 | 49.850286 | -102.248654 | 1128710 | 5558817 | 1 | 189.14 | Local |
| M7 | 06/17/2012 0:00 | 12 | 49.851157 | -102.250899 | 1128538 | 5558895 | 1 | 179.62 | Local |
| M7 | 06/17/2012 0:00 | 15 | 49.850369 | -102.248732 | 1128704 | 5558826 | 0 | NA | Local |
